# Identification and Characterization of 293T Cell-Derived Exosomes by Profiling the Protein, mRNA and MicroRNA Components

**DOI:** 10.1371/journal.pone.0163043

**Published:** 2016-09-20

**Authors:** Jing Li, Xiulan Chen, Jiao Yi, Yuchen Liu, Dameng Li, Jifeng Wang, Dongxia Hou, Xiaohong Jiang, Junfeng Zhang, Jin Wang, Ke Zen, Fuquan Yang, Chen-Yu Zhang, Yujing Zhang

**Affiliations:** 1 State Key Laboratory of Pharmaceutical Biotechnology, Jiangsu Engineering Research Center for MicroRNA Biology and Biotechnology, NJU Advanced Institute for Life Sciences, School of Life Sciences, Nanjing University, Nanjing, Jiangsu, China; 2 Key Laboratory of Protein and Peptide Pharmaceuticals & Laboratory of Proteomics, Institute of Biophysics, Chinese Academy of Sciences, Beijing, China; University of Surrey, UNITED KINGDOM

## Abstract

Cell-derived exosomes are leading candidates for *in vivo* drug delivery carriers. In particular, exosomes derived from 293T cells are used most frequently, although exosome dosing has varied greatly among studies. Considering their biological origin, it is crucial to characterize the molecular composition of exosomes if large doses are to be administered in clinical settings. In this study, we present the first comprehensive analysis of the protein, messenger RNA and microRNA profiles of 293T cell-derived exosomes; then, we characterized these data using Gene Ontology annotation and Kyoto Encyclopedia for Genes and Genomes pathway analysis. Our study will provide the basis for the selection of 293T cell-derived exosome drug delivery systems. Profiling the exosomal signatures of 293T cells will lead to a better understanding of 293T exosome biology and will aid in the identification of any harmful factors in exosomes that could cause adverse clinical effects.

## Introduction

Exosomes are small (30–120 nm) membrane vesicles of endocytic origin that are released into the extracellular environment through the fusion of multivesicular bodies with the plasma membrane [1]. Cells release exosomes under both normal and pathological conditions, and they can be isolated from extracellular fluids, including blood, urine, amniotic fluid, saliva, milk, malignant ascites, synovial fluid and cerebrospinal fluid [2]. Many different types of cells can secret exosomes, such as platelets, B cells, T cells, mast cells, dendritic cells (DCs), epithelial cells and tumor cells [3–9]. Depending on their cellular origin, exosomes contain specific profiles of cellular proteins, signaling proteins and/or peptides, microRNAs (miRNAs), messenger RNAs (mRNAs) and lipids. These components are often intact and functional, and they can be altered by stress or pathological conditions [2]. Thus, the protein, mRNA and miRNA profiles of circulating exosomes can be used for clinical diagnostics and prognostics and may have therapeutic implications.

Exosomes are involved in many biological functions, including immune response regulation, antigen presentation, tumor proliferation and intercellular communication [10,11]. Recently, several studies have suggested that exosomes transfer protein, mRNA and miRNA cargo to target cells [12–17]. As exosomes have fewer immunogenic properties than other foreign small interfering RNA (siRNA) delivery vehicles (e.g., viruses, lipid nanoparticles and polymeric nanoparticles) [18], the development of exosome-based drug delivery systems has exciting prospects for future clinical use.

Many studies have demonstrated how exosome-based drug delivery systems can improve specific disease conditions [17,19–24]. The choice of an optimal donor cell type is one initial requirement for developing an efficient exosome-based drug delivery system. Furthermore, exosomes must remain stable in circulation for sufficiently long to deliver their cargo with only minor immune-stimulating activity to prevent inflammatory responses. A variety of cell types have been used experimentally to secrete exosomes, although model cell lines, such as 293T and HeLa, have been used more frequently than murine melanoma cell lines (e.g., B16-F10, B16-BL6 and B16-F1), immature DCs and mesenchymal stem cells (MSCs) [25,26]. However, the dosing of exosomes in past studies has varied greatly, ranging from 1 to 250 μg per in vivo injection [27], and if large doses are to be administered in clinical settings, it is important that we fully characterize the composition of these exosomes. Such analyses will be crucial for identifying potential hazards within exosomes and avoiding adverse effects in patients.

In this study, we present the first full description of the protein, mRNA and miRNA profiles of 293T cell-derived exosomes, which were characterized using Gene Ontology (GO) annotation and Kyoto Encyclopedia for Genes and Genomes (KEGG) pathway analysis. Profiling exosomal signatures will help us better understand the molecular mechanisms mediated by 293T cell-derived exosomes and identify any potentially harmful exosomal factors, which together will improve the prospects for drug delivery applications.

## Materials and Methods

### Cell lines

293T cells were cultured in high-glucose Dulbecco’s modified Eagle’s medium supplemented with 10% exosome-depleted fetal bovine serum (FBS) and antibiotics (Gibco, CA, USA). Cells were incubated at 37°C in 5% CO_2_. Exosomes in the FBS were depleted by filtration through a 0.22-μm filter and ultracentrifugation at 110,000 g for 2 h.

### Isolation of 293T cell-derived exosomes

293T cells were incubated in 225-cm^2^ flasks (Corning, NY, USA), and the supernatants were harvested after 48 h of incubation with FBS-free culture medium. The samples were immediately subjected to serial differential centrifugation at 300 g*5 min, 3000 g*30 min and 10,000 g*60 min to remove cells, cell fragments and shedding vesicles. Then, exosomes were isolated from the cell culture medium using an Exosome Isolation Kit (Invitrogen) according to the manufacturer’s recommended protocols [21,28]. Exosomes were collected from the pellets and re-suspended in phosphate-buffered saline (PBS). After re-suspending the exosomes in PBS, a 0.22-μm polyvinylidene difluoride (PVDF) membrane filter was used to remove any remaining cells or debris contaminating the crude samples.

### Transmission electron microscopy (TEM)

For TEM, after the exosome samples were prepared as previously described, the purified exosomes were re-centrifuged using an Exosome Isolation Kit to collect exosome pellets. Briefly, each exosome pellet was placed in a droplet of 2.5% glutaraldehyde in PBS buffer and fixed overnight at 4°C. The exosome samples were rinsed 3 times in PBS for 10 min each and then fixed in 1% osmium tetroxide for 60 min at room temperature. Then, the samples were embedded in 10% gelatin, fixed in glutaraldehyde at 4°C and cut into small blocks (less than 1 mm^3^). The samples were dehydrated in increasing concentrations of alcohol, placed in propylene oxide and infiltrated with increasing concentrations of Quetol-812 epoxy resin mixed with propylene oxide for 3 h per step. Finally, the samples were embedded in pure, fresh Quetol-812 epoxy resin, which was allowed to polymerize at 35°C for 12 h, 45°C for 12 h, and 60°C for 24 h. Ultrathin sections were cut using a Leica UC6 ultra-microtome and stained with uranyl acetate for 10 min and lead citrate for 5 min at room temperature. The samples were then observed with a transmission electron microscope (JEM-1010) at a voltage of 110 kV.

### Nanoparticle tracking analysis (NTA)

Exosomes re-suspended in PBS at a concentration of 5 μg of protein/ml were further diluted 100- to 500-fold to achieve between 20–100 objects per frame. Samples were manually injected into the sample chamber at ambient temperature. Each sample was measured in triplicate at camera setting 13 with an acquisition time of 30 s and detection threshold setting of 7. At least 200 completed tracks were analyzed per video. NTA analytical software version 2.3 was used for capturing and analyzing the data.

### RNA isolation

Total RNA from 293T cell-derived exosomes was extracted using TRIzol LS Reagent (Invitrogen) according to the manufacturer’s recommended protocols.

### Western blot

The levels of CD9 and CD63 protein in 293T cells and exosomes were quantified by Western blot analysis using antibodies against CD9 (H-110) (Santa Cruz, sc-9148) and CD63 (H-193) (Santa Cruz, sc-15363).

### In-solution digestion and desalting

For liquid chromatography-tandem mass spectrometry (LC-MS/MS), after the exosome samples were prepared as previously described, the purified exosomes were re-centrifuged using an Exosome Isolation Kit, and the exosome pellets were lysed in 8 M urea buffer. Three batches of 293T cell-derived exosome proteins were digested in solution into peptides as previously described [29]. Briefly, the protein mixture was reduced and alkylated before the addition of Lys-C and subsequent digestion at 37°C for 6 h. After diluting the urea concentration to 1 M with 25 mM NH_4_HCO_3_, sequence-grade trypsin (Promega, Madison, WI) was added at an enzyme/protein ratio of 1:50; the digestion was performed at 37°C overnight and was stopped by adding formic acid (FA) at a final concentration of 0.1%. The digested peptide mixture was centrifuged at 20,000 g for 10 min, and the resulting supernatant was collected for desalting.

Peptide desalting was performed with a Waters Oasis HLB column (1 cc, 30 mg). Briefly, the column was washed once with 1 ml of methanol and 1 ml of acetonitrile (ACN), followed by three equilibration steps with 1 ml of 0.1% FA each. Then, samples were loaded onto the column three times. Next, the column was washed three times with 1 ml of 0.1% FA for desalting, and the samples were eluted sequentially with 0.5 ml of 40% ACN/0.1% FA and 0.5 ml of 60% ACN/0.1% FA. The combined eluate was concentrated in a Benchtop Centrifugal Vacuum Concentrator (Labconco, MO, USA).

### Online high pH fractionation

The concentrated samples were dissolved in high pH fraction buffer A (98% H_2_O/2% ACN, pH 10, pH adjusted with ammonium hydroxide) and loaded onto an XBridge C18 basic reversed-phase LC column (150×2.1 mm, 3.5 μm particles) (Waters). Peptides were separated in a Rigol L-3000 LC system (Beijing, China) with a binary buffer system of buffer A and buffer B (98% ACN/2% H_2_O, pH 10). The gradient of buffer B was set as follows: 5–8% for 5 min, 8–18% for 35 min, 18–32% for 22 min, 32–95% for 2 min, and 95% for 4 min. Eluates were collected every 90 s, and a total of 42 fractions were obtained. These fractions were combined into 14 fractions by merging fractions 1, 15, and 29; fractions 2, 16, and 30; and so on. Then, all the combined fractions were dried in a Benchtop Centrifugal Vacuum Concentrator and stored at -20°C for further MS analysis.

### LC-MS/MS analysis

Samples were analyzed by reversed-phase LC on an EASY-nLC 1000 system directly coupled to a Q Exactive mass spectrometer (Thermo Fisher Scientific) using a nanoelectrospray source (Thermo Fisher Scientific). High performance liquid chromatography (HPLC) columns 20 cm in length and 75 μm in inner diameter were packed in house with ReproSil-Pur 130 C18-AQ 3 μm particles (Dr. Maisch HPLC GmbH, Germany). Peptide mixtures were separated using linear gradients of 78 min and a two buffer system: buffer A (0.1% FA) and buffer B (ACN/0.1% FA). The flow rate was set to 280 nl/min. Peptides eluting from the column were directly sprayed into the mass spectrometer with a spray voltage of 2.0 kV and a capillary temperature of 320°C. The mass spectrometer was operated in a data-dependent mode, acquiring survey scans at a resolution of 70,000 with an automatic gain control target of 3E6 ions and a maximum ion injection time of 60 ms. Subsequently, the top 20 most abundant peaks were selected for fragmentation with an isolation window of 2 m/z and were fragmented by higher energy collisional dissociation with a normalized collision energy of 27. Fragmentation spectra were acquired at a resolution of 17,500 with a target value of 5E4 ions and a maximum ion injection time of 60 ms. To minimize peptide re-sequencing, dynamic exclusion was enabled within a time window of 50 s.

### Raw MS data analysis

Raw MS files were processed using Proteome Discoverer (Version 1.4.0.288, Thermo Fisher Scientific, Bremen, Germany) with SEQUEST as the search engine. MS/MS spectra were searched against the UniprotKB human database (downloaded on May 11, 2015) and supplemented with known contaminants. Cysteine carbamidomethylation was set as a fixed modification, and N-terminal acetylation and methionine oxidation were set as variable modifications. Peptide mass and fragment mass tolerances were set at 10 ppm and 20 mDa, respectively, and a maximum of 2 missed cleavage sites were allowed. Peptide identifications were filtered at a 1% false discovery rate.

### Microarrays

Human genome expression analyses were conducted using the Affymetrix GeneChip^®^ Human Genome U133 Plus 2.0 Array, and miRNA analyses were performed using the Affymetrix GeneChip^®^ miRNA 3.0 Array (Affymetrix, CA, USA). Affymetrix^®^ Expression Console^®^ software was used for the profiling analysis.

### Data analysis for GO and KEGG pathways

To identify the most significantly enriched signal transduction pathways in the data set, the proteins with significantly different expression levels, as determined by our proteomics analysis, were categorized by GO and KEGG pathway analyses using DAVID (Database for Annotation, Visualization and Integrated Discovery) Bioinformatics Resources 6.7 (http://david.abcc.ncifcrf.gov/) [30,31].

## Results

### Production and characterization of exosomes derived from 293T cells

**Fig 1:**
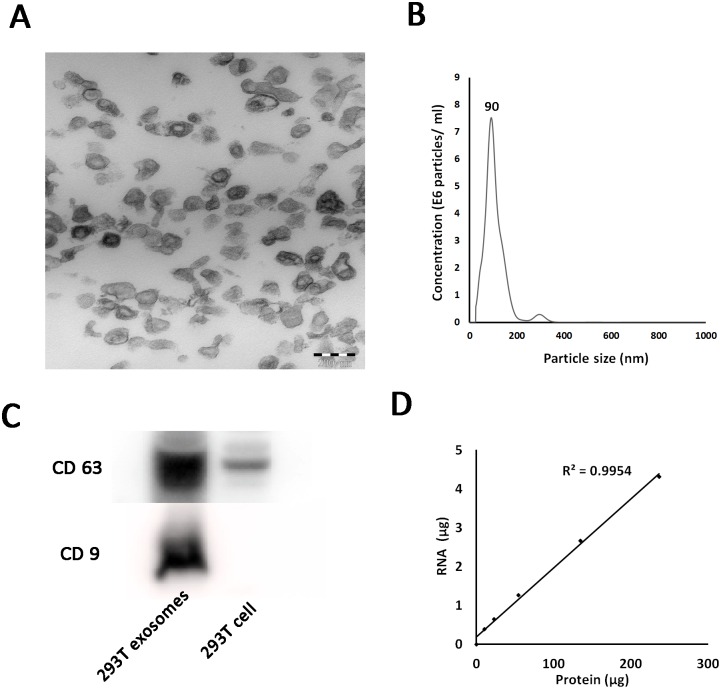
The characterization of 293T cell-derived exosomes. (A) TEM image of exosomes isolated from the culture medium of 293T cells. (B) Exosomes in the supernatants from cell cultures were measured using a Nanosight NS 300 system. The histogram represents the particle size distribution. (C) Western blot analysis of CD9 and CD63 protein levels of 293T cell-derived exosomes. (D) Pearson correlation coefficient (R) between RNA and protein levels of 293T cell-derived exosomes.

To characterize the proteins and RNAs that are enriched in exosomes from 293T cells, we first used an Exosome Isolation Kit to isolate crude exosomes from 293T cell culture supernatants after differential centrifugation. Then, after re-suspending the exosomes in PBS, a 0.22-μm PVDF membrane filter was used to remove any contaminating cells or debris remaining in the crude sample. The purified exosomes samples were then re-centrifuged using an Exosome Isolation Kit, and the pellets were examined by TEM, which showed that the purified vesicles were membrane bound, round and heterogeneous in size, ranging from approximately 40 to 120 nm (Fig 1A); these data were consistent with the NTA, which also showed the same size distribution (Fig 1B). The Western blot analysis revealed that the exosome-specific proteins CD63 and CD9 were both enriched in the exosome samples but not in the cell lysates (Fig 1C). To determine the purity of the isolated exosomes, we detected total RNA and protein levels in samples containing different concentrations of exosomes. Increasing the exosome input led to corresponding increases in RNA and protein levels, with a Pearson correlation coefficient (R) between RNA and protein levels close to 1 (Fig 1D). Taken together, these results confirmed that the isolated fractions were exosomes and that our exosome isolation method was accurate.

### Proteomic analysis of 293T cell-derived exosomes

**Fig 2:**
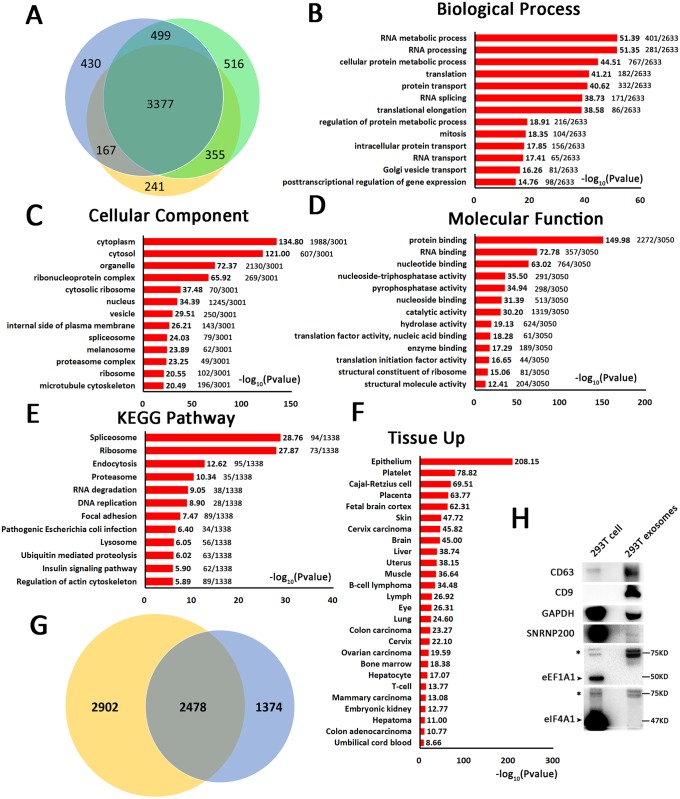
Proteomic analysis of 293T cell-derived exosomes. (A) Venn diagram of identified proteins from three replicate samples of 293T cell-derived exosomes by LC-MS/MS. A total of 3377 proteins were common to all three samples. The identified proteins in 293T cell-derived exosomes were analyzed by GO (B) biological process, (C) cellular component, and (D) molecular function annotation, as well as (E) KEGG pathway and (F) tissue specificity analyses using DAVID Bioinformatics Resources 6.7. (G) Venn diagram of identified proteins and proteins in the ExoCarta database. The yellow part represents proteins from ExoCarta, and the blue part represents proteins we identified. (H) Verification of the new proteins in 293T cell-derived exosomes by Western blot.

To examine the proteomic profile of exosomes secreted by 293T cells, LC-MS/MS analysis was performed following the in-solution digestion of isolated exosome proteins from three independent exosome samples and high pH reversed-phase fractionation of the digested peptides, as previously described. Then, the fractionated samples were analyzed by reversed-phase LC on an EASY-nLC 1000 system directly coupled to a Q Exactive mass spectrometer. The resulting MS/MS spectra were analyzed using SEQUEST and searched against the UniprotKB human sequence database supplemented with known contaminants. Protein database search showed the identification of 4594, 4742 and 4140 unique proteins and/or protein groups in the three replicate samples of 293T cell-derived exosomes. As shown in a Venn diagram (Fig 2A), a total of 3377 proteins were common to all three samples (S1 Table).

Using GO analysis in DAVID Bioinformatics Resources 6.7. (http://david.abcc.ncifcrf.gov/), exosome-derived proteins were categorized by biological process (Fig 2B), cellular component (Fig 2C) and molecular function (Fig 2D); these categories represent the annotation of the functional enrichment of specific proteins, and the data are presented as the -log10 P value. A higher value represents greater functional enrichment of a relative term. The biological processes associated with the identified proteins were focused on RNA and protein synthesis, processing and metabolism, including RNA metabolic processes (51.39), RNA processing (51.35), cellular protein metabolic processes (44.51), translation (41.21), protein transport (40.62), RNA splicing (38.73), translational elongation (38.58), and regulation of protein metabolic processes (18.91) (Fig 2B). Of the major cellular component classifications, cytoplasm and cytosol proteins were the most highly represented (134.80 and 121.00, respectively), with lower percentages for organelle proteins (72.37) and ribonucleoprotein complex proteins (65.92). This analysis also revealed relatively low proportions of the cytosolic ribosome (37.48), nucleus (34.39), vesicle (29.51), internal side of plasma membrane (26.21), spliceosome (24.03), melanosome (23.89), proteasome complex (23.25), ribosome (20.55) and microtubule cytoskeleton (20.49) classifications. The distribution of subcellular localization is shown graphically in Fig 2C. Certain functional activities, such as binding activity (protein binding, RNA binding, nucleotide binding, nucleoside binding, nucleic acid binding, and enzyme binding activity were 149.98, 72.78, 63.02, 31.39, 18.28 and 17.29, respectively), catalytic activity (30.20), and enzymatic activity (nucleoside-triphosphatase activity, pyrophosphatase activity and hydrolase activity were 35.50, 34.94 and 19.13, respectively), were significantly over-represented, suggesting that these activities may be essential for the biological functions of 293T cell-derived exosomes (Fig 2D). A KEGG pathway analysis of these proteins was mapped using the DAVID Bioinformatics Resource. A significant number of enriched proteins were associated with spliceosome (28.76) and ribosome (27.87), with weaker associations with endocytosis (12.62), proteasome (10.34), RNA degradation (9.05) and DNA replication (8.90) (Fig 2E), suggesting that 293T cell-derived exosomes may play functional roles in the synthesis, processing and degradation of DNA, RNA and protein, which is consistent with the biological process analysis. Using the UP_TISSUE tool in the DAVID Bioinformatics Resource, we compared this protein distribution with tissue expression databases and determined that these proteins are associated with epithelial tissues and less related to the protein profiles of other tissues, such as platelets, placenta, fetal brain cortex, skin, brain, liver, uterus, muscle, lymph and lung (Fig 2F). In addition, we compared our list of identified proteins to previously published exosome data in the ExoCarta database [32] (www.exocarta.org) and found that 2478 of these proteins had been previously observed, including typical exosome protein markers, e.g., CD9, CD63 and CD81, as well as the ESCRT component TSG101 and various VPS3 and CHMP components (S2 Table). Furthermore, 1374 proteins were detected in all three of the 293T cell-derived exosome samples but were not present in the ExoCarta database (S3 Table, Fig 2G). We chose some of the highly expressed proteins from these new proteins for confirmation, including GAPDH, SNRNP200, eEF1A1, and eIF4A1. The Western blot results showed that GAPDH and SNRNP200 were present in the 293T cell-derived exosomes, although the protein level of SNRNP200 was very low. Unfortunately, we did not detect the expression of eEF1A1 and eIF4A1, but we found larger version of both proteins approximately 75 kDa in size, which are labeled with asterisks; normally, these proteins are 50 kDa and 47 kDa in size, respectively, as indicated by arrows (Fig 2H). In an earlier study, the 75-kDa protein of eEF1A1 was considered a nonspecific band [33]. However, the 75-kDa proteins observed in this study could be new proteins containing the same domains as eEF1A1 and eIF4A1, as they were relatively enriched in 293T cell-derived exosomes. To identify any relationships between these proteins and disease, we analyzed associations between our data set and known disease-related genes using the OMIM_DISEASE tool in the DAVID Bioinformatics Resource, but we found only a few poorly correlated associations (S1 Fig).

### Conjoint analysis of mRNA and protein profiles of 293T cell-derived exosomes

**Fig 3:**
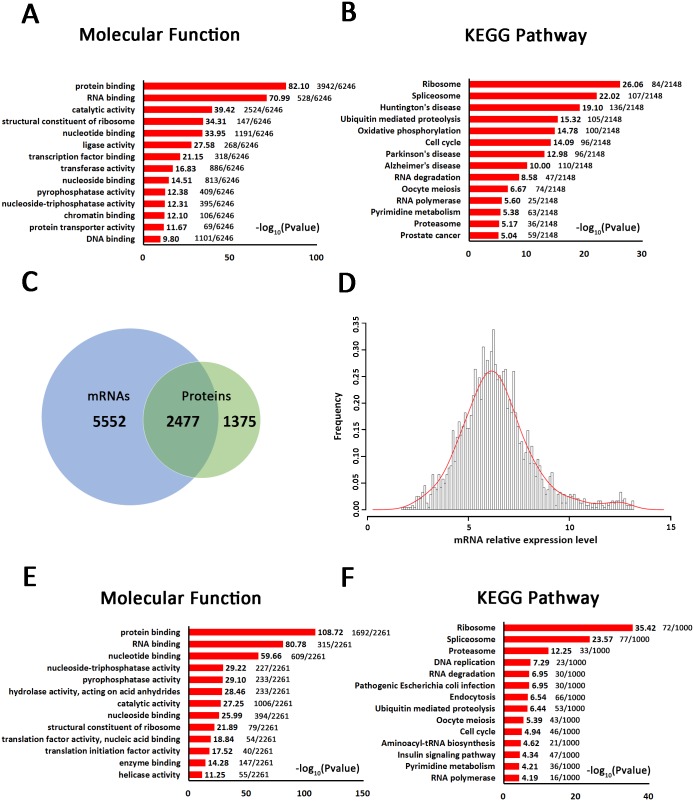
Conjoint analysis of mRNA and protein profiles of 293T cell-derived exosomes. (A) The 8029 mRNAs identified from 293T cell-derived exosomes were classified by molecular function. (B) KEGG pathway analysis of the mRNAs identified from 293T cell-derived exosomes. (C) Venn diagram of identified mRNAs and proteins from 293T cell-derived exosomes. The blue part represents proteins, and the green part represents mRNAs. Both the proteins and mRNAs of 2477 genes were present in the 293T cell-derived exosomes. (D) Relative expression levels of these 2477 mRNAs in 293T cell-derived exosomes. (E) Molecular functions and (F) KEGG pathway analysis of these 2477 genes.

RNA was isolated from exosomes released by 293T cells. Whole human genome expression arrays were utilized to identify genes expressed in these exosomes, and 8029 expression signals were identified as present in all three samples (S4 Table). Interestingly, the molecular function analysis of these mRNA transcripts revealed the greatest involvement in binding activity (the -log10 P values for protein binding, RNA binding, nucleotide binding, transcription factor binding, nucleoside binding, chromatin binding and DNA binding activity were 82.10, 70.99, 33.95, 21.15, 14.51, 12.10 and 9.80, respectively), catalytic activity (39.42), and enzymatic activity (pyrophosphatase activity and nucleoside-triphosphatase activity were 12.38 and 12.31, respectively) (Fig 3A). The KEGG pathway analysis showed that these genes were mostly involved in the ribosome (26.06), spliceosome (22.02), RNA degradation (8.58) and proteasome (5.17) (Fig 3B), which was similar to the protein analysis. As shown in the Venn diagram, 2477 mRNA transcripts were overlapped with the protein set, accounting for nearly 64.30% of the exosomal proteins identified by LC-MS/MS (Fig 3C); the relative expression levels of these mRNAs were normally distributed (Fig 3D). In addition, most of these mRNAs were associated with DNA replication, RNA transcript and translation, and protein processing, as shown in the molecular function analysis (Fig 3E) and KEGG pathway analysis (Fig 3F). In addition to these mRNAs that overlapped with the identified proteins, some mRNA transcripts were associated with ligase activity (27.58) and transferase activity (16.83) in the molecular function analysis (Fig 3A) and with Huntington’s disease (19.10), oxidative phosphorylation (14.78), Parkinson’s disease (12.98), Alzheimer’s disease (10.00) and prostate cancer (5.04) in the KEGG pathway analysis (Fig 3B). Taken together, these data suggest that exosomes were enriched with DNA, RNA and proteins related with protein and miRNA processing.

### Gene target analysis of miRNA profile of 293T cell-derived exosomes

**Fig 4:**
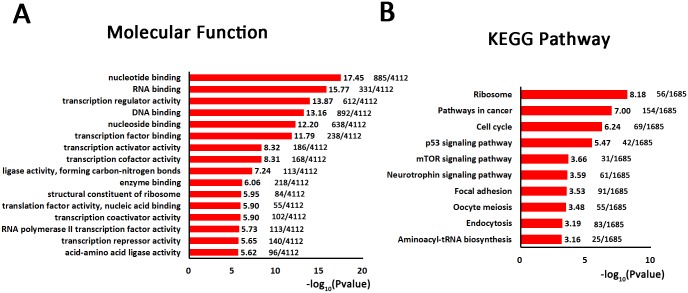
Gene target analysis of miRNA profile of 293T cell-derived exosomes. In all, 83 miRNAs were analyzed by TargetScan Human 7.0, and 6194 potential target transcripts were identified. (A) Molecular functions of these 6194 potential target transcripts. (B) KEGG pathway analysis of these 6194 potential target transcripts.

To profile miRNAs, total RNA isolated from 293T cell-derived exosomes was analyzed using miRNA microarrays. In total, 467 miRNAs were identified, and 83 were above the normalized threshold based on the 95^th^ percentile of the negative control probe signal in the exosomes and present in all three samples (S5 Table). The expression of the hsa-miR-3960, hsa-miR-1469, hsa-miR-4497, hsa-miR-3665, hsa-miR-4787-5p, miR-17 and miR-221/222 miRNA clusters was comparatively high. To better understand the function of the miRNAs present in 293T cell-derived exosomes, potential miRNA targets in the human genome were explored using TargetScan Human 7.0, and 6194 potential target transcripts were identified (S6 Table). A molecular function analysis of these predicted targets was conducted to determine the potential function of these miRNAs in target cells; most of the identified functions were consistent with the protein and mRNA analyses, including nucleotide binding (17.45), RNA binding (15.77), transcription regulator activity (13.87), DNA binding (13.16), nucleoside binding (12.20), and transcription factor binding (11.79) (Fig 4A). As shown by the KEGG pathway analysis (Fig 4B), the most related pathway was ribosome (8.18), with very few transcripts in cell signaling pathways (pathways in cancer, p53 signaling pathway, mTOR signaling and neurotrophin signaling pathway).

## Discussion

Exosomes have been used for drug delivery in many studies due to their ability to transmit RNA species between cells, a feature that is crucial for some types of targeted therapy. These vesicles exert their biological effects in a pleiotropic manner, directly activating cell surface receptors via protein and bioactive ligands, merging their membrane contents with the recipient cell plasma membrane and delivering effector proteins, mRNAs and miRNAs into recipient cells. In this way, exosomes participate in the maintenance of normal physiology during stem cell maintenance, tissue repair and immune surveillance [34,35], but they can also play important roles in disease pathogenesis [14–16]. Therefore, it is crucial that we identify and characterize the content of exosomes to determine their potential as drug delivery vehicles for future clinical applications. As 293T cell-derived exosomes are one of the most extensively used tools in drug delivery system research, we decided to characterize the protein, mRNA and miRNA profiles of these structures.

In this study, we isolated exosomes from the cell culture medium of 293T cells in accordance with the criteria for exosomes proposed by Théry and colleagues [34]. Then, we investigated the protein, mRNA and miRNA profiles of these exosomes using LC-MS/MS analysis, whole human genome expression arrays and miRNA arrays, respectively; target genes for the identified miRNAs were predicted using TargetScan Human 7.0. This is the first study to systematically characterize exosomes released by 293T cells, and these data will aid in our understanding of their molecular composition and provide support to other research groups studying 293T cell-derived exosomes.

To be an ideal drug delivery system, exosomes should contain cargo that causes minimal negative effects. Our results showed that few disease-related or cancer-related pathways were enriched in 293T cell-derived exosomes as determined by the GO and KEGG pathway analyses of the LC-MS/MS proteomic, mRNA microarray and miRNA microarray data. MS data analyzed using the UP_TISSUE tool showed that the protein expression profile of 293T cell-derived exosomes was highly related to only epithelial tissues, even though 293T cells originated from an embryonic kidney. Considering the high transfection efficiency of 293T cells, it is easy to load therapeutic small RNAs and modify exosomes by transfection; this characteristic is advantageous for potential industrial applications. These characteristics combined with the limited tissue specificity and disease correlations support the selection of 293T cell-derived exosomes for in vivo drug delivery applications.

Another characteristic of an ideal drug delivery system is targeting ability. Except for the epithelium, which is the most likely tissue of origin, various tissues, such as platelets, Cajal-Retzius cells, placenta, fetal brain cortex, skin, cervical carcinoma, brain, liver, uterus, muscle, B-cell lymphoma, lymph, eye, lung, bone marrow, and hepatocytes, were included in the tissue distribution of identified proteins from 293T cell-derived exosomes (Fig 2F). The comprehensive tissue coverage indicates the 293T cell-derived exosomes may have similar properties to these tissues, and similarities at the membrane level will help enable exosome membrane fusion in these tissues. Perhaps this explains the universal application of 293T cell-derived exosomes as drug or siRNA delivery systems for many types of target tissues. However, too many target tissues may cause off-target effects; 293T cell-derived exosomes that are modified to target specific cells may have considerably better application prospects. The KEGG pathway analysis of proteins, mRNAs and predicted miRNA targets revealed that 293T cell-derived exosomes have few relationships with some physiological and pathological processes (Figs 2–4). This result suggests that 293T cell-derived exosomes are suitable for use as in vivo drug delivery vehicles.

In addition to the potential applicability in drug delivery, we also found many other interesting features of exosomes that have not been previously reported. First, the proteins in exosomes were mostly concentrated in the categories of RNA and protein synthesis and modification (Fig 2D and 2E), indicating that these exosomes may contain active and functional assembly and processing machinery instead of randomly packaged independent and irrelevant molecules. These proteins may then be able to regulate gene expression in target cells, which could explain why exosomes with limited mRNA and protein expression have such a marked influence on target cells, as we found in previous studies.

Second, the molecular function and KEGG pathway analyses revealed that 64.30% of the exosomal proteins identified by LC-MS/MS were consistent with the exosomal mRNAs. Interestingly, these exosomes also contain proteins related to translation and protein modification, which suggests that these proteins might be translated from the mRNAs after being packaged into the exosomes; in addition, the mRNAs could potentially not only function in target cells but also be expressed in the exosomes after secretion. Most of these exosomal proteins were related to RNA and protein synthesis and processing machinery, suggesting that the exosomes harbor not only ready-to-use protein machinery but also mRNAs that can be translated if necessary. These are very interesting observations that require further study for confirmation.

Third, and most interestingly, the predicted miRNA targets were also consistent with the mRNA and protein analyses; the primary targets were RNA and protein processing machinery. These data may indicate that exosomal miRNAs play a role in regulating mRNA translation. This goes far beyond the traditional thinking that exosomes are carriers of proteins, mRNAs and miRNAs that may function in target cells and mediate intercellular and tissue interactions. However, we must now re-examine exosomes as not only a means of transport but also a systematic processing factory with precise regulation; more studies are necessary to identify specific mechanisms. Similarly, whether these are unique characteristics of 293T cell-derived exosomes or universal phenomena in all exosomes must be determined.

In addition, certain oncogenic and immunogenic molecules, such as epidermal growth factor receptor, Src kinase and Raf kinase, were present as both protein and mRNA in the exosomes. Recently, exosomes isolated from both urine and from embryonic MSCs have been shown to be bioactive vesicles that consume oxygen to aerobically synthesize ATP capable of oxidative phosphorylation[36–38]. This metabolic function emerging for human exosomes should be considered in the clinical use of exosomes as carriers. Therefore, when 293T cell-derived exosomes are used for drug delivery, the influence of these factors, which could be pathogenic, should be taken into account, as well as the dosage of exosomes and the related management of these factors. In addition, although the exosomes were harvested from FBS-free culture medium, many FBS-derived proteins were detected by LC-MS/MS. As it is unclear whether these proteins would influence drug delivery in clinical practice, specific cell culture methods should be explored. Moreover, 293T cells carry a temperature-sensitive T antigen co-selected with neomycin, but whether the exosomes contained this protein could not be established from our data because the LC-MS/MS data were analyzed only using a human protein database. Further studies should be performed to ensure that the cited proteins are present. Aside from the advantages of these exosomes as a drug delivery system, certain pathogenic factors of the exosomes remained; as such, the related disposal methods and the proper dosage of exosomes should be carefully considered.

In summary, our findings present the first comprehensive analysis of the protein, mRNA and miRNA profiles of 293T cell-derived exosomes. This study presents an important step toward using 293T cell-derived exosomes as a drug delivery system and launches a new area of research into the function and classification of exosomes.

## Supporting Information

S1 Fig A total of 3377 proteins were analyzed by the OMIM_DISEASE tool. (TIF)

S1 Table A total 3377 proteins were identified by LC-MS/MS. (PDF)

S2 Table A total of 2478 identified proteins had been previously observed in the ExoCarta database. (PDF)

S3 Table A total of 1374 identified proteins were not present in the ExoCarta database. (PDF)

S4 Table A total of 8029 mRNAs were identified by microarray. (PDF)

S5 Table A total of 83 miRNAs above the normalized threshold were identified by microarray. (PDF)

S6 Table A total of 6194 potential target transcripts were identified by using TargetScan Human 7.0. (PDF)
