# Supplementary figures and images for: Identification and Characterization of 293T Cell-Derived Exosomes by Profiling the Protein, mRNA and MicroRNA Components
Source: PLoS One. 2016 Sep 20;11(9):e0163043. doi: 10.1371/journal.pone.0163043 (PMC5029934; doi:10.1371/journal.pone.0163043)

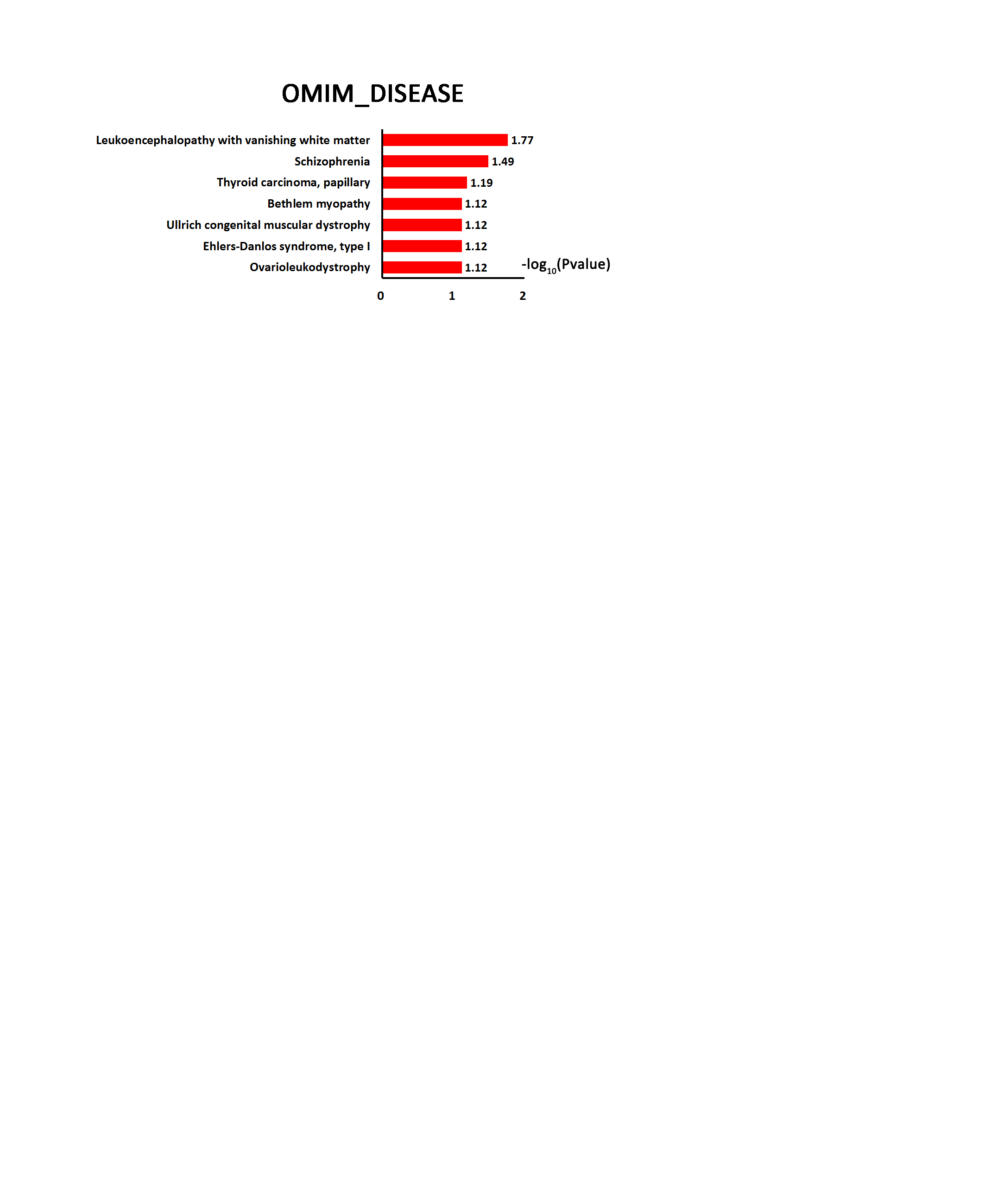

Supplement: S1 Fig — (TIF) [file pone.0163043.s001.tif]
