# Supplementary material for: Identification and Characterization of 293T Cell-Derived Exosomes by Profiling the Protein, mRNA and MicroRNA Components
Source: PLoS One. 2016 Sep 20;11(9):e0163043. doi: 10.1371/journal.pone.0163043 (PMC5029934; doi:10.1371/journal.pone.0163043)
Supplement: S1 Table — (PDF) [file pone.0163043.s002.pdf]

UNIPROT_ACCESSION	Name
P0CG48	Polyubiquitin-C OS=Homo sapiens GN=UBC PE=1 SV=3 - [UBC_HUMAN]
P07737	Profilin-1 OS=Homo sapiens GN=PFN1 PE=1 SV=2 - [PROF1_HUMAN]
P63244	Guanine nucleotide-binding protein subunit beta-2-like 1 OS=Homo sapiens GN=GNB2L1 PE=1 SV=3 - [GBLP_HUMAN]
Q9BQE3	Tubulin alpha-1C chain OS=Homo sapiens GN=TUBA1C PE=1 SV=1 - [TBA1C_HUMAN]
P05387	60S acidic ribosomal protein P2 OS=Homo sapiens GN=RPLP2 PE=1 SV=1 - [RLA2_HUMAN]
P07437	Tubulin beta chain OS=Homo sapiens GN=TUBB PE=1 SV=2 - [TBB5_HUMAN]
P68371	Tubulin beta-4B chain OS=Homo sapiens GN=TUBB4B PE=1 SV=1 - [TBB4B_HUMAN]
P04350	Tubulin beta-4A chain OS=Homo sapiens GN=TUBB4A PE=1 SV=2 - [TBB4A_HUMAN]
P49773	Histidine triad nucleotide-binding protein 1 OS=Homo sapiens GN=HINT1 PE=1 SV=2 - [HINT1_HUMAN]
Q15365	Poly(rC)-binding protein 1 OS=Homo sapiens GN=PCBP1 PE=1 SV=2 - [PCBP1_HUMAN]
Q9BVA1	Tubulin beta-2B chain OS=Homo sapiens GN=TUBB2B PE=1 SV=1 - [TBB2B_HUMAN]
O00560	Syntenin-1 OS=Homo sapiens GN=SDCBP PE=1 SV=1 - [SDCB1_HUMAN]
P62937	Peptidyl-prolyl cis-trans isomerase A OS=Homo sapiens GN=PPIA PE=1 SV=2 - [PPIA_HUMAN]
P60660	Myosin light polypeptide 6 OS=Homo sapiens GN=MYL6 PE=1 SV=2 - [MYL6_HUMAN]
P68104	Elongation factor 1-alpha 1 OS=Homo sapiens GN=EEF1A1 PE=1 SV=1 - [EF1A1_HUMAN]
Q9UMS4	Pre-mRNA-processing factor 19 OS=Homo sapiens GN=PRPF19 PE=1 SV=1 - [PRP19_HUMAN]
Q9BQA1	Methylosome protein 50 OS=Homo sapiens GN=WDR77 PE=1 SV=1 - [MEP50_HUMAN]
Q13347	Eukaryotic translation initiation factor 3 subunit I OS=Homo sapiens GN=EIF3I PE=1 SV=1 - [EIF3I_HUMAN]
P12277	Creatine kinase B-type OS=Homo sapiens GN=CKB PE=1 SV=1 - [KCRB_HUMAN]
P60709	Actin, cytoplasmic 1 OS=Homo sapiens GN=ACTB PE=1 SV=1 - [ACTB HUMAN]
P63218	Guanine nucleotide-binding protein G(I)/G(S)/G(O) subunit gamma-5 OS=Homo sapiens GN=GNG5 PE=1 SV=3 - [GBG5_HUMAN]
P04075	Fructose-bisphosphate aldolase A OS=Homo sapiens GN=ALDOA PE=1 SV=2 - [ALDOA_HUMAN]
P35244	Replication protein A 14 kDa subunit OS=Homo sapiens GN=RPA3 PE=1 SV=1 - [RFA3_HUMAN]
P19623	Spermidine synthase OS=Homo sapiens GN=SRM PE=1 SV=1 - [SPEE_HUMAN]
Q08431	Lactadherin OS=Homo sapiens GN=MFGE8 PE=1 SV=2 - [MFGM_HUMAN]
P14618	Pyruvate kinase PKM OS=Homo sapiens GN=PKM PE=1 SV=4 - [KPYM_HUMAN]
P23528	Cofilin-1 OS=Homo sapiens GN=CFL1 PE=1 SV=3 - [COF1_HUMAN]
P02787	Serotransferrin OS=Homo sapiens GN=TF PE=1 SV=3 - [TRFE_HUMAN]
P14543	Nidogen-1 OS=Homo sapiens GN=NID1 PE=1 SV=3 - [NID1_HUMAN]
Q9BUF5	Tubulin beta-6 chain OS=Homo sapiens GN=TUBB6 PE=1 SV=1 - [TBB6_HUMAN]
P08670	Vimentin OS=Homo sapiens GN=VIM PE=1 SV=4 - [VIME_HUMAN]
P35998	26S protease regulatory subunit 7 OS=Homo sapiens GN=PSMC2 PE=1 SV=3 - [PRS7_HUMAN]
P50990	T-complex protein 1 subunit theta OS=Homo sapiens GN=CCT8 PE=1 SV=4 - [TCPQ_HUMAN]
Q13526	Peptidyl-prolyl cis-trans isomerase NIMA-interacting 1 OS=Homo sapiens GN=PIN1 PE=1 SV=1 - [PIN1_HUMAN]
Q99832	T-complex protein 1 subunit eta OS=Homo sapiens GN=CCT7 PE=1 SV=2 - [TCPH_HUMAN]
P35606	Coatomer subunit beta' OS=Homo sapiens GN=COPB2 PE=1 SV=2 - [COPB2_HUMAN]
P49721	Proteasome subunit beta type-2 OS=Homo sapiens GN=PSMB2 PE=1 SV=1 - [PSB2_HUMAN]
P51149	Ras-related protein Rab-7a OS=Homo sapiens GN=RAB7A PE=1 SV=1 - [RAB7A_HUMAN]
P62979	Ubiquitin-40S ribosomal protein S27a OS=Homo sapiens GN=RPS27A PE=1 SV=2 - [RS27A_HUMAN]
P60174	Triosephosphate isomerase OS=Homo sapiens GN=TPI1 PE=1 SV=3 - [TPIS_HUMAN]
P62244	40S ribosomal protein S15a OS=Homo sapiens GN=RPS15A PE=1 SV=2 - [RS15A_HUMAN]
Q9UNM6	26S proteasome non-ATPase regulatory subunit 13 OS=Homo sapiens GN=PSMD13 PE=1 SV=2 - [PSD13_HUMAN]
Q9UK41	Vacuolar protein sorting-associated protein 28 homolog OS=Homo sapiens GN=VPS28 PE=1 SV=1 - [VPS28_HUMAN]
Q13885	Tubulin beta-2A chain OS=Homo sapiens GN=TUBB2A PE=1 SV=1 - [TBB2A_HUMAN]
Q9H4M9	EH domain-containing protein 1 OS=Homo sapiens GN=EHD1 PE=1 SV=2 - [EHD1_HUMAN]
P68366	Tubulin alpha-4A chain OS=Homo sapiens GN=TUBA4A PE=1 SV=1 - [TBA4A_HUMAN]
Q8WUM4	Programmed cell death 6-interacting protein OS=Homo sapiens GN=PDCD6IP PE=1 SV=1 - [PDC6I_HUMAN]
O75369	Filamin-B OS=Homo sapiens GN=FLNB PE=1 SV=2 - [FLNB_HUMAN]
P00492	Hypoxanthine-guanine phosphoribosyltransferase OS=Homo sapiens GN=HPRT1 PE=1 SV=2 - [HPRT_HUMAN]
P40227	T-complex protein 1 subunit zeta OS=Homo sapiens GN=CCT6A PE=1 SV=3 - [TCPZ_HUMAN]
Q05639	Elongation factor 1-alpha 2 OS=Homo sapiens GN=EEF1A2 PE=1 SV=1 - [EF1A2_HUMAN]
P62263	40S ribosomal protein S14 OS=Homo sapiens GN=RPS14 PE=1 SV=3 - [RS14_HUMAN]
Q9BWJ5	Splicing factor 3B subunit 5 OS=Homo sapiens GN=SF3B5 PE=1 SV=1 - [SF3B5_HUMAN]
Q99873	Protein arginine N-methyltransferase 1 OS=Homo sapiens GN=PRMT1 PE=1 SV=2 - [ANM1_HUMAN]
P50991	T-complex protein 1 subunit delta OS=Homo sapiens GN=CCT4 PE=1 SV=4 - [TCPD_HUMAN]
P62714	Serine/threonine-protein phosphatase 2A catalytic subunit beta isoform OS=Homo sapiens GN=PPP2CB PE=1 SV=1 - [PP2AB_HUMAN]
P67775	Serine/threonine-protein phosphatase 2A catalytic subunit alpha isoform OS=Homo sapiens GN=PPP2CA PE=1 SV=1 - [PP2AA_HUMAN]
P60900	Proteasome subunit alpha type-6 OS=Homo sapiens GN=PSMA6 PE=1 SV=1 - [PSA6_HUMAN]
P25786	Proteasome subunit alpha type-1 OS=Homo sapiens GN=PSMA1 PE=1 SV=1 - [PSA1_HUMAN]
Q92743	Serine protease HTRA1 OS=Homo sapiens GN=HTRA1 PE=1 SV=1 - [HTRA1_HUMAN]
P02751	Fibronectin OS=Homo sapiens GN=FN1 PE=1 SV=4 - [FINC_HUMAN]
P53621	Coatomer subunit alpha OS=Homo sapiens GN=COPA PE=1 SV=2 - [COPA_HUMAN]
P62304	Small nuclear ribonucleoprotein E OS=Homo sapiens GN=SNRPE PE=1 SV=1 - [RUXE_HUMAN]
Q05048	Cleavage stimulation factor subunit 1 OS=Homo sapiens GN=CSTF1 PE=1 SV=1 - [CSTF1_HUMAN]
P28066	Proteasome subunit alpha type-5 OS=Homo sapiens GN=PSMA5 PE=1 SV=3 - [PSA5_HUMAN]
P55072	Transitional endoplasmic reticulum ATPase OS=Homo sapiens GN=VCP PE=1 SV=4 - [TERA_HUMAN]
P04406	Glyceraldehyde-3-phosphate dehydrogenase OS=Homo sapiens GN=GAPDH PE=1 SV=3 - [G3P_HUMAN]
P06493	Cyclin-dependent kinase 1 OS=Homo sapiens GN=CDK1 PE=1 SV=3 - [CDK1_HUMAN]
P30086	Phosphatidylethanolamine-binding protein 1 OS=Homo sapiens GN=PEBP1 PE=1 SV=3 - [PEBP1_HUMAN]
P31939	Bifunctional purine biosynthesis protein PURH OS=Homo sapiens GN=ATIC PE=1 SV=3 - [PUR9_HUMAN]
P23396	40S ribosomal protein S3 OS=Homo sapiens GN=RPS3 PE=1 SV=2 - [RS3_HUMAN]
Q9Y315	Deoxyribose-phosphate aldolase OS=Homo sapiens GN=DERA PE=1 SV=2 - [DEOC_HUMAN]
P07108	Acyl-CoA-binding protein OS=Homo sapiens GN=DBI PE=1 SV=2 - [ACBP_HUMAN]
P43686	26S protease regulatory subunit 6B OS=Homo sapiens GN=PSMC4 PE=1 SV=2 - [PRS6B_HUMAN]
Q06830	Peroxiredoxin-1 OS=Homo sapiens GN=PRDX1 PE=1 SV=1 - [PRDX1_HUMAN]
P01834	Ig kappa chain C region OS=Homo sapiens GN=IGKC PE=1 SV=1 - [IGKC_HUMAN]
P62136	Serine/threonine-protein phosphatase PP1-alpha catalytic subunit OS=Homo sapiens GN=PPP1CA PE=1 SV=1 - [PP1A_HUMAN]
P49327	Fatty acid synthase OS=Homo sapiens GN=FASN PE=1 SV=3 - [FAS_HUMAN]
Q9P000	COMM domain-containing protein 9 OS=Homo sapiens GN=COMMD9 PE=1 SV=2 - [COMD9_HUMAN]
P62888	60S ribosomal protein L30 OS=Homo sapiens GN=RPL30 PE=1 SV=2 - [RL30_HUMAN]
Q9HCN8	Stromal cell-derived factor 2-like protein 1 OS=Homo sapiens GN=SDF2L1 PE=1 SV=2 - [SDF2L_HUMAN]
P63241	Eukaryotic translation initiation factor 5A-1 OS=Homo sapiens GN=EIF5A PE=1 SV=2 - [IF5A1_HUMAN]
P62829	60S ribosomal protein L23 OS=Homo sapiens GN=RPL23 PE=1 SV=1 - [RL23_HUMAN]
P53396	ATP-citrate synthase OS=Homo sapiens GN=ACLY PE=1 SV=3 - [ACLY HUMAN]
O00231	26S proteasome non-ATPase regulatory subunit 11 OS=Homo sapiens GN=PSMD11 PE=1 SV=3 - [PSD11_HUMAN]
Q92747	Actin-related protein 2/3 complex subunit 1A OS=Homo sapiens GN=ARPC1A PE=1 SV=2 - [ARC1A_HUMAN]
P61964	WD repeat-containing protein 5 OS=Homo sapiens GN=WDR5 PE=1 SV=1 - [WDR5_HUMAN]
P21333	Filamin-A OS=Homo sapiens GN=FLNA PE=1 SV=4 - [FLNA_HUMAN]
P62805	Histone H4 OS=Homo sapiens GN=HIST1H4A PE=1 SV=2 - [H4_HUMAN]
P32119	Peroxiredoxin-2 OS=Homo sapiens GN=PRDX2 PE=1 SV=5 - [PRDX2_HUMAN]
P62195	26S protease regulatory subunit 8 OS=Homo sapiens GN=PSMC5 PE=1 SV=1 - [PRS8_HUMAN]
Q9BRG1	Vacuolar protein-sorting-associated protein 25 OS=Homo sapiens GN=VPS25 PE=1 SV=1 - [VPS25_HUMAN]
P49368	T-complex protein 1 subunit gamma OS=Homo sapiens GN=CCT3 PE=1 SV=4 - [TCPG_HUMAN]
Q00610	Clathrin heavy chain 1 OS=Homo sapiens GN=CLTC PE=1 SV=5 - [CLH1_HUMAN]
P50151	Guanine nucleotide-binding protein G(I)/G(S)/G(O) subunit gamma-10 OS=Homo sapiens GN=GNG10 PE=1 SV=1 - [GBG10_HUMAN]
P02794	Ferritin heavy chain OS=Homo sapiens GN=FTH1 PE=1 SV=2 - [FRIH HUMAN]
P62820	Ras-related protein Rab-1A OS=Homo sapiens GN=RAB1A PE=1 SV=3 - [RAB1A_HUMAN]
P78371	T-complex protein 1 subunit beta OS=Homo sapiens GN=CCT2 PE=1 SV=4 - [TCPB_HUMAN]
Q9GZS3	WD repeat-containing protein 61 OS=Homo sapiens GN=WDR61 PE=1 SV=1 - [WDR61_HUMAN]
P19784	Casein kinase II subunit alpha' OS=Homo sapiens GN=CSNK2A2 PE=1 SV=1 - [CSK22_HUMAN]
P51148	Ras-related protein Rab-5C OS=Homo sapiens GN=RAB5C PE=1 SV=2 - [RAB5C_HUMAN]
Q02809	Procollagen-lysine,2-oxoglutarate 5-dioxygenase 1 OS=Homo sapiens GN=PLOD1 PE=1 SV=2 - [PLOD1_HUMAN]
O75934	Pre-mRNA-splicing factor SPF27 OS=Homo sapiens GN=BCAS2 PE=1 SV=1 - [SPF27_HUMAN]
P27708	CAD protein OS=Homo sapiens GN=CAD PE=1 SV=3 - [PYR1_HUMAN]
Q12905	Interleukin enhancer-binding factor 2 OS=Homo sapiens GN=ILF2 PE=1 SV=2 - [ILF2_HUMAN]
Q9Y265	RuvB-like 1 OS=Homo sapiens GN=RUVBL1 PE=1 SV=1 - [RUVB1_HUMAN]
Q9Y678	Coatomer subunit gamma-1 OS=Homo sapiens GN=COPG1 PE=1 SV=1 - [COPG1_HUMAN]
Q9H4G4	Golgi-associated plant pathogenesis-related protein 1 OS=Homo sapiens GN=GLIPR2 PE=1 SV=3 - [GAPR1_HUMAN]
Q9Y5K6	CD2-associated protein OS=Homo sapiens GN=CD2AP PE=1 SV=1 - [CD2AP_HUMAN]
P61106	Ras-related protein Rab-14 OS=Homo sapiens GN=RAB14 PE=1 SV=4 - [RAB14_HUMAN]
P17987	T-complex protein 1 subunit alpha OS=Homo sapiens GN=TCP1 PE=1 SV=1 - [TCPA_HUMAN]
P62701	40S ribosomal protein S4, X isoform OS=Homo sapiens GN=RPS4X PE=1 SV=2 - [RS4X_HUMAN]
Q9BQ67	Glutamate-rich WD repeat-containing protein 1 OS=Homo sapiens GN=GRWD1 PE=1 SV=1 - [GRWD1_HUMAN]
P62140	Serine/threonine-protein phosphatase PP1-beta catalytic subunit OS=Homo sapiens GN=PPP1CB PE=1 SV=3 - [PP1B_HUMAN]

P62942	Peptidyl-prolyl cis-trans isomerase FKBP1A OS=Homo sapiens GN=FKBP1A PE=1 SV=2 - [FKB1A_HUMAN]
P17980	26S protease regulatory subunit 6A OS=Homo sapiens GN=PSMC3 PE=1 SV=3 - [PRS6A_HUMAN]
P48643	T-complex protein 1 subunit epsilon OS=Homo sapiens GN=CCT5 PE=1 SV=1 - [TCPE_HUMAN]
P28074	Proteasome subunit beta type-5 OS=Homo sapiens GN=PSMB5 PE=1 SV=3 - [PSB5_HUMAN]
P18085	ADP-ribosylation factor 4 OS=Homo sapiens GN=ARF4 PE=1 SV=3 - [ARF4_HUMAN]
P05388	60S acidic ribosomal protein P0 OS=Homo sapiens GN=RPLP0 PE=1 SV=1 - [RLA0_HUMAN]
Q8TB73	Protein NDNF OS=Homo sapiens GN=NDNF PE=2 SV=2 - [NDNF_HUMAN]
P25789	Proteasome subunit alpha type-4 OS=Homo sapiens GN=PSMA4 PE=1 SV=1 - [PSA4_HUMAN]
P00441	Superoxide dismutase [Cu-Zn] OS=Homo sapiens GN=SOD1 PE=1 SV=2 - [SODC_HUMAN]
Q9H0U4	Ras-related protein Rab-1B OS=Homo sapiens GN=RAB1B PE=1 SV=1 - [RAB1B_HUMAN]
P84095	Rho-related GTP-binding protein RhoG OS=Homo sapiens GN=RHOG PE=1 SV=1 - [RHOG_HUMAN]
P62333	26S protease regulatory subunit 10B OS=Homo sapiens GN=PSMC6 PE=1 SV=1 - [PRS10_HUMAN]
P08107	Heat shock 70 kDa protein 1A/1B OS=Homo sapiens GN=HSPA1A PE=1 SV=5 - [HSP71_HUMAN]
P61026	Ras-related protein Rab-10 OS=Homo sapiens GN=RAB10 PE=1 SV=1 - [RAB10_HUMAN]
P11940	Polyadenylate-binding protein 1 OS=Homo sapiens GN=PABPC1 PE=1 SV=2 - [PABP1_HUMAN]
Q15126	Phosphomevalonate kinase OS=Homo sapiens GN=PMVK PE=1 SV=3 - [PMVK_HUMAN]
P68036	Ubiquitin-conjugating enzyme E2 L3 OS=Homo sapiens GN=UBE2L3 PE=1 SV=1 - [UB2L3_HUMAN]
P22061	Protein-L-isoaspartate(D-aspartate) O-methyltransferase OS=Homo sapiens GN=PCMT1 PE=1 SV=4 - [PIMT_HUMAN]
O15144	Actin-related protein 2/3 complex subunit 2 OS=Homo sapiens GN=ARPC2 PE=1 SV=1 - [ARPC2_HUMAN]
Q92626	Peroxidasin homolog OS=Homo sapiens GN=PXDN PE=1 SV=2 - [PXDN_HUMAN]
P61006	Ras-related protein Rab-8A OS=Homo sapiens GN=RAB8A PE=1 SV=1 - [RAB8A_HUMAN]
P23142	Fibulin-1 OS=Homo sapiens GN=FBLN1 PE=1 SV=4 - [FBLN1_HUMAN]
P34897	Serine hydroxymethyltransferase, mitochondrial OS=Homo sapiens GN=SHMT2 PE=1 SV=3 - [GLYM_HUMAN]
O14818	Proteasome subunit alpha type-7 OS=Homo sapiens GN=PSMA7 PE=1 SV=1 - [PSA7_HUMAN]
P25787	Proteasome subunit alpha type-2 OS=Homo sapiens GN=PSMA2 PE=1 SV=2 - [PSA2_HUMAN]
P00736	Complement C1r subcomponent OS=Homo sapiens GN=C1R PE=1 SV=2 - [C1R_HUMAN]
P00558	Phosphoglycerate kinase 1 OS=Homo sapiens GN=PGK1 PE=1 SV=3 - [PGK1_HUMAN]
P60953	Cell division control protein 42 homolog OS=Homo sapiens GN=CDC42 PE=1 SV=2 - [CDC42_HUMAN]
P05386	60S acidic ribosomal protein P1 OS=Homo sapiens GN=RPLP1 PE=1 SV=1 - [RLA1_HUMAN]
P11142	Heat shock cognate 71 kDa protein OS=Homo sapiens GN=HSPA8 PE=1 SV=1 - [HSP7C_HUMAN]
P29692	Elongation factor 1-delta OS=Homo sapiens GN=EEF1D PE=1 SV=5 - [EF1D_HUMAN]
O15372	Eukaryotic translation initiation factor 3 subunit H OS=Homo sapiens GN=EIF3H PE=1 SV=1 - [EIF3H_HUMAN]
P54136	Arginine--tRNA ligase, cytoplasmic OS=Homo sapiens GN=RARS PE=1 SV=2 - [SYRC_HUMAN]
P23258	Tubulin gamma-1 chain OS=Homo sapiens GN=TUBG1 PE=1 SV=2 - [TBG1_HUMAN]
P19105	Myosin regulatory light chain 12A OS=Homo sapiens GN=MYL12A PE=1 SV=2 - [ML12A_HUMAN]
Q92499	ATP-dependent RNA helicase DDX1 OS=Homo sapiens GN=DDX1 PE=1 SV=2 - [DDX1_HUMAN]
Q6UXN9	WD repeat-containing protein 82 OS=Homo sapiens GN=WDR82 PE=1 SV=1 - [WDR82_HUMAN]
Q9BT78	COP9 signalosome complex subunit 4 OS=Homo sapiens GN=COPS4 PE=1 SV=1 - [CSN4_HUMAN]
Q9Y224	UPF0568 protein C14orf166 OS=Homo sapiens GN=C14orf166 PE=1 SV=1 - [CN166_HUMAN]
P62191	26S protease regulatory subunit 4 OS=Homo sapiens GN=PSMC1 PE=1 SV=1 - [PRS4_HUMAN]
Q9HB71	Calcyclin-binding protein OS=Homo sapiens GN=CACYBP PE=1 SV=2 - [CYBP_HUMAN]
P07741	Adenine phosphoribosyltransferase OS=Homo sapiens GN=APRT PE=1 SV=2 - [APT_HUMAN]
P14868	Aspartate--tRNA ligase, cytoplasmic OS=Homo sapiens GN=DARS PE=1 SV=2 - [SYDC_HUMAN]
Q96EY5	Multivesicular body subunit 12A OS=Homo sapiens GN=MVB12A PE=1 SV=1 - [MB12A_HUMAN]
Q16576	Histone-binding protein RBBP7 OS=Homo sapiens GN=RBBP7 PE=1 SV=1 - [RBBP7_HUMAN]
P35625	Metalloproteinase inhibitor 3 OS=Homo sapiens GN=TIMP3 PE=1 SV=2 - [TIMP3_HUMAN]
P13693	Translationally-controlled tumor protein OS=Homo sapiens GN=TPT1 PE=1 SV=1 - [TCTP_HUMAN]
P08865	40S ribosomal protein SA OS=Homo sapiens GN=RPSA PE=1 SV=4 - [RSSA_HUMAN]
Q9Y281	Cofilin-2 OS=Homo sapiens GN=CFL2 PE=1 SV=1 - [COF2_HUMAN]
P22234	Multifunctional protein ADE2 OS=Homo sapiens GN=PAICS PE=1 SV=3 - [PUR6_HUMAN]
O43405	Cochlin OS=Homo sapiens GN=COCH PE=1 SV=1 - [COCH_HUMAN]
P05198	Eukaryotic translation initiation factor 2 subunit 1 OS=Homo sapiens GN=EIF2S1 PE=1 SV=3 - [IF2A_HUMAN]
Q15286	Ras-related protein Rab-35 OS=Homo sapiens GN=RAB35 PE=1 SV=1 - [RAB35_HUMAN]
O00303	Eukaryotic translation initiation factor 3 subunit F OS=Homo sapiens GN=EIF3F PE=1 SV=1 - [EIF3F_HUMAN]
Q9UN37	Vacuolar protein sorting-associated protein 4A OS=Homo sapiens GN=VPS4A PE=1 SV=1 - [VPS4A_HUMAN]
P43034	Platelet-activating factor acetylhydrolase IB subunit alpha OS=Homo sapiens GN=PAFAH1B1 PE=1 SV=2 - [LIS1_HUMAN]
O75531	Barrier-to-autointegration factor OS=Homo sapiens GN=BANF1 PE=1 SV=1 - [BAF_HUMAN]
Q15008	26S proteasome non-ATPase regulatory subunit 6 OS=Homo sapiens GN=PSMD6 PE=1 SV=1 - [PSMD6_HUMAN]
P00750	Tissue-type plasminogen activator OS=Homo sapiens GN=PLAT PE=1 SV=1 - [TPA_HUMAN]
P61313	60S ribosomal protein L15 OS=Homo sapiens GN=RPL15 PE=1 SV=2 - [RL15_HUMAN]
O00487	26S proteasome non-ATPase regulatory subunit 14 OS=Homo sapiens GN=PSMD14 PE=1 SV=1 - [PSDE_HUMAN]
Q7L5N1	COP9 signalosome complex subunit 6 OS=Homo sapiens GN=COPS6 PE=1 SV=1 - [CSN6_HUMAN]
P49247	Ribose-5-phosphate isomerase OS=Homo sapiens GN=RPIA PE=1 SV=3 - [RPIA_HUMAN]
Q13162	Peroxiredoxin-4 OS=Homo sapiens GN=PRDX4 PE=1 SV=1 - [PRDX4_HUMAN]
Q09028	Histone-binding protein RBBP4 OS=Homo sapiens GN=RBBP4 PE=1 SV=3 - [RBBP4_HUMAN]
Q15113	Procollagen C-endopeptidase enhancer 1 OS=Homo sapiens GN=PCOLCE PE=1 SV=2 - [PCOC1_HUMAN]
P25398	40S ribosomal protein S12 OS=Homo sapiens GN=RPS12 PE=1 SV=3 - [RS12_HUMAN]
Q9NQT4	Exosome complex component RRP46 OS=Homo sapiens GN=EXOSC5 PE=1 SV=1 - [EXOS5_HUMAN]
P60842	Eukaryotic initiation factor 4A-I OS=Homo sapiens GN=EIF4A1 PE=1 SV=1 - [IF4A1_HUMAN]
Q9Y230	RuvB-like 2 OS=Homo sapiens GN=RUVBL2 PE=1 SV=3 - [RUVB2_HUMAN]
P28070	Proteasome subunit beta type-4 OS=Homo sapiens GN=PSMB4 PE=1 SV=4 - [PSB4_HUMAN]
P61204	ADP-ribosylation factor 3 OS=Homo sapiens GN=ARF3 PE=1 SV=2 - [ARF3_HUMAN]
P62873	Guanine nucleotide-binding protein G(I)/G(S)/G(T) subunit beta-1 OS=Homo sapiens GN=GNB1 PE=1 SV=3 - [GBB1_HUMAN]
P62879	Guanine nucleotide-binding protein G(I)/G(S)/G(T) subunit beta-2 OS=Homo sapiens GN=GNB2 PE=1 SV=3 - [GBB2_HUMAN]
P62249	40S ribosomal protein S16 OS=Homo sapiens GN=RPS16 PE=1 SV=2 - [RS16_HUMAN]
P52292	Importin subunit alpha-1 OS=Homo sapiens GN=KPNA2 PE=1 SV=1 - [IMA1_HUMAN]
P07093	Glia-derived nexin OS=Homo sapiens GN=SERPINE2 PE=1 SV=1 - [GDN_HUMAN]
P01111	GTPase NRas OS=Homo sapiens GN=NRAS PE=1 SV=1 - [RASN_HUMAN]
P53618	Coatomer subunit beta OS=Homo sapiens GN=COPB1 PE=1 SV=3 - [COPB_HUMAN]
P0C0S5	Histone H2A.Z OS=Homo sapiens GN=H2AFZ PE=1 SV=2 - [H2AZ_HUMAN]
P33992	DNA replication licensing factor MCM5 OS=Homo sapiens GN=MCM5 PE=1 SV=5 - [MCM5_HUMAN]
P61247	40S ribosomal protein S3a OS=Homo sapiens GN=RPS3A PE=1 SV=2 - [RS3A_HUMAN]
Q9HCE1	Putative helicase MOV-10 OS=Homo sapiens GN=MOV10 PE=1 SV=2 - [MOV10_HUMAN]
Q15369	Transcription elongation factor B polypeptide 1 OS=Homo sapiens GN=TCEB1 PE=1 SV=1 - [ELOC_HUMAN]
Q08380	Galectin-3-binding protein OS=Homo sapiens GN=LGALS3BP PE=1 SV=1 - [LG3BP_HUMAN]
P16152	Carbonyl reductase [NADPH] 1 OS=Homo sapiens GN=CBR1 PE=1 SV=3 - [CBR1_HUMAN]
P37802	Transgelin-2 OS=Homo sapiens GN=TAGLN2 PE=1 SV=3 - [TAGL2_HUMAN]
Q969P0	Immunoglobulin superfamily member 8 OS=Homo sapiens GN=IGSF8 PE=1 SV=1 - [IGSF8_HUMAN]
P01033	Metalloproteinase inhibitor 1 OS=Homo sapiens GN=TIMP1 PE=1 SV=1 - [TIMP1_HUMAN]
P21281	V-type proton ATPase subunit B, brain isoform OS=Homo sapiens GN=ATP6V1B2 PE=1 SV=3 - [VATB2_HUMAN]
P61353	60S ribosomal protein L27 OS=Homo sapiens GN=RPL27 PE=1 SV=2 - [RL27_HUMAN]
Q15019	Septin-2 OS=Homo sapiens GN=SEPT2 PE=1 SV=1 - [SEPT2_HUMAN]
P33991	DNA replication licensing factor MCM4 OS=Homo sapiens GN=MCM4 PE=1 SV=5 - [MCM4_HUMAN]
P08758	Annexin A5 OS=Homo sapiens GN=ANXA5 PE=1 SV=2 - [ANXA5_HUMAN]
P13639	Elongation factor 2 OS=Homo sapiens GN=EEF2 PE=1 SV=4 - [EF2_HUMAN]
Q8IUL8	Cartilage intermediate layer protein 2 OS=Homo sapiens GN=CILP2 PE=2 SV=2 - [CILP2_HUMAN]
P22102	Trifunctional purine biosynthetic protein adenosine-3 OS=Homo sapiens GN=GART PE=1 SV=1 - [PUR2_HUMAN]
Q9BXS5	AP-1 complex subunit mu-1 OS=Homo sapiens GN=AP1M1 PE=1 SV=3 - [AP1M1_HUMAN]
Q16531	DNA damage-binding protein 1 OS=Homo sapiens GN=DDB1 PE=1 SV=1 - [DDB1_HUMAN]
P10599	Thioredoxin OS=Homo sapiens GN=TXN PE=1 SV=3 - [THIO_HUMAN]
O75340	Programmed cell death protein 6 OS=Homo sapiens GN=PDCD6 PE=1 SV=1 - [PDCD6_HUMAN]
P24941	Cyclin-dependent kinase 2 OS=Homo sapiens GN=CDK2 PE=1 SV=2 - [CDK2_HUMAN]
P62913	60S ribosomal protein L11 OS=Homo sapiens GN=RPL11 PE=1 SV=2 - [RL11_HUMAN]
Q12906	Interleukin enhancer-binding factor 3 OS=Homo sapiens GN=ILF3 PE=1 SV=3 - [ILF3_HUMAN]
P30041	Peroxiredoxin-6 OS=Homo sapiens GN=PRDX6 PE=1 SV=3 - [PRDX6_HUMAN]
P46777	60S ribosomal protein L5 OS=Homo sapiens GN=RPL5 PE=1 SV=3 - [RL5_HUMAN]
O43684	Mitotic checkpoint protein BUB3 OS=Homo sapiens GN=BUB3 PE=1 SV=1 - [BUB3_HUMAN]
P11047	Laminin subunit gamma-1 OS=Homo sapiens GN=LAMC1 PE=1 SV=3 - [LAMC1_HUMAN]
P78539	Sushi repeat-containing protein SRPX OS=Homo sapiens GN=SRPX PE=1 SV=1 - [SRPX_HUMAN]
P04792	Heat shock protein beta-1 OS=Homo sapiens GN=HSPB1 PE=1 SV=2 - [HSPB1_HUMAN]
Q93009	Ubiquitin carboxyl-terminal hydrolase 7 OS=Homo sapiens GN=USP7 PE=1 SV=2 - [UBP7_HUMAN]
P08238	Heat shock protein HSP 90-beta OS=Homo sapiens GN=HSP90AB1 PE=1 SV=4 - [HS90B_HUMAN]
Q99417	C-Myc-binding protein OS=Homo sapiens GN=MYCBP PE=1 SV=3 - [MYCBP_HUMAN]
P27348	14-3-3 protein theta OS=Homo sapiens GN=YWHAQ PE=1 SV=1 - [1433T_HUMAN]
Q9UBQ5	Eukaryotic translation initiation factor 3 subunit K OS=Homo sapiens GN=EIF3K PE=1 SV=1 - [EIF3K_HUMAN]

Q7L2H7	Eukaryotic translation initiation factor 3 subunit M OS=Homo sapiens GN=EIF3M PE=1 SV=1 - [EIF3M_HUMAN]
P22392	Nucleoside diphosphate kinase B OS=Homo sapiens GN=NME2 PE=1 SV=1 - [NDKB_HUMAN]
P98160	Basement membrane-specific heparan sulfate proteoglycan core protein OS=Homo sapiens GN=HSPG2 PE=1 SV=4 - [PGBM_HUMAN]
Q9BRA2	Thioredoxin domain-containing protein 17 OS=Homo sapiens GN=TXNDC17 PE=1 SV=1 - [TXD17_HUMAN]
Q15181	Inorganic pyrophosphatase OS=Homo sapiens GN=PPA1 PE=1 SV=2 - [IPYR_HUMAN]
P11802	Cyclin-dependent kinase 4 OS=Homo sapiens GN=CDK4 PE=1 SV=2 - [CDK4_HUMAN]
Q9UBB4	Ataxin-10 OS=Homo sapiens GN=ATXN10 PE=1 SV=1 - [ATX10_HUMAN]
Q9Y3F4	Serine-threonine kinase receptor-associated protein OS=Homo sapiens GN=STRAP PE=1 SV=1 - [STRAP_HUMAN]
P07814	Bifunctional glutamate/proline--tRNA ligase OS=Homo sapiens GN=EPRS PE=1 SV=5 - [SYEP_HUMAN]
Q14204	Cytoplasmic dynein 1 heavy chain 1 OS=Homo sapiens GN=DYNC1H1 PE=1 SV=5 - [DYHC1_HUMAN]
P61513	60S ribosomal protein L37a OS=Homo sapiens GN=RPL37A PE=1 SV=2 - [RL37A_HUMAN]
P46782	40S ribosomal protein S5 OS=Homo sapiens GN=RPS5 PE=1 SV=4 - [RS5_HUMAN]
Q9Y2T2	AP-3 complex subunit mu-1 OS=Homo sapiens GN=AP3M1 PE=1 SV=1 - [AP3M1_HUMAN]
Q8WUD1	Ras-related protein Rab-2B OS=Homo sapiens GN=RAB2B PE=1 SV=1 - [RAB2B_HUMAN]
Q7RTV0	PHD finger-like domain-containing protein 5A OS=Homo sapiens GN=PHF5A PE=1 SV=1 - [PHF5A_HUMAN]
Q00535	Cyclin-dependent-like kinase 5 OS=Homo sapiens GN=CDK5 PE=1 SV=3 - [CDK5_HUMAN]
P26639	Threonine--tRNA ligase, cytoplasmic OS=Homo sapiens GN=TARS PE=1 SV=3 - [SYTC_HUMAN]
Q13561	Dynactin subunit 2 OS=Homo sapiens GN=DCTN2 PE=1 SV=4 - [DCTN2_HUMAN]
P23526	Adenosylhomocysteinase OS=Homo sapiens GN=AHCY PE=1 SV=4 - [SAHH_HUMAN]
Q13509	Tubulin beta-3 chain OS=Homo sapiens GN=TUBB3 PE=1 SV=2 - [TBB3_HUMAN]
Q9UJW0	Dynactin subunit 4 OS=Homo sapiens GN=DCTN4 PE=1 SV=1 - [DCTN4_HUMAN]
P68400	Casein kinase II subunit alpha OS=Homo sapiens GN=CSNK2A1 PE=1 SV=1 - [CSK21_HUMAN]
O43242	26S proteasome non-ATPase regulatory subunit 3 OS=Homo sapiens GN=PSMD3 PE=1 SV=2 - [PSMD3_HUMAN]
P52907	F-actin-capping protein subunit alpha-1 OS=Homo sapiens GN=CAPZA1 PE=1 SV=3 - [CAZA1_HUMAN]
O75083	WD repeat-containing protein 1 OS=Homo sapiens GN=WDR1 PE=1 SV=4 - [WDR1_HUMAN]
P28072	Proteasome subunit beta type-6 OS=Homo sapiens GN=PSMB6 PE=1 SV=4 - [PSB6_HUMAN]
Q8IZ52	Chondroitin sulfate synthase 2 OS=Homo sapiens GN=CHPF PE=1 SV=2 - [CHSS2_HUMAN]
P30626	Sorcin OS=Homo sapiens GN=SRI PE=1 SV=1 - [SORCN_HUMAN]
Q99536	Synaptic vesicle membrane protein VAT-1 homolog OS=Homo sapiens GN=VAT1 PE=1 SV=2 - [VAT1_HUMAN]
P11586	C-1-tetrahydrofolate synthase, cytoplasmic OS=Homo sapiens GN=MTHFD1 PE=1 SV=3 - [C1TC_HUMAN]
P20618	Proteasome subunit beta type-1 OS=Homo sapiens GN=PSMB1 PE=1 SV=2 - [PSB1_HUMAN]
Q7KZF4	Staphylococcal nuclease domain-containing protein 1 OS=Homo sapiens GN=SND1 PE=1 SV=1 - [SND1_HUMAN]
P63267	Actin, gamma-enteric smooth muscle OS=Homo sapiens GN=ACTG2 PE=1 SV=1 - [ACTH_HUMAN]
Q9Y6G9	Cytoplasmic dynein 1 light intermediate chain 1 OS=Homo sapiens GN=DYNC1LI1 PE=1 SV=3 - [DC1L1_HUMAN]
P07900	Heat shock protein HSP 90-alpha OS=Homo sapiens GN=HSP90AA1 PE=1 SV=5 - [HS90A_HUMAN]
P41567	Eukaryotic translation initiation factor 1 OS=Homo sapiens GN=EIF1 PE=1 SV=1 - [EIF1_HUMAN]
P19388	DNA-directed RNA polymerases I, II, and III subunit RPABC1 OS=Homo sapiens GN=POLR2E PE=1 SV=4 - [RPAB1_HUMAN]
P63010	AP-2 complex subunit beta OS=Homo sapiens GN=AP2B1 PE=1 SV=1 - [AP2B1_HUMAN]
Q86YQ8	Copine-8 OS=Homo sapiens GN=CPNE8 PE=1 SV=2 - [CPNE8_HUMAN]
Q14166	Tubulin--tyrosine ligase-like protein 12 OS=Homo sapiens GN=TTLL12 PE=1 SV=2 - [TTL12_HUMAN]
P06576	ATP synthase subunit beta, mitochondrial OS=Homo sapiens GN=ATP5B PE=1 SV=3 - [ATPB_HUMAN]
P06733	Alpha-enolase OS=Homo sapiens GN=ENO1 PE=1 SV=2 - [ENOA_HUMAN]
Q86X83	COMM domain-containing protein 2 OS=Homo sapiens GN=COMMD2 PE=1 SV=2 - [COMD2_HUMAN]
O75436	Vacuolar protein sorting-associated protein 26A OS=Homo sapiens GN=VPS26A PE=1 SV=2 - [VP26A_HUMAN]
P01112	GTPase HRas OS=Homo sapiens GN=HRAS PE=1 SV=1 - [RASH_HUMAN]
Q9UFG5	UPF0449 protein C19orf25 OS=Homo sapiens GN=C19orf25 PE=1 SV=2 - [CS025_HUMAN]
P41252	Isoleucine--tRNA ligase, cytoplasmic OS=Homo sapiens GN=IARS PE=1 SV=2 - [SYIC_HUMAN]
P21796	Voltage-dependent anion-selective channel protein 1 OS=Homo sapiens GN=VDAC1 PE=1 SV=2 - [VDAC1_HUMAN]
Q14112	Nidogen-2 OS=Homo sapiens GN=NID2 PE=1 SV=3 - [NID2_HUMAN]
P26038	Moesin OS=Homo sapiens GN=MSN PE=1 SV=3 - [MOES_HUMAN]
P39019	40S ribosomal protein S19 OS=Homo sapiens GN=RPS19 PE=1 SV=2 - [RS19_HUMAN]
P62266	40S ribosomal protein S23 OS=Homo sapiens GN=RPS23 PE=1 SV=3 - [RS23_HUMAN]
Q93063	Exostosin-2 OS=Homo sapiens GN=EXT2 PE=1 SV=1 - [EXT2_HUMAN]
P54577	Tyrosine--tRNA ligase, cytoplasmic OS=Homo sapiens GN=YARS PE=1 SV=4 - [SYYC_HUMAN]
P62993	Growth factor receptor-bound protein 2 OS=Homo sapiens GN=GRB2 PE=1 SV=1 - [GRB2_HUMAN]
Q14232	Translation initiation factor eIF-2B subunit alpha OS=Homo sapiens GN=EIF2B1 PE=1 SV=1 - [EI2BA_HUMAN]
P07355	Annexin A2 OS=Homo sapiens GN=ANXA2 PE=1 SV=2 - [ANXA2_HUMAN]
P61163	Alpha-centractin OS=Homo sapiens GN=ACTR1A PE=1 SV=1 - [ACTZ_HUMAN]
Q15366	Poly(rC)-binding protein 2 OS=Homo sapiens GN=PCBP2 PE=1 SV=1 - [PCBP2_HUMAN]
P51665	26S proteasome non-ATPase regulatory subunit 7 OS=Homo sapiens GN=PSMD7 PE=1 SV=2 - [PSMD7_HUMAN]
P63000	Ras-related C3 botulinum toxin substrate 1 OS=Homo sapiens GN=RAC1 PE=1 SV=1 - [RAC1_HUMAN]
Q9UKZ9	Procollagen C-endopeptidase enhancer 2 OS=Homo sapiens GN=PCOLCE2 PE=1 SV=1 - [PCOC2_HUMAN]
O14579	Coatomer subunit epsilon OS=Homo sapiens GN=COPE PE=1 SV=3 - [COPE_HUMAN]
Q99436	Proteasome subunit beta type-7 OS=Homo sapiens GN=PSMB7 PE=1 SV=1 - [PSB7_HUMAN]
P35556	Fibrillin-2 OS=Homo sapiens GN=FBN2 PE=1 SV=3 - [FBN2_HUMAN]
P22314	Ubiquitin-like modifier-activating enzyme 1 OS=Homo sapiens GN=UBA1 PE=1 SV=3 - [UBA1_HUMAN]
Q92900	Regulator of nonsense transcripts 1 OS=Homo sapiens GN=UPF1 PE=1 SV=2 - [RENT1_HUMAN]
O00299	Chloride intracellular channel protein 1 OS=Homo sapiens GN=CLIC1 PE=1 SV=4 - [CLIC1_HUMAN]
P42771	Cyclin-dependent kinase inhibitor 2A OS=Homo sapiens GN=CDKN2A PE=1 SV=2 - [CDN2A_HUMAN]
O43809	Cleavage and polyadenylation specificity factor subunit 5 OS=Homo sapiens GN=NUDT21 PE=1 SV=1 - [CPSF5_HUMAN]
Q9Y3B4	Splicing factor 3B subunit 6 OS=Homo sapiens GN=SF3B6 PE=1 SV=1 - [SF3B6_HUMAN]
P10809	60 kDa heat shock protein, mitochondrial OS=Homo sapiens GN=HSPD1 PE=1 SV=2 - [CH60_HUMAN]
O00161	Synaptosomal-associated protein 23 OS=Homo sapiens GN=SNAP23 PE=1 SV=1 - [SNP23_HUMAN]
Q13813	Spectrin alpha chain, non-erythrocytic 1 OS=Homo sapiens GN=SPTAN1 PE=1 SV=3 - [SPTN1_HUMAN]
Q15393	Splicing factor 3B subunit 3 OS=Homo sapiens GN=SF3B3 PE=1 SV=4 - [SF3B3_HUMAN]
O00148	ATP-dependent RNA helicase DDX39A OS=Homo sapiens GN=DDX39A PE=1 SV=2 - [DX39A_HUMAN]
P46063	ATP-dependent DNA helicase Q1 OS=Homo sapiens GN=RECQL PE=1 SV=3 - [RECQ1_HUMAN]
Q9H223	EH domain-containing protein 4 OS=Homo sapiens GN=EHD4 PE=1 SV=1 - [EHD4_HUMAN]
P08134	Rho-related GTP-binding protein RhoC OS=Homo sapiens GN=RHOC PE=1 SV=1 - [RHOC_HUMAN]
P14854	Cytochrome c oxidase subunit 6B1 OS=Homo sapiens GN=COX6B1 PE=1 SV=2 - [CX6B1_HUMAN]
Q9NZN3	EH domain-containing protein 3 OS=Homo sapiens GN=EHD3 PE=1 SV=2 - [EHD3_HUMAN]
P62158	Calmodulin OS=Homo sapiens GN=CALM1 PE=1 SV=2 - [CALM_HUMAN]
P39023	60S ribosomal protein L3 OS=Homo sapiens GN=RPL3 PE=1 SV=2 - [RL3_HUMAN]
Q15029	116 kDa U5 small nuclear ribonucleoprotein component OS=Homo sapiens GN=EFTUD2 PE=1 SV=1 - [U5S1_HUMAN]
P59998	Actin-related protein 2/3 complex subunit 4 OS=Homo sapiens GN=ARPC4 PE=1 SV=3 - [ARPC4_HUMAN]
P62318	Small nuclear ribonucleoprotein Sm D3 OS=Homo sapiens GN=SNRPD3 PE=1 SV=1 - [SMD3_HUMAN]
P61158	Actin-related protein 3 OS=Homo sapiens GN=ACTR3 PE=1 SV=3 - [ARP3_HUMAN]
Q06210	Glutamine--fructose-6-phosphate aminotransferase [isomerizing] 1 OS=Homo sapiens GN=GFPT1 PE=1 SV=3 - [GFPT1_HUMAN]
P24666	Low molecular weight phosphotyrosine protein phosphatase OS=Homo sapiens GN=ACP1 PE=1 SV=3 - [PPAC_HUMAN]
P62316	Small nuclear ribonucleoprotein Sm D2 OS=Homo sapiens GN=SNRPD2 PE=1 SV=1 - [SMD2_HUMAN]
O00151	PDZ and LIM domain protein 1 OS=Homo sapiens GN=PDLIM1 PE=1 SV=4 - [PDLI1_HUMAN]
P49588	Alanine--tRNA ligase, cytoplasmic OS=Homo sapiens GN=AARS PE=1 SV=2 - [SYAC_HUMAN]
P32969	60S ribosomal protein L9 OS=Homo sapiens GN=RPL9 PE=1 SV=1 - [RL9_HUMAN]
O00232	26S proteasome non-ATPase regulatory subunit 12 OS=Homo sapiens GN=PSMD12 PE=1 SV=3 - [PSD12_HUMAN]
P40937	Replication factor C subunit 5 OS=Homo sapiens GN=RFC5 PE=1 SV=1 - [RFC5_HUMAN]
P30101	Protein disulfide-isomerase A3 OS=Homo sapiens GN=PDIA3 PE=1 SV=4 - [PDIA3_HUMAN]
P30050	60S ribosomal protein L12 OS=Homo sapiens GN=RPL12 PE=1 SV=1 - [RL12_HUMAN]
P08243	Asparagine synthetase [glutamine-hydrolyzing] OS=Homo sapiens GN=ASNS PE=1 SV=4 - [ASNS_HUMAN]
Q53EZ4	Centrosomal protein of 55 kDa OS=Homo sapiens GN=CEP55 PE=1 SV=3 - [CEP55_HUMAN]
P60228	Eukaryotic translation initiation factor 3 subunit E OS=Homo sapiens GN=EIF3E PE=1 SV=1 - [EIF3E_HUMAN]
Q9UNF0	Protein kinase C and casein kinase substrate in neurons protein 2 OS=Homo sapiens GN=PACSIN2 PE=1 SV=2 - [PACN2_HUMAN]
P24534	Elongation factor 1-beta OS=Homo sapiens GN=EEF1B2 PE=1 SV=3 - [EF1B_HUMAN]
Q8N1F7	Nuclear pore complex protein Nup93 OS=Homo sapiens GN=NUP93 PE=1 SV=2 - [NUP93_HUMAN]
Q9H444	Charged multivesicular body protein 4b OS=Homo sapiens GN=CHMP4B PE=1 SV=1 - [CHM4B_HUMAN]
P42025	Beta-centractin OS=Homo sapiens GN=ACTR1B PE=1 SV=1 - [ACTY_HUMAN]
P51153	Ras-related protein Rab-13 OS=Homo sapiens GN=RAB13 PE=1 SV=1 - [RAB13_HUMAN]
P50395	Rab GDP dissociation inhibitor beta OS=Homo sapiens GN=GDI2 PE=1 SV=2 - [GDIB_HUMAN]
Q9BZM4	NKG2D ligand 3 OS=Homo sapiens GN=ULBP3 PE=1 SV=1 - [N2DL3_HUMAN]
O95782	AP-2 complex subunit alpha-1 OS=Homo sapiens GN=AP2A1 PE=1 SV=3 - [AP2A1_HUMAN]
P17858	ATP-dependent 6-phosphofructokinase, liver type OS=Homo sapiens GN=PFKL PE=1 SV=6 - [PFKAL_HUMAN]
Q8WV92	MIT domain-containing protein 1 OS=Homo sapiens GN=MITD1 PE=1 SV=1 - [MITD1_HUMAN]
O94776	Metastasis-associated protein MTA2 OS=Homo sapiens GN=MTA2 PE=1 SV=1 - [MTA2_HUMAN]
Q0VDF9	Heat shock 70 kDa protein 14 OS=Homo sapiens GN=HSPA14 PE=1 SV=1 - [HSP7E_HUMAN]
Q99497	Protein deglycase DJ-1 OS=Homo sapiens GN=PARK7 PE=1 SV=2 - [PARK7_HUMAN]
Q9UHI8	A disintegrin and metalloproteinase with thrombospondin motifs 1 OS=Homo sapiens GN=ADAMTS1 PE=1 SV=4 - [ATS1_HUMAN]

Q9NWU2	Glucose-induced degradation protein 8 homolog OS=Homo sapiens GN=GID8 PE=1 SV=1 - [GID8_HUMAN]
O15498	Synaptobrevin homolog YKT6 OS=Homo sapiens GN=YKT6 PE=1 SV=1 - [YKT6_HUMAN]
P61289	Proteasome activator complex subunit 3 OS=Homo sapiens GN=PSME3 PE=1 SV=1 - [PSME3_HUMAN]
Q9UKS6	Protein kinase C and casein kinase substrate in neurons protein 3 OS=Homo sapiens GN=PACSIN3 PE=1 SV=2 - [PACN3_HUMAN]
Q9H9H4	Vacuolar protein sorting-associated protein 37B OS=Homo sapiens GN=VPS37B PE=1 SV=1 - [VP37B_HUMAN]
Q9Y295	Developmentally-regulated GTP-binding protein 1 OS=Homo sapiens GN=DRG1 PE=1 SV=1 - [DRG1_HUMAN]
Q16394	Exostosin-1 OS=Homo sapiens GN=EXT1 PE=1 SV=2 - [EXT1_HUMAN]
P38919	Eukaryotic initiation factor 4A-III OS=Homo sapiens GN=EIF4A3 PE=1 SV=4 - [IF4A3_HUMAN]
P60891	Ribose-phosphate pyrophosphokinase 1 OS=Homo sapiens GN=PRPS1 PE=1 SV=2 - [PRPS1_HUMAN]
O00391	Sulfhydryl oxidase 1 OS=Homo sapiens GN=QSOX1 PE=1 SV=3 - [QSOX1_HUMAN]
P62241	40S ribosomal protein S8 OS=Homo sapiens GN=RPS8 PE=1 SV=2 - [RS8_HUMAN]
Q9UBQ0	Vacuolar protein sorting-associated protein 29 OS=Homo sapiens GN=VPS29 PE=1 SV=1 - [VPS29_HUMAN]
P25391	Laminin subunit alpha-1 OS=Homo sapiens GN=LAMA1 PE=1 SV=2 - [LAMA1_HUMAN]
P84085	ADP-ribosylation factor 5 OS=Homo sapiens GN=ARF5 PE=1 SV=2 - [ARF5_HUMAN]
Q96S86	Hyaluronan and proteoglycan link protein 3 OS=Homo sapiens GN=HAPLN3 PE=2 SV=1 - [HPLN3_HUMAN]
P11021	78 kDa glucose-regulated protein OS=Homo sapiens GN=HSPA5 PE=1 SV=2 - [GRP78_HUMAN]
P04083	Annexin A1 OS=Homo sapiens GN=ANXA1 PE=1 SV=2 - [ANXA1_HUMAN]
Q9Y3I0	tRNA-splicing ligase RtcB homolog OS=Homo sapiens GN=RTCB PE=1 SV=1 - [RTCB_HUMAN]
P13010	X-ray repair cross-complementing protein 5 OS=Homo sapiens GN=XRCC5 PE=1 SV=3 - [XRCC5_HUMAN]
P62081	40S ribosomal protein S7 OS=Homo sapiens GN=RPS7 PE=1 SV=1 - [RS7_HUMAN]
P18754	Regulator of chromosome condensation OS=Homo sapiens GN=RCC1 PE=1 SV=1 - [RCC1_HUMAN]
Q9P258	Protein RCC2 OS=Homo sapiens GN=RCC2 PE=1 SV=2 - [RCC2_HUMAN]
P61221	ATP-binding cassette sub-family E member 1 OS=Homo sapiens GN=ABCE1 PE=1 SV=1 - [ABCE1_HUMAN]
Q53HC9	Protein TSSC1 OS=Homo sapiens GN=TSSC1 PE=1 SV=2 - [TSSC1_HUMAN]
P63104	14-3-3 protein zeta/delta OS=Homo sapiens GN=YWHAZ PE=1 SV=1 - [1433Z_HUMAN]
P36578	60S ribosomal protein L4 OS=Homo sapiens GN=RPL4 PE=1 SV=5 - [RL4_HUMAN]
Q9P2J5	Leucine--tRNA ligase, cytoplasmic OS=Homo sapiens GN=LARS PE=1 SV=2 - [SYLC_HUMAN]
Q9UBI1	COMM domain-containing protein 3 OS=Homo sapiens GN=COMMD3 PE=1 SV=1 - [COMD3_HUMAN]
O75821	Eukaryotic translation initiation factor 3 subunit G OS=Homo sapiens GN=EIF3G PE=1 SV=2 - [EIF3G_HUMAN]
O75131	Copine-3 OS=Homo sapiens GN=CPNE3 PE=1 SV=1 - [CPNE3_HUMAN]
Q53H96	Pyrroline-5-carboxylate reductase 3 OS=Homo sapiens GN=PYCRL PE=1 SV=3 - [P5CR3_HUMAN]
P62280	40S ribosomal protein S11 OS=Homo sapiens GN=RPS11 PE=1 SV=3 - [RS11_HUMAN]
P07942	Laminin subunit beta-1 OS=Homo sapiens GN=LAMB1 PE=1 SV=2 - [LAMB1_HUMAN]
P08754	Guanine nucleotide-binding protein G(k) subunit alpha OS=Homo sapiens GN=GNAI3 PE=1 SV=3 - [GNAI3 HUMAN]
Q9Y5S9	RNA-binding protein 8A OS=Homo sapiens GN=RBM8A PE=1 SV=1 - [RBM8A_HUMAN]
P10768	S-formylglutathione hydrolase OS=Homo sapiens GN=ESD PE=1 SV=2 - [ESTD_HUMAN]
Q96K80	Zinc finger CCCH domain-containing protein 10 OS=Homo sapiens GN=ZC3H10 PE=1 SV=1 - [ZC3HA_HUMAN]
Q9BTV5	Fibronectin type III and SPRY domain-containing protein 1 OS=Homo sapiens GN=FSD1 PE=1 SV=1 - [FSD1_HUMAN]
Q01082	Spectrin beta chain, non-erythrocytic 1 OS=Homo sapiens GN=SPTBN1 PE=1 SV=2 - [SPTB2_HUMAN]
P35268	60S ribosomal protein L22 OS=Homo sapiens GN=RPL22 PE=1 SV=2 - [RL22_HUMAN]
Q9P2B2	Prostaglandin F2 receptor negative regulator OS=Homo sapiens GN=PTGFRN PE=1 SV=2 - [FPRP_HUMAN]
P40926	Malate dehydrogenase, mitochondrial OS=Homo sapiens GN=MDH2 PE=1 SV=3 - [MDHM_HUMAN]
P30876	DNA-directed RNA polymerase II subunit RPB2 OS=Homo sapiens GN=POLR2B PE=1 SV=1 - [RPB2_HUMAN]
Q9Y296	Trafficking protein particle complex subunit 4 OS=Homo sapiens GN=TRAPPC4 PE=1 SV=1 - [TPPC4_HUMAN]
Q08211	ATP-dependent RNA helicase A OS=Homo sapiens GN=DHX9 PE=1 SV=4 - [DHX9_HUMAN]
P11766	Alcohol dehydrogenase class-3 OS=Homo sapiens GN=ADH5 PE=1 SV=4 - [ADHX_HUMAN]
P49643	DNA primase large subunit OS=Homo sapiens GN=PRIM2 PE=1 SV=2 - [PRI2_HUMAN]
O00571	ATP-dependent RNA helicase DDX3X OS=Homo sapiens GN=DDX3X PE=1 SV=3 - [DDX3X_HUMAN]
Q14141	Septin-6 OS=Homo sapiens GN=SEPT6 PE=1 SV=4 - [SEPT6_HUMAN]
P12268	Inosine-5'-monophosphate dehydrogenase 2 OS=Homo sapiens GN=IMPDH2 PE=1 SV=2 - [IMDH2_HUMAN]
P26022	Pentraxin-related protein PTX3 OS=Homo sapiens GN=PTX3 PE=1 SV=3 - [PTX3_HUMAN]
P25325	3-mercaptopyruvate sulfurtransferase OS=Homo sapiens GN=MPST PE=1 SV=3 - [THTM_HUMAN]
Q9Y2L1	Exosome complex exonuclease RRP44 OS=Homo sapiens GN=DIS3 PE=1 SV=2 - [RRP44_HUMAN]
Q92572	AP-3 complex subunit sigma-1 OS=Homo sapiens GN=AP3S1 PE=1 SV=1 - [AP3S1_HUMAN]
Q13263	Transcription intermediary factor 1-beta OS=Homo sapiens GN=TRIM28 PE=1 SV=5 - [TIF1B_HUMAN]
Q99460	26S proteasome non-ATPase regulatory subunit 1 OS=Homo sapiens GN=PSMD1 PE=1 SV=2 - [PSMD1_HUMAN]
Q8NFH3	Nucleoporin Nup43 OS=Homo sapiens GN=NUP43 PE=1 SV=1 - [NUP43_HUMAN]
P49720	Proteasome subunit beta type-3 OS=Homo sapiens GN=PSMB3 PE=1 SV=2 - [PSB3_HUMAN]
P68431	Histone H3.1 OS=Homo sapiens GN=HIST1H3A PE=1 SV=2 - [H31_HUMAN]
Q9NSD9	Phenylalanine--tRNA ligase beta subunit OS=Homo sapiens GN=FARSB PE=1 SV=3 - [SYFB_HUMAN]
Q9UBF2	Coatomer subunit gamma-2 OS=Homo sapiens GN=COPG2 PE=1 SV=1 - [COPG2_HUMAN]
P11717	Cation-independent mannose-6-phosphate receptor OS=Homo sapiens GN=IGF2R PE=1 SV=3 - [MPRI_HUMAN]
P26196	Probable ATP-dependent RNA helicase DDX6 OS=Homo sapiens GN=DDX6 PE=1 SV=2 - [DDX6_HUMAN]
P56192	Methionine--tRNA ligase, cytoplasmic OS=Homo sapiens GN=MARS PE=1 SV=2 - [SYMC_HUMAN]
P12956	X-ray repair cross-complementing protein 6 OS=Homo sapiens GN=XRCC6 PE=1 SV=2 - [XRCC6_HUMAN]
O43143	Pre-mRNA-splicing factor ATP-dependent RNA helicase DHX15 OS=Homo sapiens GN=DHX15 PE=1 SV=2 - [DHX15_HUMAN]
O15230	Laminin subunit alpha-5 OS=Homo sapiens GN=LAMA5 PE=1 SV=8 - [LAMA5_HUMAN]
Q13838	Spliceosome RNA helicase DDX39B OS=Homo sapiens GN=DDX39B PE=1 SV=1 - [DX39B_HUMAN]
P61586	Transforming protein RhoA OS=Homo sapiens GN=RHOA PE=1 SV=1 - [RHOA_HUMAN]
Q9Y5X1	Sorting nexin-9 OS=Homo sapiens GN=SNX9 PE=1 SV=1 - [SNX9_HUMAN]
O75643	U5 small nuclear ribonucleoprotein 200 kDa helicase OS=Homo sapiens GN=SNRNP200 PE=1 SV=2 - [U520 HUMAN]
P08174	Complement decay-accelerating factor OS=Homo sapiens GN=CD55 PE=1 SV=4 - [DAF_HUMAN]
O00468	Agrin OS=Homo sapiens GN=AGRN PE=1 SV=5 - [AGRIN_HUMAN]
P33993	DNA replication licensing factor MCM7 OS=Homo sapiens GN=MCM7 PE=1 SV=4 - [MCM7_HUMAN]
Q14240	Eukaryotic initiation factor 4A-II OS=Homo sapiens GN=EIF4A2 PE=1 SV=2 - [IF4A2_HUMAN]
Q14697	Neutral alpha-glucosidase AB OS=Homo sapiens GN=GANAB PE=1 SV=3 - [GANAB_HUMAN]
Q6P2Q9	Pre-mRNA-processing-splicing factor 8 OS=Homo sapiens GN=PRPF8 PE=1 SV=2 - [PRP8_HUMAN]
Q13155	Aminoacyl tRNA synthase complex-interacting multifunctional protein 2 OS=Homo sapiens GN=AIMP2 PE=1 SV=2 - [AIMP2_HUMAN]
Q9NR50	Translation initiation factor eIF-2B subunit gamma OS=Homo sapiens GN=EIF2B3 PE=1 SV=1 - [EI2BG_HUMAN]
Q99961	Endophilin-A2 OS=Homo sapiens GN=SH3GL1 PE=1 SV=1 - [SH3G1_HUMAN]
P50150	Guanine nucleotide-binding protein G(I)/G(S)/G(O) subunit gamma-4 OS=Homo sapiens GN=GNG4 PE=1 SV=1 - [GBG4_HUMAN]
Q8WUW1	Protein BRICK1 OS=Homo sapiens GN=BRK1 PE=1 SV=1 - [BRK1_HUMAN]
Q14258	E3 ubiquitin/ISG15 ligase TRIM25 OS=Homo sapiens GN=TRIM25 PE=1 SV=2 - [TRI25_HUMAN]
P18124	60S ribosomal protein L7 OS=Homo sapiens GN=RPL7 PE=1 SV=1 - [RL7_HUMAN]
P04899	Guanine nucleotide-binding protein G(i) subunit alpha-2 OS=Homo sapiens GN=GNAI2 PE=1 SV=3 - [GNAI2_HUMAN]
P27635	60S ribosomal protein L10 OS=Homo sapiens GN=RPL10 PE=1 SV=4 - [RL10_HUMAN]
Q12996	Cleavage stimulation factor subunit 3 OS=Homo sapiens GN=CSTF3 PE=1 SV=1 - [CSTF3_HUMAN]
Q12904	Aminoacyl tRNA synthase complex-interacting multifunctional protein 1 OS=Homo sapiens GN=AIMP1 PE=1 SV=2 - [AIMP1_HUMAN]
Q96CW1	AP-2 complex subunit mu OS=Homo sapiens GN=AP2M1 PE=1 SV=2 - [AP2M1_HUMAN]
Q9BZH6	WD repeat-containing protein 11 OS=Homo sapiens GN=WDR11 PE=1 SV=1 - [WDR11_HUMAN]
P61326	Protein mago nashi homolog OS=Homo sapiens GN=MAGOH PE=1 SV=1 - [MGN_HUMAN]
Q6P2E9	Enhancer of mRNA-decapping protein 4 OS=Homo sapiens GN=EDC4 PE=1 SV=1 - [EDC4_HUMAN]
Q14315	Filamin-C OS=Homo sapiens GN=FLNC PE=1 SV=3 - [FLNC_HUMAN]
P46940	Ras GTPase-activating-like protein IQGAP1 OS=Homo sapiens GN=IQGAP1 PE=1 SV=1 - [IQGA1_HUMAN]
Q8IVM0	Coiled-coil domain-containing protein 50 OS=Homo sapiens GN=CCDC50 PE=1 SV=1 - [CCD50_HUMAN]
Q9Y262	Eukaryotic translation initiation factor 3 subunit L OS=Homo sapiens GN=EIF3L PE=1 SV=1 - [EIF3L_HUMAN]
Q01813	ATP-dependent 6-phosphofructokinase, platelet type OS=Homo sapiens GN=PFKP PE=1 SV=2 - [PFKAP_HUMAN]
P54920	Alpha-soluble NSF attachment protein OS=Homo sapiens GN=NAPA PE=1 SV=3 - [SNAA_HUMAN]
P26641	Elongation factor 1-gamma OS=Homo sapiens GN=EEF1G PE=1 SV=3 - [EF1G_HUMAN]
P83110	Serine protease HTRA3 OS=Homo sapiens GN=HTRA3 PE=1 SV=2 - [HTRA3_HUMAN]
O60678	Protein arginine N-methyltransferase 3 OS=Homo sapiens GN=PRMT3 PE=1 SV=3 - [ANM3_HUMAN]
P15880	40S ribosomal protein S2 OS=Homo sapiens GN=RPS2 PE=1 SV=2 - [RS2_HUMAN]
O60814	Histone H2B type 1-K OS=Homo sapiens GN=HIST1H2BK PE=1 SV=3 - [H2B1K_HUMAN]
P06899	Histone H2B type 1-J OS=Homo sapiens GN=HIST1H2BJ PE=1 SV=3 - [H2B1J_HUMAN]
P17844	Probable ATP-dependent RNA helicase DDX5 OS=Homo sapiens GN=DDX5 PE=1 SV=1 - [DDX5_HUMAN]
Q9Y287	Integral membrane protein 2B OS=Homo sapiens GN=ITM2B PE=1 SV=1 - [ITM2B_HUMAN]
Q9Y2S0	DNA-directed RNA polymerases I and III subunit RPAC2 OS=Homo sapiens GN=POLR1D PE=1 SV=1 - [RPAC2 HUMAN]
O96019	Actin-like protein 6A OS=Homo sapiens GN=ACTL6A PE=1 SV=1 - [ACL6A_HUMAN]
P26368	Splicing factor U2AF 65 kDa subunit OS=Homo sapiens GN=U2AF2 PE=1 SV=4 - [U2AF2_HUMAN]
Q02790	Peptidyl-prolyl cis-trans isomerase FKBP4 OS=Homo sapiens GN=FKBP4 PE=1 SV=3 - [FKBP4_HUMAN]
P78527	DNA-dependent protein kinase catalytic subunit OS=Homo sapiens GN=PRKDC PE=1 SV=3 - [PRKDC_HUMAN]
Q92600	Cell differentiation protein RCD1 homolog OS=Homo sapiens GN=RQCD1 PE=1 SV=1 - [RCD1_HUMAN]
Q8TAF3	WD repeat-containing protein 48 OS=Homo sapiens GN=WDR48 PE=1 SV=1 - [WDR48_HUMAN]
P15531	Nucleoside diphosphate kinase A OS=Homo sapiens GN=NME1 PE=1 SV=1 - [NDKA_HUMAN]
O15212	Prefoldin subunit 6 OS=Homo sapiens GN=PFDN6 PE=1 SV=1 - [PFD6 HUMAN]

P12532	Creatine kinase U-type, mitochondrial OS=Homo sapiens GN=CKMT1A PE=1 SV=1 - [KCRU_HUMAN]
P61978	Heterogeneous nuclear ribonucleoprotein K OS=Homo sapiens GN=HNRNPK PE=1 SV=1 - [HNRPK_HUMAN]
P61019	Ras-related protein Rab-2A OS=Homo sapiens GN=RAB2A PE=1 SV=1 - [RAB2A_HUMAN]
P35232	Prohibitin OS=Homo sapiens GN=PHB PE=1 SV=1 - [PHB_HUMAN]
Q6PCB0	von Willebrand factor A domain-containing protein 1 OS=Homo sapiens GN=VWA1 PE=2 SV=1 - [VWA1_HUMAN]
P09651	Heterogeneous nuclear ribonucleoprotein A1 OS=Homo sapiens GN=HNRNPA1 PE=1 SV=5 - [ROA1_HUMAN]
Q9NQT5	Exosome complex component RRP40 OS=Homo sapiens GN=EXOSC3 PE=1 SV=3 - [EXOS3_HUMAN]
P61020	Ras-related protein Rab-5B OS=Homo sapiens GN=RAB5B PE=1 SV=1 - [RAB5B_HUMAN]
Q05707	Collagen alpha-1(XIV) chain OS=Homo sapiens GN=COL14A1 PE=1 SV=3 - [COEA1_HUMAN]
O15371	Eukaryotic translation initiation factor 3 subunit D OS=Homo sapiens GN=EIF3D PE=1 SV=1 - [EIF3D_HUMAN]
O14737	Programmed cell death protein 5 OS=Homo sapiens GN=PDCD5 PE=1 SV=3 - [PDCD5_HUMAN]
P49591	Serine--tRNA ligase, cytoplasmic OS=Homo sapiens GN=SARS PE=1 SV=3 - [SYSC_HUMAN]
Q9UHC7	E3 ubiquitin-protein ligase makorin-1 OS=Homo sapiens GN=MKRN1 PE=1 SV=3 - [MKRN1_HUMAN]
O43324	Eukaryotic translation elongation factor 1 epsilon-1 OS=Homo sapiens GN=EEF1E1 PE=1 SV=1 - [MCA3_HUMAN]
Q15021	Condensin complex subunit 1 OS=Homo sapiens GN=NCAPD2 PE=1 SV=3 - [CND1_HUMAN]
O60547	GDP-mannose 4,6 dehydratase OS=Homo sapiens GN=GMDS PE=1 SV=1 - [GMDS_HUMAN]
Q6IA86	Elongator complex protein 2 OS=Homo sapiens GN=ELP2 PE=1 SV=2 - [ELP2_HUMAN]
P31948	Stress-induced-phosphoprotein 1 OS=Homo sapiens GN=STIP1 PE=1 SV=1 - [STIP1_HUMAN]
P02792	Ferritin light chain OS=Homo sapiens GN=FTL PE=1 SV=2 - [FRIL_HUMAN]
P21964	Catechol O-methyltransferase OS=Homo sapiens GN=COMT PE=1 SV=2 - [COMT_HUMAN]
O15160	DNA-directed RNA polymerases I and III subunit RPAC1 OS=Homo sapiens GN=POLR1C PE=1 SV=1 - [RPAC1_HUMAN]
Q6P2C8	Mediator of RNA polymerase II transcription subunit 27 OS=Homo sapiens GN=MED27 PE=1 SV=1 - [MED27_HUMAN]
P62258	14-3-3 protein epsilon OS=Homo sapiens GN=YWHAE PE=1 SV=1 - [1433E_HUMAN]
P49915	GMP synthase [glutamine-hydrolyzing] OS=Homo sapiens GN=GMPS PE=1 SV=1 - [GUAA_HUMAN]
O60506	Heterogeneous nuclear ribonucleoprotein Q OS=Homo sapiens GN=SYNCRIP PE=1 SV=2 - [HNRPQ_HUMAN]
Q5VYK3	Proteasome-associated protein ECM29 homolog OS=Homo sapiens GN=ECM29 PE=1 SV=2 - [ECM29_HUMAN]
P02786	Transferrin receptor protein 1 OS=Homo sapiens GN=TFRC PE=1 SV=2 - [TFR1_HUMAN]
O43175	D-3-phosphoglycerate dehydrogenase OS=Homo sapiens GN=PHGDH PE=1 SV=4 - [SERA_HUMAN]
P01137	Transforming growth factor beta-1 OS=Homo sapiens GN=TGFB1 PE=1 SV=2 - [TGFB1_HUMAN]
O43854	EGF-like repeat and discoidin I-like domain-containing protein 3 OS=Homo sapiens GN=EDIL3 PE=1 SV=1 - [EDIL3_HUMAN]
Q12899	Tripartite motif-containing protein 26 OS=Homo sapiens GN=TRIM26 PE=1 SV=1 - [TRI26_HUMAN]
P62424	60S ribosomal protein L7a OS=Homo sapiens GN=RPL7A PE=1 SV=2 - [RL7A_HUMAN]
Q9H6D7	HAUS augmin-like complex subunit 4 OS=Homo sapiens GN=HAUS4 PE=1 SV=1 - [HAUS4_HUMAN]
O00267	Transcription elongation factor SPT5 OS=Homo sapiens GN=SUPT5H PE=1 SV=1 - [SPT5H_HUMAN]
O43707	Alpha-actinin-4 OS=Homo sapiens GN=ACTN4 PE=1 SV=2 - [ACTN4_HUMAN]
P33176	Kinesin-1 heavy chain OS=Homo sapiens GN=KIF5B PE=1 SV=1 - [KINH_HUMAN]
P55735	Protein SEC13 homolog OS=Homo sapiens GN=SEC13 PE=1 SV=3 - [SEC13_HUMAN]
Q12768	WASH complex subunit strumpellin OS=Homo sapiens GN=KIAA0196 PE=1 SV=1 - [STRUM_HUMAN]
O00429	Dynamin-1-like protein OS=Homo sapiens GN=DNM1L PE=1 SV=2 - [DNM1L_HUMAN]
P52272	Heterogeneous nuclear ribonucleoprotein M OS=Homo sapiens GN=HNRNPM PE=1 SV=3 - [HNRPM_HUMAN]
O95163	Elongator complex protein 1 OS=Homo sapiens GN=IKBKAP PE=1 SV=3 - [ELP1_HUMAN]
Q13868	Exosome complex component RRP4 OS=Homo sapiens GN=EXOSC2 PE=1 SV=2 - [EXOS2_HUMAN]
Q13547	Histone deacetylase 1 OS=Homo sapiens GN=HDAC1 PE=1 SV=1 - [HDAC1_HUMAN]
Q9UJX3	Anaphase-promoting complex subunit 7 OS=Homo sapiens GN=ANAPC7 PE=1 SV=4 - [APC7_HUMAN]
Q8TF74	WAS/WASL-interacting protein family member 2 OS=Homo sapiens GN=WIPF2 PE=1 SV=1 - [WIPF2_HUMAN]
P15121	Aldose reductase OS=Homo sapiens GN=AKR1B1 PE=1 SV=3 - [ALDR_HUMAN]
Q9H7D7	WD repeat-containing protein 26 OS=Homo sapiens GN=WDR26 PE=1 SV=3 - [WDR26_HUMAN]
Q9BVC4	Target of rapamycin complex subunit LST8 OS=Homo sapiens GN=MLST8 PE=1 SV=1 - [LST8_HUMAN]
P62330	ADP-ribosylation factor 6 OS=Homo sapiens GN=ARF6 PE=1 SV=2 - [ARF6_HUMAN]
Q86Y56	Dynein assembly factor 5, axonemal OS=Homo sapiens GN=DNAAF5 PE=1 SV=4 - [DAAF5_HUMAN]
P62917	60S ribosomal protein L8 OS=Homo sapiens GN=RPL8 PE=1 SV=2 - [RL8_HUMAN]
P61160	Actin-related protein 2 OS=Homo sapiens GN=ACTR2 PE=1 SV=1 - [ARP2_HUMAN]
O14744	Protein arginine N-methyltransferase 5 OS=Homo sapiens GN=PRMT5 PE=1 SV=4 - [ANM5_HUMAN]
Q8N668	COMM domain-containing protein 1 OS=Homo sapiens GN=COMMD1 PE=1 SV=1 - [COMD1_HUMAN]
O60884	DnaJ homolog subfamily A member 2 OS=Homo sapiens GN=DNAJA2 PE=1 SV=1 - [DNJA2_HUMAN]
P48444	Coatomer subunit delta OS=Homo sapiens GN=ARCN1 PE=1 SV=1 - [COPD_HUMAN]
P24821	Tenascin OS=Homo sapiens GN=TNC PE=1 SV=3 - [TENA_HUMAN]
O00567	Nucleolar protein 56 OS=Homo sapiens GN=NOP56 PE=1 SV=4 - [NOP56_HUMAN]
O15143	Actin-related protein 2/3 complex subunit 1B OS=Homo sapiens GN=ARPC1B PE=1 SV=3 - [ARC1B_HUMAN]
Q4G0F5	Vacuolar protein sorting-associated protein 26B OS=Homo sapiens GN=VPS26B PE=1 SV=2 - [VP26B_HUMAN]
P62699	Protein yippee-like 5 OS=Homo sapiens GN=YPEL5 PE=1 SV=1 - [YPEL5_HUMAN]
Q14974	Importin subunit beta-1 OS=Homo sapiens GN=KPNB1 PE=1 SV=2 - [IMB1_HUMAN]
P46781	40S ribosomal protein S9 OS=Homo sapiens GN=RPS9 PE=1 SV=3 - [RS9_HUMAN]
O14828	Secretory carrier-associated membrane protein 3 OS=Homo sapiens GN=SCAMP3 PE=1 SV=3 - [SCAM3_HUMAN]
P48556	26S proteasome non-ATPase regulatory subunit 8 OS=Homo sapiens GN=PSMD8 PE=1 SV=2 - [PSMD8_HUMAN]
P53350	Serine/threonine-protein kinase PLK1 OS=Homo sapiens GN=PLK1 PE=1 SV=1 - [PLK1_HUMAN]
Q9H1Y0	Autophagy protein 5 OS=Homo sapiens GN=ATG5 PE=1 SV=2 - [ATG5_HUMAN]
O75794	Cell division cycle protein 123 homolog OS=Homo sapiens GN=CDC123 PE=1 SV=1 - [CD123_HUMAN]
Q92930	Ras-related protein Rab-8B OS=Homo sapiens GN=RAB8B PE=1 SV=2 - [RAB8B_HUMAN]
P51991	Heterogeneous nuclear ribonucleoprotein A3 OS=Homo sapiens GN=HNRNPA3 PE=1 SV=2 - [ROA3_HUMAN]
P18669	Phosphoglycerate mutase 1 OS=Homo sapiens GN=PGAM1 PE=1 SV=2 - [PGAM1_HUMAN]
O60256	Phosphoribosyl pyrophosphate synthase-associated protein 2 OS=Homo sapiens GN=PRPSAP2 PE=1 SV=1 - [KPRB_HUMAN]
P46060	Ran GTPase-activating protein 1 OS=Homo sapiens GN=RANGAP1 PE=1 SV=1 - [RAGP1_HUMAN]
Q5VW32	BRO1 domain-containing protein BROX OS=Homo sapiens GN=BROX PE=1 SV=1 - [BROX_HUMAN]
Q9BRJ7	Protein syndesmos OS=Homo sapiens GN=NUDT16L1 PE=1 SV=1 - [SDOS_HUMAN]
Q13200	26S proteasome non-ATPase regulatory subunit 2 OS=Homo sapiens GN=PSMD2 PE=1 SV=3 - [PSMD2_HUMAN]
Q6SZW1	Sterile alpha and TIR motif-containing protein 1 OS=Homo sapiens GN=SARM1 PE=1 SV=1 - [SARM1_HUMAN]
Q96P16	Regulation of nuclear pre-mRNA domain-containing protein 1A OS=Homo sapiens GN=RPRD1A PE=1 SV=1 - [RPR1A_HUMAN]
O43264	Centromere/kinetochore protein zw10 homolog OS=Homo sapiens GN=ZW10 PE=1 SV=3 - [ZW10_HUMAN]
Q9Y490	Talin-1 OS=Homo sapiens GN=TLN1 PE=1 SV=3 - [TLN1_HUMAN]
P38606	V-type proton ATPase catalytic subunit A OS=Homo sapiens GN=ATP6V1A PE=1 SV=2 - [VATA_HUMAN]
P41091	Eukaryotic translation initiation factor 2 subunit 3 OS=Homo sapiens GN=EIF2S3 PE=1 SV=3 - [IF2G_HUMAN]
P00338	L-lactate dehydrogenase A chain OS=Homo sapiens GN=LDHA PE=1 SV=2 - [LDHA_HUMAN]
Q15185	Prostaglandin E synthase 3 OS=Homo sapiens GN=PTGES3 PE=1 SV=1 - [TEBP_HUMAN]
Q9NQX3	Gephyrin OS=Homo sapiens GN=GPHN PE=1 SV=1 - [GEPH_HUMAN]
P13807	Glycogen [starch] synthase, muscle OS=Homo sapiens GN=GYS1 PE=1 SV=2 - [GYS1_HUMAN]
P47755	F-actin-capping protein subunit alpha-2 OS=Homo sapiens GN=CAPZA2 PE=1 SV=3 - [CAZA2_HUMAN]
P63208	S-phase kinase-associated protein 1 OS=Homo sapiens GN=SKP1 PE=1 SV=2 - [SKP1_HUMAN]
O94832	Unconventional myosin-Id OS=Homo sapiens GN=MYO1D PE=1 SV=2 - [MYO1D_HUMAN]
P63167	Dynein light chain 1, cytoplasmic OS=Homo sapiens GN=DYNLL1 PE=1 SV=1 - [DYL1_HUMAN]
P61225	Ras-related protein Rap-2b OS=Homo sapiens GN=RAP2B PE=1 SV=1 - [RAP2B_HUMAN]
P27361	Mitogen-activated protein kinase 3 OS=Homo sapiens GN=MAPK3 PE=1 SV=4 - [MK03_HUMAN]
Q13310	Polyadenylate-binding protein 4 OS=Homo sapiens GN=PABPC4 PE=1 SV=1 - [PABP4_HUMAN]
Q9UQ80	Proliferation-associated protein 2G4 OS=Homo sapiens GN=PA2G4 PE=1 SV=3 - [PA2G4_HUMAN]
P61769	Beta-2-microglobulin OS=Homo sapiens GN=B2M PE=1 SV=1 - [B2MG_HUMAN]
Q96FH0	Protein MEF2BNB OS=Homo sapiens GN=MEF2BNB PE=1 SV=1 - [MF2NB_HUMAN]
P13797	Plastin-3 OS=Homo sapiens GN=PLS3 PE=1 SV=4 - [PLST_HUMAN]
Q07955	Serine/arginine-rich splicing factor 1 OS=Homo sapiens GN=SRSF1 PE=1 SV=2 - [SRSF1_HUMAN]
Q12874	Splicing factor 3A subunit 3 OS=Homo sapiens GN=SF3A3 PE=1 SV=1 - [SF3A3_HUMAN]
Q9Y696	Chloride intracellular channel protein 4 OS=Homo sapiens GN=CLIC4 PE=1 SV=4 - [CLIC4_HUMAN]
P18065	Insulin-like growth factor-binding protein 2 OS=Homo sapiens GN=IGFBP2 PE=1 SV=2 - [IBP2_HUMAN]
P78346	Ribonuclease P protein subunit p30 OS=Homo sapiens GN=RPP30 PE=1 SV=1 - [RPP30_HUMAN]
P11233	Ras-related protein Ral-A OS=Homo sapiens GN=RALA PE=1 SV=1 - [RALA_HUMAN]
Q08AM6	Protein VAC14 homolog OS=Homo sapiens GN=VAC14 PE=1 SV=1 - [VAC14_HUMAN]
P13497	Bone morphogenetic protein 1 OS=Homo sapiens GN=BMP1 PE=1 SV=2 - [BMP1_HUMAN]
P49642	DNA primase small subunit OS=Homo sapiens GN=PRIM1 PE=1 SV=1 - [PRI1_HUMAN]
Q92485	Acid sphingomyelinase-like phosphodiesterase 3b OS=Homo sapiens GN=SMPDL3B PE=2 SV=2 - [ASM3B_HUMAN]
Q16555	Dihydropyrimidinase-related protein 2 OS=Homo sapiens GN=DPYSL2 PE=1 SV=1 - [DPYL2_HUMAN]
P61604	10 kDa heat shock protein, mitochondrial OS=Homo sapiens GN=HSPE1 PE=1 SV=2 - [CH10_HUMAN]
O60826	Coiled-coil domain-containing protein 22 OS=Homo sapiens GN=CCDC22 PE=1 SV=1 - [CCD22_HUMAN]
P55058	Phospholipid transfer protein OS=Homo sapiens GN=PLTP PE=1 SV=1 - [PLTP_HUMAN]
P62269	40S ribosomal protein S18 OS=Homo sapiens GN=RPS18 PE=1 SV=3 - [RS18_HUMAN]
P10586	Receptor-type tyrosine-protein phosphatase F OS=Homo sapiens GN=PTPRF PE=1 SV=2 - [PTPRF_HUMAN]
P25205	DNA replication licensing factor MCM3 OS=Homo sapiens GN=MCM3 PE=1 SV=3 - [MCM3_HUMAN]
P54727	UV excision repair protein RAD23 homolog B OS=Homo sapiens GN=RAD23B PE=1 SV=1 - [RD23B_HUMAN]

P50542	Peroxisomal targeting signal 1 receptor OS=Homo sapiens GN=PEX5 PE=1 SV=3 - [PEX5_HUMAN]
P46459	Vesicle-fusing ATPase OS=Homo sapiens GN=NSF PE=1 SV=3 - [NSF_HUMAN]
Q9NZ32	Actin-related protein 10 OS=Homo sapiens GN=ACTR10 PE=1 SV=1 - [ARP10_HUMAN]
Q10567	AP-1 complex subunit beta-1 OS=Homo sapiens GN=AP1B1 PE=1 SV=2 - [AP1B1_HUMAN]
Q9UJX2	Cell division cycle protein 23 homolog OS=Homo sapiens GN=CDC23 PE=1 SV=3 - [CDC23_HUMAN]
P0CW22	40S ribosomal protein S17-like OS=Homo sapiens GN=RPS17L PE=1 SV=1 - [RS17L_HUMAN]
Q15836	Vesicle-associated membrane protein 3 OS=Homo sapiens GN=VAMP3 PE=1 SV=3 - [VAMP3_HUMAN]
Q9Y333	U6 snRNA-associated Sm-like protein LSm2 OS=Homo sapiens GN=LSM2 PE=1 SV=1 - [LSM2_HUMAN]
Q9Y2A7	Nck-associated protein 1 OS=Homo sapiens GN=NCKAP1 PE=1 SV=1 - [NCKP1_HUMAN]
Q9UIA9	Exportin-7 OS=Homo sapiens GN=XPO7 PE=1 SV=3 - [XPO7_HUMAN]
P07602	Prosaposin OS=Homo sapiens GN=PSAP PE=1 SV=2 - [SAP_HUMAN]
Q02543	60S ribosomal protein L18a OS=Homo sapiens GN=RPL18A PE=1 SV=2 - [RL18A_HUMAN]
Q13405	39S ribosomal protein L49, mitochondrial OS=Homo sapiens GN=MRPL49 PE=1 SV=1 - [RM49_HUMAN]
P14625	Endoplasmin OS=Homo sapiens GN=HSP90B1 PE=1 SV=1 - [ENPL_HUMAN]
Q15459	Splicing factor 3A subunit 1 OS=Homo sapiens GN=SF3A1 PE=1 SV=1 - [SF3A1_HUMAN]
Q5TDH0	Protein DDI1 homolog 2 OS=Homo sapiens GN=DDI2 PE=1 SV=1 - [DDI2_HUMAN]
Q15818	Neuronal pentraxin-1 OS=Homo sapiens GN=NPTX1 PE=2 SV=2 - [NPTX1_HUMAN]
Q9NR45	Sialic acid synthase OS=Homo sapiens GN=NANS PE=1 SV=2 - [SIAS_HUMAN]
A0AVT1	Ubiquitin-like modifier-activating enzyme 6 OS=Homo sapiens GN=UBA6 PE=1 SV=1 - [UBA6_HUMAN]
P54709	Sodium/potassium-transporting ATPase subunit beta-3 OS=Homo sapiens GN=ATP1B3 PE=1 SV=1 - [AT1B3_HUMAN]
P22626	Heterogeneous nuclear ribonucleoproteins A2/B1 OS=Homo sapiens GN=HNRNPA2B1 PE=1 SV=2 - [ROA2_HUMAN]
Q15436	Protein transport protein Sec23A OS=Homo sapiens GN=SEC23A PE=1 SV=2 - [SC23A_HUMAN]
Q13151	Heterogeneous nuclear ribonucleoprotein A0 OS=Homo sapiens GN=HNRNPA0 PE=1 SV=1 - [ROA0_HUMAN]
Q9P265	Disco-interacting protein 2 homolog B OS=Homo sapiens GN=DIP2B PE=1 SV=3 - [DIP2B_HUMAN]
Q9Y3L5	Ras-related protein Rap-2c OS=Homo sapiens GN=RAP2C PE=1 SV=1 - [RAP2C_HUMAN]
P16870	Carboxypeptidase E OS=Homo sapiens GN=CPE PE=1 SV=1 - [CBPE_HUMAN]
O43865	Putative adenosylhomocysteinase 2 OS=Homo sapiens GN=AHCYL1 PE=1 SV=2 - [SAHH2_HUMAN]
Q9UKV8	Protein argonaute-2 OS=Homo sapiens GN=AGO2 PE=1 SV=3 - [AGO2_HUMAN]
Q9H7P6	Multivesicular body subunit 12B OS=Homo sapiens GN=MVB12B PE=1 SV=2 - [MB12B_HUMAN]
O14672	Disintegrin and metalloproteinase domain-containing protein 10 OS=Homo sapiens GN=ADAM10 PE=1 SV=1 - [ADA10_HUMAN]
O60568	Procollagen-lysine,2-oxoglutarate 5-dioxygenase 3 OS=Homo sapiens GN=PLOD3 PE=1 SV=1 - [PLOD3_HUMAN]
P06748	Nucleophosmin OS=Homo sapiens GN=NPM1 PE=1 SV=2 - [NPM_HUMAN]
Q07866	Kinesin light chain 1 OS=Homo sapiens GN=KLC1 PE=1 SV=2 - [KLC1_HUMAN]
Q92841	Probable ATP-dependent RNA helicase DDX17 OS=Homo sapiens GN=DDX17 PE=1 SV=2 - [DDX17_HUMAN]
Q13557	Calcium/calmodulin-dependent protein kinase type II subunit delta OS=Homo sapiens GN=CAMK2D PE=1 SV=3 - [KCC2D_HUMAN]
P61916	Epididymal secretory protein E1 OS=Homo sapiens GN=NPC2 PE=1 SV=1 - [NPC2_HUMAN]
Q15907	Ras-related protein Rab-11B OS=Homo sapiens GN=RAB11B PE=1 SV=4 - [RB11B_HUMAN]
O60513	Beta-1,4-galactosyltransferase 4 OS=Homo sapiens GN=B4GALT4 PE=1 SV=1 - [B4GT4_HUMAN]
O76071	Probable cytosolic iron-sulfur protein assembly protein CIAO1 OS=Homo sapiens GN=CIAO1 PE=1 SV=1 - [CIAO1_HUMAN]
Q8N1G4	Leucine-rich repeat-containing protein 47 OS=Homo sapiens GN=LRRC47 PE=1 SV=1 - [LRC47_HUMAN]
Q9Y4E8	Ubiquitin carboxyl-terminal hydrolase 15 OS=Homo sapiens GN=USP15 PE=1 SV=3 - [UBP15_HUMAN]
Q9NVA2	Septin-11 OS=Homo sapiens GN=SEPT11 PE=1 SV=3 - [SEP11_HUMAN]
P17812	CTP synthase 1 OS=Homo sapiens GN=CTPS1 PE=1 SV=2 - [PYRG1_HUMAN]
P43490	Nicotinamide phosphoribosyltransferase OS=Homo sapiens GN=NAMPT PE=1 SV=1 - [NAMPT_HUMAN]
P50570	Dynamin-2 OS=Homo sapiens GN=DNM2 PE=1 SV=2 - [DYN2_HUMAN]
P46783	40S ribosomal protein S10 OS=Homo sapiens GN=RPS10 PE=1 SV=1 - [RS10_HUMAN]
P55884	Eukaryotic translation initiation factor 3 subunit B OS=Homo sapiens GN=EIF3B PE=1 SV=3 - [EIF3B_HUMAN]
P23919	Thymidylate kinase OS=Homo sapiens GN=DTYMK PE=1 SV=4 - [KTHY_HUMAN]
O94813	Slit homolog 2 protein OS=Homo sapiens GN=SLIT2 PE=1 SV=1 - [SLIT2_HUMAN]
P01892	HLA class I histocompatibility antigen, A-2 alpha chain OS=Homo sapiens GN=HLA-A PE=1 SV=1 - [1A02_HUMAN]
P01116	GTPase KRas OS=Homo sapiens GN=KRAS PE=1 SV=1 - [RASK_HUMAN]
Q9BQ16	Testican-3 OS=Homo sapiens GN=SPOCK3 PE=1 SV=2 - [TICN3_HUMAN]
Q9Y4K3	TNF receptor-associated factor 6 OS=Homo sapiens GN=TRAF6 PE=1 SV=1 - [TRAF6_HUMAN]
Q13085	Acetyl-CoA carboxylase 1 OS=Homo sapiens GN=ACACA PE=1 SV=2 - [ACACA_HUMAN]
P08133	Annexin A6 OS=Homo sapiens GN=ANXA6 PE=1 SV=3 - [ANXA6_HUMAN]
Q14558	Phosphoribosyl pyrophosphate synthase-associated protein 1 OS=Homo sapiens GN=PRPSAP1 PE=1 SV=2 - [KPRA_HUMAN]
Q86UD1	Out at first protein homolog OS=Homo sapiens GN=OAF PE=2 SV=1 - [OAF_HUMAN]
Q96L92	Sorting nexin-27 OS=Homo sapiens GN=SNX27 PE=1 SV=2 - [SNX27_HUMAN]
P62277	40S ribosomal protein S13 OS=Homo sapiens GN=RPS13 PE=1 SV=2 - [RS13_HUMAN]
O95816	BAG family molecular chaperone regulator 2 OS=Homo sapiens GN=BAG2 PE=1 SV=1 - [BAG2_HUMAN]
P49736	DNA replication licensing factor MCM2 OS=Homo sapiens GN=MCM2 PE=1 SV=4 - [MCM2_HUMAN]
P22087	rRNA 2'-O-methyltransferase fibrillarin OS=Homo sapiens GN=FBL PE=1 SV=2 - [FBRL_HUMAN]
P83436	Conserved oligomeric Golgi complex subunit 7 OS=Homo sapiens GN=COG7 PE=1 SV=1 - [COG7_HUMAN]
P29144	Tripeptidyl-peptidase 2 OS=Homo sapiens GN=TPP2 PE=1 SV=4 - [TPP2_HUMAN]
P08195	4F2 cell-surface antigen heavy chain OS=Homo sapiens GN=SLC3A2 PE=1 SV=3 - [4F2_HUMAN]
Q92598	Heat shock protein 105 kDa OS=Homo sapiens GN=HSPH1 PE=1 SV=1 - [HS105_HUMAN]
Q9Y223	Bifunctional UDP-N-acetylglucosamine 2-epimerase/N-acetylmannosamine kinase OS=Homo sapiens GN=GNE PE=1 SV=1 - [GLCNE_HUMAN]
O60232	Sjoegren syndrome/scleroderma autoantigen 1 OS=Homo sapiens GN=SSSCA1 PE=1 SV=1 - [SSA27_HUMAN]
Q9UI12	V-type proton ATPase subunit H OS=Homo sapiens GN=ATP6V1H PE=1 SV=1 - [VATH_HUMAN]
P08582	Melanotransferrin OS=Homo sapiens GN=MFI2 PE=1 SV=2 - [TRFM_HUMAN]
Q9NUP9	Protein lin-7 homolog C OS=Homo sapiens GN=LIN7C PE=1 SV=1 - [LIN7C_HUMAN]
O43396	Thioredoxin-like protein 1 OS=Homo sapiens GN=TXNL1 PE=1 SV=3 - [TXNL1_HUMAN]
Q13045	Protein flightless-1 homolog OS=Homo sapiens GN=FLII PE=1 SV=2 - [FLII_HUMAN]
Q04760	Lactoylglutathione lyase OS=Homo sapiens GN=GLO1 PE=1 SV=4 - [LGUL_HUMAN]
P14866	Heterogeneous nuclear ribonucleoprotein L OS=Homo sapiens GN=HNRNPL PE=1 SV=2 - [HNRPL_HUMAN]
P48739	Phosphatidylinositol transfer protein beta isoform OS=Homo sapiens GN=PITPNB PE=1 SV=2 - [PIPNB_HUMAN]
Q14566	DNA replication licensing factor MCM6 OS=Homo sapiens GN=MCM6 PE=1 SV=1 - [MCM6_HUMAN]
Q15599	Na(+)/H(+) exchange regulatory cofactor NHE-RF2 OS=Homo sapiens GN=SLC9A3R2 PE=1 SV=2 - [NHRF2_HUMAN]
Q92945	Far upstream element-binding protein 2 OS=Homo sapiens GN=KHSRP PE=1 SV=4 - [FUBP2_HUMAN]
O94985	Calsyntenin-1 OS=Homo sapiens GN=CLSTN1 PE=1 SV=1 - [CSTN1_HUMAN]
P40938	Replication factor C subunit 3 OS=Homo sapiens GN=RFC3 PE=1 SV=2 - [RFC3_HUMAN]
Q9H9A6	Leucine-rich repeat-containing protein 40 OS=Homo sapiens GN=LRRC40 PE=1 SV=1 - [LRC40_HUMAN]
P07339	Cathepsin D OS=Homo sapiens GN=CTSD PE=1 SV=1 - [CATD_HUMAN]
P51452	Dual specificity protein phosphatase 3 OS=Homo sapiens GN=DUSP3 PE=1 SV=1 - [DUS3_HUMAN]
Q86W42	THO complex subunit 6 homolog OS=Homo sapiens GN=THOC6 PE=1 SV=1 - [THOC6_HUMAN]
Q9BUI4	DNA-directed RNA polymerase III subunit RPC3 OS=Homo sapiens GN=POLR3C PE=1 SV=1 - [RPC3_HUMAN]
P62314	Small nuclear ribonucleoprotein Sm D1 OS=Homo sapiens GN=SNRPD1 PE=1 SV=1 - [SMD1_HUMAN]
P62750	60S ribosomal protein L23a OS=Homo sapiens GN=RPL23A PE=1 SV=1 - [RL23A_HUMAN]
P35579	Myosin-9 OS=Homo sapiens GN=MYH9 PE=1 SV=4 - [MYH9_HUMAN]
Q86VP6	Cullin-associated NEDD8-dissociated protein 1 OS=Homo sapiens GN=CAND1 PE=1 SV=2 - [CAND1_HUMAN]
Q92616	Translational activator GCN1 OS=Homo sapiens GN=GCN1L1 PE=1 SV=6 - [GCN1L_HUMAN]
P29401	Transketolase OS=Homo sapiens GN=TKT PE=1 SV=3 - [TKT_HUMAN]
Q06203	Amidophosphoribosyltransferase OS=Homo sapiens GN=PPAT PE=1 SV=1 - [PUR1_HUMAN]
Q8TD19	Serine/threonine-protein kinase Nek9 OS=Homo sapiens GN=NEK9 PE=1 SV=2 - [NEK9_HUMAN]
Q9UI10	Translation initiation factor eIF-2B subunit delta OS=Homo sapiens GN=EIF2B4 PE=1 SV=2 - [EI2BD_HUMAN]
O60493	Sorting nexin-3 OS=Homo sapiens GN=SNX3 PE=1 SV=3 - [SNX3_HUMAN]
P31943	Heterogeneous nuclear ribonucleoprotein H OS=Homo sapiens GN=HNRNPH1 PE=1 SV=4 - [HNRH1_HUMAN]
P05023	Sodium/potassium-transporting ATPase subunit alpha-1 OS=Homo sapiens GN=ATP1A1 PE=1 SV=1 - [AT1A1 HUMAN]
P42285	Superkiller viralicidic activity 2-like 2 OS=Homo sapiens GN=SKIV2L2 PE=1 SV=3 - [SK2L2_HUMAN]
P62851	40S ribosomal protein S25 OS=Homo sapiens GN=RPS25 PE=1 SV=1 - [RS25_HUMAN]
Q15003	Condensin complex subunit 2 OS=Homo sapiens GN=NCAPH PE=1 SV=3 - [CND2_HUMAN]
P13987	CD59 glycoprotein OS=Homo sapiens GN=CD59 PE=1 SV=1 - [CD59_HUMAN]
P61088	Ubiquitin-conjugating enzyme E2 N OS=Homo sapiens GN=UBE2N PE=1 SV=1 - [UBE2N_HUMAN]
Q9UBI6	Guanine nucleotide-binding protein G(I)/G(S)/G(O) subunit gamma-12 OS=Homo sapiens GN=GNG12 PE=1 SV=3 - [GBG12_HUMAN]
O60701	UDP-glucose 6-dehydrogenase OS=Homo sapiens GN=UGDH PE=1 SV=1 - [UGDH_HUMAN]
Q9Y3C7	Mediator of RNA polymerase II transcription subunit 31 OS=Homo sapiens GN=MED31 PE=1 SV=1 - [MED31_HUMAN]
Q15654	Thyroid receptor-interacting protein 6 OS=Homo sapiens GN=TRIP6 PE=1 SV=3 - [TRIP6_HUMAN]
O00743	Serine/threonine-protein phosphatase 6 catalytic subunit OS=Homo sapiens GN=PPP6C PE=1 SV=1 - [PPP6_HUMAN]
P21266	Glutathione S-transferase Mu 3 OS=Homo sapiens GN=GSTM3 PE=1 SV=3 - [GSTM3_HUMAN]
Q13610	Periodic tryptophan protein 1 homolog OS=Homo sapiens GN=PWP1 PE=1 SV=1 - [PWP1_HUMAN]
P19021	Peptidyl-glycine alpha-amidating monooxygenase OS=Homo sapiens GN=PAM PE=1 SV=2 - [AMD_HUMAN]
P61254	60S ribosomal protein L26 OS=Homo sapiens GN=RPL26 PE=1 SV=1 - [RL26_HUMAN]
P28482	Mitogen-activated protein kinase 1 OS=Homo sapiens GN=MAPK1 PE=1 SV=3 - [MK01_HUMAN]
P02545	Prelamin-A/C OS=Homo sapiens GN=LMNA PE=1 SV=1 - [LMNA_HUMAN]
P36543	V-type proton ATPase subunit E 1 OS=Homo sapiens GN=ATP6V1E1 PE=1 SV=1 - [VATE1_HUMAN]

P12004	Proliferating cell nuclear antigen OS=Homo sapiens GN=PCNA PE=1 SV=1 - [PCNA_HUMAN]
P20839	Inosine-5'-monophosphate dehydrogenase 1 OS=Homo sapiens GN=IMPDH1 PE=1 SV=2 - [IMDH1_HUMAN]
Q9NSK0	Kinesin light chain 4 OS=Homo sapiens GN=KLC4 PE=1 SV=3 - [KLC4_HUMAN]
P56537	Eukaryotic translation initiation factor 6 OS=Homo sapiens GN=EIF6 PE=1 SV=1 - [IF6_HUMAN]
P11908	Ribose-phosphate pyrophosphokinase 2 OS=Homo sapiens GN=PRPS2 PE=1 SV=2 - [PRPS2_HUMAN]
Q15024	Exosome complex component RRP42 OS=Homo sapiens GN=EXOSC7 PE=1 SV=3 - [EXOS7_HUMAN]
Q13564	NEDD8-activating enzyme E1 regulatory subunit OS=Homo sapiens GN=NAE1 PE=1 SV=1 - [ULA1_HUMAN]
Q16363	Laminin subunit alpha-4 OS=Homo sapiens GN=LAMA4 PE=1 SV=4 - [LAMA4_HUMAN]
O95757	Heat shock 70 kDa protein 4L OS=Homo sapiens GN=HSPA4L PE=1 SV=3 - [HS74L_HUMAN]
P62826	GTP-binding nuclear protein Ran OS=Homo sapiens GN=RAN PE=1 SV=3 - [RAN_HUMAN]
Q08J23	tRNA (cytosine(34)-C(5))-methyltransferase OS=Homo sapiens GN=NSUN2 PE=1 SV=2 - [NSUN2_HUMAN]
Q92621	Nuclear pore complex protein Nup205 OS=Homo sapiens GN=NUP205 PE=1 SV=3 - [NU205_HUMAN]
P61086	Ubiquitin-conjugating enzyme E2 K OS=Homo sapiens GN=UBE2K PE=1 SV=3 - [UBE2K_HUMAN]
P18621	60S ribosomal protein L17 OS=Homo sapiens GN=RPL17 PE=1 SV=3 - [RL17_HUMAN]
Q9Y3B2	Exosome complex component CSL4 OS=Homo sapiens GN=EXOSC1 PE=1 SV=1 - [EXOS1_HUMAN]
Q8TCG1	Protein CIP2A OS=Homo sapiens GN=KIAA1524 PE=1 SV=2 - [CIP2A_HUMAN]
Q96IJ6	Mannose-1-phosphate guanyltransferase alpha OS=Homo sapiens GN=GMPPA PE=1 SV=1 - [GMPPA_HUMAN]
Q9Y3Z3	Deoxynucleoside triphosphate triphosphohydrolase SAMHD1 OS=Homo sapiens GN=SAMHD1 PE=1 SV=2 - [SAMH1_HUMAN]
Q8TAT6	Nuclear protein localization protein 4 homolog OS=Homo sapiens GN=NPLOC4 PE=1 SV=3 - [NPL4_HUMAN]
Q99615	DnaJ homolog subfamily C member 7 OS=Homo sapiens GN=DNAJC7 PE=1 SV=2 - [DNJC7_HUMAN]
P26992	Ciliary neurotrophic factor receptor subunit alpha OS=Homo sapiens GN=CNTFR PE=1 SV=2 - [CNTFR_HUMAN]
P27105	Erythrocyte band 7 integral membrane protein OS=Homo sapiens GN=STOM PE=1 SV=3 - [STOM_HUMAN]
O95630	STAM-binding protein OS=Homo sapiens GN=STAMBP PE=1 SV=1 - [STABP_HUMAN]
P28799	Granulins OS=Homo sapiens GN=GRN PE=1 SV=2 - [GRN_HUMAN]
P49207	60S ribosomal protein L34 OS=Homo sapiens GN=RPL34 PE=1 SV=3 - [RL34_HUMAN]
Q9UHD1	Cysteine and histidine-rich domain-containing protein 1 OS=Homo sapiens GN=CHORDC1 PE=1 SV=2 - [CHRD1_HUMAN]
Q9Y277	Voltage-dependent anion-selective channel protein 3 OS=Homo sapiens GN=VDAC3 PE=1 SV=1 - [VDAC3_HUMAN]
P20339	Ras-related protein Rab-5A OS=Homo sapiens GN=RAB5A PE=1 SV=2 - [RAB5A_HUMAN]
P63151	Serine/threonine-protein phosphatase 2A 55 kDa regulatory subunit B alpha isoform OS=Homo sapiens GN=PPP2R2A PE=1 SV=1 - [2ABA_HUMAN]
Q96DI7	U5 small nuclear ribonucleoprotein 40 kDa protein OS=Homo sapiens GN=SNRNP40 PE=1 SV=1 - [SNR40_HUMAN]
Q9UQE7	Structural maintenance of chromosomes protein 3 OS=Homo sapiens GN=SMC3 PE=1 SV=2 - [SMC3_HUMAN]
Q96S59	Ran-binding protein 9 OS=Homo sapiens GN=RANBP9 PE=1 SV=1 - [RANB9_HUMAN]
Q9Y285	Phenylalanine--tRNA ligase alpha subunit OS=Homo sapiens GN=FARSA PE=1 SV=3 - [SYFA_HUMAN]
Q12792	Twinfilin-1 OS=Homo sapiens GN=TWF1 PE=1 SV=3 - [TWF1_HUMAN]
Q96J02	E3 ubiquitin-protein ligase Itchy homolog OS=Homo sapiens GN=ITCH PE=1 SV=2 - [ITCH_HUMAN]
O75607	Nucleoplasmin-3 OS=Homo sapiens GN=NPM3 PE=1 SV=3 - [NPM3_HUMAN]
Q12907	Vesicular integral-membrane protein VIP36 OS=Homo sapiens GN=LMAN2 PE=1 SV=1 - [LMAN2_HUMAN]
O14980	Exportin-1 OS=Homo sapiens GN=XPO1 PE=1 SV=1 - [XPO1_HUMAN]
Q15437	Protein transport protein Sec23B OS=Homo sapiens GN=SEC23B PE=1 SV=2 - [SC23B_HUMAN]
Q9ULT8	E3 ubiquitin-protein ligase HECTD1 OS=Homo sapiens GN=HECTD1 PE=1 SV=3 - [HECD1_HUMAN]
P28340	DNA polymerase delta catalytic subunit OS=Homo sapiens GN=POLD1 PE=1 SV=2 - [DPOD1_HUMAN]
P30153	Serine/threonine-protein phosphatase 2A 65 kDa regulatory subunit A alpha isoform OS=Homo sapiens GN=PPP2R1A PE=1 SV=4 - [2AAA_HUMAN]
Q6P1N0	Coiled-coil and C2 domain-containing protein 1A OS=Homo sapiens GN=CC2D1A PE=1 SV=1 - [C2D1A_HUMAN]
P30044	Peroxiredoxin-5, mitochondrial OS=Homo sapiens GN=PRDX5 PE=1 SV=4 - [PRDX5_HUMAN]
Q14203	Dynactin subunit 1 OS=Homo sapiens GN=DCTN1 PE=1 SV=3 - [DCTN1 HUMAN]
P60981	Destrin OS=Homo sapiens GN=DSTN PE=1 SV=3 - [DEST_HUMAN]
Q13740	CD166 antigen OS=Homo sapiens GN=ALCAM PE=1 SV=2 - [CD166_HUMAN]
Q13616	Cullin-1 OS=Homo sapiens GN=CUL1 PE=1 SV=2 - [CUL1_HUMAN]
Q14746	Conserved oligomeric Golgi complex subunit 2 OS=Homo sapiens GN=COG2 PE=1 SV=1 - [COG2_HUMAN]
Q9UNE7	E3 ubiquitin-protein ligase CHIP OS=Homo sapiens GN=STUB1 PE=1 SV=2 - [CHIP_HUMAN]
Q9H9T3	Elongator complex protein 3 OS=Homo sapiens GN=ELP3 PE=1 SV=2 - [ELP3_HUMAN]
Q96RS6	NudC domain-containing protein 1 OS=Homo sapiens GN=NUDCD1 PE=1 SV=2 - [NUDC1_HUMAN]
P61923	Coatomer subunit zeta-1 OS=Homo sapiens GN=COPZ1 PE=1 SV=1 - [COPZ1_HUMAN]
Q9UMR2	ATP-dependent RNA helicase DDX19B OS=Homo sapiens GN=DDX19B PE=1 SV=1 - [DD19B_HUMAN]
P23284	Peptidyl-prolyl cis-trans isomerase B OS=Homo sapiens GN=PPIB PE=1 SV=2 - [PPIB_HUMAN]
P47756	F-actin-capping protein subunit beta OS=Homo sapiens GN=CAPZB PE=1 SV=4 - [CAPZB_HUMAN]
O95983	Methyl-CpG-binding domain protein 3 OS=Homo sapiens GN=MBD3 PE=1 SV=1 - [MBD3_HUMAN]
P26640	Valine--tRNA ligase OS=Homo sapiens GN=VARS PE=1 SV=4 - [SYVC_HUMAN]
P35555	Fibrillin-1 OS=Homo sapiens GN=FBN1 PE=1 SV=3 - [FBN1_HUMAN]
Q92466	DNA damage-binding protein 2 OS=Homo sapiens GN=DDB2 PE=1 SV=1 - [DDB2_HUMAN]
Q13098	COP9 signalosome complex subunit 1 OS=Homo sapiens GN=GPS1 PE=1 SV=4 - [CSN1_HUMAN]
Q9H3K6	BolA-like protein 2 OS=Homo sapiens GN=BOLA2 PE=1 SV=1 - [BOLA2_HUMAN]
Q96KK5	Histone H2A type 1-H OS=Homo sapiens GN=HIST1H2AH PE=1 SV=3 - [H2A1H_HUMAN]
Q92905	COP9 signalosome complex subunit 5 OS=Homo sapiens GN=COPS5 PE=1 SV=4 - [CSN5_HUMAN]
Q15084	Protein disulfide-isomerase A6 OS=Homo sapiens GN=PDIA6 PE=1 SV=1 - [PDIA6_HUMAN]
Q7Z3B4	Nucleoporin p54 OS=Homo sapiens GN=NUP54 PE=1 SV=2 - [NUP54_HUMAN]
Q13330	Metastasis-associated protein MTA1 OS=Homo sapiens GN=MTA1 PE=1 SV=2 - [MTA1_HUMAN]
P27694	Replication protein A 70 kDa DNA-binding subunit OS=Homo sapiens GN=RPA1 PE=1 SV=2 - [RFA1_HUMAN]
P63173	60S ribosomal protein L38 OS=Homo sapiens GN=RPL38 PE=1 SV=2 - [RL38_HUMAN]
Q8WV22	Non-structural maintenance of chromosomes element 1 homolog OS=Homo sapiens GN=NSMCE1 PE=1 SV=5 - [NSE1_HUMAN]
P48059	LIM and senescent cell antigen-like-containing domain protein 1 OS=Homo sapiens GN=LIMS1 PE=1 SV=4 - [LIMS1_HUMAN]
Q9Y312	Protein AAR2 homolog OS=Homo sapiens GN=AAR2 PE=1 SV=2 - [AAR2 HUMAN]
P61962	DDB1- and CUL4-associated factor 7 OS=Homo sapiens GN=DCAF7 PE=1 SV=1 - [DCAF7_HUMAN]
Q96J84	Kin of IRRE-like protein 1 OS=Homo sapiens GN=KIRREL PE=1 SV=2 - [KIRR1_HUMAN]
Q92769	Histone deacetylase 2 OS=Homo sapiens GN=HDAC2 PE=1 SV=2 - [HDAC2_HUMAN]
Q07020	60S ribosomal protein L18 OS=Homo sapiens GN=RPL18 PE=1 SV=2 - [RL18_HUMAN]
P10909	Clusterin OS=Homo sapiens GN=CLU PE=1 SV=1 - [CLUS_HUMAN]
Q9BZL1	Ubiquitin-like protein 5 OS=Homo sapiens GN=UBL5 PE=1 SV=1 - [UBL5_HUMAN]
Q15370	Transcription elongation factor B polypeptide 2 OS=Homo sapiens GN=TCEB2 PE=1 SV=1 - [ELOB_HUMAN]
P12814	Alpha-actinin-1 OS=Homo sapiens GN=ACTN1 PE=1 SV=2 - [ACTN1_HUMAN]
Q14344	Guanine nucleotide-binding protein subunit alpha-13 OS=Homo sapiens GN=GNA13 PE=1 SV=2 - [GNA13_HUMAN]
Q15020	Squamous cell carcinoma antigen recognized by T-cells 3 OS=Homo sapiens GN=SART3 PE=1 SV=1 - [SART3_HUMAN]
Q7Z2W4	Zinc finger CCCH-type antiviral protein 1 OS=Homo sapiens GN=ZC3HAV1 PE=1 SV=3 - [ZCCHV_HUMAN]
Q9BSJ2	Gamma-tubulin complex component 2 OS=Homo sapiens GN=TUBGCP2 PE=1 SV=2 - [GCP2_HUMAN]
Q9Y5Q8	General transcription factor 3C polypeptide 5 OS=Homo sapiens GN=GTF3C5 PE=1 SV=2 - [TF3C5_HUMAN]
P09525	Annexin A4 OS=Homo sapiens GN=ANXA4 PE=1 SV=4 - [ANXA4_HUMAN]
O00425	Insulin-like growth factor 2 mRNA-binding protein 3 OS=Homo sapiens GN=IGF2BP3 PE=1 SV=2 - [IF2B3 HUMAN]
Q9BZK7	F-box-like/WD repeat-containing protein TBL1XR1 OS=Homo sapiens GN=TBL1XR1 PE=1 SV=1 - [TBL1R_HUMAN]
Q5ZPR3	CD276 antigen OS=Homo sapiens GN=CD276 PE=1 SV=1 - [CD276_HUMAN]
P04908	Histone H2A type 1-B/E OS=Homo sapiens GN=HIST1H2AB PE=1 SV=2 - [H2A1B_HUMAN]
Q96C90	Protein phosphatase 1 regulatory subunit 14B OS=Homo sapiens GN=PPP1R14B PE=1 SV=3 - [PP14B_HUMAN]
O75351	Vacuolar protein sorting-associated protein 4B OS=Homo sapiens GN=VPS4B PE=1 SV=2 - [VPS4B_HUMAN]
P61224	Ras-related protein Rap-1b OS=Homo sapiens GN=RAP1B PE=1 SV=1 - [RAP1B_HUMAN]
P62834	Ras-related protein Rap-1A OS=Homo sapiens GN=RAP1A PE=1 SV=1 - [RAP1A_HUMAN]
P41250	Glycine--tRNA ligase OS=Homo sapiens GN=GARS PE=1 SV=3 - [SYG_HUMAN]
P48729	Casein kinase I isoform alpha OS=Homo sapiens GN=CSNK1A1 PE=1 SV=2 - [KC1A_HUMAN]
O60684	Importin subunit alpha-7 OS=Homo sapiens GN=KPNA6 PE=1 SV=1 - [IMA7_HUMAN]
P04062	Glucosylceramidase OS=Homo sapiens GN=GBA PE=1 SV=3 - [GLCM_HUMAN]
P62166	Neuronal calcium sensor 1 OS=Homo sapiens GN=NCS1 PE=1 SV=2 - [NCS1_HUMAN]
P23921	Ribonucleoside-diphosphate reductase large subunit OS=Homo sapiens GN=RRM1 PE=1 SV=1 - [RIR1_HUMAN]
Q9UNS2	COP9 signalosome complex subunit 3 OS=Homo sapiens GN=COPS3 PE=1 SV=3 - [CSN3_HUMAN]
Q13137	Calcium-binding and coiled-coil domain-containing protein 2 OS=Homo sapiens GN=CALCOCO2 PE=1 SV=1 - [CACO2_HUMAN]
Q92599	Septin-8 OS=Homo sapiens GN=SEPT8 PE=1 SV=4 - [SEPT8_HUMAN]
P50454	Serpin H1 OS=Homo sapiens GN=SERPINH1 PE=1 SV=2 - [SERPH_HUMAN]
P08253	72 kDa type IV collagenase OS=Homo sapiens GN=MMP2 PE=1 SV=2 - [MMP2_HUMAN]
O00186	Syntaxin-binding protein 3 OS=Homo sapiens GN=STXBP3 PE=1 SV=2 - [STXB3_HUMAN]
P61626	Lysozyme C OS=Homo sapiens GN=LYZ PE=1 SV=1 - [LYSC_HUMAN]
Q01780	Exosome component 10 OS=Homo sapiens GN=EXOSC10 PE=1 SV=2 - [EXOSX_HUMAN]
Q96MW5	Conserved oligomeric Golgi complex subunit 8 OS=Homo sapiens GN=COG8 PE=1 SV=2 - [COG8_HUMAN]
Q8TEQ6	Gem-associated protein 5 OS=Homo sapiens GN=GEMIN5 PE=1 SV=3 - [GEMI5_HUMAN]
Q9UBU9	Nuclear RNA export factor 1 OS=Homo sapiens GN=NXF1 PE=1 SV=1 - [NXF1_HUMAN]
Q9BTE3	Mini-chromosome maintenance complex-binding protein OS=Homo sapiens GN=MCMBP PE=1 SV=2 - [MCMBP_HUMAN]
O60488	Long-chain-fatty-acid--CoA ligase 4 OS=Homo sapiens GN=ACSL4 PE=1 SV=2 - [ACSL4_HUMAN]
Q9NS15	Latent-transforming growth factor beta-binding protein 3 OS=Homo sapiens GN=LTBP3 PE=1 SV=4 - [LTBP3_HUMAN]
Q9NQG5	Regulation of nuclear pre-mRNA domain-containing protein 1B OS=Homo sapiens GN=RPRD1B PE=1 SV=1 - [RPR1B_HUMAN]

O94973	AP-2 complex subunit alpha-2 OS=Homo sapiens GN=AP2A2 PE=1 SV=2 - [AP2A2_HUMAN]
Q99627	COP9 signalosome complex subunit 8 OS=Homo sapiens GN=COPS8 PE=1 SV=1 - [CSN8_HUMAN]
Q9NWA0	Mediator of RNA polymerase II transcription subunit 9 OS=Homo sapiens GN=MED9 PE=1 SV=1 - [MED9_HUMAN]
Q9Y4L1	Hypoxia up-regulated protein 1 OS=Homo sapiens GN=HYOU1 PE=1 SV=1 - [HYOU1_HUMAN]
Q4LDE5	Sushi, von Willebrand factor type A, EGF and pentraxin domain-containing protein 1 OS=Homo sapiens GN=SVEP1 PE=1 SV=3 - [SVEP1_HUMAN]
O43747	AP-1 complex subunit gamma-1 OS=Homo sapiens GN=AP1G1 PE=1 SV=5 - [AP1G1_HUMAN]
O75787	Renin receptor OS=Homo sapiens GN=ATP6AP2 PE=1 SV=2 - [RENR_HUMAN]
Q9NP79	Vacuolar protein sorting-associated protein VTA1 homolog OS=Homo sapiens GN=VTA1 PE=1 SV=1 - [VTA1_HUMAN]
Q9NZI8	Insulin-like growth factor 2 mRNA-binding protein 1 OS=Homo sapiens GN=IGF2BP1 PE=1 SV=2 - [IF2B1 HUMAN]
Q9GZQ3	COMM domain-containing protein 5 OS=Homo sapiens GN=COMMD5 PE=1 SV=1 - [COMD5_HUMAN]
P31689	DnaJ homolog subfamily A member 1 OS=Homo sapiens GN=DNAJA1 PE=1 SV=2 - [DNJA1_HUMAN]
P84103	Serine/arginine-rich splicing factor 3 OS=Homo sapiens GN=SRSF3 PE=1 SV=1 - [SRSF3_HUMAN]
Q5BLP8	Neuropeptide-like protein C4orf48 OS=Homo sapiens GN=C4orf48 PE=1 SV=3 - [CD048_HUMAN]
Q9UPU5	Ubiquitin carboxyl-terminal hydrolase 24 OS=Homo sapiens GN=USP24 PE=1 SV=3 - [UBP24_HUMAN]
O95602	DNA-directed RNA polymerase I subunit RPA1 OS=Homo sapiens GN=POLR1A PE=1 SV=2 - [RPA1_HUMAN]
Q969J3	Loss of heterozygosity 12 chromosomal region 1 protein OS=Homo sapiens GN=LOH12CR1 PE=1 SV=1 - [L12R1_HUMAN]
P15311	Ezrin OS=Homo sapiens GN=EZR PE=1 SV=4 - [EZRI_HUMAN]
P36405	ADP-ribosylation factor-like protein 3 OS=Homo sapiens GN=ARL3 PE=1 SV=2 - [ARL3_HUMAN]
P35052	Glypican-1 OS=Homo sapiens GN=GPC1 PE=1 SV=2 - [GPC1_HUMAN]
P62847	40S ribosomal protein S24 OS=Homo sapiens GN=RPS24 PE=1 SV=1 - [RS24_HUMAN]
Q93008	Probable ubiquitin carboxyl-terminal hydrolase FAF-X OS=Homo sapiens GN=USP9X PE=1 SV=3 - [USP9X_HUMAN]
P15104	Glutamine synthetase OS=Homo sapiens GN=GLUL PE=1 SV=4 - [GLNA HUMAN]
Q15291	Retinoblastoma-binding protein 5 OS=Homo sapiens GN=RBBP5 PE=1 SV=2 - [RBBP5_HUMAN]
P05141	ADP/ATP translocase 2 OS=Homo sapiens GN=SLC25A5 PE=1 SV=7 - [ADT2_HUMAN]
Q7L576	Cytoplasmic FMR1-interacting protein 1 OS=Homo sapiens GN=CYFIP1 PE=1 SV=1 - [CYFP1_HUMAN]
P63092	Guanine nucleotide-binding protein G(s) subunit alpha isoforms short OS=Homo sapiens GN=GNAS PE=1 SV=1 - [GNAS2_HUMAN]
P63027	Vesicle-associated membrane protein 2 OS=Homo sapiens GN=VAMP2 PE=1 SV=3 - [VAMP2_HUMAN]
O60825	6-phosphofructo-2-kinase/fructose-2,6-bisphosphatase 2 OS=Homo sapiens GN=PFKFB2 PE=1 SV=2 - [F262_HUMAN]
A5YKK6	CCR4-NOT transcription complex subunit 1 OS=Homo sapiens GN=CNOT1 PE=1 SV=2 - [CNOT1_HUMAN]
Q9HAN9	Nicotinamide/nicotinic acid mononucleotide adenylyltransferase 1 OS=Homo sapiens GN=NMNAT1 PE=1 SV=1 - [NMNA1_HUMAN]
P53990	IST1 homolog OS=Homo sapiens GN=IST1 PE=1 SV=1 - [IST1_HUMAN]
Q9C005	Protein dpy-30 homolog OS=Homo sapiens GN=DPY30 PE=1 SV=1 - [DPY30_HUMAN]
P05455	Lupus La protein OS=Homo sapiens GN=SSB PE=1 SV=2 - [LA_HUMAN]
O75153	Clustered mitochondria protein homolog OS=Homo sapiens GN=CLUH PE=1 SV=2 - [CLU_HUMAN]
P05067	Amyloid beta A4 protein OS=Homo sapiens GN=APP PE=1 SV=3 - [A4 HUMAN]
Q13418	Integrin-linked protein kinase OS=Homo sapiens GN=ILK PE=1 SV=2 - [ILK_HUMAN]
Q15056	Eukaryotic translation initiation factor 4H OS=Homo sapiens GN=EIF4H PE=1 SV=5 - [IF4H_HUMAN]
Q96EK6	Glucosamine 6-phosphate N-acetyltransferase OS=Homo sapiens GN=GNPNAT1 PE=1 SV=1 - [GNA1_HUMAN]
Q9NRW3	DNA dC->dU-editing enzyme APOBEC-3C OS=Homo sapiens GN=APOBEC3C PE=1 SV=2 - [ABC3C_HUMAN]
O43681	ATPase ASNA1 OS=Homo sapiens GN=ASNA1 PE=1 SV=2 - [ASNA_HUMAN]
Q8N5N7	39S ribosomal protein L50, mitochondrial OS=Homo sapiens GN=MRPL50 PE=1 SV=2 - [RM50_HUMAN]
Q9Y5U9	Immediate early response 3-interacting protein 1 OS=Homo sapiens GN=IER3IP1 PE=1 SV=1 - [IR3IP_HUMAN]
Q12974	Protein tyrosine phosphatase type IVA 2 OS=Homo sapiens GN=PTP4A2 PE=1 SV=1 - [TP4A2_HUMAN]
Q14145	Kelch-like ECH-associated protein 1 OS=Homo sapiens GN=KEAP1 PE=1 SV=2 - [KEAP1_HUMAN]
Q8NCA5	Protein FAM98A OS=Homo sapiens GN=FAM98A PE=1 SV=1 - [FA98A_HUMAN]
Q01469	Fatty acid-binding protein, epidermal OS=Homo sapiens GN=FABP5 PE=1 SV=3 - [FABP5_HUMAN]
O43660	Pleiotropic regulator 1 OS=Homo sapiens GN=PLRG1 PE=1 SV=1 - [PLRG1_HUMAN]
Q96QK1	Vacuolar protein sorting-associated protein 35 OS=Homo sapiens GN=VPS35 PE=1 SV=2 - [VPS35_HUMAN]
Q12788	Transducin beta-like protein 3 OS=Homo sapiens GN=TBL3 PE=1 SV=2 - [TBL3_HUMAN]
Q9Y5B9	FACT complex subunit SPT16 OS=Homo sapiens GN=SUPT16H PE=1 SV=1 - [SP16H_HUMAN]
Q08945	FACT complex subunit SSRP1 OS=Homo sapiens GN=SSRP1 PE=1 SV=1 - [SSRP1_HUMAN]
Q86V97	Kelch repeat and BTB domain-containing protein 6 OS=Homo sapiens GN=KBTBD6 PE=1 SV=1 - [KBTB6_HUMAN]
Q15233	Non-POU domain-containing octamer-binding protein OS=Homo sapiens GN=NONO PE=1 SV=4 - [NONO_HUMAN]
O75534	Cold shock domain-containing protein E1 OS=Homo sapiens GN=CSDE1 PE=1 SV=2 - [CSDE1_HUMAN]
P53582	Methionine aminopeptidase 1 OS=Homo sapiens GN=METAP1 PE=1 SV=2 - [MAP11_HUMAN]
P12236	ADP/ATP translocase 3 OS=Homo sapiens GN=SLC25A6 PE=1 SV=4 - [ADT3_HUMAN]
Q00796	Sorbitol dehydrogenase OS=Homo sapiens GN=SORD PE=1 SV=4 - [DHSO_HUMAN]
Q9NUU7	ATP-dependent RNA helicase DDX19A OS=Homo sapiens GN=DDX19A PE=1 SV=1 - [DD19A_HUMAN]
Q9NW08	DNA-directed RNA polymerase III subunit RPC2 OS=Homo sapiens GN=POLR3B PE=1 SV=2 - [RPC2_HUMAN]
Q9H4F8	SPARC-related modular calcium-binding protein 1 OS=Homo sapiens GN=SMOC1 PE=1 SV=1 - [SMOC1_HUMAN]
P09874	Poly [ADP-ribose] polymerase 1 OS=Homo sapiens GN=PARP1 PE=1 SV=4 - [PARP1_HUMAN]
P37108	Signal recognition particle 14 kDa protein OS=Homo sapiens GN=SRP14 PE=1 SV=2 - [SRP14_HUMAN]
Q14766	Latent-transforming growth factor beta-binding protein 1 OS=Homo sapiens GN=LTBP1 PE=1 SV=4 - [LTBP1_HUMAN]
Q9UBT2	SUMO-activating enzyme subunit 2 OS=Homo sapiens GN=UBA2 PE=1 SV=2 - [SAE2_HUMAN]
P35241	Radixin OS=Homo sapiens GN=RDX PE=1 SV=1 - [RADI_HUMAN]
Q16629	Serine/arginine-rich splicing factor 7 OS=Homo sapiens GN=SRSF7 PE=1 SV=1 - [SRSF7_HUMAN]
Q15075	Early endosome antigen 1 OS=Homo sapiens GN=EEA1 PE=1 SV=2 - [EEA1_HUMAN]
Q13126	S-methyl-5'-thioadenosine phosphorylase OS=Homo sapiens GN=MTAP PE=1 SV=2 - [MTAP_HUMAN]
Q96JB2	Conserved oligomeric Golgi complex subunit 3 OS=Homo sapiens GN=COG3 PE=1 SV=3 - [COG3_HUMAN]
Q8NDZ4	Deleted in autism protein 1 OS=Homo sapiens GN=C3orf58 PE=1 SV=1 - [DIA1_HUMAN]
Q13242	Serine/arginine-rich splicing factor 9 OS=Homo sapiens GN=SRSF9 PE=1 SV=1 - [SRSF9_HUMAN]
Q9P0V9	Septin-10 OS=Homo sapiens GN=SEPT10 PE=1 SV=2 - [SEP10_HUMAN]
P52294	Importin subunit alpha-5 OS=Homo sapiens GN=KPNA1 PE=1 SV=3 - [IMA5_HUMAN]
O75487	Glypican-4 OS=Homo sapiens GN=GPC4 PE=1 SV=4 - [GPC4_HUMAN]
O43390	Heterogeneous nuclear ribonucleoprotein R OS=Homo sapiens GN=HNRNPR PE=1 SV=1 - [HNRPR_HUMAN]
Q04917	14-3-3 protein eta OS=Homo sapiens GN=YWHAH PE=1 SV=4 - [1433F HUMAN]
Q96EE3	Nucleoporin SEH1 OS=Homo sapiens GN=SEH1L PE=1 SV=3 - [SEH1_HUMAN]
Q96J01	THO complex subunit 3 OS=Homo sapiens GN=THOC3 PE=1 SV=1 - [THOC3_HUMAN]
Q99816	Tumor susceptibility gene 101 protein OS=Homo sapiens GN=TSG101 PE=1 SV=2 - [TS101_HUMAN]
Q9UKK9	ADP-sugar pyrophosphatase OS=Homo sapiens GN=NUDT5 PE=1 SV=1 - [NUDT5_HUMAN]
O00159	Unconventional myosin-Ic OS=Homo sapiens GN=MYO1C PE=1 SV=4 - [MYO1C_HUMAN]
P09936	Ubiquitin carboxyl-terminal hydrolase isozyme L1 OS=Homo sapiens GN=UCHL1 PE=1 SV=2 - [UCHL1_HUMAN]
Q14683	Structural maintenance of chromosomes protein 1A OS=Homo sapiens GN=SMC1A PE=1 SV=2 - [SMC1A_HUMAN]
O15084	Serine/threonine-protein phosphatase 6 regulatory ankyrin repeat subunit A OS=Homo sapiens GN=ANKRD28 PE=1 SV=5 - [ANR28_HUMAN]
P46778	60S ribosomal protein L21 OS=Homo sapiens GN=RPL21 PE=1 SV=2 - [RL21_HUMAN]
P56377	AP-1 complex subunit sigma-2 OS=Homo sapiens GN=AP1S2 PE=1 SV=1 - [AP1S2_HUMAN]
P57678	Gem-associated protein 4 OS=Homo sapiens GN=GEMIN4 PE=1 SV=2 - [GEMI4_HUMAN]
P20338	Ras-related protein Rab-4A OS=Homo sapiens GN=RAB4A PE=1 SV=3 - [RAB4A_HUMAN]
O95347	Structural maintenance of chromosomes protein 2 OS=Homo sapiens GN=SMC2 PE=1 SV=2 - [SMC2_HUMAN]
Q86Y82	Syntaxin-12 OS=Homo sapiens GN=STX12 PE=1 SV=1 - [STX12_HUMAN]
O95336	6-phosphogluconolactonase OS=Homo sapiens GN=PGLS PE=1 SV=2 - [6PGL_HUMAN]
P04843	Dolichyl-diphosphooligosaccharide--protein glycosyltransferase subunit 1 OS=Homo sapiens GN=RPN1 PE=1 SV=1 - [RPN1_HUMAN]
P43487	Ran-specific GTPase-activating protein OS=Homo sapiens GN=RANBP1 PE=1 SV=1 - [RANG_HUMAN]
Q01518	Adenylyl cyclase-associated protein 1 OS=Homo sapiens GN=CAP1 PE=1 SV=5 - [CAP1_HUMAN]
Q9HAS0	Protein Njmu-R1 OS=Homo sapiens GN=C17orf75 PE=1 SV=2 - [NJMU_HUMAN]
Q9UET6	Putative tRNA (cytidine(32)/guanosine(34)-2'-O)-methyltransferase OS=Homo sapiens GN=FTSJ1 PE=1 SV=2 - [TRM7_HUMAN]
P08237	ATP-dependent 6-phosphofructokinase, muscle type OS=Homo sapiens GN=PFKM PE=1 SV=2 - [PFKAM_HUMAN]
P55769	NHP2-like protein 1 OS=Homo sapiens GN=NHP2L1 PE=1 SV=3 - [NH2L1_HUMAN]
Q96KG9	N-terminal kinase-like protein OS=Homo sapiens GN=SCYL1 PE=1 SV=1 - [NTKL_HUMAN]
O43447	Peptidyl-prolyl cis-trans isomerase H OS=Homo sapiens GN=PPIH PE=1 SV=1 - [PPIH_HUMAN]
P47897	Glutamine--tRNA ligase OS=Homo sapiens GN=QARS PE=1 SV=1 - [SYQ_HUMAN]
O60462	Neuropilin-2 OS=Homo sapiens GN=NRP2 PE=1 SV=2 - [NRP2_HUMAN]
Q5T4S7	E3 ubiquitin-protein ligase UBR4 OS=Homo sapiens GN=UBR4 PE=1 SV=1 - [UBR4_HUMAN]
Q12933	TNF receptor-associated factor 2 OS=Homo sapiens GN=TRAF2 PE=1 SV=2 - [TRAF2_HUMAN]
Q9NXX6	Non-structural maintenance of chromosomes element 4 homolog A OS=Homo sapiens GN=NSMCE4A PE=1 SV=2 - [NSE4A_HUMAN]
Q9BPX5	Actin-related protein 2/3 complex subunit 5-like protein OS=Homo sapiens GN=ARPC5L PE=1 SV=1 - [ARP5L_HUMAN]
P13861	cAMP-dependent protein kinase type II-alpha regulatory subunit OS=Homo sapiens GN=PRKAR2A PE=1 SV=2 - [KAP2_HUMAN]
Q14126	Desmoglein-2 OS=Homo sapiens GN=DSG2 PE=1 SV=2 - [DSG2_HUMAN]
Q53GT1	Kelch-like protein 22 OS=Homo sapiens GN=KLHL22 PE=1 SV=2 - [KLH22_HUMAN]
Q9Y244	Proteasome maturation protein OS=Homo sapiens GN=POMP PE=1 SV=1 - [POMP_HUMAN]
P06396	Gelsolin OS=Homo sapiens GN=GSN PE=1 SV=1 - [GELS_HUMAN]
P46736	Lys-63-specific deubiquitinase BRCC36 OS=Homo sapiens GN=BRCC3 PE=1 SV=2 - [BRCC3_HUMAN]
P62910	60S ribosomal protein L32 OS=Homo sapiens GN=RPL32 PE=1 SV=2 - [RL32_HUMAN]
Q9Y2V7	Conserved oligomeric Golgi complex subunit 6 OS=Homo sapiens GN=COG6 PE=1 SV=2 - [COG6_HUMAN]
Q9NP72	Ras-related protein Rab-18 OS=Homo sapiens GN=RAB18 PE=1 SV=1 - [RAB18_HUMAN]

P45973	Chromobox protein homolog 5 OS=Homo sapiens GN=CBX5 PE=1 SV=1 - [CBX5_HUMAN]
P69905	Hemoglobin subunit alpha OS=Homo sapiens GN=HBA1 PE=1 SV=2 - [HBA_HUMAN]
Q9NXR7	BRCA1-A complex subunit BRE OS=Homo sapiens GN=BRE PE=1 SV=2 - [BRE_HUMAN]
Q9UL63	Muskelin OS=Homo sapiens GN=MKLN1 PE=1 SV=2 - [MKLN1_HUMAN]
Q9H900	Protein zwilch homolog OS=Homo sapiens GN=ZWILCH PE=1 SV=2 - [ZWILC_HUMAN]
P07099	Epoxide hydrolase 1 OS=Homo sapiens GN=EPHX1 PE=1 SV=1 - [HYEP_HUMAN]
P50148	Guanine nucleotide-binding protein G(q) subunit alpha OS=Homo sapiens GN=GNAQ PE=1 SV=4 - [GNAQ_HUMAN]
O75832	26S proteasome non-ATPase regulatory subunit 10 OS=Homo sapiens GN=PSMD10 PE=1 SV=1 - [PSD10_HUMAN]
Q00577	Transcriptional activator protein Pur-alpha OS=Homo sapiens GN=PURA PE=1 SV=2 - [PURA_HUMAN]
O00178	GTP-binding protein 1 OS=Homo sapiens GN=GTPBP1 PE=1 SV=3 - [GTPB1_HUMAN]
O15318	DNA-directed RNA polymerase III subunit RPC7 OS=Homo sapiens GN=POLR3G PE=1 SV=2 - [RPC7_HUMAN]
P34932	Heat shock 70 kDa protein 4 OS=Homo sapiens GN=HSPA4 PE=1 SV=4 - [HSP74_HUMAN]
Q9NT22	EMILIN-3 OS=Homo sapiens GN=EMILIN3 PE=2 SV=2 - [EMIL3_HUMAN]
O00471	Exocyst complex component 5 OS=Homo sapiens GN=EXOC5 PE=1 SV=1 - [EXOC5_HUMAN]
P30046	D-dopachrome decarboxylase OS=Homo sapiens GN=DDT PE=1 SV=3 - [DOPD_HUMAN]
Q6YHK3	CD109 antigen OS=Homo sapiens GN=CD109 PE=1 SV=2 - [CD109_HUMAN]
P09661	U2 small nuclear ribonucleoprotein A' OS=Homo sapiens GN=SNRPA1 PE=1 SV=2 - [RU2A_HUMAN]
Q9H0A8	COMM domain-containing protein 4 OS=Homo sapiens GN=COMMD4 PE=1 SV=1 - [COMD4_HUMAN]
P62753	40S ribosomal protein S6 OS=Homo sapiens GN=RPS6 PE=1 SV=1 - [RS6_HUMAN]
Q9H0E2	Toll-interacting protein OS=Homo sapiens GN=TOLLIP PE=1 SV=1 - [TOLIP_HUMAN]
P13489	Ribonuclease inhibitor OS=Homo sapiens GN=RNH1 PE=1 SV=2 - [RINI_HUMAN]
P09429	High mobility group protein B1 OS=Homo sapiens GN=HMGB1 PE=1 SV=3 - [HMGB1_HUMAN]
P55795	Heterogeneous nuclear ribonucleoprotein H2 OS=Homo sapiens GN=HNRNPH2 PE=1 SV=1 - [HNRH2_HUMAN]
Q32P28	Prolyl 3-hydroxylase 1 OS=Homo sapiens GN=LEPRE1 PE=1 SV=2 - [P3H1_HUMAN]
Q9HAV0	Guanine nucleotide-binding protein subunit beta-4 OS=Homo sapiens GN=GNB4 PE=1 SV=3 - [GBB4_HUMAN]
O00629	Importin subunit alpha-3 OS=Homo sapiens GN=KPNA4 PE=1 SV=1 - [IMA3_HUMAN]
P19387	DNA-directed RNA polymerase II subunit RPB3 OS=Homo sapiens GN=POLR2C PE=1 SV=2 - [RPB3_HUMAN]
P41240	Tyrosine-protein kinase CSK OS=Homo sapiens GN=CSK PE=1 SV=1 - [CSK_HUMAN]
Q99733	Nucleosome assembly protein 1-like 4 OS=Homo sapiens GN=NAP1L4 PE=1 SV=1 - [NP1L4_HUMAN]
O75533	Splicing factor 3B subunit 1 OS=Homo sapiens GN=SF3B1 PE=1 SV=3 - [SF3B1_HUMAN]
Q9BY43	Charged multivesicular body protein 4a OS=Homo sapiens GN=CHMP4A PE=1 SV=3 - [CHM4A_HUMAN]
Q92973	Transportin-1 OS=Homo sapiens GN=TNPO1 PE=1 SV=2 - [TNPO1_HUMAN]
P30154	Serine/threonine-protein phosphatase 2A 65 kDa regulatory subunit A beta isoform OS=Homo sapiens GN=PPP2R1B PE=1 SV=3 - [2AAB_HUMAN]
Q9NR12	PDZ and LIM domain protein 7 OS=Homo sapiens GN=PDLIM7 PE=1 SV=1 - [PDLI7_HUMAN]
P41223	Protein BUD31 homolog OS=Homo sapiens GN=BUD31 PE=1 SV=2 - [BUD31_HUMAN]
P35580	Myosin-10 OS=Homo sapiens GN=MYH10 PE=1 SV=3 - [MYH10_HUMAN]
Q9Y314	Nitric oxide synthase-interacting protein OS=Homo sapiens GN=NOSIP PE=1 SV=1 - [NOSIP_HUMAN]
P11172	Uridine 5'-monophosphate synthase OS=Homo sapiens GN=UMPS PE=1 SV=1 - [UMPS_HUMAN]
P61201	COP9 signalosome complex subunit 2 OS=Homo sapiens GN=COPS2 PE=1 SV=1 - [CSN2_HUMAN]
P67809	Nuclease-sensitive element-binding protein 1 OS=Homo sapiens GN=YBX1 PE=1 SV=3 - [YBOX1_HUMAN]
Q93096	Protein tyrosine phosphatase type IVA 1 OS=Homo sapiens GN=PTP4A1 PE=1 SV=2 - [TP4A1_HUMAN]
A0AVF1	Intraflagellar transport protein 56 OS=Homo sapiens GN=TTC26 PE=2 SV=1 - [IFT56_HUMAN]
P46776	60S ribosomal protein L27a OS=Homo sapiens GN=RPL27A PE=1 SV=2 - [RL27A_HUMAN]
Q9UHB9	Signal recognition particle subunit SRP68 OS=Homo sapiens GN=SRP68 PE=1 SV=2 - [SRP68_HUMAN]
Q8WVV9	Heterogeneous nuclear ribonucleoprotein L-like OS=Homo sapiens GN=HNRNPLL PE=1 SV=1 - [HNRLL_HUMAN]
Q9H0F7	ADP-ribosylation factor-like protein 6 OS=Homo sapiens GN=ARL6 PE=1 SV=1 - [ARL6_HUMAN]
Q9NVJ2	ADP-ribosylation factor-like protein 8B OS=Homo sapiens GN=ARL8B PE=1 SV=1 - [ARL8B_HUMAN]
P43246	DNA mismatch repair protein Msh2 OS=Homo sapiens GN=MSH2 PE=1 SV=1 - [MSH2_HUMAN]
O75818	Ribonuclease P protein subunit p40 OS=Homo sapiens GN=RPP40 PE=1 SV=3 - [RPP40_HUMAN]
Q9NW64	Pre-mRNA-splicing factor RBM22 OS=Homo sapiens GN=RBM22 PE=1 SV=1 - [RBM22_HUMAN]
Q96GM5	SWI/SNF-related matrix-associated actin-dependent regulator of chromatin subfamily D member 1 OS=Homo sapiens GN=SMARCD1 PE=1 SV=2 - [SMRD1_HUMAN]
Q96IV0	Peptide-N(4)-(N-acetyl-beta-glucosaminyl)asparagine amidase OS=Homo sapiens GN=NGLY1 PE=1 SV=1 - [NGLY1_HUMAN]
Q9H944	Mediator of RNA polymerase II transcription subunit 20 OS=Homo sapiens GN=MED20 PE=1 SV=1 - [MED20_HUMAN]
Q9UBS4	DnaJ homolog subfamily B member 11 OS=Homo sapiens GN=DNAJB11 PE=1 SV=1 - [DJB11_HUMAN]
P00374	Dihydrofolate reductase OS=Homo sapiens GN=DHFR PE=1 SV=2 - [DYR_HUMAN]
P02788	Lactotransferrin OS=Homo sapiens GN=LTF PE=1 SV=6 - [TRFL_HUMAN]
Q14152	Eukaryotic translation initiation factor 3 subunit A OS=Homo sapiens GN=EIF3A PE=1 SV=1 - [EIF3A_HUMAN]
Q6P597	Kinesin light chain 3 OS=Homo sapiens GN=KLC3 PE=1 SV=2 - [KLC3_HUMAN]
Q9UKB1	F-box/WD repeat-containing protein 11 OS=Homo sapiens GN=FBXW11 PE=1 SV=1 - [FBW1B_HUMAN]
Q9NT62	Ubiquitin-like-conjugating enzyme ATG3 OS=Homo sapiens GN=ATG3 PE=1 SV=1 - [ATG3_HUMAN]
P24928	DNA-directed RNA polymerase II subunit RPB1 OS=Homo sapiens GN=POLR2A PE=1 SV=2 - [RPB1_HUMAN]
Q641Q3	Meteorin-like protein OS=Homo sapiens GN=METRNL PE=2 SV=1 - [METRL_HUMAN]
Q9H2M9	Rab3 GTPase-activating protein non-catalytic subunit OS=Homo sapiens GN=RAB3GAP2 PE=1 SV=1 - [RBGPR_HUMAN]
P41227	N-alpha-acetyltransferase 10 OS=Homo sapiens GN=NAA10 PE=1 SV=1 - [NAA10_HUMAN]
Q9NVX0	HAUS augmin-like complex subunit 2 OS=Homo sapiens GN=HAUS2 PE=1 SV=1 - [HAUS2_HUMAN]
P13667	Protein disulfide-isomerase A4 OS=Homo sapiens GN=PDIA4 PE=1 SV=2 - [PDIA4_HUMAN]
P21741	Midkine OS=Homo sapiens GN=MDK PE=1 SV=1 - [MK_HUMAN]
P52701	DNA mismatch repair protein Msh6 OS=Homo sapiens GN=MSH6 PE=1 SV=2 - [MSH6_HUMAN]
Q9P2R3	Rabankyrin-5 OS=Homo sapiens GN=ANKFY1 PE=1 SV=2 - [ANFY1_HUMAN]
P07910	Heterogeneous nuclear ribonucleoproteins C1/C2 OS=Homo sapiens GN=HNRNPC PE=1 SV=4 - [HNRPC_HUMAN]
P35250	Replication factor C subunit 2 OS=Homo sapiens GN=RFC2 PE=1 SV=3 - [RFC2_HUMAN]
P49770	Translation initiation factor eIF-2B subunit beta OS=Homo sapiens GN=EIF2B2 PE=1 SV=3 - [EI2BB_HUMAN]
Q6PJ69	Tripartite motif-containing protein 65 OS=Homo sapiens GN=TRIM65 PE=1 SV=3 - [TRI65_HUMAN]
P62854	40S ribosomal protein S26 OS=Homo sapiens GN=RPS26 PE=1 SV=3 - [RS26_HUMAN]
Q13425	Beta-2-syntrophin OS=Homo sapiens GN=SNTB2 PE=1 SV=1 - [SNTB2_HUMAN]
O00401	Neural Wiskott-Aldrich syndrome protein OS=Homo sapiens GN=WASL PE=1 SV=2 - [WASL_HUMAN]
Q63HQ2	Pikachurin OS=Homo sapiens GN=EGFLAM PE=1 SV=2 - [EGFLA_HUMAN]
Q92890	Ubiquitin fusion degradation protein 1 homolog OS=Homo sapiens GN=UFD1L PE=1 SV=3 - [UFD1_HUMAN]
Q9NVI1	Fanconi anemia group I protein OS=Homo sapiens GN=FANCI PE=1 SV=4 - [FANCI_HUMAN]
O00622	Protein CYR61 OS=Homo sapiens GN=CYR61 PE=1 SV=1 - [CYR61_HUMAN]
Q14554	Protein disulfide-isomerase A5 OS=Homo sapiens GN=PDIA5 PE=1 SV=1 - [PDIA5_HUMAN]
P26358	DNA (cytosine-5)-methyltransferase 1 OS=Homo sapiens GN=DNMT1 PE=1 SV=2 - [DNMT1_HUMAN]
P50748	Kinetochore-associated protein 1 OS=Homo sapiens GN=KNTC1 PE=1 SV=1 - [KNTC1_HUMAN]
Q10570	Cleavage and polyadenylation specificity factor subunit 1 OS=Homo sapiens GN=CPSF1 PE=1 SV=2 - [CPSF1_HUMAN]
Q9H299	SH3 domain-binding glutamic acid-rich-like protein 3 OS=Homo sapiens GN=SH3BGRL3 PE=1 SV=1 - [SH3L3_HUMAN]
Q9Y5K8	V-type proton ATPase subunit D OS=Homo sapiens GN=ATP6V1D PE=1 SV=1 - [VATD_HUMAN]
Q13332	Receptor-type tyrosine-protein phosphatase S OS=Homo sapiens GN=PTPRS PE=1 SV=3 - [PTPRS_HUMAN]
Q13630	GDP-L-fucose synthase OS=Homo sapiens GN=TSTA3 PE=1 SV=1 - [FCL_HUMAN]
Q09161	Nuclear cap-binding protein subunit 1 OS=Homo sapiens GN=NCBP1 PE=1 SV=1 - [NCBP1_HUMAN]
Q15631	Translin OS=Homo sapiens GN=TSN PE=1 SV=1 - [TSN_HUMAN]
Q9Y508	E3 ubiquitin-protein ligase RNF114 OS=Homo sapiens GN=RNF114 PE=1 SV=1 - [RN114_HUMAN]
Q9NTJ3	Structural maintenance of chromosomes protein 4 OS=Homo sapiens GN=SMC4 PE=1 SV=2 - [SMC4_HUMAN]
Q9NZZ3	Charged multivesicular body protein 5 OS=Homo sapiens GN=CHMP5 PE=1 SV=1 - [CHMP5_HUMAN]
O14929	Histone acetyltransferase type B catalytic subunit OS=Homo sapiens GN=HAT1 PE=1 SV=1 - [HAT1_HUMAN]
P40429	60S ribosomal protein L13a OS=Homo sapiens GN=RPL13A PE=1 SV=2 - [RL13A_HUMAN]
Q9NRN5	Olfactomedin-like protein 3 OS=Homo sapiens GN=OLFML3 PE=2 SV=1 - [OLFL3_HUMAN]
Q15386	Ubiquitin-protein ligase E3C OS=Homo sapiens GN=UBE3C PE=1 SV=3 - [UBE3C_HUMAN]
Q15645	Pachytene checkpoint protein 2 homolog OS=Homo sapiens GN=TRIP13 PE=1 SV=2 - [PCH2_HUMAN]
Q9ULV4	Coronin-1C OS=Homo sapiens GN=CORO1C PE=1 SV=1 - [COR1C_HUMAN]
P59768	Guanine nucleotide-binding protein G(I)/G(S)/G(O) subunit gamma-2 OS=Homo sapiens GN=GNG2 PE=1 SV=2 - [GBG2_HUMAN]
Q2TAY7	WD40 repeat-containing protein SMU1 OS=Homo sapiens GN=SMU1 PE=1 SV=2 - [SMU1_HUMAN]
P04632	Calpain small subunit 1 OS=Homo sapiens GN=CAPNS1 PE=1 SV=1 - [CPNS1_HUMAN]
Q8NBZ7	UDP-glucuronic acid decarboxylase 1 OS=Homo sapiens GN=UXS1 PE=1 SV=1 - [UXS1_HUMAN]
O14802	DNA-directed RNA polymerase III subunit RPC1 OS=Homo sapiens GN=POLR3A PE=1 SV=2 - [RPC1_HUMAN]
O15145	Actin-related protein 2/3 complex subunit 3 OS=Homo sapiens GN=ARPC3 PE=1 SV=3 - [ARPC3_HUMAN]
O95456	Proteasome assembly chaperone 1 OS=Homo sapiens GN=PSMG1 PE=1 SV=1 - [PSMG1_HUMAN]
Q7Z478	ATP-dependent RNA helicase DHX29 OS=Homo sapiens GN=DHX29 PE=1 SV=2 - [DHX29_HUMAN]
P26599	Polypyrimidine tract-binding protein 1 OS=Homo sapiens GN=PTBP1 PE=1 SV=1 - [PTBP1_HUMAN]
Q99963	Endophilin-A3 OS=Homo sapiens GN=SH3GL3 PE=1 SV=1 - [SH3G3_HUMAN]
Q0PNE2	Elongator complex protein 6 OS=Homo sapiens GN=ELP6 PE=1 SV=1 - [ELP6_HUMAN]
O00203	AP-3 complex subunit beta-1 OS=Homo sapiens GN=AP3B1 PE=1 SV=3 - [AP3B1_HUMAN]
Q3MHD2	Protein LSM12 homolog OS=Homo sapiens GN=LSM12 PE=1 SV=2 - [LSM12_HUMAN]
Q9Y624	Junctional adhesion molecule A OS=Homo sapiens GN=F11R PE=1 SV=1 - [JAM1_HUMAN]
O75116	Rho-associated protein kinase 2 OS=Homo sapiens GN=ROCK2 PE=1 SV=4 - [ROCK2_HUMAN]
Q58EX2	Protein sidekick-2 OS=Homo sapiens GN=SDK2 PE=1 SV=3 - [SDK2_HUMAN]

O75054	Immunoglobulin superfamily member 3 OS=Homo sapiens GN=IGSF3 PE=2 SV=3 - [IGSF3_HUMAN]
Q96SI1	BTB/POZ domain-containing protein KCTD15 OS=Homo sapiens GN=KCTD15 PE=1 SV=1 - [KCD15_HUMAN]
O43795	Unconventional myosin-Ib OS=Homo sapiens GN=MYO1B PE=1 SV=3 - [MYO1B_HUMAN]
Q7KZ85	Transcription elongation factor SPT6 OS=Homo sapiens GN=SUPT6H PE=1 SV=2 - [SPT6H_HUMAN]
Q96L58	Beta-1,3-galactosyltransferase 6 OS=Homo sapiens GN=B3GALT6 PE=1 SV=2 - [B3GT6_HUMAN]
Q9NUD5	Zinc finger CCHC domain-containing protein 3 OS=Homo sapiens GN=ZCCHC3 PE=1 SV=1 - [ZCHC3_HUMAN]
P51812	Ribosomal protein S6 kinase alpha-3 OS=Homo sapiens GN=RPS6KA3 PE=1 SV=1 - [KS6A3_HUMAN]
Q9UKN8	General transcription factor 3C polypeptide 4 OS=Homo sapiens GN=GTF3C4 PE=1 SV=2 - [TF3C4_HUMAN]
P00966	Argininosuccinate synthase OS=Homo sapiens GN=ASS1 PE=1 SV=2 - [ASSY_HUMAN]
P06737	Glycogen phosphorylase, liver form OS=Homo sapiens GN=PYGL PE=1 SV=4 - [PYGL_HUMAN]
P83731	60S ribosomal protein L24 OS=Homo sapiens GN=RPL24 PE=1 SV=1 - [RL24_HUMAN]
Q13409	Cytoplasmic dynein 1 intermediate chain 2 OS=Homo sapiens GN=DYNC1I2 PE=1 SV=3 - [DC1I2_HUMAN]
Q9BPX3	Condensin complex subunit 3 OS=Homo sapiens GN=NCAPG PE=1 SV=1 - [CND3_HUMAN]
Q92734	Protein TFG OS=Homo sapiens GN=TFG PE=1 SV=2 - [TFG_HUMAN]
O95801	Tetratricopeptide repeat protein 4 OS=Homo sapiens GN=TTC4 PE=1 SV=3 - [TTC4_HUMAN]
P09211	Glutathione S-transferase P OS=Homo sapiens GN=GSTP1 PE=1 SV=2 - [GSTP1_HUMAN]
Q9Y3U8	60S ribosomal protein L36 OS=Homo sapiens GN=RPL36 PE=1 SV=3 - [RL36_HUMAN]
O43172	U4/U6 small nuclear ribonucleoprotein Prp4 OS=Homo sapiens GN=PRPF4 PE=1 SV=2 - [PRP4_HUMAN]
P20585	DNA mismatch repair protein Msh3 OS=Homo sapiens GN=MSH3 PE=1 SV=4 - [MSH3_HUMAN]
Q15382	GTP-binding protein Rheb OS=Homo sapiens GN=RHEB PE=1 SV=1 - [RHEB_HUMAN]
Q99623	Prohibitin-2 OS=Homo sapiens GN=PHB2 PE=1 SV=2 - [PHB2_HUMAN]
Q13131	5'-AMP-activated protein kinase catalytic subunit alpha-1 OS=Homo sapiens GN=PRKAA1 PE=1 SV=4 - [AAPK1_HUMAN]
P62899	60S ribosomal protein L31 OS=Homo sapiens GN=RPL31 PE=1 SV=1 - [RL31_HUMAN]
Q1KMD3	Heterogeneous nuclear ribonucleoprotein U-like protein 2 OS=Homo sapiens GN=HNRNPUL2 PE=1 SV=1 - [HNRL2_HUMAN]
Q8N3U4	Cohesin subunit SA-2 OS=Homo sapiens GN=STAG2 PE=1 SV=3 - [STAG2_HUMAN]
P31350	Ribonucleoside-diphosphate reductase subunit M2 OS=Homo sapiens GN=RRM2 PE=1 SV=1 - [RIR2_HUMAN]
O95084	Serine protease 23 OS=Homo sapiens GN=PRSS23 PE=2 SV=1 - [PRS23_HUMAN]
Q68CQ4	Digestive organ expansion factor homolog OS=Homo sapiens GN=DIEXF PE=1 SV=2 - [DIEXF_HUMAN]
P49458	Signal recognition particle 9 kDa protein OS=Homo sapiens GN=SRP9 PE=1 SV=2 - [SRP09_HUMAN]
Q14011	Cold-inducible RNA-binding protein OS=Homo sapiens GN=CIRBP PE=1 SV=1 - [CIRBP_HUMAN]
P07686	Beta-hexosaminidase subunit beta OS=Homo sapiens GN=HEXB PE=1 SV=3 - [HEXB_HUMAN]
O60573	Eukaryotic translation initiation factor 4E type 2 OS=Homo sapiens GN=EIF4E2 PE=1 SV=1 - [IF4E2_HUMAN]
Q9H270	Vacuolar protein sorting-associated protein 11 homolog OS=Homo sapiens GN=VPS11 PE=1 SV=1 - [VPS11_HUMAN]
Q9UBW8	COP9 signalosome complex subunit 7a OS=Homo sapiens GN=COPS7A PE=1 SV=1 - [CSN7A_HUMAN]
P53992	Protein transport protein Sec24C OS=Homo sapiens GN=SEC24C PE=1 SV=3 - [SC24C_HUMAN]
Q9HAV4	Exportin-5 OS=Homo sapiens GN=XPO5 PE=1 SV=1 - [XPO5_HUMAN]
P52597	Heterogeneous nuclear ribonucleoprotein F OS=Homo sapiens GN=HNRNPF PE=1 SV=3 - [HNRPF_HUMAN]
Q13555	Calcium/calmodulin-dependent protein kinase type II subunit gamma OS=Homo sapiens GN=CAMK2G PE=1 SV=3 - [KCC2G_HUMAN]
O43237	Cytoplasmic dynein 1 light intermediate chain 2 OS=Homo sapiens GN=DYNC1LI2 PE=1 SV=1 - [DC1L2_HUMAN]
P61081	NEDD8-conjugating enzyme Ubc12 OS=Homo sapiens GN=UBE2M PE=1 SV=1 - [UBC12_HUMAN]
Q92896	Golgi apparatus protein 1 OS=Homo sapiens GN=GLG1 PE=1 SV=2 - [GSLG1_HUMAN]
O14786	Neuropilin-1 OS=Homo sapiens GN=NRP1 PE=1 SV=3 - [NRP1_HUMAN]
Q9P253	Vacuolar protein sorting-associated protein 18 homolog OS=Homo sapiens GN=VPS18 PE=1 SV=2 - [VPS18_HUMAN]
P36507	Dual specificity mitogen-activated protein kinase kinase 2 OS=Homo sapiens GN=MAP2K2 PE=1 SV=1 - [MP2K2_HUMAN]
P51809	Vesicle-associated membrane protein 7 OS=Homo sapiens GN=VAMP7 PE=1 SV=3 - [VAMP7_HUMAN]
Q01081	Splicing factor U2AF 35 kDa subunit OS=Homo sapiens GN=U2AF1 PE=1 SV=3 - [U2AF1_HUMAN]
Q9Y478	5'-AMP-activated protein kinase subunit beta-1 OS=Homo sapiens GN=PRKAB1 PE=1 SV=4 - [AAKB1_HUMAN]
Q92538	Golgi-specific brefeldin A-resistance guanine nucleotide exchange factor 1 OS=Homo sapiens GN=GBF1 PE=1 SV=2 - [GBF1_HUMAN]
Q9BZD4	Kinetochore protein Nuf2 OS=Homo sapiens GN=NUF2 PE=1 SV=2 - [NUF2_HUMAN]
P68871	Hemoglobin subunit beta OS=Homo sapiens GN=HBB PE=1 SV=2 - [HBB_HUMAN]
Q96SY0	von Willebrand factor A domain-containing protein 9 OS=Homo sapiens GN=VWA9 PE=1 SV=2 - [VWA9_HUMAN]
Q9BXJ9	N-alpha-acetyltransferase 15, NatA auxiliary subunit OS=Homo sapiens GN=NAA15 PE=1 SV=1 - [NAA15_HUMAN]
Q9UNX4	WD repeat-containing protein 3 OS=Homo sapiens GN=WDR3 PE=1 SV=1 - [WDR3_HUMAN]
Q15198	Platelet-derived growth factor receptor-like protein OS=Homo sapiens GN=PDGFRL PE=1 SV=1 - [PGFRL_HUMAN]
Q9Y4R8	Telomere length regulation protein TEL2 homolog OS=Homo sapiens GN=TELO2 PE=1 SV=2 - [TELO2_HUMAN]
Q02878	60S ribosomal protein L6 OS=Homo sapiens GN=RPL6 PE=1 SV=3 - [RL6_HUMAN]
O43663	Protein regulator of cytokinesis 1 OS=Homo sapiens GN=PRC1 PE=1 SV=2 - [PRC1_HUMAN]
O76061	Stanniocalcin-2 OS=Homo sapiens GN=STC2 PE=1 SV=1 - [STC2_HUMAN]
P15735	Phosphorylase b kinase gamma catalytic chain, liver/testis isoform OS=Homo sapiens GN=PHKG2 PE=1 SV=1 - [PHKG2_HUMAN]
Q9BPU6	Dihydropyrimidinase-related protein 5 OS=Homo sapiens GN=DPYSL5 PE=1 SV=1 - [DPYL5_HUMAN]
O14965	Aurora kinase A OS=Homo sapiens GN=AURKA PE=1 SV=2 - [AURKA_HUMAN]
P35573	Glycogen debranching enzyme OS=Homo sapiens GN=AGL PE=1 SV=3 - [GDE_HUMAN]
O75165	DnaJ homolog subfamily C member 13 OS=Homo sapiens GN=DNAJC13 PE=1 SV=5 - [DJC13_HUMAN]
Q9HCB6	Spondin-1 OS=Homo sapiens GN=SPON1 PE=1 SV=2 - [SPON1_HUMAN]
P25685	DnaJ homolog subfamily B member 1 OS=Homo sapiens GN=DNAJB1 PE=1 SV=4 - [DNJB1_HUMAN]
P01857	Ig gamma-1 chain C region OS=Homo sapiens GN=IGHG1 PE=1 SV=1 - [IGHG1_HUMAN]
P47972	Neuronal pentraxin-2 OS=Homo sapiens GN=NPTX2 PE=1 SV=2 - [NPTX2_HUMAN]
O75348	V-type proton ATPase subunit G 1 OS=Homo sapiens GN=ATP6V1G1 PE=1 SV=3 - [VATG1_HUMAN]
Q9NVZ3	Adaptin ear-binding coat-associated protein 2 OS=Homo sapiens GN=NECAP2 PE=1 SV=1 - [NECP2_HUMAN]
Q676U5	Autophagy-related protein 16-1 OS=Homo sapiens GN=ATG16L1 PE=1 SV=2 - [A16L1_HUMAN]
Q9BRT9	DNA replication complex GINS protein SLD5 OS=Homo sapiens GN=GINS4 PE=1 SV=1 - [SLD5_HUMAN]
Q9HD42	Charged multivesicular body protein 1a OS=Homo sapiens GN=CHMP1A PE=1 SV=1 - [CHM1A_HUMAN]
Q14019	Coactosin-like protein OS=Homo sapiens GN=COTL1 PE=1 SV=3 - [COTL1_HUMAN]
O75367	Core histone macro-H2A.1 OS=Homo sapiens GN=H2AFY PE=1 SV=4 - [H2AY_HUMAN]
O75935	Dynactin subunit 3 OS=Homo sapiens GN=DCTN3 PE=1 SV=1 - [DCTN3 HUMAN]
Q8TBC3	SH3KBP1-binding protein 1 OS=Homo sapiens GN=SHKBP1 PE=1 SV=2 - [SHKB1_HUMAN]
Q9H269	Vacuolar protein sorting-associated protein 16 homolog OS=Homo sapiens GN=VPS16 PE=1 SV=2 - [VPS16_HUMAN]
Q15418	Ribosomal protein S6 kinase alpha-1 OS=Homo sapiens GN=RPS6KA1 PE=1 SV=2 - [KS6A1_HUMAN]
Q16181	Septin-7 OS=Homo sapiens GN=SEPT7 PE=1 SV=2 - [SEPT7_HUMAN]
Q8NCH0	Carbohydrate sulfotransferase 14 OS=Homo sapiens GN=CHST14 PE=1 SV=2 - [CHSTE_HUMAN]
P22413	Ectonucleotide pyrophosphatase/phosphodiesterase family member 1 OS=Homo sapiens GN=ENPP1 PE=1 SV=2 - [ENPP1_HUMAN]
Q9NVC6	Mediator of RNA polymerase II transcription subunit 17 OS=Homo sapiens GN=MED17 PE=1 SV=2 - [MED17_HUMAN]
P51659	Peroxisomal multifunctional enzyme type 2 OS=Homo sapiens GN=HSD17B4 PE=1 SV=3 - [DHB4_HUMAN]
Q9BSD7	Cancer-related nucleoside-triphosphatase OS=Homo sapiens GN=NTPCR PE=1 SV=1 - [NTPCR_HUMAN]
Q9NNW5	WD repeat-containing protein 6 OS=Homo sapiens GN=WDR6 PE=1 SV=1 - [WDR6_HUMAN]
Q8IZ83	Aldehyde dehydrogenase family 16 member A1 OS=Homo sapiens GN=ALDH16A1 PE=1 SV=2 - [A16A1_HUMAN]
Q15018	BRISC complex subunit Abro1 OS=Homo sapiens GN=FAM175B PE=1 SV=2 - [F175B_HUMAN]
Q8WTW3	Conserved oligomeric Golgi complex subunit 1 OS=Homo sapiens GN=COG1 PE=1 SV=1 - [COG1_HUMAN]
Q15042	Rab3 GTPase-activating protein catalytic subunit OS=Homo sapiens GN=RAB3GAP1 PE=1 SV=3 - [RB3GP_HUMAN]
Q68CZ6	HAUS augmin-like complex subunit 3 OS=Homo sapiens GN=HAUS3 PE=1 SV=1 - [HAUS3_HUMAN]
Q13501	Sequestosome-1 OS=Homo sapiens GN=SQSTM1 PE=1 SV=1 - [SQSTM_HUMAN]
Q13617	Cullin-2 OS=Homo sapiens GN=CUL2 PE=1 SV=2 - [CUL2_HUMAN]
P34096	Ribonuclease 4 OS=Homo sapiens GN=RNASE4 PE=1 SV=3 - [RNAS4_HUMAN]
P56282	DNA polymerase epsilon subunit 2 OS=Homo sapiens GN=POLE2 PE=1 SV=2 - [DPOE2_HUMAN]
Q9UHV9	Prefoldin subunit 2 OS=Homo sapiens GN=PFDN2 PE=1 SV=1 - [PFD2 HUMAN]
Q86XX4	Extracellular matrix protein FRAS1 OS=Homo sapiens GN=FRAS1 PE=1 SV=2 - [FRAS1_HUMAN]
Q9UNZ2	NSFL1 cofactor p47 OS=Homo sapiens GN=NSFL1C PE=1 SV=2 - [NSF1C_HUMAN]
Q13283	Ras GTPase-activating protein-binding protein 1 OS=Homo sapiens GN=G3BP1 PE=1 SV=1 - [G3BP1_HUMAN]
Q86V81	THO complex subunit 4 OS=Homo sapiens GN=ALYREF PE=1 SV=3 - [THOC4_HUMAN]
Q9NP97	Dynein light chain roadblock-type 1 OS=Homo sapiens GN=DYNLRB1 PE=1 SV=3 - [DLRB1_HUMAN]
Q9H974	Queuine tRNA-ribosyltransferase subunit QTRTD1 OS=Homo sapiens GN=QTRTD1 PE=1 SV=1 - [QTRD1_HUMAN]
O15511	Actin-related protein 2/3 complex subunit 5 OS=Homo sapiens GN=ARPC5 PE=1 SV=3 - [ARPC5_HUMAN]
P53999	Activated RNA polymerase II transcriptional coactivator p15 OS=Homo sapiens GN=SUB1 PE=1 SV=3 - [TCP4_HUMAN]
Q13144	Translation initiation factor eIF-2B subunit epsilon OS=Homo sapiens GN=EIF2B5 PE=1 SV=3 - [EI2BE HUMAN]
Q13443	Disintegrin and metalloproteinase domain-containing protein 9 OS=Homo sapiens GN=ADAM9 PE=1 SV=1 - [ADAM9_HUMAN]
Q9BQ52	Zinc phosphodiesterase ELAC protein 2 OS=Homo sapiens GN=ELAC2 PE=1 SV=2 - [RNZ2_HUMAN]
Q86XI2	Condensin-2 complex subunit G2 OS=Homo sapiens GN=NCAPG2 PE=1 SV=1 - [CNDG2_HUMAN]
Q9UBC2	Epidermal growth factor receptor substrate 15-like 1 OS=Homo sapiens GN=EPS15L1 PE=1 SV=1 - [EP15R_HUMAN]
Q14C86	GTPase-activating protein and VPS9 domain-containing protein 1 OS=Homo sapiens GN=GAPVD1 PE=1 SV=2 - [GAPD1_HUMAN]
P25788	Proteasome subunit alpha type-3 OS=Homo sapiens GN=PSMA3 PE=1 SV=2 - [PSA3_HUMAN]
Q96N67	Dedicator of cytokinesis protein 7 OS=Homo sapiens GN=DOCK7 PE=1 SV=4 - [DOCK7_HUMAN]
Q9Y4P1	Cysteine protease ATG4B OS=Homo sapiens GN=ATG4B PE=1 SV=2 - [ATG4B_HUMAN]
O60216	Double-strand-break repair protein rad21 homolog OS=Homo sapiens GN=RAD21 PE=1 SV=2 - [RAD21_HUMAN]
P25705	ATP synthase subunit alpha, mitochondrial OS=Homo sapiens GN=ATP5A1 PE=1 SV=1 - [ATPA_HUMAN]
P63220	40S ribosomal protein S21 OS=Homo sapiens GN=RPS21 PE=1 SV=1 - [RS21_HUMAN]

Q9UKG1	DCC-interacting protein 13-alpha OS=Homo sapiens GN=APPL1 PE=1 SV=1 - [DP13A_HUMAN]
Q92797	Symplekin OS=Homo sapiens GN=SYMPK PE=1 SV=2 - [SYMPK_HUMAN]
Q9UL25	Ras-related protein Rab-21 OS=Homo sapiens GN=RAB21 PE=1 SV=3 - [RAB21_HUMAN]
P55265	Double-stranded RNA-specific adenosine deaminase OS=Homo sapiens GN=ADAR PE=1 SV=4 - [DSRAD_HUMAN]
O15111	Inhibitor of nuclear factor kappa-B kinase subunit alpha OS=Homo sapiens GN=CHUK PE=1 SV=2 - [IKKA_HUMAN]
Q8N1G2	Cap-specific mRNA (nucleoside-2'-O-)-methyltransferase 1 OS=Homo sapiens GN=CMTR1 PE=1 SV=1 - [CMTR1_HUMAN]
Q9H1D9	DNA-directed RNA polymerase III subunit RPC6 OS=Homo sapiens GN=POLR3F PE=1 SV=1 - [RPC6_HUMAN]
O14617	AP-3 complex subunit delta-1 OS=Homo sapiens GN=AP3D1 PE=1 SV=1 - [AP3D1_HUMAN]
Q96EF6	F-box only protein 17 OS=Homo sapiens GN=FBXO17 PE=1 SV=1 - [FBX17_HUMAN]
Q9BT25	HAUS augmin-like complex subunit 8 OS=Homo sapiens GN=HAUS8 PE=1 SV=3 - [HAUS8_HUMAN]
Q9NRW7	Vacuolar protein sorting-associated protein 45 OS=Homo sapiens GN=VPS45 PE=1 SV=1 - [VPS45_HUMAN]
P04818	Thymidylate synthase OS=Homo sapiens GN=TYMS PE=1 SV=3 - [TYSY HUMAN]
Q6PJG6	BRCA1-associated ATM activator 1 OS=Homo sapiens GN=BRAT1 PE=1 SV=2 - [BRAT1_HUMAN]
P07195	L-lactate dehydrogenase B chain OS=Homo sapiens GN=LDHB PE=1 SV=2 - [LDHB_HUMAN]
P46109	Crk-like protein OS=Homo sapiens GN=CRKL PE=1 SV=1 - [CRKL_HUMAN]
P50995	Annexin A11 OS=Homo sapiens GN=ANXA11 PE=1 SV=1 - [ANX11_HUMAN]
P52788	Spermine synthase OS=Homo sapiens GN=SMS PE=1 SV=2 - [SPSY_HUMAN]
Q68D91	Metallo-beta-lactamase domain-containing protein 2 OS=Homo sapiens GN=MBLAC2 PE=2 SV=3 - [MBLC2_HUMAN]
Q96I24	Far upstream element-binding protein 3 OS=Homo sapiens GN=FUBP3 PE=1 SV=2 - [FUBP3_HUMAN]
O60341	Lysine-specific histone demethylase 1A OS=Homo sapiens GN=KDM1A PE=1 SV=2 - [KDM1A_HUMAN]
P11234	Ras-related protein Ral-B OS=Homo sapiens GN=RALB PE=1 SV=1 - [RALB_HUMAN]
Q9UID3	Vacuolar protein sorting-associated protein 51 homolog OS=Homo sapiens GN=VPS51 PE=1 SV=2 - [VPS51_HUMAN]
O00505	Importin subunit alpha-4 OS=Homo sapiens GN=KPNA3 PE=1 SV=2 - [IMA4_HUMAN]
P13612	Integrin alpha-4 OS=Homo sapiens GN=ITGA4 PE=1 SV=3 - [ITA4_HUMAN]
P62906	60S ribosomal protein L10a OS=Homo sapiens GN=RPL10A PE=1 SV=2 - [RL10A_HUMAN]
Q9NPD3	Exosome complex component RRP41 OS=Homo sapiens GN=EXOSC4 PE=1 SV=3 - [EXOS4_HUMAN]
Q15717	ELAV-like protein 1 OS=Homo sapiens GN=ELAVL1 PE=1 SV=2 - [ELAV1_HUMAN]
Q9H267	Vacuolar protein sorting-associated protein 33B OS=Homo sapiens GN=VPS33B PE=1 SV=2 - [VP33B_HUMAN]
Q8TBC4	NEDD8-activating enzyme E1 catalytic subunit OS=Homo sapiens GN=UBA3 PE=1 SV=2 - [UBA3_HUMAN]
P52732	Kinesin-like protein KIF11 OS=Homo sapiens GN=KIF11 PE=1 SV=2 - [KIF11_HUMAN]
Q8NCW5	NAD(P)H-hydrate epimerase OS=Homo sapiens GN=APOA1BP PE=1 SV=2 - [NNRE_HUMAN]
P19338	Nucleolin OS=Homo sapiens GN=NCL PE=1 SV=3 - [NUCL_HUMAN]
O60911	Cathepsin L2 OS=Homo sapiens GN=CTSV PE=1 SV=2 - [CATL2_HUMAN]
Q9NUQ3	Gamma-taxilin OS=Homo sapiens GN=TXLNG PE=1 SV=2 - [TXLNG_HUMAN]
O75694	Nuclear pore complex protein Nup155 OS=Homo sapiens GN=NUP155 PE=1 SV=1 - [NU155_HUMAN]
P60033	CD81 antigen OS=Homo sapiens GN=CD81 PE=1 SV=1 - [CD81_HUMAN]
Q6UVY6	DBH-like monooxygenase protein 1 OS=Homo sapiens GN=MOXD1 PE=1 SV=1 - [MOXD1_HUMAN]
Q86VR8	Four-jointed box protein 1 OS=Homo sapiens GN=FJX1 PE=2 SV=1 - [FJX1_HUMAN]
Q8WUM0	Nuclear pore complex protein Nup133 OS=Homo sapiens GN=NUP133 PE=1 SV=2 - [NU133_HUMAN]
Q9NR56	Muscleblind-like protein 1 OS=Homo sapiens GN=MBNL1 PE=1 SV=2 - [MBNL1_HUMAN]
P98172	Ephrin-B1 OS=Homo sapiens GN=EFNB1 PE=1 SV=1 - [EFNB1_HUMAN]
Q99575	Ribonucleases P/MRP protein subunit POP1 OS=Homo sapiens GN=POP1 PE=1 SV=2 - [POP1_HUMAN]
Q06124	Tyrosine-protein phosphatase non-receptor type 11 OS=Homo sapiens GN=PTPN11 PE=1 SV=2 - [PTN11_HUMAN]
Q15046	Lysine--tRNA ligase OS=Homo sapiens GN=KARS PE=1 SV=3 - [SYK_HUMAN]
O43913	Origin recognition complex subunit 5 OS=Homo sapiens GN=ORC5 PE=1 SV=1 - [ORC5_HUMAN]
Q9UP83	Conserved oligomeric Golgi complex subunit 5 OS=Homo sapiens GN=COG5 PE=1 SV=3 - [COG5_HUMAN]
P34931	Heat shock 70 kDa protein 1-like OS=Homo sapiens GN=HSPA1L PE=1 SV=2 - [HS71L_HUMAN]
Q7Z4G1	COMM domain-containing protein 6 OS=Homo sapiens GN=COMMD6 PE=1 SV=1 - [COMD6_HUMAN]
P86790	Vacuolar fusion protein CCZ1 homolog B OS=Homo sapiens GN=CCZ1B PE=1 SV=1 - [CCZ1B_HUMAN]
Q8TEX9	Importin-4 OS=Homo sapiens GN=IPO4 PE=1 SV=2 - [IPO4_HUMAN]
P49419	Alpha-aminoadipic semialdehyde dehydrogenase OS=Homo sapiens GN=ALDH7A1 PE=1 SV=5 - [AL7A1_HUMAN]
Q86X29	Lipolysis-stimulated lipoprotein receptor OS=Homo sapiens GN=LSR PE=1 SV=4 - [LSR_HUMAN]
Q9UJ70	N-acetyl-D-glucosamine kinase OS=Homo sapiens GN=NAGK PE=1 SV=4 - [NAGK_HUMAN]
Q96AX1	Vacuolar protein sorting-associated protein 33A OS=Homo sapiens GN=VPS33A PE=1 SV=1 - [VP33A_HUMAN]
O75718	Cartilage-associated protein OS=Homo sapiens GN=CRTAP PE=1 SV=1 - [CRTAP_HUMAN]
Q96EK5	KIF1-binding protein OS=Homo sapiens GN=KIAA1279 PE=1 SV=1 - [KBP_HUMAN]
O43598	2'-deoxynucleoside 5'-phosphate N-hydrolase 1 OS=Homo sapiens GN=DNPH1 PE=1 SV=1 - [DNPH1_HUMAN]
Q658Y4	Protein FAM91A1 OS=Homo sapiens GN=FAM91A1 PE=1 SV=3 - [F91A1_HUMAN]
Q8N3F8	MICAL-like protein 1 OS=Homo sapiens GN=MICALL1 PE=1 SV=2 - [MILK1_HUMAN]
Q96T76	MMS19 nucleotide excision repair protein homolog OS=Homo sapiens GN=MMS19 PE=1 SV=2 - [MMS19_HUMAN]
P54578	Ubiquitin carboxyl-terminal hydrolase 14 OS=Homo sapiens GN=USP14 PE=1 SV=3 - [UBP14_HUMAN]
Q14980	Nuclear mitotic apparatus protein 1 OS=Homo sapiens GN=NUMA1 PE=1 SV=2 - [NUMA1_HUMAN]
Q9H000	Probable E3 ubiquitin-protein ligase makorin-2 OS=Homo sapiens GN=MKRN2 PE=1 SV=2 - [MKRN2_HUMAN]
Q15293	Reticulocalbin-1 OS=Homo sapiens GN=RCN1 PE=1 SV=1 - [RCN1_HUMAN]
Q68E01	Integrator complex subunit 3 OS=Homo sapiens GN=INTS3 PE=1 SV=1 - [INT3_HUMAN]
Q9H204	Mediator of RNA polymerase II transcription subunit 28 OS=Homo sapiens GN=MED28 PE=1 SV=1 - [MED28_HUMAN]
Q6IBS0	Twinfilin-2 OS=Homo sapiens GN=TWF2 PE=1 SV=2 - [TWF2_HUMAN]
P31946	14-3-3 protein beta/alpha OS=Homo sapiens GN=YWHAB PE=1 SV=3 - [1433B_HUMAN]
Q6PD62	RNA polymerase-associated protein CTR9 homolog OS=Homo sapiens GN=CTR9 PE=1 SV=1 - [CTR9_HUMAN]
Q8WV41	Sorting nexin-33 OS=Homo sapiens GN=SNX33 PE=1 SV=1 - [SNX33_HUMAN]
Q99613	Eukaryotic translation initiation factor 3 subunit C OS=Homo sapiens GN=EIF3C PE=1 SV=1 - [EIF3C_HUMAN]
P54819	Adenylate kinase 2, mitochondrial OS=Homo sapiens GN=AK2 PE=1 SV=2 - [KAD2_HUMAN]
Q00341	Vigilin OS=Homo sapiens GN=HDLBP PE=1 SV=2 - [VIGLN_HUMAN]
Q00839	Heterogeneous nuclear ribonucleoprotein U OS=Homo sapiens GN=HNRNPU PE=1 SV=6 - [HNRPU_HUMAN]
Q02818	Nucleobindin-1 OS=Homo sapiens GN=NUCB1 PE=1 SV=4 - [NUCB1_HUMAN]
Q8IX18	Probable ATP-dependent RNA helicase DHX40 OS=Homo sapiens GN=DHX40 PE=1 SV=2 - [DHX40_HUMAN]
O43505	Beta-1,4-glucuronyltransferase 1 OS=Homo sapiens GN=B4GAT1 PE=1 SV=1 - [B4GA1_HUMAN]
Q16513	Serine/threonine-protein kinase N2 OS=Homo sapiens GN=PKN2 PE=1 SV=1 - [PKN2_HUMAN]
O43897	Tolloid-like protein 1 OS=Homo sapiens GN=TLL1 PE=1 SV=1 - [TLL1_HUMAN]
P61981	14-3-3 protein gamma OS=Homo sapiens GN=YWHAG PE=1 SV=2 - [1433G_HUMAN]
Q13243	Serine/arginine-rich splicing factor 5 OS=Homo sapiens GN=SRSF5 PE=1 SV=1 - [SRSF5_HUMAN]
Q96FN4	Copine-2 OS=Homo sapiens GN=CPNE2 PE=1 SV=3 - [CPNE2_HUMAN]
Q9UK76	Hematological and neurological expressed 1 protein OS=Homo sapiens GN=HN1 PE=1 SV=3 - [HN1_HUMAN]
Q8NFW8	N-acylneuraminate cytidylyltransferase OS=Homo sapiens GN=CMAS PE=1 SV=2 - [NEUA_HUMAN]
Q9UBL3	Set1/Ash2 histone methyltransferase complex subunit ASH2 OS=Homo sapiens GN=ASH2L PE=1 SV=1 - [ASH2L_HUMAN]
P40222	Alpha-taxilin OS=Homo sapiens GN=TXLNA PE=1 SV=3 - [TXLNA_HUMAN]
P40925	Malate dehydrogenase, cytoplasmic OS=Homo sapiens GN=MDH1 PE=1 SV=4 - [MDHC_HUMAN]
Q6NSI4	Uncharacterized protein CXorf57 OS=Homo sapiens GN=CXorf57 PE=1 SV=2 - [CX057_HUMAN]
P14923	Junction plakoglobin OS=Homo sapiens GN=JUP PE=1 SV=3 - [PLAK_HUMAN]
P47813	Eukaryotic translation initiation factor 1A, X-chromosomal OS=Homo sapiens GN=EIF1AX PE=1 SV=2 - [IF1AX_HUMAN]
Q9BRX5	DNA replication complex GINS protein PSF3 OS=Homo sapiens GN=GINS3 PE=1 SV=1 - [PSF3_HUMAN]
Q9NXV2	BTB/POZ domain-containing protein KCTD5 OS=Homo sapiens GN=KCTD5 PE=1 SV=1 - [KCTD5_HUMAN]
Q5T447	E3 ubiquitin-protein ligase HECTD3 OS=Homo sapiens GN=HECTD3 PE=1 SV=1 - [HECD3_HUMAN]
Q9Y5X3	Sorting nexin-5 OS=Homo sapiens GN=SNX5 PE=1 SV=1 - [SNX5_HUMAN]
Q9P287	BRCA2 and CDKN1A-interacting protein OS=Homo sapiens GN=BCCIP PE=1 SV=1 - [BCCIP_HUMAN]
Q9P0M6	Core histone macro-H2A.2 OS=Homo sapiens GN=H2AFY2 PE=1 SV=3 - [H2AW_HUMAN]
Q8N4P2	Tetratricopeptide repeat protein 30B OS=Homo sapiens GN=TTC30B PE=1 SV=2 - [TT30B_HUMAN]
P41221	Protein Wnt-5a OS=Homo sapiens GN=WNT5A PE=1 SV=2 - [WNT5A_HUMAN]
O60306	Intron-binding protein aquarius OS=Homo sapiens GN=AQR PE=1 SV=4 - [AQR_HUMAN]
Q9Y4B6	Protein VPRBP OS=Homo sapiens GN=VPRBP PE=1 SV=3 - [VPRBP_HUMAN]
P31153	S-adenosylmethionine synthase isoform type-2 OS=Homo sapiens GN=MAT2A PE=1 SV=1 - [METK2_HUMAN]
P52789	Hexokinase-2 OS=Homo sapiens GN=HK2 PE=1 SV=2 - [HXK2_HUMAN]
Q9Y4C2	TRPM8 channel-associated factor 1 OS=Homo sapiens GN=TCAF1 PE=1 SV=3 - [TCAF1_HUMAN]
O15020	Spectrin beta chain, non-erythrocytic 2 OS=Homo sapiens GN=SPTBN2 PE=1 SV=3 - [SPTN2_HUMAN]
Q96DM3	Uncharacterized protein C18orf8 OS=Homo sapiens GN=C18orf8 PE=2 SV=2 - [MIC1_HUMAN]
Q9H6T3	RNA polymerase II-associated protein 3 OS=Homo sapiens GN=RPAP3 PE=1 SV=2 - [RPAP3_HUMAN]
P54619	5'-AMP-activated protein kinase subunit gamma-1 OS=Homo sapiens GN=PRKAG1 PE=1 SV=1 - [AAKG1_HUMAN]
Q8NBJ5	Procollagen galactosyltransferase 1 OS=Homo sapiens GN=COLGALT1 PE=1 SV=1 - [GT251_HUMAN]
P12270	Nucleoprotein TPR OS=Homo sapiens GN=TPR PE=1 SV=3 - [TPR_HUMAN]
P55268	Laminin subunit beta-2 OS=Homo sapiens GN=LAMB2 PE=1 SV=2 - [LAMB2_HUMAN]
Q8IWA0	WD repeat-containing protein 75 OS=Homo sapiens GN=WDR75 PE=1 SV=1 - [WDR75_HUMAN]
Q9BVG4	Protein PBDC1 OS=Homo sapiens GN=PBDC1 PE=1 SV=1 - [PBDC1_HUMAN]
Q96F07	Cytoplasmic FMR1-interacting protein 2 OS=Homo sapiens GN=CYFIP2 PE=1 SV=2 - [CYFP2_HUMAN]
Q5JXB2	Putative ubiquitin-conjugating enzyme E2 N-like OS=Homo sapiens GN=UBE2NL PE=1 SV=1 - [UE2NL_HUMAN]
Q7Z3J2	UPF0505 protein C16orf62 OS=Homo sapiens GN=C16orf62 PE=1 SV=2 - [CP062_HUMAN]

Q9NVU0	DNA-directed RNA polymerase III subunit RPC5 OS=Homo sapiens GN=POLR3E PE=1 SV=1 - [RPC5_HUMAN]
Q9P289	Serine/threonine-protein kinase 26 OS=Homo sapiens GN=STK26 PE=1 SV=2 - [STK26_HUMAN]
O14657	Torsin-1B OS=Homo sapiens GN=TOR1B PE=1 SV=2 - [TOR1B_HUMAN]
Q86X55	Histone-arginine methyltransferase CARM1 OS=Homo sapiens GN=CARM1 PE=1 SV=3 - [CARM1_HUMAN]
Q14247	Src substrate cortactin OS=Homo sapiens GN=CTTN PE=1 SV=2 - [SRC8_HUMAN]
Q9P1U1	Actin-related protein 3B OS=Homo sapiens GN=ACTR3B PE=2 SV=1 - [ARP3B_HUMAN]
Q9UQN3	Charged multivesicular body protein 2b OS=Homo sapiens GN=CHMP2B PE=1 SV=1 - [CHM2B_HUMAN]
P23610	Factor VIII intron 22 protein OS=Homo sapiens GN=F8A1 PE=1 SV=2 - [F8I2_HUMAN]
P07237	Protein disulfide-isomerase OS=Homo sapiens GN=P4HB PE=1 SV=3 - [PDIA1_HUMAN]
Q9NQR4	Omega-amidase NIT2 OS=Homo sapiens GN=NIT2 PE=1 SV=1 - [NIT2_HUMAN]
Q9UHD8	Septin-9 OS=Homo sapiens GN=SEPT9 PE=1 SV=2 - [SEPT9_HUMAN]
Q6IBW4	Condensin-2 complex subunit H2 OS=Homo sapiens GN=NCAPH2 PE=1 SV=1 - [CNDH2_HUMAN]
P09543	2',3'-cyclic-nucleotide 3'-phosphodiesterase OS=Homo sapiens GN=CNP PE=1 SV=2 - [CN37_HUMAN]
P14678	Small nuclear ribonucleoprotein-associated proteins B and B' OS=Homo sapiens GN=SNRPB PE=1 SV=2 - [RSMB_HUMAN]
P15153	Ras-related C3 botulinum toxin substrate 2 OS=Homo sapiens GN=RAC2 PE=1 SV=1 - [RAC2_HUMAN]
P49005	DNA polymerase delta subunit 2 OS=Homo sapiens GN=POLD2 PE=1 SV=1 - [DPOD2_HUMAN]
Q5VST6	Alpha/beta hydrolase domain-containing protein 17B OS=Homo sapiens GN=ABHD17B PE=2 SV=1 - [AB17B_HUMAN]
Q96CX2	BTB/POZ domain-containing protein KCTD12 OS=Homo sapiens GN=KCTD12 PE=1 SV=1 - [KCD12_HUMAN]
P29122	Proprotein convertase subtilisin/kexin type 6 OS=Homo sapiens GN=PCSK6 PE=1 SV=1 - [PCSK6_HUMAN]
Q92820	Gamma-glutamyl hydrolase OS=Homo sapiens GN=GGH PE=1 SV=2 - [GGH_HUMAN]
Q8TAE8	Growth arrest and DNA damage-inducible proteins-interacting protein 1 OS=Homo sapiens GN=GADD45GIP1 PE=1 SV=1 - [G45IP_HUMAN]
P35249	Replication factor C subunit 4 OS=Homo sapiens GN=RFC4 PE=1 SV=2 - [RFC4_HUMAN]
Q70JA7	Chondroitin sulfate synthase 3 OS=Homo sapiens GN=CHSY3 PE=2 SV=3 - [CHSS3_HUMAN]
Q9BZE4	Nucleolar GTP-binding protein 1 OS=Homo sapiens GN=GTPBP4 PE=1 SV=3 - [NOG1_HUMAN]
Q14118	Dystroglycan OS=Homo sapiens GN=DAG1 PE=1 SV=2 - [DAG1_HUMAN]
Q14997	Proteasome activator complex subunit 4 OS=Homo sapiens GN=PSME4 PE=1 SV=2 - [PSME4_HUMAN]
P55786	Puromycin-sensitive aminopeptidase OS=Homo sapiens GN=NPEPPS PE=1 SV=2 - [PSA_HUMAN]
Q13619	Cullin-4A OS=Homo sapiens GN=CUL4A PE=1 SV=3 - [CUL4A_HUMAN]
P0DMP2	SLIT-ROBO Rho GTPase-activating protein 2B OS=Homo sapiens GN=SRGAP2B PE=3 SV=1 - [SRG2B_HUMAN]
P78347	General transcription factor II-I OS=Homo sapiens GN=GTF2I PE=1 SV=2 - [GTF2I_HUMAN]
Q9UGJ1	Gamma-tubulin complex component 4 OS=Homo sapiens GN=TUBGCP4 PE=1 SV=1 - [GCP4_HUMAN]
P16035	Metalloproteinase inhibitor 2 OS=Homo sapiens GN=TIMP2 PE=1 SV=2 - [TIMP2_HUMAN]
Q9UHI6	Probable ATP-dependent RNA helicase DDX20 OS=Homo sapiens GN=DDX20 PE=1 SV=2 - [DDX20_HUMAN]
P50897	Palmitoyl-protein thioesterase 1 OS=Homo sapiens GN=PPT1 PE=1 SV=1 - [PPT1_HUMAN]
Q9HBM1	Kinetochore protein Spc25 OS=Homo sapiens GN=SPC25 PE=1 SV=1 - [SPC25_HUMAN]
Q6UWP8	Suprabasin OS=Homo sapiens GN=SBSN PE=1 SV=2 - [SBSN_HUMAN]
P00505	Aspartate aminotransferase, mitochondrial OS=Homo sapiens GN=GOT2 PE=1 SV=3 - [AATM_HUMAN]
Q99435	Protein kinase C-binding protein NELL2 OS=Homo sapiens GN=NELL2 PE=1 SV=1 - [NELL2_HUMAN]
Q9NR30	Nucleolar RNA helicase 2 OS=Homo sapiens GN=DDX21 PE=1 SV=5 - [DDX21_HUMAN]
O75717	WD repeat and HMG-box DNA-binding protein 1 OS=Homo sapiens GN=WDHD1 PE=1 SV=1 - [WDHD1_HUMAN]
Q9NVE7	Pantothenate kinase 4 OS=Homo sapiens GN=PANK4 PE=1 SV=1 - [PANK4_HUMAN]
Q8N0V4	Leucine-rich repeat LGI family member 2 OS=Homo sapiens GN=LGI2 PE=2 SV=1 - [LGI2_HUMAN]
Q9H336	Cysteine-rich secretory protein LCCL domain-containing 1 OS=Homo sapiens GN=CRISPLD1 PE=2 SV=1 - [CRLD1_HUMAN]
Q9UNP9	Peptidyl-prolyl cis-trans isomerase E OS=Homo sapiens GN=PPIE PE=1 SV=1 - [PPIE_HUMAN]
P30048	Thioredoxin-dependent peroxide reductase, mitochondrial OS=Homo sapiens GN=PRDX3 PE=1 SV=3 - [PRDX3_HUMAN]
Q6NYC1	Bifunctional arginine demethylase and lysyl-hydroxylase JMJD6 OS=Homo sapiens GN=JMJD6 PE=1 SV=1 - [JMJD6_HUMAN]
P61077	Ubiquitin-conjugating enzyme E2 D3 OS=Homo sapiens GN=UBE2D3 PE=1 SV=1 - [UB2D3_HUMAN]
P42166	Lamina-associated polypeptide 2, isoform alpha OS=Homo sapiens GN=TMPO PE=1 SV=2 - [LAP2A_HUMAN]
P67870	Casein kinase II subunit beta OS=Homo sapiens GN=CSNK2B PE=1 SV=1 - [CSK2B_HUMAN]
P52435	DNA-directed RNA polymerase II subunit RPB11-a OS=Homo sapiens GN=POLR2J PE=1 SV=1 - [RPB11_HUMAN]
Q12824	SWI/SNF-related matrix-associated actin-dependent regulator of chromatin subfamily B member 1 OS=Homo sapiens GN=SMARCB1 PE=1 SV=2 - [SNF5_HUMAN]
Q9UJX4	Anaphase-promoting complex subunit 5 OS=Homo sapiens GN=ANAPC5 PE=1 SV=2 - [APC5_HUMAN]
Q8TED0	U3 small nucleolar RNA-associated protein 15 homolog OS=Homo sapiens GN=UTP15 PE=1 SV=3 - [UTP15_HUMAN]
Q96B26	Exosome complex component RRP43 OS=Homo sapiens GN=EXOSC8 PE=1 SV=1 - [EXOS8_HUMAN]
P14649	Myosin light chain 6B OS=Homo sapiens GN=MYL6B PE=1 SV=1 - [MYL6B_HUMAN]
Q9H6S0	Probable ATP-dependent RNA helicase YTHDC2 OS=Homo sapiens GN=YTHDC2 PE=1 SV=2 - [YTDC2_HUMAN]
P17900	Ganglioside GM2 activator OS=Homo sapiens GN=GM2A PE=1 SV=4 - [SAP3_HUMAN]
P30419	Glycylpeptide N-tetradecanoyltransferase 1 OS=Homo sapiens GN=NMT1 PE=1 SV=2 - [NMT1_HUMAN]
Q96AE4	Far upstream element-binding protein 1 OS=Homo sapiens GN=FUBP1 PE=1 SV=3 - [FUBP1_HUMAN]
P01876	Ig alpha-1 chain C region OS=Homo sapiens GN=IGHA1 PE=1 SV=2 - [IGHA1_HUMAN]
O00410	Importin-5 OS=Homo sapiens GN=IPO5 PE=1 SV=4 - [IPO5_HUMAN]
P29317	Ephrin type-A receptor 2 OS=Homo sapiens GN=EPHA2 PE=1 SV=2 - [EPHA2_HUMAN]
A8MWD9	Putative small nuclear ribonucleoprotein G-like protein 15 OS=Homo sapiens GN=SNRPGP15 PE=5 SV=2 - [RUXGL_HUMAN]
P62495	Eukaryotic peptide chain release factor subunit 1 OS=Homo sapiens GN=ETF1 PE=1 SV=3 - [ERF1_HUMAN]
Q9BYB4	Guanine nucleotide-binding protein subunit beta-like protein 1 OS=Homo sapiens GN=GNB1L PE=1 SV=2 - [GNB1L_HUMAN]
P56545	C-terminal-binding protein 2 OS=Homo sapiens GN=CTBP2 PE=1 SV=1 - [CTBP2_HUMAN]
Q5VSL9	Striatin-interacting protein 1 OS=Homo sapiens GN=STRIP1 PE=1 SV=1 - [STRP1_HUMAN]
Q7Z5G4	Golgin subfamily A member 7 OS=Homo sapiens GN=GOLGA7 PE=1 SV=2 - [GOGA7_HUMAN]
O00762	Ubiquitin-conjugating enzyme E2 C OS=Homo sapiens GN=UBE2C PE=1 SV=1 - [UBE2C_HUMAN]
P42677	40S ribosomal protein S27 OS=Homo sapiens GN=RPS27 PE=1 SV=3 - [RS27_HUMAN]
P45880	Voltage-dependent anion-selective channel protein 2 OS=Homo sapiens GN=VDAC2 PE=1 SV=2 - [VDAC2_HUMAN]
O43488	Aflatoxin B1 aldehyde reductase member 2 OS=Homo sapiens GN=AKR7A2 PE=1 SV=3 - [ARK72_HUMAN]
Q96RW7	Hemicentin-1 OS=Homo sapiens GN=HMCN1 PE=1 SV=2 - [HMCN1_HUMAN]
Q9BVQ7	Spermatogenesis-associated protein 5-like protein 1 OS=Homo sapiens GN=SPATA5L1 PE=1 SV=2 - [SPA5L_HUMAN]
P17655	Calpain-2 catalytic subunit OS=Homo sapiens GN=CAPN2 PE=1 SV=6 - [CAN2_HUMAN]
Q6VN20	Ran-binding protein 10 OS=Homo sapiens GN=RANBP10 PE=1 SV=1 - [RBP10_HUMAN]
Q96CS7	Pleckstrin homology domain-containing family B member 2 OS=Homo sapiens GN=PLEKHB2 PE=1 SV=1 - [PKHB2_HUMAN]
Q96MM7	Heparan-sulfate 6-O-sulfotransferase 2 OS=Homo sapiens GN=HS6ST2 PE=2 SV=2 - [H6ST2_HUMAN]
O75937	DnaJ homolog subfamily C member 8 OS=Homo sapiens GN=DNAJC8 PE=1 SV=2 - [DNJC8_HUMAN]
O94923	D-glucuronyl C5-epimerase OS=Homo sapiens GN=GLCE PE=1 SV=3 - [GLCE_HUMAN]
P51114	Fragile X mental retardation syndrome-related protein 1 OS=Homo sapiens GN=FXR1 PE=1 SV=3 - [FXR1_HUMAN]
Q14181	DNA polymerase alpha subunit B OS=Homo sapiens GN=POLA2 PE=1 SV=2 - [DPOA2_HUMAN]
P60866	40S ribosomal protein S20 OS=Homo sapiens GN=RPS20 PE=1 SV=1 - [RS20_HUMAN]
Q8NCM8	Cytoplasmic dynein 2 heavy chain 1 OS=Homo sapiens GN=DYNC2H1 PE=1 SV=4 - [DYHC2_HUMAN]
Q9NS86	LanC-like protein 2 OS=Homo sapiens GN=LANCL2 PE=1 SV=1 - [LANC2_HUMAN]
Q9Y3A5	Ribosome maturation protein SBDS OS=Homo sapiens GN=SBDS PE=1 SV=4 - [SBDS_HUMAN]
Q96A65	Exocyst complex component 4 OS=Homo sapiens GN=EXOC4 PE=1 SV=1 - [EXOC4_HUMAN]
Q99459	Cell division cycle 5-like protein OS=Homo sapiens GN=CDC5L PE=1 SV=2 - [CDC5L_HUMAN]
P09382	Galectin-1 OS=Homo sapiens GN=LGALS1 PE=1 SV=2 - [LEG1_HUMAN]
Q12860	Contactin-1 OS=Homo sapiens GN=CNTN1 PE=1 SV=1 - [CNTN1_HUMAN]
Q9NYU2	UDP-glucose:glycoprotein glucosyltransferase 1 OS=Homo sapiens GN=UGGT1 PE=1 SV=3 - [UGGG1_HUMAN]
O60282	Kinesin heavy chain isoform 5C OS=Homo sapiens GN=KIF5C PE=1 SV=1 - [KIF5C_HUMAN]
Q07954	Prolow-density lipoprotein receptor-related protein 1 OS=Homo sapiens GN=LRP1 PE=1 SV=2 - [LRP1_HUMAN]
O60832	H/ACA ribonucleoprotein complex subunit 4 OS=Homo sapiens GN=DKC1 PE=1 SV=3 - [DKC1_HUMAN]
Q86WT1	Tetratricopeptide repeat protein 30A OS=Homo sapiens GN=TTC30A PE=2 SV=3 - [TT30A_HUMAN]
P54289	Voltage-dependent calcium channel subunit alpha-2/delta-1 OS=Homo sapiens GN=CACNA2D1 PE=1 SV=3 - [CA2D1_HUMAN]
Q9H871	Protein RMD5 homolog A OS=Homo sapiens GN=RMND5A PE=1 SV=1 - [RMD5A_HUMAN]
O95825	Quinone oxidoreductase-like protein 1 OS=Homo sapiens GN=CRYZL1 PE=1 SV=2 - [QORL1_HUMAN]
Q8WXX5	DnaJ homolog subfamily C member 9 OS=Homo sapiens GN=DNAJC9 PE=1 SV=1 - [DNJC9_HUMAN]
Q9BRP4	Proteasomal ATPase-associated factor 1 OS=Homo sapiens GN=PAAF1 PE=1 SV=2 - [PAAF1_HUMAN]
Q9HD40	O-phosphoseryl-tRNA(Sec) selenium transferase OS=Homo sapiens GN=SEPSECS PE=1 SV=2 - [SPCS_HUMAN]
P31150	Rab GDP dissociation inhibitor alpha OS=Homo sapiens GN=GDI1 PE=1 SV=2 - [GDIA_HUMAN]
P49959	Double-strand break repair protein MRE11A OS=Homo sapiens GN=MRE11A PE=1 SV=3 - [MRE11_HUMAN]
Q86YM7	Homer protein homolog 1 OS=Homo sapiens GN=HOMER1 PE=1 SV=2 - [HOME1_HUMAN]
Q13642	Four and a half LIM domains protein 1 OS=Homo sapiens GN=FHL1 PE=1 SV=4 - [FHL1_HUMAN]
Q9Y547	Intraflagellar transport protein 25 homolog OS=Homo sapiens GN=HSPB11 PE=1 SV=1 - [IFT25_HUMAN]
O75581	Low-density lipoprotein receptor-related protein 6 OS=Homo sapiens GN=LRP6 PE=1 SV=2 - [LRP6_HUMAN]
Q99653	Calcineurin B homologous protein 1 OS=Homo sapiens GN=CHP1 PE=1 SV=3 - [CHP1_HUMAN]
Q9UJX5	Anaphase-promoting complex subunit 4 OS=Homo sapiens GN=ANAPC4 PE=1 SV=2 - [APC4_HUMAN]
P00491	Purine nucleoside phosphorylase OS=Homo sapiens GN=PNP PE=1 SV=2 - [PNPH_HUMAN]
Q7L1V2	Vacuolar fusion protein MON1 homolog B OS=Homo sapiens GN=MON1B PE=1 SV=1 - [MON1B_HUMAN]
Q13765	Nascent polypeptide-associated complex subunit alpha OS=Homo sapiens GN=NACA PE=1 SV=1 - [NACA_HUMAN]
Q6PGP7	Tetratricopeptide repeat protein 37 OS=Homo sapiens GN=TTC37 PE=1 SV=1 - [TTC37_HUMAN]
Q9H0D6	5'-3' exoribonuclease 2 OS=Homo sapiens GN=XRN2 PE=1 SV=1 - [XRN2_HUMAN]
Q9H0W8	Protein SMG9 OS=Homo sapiens GN=SMG9 PE=1 SV=1 - [SMG9_HUMAN]

A6NHR9	Structural maintenance of chromosomes flexible hinge domain-containing protein 1 OS=Homo sapiens GN=SMCHD1 PE=1 SV=2 - [SMHD1_HUMAN]
Q96KP1	Exocyst complex component 2 OS=Homo sapiens GN=EXOC2 PE=1 SV=1 - [EXOC2_HUMAN]
P56134	ATP synthase subunit f, mitochondrial OS=Homo sapiens GN=ATP5J2 PE=1 SV=3 - [ATPK_HUMAN]
P69849	Nodal modulator 3 OS=Homo sapiens GN=NOMO3 PE=3 SV=2 - [NOMO3_HUMAN]
Q16204	Coiled-coil domain-containing protein 6 OS=Homo sapiens GN=CCDC6 PE=1 SV=2 - [CCDC6_HUMAN]
Q8NB90	Spermatogenesis-associated protein 5 OS=Homo sapiens GN=SPATA5 PE=1 SV=3 - [SPAT5_HUMAN]
P62841	40S ribosomal protein S15 OS=Homo sapiens GN=RPS15 PE=1 SV=2 - [RS15_HUMAN]
Q92922	SWI/SNF complex subunit SMARCC1 OS=Homo sapiens GN=SMARCC1 PE=1 SV=3 - [SMRC1_HUMAN]
Q08378	Golgin subfamily A member 3 OS=Homo sapiens GN=GOLGA3 PE=1 SV=2 - [GOGA3_HUMAN]
P78537	Biogenesis of lysosome-related organelles complex 1 subunit 1 OS=Homo sapiens GN=BLOC1S1 PE=1 SV=2 - [BL1S1_HUMAN]
P11441	Ubiquitin-like protein 4A OS=Homo sapiens GN=UBL4A PE=1 SV=1 - [UBL4A_HUMAN]
P10155	60 kDa SS-A/Ro ribonucleoprotein OS=Homo sapiens GN=TROVE2 PE=1 SV=2 - [RO60_HUMAN]
P42345	Serine/threonine-protein kinase mTOR OS=Homo sapiens GN=MTOR PE=1 SV=1 - [MTOR_HUMAN]
Q9C0C9	E2/E3 hybrid ubiquitin-protein ligase UBE2O OS=Homo sapiens GN=UBE2O PE=1 SV=3 - [UBE2O_HUMAN]
O94874	E3 UFM1-protein ligase 1 OS=Homo sapiens GN=UFL1 PE=1 SV=2 - [UFL1_HUMAN]
O15294	UDP-N-acetylglucosamine--peptide N-acetylglucosaminyltransferase 110 kDa subunit OS=Homo sapiens GN=OGT PE=1 SV=3 - [OGT1_HUMAN]
Q96JG6	Coiled-coil domain-containing protein 132 OS=Homo sapiens GN=CCDC132 PE=1 SV=3 - [CC132_HUMAN]
Q6PD74	Alpha- and gamma-adaptin-binding protein p34 OS=Homo sapiens GN=AAGAB PE=1 SV=1 - [AAGAB_HUMAN]
P61812	Transforming growth factor beta-2 OS=Homo sapiens GN=TGFB2 PE=1 SV=1 - [TGFB2_HUMAN]
Q14160	Protein scribble homolog OS=Homo sapiens GN=SCRIB PE=1 SV=4 - [SCRIB_HUMAN]
P49767	Vascular endothelial growth factor C OS=Homo sapiens GN=VEGFC PE=1 SV=1 - [VEGFC_HUMAN]
P57737	Coronin-7 OS=Homo sapiens GN=CORO7 PE=1 SV=2 - [CORO7_HUMAN]
Q13620	Cullin-4B OS=Homo sapiens GN=CUL4B PE=1 SV=4 - [CUL4B_HUMAN]
Q9GZL7	Ribosome biogenesis protein WDR12 OS=Homo sapiens GN=WDR12 PE=1 SV=2 - [WDR12_HUMAN]
P11216	Glycogen phosphorylase, brain form OS=Homo sapiens GN=PYGB PE=1 SV=5 - [PYGB_HUMAN]
P49589	Cysteine--tRNA ligase, cytoplasmic OS=Homo sapiens GN=CARS PE=1 SV=3 - [SYCC_HUMAN]
P06858	Lipoprotein lipase OS=Homo sapiens GN=LPL PE=1 SV=1 - [LIPL_HUMAN]
Q9UL26	Ras-related protein Rab-22A OS=Homo sapiens GN=RAB22A PE=1 SV=2 - [RB22A_HUMAN]
Q2NL82	Pre-rRNA-processing protein TSR1 homolog OS=Homo sapiens GN=TSR1 PE=1 SV=1 - [TSR1_HUMAN]
P20042	Eukaryotic translation initiation factor 2 subunit 2 OS=Homo sapiens GN=EIF2S2 PE=1 SV=2 - [IF2B_HUMAN]
P28161	Glutathione S-transferase Mu 2 OS=Homo sapiens GN=GSTM2 PE=1 SV=2 - [GSTM2_HUMAN]
P49411	Elongation factor Tu, mitochondrial OS=Homo sapiens GN=TUFM PE=1 SV=2 - [EFTU_HUMAN]
P42766	60S ribosomal protein L35 OS=Homo sapiens GN=RPL35 PE=1 SV=2 - [RL35_HUMAN]
Q9Y580	RNA-binding protein 7 OS=Homo sapiens GN=RBM7 PE=1 SV=1 - [RBM7_HUMAN]
Q9BXB4	Oxysterol-binding protein-related protein 11 OS=Homo sapiens GN=OSBPL11 PE=1 SV=2 - [OSB11_HUMAN]
P09234	U1 small nuclear ribonucleoprotein C OS=Homo sapiens GN=SNRPC PE=1 SV=1 - [RU1C_HUMAN]
Q9BSV6	tRNA-splicing endonuclease subunit Sen34 OS=Homo sapiens GN=TSEN34 PE=1 SV=1 - [SEN34_HUMAN]
Q9NUQ7	Ufm1-specific protease 2 OS=Homo sapiens GN=UFSP2 PE=1 SV=3 - [UFSP2_HUMAN]
P10114	Ras-related protein Rap-2a OS=Homo sapiens GN=RAP2A PE=1 SV=1 - [RAP2A_HUMAN]
Q9NZL4	Hsp70-binding protein 1 OS=Homo sapiens GN=HSPBP1 PE=1 SV=1 - [HPBP1_HUMAN]
P55060	Exportin-2 OS=Homo sapiens GN=CSE1L PE=1 SV=3 - [XPO2_HUMAN]
Q9UHD2	Serine/threonine-protein kinase TBK1 OS=Homo sapiens GN=TBK1 PE=1 SV=1 - [TBK1_HUMAN]
Q9H0B6	Kinesin light chain 2 OS=Homo sapiens GN=KLC2 PE=1 SV=1 - [KLC2_HUMAN]
P22694	cAMP-dependent protein kinase catalytic subunit beta OS=Homo sapiens GN=PRKACB PE=1 SV=2 - [KAPCB_HUMAN]
Q92925	SWI/SNF-related matrix-associated actin-dependent regulator of chromatin subfamily D member 2 OS=Homo sapiens GN=SMARCD2 PE=1 SV=3 - [SMRD2_HUMAN]
Q96D46	60S ribosomal export protein NMD3 OS=Homo sapiens GN=NMD3 PE=1 SV=1 - [NMD3_HUMAN]
Q9BV44	THUMP domain-containing protein 3 OS=Homo sapiens GN=THUMPD3 PE=1 SV=1 - [THUM3_HUMAN]
Q92783	Signal transducing adapter molecule 1 OS=Homo sapiens GN=STAM PE=1 SV=3 - [STAM1_HUMAN]
Q9NRX1	RNA-binding protein PNO1 OS=Homo sapiens GN=PNO1 PE=1 SV=1 - [PNO1_HUMAN]
Q9UMF0	Intercellular adhesion molecule 5 OS=Homo sapiens GN=ICAM5 PE=1 SV=3 - [ICAM5_HUMAN]
Q9HC35	Echinoderm microtubule-associated protein-like 4 OS=Homo sapiens GN=EML4 PE=1 SV=3 - [EMAL4_HUMAN]
Q9UJA5	tRNA (adenine(58)-N(1))-methyltransferase non-catalytic subunit TRM6 OS=Homo sapiens GN=TRMT6 PE=1 SV=1 - [TRM6_HUMAN]
P55036	26S proteasome non-ATPase regulatory subunit 4 OS=Homo sapiens GN=PSMD4 PE=1 SV=1 - [PSMD4_HUMAN]
O14908	PDZ domain-containing protein GIPC1 OS=Homo sapiens GN=GIPC1 PE=1 SV=2 - [GIPC1_HUMAN]
P06276	Cholinesterase OS=Homo sapiens GN=BCHE PE=1 SV=1 - [CHLE_HUMAN]
Q9UIV1	CCR4-NOT transcription complex subunit 7 OS=Homo sapiens GN=CNOT7 PE=1 SV=3 - [CNOT7_HUMAN]
Q9Y2I8	WD repeat-containing protein 37 OS=Homo sapiens GN=WDR37 PE=1 SV=2 - [WDR37_HUMAN]
P17301	Integrin alpha-2 OS=Homo sapiens GN=ITGA2 PE=1 SV=1 - [ITA2_HUMAN]
Q6NZY4	Zinc finger CCHC domain-containing protein 8 OS=Homo sapiens GN=ZCCHC8 PE=1 SV=2 - [ZCHC8_HUMAN]
Q9NUP1	Biogenesis of lysosome-related organelles complex 1 subunit 4 OS=Homo sapiens GN=BLOC1S4 PE=1 SV=1 - [BL1S4_HUMAN]
Q9H9Y6	DNA-directed RNA polymerase I subunit RPA2 OS=Homo sapiens GN=POLR1B PE=1 SV=2 - [RPA2_HUMAN]
P27695	DNA-(apurinic or apyrimidinic site) lyase OS=Homo sapiens GN=APEX1 PE=1 SV=2 - [APEX1_HUMAN]
P12109	Collagen alpha-1(VI) chain OS=Homo sapiens GN=COL6A1 PE=1 SV=3 - [CO6A1_HUMAN]
Q14103	Heterogeneous nuclear ribonucleoprotein D0 OS=Homo sapiens GN=HNRNPD PE=1 SV=1 - [HNRPD_HUMAN]
Q6QNY0	Biogenesis of lysosome-related organelles complex 1 subunit 3 OS=Homo sapiens GN=BLOC1S3 PE=1 SV=1 - [BL1S3_HUMAN]
Q9BW83	Intraflagellar transport protein 27 homolog OS=Homo sapiens GN=IFT27 PE=1 SV=1 - [IFT27_HUMAN]
Q93100	Phosphorylase b kinase regulatory subunit beta OS=Homo sapiens GN=PHKB PE=1 SV=3 - [KPBB_HUMAN]
Q9Y4K0	Lysyl oxidase homolog 2 OS=Homo sapiens GN=LOXL2 PE=1 SV=1 - [LOXL2_HUMAN]
Q9BW92	Threonine--tRNA ligase, mitochondrial OS=Homo sapiens GN=TARS2 PE=1 SV=1 - [SYTM_HUMAN]
P26373	60S ribosomal protein L13 OS=Homo sapiens GN=RPL13 PE=1 SV=4 - [RL13_HUMAN]
P34896	Serine hydroxymethyltransferase, cytosolic OS=Homo sapiens GN=SHMT1 PE=1 SV=1 - [GLYC_HUMAN]
Q13148	TAR DNA-binding protein 43 OS=Homo sapiens GN=TARDBP PE=1 SV=1 - [TADBP_HUMAN]
Q5RKV6	Exosome complex component MTR3 OS=Homo sapiens GN=EXOSC6 PE=1 SV=1 - [EXOS6_HUMAN]
Q7Z6Z7	E3 ubiquitin-protein ligase HUWE1 OS=Homo sapiens GN=HUWE1 PE=1 SV=3 - [HUWE1_HUMAN]
Q9Y5K5	Ubiquitin carboxyl-terminal hydrolase isozyme L5 OS=Homo sapiens GN=UCHL5 PE=1 SV=3 - [UCHL5_HUMAN]
P01889	HLA class I histocompatibility antigen, B-7 alpha chain OS=Homo sapiens GN=HLA-B PE=1 SV=3 - [1B07_HUMAN]
P09960	Leukotriene A-4 hydrolase OS=Homo sapiens GN=LTA4H PE=1 SV=2 - [LKHA4_HUMAN]
P55209	Nucleosome assembly protein 1-like 1 OS=Homo sapiens GN=NAP1L1 PE=1 SV=1 - [NP1L1_HUMAN]
Q14847	LIM and SH3 domain protein 1 OS=Homo sapiens GN=LASP1 PE=1 SV=2 - [LASP1_HUMAN]
Q9BX67	Junctional adhesion molecule C OS=Homo sapiens GN=JAM3 PE=1 SV=1 - [JAM3_HUMAN]
Q9BZX2	Uridine-cytidine kinase 2 OS=Homo sapiens GN=UCK2 PE=1 SV=1 - [UCK2_HUMAN]
P48509	CD151 antigen OS=Homo sapiens GN=CD151 PE=1 SV=3 - [CD151_HUMAN]
P84098	60S ribosomal protein L19 OS=Homo sapiens GN=RPL19 PE=1 SV=1 - [RL19_HUMAN]
O14531	Dihydropyrimidinase-related protein 4 OS=Homo sapiens GN=DPYSL4 PE=1 SV=2 - [DPYL4_HUMAN]
O43491	Band 4.1-like protein 2 OS=Homo sapiens GN=EPB41L2 PE=1 SV=1 - [E41L2_HUMAN]
P06280	Alpha-galactosidase A OS=Homo sapiens GN=GLA PE=1 SV=1 - [AGAL_HUMAN]
O43639	Cytoplasmic protein NCK2 OS=Homo sapiens GN=NCK2 PE=1 SV=2 - [NCK2_HUMAN]
O95352	Ubiquitin-like modifier-activating enzyme ATG7 OS=Homo sapiens GN=ATG7 PE=1 SV=1 - [ATG7_HUMAN]
Q9NZN8	CCR4-NOT transcription complex subunit 2 OS=Homo sapiens GN=CNOT2 PE=1 SV=1 - [CNOT2_HUMAN]
P05026	Sodium/potassium-transporting ATPase subunit beta-1 OS=Homo sapiens GN=ATP1B1 PE=1 SV=1 - [AT1B1_HUMAN]
P20073	Annexin A7 OS=Homo sapiens GN=ANXA7 PE=1 SV=3 - [ANXA7_HUMAN]
Q96MG7	Melanoma-associated antigen G1 OS=Homo sapiens GN=NDNL2 PE=1 SV=1 - [MAGG1_HUMAN]
Q9Y2D4	Exocyst complex component 6B OS=Homo sapiens GN=EXOC6B PE=1 SV=3 - [EXC6B_HUMAN]
Q15785	Mitochondrial import receptor subunit TOM34 OS=Homo sapiens GN=TOMM34 PE=1 SV=2 - [TOM34_HUMAN]
Q9Y450	HBS1-like protein OS=Homo sapiens GN=HBS1L PE=1 SV=1 - [HBS1L_HUMAN]
Q9UPY3	Endoribonuclease Dicer OS=Homo sapiens GN=DICER1 PE=1 SV=3 - [DICER_HUMAN]
Q15007	Pre-mRNA-splicing regulator WTAP OS=Homo sapiens GN=WTAP PE=1 SV=2 - [FL2D_HUMAN]
O00754	Lysosomal alpha-mannosidase OS=Homo sapiens GN=MAN2B1 PE=1 SV=3 - [MA2B1_HUMAN]
Q8NFH4	Nucleoporin Nup37 OS=Homo sapiens GN=NUP37 PE=1 SV=1 - [NUP37_HUMAN]
Q16851	UTP--glucose-1-phosphate uridylyltransferase OS=Homo sapiens GN=UGP2 PE=1 SV=5 - [UGPA_HUMAN]
P07858	Cathepsin B OS=Homo sapiens GN=CTSB PE=1 SV=3 - [CATB_HUMAN]
Q15063	Periostin OS=Homo sapiens GN=POSTN PE=1 SV=2 - [POSTN_HUMAN]
P54886	Delta-1-pyrroline-5-carboxylate synthase OS=Homo sapiens GN=ALDH18A1 PE=1 SV=2 - [P5CS_HUMAN]
O14777	Kinetochore protein NDC80 homolog OS=Homo sapiens GN=NDC80 PE=1 SV=1 - [NDC80_HUMAN]
Q58A45	PAB-dependent poly(A)-specific ribonuclease subunit PAN3 OS=Homo sapiens GN=PAN3 PE=1 SV=3 - [PAN3_HUMAN]
Q9NWZ5	Uridine-cytidine kinase-like 1 OS=Homo sapiens GN=UCKL1 PE=1 SV=2 - [UCKL1_HUMAN]
Q8N1B4	Vacuolar protein sorting-associated protein 52 homolog OS=Homo sapiens GN=VPS52 PE=1 SV=1 - [VPS52_HUMAN]
Q9P2I0	Cleavage and polyadenylation specificity factor subunit 2 OS=Homo sapiens GN=CPSF2 PE=1 SV=2 - [CPSF2_HUMAN]
O14910	Protein lin-7 homolog A OS=Homo sapiens GN=LIN7A PE=1 SV=2 - [LIN7A_HUMAN]
O60841	Eukaryotic translation initiation factor 5B OS=Homo sapiens GN=EIF5B PE=1 SV=4 - [IF2P_HUMAN]
O95071	E3 ubiquitin-protein ligase UBR5 OS=Homo sapiens GN=UBR5 PE=1 SV=2 - [UBR5_HUMAN]
Q9HAD4	WD repeat-containing protein 41 OS=Homo sapiens GN=WDR41 PE=2 SV=3 - [WDR41_HUMAN]
Q58FF7	Putative heat shock protein HSP 90-beta-3 OS=Homo sapiens GN=HSP90AB3P PE=5 SV=1 - [H90B3_HUMAN]
Q8N2S1	Latent-transforming growth factor beta-binding protein 4 OS=Homo sapiens GN=LTBP4 PE=1 SV=2 - [LTBP4_HUMAN]
P16403	Histone H1.2 OS=Homo sapiens GN=HIST1H1C PE=1 SV=2 - [H12_HUMAN]

Q9Y597	BTB/POZ domain-containing protein KCTD3 OS=Homo sapiens GN=KCTD3 PE=1 SV=2 - [KCTD3_HUMAN]
Q9BTC8	Metastasis-associated protein MTA3 OS=Homo sapiens GN=MTA3 PE=1 SV=2 - [MTA3_HUMAN]
P55039	Developmentally-regulated GTP-binding protein 2 OS=Homo sapiens GN=DRG2 PE=1 SV=1 - [DRG2_HUMAN]
Q9P273	Teneurin-3 OS=Homo sapiens GN=TENM3 PE=2 SV=3 - [TEN3_HUMAN]
P42566	Epidermal growth factor receptor substrate 15 OS=Homo sapiens GN=EPS15 PE=1 SV=2 - [EPS15_HUMAN]
P09884	DNA polymerase alpha catalytic subunit OS=Homo sapiens GN=POLA1 PE=1 SV=2 - [DPOLA_HUMAN]
Q5SZK8	FRAS1-related extracellular matrix protein 2 OS=Homo sapiens GN=FREM2 PE=1 SV=2 - [FREM2_HUMAN]
P12235	ADP/ATP translocase 1 OS=Homo sapiens GN=SLC25A4 PE=1 SV=4 - [ADT1_HUMAN]
P58107	Epiplakin OS=Homo sapiens GN=EPPK1 PE=1 SV=2 - [EPIPL_HUMAN]
P54760	Ephrin type-B receptor 4 OS=Homo sapiens GN=EPHB4 PE=1 SV=2 - [EPHB4_HUMAN]
Q99715	Collagen alpha-1(XII) chain OS=Homo sapiens GN=COL12A1 PE=1 SV=2 - [COCA1_HUMAN]
P10600	Transforming growth factor beta-3 OS=Homo sapiens GN=TGFB3 PE=1 SV=1 - [TGFB3_HUMAN]
Q96PU5	E3 ubiquitin-protein ligase NEDD4-like OS=Homo sapiens GN=NEDD4L PE=1 SV=2 - [NED4L_HUMAN]
P23246	Splicing factor, proline- and glutamine-rich OS=Homo sapiens GN=SFPQ PE=1 SV=2 - [SFPQ_HUMAN]
Q07864	DNA polymerase epsilon catalytic subunit A OS=Homo sapiens GN=POLE PE=1 SV=5 - [DPOE1_HUMAN]
Q9BWU0	Kanadaptin OS=Homo sapiens GN=SLC4A1AP PE=1 SV=1 - [NADAP_HUMAN]
P62633	Cellular nucleic acid-binding protein OS=Homo sapiens GN=CNBP PE=1 SV=1 - [CNBP_HUMAN]
Q9H6R7	WD repeat and coiled-coil-containing protein C2orf44 OS=Homo sapiens GN=C2orf44 PE=1 SV=1 - [CB044_HUMAN]
P00568	Adenylate kinase isoenzyme 1 OS=Homo sapiens GN=AK1 PE=1 SV=3 - [KAD1_HUMAN]
Q8WXF1	Paraspeckle component 1 OS=Homo sapiens GN=PSPC1 PE=1 SV=1 - [PSPC1_HUMAN]
P51571	Translocon-associated protein subunit delta OS=Homo sapiens GN=SSR4 PE=1 SV=1 - [SSRD_HUMAN]
Q10471	Polypeptide N-acetylgalactosaminyltransferase 2 OS=Homo sapiens GN=GALNT2 PE=1 SV=1 - [GALT2_HUMAN]
Q96B97	SH3 domain-containing kinase-binding protein 1 OS=Homo sapiens GN=SH3KBP1 PE=1 SV=2 - [SH3K1_HUMAN]
Q9UG63	ATP-binding cassette sub-family F member 2 OS=Homo sapiens GN=ABCF2 PE=1 SV=2 - [ABCF2_HUMAN]
P09668	Pro-cathepsin H OS=Homo sapiens GN=CTSH PE=1 SV=4 - [CATH_HUMAN]
P49841	Glycogen synthase kinase-3 beta OS=Homo sapiens GN=GSK3B PE=1 SV=2 - [GSK3B_HUMAN]
P38646	Stress-70 protein, mitochondrial OS=Homo sapiens GN=HSPA9 PE=1 SV=2 - [GRP75_HUMAN]
Q9Y2Z0	Suppressor of G2 allele of SKP1 homolog OS=Homo sapiens GN=SUGT1 PE=1 SV=3 - [SUGT1_HUMAN]
O14654	Insulin receptor substrate 4 OS=Homo sapiens GN=IRS4 PE=1 SV=1 - [IRS4_HUMAN]
Q14444	Caprin-1 OS=Homo sapiens GN=CAPRIN1 PE=1 SV=2 - [CAPR1_HUMAN]
O60613	15 kDa selenoprotein OS=Homo sapiens GN=SEP15 PE=1 SV=3 - [SEP15_HUMAN]
Q9Y376	Calcium-binding protein 39 OS=Homo sapiens GN=CAB39 PE=1 SV=1 - [CAB39_HUMAN]
Q9Y3E7	Charged multivesicular body protein 3 OS=Homo sapiens GN=CHMP3 PE=1 SV=3 - [CHMP3_HUMAN]
Q6FI81	Anamorsin OS=Homo sapiens GN=CIAPIN1 PE=1 SV=2 - [CPIN1_HUMAN]
Q6UVK1	Chondroitin sulfate proteoglycan 4 OS=Homo sapiens GN=CSPG4 PE=1 SV=2 - [CSPG4_HUMAN]
P51151	Ras-related protein Rab-9A OS=Homo sapiens GN=RAB9A PE=1 SV=1 - [RAB9A_HUMAN]
Q96AC1	Fermitin family homolog 2 OS=Homo sapiens GN=FERMT2 PE=1 SV=1 - [FERM2_HUMAN]
P46108	Adapter molecule crk OS=Homo sapiens GN=CRK PE=1 SV=2 - [CRK_HUMAN]
Q13308	Inactive tyrosine-protein kinase 7 OS=Homo sapiens GN=PTK7 PE=1 SV=2 - [PTK7_HUMAN]
Q15813	Tubulin-specific chaperone E OS=Homo sapiens GN=TBCE PE=1 SV=1 - [TBCE_HUMAN]
Q8IUR7	Armadillo repeat-containing protein 8 OS=Homo sapiens GN=ARMC8 PE=1 SV=2 - [ARMC8_HUMAN]
Q9NPJ6	Mediator of RNA polymerase II transcription subunit 4 OS=Homo sapiens GN=MED4 PE=1 SV=1 - [MED4_HUMAN]
P49674	Casein kinase I isoform epsilon OS=Homo sapiens GN=CSNK1E PE=1 SV=1 - [KC1E_HUMAN]
Q96CT7	Coiled-coil domain-containing protein 124 OS=Homo sapiens GN=CCDC124 PE=1 SV=1 - [CC124_HUMAN]
Q9NU22	Midasin OS=Homo sapiens GN=MDN1 PE=1 SV=2 - [MDN1_HUMAN]
O60264	SWI/SNF-related matrix-associated actin-dependent regulator of chromatin subfamily A member 5 OS=Homo sapiens GN=SMARCA5 PE=1 SV=1 - [SMCA5_HUMAN]
P17174	Aspartate aminotransferase, cytoplasmic OS=Homo sapiens GN=GOT1 PE=1 SV=3 - [AATC_HUMAN]
Q9NPD7	Neuritin OS=Homo sapiens GN=NRN1 PE=2 SV=1 - [NRN1_HUMAN]
O00308	NEDD4-like E3 ubiquitin-protein ligase WWP2 OS=Homo sapiens GN=WWP2 PE=1 SV=2 - [WWP2_HUMAN]
Q9UBD5	Origin recognition complex subunit 3 OS=Homo sapiens GN=ORC3 PE=1 SV=1 - [ORC3_HUMAN]
Q9BY32	Inosine triphosphate pyrophosphatase OS=Homo sapiens GN=ITPA PE=1 SV=2 - [ITPA_HUMAN]
O43776	Asparagine--tRNA ligase, cytoplasmic OS=Homo sapiens GN=NARS PE=1 SV=1 - [SYNC_HUMAN]
P63096	Guanine nucleotide-binding protein G(i) subunit alpha-1 OS=Homo sapiens GN=GNAI1 PE=1 SV=2 - [GNAI1_HUMAN]
Q05193	Dynamin-1 OS=Homo sapiens GN=DNM1 PE=1 SV=2 - [DYN1_HUMAN]
Q9NUQ9	Protein FAM49B OS=Homo sapiens GN=FAM49B PE=1 SV=1 - [FA49B_HUMAN]
P05423	DNA-directed RNA polymerase III subunit RPC4 OS=Homo sapiens GN=POLR3D PE=1 SV=2 - [RPC4_HUMAN]
Q92974	Rho guanine nucleotide exchange factor 2 OS=Homo sapiens GN=ARHGEF2 PE=1 SV=4 - [ARHG2_HUMAN]
Q9BV19	Uncharacterized protein C1orf50 OS=Homo sapiens GN=C1orf50 PE=1 SV=2 - [CA050_HUMAN]
P11413	Glucose-6-phosphate 1-dehydrogenase OS=Homo sapiens GN=G6PD PE=1 SV=4 - [G6PD_HUMAN]
Q2M389	WASH complex subunit 7 OS=Homo sapiens GN=KIAA1033 PE=1 SV=2 - [WASH7_HUMAN]
Q8WUN7	Ubiquitin domain-containing protein 2 OS=Homo sapiens GN=UBTD2 PE=1 SV=2 - [UBTD2_HUMAN]
O94927	HAUS augmin-like complex subunit 5 OS=Homo sapiens GN=HAUS5 PE=1 SV=2 - [HAUS5_HUMAN]
P15170	Eukaryotic peptide chain release factor GTP-binding subunit ERF3A OS=Homo sapiens GN=GSPT1 PE=1 SV=1 - [ERF3A_HUMAN]
O15226	NF-kappa-B-repressing factor OS=Homo sapiens GN=NKRF PE=1 SV=2 - [NKRF_HUMAN]
P62070	Ras-related protein R-Ras2 OS=Homo sapiens GN=RRAS2 PE=1 SV=1 - [RRAS2_HUMAN]
Q9BV20	Methylthioribose-1-phosphate isomerase OS=Homo sapiens GN=MRI1 PE=1 SV=1 - [MTNA_HUMAN]
O60502	Protein O-GlcNAcase OS=Homo sapiens GN=MGEA5 PE=1 SV=2 - [OGA_HUMAN]
Q9UM54	Unconventional myosin-VI OS=Homo sapiens GN=MYO6 PE=1 SV=4 - [MYO6_HUMAN]
P07947	Tyrosine-protein kinase Yes OS=Homo sapiens GN=YES1 PE=1 SV=3 - [YES_HUMAN]
Q13435	Splicing factor 3B subunit 2 OS=Homo sapiens GN=SF3B2 PE=1 SV=2 - [SF3B2_HUMAN]
O75954	Tetraspanin-9 OS=Homo sapiens GN=TSPAN9 PE=1 SV=1 - [TSN9_HUMAN]
Q13464	Rho-associated protein kinase 1 OS=Homo sapiens GN=ROCK1 PE=1 SV=1 - [ROCK1_HUMAN]
Q719H9	BTB/POZ domain-containing protein KCTD1 OS=Homo sapiens GN=KCTD1 PE=1 SV=1 - [KCTD1_HUMAN]
Q8NE71	ATP-binding cassette sub-family F member 1 OS=Homo sapiens GN=ABCF1 PE=1 SV=2 - [ABCF1_HUMAN]
Q16658	Fascin OS=Homo sapiens GN=FSCN1 PE=1 SV=3 - [FSCN1_HUMAN]
Q9UMX0	Ubiquilin-1 OS=Homo sapiens GN=UBQLN1 PE=1 SV=2 - [UBQL1_HUMAN]
O15379	Histone deacetylase 3 OS=Homo sapiens GN=HDAC3 PE=1 SV=2 - [HDAC3_HUMAN]
P39687	Acidic leucine-rich nuclear phosphoprotein 32 family member A OS=Homo sapiens GN=ANP32A PE=1 SV=1 - [AN32A_HUMAN]
Q9H9J2	39S ribosomal protein L44, mitochondrial OS=Homo sapiens GN=MRPL44 PE=1 SV=1 - [RM44_HUMAN]
P33240	Cleavage stimulation factor subunit 2 OS=Homo sapiens GN=CSTF2 PE=1 SV=1 - [CSTF2_HUMAN]
Q8IX12	Cell division cycle and apoptosis regulator protein 1 OS=Homo sapiens GN=CCAR1 PE=1 SV=2 - [CCAR1_HUMAN]
O14979	Heterogeneous nuclear ribonucleoprotein D-like OS=Homo sapiens GN=HNRNPDL PE=1 SV=3 - [HNRDL_HUMAN]
Q8N122	Regulatory-associated protein of mTOR OS=Homo sapiens GN=RPTOR PE=1 SV=1 - [RPTOR_HUMAN]
O15232	Matrilin-3 OS=Homo sapiens GN=MATN3 PE=1 SV=2 - [MATN3_HUMAN]
P61421	V-type proton ATPase subunit d 1 OS=Homo sapiens GN=ATP6V0D1 PE=1 SV=1 - [VA0D1_HUMAN]
Q86UP2	Kinectin OS=Homo sapiens GN=KTN1 PE=1 SV=1 - [KTN1_HUMAN]
Q9UJX6	Anaphase-promoting complex subunit 2 OS=Homo sapiens GN=ANAPC2 PE=1 SV=1 - [ANC2_HUMAN]
Q13363	C-terminal-binding protein 1 OS=Homo sapiens GN=CTBP1 PE=1 SV=2 - [CTBP1_HUMAN]
Q969Y0	NXPE family member 3 OS=Homo sapiens GN=NXPE3 PE=2 SV=1 - [NXPE3_HUMAN]
A6NIH7	Protein unc-119 homolog B OS=Homo sapiens GN=UNC119B PE=1 SV=1 - [U119B_HUMAN]
Q9NR09	Baculoviral IAP repeat-containing protein 6 OS=Homo sapiens GN=BIRC6 PE=1 SV=2 - [BIRC6_HUMAN]
Q8N4T4	Rho guanine nucleotide exchange factor 39 OS=Homo sapiens GN=ARHGEF39 PE=1 SV=1 - [ARG39_HUMAN]
P51003	Poly(A) polymerase alpha OS=Homo sapiens GN=PAPOLA PE=1 SV=4 - [PAPOA_HUMAN]
P20674	Cytochrome c oxidase subunit 5A, mitochondrial OS=Homo sapiens GN=COX5A PE=1 SV=2 - [COX5A_HUMAN]
Q8IXM2	Chromatin complexes subunit BAP18 OS=Homo sapiens GN=BAP18 PE=1 SV=1 - [BAP18_HUMAN]
Q96EY4	Translation machinery-associated protein 16 OS=Homo sapiens GN=TMA16 PE=1 SV=2 - [TMA16_HUMAN]
P05156	Complement factor I OS=Homo sapiens GN=CFI PE=1 SV=2 - [CFAI_HUMAN]
Q96G46	tRNA-dihydrouridine(47) synthase [NAD(P)(+)]-like OS=Homo sapiens GN=DUS3L PE=1 SV=2 - [DUS3L_HUMAN]
O60942	mRNA-capping enzyme OS=Homo sapiens GN=RNGTT PE=1 SV=1 - [MCE1_HUMAN]
Q99570	Phosphoinositide 3-kinase regulatory subunit 4 OS=Homo sapiens GN=PIK3R4 PE=1 SV=3 - [PI3R4_HUMAN]
Q9UN86	Ras GTPase-activating protein-binding protein 2 OS=Homo sapiens GN=G3BP2 PE=1 SV=2 - [G3BP2_HUMAN]
O75150	E3 ubiquitin-protein ligase BRE1B OS=Homo sapiens GN=RNF40 PE=1 SV=4 - [BRE1B_HUMAN]
P13804	Electron transfer flavoprotein subunit alpha, mitochondrial OS=Homo sapiens GN=ETFA PE=1 SV=1 - [ETFA_HUMAN]
Q9Y5Y2	Cytosolic Fe-S cluster assembly factor NUBP2 OS=Homo sapiens GN=NUBP2 PE=1 SV=1 - [NUBP2_HUMAN]
O76094	Signal recognition particle subunit SRP72 OS=Homo sapiens GN=SRP72 PE=1 SV=3 - [SRP72_HUMAN]
P02768	Serum albumin OS=Homo sapiens GN=ALB PE=1 SV=2 - [ALBU_HUMAN]
Q96C19	EF-hand domain-containing protein D2 OS=Homo sapiens GN=EFHD2 PE=1 SV=1 - [EFHD2_HUMAN]
P11387	DNA topoisomerase 1 OS=Homo sapiens GN=TOP1 PE=1 SV=2 - [TOP1_HUMAN]
Q6YHU6	Thyroid adenoma-associated protein OS=Homo sapiens GN=THADA PE=1 SV=1 - [THADA_HUMAN]
Q7Z3U7	Protein MON2 homolog OS=Homo sapiens GN=MON2 PE=1 SV=3 - [MON2_HUMAN]
Q9H1A4	Anaphase-promoting complex subunit 1 OS=Homo sapiens GN=ANAPC1 PE=1 SV=1 - [APC1_HUMAN]
Q92824	Proprotein convertase subtilisin/kexin type 5 OS=Homo sapiens GN=PCSK5 PE=1 SV=4 - [PCSK5_HUMAN]
Q8IXB1	DnaJ homolog subfamily C member 10 OS=Homo sapiens GN=DNAJC10 PE=1 SV=2 - [DJC10_HUMAN]
Q8IY18	Structural maintenance of chromosomes protein 5 OS=Homo sapiens GN=SMC5 PE=1 SV=2 - [SMC5_HUMAN]
Q01085	Nucleolysin TIAR OS=Homo sapiens GN=TIAL1 PE=1 SV=1 - [TIAR_HUMAN]

Q16342	Programmed cell death protein 2 OS=Homo sapiens GN=PDCD2 PE=1 SV=2 - [PDCD2_HUMAN]
O95716	Ras-related protein Rab-3D OS=Homo sapiens GN=RAB3D PE=1 SV=1 - [RAB3D_HUMAN]
O43765	Small glutamine-rich tetratricopeptide repeat-containing protein alpha OS=Homo sapiens GN=SGTA PE=1 SV=1 - [SGTA_HUMAN]
Q5VTR2	E3 ubiquitin-protein ligase BRE1A OS=Homo sapiens GN=RNF20 PE=1 SV=2 - [BRE1A_HUMAN]
P20700	Lamin-B1 OS=Homo sapiens GN=LMNB1 PE=1 SV=2 - [LMNB1_HUMAN]
Q9Y6D5	Brefeldin A-inhibited guanine nucleotide-exchange protein 2 OS=Homo sapiens GN=ARFGEF2 PE=1 SV=3 - [BIG2_HUMAN]
Q96CS2	HAUS augmin-like complex subunit 1 OS=Homo sapiens GN=HAUS1 PE=1 SV=1 - [HAUS1_HUMAN]
P37235	Hippocalcin-like protein 1 OS=Homo sapiens GN=HPCAL1 PE=1 SV=3 - [HPCL1_HUMAN]
P19086	Guanine nucleotide-binding protein G(z) subunit alpha OS=Homo sapiens GN=GNAZ PE=2 SV=3 - [GNAZ_HUMAN]
Q9H8K7	Uncharacterized protein C10orf88 OS=Homo sapiens GN=C10orf88 PE=1 SV=2 - [CJ088_HUMAN]
P18074	TFIIH basal transcription factor complex helicase XPD subunit OS=Homo sapiens GN=ERCC2 PE=1 SV=1 - [ERCC2_HUMAN]
P00367	Glutamate dehydrogenase 1, mitochondrial OS=Homo sapiens GN=GLUD1 PE=1 SV=2 - [DHE3_HUMAN]
P15927	Replication protein A 32 kDa subunit OS=Homo sapiens GN=RPA2 PE=1 SV=1 - [RFA2_HUMAN]
Q92832	Protein kinase C-binding protein NELL1 OS=Homo sapiens GN=NELL1 PE=1 SV=4 - [NELL1_HUMAN]
Q92879	CUGBP Elav-like family member 1 OS=Homo sapiens GN=CELF1 PE=1 SV=2 - [CELF1_HUMAN]
P35221	Catenin alpha-1 OS=Homo sapiens GN=CTNNA1 PE=1 SV=1 - [CTNA1_HUMAN]
Q15758	Neutral amino acid transporter B(0) OS=Homo sapiens GN=SLC1A5 PE=1 SV=2 - [AAAT_HUMAN]
Q9C0D3	Protein zyg-11 homolog B OS=Homo sapiens GN=ZYG11B PE=1 SV=2 - [ZY11B_HUMAN]
Q9H9A5	CCR4-NOT transcription complex subunit 10 OS=Homo sapiens GN=CNOT10 PE=1 SV=1 - [CNO10_HUMAN]
P48730	Casein kinase I isoform delta OS=Homo sapiens GN=CSNK1D PE=1 SV=2 - [KC1D_HUMAN]
Q9NXC5	WD repeat-containing protein mio OS=Homo sapiens GN=MIOS PE=1 SV=2 - [MIO_HUMAN]
O75494	Serine/arginine-rich splicing factor 10 OS=Homo sapiens GN=SRSF10 PE=1 SV=1 - [SRS10_HUMAN]
Q9NVH2	Integrator complex subunit 7 OS=Homo sapiens GN=INTS7 PE=1 SV=1 - [INT7_HUMAN]
Q9BXW9	Fanconi anemia group D2 protein OS=Homo sapiens GN=FANCD2 PE=1 SV=2 - [FACD2_HUMAN]
O00399	Dynactin subunit 6 OS=Homo sapiens GN=DCTN6 PE=1 SV=1 - [DCTN6 HUMAN]
P54646	5'-AMP-activated protein kinase catalytic subunit alpha-2 OS=Homo sapiens GN=PRKAA2 PE=1 SV=2 - [AAPK2_HUMAN]
Q9Y5L0	Transportin-3 OS=Homo sapiens GN=TNPO3 PE=1 SV=3 - [TNPO3_HUMAN]
Q9BV68	E3 ubiquitin-protein ligase RNF126 OS=Homo sapiens GN=RNF126 PE=1 SV=1 - [RN126_HUMAN]
Q9Y2L5	Trafficking protein particle complex subunit 8 OS=Homo sapiens GN=TRAPPC8 PE=1 SV=2 - [TPPC8_HUMAN]
Q9Y294	Histone chaperone ASF1A OS=Homo sapiens GN=ASF1A PE=1 SV=1 - [ASF1A_HUMAN]
P06744	Glucose-6-phosphate isomerase OS=Homo sapiens GN=GPI PE=1 SV=4 - [G6PI_HUMAN]
Q9UPT5	Exocyst complex component 7 OS=Homo sapiens GN=EXOC7 PE=1 SV=3 - [EXOC7_HUMAN]
Q6P1J9	Parafibromin OS=Homo sapiens GN=CDC73 PE=1 SV=1 - [CDC73_HUMAN]
Q15528	Mediator of RNA polymerase II transcription subunit 22 OS=Homo sapiens GN=MED22 PE=1 SV=2 - [MED22_HUMAN]
Q86VX2	COMM domain-containing protein 7 OS=Homo sapiens GN=COMMD7 PE=1 SV=2 - [COMD7_HUMAN]
Q8N163	Cell cycle and apoptosis regulator protein 2 OS=Homo sapiens GN=CCAR2 PE=1 SV=2 - [CCAR2_HUMAN]
Q7Z392	Trafficking protein particle complex subunit 11 OS=Homo sapiens GN=TRAPPC11 PE=1 SV=2 - [TPC11_HUMAN]
Q9NTK5	Obg-like ATPase 1 OS=Homo sapiens GN=OLA1 PE=1 SV=2 - [OLA1_HUMAN]
Q9Y366	Intraflagellar transport protein 52 homolog OS=Homo sapiens GN=IFT52 PE=2 SV=3 - [IFT52_HUMAN]
O00255	Menin OS=Homo sapiens GN=MEN1 PE=1 SV=4 - [MEN1_HUMAN]
P41214	Eukaryotic translation initiation factor 2D OS=Homo sapiens GN=EIF2D PE=1 SV=3 - [EIF2D_HUMAN]
P54652	Heat shock-related 70 kDa protein 2 OS=Homo sapiens GN=HSPA2 PE=1 SV=1 - [HSP72_HUMAN]
P46779	60S ribosomal protein L28 OS=Homo sapiens GN=RPL28 PE=1 SV=3 - [RL28_HUMAN]
Q8ND04	Protein SMG8 OS=Homo sapiens GN=SMG8 PE=1 SV=1 - [SMG8_HUMAN]
Q15149	Plectin OS=Homo sapiens GN=PLEC PE=1 SV=3 - [PLEC_HUMAN]
P18206	Vinculin OS=Homo sapiens GN=VCL PE=1 SV=4 - [VINC_HUMAN]
P48723	Heat shock 70 kDa protein 13 OS=Homo sapiens GN=HSPA13 PE=1 SV=1 - [HSP13_HUMAN]
Q9P2D3	HEAT repeat-containing protein 5B OS=Homo sapiens GN=HEATR5B PE=1 SV=2 - [HTR5B_HUMAN]
Q7L2E3	Putative ATP-dependent RNA helicase DHX30 OS=Homo sapiens GN=DHX30 PE=1 SV=1 - [DHX30_HUMAN]
P07384	Calpain-1 catalytic subunit OS=Homo sapiens GN=CAPN1 PE=1 SV=1 - [CAN1_HUMAN]
O43399	Tumor protein D54 OS=Homo sapiens GN=TPD52L2 PE=1 SV=2 - [TPD54_HUMAN]
P53634	Dipeptidyl peptidase 1 OS=Homo sapiens GN=CTSC PE=1 SV=2 - [CATC_HUMAN]
O60716	Catenin delta-1 OS=Homo sapiens GN=CTNND1 PE=1 SV=1 - [CTND1_HUMAN]
Q96HE7	ERO1-like protein alpha OS=Homo sapiens GN=ERO1L PE=1 SV=2 - [ERO1A_HUMAN]
P67936	Tropomyosin alpha-4 chain OS=Homo sapiens GN=TPM4 PE=1 SV=3 - [TPM4_HUMAN]
Q8IUF1	COBW domain-containing protein 2 OS=Homo sapiens GN=CBWD2 PE=1 SV=1 - [CBWD2_HUMAN]
Q15477	Helicase SKI2W OS=Homo sapiens GN=SKIV2L PE=1 SV=3 - [SKIV2_HUMAN]
Q14137	Ribosome biogenesis protein BOP1 OS=Homo sapiens GN=BOP1 PE=1 SV=2 - [BOP1_HUMAN]
P78368	Casein kinase I isoform gamma-2 OS=Homo sapiens GN=CSNK1G2 PE=1 SV=1 - [KC1G2_HUMAN]
P05556	Integrin beta-1 OS=Homo sapiens GN=ITGB1 PE=1 SV=2 - [ITB1_HUMAN]
Q969Q5	Ras-related protein Rab-24 OS=Homo sapiens GN=RAB24 PE=1 SV=1 - [RAB24_HUMAN]
Q9NQW7	Xaa-Pro aminopeptidase 1 OS=Homo sapiens GN=XPNPEP1 PE=1 SV=3 - [XPP1_HUMAN]
Q8IYI6	Exocyst complex component 8 OS=Homo sapiens GN=EXOC8 PE=1 SV=2 - [EXOC8_HUMAN]
Q9H9E3	Conserved oligomeric Golgi complex subunit 4 OS=Homo sapiens GN=COG4 PE=1 SV=3 - [COG4_HUMAN]
Q9NY12	H/ACA ribonucleoprotein complex subunit 1 OS=Homo sapiens GN=GAR1 PE=1 SV=1 - [GAR1_HUMAN]
Q15102	Platelet-activating factor acetylhydrolase IB subunit gamma OS=Homo sapiens GN=PAFAH1B3 PE=1 SV=1 - [PA1B3_HUMAN]
O94979	Protein transport protein Sec31A OS=Homo sapiens GN=SEC31A PE=1 SV=3 - [SC31A_HUMAN]
Q01970	1-phosphatidylinositol 4,5-bisphosphate phosphodiesterase beta-3 OS=Homo sapiens GN=PLCB3 PE=1 SV=2 - [PLCB3_HUMAN]
Q15691	Microtubule-associated protein RP/EB family member 1 OS=Homo sapiens GN=MAPRE1 PE=1 SV=3 - [MARE1 HUMAN]
Q9BTT0	Acidic leucine-rich nuclear phosphoprotein 32 family member E OS=Homo sapiens GN=ANP32E PE=1 SV=1 - [AN32E_HUMAN]
O43657	Tetraspanin-6 OS=Homo sapiens GN=TSPAN6 PE=1 SV=1 - [TSN6_HUMAN]
P61011	Signal recognition particle 54 kDa protein OS=Homo sapiens GN=SRP54 PE=1 SV=1 - [SRP54_HUMAN]
P98095	Fibulin-2 OS=Homo sapiens GN=FBLN2 PE=1 SV=2 - [FBLN2_HUMAN]
Q04837	Single-stranded DNA-binding protein, mitochondrial OS=Homo sapiens GN=SSBP1 PE=1 SV=1 - [SSBP_HUMAN]
Q9H7X7	Intraflagellar transport protein 22 homolog OS=Homo sapiens GN=IFT22 PE=2 SV=1 - [IFT22_HUMAN]
Q9UI30	Multifunctional methyltransferase subunit TRM112-like protein OS=Homo sapiens GN=TRMT112 PE=1 SV=1 - [TR112_HUMAN]
P10301	Ras-related protein R-Ras OS=Homo sapiens GN=RRAS PE=1 SV=1 - [RRAS_HUMAN]
O14972	Down syndrome critical region protein 3 OS=Homo sapiens GN=DSCR3 PE=2 SV=1 - [DSCR3_HUMAN]
P53677	AP-3 complex subunit mu-2 OS=Homo sapiens GN=AP3M2 PE=2 SV=1 - [AP3M2_HUMAN]
Q96S55	ATPase WRNIP1 OS=Homo sapiens GN=WRNIP1 PE=1 SV=2 - [WRIP1_HUMAN]
P43243	Matrin-3 OS=Homo sapiens GN=MATR3 PE=1 SV=2 - [MATR3_HUMAN]
Q9NUQ8	ATP-binding cassette sub-family F member 3 OS=Homo sapiens GN=ABCF3 PE=1 SV=2 - [ABCF3_HUMAN]
P09972	Fructose-bisphosphate aldolase C OS=Homo sapiens GN=ALDOC PE=1 SV=2 - [ALDOC_HUMAN]
Q8N6T3	ADP-ribosylation factor GTPase-activating protein 1 OS=Homo sapiens GN=ARFGAP1 PE=1 SV=2 - [ARFG1 HUMAN]
O43815	Striatin OS=Homo sapiens GN=STRN PE=1 SV=4 - [STRN_HUMAN]
Q9BTY2	Plasma alpha-L-fucosidase OS=Homo sapiens GN=FUCA2 PE=1 SV=2 - [FUCO2_HUMAN]
C4AMC7	Putative WAS protein family homolog 3 OS=Homo sapiens GN=WASH3P PE=1 SV=2 - [WASH3_HUMAN]
Q92990	Glomulin OS=Homo sapiens GN=GLMN PE=1 SV=2 - [GLMN_HUMAN]
P63272	Transcription elongation factor SPT4 OS=Homo sapiens GN=SUPT4H1 PE=1 SV=1 - [SPT4H_HUMAN]
Q9UKL0	REST corepressor 1 OS=Homo sapiens GN=RCOR1 PE=1 SV=1 - [RCOR1 HUMAN]
P02647	Apolipoprotein A-I OS=Homo sapiens GN=APOA1 PE=1 SV=1 - [APOA1 HUMAN]
Q6STE5	SWI/SNF-related matrix-associated actin-dependent regulator of chromatin subfamily D member 3 OS=Homo sapiens GN=SMARCD3 PE=1 SV=1 - [SMRD3_HUMAN]
Q29RF7	Sister chromatid cohesion protein PDS5 homolog A OS=Homo sapiens GN=PDS5A PE=1 SV=1 - [PDS5A_HUMAN]
Q9UKY7	Protein CDV3 homolog OS=Homo sapiens GN=CDV3 PE=1 SV=1 - [CDV3 HUMAN]
O95433	Activator of 90 kDa heat shock protein ATPase homolog 1 OS=Homo sapiens GN=AHSA1 PE=1 SV=1 - [AHSA1_HUMAN]
P35613	Basigin OS=Homo sapiens GN=BSG PE=1 SV=2 - [BASI_HUMAN]
Q12805	EGF-containing fibulin-like extracellular matrix protein 1 OS=Homo sapiens GN=EFEMP1 PE=1 SV=2 - [FBLN3_HUMAN]
Q9BV38	WD repeat-containing protein 18 OS=Homo sapiens GN=WDR18 PE=1 SV=2 - [WDR18_HUMAN]
P32121	Beta-arrestin-2 OS=Homo sapiens GN=ARRB2 PE=1 SV=2 - [ARRB2_HUMAN]
Q9NQS1	Cell death regulator Aven OS=Homo sapiens GN=AVEN PE=1 SV=1 - [AVEN_HUMAN]
P53041	Serine/threonine-protein phosphatase 5 OS=Homo sapiens GN=PPP5C PE=1 SV=1 - [PPP5_HUMAN]
P01034	Cystatin-C OS=Homo sapiens GN=CST3 PE=1 SV=1 - [CYTC_HUMAN]
Q12840	Kinesin heavy chain isoform 5A OS=Homo sapiens GN=KIF5A PE=1 SV=2 - [KIF5A_HUMAN]
Q96GD4	Aurora kinase B OS=Homo sapiens GN=AURKB PE=1 SV=3 - [AURKB_HUMAN]
A4D1P6	WD repeat-containing protein 91 OS=Homo sapiens GN=WDR91 PE=1 SV=2 - [WDR91_HUMAN]
Q96QR8	Transcriptional activator protein Pur-beta OS=Homo sapiens GN=PURB PE=1 SV=3 - [PURB_HUMAN]
Q9BTE7	DCN1-like protein 5 OS=Homo sapiens GN=DCUN1D5 PE=1 SV=1 - [DCNL5_HUMAN]
Q9NVM9	Protein asunder homolog OS=Homo sapiens GN=ASUN PE=1 SV=2 - [ASUN_HUMAN]
P42858	Huntingtin OS=Homo sapiens GN=HTT PE=1 SV=2 - [HD_HUMAN]
P21926	CD9 antigen OS=Homo sapiens GN=CD9 PE=1 SV=4 - [CD9_HUMAN]
Q9H9G7	Protein argonaute-3 OS=Homo sapiens GN=AGO3 PE=1 SV=2 - [AGO3_HUMAN]
Q06787	Fragile X mental retardation protein 1 OS=Homo sapiens GN=FMR1 PE=1 SV=1 - [FMR1_HUMAN]
Q96FW1	Ubiquitin thioesterase OTUB1 OS=Homo sapiens GN=OTUB1 PE=1 SV=2 - [OTUB1_HUMAN]
O94966	Ubiquitin carboxyl-terminal hydrolase 19 OS=Homo sapiens GN=USP19 PE=1 SV=2 - [UBP19_HUMAN]
P98179	RNA-binding protein 3 OS=Homo sapiens GN=RBM3 PE=1 SV=1 - [RBM3_HUMAN]

Q96HY6	DDRGK domain-containing protein 1 OS=Homo sapiens GN=DDRGK1 PE=1 SV=2 - [DDRGK_HUMAN]
Q9UJC3	Protein Hook homolog 1 OS=Homo sapiens GN=HOOK1 PE=1 SV=2 - [HOOK1_HUMAN]
P52594	Arf-GAP domain and FG repeat-containing protein 1 OS=Homo sapiens GN=AGFG1 PE=1 SV=2 - [AGFG1_HUMAN]
Q9NRL3	Striatin-4 OS=Homo sapiens GN=STRN4 PE=1 SV=2 - [STRN4_HUMAN]
P99999	Cytochrome c OS=Homo sapiens GN=CYCS PE=1 SV=2 - [CYC_HUMAN]
Q96S44	TP53-regulating kinase OS=Homo sapiens GN=TP53RK PE=1 SV=2 - [PRPK_HUMAN]
Q99707	Methionine synthase OS=Homo sapiens GN=MTR PE=1 SV=2 - [METH_HUMAN]
P50914	60S ribosomal protein L14 OS=Homo sapiens GN=RPL14 PE=1 SV=4 - [RL14_HUMAN]
Q9BVI4	Nucleolar complex protein 4 homolog OS=Homo sapiens GN=NOC4L PE=1 SV=1 - [NOC4L_HUMAN]
P51570	Galactokinase OS=Homo sapiens GN=GALK1 PE=1 SV=1 - [GALK1_HUMAN]
O75197	Low-density lipoprotein receptor-related protein 5 OS=Homo sapiens GN=LRP5 PE=1 SV=2 - [LRP5_HUMAN]
Q02750	Dual specificity mitogen-activated protein kinase kinase 1 OS=Homo sapiens GN=MAP2K1 PE=1 SV=2 - [MP2K1_HUMAN]
O95631	Netrin-1 OS=Homo sapiens GN=NTN1 PE=1 SV=2 - [NET1_HUMAN]
P78344	Eukaryotic translation initiation factor 4 gamma 2 OS=Homo sapiens GN=EIF4G2 PE=1 SV=1 - [IF4G2_HUMAN]
P16278	Beta-galactosidase OS=Homo sapiens GN=GLB1 PE=1 SV=2 - [BGAL_HUMAN]
P62995	Transformer-2 protein homolog beta OS=Homo sapiens GN=TRA2B PE=1 SV=1 - [TRA2B_HUMAN]
Q6P9B6	TLD domain-containing protein 1 OS=Homo sapiens GN=TLDC1 PE=1 SV=2 - [TLDC1_HUMAN]
Q9Y6I3	Epsin-1 OS=Homo sapiens GN=EPN1 PE=1 SV=2 - [EPN1_HUMAN]
P08621	U1 small nuclear ribonucleoprotein 70 kDa OS=Homo sapiens GN=SNRNP70 PE=1 SV=2 - [RU17_HUMAN]
Q7L273	BTB/POZ domain-containing protein KCTD9 OS=Homo sapiens GN=KCTD9 PE=1 SV=1 - [KCTD9_HUMAN]
Q9Y6D6	Brefeldin A-inhibited guanine nucleotide-exchange protein 1 OS=Homo sapiens GN=ARFGEF1 PE=1 SV=2 - [BIG1_HUMAN]
P28676	Grancalcin OS=Homo sapiens GN=GCA PE=1 SV=2 - [GRAN_HUMAN]
Q562R1	Beta-actin-like protein 2 OS=Homo sapiens GN=ACTBL2 PE=1 SV=2 - [ACTBL_HUMAN]
Q8NG11	Tetraspanin-14 OS=Homo sapiens GN=TSPAN14 PE=1 SV=1 - [TSN14_HUMAN]
Q9P2W1	Homologous-pairing protein 2 homolog OS=Homo sapiens GN=PSMC3IP PE=1 SV=1 - [HOP2_HUMAN]
Q9H2U1	ATP-dependent RNA helicase DHX36 OS=Homo sapiens GN=DHX36 PE=1 SV=2 - [DHX36_HUMAN]
Q92888	Rho guanine nucleotide exchange factor 1 OS=Homo sapiens GN=ARHGEF1 PE=1 SV=2 - [ARHG1_HUMAN]
Q9BQT9	Calsyntenin-3 OS=Homo sapiens GN=CLSTN3 PE=1 SV=1 - [CSTN3_HUMAN]
Q9UFC0	Leucine-rich repeat and WD repeat-containing protein 1 OS=Homo sapiens GN=LRWD1 PE=1 SV=2 - [LRWD1_HUMAN]
O00584	Ribonuclease T2 OS=Homo sapiens GN=RNASET2 PE=1 SV=2 - [RNT2_HUMAN]
P07948	Tyrosine-protein kinase Lyn OS=Homo sapiens GN=LYN PE=1 SV=3 - [LYN_HUMAN]
O43592	Exportin-T OS=Homo sapiens GN=XPOT PE=1 SV=2 - [XPOT_HUMAN]
P51116	Fragile X mental retardation syndrome-related protein 2 OS=Homo sapiens GN=FXR2 PE=1 SV=2 - [FXR2 HUMAN]
Q9BV86	N-terminal Xaa-Pro-Lys N-methyltransferase 1 OS=Homo sapiens GN=NTMT1 PE=1 SV=3 - [NTM1A_HUMAN]
Q9BUR4	Telomerase Cajal body protein 1 OS=Homo sapiens GN=WRAP53 PE=1 SV=1 - [WAP53_HUMAN]
O95232	Luc7-like protein 3 OS=Homo sapiens GN=LUC7L3 PE=1 SV=2 - [LC7L3_HUMAN]
Q9BWF3	RNA-binding protein 4 OS=Homo sapiens GN=RBM4 PE=1 SV=1 - [RBM4_HUMAN]
P42704	Leucine-rich PPR motif-containing protein, mitochondrial OS=Homo sapiens GN=LRPPRC PE=1 SV=3 - [LPPRC_HUMAN]
Q86Z23	Complement C1q-like protein 4 OS=Homo sapiens GN=C1QL4 PE=2 SV=1 - [C1QL4_HUMAN]
Q8NC51	Plasminogen activator inhibitor 1 RNA-binding protein OS=Homo sapiens GN=SERBP1 PE=1 SV=2 - [PAIRB_HUMAN]
Q92542	Nicastrin OS=Homo sapiens GN=NCSTN PE=1 SV=2 - [NICA_HUMAN]
Q9UNN8	Endothelial protein C receptor OS=Homo sapiens GN=PROCR PE=1 SV=1 - [EPCR_HUMAN]
Q9UHX1	Poly(U)-binding-splicing factor PUF60 OS=Homo sapiens GN=PUF60 PE=1 SV=1 - [PUF60_HUMAN]
Q92882	Osteoclast-stimulating factor 1 OS=Homo sapiens GN=OSTF1 PE=1 SV=2 - [OSTF1_HUMAN]
Q9Y625	Glypican-6 OS=Homo sapiens GN=GPC6 PE=1 SV=1 - [GPC6_HUMAN]
A3KN83	Protein strawberry notch homolog 1 OS=Homo sapiens GN=SBNO1 PE=1 SV=1 - [SBNO1_HUMAN]
P52298	Nuclear cap-binding protein subunit 2 OS=Homo sapiens GN=NCBP2 PE=1 SV=1 - [NCBP2_HUMAN]
O15067	Phosphoribosylformylglycinamidine synthase OS=Homo sapiens GN=PFAS PE=1 SV=4 - [PUR4_HUMAN]
P57772	Selenocysteine-specific elongation factor OS=Homo sapiens GN=EEFSEC PE=1 SV=4 - [SELB_HUMAN]
Q86VN1	Vacuolar protein-sorting-associated protein 36 OS=Homo sapiens GN=VPS36 PE=1 SV=1 - [VPS36_HUMAN]
Q969U7	Proteasome assembly chaperone 2 OS=Homo sapiens GN=PSMG2 PE=1 SV=1 - [PSMG2_HUMAN]
Q15750	TGF-beta-activated kinase 1 and MAP3K7-binding protein 1 OS=Homo sapiens GN=TAB1 PE=1 SV=1 - [TAB1_HUMAN]
O75530	Polycomb protein EED OS=Homo sapiens GN=EED PE=1 SV=2 - [EED_HUMAN]
O95858	Tetraspanin-15 OS=Homo sapiens GN=TSPAN15 PE=1 SV=1 - [TSN15_HUMAN]
O94808	Glutamine--fructose-6-phosphate aminotransferase [isomerizing] 2 OS=Homo sapiens GN=GFPT2 PE=1 SV=3 - [GFPT2_HUMAN]
P23381	Tryptophan--tRNA ligase, cytoplasmic OS=Homo sapiens GN=WARS PE=1 SV=2 - [SYWC_HUMAN]
Q8IUX4	DNA dC->dU-editing enzyme APOBEC-3F OS=Homo sapiens GN=APOBEC3F PE=1 SV=3 - [ABC3F_HUMAN]
Q9Y2P8	RNA 3'-terminal phosphate cyclase-like protein OS=Homo sapiens GN=RCL1 PE=1 SV=3 - [RCL1_HUMAN]
P62861	40S ribosomal protein S30 OS=Homo sapiens GN=FAU PE=1 SV=1 - [RS30_HUMAN]
O95373	Importin-7 OS=Homo sapiens GN=IPO7 PE=1 SV=1 - [IPO7_HUMAN]
Q5SRE5	Nucleoporin NUP188 homolog OS=Homo sapiens GN=NUP188 PE=1 SV=1 - [NU188_HUMAN]
Q9NQ55	Suppressor of SWI4 1 homolog OS=Homo sapiens GN=PPAN PE=1 SV=1 - [SSF1_HUMAN]
Q9UBQ6	Exostosin-like 2 OS=Homo sapiens GN=EXTL2 PE=1 SV=1 - [EXTL2_HUMAN]
P49840	Glycogen synthase kinase-3 alpha OS=Homo sapiens GN=GSK3A PE=1 SV=2 - [GSK3A_HUMAN]
Q99543	DnaJ homolog subfamily C member 2 OS=Homo sapiens GN=DNAJC2 PE=1 SV=4 - [DNJC2_HUMAN]
Q9UQB8	Brain-specific angiogenesis inhibitor 1-associated protein 2 OS=Homo sapiens GN=BAIAP2 PE=1 SV=1 - [BAIP2_HUMAN]
O43633	Charged multivesicular body protein 2a OS=Homo sapiens GN=CHMP2A PE=1 SV=1 - [CHM2A_HUMAN]
P49754	Vacuolar protein sorting-associated protein 41 homolog OS=Homo sapiens GN=VPS41 PE=1 SV=3 - [VPS41_HUMAN]
P61966	AP-1 complex subunit sigma-1A OS=Homo sapiens GN=AP1S1 PE=1 SV=1 - [AP1S1_HUMAN]
Q14517	Protocadherin Fat 1 OS=Homo sapiens GN=FAT1 PE=1 SV=2 - [FAT1_HUMAN]
Q93099	Homogentisate 1,2-dioxygenase OS=Homo sapiens GN=HGD PE=1 SV=2 - [HGD_HUMAN]
Q9NTX5	Ethylmalonyl-CoA decarboxylase OS=Homo sapiens GN=ECHDC1 PE=1 SV=2 - [ECHD1_HUMAN]
O14817	Tetraspanin-4 OS=Homo sapiens GN=TSPAN4 PE=1 SV=1 - [TSN4_HUMAN]
P49585	Choline-phosphate cytidylyltransferase A OS=Homo sapiens GN=PCYT1A PE=1 SV=2 - [PCY1A_HUMAN]
Q9P0N9	TBC1 domain family member 7 OS=Homo sapiens GN=TBC1D7 PE=1 SV=1 - [TBCD7_HUMAN]
Q8N3C0	Activating signal cointegrator 1 complex subunit 3 OS=Homo sapiens GN=ASCC3 PE=1 SV=3 - [ASCC3_HUMAN]
P57740	Nuclear pore complex protein Nup107 OS=Homo sapiens GN=NUP107 PE=1 SV=1 - [NU107_HUMAN]
Q99871	HAUS augmin-like complex subunit 7 OS=Homo sapiens GN=HAUS7 PE=1 SV=3 - [HAUS7_HUMAN]
Q9H0X4	Protein ITFG3 OS=Homo sapiens GN=ITFG3 PE=1 SV=1 - [ITFG3_HUMAN]
Q9Y3P9	Rab GTPase-activating protein 1 OS=Homo sapiens GN=RABGAP1 PE=1 SV=3 - [RBGP1_HUMAN]
Q96Q15	Serine/threonine-protein kinase SMG1 OS=Homo sapiens GN=SMG1 PE=1 SV=3 - [SMG1_HUMAN]
P52209	6-phosphogluconate dehydrogenase, decarboxylating OS=Homo sapiens GN=PGD PE=1 SV=3 - [6PGD_HUMAN]
O95639	Cleavage and polyadenylation specificity factor subunit 4 OS=Homo sapiens GN=CPSF4 PE=1 SV=1 - [CPSF4_HUMAN]
P48507	Glutamate--cysteine ligase regulatory subunit OS=Homo sapiens GN=GCLM PE=1 SV=1 - [GSH0_HUMAN]
Q9HCC0	Methylcrotonoyl-CoA carboxylase beta chain, mitochondrial OS=Homo sapiens GN=MCCC2 PE=1 SV=1 - [MCCB_HUMAN]
Q9UL18	Protein argonaute-1 OS=Homo sapiens GN=AGO1 PE=1 SV=3 - [AGO1_HUMAN]
Q6P158	Putative ATP-dependent RNA helicase DHX57 OS=Homo sapiens GN=DHX57 PE=1 SV=2 - [DHX57_HUMAN]
Q9NSB8	Homer protein homolog 2 OS=Homo sapiens GN=HOMER2 PE=1 SV=1 - [HOME2_HUMAN]
P49821	NADH dehydrogenase [ubiquinone] flavoprotein 1, mitochondrial OS=Homo sapiens GN=NDUFV1 PE=1 SV=4 - [NDUV1_HUMAN]
O43617	Trafficking protein particle complex subunit 3 OS=Homo sapiens GN=TRAPPC3 PE=1 SV=1 - [TPPC3_HUMAN]
O94766	Galactosylgalactosylxylosylprotein 3-beta-glucuronosyltransferase 3 OS=Homo sapiens GN=B3GAT3 PE=1 SV=2 - [B3GA3_HUMAN]
Q52LJ0	Protein FAM98B OS=Homo sapiens GN=FAM98B PE=1 SV=1 - [FA98B_HUMAN]
Q6PI48	Aspartate--tRNA ligase, mitochondrial OS=Homo sapiens GN=DARS2 PE=1 SV=1 - [SYDM_HUMAN]
Q9HCS7	Pre-mRNA-splicing factor SYF1 OS=Homo sapiens GN=XAB2 PE=1 SV=2 - [SYF1_HUMAN]
Q96JC1	Vam6/Vps39-like protein OS=Homo sapiens GN=VPS39 PE=1 SV=2 - [VPS39_HUMAN]
P35222	Catenin beta-1 OS=Homo sapiens GN=CTNNB1 PE=1 SV=1 - [CTNB1_HUMAN]
P48426	Phosphatidylinositol 5-phosphate 4-kinase type-2 alpha OS=Homo sapiens GN=PIP4K2A PE=1 SV=2 - [PI42A_HUMAN]
Q04637	Eukaryotic translation initiation factor 4 gamma 1 OS=Homo sapiens GN=EIF4G1 PE=1 SV=4 - [IF4G1_HUMAN]
Q8TBX8	Phosphatidylinositol 5-phosphate 4-kinase type-2 gamma OS=Homo sapiens GN=PIP4K2C PE=1 SV=3 - [PI42C_HUMAN]
P53675	Clathrin heavy chain 2 OS=Homo sapiens GN=CLTCL1 PE=1 SV=2 - [CLH2_HUMAN]
Q9H7B2	Ribosome production factor 2 homolog OS=Homo sapiens GN=RPF2 PE=1 SV=2 - [RPF2_HUMAN]
Q12789	General transcription factor 3C polypeptide 1 OS=Homo sapiens GN=GTF3C1 PE=1 SV=4 - [TF3C1_HUMAN]
P57081	tRNA (guanine-N(7)-)-methyltransferase non-catalytic subunit WDR4 OS=Homo sapiens GN=WDR4 PE=1 SV=2 - [WDR4_HUMAN]
P50750	Cyclin-dependent kinase 9 OS=Homo sapiens GN=CDK9 PE=1 SV=3 - [CDK9_HUMAN]
P48960	CD97 antigen OS=Homo sapiens GN=CD97 PE=1 SV=4 - [CD97_HUMAN]
Q99729	Heterogeneous nuclear ribonucleoprotein A/B OS=Homo sapiens GN=HNRNPAB PE=1 SV=2 - [ROAA_HUMAN]
P22695	Cytochrome b-c1 complex subunit 2, mitochondrial OS=Homo sapiens GN=UQCRC2 PE=1 SV=3 - [QCR2_HUMAN]
P60510	Serine/threonine-protein phosphatase 4 catalytic subunit OS=Homo sapiens GN=PPP4C PE=1 SV=1 - [PP4C_HUMAN]
Q9Y6M1	Insulin-like growth factor 2 mRNA-binding protein 2 OS=Homo sapiens GN=IGF2BP2 PE=1 SV=2 - [IF2B2 HUMAN]
P09012	U1 small nuclear ribonucleoprotein A OS=Homo sapiens GN=SNRPA PE=1 SV=3 - [SNRPA_HUMAN]
O60645	Exocyst complex component 3 OS=Homo sapiens GN=EXOC3 PE=1 SV=2 - [EXOC3_HUMAN]
Q8NEB9	Phosphatidylinositol 3-kinase catalytic subunit type 3 OS=Homo sapiens GN=PIK3C3 PE=1 SV=1 - [PK3C3_HUMAN]
O00469	Procollagen-lysine,2-oxoglutarate 5-dioxygenase 2 OS=Homo sapiens GN=PLOD2 PE=1 SV=2 - [PLOD2_HUMAN]
Q96GA3	Protein LTV1 homolog OS=Homo sapiens GN=LTV1 PE=1 SV=1 - [LTV1 HUMAN]
Q9NRG9	Aladin OS=Homo sapiens GN=AAAS PE=1 SV=1 - [AAAS_HUMAN]

Q9P1F3	Costars family protein ABRACL OS=Homo sapiens GN=ABRACL PE=1 SV=1 - [ABRAL_HUMAN]
Q92878	DNA repair protein RAD50 OS=Homo sapiens GN=RAD50 PE=1 SV=1 - [RAD50_HUMAN]
Q6PKG0	La-related protein 1 OS=Homo sapiens GN=LARP1 PE=1 SV=2 - [LARP1_HUMAN]
Q9UHD9	Ubiquilin-2 OS=Homo sapiens GN=UBQLN2 PE=1 SV=2 - [UBQL2_HUMAN]
P45974	Ubiquitin carboxyl-terminal hydrolase 5 OS=Homo sapiens GN=USP5 PE=1 SV=2 - [UBP5_HUMAN]
Q5H9R7	Serine/threonine-protein phosphatase 6 regulatory subunit 3 OS=Homo sapiens GN=PPP6R3 PE=1 SV=2 - [PP6R3_HUMAN]
P02795	Metallothionein-2 OS=Homo sapiens GN=MT2A PE=1 SV=1 - [MT2_HUMAN]
Q8N0X7	Spartin OS=Homo sapiens GN=SPG20 PE=1 SV=1 - [SPG20_HUMAN]
P13674	Prolyl 4-hydroxylase subunit alpha-1 OS=Homo sapiens GN=P4HA1 PE=1 SV=2 - [P4HA1_HUMAN]
Q9H0L4	Cleavage stimulation factor subunit 2 tau variant OS=Homo sapiens GN=CSTF2T PE=1 SV=1 - [CSTFT_HUMAN]
Q9Y383	Putative RNA-binding protein Luc7-like 2 OS=Homo sapiens GN=LUC7L2 PE=1 SV=2 - [LC7L2_HUMAN]
B9A064	Immunoglobulin lambda-like polypeptide 5 OS=Homo sapiens GN=IGLL5 PE=2 SV=2 - [IGLL5_HUMAN]
P55084	Trifunctional enzyme subunit beta, mitochondrial OS=Homo sapiens GN=HADHB PE=1 SV=3 - [ECHB_HUMAN]
P51610	Host cell factor 1 OS=Homo sapiens GN=HCFC1 PE=1 SV=2 - [HCFC1_HUMAN]
P62891	60S ribosomal protein L39 OS=Homo sapiens GN=RPL39 PE=1 SV=2 - [RL39_HUMAN]
P23193	Transcription elongation factor A protein 1 OS=Homo sapiens GN=TCEA1 PE=1 SV=2 - [TCEA1_HUMAN]
Q06265	Exosome complex component RRP45 OS=Homo sapiens GN=EXOSC9 PE=1 SV=3 - [EXOS9_HUMAN]
Q08629	Testican-1 OS=Homo sapiens GN=SPOCK1 PE=1 SV=1 - [TICN1_HUMAN]
Q9NTI5	Sister chromatid cohesion protein PDS5 homolog B OS=Homo sapiens GN=PDS5B PE=1 SV=1 - [PDS5B_HUMAN]
Q14694	Ubiquitin carboxyl-terminal hydrolase 10 OS=Homo sapiens GN=USP10 PE=1 SV=2 - [UBP10_HUMAN]
Q96SB4	SRSF protein kinase 1 OS=Homo sapiens GN=SRPK1 PE=1 SV=2 - [SRPK1_HUMAN]
Q9UFF9	CCR4-NOT transcription complex subunit 8 OS=Homo sapiens GN=CNOT8 PE=1 SV=1 - [CNOT8_HUMAN]
Q96DH6	RNA-binding protein Musashi homolog 2 OS=Homo sapiens GN=MSI2 PE=1 SV=1 - [MSI2H_HUMAN]
P11177	Pyruvate dehydrogenase E1 component subunit beta, mitochondrial OS=Homo sapiens GN=PDHB PE=1 SV=3 - [ODPB_HUMAN]
P29992	Guanine nucleotide-binding protein subunit alpha-11 OS=Homo sapiens GN=GNA11 PE=1 SV=2 - [GNA11_HUMAN]
Q96T51	RUN and FYVE domain-containing protein 1 OS=Homo sapiens GN=RUFY1 PE=1 SV=2 - [RUFY1_HUMAN]
Q8WV99	AN1-type zinc finger protein 2B OS=Homo sapiens GN=ZFAND2B PE=1 SV=1 - [ZFN2B_HUMAN]
P78310	Coxsackievirus and adenovirus receptor OS=Homo sapiens GN=CXADR PE=1 SV=1 - [CXAR_HUMAN]
Q7L9L4	MOB kinase activator 1B OS=Homo sapiens GN=MOB1B PE=1 SV=3 - [MOB1B_HUMAN]
P49189	4-trimethylaminobutyraldehyde dehydrogenase OS=Homo sapiens GN=ALDH9A1 PE=1 SV=3 - [AL9A1_HUMAN]
O75964	ATP synthase subunit g, mitochondrial OS=Homo sapiens GN=ATP5L PE=1 SV=3 - [ATP5L_HUMAN]
P30043	Flavin reductase (NADPH) OS=Homo sapiens GN=BLVRB PE=1 SV=3 - [BLVRB_HUMAN]
O43252	Bifunctional 3'-phosphoadenosine 5'-phosphosulfate synthase 1 OS=Homo sapiens GN=PAPSS1 PE=1 SV=2 - [PAPS1_HUMAN]
P21283	V-type proton ATPase subunit C 1 OS=Homo sapiens GN=ATP6V1C1 PE=1 SV=4 - [VATC1_HUMAN]
Q14839	Chromodomain-helicase-DNA-binding protein 4 OS=Homo sapiens GN=CHD4 PE=1 SV=2 - [CHD4_HUMAN]
Q5VW36	Focadhesin OS=Homo sapiens GN=FOCAD PE=1 SV=1 - [FOCAD_HUMAN]
O60749	Sorting nexin-2 OS=Homo sapiens GN=SNX2 PE=1 SV=2 - [SNX2_HUMAN]
P28838	Cytosol aminopeptidase OS=Homo sapiens GN=LAP3 PE=1 SV=3 - [AMPL_HUMAN]
P55010	Eukaryotic translation initiation factor 5 OS=Homo sapiens GN=EIF5 PE=1 SV=2 - [IF5_HUMAN]
Q8TF46	DIS3-like exonuclease 1 OS=Homo sapiens GN=DIS3L PE=1 SV=2 - [DI3L1_HUMAN]
Q7Z406	Myosin-14 OS=Homo sapiens GN=MYH14 PE=1 SV=2 - [MYH14_HUMAN]
Q13444	Disintegrin and metalloproteinase domain-containing protein 15 OS=Homo sapiens GN=ADAM15 PE=1 SV=4 - [ADA15_HUMAN]
Q9Y6M4	Casein kinase I isoform gamma-3 OS=Homo sapiens GN=CSNK1G3 PE=1 SV=2 - [KC1G3_HUMAN]
P43121	Cell surface glycoprotein MUC18 OS=Homo sapiens GN=MCAM PE=1 SV=2 - [MUC18_HUMAN]
O95390	Growth/differentiation factor 11 OS=Homo sapiens GN=GDF11 PE=2 SV=1 - [GDF11_HUMAN]
O95861	3'(2'),5'-bisphosphate nucleotidase 1 OS=Homo sapiens GN=BPNT1 PE=1 SV=1 - [BPNT1_HUMAN]
Q8WX92	Negative elongation factor B OS=Homo sapiens GN=NELFB PE=1 SV=1 - [NELFB_HUMAN]
Q15404	Ras suppressor protein 1 OS=Homo sapiens GN=RSU1 PE=1 SV=3 - [RSU1_HUMAN]
Q9Y535	DNA-directed RNA polymerase III subunit RPC8 OS=Homo sapiens GN=POLR3H PE=1 SV=1 - [RPC8_HUMAN]
Q96KR1	Zinc finger RNA-binding protein OS=Homo sapiens GN=ZFR PE=1 SV=2 - [ZFR_HUMAN]
Q86X52	Chondroitin sulfate synthase 1 OS=Homo sapiens GN=CHSY1 PE=1 SV=3 - [CHSS1_HUMAN]
Q9BQ70	Transcription factor 25 OS=Homo sapiens GN=TCF25 PE=1 SV=1 - [TCF25_HUMAN]
O00273	DNA fragmentation factor subunit alpha OS=Homo sapiens GN=DFFA PE=1 SV=1 - [DFFA_HUMAN]
Q8NI36	WD repeat-containing protein 36 OS=Homo sapiens GN=WDR36 PE=1 SV=1 - [WDR36_HUMAN]
Q96SU4	Oxysterol-binding protein-related protein 9 OS=Homo sapiens GN=OSBPL9 PE=1 SV=2 - [OSBL9_HUMAN]
O15514	DNA-directed RNA polymerase II subunit RPB4 OS=Homo sapiens GN=POLR2D PE=1 SV=1 - [RPB4_HUMAN]
P30566	Adenylosuccinate lyase OS=Homo sapiens GN=ADSL PE=1 SV=2 - [PUR8_HUMAN]
Q9NUS5	AP-5 complex subunit sigma-1 OS=Homo sapiens GN=AP5S1 PE=1 SV=1 - [AP5S1_HUMAN]
O94855	Protein transport protein Sec24D OS=Homo sapiens GN=SEC24D PE=1 SV=2 - [SC24D_HUMAN]
O00154	Cytosolic acyl coenzyme A thioester hydrolase OS=Homo sapiens GN=ACOT7 PE=1 SV=3 - [BACH_HUMAN]
P31947	14-3-3 protein sigma OS=Homo sapiens GN=SFN PE=1 SV=1 - [1433S_HUMAN]
Q14008	Cytoskeleton-associated protein 5 OS=Homo sapiens GN=CKAP5 PE=1 SV=3 - [CKAP5_HUMAN]
Q6ZVM7	TOM1-like protein 2 OS=Homo sapiens GN=TOM1L2 PE=1 SV=1 - [TM1L2_HUMAN]
Q9Y617	Phosphoserine aminotransferase OS=Homo sapiens GN=PSAT1 PE=1 SV=2 - [SERC_HUMAN]
Q09328	Alpha-1,6-mannosylglycoprotein 6-beta-N-acetylglucosaminyltransferase A OS=Homo sapiens GN=MGAT5 PE=2 SV=1 - [MGT5A_HUMAN]
Q06481	Amyloid-like protein 2 OS=Homo sapiens GN=APLP2 PE=1 SV=2 - [APLP2_HUMAN]
Q6RW13	Type-1 angiotensin II receptor-associated protein OS=Homo sapiens GN=AGTRAP PE=1 SV=1 - [ATRAP_HUMAN]
P51617	Interleukin-1 receptor-associated kinase 1 OS=Homo sapiens GN=IRAK1 PE=1 SV=2 - [IRAK1_HUMAN]
Q9H773	dCTP pyrophosphatase 1 OS=Homo sapiens GN=DCTPP1 PE=1 SV=1 - [DCTP1_HUMAN]
Q9NRR5	Ubiquilin-4 OS=Homo sapiens GN=UBQLN4 PE=1 SV=2 - [UBQL4_HUMAN]
Q13595	Transformer-2 protein homolog alpha OS=Homo sapiens GN=TRA2A PE=1 SV=1 - [TRA2A_HUMAN]
Q66K74	Microtubule-associated protein 1S OS=Homo sapiens GN=MAP1S PE=1 SV=2 - [MAP1S_HUMAN]
Q8N999	Uncharacterized protein C12orf29 OS=Homo sapiens GN=C12orf29 PE=1 SV=2 - [CL029_HUMAN]
P10646	Tissue factor pathway inhibitor OS=Homo sapiens GN=TFPI PE=1 SV=1 - [TFPI1_HUMAN]
P20340	Ras-related protein Rab-6A OS=Homo sapiens GN=RAB6A PE=1 SV=3 - [RAB6A_HUMAN]
Q15428	Splicing factor 3A subunit 2 OS=Homo sapiens GN=SF3A2 PE=1 SV=2 - [SF3A2_HUMAN]
Q9Y263	Phospholipase A-2-activating protein OS=Homo sapiens GN=PLAA PE=1 SV=2 - [PLAP_HUMAN]
P35611	Alpha-adducin OS=Homo sapiens GN=ADD1 PE=1 SV=2 - [ADDA_HUMAN]
Q8IUF8	Bifunctional lysine-specific demethylase and histidyl-hydroxylase MINA OS=Homo sapiens GN=MINA PE=1 SV=1 - [MINA_HUMAN]
Q9ULK4	Mediator of RNA polymerase II transcription subunit 23 OS=Homo sapiens GN=MED23 PE=1 SV=2 - [MED23_HUMAN]
Q567U6	Coiled-coil domain-containing protein 93 OS=Homo sapiens GN=CCDC93 PE=1 SV=2 - [CCD93_HUMAN]
Q9BZM5	NKG2D ligand 2 OS=Homo sapiens GN=ULBP2 PE=1 SV=1 - [N2DL2_HUMAN]
Q04206	Transcription factor p65 OS=Homo sapiens GN=RELA PE=1 SV=2 - [TF65_HUMAN]
Q9H0S4	Probable ATP-dependent RNA helicase DDX47 OS=Homo sapiens GN=DDX47 PE=1 SV=1 - [DDX47_HUMAN]
Q8IVV7	Glucose-induced degradation protein 4 homolog OS=Homo sapiens GN=GID4 PE=2 SV=1 - [GID4_HUMAN]
Q8TC07	TBC1 domain family member 15 OS=Homo sapiens GN=TBC1D15 PE=1 SV=2 - [TBC15_HUMAN]
Q9BQG0	Myb-binding protein 1A OS=Homo sapiens GN=MYBBP1A PE=1 SV=2 - [MBB1A_HUMAN]
O95486	Protein transport protein Sec24A OS=Homo sapiens GN=SEC24A PE=1 SV=2 - [SC24A_HUMAN]
Q8WUA4	General transcription factor 3C polypeptide 2 OS=Homo sapiens GN=GTF3C2 PE=1 SV=2 - [TF3C2_HUMAN]
Q9UBB6	Neurochondrin OS=Homo sapiens GN=NCDN PE=1 SV=1 - [NCDN_HUMAN]
Q9Y6Y8	SEC23-interacting protein OS=Homo sapiens GN=SEC23IP PE=1 SV=1 - [S23IP_HUMAN]
Q9UKX7	Nuclear pore complex protein Nup50 OS=Homo sapiens GN=NUP50 PE=1 SV=2 - [NUP50_HUMAN]
Q13185	Chromobox protein homolog 3 OS=Homo sapiens GN=CBX3 PE=1 SV=4 - [CBX3_HUMAN]
Q9UKF6	Cleavage and polyadenylation specificity factor subunit 3 OS=Homo sapiens GN=CPSF3 PE=1 SV=1 - [CPSF3_HUMAN]
O00541	Pescadillo homolog OS=Homo sapiens GN=PES1 PE=1 SV=1 - [PESC_HUMAN]
Q6L9W6	Beta-1,4-N-acetylgalactosaminyltransferase 3 OS=Homo sapiens GN=B4GALNT3 PE=1 SV=2 - [B4GN3_HUMAN]
O60763	General vesicular transport factor p115 OS=Homo sapiens GN=USO1 PE=1 SV=2 - [USO1_HUMAN]
Q15269	Periodic tryptophan protein 2 homolog OS=Homo sapiens GN=PWP2 PE=1 SV=2 - [PWP2_HUMAN]
Q8N428	Polypeptide N-acetylgalactosaminyltransferase 16 OS=Homo sapiens GN=GALNT16 PE=1 SV=2 - [GLT16_HUMAN]
Q12769	Nuclear pore complex protein Nup160 OS=Homo sapiens GN=NUP160 PE=1 SV=3 - [NU160_HUMAN]
Q9NRN7	L-aminoadipate-semialdehyde dehydrogenase-phosphopantetheinyl transferase OS=Homo sapiens GN=AASDHPPT PE=1 SV=2 - [ADPPT_HUMAN]
P50502	Hsc70-interacting protein OS=Homo sapiens GN=ST13 PE=1 SV=2 - [F10A1_HUMAN]
Q96FV9	THO complex subunit 1 OS=Homo sapiens GN=THOC1 PE=1 SV=1 - [THOC1_HUMAN]
O43427	Acidic fibroblast growth factor intracellular-binding protein OS=Homo sapiens GN=FIBP PE=1 SV=3 - [FIBP_HUMAN]
Q7Z569	BRCA1-associated protein OS=Homo sapiens GN=BRAP PE=1 SV=2 - [BRAP_HUMAN]
Q9BW66	Cyclin-dependent kinase 2-interacting protein OS=Homo sapiens GN=CINP PE=1 SV=1 - [CINP_HUMAN]
O14787	Transportin-2 OS=Homo sapiens GN=TNPO2 PE=1 SV=3 - [TNPO2_HUMAN]
Q9UBX1	Cathepsin F OS=Homo sapiens GN=CTSF PE=1 SV=1 - [CATF_HUMAN]
Q53EL6	Programmed cell death protein 4 OS=Homo sapiens GN=PDCD4 PE=1 SV=2 - [PDCD4_HUMAN]
P00734	Prothrombin OS=Homo sapiens GN=F2 PE=1 SV=2 - [THRB_HUMAN]
Q8NI27	THO complex subunit 2 OS=Homo sapiens GN=THOC2 PE=1 SV=2 - [THOC2_HUMAN]
Q9Y221	60S ribosome subunit biogenesis protein NIP7 homolog OS=Homo sapiens GN=NIP7 PE=1 SV=1 - [NIP7_HUMAN]
Q13033	Striatin-3 OS=Homo sapiens GN=STRN3 PE=1 SV=3 - [STRN3_HUMAN]
Q8IY67	Ribonucleoprotein PTB-binding 1 OS=Homo sapiens GN=RAVER1 PE=1 SV=1 - [RAVR1_HUMAN]
Q13895	Bystin OS=Homo sapiens GN=BYSL PE=1 SV=3 - [BYST_HUMAN]

Q86U42	Polyadenylate-binding protein 2 OS=Homo sapiens GN=PABPN1 PE=1 SV=3 - [PABP2_HUMAN]
Q9UQ16	Dynamin-3 OS=Homo sapiens GN=DNM3 PE=1 SV=4 - [DYN3_HUMAN]
Q8WUA2	Peptidyl-prolyl cis-trans isomerase-like 4 OS=Homo sapiens GN=PPIL4 PE=1 SV=1 - [PPIL4_HUMAN]
O00764	Pyridoxal kinase OS=Homo sapiens GN=PDXK PE=1 SV=1 - [PDXK_HUMAN]
O75390	Citrate synthase, mitochondrial OS=Homo sapiens GN=CS PE=1 SV=2 - [CISY_HUMAN]
Q13535	Serine/threonine-protein kinase ATR OS=Homo sapiens GN=ATR PE=1 SV=3 - [ATR_HUMAN]
P06865	Beta-hexosaminidase subunit alpha OS=Homo sapiens GN=HEXA PE=1 SV=2 - [HEXA_HUMAN]
Q9Y2X3	Nucleolar protein 58 OS=Homo sapiens GN=NOP58 PE=1 SV=1 - [NOP58_HUMAN]
O75396	Vesicle-trafficking protein SEC22b OS=Homo sapiens GN=SEC22B PE=1 SV=4 - [SC22B_HUMAN]
Q13177	Serine/threonine-protein kinase PAK 2 OS=Homo sapiens GN=PAK2 PE=1 SV=3 - [PAK2_HUMAN]
Q66LE6	Serine/threonine-protein phosphatase 2A 55 kDa regulatory subunit B delta isoform OS=Homo sapiens GN=PPP2R2D PE=2 SV=1 - [2ABD_HUMAN]
Q9H583	HEAT repeat-containing protein 1 OS=Homo sapiens GN=HEATR1 PE=1 SV=3 - [HEAT1_HUMAN]
Q96JJ3	Engulfment and cell motility protein 2 OS=Homo sapiens GN=ELMO2 PE=1 SV=2 - [ELMO2_HUMAN]
Q13636	Ras-related protein Rab-31 OS=Homo sapiens GN=RAB31 PE=1 SV=1 - [RAB31_HUMAN]
Q9NW82	WD repeat-containing protein 70 OS=Homo sapiens GN=WDR70 PE=1 SV=1 - [WDR70_HUMAN]
P06756	Integrin alpha-V OS=Homo sapiens GN=ITGAV PE=1 SV=2 - [ITAV_HUMAN]
P46379	Large proline-rich protein BAG6 OS=Homo sapiens GN=BAG6 PE=1 SV=2 - [BAG6_HUMAN]
P41229	Lysine-specific demethylase 5C OS=Homo sapiens GN=KDM5C PE=1 SV=2 - [KDM5C_HUMAN]
P52434	DNA-directed RNA polymerases I, II, and III subunit RPABC3 OS=Homo sapiens GN=POLR2H PE=1 SV=4 - [RPAB3_HUMAN]
P28300	Protein-lysine 6-oxidase OS=Homo sapiens GN=LOX PE=1 SV=2 - [LYOX_HUMAN]
Q9UNF1	Melanoma-associated antigen D2 OS=Homo sapiens GN=MAGED2 PE=1 SV=2 - [MAGD2_HUMAN]
Q9Y4W2	Ribosomal biogenesis protein LAS1L OS=Homo sapiens GN=LAS1L PE=1 SV=2 - [LAS1L_HUMAN]
Q9BTW9	Tubulin-specific chaperone D OS=Homo sapiens GN=TBCD PE=1 SV=2 - [TBCD_HUMAN]
P05186	Alkaline phosphatase, tissue-nonspecific isozyme OS=Homo sapiens GN=ALPL PE=1 SV=4 - [PPBT_HUMAN]
Q13492	Phosphatidylinositol-binding clathrin assembly protein OS=Homo sapiens GN=PICALM PE=1 SV=2 - [PICAL_HUMAN]
Q8TCZ2	CD99 antigen-like protein 2 OS=Homo sapiens GN=CD99L2 PE=1 SV=1 - [C99L2_HUMAN]
Q9Y5R8	Trafficking protein particle complex subunit 1 OS=Homo sapiens GN=TRAPPC1 PE=1 SV=1 - [TPPC1_HUMAN]
O95573	Long-chain-fatty-acid--CoA ligase 3 OS=Homo sapiens GN=ACSL3 PE=1 SV=3 - [ACSL3_HUMAN]
P31942	Heterogeneous nuclear ribonucleoprotein H3 OS=Homo sapiens GN=HNRNPH3 PE=1 SV=2 - [HNRH3_HUMAN]
P27824	Calnexin OS=Homo sapiens GN=CANX PE=1 SV=2 - [CALX_HUMAN]
Q03405	Urokinase plasminogen activator surface receptor OS=Homo sapiens GN=PLAUR PE=1 SV=1 - [UPAR_HUMAN]
O95490	Latrophilin-2 OS=Homo sapiens GN=LPHN2 PE=1 SV=2 - [LPHN2_HUMAN]
P12081	Histidine--tRNA ligase, cytoplasmic OS=Homo sapiens GN=HARS PE=1 SV=2 - [SYHC_HUMAN]
P36957	Dihydrolipoyllysine-residue succinyltransferase component of 2-oxoglutarate dehydrogenase complex, mitochondrial OS=Homo sapiens GN=DLST PE=1 SV=4 - [ODO2_HUMAN]
Q9Y570	Protein phosphatase methylesterase 1 OS=Homo sapiens GN=PPME1 PE=1 SV=3 - [PPME1_HUMAN]
O14757	Serine/threonine-protein kinase Chk1 OS=Homo sapiens GN=CHEK1 PE=1 SV=2 - [CHK1_HUMAN]
P14550	Alcohol dehydrogenase [NADP(+)] OS=Homo sapiens GN=AKR1A1 PE=1 SV=3 - [AK1A1_HUMAN]
O75251	NADH dehydrogenase [ubiquinone] iron-sulfur protein 7, mitochondrial OS=Homo sapiens GN=NDUFS7 PE=1 SV=3 - [NDUS7_HUMAN]
Q92692	Nectin-2 OS=Homo sapiens GN=PVRL2 PE=1 SV=1 - [PVRL2_HUMAN]
P15924	Desmoplakin OS=Homo sapiens GN=DSP PE=1 SV=3 - [DESP_HUMAN]
Q86UK7	Zinc finger protein 598 OS=Homo sapiens GN=ZNF598 PE=1 SV=1 - [ZN598_HUMAN]
Q8IWJ2	GRIP and coiled-coil domain-containing protein 2 OS=Homo sapiens GN=GCC2 PE=1 SV=4 - [GCC2_HUMAN]
O43414	ERI1 exoribonuclease 3 OS=Homo sapiens GN=ERI3 PE=1 SV=2 - [ERI3_HUMAN]
Q92673	Sortilin-related receptor OS=Homo sapiens GN=SORL1 PE=1 SV=2 - [SORL_HUMAN]
Q9H3U1	Protein unc-45 homolog A OS=Homo sapiens GN=UNC45A PE=1 SV=1 - [UN45A_HUMAN]
P45985	Dual specificity mitogen-activated protein kinase kinase 4 OS=Homo sapiens GN=MAP2K4 PE=1 SV=1 - [MP2K4_HUMAN]
Q9Y484	WD repeat domain phosphoinositide-interacting protein 4 OS=Homo sapiens GN=WDR45 PE=2 SV=1 - [WIPI4_HUMAN]
P21399	Cytoplasmic aconitate hydratase OS=Homo sapiens GN=ACO1 PE=1 SV=3 - [ACOC_HUMAN]
P42785	Lysosomal Pro-X carboxypeptidase OS=Homo sapiens GN=PRCP PE=1 SV=1 - [PCP_HUMAN]
Q99956	Dual specificity protein phosphatase 9 OS=Homo sapiens GN=DUSP9 PE=1 SV=1 - [DUS9_HUMAN]
Q9UPT9	Ubiquitin carboxyl-terminal hydrolase 22 OS=Homo sapiens GN=USP22 PE=1 SV=2 - [UBP22_HUMAN]
P49792	E3 SUMO-protein ligase RanBP2 OS=Homo sapiens GN=RANBP2 PE=1 SV=2 - [RBP2_HUMAN]
Q9UBQ7	Glyoxylate reductase/hydroxypyruvate reductase OS=Homo sapiens GN=GRHPR PE=1 SV=1 - [GRHPR_HUMAN]
P78406	mRNA export factor OS=Homo sapiens GN=RAE1 PE=1 SV=1 - [RAE1L_HUMAN]
O95295	SNARE-associated protein Snapin OS=Homo sapiens GN=SNAPIN PE=1 SV=1 - [SNAPN_HUMAN]
P12429	Annexin A3 OS=Homo sapiens GN=ANXA3 PE=1 SV=3 - [ANXA3_HUMAN]
Q96DV4	39S ribosomal protein L38, mitochondrial OS=Homo sapiens GN=MRPL38 PE=1 SV=2 - [RM38_HUMAN]
O60508	Pre-mRNA-processing factor 17 OS=Homo sapiens GN=CDC40 PE=1 SV=1 - [PRP17_HUMAN]
O75347	Tubulin-specific chaperone A OS=Homo sapiens GN=TBCA PE=1 SV=3 - [TBCA_HUMAN]
Q9BS18	Anaphase-promoting complex subunit 13 OS=Homo sapiens GN=ANAPC13 PE=1 SV=1 - [APC13_HUMAN]
Q9GZZ9	Ubiquitin-like modifier-activating enzyme 5 OS=Homo sapiens GN=UBA5 PE=1 SV=1 - [UBA5_HUMAN]
P01130	Low-density lipoprotein receptor OS=Homo sapiens GN=LDLR PE=1 SV=1 - [LDLR_HUMAN]
Q8NEM2	SHC SH2 domain-binding protein 1 OS=Homo sapiens GN=SHCBP1 PE=1 SV=3 - [SHCBP_HUMAN]
Q86X10	Ral GTPase-activating protein subunit beta OS=Homo sapiens GN=RALGAPB PE=1 SV=1 - [RLGPB_HUMAN]
Q96G25	Mediator of RNA polymerase II transcription subunit 8 OS=Homo sapiens GN=MED8 PE=1 SV=2 - [MED8_HUMAN]
Q14168	MAGUK p55 subfamily member 2 OS=Homo sapiens GN=MPP2 PE=1 SV=3 - [MPP2_HUMAN]
P57721	Poly(rC)-binding protein 3 OS=Homo sapiens GN=PCBP3 PE=2 SV=2 - [PCBP3_HUMAN]
Q6NXR4	TELO2-interacting protein 2 OS=Homo sapiens GN=TTI2 PE=1 SV=1 - [TTI2_HUMAN]
Q96RF0	Sorting nexin-18 OS=Homo sapiens GN=SNX18 PE=1 SV=2 - [SNX18_HUMAN]
O75419	Cell division control protein 45 homolog OS=Homo sapiens GN=CDC45 PE=1 SV=1 - [CDC45_HUMAN]
P42695	Condensin-2 complex subunit D3 OS=Homo sapiens GN=NCAPD3 PE=1 SV=2 - [CNDD3_HUMAN]
Q86UV5	Ubiquitin carboxyl-terminal hydrolase 48 OS=Homo sapiens GN=USP48 PE=1 SV=1 - [UBP48_HUMAN]
Q8IXW5	Putative RNA polymerase II subunit B1 CTD phosphatase RPAP2 OS=Homo sapiens GN=RPAP2 PE=1 SV=1 - [RPAP2_HUMAN]
P36955	Pigment epithelium-derived factor OS=Homo sapiens GN=SERPINF1 PE=1 SV=4 - [PEDF_HUMAN]
Q13395	Probable methyltransferase TARBP1 OS=Homo sapiens GN=TARBP1 PE=1 SV=1 - [TARB1_HUMAN]
Q8TAQ2	SWI/SNF complex subunit SMARCC2 OS=Homo sapiens GN=SMARCC2 PE=1 SV=1 - [SMRC2_HUMAN]
P16949	Stathmin OS=Homo sapiens GN=STMN1 PE=1 SV=3 - [STMN1_HUMAN]
Q9NXF1	Testis-expressed sequence 10 protein OS=Homo sapiens GN=TEX10 PE=1 SV=2 - [TEX10_HUMAN]
Q9Y662	Heparan sulfate glucosamine 3-O-sulfotransferase 3B1 OS=Homo sapiens GN=HS3ST3B1 PE=1 SV=1 - [HS3SB_HUMAN]
Q8WWY3	U4/U6 small nuclear ribonucleoprotein Prp31 OS=Homo sapiens GN=PRPF31 PE=1 SV=2 - [PRP31_HUMAN]
O00139	Kinesin-like protein KIF2A OS=Homo sapiens GN=KIF2A PE=1 SV=3 - [KIF2A_HUMAN]
Q92562	Polyphosphoinositide phosphatase OS=Homo sapiens GN=FIG4 PE=1 SV=1 - [FIG4_HUMAN]
Q12797	Aspartyl/asparaginyl beta-hydroxylase OS=Homo sapiens GN=ASPH PE=1 SV=3 - [ASPH_HUMAN]
P40763	Signal transducer and activator of transcription 3 OS=Homo sapiens GN=STAT3 PE=1 SV=2 - [STAT3_HUMAN]
Q12884	Prolyl endopeptidase FAP OS=Homo sapiens GN=FAP PE=1 SV=5 - [SEPR_HUMAN]
P19174	1-phosphatidylinositol 4,5-bisphosphate phosphodiesterase gamma-1 OS=Homo sapiens GN=PLCG1 PE=1 SV=1 - [PLCG1_HUMAN]
Q9P2K8	Eukaryotic translation initiation factor 2-alpha kinase 4 OS=Homo sapiens GN=EIF2AK4 PE=1 SV=3 - [E2AK4_HUMAN]
Q96BJ3	Axin interactor, dorsalization-associated protein OS=Homo sapiens GN=AIDA PE=1 SV=1 - [AIDA_HUMAN]
Q5R372	Rab GTPase-activating protein 1-like OS=Homo sapiens GN=RABGAP1L PE=1 SV=1 - [RBG1L_HUMAN]
Q9UNS1	Protein timeless homolog OS=Homo sapiens GN=TIMELESS PE=1 SV=2 - [TIM_HUMAN]
P49902	Cytosolic purine 5'-nucleotidase OS=Homo sapiens GN=NT5C2 PE=1 SV=1 - [5NTC_HUMAN]
P09104	Gamma-enolase OS=Homo sapiens GN=ENO2 PE=1 SV=3 - [ENOG_HUMAN]
P13929	Beta-enolase OS=Homo sapiens GN=ENO3 PE=1 SV=5 - [ENOB_HUMAN]
Q15397	Pumilio domain-containing protein KIAA0020 OS=Homo sapiens GN=KIAA0020 PE=1 SV=3 - [K0020_HUMAN]
Q8IZ69	tRNA (uracil-5-)-methyltransferase homolog A OS=Homo sapiens GN=TRMT2A PE=1 SV=2 - [TRM2A_HUMAN]
Q13576	Ras GTPase-activating-like protein IQGAP2 OS=Homo sapiens GN=IQGAP2 PE=1 SV=4 - [IQGA2_HUMAN]
P23229	Integrin alpha-6 OS=Homo sapiens GN=ITGA6 PE=1 SV=5 - [ITA6_HUMAN]
Q8N441	Fibroblast growth factor receptor-like 1 OS=Homo sapiens GN=FGFRL1 PE=1 SV=1 - [FGRL1_HUMAN]
Q9NQ29	Putative RNA-binding protein Luc7-like 1 OS=Homo sapiens GN=LUC7L PE=1 SV=1 - [LUC7L_HUMAN]
Q5VVQ6	Ubiquitin thioesterase OTU1 OS=Homo sapiens GN=YOD1 PE=1 SV=1 - [OTU1_HUMAN]
Q6L8Q7	2',5'-phosphodiesterase 12 OS=Homo sapiens GN=PDE12 PE=1 SV=2 - [PDE12_HUMAN]
Q9BW27	Nuclear pore complex protein Nup85 OS=Homo sapiens GN=NUP85 PE=1 SV=1 - [NUP85_HUMAN]
P26583	High mobility group protein B2 OS=Homo sapiens GN=HMGB2 PE=1 SV=2 - [HMGB2_HUMAN]
P24347	Stromelysin-3 OS=Homo sapiens GN=MMP11 PE=1 SV=3 - [MMP11_HUMAN]
Q16543	Hsp90 co-chaperone Cdc37 OS=Homo sapiens GN=CDC37 PE=1 SV=1 - [CDC37_HUMAN]
O00291	Huntingtin-interacting protein 1 OS=Homo sapiens GN=HIP1 PE=1 SV=5 - [HIP1_HUMAN]
P33908	Mannosyl-oligosaccharide 1,2-alpha-mannosidase IA OS=Homo sapiens GN=MAN1A1 PE=1 SV=3 - [MA1A1_HUMAN]
Q9NRY4	Rho GTPase-activating protein 35 OS=Homo sapiens GN=ARHGAP35 PE=1 SV=3 - [RHG35_HUMAN]
Q13107	Ubiquitin carboxyl-terminal hydrolase 4 OS=Homo sapiens GN=USP4 PE=1 SV=3 - [UBP4_HUMAN]
Q8WWM7	Ataxin-2-like protein OS=Homo sapiens GN=ATXN2L PE=1 SV=2 - [ATX2L_HUMAN]
Q96ST3	Paired amphipathic helix protein Sin3a OS=Homo sapiens GN=SIN3A PE=1 SV=2 - [SIN3A_HUMAN]
O95819	Mitogen-activated protein kinase kinase kinase kinase 4 OS=Homo sapiens GN=MAP4K4 PE=1 SV=2 - [M4K4_HUMAN]
Q86SZ2	Trafficking protein particle complex subunit 6B OS=Homo sapiens GN=TRAPPC6B PE=1 SV=1 - [TPC6B_HUMAN]
Q9NZL9	Methionine adenosyltransferase 2 subunit beta OS=Homo sapiens GN=MAT2B PE=1 SV=1 - [MAT2B_HUMAN]
Q9HCP0	Casein kinase I isoform gamma-1 OS=Homo sapiens GN=CSNK1G1 PE=1 SV=1 - [KC1G1_HUMAN]

Q96SB8	Structural maintenance of chromosomes protein 6 OS=Homo sapiens GN=SMC6 PE=1 SV=2 - [SMC6_HUMAN]
Q92889	DNA repair endonuclease XPF OS=Homo sapiens GN=ERCC4 PE=1 SV=3 - [XPF_HUMAN]
Q9NXH9	tRNA (guanine(26)-N(2))-dimethyltransferase OS=Homo sapiens GN=TRMT1 PE=1 SV=1 - [TRM1_HUMAN]
Q9UNH7	Sorting nexin-6 OS=Homo sapiens GN=SNX6 PE=1 SV=1 - [SNX6_HUMAN]
Q8WVM8	Sec1 family domain-containing protein 1 OS=Homo sapiens GN=SCFD1 PE=1 SV=4 - [SCFD1_HUMAN]
P15374	Ubiquitin carboxyl-terminal hydrolase isozyme L3 OS=Homo sapiens GN=UCHL3 PE=1 SV=1 - [UCHL3_HUMAN]
Q8N5M4	Tetratricopeptide repeat protein 9C OS=Homo sapiens GN=TTC9C PE=1 SV=1 - [TTC9C_HUMAN]
Q9UPN3	Microtubule-actin cross-linking factor 1, isoforms 1/2/3/5 OS=Homo sapiens GN=MACF1 PE=1 SV=4 - [MACF1_HUMAN]
Q8TAG9	Exocyst complex component 6 OS=Homo sapiens GN=EXOC6 PE=1 SV=3 - [EXOC6_HUMAN]
Q9H4A4	Aminopeptidase B OS=Homo sapiens GN=RNPEP PE=1 SV=2 - [AMPB_HUMAN]
O60231	Putative pre-mRNA-splicing factor ATP-dependent RNA helicase DHX16 OS=Homo sapiens GN=DHX16 PE=1 SV=2 - [DHX16_HUMAN]
P40939	Trifunctional enzyme subunit alpha, mitochondrial OS=Homo sapiens GN=HADHA PE=1 SV=2 - [ECHA_HUMAN]
Q8IZL8	Proline-, glutamic acid- and leucine-rich protein 1 OS=Homo sapiens GN=PELP1 PE=1 SV=2 - [PELP1_HUMAN]
Q9NWS0	PIH1 domain-containing protein 1 OS=Homo sapiens GN=PIH1D1 PE=1 SV=1 - [PIHD1_HUMAN]
Q99547	M-phase phosphoprotein 6 OS=Homo sapiens GN=MPHOSPH6 PE=1 SV=2 - [MPH6_HUMAN]
Q9UHR6	Zinc finger HIT domain-containing protein 2 OS=Homo sapiens GN=ZNHIT2 PE=1 SV=1 - [ZNHI2_HUMAN]
Q53SF7	Cordon-bleu protein-like 1 OS=Homo sapiens GN=COBLL1 PE=1 SV=2 - [COBL1_HUMAN]
Q5VTL8	Pre-mRNA-splicing factor 38B OS=Homo sapiens GN=PRPF38B PE=1 SV=1 - [PR38B_HUMAN]
P30085	UMP-CMP kinase OS=Homo sapiens GN=CMPK1 PE=1 SV=3 - [KCY_HUMAN]
P62745	Rho-related GTP-binding protein RhoB OS=Homo sapiens GN=RHOB PE=1 SV=1 - [RHOB_HUMAN]
Q9Y316	Protein MEMO1 OS=Homo sapiens GN=MEMO1 PE=1 SV=1 - [MEMO1_HUMAN]
Q6PCB8	Embigin OS=Homo sapiens GN=EMB PE=1 SV=1 - [EMB_HUMAN]
O15042	U2 snRNP-associated SURP motif-containing protein OS=Homo sapiens GN=U2SURP PE=1 SV=2 - [SR140_HUMAN]
Q07065	Cytoskeleton-associated protein 4 OS=Homo sapiens GN=CKAP4 PE=1 SV=2 - [CKAP4_HUMAN]
Q9UPN9	E3 ubiquitin-protein ligase TRIM33 OS=Homo sapiens GN=TRIM33 PE=1 SV=3 - [TRI33_HUMAN]
Q969X6	Cirhin OS=Homo sapiens GN=CIRH1A PE=1 SV=1 - [CIR1A_HUMAN]
Q9BVP2	Guanine nucleotide-binding protein-like 3 OS=Homo sapiens GN=GNL3 PE=1 SV=2 - [GNL3_HUMAN]
Q9NYB9	Abl interactor 2 OS=Homo sapiens GN=ABI2 PE=1 SV=1 - [ABI2_HUMAN]
O60784	Target of Myb protein 1 OS=Homo sapiens GN=TOM1 PE=1 SV=2 - [TOM1_HUMAN]
P09471	Guanine nucleotide-binding protein G(o) subunit alpha OS=Homo sapiens GN=GNAO1 PE=1 SV=4 - [GNAO_HUMAN]
P49257	Protein ERGIC-53 OS=Homo sapiens GN=LMAN1 PE=1 SV=2 - [LMAN1_HUMAN]
Q02880	DNA topoisomerase 2-beta OS=Homo sapiens GN=TOP2B PE=1 SV=3 - [TOP2B_HUMAN]
Q8NBS9	Thioredoxin domain-containing protein 5 OS=Homo sapiens GN=TXNDC5 PE=1 SV=2 - [TXND5_HUMAN]
Q8WTV0	Scavenger receptor class B member 1 OS=Homo sapiens GN=SCARB1 PE=1 SV=1 - [SCRB1_HUMAN]
Q96SI9	Spermatid perinuclear RNA-binding protein OS=Homo sapiens GN=STRBP PE=1 SV=1 - [STRBP_HUMAN]
Q9NPI6	mRNA-decapping enzyme 1A OS=Homo sapiens GN=DCP1A PE=1 SV=2 - [DCP1A_HUMAN]
Q9NR31	GTP-binding protein SAR1a OS=Homo sapiens GN=SAR1A PE=1 SV=1 - [SAR1A_HUMAN]
Q7Z2T5	TRMT1-like protein OS=Homo sapiens GN=TRMT1L PE=1 SV=2 - [TRM1L_HUMAN]
Q8NB46	Serine/threonine-protein phosphatase 6 regulatory ankyrin repeat subunit C OS=Homo sapiens GN=ANKRD52 PE=1 SV=3 - [ANR52_HUMAN]
P00387	NADH-cytochrome b5 reductase 3 OS=Homo sapiens GN=CYB5R3 PE=1 SV=3 - [NB5R3_HUMAN]
Q9H0C8	Integrin-linked kinase-associated serine/threonine phosphatase 2C OS=Homo sapiens GN=ILKAP PE=1 SV=1 - [ILKAP_HUMAN]
O95248	Myotubularin-related protein 5 OS=Homo sapiens GN=SBF1 PE=1 SV=3 - [MTMR5_HUMAN]
Q7Z6J9	tRNA-splicing endonuclease subunit Sen54 OS=Homo sapiens GN=TSEN54 PE=1 SV=3 - [SEN54_HUMAN]
Q8IZP0	Abl interactor 1 OS=Homo sapiens GN=ABI1 PE=1 SV=4 - [ABI1_HUMAN]
Q99719	Septin-5 OS=Homo sapiens GN=SEPT5 PE=1 SV=1 - [SEPT5_HUMAN]
Q9P2J3	Kelch-like protein 9 OS=Homo sapiens GN=KLHL9 PE=1 SV=2 - [KLHL9_HUMAN]
P35520	Cystathionine beta-synthase OS=Homo sapiens GN=CBS PE=1 SV=2 - [CBS_HUMAN]
P52799	Ephrin-B2 OS=Homo sapiens GN=EFNB2 PE=1 SV=1 - [EFNB2_HUMAN]
Q5JU69	Torsin-2A OS=Homo sapiens GN=TOR2A PE=2 SV=1 - [TOR2A_HUMAN]
O14545	TRAF-type zinc finger domain-containing protein 1 OS=Homo sapiens GN=TRAFD1 PE=1 SV=1 - [TRAD1_HUMAN]
Q07666	KH domain-containing, RNA-binding, signal transduction-associated protein 1 OS=Homo sapiens GN=KHDRBS1 PE=1 SV=1 - [KHDR1_HUMAN]
Q8IWX8	Calcium homeostasis endoplasmic reticulum protein OS=Homo sapiens GN=CHERP PE=1 SV=3 - [CHERP_HUMAN]
P31749	RAC-alpha serine/threonine-protein kinase OS=Homo sapiens GN=AKT1 PE=1 SV=2 - [AKT1_HUMAN]
Q9NV70	Exocyst complex component 1 OS=Homo sapiens GN=EXOC1 PE=1 SV=4 - [EXOC1_HUMAN]
O75044	SLIT-ROBO Rho GTPase-activating protein 2 OS=Homo sapiens GN=SRGAP2 PE=1 SV=2 - [SRGP2_HUMAN]
Q9NS87	Kinesin-like protein KIF15 OS=Homo sapiens GN=KIF15 PE=1 SV=1 - [KIF15_HUMAN]
Q9UL33	Trafficking protein particle complex subunit 2-like protein OS=Homo sapiens GN=TRAPPC2L PE=1 SV=1 - [TPC2L_HUMAN]
Q13618	Cullin-3 OS=Homo sapiens GN=CUL3 PE=1 SV=2 - [CUL3_HUMAN]
Q9GZN1	Actin-related protein 6 OS=Homo sapiens GN=ACTR6 PE=1 SV=1 - [ARP6_HUMAN]
P08648	Integrin alpha-5 OS=Homo sapiens GN=ITGA5 PE=1 SV=2 - [ITA5_HUMAN]
Q9BYG0	Lactosylceramide 1,3-N-acetyl-beta-D-glucosaminyltransferase OS=Homo sapiens GN=B3GNT5 PE=1 SV=1 - [B3GN5_HUMAN]
P49354	Protein farnesyltransferase/geranylgeranyltransferase type-1 subunit alpha OS=Homo sapiens GN=FNTA PE=1 SV=1 - [FNTA_HUMAN]
O43149	Zinc finger ZZ-type and EF-hand domain-containing protein 1 OS=Homo sapiens GN=ZZEF1 PE=1 SV=6 - [ZZEF1_HUMAN]
Q9UM82	Spermatogenesis-associated protein 2 OS=Homo sapiens GN=SPATA2 PE=1 SV=2 - [SPAT2_HUMAN]
A6NDU8	UPF0600 protein C5orf51 OS=Homo sapiens GN=C5orf51 PE=1 SV=1 - [CE051_HUMAN]
Q9UL15	BAG family molecular chaperone regulator 5 OS=Homo sapiens GN=BAG5 PE=1 SV=1 - [BAG5_HUMAN]
Q96EP0	E3 ubiquitin-protein ligase RNF31 OS=Homo sapiens GN=RNF31 PE=1 SV=1 - [RNF31_HUMAN]
Q07021	Complement component 1 Q subcomponent-binding protein, mitochondrial OS=Homo sapiens GN=C1QBP PE=1 SV=1 - [C1QBP_HUMAN]
P15586	N-acetylglucosamine-6-sulfatase OS=Homo sapiens GN=GNS PE=1 SV=3 - [GNS_HUMAN]
P50851	Lipopolysaccharide-responsive and beige-like anchor protein OS=Homo sapiens GN=LRBA PE=1 SV=4 - [LRBA_HUMAN]
Q05655	Protein kinase C delta type OS=Homo sapiens GN=PRKCD PE=1 SV=2 - [KPCD_HUMAN]
Q9Y6W5	Wiskott-Aldrich syndrome protein family member 2 OS=Homo sapiens GN=WASF2 PE=1 SV=3 - [WASF2_HUMAN]
Q86UF1	Tetraspanin-33 OS=Homo sapiens GN=TSPAN33 PE=2 SV=1 - [TSN33_HUMAN]
P10515	Dihydrolipoyllysine-residue acetyltransferase component of pyruvate dehydrogenase complex, mitochondrial OS=Homo sapiens GN=DLAT PE=1 SV=3 - [ODP2_HUMAN]
P30520	Adenylosuccinate synthetase isozyme 2 OS=Homo sapiens GN=ADSS PE=1 SV=3 - [PURA2_HUMAN]
Q68EM7	Rho GTPase-activating protein 17 OS=Homo sapiens GN=ARHGAP17 PE=1 SV=1 - [RHG17_HUMAN]
Q96P48	Arf-GAP with Rho-GAP domain, ANK repeat and PH domain-containing protein 1 OS=Homo sapiens GN=ARAP1 PE=1 SV=3 - [ARAP1_HUMAN]
Q8WU90	Zinc finger CCCH domain-containing protein 15 OS=Homo sapiens GN=ZC3H15 PE=1 SV=1 - [ZC3HF_HUMAN]
Q14147	Probable ATP-dependent RNA helicase DHX34 OS=Homo sapiens GN=DHX34 PE=1 SV=2 - [DHX34_HUMAN]
Q9P2N7	Kelch-like protein 13 OS=Homo sapiens GN=KLHL13 PE=1 SV=3 - [KLH13_HUMAN]
Q14703	Membrane-bound transcription factor site-1 protease OS=Homo sapiens GN=MBTPS1 PE=1 SV=1 - [MBTP1_HUMAN]
P43250	G protein-coupled receptor kinase 6 OS=Homo sapiens GN=GRK6 PE=1 SV=2 - [GRK6_HUMAN]
O95985	DNA topoisomerase 3-beta-1 OS=Homo sapiens GN=TOP3B PE=1 SV=1 - [TOP3B_HUMAN]
O75096	Low-density lipoprotein receptor-related protein 4 OS=Homo sapiens GN=LRP4 PE=1 SV=4 - [LRP4_HUMAN]
P29372	DNA-3-methyladenine glycosylase OS=Homo sapiens GN=MPG PE=1 SV=3 - [3MG_HUMAN]
Q8NEL9	Phospholipase DDHD1 OS=Homo sapiens GN=DDHD1 PE=1 SV=2 - [DDHD1_HUMAN]
Q8IU81	Interferon regulatory factor 2-binding protein 1 OS=Homo sapiens GN=IRF2BP1 PE=1 SV=1 - [I2BP1_HUMAN]
Q92558	Wiskott-Aldrich syndrome protein family member 1 OS=Homo sapiens GN=WASF1 PE=1 SV=1 - [WASF1_HUMAN]
Q96CW5	Gamma-tubulin complex component 3 OS=Homo sapiens GN=TUBGCP3 PE=1 SV=2 - [GCP3_HUMAN]
P02649	Apolipoprotein E OS=Homo sapiens GN=APOE PE=1 SV=1 - [APOE_HUMAN]
Q02487	Desmocollin-2 OS=Homo sapiens GN=DSC2 PE=1 SV=1 - [DSC2_HUMAN]
O15037	Protein KHNYN OS=Homo sapiens GN=KHNYN PE=1 SV=3 - [KHNYN_HUMAN]
Q9UKZ1	CCR4-NOT transcription complex subunit 11 OS=Homo sapiens GN=CNOT11 PE=1 SV=1 - [CNO11_HUMAN]
Q00688	Peptidyl-prolyl cis-trans isomerase FKBP3 OS=Homo sapiens GN=FKBP3 PE=1 SV=1 - [FKBP3_HUMAN]
P31483	Nucleolysin TIA-1 isoform p40 OS=Homo sapiens GN=TIA1 PE=1 SV=3 - [TIA1_HUMAN]
Q15427	Splicing factor 3B subunit 4 OS=Homo sapiens GN=SF3B4 PE=1 SV=1 - [SF3B4_HUMAN]
Q9Y237	Peptidyl-prolyl cis-trans isomerase NIMA-interacting 4 OS=Homo sapiens GN=PIN4 PE=1 SV=1 - [PIN4_HUMAN]
Q96QD8	Sodium-coupled neutral amino acid transporter 2 OS=Homo sapiens GN=SLC38A2 PE=1 SV=2 - [S38A2_HUMAN]
P24043	Laminin subunit alpha-2 OS=Homo sapiens GN=LAMA2 PE=1 SV=4 - [LAMA2_HUMAN]
Q15022	Polycomb protein SUZ12 OS=Homo sapiens GN=SUZ12 PE=1 SV=3 - [SUZ12_HUMAN]
Q14393	Growth arrest-specific protein 6 OS=Homo sapiens GN=GAS6 PE=1 SV=2 - [GAS6_HUMAN]
Q9NZW5	MAGUK p55 subfamily member 6 OS=Homo sapiens GN=MPP6 PE=1 SV=2 - [MPP6_HUMAN]
P19367	Hexokinase-1 OS=Homo sapiens GN=HK1 PE=1 SV=3 - [HXK1_HUMAN]
P14314	Glucosidase 2 subunit beta OS=Homo sapiens GN=PRKCSH PE=1 SV=2 - [GLU2B_HUMAN]
P37198	Nuclear pore glycoprotein p62 OS=Homo sapiens GN=NUP62 PE=1 SV=3 - [NUP62_HUMAN]
Q96RU3	Formin-binding protein 1 OS=Homo sapiens GN=FNBP1 PE=1 SV=2 - [FNBP1_HUMAN]
P11171	Protein 4.1 OS=Homo sapiens GN=EPB41 PE=1 SV=4 - [41_HUMAN]
Q12846	Syntaxin-4 OS=Homo sapiens GN=STX4 PE=1 SV=2 - [STX4_HUMAN]
Q15582	Transforming growth factor-beta-induced protein ig-h3 OS=Homo sapiens GN=TGFBI PE=1 SV=1 - [BGH3_HUMAN]
Q6ZUX7	Lipoma HMGIC fusion partner-like 2 protein OS=Homo sapiens GN=LHFPL2 PE=2 SV=2 - [LHPL2_HUMAN]
P35637	RNA-binding protein FUS OS=Homo sapiens GN=FUS PE=1 SV=1 - [FUS_HUMAN]
Q9BYD1	39S ribosomal protein L13, mitochondrial OS=Homo sapiens GN=MRPL13 PE=1 SV=1 - [RM13_HUMAN]
Q969E2	Secretory carrier-associated membrane protein 4 OS=Homo sapiens GN=SCAMP4 PE=2 SV=1 - [SCAM4_HUMAN]
Q96HN2	Putative adenosylhomocysteinase 3 OS=Homo sapiens GN=AHCYL2 PE=1 SV=1 - [SAHH3_HUMAN]
O75955	Flotillin-1 OS=Homo sapiens GN=FLOT1 PE=1 SV=3 - [FLOT1_HUMAN]

P25098	Beta-adrenergic receptor kinase 1 OS=Homo sapiens GN=ADRBK1 PE=1 SV=2 - [ARBK1_HUMAN]
Q15650	Activating signal cointegrator 1 OS=Homo sapiens GN=TRIP4 PE=1 SV=4 - [TRIP4_HUMAN]
Q96AG4	Leucine-rich repeat-containing protein 59 OS=Homo sapiens GN=LRRC59 PE=1 SV=1 - [LRC59_HUMAN]
Q12931	Heat shock protein 75 kDa, mitochondrial OS=Homo sapiens GN=TRAP1 PE=1 SV=3 - [TRAP1_HUMAN]
Q8N6R0	Methyltransferase-like protein 13 OS=Homo sapiens GN=METTL13 PE=1 SV=1 - [MET13_HUMAN]
P42892	Endothelin-converting enzyme 1 OS=Homo sapiens GN=ECE1 PE=1 SV=2 - [ECE1_HUMAN]
Q92759	General transcription factor IIH subunit 4 OS=Homo sapiens GN=GTF2H4 PE=2 SV=1 - [TF2H4_HUMAN]
P50213	Isocitrate dehydrogenase [NAD] subunit alpha, mitochondrial OS=Homo sapiens GN=IDH3A PE=1 SV=1 - [IDH3A_HUMAN]
O95219	Sorting nexin-4 OS=Homo sapiens GN=SNX4 PE=1 SV=1 - [SNX4_HUMAN]
P49184	Deoxyribonuclease-1-like 1 OS=Homo sapiens GN=DNASE1L1 PE=1 SV=1 - [DNSL1_HUMAN]
Q9BSL1	Ubiquitin-associated domain-containing protein 1 OS=Homo sapiens GN=UBAC1 PE=1 SV=1 - [UBAC1_HUMAN]
Q9BUJ2	Heterogeneous nuclear ribonucleoprotein U-like protein 1 OS=Homo sapiens GN=HNRNPUL1 PE=1 SV=2 - [HNRL1_HUMAN]
Q9P015	39S ribosomal protein L15, mitochondrial OS=Homo sapiens GN=MRPL15 PE=1 SV=1 - [RM15_HUMAN]
P20908	Collagen alpha-1(V) chain OS=Homo sapiens GN=COL5A1 PE=1 SV=3 - [CO5A1_HUMAN]
Q9UBV8	Peflin OS=Homo sapiens GN=PEF1 PE=1 SV=1 - [PEF1_HUMAN]
Q9H3S7	Tyrosine-protein phosphatase non-receptor type 23 OS=Homo sapiens GN=PTPN23 PE=1 SV=1 - [PTN23_HUMAN]
Q9Y6J9	TAF6-like RNA polymerase II p300/CBP-associated factor-associated factor 65 kDa subunit 6L OS=Homo sapiens GN=TAF6L PE=1 SV=1 - [TAF6L_HUMAN]
Q9BXP5	Serrate RNA effector molecule homolog OS=Homo sapiens GN=SRRT PE=1 SV=1 - [SRRT_HUMAN]
Q9UHN6	Transmembrane protein 2 OS=Homo sapiens GN=TMEM2 PE=1 SV=1 - [TMEM2_HUMAN]
O95487	Protein transport protein Sec24B OS=Homo sapiens GN=SEC24B PE=1 SV=2 - [SC24B_HUMAN]
Q13112	Chromatin assembly factor 1 subunit B OS=Homo sapiens GN=CHAF1B PE=1 SV=1 - [CAF1B_HUMAN]
Q9Y4C1	Lysine-specific demethylase 3A OS=Homo sapiens GN=KDM3A PE=1 SV=4 - [KDM3A_HUMAN]
Q9H0H5	Rac GTPase-activating protein 1 OS=Homo sapiens GN=RACGAP1 PE=1 SV=1 - [RGAP1_HUMAN]
Q9Y6G5	COMM domain-containing protein 10 OS=Homo sapiens GN=COMMD10 PE=1 SV=1 - [COMDA_HUMAN]
O60243	Heparan-sulfate 6-O-sulfotransferase 1 OS=Homo sapiens GN=HS6ST1 PE=1 SV=5 - [H6ST1_HUMAN]
Q12756	Kinesin-like protein KIF1A OS=Homo sapiens GN=KIF1A PE=1 SV=2 - [KIF1A_HUMAN]
P19838	Nuclear factor NF-kappa-B p105 subunit OS=Homo sapiens GN=NFKB1 PE=1 SV=2 - [NFKB1_HUMAN]
Q96GM8	Target of EGR1 protein 1 OS=Homo sapiens GN=TOE1 PE=1 SV=1 - [TOE1_HUMAN]
Q9UBK9	Protein UXT OS=Homo sapiens GN=UXT PE=1 SV=1 - [UXT_HUMAN]
Q99829	Copine-1 OS=Homo sapiens GN=CPNE1 PE=1 SV=1 - [CPNE1_HUMAN]
Q9UER7	Death domain-associated protein 6 OS=Homo sapiens GN=DAXX PE=1 SV=2 - [DAXX_HUMAN]
Q06323	Proteasome activator complex subunit 1 OS=Homo sapiens GN=PSME1 PE=1 SV=1 - [PSME1_HUMAN]
Q86U38	Nucleolar protein 9 OS=Homo sapiens GN=NOP9 PE=1 SV=1 - [NOP9_HUMAN]
Q9HAU5	Regulator of nonsense transcripts 2 OS=Homo sapiens GN=UPF2 PE=1 SV=1 - [RENT2_HUMAN]
Q9BSJ8	Extended synaptotagmin-1 OS=Homo sapiens GN=ESYT1 PE=1 SV=1 - [ESYT1_HUMAN]
P00533	Epidermal growth factor receptor OS=Homo sapiens GN=EGFR PE=1 SV=2 - [EGFR_HUMAN]
P48147	Prolyl endopeptidase OS=Homo sapiens GN=PREP PE=1 SV=2 - [PPCE HUMAN]
O95025	Semaphorin-3D OS=Homo sapiens GN=SEMA3D PE=2 SV=2 - [SEM3D_HUMAN]
P24863	Cyclin-C OS=Homo sapiens GN=CCNC PE=1 SV=2 - [CCNC_HUMAN]
Q9BXX0	EMILIN-2 OS=Homo sapiens GN=EMILIN2 PE=1 SV=3 - [EMIL2_HUMAN]
Q15833	Syntaxin-binding protein 2 OS=Homo sapiens GN=STXBP2 PE=1 SV=2 - [STXB2_HUMAN]
O75448	Mediator of RNA polymerase II transcription subunit 24 OS=Homo sapiens GN=MED24 PE=1 SV=1 - [MED24_HUMAN]
Q99661	Kinesin-like protein KIF2C OS=Homo sapiens GN=KIF2C PE=1 SV=2 - [KIF2C_HUMAN]
Q9HB07	UPF0160 protein MYG1, mitochondrial OS=Homo sapiens GN=C12orf10 PE=1 SV=2 - [MYG1_HUMAN]
P54725	UV excision repair protein RAD23 homolog A OS=Homo sapiens GN=RAD23A PE=1 SV=1 - [RD23A_HUMAN]
Q86TU7	Histone-lysine N-methyltransferase setd3 OS=Homo sapiens GN=SETD3 PE=1 SV=1 - [SETD3_HUMAN]
Q9C0C2	182 kDa tankyrase-1-binding protein OS=Homo sapiens GN=TNKS1BP1 PE=1 SV=4 - [TB182_HUMAN]
O95671	N-acetylserotonin O-methyltransferase-like protein OS=Homo sapiens GN=ASMTL PE=1 SV=3 - [ASML_HUMAN]
Q92614	Unconventional myosin-XVIIIa OS=Homo sapiens GN=MYO18A PE=1 SV=3 - [MY18A_HUMAN]
P39656	Dolichyl-diphosphooligosaccharide--protein glycosyltransferase 48 kDa subunit OS=Homo sapiens GN=DDOST PE=1 SV=4 - [OST48_HUMAN]
Q13596	Sorting nexin-1 OS=Homo sapiens GN=SNX1 PE=1 SV=3 - [SNX1_HUMAN]
Q96T88	E3 ubiquitin-protein ligase UHRF1 OS=Homo sapiens GN=UHRF1 PE=1 SV=1 - [UHRF1_HUMAN]
O95429	BAG family molecular chaperone regulator 4 OS=Homo sapiens GN=BAG4 PE=1 SV=1 - [BAG4_HUMAN]
Q9NVQ4	Fas apoptotic inhibitory molecule 1 OS=Homo sapiens GN=FAIM PE=1 SV=1 - [FAIM1_HUMAN]
Q8N684	Cleavage and polyadenylation specificity factor subunit 7 OS=Homo sapiens GN=CPSF7 PE=1 SV=1 - [CPSF7_HUMAN]
Q16630	Cleavage and polyadenylation specificity factor subunit 6 OS=Homo sapiens GN=CPSF6 PE=1 SV=2 - [CPSF6_HUMAN]
Q92989	Polyribonucleotide 5'-hydroxyl-kinase Clp1 OS=Homo sapiens GN=CLP1 PE=1 SV=1 - [CLP1_HUMAN]
Q7L1Q6	Basic leucine zipper and W2 domain-containing protein 1 OS=Homo sapiens GN=BZW1 PE=1 SV=1 - [BZW1 HUMAN]
Q86VS8	Protein Hook homolog 3 OS=Homo sapiens GN=HOOK3 PE=1 SV=2 - [HOOK3_HUMAN]
Q9Y6E2	Basic leucine zipper and W2 domain-containing protein 2 OS=Homo sapiens GN=BZW2 PE=1 SV=1 - [BZW2 HUMAN]
Q29865	HLA class I histocompatibility antigen, Cw-18 alpha chain OS=Homo sapiens GN=HLA-C PE=1 SV=1 - [1C18_HUMAN]
P55196	Afadin OS=Homo sapiens GN=MLLT4 PE=1 SV=3 - [AFAD_HUMAN]
Q9NRF8	CTP synthase 2 OS=Homo sapiens GN=CTPS2 PE=1 SV=1 - [PYRG2_HUMAN]
Q15910	Histone-lysine N-methyltransferase EZH2 OS=Homo sapiens GN=EZH2 PE=1 SV=2 - [EZH2_HUMAN]
P39748	Flap endonuclease 1 OS=Homo sapiens GN=FEN1 PE=1 SV=1 - [FEN1_HUMAN]
Q7Z4Q2	HEAT repeat-containing protein 3 OS=Homo sapiens GN=HEATR3 PE=1 SV=2 - [HEAT3_HUMAN]
Q9H3N1	Thioredoxin-related transmembrane protein 1 OS=Homo sapiens GN=TMX1 PE=1 SV=1 - [TMX1_HUMAN]
Q96EP5	DAZ-associated protein 1 OS=Homo sapiens GN=DAZAP1 PE=1 SV=1 - [DAZP1_HUMAN]
O75874	Isocitrate dehydrogenase [NADP] cytoplasmic OS=Homo sapiens GN=IDH1 PE=1 SV=2 - [IDHC_HUMAN]
P10644	cAMP-dependent protein kinase type I-alpha regulatory subunit OS=Homo sapiens GN=PRKAR1A PE=1 SV=1 - [KAP0_HUMAN]
Q00266	S-adenosylmethionine synthase isoform type-1 OS=Homo sapiens GN=MAT1A PE=1 SV=2 - [METK1_HUMAN]
P35659	Protein DEK OS=Homo sapiens GN=DEK PE=1 SV=1 - [DEK_HUMAN]
Q71RC2	La-related protein 4 OS=Homo sapiens GN=LARP4 PE=1 SV=3 - [LARP4_HUMAN]
Q9NSV4	Protein diaphanous homolog 3 OS=Homo sapiens GN=DIAPH3 PE=1 SV=4 - [DIAP3_HUMAN]
Q9UBU8	Mortality factor 4-like protein 1 OS=Homo sapiens GN=MORF4L1 PE=1 SV=2 - [MO4L1_HUMAN]
O95905	Protein SGT1 OS=Homo sapiens GN=ECD PE=1 SV=1 - [SGT1_HUMAN]
Q9H4L7	SWI/SNF-related matrix-associated actin-dependent regulator of chromatin subfamily A containing DEAD/H box 1 OS=Homo sapiens GN=SMARCAD1 PE=1 SV=2 - [SMRCD_HUMAN]
Q7L5Y9	Macrophage erythroblast attacher OS=Homo sapiens GN=MAEA PE=1 SV=1 - [MAEA_HUMAN]
Q99986	Serine/threonine-protein kinase VRK1 OS=Homo sapiens GN=VRK1 PE=1 SV=1 - [VRK1_HUMAN]
Q14457	Beclin-1 OS=Homo sapiens GN=BECN1 PE=1 SV=2 - [BECN1_HUMAN]
Q15417	Calponin-3 OS=Homo sapiens GN=CNN3 PE=1 SV=1 - [CNN3_HUMAN]
Q6PJI9	WD repeat-containing protein 59 OS=Homo sapiens GN=WDR59 PE=1 SV=2 - [WDR59_HUMAN]
Q9Y2I1	Nischarin OS=Homo sapiens GN=NISCH PE=1 SV=3 - [NISCH_HUMAN]
Q9Y2K7	Lysine-specific demethylase 2A OS=Homo sapiens GN=KDM2A PE=1 SV=3 - [KDM2A_HUMAN]
P29323	Ephrin type-B receptor 2 OS=Homo sapiens GN=EPHB2 PE=1 SV=5 - [EPHB2_HUMAN]
O75891	Cytosolic 10-formyltetrahydrofolate dehydrogenase OS=Homo sapiens GN=ALDH1L1 PE=1 SV=2 - [AL1L1_HUMAN]
P53004	Biliverdin reductase A OS=Homo sapiens GN=BLVRA PE=1 SV=2 - [BIEA_HUMAN]
P16333	Cytoplasmic protein NCK1 OS=Homo sapiens GN=NCK1 PE=1 SV=1 - [NCK1_HUMAN]
Q5GLZ8	Probable E3 ubiquitin-protein ligase HERC4 OS=Homo sapiens GN=HERC4 PE=1 SV=1 - [HERC4_HUMAN]
Q9NZB2	Constitutive coactivator of PPAR-gamma-like protein 1 OS=Homo sapiens GN=FAM120A PE=1 SV=2 - [F120A_HUMAN]
Q96QU8	Exportin-6 OS=Homo sapiens GN=XPO6 PE=1 SV=1 - [XPO6_HUMAN]
Q9UDY4	DnaJ homolog subfamily B member 4 OS=Homo sapiens GN=DNAJB4 PE=1 SV=1 - [DNJB4_HUMAN]
P20645	Cation-dependent mannose-6-phosphate receptor OS=Homo sapiens GN=M6PR PE=1 SV=1 - [MPRD_HUMAN]
Q86WB0	Nuclear-interacting partner of ALK OS=Homo sapiens GN=ZC3HC1 PE=1 SV=1 - [NIPA_HUMAN]
Q93034	Cullin-5 OS=Homo sapiens GN=CUL5 PE=1 SV=4 - [CUL5_HUMAN]
Q96KP4	Cytosolic non-specific dipeptidase OS=Homo sapiens GN=CNDP2 PE=1 SV=2 - [CNDP2_HUMAN]
Q96H20	Vacuolar-sorting protein SNF8 OS=Homo sapiens GN=SNF8 PE=1 SV=1 - [SNF8_HUMAN]
Q9C0I1	Myotubularin-related protein 12 OS=Homo sapiens GN=MTMR12 PE=1 SV=2 - [MTMRC_HUMAN]
P61758	Prefoldin subunit 3 OS=Homo sapiens GN=VBP1 PE=1 SV=3 - [PFD3_HUMAN]
Q969H6	Ribonuclease P/MRP protein subunit POP5 OS=Homo sapiens GN=POP5 PE=1 SV=1 - [POP5_HUMAN]
O14936	Peripheral plasma membrane protein CASK OS=Homo sapiens GN=CASK PE=1 SV=3 - [CSKP_HUMAN]
P10398	Serine/threonine-protein kinase A-Raf OS=Homo sapiens GN=ARAF PE=1 SV=2 - [ARAF_HUMAN]
Q13042	Cell division cycle protein 16 homolog OS=Homo sapiens GN=CDC16 PE=1 SV=2 - [CDC16_HUMAN]
Q8IWY4	Signal peptide, CUB and EGF-like domain-containing protein 1 OS=Homo sapiens GN=SCUBE1 PE=1 SV=3 - [SCUB1_HUMAN]
Q9Y6E0	Serine/threonine-protein kinase 24 OS=Homo sapiens GN=STK24 PE=1 SV=1 - [STK24_HUMAN]
P55263	Adenosine kinase OS=Homo sapiens GN=ADK PE=1 SV=2 - [ADK_HUMAN]
P11388	DNA topoisomerase 2-alpha OS=Homo sapiens GN=TOP2A PE=1 SV=3 - [TOP2A_HUMAN]
P14735	Insulin-degrading enzyme OS=Homo sapiens GN=IDE PE=1 SV=4 - [IDE_HUMAN]
P61599	N-alpha-acetyltransferase 20 OS=Homo sapiens GN=NAA20 PE=1 SV=1 - [NAA20_HUMAN]
P41743	Protein kinase C iota type OS=Homo sapiens GN=PRKCI PE=1 SV=2 - [KPCI_HUMAN]
Q86U70	LIM domain-binding protein 1 OS=Homo sapiens GN=LDB1 PE=1 SV=2 - [LDB1_HUMAN]
O15127	Secretory carrier-associated membrane protein 2 OS=Homo sapiens GN=SCAMP2 PE=1 SV=2 - [SCAM2_HUMAN]
O60294	tRNA wybutosine-synthesizing protein 4 OS=Homo sapiens GN=LCMT2 PE=1 SV=3 - [TYW4_HUMAN]
C9JRZ8	Aldo-keto reductase family 1 member B15 OS=Homo sapiens GN=AKR1B15 PE=1 SV=2 - [AK1BF_HUMAN]
O14773	Tripeptidyl-peptidase 1 OS=Homo sapiens GN=TPP1 PE=1 SV=2 - [TPP1_HUMAN]

P11274	Breakpoint cluster region protein OS=Homo sapiens GN=BCR PE=1 SV=2 - [BCR_HUMAN]
Q2NKX8	DNA excision repair protein ERCC-6-like OS=Homo sapiens GN=ERCC6L PE=1 SV=1 - [ERC6L_HUMAN]
Q9BZF3	Oxysterol-binding protein-related protein 6 OS=Homo sapiens GN=OSBPL6 PE=1 SV=1 - [OSBL6_HUMAN]
Q5PSV4	Breast cancer metastasis-suppressor 1-like protein OS=Homo sapiens GN=BRMS1L PE=1 SV=2 - [BRM1L_HUMAN]
Q5SY16	Polynucleotide 5'-hydroxyl-kinase NOL9 OS=Homo sapiens GN=NOL9 PE=1 SV=1 - [NOL9_HUMAN]
Q9ULA0	Aspartyl aminopeptidase OS=Homo sapiens GN=DNPEP PE=1 SV=1 - [DNPEP_HUMAN]
Q9Y6W3	Calpain-7 OS=Homo sapiens GN=CAPN7 PE=1 SV=1 - [CAN7_HUMAN]
O95758	Polypyrimidine tract-binding protein 3 OS=Homo sapiens GN=PTBP3 PE=1 SV=2 - [PTBP3_HUMAN]
Q86U44	N6-adenosine-methyltransferase 70 kDa subunit OS=Homo sapiens GN=METTL3 PE=1 SV=2 - [MTA70_HUMAN]
Q8NFF5	FAD synthase OS=Homo sapiens GN=FLAD1 PE=1 SV=1 - [FAD1_HUMAN]
Q9HCU4	Cadherin EGF LAG seven-pass G-type receptor 2 OS=Homo sapiens GN=CELSR2 PE=1 SV=1 - [CELR2_HUMAN]
Q8WWI5	Choline transporter-like protein 1 OS=Homo sapiens GN=SLC44A1 PE=1 SV=1 - [CTL1_HUMAN]
Q9H0A0	N-acetyltransferase 10 OS=Homo sapiens GN=NAT10 PE=1 SV=2 - [NAT10_HUMAN]
Q96JB5	CDK5 regulatory subunit-associated protein 3 OS=Homo sapiens GN=CDK5RAP3 PE=1 SV=2 - [CK5P3_HUMAN]
P01877	Ig alpha-2 chain C region OS=Homo sapiens GN=IGHA2 PE=1 SV=3 - [IGHA2_HUMAN]
Q3SY69	Mitochondrial 10-formyltetrahydrofolate dehydrogenase OS=Homo sapiens GN=ALDH1L2 PE=1 SV=2 - [AL1L2_HUMAN]
Q6PIW4	Fidgetin-like protein 1 OS=Homo sapiens GN=FIGNL1 PE=1 SV=2 - [FIGL1_HUMAN]
Q562E7	WD repeat-containing protein 81 OS=Homo sapiens GN=WDR81 PE=1 SV=2 - [WDR81_HUMAN]
Q9UIW2	Plexin-A1 OS=Homo sapiens GN=PLXNA1 PE=1 SV=3 - [PLXA1_HUMAN]
Q8N201	Integrator complex subunit 1 OS=Homo sapiens GN=INTS1 PE=1 SV=2 - [INT1_HUMAN]
Q92688	Acidic leucine-rich nuclear phosphoprotein 32 family member B OS=Homo sapiens GN=ANP32B PE=1 SV=1 - [AN32B_HUMAN]
P52306	Rap1 GTPase-GDP dissociation stimulator 1 OS=Homo sapiens GN=RAP1GDS1 PE=1 SV=3 - [GDS1_HUMAN]
Q96LB3	Intraflagellar transport protein 74 homolog OS=Homo sapiens GN=IFT74 PE=1 SV=1 - [IFT74_HUMAN]
Q96PE3	Type I inositol 3,4-bisphosphate 4-phosphatase OS=Homo sapiens GN=INPP4A PE=1 SV=1 - [INP4A_HUMAN]
Q13362	Serine/threonine-protein phosphatase 2A 56 kDa regulatory subunit gamma isoform OS=Homo sapiens GN=PPP2R5C PE=1 SV=3 - [2A5G_HUMAN]
Q9BRX2	Protein pelota homolog OS=Homo sapiens GN=PELO PE=1 SV=2 - [PELO_HUMAN]
Q13496	Myotubularin OS=Homo sapiens GN=MTM1 PE=1 SV=2 - [MTM1_HUMAN]
Q15904	V-type proton ATPase subunit S1 OS=Homo sapiens GN=ATP6AP1 PE=1 SV=2 - [VAS1_HUMAN]
Q9UGM3	Deleted in malignant brain tumors 1 protein OS=Homo sapiens GN=DMBT1 PE=1 SV=2 - [DMBT1_HUMAN]
Q16186	Proteasomal ubiquitin receptor ADRM1 OS=Homo sapiens GN=ADRM1 PE=1 SV=2 - [ADRM1_HUMAN]
O95747	Serine/threonine-protein kinase OSR1 OS=Homo sapiens GN=OXSR1 PE=1 SV=1 - [OXSR1_HUMAN]
P20023	Complement receptor type 2 OS=Homo sapiens GN=CR2 PE=1 SV=2 - [CR2_HUMAN]
Q6P3X3	Tetratricopeptide repeat protein 27 OS=Homo sapiens GN=TTC27 PE=1 SV=1 - [TTC27_HUMAN]
O95857	Tetraspanin-13 OS=Homo sapiens GN=TSPAN13 PE=2 SV=1 - [TSN13_HUMAN]
P17948	Vascular endothelial growth factor receptor 1 OS=Homo sapiens GN=FLT1 PE=1 SV=2 - [VGFR1_HUMAN]
Q8TD16	Protein bicaudal D homolog 2 OS=Homo sapiens GN=BICD2 PE=1 SV=1 - [BICD2_HUMAN]
O43542	DNA repair protein XRCC3 OS=Homo sapiens GN=XRCC3 PE=1 SV=1 - [XRCC3_HUMAN]
Q14498	RNA-binding protein 39 OS=Homo sapiens GN=RBM39 PE=1 SV=2 - [RBM39_HUMAN]
Q504Q3	PAB-dependent poly(A)-specific ribonuclease subunit PAN2 OS=Homo sapiens GN=PAN2 PE=1 SV=3 - [PAN2_HUMAN]
O43570	Carbonic anhydrase 12 OS=Homo sapiens GN=CA12 PE=1 SV=1 - [CAH12_HUMAN]
Q8TAA9	Vang-like protein 1 OS=Homo sapiens GN=VANGL1 PE=1 SV=1 - [VANG1_HUMAN]
O75674	TOM1-like protein 1 OS=Homo sapiens GN=TOM1L1 PE=1 SV=2 - [TM1L1_HUMAN]
Q92917	G patch domain and KOW motifs-containing protein OS=Homo sapiens GN=GPKOW PE=1 SV=2 - [GPKOW_HUMAN]
Q9H3G5	Probable serine carboxypeptidase CPVL OS=Homo sapiens GN=CPVL PE=1 SV=2 - [CPVL_HUMAN]
Q9Y6K9	NF-kappa-B essential modulator OS=Homo sapiens GN=IKBKG PE=1 SV=2 - [NEMO_HUMAN]
Q00587	Cdc42 effector protein 1 OS=Homo sapiens GN=CDC42EP1 PE=1 SV=1 - [BORG5_HUMAN]
Q9Y2S6	Translation machinery-associated protein 7 OS=Homo sapiens GN=TMA7 PE=1 SV=1 - [TMA7_HUMAN]
P04049	RAF proto-oncogene serine/threonine-protein kinase OS=Homo sapiens GN=RAF1 PE=1 SV=1 - [RAF1_HUMAN]
P49406	39S ribosomal protein L19, mitochondrial OS=Homo sapiens GN=MRPL19 PE=1 SV=2 - [RM19_HUMAN]
Q8IWF2	FAD-dependent oxidoreductase domain-containing protein 2 OS=Homo sapiens GN=FOXRED2 PE=1 SV=1 - [FXRD2_HUMAN]
Q96HR3	Mediator of RNA polymerase II transcription subunit 30 OS=Homo sapiens GN=MED30 PE=1 SV=1 - [MED30_HUMAN]
Q9BUK6	Protein misato homolog 1 OS=Homo sapiens GN=MSTO1 PE=1 SV=1 - [MSTO1_HUMAN]
Q9H7B4	Histone-lysine N-methyltransferase SMYD3 OS=Homo sapiens GN=SMYD3 PE=1 SV=4 - [SMYD3_HUMAN]
Q9HB63	Netrin-4 OS=Homo sapiens GN=NTN4 PE=1 SV=2 - [NET4_HUMAN]
Q8NC42	E3 ubiquitin-protein ligase RNF149 OS=Homo sapiens GN=RNF149 PE=2 SV=2 - [RN149_HUMAN]
Q5T0F9	Coiled-coil and C2 domain-containing protein 1B OS=Homo sapiens GN=CC2D1B PE=1 SV=1 - [C2D1B_HUMAN]
Q9UJ41	Rab5 GDP/GTP exchange factor OS=Homo sapiens GN=RABGEF1 PE=1 SV=2 - [RABX5_HUMAN]
Q86XP3	ATP-dependent RNA helicase DDX42 OS=Homo sapiens GN=DDX42 PE=1 SV=1 - [DDX42_HUMAN]
O14578	Citron Rho-interacting kinase OS=Homo sapiens GN=CIT PE=1 SV=2 - [CTRO_HUMAN]
Q9H814	Phosphorylated adapter RNA export protein OS=Homo sapiens GN=PHAX PE=1 SV=1 - [PHAX_HUMAN]
P62487	DNA-directed RNA polymerase II subunit RPB7 OS=Homo sapiens GN=POLR2G PE=1 SV=1 - [RPB7_HUMAN]
Q32P41	tRNA (guanine(37)-N1)-methyltransferase OS=Homo sapiens GN=TRMT5 PE=1 SV=2 - [TRM5_HUMAN]
Q15738	Sterol-4-alpha-carboxylate 3-dehydrogenase, decarboxylating OS=Homo sapiens GN=NSDHL PE=1 SV=2 - [NSDHL_HUMAN]
Q9Y240	C-type lectin domain family 11 member A OS=Homo sapiens GN=CLEC11A PE=1 SV=1 - [CLC11_HUMAN]
P19022	Cadherin-2 OS=Homo sapiens GN=CDH2 PE=1 SV=4 - [CADH2_HUMAN]
P82094	TATA element modulatory factor OS=Homo sapiens GN=TMF1 PE=1 SV=2 - [TMF1_HUMAN]
O15173	Membrane-associated progesterone receptor component 2 OS=Homo sapiens GN=PGRMC2 PE=1 SV=1 - [PGRC2_HUMAN]
Q16563	Synaptophysin-like protein 1 OS=Homo sapiens GN=SYPL1 PE=1 SV=1 - [SYPL1_HUMAN]
Q6ZRP7	Sulfhydryl oxidase 2 OS=Homo sapiens GN=QSOX2 PE=1 SV=3 - [QSOX2_HUMAN]
O60244	Mediator of RNA polymerase II transcription subunit 14 OS=Homo sapiens GN=MED14 PE=1 SV=2 - [MED14_HUMAN]
P01344	Insulin-like growth factor II OS=Homo sapiens GN=IGF2 PE=1 SV=1 - [IGF2_HUMAN]
Q8WVT3	Trafficking protein particle complex subunit 12 OS=Homo sapiens GN=TRAPPC12 PE=1 SV=3 - [TPC12_HUMAN]
Q04726	Transducin-like enhancer protein 3 OS=Homo sapiens GN=TLE3 PE=1 SV=2 - [TLE3_HUMAN]
Q16877	6-phosphofructo-2-kinase/fructose-2,6-bisphosphatase 4 OS=Homo sapiens GN=PFKFB4 PE=2 SV=6 - [F264_HUMAN]
O14981	TATA-binding protein-associated factor 172 OS=Homo sapiens GN=BTAF1 PE=1 SV=2 - [BTAF1_HUMAN]
O15530	3-phosphoinositide-dependent protein kinase 1 OS=Homo sapiens GN=PDPK1 PE=1 SV=1 - [PDPK1_HUMAN]
Q9UPN7	Serine/threonine-protein phosphatase 6 regulatory subunit 1 OS=Homo sapiens GN=PPP6R1 PE=1 SV=5 - [PP6R1_HUMAN]
P04156	Major prion protein OS=Homo sapiens GN=PRNP PE=1 SV=1 - [PRIO_HUMAN]
Q96P47	Arf-GAP with GTPase, ANK repeat and PH domain-containing protein 3 OS=Homo sapiens GN=AGAP3 PE=1 SV=2 - [AGAP3_HUMAN]
Q6GYQ0	Ral GTPase-activating protein subunit alpha-1 OS=Homo sapiens GN=RALGAPA1 PE=1 SV=1 - [RGPA1_HUMAN]
Q9H9C1	Spermatogenesis-defective protein 39 homolog OS=Homo sapiens GN=VIPAS39 PE=1 SV=1 - [SPE39_HUMAN]
Q96PZ0	Pseudouridylate synthase 7 homolog OS=Homo sapiens GN=PUS7 PE=1 SV=2 - [PUS7_HUMAN]
Q99567	Nuclear pore complex protein Nup88 OS=Homo sapiens GN=NUP88 PE=1 SV=2 - [NUP88_HUMAN]
P36542	ATP synthase subunit gamma, mitochondrial OS=Homo sapiens GN=ATP5C1 PE=1 SV=1 - [ATPG_HUMAN]
Q9Y5X2	Sorting nexin-8 OS=Homo sapiens GN=SNX8 PE=1 SV=1 - [SNX8_HUMAN]
Q9HD45	Transmembrane 9 superfamily member 3 OS=Homo sapiens GN=TM9SF3 PE=1 SV=2 - [TM9S3_HUMAN]
Q9P2E5	Chondroitin sulfate glucuronyltransferase OS=Homo sapiens GN=CHPF2 PE=2 SV=2 - [CHPF2_HUMAN]
Q9P0U4	CXXC-type zinc finger protein 1 OS=Homo sapiens GN=CXXC1 PE=1 SV=2 - [CXXC1_HUMAN]
Q16881	Thioredoxin reductase 1, cytoplasmic OS=Homo sapiens GN=TXNRD1 PE=1 SV=3 - [TRXR1_HUMAN]
Q15276	Rab GTPase-binding effector protein 1 OS=Homo sapiens GN=RABEP1 PE=1 SV=2 - [RABE1_HUMAN]
O43818	U3 small nucleolar RNA-interacting protein 2 OS=Homo sapiens GN=RRP9 PE=1 SV=1 - [U3IP2_HUMAN]
Q7L014	Probable ATP-dependent RNA helicase DDX46 OS=Homo sapiens GN=DDX46 PE=1 SV=2 - [DDX46_HUMAN]
Q9H2P9	Diphthine synthase OS=Homo sapiens GN=DPH5 PE=1 SV=2 - [DPH5_HUMAN]
O00442	RNA 3'-terminal phosphate cyclase OS=Homo sapiens GN=RTCA PE=1 SV=1 - [RTCA_HUMAN]
Q9Y5Q9	General transcription factor 3C polypeptide 3 OS=Homo sapiens GN=GTF3C3 PE=1 SV=1 - [TF3C3_HUMAN]
Q13049	E3 ubiquitin-protein ligase TRIM32 OS=Homo sapiens GN=TRIM32 PE=1 SV=2 - [TRI32_HUMAN]
O00506	Serine/threonine-protein kinase 25 OS=Homo sapiens GN=STK25 PE=1 SV=1 - [STK25_HUMAN]
Q15043	Zinc transporter ZIP14 OS=Homo sapiens GN=SLC39A14 PE=1 SV=3 - [S39AE_HUMAN]
O15126	Secretory carrier-associated membrane protein 1 OS=Homo sapiens GN=SCAMP1 PE=1 SV=2 - [SCAM1_HUMAN]
Q9BQ90	Kelch domain-containing protein 3 OS=Homo sapiens GN=KLHDC3 PE=2 SV=1 - [KLDC3_HUMAN]
Q05086	Ubiquitin-protein ligase E3A OS=Homo sapiens GN=UBE3A PE=1 SV=4 - [UBE3A_HUMAN]
Q9H497	Torsin-3A OS=Homo sapiens GN=TOR3A PE=1 SV=1 - [TOR3A_HUMAN]
O14776	Transcription elongation regulator 1 OS=Homo sapiens GN=TCERG1 PE=1 SV=2 - [TCRG1_HUMAN]
Q9H0H0	Integrator complex subunit 2 OS=Homo sapiens GN=INTS2 PE=1 SV=2 - [INT2_HUMAN]
Q14185	Dedicator of cytokinesis protein 1 OS=Homo sapiens GN=DOCK1 PE=1 SV=2 - [DOCK1_HUMAN]
Q9HB40	Retinoid-inducible serine carboxypeptidase OS=Homo sapiens GN=SCPEP1 PE=1 SV=1 - [RISC_HUMAN]
P08572	Collagen alpha-2(IV) chain OS=Homo sapiens GN=COL4A2 PE=1 SV=4 - [CO4A2_HUMAN]
O95239	Chromosome-associated kinesin KIF4A OS=Homo sapiens GN=KIF4A PE=1 SV=3 - [KIF4A_HUMAN]
O95453	Poly(A)-specific ribonuclease PARN OS=Homo sapiens GN=PARN PE=1 SV=1 - [PARN_HUMAN]
Q8IWA5	Choline transporter-like protein 2 OS=Homo sapiens GN=SLC44A2 PE=1 SV=3 - [CTL2_HUMAN]
Q9BQS8	FYVE and coiled-coil domain-containing protein 1 OS=Homo sapiens GN=FYCO1 PE=1 SV=3 - [FYCO1_HUMAN]
Q9P2L0	WD repeat-containing protein 35 OS=Homo sapiens GN=WDR35 PE=1 SV=3 - [WDR35_HUMAN]
P08962	CD63 antigen OS=Homo sapiens GN=CD63 PE=1 SV=2 - [CD63_HUMAN]
Q8WW01	tRNA-splicing endonuclease subunit Sen15 OS=Homo sapiens GN=TSEN15 PE=1 SV=1 - [SEN15_HUMAN]
O75695	Protein XRP2 OS=Homo sapiens GN=RP2 PE=1 SV=4 - [XRP2_HUMAN]

Q01968	Inositol polyphosphate 5-phosphatase OCRL-1 OS=Homo sapiens GN=OCRL PE=1 SV=3 - [OCRL_HUMAN]
Q9UNY4	Transcription termination factor 2 OS=Homo sapiens GN=TTF2 PE=1 SV=2 - [TTF2_HUMAN]
Q8IZ07	Ankyrin repeat domain-containing protein 13A OS=Homo sapiens GN=ANKRD13A PE=1 SV=3 - [AN13A_HUMAN]
Q9Y297	F-box/WD repeat-containing protein 1A OS=Homo sapiens GN=BTRC PE=1 SV=1 - [FBW1A_HUMAN]
O94967	WD repeat-containing protein 47 OS=Homo sapiens GN=WDR47 PE=1 SV=1 - [WDR47_HUMAN]
Q04724	Transducin-like enhancer protein 1 OS=Homo sapiens GN=TLE1 PE=1 SV=2 - [TLE1_HUMAN]
Q9NVR2	Integrator complex subunit 10 OS=Homo sapiens GN=INTS10 PE=1 SV=2 - [INT10_HUMAN]
P17066	Heat shock 70 kDa protein 6 OS=Homo sapiens GN=HSPA6 PE=1 SV=2 - [HSP76_HUMAN]
Q8IZH2	5'-3' exoribonuclease 1 OS=Homo sapiens GN=XRN1 PE=1 SV=1 - [XRN1_HUMAN]
Q9NYL9	Tropomodulin-3 OS=Homo sapiens GN=TMOD3 PE=1 SV=1 - [TMOD3_HUMAN]
Q8IYS1	Peptidase M20 domain-containing protein 2 OS=Homo sapiens GN=PM20D2 PE=1 SV=2 - [P20D2_HUMAN]
Q9BR76	Coronin-1B OS=Homo sapiens GN=CORO1B PE=1 SV=1 - [COR1B_HUMAN]
P46019	Phosphorylase b kinase regulatory subunit alpha, liver isoform OS=Homo sapiens GN=PHKA2 PE=1 SV=1 - [KPB2_HUMAN]
Q13439	Golgin subfamily A member 4 OS=Homo sapiens GN=GOLGA4 PE=1 SV=1 - [GOGA4_HUMAN]
Q9Y2J2	Band 4.1-like protein 3 OS=Homo sapiens GN=EPB41L3 PE=1 SV=2 - [E41L3_HUMAN]
Q14624	Inter-alpha-trypsin inhibitor heavy chain H4 OS=Homo sapiens GN=ITIH4 PE=1 SV=4 - [ITIH4_HUMAN]
P50895	Basal cell adhesion molecule OS=Homo sapiens GN=BCAM PE=1 SV=2 - [BCAM_HUMAN]
Q9HAC8	Ubiquitin domain-containing protein 1 OS=Homo sapiens GN=UBTD1 PE=1 SV=1 - [UBTD1_HUMAN]
O15357	Phosphatidylinositol 3,4,5-trisphosphate 5-phosphatase 2 OS=Homo sapiens GN=INPPL1 PE=1 SV=2 - [SHIP2_HUMAN]
Q9UK97	F-box only protein 9 OS=Homo sapiens GN=FBXO9 PE=1 SV=1 - [FBX9_HUMAN]
O75688	Protein phosphatase 1B OS=Homo sapiens GN=PPM1B PE=1 SV=1 - [PPM1B_HUMAN]
Q14669	E3 ubiquitin-protein ligase TRIP12 OS=Homo sapiens GN=TRIP12 PE=1 SV=1 - [TRIPC_HUMAN]
Q9BZK3	Putative nascent polypeptide-associated complex subunit alpha-like protein OS=Homo sapiens GN=NACAP1 PE=5 SV=1 - [NACP1_HUMAN]
P50993	Sodium/potassium-transporting ATPase subunit alpha-2 OS=Homo sapiens GN=ATP1A2 PE=1 SV=1 - [AT1A2_HUMAN]
Q9UM47	Neurogenic locus notch homolog protein 3 OS=Homo sapiens GN=NOTCH3 PE=1 SV=2 - [NOTC3_HUMAN]
O76003	Glutaredoxin-3 OS=Homo sapiens GN=GLRX3 PE=1 SV=2 - [GLRX3_HUMAN]
Q9NY47	Voltage-dependent calcium channel subunit alpha-2/delta-2 OS=Homo sapiens GN=CACNA2D2 PE=1 SV=2 - [CA2D2_HUMAN]
Q8NCE0	tRNA-splicing endonuclease subunit Sen2 OS=Homo sapiens GN=TSEN2 PE=1 SV=2 - [SEN2_HUMAN]
P09497	Clathrin light chain B OS=Homo sapiens GN=CLTB PE=1 SV=1 - [CLCB_HUMAN]
P78324	Tyrosine-protein phosphatase non-receptor type substrate 1 OS=Homo sapiens GN=SIRPA PE=1 SV=2 - [SHPS1_HUMAN]
Q9H6R4	Nucleolar protein 6 OS=Homo sapiens GN=NOL6 PE=1 SV=2 - [NOL6_HUMAN]
Q4V328	GRIP1-associated protein 1 OS=Homo sapiens GN=GRIPAP1 PE=1 SV=1 - [GRAP1_HUMAN]
O95155	Ubiquitin conjugation factor E4 B OS=Homo sapiens GN=UBE4B PE=1 SV=1 - [UBE4B_HUMAN]
Q8IW35	Centrosomal protein of 97 kDa OS=Homo sapiens GN=CEP97 PE=1 SV=1 - [CEP97_HUMAN]
Q9NY35	Claudin domain-containing protein 1 OS=Homo sapiens GN=CLDND1 PE=1 SV=1 - [CLDN1_HUMAN]
Q9H8T0	AKT-interacting protein OS=Homo sapiens GN=AKTIP PE=1 SV=1 - [AKTIP_HUMAN]
Q9Y6R4	Mitogen-activated protein kinase kinase kinase 4 OS=Homo sapiens GN=MAP3K4 PE=1 SV=2 - [M3K4_HUMAN]
Q7Z739	YTH domain-containing family protein 3 OS=Homo sapiens GN=YTHDF3 PE=1 SV=1 - [YTHD3_HUMAN]
Q13017	Rho GTPase-activating protein 5 OS=Homo sapiens GN=ARHGAP5 PE=1 SV=2 - [RHG05_HUMAN]
Q15642	Cdc42-interacting protein 4 OS=Homo sapiens GN=TRIP10 PE=1 SV=3 - [CIP4_HUMAN]
Q14012	Calcium/calmodulin-dependent protein kinase type 1 OS=Homo sapiens GN=CAMK1 PE=1 SV=1 - [KCC1A_HUMAN]
P62877	E3 ubiquitin-protein ligase RBX1 OS=Homo sapiens GN=RBX1 PE=1 SV=1 - [RBX1_HUMAN]
O75113	NEDD4-binding protein 1 OS=Homo sapiens GN=N4BP1 PE=1 SV=4 - [N4BP1_HUMAN]
O94822	E3 ubiquitin-protein ligase listerin OS=Homo sapiens GN=LTN1 PE=1 SV=6 - [LTN1_HUMAN]
P35443	Thrombospondin-4 OS=Homo sapiens GN=THBS4 PE=1 SV=2 - [TSP4_HUMAN]
O14976	Cyclin-G-associated kinase OS=Homo sapiens GN=GAK PE=1 SV=2 - [GAK_HUMAN]
Q92574	Hamartin OS=Homo sapiens GN=TSC1 PE=1 SV=2 - [TSC1_HUMAN]
O15072	A disintegrin and metalloproteinase with thrombospondin motifs 3 OS=Homo sapiens GN=ADAMTS3 PE=2 SV=4 - [ATS3_HUMAN]
P06732	Creatine kinase M-type OS=Homo sapiens GN=CKM PE=1 SV=2 - [KCRM_HUMAN]
Q13641	Trophoblast glycoprotein OS=Homo sapiens GN=TPBG PE=1 SV=1 - [TPBG_HUMAN]
Q13188	Serine/threonine-protein kinase 3 OS=Homo sapiens GN=STK3 PE=1 SV=2 - [STK3_HUMAN]
O60610	Protein diaphanous homolog 1 OS=Homo sapiens GN=DIAPH1 PE=1 SV=2 - [DIAP1_HUMAN]
P00738	Haptoglobin OS=Homo sapiens GN=HP PE=1 SV=1 - [HPT_HUMAN]
Q9Y6D9	Mitotic spindle assembly checkpoint protein MAD1 OS=Homo sapiens GN=MAD1L1 PE=1 SV=2 - [MD1L1_HUMAN]
P42898	Methylenetetrahydrofolate reductase OS=Homo sapiens GN=MTHFR PE=1 SV=3 - [MTHR_HUMAN]
Q13247	Serine/arginine-rich splicing factor 6 OS=Homo sapiens GN=SRSF6 PE=1 SV=2 - [SRSF6_HUMAN]
P38571	Lysosomal acid lipase/cholesteryl ester hydrolase OS=Homo sapiens GN=LIPA PE=1 SV=2 - [LICH_HUMAN]
Q15036	Sorting nexin-17 OS=Homo sapiens GN=SNX17 PE=1 SV=1 - [SNX17_HUMAN]
O75886	Signal transducing adapter molecule 2 OS=Homo sapiens GN=STAM2 PE=1 SV=1 - [STAM2_HUMAN]
P10619	Lysosomal protective protein OS=Homo sapiens GN=CTSA PE=1 SV=2 - [PPGB_HUMAN]
Q6ZRQ5	Protein MMS22-like OS=Homo sapiens GN=MMS22L PE=1 SV=3 - [MMS22_HUMAN]
P54105	Methylosome subunit pICln OS=Homo sapiens GN=CLNS1A PE=1 SV=1 - [ICLN_HUMAN]
Q9UEW8	STE20/SPS1-related proline-alanine-rich protein kinase OS=Homo sapiens GN=STK39 PE=1 SV=3 - [STK39_HUMAN]
Q9Y5P4	Collagen type IV alpha-3-binding protein OS=Homo sapiens GN=COL4A3BP PE=1 SV=1 - [C43BP_HUMAN]
O14656	Torsin-1A OS=Homo sapiens GN=TOR1A PE=1 SV=1 - [TOR1A_HUMAN]
P61956	Small ubiquitin-related modifier 2 OS=Homo sapiens GN=SUMO2 PE=1 SV=3 - [SUMO2_HUMAN]
Q9Y4A5	Transformation/transcription domain-associated protein OS=Homo sapiens GN=TRRAP PE=1 SV=3 - [TRRAP_HUMAN]
Q86YS7	C2 domain-containing protein 5 OS=Homo sapiens GN=C2CD5 PE=1 SV=1 - [C2CD5_HUMAN]
Q86UX7	Fermitin family homolog 3 OS=Homo sapiens GN=FERMT3 PE=1 SV=1 - [URP2_HUMAN]
Q9Y5J1	U3 small nucleolar RNA-associated protein 18 homolog OS=Homo sapiens GN=UTP18 PE=1 SV=3 - [UTP18_HUMAN]
Q9HCN4	GPN-loop GTPase 1 OS=Homo sapiens GN=GPN1 PE=1 SV=1 - [GPN1_HUMAN]
Q15061	WD repeat-containing protein 43 OS=Homo sapiens GN=WDR43 PE=1 SV=3 - [WDR43_HUMAN]
Q7LBR1	Charged multivesicular body protein 1b OS=Homo sapiens GN=CHMP1B PE=1 SV=1 - [CHM1B_HUMAN]
P48553	Trafficking protein particle complex subunit 10 OS=Homo sapiens GN=TRAPPC10 PE=1 SV=2 - [TPC10_HUMAN]
Q08170	Serine/arginine-rich splicing factor 4 OS=Homo sapiens GN=SRSF4 PE=1 SV=2 - [SRSF4_HUMAN]
Q96LA8	Protein arginine N-methyltransferase 6 OS=Homo sapiens GN=PRMT6 PE=1 SV=1 - [ANM6_HUMAN]
P46939	Utrophin OS=Homo sapiens GN=UTRN PE=1 SV=2 - [UTRO_HUMAN]
Q86V21	Acetoacetyl-CoA synthetase OS=Homo sapiens GN=AACS PE=1 SV=1 - [AACS_HUMAN]
Q96EY1	DnaJ homolog subfamily A member 3, mitochondrial OS=Homo sapiens GN=DNAJA3 PE=1 SV=2 - [DNJA3_HUMAN]
P39060	Collagen alpha-1(XVIII) chain OS=Homo sapiens GN=COL18A1 PE=1 SV=5 - [COIA1_HUMAN]
Q8WVM7	Cohesin subunit SA-1 OS=Homo sapiens GN=STAG1 PE=1 SV=3 - [STAG1_HUMAN]
P54764	Ephrin type-A receptor 4 OS=Homo sapiens GN=EPHA4 PE=1 SV=1 - [EPHA4_HUMAN]
Q92609	TBC1 domain family member 5 OS=Homo sapiens GN=TBC1D5 PE=1 SV=1 - [TBCD5_HUMAN]
P46087	Probable 28S rRNA (cytosine(4447)-C(5))-methyltransferase OS=Homo sapiens GN=NOP2 PE=1 SV=2 - [NOP2_HUMAN]
Q8N474	Secreted frizzled-related protein 1 OS=Homo sapiens GN=SFRP1 PE=1 SV=1 - [SFRP1_HUMAN]
O60271	C-Jun-amino-terminal kinase-interacting protein 4 OS=Homo sapiens GN=SPAG9 PE=1 SV=4 - [JIP4_HUMAN]
Q68CQ7	Glycosyltransferase 8 domain-containing protein 1 OS=Homo sapiens GN=GLT8D1 PE=1 SV=2 - [GL8D1_HUMAN]
P30260	Cell division cycle protein 27 homolog OS=Homo sapiens GN=CDC27 PE=1 SV=2 - [CDC27_HUMAN]
Q9ULX3	RNA-binding protein NOB1 OS=Homo sapiens GN=NOB1 PE=1 SV=1 - [NOB1_HUMAN]
Q14999	Cullin-7 OS=Homo sapiens GN=CUL7 PE=1 SV=2 - [CUL7_HUMAN]
O95980	Reversion-inducing cysteine-rich protein with Kazal motifs OS=Homo sapiens GN=RECK PE=1 SV=1 - [RECK_HUMAN]
Q8N954	G patch domain-containing protein 11 OS=Homo sapiens GN=GPATCH11 PE=1 SV=3 - [GPT11_HUMAN]
Q96HA7	Tonsoku-like protein OS=Homo sapiens GN=TONSL PE=1 SV=2 - [TONSL_HUMAN]
O95834	Echinoderm microtubule-associated protein-like 2 OS=Homo sapiens GN=EML2 PE=1 SV=1 - [EMAL2_HUMAN]
Q8TEA7	TBC domain-containing protein kinase-like protein OS=Homo sapiens GN=TBCK PE=1 SV=4 - [TBCK_HUMAN]
Q8IUE6	Histone H2A type 2-B OS=Homo sapiens GN=HIST2H2AB PE=1 SV=3 - [H2A2B_HUMAN]
Q9H3P7	Golgi resident protein GCP60 OS=Homo sapiens GN=ACBD3 PE=1 SV=4 - [GCP60_HUMAN]
Q9Y639	Neuroplastin OS=Homo sapiens GN=NPTN PE=1 SV=2 - [NPTN_HUMAN]
P38159	RNA-binding motif protein, X chromosome OS=Homo sapiens GN=RBMX PE=1 SV=3 - [RBMX_HUMAN]
Q8IUD2	ELKS/Rab6-interacting/CAST family member 1 OS=Homo sapiens GN=ERC1 PE=1 SV=1 - [RB6I2_HUMAN]
P18084	Integrin beta-5 OS=Homo sapiens GN=ITGB5 PE=1 SV=1 - [ITB5_HUMAN]
Q14789	Golgin subfamily B member 1 OS=Homo sapiens GN=GOLGB1 PE=1 SV=2 - [GOGB1_HUMAN]
P51532	Transcription activator BRG1 OS=Homo sapiens GN=SMARCA4 PE=1 SV=2 - [SMCA4_HUMAN]
Q9Y5A9	YTH domain-containing family protein 2 OS=Homo sapiens GN=YTHDF2 PE=1 SV=2 - [YTHD2_HUMAN]
O15397	Importin-8 OS=Homo sapiens GN=IPO8 PE=1 SV=2 - [IPO8_HUMAN]
P54802	Alpha-N-acetylglucosaminidase OS=Homo sapiens GN=NAGLU PE=1 SV=2 - [ANAG_HUMAN]
Q9Y4J8	Dystrobrevin alpha OS=Homo sapiens GN=DTNA PE=1 SV=2 - [DTNA_HUMAN]
P06746	DNA polymerase beta OS=Homo sapiens GN=POLB PE=1 SV=3 - [DPOLB_HUMAN]
Q86UY6	N-alpha-acetyltransferase 40 OS=Homo sapiens GN=NAA40 PE=1 SV=1 - [NAA40_HUMAN]
Q6UXI9	Nephronectin OS=Homo sapiens GN=NPNT PE=2 SV=3 - [NPNT_HUMAN]
Q7Z2W9	39S ribosomal protein L21, mitochondrial OS=Homo sapiens GN=MRPL21 PE=1 SV=2 - [RM21_HUMAN]
O15164	Transcription intermediary factor 1-alpha OS=Homo sapiens GN=TRIM24 PE=1 SV=3 - [TIF1A_HUMAN]
Q6P996	Pyridoxal-dependent decarboxylase domain-containing protein 1 OS=Homo sapiens GN=PDXDC1 PE=1 SV=2 - [PDXD1_HUMAN]
Q8NHH9	Atlastin-2 OS=Homo sapiens GN=ATL2 PE=1 SV=2 - [ATLA2_HUMAN]
O94868	F-BAR and double SH3 domains protein 2 OS=Homo sapiens GN=FCHSD2 PE=1 SV=3 - [FCSD2_HUMAN]

Q9BYJ9	YTH domain-containing family protein 1 OS=Homo sapiens GN=YTHDF1 PE=1 SV=1 - [YTHD1_HUMAN]
Q9H078	Caseinolytic peptidase B protein homolog OS=Homo sapiens GN=CLPB PE=1 SV=1 - [CLPB_HUMAN]
Q9Y496	Kinesin-like protein KIF3A OS=Homo sapiens GN=KIF3A PE=1 SV=4 - [KIF3A_HUMAN]
Q9BZJ0	Crooked neck-like protein 1 OS=Homo sapiens GN=CRNKL1 PE=1 SV=4 - [CRNL1_HUMAN]
Q9H1B5	Xylosyltransferase 2 OS=Homo sapiens GN=XYLT2 PE=2 SV=2 - [XYLT2_HUMAN]
O75128	Protein cordon-bleu OS=Homo sapiens GN=COBL PE=1 SV=2 - [COBL_HUMAN]
Q96EX3	WD repeat-containing protein 34 OS=Homo sapiens GN=WDR34 PE=1 SV=2 - [WDR34_HUMAN]
Q9BWH6	RNA polymerase II-associated protein 1 OS=Homo sapiens GN=RPAP1 PE=1 SV=3 - [RPAP1_HUMAN]
Q9Y6L7	Tolloid-like protein 2 OS=Homo sapiens GN=TLL2 PE=1 SV=1 - [TLL2_HUMAN]
O14646	Chromodomain-helicase-DNA-binding protein 1 OS=Homo sapiens GN=CHD1 PE=1 SV=2 - [CHD1_HUMAN]
Q9H074	Polyadenylate-binding protein-interacting protein 1 OS=Homo sapiens GN=PAIP1 PE=1 SV=1 - [PAIP1_HUMAN]
P09001	39S ribosomal protein L3, mitochondrial OS=Homo sapiens GN=MRPL3 PE=1 SV=1 - [RM03_HUMAN]
P12110	Collagen alpha-2(VI) chain OS=Homo sapiens GN=COL6A2 PE=1 SV=4 - [CO6A2_HUMAN]
P15907	Beta-galactoside alpha-2,6-sialyltransferase 1 OS=Homo sapiens GN=ST6GAL1 PE=1 SV=1 - [SIAT1_HUMAN]
P55081	Microfibrillar-associated protein 1 OS=Homo sapiens GN=MFAP1 PE=1 SV=2 - [MFAP1_HUMAN]
Q07960	Rho GTPase-activating protein 1 OS=Homo sapiens GN=ARHGAP1 PE=1 SV=1 - [RHG01_HUMAN]
Q99805	Transmembrane 9 superfamily member 2 OS=Homo sapiens GN=TM9SF2 PE=1 SV=1 - [TM9S2_HUMAN]
O94856	Neurofascin OS=Homo sapiens GN=NFASC PE=1 SV=4 - [NFASC_HUMAN]
Q86YT6	E3 ubiquitin-protein ligase MIB1 OS=Homo sapiens GN=MIB1 PE=1 SV=1 - [MIB1_HUMAN]
Q8IWZ6	Bardet-Biedl syndrome 7 protein OS=Homo sapiens GN=BBS7 PE=1 SV=2 - [BBS7_HUMAN]
Q9BUE0	Mediator of RNA polymerase II transcription subunit 18 OS=Homo sapiens GN=MED18 PE=1 SV=1 - [MED18_HUMAN]
Q9HBG6	Intraflagellar transport protein 122 homolog OS=Homo sapiens GN=IFT122 PE=1 SV=2 - [IF122_HUMAN]
O75146	Huntingtin-interacting protein 1-related protein OS=Homo sapiens GN=HIP1R PE=1 SV=2 - [HIP1R_HUMAN]
P15529	Membrane cofactor protein OS=Homo sapiens GN=CD46 PE=1 SV=3 - [MCP_HUMAN]
Q8IUH3	RNA-binding protein 45 OS=Homo sapiens GN=RBM45 PE=1 SV=1 - [RBM45_HUMAN]
Q96EV8	Dysbindin OS=Homo sapiens GN=DTNBP1 PE=1 SV=1 - [DTBP1_HUMAN]
Q70CQ2	Ubiquitin carboxyl-terminal hydrolase 34 OS=Homo sapiens GN=USP34 PE=1 SV=2 - [UBP34_HUMAN]
Q8TEV9	Smith-Magenis syndrome chromosomal region candidate gene 8 protein OS=Homo sapiens GN=SMCR8 PE=1 SV=2 - [SMCR8_HUMAN]
Q9P260	LisH domain and HEAT repeat-containing protein KIAA1468 OS=Homo sapiens GN=KIAA1468 PE=1 SV=2 - [K1468_HUMAN]
O75170	Serine/threonine-protein phosphatase 6 regulatory subunit 2 OS=Homo sapiens GN=PPP6R2 PE=1 SV=2 - [PP6R2_HUMAN]
Q8WYA6	Beta-catenin-like protein 1 OS=Homo sapiens GN=CTNNBL1 PE=1 SV=1 - [CTBL1_HUMAN]
Q6ZMP0	Thrombospondin type-1 domain-containing protein 4 OS=Homo sapiens GN=THSD4 PE=2 SV=2 - [THSD4_HUMAN]
Q86VX9	Vacuolar fusion protein MON1 homolog A OS=Homo sapiens GN=MON1A PE=1 SV=2 - [MON1A_HUMAN]
Q9H7L9	Sin3 histone deacetylase corepressor complex component SDS3 OS=Homo sapiens GN=SUDS3 PE=1 SV=2 - [SDS3_HUMAN]
Q9UI14	Prenylated Rab acceptor protein 1 OS=Homo sapiens GN=RABAC1 PE=1 SV=1 - [PRAF1_HUMAN]
O75529	TAF5-like RNA polymerase II p300/CBP-associated factor-associated factor 65 kDa subunit 5L OS=Homo sapiens GN=TAF5L PE=1 SV=1 - [TAF5L_HUMAN]
O75326	Semaphorin-7A OS=Homo sapiens GN=SEMA7A PE=1 SV=1 - [SEM7A_HUMAN]
Q5VZE5	N-alpha-acetyltransferase 35, NatC auxiliary subunit OS=Homo sapiens GN=NAA35 PE=1 SV=1 - [NAA35_HUMAN]
Q8NAV1	Pre-mRNA-splicing factor 38A OS=Homo sapiens GN=PRPF38A PE=1 SV=1 - [PR38A_HUMAN]
Q9BVL2	Nucleoporin p58/p45 OS=Homo sapiens GN=NUPL1 PE=1 SV=1 - [NUPL1_HUMAN]
Q9H089	Large subunit GTPase 1 homolog OS=Homo sapiens GN=LSG1 PE=1 SV=2 - [LSG1_HUMAN]
O94864	STAGA complex 65 subunit gamma OS=Homo sapiens GN=SUPT7L PE=1 SV=1 - [ST65G_HUMAN]
P53794	Sodium/myo-inositol cotransporter OS=Homo sapiens GN=SLC5A3 PE=3 SV=2 - [SC5A3_HUMAN]
Q8WYA0	Intraflagellar transport protein 81 homolog OS=Homo sapiens GN=IFT81 PE=1 SV=1 - [IFT81_HUMAN]
Q5VZK9	Leucine-rich repeat-containing protein 16A OS=Homo sapiens GN=LRRC16A PE=1 SV=1 - [LR16A_HUMAN]
P55011	Solute carrier family 12 member 2 OS=Homo sapiens GN=SLC12A2 PE=1 SV=1 - [S12A2_HUMAN]
Q9H9B4	Sideroflexin-1 OS=Homo sapiens GN=SFXN1 PE=1 SV=4 - [SFXN1_HUMAN]
O00264	Membrane-associated progesterone receptor component 1 OS=Homo sapiens GN=PGRMC1 PE=1 SV=3 - [PGRC1_HUMAN]
Q96BH1	E3 ubiquitin-protein ligase RNF25 OS=Homo sapiens GN=RNF25 PE=1 SV=1 - [RNF25_HUMAN]
Q9NS98	Semaphorin-3G OS=Homo sapiens GN=SEMA3G PE=2 SV=1 - [SEM3G_HUMAN]
Q9H9Q2	COP9 signalosome complex subunit 7b OS=Homo sapiens GN=COPS7B PE=1 SV=1 - [CSN7B_HUMAN]
Q9UJV9	Probable ATP-dependent RNA helicase DDX41 OS=Homo sapiens GN=DDX41 PE=1 SV=2 - [DDX41_HUMAN]
Q53T59	HCLS1-binding protein 3 OS=Homo sapiens GN=HS1BP3 PE=1 SV=1 - [H1BP3_HUMAN]
Q6S8J3	POTE ankyrin domain family member E OS=Homo sapiens GN=POTEE PE=1 SV=3 - [POTEE_HUMAN]
O14964	Hepatocyte growth factor-regulated tyrosine kinase substrate OS=Homo sapiens GN=HGS PE=1 SV=1 - [HGS_HUMAN]
Q96EN8	Molybdenum cofactor sulfurase OS=Homo sapiens GN=MOCOS PE=1 SV=2 - [MOCOS_HUMAN]
Q9NTZ6	RNA-binding protein 12 OS=Homo sapiens GN=RBM12 PE=1 SV=1 - [RBM12_HUMAN]
O75822	Eukaryotic translation initiation factor 3 subunit J OS=Homo sapiens GN=EIF3J PE=1 SV=2 - [EIF3J_HUMAN]
P51970	NADH dehydrogenase [ubiquinone] 1 alpha subcomplex subunit 8 OS=Homo sapiens GN=NDUFA8 PE=1 SV=3 - [NDUA8_HUMAN]
Q13472	DNA topoisomerase 3-alpha OS=Homo sapiens GN=TOP3A PE=1 SV=1 - [TOP3A_HUMAN]
Q9Y6Q5	AP-1 complex subunit mu-2 OS=Homo sapiens GN=AP1M2 PE=1 SV=4 - [AP1M2_HUMAN]
Q9Y487	V-type proton ATPase 116 kDa subunit a isoform 2 OS=Homo sapiens GN=ATP6V0A2 PE=1 SV=2 - [VPP2_HUMAN]
Q5TAQ9	DDB1- and CUL4-associated factor 8 OS=Homo sapiens GN=DCAF8 PE=1 SV=1 - [DCAF8_HUMAN]
Q3B7T1	Erythroid differentiation-related factor 1 OS=Homo sapiens GN=EDRF1 PE=1 SV=1 - [EDRF1_HUMAN]
O75976	Carboxypeptidase D OS=Homo sapiens GN=CPD PE=1 SV=2 - [CBPD_HUMAN]
O95297	Myelin protein zero-like protein 1 OS=Homo sapiens GN=MPZL1 PE=1 SV=1 - [MPZL1_HUMAN]
P06730	Eukaryotic translation initiation factor 4E OS=Homo sapiens GN=EIF4E PE=1 SV=2 - [IF4E_HUMAN]
P22059	Oxysterol-binding protein 1 OS=Homo sapiens GN=OSBP PE=1 SV=1 - [OSBP1_HUMAN]
P02458	Collagen alpha-1(II) chain OS=Homo sapiens GN=COL2A1 PE=1 SV=3 - [CO2A1_HUMAN]
Q14574	Desmocollin-3 OS=Homo sapiens GN=DSC3 PE=1 SV=3 - [DSC3_HUMAN]
Q7L7X3	Serine/threonine-protein kinase TAO1 OS=Homo sapiens GN=TAOK1 PE=1 SV=1 - [TAOK1_HUMAN]
Q969G3	SWI/SNF-related matrix-associated actin-dependent regulator of chromatin subfamily E member 1 OS=Homo sapiens GN=SMARCE1 PE=1 SV=2 - [SMCE1_HUMAN]
P31751	RAC-beta serine/threonine-protein kinase OS=Homo sapiens GN=AKT2 PE=1 SV=2 - [AKT2_HUMAN]
Q9Y2X7	ARF GTPase-activating protein GIT1 OS=Homo sapiens GN=GIT1 PE=1 SV=2 - [GIT1_HUMAN]
Q6AI12	Ankyrin repeat domain-containing protein 40 OS=Homo sapiens GN=ANKRD40 PE=1 SV=2 - [ANR40_HUMAN]
Q8TCX1	Cytoplasmic dynein 2 light intermediate chain 1 OS=Homo sapiens GN=DYNC2LI1 PE=1 SV=1 - [DC2L1_HUMAN]
Q8TCY9	Up-regulator of cell proliferation OS=Homo sapiens GN=URGCP PE=1 SV=2 - [URGCP_HUMAN]
Q9NVP2	Histone chaperone ASF1B OS=Homo sapiens GN=ASF1B PE=1 SV=1 - [ASF1B_HUMAN]
Q8NCE2	Myotubularin-related protein 14 OS=Homo sapiens GN=MTMR14 PE=1 SV=2 - [MTMRE_HUMAN]
Q9UBX5	Fibulin-5 OS=Homo sapiens GN=FBLN5 PE=1 SV=1 - [FBLN5_HUMAN]
O75190	DnaJ homolog subfamily B member 6 OS=Homo sapiens GN=DNAJB6 PE=1 SV=2 - [DNJB6_HUMAN]
P01591	Immunoglobulin J chain OS=Homo sapiens GN=IGJ PE=1 SV=4 - [IGJ_HUMAN]
O43156	TELO2-interacting protein 1 homolog OS=Homo sapiens GN=TTI1 PE=1 SV=3 - [TTI1_HUMAN]
O75400	Pre-mRNA-processing factor 40 homolog A OS=Homo sapiens GN=PRPF40A PE=1 SV=2 - [PR40A_HUMAN]
Q00536	Cyclin-dependent kinase 16 OS=Homo sapiens GN=CDK16 PE=1 SV=1 - [CDK16_HUMAN]
Q9H4H8	Protein FAM83D OS=Homo sapiens GN=FAM83D PE=1 SV=3 - [FA83D_HUMAN]
Q96DA2	Ras-related protein Rab-39B OS=Homo sapiens GN=RAB39B PE=1 SV=1 - [RB39B_HUMAN]
Q9H8M7	Protein FAM188A OS=Homo sapiens GN=FAM188A PE=1 SV=1 - [F188A_HUMAN]
Q9UQ88	Cyclin-dependent kinase 11A OS=Homo sapiens GN=CDK11A PE=1 SV=4 - [CD11A_HUMAN]
O60333	Kinesin-like protein KIF1B OS=Homo sapiens GN=KIF1B PE=1 SV=5 - [KIF1B_HUMAN]
Q63HN8	E3 ubiquitin-protein ligase RNF213 OS=Homo sapiens GN=RNF213 PE=1 SV=3 - [RN213_HUMAN]
Q9ULR0	Pre-mRNA-splicing factor ISY1 homolog OS=Homo sapiens GN=ISY1 PE=1 SV=3 - [ISY1_HUMAN]
Q9Y520	Protein PRRC2C OS=Homo sapiens GN=PRRC2C PE=1 SV=4 - [PRC2C_HUMAN]
Q9NQ50	39S ribosomal protein L40, mitochondrial OS=Homo sapiens GN=MRPL40 PE=1 SV=1 - [RM40_HUMAN]
P55327	Tumor protein D52 OS=Homo sapiens GN=TPD52 PE=1 SV=2 - [TPD52_HUMAN]
Q09666	Neuroblast differentiation-associated protein AHNAK OS=Homo sapiens GN=AHNAK PE=1 SV=2 - [AHNK_HUMAN]
P10253	Lysosomal alpha-glucosidase OS=Homo sapiens GN=GAA PE=1 SV=4 - [LYAG_HUMAN]
P20020	Plasma membrane calcium-transporting ATPase 1 OS=Homo sapiens GN=ATP2B1 PE=1 SV=3 - [AT2B1_HUMAN]
Q9NQ84	G-protein coupled receptor family C group 5 member C OS=Homo sapiens GN=GPRC5C PE=1 SV=2 - [GPC5C_HUMAN]
Q9H1I8	Activating signal cointegrator 1 complex subunit 2 OS=Homo sapiens GN=ASCC2 PE=1 SV=3 - [ASCC2_HUMAN]
Q00403	Transcription initiation factor IIB OS=Homo sapiens GN=GTF2B PE=1 SV=1 - [TF2B_HUMAN]
Q08379	Golgin subfamily A member 2 OS=Homo sapiens GN=GOLGA2 PE=1 SV=3 - [GOGA2_HUMAN]
P21127	Cyclin-dependent kinase 11B OS=Homo sapiens GN=CDK11B PE=1 SV=3 - [CD11B_HUMAN]
P21980	Protein-glutamine gamma-glutamyltransferase 2 OS=Homo sapiens GN=TGM2 PE=1 SV=2 - [TGM2_HUMAN]
Q9HDC9	Adipocyte plasma membrane-associated protein OS=Homo sapiens GN=APMAP PE=1 SV=2 - [APMAP_HUMAN]
P23634	Plasma membrane calcium-transporting ATPase 4 OS=Homo sapiens GN=ATP2B4 PE=1 SV=2 - [AT2B4_HUMAN]
Q8N3Y1	F-box/WD repeat-containing protein 8 OS=Homo sapiens GN=FBXW8 PE=1 SV=2 - [FBXW8_HUMAN]
P36871	Phosphoglucomutase-1 OS=Homo sapiens GN=PGM1 PE=1 SV=3 - [PGM1_HUMAN]
Q15375	Ephrin type-A receptor 7 OS=Homo sapiens GN=EPHA7 PE=1 SV=3 - [EPHA7_HUMAN]
Q8IWF6	Protein DENND6A OS=Homo sapiens GN=DENND6A PE=1 SV=1 - [DEN6A_HUMAN]
Q96BY7	Autophagy-related protein 2 homolog B OS=Homo sapiens GN=ATG2B PE=1 SV=5 - [ATG2B_HUMAN]
Q96EK9	Protein KTI12 homolog OS=Homo sapiens GN=KTI12 PE=1 SV=1 - [KTI12_HUMAN]
P31323	cAMP-dependent protein kinase type II-beta regulatory subunit OS=Homo sapiens GN=PRKAR2B PE=1 SV=3 - [KAP3_HUMAN]
Q14527	Helicase-like transcription factor OS=Homo sapiens GN=HLTF PE=1 SV=2 - [HLTF_HUMAN]
P42356	Phosphatidylinositol 4-kinase alpha OS=Homo sapiens GN=PI4KA PE=1 SV=3 - [PI4KA_HUMAN]

Q9UKE5	TRAF2 and NCK-interacting protein kinase OS=Homo sapiens GN=TNIK PE=1 SV=1 - [TNIK_HUMAN]
Q71H61	Immunoglobulin-like domain-containing receptor 2 OS=Homo sapiens GN=ILDR2 PE=2 SV=1 - [ILDR2_HUMAN]
Q9UHW5	GPN-loop GTPase 3 OS=Homo sapiens GN=GPN3 PE=1 SV=2 - [GPN3_HUMAN]
Q9P2K3	REST corepressor 3 OS=Homo sapiens GN=RCOR3 PE=1 SV=2 - [RCOR3_HUMAN]
Q53HC5	Kelch-like protein 26 OS=Homo sapiens GN=KLHL26 PE=1 SV=2 - [KLH26_HUMAN]
P61601	Neurocalcin-delta OS=Homo sapiens GN=NCALD PE=1 SV=2 - [NCALD_HUMAN]
Q5VIR6	Vacuolar protein sorting-associated protein 53 homolog OS=Homo sapiens GN=VPS53 PE=1 SV=1 - [VPS53_HUMAN]
P25440	Bromodomain-containing protein 2 OS=Homo sapiens GN=BRD2 PE=1 SV=2 - [BRD2_HUMAN]
P49815	Tuberin OS=Homo sapiens GN=TSC2 PE=1 SV=2 - [TSC2_HUMAN]
Q8IXH7	Negative elongation factor C/D OS=Homo sapiens GN=NELFCD PE=1 SV=2 - [NELFD_HUMAN]
Q9BZE9	Tether containing UBX domain for GLUT4 OS=Homo sapiens GN=ASPSCR1 PE=1 SV=1 - [ASPC1_HUMAN]
Q12965	Unconventional myosin-Ie OS=Homo sapiens GN=MYO1E PE=1 SV=2 - [MYO1E_HUMAN]
Q9Y3C0	WASH complex subunit CCDC53 OS=Homo sapiens GN=CCDC53 PE=1 SV=1 - [CCD53_HUMAN]
P07711	Cathepsin L1 OS=Homo sapiens GN=CTSL PE=1 SV=2 - [CATL1_HUMAN]
Q86WJ1	Chromodomain-helicase-DNA-binding protein 1-like OS=Homo sapiens GN=CHD1L PE=1 SV=2 - [CHD1L_HUMAN]
Q9H5Z1	Probable ATP-dependent RNA helicase DHX35 OS=Homo sapiens GN=DHX35 PE=1 SV=2 - [DHX35_HUMAN]
P54803	Galactocerebrosidase OS=Homo sapiens GN=GALC PE=1 SV=2 - [GALC_HUMAN]
Q15057	Arf-GAP with coiled-coil, ANK repeat and PH domain-containing protein 2 OS=Homo sapiens GN=ACAP2 PE=1 SV=3 - [ACAP2_HUMAN]
Q32MZ4	Leucine-rich repeat flightless-interacting protein 1 OS=Homo sapiens GN=LRRFIP1 PE=1 SV=2 - [LRRF1_HUMAN]
Q96DE0	U8 snoRNA-decapping enzyme OS=Homo sapiens GN=NUDT16 PE=1 SV=2 - [NUD16_HUMAN]
Q86UE8	Serine/threonine-protein kinase tousled-like 2 OS=Homo sapiens GN=TLK2 PE=1 SV=2 - [TLK2_HUMAN]
Q9BWT6	Meiotic nuclear division protein 1 homolog OS=Homo sapiens GN=MND1 PE=1 SV=1 - [MND1_HUMAN]
Q9UH17	DNA dC->dU-editing enzyme APOBEC-3B OS=Homo sapiens GN=APOBEC3B PE=1 SV=1 - [ABC3B_HUMAN]
Q16512	Serine/threonine-protein kinase N1 OS=Homo sapiens GN=PKN1 PE=1 SV=2 - [PKN1_HUMAN]
P30622	CAP-Gly domain-containing linker protein 1 OS=Homo sapiens GN=CLIP1 PE=1 SV=2 - [CLIP1_HUMAN]
Q12841	Follistatin-related protein 1 OS=Homo sapiens GN=FSTL1 PE=1 SV=1 - [FSTL1_HUMAN]
Q14689	Disco-interacting protein 2 homolog A OS=Homo sapiens GN=DIP2A PE=1 SV=2 - [DIP2A_HUMAN]
P29320	Ephrin type-A receptor 3 OS=Homo sapiens GN=EPHA3 PE=1 SV=2 - [EPHA3_HUMAN]
P62068	Ubiquitin carboxyl-terminal hydrolase 46 OS=Homo sapiens GN=USP46 PE=1 SV=1 - [UBP46_HUMAN]
Q6N069	N-alpha-acetyltransferase 16, NatA auxiliary subunit OS=Homo sapiens GN=NAA16 PE=1 SV=2 - [NAA16_HUMAN]
Q9BX40	Protein LSM14 homolog B OS=Homo sapiens GN=LSM14B PE=1 SV=1 - [LS14B_HUMAN]
O95445	Apolipoprotein M OS=Homo sapiens GN=APOM PE=1 SV=2 - [APOM_HUMAN]
Q7KYR7	Butyrophilin subfamily 2 member A1 OS=Homo sapiens GN=BTN2A1 PE=1 SV=3 - [BT2A1_HUMAN]
Q6UN15	Pre-mRNA 3'-end-processing factor FIP1 OS=Homo sapiens GN=FIP1L1 PE=1 SV=1 - [FIP1_HUMAN]
Q7Z3C6	Autophagy-related protein 9A OS=Homo sapiens GN=ATG9A PE=1 SV=3 - [ATG9A_HUMAN]
Q96LT7	Protein C9orf72 OS=Homo sapiens GN=C9orf72 PE=1 SV=2 - [CI072_HUMAN]
Q07889	Son of sevenless homolog 1 OS=Homo sapiens GN=SOS1 PE=1 SV=1 - [SOS1_HUMAN]
Q16875	6-phosphofructo-2-kinase/fructose-2,6-bisphosphatase 3 OS=Homo sapiens GN=PFKFB3 PE=1 SV=1 - [F263_HUMAN]
Q8WUH2	Transforming growth factor-beta receptor-associated protein 1 OS=Homo sapiens GN=TGFBRAP1 PE=1 SV=1 - [TGFA1_HUMAN]
P08603	Complement factor H OS=Homo sapiens GN=CFH PE=1 SV=4 - [CFAH_HUMAN]
Q13428	Treacle protein OS=Homo sapiens GN=TCOF1 PE=1 SV=3 - [TCOF_HUMAN]
Q99717	Mothers against decapentaplegic homolog 5 OS=Homo sapiens GN=SMAD5 PE=1 SV=1 - [SMAD5_HUMAN]
P49321	Nuclear autoantigenic sperm protein OS=Homo sapiens GN=NASP PE=1 SV=2 - [NASP_HUMAN]
Q86XA9	HEAT repeat-containing protein 5A OS=Homo sapiens GN=HEATR5A PE=1 SV=2 - [HTR5A_HUMAN]
Q14671	Pumilio homolog 1 OS=Homo sapiens GN=PUM1 PE=1 SV=3 - [PUM1_HUMAN]
Q9UG01	Intraflagellar transport protein 172 homolog OS=Homo sapiens GN=IFT172 PE=1 SV=2 - [IF172_HUMAN]
P02461	Collagen alpha-1(III) chain OS=Homo sapiens GN=COL3A1 PE=1 SV=4 - [CO3A1_HUMAN]
Q9UK39	Nocturnin OS=Homo sapiens GN=CCRN4L PE=2 SV=2 - [NOCT_HUMAN]
Q69YN4	Protein virilizer homolog OS=Homo sapiens GN=KIAA1429 PE=1 SV=2 - [VIR_HUMAN]
Q96DX4	RING finger and SPRY domain-containing protein 1 OS=Homo sapiens GN=RSPRY1 PE=1 SV=1 - [RSPRY_HUMAN]
Q9P2D0	Inhibitor of Bruton tyrosine kinase OS=Homo sapiens GN=IBTK PE=1 SV=3 - [IBTK_HUMAN]
Q01130	Serine/arginine-rich splicing factor 2 OS=Homo sapiens GN=SRSF2 PE=1 SV=4 - [SRSF2_HUMAN]
Q92696	Geranylgeranyl transferase type-2 subunit alpha OS=Homo sapiens GN=RABGGTA PE=1 SV=2 - [PGTA_HUMAN]
P13611	Versican core protein OS=Homo sapiens GN=VCAN PE=1 SV=3 - [CSPG2_HUMAN]
P00352	Retinal dehydrogenase 1 OS=Homo sapiens GN=ALDH1A1 PE=1 SV=2 - [AL1A1_HUMAN]
Q69YQ0	Cytospin-A OS=Homo sapiens GN=SPECC1L PE=1 SV=2 - [CYTSA_HUMAN]
Q9BZG8	Diphthamide biosynthesis protein 1 OS=Homo sapiens GN=DPH1 PE=1 SV=2 - [DPH1_HUMAN]
Q9UHG2	ProSAAS OS=Homo sapiens GN=PCSK1N PE=1 SV=1 - [PCSK1_HUMAN]
Q8TBZ3	WD repeat-containing protein 20 OS=Homo sapiens GN=WDR20 PE=1 SV=2 - [WDR20_HUMAN]
Q9H4L5	Oxysterol-binding protein-related protein 3 OS=Homo sapiens GN=OSBPL3 PE=1 SV=1 - [OSBL3_HUMAN]
P11498	Pyruvate carboxylase, mitochondrial OS=Homo sapiens GN=PC PE=1 SV=2 - [PYC_HUMAN]
O14966	Ras-related protein Rab-7L1 OS=Homo sapiens GN=RAB29 PE=1 SV=1 - [RAB7L_HUMAN]
P19525	Interferon-induced, double-stranded RNA-activated protein kinase OS=Homo sapiens GN=EIF2AK2 PE=1 SV=2 - [E2AK2_HUMAN]
P11217	Glycogen phosphorylase, muscle form OS=Homo sapiens GN=PYGM PE=1 SV=6 - [PYGM_HUMAN]
Q9H1K0	Rabenosyn-5 OS=Homo sapiens GN=RBSN PE=1 SV=2 - [RBNS5_HUMAN]
A6NHL2	Tubulin alpha chain-like 3 OS=Homo sapiens GN=TUBAL3 PE=1 SV=2 - [TBAL3_HUMAN]
Q14653	Interferon regulatory factor 3 OS=Homo sapiens GN=IRF3 PE=1 SV=1 - [IRF3_HUMAN]
Q92804	TATA-binding protein-associated factor 2N OS=Homo sapiens GN=TAF15 PE=1 SV=1 - [RBP56_HUMAN]
P23443	Ribosomal protein S6 kinase beta-1 OS=Homo sapiens GN=RPS6KB1 PE=1 SV=2 - [KS6B1_HUMAN]
P61764	Syntaxin-binding protein 1 OS=Homo sapiens GN=STXBP1 PE=1 SV=1 - [STXB1_HUMAN]
Q9Y4G6	Talin-2 OS=Homo sapiens GN=TLN2 PE=1 SV=4 - [TLN2_HUMAN]
Q9Y6M5	Zinc transporter 1 OS=Homo sapiens GN=SLC30A1 PE=1 SV=3 - [ZNT1_HUMAN]
O96008	Mitochondrial import receptor subunit TOM40 homolog OS=Homo sapiens GN=TOMM40 PE=1 SV=1 - [TOM40_HUMAN]
Q15542	Transcription initiation factor TFIID subunit 5 OS=Homo sapiens GN=TAF5 PE=1 SV=3 - [TAF5_HUMAN]
Q6IQ22	Ras-related protein Rab-12 OS=Homo sapiens GN=RAB12 PE=1 SV=3 - [RAB12_HUMAN]
Q92979	Ribosomal RNA small subunit methyltransferase NEP1 OS=Homo sapiens GN=EMG1 PE=1 SV=4 - [NEP1_HUMAN]
P36776	Lon protease homolog, mitochondrial OS=Homo sapiens GN=LONP1 PE=1 SV=2 - [LONM_HUMAN]
O15031	Plexin-B2 OS=Homo sapiens GN=PLXNB2 PE=1 SV=3 - [PLXB2_HUMAN]
P14384	Carboxypeptidase M OS=Homo sapiens GN=CPM PE=1 SV=2 - [CBPM_HUMAN]
Q6DKI1	60S ribosomal protein L7-like 1 OS=Homo sapiens GN=RPL7L1 PE=1 SV=1 - [RL7L_HUMAN]
O95251	Histone acetyltransferase KAT7 OS=Homo sapiens GN=KAT7 PE=1 SV=1 - [KAT7_HUMAN]
Q9Y3I1	F-box only protein 7 OS=Homo sapiens GN=FBXO7 PE=1 SV=1 - [FBX7_HUMAN]
Q14674	Separin OS=Homo sapiens GN=ESPL1 PE=1 SV=3 - [ESPL1_HUMAN]
P18887	DNA repair protein XRCC1 OS=Homo sapiens GN=XRCC1 PE=1 SV=2 - [XRCC1_HUMAN]
Q5JTH9	RRP12-like protein OS=Homo sapiens GN=RRP12 PE=1 SV=2 - [RRP12_HUMAN]
P78362	SRSF protein kinase 2 OS=Homo sapiens GN=SRPK2 PE=1 SV=3 - [SRPK2_HUMAN]
Q5UIP0	Telomere-associated protein RIF1 OS=Homo sapiens GN=RIF1 PE=1 SV=2 - [RIF1_HUMAN]
Q8IYD1	Eukaryotic peptide chain release factor GTP-binding subunit ERF3B OS=Homo sapiens GN=GSPT2 PE=1 SV=2 - [ERF3B_HUMAN]
Q7LGC8	Carbohydrate sulfotransferase 3 OS=Homo sapiens GN=CHST3 PE=1 SV=3 - [CHST3_HUMAN]
O60245	Protocadherin-7 OS=Homo sapiens GN=PCDH7 PE=1 SV=2 - [PCDH7_HUMAN]
P53801	Pituitary tumor-transforming gene 1 protein-interacting protein OS=Homo sapiens GN=PTTG1IP PE=1 SV=1 - [PTTG_HUMAN]
Q13432	Protein unc-119 homolog A OS=Homo sapiens GN=UNC119 PE=1 SV=1 - [U119A_HUMAN]
O94830	Phospholipase DDHD2 OS=Homo sapiens GN=DDHD2 PE=1 SV=2 - [DDHD2_HUMAN]
Q13601	KRR1 small subunit processome component homolog OS=Homo sapiens GN=KRR1 PE=1 SV=4 - [KRR1_HUMAN]
O75330	Hyaluronan mediated motility receptor OS=Homo sapiens GN=HMMR PE=1 SV=2 - [HMMR_HUMAN]
Q9H981	Actin-related protein 8 OS=Homo sapiens GN=ACTR8 PE=1 SV=2 - [ARP8_HUMAN]
P08581	Hepatocyte growth factor receptor OS=Homo sapiens GN=MET PE=1 SV=4 - [MET_HUMAN]
Q14165	Malectin OS=Homo sapiens GN=MLEC PE=1 SV=1 - [MLEC_HUMAN]
P48029	Sodium- and chloride-dependent creatine transporter 1 OS=Homo sapiens GN=SLC6A8 PE=1 SV=1 - [SC6A8_HUMAN]
Q92859	Neogenin OS=Homo sapiens GN=NEO1 PE=1 SV=2 - [NEO1_HUMAN]
P46020	Phosphorylase b kinase regulatory subunit alpha, skeletal muscle isoform OS=Homo sapiens GN=PHKA1 PE=1 SV=2 - [KPB1_HUMAN]
Q99519	Sialidase-1 OS=Homo sapiens GN=NEU1 PE=1 SV=1 - [NEUR1_HUMAN]
Q6ZMI0	Protein phosphatase 1 regulatory subunit 21 OS=Homo sapiens GN=PPP1R21 PE=1 SV=1 - [PPR21_HUMAN]
Q9H0M0	NEDD4-like E3 ubiquitin-protein ligase WWP1 OS=Homo sapiens GN=WWP1 PE=1 SV=1 - [WWP1_HUMAN]
Q15637	Splicing factor 1 OS=Homo sapiens GN=SF1 PE=1 SV=4 - [SF01_HUMAN]
Q9UII4	E3 ISG15--protein ligase HERC5 OS=Homo sapiens GN=HERC5 PE=1 SV=2 - [HERC5_HUMAN]
Q9NZQ3	NCK-interacting protein with SH3 domain OS=Homo sapiens GN=NCKIPSD PE=1 SV=1 - [SPN90_HUMAN]
Q5PRF9	Protein Smaug homolog 2 OS=Homo sapiens GN=SAMD4B PE=1 SV=1 - [SMAG2_HUMAN]
Q96BD8	Spindle and kinetochore-associated protein 1 OS=Homo sapiens GN=SKA1 PE=1 SV=1 - [SKA1_HUMAN]
Q9C0B5	Palmitoyltransferase ZDHHC5 OS=Homo sapiens GN=ZDHHC5 PE=1 SV=2 - [ZDHC5_HUMAN]
Q86YR5	G-protein-signaling modulator 1 OS=Homo sapiens GN=GPSM1 PE=1 SV=2 - [GPSM1_HUMAN]
Q8IWR0	Zinc finger CCCH domain-containing protein 7A OS=Homo sapiens GN=ZC3H7A PE=1 SV=1 - [Z3H7A_HUMAN]
Q9P016	Thymocyte nuclear protein 1 OS=Homo sapiens GN=THYN1 PE=1 SV=1 - [THYN1_HUMAN]
O94889	Kelch-like protein 18 OS=Homo sapiens GN=KLHL18 PE=1 SV=3 - [KLH18_HUMAN]
Q9ULF5	Zinc transporter ZIP10 OS=Homo sapiens GN=SLC39A10 PE=1 SV=2 - [S39AA_HUMAN]

P11310	Medium-chain specific acyl-CoA dehydrogenase, mitochondrial OS=Homo sapiens GN=ACADM PE=1 SV=1 - [ACADM_HUMAN]
O75582	Ribosomal protein S6 kinase alpha-5 OS=Homo sapiens GN=RPS6KA5 PE=1 SV=1 - [KS6A5_HUMAN]
O95793	Double-stranded RNA-binding protein Staufen homolog 1 OS=Homo sapiens GN=STAU1 PE=1 SV=2 - [STAU1_HUMAN]
Q92620	Pre-mRNA-splicing factor ATP-dependent RNA helicase PRP16 OS=Homo sapiens GN=DHX38 PE=1 SV=2 - [PRP16_HUMAN]
Q68D85	Natural cytotoxicity triggering receptor 3 ligand 1 OS=Homo sapiens GN=NCR3LG1 PE=1 SV=1 - [NR3L1_HUMAN]
P06241	Tyrosine-protein kinase Fyn OS=Homo sapiens GN=FYN PE=1 SV=3 - [FYN_HUMAN]
O43347	RNA-binding protein Musashi homolog 1 OS=Homo sapiens GN=MSI1 PE=1 SV=1 - [MSI1H_HUMAN]
Q8IYT4	Katanin p60 ATPase-containing subunit A-like 2 OS=Homo sapiens GN=KATNAL2 PE=2 SV=3 - [KATL2_HUMAN]
P54762	Ephrin type-B receptor 1 OS=Homo sapiens GN=EPHB1 PE=1 SV=1 - [EPHB1_HUMAN]
Q8IZA0	Dyslexia-associated protein KIAA0319-like protein OS=Homo sapiens GN=KIAA0319L PE=1 SV=2 - [K319L_HUMAN]
Q5T9L3	Protein wntless homolog OS=Homo sapiens GN=WLS PE=1 SV=2 - [WLS_HUMAN]
O00628	Peroxisomal targeting signal 2 receptor OS=Homo sapiens GN=PEX7 PE=1 SV=1 - [PEX7_HUMAN]
Q4G0M1	Erythroferrone OS=Homo sapiens GN=FAM132B PE=2 SV=2 - [ERFE_HUMAN]
Q8TBF2	Prostamide/prostaglandin F synthase OS=Homo sapiens GN=FAM213B PE=2 SV=1 - [PGFS_HUMAN]
Q9NPQ8	Synembryn-A OS=Homo sapiens GN=RIC8A PE=1 SV=3 - [RIC8A_HUMAN]
P28370	Probable global transcription activator SNF2L1 OS=Homo sapiens GN=SMARCA1 PE=1 SV=2 - [SMCA1_HUMAN]
P58004	Sestrin-2 OS=Homo sapiens GN=SESN2 PE=1 SV=1 - [SESN2_HUMAN]
Q8N4N3	Kelch-like protein 36 OS=Homo sapiens GN=KLHL36 PE=1 SV=1 - [KLH36_HUMAN]
O75915	PRA1 family protein 3 OS=Homo sapiens GN=ARL6IP5 PE=1 SV=1 - [PRAF3_HUMAN]
Q9NRA1	Platelet-derived growth factor C OS=Homo sapiens GN=PDGFC PE=1 SV=2 - [PDGFC_HUMAN]
Q9P2E9	Ribosome-binding protein 1 OS=Homo sapiens GN=RRBP1 PE=1 SV=4 - [RRBP1_HUMAN]
P55212	Caspase-6 OS=Homo sapiens GN=CASP6 PE=1 SV=2 - [CASP6_HUMAN]
Q00537	Cyclin-dependent kinase 17 OS=Homo sapiens GN=CDK17 PE=1 SV=2 - [CDK17_HUMAN]
Q93050	V-type proton ATPase 116 kDa subunit a isoform 1 OS=Homo sapiens GN=ATP6V0A1 PE=1 SV=3 - [VPP1_HUMAN]
Q05519	Serine/arginine-rich splicing factor 11 OS=Homo sapiens GN=SRSF11 PE=1 SV=1 - [SRS11_HUMAN]
O00443	Phosphatidylinositol 4-phosphate 3-kinase C2 domain-containing subunit alpha OS=Homo sapiens GN=PIK3C2A PE=1 SV=2 - [P3C2A_HUMAN]
Q15334	Lethal(2) giant larvae protein homolog 1 OS=Homo sapiens GN=LLGL1 PE=1 SV=3 - [L2GL1_HUMAN]
Q9Y3T9	Nucleolar complex protein 2 homolog OS=Homo sapiens GN=NOC2L PE=1 SV=4 - [NOC2L_HUMAN]
P50552	Vasodilator-stimulated phosphoprotein OS=Homo sapiens GN=VASP PE=1 SV=3 - [VASP_HUMAN]
Q6NUQ1	RAD50-interacting protein 1 OS=Homo sapiens GN=RINT1 PE=1 SV=1 - [RINT1_HUMAN]
Q08752	Peptidyl-prolyl cis-trans isomerase D OS=Homo sapiens GN=PPID PE=1 SV=3 - [PPID_HUMAN]
Q96HW7	Integrator complex subunit 4 OS=Homo sapiens GN=INTS4 PE=1 SV=2 - [INT4_HUMAN]
Q99985	Semaphorin-3C OS=Homo sapiens GN=SEMA3C PE=2 SV=2 - [SEM3C_HUMAN]
Q9NRB3	Carbohydrate sulfotransferase 12 OS=Homo sapiens GN=CHST12 PE=1 SV=2 - [CHSTC_HUMAN]
Q9NZU0	Leucine-rich repeat transmembrane protein FLRT3 OS=Homo sapiens GN=FLRT3 PE=1 SV=1 - [FLRT3_HUMAN]
P30825	High affinity cationic amino acid transporter 1 OS=Homo sapiens GN=SLC7A1 PE=1 SV=1 - [CTR1_HUMAN]
Q96LI5	CCR4-NOT transcription complex subunit 6-like OS=Homo sapiens GN=CNOT6L PE=1 SV=2 - [CNO6L_HUMAN]
Q99700	Ataxin-2 OS=Homo sapiens GN=ATXN2 PE=1 SV=2 - [ATX2_HUMAN]
P46821	Microtubule-associated protein 1B OS=Homo sapiens GN=MAP1B PE=1 SV=2 - [MAP1B_HUMAN]
P52948	Nuclear pore complex protein Nup98-Nup96 OS=Homo sapiens GN=NUP98 PE=1 SV=4 - [NUP98_HUMAN]
Q9NYF8	Bcl-2-associated transcription factor 1 OS=Homo sapiens GN=BCLAF1 PE=1 SV=2 - [BCLF1_HUMAN]
Q92729	Receptor-type tyrosine-protein phosphatase U OS=Homo sapiens GN=PTPRU PE=1 SV=2 - [PTPRU_HUMAN]
P46934	E3 ubiquitin-protein ligase NEDD4 OS=Homo sapiens GN=NEDD4 PE=1 SV=4 - [NEDD4_HUMAN]
Q7Z5K2	Wings apart-like protein homolog OS=Homo sapiens GN=WAPAL PE=1 SV=1 - [WAPL_HUMAN]
Q96JH7	Deubiquitinating protein VCIP135 OS=Homo sapiens GN=VCPIP1 PE=1 SV=2 - [VCIP1_HUMAN]
Q9UI26	Importin-11 OS=Homo sapiens GN=IPO11 PE=1 SV=1 - [IPO11_HUMAN]
Q9Y3A3	MOB-like protein phocein OS=Homo sapiens GN=MOB4 PE=1 SV=1 - [PHOCN_HUMAN]
Q13228	Selenium-binding protein 1 OS=Homo sapiens GN=SELENBP1 PE=1 SV=2 - [SBP1_HUMAN]
P06400	Retinoblastoma-associated protein OS=Homo sapiens GN=RB1 PE=1 SV=2 - [RB_HUMAN]
P22681	E3 ubiquitin-protein ligase CBL OS=Homo sapiens GN=CBL PE=1 SV=2 - [CBL_HUMAN]
Q5T6F0	DDB1- and CUL4-associated factor 12 OS=Homo sapiens GN=DCAF12 PE=1 SV=1 - [DCA12_HUMAN]
O75179	Ankyrin repeat domain-containing protein 17 OS=Homo sapiens GN=ANKRD17 PE=1 SV=3 - [ANR17_HUMAN]
Q8TDY2	RB1-inducible coiled-coil protein 1 OS=Homo sapiens GN=RB1CC1 PE=1 SV=3 - [RBCC1_HUMAN]
Q6P2H3	Centrosomal protein of 85 kDa OS=Homo sapiens GN=CEP85 PE=1 SV=1 - [CEP85_HUMAN]
Q9BTU6	Phosphatidylinositol 4-kinase type 2-alpha OS=Homo sapiens GN=PI4K2A PE=1 SV=1 - [P4K2A_HUMAN]
Q9H7Z3	Protein NRDE2 homolog OS=Homo sapiens GN=NRDE2 PE=1 SV=3 - [NRDE2_HUMAN]
Q8TE02	Elongator complex protein 5 OS=Homo sapiens GN=ELP5 PE=1 SV=2 - [ELP5_HUMAN]
P07225	Vitamin K-dependent protein S OS=Homo sapiens GN=PROS1 PE=1 SV=1 - [PROS_HUMAN]
Q6P1X5	Transcription initiation factor TFIID subunit 2 OS=Homo sapiens GN=TAF2 PE=1 SV=3 - [TAF2_HUMAN]
Q92995	Ubiquitin carboxyl-terminal hydrolase 13 OS=Homo sapiens GN=USP13 PE=1 SV=2 - [UBP13_HUMAN]
Q9Y2E4	Disco-interacting protein 2 homolog C OS=Homo sapiens GN=DIP2C PE=1 SV=2 - [DIP2C_HUMAN]
Q86W56	Poly(ADP-ribose) glycohydrolase OS=Homo sapiens GN=PARG PE=1 SV=1 - [PARG_HUMAN]
P36896	Activin receptor type-1B OS=Homo sapiens GN=ACVR1B PE=1 SV=1 - [ACV1B_HUMAN]
O60637	Tetraspanin-3 OS=Homo sapiens GN=TSPAN3 PE=2 SV=1 - [TSN3_HUMAN]
Q8WU79	Stromal membrane-associated protein 2 OS=Homo sapiens GN=SMAP2 PE=1 SV=1 - [SMAP2_HUMAN]
O00423	Echinoderm microtubule-associated protein-like 1 OS=Homo sapiens GN=EML1 PE=1 SV=3 - [EMAL1_HUMAN]
Q9UPR3	Protein SMG5 OS=Homo sapiens GN=SMG5 PE=1 SV=3 - [SMG5_HUMAN]
P51784	Ubiquitin carboxyl-terminal hydrolase 11 OS=Homo sapiens GN=USP11 PE=1 SV=3 - [UBP11_HUMAN]
Q9UNW1	Multiple inositol polyphosphate phosphatase 1 OS=Homo sapiens GN=MINPP1 PE=1 SV=1 - [MINP1_HUMAN]
Q96JK2	DDB1- and CUL4-associated factor 5 OS=Homo sapiens GN=DCAF5 PE=1 SV=2 - [DCAF5_HUMAN]
Q9Y6N7	Roundabout homolog 1 OS=Homo sapiens GN=ROBO1 PE=1 SV=1 - [ROBO1_HUMAN]
P18031	Tyrosine-protein phosphatase non-receptor type 1 OS=Homo sapiens GN=PTPN1 PE=1 SV=1 - [PTN1_HUMAN]
Q9UHY1	Nuclear receptor-binding protein OS=Homo sapiens GN=NRBP1 PE=1 SV=1 - [NRBP_HUMAN]
O15234	Protein CASC3 OS=Homo sapiens GN=CASC3 PE=1 SV=2 - [CASC3_HUMAN]
Q7L523	Ras-related GTP-binding protein A OS=Homo sapiens GN=RRAGA PE=1 SV=1 - [RRAGA_HUMAN]
Q96K76	Ubiquitin carboxyl-terminal hydrolase 47 OS=Homo sapiens GN=USP47 PE=1 SV=3 - [UBP47_HUMAN]
P46977	Dolichyl-diphosphooligosaccharide--protein glycosyltransferase subunit STT3A OS=Homo sapiens GN=STT3A PE=1 SV=2 - [STT3A_HUMAN]
O60941	Dystrobrevin beta OS=Homo sapiens GN=DTNB PE=1 SV=1 - [DTNB_HUMAN]
Q7LBC6	Lysine-specific demethylase 3B OS=Homo sapiens GN=KDM3B PE=1 SV=2 - [KDM3B_HUMAN]
Q8IWV7	E3 ubiquitin-protein ligase UBR1 OS=Homo sapiens GN=UBR1 PE=1 SV=1 - [UBR1_HUMAN]
O43301	Heat shock 70 kDa protein 12A OS=Homo sapiens GN=HSPA12A PE=1 SV=2 - [HS12A_HUMAN]
O15360	Fanconi anemia group A protein OS=Homo sapiens GN=FANCA PE=1 SV=2 - [FANCA_HUMAN]
Q8WXE1	ATR-interacting protein OS=Homo sapiens GN=ATRIP PE=1 SV=1 - [ATRIP_HUMAN]
Q9UKA9	Polypyrimidine tract-binding protein 2 OS=Homo sapiens GN=PTBP2 PE=1 SV=1 - [PTBP2_HUMAN]
Q05397	Focal adhesion kinase 1 OS=Homo sapiens GN=PTK2 PE=1 SV=2 - [FAK1_HUMAN]
Q08209	Serine/threonine-protein phosphatase 2B catalytic subunit alpha isoform OS=Homo sapiens GN=PPP3CA PE=1 SV=1 - [PP2BA_HUMAN]
Q8N3P4	Vacuolar protein sorting-associated protein 8 homolog OS=Homo sapiens GN=VPS8 PE=1 SV=3 - [VPS8_HUMAN]
O00750	Phosphatidylinositol 4-phosphate 3-kinase C2 domain-containing subunit beta OS=Homo sapiens GN=PIK3C2B PE=1 SV=2 - [P3C2B_HUMAN]
O00499	Myc box-dependent-interacting protein 1 OS=Homo sapiens GN=BIN1 PE=1 SV=1 - [BIN1_HUMAN]
P46531	Neurogenic locus notch homolog protein 1 OS=Homo sapiens GN=NOTCH1 PE=1 SV=4 - [NOTC1_HUMAN]
Q12800	Alpha-globin transcription factor CP2 OS=Homo sapiens GN=TFCP2 PE=1 SV=2 - [TFCP2_HUMAN]
Q9H832	Ubiquitin-conjugating enzyme E2 Z OS=Homo sapiens GN=UBE2Z PE=1 SV=2 - [UBE2Z_HUMAN]
P11166	Solute carrier family 2, facilitated glucose transporter member 1 OS=Homo sapiens GN=SLC2A1 PE=1 SV=2 - [GTR1_HUMAN]
Q7L099	Protein RUFY3 OS=Homo sapiens GN=RUFY3 PE=1 SV=1 - [RUFY3_HUMAN]
A5D8V6	Vacuolar protein sorting-associated protein 37C OS=Homo sapiens GN=VPS37C PE=1 SV=2 - [VP37C_HUMAN]
Q66K14	TBC1 domain family member 9B OS=Homo sapiens GN=TBC1D9B PE=1 SV=3 - [TBC9B_HUMAN]
P78509	Reelin OS=Homo sapiens GN=RELN PE=1 SV=3 - [RELN_HUMAN]
Q05682	Caldesmon OS=Homo sapiens GN=CALD1 PE=1 SV=3 - [CALD1_HUMAN]
Q9BZI7	Regulator of nonsense transcripts 3B OS=Homo sapiens GN=UPF3B PE=1 SV=1 - [REN3B_HUMAN]
Q8NEZ5	F-box only protein 22 OS=Homo sapiens GN=FBXO22 PE=1 SV=1 - [FBX22_HUMAN]
P01024	Complement C3 OS=Homo sapiens GN=C3 PE=1 SV=2 - [CO3_HUMAN]
Q01581	Hydroxymethylglutaryl-CoA synthase, cytoplasmic OS=Homo sapiens GN=HMGCS1 PE=1 SV=2 - [HMCS1_HUMAN]
Q16643	Drebrin OS=Homo sapiens GN=DBN1 PE=1 SV=4 - [DREB_HUMAN]
Q9H8Y8	Golgi reassembly-stacking protein 2 OS=Homo sapiens GN=GORASP2 PE=1 SV=3 - [GORS2_HUMAN]
Q9NZJ4	Sacsin OS=Homo sapiens GN=SACS PE=1 SV=2 - [SACS_HUMAN]
Q13123	Protein Red OS=Homo sapiens GN=IK PE=1 SV=3 - [RED_HUMAN]
Q9BSA4	Protein tweety homolog 2 OS=Homo sapiens GN=TTYH2 PE=1 SV=3 - [TTYH2_HUMAN]
Q14146	Unhealthy ribosome biogenesis protein 2 homolog OS=Homo sapiens GN=URB2 PE=2 SV=2 - [URB2_HUMAN]
Q7Z4L5	Tetratricopeptide repeat protein 21B OS=Homo sapiens GN=TTC21B PE=1 SV=2 - [TT21B_HUMAN]
P49746	Thrombospondin-3 OS=Homo sapiens GN=THBS3 PE=1 SV=1 - [TSP3_HUMAN]
Q14108	Lysosome membrane protein 2 OS=Homo sapiens GN=SCARB2 PE=1 SV=2 - [SCRB2_HUMAN]
Q147X3	N-alpha-acetyltransferase 30 OS=Homo sapiens GN=NAA30 PE=1 SV=1 - [NAA30_HUMAN]
Q01844	RNA-binding protein EWS OS=Homo sapiens GN=EWSR1 PE=1 SV=1 - [EWS_HUMAN]
Q16401	26S proteasome non-ATPase regulatory subunit 5 OS=Homo sapiens GN=PSMD5 PE=1 SV=3 - [PSMD5_HUMAN]
Q8TE59	A disintegrin and metalloproteinase with thrombospondin motifs 19 OS=Homo sapiens GN=ADAMTS19 PE=2 SV=2 - [ATS19_HUMAN]

Q9Y5S2	Serine/threonine-protein kinase MRCK beta OS=Homo sapiens GN=CDC42BPB PE=1 SV=2 - [MRCKB_HUMAN]
P23468	Receptor-type tyrosine-protein phosphatase delta OS=Homo sapiens GN=PTPRD PE=1 SV=2 - [PTPRD_HUMAN]
P16885	1-phosphatidylinositol 4,5-bisphosphate phosphodiesterase gamma-2 OS=Homo sapiens GN=PLCG2 PE=1 SV=4 - [PLCG2_HUMAN]
Q9C0E2	Exportin-4 OS=Homo sapiens GN=XPO4 PE=1 SV=2 - [XPO4_HUMAN]
Q9HB21	Pleckstrin homology domain-containing family A member 1 OS=Homo sapiens GN=PLEKHA1 PE=1 SV=2 - [PKHA1_HUMAN]
O75882	Attractin OS=Homo sapiens GN=ATRN PE=1 SV=2 - [ATRN_HUMAN]
Q9BXB5	Oxysterol-binding protein-related protein 10 OS=Homo sapiens GN=OSBPL10 PE=1 SV=2 - [OSB10_HUMAN]
Q58FG1	Putative heat shock protein HSP 90-alpha A4 OS=Homo sapiens GN=HSP90AA4P PE=5 SV=1 - [HS904_HUMAN]
Q8IV08	Phospholipase D3 OS=Homo sapiens GN=PLD3 PE=1 SV=1 - [PLD3_HUMAN]
Q8WVS4	WD repeat-containing protein 60 OS=Homo sapiens GN=WDR60 PE=1 SV=3 - [WDR60_HUMAN]
P25686	DnaJ homolog subfamily B member 2 OS=Homo sapiens GN=DNAJB2 PE=1 SV=3 - [DNJB2_HUMAN]
Q7Z401	C-myc promoter-binding protein OS=Homo sapiens GN=DENND4A PE=1 SV=2 - [MYCPP_HUMAN]
Q9C0B7	Transport and Golgi organization protein 6 homolog OS=Homo sapiens GN=TANGO6 PE=1 SV=2 - [TNG6_HUMAN]
Q9UHB6	LIM domain and actin-binding protein 1 OS=Homo sapiens GN=LIMA1 PE=1 SV=1 - [LIMA1_HUMAN]
O75147	Obscurin-like protein 1 OS=Homo sapiens GN=OBSL1 PE=1 SV=4 - [OBSL1_HUMAN]
Q3B726	DNA-directed RNA polymerase I subunit RPA43 OS=Homo sapiens GN=TWISTNB PE=1 SV=1 - [RPA43_HUMAN]
P11117	Lysosomal acid phosphatase OS=Homo sapiens GN=ACP2 PE=1 SV=3 - [PPAL_HUMAN]
Q8IWU6	Extracellular sulfatase Sulf-1 OS=Homo sapiens GN=SULF1 PE=1 SV=1 - [SULF1_HUMAN]
Q8NFD5	AT-rich interactive domain-containing protein 1B OS=Homo sapiens GN=ARID1B PE=1 SV=2 - [ARI1B_HUMAN]
O15027	Protein transport protein Sec16A OS=Homo sapiens GN=SEC16A PE=1 SV=3 - [SC16A_HUMAN]
O14727	Apoptotic protease-activating factor 1 OS=Homo sapiens GN=APAF1 PE=1 SV=2 - [APAF_HUMAN]
Q9NVG8	TBC1 domain family member 13 OS=Homo sapiens GN=TBC1D13 PE=1 SV=3 - [TBC13_HUMAN]
Q9NYJ8	TGF-beta-activated kinase 1 and MAP3K7-binding protein 2 OS=Homo sapiens GN=TAB2 PE=1 SV=1 - [TAB2_HUMAN]
Q9H2C0	Gigaxonin OS=Homo sapiens GN=GAN PE=1 SV=1 - [GAN_HUMAN]
P62256	Ubiquitin-conjugating enzyme E2 H OS=Homo sapiens GN=UBE2H PE=1 SV=1 - [UBE2H_HUMAN]
P10915	Hyaluronan and proteoglycan link protein 1 OS=Homo sapiens GN=HAPLN1 PE=2 SV=2 - [HPLN1_HUMAN]
Q96EB6	NAD-dependent protein deacetylase sirtuin-1 OS=Homo sapiens GN=SIRT1 PE=1 SV=2 - [SIR1_HUMAN]
P11279	Lysosome-associated membrane glycoprotein 1 OS=Homo sapiens GN=LAMP1 PE=1 SV=3 - [LAMP1_HUMAN]
Q3ZCW2	Galectin-related protein OS=Homo sapiens GN=LGALSL PE=1 SV=2 - [LEGL_HUMAN]
Q9NRZ9	Lymphoid-specific helicase OS=Homo sapiens GN=HELLS PE=1 SV=1 - [HELLS_HUMAN]
Q9Y4B4	Helicase ARIP4 OS=Homo sapiens GN=RAD54L2 PE=1 SV=4 - [ARIP4_HUMAN]
O43823	A-kinase anchor protein 8 OS=Homo sapiens GN=AKAP8 PE=1 SV=1 - [AKAP8_HUMAN]
O43719	HIV Tat-specific factor 1 OS=Homo sapiens GN=HTATSF1 PE=1 SV=1 - [HTSF1_HUMAN]
Q8WY07	Cationic amino acid transporter 3 OS=Homo sapiens GN=SLC7A3 PE=1 SV=1 - [CTR3_HUMAN]
Q96RU2	Ubiquitin carboxyl-terminal hydrolase 28 OS=Homo sapiens GN=USP28 PE=1 SV=1 - [UBP28_HUMAN]
Q56P03	E2F-associated phosphoprotein OS=Homo sapiens GN=EAPP PE=1 SV=4 - [EAPP_HUMAN]
Q5JSL3	Dedicator of cytokinesis protein 11 OS=Homo sapiens GN=DOCK11 PE=1 SV=2 - [DOC11_HUMAN]
Q9P0K7	Ankycorbin OS=Homo sapiens GN=RAI14 PE=1 SV=2 - [RAI14_HUMAN]
Q6UX04	Peptidyl-prolyl cis-trans isomerase CWC27 homolog OS=Homo sapiens GN=CWC27 PE=1 SV=1 - [CWC27_HUMAN]
P12931	Proto-oncogene tyrosine-protein kinase Src OS=Homo sapiens GN=SRC PE=1 SV=3 - [SRC_HUMAN]
Q04721	Neurogenic locus notch homolog protein 2 OS=Homo sapiens GN=NOTCH2 PE=1 SV=3 - [NOTC2_HUMAN]
Q9HDB5	Neurexin-3-beta OS=Homo sapiens GN=NRXN3 PE=1 SV=4 - [NRX3B_HUMAN]
Q9P035	Very-long-chain (3R)-3-hydroxyacyl-CoA dehydratase 3 OS=Homo sapiens GN=HACD3 PE=1 SV=2 - [HACD3_HUMAN]
Q86TI2	Dipeptidyl peptidase 9 OS=Homo sapiens GN=DPP9 PE=1 SV=3 - [DPP9_HUMAN]
Q8WU10	Pyridine nucleotide-disulfide oxidoreductase domain-containing protein 1 OS=Homo sapiens GN=PYROXD1 PE=1 SV=1 - [PYRD1_HUMAN]
Q5VWQ0	Round spermatid basic protein 1 OS=Homo sapiens GN=RSBN1 PE=1 SV=2 - [RSBN1_HUMAN]
Q9UL42	Paraneoplastic antigen Ma2 OS=Homo sapiens GN=PNMA2 PE=1 SV=2 - [PNMA2_HUMAN]
Q99962	Endophilin-A1 OS=Homo sapiens GN=SH3GL2 PE=1 SV=1 - [SH3G2_HUMAN]
Q96A83	Collagen alpha-1(XXVI) chain OS=Homo sapiens GN=COL26A1 PE=1 SV=1 - [COQA1_HUMAN]
P49593	Protein phosphatase 1F OS=Homo sapiens GN=PPM1F PE=1 SV=3 - [PPM1F_HUMAN]
P78316	Nucleolar protein 14 OS=Homo sapiens GN=NOP14 PE=1 SV=3 - [NOP14_HUMAN]
Q12913	Receptor-type tyrosine-protein phosphatase eta OS=Homo sapiens GN=PTPRJ PE=1 SV=3 - [PTPRJ_HUMAN]
Q13491	Neuronal membrane glycoprotein M6-b OS=Homo sapiens GN=GPM6B PE=1 SV=2 - [GPM6B_HUMAN]
P49590	Probable histidine--tRNA ligase, mitochondrial OS=Homo sapiens GN=HARS2 PE=1 SV=1 - [SYHM_HUMAN]
P0C0L4	Complement C4-A OS=Homo sapiens GN=C4A PE=1 SV=2 - [CO4A_HUMAN]
P02771	Alpha-fetoprotein OS=Homo sapiens GN=AFP PE=1 SV=1 - [FETA_HUMAN]
Q9H857	5'-nucleotidase domain-containing protein 2 OS=Homo sapiens GN=NT5DC2 PE=1 SV=1 - [NT5D2_HUMAN]
Q9HAR2	Latrophilin-3 OS=Homo sapiens GN=LPHN3 PE=2 SV=2 - [LPHN3_HUMAN]
Q6P3W7	SCY1-like protein 2 OS=Homo sapiens GN=SCYL2 PE=1 SV=1 - [SCYL2_HUMAN]
O75155	Cullin-associated NEDD8-dissociated protein 2 OS=Homo sapiens GN=CAND2 PE=1 SV=3 - [CAND2_HUMAN]
Q27J81	Inverted formin-2 OS=Homo sapiens GN=INF2 PE=1 SV=2 - [INF2_HUMAN]
Q9Y5B6	PAX3- and PAX7-binding protein 1 OS=Homo sapiens GN=PAXBP1 PE=1 SV=2 - [PAXB1_HUMAN]
P14174	Macrophage migration inhibitory factor OS=Homo sapiens GN=MIF PE=1 SV=4 - [MIF_HUMAN]
P18858	DNA ligase 1 OS=Homo sapiens GN=LIG1 PE=1 SV=1 - [DNLI1_HUMAN]
Q15058	Kinesin-like protein KIF14 OS=Homo sapiens GN=KIF14 PE=1 SV=1 - [KIF14_HUMAN]
Q9UKV3	Apoptotic chromatin condensation inducer in the nucleus OS=Homo sapiens GN=ACIN1 PE=1 SV=2 - [ACINU_HUMAN]
P12259	Coagulation factor V OS=Homo sapiens GN=F5 PE=1 SV=4 - [FA5_HUMAN]
Q96DT7	Zinc finger and BTB domain-containing protein 10 OS=Homo sapiens GN=ZBTB10 PE=1 SV=2 - [ZBT10_HUMAN]
Q14562	ATP-dependent RNA helicase DHX8 OS=Homo sapiens GN=DHX8 PE=1 SV=1 - [DHX8_HUMAN]
Q86VI3	Ras GTPase-activating-like protein IQGAP3 OS=Homo sapiens GN=IQGAP3 PE=1 SV=2 - [IQGA3_HUMAN]
Q12888	Tumor suppressor p53-binding protein 1 OS=Homo sapiens GN=TP53BP1 PE=1 SV=2 - [TP53B_HUMAN]
Q93074	Mediator of RNA polymerase II transcription subunit 12 OS=Homo sapiens GN=MED12 PE=1 SV=4 - [MED12_HUMAN]
O43741	5'-AMP-activated protein kinase subunit beta-2 OS=Homo sapiens GN=PRKAB2 PE=1 SV=1 - [AAKB2_HUMAN]
O60879	Protein diaphanous homolog 2 OS=Homo sapiens GN=DIAPH2 PE=1 SV=1 - [DIAP2_HUMAN]
Q4ZHG4	Fibronectin type III domain-containing protein 1 OS=Homo sapiens GN=FNDC1 PE=2 SV=4 - [FNDC1_HUMAN]
Q8IYB5	Stromal membrane-associated protein 1 OS=Homo sapiens GN=SMAP1 PE=1 SV=2 - [SMAP1_HUMAN]
Q9UKI8	Serine/threonine-protein kinase tousled-like 1 OS=Homo sapiens GN=TLK1 PE=1 SV=2 - [TLK1_HUMAN]
Q2PPJ7	Ral GTPase-activating protein subunit alpha-2 OS=Homo sapiens GN=RALGAPA2 PE=1 SV=2 - [RGPA2_HUMAN]
Q96EK7	Constitutive coactivator of peroxisome proliferator-activated receptor gamma OS=Homo sapiens GN=FAM120B PE=1 SV=1 - [F120B_HUMAN]
Q9NYK5	39S ribosomal protein L39, mitochondrial OS=Homo sapiens GN=MRPL39 PE=1 SV=3 - [RM39_HUMAN]
Q9UHF1	Epidermal growth factor-like protein 7 OS=Homo sapiens GN=EGFL7 PE=1 SV=3 - [EGFL7_HUMAN]
Q9HCN3	Transmembrane protein 8A OS=Homo sapiens GN=TMEM8A PE=1 SV=3 - [TMM8A_HUMAN]
O94804	Serine/threonine-protein kinase 10 OS=Homo sapiens GN=STK10 PE=1 SV=1 - [STK10_HUMAN]
O60343	TBC1 domain family member 4 OS=Homo sapiens GN=TBC1D4 PE=1 SV=2 - [TBCD4_HUMAN]
O60524	Nuclear export mediator factor NEMF OS=Homo sapiens GN=NEMF PE=1 SV=4 - [NEMF_HUMAN]
Q2TAL8	Glutamine-rich protein 1 OS=Homo sapiens GN=QRICH1 PE=1 SV=1 - [QRIC1_HUMAN]
P09486	SPARC OS=Homo sapiens GN=SPARC PE=1 SV=1 - [SPRC_HUMAN]
Q13510	Acid ceramidase OS=Homo sapiens GN=ASAH1 PE=1 SV=5 - [ASAH1_HUMAN]
P00742	Coagulation factor X OS=Homo sapiens GN=F10 PE=1 SV=2 - [FA10_HUMAN]
P35240	Merlin OS=Homo sapiens GN=NF2 PE=1 SV=1 - [MERL_HUMAN]
Q9NVN8	Guanine nucleotide-binding protein-like 3-like protein OS=Homo sapiens GN=GNL3L PE=1 SV=1 - [GNL3L_HUMAN]
Q4KMP7	TBC1 domain family member 10B OS=Homo sapiens GN=TBC1D10B PE=1 SV=3 - [TB10B_HUMAN]
Q6ZRS2	Helicase SRCAP OS=Homo sapiens GN=SRCAP PE=1 SV=3 - [SRCAP_HUMAN]
Q9BW71	HIRA-interacting protein 3 OS=Homo sapiens GN=HIRIP3 PE=1 SV=3 - [HIRP3_HUMAN]
Q9H0R1	AP-5 complex subunit mu-1 OS=Homo sapiens GN=AP5M1 PE=1 SV=2 - [AP5M1_HUMAN]
Q9Y274	Type 2 lactosamine alpha-2,3-sialyltransferase OS=Homo sapiens GN=ST3GAL6 PE=1 SV=1 - [SIA10_HUMAN]
Q6AI08	HEAT repeat-containing protein 6 OS=Homo sapiens GN=HEATR6 PE=1 SV=1 - [HEAT6_HUMAN]
Q2TAZ0	Autophagy-related protein 2 homolog A OS=Homo sapiens GN=ATG2A PE=1 SV=3 - [ATG2A_HUMAN]
Q5SVZ6	Zinc finger MYM-type protein 1 OS=Homo sapiens GN=ZMYM1 PE=2 SV=1 - [ZMYM1_HUMAN]
Q15054	DNA polymerase delta subunit 3 OS=Homo sapiens GN=POLD3 PE=1 SV=2 - [DPOD3_HUMAN]
P56199	Integrin alpha-1 OS=Homo sapiens GN=ITGA1 PE=1 SV=2 - [ITA1_HUMAN]
Q00325	Phosphate carrier protein, mitochondrial OS=Homo sapiens GN=SLC25A3 PE=1 SV=2 - [MPCP_HUMAN]
Q14149	MORC family CW-type zinc finger protein 3 OS=Homo sapiens GN=MORC3 PE=1 SV=3 - [MORC3_HUMAN]
Q8NEZ3	WD repeat-containing protein 19 OS=Homo sapiens GN=WDR19 PE=1 SV=2 - [WDR19_HUMAN]
P18075	Bone morphogenetic protein 7 OS=Homo sapiens GN=BMP7 PE=1 SV=1 - [BMP7_HUMAN]
P51531	Probable global transcription activator SNF2L2 OS=Homo sapiens GN=SMARCA2 PE=1 SV=2 - [SMCA2_HUMAN]
Q15527	Surfeit locus protein 2 OS=Homo sapiens GN=SURF2 PE=1 SV=3 - [SURF2_HUMAN]
Q9BTA9	WW domain-containing adapter protein with coiled-coil OS=Homo sapiens GN=WAC PE=1 SV=3 - [WAC_HUMAN]
Q0JRZ9	F-BAR domain only protein 2 OS=Homo sapiens GN=FCHO2 PE=1 SV=2 - [FCHO2_HUMAN]
P78357	Contactin-associated protein 1 OS=Homo sapiens GN=CNTNAP1 PE=1 SV=1 - [CNTP1_HUMAN]
P85037	Forkhead box protein K1 OS=Homo sapiens GN=FOXK1 PE=1 SV=1 - [FOXK1_HUMAN]
Q96PY5	Formin-like protein 2 OS=Homo sapiens GN=FMNL2 PE=1 SV=3 - [FMNL2_HUMAN]
O75792	Ribonuclease H2 subunit A OS=Homo sapiens GN=RNASEH2A PE=1 SV=2 - [RNH2A_HUMAN]
Q6DKJ4	Nucleoredoxin OS=Homo sapiens GN=NXN PE=1 SV=2 - [NXN_HUMAN]
Q9NWX5	Ankyrin repeat and SOCS box protein 6 OS=Homo sapiens GN=ASB6 PE=1 SV=1 - [ASB6_HUMAN]

Q9UBF8	Phosphatidylinositol 4-kinase beta OS=Homo sapiens GN=PI4KB PE=1 SV=1 - [PI4KB_HUMAN]
O75122	CLIP-associating protein 2 OS=Homo sapiens GN=CLASP2 PE=1 SV=2 - [CLAP2_HUMAN]
O60566	Mitotic checkpoint serine/threonine-protein kinase BUB1 beta OS=Homo sapiens GN=BUB1B PE=1 SV=3 - [BUB1B_HUMAN]
Q9BVC5	Ashwin OS=Homo sapiens GN=C2orf49 PE=1 SV=1 - [ASHWN_HUMAN]
P48740	Mannan-binding lectin serine protease 1 OS=Homo sapiens GN=MASP1 PE=1 SV=3 - [MASP1_HUMAN]
Q96P70	Importin-9 OS=Homo sapiens GN=IPO9 PE=1 SV=3 - [IPO9_HUMAN]
Q9HCK8	Chromodomain-helicase-DNA-binding protein 8 OS=Homo sapiens GN=CHD8 PE=1 SV=5 - [CHD8_HUMAN]
Q9P2H3	Intraflagellar transport protein 80 homolog OS=Homo sapiens GN=IFT80 PE=1 SV=3 - [IFT80_HUMAN]
Q9Y4E1	WASH complex subunit FAM21C OS=Homo sapiens GN=FAM21C PE=1 SV=3 - [FA21C_HUMAN]
P81877	Single-stranded DNA-binding protein 2 OS=Homo sapiens GN=SSBP2 PE=1 SV=2 - [SSBP2_HUMAN]
Q6IN85	Serine/threonine-protein phosphatase 4 regulatory subunit 3A OS=Homo sapiens GN=SMEK1 PE=1 SV=1 - [P4R3A_HUMAN]
Q10469	Alpha-1,6-mannosyl-glycoprotein 2-beta-N-acetylglucosaminyltransferase OS=Homo sapiens GN=MGAT2 PE=1 SV=1 - [MGAT2_HUMAN]
Q641Q2	WASH complex subunit FAM21A OS=Homo sapiens GN=FAM21A PE=2 SV=3 - [FA21A_HUMAN]
Q9NRG4	N-lysine methyltransferase SMYD2 OS=Homo sapiens GN=SMYD2 PE=1 SV=2 - [SMYD2_HUMAN]
P17405	Sphingomyelin phosphodiesterase OS=Homo sapiens GN=SMPD1 PE=1 SV=4 - [ASM_HUMAN]
P21359	Neurofibromin OS=Homo sapiens GN=NF1 PE=1 SV=2 - [NF1_HUMAN]
A1X283	SH3 and PX domain-containing protein 2B OS=Homo sapiens GN=SH3PXD2B PE=1 SV=3 - [SPD2B_HUMAN]
O94817	Ubiquitin-like protein ATG12 OS=Homo sapiens GN=ATG12 PE=1 SV=1 - [ATG12_HUMAN]
Q96Q42	Alsin OS=Homo sapiens GN=ALS2 PE=1 SV=2 - [ALS2_HUMAN]
P02452	Collagen alpha-1(I) chain OS=Homo sapiens GN=COL1A1 PE=1 SV=5 - [CO1A1_HUMAN]
Q15048	Leucine-rich repeat-containing protein 14 OS=Homo sapiens GN=LRRC14 PE=2 SV=1 - [LRC14_HUMAN]
Q96TA1	Niban-like protein 1 OS=Homo sapiens GN=FAM129B PE=1 SV=3 - [NIBL1_HUMAN]
Q9P0T7	Transmembrane protein 9 OS=Homo sapiens GN=TMEM9 PE=1 SV=1 - [TMEM9_HUMAN]
O14662	Syntaxin-16 OS=Homo sapiens GN=STX16 PE=1 SV=3 - [STX16_HUMAN]
O75962	Triple functional domain protein OS=Homo sapiens GN=TRIO PE=1 SV=2 - [TRIO_HUMAN]
P13473	Lysosome-associated membrane glycoprotein 2 OS=Homo sapiens GN=LAMP2 PE=1 SV=2 - [LAMP2_HUMAN]
Q9C0H2	Protein tweety homolog 3 OS=Homo sapiens GN=TTYH3 PE=1 SV=3 - [TTYH3_HUMAN]
Q5VT25	Serine/threonine-protein kinase MRCK alpha OS=Homo sapiens GN=CDC42BPA PE=1 SV=1 - [MRCKA_HUMAN]
Q96R06	Sperm-associated antigen 5 OS=Homo sapiens GN=SPAG5 PE=1 SV=2 - [SPAG5_HUMAN]
Q5D862	Filaggrin-2 OS=Homo sapiens GN=FLG2 PE=1 SV=1 - [FILA2_HUMAN]
Q9UBG0	C-type mannose receptor 2 OS=Homo sapiens GN=MRC2 PE=1 SV=2 - [MRC2_HUMAN]
Q13367	AP-3 complex subunit beta-2 OS=Homo sapiens GN=AP3B2 PE=1 SV=2 - [AP3B2_HUMAN]
Q9BUZ4	TNF receptor-associated factor 4 OS=Homo sapiens GN=TRAF4 PE=1 SV=1 - [TRAF4_HUMAN]
Q5T5X7	BEN domain-containing protein 3 OS=Homo sapiens GN=BEND3 PE=1 SV=1 - [BEND3_HUMAN]
P98175	RNA-binding protein 10 OS=Homo sapiens GN=RBM10 PE=1 SV=3 - [RBM10_HUMAN]
P35626	Beta-adrenergic receptor kinase 2 OS=Homo sapiens GN=ADRBK2 PE=1 SV=2 - [ARBK2_HUMAN]
Q9ULX6	A-kinase anchor protein 8-like OS=Homo sapiens GN=AKAP8L PE=1 SV=3 - [AKP8L_HUMAN]
O43422	52 kDa repressor of the inhibitor of the protein kinase OS=Homo sapiens GN=PRKRIR PE=1 SV=2 - [P52K_HUMAN]
Q14520	Hyaluronan-binding protein 2 OS=Homo sapiens GN=HABP2 PE=1 SV=1 - [HABP2_HUMAN]
Q15648	Mediator of RNA polymerase II transcription subunit 1 OS=Homo sapiens GN=MED1 PE=1 SV=4 - [MED1_HUMAN]
P49790	Nuclear pore complex protein Nup153 OS=Homo sapiens GN=NUP153 PE=1 SV=2 - [NU153_HUMAN]
Q9Y6A5	Transforming acidic coiled-coil-containing protein 3 OS=Homo sapiens GN=TACC3 PE=1 SV=1 - [TACC3_HUMAN]
O14497	AT-rich interactive domain-containing protein 1A OS=Homo sapiens GN=ARID1A PE=1 SV=3 - [ARI1A_HUMAN]
O75175	CCR4-NOT transcription complex subunit 3 OS=Homo sapiens GN=CNOT3 PE=1 SV=1 - [CNOT3_HUMAN]
Q8ND56	Protein LSM14 homolog A OS=Homo sapiens GN=LSM14A PE=1 SV=3 - [LS14A_HUMAN]
O43670	BUB3-interacting and GLEBS motif-containing protein ZNF207 OS=Homo sapiens GN=ZNF207 PE=1 SV=1 - [ZN207_HUMAN]
Q14157	Ubiquitin-associated protein 2-like OS=Homo sapiens GN=UBAP2L PE=1 SV=2 - [UBP2L_HUMAN]
Q6ZT12	E3 ubiquitin-protein ligase UBR3 OS=Homo sapiens GN=UBR3 PE=2 SV=2 - [UBR3_HUMAN]
O43432	Eukaryotic translation initiation factor 4 gamma 3 OS=Homo sapiens GN=EIF4G3 PE=1 SV=2 - [IF4G3_HUMAN]
O00459	Phosphatidylinositol 3-kinase regulatory subunit beta OS=Homo sapiens GN=PIK3R2 PE=1 SV=2 - [P85B HUMAN]
Q16787	Laminin subunit alpha-3 OS=Homo sapiens GN=LAMA3 PE=1 SV=2 - [LAMA3_HUMAN]
Q9Y485	DmX-like protein 1 OS=Homo sapiens GN=DMXL1 PE=1 SV=3 - [DMXL1 HUMAN]
Q9Y613	FH1/FH2 domain-containing protein 1 OS=Homo sapiens GN=FHOD1 PE=1 SV=3 - [FHOD1_HUMAN]
Q53H47	Histone-lysine N-methyltransferase SETMAR OS=Homo sapiens GN=SETMAR PE=1 SV=2 - [SETMR_HUMAN]
Q9C0C7	Activating molecule in BECN1-regulated autophagy protein 1 OS=Homo sapiens GN=AMBRA1 PE=1 SV=2 - [AMRA1_HUMAN]
Q9Y6Z7	Collectin-10 OS=Homo sapiens GN=COLEC10 PE=2 SV=2 - [COL10_HUMAN]
P35749	Myosin-11 OS=Homo sapiens GN=MYH11 PE=1 SV=3 - [MYH11_HUMAN]
Q9NZI7	Upstream-binding protein 1 OS=Homo sapiens GN=UBP1 PE=1 SV=1 - [UBIP1_HUMAN]
P0CG39	POTE ankyrin domain family member J OS=Homo sapiens GN=POTEJ PE=3 SV=1 - [POTEJ_HUMAN]
Q86TP1	Protein prune homolog OS=Homo sapiens GN=PRUNE PE=1 SV=2 - [PRUNE_HUMAN]
Q9H081	Protein MIS12 homolog OS=Homo sapiens GN=MIS12 PE=1 SV=1 - [MIS12_HUMAN]
P29590	Protein PML OS=Homo sapiens GN=PML PE=1 SV=3 - [PML_HUMAN]
O60636	Tetraspanin-2 OS=Homo sapiens GN=TSPAN2 PE=2 SV=2 - [TSN2_HUMAN]
P35658	Nuclear pore complex protein Nup214 OS=Homo sapiens GN=NUP214 PE=1 SV=2 - [NU214_HUMAN]
Q9UNK0	Syntaxin-8 OS=Homo sapiens GN=STX8 PE=1 SV=2 - [STX8_HUMAN]
O96013	Serine/threonine-protein kinase PAK 4 OS=Homo sapiens GN=PAK4 PE=1 SV=1 - [PAK4_HUMAN]
Q7Z460	CLIP-associating protein 1 OS=Homo sapiens GN=CLASP1 PE=1 SV=1 - [CLAP1_HUMAN]
Q14767	Latent-transforming growth factor beta-binding protein 2 OS=Homo sapiens GN=LTBP2 PE=1 SV=3 - [LTBP2_HUMAN]
O60504	Vinexin OS=Homo sapiens GN=SORBS3 PE=1 SV=2 - [VINEX_HUMAN]
Q8IXT5	RNA-binding protein 12B OS=Homo sapiens GN=RBM12B PE=1 SV=2 - [RB12B_HUMAN]
P05997	Collagen alpha-2(V) chain OS=Homo sapiens GN=COL5A2 PE=1 SV=3 - [CO5A2_HUMAN]
P16383	GC-rich sequence DNA-binding factor 2 OS=Homo sapiens GN=GCFC2 PE=1 SV=2 - [GCFC2_HUMAN]
Q7Z5N4	Protein sidekick-1 OS=Homo sapiens GN=SDK1 PE=2 SV=3 - [SDK1_HUMAN]
Q96RY7	Intraflagellar transport protein 140 homolog OS=Homo sapiens GN=IFT140 PE=1 SV=1 - [IF140_HUMAN]
O43318	Mitogen-activated protein kinase kinase kinase 7 OS=Homo sapiens GN=MAP3K7 PE=1 SV=1 - [M3K7_HUMAN]
P49454	Centromere protein F OS=Homo sapiens GN=CENPF PE=1 SV=2 - [CENPF_HUMAN]
Q2VPK5	Cytoplasmic tRNA 2-thiolation protein 2 OS=Homo sapiens GN=CTU2 PE=1 SV=1 - [CTU2_HUMAN]
Q6PI26	Protein SHQ1 homolog OS=Homo sapiens GN=SHQ1 PE=1 SV=2 - [SHQ1 HUMAN]
Q8N7H5	RNA polymerase II-associated factor 1 homolog OS=Homo sapiens GN=PAF1 PE=1 SV=2 - [PAF1_HUMAN]
Q709C8	Vacuolar protein sorting-associated protein 13C OS=Homo sapiens GN=VPS13C PE=1 SV=1 - [VP13C_HUMAN]
Q5JSH3	WD repeat-containing protein 44 OS=Homo sapiens GN=WDR44 PE=1 SV=1 - [WDR44_HUMAN]
Q9H813	Transmembrane protein 206 OS=Homo sapiens GN=TMEM206 PE=1 SV=1 - [TM206_HUMAN]
Q9UBP4	Dickkopf-related protein 3 OS=Homo sapiens GN=DKK3 PE=1 SV=2 - [DKK3_HUMAN]
P54756	Ephrin type-A receptor 5 OS=Homo sapiens GN=EPHA5 PE=1 SV=3 - [EPHA5_HUMAN]
Q6ZSZ5	Rho guanine nucleotide exchange factor 18 OS=Homo sapiens GN=ARHGEF18 PE=1 SV=3 - [ARHGI_HUMAN]
Q9Y5T5	Ubiquitin carboxyl-terminal hydrolase 16 OS=Homo sapiens GN=USP16 PE=1 SV=1 - [UBP16_HUMAN]
Q9Y5V3	Melanoma-associated antigen D1 OS=Homo sapiens GN=MAGED1 PE=1 SV=3 - [MAGD1_HUMAN]
P47712	Cytosolic phospholipase A2 OS=Homo sapiens GN=PLA2G4A PE=1 SV=2 - [PA24A_HUMAN]
Q92544	Transmembrane 9 superfamily member 4 OS=Homo sapiens GN=TM9SF4 PE=1 SV=2 - [TM9S4_HUMAN]
Q96HC4	PDZ and LIM domain protein 5 OS=Homo sapiens GN=PDLIM5 PE=1 SV=5 - [PDLI5_HUMAN]
P49756	RNA-binding protein 25 OS=Homo sapiens GN=RBM25 PE=1 SV=3 - [RBM25_HUMAN]
Q9BWT3	Poly(A) polymerase gamma OS=Homo sapiens GN=PAPOLG PE=1 SV=2 - [PAPOG_HUMAN]
Q2VPB7	AP-5 complex subunit beta-1 OS=Homo sapiens GN=AP5B1 PE=1 SV=4 - [AP5B1_HUMAN]
Q15059	Bromodomain-containing protein 3 OS=Homo sapiens GN=BRD3 PE=1 SV=1 - [BRD3_HUMAN]
Q7Z7M0	Multiple epidermal growth factor-like domains protein 8 OS=Homo sapiens GN=MEGF8 PE=1 SV=2 - [MEGF8_HUMAN]
Q8NFP9	Neurobeachin OS=Homo sapiens GN=NBEA PE=1 SV=3 - [NBEA_HUMAN]
Q4LE39	AT-rich interactive domain-containing protein 4B OS=Homo sapiens GN=ARID4B PE=1 SV=2 - [ARI4B_HUMAN]
Q9Y5U2	Protein TSSC4 OS=Homo sapiens GN=TSSC4 PE=1 SV=3 - [TSSC4_HUMAN]
Q13427	Peptidyl-prolyl cis-trans isomerase G OS=Homo sapiens GN=PPIG PE=1 SV=2 - [PPIG_HUMAN]
Q07002	Cyclin-dependent kinase 18 OS=Homo sapiens GN=CDK18 PE=1 SV=3 - [CDK18_HUMAN]
Q9BVS4	Serine/threonine-protein kinase RIO2 OS=Homo sapiens GN=RIOK2 PE=1 SV=2 - [RIOK2_HUMAN]
Q9C0B1	Alpha-ketoglutarate-dependent dioxygenase FTO OS=Homo sapiens GN=FTO PE=1 SV=3 - [FTO_HUMAN]
Q8N6H7	ADP-ribosylation factor GTPase-activating protein 2 OS=Homo sapiens GN=ARFGAP2 PE=1 SV=1 - [ARFG2 HUMAN]
Q8N4C8	Misshapen-like kinase 1 OS=Homo sapiens GN=MINK1 PE=1 SV=2 - [MINK1_HUMAN]
Q5VYS8	Terminal uridylyltransferase 7 OS=Homo sapiens GN=ZCCHC6 PE=1 SV=1 - [TUT7_HUMAN]
Q68DK2	Zinc finger FYVE domain-containing protein 26 OS=Homo sapiens GN=ZFYVE26 PE=1 SV=3 - [ZFY26_HUMAN]
Q99941	Cyclic AMP-dependent transcription factor ATF-6 beta OS=Homo sapiens GN=ATF6B PE=1 SV=2 - [ATF6B_HUMAN]
Q15283	Ras GTPase-activating protein 2 OS=Homo sapiens GN=RASA2 PE=1 SV=3 - [RASA2_HUMAN]
Q13136	Liprin-alpha-1 OS=Homo sapiens GN=PPFIA1 PE=1 SV=1 - [LIPA1_HUMAN]
Q9ULQ0	Striatin-interacting protein 2 OS=Homo sapiens GN=STRIP2 PE=1 SV=2 - [STRP2_HUMAN]
Q14563	Semaphorin-3A OS=Homo sapiens GN=SEMA3A PE=1 SV=1 - [SEM3A_HUMAN]
O60551	Glycylpeptide N-tetradecanoyltransferase 2 OS=Homo sapiens GN=NMT2 PE=1 SV=1 - [NMT2_HUMAN]
O60831	PRA1 family protein 2 OS=Homo sapiens GN=PRAF2 PE=1 SV=1 - [PRAF2_HUMAN]
Q96MX6	WD repeat-containing protein 92 OS=Homo sapiens GN=WDR92 PE=1 SV=1 - [WDR92_HUMAN]
Q9UNN5	FAS-associated factor 1 OS=Homo sapiens GN=FAF1 PE=1 SV=2 - [FAF1_HUMAN]

Q15751	Probable E3 ubiquitin-protein ligase HERC1 OS=Homo sapiens GN=HERC1 PE=1 SV=2 - [HERC1_HUMAN]
Q6KC79	Nipped-B-like protein OS=Homo sapiens GN=NIPBL PE=1 SV=2 - [NIPBL_HUMAN]
Q6V0I7	Protocadherin Fat 4 OS=Homo sapiens GN=FAT4 PE=1 SV=2 - [FAT4_HUMAN]
O75493	Carbonic anhydrase-related protein 11 OS=Homo sapiens GN=CA11 PE=1 SV=2 - [CAH11_HUMAN]
P23470	Receptor-type tyrosine-protein phosphatase gamma OS=Homo sapiens GN=PTPRG PE=1 SV=4 - [PTPRG_HUMAN]
O15550	Lysine-specific demethylase 6A OS=Homo sapiens GN=KDM6A PE=1 SV=2 - [KDM6A_HUMAN]
O94915	Protein furry homolog-like OS=Homo sapiens GN=FRYL PE=1 SV=2 - [FRYL_HUMAN]
O95544	NAD kinase OS=Homo sapiens GN=NADK PE=1 SV=1 - [NADK_HUMAN]
Q92556	Engulfment and cell motility protein 1 OS=Homo sapiens GN=ELMO1 PE=1 SV=2 - [ELMO1_HUMAN]
Q99523	Sortilin OS=Homo sapiens GN=SORT1 PE=1 SV=3 - [SORT_HUMAN]
Q8TEU7	Rap guanine nucleotide exchange factor 6 OS=Homo sapiens GN=RAPGEF6 PE=1 SV=2 - [RPGF6_HUMAN]
O43379	WD repeat-containing protein 62 OS=Homo sapiens GN=WDR62 PE=1 SV=4 - [WDR62_HUMAN]
O15047	Histone-lysine N-methyltransferase SETD1A OS=Homo sapiens GN=SETD1A PE=1 SV=3 - [SET1A_HUMAN]
Q5VZ89	DENN domain-containing protein 4C OS=Homo sapiens GN=DENND4C PE=1 SV=2 - [DEN4C_HUMAN]
Q9UIQ6	Leucyl-cystinyl aminopeptidase OS=Homo sapiens GN=LNPEP PE=1 SV=3 - [LCAP_HUMAN]
Q9C0B0	RING finger protein unkempt homolog OS=Homo sapiens GN=UNK PE=1 SV=2 - [UNK_HUMAN]
P29558	RNA-binding motif, single-stranded-interacting protein 1 OS=Homo sapiens GN=RBMS1 PE=1 SV=3 - [RBMS1_HUMAN]
Q4VCS5	Angiomotin OS=Homo sapiens GN=AMOT PE=1 SV=1 - [AMOT_HUMAN]
Q14004	Cyclin-dependent kinase 13 OS=Homo sapiens GN=CDK13 PE=1 SV=2 - [CDK13_HUMAN]
Q08722	Leukocyte surface antigen CD47 OS=Homo sapiens GN=CD47 PE=1 SV=1 - [CD47_HUMAN]
Q969F9	Hermansky-Pudlak syndrome 3 protein OS=Homo sapiens GN=HPS3 PE=1 SV=1 - [HPS3_HUMAN]
Q96RE7	Nucleus accumbens-associated protein 1 OS=Homo sapiens GN=NACC1 PE=1 SV=1 - [NACC1_HUMAN]
Q9C0D5	Protein TANC1 OS=Homo sapiens GN=TANC1 PE=1 SV=3 - [TANC1_HUMAN]
P01031	Complement C5 OS=Homo sapiens GN=C5 PE=1 SV=4 - [CO5_HUMAN]
Q9UQ35	Serine/arginine repetitive matrix protein 2 OS=Homo sapiens GN=SRRM2 PE=1 SV=2 - [SRRM2_HUMAN]
Q9H4Z3	Phosphorylated CTD-interacting factor 1 OS=Homo sapiens GN=PCIF1 PE=1 SV=1 - [PCIF1_HUMAN]
Q15345	Leucine-rich repeat-containing protein 41 OS=Homo sapiens GN=LRRC41 PE=1 SV=3 - [LRC41_HUMAN]
Q5W0B1	RING finger protein 219 OS=Homo sapiens GN=RNF219 PE=1 SV=1 - [RN219_HUMAN]
P23434	Glycine cleavage system H protein, mitochondrial OS=Homo sapiens GN=GCSH PE=1 SV=2 - [GCSH_HUMAN]
Q9Y4D8	Probable E3 ubiquitin-protein ligase HECTD4 OS=Homo sapiens GN=HECTD4 PE=1 SV=5 - [HECD4_HUMAN]
Q9UKP4	A disintegrin and metalloproteinase with thrombospondin motifs 7 OS=Homo sapiens GN=ADAMTS7 PE=1 SV=2 - [ATS7_HUMAN]
Q8IWZ3	Ankyrin repeat and KH domain-containing protein 1 OS=Homo sapiens GN=ANKHD1 PE=1 SV=1 - [ANKH1_HUMAN]
Q96I25	Splicing factor 45 OS=Homo sapiens GN=RBM17 PE=1 SV=1 - [SPF45 HUMAN]
O94888	UBX domain-containing protein 7 OS=Homo sapiens GN=UBXN7 PE=1 SV=2 - [UBXN7_HUMAN]
Q8IZP7	Heparan-sulfate 6-O-sulfotransferase 3 OS=Homo sapiens GN=HS6ST3 PE=2 SV=3 - [H6ST3_HUMAN]
O00763	Acetyl-CoA carboxylase 2 OS=Homo sapiens GN=ACACB PE=1 SV=3 - [ACACB_HUMAN]
Q14CX7	N-alpha-acetyltransferase 25, NatB auxiliary subunit OS=Homo sapiens GN=NAA25 PE=1 SV=1 - [NAA25_HUMAN]
Q75QN2	Integrator complex subunit 8 OS=Homo sapiens GN=INTS8 PE=1 SV=1 - [INT8_HUMAN]
O75145	Liprin-alpha-3 OS=Homo sapiens GN=PPFIA3 PE=1 SV=3 - [LIPA3_HUMAN]
O94782	Ubiquitin carboxyl-terminal hydrolase 1 OS=Homo sapiens GN=USP1 PE=1 SV=1 - [UBP1_HUMAN]
Q12830	Nucleosome-remodeling factor subunit BPTF OS=Homo sapiens GN=BPTF PE=1 SV=3 - [BPTF_HUMAN]
Q58WW2	DDB1- and CUL4-associated factor 6 OS=Homo sapiens GN=DCAF6 PE=1 SV=1 - [DCAF6_HUMAN]
Q15435	Protein phosphatase 1 regulatory subunit 7 OS=Homo sapiens GN=PPP1R7 PE=1 SV=1 - [PP1R7_HUMAN]
P33527	Multidrug resistance-associated protein 1 OS=Homo sapiens GN=ABCC1 PE=1 SV=3 - [MRP1_HUMAN]
Q8IY95	Transmembrane protein 192 OS=Homo sapiens GN=TMEM192 PE=1 SV=1 - [TM192_HUMAN]
P02462	Collagen alpha-1(IV) chain OS=Homo sapiens GN=COL4A1 PE=1 SV=3 - [CO4A1_HUMAN]
Q3V6T2	Girdin OS=Homo sapiens GN=CCDC88A PE=1 SV=2 - [GRDN_HUMAN]
Q8WWQ0	PH-interacting protein OS=Homo sapiens GN=PHIP PE=1 SV=2 - [PHIP_HUMAN]
Q5VT52	Regulation of nuclear pre-mRNA domain-containing protein 2 OS=Homo sapiens GN=RPRD2 PE=1 SV=1 - [RPRD2_HUMAN]
O43464	Serine protease HTRA2, mitochondrial OS=Homo sapiens GN=HTRA2 PE=1 SV=2 - [HTRA2_HUMAN]
Q7Z3K3	Pogo transposable element with ZNF domain OS=Homo sapiens GN=POGZ PE=1 SV=2 - [POGZ_HUMAN]
Q9UGR2	Zinc finger CCCH domain-containing protein 7B OS=Homo sapiens GN=ZC3H7B PE=1 SV=1 - [Z3H7B_HUMAN]
Q9ULI3	Protein HEG homolog 1 OS=Homo sapiens GN=HEG1 PE=1 SV=3 - [HEG1_HUMAN]
P16112	Aggrecan core protein OS=Homo sapiens GN=ACAN PE=1 SV=2 - [PGCA_HUMAN]
Q9Y608	Leucine-rich repeat flightless-interacting protein 2 OS=Homo sapiens GN=LRRFIP2 PE=1 SV=1 - [LRRF2_HUMAN]
Q93075	Putative deoxyribonuclease TATDN2 OS=Homo sapiens GN=TATDN2 PE=2 SV=2 - [TATD2_HUMAN]
P56159	GDNF family receptor alpha-1 OS=Homo sapiens GN=GFRA1 PE=2 SV=2 - [GFRA1_HUMAN]
P18850	Cyclic AMP-dependent transcription factor ATF-6 alpha OS=Homo sapiens GN=ATF6 PE=1 SV=3 - [ATF6A_HUMAN]
Q9ULP9	TBC1 domain family member 24 OS=Homo sapiens GN=TBC1D24 PE=1 SV=2 - [TBC24_HUMAN]
Q9BPZ7	Target of rapamycin complex 2 subunit MAPKAP1 OS=Homo sapiens GN=MAPKAP1 PE=1 SV=2 - [SIN1_HUMAN]
Q9UL54	Serine/threonine-protein kinase TAO2 OS=Homo sapiens GN=TAOK2 PE=1 SV=2 - [TAOK2_HUMAN]
Q13480	GRB2-associated-binding protein 1 OS=Homo sapiens GN=GAB1 PE=1 SV=2 - [GAB1_HUMAN]
Q5XPI4	E3 ubiquitin-protein ligase RNF123 OS=Homo sapiens GN=RNF123 PE=1 SV=1 - [RN123_HUMAN]
Q6NW29	RWD domain-containing protein 4 OS=Homo sapiens GN=RWDD4 PE=1 SV=3 - [RWDD4_HUMAN]
Q9ULU4	Protein kinase C-binding protein 1 OS=Homo sapiens GN=ZMYND8 PE=1 SV=2 - [PKCB1_HUMAN]
Q12873	Chromodomain-helicase-DNA-binding protein 3 OS=Homo sapiens GN=CHD3 PE=1 SV=3 - [CHD3_HUMAN]
Q5TCZ1	SH3 and PX domain-containing protein 2A OS=Homo sapiens GN=SH3PXD2A PE=1 SV=1 - [SPD2A_HUMAN]
P98153	Integral membrane protein DGCR2/IDD OS=Homo sapiens GN=DGCR2 PE=2 SV=1 - [IDD_HUMAN]
Q15555	Microtubule-associated protein RP/EB family member 2 OS=Homo sapiens GN=MAPRE2 PE=1 SV=1 - [MARE2 HUMAN]
Q9H5H4	Zinc finger protein 768 OS=Homo sapiens GN=ZNF768 PE=1 SV=2 - [ZN768_HUMAN]
Q92845	Kinesin-associated protein 3 OS=Homo sapiens GN=KIFAP3 PE=1 SV=2 - [KIFA3_HUMAN]
O43759	Synaptogyrin-1 OS=Homo sapiens GN=SYNGR1 PE=1 SV=2 - [SNG1_HUMAN]
O60885	Bromodomain-containing protein 4 OS=Homo sapiens GN=BRD4 PE=1 SV=2 - [BRD4_HUMAN]
Q13057	Bifunctional coenzyme A synthase OS=Homo sapiens GN=COASY PE=1 SV=4 - [COASY_HUMAN]
Q14031	Collagen alpha-6(IV) chain OS=Homo sapiens GN=COL4A6 PE=1 SV=3 - [CO4A6_HUMAN]
Q15262	Receptor-type tyrosine-protein phosphatase kappa OS=Homo sapiens GN=PTPRK PE=1 SV=2 - [PTPRK_HUMAN]
Q96RQ3	Methylcrotonoyl-CoA carboxylase subunit alpha, mitochondrial OS=Homo sapiens GN=MCCC1 PE=1 SV=3 - [MCCA_HUMAN]
Q9NVR0	Kelch-like protein 11 OS=Homo sapiens GN=KLHL11 PE=1 SV=1 - [KLH11_HUMAN]
Q9UGV2	Protein NDRG3 OS=Homo sapiens GN=NDRG3 PE=1 SV=2 - [NDRG3_HUMAN]
Q8NF37	Lysophosphatidylcholine acyltransferase 1 OS=Homo sapiens GN=LPCAT1 PE=1 SV=2 - [PCAT1_HUMAN]
Q13867	Bleomycin hydrolase OS=Homo sapiens GN=BLMH PE=1 SV=1 - [BLMH_HUMAN]
P47914	60S ribosomal protein L29 OS=Homo sapiens GN=RPL29 PE=1 SV=2 - [RL29_HUMAN]
P01023	Alpha-2-macroglobulin OS=Homo sapiens GN=A2M PE=1 SV=3 - [A2MG HUMAN]
Q13315	Serine-protein kinase ATM OS=Homo sapiens GN=ATM PE=1 SV=4 - [ATM_HUMAN]
Q8N9N2	Activating signal cointegrator 1 complex subunit 1 OS=Homo sapiens GN=ASCC1 PE=1 SV=1 - [ASCC1_HUMAN]
Q96IZ0	PRKC apoptosis WT1 regulator protein OS=Homo sapiens GN=PAWR PE=1 SV=1 - [PAWR_HUMAN]
Q96T23	Remodeling and spacing factor 1 OS=Homo sapiens GN=RSF1 PE=1 SV=2 - [RSF1_HUMAN]
Q7Z6B0	Coiled-coil domain-containing protein 91 OS=Homo sapiens GN=CCDC91 PE=1 SV=2 - [CCD91_HUMAN]
Q9HD26	Golgi-associated PDZ and coiled-coil motif-containing protein OS=Homo sapiens GN=GOPC PE=1 SV=1 - [GOPC_HUMAN]
O75808	Calpain-15 OS=Homo sapiens GN=CAPN15 PE=1 SV=1 - [CAN15_HUMAN]
Q96GX9	Methylthioribulose-1-phosphate dehydratase OS=Homo sapiens GN=APIP PE=1 SV=1 - [MTNB_HUMAN]
P42694	Probable helicase with zinc finger domain OS=Homo sapiens GN=HELZ PE=1 SV=2 - [HELZ_HUMAN]
Q86UW7	Calcium-dependent secretion activator 2 OS=Homo sapiens GN=CADPS2 PE=1 SV=2 - [CAPS2_HUMAN]
Q15041	ADP-ribosylation factor-like protein 6-interacting protein 1 OS=Homo sapiens GN=ARL6IP1 PE=1 SV=2 - [AR6P1_HUMAN]
O75489	NADH dehydrogenase [ubiquinone] iron-sulfur protein 3, mitochondrial OS=Homo sapiens GN=NDUFS3 PE=1 SV=1 - [NDUS3_HUMAN]
Q6ZS17	Protein FAM65A OS=Homo sapiens GN=FAM65A PE=1 SV=1 - [FA65A_HUMAN]
Q6Y7W6	PERQ amino acid-rich with GYF domain-containing protein 2 OS=Homo sapiens GN=GIGYF2 PE=1 SV=1 - [PERQ2_HUMAN]
Q13277	Syntaxin-3 OS=Homo sapiens GN=STX3 PE=1 SV=3 - [STX3_HUMAN]
Q8IZQ1	WD repeat and FYVE domain-containing protein 3 OS=Homo sapiens GN=WDFY3 PE=1 SV=2 - [WDFY3_HUMAN]
Q15643	Thyroid receptor-interacting protein 11 OS=Homo sapiens GN=TRIP11 PE=1 SV=3 - [TRIPB_HUMAN]
Q01433	AMP deaminase 2 OS=Homo sapiens GN=AMPD2 PE=1 SV=2 - [AMPD2_HUMAN]
Q5TAX3	Terminal uridylyltransferase 4 OS=Homo sapiens GN=ZCCHC11 PE=1 SV=3 - [TUT4_HUMAN]
Q8NHU6	Tudor domain-containing protein 7 OS=Homo sapiens GN=TDRD7 PE=1 SV=2 - [TDRD7_HUMAN]
P30613	Pyruvate kinase PKLR OS=Homo sapiens GN=PKLR PE=1 SV=2 - [KPYR HUMAN]
Q9Y4E6	WD repeat-containing protein 7 OS=Homo sapiens GN=WDR7 PE=1 SV=2 - [WDR7_HUMAN]
P35442	Thrombospondin-2 OS=Homo sapiens GN=THBS2 PE=1 SV=2 - [TSP2_HUMAN]
O94763	Unconventional prefoldin RPB5 interactor 1 OS=Homo sapiens GN=URI1 PE=1 SV=3 - [RMP_HUMAN]
Q7Z2Z2	Elongation factor Tu GTP-binding domain-containing protein 1 OS=Homo sapiens GN=EFTUD1 PE=1 SV=2 - [ETUD1_HUMAN]
Q96AY3	Peptidyl-prolyl cis-trans isomerase FKBP10 OS=Homo sapiens GN=FKBP10 PE=1 SV=1 - [FKB10_HUMAN]
Q96HP0	Dedicator of cytokinesis protein 6 OS=Homo sapiens GN=DOCK6 PE=1 SV=3 - [DOCK6_HUMAN]
Q9UMR5	Lysosomal thioesterase PPT2 OS=Homo sapiens GN=PPT2 PE=1 SV=4 - [PPT2_HUMAN]
Q8N766	ER membrane protein complex subunit 1 OS=Homo sapiens GN=EMC1 PE=1 SV=1 - [EMC1_HUMAN]
Q9NYV4	Cyclin-dependent kinase 12 OS=Homo sapiens GN=CDK12 PE=1 SV=2 - [CDK12_HUMAN]
Q32P44	Echinoderm microtubule-associated protein-like 3 OS=Homo sapiens GN=EML3 PE=1 SV=1 - [EMAL3_HUMAN]
Q9Y5A7	NEDD8 ultimate buster 1 OS=Homo sapiens GN=NUB1 PE=1 SV=2 - [NUB1_HUMAN]

Q01650	Large neutral amino acids transporter small subunit 1 OS=Homo sapiens GN=SLC7A5 PE=1 SV=2 - [LAT1 HUMAN]
Q9NZM3	Intersectin-2 OS=Homo sapiens GN=ITSN2 PE=1 SV=3 - [ITSN2_HUMAN]
P23458	Tyrosine-protein kinase JAK1 OS=Homo sapiens GN=JAK1 PE=1 SV=2 - [JAK1_HUMAN]
Q8WXG6	MAP kinase-activating death domain protein OS=Homo sapiens GN=MADD PE=1 SV=2 - [MADD_HUMAN]
Q9BYK8	Helicase with zinc finger domain 2 OS=Homo sapiens GN=HELZ2 PE=1 SV=6 - [HELZ2_HUMAN]
O15013	Rho guanine nucleotide exchange factor 10 OS=Homo sapiens GN=ARHGEF10 PE=1 SV=4 - [ARHGA_HUMAN]
O75334	Liprin-alpha-2 OS=Homo sapiens GN=PPFIA2 PE=1 SV=2 - [LIPA2_HUMAN]
A1L4H1	Soluble scavenger receptor cysteine-rich domain-containing protein SSC5D OS=Homo sapiens GN=SSC5D PE=1 SV=3 - [SRCRL_HUMAN]
P35269	General transcription factor IIF subunit 1 OS=Homo sapiens GN=GTF2F1 PE=1 SV=2 - [T2FA_HUMAN]
Q6R327	Rapamycin-insensitive companion of mTOR OS=Homo sapiens GN=RICTOR PE=1 SV=1 - [RICTR_HUMAN]
Q99996	A-kinase anchor protein 9 OS=Homo sapiens GN=AKAP9 PE=1 SV=3 - [AKAP9_HUMAN]
O43847	Nardilysin OS=Homo sapiens GN=NRD1 PE=1 SV=2 - [NRDC_HUMAN]
Q9NV96	Cell cycle control protein 50A OS=Homo sapiens GN=TMEM30A PE=1 SV=1 - [CC50A_HUMAN]
Q9Y2I7	1-phosphatidylinositol 3-phosphate 5-kinase OS=Homo sapiens GN=PIKFYVE PE=1 SV=3 - [FYV1_HUMAN]
Q8IYB7	DIS3-like exonuclease 2 OS=Homo sapiens GN=DIS3L2 PE=1 SV=4 - [DI3L2_HUMAN]
Q9NXU5	ADP-ribosylation factor-like protein 15 OS=Homo sapiens GN=ARL15 PE=1 SV=1 - [ARL15_HUMAN]
Q12955	Ankyrin-3 OS=Homo sapiens GN=ANK3 PE=1 SV=3 - [ANK3_HUMAN]
Q96JN8	Neuralized-like protein 4 OS=Homo sapiens GN=NEURL4 PE=1 SV=2 - [NEUL4_HUMAN]
Q6BDS2	UHRF1-binding protein 1 OS=Homo sapiens GN=UHRF1BP1 PE=1 SV=1 - [URFB1_HUMAN]
Q13724	Mannosyl-oligosaccharide glucosidase OS=Homo sapiens GN=MOGS PE=1 SV=5 - [MOGS_HUMAN]
Q8IV48	3'-5' exoribonuclease 1 OS=Homo sapiens GN=ERI1 PE=1 SV=3 - [ERI1_HUMAN]
O94887	FERM, RhoGEF and pleckstrin domain-containing protein 2 OS=Homo sapiens GN=FARP2 PE=1 SV=3 - [FARP2_HUMAN]
Q15223	Nectin-1 OS=Homo sapiens GN=PVRL1 PE=1 SV=3 - [PVRL1_HUMAN]
Q9HBI1	Beta-parvin OS=Homo sapiens GN=PARVB PE=1 SV=1 - [PARVB_HUMAN]
Q9ULH0	Kinase D-interacting substrate of 220 kDa OS=Homo sapiens GN=KIDINS220 PE=1 SV=3 - [KDIS_HUMAN]
Q96RS0	Trimethylguanosine synthase OS=Homo sapiens GN=TGS1 PE=1 SV=3 - [TGS1_HUMAN]
Q8IZU2	WD repeat-containing protein 17 OS=Homo sapiens GN=WDR17 PE=2 SV=2 - [WDR17_HUMAN]
Q9Y6Q2	Stonin-1 OS=Homo sapiens GN=STON1 PE=1 SV=2 - [STON1_HUMAN]
Q8NB49	Phospholipid-transporting ATPase IG OS=Homo sapiens GN=ATP11C PE=1 SV=3 - [AT11C_HUMAN]
Q9HCK4	Roundabout homolog 2 OS=Homo sapiens GN=ROBO2 PE=1 SV=2 - [ROBO2_HUMAN]
Q659C4	La-related protein 1B OS=Homo sapiens GN=LARP1B PE=1 SV=2 - [LAR1B_HUMAN]
Q9H2G2	STE20-like serine/threonine-protein kinase OS=Homo sapiens GN=SLK PE=1 SV=1 - [SLK_HUMAN]
Q9NSE4	Isoleucine--tRNA ligase, mitochondrial OS=Homo sapiens GN=IARS2 PE=1 SV=2 - [SYIM_HUMAN]
A0FGR8	Extended synaptotagmin-2 OS=Homo sapiens GN=ESYT2 PE=1 SV=1 - [ESYT2_HUMAN]
O95714	E3 ubiquitin-protein ligase HERC2 OS=Homo sapiens GN=HERC2 PE=1 SV=2 - [HERC2_HUMAN]
Q8ND24	RING finger protein 214 OS=Homo sapiens GN=RNF214 PE=1 SV=2 - [RN214_HUMAN]
O95382	Mitogen-activated protein kinase kinase kinase 6 OS=Homo sapiens GN=MAP3K6 PE=1 SV=3 - [M3K6_HUMAN]
P08123	Collagen alpha-2(I) chain OS=Homo sapiens GN=COL1A2 PE=1 SV=7 - [CO1A2_HUMAN]
P31327	Carbamoyl-phosphate synthase [ammonia], mitochondrial OS=Homo sapiens GN=CPS1 PE=1 SV=2 - [CPSM_HUMAN]
Q9UPW5	Cytosolic carboxypeptidase 1 OS=Homo sapiens GN=AGTPBP1 PE=1 SV=3 - [CBPC1_HUMAN]
Q6P2P2	Putative protein arginine N-methyltransferase 9 OS=Homo sapiens GN=PRMT9 PE=2 SV=1 - [ANM9_HUMAN]
Q9UPR0	Inactive phospholipase C-like protein 2 OS=Homo sapiens GN=PLCL2 PE=1 SV=2 - [PLCL2_HUMAN]
Q9NZN5	Rho guanine nucleotide exchange factor 12 OS=Homo sapiens GN=ARHGEF12 PE=1 SV=1 - [ARHGC_HUMAN]
O43251	RNA binding protein fox-1 homolog 2 OS=Homo sapiens GN=RBFOX2 PE=1 SV=3 - [RFOX2_HUMAN]
Q13459	Unconventional myosin-IXb OS=Homo sapiens GN=MYO9B PE=1 SV=3 - [MYO9B_HUMAN]
P36406	E3 ubiquitin-protein ligase TRIM23 OS=Homo sapiens GN=TRIM23 PE=1 SV=1 - [TRI23_HUMAN]
Q66K64	DDB1- and CUL4-associated factor 15 OS=Homo sapiens GN=DCAF15 PE=1 SV=1 - [DCA15_HUMAN]
Q96BR1	Serine/threonine-protein kinase Sgk3 OS=Homo sapiens GN=SGK3 PE=1 SV=1 - [SGK3_HUMAN]
Q16352	Alpha-internexin OS=Homo sapiens GN=INA PE=1 SV=2 - [AINX_HUMAN]
O43683	Mitotic checkpoint serine/threonine-protein kinase BUB1 OS=Homo sapiens GN=BUB1 PE=1 SV=1 - [BUB1 HUMAN]
Q9NVM4	Protein arginine N-methyltransferase 7 OS=Homo sapiens GN=PRMT7 PE=1 SV=1 - [ANM7_HUMAN]
Q96S97	Myeloid-associated differentiation marker OS=Homo sapiens GN=MYADM PE=1 SV=2 - [MYADM_HUMAN]
Q7Z4S6	Kinesin-like protein KIF21A OS=Homo sapiens GN=KIF21A PE=1 SV=2 - [KI21A_HUMAN]
Q9H1E3	Nuclear ubiquitous casein and cyclin-dependent kinase substrate 1 OS=Homo sapiens GN=NUCKS1 PE=1 SV=1 - [NUCKS_HUMAN]
Q9HA65	TBC1 domain family member 17 OS=Homo sapiens GN=TBC1D17 PE=1 SV=2 - [TBC17_HUMAN]
Q14161	ARF GTPase-activating protein GIT2 OS=Homo sapiens GN=GIT2 PE=1 SV=2 - [GIT2_HUMAN]
Q9H892	Tetratricopeptide repeat protein 12 OS=Homo sapiens GN=TTC12 PE=1 SV=2 - [TTC12_HUMAN]
Q9P2K5	Myelin expression factor 2 OS=Homo sapiens GN=MYEF2 PE=1 SV=3 - [MYEF2_HUMAN]
Q6P9A2	Polypeptide N-acetylgalactosaminyltransferase 18 OS=Homo sapiens GN=GALNT18 PE=2 SV=2 - [GLT18_HUMAN]
Q86UX2	Inter-alpha-trypsin inhibitor heavy chain H5 OS=Homo sapiens GN=ITIH5 PE=2 SV=2 - [ITIH5_HUMAN]
Q9UPM8	AP-4 complex subunit epsilon-1 OS=Homo sapiens GN=AP4E1 PE=1 SV=2 - [AP4E1_HUMAN]
Q9H0K6	Pseudouridylate synthase 7 homolog-like protein OS=Homo sapiens GN=PUS7L PE=1 SV=1 - [PUS7L_HUMAN]
Q8TDJ6	DmX-like protein 2 OS=Homo sapiens GN=DMXL2 PE=1 SV=2 - [DMXL2 HUMAN]
P00488	Coagulation factor XIII A chain OS=Homo sapiens GN=F13A1 PE=1 SV=4 - [F13A_HUMAN]
Q9NQH7	Probable Xaa-Pro aminopeptidase 3 OS=Homo sapiens GN=XPNPEP3 PE=1 SV=1 - [XPP3_HUMAN]
Q9NXV6	CDKN2A-interacting protein OS=Homo sapiens GN=CDKN2AIP PE=1 SV=3 - [CARF_HUMAN]
Q9UHV7	Mediator of RNA polymerase II transcription subunit 13 OS=Homo sapiens GN=MED13 PE=1 SV=3 - [MED13_HUMAN]
Q8TEM1	Nuclear pore membrane glycoprotein 210 OS=Homo sapiens GN=NUP210 PE=1 SV=3 - [PO210_HUMAN]
P12111	Collagen alpha-3(VI) chain OS=Homo sapiens GN=COL6A3 PE=1 SV=5 - [CO6A3_HUMAN]
Q8NBN3	Transmembrane protein 87A OS=Homo sapiens GN=TMEM87A PE=1 SV=3 - [TM87A_HUMAN]
Q15811	Intersectin-1 OS=Homo sapiens GN=ITSN1 PE=1 SV=3 - [ITSN1_HUMAN]
O14647	Chromodomain-helicase-DNA-binding protein 2 OS=Homo sapiens GN=CHD2 PE=1 SV=2 - [CHD2_HUMAN]
Q13371	Phosducin-like protein OS=Homo sapiens GN=PDCL PE=1 SV=3 - [PHLP_HUMAN]
P04114	Apolipoprotein B-100 OS=Homo sapiens GN=APOB PE=1 SV=2 - [APOB HUMAN]
Q96RK4	Bardet-Biedl syndrome 4 protein OS=Homo sapiens GN=BBS4 PE=1 SV=2 - [BBS4_HUMAN]
Q96JG8	Melanoma-associated antigen D4 OS=Homo sapiens GN=MAGED4 PE=1 SV=3 - [MAGD4_HUMAN]
O15066	Kinesin-like protein KIF3B OS=Homo sapiens GN=KIF3B PE=1 SV=1 - [KIF3B_HUMAN]
Q92954	Proteoglycan 4 OS=Homo sapiens GN=PRG4 PE=1 SV=2 - [PRG4_HUMAN]
P46937	Transcriptional coactivator YAP1 OS=Homo sapiens GN=YAP1 PE=1 SV=2 - [YAP1_HUMAN]
Q8NG31	Protein CASC5 OS=Homo sapiens GN=CASC5 PE=1 SV=3 - [CASC5_HUMAN]
Q9H3C7	Gametogenetin-binding protein 2 OS=Homo sapiens GN=GGNBP2 PE=1 SV=1 - [GGNB2_HUMAN]
Q86WG5	Myotubularin-related protein 13 OS=Homo sapiens GN=SBF2 PE=1 SV=1 - [MTMRD_HUMAN]
P20742	Pregnancy zone protein OS=Homo sapiens GN=PZP PE=1 SV=4 - [PZP HUMAN]
Q8WX93	Palladin OS=Homo sapiens GN=PALLD PE=1 SV=3 - [PALLD_HUMAN]
Q9NVI7	ATPase family AAA domain-containing protein 3A OS=Homo sapiens GN=ATAD3A PE=1 SV=2 - [ATD3A_HUMAN]
Q9UPY5	Cystine/glutamate transporter OS=Homo sapiens GN=SLC7A11 PE=1 SV=1 - [XCT_HUMAN]
Q9UBW7	Zinc finger MYM-type protein 2 OS=Homo sapiens GN=ZMYM2 PE=1 SV=1 - [ZMYM2_HUMAN]
Q07157	Tight junction protein ZO-1 OS=Homo sapiens GN=TJP1 PE=1 SV=3 - [ZO1_HUMAN]
Q7RTP6	Protein-methionine sulfoxide oxidase MICAL3 OS=Homo sapiens GN=MICAL3 PE=1 SV=2 - [MICA3_HUMAN]
Q7Z3E5	LisH domain-containing protein ARMC9 OS=Homo sapiens GN=ARMC9 PE=1 SV=2 - [ARMC9_HUMAN]
P08069	Insulin-like growth factor 1 receptor OS=Homo sapiens GN=IGF1R PE=1 SV=1 - [IGF1R_HUMAN]
Q96PU8	Protein quaking OS=Homo sapiens GN=QKI PE=1 SV=1 - [QKI_HUMAN]
Q9BXW6	Oxysterol-binding protein-related protein 1 OS=Homo sapiens GN=OSBPL1A PE=1 SV=2 - [OSBL1_HUMAN]
O75592	E3 ubiquitin-protein ligase MYCBP2 OS=Homo sapiens GN=MYCBP2 PE=1 SV=3 - [MYCB2_HUMAN]
O75084	Frizzled-7 OS=Homo sapiens GN=FZD7 PE=1 SV=2 - [FZD7_HUMAN]
Q8WUF5	RelA-associated inhibitor OS=Homo sapiens GN=PPP1R13L PE=1 SV=4 - [IASPP_HUMAN]
P46100	Transcriptional regulator ATRX OS=Homo sapiens GN=ATRX PE=1 SV=5 - [ATRX_HUMAN]
Q5TKA1	Protein lin-9 homolog OS=Homo sapiens GN=LIN9 PE=1 SV=1 - [LIN9_HUMAN]
Q8WXA9	Splicing regulatory glutamine/lysine-rich protein 1 OS=Homo sapiens GN=SREK1 PE=1 SV=1 - [SREK1_HUMAN]
Q96GX5	Serine/threonine-protein kinase greatwall OS=Homo sapiens GN=MASTL PE=1 SV=1 - [GWL_HUMAN]
O43896	Kinesin-like protein KIF1C OS=Homo sapiens GN=KIF1C PE=1 SV=3 - [KIF1C_HUMAN]
Q8TCT8	Signal peptide peptidase-like 2A OS=Homo sapiens GN=SPPL2A PE=1 SV=2 - [SPP2A_HUMAN]
Q9C0J8	pre-mRNA 3' end processing protein WDR33 OS=Homo sapiens GN=WDR33 PE=1 SV=2 - [WDR33_HUMAN]
Q76LX8	A disintegrin and metalloproteinase with thrombospondin motifs 13 OS=Homo sapiens GN=ADAMTS13 PE=1 SV=1 - [ATS13_HUMAN]
Q01484	Ankyrin-2 OS=Homo sapiens GN=ANK2 PE=1 SV=4 - [ANK2_HUMAN]
Q13099	Intraflagellar transport protein 88 homolog OS=Homo sapiens GN=IFT88 PE=2 SV=2 - [IFT88_HUMAN]
Q6ZNJ1	Neurobeachin-like protein 2 OS=Homo sapiens GN=NBEAL2 PE=1 SV=2 - [NBEL2_HUMAN]
P05107	Integrin beta-2 OS=Homo sapiens GN=ITGB2 PE=1 SV=2 - [ITB2_HUMAN]
Q9P2D1	Chromodomain-helicase-DNA-binding protein 7 OS=Homo sapiens GN=CHD7 PE=1 SV=3 - [CHD7_HUMAN]
P14780	Matrix metalloproteinase-9 OS=Homo sapiens GN=MMP9 PE=1 SV=3 - [MMP9_HUMAN]
Q13433	Zinc transporter ZIP6 OS=Homo sapiens GN=SLC39A6 PE=1 SV=3 - [S39A6_HUMAN]
Q9H2X0	Chordin OS=Homo sapiens GN=CHRD PE=1 SV=2 - [CHRD_HUMAN]
O14686	Histone-lysine N-methyltransferase 2D OS=Homo sapiens GN=KMT2D PE=1 SV=2 - [KMT2D_HUMAN]
Q9UPN6	Protein SCAF8 OS=Homo sapiens GN=SCAF8 PE=1 SV=1 - [SCAF8_HUMAN]
P29400	Collagen alpha-5(IV) chain OS=Homo sapiens GN=COL4A5 PE=1 SV=2 - [CO4A5_HUMAN]

A1A4S6	Rho GTPase-activating protein 10 OS=Homo sapiens GN=ARHGAP10 PE=1 SV=1 - [RHG10_HUMAN]
Q9P2K2	Thioredoxin domain-containing protein 16 OS=Homo sapiens GN=TXNDC16 PE=2 SV=4 - [TXD16_HUMAN]
Q96PV6	Leukocyte receptor cluster member 8 OS=Homo sapiens GN=LENG8 PE=1 SV=2 - [LENG8_HUMAN]
Q5TH69	Brefeldin A-inhibited guanine nucleotide-exchange protein 3 OS=Homo sapiens GN=ARFGEF3 PE=1 SV=3 - [BIG3_HUMAN]
Q9H0E9	Bromodomain-containing protein 8 OS=Homo sapiens GN=BRD8 PE=1 SV=2 - [BRD8_HUMAN]
Q9NZP5	Olfactory receptor 5AC2 OS=Homo sapiens GN=OR5AC2 PE=3 SV=2 - [O5AC2_HUMAN]
Q9H410	Kinetochore-associated protein DSN1 homolog OS=Homo sapiens GN=DSN1 PE=1 SV=2 - [DSN1_HUMAN]
P22455	Fibroblast growth factor receptor 4 OS=Homo sapiens GN=FGFR4 PE=1 SV=2 - [FGFR4_HUMAN]
Q8NF91	Nesprin-1 OS=Homo sapiens GN=SYNE1 PE=1 SV=4 - [SYNE1_HUMAN]
P22105	Tenascin-X OS=Homo sapiens GN=TNXB PE=1 SV=3 - [TENX_HUMAN]
Q8WXE9	Stonin-2 OS=Homo sapiens GN=STON2 PE=1 SV=1 - [STON2_HUMAN]
P09603	Macrophage colony-stimulating factor 1 OS=Homo sapiens GN=CSF1 PE=1 SV=2 - [CSF1_HUMAN]
Q01955	Collagen alpha-3(IV) chain OS=Homo sapiens GN=COL4A3 PE=1 SV=3 - [CO4A3_HUMAN]
Q03001	Dystonin OS=Homo sapiens GN=DST PE=1 SV=4 - [DYST_HUMAN]
Q15154	Pericentriolar material 1 protein OS=Homo sapiens GN=PCM1 PE=1 SV=4 - [PCM1_HUMAN]
Q2T9J0	Peroxisomal leader peptide-processing protease OS=Homo sapiens GN=TYSND1 PE=1 SV=3 - [TYSD1_HUMAN]
A0JNW5	UHRF1-binding protein 1-like OS=Homo sapiens GN=UHRF1BP1L PE=1 SV=2 - [UH1BL_HUMAN]
Q03692	Collagen alpha-1(X) chain OS=Homo sapiens GN=COL10A1 PE=1 SV=2 - [COAA1_HUMAN]
Q96RN5	Mediator of RNA polymerase II transcription subunit 15 OS=Homo sapiens GN=MED15 PE=1 SV=2 - [MED15_HUMAN]
Q2M2I8	AP2-associated protein kinase 1 OS=Homo sapiens GN=AAK1 PE=1 SV=3 - [AAK1_HUMAN]
Q5VZL5	Zinc finger MYM-type protein 4 OS=Homo sapiens GN=ZMYM4 PE=1 SV=1 - [ZMYM4_HUMAN]
O95197	Reticulon-3 OS=Homo sapiens GN=RTN3 PE=1 SV=2 - [RTN3_HUMAN]
O75691	Small subunit processome component 20 homolog OS=Homo sapiens GN=UTP20 PE=1 SV=3 - [UTP20_HUMAN]
P11169	Solute carrier family 2, facilitated glucose transporter member 3 OS=Homo sapiens GN=SLC2A3 PE=2 SV=1 - [GTR3_HUMAN]
Q9ULG1	DNA helicase INO80 OS=Homo sapiens GN=INO80 PE=1 SV=2 - [INO80 HUMAN]
P13671	Complement component C6 OS=Homo sapiens GN=C6 PE=1 SV=3 - [CO6 HUMAN]
Q96JB3	Hypermethylated in cancer 2 protein OS=Homo sapiens GN=HIC2 PE=1 SV=2 - [HIC2_HUMAN]
Q49MI3	Ceramide kinase-like protein OS=Homo sapiens GN=CERKL PE=1 SV=1 - [CERKL_HUMAN]
Q9P2N6	KAT8 regulatory NSL complex subunit 3 OS=Homo sapiens GN=KANSL3 PE=1 SV=2 - [KANL3_HUMAN]
Q9BV73	Centrosome-associated protein CEP250 OS=Homo sapiens GN=CEP250 PE=1 SV=2 - [CP250_HUMAN]
Q9UPQ9	Trinucleotide repeat-containing gene 6B protein OS=Homo sapiens GN=TNRC6B PE=1 SV=4 - [TNR6B_HUMAN]
Q9H2D6	TRIO and F-actin-binding protein OS=Homo sapiens GN=TRIOBP PE=1 SV=3 - [TARA_HUMAN]
Q9NZT2	Opioid growth factor receptor OS=Homo sapiens GN=OGFR PE=1 SV=3 - [OGFR_HUMAN]
Q9NYU1	UDP-glucose:glycoprotein glucosyltransferase 2 OS=Homo sapiens GN=UGGT2 PE=1 SV=4 - [UGGG2_HUMAN]
Q9NQT8	Kinesin-like protein KIF13B OS=Homo sapiens GN=KIF13B PE=1 SV=2 - [KI13B_HUMAN]
Q9Y618	Nuclear receptor corepressor 2 OS=Homo sapiens GN=NCOR2 PE=1 SV=2 - [NCOR2_HUMAN]
Q9NZM1	Myoferlin OS=Homo sapiens GN=MYOF PE=1 SV=1 - [MYOF_HUMAN]
P35251	Replication factor C subunit 1 OS=Homo sapiens GN=RFC1 PE=1 SV=4 - [RFC1_HUMAN]
P11532	Dystrophin OS=Homo sapiens GN=DMD PE=1 SV=3 - [DMD_HUMAN]
Q5TZA2	Rootletin OS=Homo sapiens GN=CROCC PE=1 SV=1 - [CROCC_HUMAN]
Q9H4D0	Calsyntenin-2 OS=Homo sapiens GN=CLSTN2 PE=1 SV=2 - [CSTN2_HUMAN]
P00451	Coagulation factor VIII OS=Homo sapiens GN=F8 PE=1 SV=1 - [FA8 HUMAN]
Q96F46	Interleukin-17 receptor A OS=Homo sapiens GN=IL17RA PE=1 SV=2 - [I17RA_HUMAN]
