# Supplementary material for: Identification and Characterization of 293T Cell-Derived Exosomes by Profiling the Protein, mRNA and MicroRNA Components
Source: PLoS One. 2016 Sep 20;11(9):e0163043. doi: 10.1371/journal.pone.0163043 (PMC5029934; doi:10.1371/journal.pone.0163043)
Supplement: S2 Table — (PDF) [file pone.0163043.s003.pdf]

ENTREZ_GENE_ID	Name	Species
91	activin A receptor, type IB	Homo sapiens
1509	cathepsin D	Homo sapiens
23204	ADP-ribosylation factor-like 6 interacting protein 1	Homo sapiens
26986	poly(A) binding protein, cytoplasmic pseudogene 5; poly(A) binding protein, cytoplasmic 1	Homo sapiens
23277	KIAA0664	Homo sapiens
89853	family with sequence similarity 125, member B	Homo sapiens
79892	chromosome 10 open reading frame 119	Homo sapiens
79809	tetratricopeptide repeat domain 21B	Homo sapiens
2321	fms-related tyrosine kinase 1 (vascular endothelial growth factor/vascular permeability factor receptor)	Homo sapiens
5118	procollagen C-endopeptidase enhancer	Homo sapiens
5985	replication factor C (activator 1) 5, 36.5kDa	Homo sapiens
57510	exportin 5	Homo sapiens
4121	mannosidase, alpha, class 1A, member 1	Homo sapiens
2027	enolase 3 (beta, muscle)	Homo sapiens
29094	galectin-related protein	Homo sapiens
23608	makorin ring finger protein pseudogene 6; makorin ring finger protein 1	Homo sapiens
2153	coagulation factor V (proaccelerin, labile factor)	Homo sapiens
53944	casein kinase 1, gamma 1	Homo sapiens
22826	DnaJ (Hsp40) homolog, subfamily C, member 8	Homo sapiens
7153	topoisomerase (DNA) II alpha 170kDa	Homo sapiens
128866	chromatin modifying protein 4B	Homo sapiens
3066	histone deacetylase 2	Homo sapiens
9747	family with sequence similarity 115, member A; family with sequence similarity 115, member B (pseudogene)	Homo sapiens
5515	protein phosphatase 2 (formerly 2A), catalytic subunit, alpha isoform	Homo sapiens
128	alcohol dehydrogenase 5 (class III), chi polypeptide, pseudogene 4; alcohol dehydrogenase 5 (class III), chi polypeptide	Homo sapiens
2734	golgi apparatus protein 1	Homo sapiens
10533	ATG7 autophagy related 7 homolog (S. cerevisiae)	Homo sapiens
23646	phospholipase D family, member 3	Homo sapiens
6201	ribosomal protein S7; ribosomal protein S7 pseudogene 11; ribosomal protein S7 pseudogene 4; ribosomal protein S7 pseudogene 10	Homo sapiens
8655	dynein, light chain, LC8-type 1	Homo sapiens
83986	integrin alpha FG-GAP repeat containing 3	Homo sapiens
7205	thyroid hormone receptor interactor 6	Homo sapiens
818	calcium/calmodulin-dependent protein kinase II gamma	Homo sapiens
5685	proteasome (prosome, macropain) subunit, alpha type, 4	Homo sapiens
81567	thioredoxin domain containing 5 (endoplasmic reticulum); muted homolog (mouse)	Homo sapiens
63915	thioredoxin domain containing 5 (endoplasmic reticulum); muted homolog (mouse)	Homo sapiens
4176	minichromosome maintenance complex component 7	Homo sapiens
55968	NSFL1 (p97) cofactor (p47)	Homo sapiens
9328	general transcription factor IIIC, polypeptide 5, 63kDa	Homo sapiens
8614	stanniocalcin 2	Homo sapiens
388	ras homolog gene family, member B	Homo sapiens
64175	leucine proline-enriched proteoglycan (leprecan) 1	Homo sapiens
9254	calcium channel, voltage-dependent, alpha 2/delta subunit 2	Homo sapiens
55571	chromosome 2 open reading frame 29	Homo sapiens
3035	histidyl-tRNA synthetase	Homo sapiens
79720	vacuolar protein sorting 37 homolog B (S. cerevisiae)	Homo sapiens
51194	importin 11	Homo sapiens
10291	splicing factor 3a, subunit 1, 120kDa	Homo sapiens
27000	DnaJ (Hsp40) homolog, subfamily C, member 2	Homo sapiens
55119	PRP38 pre-mRNA processing factor 38 (yeast) domain containing B	Homo sapiens
55327	lin-7 homolog C (C. elegans)	Homo sapiens
79077	dCTP pyrophosphatase 1	Homo sapiens
5965	RecQ protein-like (DNA helicase Q1-like)	Homo sapiens
139818	dedicator of cytokinesis 11	Homo sapiens
65986	zinc finger and BTB domain containing 10	Homo sapiens
133418	embigin homolog (mouse)	Homo sapiens
5339	similar to Plectin 1 (PLTN) (PCN) (Hemidesmosomal protein 1) (HD1); plectin 1, intermediate filament binding protein 500kDa	Homo sapiens
25980	chromosome 20 open reading frame 4	Homo sapiens
6730	signal recognition particle 68kDa	Homo sapiens
31	acetyl-Coenzyme A carboxylase alpha	Homo sapiens
115207	potassium channel tetramerisation domain containing 12	Homo sapiens
23335	WD repeat domain 7	Homo sapiens
2200	fibrillin 1	Homo sapiens
9230	RAB11B, member RAS oncogene family	Homo sapiens
6624	fascin homolog 1, actin-bundling protein (Strongylocentrotus purpuratus)	Homo sapiens
966	CD59 molecule, complement regulatory protein	Homo sapiens
23181	DIP2 disco-interacting protein 2 homolog A (Drosophila)	Homo sapiens
3799	kinesin family member 5B	Homo sapiens
51606	ATPase, H+ transporting, lysosomal 50/57kDa, V1 subunit H	Homo sapiens
5495	protein phosphatase 1B (formerly 2C), magnesium-dependent, beta isoform	Homo sapiens
10383	tubulin, beta 2C	Homo sapiens
55660	PRP40 pre-mRNA processing factor 40 homolog A (S. cerevisiae)	Homo sapiens
646949	ribosomal protein L23 pseudogene 6; ribosomal protein L23	Homo sapiens
9349	ribosomal protein L23 pseudogene 6; ribosomal protein L23	Homo sapiens
9696	ciliary rootlet coiled-coil, rootletin	Homo sapiens
51602	NOP58 ribonucleoprotein homolog (yeast)	Homo sapiens
9902	mannose receptor, C type 2	Homo sapiens
26999	cytoplasmic FMR1 interacting protein 2	Homo sapiens
80232	WD repeat domain 26	Homo sapiens
10631	periostin, osteoblast specific factor	Homo sapiens

64151	non-SMC condensin I complex, subunit G	Homo sapiens
10527	importin 7	Homo sapiens
140885	signal-regulatory protein alpha	Homo sapiens
392	Rho GTPase activating protein 1	Homo sapiens
528	ATPase, H+ transporting, lysosomal 42kDa, V1 subunit C1	Homo sapiens
200081	taxilin alpha	Homo sapiens
8662	eukaryotic translation initiation factor 3, subunit B	Homo sapiens
79180	EF-hand domain family, member D2	Homo sapiens
6130	ribosomal protein L7a pseudogene 70; ribosomal protein L7a; ribosomal protein L7a pseudogene 30; ribosomal protein L7a pseudogene 66; ribosomal protein L7a pseudogene 27; ribosomal protein L7a pseudogene 11; ribosomal protein L7a pseudogene 62	Homo sapiens
4023	lipoprotein lipase	Homo sapiens
1315	coatomer protein complex, subunit beta 1	Homo sapiens
25939	SAM domain and HD domain 1	Homo sapiens
3913	laminin, beta 2 (laminin S)	Homo sapiens
22837	COBL-like 1	Homo sapiens
7353	ubiquitin fusion degradation 1 like (yeast)	Homo sapiens
2810	stratifin	Homo sapiens
55506	H2A histone family, member Y2	Homo sapiens
6009	Ras homolog enriched in brain	Homo sapiens
5908	RAP1B, member of RAS oncogene family	Homo sapiens
6161	small nucleolar RNA, H/ACA box 7A; small nucleolar RNA, H/ACA box 7B; ribosomal protein L32	Homo sapiens
53834	fibroblast growth factor receptor-like 1	Homo sapiens
2767	guanine nucleotide binding protein (G protein), alpha 11 (Gq class)	Homo sapiens
84624	fibronectin type III domain containing 1	Homo sapiens
9508	ADAM metallopeptidase with thrombospondin type 1 motif, 3	Homo sapiens
345651	actin, beta-like 2	Homo sapiens
10197	proteasome (prosome, macropain) activator subunit 3 (PA28 gamma; Ki)	Homo sapiens
56474	CTP synthase II	Homo sapiens
201475	RAB12, member RAS oncogene family	Homo sapiens
23198	proteasome (prosome, macropain) activator subunit 4	Homo sapiens
5289	phosphoinositide-3-kinase, class 3	Homo sapiens
5605	mitogen-activated protein kinase kinase 2 pseudogene; mitogen-activated protein kinase kinase 2	Homo sapiens
2548	glucosidase, alpha; acid	Homo sapiens
79869	cleavage and polyadenylation specific factor 7, 59kDa	Homo sapiens
5917	arginyl-tRNA synthetase	Homo sapiens
3915	laminin, gamma 1 (formerly LAMB2)	Homo sapiens
55696	RNA binding motif protein 22	Homo sapiens
5935	RNA binding motif (RNP1, RRM) protein 3	Homo sapiens
11054	opioid growth factor receptor	Homo sapiens
57132	chromatin modifying protein 1B	Homo sapiens
1152	creatine kinase, brain	Homo sapiens
249	alkaline phosphatase, liver/bone/kidney	Homo sapiens
10452	translocase of outer mitochondrial membrane 40 homolog (yeast)	Homo sapiens
118460	exosome component 6	Homo sapiens
55201	microtubule-associated protein 1S	Homo sapiens
7529	tyrosine 3-monooxygenase/tryptophan 5-monooxygenase activation protein, beta polypeptide	Homo sapiens
7919	HLA-B associated transcript 1	Homo sapiens
9351	solute carrier family 9 (sodium/hydrogen exchanger), member 3 regulator 2	Homo sapiens
11127	kinesin family member 3A	Homo sapiens
57470	leucine rich repeat containing 47	Homo sapiens
57650	KIAA1524	Homo sapiens
3032	hydroxyacyl-Coenzyme A dehydrogenase/3-ketoacyl-Coenzyme A thiolase/enoyl-Coenzyme A hydratase (trifunctional protein), beta subunit	Homo sapiens
10867	tetraspanin 9	Homo sapiens
2805	glutamic-oxaloacetic transaminase 1, soluble (aspartate aminotransferase 1)	Homo sapiens
51340	crooked neck pre-mRNA splicing factor-like 1 (Drosophila)	Homo sapiens
10672	guanine nucleotide binding protein (G protein), alpha 13	Homo sapiens
112574	sorting nexin 18	Homo sapiens
372	archain 1	Homo sapiens
6601	SWI/SNF related, matrix associated, actin dependent regulator of chromatin, subfamily c, member 2	Homo sapiens
29883	CCR4-NOT transcription complex, subunit 7	Homo sapiens
10049	DnaJ (Hsp40) homolog, subfamily B, member 6	Homo sapiens
5520	protein phosphatase 2 (formerly 2A), regulatory subunit B, alpha isoform	Homo sapiens
7965	aminoacyl tRNA synthetase complex-interacting multifunctional protein 2; stromal antigen 3-like 3	Homo sapiens
57674	ring finger protein 213	Homo sapiens
57181	solute carrier family 39 (zinc transporter), member 10	Homo sapiens
2969	general transcription factor II, i; general transcription factor II, i, pseudogene	Homo sapiens
103	adenosine deaminase, RNA-specific	Homo sapiens
26229	beta-1,3-glucuronyltransferase 3 (glucuronosyltransferase I)	Homo sapiens
3183	heterogeneous nuclear ribonucleoprotein C (C1/C2)	Homo sapiens
27101	similar to calcyclin binding protein; calcyclin binding protein	Homo sapiens
10262	splicing factor 3b, subunit 4, 49kDa	Homo sapiens
341640	FRAS1 related extracellular matrix protein 2	Homo sapiens
29896	transformer 2 alpha homolog (Drosophila)	Homo sapiens
84844	PHD finger protein 5A	Homo sapiens
2597	glyceraldehyde-3-phosphate dehydrogenase-like 6; hypothetical protein LOC100133042; glyceraldehyde-3-phosphate dehydrogenase	Homo sapiens
54881	testis expressed 10	Homo sapiens
1459	casein kinase 2, alpha prime polypeptide	Homo sapiens
54545	myotubularin related protein 12	Homo sapiens
55898	unc-45 homolog A (C. elegans)	Homo sapiens
2580	cyclin G associated kinase	Homo sapiens
1073	cofilin 2 (muscle)	Homo sapiens

6157	ribosomal protein L27a	Homo sapiens
3337	DnaJ (Hsp40) homolog, subfamily B, member 1	Homo sapiens
6426	splicing factor, arginine/serine-rich 1	Homo sapiens
51495	protein tyrosine phosphatase-like A domain containing 1	Homo sapiens
9371	kinesin family member 3B	Homo sapiens
57819	LSM2 homolog, U6 small nuclear RNA associated (S. cerevisiae)	Homo sapiens
10422	UBA domain containing 1	Homo sapiens
63935	PDX1 C-terminal inhibiting factor 1	Homo sapiens
201627	family with sequence similarity 116, member A	Homo sapiens
25836	Nipped-B homolog (Drosophila)	Homo sapiens
7150	topoisomerase (DNA) I	Homo sapiens
3323	heat shock protein 90kDa alpha (cytosolic), class A member 4 (pseudogene)	Homo sapiens
2730	glutamate-cysteine ligase, modifier subunit	Homo sapiens
55860	actin-related protein 10 homolog (S. cerevisiae)	Homo sapiens
55234	smu-1 suppressor of mec-8 and unc-52 homolog (C. elegans)	Homo sapiens
10606	phosphoribosylaminoimidazole carboxylase, phosphoribosylaminoimidazole succinocarboxamide synthetase	Homo sapiens
6209	ribosomal protein S15 pseudogene 5; ribosomal protein S15	Homo sapiens
6897	threonyl-tRNA synthetase	Homo sapiens
445582	POTE ankyrin domain family, member E	Homo sapiens
57491	aryl-hydrocarbon receptor repressor; programmed cell death 6	Homo sapiens
10016	aryl-hydrocarbon receptor repressor; programmed cell death 6	Homo sapiens
64853	hypothetical LOC653631; hypothetical LOC646050; hypothetical LOC646890; axin interactor, dorsalization associated	Homo sapiens
348995	nucleoporin 43kDa	Homo sapiens
2042	EPH receptor A3	Homo sapiens
84284	chromosome 1 open reading frame 57	Homo sapiens
5627	protein S (alpha)	Homo sapiens
715	complement component 1, r subcomponent	Homo sapiens
55718	polymerase (RNA) III (DNA directed) polypeptide E (80kD)	Homo sapiens
6455	SH3-domain GRB2-like 1	Homo sapiens
23214	exportin 6	Homo sapiens
51191	hect domain and RLD 5	Homo sapiens
5903	RAN binding protein 2	Homo sapiens
11120	butyrophilin, subfamily 2, member A1	Homo sapiens
3043	hemoglobin, beta	Homo sapiens
23376	KIAA0776	Homo sapiens
27043	proline, glutamate and leucine rich protein 1	Homo sapiens
3006	histone cluster 1, H1c	Homo sapiens
6092	roundabout, axon guidance receptor, homolog 2 (Drosophila)	Homo sapiens
102	ADAM metallopeptidase domain 10	Homo sapiens
57187	THO complex 2	Homo sapiens
8664	eukaryotic translation initiation factor 3, subunit D	Homo sapiens
9181	Rho/Rac guanine nucleotide exchange factor (GEF) 2	Homo sapiens
54431	DnaJ (Hsp40) homolog, subfamily C, member 10	Homo sapiens
5223	phosphoglycerate mutase 1 (brain)	Homo sapiens
54888	NOL1/NOP2/Sun domain family, member 2	Homo sapiens
8894	eukaryotic translation initiation factor 2, subunit 2 beta, 38kDa	Homo sapiens
10494	serine/threonine kinase 25 (STE20 homolog, yeast)	Homo sapiens
9344	TAO kinase 2	Homo sapiens
9520	hypothetical protein FLJ11822; aminopeptidase puromycin sensitive	Homo sapiens
3182	heterogeneous nuclear ribonucleoprotein A/B	Homo sapiens
9184	budding uninhibited by benzimidazoles 3 homolog (yeast)	Homo sapiens
7415	valosin-containing protein	Homo sapiens
23043	TRAF2 and NCK interacting kinase	Homo sapiens
8729	golgi-specific brefeldin A resistant guanine nucleotide exchange factor 1	Homo sapiens
967	CD63 molecule	Homo sapiens
3655	integrin, alpha 6	Homo sapiens
2760	GM2 ganglioside activator	Homo sapiens
826	calpain, small subunit 1	Homo sapiens
535	ATPase, H+ transporting, lysosomal V0 subunit a1	Homo sapiens
10576	chaperonin containing TCP1, subunit 2 (beta)	Homo sapiens
1019	cyclin-dependent kinase 4	Homo sapiens
10564	ADP-ribosylation factor guanine nucleotide-exchange factor 2 (brefeldin A-inhibited)	Homo sapiens
5479	peptidylprolyl isomerase B (cyclophilin B)	Homo sapiens
57610	RAN binding protein 10	Homo sapiens
10910	SGT1, suppressor of G2 allele of SKP1 (S. cerevisiae)	Homo sapiens
81542	thioredoxin-related transmembrane protein 1	Homo sapiens
5527	protein phosphatase 2, regulatory subunit B', gamma isoform	Homo sapiens
60678	eukaryotic elongation factor, selenocysteine-tRNA-specific	Homo sapiens
9114	ATPase, H+ transporting, lysosomal 38kDa, V0 subunit d1	Homo sapiens
7408	vasodilator-stimulated phosphoprotein	Homo sapiens
9578	CDC42 binding protein kinase beta (DMPK-like)	Homo sapiens
7332	ubiquitin-conjugating enzyme E2L 3	Homo sapiens
10528	NOP56 ribonucleoprotein homolog (yeast)	Homo sapiens
11315	Parkinson disease (autosomal recessive, early onset) 7	Homo sapiens
2773	guanine nucleotide binding protein (G protein), alpha inhibiting activity polypeptide 3	Homo sapiens
5692	proteasome (prosome, macropain) subunit, beta type, 4	Homo sapiens
2052	epoxide hydrolase 1, microsomal (xenobiotic)	Homo sapiens
22824	heat shock 70kDa protein 4-like	Homo sapiens
27037	TRM2 tRNA methyltransferase 2 homolog A (S. cerevisiae)	Homo sapiens
51433	anaphase promoting complex subunit 5	Homo sapiens
5563	protein kinase, AMP-activated, alpha 2 catalytic subunit	Homo sapiens
4048	leukotriene A4 hydrolase	Homo sapiens
2157	coagulation factor VIII, procoagulant component	Homo sapiens
5127	PCTAIRE protein kinase 1	Homo sapiens

6712	spectrin, beta, non-erythrocytic 2	Homo sapiens
975	CD81 molecule	Homo sapiens
9601	protein disulfide isomerase family A, member 4	Homo sapiens
8189	symplekin	Homo sapiens
2048	EPH receptor B2	Homo sapiens
6431	splicing factor, arginine/serine-rich 6; similar to arginine/serine-rich splicing factor 6	Homo sapiens
6147	ribosomal protein L23a pseudogene 63; ribosomal protein L23a pseudogene 75; ribosomal protein L23a pseudogene 37; ribosomal protein L23a pseudogene 65; ribosomal protein L23a pseudogene 43; ribosomal protein L23a pseudogene 44; ribosomal protein L23a	Homo sapiens
6102	retinitis pigmentosa 2 (X-linked recessive)	Homo sapiens
64837	kinesin light chain 2	Homo sapiens
3843	importin 5	Homo sapiens
537	ATPase, H+ transporting, lysosomal accessory protein 1	Homo sapiens
10767	HBS1-like (S. cerevisiae)	Homo sapiens
56829	zinc finger CCCH-type, antiviral 1	Homo sapiens
112936	vacuolar protein sorting 26 homolog B (S. pombe)	Homo sapiens
832	capping protein (actin filament) muscle Z-line, beta	Homo sapiens
7525	v-yes-1 Yamaguchi sarcoma viral oncogene homolog 1	Homo sapiens
10670	Ras-related GTP binding A	Homo sapiens
51386	eukaryotic translation initiation factor 3, subunit L	Homo sapiens
9118	internexin neuronal intermediate filament protein, alpha	Homo sapiens
3106	major histocompatibility complex, class I, C; major histocompatibility complex, class I, B	Homo sapiens
3107	major histocompatibility complex, class I, C; major histocompatibility complex, class I, B	Homo sapiens
1290	collagen, type V, alpha 2	Homo sapiens
285636	chromosome 5 open reading frame 51	Homo sapiens
1627	drebrin 1	Homo sapiens
51552	RAB14, member RAS oncogene family	Homo sapiens
7086	transketolase	Homo sapiens
2931	glycogen synthase kinase 3 alpha	Homo sapiens
9255	aminoacyl tRNA synthetase complex-interacting multifunctional protein 1	Homo sapiens
5230	phosphoglycerate kinase 1	Homo sapiens
23499	microtubule-actin crosslinking factor 1	Homo sapiens
3909	laminin, alpha 3	Homo sapiens
2159	coagulation factor X	Homo sapiens
3327	heat shock protein 90kDa alpha (cytosolic), class B member 3 (pseudogene)	Homo sapiens
51082	polymerase (RNA) I polypeptide D, 16kDa	Homo sapiens
1147	conserved helix-loop-helix ubiquitous kinase	Homo sapiens
293	solute carrier family 25 (mitochondrial carrier; adenine nucleotide translocator), member 6	Homo sapiens
50618	intersectin 2	Homo sapiens
10644	insulin-like growth factor 2 mRNA binding protein 2	Homo sapiens
51112	tetratricopeptide repeat domain 15	Homo sapiens
5305	phosphatidylinositol-5-phosphate 4-kinase, type II, alpha	Homo sapiens
91949	component of oligomeric golgi complex 7	Homo sapiens
51125	golgi autoantigen, golgin subfamily a, 7	Homo sapiens
8936	WAS protein family, member 1	Homo sapiens
1654	DEAD (Asp-Glu-Ala-Asp) box polypeptide 3, X-linked	Homo sapiens
7347	ubiquitin carboxyl-terminal esterase L3 (ubiquitin thiolesterase)	Homo sapiens
54187	N-acetylneuraminic acid synthase	Homo sapiens
1824	desmocollin 2	Homo sapiens
10615	sperm associated antigen 5	Homo sapiens
57153	solute carrier family 44, member 2	Homo sapiens
55342	spermatid perinuclear RNA binding protein	Homo sapiens
26128	KIAA1279	Homo sapiens
50848	F11 receptor	Homo sapiens
220988	heterogeneous nuclear ribonucleoprotein A3	Homo sapiens
8370	histone cluster 1, H4l; histone cluster 1, H4k; histone cluster 4, H4; histone cluster 1, H4h; histone cluster 1, H4j; histone cluster 1, H4i; histone cluster 1, H4d; histone cluster 1, H4c; histone cluster 1, H4f; histone cluster 1, H4e; histone cluster 1, H4b; histone cluster 1, H4a; histone cluster 2, H4a; histone cluster 2, H4b	Homo sapiens
554313	histone cluster 1, H4l; histone cluster 1, H4k; histone cluster 4, H4; histone cluster 1, H4h; histone cluster 1, H4j; histone cluster 1, H4i; histone cluster 1, H4d; histone cluster 1, H4c; histone cluster 1, H4f; histone cluster 1, H4e; histone cluster 1, H4b; histone cluster 1, H4a; histone cluster 2, H4a; histone cluster 2, H4b	Homo sapiens
8360	histone cluster 1, H4l; histone cluster 1, H4k; histone cluster 4, H4; histone cluster 1, H4h; histone cluster 1, H4j; histone cluster 1, H4i; histone cluster 1, H4d; histone cluster 1, H4c; histone cluster 1, H4f; histone cluster 1, H4e; histone cluster 1, H4b; histone cluster 1, H4a; histone cluster 2, H4a; histone cluster 2, H4b	Homo sapiens
8363	histone cluster 1, H4l; histone cluster 1, H4k; histone cluster 4, H4; histone cluster 1, H4h; histone cluster 1, H4j; histone cluster 1, H4i; histone cluster 1, H4d; histone cluster 1, H4c; histone cluster 1, H4f; histone cluster 1, H4e; histone cluster 1, H4b; histone cluster 1, H4a; histone cluster 2, H4a; histone cluster 2, H4b	Homo sapiens
8368	histone cluster 1, H4l; histone cluster 1, H4k; histone cluster 4, H4; histone cluster 1, H4h; histone cluster 1, H4j; histone cluster 1, H4i; histone cluster 1, H4d; histone cluster 1, H4c; histone cluster 1, H4f; histone cluster 1, H4e; histone cluster 1, H4b; histone cluster 1, H4a; histone cluster 2, H4a; histone cluster 2, H4b	Homo sapiens
8365	histone cluster 1, H4l; histone cluster 1, H4k; histone cluster 4, H4; histone cluster 1, H4h; histone cluster 1, H4j; histone cluster 1, H4i; histone cluster 1, H4d; histone cluster 1, H4c; histone cluster 1, H4f; histone cluster 1, H4e; histone cluster 1, H4b; histone cluster 1, H4a; histone cluster 2, H4a; histone cluster 2, H4b	Homo sapiens
8294	histone cluster 1, H4l; histone cluster 1, H4k; histone cluster 4, H4; histone cluster 1, H4h; histone cluster 1, H4j; histone cluster 1, H4i; histone cluster 1, H4d; histone cluster 1, H4c; histone cluster 1, H4f; histone cluster 1, H4e; histone cluster 1, H4b; histone cluster 1, H4a; histone cluster 2, H4a; histone cluster 2, H4b	Homo sapiens

8364	histone cluster 1, H4l; histone cluster 1, H4k; histone cluster 4, H4; histone cluster 1, H4h; histone cluster 1, H4j; histone cluster 1, H4i; histone cluster 1, H4d; histone cluster 1, H4c; histone cluster 1, H4f; histone cluster 1, H4e; histone cluster 1, H4b; histone cluster 1, H4a; histone cluster 2, H4a; histone cluster 2, H4b	Homo sapiens
8361	histone cluster 1, H4l; histone cluster 1, H4k; histone cluster 4, H4; histone cluster 1, H4h; histone cluster 1, H4j; histone cluster 1, H4i; histone cluster 1, H4d; histone cluster 1, H4c; histone cluster 1, H4f; histone cluster 1, H4e; histone cluster 1, H4b; histone cluster 1, H4a; histone cluster 2, H4a; histone cluster 2, H4b	Homo sapiens
8366	histone cluster 1, H4l; histone cluster 1, H4k; histone cluster 4, H4; histone cluster 1, H4h; histone cluster 1, H4j; histone cluster 1, H4i; histone cluster 1, H4d; histone cluster 1, H4c; histone cluster 1, H4f; histone cluster 1, H4e; histone cluster 1, H4b; histone cluster 1, H4a; histone cluster 2, H4a; histone cluster 2, H4b	Homo sapiens
8362	histone cluster 1, H4l; histone cluster 1, H4k; histone cluster 4, H4; histone cluster 1, H4h; histone cluster 1, H4j; histone cluster 1, H4i; histone cluster 1, H4d; histone cluster 1, H4c; histone cluster 1, H4f; histone cluster 1, H4e; histone cluster 1, H4b; histone cluster 1, H4a; histone cluster 2, H4a; histone cluster 2, H4b	Homo sapiens
121504	histone cluster 1, H4l; histone cluster 1, H4k; histone cluster 4, H4; histone cluster 1, H4h; histone cluster 1, H4j; histone cluster 1, H4i; histone cluster 1, H4d; histone cluster 1, H4c; histone cluster 1, H4f; histone cluster 1, H4e; histone cluster 1, H4b; histone cluster 1, H4a; histone cluster 2, H4a; histone cluster 2, H4b	Homo sapiens
8359	histone cluster 1, H4l; histone cluster 1, H4k; histone cluster 4, H4; histone cluster 1, H4h; histone cluster 1, H4j; histone cluster 1, H4i; histone cluster 1, H4d; histone cluster 1, H4c; histone cluster 1, H4f; histone cluster 1, H4e; histone cluster 1, H4b; histone cluster 1, H4a; histone cluster 2, H4a; histone cluster 2, H4b	Homo sapiens
8367	histone cluster 1, H4l; histone cluster 1, H4k; histone cluster 4, H4; histone cluster 1, H4h; histone cluster 1, H4j; histone cluster 1, H4i; histone cluster 1, H4d; histone cluster 1, H4c; histone cluster 1, H4f; histone cluster 1, H4e; histone cluster 1, H4b; histone cluster 1, H4a; histone cluster 2, H4a; histone cluster 2, H4b	Homo sapiens
60	actin, beta	Homo sapiens
552900	bolA homolog 2 (E. coli); bolA homolog 2B (E. coli)	Homo sapiens
654483	bolA homolog 2 (E. coli); bolA homolog 2B (E. coli)	Homo sapiens
10066	secretory carrier membrane protein 2	Homo sapiens
6635	small nuclear ribonucleoprotein polypeptide E-like 1; small nuclear ribonucleoprotein polypeptide E; similar to hCG23490	Homo sapiens
6813	syntaxin binding protein 2	Homo sapiens
2539	glucose-6-phosphate dehydrogenase	Homo sapiens
23644	enhancer of mRNA decapping 4	Homo sapiens
9861	proteasome (prosome, macropain) 26S subunit, non-ATPase, 6	Homo sapiens
23041	MON2 homolog (S. cerevisiae)	Homo sapiens
5464	pyrophosphatase (inorganic) 1	Homo sapiens
84617	tubulin, beta 6	Homo sapiens
5110	protein-L-isoaspartate (D-aspartate) O-methyltransferase	Homo sapiens
2946	glutathione S-transferase mu 2 (muscle)	Homo sapiens
5423	polymerase (DNA directed), beta	Homo sapiens
7358	UDP-glucose dehydrogenase	Homo sapiens
10552	actin related protein 2/3 complex, subunit 1A, 41kDa	Homo sapiens
10982	microtubule-associated protein, RP/EB family, member 2	Homo sapiens
727	complement component 5	Homo sapiens
55768	N-glycanase 1	Homo sapiens
11338	U2 small nuclear RNA auxiliary factor 2	Homo sapiens
55610	coiled-coil domain containing 132	Homo sapiens
6135	ribosomal protein L11	Homo sapiens
5236	phosphoglucomutase 1	Homo sapiens
8880	far upstream element (FUSE) binding protein 1	Homo sapiens
5547	prolylcarboxypeptidase (angiotensinase C)	Homo sapiens
5694	proteasome (prosome, macropain) subunit, beta type, 6	Homo sapiens
6272	sortilin 1	Homo sapiens
3030	hydroxyacyl-Coenzyme A dehydrogenase/3-ketoacyl-Coenzyme A thiolase/enoyl-Coenzyme A hydratase (trifunctional protein), alpha subunit	Homo sapiens
23119	hypermethylated in cancer 2	Homo sapiens
23028	lysine (K)-specific demethylase 1	Homo sapiens
7311	ubiquitin A-52 residue ribosomal protein fusion product 1	Homo sapiens
10238	WD repeat domain 68	Homo sapiens
8751	ADAM metallopeptidase domain 15	Homo sapiens
5270	serpin peptidase inhibitor, clade E (nexin, plasminogen activator inhibitor type 1), member 2	Homo sapiens
208	v-akt murine thymoma viral oncogene homolog 2	Homo sapiens
79709	glycosyltransferase 25 domain containing 1	Homo sapiens
6520	solute carrier family 3 (activators of dibasic and neutral amino acid transport), member 2	Homo sapiens
23345	spectrin repeat containing, nuclear envelope 1	Homo sapiens
408050	NODAL modulator 3; NODAL modulator 1; NODAL modulator 2	Homo sapiens
23420	NODAL modulator 3; NODAL modulator 1; NODAL modulator 2	Homo sapiens
283820	NODAL modulator 3; NODAL modulator 1; NODAL modulator 2	Homo sapiens
10189	THO complex 4	Homo sapiens
311	annexin A11	Homo sapiens
6232	ribosomal protein S27 pseudogene 29; ribosomal protein S27 pseudogene 9; ribosomal protein S27 pseudogene 23; ribosomal protein S27 pseudogene 13; ribosomal protein S27; ribosomal protein S27 pseudogene 21; ribosomal protein S27 pseudogene 7; ribosomal protein S27 pseudogene 6; ribosomal protein S27 pseudogene 19	Homo sapiens
317772	histone cluster 2, H2ab	Homo sapiens
64149	chromosome 17 open reading frame 75	Homo sapiens
11093	ADAM metallopeptidase with thrombospondin type 1 motif, 13	Homo sapiens
1398	v-crk sarcoma virus CT10 oncogene homolog (avian)	Homo sapiens
56650	claudin domain containing 1	Homo sapiens
369	v-raf murine sarcoma 3611 viral oncogene homolog	Homo sapiens
506	ATP synthase, H+ transporting, mitochondrial F1 complex, beta polypeptide	Homo sapiens

4860	nucleoside phosphorylase	Homo sapiens
10399	guanine nucleotide binding protein (G protein), beta polypeptide 2-like 1	Homo sapiens
58533	sorting nexin 6	Homo sapiens
4924	nucleobindin 1	Homo sapiens
114876	oxysterol binding protein-like 1A	Homo sapiens
3181	heterogeneous nuclear ribonucleoprotein A2/B1	Homo sapiens
5717	proteasome (prosome, macropain) 26S subunit, non-ATPase, 11	Homo sapiens
9391	cytosolic iron-sulfur protein assembly 1 homolog (S. cerevisiae)	Homo sapiens
6904	tubulin folding cofactor D	Homo sapiens
80230	RUN and FYVE domain containing 1	Homo sapiens
1652	D-dopachrome tautomerase	Homo sapiens
6132	ribosomal protein L8; ribosomal protein L8 pseudogene 2	Homo sapiens
2799	glucosamine (N-acetyl)-6-sulfatase	Homo sapiens
140890	splicing factor, arginine/serine-rich 12	Homo sapiens
1727	cytochrome b5 reductase 3	Homo sapiens
3491	cysteine-rich, angiogenic inducer, 61	Homo sapiens
11146	glomulin, FKBP associated protein	Homo sapiens
10137	RNA binding motif protein 12; copine I	Homo sapiens
8904	RNA binding motif protein 12; copine I	Homo sapiens
6949	Treacher Collins-Franceschetti syndrome 1	Homo sapiens
3494	immunoglobulin heavy constant alpha 2 (A2m marker)	Homo sapiens
5962	radixin	Homo sapiens
253260	RPTOR independent companion of MTOR, complex 2	Homo sapiens
3312	heat shock 70kDa protein 8	Homo sapiens
3417	isocitrate dehydrogenase 1 (NADP+), soluble	Homo sapiens
9352	thioredoxin-like 1	Homo sapiens
3646	eukaryotic translation initiation factor 3, subunit E	Homo sapiens
6767	similar to heat shock 70kD protein binding protein; suppression of tumorigenicity 13 (colon carcinoma) (Hsp70 interacting protein)	Homo sapiens
4869	nucleophosmin 1 (nucleolar phosphoprotein B23, numatrin) pseudogene 21; hypothetical LOC100131044; similar to nucleophosmin 1; nucleophosmin (nucleolar phosphoprotein B23, numatrin)	Homo sapiens
27161	eukaryotic translation initiation factor 2C, 2	Homo sapiens
6809	syntaxin 3	Homo sapiens
4076	cell cycle associated protein 1	Homo sapiens
10844	tubulin, gamma complex associated protein 2	Homo sapiens
9482	syntaxin 8	Homo sapiens
10432	RNA binding motif protein 14; RNA binding motif protein 4	Homo sapiens
5936	RNA binding motif protein 14; RNA binding motif protein 4	Homo sapiens
6222	ribosomal protein S18 pseudogene 12; ribosomal protein S18 pseudogene 5; ribosomal protein S18	Homo sapiens
388339	ribosomal protein S18 pseudogene 12; ribosomal protein S18 pseudogene 5; ribosomal protein S18	Homo sapiens
100131863	ribosomal protein S18 pseudogene 12; ribosomal protein S18 pseudogene 5; ribosomal protein S18	Homo sapiens
3996	lethal giant larvae homolog 1 (Drosophila)	Homo sapiens
5287	phosphoinositide-3-kinase, class 2, beta polypeptide	Homo sapiens
5531	protein phosphatase 4 (formerly X), catalytic subunit	Homo sapiens
2629	glucosidase, beta; acid (includes glucosylceramidase)	Homo sapiens
4666	nascent polypeptide-associated complex alpha subunit	Homo sapiens
10211	flotillin 1	Homo sapiens
643531	ribosomal protein L29 pseudogene 9; ribosomal protein L29 pseudogene 12; ribosomal protein L29 pseudogene 11; ribosomal protein L29; ribosomal protein L29 pseudogene 26	Homo sapiens
283412	ribosomal protein L29 pseudogene 9; ribosomal protein L29 pseudogene 12; ribosomal protein L29 pseudogene 11; ribosomal protein L29; ribosomal protein L29 pseudogene 26	Homo sapiens
100131713	ribosomal protein L29 pseudogene 9; ribosomal protein L29 pseudogene 12; ribosomal protein L29 pseudogene 11; ribosomal protein L29; ribosomal protein L29 pseudogene 26	Homo sapiens
647285	ribosomal protein L29 pseudogene 9; ribosomal protein L29 pseudogene 12; ribosomal protein L29 pseudogene 11; ribosomal protein L29; ribosomal protein L29 pseudogene 26	Homo sapiens
6159	ribosomal protein L29 pseudogene 9; ribosomal protein L29 pseudogene 12; ribosomal protein L29 pseudogene 11; ribosomal protein L29; ribosomal protein L29 pseudogene 26	Homo sapiens
3482	insulin-like growth factor 2 receptor	Homo sapiens
5594	mitogen-activated protein kinase 1	Homo sapiens
3064	huntingtin	Homo sapiens
5861	RAB1A, member RAS oncogene family	Homo sapiens
7453	tryptophanyl-tRNA synthetase	Homo sapiens
317	apoptotic peptidase activating factor 1	Homo sapiens
27335	eukaryotic translation initiation factor 3, subunit K	Homo sapiens
23193	glucosidase, alpha; neutral AB	Homo sapiens
27430	methionine adenosyltransferase II, beta	Homo sapiens
25963	transmembrane protein 87A	Homo sapiens
100128936	ribosomal protein L10a pseudogene 6; ribosomal protein L10a; ribosomal protein L10a pseudogene 9	Homo sapiens
4736	ribosomal protein L10a pseudogene 6; ribosomal protein L10a; ribosomal protein L10a pseudogene 9	Homo sapiens
728979	ribosomal protein L10a pseudogene 6; ribosomal protein L10a; ribosomal protein L10a pseudogene 9	Homo sapiens
83872	hemicentin 1	Homo sapiens
79065	ATG9 autophagy related 9 homolog A (S. cerevisiae)	Homo sapiens
9554	SEC22 vesicle trafficking protein homolog B (S. cerevisiae)	Homo sapiens
23157	septin 6	Homo sapiens
64841	glucosamine-phosphate N-acetyltransferase 1	Homo sapiens
284207	meteorin, glial cell differentiation regulator-like; similar to meteorin, glial cell differentiation regulator-like	Homo sapiens
5052	peroxiredoxin 1	Homo sapiens
9577	brain and reproductive organ-expressed (TNFRSF1A modulator)	Homo sapiens
9945	glutamine-fructose-6-phosphate transaminase 2	Homo sapiens
9690	ubiquitin protein ligase E3C	Homo sapiens
720	complement component 4A (Rodgers blood group)	Homo sapiens

1499	catenin (cadherin-associated protein), beta 1, 88kDa	Homo sapiens
5335	phospholipase C, gamma 1	Homo sapiens
5216	profilin 1	Homo sapiens
440589	ribosomal protein S2 pseudogene 8; ribosomal protein S2 pseudogene 11; ribosomal protein S2 pseudogene 5; ribosomal protein S2 pseudogene 12; ribosomal protein S2 pseudogene 51; ribosomal protein S2 pseudogene 17; ribosomal protein S2 pseudogene 55; ribosomal protein S2 pseudogene 20; ribosomal protein S2	Homo sapiens
286444	ribosomal protein S2 pseudogene 8; ribosomal protein S2 pseudogene 11; ribosomal protein S2 pseudogene 5; ribosomal protein S2 pseudogene 12; ribosomal protein S2 pseudogene 51; ribosomal protein S2 pseudogene 17; ribosomal protein S2 pseudogene 55; ribosomal protein S2 pseudogene 20; ribosomal protein S2	Homo sapiens
729679	ribosomal protein S2 pseudogene 8; ribosomal protein S2 pseudogene 11; ribosomal protein S2 pseudogene 5; ribosomal protein S2 pseudogene 12; ribosomal protein S2 pseudogene 51; ribosomal protein S2 pseudogene 17; ribosomal protein S2 pseudogene 55; ribosomal protein S2 pseudogene 20; ribosomal protein S2	Homo sapiens
650901	ribosomal protein S2 pseudogene 8; ribosomal protein S2 pseudogene 11; ribosomal protein S2 pseudogene 5; ribosomal protein S2 pseudogene 12; ribosomal protein S2 pseudogene 51; ribosomal protein S2 pseudogene 17; ribosomal protein S2 pseudogene 55; ribosomal protein S2 pseudogene 20; ribosomal protein S2	Homo sapiens
100130562	ribosomal protein S2 pseudogene 8; ribosomal protein S2 pseudogene 11; ribosomal protein S2 pseudogene 5; ribosomal protein S2 pseudogene 12; ribosomal protein S2 pseudogene 51; ribosomal protein S2 pseudogene 17; ribosomal protein S2 pseudogene 55; ribosomal protein S2 pseudogene 20; ribosomal protein S2	Homo sapiens
343184	ribosomal protein S2 pseudogene 8; ribosomal protein S2 pseudogene 11; ribosomal protein S2 pseudogene 5; ribosomal protein S2 pseudogene 12; ribosomal protein S2 pseudogene 51; ribosomal protein S2 pseudogene 17; ribosomal protein S2 pseudogene 55; ribosomal protein S2 pseudogene 20; ribosomal protein S2	Homo sapiens
400963	ribosomal protein S2 pseudogene 8; ribosomal protein S2 pseudogene 11; ribosomal protein S2 pseudogene 5; ribosomal protein S2 pseudogene 12; ribosomal protein S2 pseudogene 51; ribosomal protein S2 pseudogene 17; ribosomal protein S2 pseudogene 55; ribosomal protein S2 pseudogene 20; ribosomal protein S2	Homo sapiens
645018	ribosomal protein S2 pseudogene 8; ribosomal protein S2 pseudogene 11; ribosomal protein S2 pseudogene 5; ribosomal protein S2 pseudogene 12; ribosomal protein S2 pseudogene 51; ribosomal protein S2 pseudogene 17; ribosomal protein S2 pseudogene 55; ribosomal protein S2 pseudogene 20; ribosomal protein S2	Homo sapiens
6187	ribosomal protein S2 pseudogene 8; ribosomal protein S2 pseudogene 11; ribosomal protein S2 pseudogene 5; ribosomal protein S2 pseudogene 12; ribosomal protein S2 pseudogene 51; ribosomal protein S2 pseudogene 17; ribosomal protein S2 pseudogene 55; ribosomal protein S2 pseudogene 20; ribosomal protein S2	Homo sapiens
3265	v-Ha-ras Harvey rat sarcoma viral oncogene homolog	Homo sapiens
10294	DnaJ (Hsp40) homolog, subfamily A, member 2	Homo sapiens
9550	ATPase, H+ transporting, lysosomal 13kDa, V1 subunit G1	Homo sapiens
2674	GDNF family receptor alpha 1	Homo sapiens
85439	stonin 2	Homo sapiens
149371	exocyst complex component 8	Homo sapiens
23753	stromal cell-derived factor 2-like 1	Homo sapiens
8675	syntaxin 16	Homo sapiens
23	ATP-binding cassette, sub-family F (GCN20), member 1	Homo sapiens
2273	four and a half LIM domains 1	Homo sapiens
5432	polymerase (RNA) II (DNA directed) polypeptide C, 33kDa	Homo sapiens
1759	dynamin 1	Homo sapiens
10163	WAS protein family, member 2	Homo sapiens
8907	adaptor-related protein complex 1, mu 1 subunit	Homo sapiens
83473	katanin p60 subunit A-like 2	Homo sapiens
5806	pentraxin-related gene, rapidly induced by IL-1 beta	Homo sapiens
9793	cytoskeleton associated protein 5	Homo sapiens
3512	immunoglobulin J polypeptide, linker protein for immunoglobulin alpha and mu polypeptides	Homo sapiens
553115	penta-EF-hand domain containing 1	Homo sapiens
285527	FRY-like	Homo sapiens
2017	cortactin	Homo sapiens
3688	integrin, beta 1 (fibronectin receptor, beta polypeptide, antigen CD29 includes MDF2, MSK12)	Homo sapiens
80347	Coenzyme A synthase	Homo sapiens
2132	exostoses (multiple) 2	Homo sapiens
10130	protein disulfide isomerase family A, member 6	Homo sapiens
3148	high-mobility group box 2	Homo sapiens
4125	mannosidase, alpha, class 2B, member 1	Homo sapiens
1173	adaptor-related protein complex 2, mu 1 subunit	Homo sapiens
10051	structural maintenance of chromosomes 4	Homo sapiens
1456	casein kinase 1, gamma 3	Homo sapiens
6158	ribosomal protein L28	Homo sapiens
10890	RAB10, member RAS oncogene family	Homo sapiens
987	LPS-responsive vesicle trafficking, beach and anchor containing	Homo sapiens
374897	suprabasin	Homo sapiens
4179	CD46 molecule, complement regulatory protein	Homo sapiens
23233	exocyst complex component 6B	Homo sapiens
10085	EGF-like repeats and discoidin I-like domains 3	Homo sapiens
7280	tubulin, beta 2A	Homo sapiens
9319	thyroid hormone receptor interactor 13	Homo sapiens
1271	ciliary neurotrophic factor receptor	Homo sapiens
57493	HEG homolog 1 (zebrafish)	Homo sapiens
116143	WD repeat domain 92	Homo sapiens
4690	NCK adaptor protein 1	Homo sapiens
7249	tuberous sclerosis 2	Homo sapiens
2512	similar to ferritin, light polypeptide; ferritin, light polypeptide	Homo sapiens
2068	excision repair cross-complementing rodent repair deficiency, complementation group 2	Homo sapiens

56944	olfactomedin-like 3	Homo sapiens
7837	peroxidasin homolog (Drosophila)	Homo sapiens
55970	guanine nucleotide binding protein (G protein), gamma 12	Homo sapiens
94015	tweety homolog 2 (Drosophila)	Homo sapiens
11100	heterogeneous nuclear ribonucleoprotein U-like 1	Homo sapiens
8140	solute carrier family 7 (cationic amino acid transporter, y+ system), member 5	Homo sapiens
399687	myosin XVIIIA	Homo sapiens
10788	IQ motif containing GTPase activating protein 2	Homo sapiens
1435	colony stimulating factor 1 (macrophage)	Homo sapiens
4041	low density lipoprotein receptor-related protein 5	Homo sapiens
7109	trafficking protein particle complex 10	Homo sapiens
9043	sperm associated antigen 9	Homo sapiens
23066	cullin-associated and neddylation-dissociated 2 (putative)	Homo sapiens
51520	leucyl-tRNA synthetase	Homo sapiens
57148	KIAA1219	Homo sapiens
6741	Sjogren syndrome antigen B (autoantigen La)	Homo sapiens
26259	F-box and WD repeat domain containing 8	Homo sapiens
10216	proteoglycan 4	Homo sapiens
308	annexin A5	Homo sapiens
152007	GLI pathogenesis-related 2	Homo sapiens
439	arsA arsenite transporter, ATP-binding, homolog 1 (bacterial)	Homo sapiens
64746	acyl-Coenzyme A binding domain containing 3	Homo sapiens
81876	RAB1B, member RAS oncogene family	Homo sapiens
2932	glycogen synthase kinase 3 beta	Homo sapiens
5635	phosphoribosyl pyrophosphate synthetase-associated protein 1	Homo sapiens
1278	collagen, type I, alpha 2	Homo sapiens
2873	G protein pathway suppressor 1	Homo sapiens
950	scavenger receptor class B, member 2	Homo sapiens
338	apolipoprotein B (including Ag(x) antigen)	Homo sapiens
10277	ubiquitination factor E4B (UFD2 homolog, yeast)	Homo sapiens
51382	ATPase, H+ transporting, lysosomal 34kDa, V1 subunit D	Homo sapiens
29978	ubiquilin 2	Homo sapiens
351	amyloid beta (A4) precursor protein	Homo sapiens
3925	stathmin 1	Homo sapiens
58513	epidermal growth factor receptor pathway substrate 15-like 1	Homo sapiens
9522	secretory carrier membrane protein 1	Homo sapiens
6597	SWI/SNF related, matrix associated, actin dependent regulator of chromatin, subfamily a, member 4	Homo sapiens
3306	heat shock 70kDa protein 2	Homo sapiens
10054	ubiquitin-like modifier activating enzyme 2	Homo sapiens
7779	solute carrier family 30 (zinc transporter), member 1	Homo sapiens
6917	transcription elongation factor A (SII), 1 pseudogene 2; transcription elongation factor A (SII), 1	Homo sapiens
55100	WD repeat domain 70	Homo sapiens
9044	BTAF1 RNA polymerase II, B-TFIID transcription factor-associated, 170kDa (Mot1 homolog, S. cerevisiae)	Homo sapiens
5902	similar to RAN binding protein 1; RAN binding protein 1	Homo sapiens
10526	importin 8	Homo sapiens
57599	WD repeat domain 48	Homo sapiens
1477	cleavage stimulation factor, 3' pre-RNA, subunit 1, 50kDa	Homo sapiens
1267	2',3'-cyclic nucleotide 3' phosphodiesterase	Homo sapiens
7110	TATA element modulatory factor 1	Homo sapiens
5654	HtrA serine peptidase 1	Homo sapiens
9353	slit homolog 2 (Drosophila)	Homo sapiens
7879	RAB7A, member RAS oncogene family	Homo sapiens
3912	laminin, beta 1	Homo sapiens
8533	COP9 constitutive photomorphogenic homolog subunit 3 (Arabidopsis)	Homo sapiens
4318	matrix metallopeptidase 9 (gelatinase B, 92kDa gelatinase, 92kDa type IV collagenase)	Homo sapiens
9775	eukaryotic translation initiation factor 4A, isoform 3	Homo sapiens
81624	diaphanous homolog 3 (Drosophila)	Homo sapiens
5708	proteasome (prosome, macropain) 26S subunit, non-ATPase, 2	Homo sapiens
161424	chromosome 14 open reading frame 21	Homo sapiens
23011	RAB21, member RAS oncogene family	Homo sapiens
6231	ribosomal protein S26 pseudogene 38; ribosomal protein S26 pseudogene 39; ribosomal protein S26 pseudogene 35; ribosomal protein S26 pseudogene 31; ribosomal protein S26 pseudogene 20; ribosomal protein S26 pseudogene 54; ribosomal protein S26 pseudogene 2; ribosomal protein S26 pseudogene 53; ribosomal protein S26 pseudogene 25; ribosomal protein S26 pseudogene 50; ribosomal protein S26 pseudogene 6; ribosomal protein S26 pseudogene 8; ribosomal protein S26	Homo sapiens
4144	methionine adenosyltransferase II, alpha	Homo sapiens
54512	exosome component 4	Homo sapiens
5899	v-ral simian leukemia viral oncogene homolog B (ras related; GTP binding protein)	Homo sapiens
79876	ubiquitin-like modifier activating enzyme 5	Homo sapiens
55250	elongation protein 2 homolog (S. cerevisiae)	Homo sapiens
8518	inhibitor of kappa light polypeptide gene enhancer in B-cells, kinase complex-associated protein	Homo sapiens
51162	EGF-like-domain, multiple 7	Homo sapiens
6605	SWI/SNF related, matrix associated, actin dependent regulator of chromatin, subfamily e, member 1	Homo sapiens
58517	RNA binding motif protein 25	Homo sapiens
5255	phosphorylase kinase, alpha 1 pseudogene 1; phosphorylase kinase, alpha 1 (muscle)	Homo sapiens
2519	fucosidase, alpha-L- 2, plasma	Homo sapiens
29926	GDP-mannose pyrophosphorylase A	Homo sapiens
5910	RAP1, GTP-GDP dissociation stimulator 1	Homo sapiens
79718	transducin (beta)-like 1 X-linked receptor 1	Homo sapiens
7917	HLA-B associated transcript 3	Homo sapiens
4650	myosin IXB	Homo sapiens

5576	protein kinase, cAMP-dependent, regulatory, type II, alpha	Homo sapiens
708	complement component 1, q subcomponent binding protein	Homo sapiens
24137	kinesin family member 4B; kinesin family member 4A	Homo sapiens
3069	high density lipoprotein binding protein	Homo sapiens
25839	component of oligomeric golgi complex 4	Homo sapiens
10450	peptidylprolyl isomerase E (cyclophilin E)	Homo sapiens
10067	secretory carrier membrane protein 3	Homo sapiens
6434	transformer 2 beta homolog (Drosophila)	Homo sapiens
4131	microtubule-associated protein 1B	Homo sapiens
1453	casein kinase 1, delta	Homo sapiens
11078	TRIO and F-actin binding protein	Homo sapiens
1778	dynein, cytoplasmic 1, heavy chain 1	Homo sapiens
9343	elongation factor Tu GTP binding domain containing 2	Homo sapiens
996	cell division cycle 27 homolog (S. cerevisiae)	Homo sapiens
10059	dynamin 1-like	Homo sapiens
169714	quiescin Q6 sulfhydryl oxidase 2	Homo sapiens
1938	eukaryotic translation elongation factor 2	Homo sapiens
9844	engulfment and cell motility 1	Homo sapiens
6733	SFRS protein kinase 2	Homo sapiens
27095	trafficking protein particle complex 3	Homo sapiens
6515	solute carrier family 2 (facilitated glucose transporter), member 3	Homo sapiens
6541	solute carrier family 7 (cationic amino acid transporter, y+ system), member 1	Homo sapiens
3840	karyopherin alpha 4 (importin alpha 3)	Homo sapiens
23435	TAR DNA binding protein	Homo sapiens
51434	anaphase promoting complex subunit 7	Homo sapiens
27131	sorting nexin 5	Homo sapiens
63929	X-prolyl aminopeptidase (aminopeptidase P) 3, putative	Homo sapiens
3074	hexosaminidase B (beta polypeptide)	Homo sapiens
5352	procollagen-lysine, 2-oxoglutarate 5-dioxygenase 2	Homo sapiens
4012	leucyl/cystinyl aminopeptidase	Homo sapiens
3735	lysyl-tRNA synthetase	Homo sapiens
3609	interleukin enhancer binding factor 3, 90kDa	Homo sapiens
5208	6-phosphofructo-2-kinase/fructose-2,6-biphosphatase 2	Homo sapiens
387	ras homolog gene family, member A	Homo sapiens
160	adaptor-related protein complex 2, alpha 1 subunit	Homo sapiens
4841	non-POU domain containing, octamer-binding	Homo sapiens
29082	chromatin modifying protein 4A	Homo sapiens
5430	polymerase (RNA) II (DNA directed) polypeptide A, 220kDa	Homo sapiens
79987	sushi, von Willebrand factor type A, EGF and pentraxin domain containing 1	Homo sapiens
5202	prefoldin subunit 2	Homo sapiens
79861	tubulin, alpha-like 3	Homo sapiens
2091	fibrillarin	Homo sapiens
4054	latent transforming growth factor beta binding protein 3	Homo sapiens
9208	leucine rich repeat (in FLII) interacting protein 1	Homo sapiens
81611	acidic (leucine-rich) nuclear phosphoprotein 32 family, member E	Homo sapiens
203	adenylate kinase 1	Homo sapiens
85440	dedicator of cytokinesis 7	Homo sapiens
6168	ribosomal protein L37a	Homo sapiens
5870	RAB6C, member RAS oncogene family; RAB6A, member RAS oncogene family; hypothetical LOC100130819; RAB6C-like	Homo sapiens
150786	RAB6C, member RAS oncogene family; RAB6A, member RAS oncogene family; hypothetical LOC100130819; RAB6C-like	Homo sapiens
84084	RAB6C, member RAS oncogene family; RAB6A, member RAS oncogene family; hypothetical LOC100130819; RAB6C-like	Homo sapiens
135293	peptidase M20 domain containing 2	Homo sapiens
5519	protein phosphatase 2 (formerly 2A), regulatory subunit A, beta isoform	Homo sapiens
6144	ribosomal protein L21 pseudogene 134; ribosomal protein L21 pseudogene 80; ribosomal protein L21 pseudogene 20; ribosomal protein L21 pseudogene 46; ribosomal protein L21 pseudogene 45; ribosomal protein L21 pseudogene 131; ribosomal protein L21 pseudogene 16; ribosomal protein L21 pseudogene 53; ribosomal protein L21 pseudogene 120; ribosomal protein L21 pseudogene 37; ribosomal protein L21 pseudogene 93; ribosomal protein L21 pseudogene 39; ribosomal protein L21 pseudogene 29; ribosomal protein L21 pseudogene 28; ribosomal protein L21 pseudogene 14; ribosomal protein L21 pseudogene 98; ribosomal protein L21 pseudogene 105; ribosomal protein L21 pseudogene 87; ribosomal protein L21 pseudogene 128; ribosomal protein L21 pseudogene 69; ribosomal protein L21 pseudogene 97; ribosomal protein L21; ribosomal protein L21 pseudogene 119; ribosomal protein L21 pseudogene 125	Homo sapiens
56920	sema domain, immunoglobulin domain (Ig), short basic domain, secreted, (semaphorin) 3G	Homo sapiens
204	adenylate kinase 2	Homo sapiens
6237	related RAS viral (r-ras) oncogene homolog	Homo sapiens
11171	serine/threonine kinase receptor associated protein	Homo sapiens
56915	exosome component 5	Homo sapiens
3998	lectin, mannose-binding, 1	Homo sapiens
23170	tubulin tyrosine ligase-like family, member 12	Homo sapiens
10550	ADP-ribosylation-like factor 6 interacting protein 5	Homo sapiens
1373	carbamoyl-phosphate synthetase 1, mitochondrial	Homo sapiens
10313	reticulon 3	Homo sapiens
2746	glutamate dehydrogenase 1	Homo sapiens
51699	vacuolar protein sorting 29 homolog (S. cerevisiae)	Homo sapiens
9439	mediator complex subunit 23	Homo sapiens
7431	vimentin	Homo sapiens
23451	splicing factor 3b, subunit 1, 155kDa	Homo sapiens
23246	block of proliferation 1	Homo sapiens
6197	ribosomal protein S6 kinase, 90kDa, polypeptide 3	Homo sapiens
3188	ribosomal protein L36a pseudogene 51; ribosomal protein L36a pseudogene 37; ribosomal protein L36a pseudogene 49; heterogeneous nuclear ribonucleoprotein H2 (H'); ribosomal protein L36a	Homo sapiens
7812	cold shock domain containing E1, RNA-binding	Homo sapiens

84313	vacuolar protein sorting 25 homolog (S. cerevisiae)	Homo sapiens
10801	septin 9	Homo sapiens
6723	spermidine synthase	Homo sapiens
6449	small glutamine-rich tetratricopeptide repeat (TPR)-containing, alpha	Homo sapiens
9126	structural maintenance of chromosomes 3	Homo sapiens
9810	ring finger protein 40	Homo sapiens
977	CD151 molecule (Raph blood group)	Homo sapiens
8825	lin-7 homolog A (C. elegans)	Homo sapiens
2193	phenylalanyl-tRNA synthetase, alpha subunit	Homo sapiens
9675	KIAA0406	Homo sapiens
9510	ADAM metallopeptidase with thrombospondin type 1 motif, 1	Homo sapiens
5691	proteasome (prosome, macropain) subunit, beta type, 3	Homo sapiens
129531	MIT, microtubule interacting and transport, domain containing 1	Homo sapiens
79631	elongation factor Tu GTP binding domain containing 1	Homo sapiens
9040	ubiquitin-conjugating enzyme E2M (UBC12 homolog, yeast); ubiquitin-conjugating enzyme E2M pseudogene 1	Homo sapiens
201292	tripartite motif-containing 65	Homo sapiens
151011	septin 10	Homo sapiens
3693	integrin, beta 5	Homo sapiens
7042	transforming growth factor, beta 2	Homo sapiens
10627	myosin, light chain 12A, regulatory, non-sarcomeric	Homo sapiens
55269	paraspeckle component 1; paraspeckle protein 1 pseudogene	Homo sapiens
2060	epidermal growth factor receptor pathway substrate 15	Homo sapiens
22803	5'-3' exoribonuclease 2	Homo sapiens
29109	formin homology 2 domain containing 1	Homo sapiens
60412	exocyst complex component 4	Homo sapiens
9375	transmembrane 9 superfamily member 2	Homo sapiens
7094	talin 1	Homo sapiens
55324	ATP-binding cassette, sub-family F (GCN20), member 3	Homo sapiens
8567	MAP-kinase activating death domain	Homo sapiens
5796	protein tyrosine phosphatase, receptor type, K	Homo sapiens
145864	hyaluronan and proteoglycan link protein 3	Homo sapiens
55207	ADP-ribosylation factor-like 8B	Homo sapiens
88455	ankyrin repeat domain 13A	Homo sapiens
51072	mediator of cell motility 1; similar to mediator of cell motility 1	Homo sapiens
55275	vacuolar protein sorting 53 homolog (S. cerevisiae)	Homo sapiens
9318	COP9 constitutive photomorphogenic homolog subunit 2 (Arabidopsis)	Homo sapiens
1200	tripeptidyl peptidase I	Homo sapiens
1460	lymphocyte antigen 6 complex, locus G5B; casein kinase 2, beta polypeptide	Homo sapiens
53918	pelota homolog (Drosophila)	Homo sapiens
7251	tumor susceptibility gene 101	Homo sapiens
3800	kinesin family member 5C	Homo sapiens
84955	NudC domain containing 1	Homo sapiens
9382	component of oligomeric golgi complex 1	Homo sapiens
23545	ATPase, H+ transporting, lysosomal V0 subunit a2	Homo sapiens
55754	transmembrane protein 30A	Homo sapiens
11103	KRR1, small subunit (SSU) processome component, homolog (yeast)	Homo sapiens
5046	proprotein convertase subtilisin/kexin type 6	Homo sapiens
10360	nucleophosmin/nucleoplasmin, 3	Homo sapiens
23446	solute carrier family 44, member 1	Homo sapiens
8467	SWI/SNF related, matrix associated, actin dependent regulator of chromatin, subfamily a, member 5	Homo sapiens
5832	aldehyde dehydrogenase 18 family, member A1	Homo sapiens
2584	galactokinase 1	Homo sapiens
9532	BCL2-associated athanogene 2	Homo sapiens
5869	RAB5B, member RAS oncogene family	Homo sapiens
4241	antigen p97 (melanoma associated) identified by monoclonal antibodies 133.2 and 96.5	Homo sapiens
6627	small nuclear ribonucleoprotein polypeptide A'	Homo sapiens
3039	hemoglobin, alpha 2; hemoglobin, alpha 1	Homo sapiens
3040	hemoglobin, alpha 2; hemoglobin, alpha 1	Homo sapiens
7407	valyl-tRNA synthetase	Homo sapiens
5478	similar to TRIMCyp; peptidylprolyl isomerase A (cyclophilin A); peptidylprolyl isomerase A (cyclophilin A)-like 3	Homo sapiens
653214	similar to TRIMCyp; peptidylprolyl isomerase A (cyclophilin A); peptidylprolyl isomerase A (cyclophilin A)-like 3	Homo sapiens
439953	similar to TRIMCyp; peptidylprolyl isomerase A (cyclophilin A); peptidylprolyl isomerase A (cyclophilin A)-like 3	Homo sapiens
7841	mannosyl-oligosaccharide glucosidase	Homo sapiens
51497	TH1-like (Drosophila)	Homo sapiens
3105	major histocompatibility complex, class I, A	Homo sapiens
5567	protein kinase, cAMP-dependent, catalytic, beta	Homo sapiens
56949	XPA binding protein 2	Homo sapiens
875	cystathionine-beta-synthase	Homo sapiens
9777	transmembrane 9 superfamily protein member 4	Homo sapiens
2237	flap structure-specific endonuclease 1	Homo sapiens
4677	asparaginyl-tRNA synthetase	Homo sapiens
8881	cell division cycle 16 homolog (S. cerevisiae)	Homo sapiens
51727	cytidine monophosphate (UMP-CMP) kinase 1, cytosolic	Homo sapiens
6093	similar to Rho-associated, coiled-coil containing protein kinase 1; Rho-associated, coiled-coil containing protein kinase 1	Homo sapiens
4141	methionyl-tRNA synthetase	Homo sapiens
1743	dihydrolipoamide S-succinyltransferase (E2 component of 2-oxo-glutarate complex); dihydrolipoamide S-succinyltransferase pseudogene (E2 component of 2-oxo-glutarate complex)	Homo sapiens
6203	ribosomal protein S9; ribosomal protein S9 pseudogene 4	Homo sapiens
6470	serine hydroxymethyltransferase 1 (soluble)	Homo sapiens

3178	heterogeneous nuclear ribonucleoprotein A1-like 3; similar to heterogeneous nuclear ribonucleoprotein A1; heterogeneous nuclear ribonucleoprotein A1 pseudogene 2; heterogeneous nuclear ribonucleoprotein A1; heterogeneous nuclear ribonucleoprotein A1 pseudogene	Homo sapiens
1861	torsin family 1, member A (torsin A)	Homo sapiens
5880	ras-related C3 botulinum toxin substrate 2 (rho family, small GTP binding protein Rac2)	Homo sapiens
5813	purine-rich element binding protein A	Homo sapiens
1841	deoxythymidylate kinase (thymidylate kinase); similar to Deoxythymidylate kinase (thymidylate kinase)	Homo sapiens
388698	filaggrin family member 2	Homo sapiens
55745	MU-2/AP1M2 domain containing, death-inducing	Homo sapiens
3845	v-Ki-ras2 Kirsten rat sarcoma viral oncogene homolog	Homo sapiens
1272	contactin 1	Homo sapiens
22883	calsyntenin 1	Homo sapiens
4763	neurofibromin 1	Homo sapiens
57521	regulatory associated protein of MTOR, complex 1	Homo sapiens
10768	adenosylhomocysteinase-like 1	Homo sapiens
23657	solute carrier family 7, (cationic amino acid transporter, y+ system) member 11	Homo sapiens
9939	RNA binding motif protein 8A	Homo sapiens
10953	translocase of outer mitochondrial membrane 34	Homo sapiens
643358	ribosomal protein S27a pseudogene 12; ribosomal protein S27a; ribosomal protein S27a pseudogene 11; ribosomal protein S27a pseudogene 16	Homo sapiens
6233	ribosomal protein S27a pseudogene 12; ribosomal protein S27a; ribosomal protein S27a pseudogene 11; ribosomal protein S27a pseudogene 16	Homo sapiens
728590	ribosomal protein S27a pseudogene 12; ribosomal protein S27a; ribosomal protein S27a pseudogene 11; ribosomal protein S27a pseudogene 16	Homo sapiens
100130446	ribosomal protein S27a pseudogene 12; ribosomal protein S27a; ribosomal protein S27a pseudogene 11; ribosomal protein S27a pseudogene 16	Homo sapiens
55501	carbohydrate (chondroitin 4) sulfotransferase 12	Homo sapiens
718	similar to Complement C3 precursor; complement component 3; hypothetical protein LOC100133511	Homo sapiens
16	alanyl-tRNA synthetase	Homo sapiens
9689	basic leucine zipper and W2 domains 1 pseudogene 1; basic leucine zipper and W2 domains 1 like 1; basic leucine zipper and W2 domains 1	Homo sapiens
9716	aquarius homolog (mouse)	Homo sapiens
7514	exportin 1 (CRM1 homolog, yeast)	Homo sapiens
8295	transformation/transcription domain-associated protein	Homo sapiens
2199	fibulin 2	Homo sapiens
7060	thrombospondin 4	Homo sapiens
3192	heterogeneous nuclear ribonucleoprotein U (scaffold attachment factor A)	Homo sapiens
10298	p21 protein (Cdc42/Rac)-activated kinase 4	Homo sapiens
153364	metallo-beta-lactamase domain containing 2	Homo sapiens
1786	DNA (cytosine-5-)-methyltransferase 1	Homo sapiens
55140	elongation protein 3 homolog (S. cerevisiae)	Homo sapiens
11052	cleavage and polyadenylation specific factor 6, 68kDa	Homo sapiens
291	solute carrier family 25 (mitochondrial carrier; adenine nucleotide translocator), member 4	Homo sapiens
2202	EGF-containing fibulin-like extracellular matrix protein 1	Homo sapiens
170506	DEAH (Asp-Glu-Ala-His) box polypeptide 36	Homo sapiens
8615	USO1 homolog, vesicle docking protein (yeast)	Homo sapiens
2547	X-ray repair complementing defective repair in Chinese hamster cells 6; similar to ATP-dependent DNA helicase II, 70 kDa subunit	Homo sapiens
10856	RuvB-like 2 (E. coli)	Homo sapiens
1108	chromodomain helicase DNA binding protein 4	Homo sapiens
3081	homogentisate 1,2-dioxygenase (homogentisate oxidase)	Homo sapiens
6625	small nuclear ribonucleoprotein 70kDa (U1)	Homo sapiens
1508	cathepsin B	Homo sapiens
84904	chromosome 9 open reading frame 100	Homo sapiens
252839	transmembrane protein 9	Homo sapiens
10632	ATP synthase, H+ transporting, mitochondrial F0 complex, subunit G	Homo sapiens
59338	pleckstrin homology domain containing, family A (phosphoinositide binding specific) member 1	Homo sapiens
1191	clusterin	Homo sapiens
25940	family with sequence similarity 98, member A	Homo sapiens
4507	methylthioadenosine phosphorylase	Homo sapiens
310	annexin A7	Homo sapiens
6224	ribosomal protein S20	Homo sapiens
8480	RAE1 RNA export 1 homolog (S. pombe)	Homo sapiens
3492	immunoglobulin heavy constant gamma 1 (G1m marker); immunoglobulin heavy constant mu; immunoglobulin heavy variable 3-7; immunoglobulin heavy constant gamma 3 (G3m marker); immunoglobulin heavy variable 3-11 (gene/pseudogene); immunoglobulin heavy variable 4-31; immunoglobulin heavy locus	Homo sapiens
28452	immunoglobulin heavy constant gamma 1 (G1m marker); immunoglobulin heavy constant mu; immunoglobulin heavy variable 3-7; immunoglobulin heavy constant gamma 3 (G3m marker); immunoglobulin heavy variable 3-11 (gene/pseudogene); immunoglobulin heavy variable 4-31; immunoglobulin heavy locus	Homo sapiens
3507	immunoglobulin heavy constant gamma 1 (G1m marker); immunoglobulin heavy constant mu; immunoglobulin heavy variable 3-7; immunoglobulin heavy constant gamma 3 (G3m marker); immunoglobulin heavy variable 3-11 (gene/pseudogene); immunoglobulin heavy variable 4-31; immunoglobulin heavy locus	Homo sapiens
3502	immunoglobulin heavy constant gamma 1 (G1m marker); immunoglobulin heavy constant mu; immunoglobulin heavy variable 3-7; immunoglobulin heavy constant gamma 3 (G3m marker); immunoglobulin heavy variable 3-11 (gene/pseudogene); immunoglobulin heavy variable 4-31; immunoglobulin heavy locus	Homo sapiens
3500	immunoglobulin heavy constant gamma 1 (G1m marker); immunoglobulin heavy constant mu; immunoglobulin heavy variable 3-7; immunoglobulin heavy constant gamma 3 (G3m marker); immunoglobulin heavy variable 3-11 (gene/pseudogene); immunoglobulin heavy variable 4-31; immunoglobulin heavy locus	Homo sapiens

28450	immunoglobulin heavy constant gamma 1 (G1m marker); immunoglobulin heavy constant mu; immunoglobulin heavy variable 3-7; immunoglobulin heavy constant gamma 3 (G3m marker); immunoglobulin heavy variable 3-11 (gene/pseudogene); immunoglobulin heavy variable 4-31; immunoglobulin heavy locus	Homo sapiens
28396	immunoglobulin heavy constant gamma 1 (G1m marker); immunoglobulin heavy constant mu; immunoglobulin heavy variable 3-7; immunoglobulin heavy constant gamma 3 (G3m marker); immunoglobulin heavy variable 3-11 (gene/pseudogene); immunoglobulin heavy variable 4-31; immunoglobulin heavy locus	Homo sapiens
28969	basic leucine zipper and W2 domains 2	Homo sapiens
8634	RNA terminal phosphate cyclase domain 1	Homo sapiens
5573	protein kinase, cAMP-dependent, regulatory, type I, alpha (tissue specific extinguisher 1)	Homo sapiens
6642	sorting nexin 1	Homo sapiens
47	ATP citrate lyase	Homo sapiens
54536	exocyst complex component 6	Homo sapiens
51104	family with sequence similarity 108, member B1	Homo sapiens
5922	RAS p21 protein activator 2	Homo sapiens
9448	mitogen-activated protein kinase kinase kinase kinase 4	Homo sapiens
22796	component of oligomeric golgi complex 2	Homo sapiens
1832	desmoplakin	Homo sapiens
79084	WD repeat domain 77	Homo sapiens
5770	protein tyrosine phosphatase, non-receptor type 1	Homo sapiens
8570	KH-type splicing regulatory protein	Homo sapiens
3916	lysosomal-associated membrane protein 1	Homo sapiens
23140	zinc finger, ZZ-type with EF-hand domain 1	Homo sapiens
10101	nucleotide binding protein 2 (MinD homolog, E. coli)	Homo sapiens
9557	chromodomain helicase DNA binding protein 1-like	Homo sapiens
6774	signal transducer and activator of transcription 3 (acute-phase response factor)	Homo sapiens
6626	small nuclear ribonucleoprotein polypeptide A	Homo sapiens
5286	phosphoinositide-3-kinase, class 2, alpha polypeptide	Homo sapiens
1954	multiple EGF-like-domains 8	Homo sapiens
7165	tumor protein D52-like 2	Homo sapiens
11311	vacuolar protein sorting 45 homolog (S. cerevisiae)	Homo sapiens
65992	DDRGK domain containing 1	Homo sapiens
7073	TIA1 cytotoxic granule-associated RNA binding protein-like 1	Homo sapiens
10402	ST3 beta-galactoside alpha-2,3-sialyltransferase 6	Homo sapiens
5928	hypothetical LOC642954; retinoblastoma binding protein 4	Homo sapiens
8330	histone cluster 1, H2ag; histone cluster 1, H2ah; histone cluster 1, H2ai; histone cluster 1, H2ak; histone cluster 1, H2al; histone cluster 1, H2am	Homo sapiens
8336	histone cluster 1, H2ag; histone cluster 1, H2ah; histone cluster 1, H2ai; histone cluster 1, H2ak; histone cluster 1, H2al; histone cluster 1, H2am	Homo sapiens
85235	histone cluster 1, H2ag; histone cluster 1, H2ah; histone cluster 1, H2ai; histone cluster 1, H2ak; histone cluster 1, H2al; histone cluster 1, H2am	Homo sapiens
8329	histone cluster 1, H2ag; histone cluster 1, H2ah; histone cluster 1, H2ai; histone cluster 1, H2ak; histone cluster 1, H2al; histone cluster 1, H2am	Homo sapiens
8969	histone cluster 1, H2ag; histone cluster 1, H2ah; histone cluster 1, H2ai; histone cluster 1, H2ak; histone cluster 1, H2al; histone cluster 1, H2am	Homo sapiens
8332	histone cluster 1, H2ag; histone cluster 1, H2ah; histone cluster 1, H2ai; histone cluster 1, H2ak; histone cluster 1, H2al; histone cluster 1, H2am	Homo sapiens
6749	structure specific recognition protein 1	Homo sapiens
833	cysteinyl-tRNA synthetase	Homo sapiens
10093	tubulin tyrosine ligase-like family, member 3; actin related protein 2/3 complex, subunit 4, 20kDa	Homo sapiens
26140	tubulin tyrosine ligase-like family, member 3; actin related protein 2/3 complex, subunit 4, 20kDa	Homo sapiens
23603	coronin, actin binding protein, 1C	Homo sapiens
26064	retinoic acid induced 14	Homo sapiens
9588	peroxiredoxin 6	Homo sapiens
5867	RAB4A, member RAS oncogene family	Homo sapiens
7458	eukaryotic translation initiation factor 4H	Homo sapiens
2037	erythrocyte membrane protein band 4.1-like 2	Homo sapiens
51071	2-deoxyribose-5-phosphate aldolase homolog (C. elegans)	Homo sapiens
567	beta-2-microglobulin	Homo sapiens
6500	S-phase kinase-associated protein 1	Homo sapiens
55339	WD repeat domain 33	Homo sapiens
6117	replication protein A1, 70kDa	Homo sapiens
2720	galactosidase, beta 1	Homo sapiens
5682	proteasome (prosome, macropain) subunit, alpha type, 1	Homo sapiens
10040	target of myb1 (chicken)-like 1	Homo sapiens
4735	septin 2	Homo sapiens
6122	ribosomal protein L3; similar to 60S ribosomal protein L3 (L4)	Homo sapiens
2947	glutathione S-transferase mu 3 (brain)	Homo sapiens
490	ATPase, Ca++ transporting, plasma membrane 1	Homo sapiens
201931	transmembrane protein 192	Homo sapiens
8085	myeloid/lymphoid or mixed-lineage leukemia 2	Homo sapiens
65082	vacuolar protein sorting 33 homolog A (S. cerevisiae)	Homo sapiens
51726	DnaJ (Hsp40) homolog, subfamily B, member 11	Homo sapiens
1020	cyclin-dependent kinase 5	Homo sapiens
64225	atlastin GTPase 2	Homo sapiens
8237	ubiquitin specific peptidase 11	Homo sapiens
6902	tubulin folding cofactor A	Homo sapiens
26276	vacuolar protein sorting 33 homolog B (yeast)	Homo sapiens
1285	collagen, type IV, alpha 3 (Goodpasture antigen)	Homo sapiens
8602	NOP14 nucleolar protein homolog (yeast)	Homo sapiens
6154	ribosomal protein L26 pseudogene 33; ribosomal protein L26; ribosomal protein L26 pseudogene 16; ribosomal protein L26 pseudogene 19; ribosomal protein L26 pseudogene 6	Homo sapiens
4733	developmentally regulated GTP binding protein 1	Homo sapiens

11198	suppressor of Ty 16 homolog (S. cerevisiae); suppressor of Ty 16 homolog (S. cerevisiae) pseudogene	Homo sapiens
56902	partner of NOB1 homolog (S. cerevisiae)	Homo sapiens
11164	nudix (nucleoside diphosphate linked moiety X)-type motif 5	Homo sapiens
6558	solute carrier family 12 (sodium/potassium/chloride transporters), member 2	Homo sapiens
56160	necdin-like 2	Homo sapiens
7204	triple functional domain (PTPRF interacting)	Homo sapiens
2317	filamin B, beta (actin binding protein 278)	Homo sapiens
3978	ligase I, DNA, ATP-dependent	Homo sapiens
10802	SEC24 family, member A (S. cerevisiae)	Homo sapiens
2804	golgin B1, golgi integral membrane protein	Homo sapiens
3295	hydroxysteroid (17-beta) dehydrogenase 4	Homo sapiens
8833	guanine monphosphate synthetase	Homo sapiens
6611	spermine synthase; similar to spermine synthase	Homo sapiens
2044	EPH receptor A5	Homo sapiens
5093	poly(rC) binding protein 1	Homo sapiens
286410	ATPase, class VI, type 11C	Homo sapiens
55361	phosphatidylinositol 4-kinase type 2 alpha	Homo sapiens
5894	v-raf-1 murine leukemia viral oncogene homolog 1	Homo sapiens
55830	glycosyltransferase 8 domain containing 1	Homo sapiens
10220	growth differentiation factor 11	Homo sapiens
25814	ataxin 10	Homo sapiens
53	acid phosphatase 2, lysosomal	Homo sapiens
23473	calpain 7	Homo sapiens
26578	osteoclast stimulating factor 1	Homo sapiens
5720	proteasome (prosome, macropain) activator subunit 1 (PA28 alpha)	Homo sapiens
6241	ribonucleotide reductase M2 polypeptide	Homo sapiens
7323	ubiquitin-conjugating enzyme E2D 3 (UBC4/5 homolog, yeast); ubiquitin-conjugating enzyme E2D 3 pseudogene	Homo sapiens
3308	heat shock 70kDa protein 4	Homo sapiens
60684	chromosome 4 open reading frame 41	Homo sapiens
1399	v-crk sarcoma virus CT10 oncogene homolog (avian)-like	Homo sapiens
56254	ring finger protein 20	Homo sapiens
501	aldehyde dehydrogenase 7 family, member A1	Homo sapiens
5360	phospholipid transfer protein	Homo sapiens
27020	neuroplastin	Homo sapiens
3075	complement factor H	Homo sapiens
2618	phosphoribosylglycinamide formyltransferase, phosphoribosylglycinamide synthetase, phosphoribosylaminoimidazole synthetase	Homo sapiens
55692	LUC7-like (S. cerevisiae)	Homo sapiens
3493	immunoglobulin heavy constant alpha 1	Homo sapiens
6628	small nuclear ribonucleoprotein polypeptides B and B1	Homo sapiens
63979	fidgetin-like 1	Homo sapiens
4430	myosin IB	Homo sapiens
655	bone morphogenetic protein 7	Homo sapiens
10623	polymerase (RNA) III (DNA directed) polypeptide C (62kD)	Homo sapiens
27347	serine threonine kinase 39 (STE20/SPS1 homolog, yeast)	Homo sapiens
2997	glycogen synthase 1 (muscle)	Homo sapiens
32	acetyl-Coenzyme A carboxylase beta	Homo sapiens
85236	histone cluster 1, H2bk	Homo sapiens
7417	voltage-dependent anion channel 2	Homo sapiens
1783	dynein, cytoplasmic 1, light intermediate chain 2	Homo sapiens
23234	DnaJ (Hsp40) homolog, subfamily C, member 9	Homo sapiens
5595	hypothetical LOC100271831; mitogen-activated protein kinase 3	Homo sapiens
585	Bardet-Biedl syndrome 4	Homo sapiens
54549	sidekick homolog 2 (chicken)	Homo sapiens
9559	vacuolar protein sorting 26 homolog A (S. pombe)	Homo sapiens
113189	carbohydrate (N-acetylgalactosamine 4-0) sulfotransferase 14	Homo sapiens
2534	FYN oncogene related to SRC, FGR, YES	Homo sapiens
11190	centrosomal protein 250kDa	Homo sapiens
27044	staphylococcal nuclease and tudor domain containing 1	Homo sapiens
10094	similar to actin related protein 2/3 complex subunit 3; hypothetical LOC729841; actin related protein 2/3 complex, subunit 3, 21kDa	Homo sapiens
2717	galactosidase, alpha	Homo sapiens
1615	aspartyl-tRNA synthetase	Homo sapiens
5719	proteasome (prosome, macropain) 26S subunit, non-ATPase, 13	Homo sapiens
10923	SUB1 homolog (S. cerevisiae)	Homo sapiens
57180	ARP3 actin-related protein 3 homolog B (yeast)	Homo sapiens
5331	phospholipase C, beta 3 (phosphatidylinositol-specific)	Homo sapiens
6905	tubulin folding cofactor E	Homo sapiens
57410	SCY1-like 1 (S. cerevisiae)	Homo sapiens
5313	pyruvate kinase, liver and RBC	Homo sapiens
5713	proteasome (prosome, macropain) 26S subunit, non-ATPase, 7	Homo sapiens
79875	thrombospondin, type I, domain containing 4	Homo sapiens
4074	mannose-6-phosphate receptor (cation dependent)	Homo sapiens
5872	RAB13, member RAS oncogene family; similar to hCG24991	Homo sapiens
5634	phosphoribosyl pyrophosphate synthetase 2	Homo sapiens
8500	protein tyrosine phosphatase, receptor type, f polypeptide (PTPRF), interacting protein (liprin), alpha 1	Homo sapiens
3837	karyopherin (importin) beta 1	Homo sapiens
10985	GCN1 general control of amino-acid synthesis 1-like 1 (yeast)	Homo sapiens
23243	ankyrin repeat domain 28	Homo sapiens
667	dystonin	Homo sapiens
4057	lactotransferrin	Homo sapiens
5300	peptidylprolyl cis/trans isomerase, NIMA-interacting 1	Homo sapiens
2280	FK506 binding protein 1A, 12kDa	Homo sapiens

6535	solute carrier family 6 (neurotransmitter transporter, creatine), member 8	Homo sapiens
6189	ribosomal protein S3A pseudogene 5; ribosomal protein S3a pseudogene 47; ribosomal protein S3a pseudogene 49; ribosomal protein S3A; hypothetical LOC100131699; hypothetical LOC100130107	Homo sapiens
7337	ubiquitin protein ligase E3A	Homo sapiens
25920	cofactor of BRCA1	Homo sapiens
9704	DEAH (Asp-Glu-Ala-His) box polypeptide 34	Homo sapiens
83481	epiplakin 1	Homo sapiens
7371	uridine-cytidine kinase 2	Homo sapiens
10979	fermitin family homolog 2 (Drosophila)	Homo sapiens
3426	complement factor I	Homo sapiens
64210	MMS19 nucleotide excision repair homolog (S. cerevisiae)	Homo sapiens
1380	complement component (3d/Epstein Barr virus) receptor 2	Homo sapiens
6204	ribosomal protein S10; ribosomal protein S10 pseudogene 4; ribosomal protein S10 pseudogene 11; ribosomal protein S10 pseudogene 22; ribosomal protein S10 pseudogene 7; ribosomal protein S10 pseudogene 13	Homo sapiens
401817	ribosomal protein S10; ribosomal protein S10 pseudogene 4; ribosomal protein S10 pseudogene 11; ribosomal protein S10 pseudogene 22; ribosomal protein S10 pseudogene 7; ribosomal protein S10 pseudogene 13	Homo sapiens
646785	ribosomal protein S10; ribosomal protein S10 pseudogene 4; ribosomal protein S10 pseudogene 11; ribosomal protein S10 pseudogene 22; ribosomal protein S10 pseudogene 7; ribosomal protein S10 pseudogene 13	Homo sapiens
391833	ribosomal protein S10; ribosomal protein S10 pseudogene 4; ribosomal protein S10 pseudogene 11; ribosomal protein S10 pseudogene 22; ribosomal protein S10 pseudogene 7; ribosomal protein S10 pseudogene 13	Homo sapiens
728791	ribosomal protein S10; ribosomal protein S10 pseudogene 4; ribosomal protein S10 pseudogene 11; ribosomal protein S10 pseudogene 22; ribosomal protein S10 pseudogene 7; ribosomal protein S10 pseudogene 13	Homo sapiens
376693	ribosomal protein S10; ribosomal protein S10 pseudogene 4; ribosomal protein S10 pseudogene 11; ribosomal protein S10 pseudogene 22; ribosomal protein S10 pseudogene 7; ribosomal protein S10 pseudogene 13	Homo sapiens
9688	nucleoporin 93kDa	Homo sapiens
1994	ELAV (embryonic lethal, abnormal vision, Drosophila)-like 1 (Hu antigen R)	Homo sapiens
79654	HECT domain containing 3	Homo sapiens
80349	WD repeat domain 61	Homo sapiens
781	calcium channel, voltage-dependent, alpha 2/delta subunit 1	Homo sapiens
26003	golgi reassembly stacking protein 2, 55kDa	Homo sapiens
113178	secretory carrier membrane protein 4	Homo sapiens
148362	chromosome 1 open reading frame 58	Homo sapiens
25852	armadillo repeat containing 8	Homo sapiens
51056	leucine aminopeptidase 3	Homo sapiens
5424	polymerase (DNA directed), delta 1, catalytic subunit 125kDa	Homo sapiens
3320	heat shock protein 90kDa alpha (cytosolic), class A member 2; heat shock protein 90kDa alpha (cytosolic), class A member 1	Homo sapiens
3324	heat shock protein 90kDa alpha (cytosolic), class A member 2; heat shock protein 90kDa alpha (cytosolic), class A member 1	Homo sapiens
9045	ribosomal protein L14	Homo sapiens
5911	RAP2A, member of RAS oncogene family	Homo sapiens
7334	ubiquitin-conjugating enzyme E2N (UBC13 homolog, yeast)	Homo sapiens
23517	superkiller viralicidic activity 2-like 2 (S. cerevisiae)	Homo sapiens
9320	thyroid hormone receptor interactor 12	Homo sapiens
11269	DEAD (Asp-Glu-Ala-As) box polypeptide 19B	Homo sapiens
645548	heat shock 60kDa protein 1 (chaperonin) pseudogene 5; heat shock 60kDa protein 1 (chaperonin) pseudogene 6; heat shock 60kDa protein 1 (chaperonin) pseudogene 1; heat shock 60kDa protein 1 (chaperonin) pseudogene 4; heat shock 60kDa protein 1 (chaperonin)	Homo sapiens
643300	heat shock 60kDa protein 1 (chaperonin) pseudogene 5; heat shock 60kDa protein 1 (chaperonin) pseudogene 6; heat shock 60kDa protein 1 (chaperonin) pseudogene 1; heat shock 60kDa protein 1 (chaperonin) pseudogene 4; heat shock 60kDa protein 1 (chaperonin)	Homo sapiens
3329	heat shock 60kDa protein 1 (chaperonin) pseudogene 5; heat shock 60kDa protein 1 (chaperonin) pseudogene 6; heat shock 60kDa protein 1 (chaperonin) pseudogene 1; heat shock 60kDa protein 1 (chaperonin) pseudogene 4; heat shock 60kDa protein 1 (chaperonin)	Homo sapiens
644745	heat shock 60kDa protein 1 (chaperonin) pseudogene 5; heat shock 60kDa protein 1 (chaperonin) pseudogene 6; heat shock 60kDa protein 1 (chaperonin) pseudogene 1; heat shock 60kDa protein 1 (chaperonin) pseudogene 4; heat shock 60kDa protein 1 (chaperonin)	Homo sapiens
345041	heat shock 60kDa protein 1 (chaperonin) pseudogene 5; heat shock 60kDa protein 1 (chaperonin) pseudogene 6; heat shock 60kDa protein 1 (chaperonin) pseudogene 1; heat shock 60kDa protein 1 (chaperonin) pseudogene 4; heat shock 60kDa protein 1 (chaperonin)	Homo sapiens
7345	ubiquitin carboxyl-terminal esterase L1 (ubiquitin thiolesterase)	Homo sapiens
116985	ArfGAP with RhoGAP domain, ankyrin repeat and PH domain 1	Homo sapiens
23637	RAB GTPase activating protein 1	Homo sapiens
4643	myosin IE	Homo sapiens
23265	exocyst complex component 7	Homo sapiens
27350	apolipoprotein B mRNA editing enzyme, catalytic polypeptide-like 3C	Homo sapiens
81	actinin, alpha 4	Homo sapiens
5580	protein kinase C, delta	Homo sapiens
5610	eukaryotic translation initiation factor 2-alpha kinase 2	Homo sapiens
5413	septin 5	Homo sapiens
51534	Vps20-associated 1 homolog (S. cerevisiae)	Homo sapiens
11260	exportin, tRNA (nuclear export receptor for tRNAs); similar to Exportin-T (tRNA exportin) (Exportin(tRNA))	Homo sapiens
9533	polymerase (RNA) I polypeptide C, 30kDa	Homo sapiens
3098	hexokinase 1	Homo sapiens
5781	protein tyrosine phosphatase, non-receptor type 11; similar to protein tyrosine phosphatase, non-receptor type 11	Homo sapiens
10096	ARP3 actin-related protein 3 homolog (yeast)	Homo sapiens
824	calpain 2, (m/II) large subunit	Homo sapiens

2288	FK506 binding protein 4, 59kDa	Homo sapiens
27072	vacuolar protein sorting 41 homolog (S. cerevisiae)	Homo sapiens
5091	pyruvate carboxylase	Homo sapiens
6643	sorting nexin 2	Homo sapiens
5586	protein kinase N2	Homo sapiens
6429	splicing factor, arginine/serine-rich 4	Homo sapiens
166378	spermatogenesis associated 5	Homo sapiens
55770	exocyst complex component 2	Homo sapiens
10487	CAP, adenylate cyclase-associated protein 1 (yeast)	Homo sapiens
6051	arginyl aminopeptidase (aminopeptidase B)	Homo sapiens
8939	far upstream element (FUSE) binding protein 3	Homo sapiens
9821	RB1-inducible coiled-coil 1	Homo sapiens
55922	NFKB repressing factor	Homo sapiens
4839	NOP2 nucleolar protein homolog (yeast)	Homo sapiens
1781	similar to dynein cytoplasmic 1 intermediate chain 2; dynein, cytoplasmic 1, intermediate chain 2	Homo sapiens
11051	nudix (nucleoside diphosphate linked moiety X)-type motif 21	Homo sapiens
2790	DnaJ (Hsp40) homolog, subfamily C , member 25; guanine nucleotide binding protein (G protein), gamma 10; DNAJC25-GNG10 readthrough transcript	Homo sapiens
8895	copine III	Homo sapiens
2192	fibulin 1	Homo sapiens
7411	von Hippel-Lindau binding protein 1	Homo sapiens
6793	serine/threonine kinase 10	Homo sapiens
10541	similar to Acidic leucine-rich nuclear phosphoprotein 32 family member B (PHAPI2 protein) (Silver-stainable protein SSP29) (Acidic protein rich in leucines); acidic (leucine-rich) nuclear phosphoprotein 32 family, member B	Homo sapiens
8450	cullin 4B	Homo sapiens
11316	coatomer protein complex, subunit epsilon	Homo sapiens
7058	thrombospondin 2	Homo sapiens
10483	Sec23 homolog B (S. cerevisiae)	Homo sapiens
5351	procollagen-lysine 1, 2-oxoglutarate 5-dioxygenase 1	Homo sapiens
10159	ATPase, H+ transporting, lysosomal accessory protein 2	Homo sapiens
1212	clathrin, light chain (Lcb)	Homo sapiens
56922	methylcrotonoyl-Coenzyme A carboxylase 1 (alpha)	Homo sapiens
54039	poly(rC) binding protein 3	Homo sapiens
23019	CCR4-NOT transcription complex, subunit 1	Homo sapiens
5756	twinfilin, actin-binding protein, homolog 1 (Drosophila)	Homo sapiens
2787	guanine nucleotide binding protein (G protein), gamma 5	Homo sapiens
301	annexin A1	Homo sapiens
5684	proteasome (prosome, macropain) subunit, alpha type, 3	Homo sapiens
9567	GTP binding protein 1	Homo sapiens
23365	Rho guanine nucleotide exchange factor (GEF) 12	Homo sapiens
5621	prion protein	Homo sapiens
1075	cathepsin C	Homo sapiens
64422	ATG3 autophagy related 3 homolog (S. cerevisiae)	Homo sapiens
27348	torsin family 1, member B (torsin B)	Homo sapiens
6205	ribosomal protein S11 pseudogene 5; ribosomal protein S11	Homo sapiens
56889	transmembrane 9 superfamily member 3	Homo sapiens
23350	U2-associated SR140 protein	Homo sapiens
34	acyl-Coenzyme A dehydrogenase, C-4 to C-12 straight chain	Homo sapiens
29763	protein kinase C and casein kinase substrate in neurons 3	Homo sapiens
91056	DKFZp761E198 protein	Homo sapiens
11021	similar to hCG1778032; RAB35, member RAS oncogene family	Homo sapiens
54892	non-SMC condensin II complex, subunit G2	Homo sapiens
6653	sortilin-related receptor, L(DLR class) A repeats-containing	Homo sapiens
163	adaptor-related protein complex 2, beta 1 subunit	Homo sapiens
2621	similar to growth arrest-specific 6; growth arrest-specific 6	Homo sapiens
7266	DnaJ (Hsp40) homolog, subfamily C, member 7	Homo sapiens
7105	tetraspanin 6	Homo sapiens
51155	hematological and neurological expressed 1	Homo sapiens
440917	similar to 14-3-3 protein epsilon (14-3-3E) (Mitochondrial import stimulation factor L subunit) (MSF L); tyrosine 3-monooxygenase/tryptophan 5-monooxygenase activation protein, epsilon polypeptide	Homo sapiens
7531	similar to 14-3-3 protein epsilon (14-3-3E) (Mitochondrial import stimulation factor L subunit) (MSF L); tyrosine 3-monooxygenase/tryptophan 5-monooxygenase activation protein, epsilon polypeptide	Homo sapiens
116442	RAB39B, member RAS oncogene family	Homo sapiens
5134	programmed cell death 2	Homo sapiens
80185	chromosome 8 open reading frame 41	Homo sapiens
8073	protein tyrosine phosphatase type IVA, member 2	Homo sapiens
3094	histidine triad nucleotide binding protein 1	Homo sapiens
11188	nischarin	Homo sapiens
55229	pantothenate kinase 4	Homo sapiens
3251	hypoxanthine phosphoribosyltransferase 1	Homo sapiens
26046	ring finger protein 160	Homo sapiens
2950	glutathione S-transferase pi 1	Homo sapiens
27339	PRP19/PSO4 pre-mRNA processing factor 19 homolog (S. cerevisiae)	Homo sapiens
10273	STIP1 homology and U-box containing protein 1	Homo sapiens
2040	stomatin	Homo sapiens
9125	RCD1 required for cell differentiation1 homolog (S. pombe)	Homo sapiens
1825	desmocollin 3	Homo sapiens
4830	non-metastatic cells 1, protein (NM23A) expressed in; NME1-NME2 readthrough transcript; non-metastatic cells 2, protein (NM23B) expressed in	Homo sapiens
4831	non-metastatic cells 1, protein (NM23A) expressed in; NME1-NME2 readthrough transcript; non-metastatic cells 2, protein (NM23B) expressed in	Homo sapiens

654364	non-metastatic cells 1, protein (NM23A) expressed in; NME1-NME2 readthrough transcript; non-metastatic cells 2, protein (NM23B) expressed in	Homo sapiens
26973	cysteine and histidine-rich domain (CHORD)-containing 1; cysteine and histidine-rich domain (CHORD)-containing 1 pseudogene	Homo sapiens
51599	lipolysis stimulated lipoprotein receptor	Homo sapiens
146691	target of myb1-like 2 (chicken)	Homo sapiens
2771	guanine nucleotide binding protein (G protein), alpha inhibiting activity polypeptide 2	Homo sapiens
55577	N-acetylglucosamine kinase	Homo sapiens
27183	vacuolar protein sorting 4 homolog A (S. cerevisiae)	Homo sapiens
10121	ARP1 actin-related protein 1 homolog A, centractin alpha (yeast)	Homo sapiens
6645	syntrophin, beta 2 (dystrophin-associated protein A1, 59kDa, basic component 2)	Homo sapiens
26002	monooxygenase, DBH-like 1	Homo sapiens
10938	EH-domain containing 1	Homo sapiens
10755	GIPC PDZ domain containing family, member 1	Homo sapiens
1514	cathepsin L1	Homo sapiens
5050	platelet-activating factor acetylhydrolase, isoform Ib, subunit 3 (29kDa)	Homo sapiens
5706	proteasome (prosome, macropain) 26S subunit, ATPase, 6	Homo sapiens
391	ras homolog gene family, member G (rho G)	Homo sapiens
23284	latrophilin 3	Homo sapiens
10213	proteasome (prosome, macropain) 26S subunit, non-ATPase, 14	Homo sapiens
79784	myosin, heavy chain 14	Homo sapiens
5062	p21 protein (Cdc42/Rac)-activated kinase 2	Homo sapiens
10006	abl-interactor 1	Homo sapiens
10652	YKT6 v-SNARE homolog (S. cerevisiae)	Homo sapiens
7372	uridine monophosphate synthetase	Homo sapiens
10920	COP9 constitutive photomorphogenic homolog subunit 8 (Arabidopsis)	Homo sapiens
8454	cullin 1	Homo sapiens
7360	UDP-glucose pyrophosphorylase 2	Homo sapiens
2239	glypican 4	Homo sapiens
2318	filamin C, gamma (actin binding protein 280)	Homo sapiens
55703	polymerase (RNA) III (DNA directed) polypeptide B	Homo sapiens
8624	proteasome (prosome, macropain) assembly chaperone 1	Homo sapiens
9943	oxidative-stress responsive 1	Homo sapiens
5007	oxysterol binding protein	Homo sapiens
9521	eukaryotic translation elongation factor 1 epsilon 1	Homo sapiens
2314	flightless I homolog (Drosophila)	Homo sapiens
5837	phosphorylase, glycogen, muscle	Homo sapiens
6193	ribosomal protein S5	Homo sapiens
2035	erythrocyte membrane protein band 4.1 (elliptocytosis 1, RH-linked)	Homo sapiens
1293	collagen, type VI, alpha 3	Homo sapiens
10694	similar to chaperonin containing TCP1, subunit 8 (theta); chaperonin containing TCP1, subunit 8 (theta)	Homo sapiens
10598	AHA1, activator of heat shock 90kDa protein ATPase homolog 1 (yeast)	Homo sapiens
9146	hepatocyte growth factor-regulated tyrosine kinase substrate	Homo sapiens
9918	non-SMC condensin I complex, subunit D2	Homo sapiens
55737	hypothetical protein LOC100133770; vacuolar protein sorting 35 homolog (S. cerevisiae)	Homo sapiens
8411	early endosome antigen 1	Homo sapiens
23406	coactosin-like 1 (Dictyostelium)	Homo sapiens
23047	PDS5, regulator of cohesion maintenance, homolog B (S. cerevisiae)	Homo sapiens
5431	polymerase (RNA) II (DNA directed) polypeptide B, 140kDa	Homo sapiens
2775	guanine nucleotide binding protein (G protein), alpha activating activity polypeptide O	Homo sapiens
373	tripartite motif-containing 23	Homo sapiens
1917	eukaryotic translation elongation factor 1 alpha 2	Homo sapiens
10970	cytoskeleton-associated protein 4	Homo sapiens
56647	BRCA2 and CDKN1A interacting protein	Homo sapiens
7316	ubiquitin C	Homo sapiens
5802	protein tyrosine phosphatase, receptor type, S	Homo sapiens
3838	karyopherin alpha 2 (RAG cohort 1, importin alpha 1); karyopherin alpha-2 subunit like	Homo sapiens
1650	dolichyl-diphosphooligosaccharide-protein glycosyltransferase	Homo sapiens
6633	small nuclear ribonucleoprotein D2 polypeptide 16.5kDa; similar to hCG2040270	Homo sapiens
22948	chaperonin containing TCP1, subunit 5 (epsilon)	Homo sapiens
1487	C-terminal binding protein 1	Homo sapiens
334	amyloid beta (A4) precursor-like protein 2	Homo sapiens
7317	ubiquitin-like modifier activating enzyme 1	Homo sapiens
9529	BCL2-associated athanogene 5	Homo sapiens
23413	frequenin homolog (Drosophila)	Homo sapiens
3987	LIM and senescent cell antigen-like domains 1	Homo sapiens
54407	solute carrier family 38, member 2	Homo sapiens
1072	cofilin 1 (non-muscle)	Homo sapiens
8218	clathrin, heavy chain-like 1	Homo sapiens
1292	collagen, type VI, alpha 2	Homo sapiens
79659	dynein, cytoplasmic 2, heavy chain 1	Homo sapiens
7533	tyrosine 3-monooxygenase/tryptophan 5-monooxygenase activation protein, eta polypeptide	Homo sapiens
928	CD9 molecule	Homo sapiens
221184	copine II	Homo sapiens
5327	plasminogen activator, tissue	Homo sapiens
5499	protein phosphatase 1, catalytic subunit, alpha isoform	Homo sapiens
7416	voltage-dependent anion channel 1; similar to voltage-dependent anion channel 1	Homo sapiens
23347	structural maintenance of chromosomes flexible hinge domain containing 1	Homo sapiens
3685	integrin, alpha V (vitronectin receptor, alpha polypeptide, antigen CD51)	Homo sapiens
6228	ribosomal protein S23	Homo sapiens
84661	dpy-30 homolog (C. elegans)	Homo sapiens
10658	CUG triplet repeat, RNA binding protein 1	Homo sapiens
9219	metastasis associated 1 family, member 2	Homo sapiens
10327	aldo-keto reductase family 1, member A1 (aldehyde reductase)	Homo sapiens
1737	dihydrolipoamide S-acetyltransferase	Homo sapiens

51068	NMD3 homolog (S. cerevisiae)	Homo sapiens
51028	vacuolar protein sorting 36 homolog (S. cerevisiae)	Homo sapiens
22818	coatomer protein complex, subunit zeta 1	Homo sapiens
8826	IQ motif containing GTPase activating protein 1	Homo sapiens
8878	sequestosome 1	Homo sapiens
8471	insulin receptor substrate 4	Homo sapiens
51013	exosome component 1	Homo sapiens
10480	eukaryotic translation initiation factor 3, subunit M	Homo sapiens
4926	nuclear mitotic apparatus protein 1	Homo sapiens
60496	aminoadipate-semialdehyde dehydrogenase-phosphopantetheinyl transferase	Homo sapiens
6844	vesicle-associated membrane protein 2 (synaptobrevin 2)	Homo sapiens
382	ADP-ribosylation factor 6	Homo sapiens
174	alpha-fetoprotein	Homo sapiens
51399	trafficking protein particle complex 4	Homo sapiens
10971	tyrosine 3-monooxygenase/tryptophan 5-monooxygenase activation protein, theta polypeptide	Homo sapiens
23122	cytoplasmic linker associated protein 2	Homo sapiens
25782	RAB3 GTPase activating protein subunit 2 (non-catalytic)	Homo sapiens
8892	eukaryotic translation initiation factor 2B, subunit 2 beta, 39kDa	Homo sapiens
3305	heat shock 70kDa protein 1-like	Homo sapiens
3371	tenascin C	Homo sapiens
201626	phosphodiesterase 12	Homo sapiens
29780	parvin, beta	Homo sapiens
7077	TIMP metallopeptidase inhibitor 2	Homo sapiens
2195	FAT tumor suppressor homolog 1 (Drosophila)	Homo sapiens
1445	c-src tyrosine kinase	Homo sapiens
9061	3'-phosphoadenosine 5'-phosphosulfate synthase 1	Homo sapiens
6422	secreted frizzled-related protein 1	Homo sapiens
79932	KIAA0319-like	Homo sapiens
59342	serine carboxypeptidase 1	Homo sapiens
56984	proteasome (prosome, macropain) assembly chaperone 2	Homo sapiens
22827	poly-U binding splicing factor 60KDa	Homo sapiens
3185	heterogeneous nuclear ribonucleoprotein F	Homo sapiens
1729	diaphanous homolog 1 (Drosophila)	Homo sapiens
5119	chromatin modifying protein 1A	Homo sapiens
9742	intraflagellar transport 140 homolog (Chlamydomonas)	Homo sapiens
5538	palmitoyl-protein thioesterase 1	Homo sapiens
4646	myosin VI	Homo sapiens
83442	SH3 domain binding glutamic acid-rich protein like 3	Homo sapiens
7520	X-ray repair complementing defective repair in Chinese hamster cells 5 (double-strand-break rejoining)	Homo sapiens
682	basigin (Ok blood group)	Homo sapiens
11273	ataxin 2-like	Homo sapiens
23136	erythrocyte membrane protein band 4.1-like 3	Homo sapiens
23354	HAUS augmin-like complex, subunit 5	Homo sapiens
11091	WD repeat domain 5	Homo sapiens
3703	STT3, subunit of the oligosaccharyltransferase complex, homolog A (S. cerevisiae)	Homo sapiens
9618	TNF receptor-associated factor 4	Homo sapiens
64682	anaphase promoting complex subunit 1; similar to anaphase promoting complex subunit 1	Homo sapiens
10544	protein C receptor, endothelial (EPCR)	Homo sapiens
9919	SEC16 homolog A (S. cerevisiae)	Homo sapiens
10142	A kinase (PRKA) anchor protein (yotiao) 9	Homo sapiens
873	carbonyl reductase 1	Homo sapiens
83737	itchy E3 ubiquitin protein ligase homolog (mouse)	Homo sapiens
10048	RAN binding protein 9	Homo sapiens
9019	myelin protein zero-like 1	Homo sapiens
1756	dystrophin	Homo sapiens
23534	transportin 3	Homo sapiens
3956	lectin, galactoside-binding, soluble, 1	Homo sapiens
8841	histone deacetylase 3	Homo sapiens
7167	TPI1 pseudogene; triosephosphate isomerase 1	Homo sapiens
729708	TPI1 pseudogene; triosephosphate isomerase 1	Homo sapiens
92597	MOB1, Mps One Binder kinase activator-like 1A (yeast)	Homo sapiens
2778	GNAS complex locus	Homo sapiens
23016	exosome component 7	Homo sapiens
85461	tetratricopeptide repeat, ankyrin repeat and coiled-coil containing 1	Homo sapiens
1464	chondroitin sulfate proteoglycan 4	Homo sapiens
80781	collagen, type XVIII, alpha 1	Homo sapiens
22978	5'-nucleotidase, cytosolic II	Homo sapiens
8289	AT rich interactive domain 1A (SWI-like)	Homo sapiens
57609	DIP2 disco-interacting protein 2 homolog B (Drosophila)	Homo sapiens
8723	sorting nexin 4	Homo sapiens
5792	protein tyrosine phosphatase, receptor type, F	Homo sapiens
523	ATPase, H+ transporting, lysosomal 70kDa, V1 subunit A	Homo sapiens
1936	eukaryotic translation elongation factor 1 delta (guanine nucleotide exchange protein)	Homo sapiens
51637	chromosome 14 open reading frame 166	Homo sapiens
4343	Mov10, Moloney leukemia virus 10, homolog (mouse)	Homo sapiens
6526	solute carrier family 5 (sodium/myo-inositol cotransporter), member 3	Homo sapiens
2770	guanine nucleotide binding protein (G protein), alpha inhibiting activity polypeptide 1	Homo sapiens
6742	single-stranded DNA binding protein 1	Homo sapiens
4811	nidogen 1	Homo sapiens
1207	chloride channel, nucleotide-sensitive, 1A	Homo sapiens
10575	chaperonin containing TCP1, subunit 4 (delta)	Homo sapiens
2475	mechanistic target of rapamycin (serine/threonine kinase)	Homo sapiens
83660	talin 2	Homo sapiens
5037	phosphatidylethanolamine binding protein 1	Homo sapiens
493	ATPase, Ca++ transporting, plasma membrane 4	Homo sapiens

738	chromosome 11 open reading frame2	Homo sapiens
23228	phospholipase C-like 2	Homo sapiens
10491	cartilage associated protein	Homo sapiens
6602	SWI/SNF related, matrix associated, actin dependent regulator of chromatin, subfamily d, member 1	Homo sapiens
1889	endothelin converting enzyme 1	Homo sapiens
1793	dedicator of cytokinesis 1	Homo sapiens
10419	protein arginine methyltransferase 5	Homo sapiens
22931	RAB18, member RAS oncogene family	Homo sapiens
5879	ras-related C3 botulinum toxin substrate 1 (rho family, small GTP binding protein Rac1)	Homo sapiens
3911	laminin, alpha 5	Homo sapiens
6184	ribophorin I	Homo sapiens
5862	RAB2A, member RAS oncogene family	Homo sapiens
1984	eukaryotic translation initiation factor 5A; eukaryotic translation initiation factor 5A-like 1	Homo sapiens
143244	eukaryotic translation initiation factor 5A; eukaryotic translation initiation factor 5A-like 1	Homo sapiens
57136	chromosome 20 open reading frame 3	Homo sapiens
1639	dynactin 1 (p150, glued homolog, Drosophila)	Homo sapiens
6894	TAR (HIV-1) RNA binding protein 1	Homo sapiens
23760	phosphatidylinositol transfer protein, beta	Homo sapiens
118	adducin 1 (alpha)	Homo sapiens
9138	Rho guanine nucleotide exchange factor (GEF) 1	Homo sapiens
4313	matrix metallopeptidase 2 (gelatinase A, 72kDa gelatinase, 72kDa type IV collagenase)	Homo sapiens
6230	ribosomal protein S25 pseudogene 8; ribosomal protein S25	Homo sapiens
5167	ectonucleotide pyrophosphatase/phosphodiesterase 1	Homo sapiens
72	actin, gamma 2, smooth muscle, enteric	Homo sapiens
5754	PTK7 protein tyrosine kinase 7	Homo sapiens
729	complement component 6	Homo sapiens
1937	eukaryotic translation elongation factor 1 gamma	Homo sapiens
4001	lamin B1	Homo sapiens
10640	exocyst complex component 5	Homo sapiens
10946	splicing factor 3a, subunit 3, 60kDa	Homo sapiens
10512	sema domain, immunoglobulin domain (Ig), short basic domain, secreted, (semaphorin) 3C	Homo sapiens
10514	MYB binding protein (P160) 1a	Homo sapiens
79228	THO complex 6 homolog (Drosophila)	Homo sapiens
2495	ferritin, heavy polypeptide 1; ferritin, heavy polypeptide-like 16; similar to ferritin, heavy polypeptide 1; ferritin, heavy polypeptide-like 3 pseudogene	Homo sapiens
81929	SEH1-like (S. cerevisiae)	Homo sapiens
2664	GDP dissociation inhibitor 1	Homo sapiens
23633	karyopherin alpha 6 (importin alpha 7)	Homo sapiens
4143	methionine adenosyltransferase I, alpha	Homo sapiens
55720	TSR1, 20S rRNA accumulation, homolog (S. cerevisiae)	Homo sapiens
4851	Notch homolog 1, translocation-associated (Drosophila)	Homo sapiens
7284	Tu translation elongation factor, mitochondrial	Homo sapiens
135228	CD109 molecule	Homo sapiens
6678	secreted protein, acidic, cysteine-rich (osteonectin)	Homo sapiens
10949	heterogeneous nuclear ribonucleoprotein A0	Homo sapiens
6386	syndecan binding protein (syntenin)	Homo sapiens
10043	hypothetical LOC100128526; target of myb1 (chicken)	Homo sapiens
91663	myeloid-associated differentiation marker	Homo sapiens
8428	serine/threonine kinase 24 (STE20 homolog, yeast)	Homo sapiens
3700	inter-alpha (globulin) inhibitor H4 (plasma Kallikrein-sensitive glycoprotein)	Homo sapiens
57020	chromosome 16 open reading frame 62	Homo sapiens
51119	Shwachman-Bodian-Diamond syndrome pseudogene; Shwachman-Bodian-Diamond syndrome	Homo sapiens
23521	ribosomal protein L13a pseudogene 7; ribosomal protein L13a pseudogene 5; ribosomal protein L13a pseudogene 16; ribosomal protein L13a; ribosomal protein L13a pseudogene 18	Homo sapiens
51202	DEAD (Asp-Glu-Ala-Asp) box polypeptide 47	Homo sapiens
8661	eukaryotic translation initiation factor 3, subunit A	Homo sapiens
115290	F-box protein 17	Homo sapiens
5768	quiescin Q6 sulfhydryl oxidase 1	Homo sapiens
5584	protein kinase C, iota	Homo sapiens
529	ATPase, H+ transporting, lysosomal 31kDa, V1 subunit E1	Homo sapiens
5705	proteasome (prosome, macropain) 26S subunit, ATPase, 5	Homo sapiens
9445	integral membrane protein 2B	Homo sapiens
9871	SEC24 family, member D (S. cerevisiae)	Homo sapiens
55622	tetratricopeptide repeat domain 27	Homo sapiens
2043	EPH receptor A4	Homo sapiens
8702	UDP-Gal:betaGlcNAc beta 1,4- galactosyltransferase, polypeptide 4	Homo sapiens
6133	ribosomal protein L9; ribosomal protein L9 pseudogene 25	Homo sapiens
3419	isocitrate dehydrogenase 3 (NAD+) alpha	Homo sapiens
63891	ring finger protein 123	Homo sapiens
4629	myosin, heavy chain 11, smooth muscle	Homo sapiens
56954	nitrilase family, member 2	Homo sapiens
26227	phosphoglycerate dehydrogenase	Homo sapiens
7052	transglutaminase 2 (C polypeptide, protein-glutamine-gamma-glutamyltransferase)	Homo sapiens
5036	proliferation-associated 2G4, 38kDa; proliferation-associated 2G4 pseudogene 4	Homo sapiens
10992	splicing factor 3b, subunit 2, 145kDa	Homo sapiens
8621	cell division cycle 2-like 5 (cholinesterase-related cell division controller)	Homo sapiens
3184	heterogeneous nuclear ribonucleoprotein D (AU-rich element RNA binding protein 1, 37kDa)	Homo sapiens
10728	prostaglandin E synthase 3 (cytosolic)	Homo sapiens
23352	ubiquitin protein ligase E3 component n-recognin 4	Homo sapiens
7045	transforming growth factor, beta-induced, 68kDa	Homo sapiens
9894	TEL2, telomere maintenance 2, homolog (S. cerevisiae)	Homo sapiens
27336	HIV-1 Tat specific factor 1	Homo sapiens
6427	splicing factor, arginine/serine-rich 2	Homo sapiens
8425	latent transforming growth factor beta binding protein 4	Homo sapiens
6782	heat shock protein 70kDa family, member 13	Homo sapiens

57465	TBC1 domain family, member 24	Homo sapiens
23256	sec1 family domain containing 1	Homo sapiens
6301	seryl-tRNA synthetase	Homo sapiens
26000	TBC1 domain family, member 10B	Homo sapiens
829	capping protein (actin filament) muscle Z-line, alpha 1	Homo sapiens
4756	neogenin homolog 1 (chicken)	Homo sapiens
8175	splicing factor 3a, subunit 2, 66kDa	Homo sapiens
5033	prolyl 4-hydroxylase, alpha polypeptide I	Homo sapiens
2617	glycyl-tRNA synthetase	Homo sapiens
4301	similar to Afadin (Protein AF-6); myeloid/lymphoid or mixed-lineage leukemia (trithorax homolog, Drosophila); translocated to, 4	Homo sapiens
79633	FAT tumor suppressor homolog 4 (Drosophila)	Homo sapiens
9798	similar to CG10103; KIAA0174	Homo sapiens
728533	similar to CG10103; KIAA0174	Homo sapiens
6164	ribosomal protein L34	Homo sapiens
9978	ring-box 1	Homo sapiens
6810	syntaxin 4	Homo sapiens
2287	FK506 binding protein 3, 25kDa	Homo sapiens
23476	bromodomain containing 4	Homo sapiens
4436	mutS homolog 2, colon cancer, nonpolyposis type 1 (E. coli)	Homo sapiens
3796	kinesin heavy chain member 2A	Homo sapiens
1983	eukaryotic translation initiation factor 5	Homo sapiens
216	aldehyde dehydrogenase 1 family, member A1	Homo sapiens
2316	filamin A, alpha (actin binding protein 280)	Homo sapiens
10135	nicotinamide phosphoribosyltransferase	Homo sapiens
10436	EMG1 nucleolar protein homolog (S. cerevisiae)	Homo sapiens
23513	scribbled homolog (Drosophila)	Homo sapiens
7419	voltage-dependent anion channel 3	Homo sapiens
79443	FYVE and coiled-coil domain containing 1	Homo sapiens
27250	programmed cell death 4 (neoplastic transformation inhibitor)	Homo sapiens
6134	ribosomal protein L10; ribosomal protein L10 pseudogene 15; ribosomal protein L10 pseudogene 6; ribosomal protein L10 pseudogene 16; ribosomal protein L10 pseudogene 9	Homo sapiens
4641	myosin IC	Homo sapiens
55845	chromosome 3 open reading frame 10	Homo sapiens
10584	collectin sub-family member 10 (C-type lectin)	Homo sapiens
3636	inositol polyphosphate phosphatase-like 1	Homo sapiens
5795	protein tyrosine phosphatase, receptor type, J	Homo sapiens
51735	Rap guanine nucleotide exchange factor (GEF) 6	Homo sapiens
60314	chromosome 12 open reading frame 10	Homo sapiens
8260	ARD1 homolog A, N-acetyltransferase (S. cerevisiae)	Homo sapiens
4361	MRE11 meiotic recombination 11 homolog A (S. cerevisiae)	Homo sapiens
23176	septin 8	Homo sapiens
22894	DIS3 mitotic control homolog (S. cerevisiae)	Homo sapiens
830	capping protein (actin filament) muscle Z-line, alpha 2	Homo sapiens
259217	heat shock 70kDa protein 12A	Homo sapiens
83690	cysteine-rich secretory protein LCCL domain containing 1	Homo sapiens
3631	inositol polyphosphate-4-phosphatase, type I, 107kDa	Homo sapiens
5906	RAP1A, member of RAS oncogene family	Homo sapiens
92181	ubiquitin domain containing 2	Homo sapiens
129563	DIS3 mitotic control homolog (S. cerevisiae)-like 2	Homo sapiens
1362	carboxypeptidase D	Homo sapiens
7511	X-prolyl aminopeptidase (aminopeptidase P) 1, soluble	Homo sapiens
3689	integrin, beta 2 (complement component 3 receptor 3 and 4 subunit)	Homo sapiens
22982	DIP2 disco-interacting protein 2 homolog C (Drosophila)	Homo sapiens
3678	integrin, alpha 5 (fibronectin receptor, alpha polypeptide)	Homo sapiens
231	aldo-keto reductase family 1, member B1 (aldose reductase)	Homo sapiens
57614	KIAA1468	Homo sapiens
10152	abl interactor 2	Homo sapiens
23397	non-SMC condensin I complex, subunit H	Homo sapiens
57805	KIAA1967	Homo sapiens
23516	solute carrier family 39 (zinc transporter), member 14	Homo sapiens
59345	guanine nucleotide binding protein (G protein), beta polypeptide 4	Homo sapiens
2098	esterase D/formylglutathione hydrolase	Homo sapiens
3339	heparan sulfate proteoglycan 2	Homo sapiens
8669	eukaryotic translation initiation factor 3, subunit J	Homo sapiens
6143	ribosomal protein L19; ribosomal protein L19 pseudogene 12	Homo sapiens
23042	pyridoxal-dependent decarboxylase domain containing 1	Homo sapiens
6142	ribosomal protein L18a pseudogene 6; ribosomal protein L18a	Homo sapiens
10960	lectin, mannose-binding 2	Homo sapiens
6202	ribosomal protein S8; ribosomal protein S8 pseudogene 8; ribosomal protein S8 pseudogene 10	Homo sapiens
57504	metastasis associated 1 family, member 3	Homo sapiens
8566	pyridoxal (pyridoxine, vitamin B6) kinase	Homo sapiens
64795	required for meiotic nuclear division 5 homolog A (S. cerevisiae)	Homo sapiens
10525	hypoxia up-regulated 1	Homo sapiens
2896	granulin	Homo sapiens
6416	mitogen-activated protein kinase kinase 4	Homo sapiens
1153	cold inducible RNA binding protein	Homo sapiens
23332	cytoplasmic linker associated protein 1	Homo sapiens
1457	casein kinase 2, alpha 1 polypeptide pseudogene; casein kinase 2, alpha 1 polypeptide	Homo sapiens
5983	replication factor C (activator 1) 3, 38kDa	Homo sapiens
5094	poly(rC) binding protein 2	Homo sapiens
645	biliverdin reductase B (flavin reductase (NADPH))	Homo sapiens
6146	ribosomal protein L22 pseudogene 11; ribosomal protein L22	Homo sapiens
1622	diazepam binding inhibitor (GABA receptor modulator, acyl-Coenzyme A binding protein)	Homo sapiens
57511	component of oligomeric golgi complex 6	Homo sapiens
5214	phosphofructokinase, platelet	Homo sapiens

80019	ubiquitin domain containing 1	Homo sapiens
55165	centrosomal protein 55kDa	Homo sapiens
307	annexin A4	Homo sapiens
5211	phosphofructokinase, liver	Homo sapiens
3692	eukaryotic translation initiation factor 6	Homo sapiens
11128	polymerase (RNA) III (DNA directed) polypeptide A, 155kDa	Homo sapiens
91754	NIMA (never in mitosis gene a)- related kinase 9	Homo sapiens
6208	ribosomal protein S14	Homo sapiens
60626	resistance to inhibitors of cholinesterase 8 homolog A (C. elegans)	Homo sapiens
5690	proteasome (prosome, macropain) subunit, beta type, 2	Homo sapiens
84817	thioredoxin domain containing 17	Homo sapiens
481	ATPase, Na+/K+ transporting, beta 1 polypeptide	Homo sapiens
5565	protein kinase, AMP-activated, beta 2 non-catalytic subunit	Homo sapiens
6453	intersectin 1 (SH3 domain protein)	Homo sapiens
10254	signal transducing adaptor molecule (SH3 domain and ITAM motif) 2	Homo sapiens
10311	Down syndrome critical region gene 3	Homo sapiens
158078	eukaryotic translation elongation factor 1 alpha-like 7; eukaryotic translation elongation factor 1 alpha-like 3; similar to eukaryotic translation elongation factor 1 alpha 1; eukaryotic translation elongation factor 1 alpha 1	Homo sapiens
1915	eukaryotic translation elongation factor 1 alpha-like 7; eukaryotic translation elongation factor 1 alpha-like 3; similar to eukaryotic translation elongation factor 1 alpha 1; eukaryotic translation elongation factor 1 alpha 1	Homo sapiens
8970	histone cluster 1, H2bj	Homo sapiens
2171	fatty acid binding protein 5-like 2; fatty acid binding protein 5 (psoriasis-associated); fatty acid binding protein 5-like 8; fatty acid binding protein 5-like 7; fatty acid binding protein 5-like 9	Homo sapiens
728641	fatty acid binding protein 5-like 2; fatty acid binding protein 5 (psoriasis-associated); fatty acid binding protein 5-like 8; fatty acid binding protein 5-like 7; fatty acid binding protein 5-like 9	Homo sapiens
114034	target of EGR1, member 1 (nuclear)	Homo sapiens
64601	vacuolar protein sorting 16 homolog A (S. cerevisiae)	Homo sapiens
1774	deoxyribonuclease I-like 1	Homo sapiens
58490	regulation of nuclear pre-mRNA domain containing 1B	Homo sapiens
64710	nuclear casein kinase and cyclin-dependent kinase substrate 1	Homo sapiens
5176	serpin peptidase inhibitor, clade F (alpha-2 antiplasmin, pigment epithelium derived factor), member 1	Homo sapiens
23355	vacuolar protein sorting 8 homolog (S. cerevisiae)	Homo sapiens
158	adenylosuccinate lyase	Homo sapiens
4053	latent transforming growth factor beta binding protein 2	Homo sapiens
9513	fragile X mental retardation, autosomal homolog 2	Homo sapiens
10954	protein disulfide isomerase family A, member 5	Homo sapiens
8407	transgelin 2	Homo sapiens
7106	tetraspanin 4	Homo sapiens
2182	acyl-CoA synthetase long-chain family member 4	Homo sapiens
1281	collagen, type III, alpha 1	Homo sapiens
8451	cullin 4A	Homo sapiens
7373	collagen, type XIV, alpha 1	Homo sapiens
10772	FUS interacting protein (serine/arginine-rich) 1; similar to FUS interacting protein (serine-arginine rich) 1	Homo sapiens
23196	family with sequence similarity 120A	Homo sapiens
5315	similar to Pyruvate kinase, isozymes M1/M2 (Pyruvate kinase muscle isozyme) (Cytosolic thyroid hormone-binding protein) (CTHBP) (THBP1); pyruvate kinase, muscle	Homo sapiens
22916	nuclear cap binding protein subunit 2, 20kDa	Homo sapiens
6612	SMT3 suppressor of mif two 3 homolog 2 (S. cerevisiae) pseudogene; SMT3 suppressor of mif two 3 homolog 2 (S. cerevisiae); SMT3 suppressor of mif two 3 homolog 3 (S. cerevisiae)	Homo sapiens
6613	SMT3 suppressor of mif two 3 homolog 2 (S. cerevisiae) pseudogene; SMT3 suppressor of mif two 3 homolog 2 (S. cerevisiae); SMT3 suppressor of mif two 3 homolog 3 (S. cerevisiae)	Homo sapiens
5738	prostaglandin F2 receptor negative regulator	Homo sapiens
80727	tweety homolog 3 (Drosophila)	Homo sapiens
22907	DEAH (Asp-Glu-Ala-His) box polypeptide 30	Homo sapiens
2181	acyl-CoA synthetase long-chain family member 3	Homo sapiens
11124	Fas (TNFRSF6) associated factor 1	Homo sapiens
2162	coagulation factor XIII, A1 polypeptide	Homo sapiens
7283	tubulin, gamma 1; similar to Tubulin, gamma 1	Homo sapiens
10787	NCK-associated protein 1	Homo sapiens
10484	Sec23 homolog A (S. cerevisiae)	Homo sapiens
25873	ribosomal protein L36; ribosomal protein L36 pseudogene 14	Homo sapiens
54331	guanine nucleotide binding protein (G protein), gamma 2	Homo sapiens
84245	methylthioribose-1-phosphate isomerase homolog (S. cerevisiae)	Homo sapiens
5297	phosphatidylinositol 4-kinase, catalytic, alpha	Homo sapiens
1973	similar to eukaryotic translation initiation factor 4A; small nucleolar RNA, H/ACA box 67; eukaryotic translation initiation factor 4A, isoform 1	Homo sapiens
9898	ubiquitin associated protein 2-like	Homo sapiens
23392	KIAA0368	Homo sapiens
23503	zinc finger, FYVE domain containing 26	Homo sapiens
1192	chloride intracellular channel 1	Homo sapiens
56681	SAR1 homolog A (S. cerevisiae)	Homo sapiens
5425	polymerase (DNA directed), delta 2, regulatory subunit 50kDa	Homo sapiens
11196	SEC23 interacting protein	Homo sapiens
55573	CDV3 homolog (mouse)	Homo sapiens
89970	ring finger and SPRY domain containing 1	Homo sapiens
89953	kinesin light chain 4	Homo sapiens
10418	spondin 1, extracellular matrix protein	Homo sapiens
5976	UPF1 regulator of nonsense transcripts homolog (yeast)	Homo sapiens
613	breakpoint cluster region	Homo sapiens

8324	frizzled homolog 7 (Drosophila)	Homo sapiens
8623	acetylserotonin O-methyltransferase-like	Homo sapiens
6217	ribosomal protein S16 pseudogene 1; ribosomal protein S16 pseudogene 10; ribosomal protein S16	Homo sapiens
441876	ribosomal protein S16 pseudogene 1; ribosomal protein S16 pseudogene 10; ribosomal protein S16	Homo sapiens
729903	ribosomal protein S16 pseudogene 1; ribosomal protein S16 pseudogene 10; ribosomal protein S16	Homo sapiens
6118	replication protein A2, 32kDa	Homo sapiens
54584	guanine nucleotide binding protein (G protein), beta polypeptide 1-like	Homo sapiens
4069	lysozyme (renal amyloidosis)	Homo sapiens
152137	coiled-coil domain containing 50	Homo sapiens
5793	protein tyrosine phosphatase, receptor type, G	Homo sapiens
6814	syntaxin binding protein 3	Homo sapiens
6195	ribosomal protein S6 kinase, 90kDa, polypeptide 1	Homo sapiens
3073	hexosaminidase A (alpha polypeptide)	Homo sapiens
5954	reticulocalbin 1, EF-hand calcium binding domain	Homo sapiens
8985	procollagen-lysine, 2-oxoglutarate 5-dioxygenase 3	Homo sapiens
6129	ribosomal protein L7 pseudogene 26; ribosomal protein L7 pseudogene 16; ribosomal protein L7; ribosomal protein L7 pseudogene 32; ribosomal protein L7 pseudogene 23; ribosomal protein L7 pseudogene 24; ribosomal protein L7 pseudogene 20	Homo sapiens
9987	heterogeneous nuclear ribonucleoprotein D-like	Homo sapiens
1974	similar to eukaryotic translation initiation factor 4A2; eukaryotic translation initiation factor 4A, isoform 2	Homo sapiens
7803	protein tyrosine phosphatase type IVA, member 1	Homo sapiens
6305	SET binding factor 1; SET binding factor 1 pseudogene 1	Homo sapiens
6059	similar to ATP-binding cassette, sub-family E, member 1; ATP-binding cassette, sub-family E (OABP), member 1	Homo sapiens
6717	sorcin	Homo sapiens
64856	von Willebrand factor A domain containing 1	Homo sapiens
7726	tripartite motif-containing 26	Homo sapiens
65123	integrator complex subunit 3	Homo sapiens
83658	dynein, light chain, roadblock-type 1	Homo sapiens
64222	torsin family 3, member A	Homo sapiens
3716	Janus kinase 1	Homo sapiens
477	ATPase, Na+/K+ transporting, alpha 2 (+) polypeptide	Homo sapiens
4678	nuclear autoantigenic sperm protein (histone-binding)	Homo sapiens
8574	aldo-keto reductase family 7, member A2 (aflatoxin aldehyde reductase)	Homo sapiens
10840	aldehyde dehydrogenase 1 family, member L1	Homo sapiens
6293	vacuolar protein sorting 52 homolog (S. cerevisiae)	Homo sapiens
23515	MORC family CW-type zinc finger 3	Homo sapiens
25929	gem (nuclear organelle) associated protein 5	Homo sapiens
51400	protein phosphatase methylesterase 1	Homo sapiens
7112	thymopoietin	Homo sapiens
3065	histone deacetylase 1	Homo sapiens
23344	family with sequence similarity 62 (C2 domain containing), member A	Homo sapiens
988	CDC5 cell division cycle 5-like (S. pombe)	Homo sapiens
63916	engulfment and cell motility 2	Homo sapiens
348	hypothetical LOC100129500; apolipoprotein E	Homo sapiens
84309	nudix (nucleoside diphosphate linked moiety X)-type motif 16-like 1	Homo sapiens
10980	COP9 constitutive photomorphogenic homolog subunit 6 (Arabidopsis)	Homo sapiens
6124	ribosomal protein L4; ribosomal protein L4 pseudogene 5; ribosomal protein L4 pseudogene 4	Homo sapiens
284217	laminin, alpha 1	Homo sapiens
5689	proteasome (prosome, macropain) subunit, beta type, 1	Homo sapiens
221092	heterogeneous nuclear ribonucleoprotein U-like 2	Homo sapiens
440	asparagine synthetase	Homo sapiens
9128	PRP4 pre-mRNA processing factor 4 homolog (yeast)	Homo sapiens
10382	tubulin, beta 4	Homo sapiens
328	APEX nuclease (multifunctional DNA repair enzyme) 1	Homo sapiens
7424	vascular endothelial growth factor C	Homo sapiens
8239	ubiquitin specific peptidase 9, X-linked	Homo sapiens
51652	vacuolar protein sorting 24 homolog (S. cerevisiae); ring finger protein 103	Homo sapiens
230	aldolase C, fructose-bisphosphate	Homo sapiens
30001	ERO1-like (S. cerevisiae)	Homo sapiens
4052	latent transforming growth factor beta binding protein 1	Homo sapiens
23708	G1 to S phase transition 2	Homo sapiens
4233	met proto-oncogene (hepatocyte growth factor receptor)	Homo sapiens
55749	cell division cycle and apoptosis regulator 1	Homo sapiens
5437	polymerase (RNA) II (DNA directed) polypeptide H	Homo sapiens
483	ATPase, Na+/K+ transporting, beta 3 polypeptide	Homo sapiens
9252	ribosomal protein S6 kinase, 90kDa, polypeptide 5	Homo sapiens
8565	tyrosyl-tRNA synthetase	Homo sapiens
11034	destrin (actin depolymerizing factor)	Homo sapiens
81627	chromosome 1 open reading frame 25	Homo sapiens
1785	dynamin 2	Homo sapiens
9748	STE20-like kinase (yeast)	Homo sapiens
5111	proliferating cell nuclear antigen	Homo sapiens
79029	spermatogenesis associated 5-like 1	Homo sapiens
4221	multiple endocrine neoplasia I	Homo sapiens
51639	splicing factor 3B, 14 kDa subunit	Homo sapiens
57403	RAB22A, member RAS oncogene family	Homo sapiens
7178	similar to tumor protein, translationally-controlled 1; tumor protein, translationally-controlled 1	Homo sapiens
55697	Vac14 homolog (S. cerevisiae)	Homo sapiens
22878	KIAA1012	Homo sapiens
10056	phenylalanyl-tRNA synthetase, beta subunit	Homo sapiens
498	ATP synthase, H+ transporting, mitochondrial F1 complex, alpha subunit 1, cardiac muscle	Homo sapiens
5836	phosphorylase, glycogen, liver	Homo sapiens
4175	minichromosome maintenance complex component 6	Homo sapiens

54919	HEAT repeat containing 2	Homo sapiens
353	adenine phosphoribosyltransferase	Homo sapiens
10987	COP9 constitutive photomorphogenic homolog subunit 5 (Arabidopsis)	Homo sapiens
1488	C-terminal binding protein 2	Homo sapiens
5905	Ran GTPase activating protein 1	Homo sapiens
9652	tetratricopeptide repeat domain 37	Homo sapiens
223	aldehyde dehydrogenase 9 family, member A1	Homo sapiens
52	acid phosphatase 1, soluble	Homo sapiens
132	adenosine kinase	Homo sapiens
2934	gelsolin (amyloidosis, Finnish type)	Homo sapiens
23468	chromobox homolog 5 (HP1 alpha homolog, Drosophila)	Homo sapiens
2047	EPH receptor B1	Homo sapiens
124801	LSM12 homolog (S. cerevisiae)	Homo sapiens
4669	N-acetylglucosaminidase, alpha-	Homo sapiens
7307	U2 small nuclear RNA auxiliary factor 1	Homo sapiens
126961	histone cluster 1, H3j; histone cluster 1, H3i; histone cluster 1, H3h; histone cluster 1, H3g; histone cluster 1, H3f; histone cluster 1, H3e; histone cluster 1, H3d; histone cluster 1, H3c; histone cluster 1, H3b; histone cluster 1, H3a; histone cluster 1, H2ad; histone cluster 2, H3a; histone cluster 2, H3c; histone cluster 2, H3d	Homo sapiens
3013	histone cluster 1, H3j; histone cluster 1, H3i; histone cluster 1, H3h; histone cluster 1, H3g; histone cluster 1, H3f; histone cluster 1, H3e; histone cluster 1, H3d; histone cluster 1, H3c; histone cluster 1, H3b; histone cluster 1, H3a; histone cluster 1, H2ad; histone cluster 2, H3a; histone cluster 2, H3c; histone cluster 2, H3d	Homo sapiens
8356	histone cluster 1, H3j; histone cluster 1, H3i; histone cluster 1, H3h; histone cluster 1, H3g; histone cluster 1, H3f; histone cluster 1, H3e; histone cluster 1, H3d; histone cluster 1, H3c; histone cluster 1, H3b; histone cluster 1, H3a; histone cluster 1, H2ad; histone cluster 2, H3a; histone cluster 2, H3c; histone cluster 2, H3d	Homo sapiens
8351	histone cluster 1, H3j; histone cluster 1, H3i; histone cluster 1, H3h; histone cluster 1, H3g; histone cluster 1, H3f; histone cluster 1, H3e; histone cluster 1, H3d; histone cluster 1, H3c; histone cluster 1, H3b; histone cluster 1, H3a; histone cluster 1, H2ad; histone cluster 2, H3a; histone cluster 2, H3c; histone cluster 2, H3d	Homo sapiens
653604	histone cluster 1, H3j; histone cluster 1, H3i; histone cluster 1, H3h; histone cluster 1, H3g; histone cluster 1, H3f; histone cluster 1, H3e; histone cluster 1, H3d; histone cluster 1, H3c; histone cluster 1, H3b; histone cluster 1, H3a; histone cluster 1, H2ad; histone cluster 2, H3a; histone cluster 2, H3c; histone cluster 2, H3d	Homo sapiens
333932	histone cluster 1, H3j; histone cluster 1, H3i; histone cluster 1, H3h; histone cluster 1, H3g; histone cluster 1, H3f; histone cluster 1, H3e; histone cluster 1, H3d; histone cluster 1, H3c; histone cluster 1, H3b; histone cluster 1, H3a; histone cluster 1, H2ad; histone cluster 2, H3a; histone cluster 2, H3c; histone cluster 2, H3d	Homo sapiens
8968	histone cluster 1, H3j; histone cluster 1, H3i; histone cluster 1, H3h; histone cluster 1, H3g; histone cluster 1, H3f; histone cluster 1, H3e; histone cluster 1, H3d; histone cluster 1, H3c; histone cluster 1, H3b; histone cluster 1, H3a; histone cluster 1, H2ad; histone cluster 2, H3a; histone cluster 2, H3c; histone cluster 2, H3d	Homo sapiens
8354	histone cluster 1, H3j; histone cluster 1, H3i; histone cluster 1, H3h; histone cluster 1, H3g; histone cluster 1, H3f; histone cluster 1, H3e; histone cluster 1, H3d; histone cluster 1, H3c; histone cluster 1, H3b; histone cluster 1, H3a; histone cluster 1, H2ad; histone cluster 2, H3a; histone cluster 2, H3c; histone cluster 2, H3d	Homo sapiens
8357	histone cluster 1, H3j; histone cluster 1, H3i; histone cluster 1, H3h; histone cluster 1, H3g; histone cluster 1, H3f; histone cluster 1, H3e; histone cluster 1, H3d; histone cluster 1, H3c; histone cluster 1, H3b; histone cluster 1, H3a; histone cluster 1, H2ad; histone cluster 2, H3a; histone cluster 2, H3c; histone cluster 2, H3d	Homo sapiens
8350	histone cluster 1, H3j; histone cluster 1, H3i; histone cluster 1, H3h; histone cluster 1, H3g; histone cluster 1, H3f; histone cluster 1, H3e; histone cluster 1, H3d; histone cluster 1, H3c; histone cluster 1, H3b; histone cluster 1, H3a; histone cluster 1, H2ad; histone cluster 2, H3a; histone cluster 2, H3c; histone cluster 2, H3d	Homo sapiens
8355	histone cluster 1, H3j; histone cluster 1, H3i; histone cluster 1, H3h; histone cluster 1, H3g; histone cluster 1, H3f; histone cluster 1, H3e; histone cluster 1, H3d; histone cluster 1, H3c; histone cluster 1, H3b; histone cluster 1, H3a; histone cluster 1, H2ad; histone cluster 2, H3a; histone cluster 2, H3c; histone cluster 2, H3d	Homo sapiens
8352	histone cluster 1, H3j; histone cluster 1, H3i; histone cluster 1, H3h; histone cluster 1, H3g; histone cluster 1, H3f; histone cluster 1, H3e; histone cluster 1, H3d; histone cluster 1, H3c; histone cluster 1, H3b; histone cluster 1, H3a; histone cluster 1, H2ad; histone cluster 2, H3a; histone cluster 2, H3c; histone cluster 2, H3d	Homo sapiens
8353	histone cluster 1, H3j; histone cluster 1, H3i; histone cluster 1, H3h; histone cluster 1, H3g; histone cluster 1, H3f; histone cluster 1, H3e; histone cluster 1, H3d; histone cluster 1, H3c; histone cluster 1, H3b; histone cluster 1, H3a; histone cluster 1, H2ad; histone cluster 2, H3a; histone cluster 2, H3c; histone cluster 2, H3d	Homo sapiens
8358	histone cluster 1, H3j; histone cluster 1, H3i; histone cluster 1, H3h; histone cluster 1, H3g; histone cluster 1, H3f; histone cluster 1, H3e; histone cluster 1, H3d; histone cluster 1, H3c; histone cluster 1, H3b; histone cluster 1, H3a; histone cluster 1, H2ad; histone cluster 2, H3a; histone cluster 2, H3c; histone cluster 2, H3d	Homo sapiens
590	butyrylcholinesterase	Homo sapiens
6695	sparc/osteonectin, cwcv and kazal-like domains proteoglycan (testican) 1	Homo sapiens
998	cell division cycle 42 (GTP binding protein, 25kDa); cell division cycle 42 pseudogene 2	Homo sapiens
9782	matrin 3	Homo sapiens
3704	inosine triphosphatase (nucleoside triphosphate pyrophosphatase)	Homo sapiens
5747	PTK2 protein tyrosine kinase 2	Homo sapiens
1500	catenin (cadherin-associated protein), delta 1	Homo sapiens
526	ATPase, H+ transporting, lysosomal 56/58kDa, V1 subunit B2	Homo sapiens
6780	staufen, RNA binding protein, homolog 1 (Drosophila)	Homo sapiens
29766	tropomodulin 3 (ubiquitous)	Homo sapiens
27122	dickkopf homolog 3 (Xenopus laevis)	Homo sapiens
6923	transcription elongation factor B (SIII), polypeptide 2 (18kDa, elongin B)	Homo sapiens
403	ADP-ribosylation factor-like 3	Homo sapiens
56034	platelet derived growth factor C	Homo sapiens

10539	glutaredoxin 3	Homo sapiens
7296	thioredoxin reductase 1; hypothetical LOC100130902	Homo sapiens
57498	kinase D-interacting substrate, 220kDa	Homo sapiens
10236	heterogeneous nuclear ribonucleoprotein R	Homo sapiens
7040	transforming growth factor, beta 1	Homo sapiens
1266	calponin 3, acidic	Homo sapiens
8243	structural maintenance of chromosomes 1A	Homo sapiens
8815	similar to barrier-to-autointegration factor; barrier to autointegration factor 1	Homo sapiens
2783	guanine nucleotide binding protein (G protein), beta polypeptide 2	Homo sapiens
100130624	ribosomal protein L15 pseudogene 22; ribosomal protein L15 pseudogene 18; ribosomal protein L15 pseudogene 17; ribosomal protein L15 pseudogene 3; ribosomal protein L15 pseudogene 7; ribosomal protein L15	Homo sapiens
728576	ribosomal protein L15 pseudogene 22; ribosomal protein L15 pseudogene 18; ribosomal protein L15 pseudogene 17; ribosomal protein L15 pseudogene 3; ribosomal protein L15 pseudogene 7; ribosomal protein L15	Homo sapiens
653232	ribosomal protein L15 pseudogene 22; ribosomal protein L15 pseudogene 18; ribosomal protein L15 pseudogene 17; ribosomal protein L15 pseudogene 3; ribosomal protein L15 pseudogene 7; ribosomal protein L15	Homo sapiens
6138	ribosomal protein L15 pseudogene 22; ribosomal protein L15 pseudogene 18; ribosomal protein L15 pseudogene 17; ribosomal protein L15 pseudogene 3; ribosomal protein L15 pseudogene 7; ribosomal protein L15	Homo sapiens
728002	ribosomal protein L15 pseudogene 22; ribosomal protein L15 pseudogene 18; ribosomal protein L15 pseudogene 17; ribosomal protein L15 pseudogene 3; ribosomal protein L15 pseudogene 7; ribosomal protein L15	Homo sapiens
728088	ribosomal protein L15 pseudogene 22; ribosomal protein L15 pseudogene 18; ribosomal protein L15 pseudogene 17; ribosomal protein L15 pseudogene 3; ribosomal protein L15 pseudogene 7; ribosomal protein L15	Homo sapiens
9897	KIAA0196	Homo sapiens
2026	enolase 2 (gamma, neuronal)	Homo sapiens
2147	coagulation factor II (thrombin)	Homo sapiens
8829	neuropilin 1	Homo sapiens
8541	protein tyrosine phosphatase, receptor type, f polypeptide (PTPRF), interacting protein (liprin), alpha 3	Homo sapiens
56931	dihydrouridine synthase 3-like (S. cerevisiae)	Homo sapiens
129285	KLRAQ motif containing 1	Homo sapiens
6038	ribonuclease, RNase A family, 4	Homo sapiens
6176	ribosomal protein, large, P1	Homo sapiens
22795	nidogen 2 (osteonidogen)	Homo sapiens
6856	synaptophysin-like 1	Homo sapiens
309	annexin A6	Homo sapiens
5250	solute carrier family 25 (mitochondrial carrier; phosphate carrier), member 3	Homo sapiens
23303	kinesin family member 13B	Homo sapiens
5649	reelin	Homo sapiens
11267	SNF8, ESCRT-II complex subunit, homolog (S. cerevisiae)	Homo sapiens
64848	YTH domain containing 2	Homo sapiens
10424	progesterone receptor membrane component 2	Homo sapiens
3798	kinesin family member 5A	Homo sapiens
11336	exocyst complex component 3	Homo sapiens
7162	trophoblast glycoprotein	Homo sapiens
6125	ribosomal protein L5 pseudogene 34; ribosomal protein L5 pseudogene 1; ribosomal protein L5	Homo sapiens
84861	kelch-like 22 (Drosophila)	Homo sapiens
3301	DnaJ (Hsp40) homolog, subfamily A, member 1	Homo sapiens
23317	DnaJ (Hsp40) homolog, subfamily C, member 13	Homo sapiens
126133	aldehyde dehydrogenase 16 family, member A1	Homo sapiens
6950	hypothetical gene supported by BC000665; t-complex 1	Homo sapiens
3311	heat shock 70kDa protein 7 (HSP70B); heat shock 70kDa protein 6 (HSP70B')	Homo sapiens
3310	heat shock 70kDa protein 7 (HSP70B); heat shock 70kDa protein 6 (HSP70B')	Homo sapiens
347733	tubulin, beta 2B	Homo sapiens
55226	N-acetyltransferase 10 (GCN5-related)	Homo sapiens
51719	calcium binding protein 39	Homo sapiens
56886	UDP-glucose ceramide glucosyltransferase-like 1	Homo sapiens
127281	chromosome 1 open reading frame 93	Homo sapiens
51603	methyltransferase like 13	Homo sapiens
3945	lactate dehydrogenase B	Homo sapiens
51692	cleavage and polyadenylation specific factor 3, 73kDa	Homo sapiens
5034	prolyl 4-hydroxylase, beta polypeptide	Homo sapiens
7474	wingless-type MMTV integration site family, member 5A	Homo sapiens
7174	tripeptidyl peptidase II	Homo sapiens
3676	integrin, alpha 4 (antigen CD49D, alpha 4 subunit of VLA-4 receptor)	Homo sapiens
23384	cytospin A	Homo sapiens
7163	tumor protein D52	Homo sapiens
5500	protein phosphatase 1, catalytic subunit, beta isoform; speedy homolog A (Xenopus laevis)	Homo sapiens
79026	AHNAK nucleoprotein	Homo sapiens
80146	UDP-glucuronate decarboxylase 1	Homo sapiens
22872	SEC31 homolog A (S. cerevisiae)	Homo sapiens
7295	thioredoxin	Homo sapiens
48	aconitase 1, soluble	Homo sapiens
10808	heat shock 105kDa/110kDa protein 1	Homo sapiens
6229	ribosomal protein S24	Homo sapiens
6137	ribosomal protein L13 pseudogene 12; ribosomal protein L13	Homo sapiens
8520	histone acetyltransferase 1	Homo sapiens
10973	activating signal cointegrator 1 complex subunit 3	Homo sapiens
5858	pregnancy-zone protein	Homo sapiens
84932	RAB2B, member RAS oncogene family	Homo sapiens
5434	polymerase (RNA) II (DNA directed) polypeptide E, 25kDa	Homo sapiens

56893	ubiquilin 4	Homo sapiens
9136	ribosomal RNA processing 9, small subunit (SSU) processome component, homolog (yeast)	Homo sapiens
6513	solute carrier family 2 (facilitated glucose transporter), member 1	Homo sapiens
79830	zinc finger, MYM-type 1	Homo sapiens
4627	myosin, heavy chain 9, non-muscle	Homo sapiens
5725	polypyrimidine tract binding protein 1	Homo sapiens
55210	ATPase family, AAA domain containing 3A	Homo sapiens
303	annexin A2 pseudogene 3; annexin A2; annexin A2 pseudogene 1	Homo sapiens
302	annexin A2 pseudogene 3; annexin A2; annexin A2 pseudogene 1	Homo sapiens
26354	guanine nucleotide binding protein-like 3 (nucleolar)	Homo sapiens
5213	phosphofructokinase, muscle	Homo sapiens
156	adrenergic, beta, receptor kinase 1	Homo sapiens
4035	low density lipoprotein-related protein 1 (alpha-2-macroglobulin receptor)	Homo sapiens
9669	eukaryotic translation initiation factor 5B	Homo sapiens
29110	TANK-binding kinase 1	Homo sapiens
8732	RNA guanylyltransferase and 5'-phosphatase	Homo sapiens
5636	phosphoribosyl pyrophosphate synthetase-associated protein 2	Homo sapiens
5631	phosphoribosyl pyrophosphate synthetase 1; phosphoribosyl pyrophosphate synthetase 1-like 1	Homo sapiens
221823	phosphoribosyl pyrophosphate synthetase 1; phosphoribosyl pyrophosphate synthetase 1-like 1	Homo sapiens
3250	haptoglobin-related protein; haptoglobin	Homo sapiens
3240	haptoglobin-related protein; haptoglobin	Homo sapiens
80124	valosin containing protein (p97)/p47 complex interacting protein 1	Homo sapiens
3728	junction plakoglobin	Homo sapiens
128239	IQ motif containing GTPase activating protein 3	Homo sapiens
3673	integrin, alpha 2 (CD49B, alpha 2 subunit of VLA-2 receptor)	Homo sapiens
80210	armadillo repeat containing 9	Homo sapiens
6652	sorbitol dehydrogenase	Homo sapiens
22934	ribose 5-phosphate isomerase A	Homo sapiens
2762	GDP-mannose 4,6-dehydratase	Homo sapiens
28952	coiled-coil domain containing 22	Homo sapiens
5716	proteasome (prosome, macropain) 26S subunit, non-ATPase, 10	Homo sapiens
140465	myosin, light chain 6B, alkali, smooth muscle and non-muscle	Homo sapiens
378	ADP-ribosylation factor 4	Homo sapiens
83700	junctional adhesion molecule 3	Homo sapiens
84498	family with sequence similarity 120B	Homo sapiens
4670	heterogeneous nuclear ribonucleoprotein M	Homo sapiens
1665	DEAH (Asp-Glu-Ala-His) box polypeptide 15	Homo sapiens
29128	ubiquitin-like with PHD and ring finger domains 1	Homo sapiens
8065	cullin 5	Homo sapiens
8452	cullin 3	Homo sapiens
476	ATPase, Na+/K+ transporting, alpha 1 polypeptide	Homo sapiens
1656	DEAD (Asp-Glu-Ala-Asp) box polypeptide 6	Homo sapiens
2023	enolase 1, (alpha)	Homo sapiens
64359	nucleoredoxin	Homo sapiens
3321	immunoglobulin superfamily, member 3	Homo sapiens
3191	similar to heterogeneous nuclear ribonucleoprotein L-like; heterogeneous nuclear ribonucleoprotein L	Homo sapiens
5129	PCTAIRE protein kinase 3	Homo sapiens
93973	ARP8 actin-related protein 8 homolog (yeast)	Homo sapiens
7414	vinculin	Homo sapiens
9276	coatomer protein complex, subunit beta 2 (beta prime)	Homo sapiens
11344	twinfilin, actin-binding protein, homolog 2 (Drosophila)	Homo sapiens
10574	chaperonin containing TCP1, subunit 7 (eta)	Homo sapiens
8836	gamma-glutamyl hydrolase (conjugase, folylpolygammaglutamyl hydrolase)	Homo sapiens
306	annexin A3	Homo sapiens
283742	family with sequence similarity 98, member B	Homo sapiens
5898	v-ral simian leukemia viral oncogene homolog A (ras related)	Homo sapiens
1967	eukaryotic translation initiation factor 2B, subunit 1 alpha, 26kDa	Homo sapiens
5471	phosphoribosyl pyrophosphate amidotransferase	Homo sapiens
9477	mediator complex subunit 20	Homo sapiens
1605	dystroglycan 1 (dystrophin-associated glycoprotein 1)	Homo sapiens
8697	cell division cycle 23 homolog (S. cerevisiae)	Homo sapiens
83743	glutamate-rich WD repeat containing 1	Homo sapiens
1471	cystatin C	Homo sapiens
5648	mannan-binding lectin serine peptidase 1 (C4/C2 activating component of Ra-reactive factor)	Homo sapiens
7314	ubiquitin B	Homo sapiens
65263	pyrroline-5-carboxylate reductase-like	Homo sapiens
87	actinin, alpha 1	Homo sapiens
5711	proteasome (prosome, macropain) 26S subunit, non-ATPase, 5	Homo sapiens
55379	leucine rich repeat containing 59	Homo sapiens
5577	protein kinase, cAMP-dependent, regulatory, type II, beta	Homo sapiens
908	chaperonin containing TCP1, subunit 6A (zeta 1)	Homo sapiens
11060	WW domain containing E3 ubiquitin protein ligase 2	Homo sapiens
22870	SAPS domain family, member 1	Homo sapiens
8078	ubiquitin specific peptidase 5 (isopeptidase T)	Homo sapiens
9847	KIAA0528	Homo sapiens
54862	coiled-coil and C2 domain containing 1A	Homo sapiens
55823	vacuolar protein sorting 11 homolog (S. cerevisiae)	Homo sapiens
114793	formin-like 2	Homo sapiens
63892	thyroid adenoma associated	Homo sapiens
9733	squamous cell carcinoma antigen recognized by T cells 3	Homo sapiens
157769	family with sequence similarity 91, member A1	Homo sapiens
11059	WW domain containing E3 ubiquitin protein ligase 1	Homo sapiens
51479	ankyrin repeat and FYVE domain containing 1	Homo sapiens
4673	nucleosome assembly protein 1-like 1	Homo sapiens
8406	sushi-repeat-containing protein, X-linked	Homo sapiens

25978	chromatin modifying protein 2B	Homo sapiens
8573	calcium/calmodulin-dependent serine protein kinase (MAGUK family)	Homo sapiens
1284	collagen, type IV, alpha 2	Homo sapiens
9380	glyoxylate reductase/hydroxypyruvate reductase	Homo sapiens
7184	heat shock protein 90kDa beta (Grp94), member 1	Homo sapiens
100133211	related RAS viral (r-ras) oncogene homolog 2; similar to related RAS viral (r-ras) oncogene homolog 2	Homo sapiens
22800	related RAS viral (r-ras) oncogene homolog 2; similar to related RAS viral (r-ras) oncogene homolog 2	Homo sapiens
5912	RAP2B, member of RAS oncogene family	Homo sapiens
1719	dihydrofolate reductase	Homo sapiens
26135	SERPINE1 mRNA binding protein 1	Homo sapiens
25998	inhibitor of Bruton agammaglobulinemia tyrosine kinase	Homo sapiens
642	bleomycin hydrolase	Homo sapiens
8724	sorting nexin 3	Homo sapiens
23218	neurobeachin-like 2	Homo sapiens
57488	family with sequence similarity 62 (C2 domain containing), member B	Homo sapiens
4809	NHP2 non-histone chromosome protein 2-like 1 (S. cerevisiae)	Homo sapiens
27293	sphingomyelin phosphodiesterase, acid-like 3B	Homo sapiens
5336	phospholipase C, gamma 2 (phosphatidylinositol-specific)	Homo sapiens
5476	cathepsin A	Homo sapiens
1939	ligatin	Homo sapiens
3276	protein arginine methyltransferase 1	Homo sapiens
29968	chromosome 8 open reading frame 62; phosphoserine aminotransferase 1	Homo sapiens
2801	golgi autoantigen, golgin subfamily a, 2	Homo sapiens
9097	ubiquitin specific peptidase 14 (tRNA-guanine transglycosylase)	Homo sapiens
10184	lipoma HMGIC fusion partner-like 2	Homo sapiens
25930	protein tyrosine phosphatase, non-receptor type 23	Homo sapiens
51571	family with sequence similarity 49, member B	Homo sapiens
65264	ubiquitin-conjugating enzyme E2Z	Homo sapiens
471	5-aminoimidazole-4-carboxamide ribonucleotide formyltransferase/IMP cyclohydrolase	Homo sapiens
4905	N-ethylmaleimide-sensitive factor	Homo sapiens
5298	phosphatidylinositol 4-kinase, catalytic, beta	Homo sapiens
9646	Ctr9, Paf1/RNA polymerase II complex component, homolog (S. cerevisiae)	Homo sapiens
6194	ribosomal protein S6 pseudogene 25; ribosomal protein S6; ribosomal protein S6 pseudogene 1	Homo sapiens
8546	adaptor-related protein complex 3, beta 1 subunit	Homo sapiens
22820	coatomer protein complex, subunit gamma	Homo sapiens
6714	v-src sarcoma (Schmidt-Ruppin A-2) viral oncogene homolog (avian)	Homo sapiens
4218	RAB8A, member RAS oncogene family	Homo sapiens
10465	peptidylprolyl isomerase H (cyclophilin H)	Homo sapiens
25801	grancalcin, EF-hand calcium binding protein	Homo sapiens
10935	peroxiredoxin 3	Homo sapiens
27243	chromatin modifying protein 2A	Homo sapiens
9933	KIAA0020	Homo sapiens
23266	latrophilin 2	Homo sapiens
10592	structural maintenance of chromosomes 2	Homo sapiens
1314	coatomer protein complex, subunit alpha	Homo sapiens
2107	eukaryotic translation termination factor 1	Homo sapiens
4191	malate dehydrogenase 2, NAD (mitochondrial)	Homo sapiens
6251	Ras suppressor protein 1	Homo sapiens
55862	enoyl Coenzyme A hydratase domain containing 1	Homo sapiens
3614	IMP (inosine monophosphate) dehydrogenase 1	Homo sapiens
51138	COP9 constitutive photomorphogenic homolog subunit 4 (Arabidopsis)	Homo sapiens
8453	cullin 2	Homo sapiens
80895	integrin-linked kinase-associated serine/threonine phosphatase 2C	Homo sapiens
6829	suppressor of Ty 5 homolog (S. cerevisiae)	Homo sapiens
1947	ephrin-B1	Homo sapiens
25921	zinc finger, DHHC-type containing 5	Homo sapiens
10785	WD repeat domain 4	Homo sapiens
5303	protein (peptidylprolyl cis/trans isomerase) NIMA-interacting, 4 (parvulin)	Homo sapiens
9958	ubiquitin specific peptidase 15	Homo sapiens
6432	splicing factor, arginine/serine-rich 7, 35kDa	Homo sapiens
6188	ribosomal protein S3 pseudogene 3; ribosomal protein S3	Homo sapiens
440991	ribosomal protein S3 pseudogene 3; ribosomal protein S3	Homo sapiens
30844	EH-domain containing 4	Homo sapiens
6598	SWI/SNF related, matrix associated, actin dependent regulator of chromatin, subfamily b, member 1	Homo sapiens
7082	tight junction protein 1 (zona occludens 1)	Homo sapiens
2870	G protein-coupled receptor kinase 6	Homo sapiens
26173	integrator complex subunit 1	Homo sapiens
11098	protease, serine, 23	Homo sapiens
23020	similar to U5 snRNP-specific protein, 200 kDa; small nuclear ribonucleoprotein 200kDa (U5)	Homo sapiens
79837	phosphatidylinositol-5-phosphate 4-kinase, type II, gamma	Homo sapiens
51377	ubiquitin carboxyl-terminal hydrolase L5	Homo sapiens
85369	family with sequence similarity 40, member A	Homo sapiens
6732	SFRS protein kinase 1	Homo sapiens
55236	ubiquitin-like modifier activating enzyme 6	Homo sapiens
4363	ATP-binding cassette, sub-family C (CFTR/MRP), member 1	Homo sapiens
3949	low density lipoprotein receptor	Homo sapiens
83548	component of oligomeric golgi complex 3	Homo sapiens
5329	plasminogen activator, urokinase receptor	Homo sapiens
335	apolipoprotein A-I	Homo sapiens
3157	3-hydroxy-3-methylglutaryl-Coenzyme A synthase 1 (soluble)	Homo sapiens
7087	intercellular adhesion molecule 5, telencephalin	Homo sapiens
8301	phosphatidylinositol binding clathrin assembly protein	Homo sapiens
1303	collagen, type XII, alpha 1	Homo sapiens

214	hypothetical protein LOC100133690; activated leukocyte cell adhesion molecule	Homo sapiens
8086	achalasia, adrenocortical insufficiency, alacrimia (Allgrove, triple-A)	Homo sapiens
9410	small nuclear ribonucleoprotein 40kDa (U5)	Homo sapiens
56850	GRIP1 associated protein 1	Homo sapiens
159	adenylosuccinate synthase	Homo sapiens
51143	dynein, cytoplasmic 1, light intermediate chain 1	Homo sapiens
55604	leucine rich repeat containing 16A	Homo sapiens
55605	kinesin family member 21A	Homo sapiens
7035	tissue factor pathway inhibitor (lipoprotein-associated coagulation inhibitor)	Homo sapiens
1277	collagen, type I, alpha 1	Homo sapiens
84888	signal peptide peptidase-like 2A	Homo sapiens
3939	lactate dehydrogenase A	Homo sapiens
2135	exostoses (multiple)-like 2	Homo sapiens
1956	epidermal growth factor receptor (erythroblastic leukemia viral (v-erb-b) oncogene homolog, avian)	Homo sapiens
2521	fusion (involved in t(12;16) in malignant liposarcoma)	Homo sapiens
10120	ARP1 actin-related protein 1 homolog B, centractin beta (yeast)	Homo sapiens
6472	serine hydroxymethyltransferase 2 (mitochondrial)	Homo sapiens
9209	leucine rich repeat (in FLII) interacting protein 2	Homo sapiens
444	aspartate beta-hydroxylase	Homo sapiens
3315	heat shock 27kDa protein-like 2 pseudogene; heat shock 27kDa protein 1	Homo sapiens
653553	heat shock 27kDa protein-like 2 pseudogene; heat shock 27kDa protein 1	Homo sapiens
10380	3'(2'), 5'-bisphosphate nucleotidase 1	Homo sapiens
64423	inverted formin, FH2 and WH2 domain containing	Homo sapiens
6156	ribosomal protein L30	Homo sapiens
3480	insulin-like growth factor 1 receptor	Homo sapiens
6227	ribosomal protein S21	Homo sapiens
55752	septin 11	Homo sapiens
2739	glyoxalase I	Homo sapiens
3842	transportin 1	Homo sapiens
55937	apolipoprotein M	Homo sapiens
4282	macrophage migration inhibitory factor (glycosylation-inhibiting factor)	Homo sapiens
6430	splicing factor, arginine/serine-rich 5	Homo sapiens
4090	SMAD family member 5	Homo sapiens
2782	guanine nucleotide binding protein (G protein), beta polypeptide 1	Homo sapiens
57826	RAP2C, member of RAS oncogene family	Homo sapiens
30849	phosphoinositide-3-kinase, regulatory subunit 4	Homo sapiens
5984	replication factor C (activator 1) 4, 37kDa	Homo sapiens
64855	family with sequence similarity 129, member B	Homo sapiens
256364	echinoderm microtubule associated protein like 3	Homo sapiens
5589	protein kinase C substrate 80K-H	Homo sapiens
29894	cleavage and polyadenylation specific factor 1, 160kDa	Homo sapiens
3070	helicase, lymphoid-specific	Homo sapiens
23385	nicastrin	Homo sapiens
26263	FBXO22 opposite strand (non-protein coding); F-box protein 22	Homo sapiens
274	bridging integrator 1	Homo sapiens
6731	signal recognition particle 72kDa	Homo sapiens
9373	phospholipase A2-activating protein	Homo sapiens
9991	ROD1 regulator of differentiation 1 (S. pombe)	Homo sapiens
28989	methyltransferase like 11A	Homo sapiens
8893	eukaryotic translation initiation factor 2B, subunit 5 epsilon, 82kDa	Homo sapiens
823	calpain 1, (mu/I) large subunit	Homo sapiens
8087	fragile X mental retardation, autosomal homolog 1	Homo sapiens
23358	ubiquitin specific peptidase 24	Homo sapiens
400389	ribosomal protein L12 pseudogene 2; ribosomal protein L12 pseudogene 32; ribosomal protein L12 pseudogene 35; ribosomal protein L12 pseudogene 19; ribosomal protein L12 pseudogene 6; ribosomal protein L12; ribosomal protein L12 pseudogene 14	Homo sapiens
440176	ribosomal protein L12 pseudogene 2; ribosomal protein L12 pseudogene 32; ribosomal protein L12 pseudogene 35; ribosomal protein L12 pseudogene 19; ribosomal protein L12 pseudogene 6; ribosomal protein L12; ribosomal protein L12 pseudogene 14	Homo sapiens
6136	ribosomal protein L12 pseudogene 2; ribosomal protein L12 pseudogene 32; ribosomal protein L12 pseudogene 35; ribosomal protein L12 pseudogene 19; ribosomal protein L12 pseudogene 6; ribosomal protein L12; ribosomal protein L12 pseudogene 14	Homo sapiens
646875	ribosomal protein L12 pseudogene 2; ribosomal protein L12 pseudogene 32; ribosomal protein L12 pseudogene 35; ribosomal protein L12 pseudogene 19; ribosomal protein L12 pseudogene 6; ribosomal protein L12; ribosomal protein L12 pseudogene 14	Homo sapiens
100129982	ribosomal protein L12 pseudogene 2; ribosomal protein L12 pseudogene 32; ribosomal protein L12 pseudogene 35; ribosomal protein L12 pseudogene 19; ribosomal protein L12 pseudogene 6; ribosomal protein L12; ribosomal protein L12 pseudogene 14	Homo sapiens
729500	ribosomal protein L12 pseudogene 2; ribosomal protein L12 pseudogene 32; ribosomal protein L12 pseudogene 35; ribosomal protein L12 pseudogene 19; ribosomal protein L12 pseudogene 6; ribosomal protein L12; ribosomal protein L12 pseudogene 14	Homo sapiens
100132795	ribosomal protein L12 pseudogene 2; ribosomal protein L12 pseudogene 32; ribosomal protein L12 pseudogene 35; ribosomal protein L12 pseudogene 19; ribosomal protein L12 pseudogene 6; ribosomal protein L12; ribosomal protein L12 pseudogene 14	Homo sapiens
10591	chromosome 6 open reading frame 108	Homo sapiens
2956	mutS homolog 6 (E. coli)	Homo sapiens
55915	LanC lantibiotic synthetase component C-like 2 (bacterial)	Homo sapiens
9145	synaptogyrin 1	Homo sapiens
9183	ZW10, kinetochore associated, homolog (Drosophila)	Homo sapiens
10493	vesicle amine transport protein 1 homolog (T. californica)	Homo sapiens
8775	N-ethylmaleimide-sensitive factor attachment protein, alpha	Homo sapiens
4437	mutS homolog 3 (E. coli)	Homo sapiens
10128	leucine-rich PPR-motif containing	Homo sapiens
1312	catechol-O-methyltransferase	Homo sapiens

6428	splicing factor, arginine/serine-rich 3	Homo sapiens
7532	tyrosine 3-monooxygenase/tryptophan 5-monooxygenase activation protein, gamma polypeptide	Homo sapiens
7534	tyrosine 3-monooxygenase/tryptophan 5-monooxygenase activation protein, zeta polypeptide	Homo sapiens
6206	ribosomal protein S12; ribosomal protein S12 pseudogene 4; ribosomal protein S12 pseudogene 11; ribosomal protein S12 pseudogene 9	Homo sapiens
10617	STAM binding protein	Homo sapiens
7203	chaperonin containing TCP1, subunit 3 (gamma)	Homo sapiens
29789	Obg-like ATPase 1	Homo sapiens
9984	THO complex 1	Homo sapiens
50814	NAD(P) dependent steroid dehydrogenase-like	Homo sapiens
1808	dihydropyrimidinase-like 2	Homo sapiens
3189	heterogeneous nuclear ribonucleoprotein H3 (2H9)	Homo sapiens
10654	phosphomevalonate kinase	Homo sapiens
54832	vacuolar protein sorting 13 homolog C (S. cerevisiae)	Homo sapiens
6396	SEC13 homolog (S. cerevisiae)	Homo sapiens
10082	glypican 6	Homo sapiens
10492	synaptotagmin binding, cytoplasmic RNA interacting protein	Homo sapiens
5066	peptidylglycine alpha-amidating monooxygenase	Homo sapiens
10171	RNA terminal phosphate cyclase-like 1	Homo sapiens
1965	eukaryotic translation initiation factor 2, subunit 1 alpha, 35kDa	Homo sapiens
55048	vacuolar protein sorting 37 homolog C (S. cerevisiae)	Homo sapiens
2776	guanine nucleotide binding protein (G protein), q polypeptide	Homo sapiens
54520	coiled-coil domain containing 93	Homo sapiens
3927	LIM and SH3 protein 1	Homo sapiens
65985	acetoacetyl-CoA synthetase	Homo sapiens
79585	coronin 7	Homo sapiens
55890	G protein-coupled receptor, family C, group 5, member C	Homo sapiens
10381	tubulin, beta 3; melanocortin 1 receptor (alpha melanocyte stimulating hormone receptor)	Homo sapiens
4157	tubulin, beta 3; melanocortin 1 receptor (alpha melanocyte stimulating hormone receptor)	Homo sapiens
10342	TRK-fused gene	Homo sapiens
5257	phosphorylase kinase, beta	Homo sapiens
57085	angiotensin II receptor-associated protein	Homo sapiens
5695	proteasome (prosome, macropain) subunit, beta type, 7	Homo sapiens
83692	CD99 molecule-like 2	Homo sapiens
161	adaptor-related protein complex 2, alpha 2 subunit	Homo sapiens
5604	mitogen-activated protein kinase kinase 1	Homo sapiens
23191	cytoplasmic FMR1 interacting protein 1	Homo sapiens
1512	cathepsin H	Homo sapiens
164	adaptor-related protein complex 1, gamma 1 subunit	Homo sapiens
29882	anaphase promoting complex subunit 2	Homo sapiens
6738	TROVE domain family, member 2	Homo sapiens
4059	basal cell adhesion molecule (Lutheran blood group)	Homo sapiens
644	biliverdin reductase A	Homo sapiens
10427	SEC24 family, member B (S. cerevisiae)	Homo sapiens
10940	processing of precursor 1, ribonuclease P/MRP subunit (S. cerevisiae)	Homo sapiens
5209	6-phosphofructo-2-kinase/fructose-2,6-biphosphatase 3	Homo sapiens
4357	mercaptopyruvate sulfurtransferase	Homo sapiens
8335	histone cluster 1, H2ae; histone cluster 1, H2ab	Homo sapiens
3012	histone cluster 1, H2ae; histone cluster 1, H2ab	Homo sapiens
23327	neural precursor cell expressed, developmentally down-regulated 4-like	Homo sapiens
6152	ribosomal protein L24; ribosomal protein L24 pseudogene 6	Homo sapiens
4642	myosin ID	Homo sapiens
79707	nucleolar protein 9	Homo sapiens
22919	microtubule-associated protein, RP/EB family, member 1	Homo sapiens
23555	tetraspanin 15	Homo sapiens
94081	sideroflexin 1	Homo sapiens
27316	similar to RNA binding motif protein, X-linked; similar to hCG2011544; RNA binding motif protein, X-linked	Homo sapiens
55748	CNDP dipeptidase 2 (metallopeptidase M20 family)	Homo sapiens
9555	H2A histone family, member Y	Homo sapiens
26060	adaptor protein, phosphotyrosine interaction, PH domain and leucine zipper containing 1	Homo sapiens
10643	insulin-like growth factor 2 mRNA binding protein 3	Homo sapiens
427	N-acylsphingosine amidohydrolase (acid ceramidase) 1	Homo sapiens
26278	spastic ataxia of Charlevoix-Saguenay (sacsin)	Homo sapiens
5537	protein phosphatase 6, catalytic subunit	Homo sapiens
93343	family with sequence similarity 125, member A	Homo sapiens
10735	stromal antigen 2	Homo sapiens
1515	cathepsin L2	Homo sapiens
2108	electron-transfer-flavoprotein, alpha polypeptide	Homo sapiens
23339	vacuolar protein sorting 39 homolog (S. cerevisiae)	Homo sapiens
5819	poliovirus receptor-related 2 (herpesvirus entry mediator B)	Homo sapiens
5530	protein phosphatase 3 (formerly 2B), catalytic subunit, alpha isoform	Homo sapiens
3672	integrin, alpha 1	Homo sapiens
10458	BAI1-associated protein 2	Homo sapiens
3187	heterogeneous nuclear ribonucleoprotein H1 (H)	Homo sapiens
64223	MTOR associated protein, LST8 homolog (S. cerevisiae)	Homo sapiens
6603	SWI/SNF related, matrix associated, actin dependent regulator of chromatin, subfamily d, member 2	Homo sapiens
1977	eukaryotic translation initiation factor 4E; similar to hCG1777996; similar to eukaryotic translation initiation factor 4E	Homo sapiens
2194	fatty acid synthase	Homo sapiens
989	septin 7	Homo sapiens
6812	syntaxin binding protein 1	Homo sapiens
8754	ADAM metallopeptidase domain 9 (meltrin gamma)	Homo sapiens
6729	similar to signal recognition particle 54kDa; signal recognition particle 54kDa	Homo sapiens
26958	coatomer protein complex, subunit gamma 2	Homo sapiens

7155	topoisomerase (DNA) II beta 180kDa	Homo sapiens
7076	TIMP metallopeptidase inhibitor 1	Homo sapiens
27229	tubulin, gamma complex associated protein 4	Homo sapiens
1213	clathrin, heavy chain (Hc)	Homo sapiens
10521	DEAD (Asp-Glu-Ala-Asp) box polypeptide 17	Homo sapiens
4038	low density lipoprotein receptor-related protein 4	Homo sapiens
388524	ribosomal protein SA pseudogene 9; ribosomal protein SA pseudogene 8; ribosomal protein SA pseudogene 58; ribosomal protein SA pseudogene 19; ribosomal protein SA pseudogene 18; ribosomal protein SA; ribosomal protein SA pseudogene 15; ribosomal protein SA pseudogene 61; ribosomal protein SA pseudogene 29; ribosomal protein SA pseudogene 12	Homo sapiens
389141	ribosomal protein SA pseudogene 9; ribosomal protein SA pseudogene 8; ribosomal protein SA pseudogene 58; ribosomal protein SA pseudogene 19; ribosomal protein SA pseudogene 18; ribosomal protein SA; ribosomal protein SA pseudogene 15; ribosomal protein SA pseudogene 61; ribosomal protein SA pseudogene 29; ribosomal protein SA pseudogene 12	Homo sapiens
3921	ribosomal protein SA pseudogene 9; ribosomal protein SA pseudogene 8; ribosomal protein SA pseudogene 58; ribosomal protein SA pseudogene 19; ribosomal protein SA pseudogene 18; ribosomal protein SA; ribosomal protein SA pseudogene 15; ribosomal protein SA pseudogene 61; ribosomal protein SA pseudogene 29; ribosomal protein SA pseudogene 12	Homo sapiens
387867	ribosomal protein SA pseudogene 9; ribosomal protein SA pseudogene 8; ribosomal protein SA pseudogene 58; ribosomal protein SA pseudogene 19; ribosomal protein SA pseudogene 18; ribosomal protein SA; ribosomal protein SA pseudogene 15; ribosomal protein SA pseudogene 61; ribosomal protein SA pseudogene 29; ribosomal protein SA pseudogene 12	Homo sapiens
643617	ribosomal protein SA pseudogene 9; ribosomal protein SA pseudogene 8; ribosomal protein SA pseudogene 58; ribosomal protein SA pseudogene 19; ribosomal protein SA pseudogene 18; ribosomal protein SA; ribosomal protein SA pseudogene 15; ribosomal protein SA pseudogene 61; ribosomal protein SA pseudogene 29; ribosomal protein SA pseudogene 12	Homo sapiens
388707	ribosomal protein SA pseudogene 9; ribosomal protein SA pseudogene 8; ribosomal protein SA pseudogene 58; ribosomal protein SA pseudogene 19; ribosomal protein SA pseudogene 18; ribosomal protein SA; ribosomal protein SA pseudogene 15; ribosomal protein SA pseudogene 61; ribosomal protein SA pseudogene 29; ribosomal protein SA pseudogene 12	Homo sapiens
653162	ribosomal protein SA pseudogene 9; ribosomal protein SA pseudogene 8; ribosomal protein SA pseudogene 58; ribosomal protein SA pseudogene 19; ribosomal protein SA pseudogene 18; ribosomal protein SA; ribosomal protein SA pseudogene 15; ribosomal protein SA pseudogene 61; ribosomal protein SA pseudogene 29; ribosomal protein SA pseudogene 12	Homo sapiens
730029	ribosomal protein SA pseudogene 9; ribosomal protein SA pseudogene 8; ribosomal protein SA pseudogene 58; ribosomal protein SA pseudogene 19; ribosomal protein SA pseudogene 18; ribosomal protein SA; ribosomal protein SA pseudogene 15; ribosomal protein SA pseudogene 61; ribosomal protein SA pseudogene 29; ribosomal protein SA pseudogene 12	Homo sapiens
220885	ribosomal protein SA pseudogene 9; ribosomal protein SA pseudogene 8; ribosomal protein SA pseudogene 58; ribosomal protein SA pseudogene 19; ribosomal protein SA pseudogene 18; ribosomal protein SA; ribosomal protein SA pseudogene 15; ribosomal protein SA pseudogene 61; ribosomal protein SA pseudogene 29; ribosomal protein SA pseudogene 12	Homo sapiens
644464	ribosomal protein SA pseudogene 9; ribosomal protein SA pseudogene 8; ribosomal protein SA pseudogene 58; ribosomal protein SA pseudogene 19; ribosomal protein SA pseudogene 18; ribosomal protein SA; ribosomal protein SA pseudogene 15; ribosomal protein SA pseudogene 61; ribosomal protein SA pseudogene 29; ribosomal protein SA pseudogene 12	Homo sapiens
8891	eukaryotic translation initiation factor 2B, subunit 3 gamma, 58kDa	Homo sapiens
51667	negative regulator of ubiquitin-like proteins 1	Homo sapiens
5562	protein kinase, AMP-activated, alpha 1 catalytic subunit	Homo sapiens
54504	carboxypeptidase, vitellogenic-like	Homo sapiens
220717	ribosomal protein, large, P0 pseudogene 2; ribosomal protein, large, P0 pseudogene 3; ribosomal protein, large, P0 pseudogene 6; ribosomal protein, large, P0	Homo sapiens
113157	ribosomal protein, large, P0 pseudogene 2; ribosomal protein, large, P0 pseudogene 3; ribosomal protein, large, P0 pseudogene 6; ribosomal protein, large, P0	Homo sapiens
6175	ribosomal protein, large, P0 pseudogene 2; ribosomal protein, large, P0 pseudogene 3; ribosomal protein, large, P0 pseudogene 6; ribosomal protein, large, P0	Homo sapiens
122589	ribosomal protein, large, P0 pseudogene 2; ribosomal protein, large, P0 pseudogene 3; ribosomal protein, large, P0 pseudogene 6; ribosomal protein, large, P0	Homo sapiens
1755	deleted in malignant brain tumors 1	Homo sapiens
4893	neuroblastoma RAS viral (v-ras) oncogene homolog	Homo sapiens
5709	proteasome (prosome, macropain) 26S subunit, non-ATPase, 3	Homo sapiens
5550	prolyl endopeptidase	Homo sapiens
1933	eukaryotic translation elongation factor 1 beta 2; eukaryotic translation elongation factor 1 beta 2-like	Homo sapiens
10540	dynactin 2 (p50)	Homo sapiens
255743	nephronectin	Homo sapiens
1431	citrate synthase	Homo sapiens
3831	kinesin light chain 1	Homo sapiens
5686	proteasome (prosome, macropain) subunit, alpha type, 5	Homo sapiens
1174	adaptor-related protein complex 1, sigma 1 subunit	Homo sapiens
3630	insulin-like growth factor 2 (somatomedin A); insulin; INS-IGF2 readthrough transcript	Homo sapiens
3481	insulin-like growth factor 2 (somatomedin A); insulin; INS-IGF2 readthrough transcript	Homo sapiens
8976	Wiskott-Aldrich syndrome-like	Homo sapiens
55243	kin of IRRE like (Drosophila)	Homo sapiens
3313	heat shock 70kDa protein 9 (mortalin)	Homo sapiens
2665	GDP dissociation inhibitor 2	Homo sapiens
2923	protein disulfide isomerase family A, member 3	Homo sapiens
1291	collagen, type VI, alpha 1	Homo sapiens
284996	ring finger protein 149	Homo sapiens
11047	adhesion regulating molecule 1	Homo sapiens
8683	splicing factor, arginine/serine-rich 9	Homo sapiens
10286	breast carcinoma amplified sequence 2	Homo sapiens
23279	nucleoporin 160kDa	Homo sapiens
79980	DSN1, MIND kinetochore complex component, homolog (S. cerevisiae)	Homo sapiens
23524	serine/arginine repetitive matrix 2; hypothetical LOC100132779	Homo sapiens

5128	PCTAIRE protein kinase 2	Homo sapiens
57617	vacuolar protein sorting 18 homolog (S. cerevisiae)	Homo sapiens
7430	hypothetical protein LOC100129652; ezrin	Homo sapiens
5885	RAD21 homolog (S. pombe)	Homo sapiens
6711	spectrin, beta, non-erythrocytic 1	Homo sapiens
51474	LIM domain and actin binding 1	Homo sapiens
4836	N-myristoyltransferase 1	Homo sapiens
2909	glucocorticoid receptor DNA binding factor 1	Homo sapiens
55705	importin 9	Homo sapiens
9545	RAB3D, member RAS oncogene family	Homo sapiens
11258	dynactin 3 (p22)	Homo sapiens
8761	poly(A) binding protein, cytoplasmic 4 (inducible form)	Homo sapiens
55714	odz, odd Oz/ten-m homolog 3 (Drosophila)	Homo sapiens
4758	sialidase 1 (lysosomal sialidase)	Homo sapiens
1736	dyskeratosis congenita 1, dyskerin	Homo sapiens
54521	WD repeat domain 44	Homo sapiens
80335	WD repeat domain 82	Homo sapiens
7402	utrophin	Homo sapiens
9784	sorting nexin 17	Homo sapiens
5481	peptidylprolyl isomerase D	Homo sapiens
3910	laminin, alpha 4	Homo sapiens
23673	syntaxin 12	Homo sapiens
55666	nuclear protein localization 4 homolog (S. cerevisiae)	Homo sapiens
3376	isoleucyl-tRNA synthetase	Homo sapiens
6141	ribosomal protein L18	Homo sapiens
6421	splicing factor proline/glutamine-rich (polypyrimidine tract binding protein associated)	Homo sapiens
11113	citron (rho-interacting, serine/threonine kinase 21)	Homo sapiens
2885	growth factor receptor-bound protein 2	Homo sapiens
191	adenosylhomocysteinase	Homo sapiens
51755	Cdc2-related kinase, arginine/serine-rich	Homo sapiens
1660	DEAH (Asp-Glu-Ala-His) box polypeptide 9	Homo sapiens
10549	peroxiredoxin 4	Homo sapiens
4771	neurofibromin 2 (merlin)	Homo sapiens
1404	hyaluronan and proteoglycan link protein 1	Homo sapiens
207	v-akt murine thymoma viral oncogene homolog 1	Homo sapiens
57418	WD repeat domain 18	Homo sapiens
10678	UDP-GlcNAc:betaGal beta-1,3-N-acetylglucosaminyltransferase 1; UDP-GlcNAc:betaGal beta-1,3-N-acetylglucosaminyltransferase 2	Homo sapiens
26985	adaptor-related protein complex 3, mu 1 subunit	Homo sapiens
22930	RAB3 GTPase activating protein subunit 1 (catalytic)	Homo sapiens
1462	versican	Homo sapiens
23654	plexin B2	Homo sapiens
6191	ribosomal protein S4X pseudogene 6; ribosomal protein S4X pseudogene 13; ribosomal protein S4, X-linked	Homo sapiens
390183	ribosomal protein S4X pseudogene 6; ribosomal protein S4X pseudogene 13; ribosomal protein S4, X-linked	Homo sapiens
391777	ribosomal protein S4X pseudogene 6; ribosomal protein S4X pseudogene 13; ribosomal protein S4, X-linked	Homo sapiens
3146	high-mobility group box 1; high-mobility group box 1-like 10	Homo sapiens
4154	muscleblind-like (Drosophila)	Homo sapiens
10482	nuclear RNA export factor 1	Homo sapiens
81873	actin related protein 2/3 complex, subunit 5-like	Homo sapiens
400750	hypothetical gene supported by AF216292; NM_005347; heat shock 70kDa protein 5 (glucose-regulated protein, 78kDa)	Homo sapiens
3309	hypothetical gene supported by AF216292; NM_005347; heat shock 70kDa protein 5 (glucose-regulated protein, 78kDa)	Homo sapiens
23481	pescadillo homolog 1, containing BRCT domain (zebrafish)	Homo sapiens
1434	CSE1 chromosome segregation 1-like (yeast)	Homo sapiens
871	serpin peptidase inhibitor, clade H (heat shock protein 47), member 1, (collagen binding protein 1)	Homo sapiens
805	calmodulin 3 (phosphorylase kinase, delta); calmodulin 2 (phosphorylase kinase, delta); calmodulin 1 (phosphorylase kinase, delta)	Homo sapiens
808	calmodulin 3 (phosphorylase kinase, delta); calmodulin 2 (phosphorylase kinase, delta); calmodulin 1 (phosphorylase kinase, delta)	Homo sapiens
801	calmodulin 3 (phosphorylase kinase, delta); calmodulin 2 (phosphorylase kinase, delta); calmodulin 1 (phosphorylase kinase, delta)	Homo sapiens
728689	eukaryotic translation initiation factor 3, subunit C-like	Homo sapiens
55308	DEAD (Asp-Glu-Ala-As) box polypeptide 19A	Homo sapiens
142	poly (ADP-ribose) polymerase 1	Homo sapiens
57461	ISY1 splicing factor homolog (S. cerevisiae)	Homo sapiens
55763	exocyst complex component 1	Homo sapiens
445	argininosuccinate synthetase 1	Homo sapiens
1503	CTP synthase	Homo sapiens
389898	ubiquitin-conjugating enzyme E2N-like	Homo sapiens
7171	tropomyosin 4	Homo sapiens
79567	family with sequence similarity 65, member A	Homo sapiens
56897	Werner helicase interacting protein 1	Homo sapiens
5518	protein phosphatase 2 (formerly 2A), regulatory subunit A, alpha isoform	Homo sapiens
55844	protein phosphatase 2, regulatory subunit B, delta isoform	Homo sapiens
283450	chromosome 12 open reading frame 51	Homo sapiens
23549	aspartyl aminopeptidase	Homo sapiens
3336	heat shock 10kDa protein 1 (chaperonin 10)	Homo sapiens
2802	golgi autoantigen, golgin subfamily a, 3	Homo sapiens
2	alpha-2-macroglobulin	Homo sapiens
1000	cadherin 2, type 1, N-cadherin (neuronal)	Homo sapiens

8473	O-linked N-acetylglucosamine (GlcNAc) transferase (UDP-N-acetylglucosamine:polypeptide-N-acetylglucosaminyl transferase)	Homo sapiens
790	carbamoyl-phosphate synthetase 2, aspartate transcarbamylase, and dihydroorotase	Homo sapiens
79711	importin 4	Homo sapiens
11332	acyl-CoA thioesterase 7	Homo sapiens
867	Cas-Br-M (murine) ecotropic retroviral transforming sequence	Homo sapiens
200014	coiled-coil and C2 domain containing 1B	Homo sapiens
10155	tripartite motif-containing 28	Homo sapiens
1280	collagen, type II, alpha 1	Homo sapiens
81619	tetraspanin 14	Homo sapiens
23380	SLIT-ROBO Rho GTPase activating protein 2	Homo sapiens
10109	actin related protein 2/3 complex, subunit 2, 34kDa	Homo sapiens
1452	casein kinase 1, alpha 1	Homo sapiens
83988	neurocalcin delta	Homo sapiens
5687	proteasome (prosome, macropain) subunit, alpha type, 6	Homo sapiens
2781	guanine nucleotide binding protein (G protein), alpha z polypeptide	Homo sapiens
29979	ubiquilin 1	Homo sapiens
29087	thymocyte nuclear protein 1	Homo sapiens
54622	ADP-ribosylation factor-like 15	Homo sapiens
5901	RAN, member RAS oncogene family	Homo sapiens
27340	similar to Down-regulated in metastasis protein (Key-1A6 protein) (Novel nucleolar protein 73) (NNP73); UTP20, small subunit (SSU) processome component, homolog (yeast)	Homo sapiens
8125	hepatopoietin PCn127; acidic (leucine-rich) nuclear phosphoprotein 32 family, member A	Homo sapiens
3959	lectin, galactoside-binding, soluble, 3 binding protein	Homo sapiens
3190	heterogeneous nuclear ribonucleoprotein K; similar to heterogeneous nuclear ribonucleoprotein K	Homo sapiens
5981	replication factor C (activator 1) 1, 145kDa	Homo sapiens
10212	DEAD (Asp-Glu-Ala-Asp) box polypeptide 39	Homo sapiens
6632	small nuclear ribonucleoprotein D1 polypeptide 16kDa; hypothetical protein LOC100129492	Homo sapiens
100129492	small nuclear ribonucleoprotein D1 polypeptide 16kDa; hypothetical protein LOC100129492	Homo sapiens
57446	NDRG family member 3	Homo sapiens
5707	proteasome (prosome, macropain) 26S subunit, non-ATPase, 1	Homo sapiens
9322	thyroid hormone receptor interactor 10	Homo sapiens
51510	chromatin modifying protein 5	Homo sapiens
984	similar to cell division cycle 2-like 1 (PITSLRE proteins); cell division cycle 2-like 1 (PITSLRE proteins); cell division cycle 2-like 2 (PITSLRE proteins)	Homo sapiens
221927	chromosome 7 open reading frame 27	Homo sapiens
6647	superoxide dismutase 1, soluble	Homo sapiens
8934	RAB7, member RAS oncogene family-like 1	Homo sapiens
3836	karyopherin alpha 1 (importin alpha 5)	Homo sapiens
1368	carboxypeptidase M	Homo sapiens
5245	prohibitin	Homo sapiens
4628	myosin, heavy chain 10, non-muscle	Homo sapiens
9948	WD repeat domain 1	Homo sapiens
27436	echinoderm microtubule associated protein like 4	Homo sapiens
25796	6-phosphogluconolactonase	Homo sapiens
55681	SCY1-like 2 (S. cerevisiae)	Homo sapiens
10075	HECT, UBA and WWE domain containing 1	Homo sapiens
8668	eukaryotic translation initiation factor 3, subunit I	Homo sapiens
26121	PRP31 pre-mRNA processing factor 31 homolog (S. cerevisiae)	Homo sapiens
5256	phosphorylase kinase, alpha 2 (liver)	Homo sapiens
10857	progesterone receptor membrane component 1	Homo sapiens
5591	similar to protein kinase, DNA-activated, catalytic polypeptide; protein kinase, DNA-activated, catalytic polypeptide	Homo sapiens
29966	striatin, calmodulin binding protein 3	Homo sapiens
1653	DEAD (Asp-Glu-Ala-Asp) box polypeptide 1	Homo sapiens
23244	PDS5, regulator of cohesion maintenance, homolog A (S. cerevisiae)	Homo sapiens
51762	RAB8B, member RAS oncogene family	Homo sapiens
9392	transforming growth factor, beta receptor associated protein 1	Homo sapiens
9582	apolipoprotein B mRNA editing enzyme, catalytic polypeptide-like 3B	Homo sapiens
389	ras homolog gene family, member C	Homo sapiens
55920	regulator of chromosome condensation 2	Homo sapiens
5688	proteasome (prosome, macropain) subunit, alpha type, 7	Homo sapiens
821	calnexin	Homo sapiens
6709	spectrin, alpha, non-erythrocytic 1 (alpha-fodrin)	Homo sapiens
1282	collagen, type IV, alpha 1	Homo sapiens
162	adaptor-related protein complex 1, beta 1 subunit	Homo sapiens
1017	cyclin-dependent kinase 2	Homo sapiens
26509	myoferlin	Homo sapiens
9397	N-myristoyltransferase 2	Homo sapiens
10097	ARP2 actin-related protein 2 homolog (yeast)	Homo sapiens
6320	C-type lectin domain family 11, member A	Homo sapiens
2335	fibronectin 1	Homo sapiens
10174	sorbin and SH3 domain containing 3	Homo sapiens
3326	heat shock protein 90kDa alpha (cytosolic), class B member 1	Homo sapiens
6160	ribosomal protein L31 pseudogene 49; ribosomal protein L31 pseudogene 17; ribosomal protein L31	Homo sapiens
255967	PAN3 poly(A) specific ribonuclease subunit homolog (S. cerevisiae)	Homo sapiens
4067	v-yes-1 Yamaguchi sarcoma viral related oncogene homolog	Homo sapiens
23670	transmembrane protein 2	Homo sapiens
23225	nucleoporin 210kDa	Homo sapiens
10111	RAD50 homolog (S. cerevisiae)	Homo sapiens
1968	eukaryotic translation initiation factor 2, subunit 3 gamma, 52kDa	Homo sapiens
1604	CD55 molecule, decay accelerating factor for complement (Cromer blood group)	Homo sapiens
3611	integrin-linked kinase	Homo sapiens
23111	spastic paraplegia 20 (Troyer syndrome)	Homo sapiens
51493	chromosome 22 open reading frame 28	Homo sapiens
5887	RAD23 homolog B (S. cerevisiae)	Homo sapiens

23070	FtsJ methyltransferase domain containing 2	Homo sapiens
6921	similar to elongin C; transcription elongation factor B (SIII), polypeptide 1 (15kDa, elongin C)	Homo sapiens
80155	NMDA receptor regulated 1	Homo sapiens
4172	minichromosome maintenance complex component 3	Homo sapiens
10061	ATP-binding cassette, sub-family F (GCN20), member 2	Homo sapiens
30000	transportin 2	Homo sapiens
23607	CD2-associated protein	Homo sapiens
9946	crystallin, zeta (quinone reductase)-like 1	Homo sapiens
377	ADP-ribosylation factor 3	Homo sapiens
4162	melanoma cell adhesion molecule	Homo sapiens
5718	proteasome (prosome, macropain) 26S subunit, non-ATPase, 12	Homo sapiens
178	amylo-1, 6-glucosidase, 4-alpha-glucanotransferase	Homo sapiens
6845	vesicle-associated membrane protein 7	Homo sapiens
10535	ribonuclease H2, subunit A	Homo sapiens
8455	attractin	Homo sapiens
9361	lon peptidase 1, mitochondrial	Homo sapiens
9632	SEC24 family, member C (S. cerevisiae)	Homo sapiens
4927	nucleoporin 88kDa	Homo sapiens
5868	RAB5A, member RAS oncogene family	Homo sapiens
1819	developmentally regulated GTP binding protein 2	Homo sapiens
8943	adaptor-related protein complex 3, delta 1 subunit	Homo sapiens
4000	lamin A/C	Homo sapiens
9188	DEAD (Asp-Glu-Ala-Asp) box polypeptide 21	Homo sapiens
8890	eukaryotic translation initiation factor 2B, subunit 4 delta, 67kDa	Homo sapiens
23137	structural maintenance of chromosomes 5	Homo sapiens
64786	TBC1 domain family, member 15	Homo sapiens
94031	HtrA serine peptidase 3	Homo sapiens
6510	solute carrier family 1 (neutral amino acid transporter), member 5	Homo sapiens
11335	similar to chromobox homolog 3; chromobox homolog 3 (HP1 gamma homolog, Drosophila)	Homo sapiens
3895	kinectin 1 (kinesin receptor)	Homo sapiens
9341	vesicle-associated membrane protein 3 (cellubrevin)	Homo sapiens
7247	translin	Homo sapiens
51504	tRNA methyltransferase 11-2 homolog (S. cerevisiae); similar to CG12975	Homo sapiens
2339	farnesyltransferase, CAAX box, alpha	Homo sapiens
10095	actin related protein 2/3 complex, subunit 1B, 41kDa; similar to Actin-related protein 2/3 complex subunit 1B (ARP2/3 complex 41 kDa subunit) (p41-ARC)	Homo sapiens
6748	signal sequence receptor, delta (translocon-associated protein delta)	Homo sapiens
51678	membrane protein, palmitoylated 6 (MAGUK p55 subfamily member 6)	Homo sapiens
83706	fermitin family homolog 3 (Drosophila)	Homo sapiens
10611	PDZ and LIM domain 5	Homo sapiens
8440	NCK adaptor protein 2	Homo sapiens
4676	nucleosome assembly protein 1-like 4	Homo sapiens
7385	ubiquinol-cytochrome c reductase core protein II	Homo sapiens
10657	KH domain containing, RNA binding, signal transduction associated 1	Homo sapiens
6128	ribosomal protein L6 pseudogene 27; ribosomal protein L6 pseudogene 19; ribosomal protein L6; ribosomal protein L6 pseudogene 10	Homo sapiens
2050	EPH receptor B4	Homo sapiens
6139	ribosomal protein L17 pseudogene 22; ribosomal protein L17 pseudogene 36; ribosomal protein L17 pseudogene 20; similar to ribosomal protein L17; ribosomal protein L17 pseudogene 33; ribosomal protein L17 pseudogene 34; ribosomal protein L17 pseudogene 9; ribosomal protein L17; ribosomal protein L17 pseudogene 18; ribosomal protein L17 pseudogene 7; ribosomal protein L17 pseudogene 39	Homo sapiens
1845	dual specificity phosphatase 3	Homo sapiens
64328	exportin 4	Homo sapiens
23438	histidyl-tRNA synthetase 2, mitochondrial (putative); D-tyrosyl-tRNA deacylase 1 homolog (S. cerevisiae)	Homo sapiens
25932	chloride intracellular channel 4	Homo sapiens
23165	nucleoporin 205kDa	Homo sapiens
6727	signal recognition particle 14kDa (homologous Alu RNA binding protein) pseudogene 1; signal recognition particle 14kDa (homologous Alu RNA binding protein)	Homo sapiens
9761	malectin	Homo sapiens
84790	tubulin, alpha 1c	Homo sapiens
54623	Paf1, RNA polymerase II associated factor, homolog (S. cerevisiae)	Homo sapiens
4637	myosin, light chain 6, alkali, smooth muscle and non-muscle	Homo sapiens
11224	ribosomal protein L35; ribosomal protein L35 pseudogene 1; ribosomal protein L35 pseudogene 2	Homo sapiens
3608	interleukin enhancer binding factor 2, 45kDa	Homo sapiens
3241	hippocalcin-like 1	Homo sapiens
1969	EPH receptor A2	Homo sapiens
9525	vacuolar protein sorting 4 homolog B (S. cerevisiae)	Homo sapiens
10146	GTPase activating protein (SH3 domain) binding protein 1	Homo sapiens
11140	cell division cycle 37 homolog (S. cerevisiae)	Homo sapiens
124540	musashi homolog 2 (Drosophila)	Homo sapiens
112858	TP53 regulating kinase	Homo sapiens
23223	ribosomal RNA processing 12 homolog (S. cerevisiae)	Homo sapiens
8666	eukaryotic translation initiation factor 3, subunit G	Homo sapiens
1829	desmoglein 2	Homo sapiens
84100	ADP-ribosylation factor-like 6	Homo sapiens
8665	eukaryotic translation initiation factor 3, subunit F; similar to hCG2040283	Homo sapiens
4192	midkine (neurite growth-promoting factor 2)	Homo sapiens
9260	PDZ and LIM domain 7 (enigma)	Homo sapiens
7277	tubulin, alpha 4a	Homo sapiens
54994	chromosome 20 open reading frame 11	Homo sapiens
81887	LAS1-like (S. cerevisiae)	Homo sapiens
10020	glucosamine (UDP-N-acetyl)-2-epimerase/N-acetylmannosamine kinase	Homo sapiens
949	scavenger receptor class B, member 1	Homo sapiens

2673	glutamine-fructose-6-phosphate transaminase 1	Homo sapiens
5814	purine-rich element binding protein B	Homo sapiens
86	actin-like 6A	Homo sapiens
1655	DEAD (Asp-Glu-Ala-Asp) box polypeptide 5	Homo sapiens
5660	prosaposin	Homo sapiens
7037	transferrin receptor (p90, CD71)	Homo sapiens
55215	Fanconi anemia, complementation group I	Homo sapiens
1025	cyclin-dependent kinase 9	Homo sapiens
9908	GTPase activating protein (SH3 domain) binding protein 2	Homo sapiens
6181	ribosomal protein, large, P2 pseudogene 3; ribosomal protein, large, P2	Homo sapiens
23095	kinesin family member 1B	Homo sapiens
50488	misshapen-like kinase 1 (zebrafish)	Homo sapiens
9321	hypothetical LOC341378; thyroid hormone receptor interactor 11	Homo sapiens
1495	catenin (cadherin-associated protein), alpha 1, 102kDa	Homo sapiens
23195	MDN1, midasin homolog (yeast)	Homo sapiens
92755	tubulin, beta; similar to tubulin, beta 5; tubulin, beta pseudogene 2; tubulin, beta pseudogene 1	Homo sapiens
647000	tubulin, beta; similar to tubulin, beta 5; tubulin, beta pseudogene 2; tubulin, beta pseudogene 1	Homo sapiens
203068	tubulin, beta; similar to tubulin, beta 5; tubulin, beta pseudogene 2; tubulin, beta pseudogene 1	Homo sapiens
442308	tubulin, beta; similar to tubulin, beta 5; tubulin, beta pseudogene 2; tubulin, beta pseudogene 1	Homo sapiens
9124	PDZ and LIM domain 1	Homo sapiens
8027	signal transducing adaptor molecule (SH3 domain and ITAM motif) 1	Homo sapiens
8663	eukaryotic translation initiation factor 3, subunit C	Homo sapiens
2752	glutamate-ammonia ligase (glutamine synthetase)	Homo sapiens
1690	coagulation factor C homolog, cochlin (Limulus polyphemus)	Homo sapiens
54472	toll interacting protein	Homo sapiens
5702	proteasome (prosome, macropain) 26S subunit, ATPase, 3	Homo sapiens
55717	bromodomain and WD repeat domain containing 2	Homo sapiens
817	calcium/calmodulin-dependent protein kinase II delta	Homo sapiens
2959	general transcription factor IIB	Homo sapiens
10426	tubulin, gamma complex associated protein 3	Homo sapiens
54433	GAR1 ribonucleoprotein homolog (yeast)	Homo sapiens
2935	G1 to S phase transition 1	Homo sapiens
79612	NMDA receptor regulated 1-like	Homo sapiens
7260	tumor suppressing subtransferable candidate 1	Homo sapiens
4853	Notch homolog 2 (Drosophila)	Homo sapiens
22954	tripartite motif-containing 32	Homo sapiens
292	solute carrier family 25 (mitochondrial carrier; adenine nucleotide translocator), member 5; solute carrier family 25 (mitochondrial carrier; adenine nucleotide translocator), member 5 pseudogene 8	Homo sapiens
226	aldolase A, fructose-bisphosphate	Homo sapiens
5704	similar to 26S protease regulatory subunit 6B (MIP224) (MB67-interacting protein) (TAT-binding protein 7) (TBP-7); proteasome (prosome, macropain) 26S subunit, ATPase, 4	Homo sapiens
2821	glucose phosphate isomerase	Homo sapiens
1525	coxsackie virus and adenovirus receptor pseudogene 2; coxsackie virus and adenovirus receptor	Homo sapiens
1104	regulator of chromosome condensation 1; SNHG3-RCC1 readthrough transcript	Homo sapiens
9584	similar to RNA binding motif protein 39; RNA binding motif protein 39	Homo sapiens
80381	CD276 molecule	Homo sapiens
3093	ubiquitin-conjugating enzyme E2K (UBC1 homolog, yeast)	Homo sapiens
5859	glutaminyl-tRNA synthetase	Homo sapiens
4240	milk fat globule-EGF factor 8 protein	Homo sapiens
6240	ribonucleotide reductase M1	Homo sapiens
5536	protein phosphatase 5, catalytic subunit	Homo sapiens
381	ADP-ribosylation factor 5	Homo sapiens
7706	tripartite motif-containing 25	Homo sapiens
4686	nuclear cap binding protein subunit 1, 80kDa	Homo sapiens
5693	proteasome (prosome, macropain) subunit, beta type, 5	Homo sapiens
3015	H2A histone family, member Z	Homo sapiens
23450	splicing factor 3b, subunit 3, 130kDa	Homo sapiens
5361	plexin A1	Homo sapiens
11331	prohibitin 2	Homo sapiens
8517	inhibitor of kappa light polypeptide gene enhancer in B-cells, kinase gamma	Homo sapiens
976	CD97 molecule	Homo sapiens
2058	glutamyl-prolyl-tRNA synthetase	Homo sapiens
7874	ubiquitin specific peptidase 7 (herpes virus-associated)	Homo sapiens
29127	Rac GTPase activating protein 1 pseudogene; Rac GTPase activating protein 1	Homo sapiens
5510	protein phosphatase 1, regulatory (inhibitor) subunit 7	Homo sapiens
30845	EH-domain containing 3	Homo sapiens
23077	MYC binding protein 2	Homo sapiens
2806	glutamic-oxaloacetic transaminase 2, mitochondrial (aspartate aminotransferase 2)	Homo sapiens
10131	TNF receptor-associated protein 1	Homo sapiens
3416	insulin-degrading enzyme	Homo sapiens
8449	DEAH (Asp-Glu-Ala-His) box polypeptide 16	Homo sapiens
51160	vacuolar protein sorting 28 homolog (S. cerevisiae)	Homo sapiens
55832	cullin-associated and neddylation-dissociated 1	Homo sapiens
8607	RuvB-like 1 (E. coli)	Homo sapiens
23382	adenosylhomocysteinase-like 2	Homo sapiens
10963	stress-induced-phosphoprotein 1	Homo sapiens
983	cell division cycle 2, G1 to S and G2 to M	Homo sapiens
7913	DEK oncogene	Homo sapiens
509	ATP synthase, H+ transporting, mitochondrial F1 complex, gamma polypeptide 1	Homo sapiens
3615	IMP (inosine monophosphate) dehydrogenase 2	Homo sapiens
25800	solute carrier family 39 (zinc transporter), member 6	Homo sapiens

6169	ribosomal protein L38	Homo sapiens
839	caspase 6, apoptosis-related cysteine peptidase	Homo sapiens
10099	tetraspanin 3	Homo sapiens
3303	heat shock 70kDa protein 1A; heat shock 70kDa protein 1B	Homo sapiens
3304	heat shock 70kDa protein 1A; heat shock 70kDa protein 1B	Homo sapiens
11261	calcium binding protein P22	Homo sapiens
23172	family with sequence similarity 175, member B	Homo sapiens
4952	oculocerebrorenal syndrome of Lowe	Homo sapiens
9141	programmed cell death 5	Homo sapiens
5834	phosphorylase, glycogen; brain	Homo sapiens
9879	DEAD (Asp-Glu-Ala-Asp) box polypeptide 46	Homo sapiens
93185	immunoglobulin superfamily, member 8	Homo sapiens
84747	unc-119 homolog B (C. elegans)	Homo sapiens
51010	exosome component 3	Homo sapiens
9367	RAB9A, member RAS oncogene family	Homo sapiens
6207	ribosomal protein S13 pseudogene 8; ribosomal protein S13; ribosomal protein S13 pseudogene 2	Homo sapiens
64854	ubiquitin specific peptidase 46	Homo sapiens
4174	minichromosome maintenance complex component 5	Homo sapiens
4190	malate dehydrogenase 1, NAD (soluble)	Homo sapiens
4171	minichromosome maintenance complex component 2	Homo sapiens
81609	sorting nexin family member 27	Homo sapiens
961	CD47 molecule	Homo sapiens
2201	fibrillin 2	Homo sapiens
85456	tankyrase 1 binding protein 1, 182kDa	Homo sapiens
57544	thioredoxin domain containing 16	Homo sapiens
271	adenosine monophosphate deaminase 2 (isoform L)	Homo sapiens
79598	centrosomal protein 97kDa	Homo sapiens
23325	KIAA1033	Homo sapiens
55611	OTU domain, ubiquitin aldehyde binding 1	Homo sapiens
8991	selenium binding protein 1	Homo sapiens
213	albumin	Homo sapiens
23318	zinc finger, CCHC domain containing 11	Homo sapiens
10594	PRP8 pre-mRNA processing factor 8 homolog (S. cerevisiae)	Homo sapiens
10015	programmed cell death 6 interacting protein	Homo sapiens
771	carbonic anhydrase XII	Homo sapiens
5358	plastin 3 (T isoform)	Homo sapiens
3514	similar to hCG26659; immunoglobulin kappa constant; similar to Ig kappa chain V-I region HK102 precursor	Homo sapiens
100130100	similar to hCG26659; immunoglobulin kappa constant; similar to Ig kappa chain V-I region HK102 precursor	Homo sapiens
8499	protein tyrosine phosphatase, receptor type, f polypeptide (PTPRF), interacting protein (liprin), alpha 2	Homo sapiens
6599	SWI/SNF related, matrix associated, actin dependent regulator of chromatin, subfamily c, member 1	Homo sapiens
51765	serine/threonine protein kinase MST4	Homo sapiens
81839	vang-like 1 (van gogh, Drosophila)	Homo sapiens
1479	cleavage stimulation factor, 3' pre-RNA, subunit 3, 77kDa	Homo sapiens
1981	eukaryotic translation initiation factor 4 gamma, 1	Homo sapiens
51593	serrate RNA effector molecule homolog (Arabidopsis)	Homo sapiens
10092	actin related protein 2/3 complex, subunit 5, 16kDa	Homo sapiens
4522	methylenetetrahydrofolate dehydrogenase (NADP+ dependent) 1, methenyltetrahydrofolate cyclohydrolase, formyltetrahydrofolate synthetase	Homo sapiens
9475	Rho-associated, coiled-coil containing protein kinase 2	Homo sapiens
6210	ribosomal protein S15a pseudogene 17; ribosomal protein S15a pseudogene 19; ribosomal protein S15a pseudogene 12; ribosomal protein S15a pseudogene 24; ribosomal protein S15a pseudogene 11; ribosomal protein S15a	Homo sapiens
5516	protein phosphatase 2 (formerly 2A), catalytic subunit, beta isoform	Homo sapiens
5982	replication factor C (activator 1) 2, 40kDa	Homo sapiens
5571	protein kinase, AMP-activated, gamma 1 non-catalytic subunit	Homo sapiens
3054	host cell factor C1 (VP16-accessory protein)	Homo sapiens
7078	TIMP metallopeptidase inhibitor 3	Homo sapiens
10053	adaptor-related protein complex 1, mu 2 subunit	Homo sapiens
23412	COMM domain containing 3	Homo sapiens
23039	exportin 7	Homo sapiens
3920	lysosomal-associated membrane protein 2	Homo sapiens
340348	tetraspanin 33	Homo sapiens
8883	NEDD8 activating enzyme E1 subunit 1	Homo sapiens
4040	low density lipoprotein receptor-related protein 6	Homo sapiens
50628	gem (nuclear organelle) associated protein 4	Homo sapiens
6050	ribonuclease/angiogenin inhibitor 1	Homo sapiens
5683	proteasome (prosome, macropain) subunit, alpha type, 2	Homo sapiens
8476	CDC42 binding protein kinase alpha (DMPK-like)	Homo sapiens
57175	coronin, actin binding protein, 1B	Homo sapiens
5700	proteasome (prosome, macropain) 26S subunit, ATPase, 1; similar to protease (prosome, macropain) 26S subunit, ATPase 1	Homo sapiens
2191	fibroblast activation protein, alpha	Homo sapiens
4734	neural precursor cell expressed, developmentally down-regulated 4	Homo sapiens
22884	WD repeat domain 37	Homo sapiens
55041	pleckstrin homology domain containing, family B (evectins) member 2	Homo sapiens
144402	copine VIII	Homo sapiens
6726	signal recognition particle 9-like 1; signal recognition particle 9kDa	Homo sapiens
5226	phosphogluconate dehydrogenase	Homo sapiens
5931	retinoblastoma binding protein 7	Homo sapiens
754	pituitary tumor-transforming 1 interacting protein	Homo sapiens
8773	synaptosomal-associated protein, 23kDa	Homo sapiens
25824	peroxiredoxin 5	Homo sapiens

10556	ribonuclease P/MRP 30kDa subunit	Homo sapiens
6634	small nuclear ribonucleoprotein D3 polypeptide 18kDa	Homo sapiens
23370	Rho/Rac guanine nucleotide exchange factor (GEF) 18	Homo sapiens
1642	damage-specific DNA binding protein 1, 127kDa	Homo sapiens
5714	proteasome (prosome, macropain) 26S subunit, non-ATPase, 8	Homo sapiens
53615	methyl-CpG binding domain protein 3	Homo sapiens
7264	tissue specific transplantation antigen P35B	Homo sapiens
8828	neuropilin 2	Homo sapiens
10466	component of oligomeric golgi complex 5	Homo sapiens
57551	TAO kinase 1	Homo sapiens
4904	Y box binding protein 1	Homo sapiens
63893	ubiquitin-conjugating enzyme E2O	Homo sapiens
4249	mannosyl (alpha-1,6-)-glycoprotein beta-1,6-N-acetyl-glucosaminyltransferase; hypothetical LOC151162	Homo sapiens
7443	vaccinia related kinase 1	Homo sapiens
7398	ubiquitin specific peptidase 1	Homo sapiens
7375	ubiquitin specific peptidase 4 (proto-oncogene)	Homo sapiens
30011	SH3-domain kinase binding protein 1	Homo sapiens
56992	kinesin family member 15	Homo sapiens
4478	moesin	Homo sapiens
2817	glypican 1	Homo sapiens
4017	lysyl oxidase-like 2	Homo sapiens
2590	UDP-N-acetyl-alpha-D-galactosamine:polypeptide N-acetylgalactosaminyltransferase 2 (GalNAc-T2)	Homo sapiens
6499	superkiller viralicidic activity 2-like (S. cerevisiae)	Homo sapiens
2264	fibroblast growth factor receptor 4	Homo sapiens
257364	sorting nexin 33	Homo sapiens
8667	eukaryotic translation initiation factor 3, subunit H	Homo sapiens
51429	sorting nexin 9	Homo sapiens
26052	dynamin 3	Homo sapiens
5356	pleiotropic regulator 1 (PRL1 homolog, Arabidopsis)	Homo sapiens
6223	ribosomal protein S19 pseudogene 3; ribosomal protein S19	Homo sapiens
548596	creatine kinase, mitochondrial 1A; creatine kinase, mitochondrial 1B	Homo sapiens
1159	creatine kinase, mitochondrial 1A; creatine kinase, mitochondrial 1B	Homo sapiens
84034	elastin microfibril interfacer 2	Homo sapiens
4691	nucleolin	Homo sapiens
7018	transferrin	Homo sapiens
6155	ribosomal protein L27	Homo sapiens
4173	minichromosome maintenance complex component 4	Homo sapiens
5701	proteasome (prosome, macropain) 26S subunit, ATPase, 2	Homo sapiens
5710	proteasome (prosome, macropain) 26S subunit, non-ATPase, 4	Homo sapiens
5048	platelet-activating factor acetylhydrolase, isoform Ib, subunit 1 (45kDa)	Homo sapiens
7001	peroxiredoxin 2	Homo sapiens
5878	RAB5C, member RAS oncogene family	Homo sapiens
10916	melanoma antigen family D, 2	Homo sapiens
3485	insulin-like growth factor binding protein 2, 36kDa	Homo sapiens
11252	protein kinase C and casein kinase substrate in neurons 2	Homo sapiens
375790	agrin	Homo sapiens
1289	collagen, type V, alpha 1	Homo sapiens
