# Supplementary material for: Identification and Characterization of 293T Cell-Derived Exosomes by Profiling the Protein, mRNA and MicroRNA Components
Source: PLoS One. 2016 Sep 20;11(9):e0163043. doi: 10.1371/journal.pone.0163043 (PMC5029934; doi:10.1371/journal.pone.0163043)
Supplement: S3 Table — (PDF) [file pone.0163043.s004.pdf]

ENTREZ_GENE_ID	Name	Species
63897	HEAT repeat containing 6	Homo sapiens
26057	ankyrin repeat domain 17	Homo sapiens
55295	kelch-like 26 (Drosophila)	Homo sapiens
5822	PWP2 periodic tryptophan protein homolog (yeast)	Homo sapiens
341315	poly(A) binding protein, cytoplasmic pseudogene 5; poly(A) binding protein, cytoplasmic 1	Homo sapiens
4753	NEL-like 2 (chicken)	Homo sapiens
100128526	hypothetical LOC100128526; target of myb1 (chicken)	Homo sapiens
64895	poly(A) polymerase gamma	Homo sapiens
57405	SPC25, NDC80 kinetochore complex component, homolog (S. cerevisiae)	Homo sapiens
9282	mediator complex subunit 14	Homo sapiens
84057	meiotic nuclear divisions 1 homolog (S. cerevisiae)	Homo sapiens
5394	exosome component 10	Homo sapiens
81610	family with sequence similarity 83, member D	Homo sapiens
400058	makorin ring finger protein pseudogene 6; makorin ring finger protein 1	Homo sapiens
23424	tudor domain containing 7	Homo sapiens
155370	Shwachman-Bodian-Diamond syndrome pseudogene; Shwachman-Bodian-Diamond syndrome	Homo sapiens
100129553	ribosomal protein L13a pseudogene 7; ribosomal protein L13a pseudogene 5; ribosomal protein L13a pseudogene 16; ribosomal protein L13a; ribosomal protein L13a pseudogene 18	Homo sapiens
284821	ribosomal protein L13a pseudogene 7; ribosomal protein L13a pseudogene 5; ribosomal protein L13a pseudogene 16; ribosomal protein L13a; ribosomal protein L13a pseudogene 18	Homo sapiens
728658	ribosomal protein L13a pseudogene 7; ribosomal protein L13a pseudogene 5; ribosomal protein L13a pseudogene 16; ribosomal protein L13a; ribosomal protein L13a pseudogene 18	Homo sapiens
402342	ribosomal protein L13a pseudogene 7; ribosomal protein L13a pseudogene 5; ribosomal protein L13a pseudogene 16; ribosomal protein L13a; ribosomal protein L13a pseudogene 18	Homo sapiens
79187	fibronectin type III and SPRY domain containing 1	Homo sapiens
5789	protein tyrosine phosphatase, receptor type, D	Homo sapiens
653199	family with sequence similarity 115, member A; family with sequence similarity 115, member B (pseudogene)	Homo sapiens
642443	alcohol dehydrogenase 5 (class III), chi polypeptide, pseudogene 4; alcohol dehydrogenase 5 (class III), chi polypeptide	Homo sapiens
7403	lysine (K)-specific demethylase 6A	Homo sapiens
149224	ribosomal protein S7; ribosomal protein S7 pseudogene 11; ribosomal protein S7 pseudogene 4; ribosomal protein S7 pseudogene 10	Homo sapiens
100128060	ribosomal protein S7; ribosomal protein S7 pseudogene 11; ribosomal protein S7 pseudogene 4; ribosomal protein S7 pseudogene 10	Homo sapiens
644315	ribosomal protein S7; ribosomal protein S7 pseudogene 11; ribosomal protein S7 pseudogene 4; ribosomal protein S7 pseudogene 10	Homo sapiens
283373	ankyrin repeat domain 52	Homo sapiens
654350	ribosomal protein L9; ribosomal protein L9 pseudogene 25	Homo sapiens
84301	DDI1, DNA-damage inducible 1, homolog 2 (S. cerevisiae)	Homo sapiens
8861	LIM domain binding 1	Homo sapiens
8100	intraflagellar transport 88 homolog (Chlamydomonas)	Homo sapiens
647033	proliferation-associated 2G4, 38kDa; proliferation-associated 2G4 pseudogene 4	Homo sapiens
8872	cell division cycle 123 homolog (S. cerevisiae)	Homo sapiens
7146	tenascin XB; tenascin XA pseudogene	Homo sapiens
7148	tenascin XB; tenascin XA pseudogene	Homo sapiens
80746	tRNA splicing endonuclease 2 homolog (S. cerevisiae)	Homo sapiens
5125	proprotein convertase subtilisin/kexin type 5	Homo sapiens
642132	roundabout, axon guidance receptor, homolog 1 (Drosophila); similar to roundabout 1 isoform b	Homo sapiens
6091	roundabout, axon guidance receptor, homolog 1 (Drosophila); similar to roundabout 1 isoform b	Homo sapiens
85377	MICAL-like 1	Homo sapiens
81570	ClpB caseinolytic peptidase B homolog (E. coli)	Homo sapiens
728620	ribosomal protein L7-like 1; ribosomal protein L7 pseudogene 14; ribosomal protein L7 pseudogene 21; ribosomal protein L7 pseudogene 22; ribosomal protein L7 pseudogene 46	Homo sapiens
642451	ribosomal protein L7-like 1; ribosomal protein L7 pseudogene 14; ribosomal protein L7 pseudogene 21; ribosomal protein L7 pseudogene 22; ribosomal protein L7 pseudogene 46	Homo sapiens
729123	ribosomal protein L7-like 1; ribosomal protein L7 pseudogene 14; ribosomal protein L7 pseudogene 21; ribosomal protein L7 pseudogene 22; ribosomal protein L7 pseudogene 46	Homo sapiens
100129743	ribosomal protein L7-like 1; ribosomal protein L7 pseudogene 14; ribosomal protein L7 pseudogene 21; ribosomal protein L7 pseudogene 22; ribosomal protein L7 pseudogene 46	Homo sapiens
285855	ribosomal protein L7-like 1; ribosomal protein L7 pseudogene 14; ribosomal protein L7 pseudogene 21; ribosomal protein L7 pseudogene 22; ribosomal protein L7 pseudogene 46	Homo sapiens
9202	zinc finger, MYM-type 4	Homo sapiens
26065	LSM14A, SCD6 homolog A (S. cerevisiae)	Homo sapiens
84193	SET domain containing 3	Homo sapiens

9928	kinesin family member 14	Homo sapiens
9377	cytochrome c oxidase subunit Va	Homo sapiens
26043	UBX domain protein 7	Homo sapiens
60528	elaC homolog 2 (E. coli)	Homo sapiens
1300	collagen, type X, alpha 1	Homo sapiens
23118	mitogen-activated protein kinase kinase kinase 7 interacting protein 2	Homo sapiens
6837	mediator complex subunit 22	Homo sapiens
79078	chromosome 1 open reading frame 50	Homo sapiens
1176	adaptor-related protein complex 3, sigma 1 subunit	Homo sapiens
25793	F-box protein 7	Homo sapiens
8637	ankyrin repeat and KH domain containing 1; ANKHD1-EIF4EBP3 readthrough transcript; eukaryotic translation initiation factor 4E binding protein 3	Homo sapiens
54882	ankyrin repeat and KH domain containing 1; ANKHD1-EIF4EBP3 readthrough transcript; eukaryotic translation initiation factor 4E binding protein 3	Homo sapiens
404734	ankyrin repeat and KH domain containing 1; ANKHD1-EIF4EBP3 readthrough transcript; eukaryotic translation initiation factor 4E binding protein 3	Homo sapiens
22794	cancer susceptibility candidate 3	Homo sapiens
84376	hook homolog 3 (Drosophila)	Homo sapiens
652460	similar to Plectin 1 (PLTN) (PCN) (Hemidesmosomal protein 1) (HD1); plectin 1, intermediate filament binding protein 500kDa	Homo sapiens
23126	pogo transposable element with ZNF domain	Homo sapiens
6936	chromosome 2 open reading frame 3	Homo sapiens
7515	X-ray repair complementing defective repair in Chinese hamster cells 1	Homo sapiens
10749	kinesin family member 1C	Homo sapiens
730031	similar to Afadin (Protein AF-6); myeloid/lymphoid or mixed-lineage leukemia (trithorax homolog, Drosophila); translocated to, 4	Homo sapiens
64342	HCLS1 binding protein 3	Homo sapiens
9647	protein phosphatase 1F (PP2C domain containing)	Homo sapiens
8242	lysine (K)-specific demethylase 5C	Homo sapiens
79068	fat mass and obesity associated	Homo sapiens
90826	protein arginine methyltransferase 10 (putative)	Homo sapiens
9896	FIG4 homolog (S. cerevisiae)	Homo sapiens
79893	gametogenetin binding protein 2	Homo sapiens
6801	striatin, calmodulin binding protein	Homo sapiens
1801	DPH1 homolog (S. cerevisiae)	Homo sapiens
150737	tetratricopeptide repeat domain 30B	Homo sapiens
389342	ribosomal protein L10; ribosomal protein L10 pseudogene 15; ribosomal protein L10 pseudogene 6; ribosomal protein L10 pseudogene 16; ribosomal protein L10 pseudogene 9	Homo sapiens
100129975	ribosomal protein L10; ribosomal protein L10 pseudogene 15; ribosomal protein L10 pseudogene 6; ribosomal protein L10 pseudogene 16; ribosomal protein L10 pseudogene 9	Homo sapiens
285176	ribosomal protein L10; ribosomal protein L10 pseudogene 15; ribosomal protein L10 pseudogene 6; ribosomal protein L10 pseudogene 16; ribosomal protein L10 pseudogene 9	Homo sapiens
284393	ribosomal protein L10; ribosomal protein L10 pseudogene 15; ribosomal protein L10 pseudogene 6; ribosomal protein L10 pseudogene 16; ribosomal protein L10 pseudogene 9	Homo sapiens
55095	sterile alpha motif domain containing 4B	Homo sapiens
23678	serum/glucocorticoid regulated kinase family, member 3	Homo sapiens
154796	angiomotin	Homo sapiens
55325	UFM1-specific peptidase 2	Homo sapiens
23636	nucleoporin 62kDa	Homo sapiens
4898	nardilysin (N-arginine dibasic convertase)	Homo sapiens
10629	TAF6-like RNA polymerase II, p300/CBP-associated factor (PCAF)-associated factor, 65kDa	Homo sapiens
51096	UTP18, small subunit (SSU) processome component, homolog (yeast)	Homo sapiens
11167	follistatin-like 1	Homo sapiens
9337	CCR4-NOT transcription complex, subunit 8	Homo sapiens
200576	phosphoinositide kinase, FYVE finger containing	Homo sapiens
116138	kelch domain containing 3	Homo sapiens
55621	TRM1 tRNA methyltransferase 1 homolog (S. cerevisiae)	Homo sapiens
3026	hyaluronan binding protein 2	Homo sapiens
221830	TWIST neighbor	Homo sapiens
644029	ribosomal protein L7a pseudogene 70; ribosomal protein L7a; ribosomal protein L7a pseudogene 30; ribosomal protein L7a pseudogene 66; ribosomal protein L7a pseudogene 27; ribosomal protein L7a pseudogene 11; ribosomal protein L7a pseudogene 62	Homo sapiens
152663	ribosomal protein L7a pseudogene 70; ribosomal protein L7a; ribosomal protein L7a pseudogene 30; ribosomal protein L7a pseudogene 66; ribosomal protein L7a pseudogene 27; ribosomal protein L7a pseudogene 11; ribosomal protein L7a pseudogene 62	Homo sapiens
728139	ribosomal protein L7a pseudogene 70; ribosomal protein L7a; ribosomal protein L7a pseudogene 30; ribosomal protein L7a pseudogene 66; ribosomal protein L7a pseudogene 27; ribosomal protein L7a pseudogene 11; ribosomal protein L7a pseudogene 62	Homo sapiens
728992	ribosomal protein L7a pseudogene 70; ribosomal protein L7a; ribosomal protein L7a pseudogene 30; ribosomal protein L7a pseudogene 66; ribosomal protein L7a pseudogene 27; ribosomal protein L7a pseudogene 11; ribosomal protein L7a pseudogene 62	Homo sapiens

388474	ribosomal protein L7a pseudogene 70; ribosomal protein L7a; ribosomal protein L7a pseudogene 30; ribosomal protein L7a pseudogene 66; ribosomal protein L7a pseudogene 27; ribosomal protein L7a pseudogene 11; ribosomal protein L7a pseudogene 62	Homo sapiens
441034	ribosomal protein L7a pseudogene 70; ribosomal protein L7a; ribosomal protein L7a pseudogene 30; ribosomal protein L7a pseudogene 66; ribosomal protein L7a pseudogene 27; ribosomal protein L7a pseudogene 11; ribosomal protein L7a pseudogene 62	Homo sapiens
55102	ATG2 autophagy related 2 homolog B (S. cerevisiae)	Homo sapiens
10622	polymerase (RNA) III (DNA directed) polypeptide G (32kD)	Homo sapiens
54468	missing oocyte, meiosis regulator, homolog (Drosophila)	Homo sapiens
27342	RAB guanine nucleotide exchange factor (GEF) 1	Homo sapiens
10577	Niemann-Pick disease, type C2	Homo sapiens
11180	WD repeat domain 6	Homo sapiens
677797	small nucleolar RNA, H/ACA box 7A; small nucleolar RNA, H/ACA box 7B; ribosomal protein L32	Homo sapiens
619563	small nucleolar RNA, H/ACA box 7A; small nucleolar RNA, H/ACA box 7B; ribosomal protein L32	Homo sapiens
401115	chromosome 4 open reading frame 48	Homo sapiens
728599	cytokine induced apoptosis inhibitor 1; cytokine induced apoptosis inhibitor 1 pseudogene	Homo sapiens
57019	cytokine induced apoptosis inhibitor 1; cytokine induced apoptosis inhibitor 1 pseudogene	Homo sapiens
5818	poliovirus receptor-related 1 (herpesvirus entry mediator C)	Homo sapiens
51780	lysine (K)-specific demethylase 3B	Homo sapiens
51611	DPH5 homolog (S. cerevisiae)	Homo sapiens
80173	intraflagellar transport 74 homolog (Chlamydomonas)	Homo sapiens
51091	Sep (O-phosphoserine) tRNA:Sec (selenocysteine) tRNA synthase	Homo sapiens
5426	polymerase (DNA directed), epsilon	Homo sapiens
5157	platelet-derived growth factor receptor-like	Homo sapiens
407835	mitogen-activated protein kinase kinase 2 pseudogene; mitogen-activated protein kinase kinase 2	Homo sapiens
7342	upstream binding protein 1 (LBP-1a)	Homo sapiens
100129424	ribosomal protein L19; ribosomal protein L19 pseudogene 12	Homo sapiens
3654	interleukin-1 receptor-associated kinase 1	Homo sapiens
285053	ribosomal protein L18a pseudogene 6; ribosomal protein L18a	Homo sapiens
388076	ribosomal protein S8; ribosomal protein S8 pseudogene 8; ribosomal protein S8 pseudogene 10	Homo sapiens
728553	ribosomal protein S8; ribosomal protein S8 pseudogene 8; ribosomal protein S8 pseudogene 10	Homo sapiens
57492	AT rich interactive domain 1B (SWI1-like)	Homo sapiens
283106	casein kinase 2, alpha 1 polypeptide pseudogene; casein kinase 2, alpha 1 polypeptide	Homo sapiens
7536	splicing factor 1	Homo sapiens
9295	splicing factor, arginine/serine-rich 11	Homo sapiens
84991	RNA binding motif protein 17	Homo sapiens
57120	golgi associated PDZ and coiled-coil motif containing	Homo sapiens
283989	tRNA splicing endonuclease 54 homolog (S. cerevisiae)	Homo sapiens
402100	ribosomal protein L22 pseudogene 11; ribosomal protein L22	Homo sapiens
8030	coiled-coil domain containing 6	Homo sapiens
23022	palladin, cytoskeletal associated protein	Homo sapiens
55665	up-regulated gene 4	Homo sapiens
2072	excision repair cross-complementing rodent repair deficiency, complementation group 4	Homo sapiens
55181	chromosome 17 open reading frame 71	Homo sapiens
4854	Notch homolog 3 (Drosophila)	Homo sapiens
23160	WD repeat domain 43	Homo sapiens
124944	chromosome 17 open reading frame 49	Homo sapiens
84296	GINS complex subunit 4 (Sld5 homolog)	Homo sapiens
57221	KIAA1244	Homo sapiens
90187	elastin microfibril interfacer 3	Homo sapiens
23210	jumonji domain containing 6	Homo sapiens
442578	aminoacyl tRNA synthetase complex-interacting multifunctional protein 2; stromal antigen 3-like 3	Homo sapiens
26993	A kinase (PRKA) anchor protein 8-like	Homo sapiens
56950	SET and MYND domain containing 2	Homo sapiens
100093631	general transcription factor II, i; general transcription factor II, i, pseudogene	Homo sapiens
645715	eukaryotic translation elongation factor 1 alpha-like 7; eukaryotic translation elongation factor 1 alpha-like 3; similar to eukaryotic translation elongation factor 1 alpha 1; eukaryotic translation elongation factor 1 alpha 1	Homo sapiens
441032	eukaryotic translation elongation factor 1 alpha-like 7; eukaryotic translation elongation factor 1 alpha-like 3; similar to eukaryotic translation elongation factor 1 alpha 1; eukaryotic translation elongation factor 1 alpha 1	Homo sapiens
23381	Smg-5 homolog, nonsense mediated mRNA decay factor (C. elegans)	Homo sapiens
729163	fatty acid binding protein 5-like 2; fatty acid binding protein 5 (psoriasis-associated); fatty acid binding protein 5-like 8; fatty acid binding protein 5-like 7; fatty acid binding protein 5-like 9	Homo sapiens

642956	fatty acid binding protein 5-like 2; fatty acid binding protein 5 (psoriasis-associated); fatty acid binding protein 5-like 8; fatty acid binding protein 5-like 7; fatty acid binding protein 5-like 9	Homo sapiens
728729	fatty acid binding protein 5-like 2; fatty acid binding protein 5 (psoriasis-associated); fatty acid binding protein 5-like 8; fatty acid binding protein 5-like 7; fatty acid binding protein 5-like 9	Homo sapiens
644877	similar to calcyclin binding protein; calcyclin binding protein	Homo sapiens
6654	son of sevenless homolog 1 (Drosophila)	Homo sapiens
200316	apolipoprotein B mRNA editing enzyme, catalytic polypeptide-like 3F	Homo sapiens
2045	EPH receptor A7	Homo sapiens
257160	ring finger protein 214	Homo sapiens
6650	small optic lobes homolog (Drosophila)	Homo sapiens
219927	mitochondrial ribosomal protein L21	Homo sapiens
51550	cyclin-dependent kinase 2-interacting protein	Homo sapiens
10260	DENN/MADD domain containing 4A	Homo sapiens
148223	chromosome 19 open reading frame 25	Homo sapiens
100133042	glyceraldehyde-3-phosphate dehydrogenase-like 6; hypothetical protein LOC100133042; glyceraldehyde-3-phosphate dehydrogenase	Homo sapiens
729403	glyceraldehyde-3-phosphate dehydrogenase-like 6; hypothetical protein LOC100133042; glyceraldehyde-3-phosphate dehydrogenase	Homo sapiens
7090	transducin-like enhancer of split 3 (E(sp1) homolog, Drosophila)	Homo sapiens
9836	leucine carboxyl methyltransferase 2	Homo sapiens
10869	ubiquitin specific peptidase 19	Homo sapiens
8635	ribonuclease T2	Homo sapiens
100130746	AKT interacting protein; similar to AKT interacting protein	Homo sapiens
64400	AKT interacting protein; similar to AKT interacting protein	Homo sapiens
7750	zinc finger, MYM-type 2	Homo sapiens
55248	transmembrane protein 206	Homo sapiens
10274	stromal antigen 1	Homo sapiens
653884	FUS interacting protein (serine/arginine-rich) 1; similar to FUS interacting protein (serine-arginine rich) 1	Homo sapiens
800	caldesmon 1	Homo sapiens
11020	RAB, member of RAS oncogene family-like 4	Homo sapiens
10762	nucleoporin 50kDa	Homo sapiens
58155	polypyrimidine tract binding protein 2	Homo sapiens
51428	DEAD (Asp-Glu-Ala-Asp) box polypeptide 41	Homo sapiens
79871	RNA polymerase II associated protein 2	Homo sapiens
652797	similar to Pyruvate kinase, isozymes M1/M2 (Pyruvate kinase muscle isozyme) (Cytosolic thyroid hormone-binding protein) (CTHBP) (THBP1); pyruvate kinase, muscle	Homo sapiens
5001	origin recognition complex, subunit 5-like (yeast)	Homo sapiens
285829	SMT3 suppressor of mif two 3 homolog 2 (S. cerevisiae) pseudogene; SMT3 suppressor of mif two 3 homolog 2 (S. cerevisiae); SMT3 suppressor of mif two 3 homolog 3 (S. cerevisiae)	Homo sapiens
23061	TBC1 domain family, member 9B (with GRAM domain)	Homo sapiens
5585	protein kinase N1	Homo sapiens
729789	ribosomal protein S15 pseudogene 5; ribosomal protein S15	Homo sapiens
545	ataxia telangiectasia and Rad3 related; similar to ataxia telangiectasia and Rad3 related protein	Homo sapiens
648152	ataxia telangiectasia and Rad3 related; similar to ataxia telangiectasia and Rad3 related protein	Homo sapiens
651921	ataxia telangiectasia and Rad3 related; similar to ataxia telangiectasia and Rad3 related protein	Homo sapiens
23613	zinc finger, MYND-type containing 8	Homo sapiens
653631	hypothetical LOC653631; hypothetical LOC646050; hypothetical LOC646890; axin interactor, dorsalization associated	Homo sapiens
646050	hypothetical LOC653631; hypothetical LOC646050; hypothetical LOC646890; axin interactor, dorsalization associated	Homo sapiens
646890	hypothetical LOC653631; hypothetical LOC646050; hypothetical LOC646890; axin interactor, dorsalization associated	Homo sapiens
22848	AP2 associated kinase 1	Homo sapiens
23640	HSPA (heat shock 70kDa) binding protein, cytoplasmic cochaperone 1	Homo sapiens
100133673	tubulin, gamma 1; similar to Tubulin, gamma 1	Homo sapiens
26960	neurobeachin	Homo sapiens
1454	casein kinase 1, epsilon	Homo sapiens
347292	ribosomal protein L36; ribosomal protein L36 pseudogene 14	Homo sapiens
64145	zinc finger, FYVE domain containing 20	Homo sapiens
8139	gigaxonin	Homo sapiens
6877	TAF5 RNA polymerase II, TATA box binding protein (TBP)-associated factor, 100kDa	Homo sapiens
54780	non-SMC element 4 homolog A (S. cerevisiae)	Homo sapiens
57534	mindbomb homolog 1 (Drosophila)	Homo sapiens
55764	intraflagellar transport 122 homolog (Chlamydomonas)	Homo sapiens
11325	DEAD (Asp-Glu-Ala-Asp) box polypeptide 42	Homo sapiens
79912	pyridine nucleotide-disulphide oxidoreductase domain 1	Homo sapiens
26155	nucleolar complex associated 2 homolog (S. cerevisiae)	Homo sapiens
23560	GTP binding protein 4	Homo sapiens
100130802	ribosomal protein L39 pseudogene 10; ribosomal protein L39 pseudogene 20; ribosomal protein L39 pseudogene 27; ribosomal protein L39; ribosomal protein L39 pseudogene 13; ribosomal protein L39 pseudogene 32	Homo sapiens

6170	ribosomal protein L39 pseudogene 10; ribosomal protein L39 pseudogene 20; ribosomal protein L39 pseudogene 27; ribosomal protein L39; ribosomal protein L39 pseudogene 13; ribosomal protein L39 pseudogene 32	Homo sapiens
100131160	ribosomal protein L39 pseudogene 10; ribosomal protein L39 pseudogene 20; ribosomal protein L39 pseudogene 27; ribosomal protein L39; ribosomal protein L39 pseudogene 13; ribosomal protein L39 pseudogene 32	Homo sapiens
100131387	ribosomal protein L39 pseudogene 10; ribosomal protein L39 pseudogene 20; ribosomal protein L39 pseudogene 27; ribosomal protein L39; ribosomal protein L39 pseudogene 13; ribosomal protein L39 pseudogene 32	Homo sapiens
100129952	ribosomal protein L39 pseudogene 10; ribosomal protein L39 pseudogene 20; ribosomal protein L39 pseudogene 27; ribosomal protein L39; ribosomal protein L39 pseudogene 13; ribosomal protein L39 pseudogene 32	Homo sapiens
100133222	ribosomal protein L39 pseudogene 10; ribosomal protein L39 pseudogene 20; ribosomal protein L39 pseudogene 27; ribosomal protein L39; ribosomal protein L39 pseudogene 13; ribosomal protein L39 pseudogene 32	Homo sapiens
728698	similar to eukaryotic translation initiation factor 4A; small nucleolar RNA, H/ACA box 67; eukaryotic translation initiation factor 4A, isoform 1	Homo sapiens
26781	similar to eukaryotic translation initiation factor 4A; small nucleolar RNA, H/ACA box 67; eukaryotic translation initiation factor 4A, isoform 1	Homo sapiens
546	alpha thalassemia/mental retardation syndrome X-linked (RAD54 homolog, S. cerevisiae)	Homo sapiens
5937	RNA binding motif, single stranded interacting protein 1	Homo sapiens
84002	UDP-GlcNAc:betaGal beta-1,3-N-acetylglucosaminyltransferase 5	Homo sapiens
55757	UDP-glucose ceramide glucosyltransferase-like 2	Homo sapiens
440434	hypothetical protein FLJ11822; aminopeptidase puromycin sensitive	Homo sapiens
93664	Ca++-dependent secretion activator 2	Homo sapiens
84889	solute carrier family 7 (cationic amino acid transporter, y+ system), member 3	Homo sapiens
8896	BUD31 homolog (S. cerevisiae)	Homo sapiens
23291	F-box and WD repeat domain containing 11	Homo sapiens
126792	UDP-Gal:betaGal beta 1,3-galactosyltransferase polypeptide 6	Homo sapiens
2186	bromodomain PHD finger transcription factor	Homo sapiens
65080	mitochondrial ribosomal protein L44	Homo sapiens
54205	cytochrome c, somatic	Homo sapiens
51367	processing of precursor 5, ribonuclease P/MRP subunit (S. cerevisiae)	Homo sapiens
10076	protein tyrosine phosphatase, receptor type, U	Homo sapiens
5162	pyruvate dehydrogenase (lipoamide) beta	Homo sapiens
648000	ribosomal protein L7 pseudogene 26; ribosomal protein L7 pseudogene 16; ribosomal protein L7; ribosomal protein L7 pseudogene 32; ribosomal protein L7 pseudogene 23; ribosomal protein L7 pseudogene 24; ribosomal protein L7 pseudogene 20	Homo sapiens
728843	ribosomal protein L7 pseudogene 26; ribosomal protein L7 pseudogene 16; ribosomal protein L7; ribosomal protein L7 pseudogene 32; ribosomal protein L7 pseudogene 23; ribosomal protein L7 pseudogene 24; ribosomal protein L7 pseudogene 20	Homo sapiens
729677	ribosomal protein L7 pseudogene 26; ribosomal protein L7 pseudogene 16; ribosomal protein L7; ribosomal protein L7 pseudogene 32; ribosomal protein L7 pseudogene 23; ribosomal protein L7 pseudogene 24; ribosomal protein L7 pseudogene 20	Homo sapiens
728380	ribosomal protein L7 pseudogene 26; ribosomal protein L7 pseudogene 16; ribosomal protein L7; ribosomal protein L7 pseudogene 32; ribosomal protein L7 pseudogene 23; ribosomal protein L7 pseudogene 24; ribosomal protein L7 pseudogene 20	Homo sapiens
100130892	ribosomal protein L7 pseudogene 26; ribosomal protein L7 pseudogene 16; ribosomal protein L7; ribosomal protein L7 pseudogene 32; ribosomal protein L7 pseudogene 23; ribosomal protein L7 pseudogene 24; ribosomal protein L7 pseudogene 20	Homo sapiens
100127893	ribosomal protein L7 pseudogene 26; ribosomal protein L7 pseudogene 16; ribosomal protein L7; ribosomal protein L7 pseudogene 32; ribosomal protein L7 pseudogene 23; ribosomal protein L7 pseudogene 24; ribosomal protein L7 pseudogene 20	Homo sapiens
286512	similar to eukaryotic translation initiation factor 4A2; eukaryotic translation initiation factor 4A, isoform 2	Homo sapiens
100133234	SET binding factor 1; SET binding factor 1 pseudogene 1	Homo sapiens
4216	mitogen-activated protein kinase kinase kinase 4	Homo sapiens
9785	DEAH (Asp-Glu-Ala-His) box polypeptide 38	Homo sapiens
51397	COMM domain containing 10	Homo sapiens
58485	trafficking protein particle complex 1	Homo sapiens
81050	olfactory receptor, family 5, subfamily AC, member 2	Homo sapiens
647150	similar to ATP-binding cassette, sub-family E, member 1; ATP-binding cassette, sub-family E (OABP), member 1	Homo sapiens
8479	HIRA interacting protein 3	Homo sapiens
10621	polymerase (RNA) III (DNA directed) polypeptide F, 39 kDa	Homo sapiens
5469	mediator complex subunit 1	Homo sapiens
26528	DAZ associated protein 1	Homo sapiens
29062	WD repeat domain 91	Homo sapiens
54617	INO80 homolog (S. cerevisiae)	Homo sapiens
80007	chromosome 10 open reading frame 88	Homo sapiens
701	budding uninhibited by benzimidazoles 1 homolog beta (yeast)	Homo sapiens
80144	Fraser syndrome 1	Homo sapiens

55683	KIAA1310	Homo sapiens
100133829	similar to hCG1991431; similar to COMPase; ADAM metallopeptidase with thrombospondin type 1 motif, 7	Homo sapiens
11173	similar to hCG1991431; similar to COMPase; ADAM metallopeptidase with thrombospondin type 1 motif, 7	Homo sapiens
642935	similar to hCG1991431; similar to COMPase; ADAM metallopeptidase with thrombospondin type 1 motif, 7	Homo sapiens
122553	trafficking protein particle complex 6B	Homo sapiens
29781	non-SMC condensin II complex, subunit H2	Homo sapiens
65220	NAD kinase	Homo sapiens
8208	chromatin assembly factor 1, subunit B (p60)	Homo sapiens
644422	splicing factor, arginine/serine-rich 6; similar to arginine/serine-rich splicing factor 6	Homo sapiens
130773	ribosomal protein L23a pseudogene 63; ribosomal protein L23a pseudogene 75; ribosomal protein L23a pseudogene 37; ribosomal protein L23a pseudogene 65; ribosomal protein L23a pseudogene 43; ribosomal protein L23a pseudogene 44; ribosomal protein L23a	Homo sapiens
729798	ribosomal protein L23a pseudogene 63; ribosomal protein L23a pseudogene 75; ribosomal protein L23a pseudogene 37; ribosomal protein L23a pseudogene 65; ribosomal protein L23a pseudogene 43; ribosomal protein L23a pseudogene 44; ribosomal protein L23a	Homo sapiens
389101	ribosomal protein L23a pseudogene 63; ribosomal protein L23a pseudogene 75; ribosomal protein L23a pseudogene 37; ribosomal protein L23a pseudogene 65; ribosomal protein L23a pseudogene 43; ribosomal protein L23a pseudogene 44; ribosomal protein L23a	Homo sapiens
728207	ribosomal protein L23a pseudogene 63; ribosomal protein L23a pseudogene 75; ribosomal protein L23a pseudogene 37; ribosomal protein L23a pseudogene 65; ribosomal protein L23a pseudogene 43; ribosomal protein L23a pseudogene 44; ribosomal protein L23a	Homo sapiens
440027	ribosomal protein L23a pseudogene 63; ribosomal protein L23a pseudogene 75; ribosomal protein L23a pseudogene 37; ribosomal protein L23a pseudogene 65; ribosomal protein L23a pseudogene 43; ribosomal protein L23a pseudogene 44; ribosomal protein L23a	Homo sapiens
391825	ribosomal protein L23a pseudogene 63; ribosomal protein L23a pseudogene 75; ribosomal protein L23a pseudogene 37; ribosomal protein L23a pseudogene 65; ribosomal protein L23a pseudogene 43; ribosomal protein L23a pseudogene 44; ribosomal protein L23a	Homo sapiens
100129500	hypothetical LOC100129500; apolipoprotein E	Homo sapiens
4116	mago-nashi homolog, proliferation-associated (Drosophila)	Homo sapiens
9972	nucleoporin 153kDa	Homo sapiens
1948	ephrin-B2	Homo sapiens
158345	ribosomal protein L4; ribosomal protein L4 pseudogene 5; ribosomal protein L4 pseudogene 4	Homo sapiens
647276	ribosomal protein L4; ribosomal protein L4 pseudogene 5; ribosomal protein L4 pseudogene 4	Homo sapiens
150684	copper metabolism (Murr1) domain containing 1	Homo sapiens
4502	metallothionein 2A	Homo sapiens
6594	SWI/SNF related, matrix associated, actin dependent regulator of chromatin, subfamily a, member 1	Homo sapiens
84128	WD repeat domain 75	Homo sapiens
7844	vacuolar protein sorting 24 homolog (S. cerevisiae); ring finger protein 103	Homo sapiens
6885	mitogen-activated protein kinase kinase kinase 7	Homo sapiens
11004	kinesin family member 2C	Homo sapiens
23312	Dmx-like 2	Homo sapiens
9797	TatD DNase domain containing 2	Homo sapiens
1455	casein kinase 1, gamma 2	Homo sapiens
79441	HAUS augmin-like complex, subunit 3	Homo sapiens
9817	kelch-like ECH-associated protein 1	Homo sapiens
92799	SH3KBP1 binding protein 1	Homo sapiens
23287	ATP/GTP binding protein 1	Homo sapiens
118426	loss of heterozygosity, 12, chromosomal region 1	Homo sapiens
6873	TAF2 RNA polymerase II, TATA box binding protein (TBP)-associated factor, 150kDa	Homo sapiens
80821	DDHD domain containing 1	Homo sapiens
9094	unc-119 homolog (C. elegans)	Homo sapiens
9369	neurexin 3	Homo sapiens
285741	similar to tumor protein, translationally-controlled 1; tumor protein, translationally-controlled 1	Homo sapiens
389787	similar to tumor protein, translationally-controlled 1; tumor protein, translationally-controlled 1	Homo sapiens
25843	MOB1, Mps One Binder kinase activator-like 3 (yeast)	Homo sapiens
4247	mannosyl (alpha-1,6-)-glycoprotein beta-1,2-N-acetylglucosaminyltransferase	Homo sapiens
2581	galactosylceramidase	Homo sapiens
79074	chromosome 2 open reading frame 49	Homo sapiens
29886	sorting nexin 8	Homo sapiens
26058	GRB10 interacting GYF protein 2	Homo sapiens
203228	chromosome 9 open reading frame 72	Homo sapiens
79465	UL16 binding protein 3	Homo sapiens
84196	ubiquitin specific peptidase 48	Homo sapiens
171568	polymerase (RNA) III (DNA directed) polypeptide H (22.9kD)	Homo sapiens

729991	hypothetical protein LOC729991	Homo sapiens
2975	general transcription factor IIIC, polypeptide 1, alpha 220kDa	Homo sapiens
100290337	similar to damage-specific DNA binding protein 1	Homo sapiens
414153	small nuclear ribonucleoprotein polypeptide E-like 1; small nuclear ribonucleoprotein polypeptide E; similar to hCG23490	Homo sapiens
100130109	small nuclear ribonucleoprotein polypeptide E-like 1; small nuclear ribonucleoprotein polypeptide E; similar to hCG23490	Homo sapiens
91833	WD repeat domain 20	Homo sapiens
9968	mediator complex subunit 12	Homo sapiens
8379	MAD1 mitotic arrest deficient-like 1 (yeast)	Homo sapiens
246175	CCR4-NOT transcription complex, subunit 6-like	Homo sapiens
5170	3-phosphoinositide dependent protein kinase-1	Homo sapiens
64320	ring finger protein 25	Homo sapiens
1288	collagen, type IV, alpha 6	Homo sapiens
643336	cell division cycle 42 (GTP binding protein, 25kDa); cell division cycle 42 pseudogene 2	Homo sapiens
4702	NADH dehydrogenase (ubiquinone) 1 alpha subcomplex, 8, 19kDa	Homo sapiens
220323	OAF homolog (Drosophila)	Homo sapiens
5558	primase, DNA, polypeptide 2 (58kDa)	Homo sapiens
5925	retinoblastoma 1	Homo sapiens
11031	RAB31, member RAS oncogene family	Homo sapiens
80308	FAD1 flavin adenine dinucleotide synthetase homolog (S. cerevisiae)	Homo sapiens
90379	chromosome 19 open reading frame 72	Homo sapiens
5261	phosphorylase kinase, gamma 2 (testis)	Homo sapiens
4723	NADH dehydrogenase (ubiquinone) flavoprotein 1, 51kDa	Homo sapiens
22926	activating transcription factor 6	Homo sapiens
10567	Rab acceptor 1 (prenylated)	Homo sapiens
81846	SET binding factor 2	Homo sapiens
55135	WD repeat containing, antisense to TP53	Homo sapiens
10915	transcription elongation regulator 1	Homo sapiens
100130902	thioredoxin reductase 1; hypothetical LOC100130902	Homo sapiens
55255	WD repeat domain 41	Homo sapiens
7093	tolloid-like 2	Homo sapiens
150472	COBW domain containing 2	Homo sapiens
645870	similar to barrier-to-autointegration factor; barrier to autointegration factor 1	Homo sapiens
114823	leukocyte receptor cluster (LRC) member 8	Homo sapiens
26268	F-box protein 9	Homo sapiens
6835	surfeit 2	Homo sapiens
53371	nucleoporin 54kDa	Homo sapiens
29924	epsin 1	Homo sapiens
55559	three prime repair exonuclease 2; HAUS augmin-like complex, subunit 7	Homo sapiens
11219	three prime repair exonuclease 2; HAUS augmin-like complex, subunit 7	Homo sapiens
9135	rabaptin, RAB GTPase binding effector protein 1	Homo sapiens
100131572	ribosomal protein S27 pseudogene 29; ribosomal protein S27 pseudogene 9; ribosomal protein S27 pseudogene 23; ribosomal protein S27 pseudogene 13; ribosomal protein S27; ribosomal protein S27 pseudogene 21; ribosomal protein S27 pseudogene 7; ribosomal protein S27 pseudogene 6; ribosomal protein S27 pseudogene 19	Homo sapiens
100132488	ribosomal protein S27 pseudogene 29; ribosomal protein S27 pseudogene 9; ribosomal protein S27 pseudogene 23; ribosomal protein S27 pseudogene 13; ribosomal protein S27; ribosomal protein S27 pseudogene 21; ribosomal protein S27 pseudogene 7; ribosomal protein S27 pseudogene 6; ribosomal protein S27 pseudogene 19	Homo sapiens
100131905	ribosomal protein S27 pseudogene 29; ribosomal protein S27 pseudogene 9; ribosomal protein S27 pseudogene 23; ribosomal protein S27 pseudogene 13; ribosomal protein S27; ribosomal protein S27 pseudogene 21; ribosomal protein S27 pseudogene 7; ribosomal protein S27 pseudogene 6; ribosomal protein S27 pseudogene 19	Homo sapiens
100130775	ribosomal protein S27 pseudogene 29; ribosomal protein S27 pseudogene 9; ribosomal protein S27 pseudogene 23; ribosomal protein S27 pseudogene 13; ribosomal protein S27; ribosomal protein S27 pseudogene 21; ribosomal protein S27 pseudogene 7; ribosomal protein S27 pseudogene 6; ribosomal protein S27 pseudogene 19	Homo sapiens
100130070	ribosomal protein S27 pseudogene 29; ribosomal protein S27 pseudogene 9; ribosomal protein S27 pseudogene 23; ribosomal protein S27 pseudogene 13; ribosomal protein S27; ribosomal protein S27 pseudogene 21; ribosomal protein S27 pseudogene 7; ribosomal protein S27 pseudogene 6; ribosomal protein S27 pseudogene 19	Homo sapiens
100129905	ribosomal protein S27 pseudogene 29; ribosomal protein S27 pseudogene 9; ribosomal protein S27 pseudogene 23; ribosomal protein S27 pseudogene 13; ribosomal protein S27; ribosomal protein S27 pseudogene 21; ribosomal protein S27 pseudogene 7; ribosomal protein S27 pseudogene 6; ribosomal protein S27 pseudogene 19	Homo sapiens
100132291	ribosomal protein S27 pseudogene 29; ribosomal protein S27 pseudogene 9; ribosomal protein S27 pseudogene 23; ribosomal protein S27 pseudogene 13; ribosomal protein S27; ribosomal protein S27 pseudogene 21; ribosomal protein S27 pseudogene 7; ribosomal protein S27 pseudogene 6; ribosomal protein S27 pseudogene 19	Homo sapiens

100131787	ribosomal protein S27 pseudogene 29; ribosomal protein S27 pseudogene 9; ribosomal protein S27 pseudogene 23; ribosomal protein S27 pseudogene 13; ribosomal protein S27; ribosomal protein S27 pseudogene 21; ribosomal protein S27 pseudogene 7; ribosomal protein S27 pseudogene 6; ribosomal protein S27 pseudogene 19	Homo sapiens
29945	anaphase promoting complex subunit 4	Homo sapiens
50859	sparc/osteonectin, cwcv and kazal-like domains proteoglycan (testican) 3	Homo sapiens
8241	RNA binding motif protein 10	Homo sapiens
57082	cancer susceptibility candidate 5	Homo sapiens
23767	fibronectin leucine rich transmembrane protein 3	Homo sapiens
51646	yippee-like 5 (Drosophila)	Homo sapiens
55330	cappuccino homolog (mouse)	Homo sapiens
10714	polymerase (DNA-directed), delta 3, accessory subunit	Homo sapiens
55631	leucine rich repeat containing 40	Homo sapiens
65083	nucleolar protein family 6 (RNA-associated)	Homo sapiens
388907	ribosomal protein L5 pseudogene 34; ribosomal protein L5 pseudogene 1; ribosomal protein L5	Homo sapiens
647436	ribosomal protein L5 pseudogene 34; ribosomal protein L5 pseudogene 1; ribosomal protein L5	Homo sapiens
400013	hypothetical gene supported by BC000665; t-complex 1	Homo sapiens
10413	Yes-associated protein 1, 65kDa	Homo sapiens
5296	phosphoinositide-3-kinase, regulatory subunit 2 (beta)	Homo sapiens
9639	Rho guanine nucleotide exchange factor (GEF) 10	Homo sapiens
7043	transforming growth factor, beta 3	Homo sapiens
54887	UHRF1 binding protein 1	Homo sapiens
8482	semaphorin 7A, GPI membrane anchor (John Milton Hagen blood group)	Homo sapiens
55051	chromosome 14 open reading frame 102	Homo sapiens
23765	interleukin 17 receptor A	Homo sapiens
60560	MAK10 homolog, amino-acid N-acetyltransferase subunit (S. cerevisiae)	Homo sapiens
348180	chromosome 16 open reading frame 84	Homo sapiens
51124	immediate early response 3 interacting protein 1	Homo sapiens
100128766	ribosomal protein L8; ribosomal protein L8 pseudogene 2	Homo sapiens
136227	EMI domain containing 2	Homo sapiens
27238	G patch domain and KOW motifs	Homo sapiens
4015	lysyl oxidase	Homo sapiens
8678	beclin 1, autophagy related	Homo sapiens
23242	cordon-bleu homolog (mouse)	Homo sapiens
8945	beta-transducin repeat containing	Homo sapiens
375298	ceramide kinase-like	Homo sapiens
23065	KIAA0090	Homo sapiens
730107	similar to Glycine cleavage system H protein, mitochondrial precursor; glycine cleavage system protein H (aminomethyl carrier); similar to Glycine cleavage system H protein, mitochondrial	Homo sapiens
654085	similar to Glycine cleavage system H protein, mitochondrial precursor; glycine cleavage system protein H (aminomethyl carrier); similar to Glycine cleavage system H protein, mitochondrial	Homo sapiens
729080	similar to Glycine cleavage system H protein, mitochondrial precursor; glycine cleavage system protein H (aminomethyl carrier); similar to Glycine cleavage system H protein, mitochondrial	Homo sapiens
2653	similar to Glycine cleavage system H protein, mitochondrial precursor; glycine cleavage system protein H (aminomethyl carrier); similar to Glycine cleavage system H protein, mitochondrial	Homo sapiens
9070	ash2 (absent, small, or homeotic)-like (Drosophila)	Homo sapiens
1107	chromodomain helicase DNA binding protein 3	Homo sapiens
147179	WAS/WASL interacting protein family, member 2	Homo sapiens
245711	protein phosphatase 1, catalytic subunit, beta isoform; speedy homolog A (Xenopus laevis)	Homo sapiens
26130	GTPase activating protein and VPS9 domains 1	Homo sapiens
30827	CXXC finger 1 (PHD domain)	Homo sapiens
9612	nuclear receptor co-repressor 2	Homo sapiens
10642	insulin-like growth factor 2 mRNA binding protein 1	Homo sapiens
399804	nucleophosmin 1 (nucleolar phosphoprotein B23, numatrin) pseudogene 21; hypothetical LOC100131044; similar to nucleophosmin 1; nucleophosmin (nucleolar phosphoprotein B23, numatrin)	Homo sapiens
100129237	nucleophosmin 1 (nucleolar phosphoprotein B23, numatrin) pseudogene 21; hypothetical LOC100131044; similar to nucleophosmin 1; nucleophosmin (nucleolar phosphoprotein B23, numatrin)	Homo sapiens
440577	nucleophosmin 1 (nucleolar phosphoprotein B23, numatrin) pseudogene 21; hypothetical LOC100131044; similar to nucleophosmin 1; nucleophosmin (nucleolar phosphoprotein B23, numatrin)	Homo sapiens
729686	nucleophosmin 1 (nucleolar phosphoprotein B23, numatrin) pseudogene 21; hypothetical LOC100131044; similar to nucleophosmin 1; nucleophosmin (nucleolar phosphoprotein B23, numatrin)	Homo sapiens
100131044	nucleophosmin 1 (nucleolar phosphoprotein B23, numatrin) pseudogene 21; hypothetical LOC100131044; similar to nucleophosmin 1; nucleophosmin (nucleolar phosphoprotein B23, numatrin)	Homo sapiens
729342	nucleophosmin 1 (nucleolar phosphoprotein B23, numatrin) pseudogene 21; hypothetical LOC100131044; similar to nucleophosmin 1; nucleophosmin (nucleolar phosphoprotein B23, numatrin)	Homo sapiens

729992	similar to heat shock 70kD protein binding protein; suppression of tumorigenicity 13 (colon carcinoma) (Hsp70 interacting protein)	Homo sapiens
344328	similar to heat shock 70kD protein binding protein; suppression of tumorigenicity 13 (colon carcinoma) (Hsp70 interacting protein)	Homo sapiens
388344	ribosomal protein L13 pseudogene 12; ribosomal protein L13	Homo sapiens
50804	myelin expression factor 2	Homo sapiens
84872	zinc finger CCCH-type containing 10	Homo sapiens
1730	diaphanous homolog 2 (Drosophila)	Homo sapiens
1616	death-domain associated protein	Homo sapiens
201965	RWD domain containing 4A	Homo sapiens
51441	YTH domain family, member 2	Homo sapiens
79902	nucleoporin 85kDa	Homo sapiens
11152	WD repeat domain 45	Homo sapiens
305	annexin A2 pseudogene 3; annexin A2; annexin A2 pseudogene 1	Homo sapiens
51362	cell division cycle 40 homolog (S. cerevisiae)	Homo sapiens
53981	cleavage and polyadenylation specific factor 2, 100kDa	Homo sapiens
58527	chromosome 6 open reading frame 115	Homo sapiens
81556	chromosome 15 open reading frame 44	Homo sapiens
253943	YTH domain family, member 3	Homo sapiens
84343	Hermansky-Pudlak syndrome 3	Homo sapiens
10498	coactivator-associated arginine methyltransferase 1	Homo sapiens
57680	chromodomain helicase DNA binding protein 8	Homo sapiens
89891	WD repeat domain 34	Homo sapiens
653506	meteorin, glial cell differentiation regulator-like; similar to meteorin, glial cell differentiation regulator-like	Homo sapiens
642513	similar to Potassium channel tetramerisation domain containing 9; potassium channel tetramerisation domain containing 9; similar to potassium channel tetramerisation domain containing 9	Homo sapiens
647013	similar to Potassium channel tetramerisation domain containing 9; potassium channel tetramerisation domain containing 9; similar to potassium channel tetramerisation domain containing 9	Homo sapiens
54793	similar to Potassium channel tetramerisation domain containing 9; potassium channel tetramerisation domain containing 9; similar to potassium channel tetramerisation domain containing 9	Homo sapiens
2146	enhancer of zeste homolog 2 (Drosophila)	Homo sapiens
9862	mediator complex subunit 24	Homo sapiens
60681	FK506 binding protein 10, 65 kDa	Homo sapiens
57186	chromosome 20 open reading frame 74	Homo sapiens
9093	DnaJ (Hsp40) homolog, subfamily A, member 3	Homo sapiens
7189	TNF receptor-associated factor 6	Homo sapiens
23649	polymerase (DNA directed), alpha 2 (70kD subunit)	Homo sapiens
23404	exosome component 2	Homo sapiens
64785	GINS complex subunit 3 (Psf3 homolog)	Homo sapiens
770	carbonic anhydrase XI	Homo sapiens
3161	hyaluronan-mediated motility receptor (RHAMM)	Homo sapiens
100132364	NIN1/RPN12 binding protein 1 homolog (S. cerevisiae); hypothetical LOC100132364	Homo sapiens
28987	NIN1/RPN12 binding protein 1 homolog (S. cerevisiae); hypothetical LOC100132364	Homo sapiens
8975	ubiquitin specific peptidase 13 (isopeptidase T-3)	Homo sapiens
51019	coiled-coil domain containing 53	Homo sapiens
151176	family with sequence similarity 132, member B	Homo sapiens
22879	MON1 homolog B (yeast)	Homo sapiens
2009	echinoderm microtubule associated protein like 1	Homo sapiens
84315	MON1 homolog A (yeast)	Homo sapiens
55758	REST corepressor 3	Homo sapiens
23264	zinc finger CCCH-type containing 7B	Homo sapiens
51008	activating signal cointegrator 1 complex subunit 1	Homo sapiens
24139	echinoderm microtubule associated protein like 2	Homo sapiens
54442	potassium channel tetramerisation domain containing 5	Homo sapiens
3300	DnaJ (Hsp40) homolog, subfamily B, member 2	Homo sapiens
51361	hook homolog 1 (Drosophila)	Homo sapiens
5210	6-phosphofructo-2-kinase/fructose-2,6-biphosphatase 4	Homo sapiens
25831	HECT domain containing 1	Homo sapiens
55704	coiled-coil domain containing 88A	Homo sapiens
56896	dihydropyrimidinase-like 5	Homo sapiens
644390	similar to heterogeneous nuclear ribonucleoprotein L-like; heterogeneous nuclear ribonucleoprotein L	Homo sapiens
7158	tumor protein p53 binding protein 1	Homo sapiens
7248	tuberous sclerosis 1	Homo sapiens
409	arrestin, beta 2	Homo sapiens
10270	A kinase (PRKA) anchor protein 8	Homo sapiens
55090	mediator complex subunit 9	Homo sapiens
26098	chromosome 10 open reading frame 137	Homo sapiens
9330	general transcription factor IIIC, polypeptide 3, 102kDa	Homo sapiens
9455	homer homolog 2 (Drosophila)	Homo sapiens
4350	N-methylpurine-DNA glycosylase	Homo sapiens
392437	similar to ferritin, light polypeptide; ferritin, light polypeptide	Homo sapiens
741	zinc finger, HIT type 2	Homo sapiens
9701	SAPS domain family, member 2	Homo sapiens

642546	hexokinase 2 pseudogene; hexokinase 2	Homo sapiens
3099	hexokinase 2 pseudogene; hexokinase 2	Homo sapiens
2175	Fanconi anemia, complementation group A	Homo sapiens
55905	ring finger protein 114	Homo sapiens
4849	CCR4-NOT transcription complex, subunit 3	Homo sapiens
55297	coiled-coil domain containing 91	Homo sapiens
23557	SNAP-associated protein	Homo sapiens
54662	TBC1 domain family, member 13	Homo sapiens
57679	amyotrophic lateral sclerosis 2 (juvenile)	Homo sapiens
5321	phospholipase A2, group IVA (cytosolic, calcium-dependent)	Homo sapiens
79658	Rho GTPase activating protein 10	Homo sapiens
28981	intraflagellar transport 81 homolog (Chlamydomonas)	Homo sapiens
23276	kelch-like 18 (Drosophila)	Homo sapiens
729608	brix domain containing 1 pseudogene; brix domain containing 1	Homo sapiens
84154	brix domain containing 1 pseudogene; brix domain containing 1	Homo sapiens
23635	single-stranded DNA binding protein 2	Homo sapiens
5439	polymerase (RNA) II (DNA directed) polypeptide J, 13.3kDa	Homo sapiens
23213	sulfatase 1	Homo sapiens
84172	polymerase (RNA) I polypeptide B, 128kDa	Homo sapiens
57560	intraflagellar transport 80 homolog (Chlamydomonas)	Homo sapiens
26472	protein phosphatase 1, regulatory (inhibitor) subunit 14B	Homo sapiens
55907	cytidine monophosphate N-acetylneuraminic acid synthetase	Homo sapiens
114883	oxysterol binding protein-like 9	Homo sapiens
84864	MYC induced nuclear antigen	Homo sapiens
9818	nucleoporin like 1	Homo sapiens
9820	cullin 7	Homo sapiens
92906	heterogeneous nuclear ribonucleoprotein L-like	Homo sapiens
149986	LSM14B, SCD6 homolog B (S. cerevisiae)	Homo sapiens
197131	ubiquitin protein ligase E3 component n-recognin 1	Homo sapiens
5130	phosphate cytidylyltransferase 1, choline, alpha	Homo sapiens
116988	ArfGAP with GTPase domain, ankyrin repeat and PH domain 3	Homo sapiens
4320	matrix metallopeptidase 11 (stromelysin 3)	Homo sapiens
7756	zinc finger protein 207	Homo sapiens
79888	lysophosphatidylcholine acyltransferase 1	Homo sapiens
91298	chromosome 12 open reading frame 29	Homo sapiens
64792	RAB, member RAS oncogene family-like 5	Homo sapiens
64802	nicotinamide nucleotide adenylyltransferase 1	Homo sapiens
56259	catenin, beta like 1	Homo sapiens
10516	fibulin 5	Homo sapiens
64976	mitochondrial ribosomal protein L40	Homo sapiens
51372	coiled-coil domain containing 72; hypothetical LOC729973; hypothetical LOC728416	Homo sapiens
728416	coiled-coil domain containing 72; hypothetical LOC729973; hypothetical LOC728416	Homo sapiens
729973	coiled-coil domain containing 72; hypothetical LOC729973; hypothetical LOC728416	Homo sapiens
1106	chromodomain helicase DNA binding protein 2	Homo sapiens
55027	HEAT repeat containing 3	Homo sapiens
55667	DENN/MADD domain containing 4C	Homo sapiens
29893	PSMC3 interacting protein	Homo sapiens
645381	similar to transducin-like enhancer of split 1 (E(sp1) homolog, Drosophila); transducin-like enhancer of split 1 (E(sp1) homolog, Drosophila)	Homo sapiens
7088	similar to transducin-like enhancer of split 1 (E(sp1) homolog, Drosophila); transducin-like enhancer of split 1 (E(sp1) homolog, Drosophila)	Homo sapiens
3908	laminin, alpha 2	Homo sapiens
399511	transcription elongation factor A (SII), 1 pseudogene 2; transcription elongation factor A (SII), 1	Homo sapiens
1952	cadherin, EGF LAG seven-pass G-type receptor 2 (flamingo homolog, Drosophila)	Homo sapiens
4355	membrane protein, palmitoylated 2 (MAGUK p55 subfamily member 2)	Homo sapiens
9924	PAN2 poly(A) specific ribonuclease subunit homolog (S. cerevisiae)	Homo sapiens
661	polymerase (RNA) III (DNA directed) polypeptide D, 44kDa	Homo sapiens
285590	SH3 and PX domains 2B	Homo sapiens
389842	similar to RAN binding protein 1; RAN binding protein 1	Homo sapiens
727803	similar to RAN binding protein 1; RAN binding protein 1	Homo sapiens
137133	chromosome 8 open reading frame 62; phosphoserine aminotransferase 1	Homo sapiens
84312	breast cancer metastasis-suppressor 1-like	Homo sapiens
51530	zinc finger, C3HC-type containing 1	Homo sapiens
130617	zinc finger, AN1-type domain 2B	Homo sapiens
112939	nucleus accumbens associated 1, BEN and BTB (POZ) domain containing	Homo sapiens
55656	integrator complex subunit 8	Homo sapiens
23411	sirtuin (silent mating type information regulation 2 homolog) 1 (S. cerevisiae)	Homo sapiens
729389	ribosomal protein S6 pseudogene 25; ribosomal protein S6; ribosomal protein S6 pseudogene 1	Homo sapiens
440086	ribosomal protein S6 pseudogene 25; ribosomal protein S6; ribosomal protein S6 pseudogene 1	Homo sapiens
55183	RAP1 interacting factor homolog (yeast)	Homo sapiens
547	kinesin family member 1A	Homo sapiens
51074	APAF1 interacting protein; similar to APAF1 interacting protein	Homo sapiens

100128086	APAF1 interacting protein; similar to APAF1 interacting protein	Homo sapiens
4534	myotubularin 1	Homo sapiens
57673	BEN domain containing 3	Homo sapiens
55781	RIO kinase 2 (yeast)	Homo sapiens
55031	ubiquitin specific peptidase 47	Homo sapiens
337876	chondroitin sulfate synthase 3	Homo sapiens
645979	ribosomal protein S26 pseudogene 38; ribosomal protein S26 pseudogene 39; ribosomal protein S26 pseudogene 35; ribosomal protein S26 pseudogene 31; ribosomal protein S26 pseudogene 20; ribosomal protein S26 pseudogene 54; ribosomal protein S26 pseudogene 2; ribosomal protein S26 pseudogene 53; ribosomal protein S26 pseudogene 25; ribosomal protein S26 pseudogene 50; ribosomal protein S26 pseudogene 6; ribosomal protein S26 pseudogene 8; ribosomal protein S26	Homo sapiens
100131971	ribosomal protein S26 pseudogene 38; ribosomal protein S26 pseudogene 39; ribosomal protein S26 pseudogene 35; ribosomal protein S26 pseudogene 31; ribosomal protein S26 pseudogene 20; ribosomal protein S26 pseudogene 54; ribosomal protein S26 pseudogene 2; ribosomal protein S26 pseudogene 53; ribosomal protein S26 pseudogene 25; ribosomal protein S26 pseudogene 50; ribosomal protein S26 pseudogene 6; ribosomal protein S26 pseudogene 8; ribosomal protein S26	Homo sapiens
646753	ribosomal protein S26 pseudogene 38; ribosomal protein S26 pseudogene 39; ribosomal protein S26 pseudogene 35; ribosomal protein S26 pseudogene 31; ribosomal protein S26 pseudogene 20; ribosomal protein S26 pseudogene 54; ribosomal protein S26 pseudogene 2; ribosomal protein S26 pseudogene 53; ribosomal protein S26 pseudogene 25; ribosomal protein S26 pseudogene 50; ribosomal protein S26 pseudogene 6; ribosomal protein S26 pseudogene 8; ribosomal protein S26	Homo sapiens
644934	ribosomal protein S26 pseudogene 38; ribosomal protein S26 pseudogene 39; ribosomal protein S26 pseudogene 35; ribosomal protein S26 pseudogene 31; ribosomal protein S26 pseudogene 20; ribosomal protein S26 pseudogene 54; ribosomal protein S26 pseudogene 2; ribosomal protein S26 pseudogene 53; ribosomal protein S26 pseudogene 25; ribosomal protein S26 pseudogene 50; ribosomal protein S26 pseudogene 6; ribosomal protein S26 pseudogene 8; ribosomal protein S26	Homo sapiens
441377	ribosomal protein S26 pseudogene 38; ribosomal protein S26 pseudogene 39; ribosomal protein S26 pseudogene 35; ribosomal protein S26 pseudogene 31; ribosomal protein S26 pseudogene 20; ribosomal protein S26 pseudogene 54; ribosomal protein S26 pseudogene 2; ribosomal protein S26 pseudogene 53; ribosomal protein S26 pseudogene 25; ribosomal protein S26 pseudogene 50; ribosomal protein S26 pseudogene 6; ribosomal protein S26 pseudogene 8; ribosomal protein S26	Homo sapiens
100128168	ribosomal protein S26 pseudogene 38; ribosomal protein S26 pseudogene 39; ribosomal protein S26 pseudogene 35; ribosomal protein S26 pseudogene 31; ribosomal protein S26 pseudogene 20; ribosomal protein S26 pseudogene 54; ribosomal protein S26 pseudogene 2; ribosomal protein S26 pseudogene 53; ribosomal protein S26 pseudogene 25; ribosomal protein S26 pseudogene 50; ribosomal protein S26 pseudogene 6; ribosomal protein S26 pseudogene 8; ribosomal protein S26	Homo sapiens
728937	ribosomal protein S26 pseudogene 38; ribosomal protein S26 pseudogene 39; ribosomal protein S26 pseudogene 35; ribosomal protein S26 pseudogene 31; ribosomal protein S26 pseudogene 20; ribosomal protein S26 pseudogene 54; ribosomal protein S26 pseudogene 2; ribosomal protein S26 pseudogene 53; ribosomal protein S26 pseudogene 25; ribosomal protein S26 pseudogene 50; ribosomal protein S26 pseudogene 6; ribosomal protein S26 pseudogene 8; ribosomal protein S26	Homo sapiens
644166	ribosomal protein S26 pseudogene 38; ribosomal protein S26 pseudogene 39; ribosomal protein S26 pseudogene 35; ribosomal protein S26 pseudogene 31; ribosomal protein S26 pseudogene 20; ribosomal protein S26 pseudogene 54; ribosomal protein S26 pseudogene 2; ribosomal protein S26 pseudogene 53; ribosomal protein S26 pseudogene 25; ribosomal protein S26 pseudogene 50; ribosomal protein S26 pseudogene 6; ribosomal protein S26 pseudogene 8; ribosomal protein S26	Homo sapiens
100129552	ribosomal protein S26 pseudogene 38; ribosomal protein S26 pseudogene 39; ribosomal protein S26 pseudogene 35; ribosomal protein S26 pseudogene 31; ribosomal protein S26 pseudogene 20; ribosomal protein S26 pseudogene 54; ribosomal protein S26 pseudogene 2; ribosomal protein S26 pseudogene 53; ribosomal protein S26 pseudogene 25; ribosomal protein S26 pseudogene 50; ribosomal protein S26 pseudogene 6; ribosomal protein S26 pseudogene 8; ribosomal protein S26	Homo sapiens
392256	ribosomal protein S26 pseudogene 38; ribosomal protein S26 pseudogene 39; ribosomal protein S26 pseudogene 35; ribosomal protein S26 pseudogene 31; ribosomal protein S26 pseudogene 20; ribosomal protein S26 pseudogene 54; ribosomal protein S26 pseudogene 2; ribosomal protein S26 pseudogene 53; ribosomal protein S26 pseudogene 25; ribosomal protein S26 pseudogene 50; ribosomal protein S26 pseudogene 6; ribosomal protein S26 pseudogene 8; ribosomal protein S26	Homo sapiens

728823	ribosomal protein S26 pseudogene 38; ribosomal protein S26 pseudogene 39; ribosomal protein S26 pseudogene 35; ribosomal protein S26 pseudogene 31; ribosomal protein S26 pseudogene 20; ribosomal protein S26 pseudogene 54; ribosomal protein S26 pseudogene 2; ribosomal protein S26 pseudogene 53; ribosomal protein S26 pseudogene 25; ribosomal protein S26 pseudogene 50; ribosomal protein S26 pseudogene 6; ribosomal protein S26 pseudogene 8; ribosomal protein S26	Homo sapiens
644191	ribosomal protein S26 pseudogene 38; ribosomal protein S26 pseudogene 39; ribosomal protein S26 pseudogene 35; ribosomal protein S26 pseudogene 31; ribosomal protein S26 pseudogene 20; ribosomal protein S26 pseudogene 54; ribosomal protein S26 pseudogene 2; ribosomal protein S26 pseudogene 53; ribosomal protein S26 pseudogene 25; ribosomal protein S26 pseudogene 50; ribosomal protein S26 pseudogene 6; ribosomal protein S26 pseudogene 8; ribosomal protein S26	Homo sapiens
10087	collagen, type IV, alpha 3 (Goodpasture antigen) binding protein	Homo sapiens
11143	MYST histone acetyltransferase 2; similar to MYST histone acetyltransferase 2	Homo sapiens
648927	MYST histone acetyltransferase 2; similar to MYST histone acetyltransferase 2	Homo sapiens
23098	sterile alpha and TIR motif containing 1	Homo sapiens
90459	exoribonuclease 1	Homo sapiens
10200	M-phase phosphoprotein 6	Homo sapiens
115106	HAUS augmin-like complex, subunit 1	Homo sapiens
646780	phosphorylase kinase, alpha 1 pseudogene 1; phosphorylase kinase, alpha 1 (muscle)	Homo sapiens
8914	timeless homolog (Drosophila)	Homo sapiens
6311	ataxin 2	Homo sapiens
5073	poly(A)-specific ribonuclease (deadenylation nuclease)	Homo sapiens
23310	non-SMC condensin II complex, subunit D3	Homo sapiens
55602	CDKN2A interacting protein	Homo sapiens
285643	kinesin family member 4B; kinesin family member 4A	Homo sapiens
24147	four jointed box 1 (Drosophila)	Homo sapiens
128240	apolipoprotein A-I binding protein	Homo sapiens
652147	similar to U5 snRNP-specific protein, 200 kDa; small nuclear ribonucleoprotein 200kDa (U5)	Homo sapiens
8925	hect (homologous to the E6-AP (UBE3A) carboxyl terminus) domain and RCC1 (CHC1)-like domain (RLD) 1	Homo sapiens
23192	ATG4 autophagy related 4 homolog B (S. cerevisiae)	Homo sapiens
5074	PRKC, apoptosis, WT1, regulator	Homo sapiens
8672	eukaryotic translation initiation factor 4 gamma, 3	Homo sapiens
8646	chordin	Homo sapiens
23259	DDHD domain containing 2	Homo sapiens
8940	topoisomerase (DNA) III beta	Homo sapiens
84930	microtubule associated serine/threonine kinase-like	Homo sapiens
55671	SMEK homolog 1, suppressor of mek1 (Dictyostelium)	Homo sapiens
10605	poly(A) binding protein interacting protein 1; similar to poly(A) binding protein interacting protein 1	Homo sapiens
645139	poly(A) binding protein interacting protein 1; similar to poly(A) binding protein interacting protein 1	Homo sapiens
64084	calsyntenin 2	Homo sapiens
54930	HAUS augmin-like complex, subunit 4	Homo sapiens
60625	DEAH (Asp-Glu-Ala-His) box polypeptide 35	Homo sapiens
79625	chromosome 4 open reading frame 31	Homo sapiens
54797	mediator complex subunit 18	Homo sapiens
25938	HEAT repeat containing 5A	Homo sapiens
100133690	hypothetical protein LOC100133690; activated leukocyte cell adhesion molecule	Homo sapiens
51592	tripartite motif-containing 33	Homo sapiens
28964	G protein-coupled receptor kinase interacting ArfGAP 1	Homo sapiens
9953	heparan sulfate (glucosamine) 3-O-sulfotransferase 3B1	Homo sapiens
1964	eukaryotic translation initiation factor 1A, X-linked	Homo sapiens
10565	ADP-ribosylation factor guanine nucleotide-exchange factor 1(brefeldin A-inhibited)	Homo sapiens
10687	paraneoplastic antigen MA2	Homo sapiens
112950	mediator complex subunit 8	Homo sapiens
85451	unkempt homolog (Drosophila)	Homo sapiens
79801	SHC SH2-domain binding protein 1	Homo sapiens
22985	apoptotic chromatin condensation inducer 1	Homo sapiens
55175	kelch-like 11 (Drosophila)	Homo sapiens
115098	coiled-coil domain containing 124	Homo sapiens
55157	aspartyl-tRNA synthetase 2, mitochondrial	Homo sapiens
54870	glutamine-rich 1	Homo sapiens
3661	interferon regulatory factor 3	Homo sapiens
64419	myotubularin related protein 14	Homo sapiens
2976	general transcription factor IIIC, polypeptide 2, beta 110kDa	Homo sapiens
9100	ubiquitin specific peptidase 10	Homo sapiens
9931	helicase with zinc finger	Homo sapiens
9394	heparan sulfate 6-O-sulfotransferase 1	Homo sapiens
1388	activating transcription factor 6 beta	Homo sapiens
7072	TIA1 cytotoxic granule-associated RNA binding protein	Homo sapiens

23154	neurochondrin	Homo sapiens
79691	queuine tRNA-ribosyltransferase domain containing 1	Homo sapiens
10489	leucine rich repeat containing 41	Homo sapiens
100130819	RAB6C, member RAS oncogene family; RAB6A, member RAS oncogene family; hypothetical LOC100130819; RAB6C-like	Homo sapiens
1659	DEAH (Asp-Glu-Ala-His) box polypeptide 8	Homo sapiens
171019	ADAM metallopeptidase with thrombospondin type 1 motif, 19	Homo sapiens
731688	ribosomal protein L21 pseudogene 134; ribosomal protein L21 pseudogene 80; ribosomal protein L21 pseudogene 20; ribosomal protein L21 pseudogene 46; ribosomal protein L21 pseudogene 45; ribosomal protein L21 pseudogene 131; ribosomal protein L21 pseudogene 16; ribosomal protein L21 pseudogene 53; ribosomal protein L21 pseudogene 120; ribosomal protein L21 pseudogene 37; ribosomal protein L21 pseudogene 93; ribosomal protein L21 pseudogene 39; ribosomal protein L21 pseudogene 29; ribosomal protein L21 pseudogene 28; ribosomal protein L21 pseudogene 14; ribosomal protein L21 pseudogene 98; ribosomal protein L21 pseudogene 105; ribosomal protein L21 pseudogene 87; ribosomal protein L21 pseudogene 128; ribosomal protein L21 pseudogene 69; ribosomal protein L21 pseudogene 97; ribosomal protein L21; ribosomal protein L21 pseudogene 119; ribosomal protein L21 pseudogene 125	Homo sapiens
100129685	ribosomal protein L21 pseudogene 134; ribosomal protein L21 pseudogene 80; ribosomal protein L21 pseudogene 20; ribosomal protein L21 pseudogene 46; ribosomal protein L21 pseudogene 45; ribosomal protein L21 pseudogene 131; ribosomal protein L21 pseudogene 16; ribosomal protein L21 pseudogene 53; ribosomal protein L21 pseudogene 120; ribosomal protein L21 pseudogene 37; ribosomal protein L21 pseudogene 93; ribosomal protein L21 pseudogene 39; ribosomal protein L21 pseudogene 29; ribosomal protein L21 pseudogene 28; ribosomal protein L21 pseudogene 14; ribosomal protein L21 pseudogene 98; ribosomal protein L21 pseudogene 105; ribosomal protein L21 pseudogene 87; ribosomal protein L21 pseudogene 128; ribosomal protein L21 pseudogene 69; ribosomal protein L21 pseudogene 97; ribosomal protein L21; ribosomal protein L21 pseudogene 119; ribosomal protein L21 pseudogene 125	Homo sapiens
729208	ribosomal protein L21 pseudogene 134; ribosomal protein L21 pseudogene 80; ribosomal protein L21 pseudogene 20; ribosomal protein L21 pseudogene 46; ribosomal protein L21 pseudogene 45; ribosomal protein L21 pseudogene 131; ribosomal protein L21 pseudogene 16; ribosomal protein L21 pseudogene 53; ribosomal protein L21 pseudogene 120; ribosomal protein L21 pseudogene 37; ribosomal protein L21 pseudogene 93; ribosomal protein L21 pseudogene 39; ribosomal protein L21 pseudogene 29; ribosomal protein L21 pseudogene 28; ribosomal protein L21 pseudogene 14; ribosomal protein L21 pseudogene 98; ribosomal protein L21 pseudogene 105; ribosomal protein L21 pseudogene 87; ribosomal protein L21 pseudogene 128; ribosomal protein L21 pseudogene 69; ribosomal protein L21 pseudogene 97; ribosomal protein L21; ribosomal protein L21 pseudogene 119; ribosomal protein L21 pseudogene 125	Homo sapiens
727821	ribosomal protein L21 pseudogene 134; ribosomal protein L21 pseudogene 80; ribosomal protein L21 pseudogene 20; ribosomal protein L21 pseudogene 46; ribosomal protein L21 pseudogene 45; ribosomal protein L21 pseudogene 131; ribosomal protein L21 pseudogene 16; ribosomal protein L21 pseudogene 53; ribosomal protein L21 pseudogene 120; ribosomal protein L21 pseudogene 37; ribosomal protein L21 pseudogene 93; ribosomal protein L21 pseudogene 39; ribosomal protein L21 pseudogene 29; ribosomal protein L21 pseudogene 28; ribosomal protein L21 pseudogene 14; ribosomal protein L21 pseudogene 98; ribosomal protein L21 pseudogene 105; ribosomal protein L21 pseudogene 87; ribosomal protein L21 pseudogene 128; ribosomal protein L21 pseudogene 69; ribosomal protein L21 pseudogene 97; ribosomal protein L21; ribosomal protein L21 pseudogene 119; ribosomal protein L21 pseudogene 125	Homo sapiens
653665	ribosomal protein L21 pseudogene 134; ribosomal protein L21 pseudogene 80; ribosomal protein L21 pseudogene 20; ribosomal protein L21 pseudogene 46; ribosomal protein L21 pseudogene 45; ribosomal protein L21 pseudogene 131; ribosomal protein L21 pseudogene 16; ribosomal protein L21 pseudogene 53; ribosomal protein L21 pseudogene 120; ribosomal protein L21 pseudogene 37; ribosomal protein L21 pseudogene 93; ribosomal protein L21 pseudogene 39; ribosomal protein L21 pseudogene 29; ribosomal protein L21 pseudogene 28; ribosomal protein L21 pseudogene 14; ribosomal protein L21 pseudogene 98; ribosomal protein L21 pseudogene 105; ribosomal protein L21 pseudogene 87; ribosomal protein L21 pseudogene 128; ribosomal protein L21 pseudogene 69; ribosomal protein L21 pseudogene 97; ribosomal protein L21; ribosomal protein L21 pseudogene 119; ribosomal protein L21 pseudogene 125	Homo sapiens
729402	ribosomal protein L21 pseudogene 134; ribosomal protein L21 pseudogene 80; ribosomal protein L21 pseudogene 20; ribosomal protein L21 pseudogene 46; ribosomal protein L21 pseudogene 45; ribosomal protein L21 pseudogene 131; ribosomal protein L21 pseudogene 16; ribosomal protein L21 pseudogene 53; ribosomal protein L21 pseudogene 120; ribosomal protein L21 pseudogene 37; ribosomal protein L21 pseudogene 93; ribosomal protein L21 pseudogene 39; ribosomal protein L21 pseudogene 29; ribosomal protein L21 pseudogene 28; ribosomal protein L21 pseudogene 14; ribosomal protein L21 pseudogene 98; ribosomal protein L21 pseudogene 105; ribosomal protein L21 pseudogene 87; ribosomal protein L21 pseudogene 128; ribosomal protein L21 pseudogene 69; ribosomal protein L21 pseudogene 97; ribosomal protein L21; ribosomal protein L21 pseudogene 119; ribosomal protein L21 pseudogene 125	Homo sapiens

653737	ribosomal protein L21 pseudogene 134; ribosomal protein L21 pseudogene 80; ribosomal protein L21 pseudogene 20; ribosomal protein L21 pseudogene 46; ribosomal protein L21 pseudogene 45; ribosomal protein L21 pseudogene 131; ribosomal protein L21 pseudogene 16; ribosomal protein L21 pseudogene 53; ribosomal protein L21 pseudogene 120; ribosomal protein L21 pseudogene 37; ribosomal protein L21 pseudogene 93; ribosomal protein L21 pseudogene 39; ribosomal protein L21 pseudogene 29; ribosomal protein L21 pseudogene 28; ribosomal protein L21 pseudogene 14; ribosomal protein L21 pseudogene 98; ribosomal protein L21 pseudogene 105; ribosomal protein L21 pseudogene 87; ribosomal protein L21 pseudogene 128; ribosomal protein L21 pseudogene 69; ribosomal protein L21 pseudogene 97; ribosomal protein L21; ribosomal protein L21 pseudogene 119; ribosomal protein L21 pseudogene 125	Homo sapiens
387753	ribosomal protein L21 pseudogene 134; ribosomal protein L21 pseudogene 80; ribosomal protein L21 pseudogene 20; ribosomal protein L21 pseudogene 46; ribosomal protein L21 pseudogene 45; ribosomal protein L21 pseudogene 131; ribosomal protein L21 pseudogene 16; ribosomal protein L21 pseudogene 53; ribosomal protein L21 pseudogene 120; ribosomal protein L21 pseudogene 37; ribosomal protein L21 pseudogene 93; ribosomal protein L21 pseudogene 39; ribosomal protein L21 pseudogene 29; ribosomal protein L21 pseudogene 28; ribosomal protein L21 pseudogene 14; ribosomal protein L21 pseudogene 98; ribosomal protein L21 pseudogene 105; ribosomal protein L21 pseudogene 87; ribosomal protein L21 pseudogene 128; ribosomal protein L21 pseudogene 69; ribosomal protein L21 pseudogene 97; ribosomal protein L21; ribosomal protein L21 pseudogene 119; ribosomal protein L21 pseudogene 125	Homo sapiens
728782	ribosomal protein L21 pseudogene 134; ribosomal protein L21 pseudogene 80; ribosomal protein L21 pseudogene 20; ribosomal protein L21 pseudogene 46; ribosomal protein L21 pseudogene 45; ribosomal protein L21 pseudogene 131; ribosomal protein L21 pseudogene 16; ribosomal protein L21 pseudogene 53; ribosomal protein L21 pseudogene 120; ribosomal protein L21 pseudogene 37; ribosomal protein L21 pseudogene 93; ribosomal protein L21 pseudogene 39; ribosomal protein L21 pseudogene 29; ribosomal protein L21 pseudogene 28; ribosomal protein L21 pseudogene 14; ribosomal protein L21 pseudogene 98; ribosomal protein L21 pseudogene 105; ribosomal protein L21 pseudogene 87; ribosomal protein L21 pseudogene 128; ribosomal protein L21 pseudogene 69; ribosomal protein L21 pseudogene 97; ribosomal protein L21; ribosomal protein L21 pseudogene 119; ribosomal protein L21 pseudogene 125	Homo sapiens
653156	ribosomal protein L21 pseudogene 134; ribosomal protein L21 pseudogene 80; ribosomal protein L21 pseudogene 20; ribosomal protein L21 pseudogene 46; ribosomal protein L21 pseudogene 45; ribosomal protein L21 pseudogene 131; ribosomal protein L21 pseudogene 16; ribosomal protein L21 pseudogene 53; ribosomal protein L21 pseudogene 120; ribosomal protein L21 pseudogene 37; ribosomal protein L21 pseudogene 93; ribosomal protein L21 pseudogene 39; ribosomal protein L21 pseudogene 29; ribosomal protein L21 pseudogene 28; ribosomal protein L21 pseudogene 14; ribosomal protein L21 pseudogene 98; ribosomal protein L21 pseudogene 105; ribosomal protein L21 pseudogene 87; ribosomal protein L21 pseudogene 128; ribosomal protein L21 pseudogene 69; ribosomal protein L21 pseudogene 97; ribosomal protein L21; ribosomal protein L21 pseudogene 119; ribosomal protein L21 pseudogene 125	Homo sapiens
653079	ribosomal protein L21 pseudogene 134; ribosomal protein L21 pseudogene 80; ribosomal protein L21 pseudogene 20; ribosomal protein L21 pseudogene 46; ribosomal protein L21 pseudogene 45; ribosomal protein L21 pseudogene 131; ribosomal protein L21 pseudogene 16; ribosomal protein L21 pseudogene 53; ribosomal protein L21 pseudogene 120; ribosomal protein L21 pseudogene 37; ribosomal protein L21 pseudogene 93; ribosomal protein L21 pseudogene 39; ribosomal protein L21 pseudogene 29; ribosomal protein L21 pseudogene 28; ribosomal protein L21 pseudogene 14; ribosomal protein L21 pseudogene 98; ribosomal protein L21 pseudogene 105; ribosomal protein L21 pseudogene 87; ribosomal protein L21 pseudogene 128; ribosomal protein L21 pseudogene 69; ribosomal protein L21 pseudogene 97; ribosomal protein L21; ribosomal protein L21 pseudogene 119; ribosomal protein L21 pseudogene 125	Homo sapiens
100131672	ribosomal protein L21 pseudogene 134; ribosomal protein L21 pseudogene 80; ribosomal protein L21 pseudogene 20; ribosomal protein L21 pseudogene 46; ribosomal protein L21 pseudogene 45; ribosomal protein L21 pseudogene 131; ribosomal protein L21 pseudogene 16; ribosomal protein L21 pseudogene 53; ribosomal protein L21 pseudogene 120; ribosomal protein L21 pseudogene 37; ribosomal protein L21 pseudogene 93; ribosomal protein L21 pseudogene 39; ribosomal protein L21 pseudogene 29; ribosomal protein L21 pseudogene 28; ribosomal protein L21 pseudogene 14; ribosomal protein L21 pseudogene 98; ribosomal protein L21 pseudogene 105; ribosomal protein L21 pseudogene 87; ribosomal protein L21 pseudogene 128; ribosomal protein L21 pseudogene 69; ribosomal protein L21 pseudogene 97; ribosomal protein L21; ribosomal protein L21 pseudogene 119; ribosomal protein L21 pseudogene 125	Homo sapiens

100133273	ribosomal protein L21 pseudogene 134; ribosomal protein L21 pseudogene 80; ribosomal protein L21 pseudogene 20; ribosomal protein L21 pseudogene 46; ribosomal protein L21 pseudogene 45; ribosomal protein L21 pseudogene 131; ribosomal protein L21 pseudogene 16; ribosomal protein L21 pseudogene 53; ribosomal protein L21 pseudogene 120; ribosomal protein L21 pseudogene 37; ribosomal protein L21 pseudogene 93; ribosomal protein L21 pseudogene 39; ribosomal protein L21 pseudogene 29; ribosomal protein L21 pseudogene 28; ribosomal protein L21 pseudogene 14; ribosomal protein L21 pseudogene 98; ribosomal protein L21 pseudogene 105; ribosomal protein L21 pseudogene 87; ribosomal protein L21 pseudogene 128; ribosomal protein L21 pseudogene 69; ribosomal protein L21 pseudogene 97; ribosomal protein L21; ribosomal protein L21 pseudogene 119; ribosomal protein L21 pseudogene 125	Homo sapiens
100131205	ribosomal protein L21 pseudogene 134; ribosomal protein L21 pseudogene 80; ribosomal protein L21 pseudogene 20; ribosomal protein L21 pseudogene 46; ribosomal protein L21 pseudogene 45; ribosomal protein L21 pseudogene 131; ribosomal protein L21 pseudogene 16; ribosomal protein L21 pseudogene 53; ribosomal protein L21 pseudogene 120; ribosomal protein L21 pseudogene 37; ribosomal protein L21 pseudogene 93; ribosomal protein L21 pseudogene 39; ribosomal protein L21 pseudogene 29; ribosomal protein L21 pseudogene 28; ribosomal protein L21 pseudogene 14; ribosomal protein L21 pseudogene 98; ribosomal protein L21 pseudogene 105; ribosomal protein L21 pseudogene 87; ribosomal protein L21 pseudogene 128; ribosomal protein L21 pseudogene 69; ribosomal protein L21 pseudogene 97; ribosomal protein L21; ribosomal protein L21 pseudogene 119; ribosomal protein L21 pseudogene 125	Homo sapiens
440575	ribosomal protein L21 pseudogene 134; ribosomal protein L21 pseudogene 80; ribosomal protein L21 pseudogene 20; ribosomal protein L21 pseudogene 46; ribosomal protein L21 pseudogene 45; ribosomal protein L21 pseudogene 131; ribosomal protein L21 pseudogene 16; ribosomal protein L21 pseudogene 53; ribosomal protein L21 pseudogene 120; ribosomal protein L21 pseudogene 37; ribosomal protein L21 pseudogene 93; ribosomal protein L21 pseudogene 39; ribosomal protein L21 pseudogene 29; ribosomal protein L21 pseudogene 28; ribosomal protein L21 pseudogene 14; ribosomal protein L21 pseudogene 98; ribosomal protein L21 pseudogene 105; ribosomal protein L21 pseudogene 87; ribosomal protein L21 pseudogene 128; ribosomal protein L21 pseudogene 69; ribosomal protein L21 pseudogene 97; ribosomal protein L21; ribosomal protein L21 pseudogene 119; ribosomal protein L21 pseudogene 125	Homo sapiens
388532	ribosomal protein L21 pseudogene 134; ribosomal protein L21 pseudogene 80; ribosomal protein L21 pseudogene 20; ribosomal protein L21 pseudogene 46; ribosomal protein L21 pseudogene 45; ribosomal protein L21 pseudogene 131; ribosomal protein L21 pseudogene 16; ribosomal protein L21 pseudogene 53; ribosomal protein L21 pseudogene 120; ribosomal protein L21 pseudogene 37; ribosomal protein L21 pseudogene 93; ribosomal protein L21 pseudogene 39; ribosomal protein L21 pseudogene 29; ribosomal protein L21 pseudogene 28; ribosomal protein L21 pseudogene 14; ribosomal protein L21 pseudogene 98; ribosomal protein L21 pseudogene 105; ribosomal protein L21 pseudogene 87; ribosomal protein L21 pseudogene 128; ribosomal protein L21 pseudogene 69; ribosomal protein L21 pseudogene 97; ribosomal protein L21; ribosomal protein L21 pseudogene 119; ribosomal protein L21 pseudogene 125	Homo sapiens
389156	ribosomal protein L21 pseudogene 134; ribosomal protein L21 pseudogene 80; ribosomal protein L21 pseudogene 20; ribosomal protein L21 pseudogene 46; ribosomal protein L21 pseudogene 45; ribosomal protein L21 pseudogene 131; ribosomal protein L21 pseudogene 16; ribosomal protein L21 pseudogene 53; ribosomal protein L21 pseudogene 120; ribosomal protein L21 pseudogene 37; ribosomal protein L21 pseudogene 93; ribosomal protein L21 pseudogene 39; ribosomal protein L21 pseudogene 29; ribosomal protein L21 pseudogene 28; ribosomal protein L21 pseudogene 14; ribosomal protein L21 pseudogene 98; ribosomal protein L21 pseudogene 105; ribosomal protein L21 pseudogene 87; ribosomal protein L21 pseudogene 128; ribosomal protein L21 pseudogene 69; ribosomal protein L21 pseudogene 97; ribosomal protein L21; ribosomal protein L21 pseudogene 119; ribosomal protein L21 pseudogene 125	Homo sapiens
388621	ribosomal protein L21 pseudogene 134; ribosomal protein L21 pseudogene 80; ribosomal protein L21 pseudogene 20; ribosomal protein L21 pseudogene 46; ribosomal protein L21 pseudogene 45; ribosomal protein L21 pseudogene 131; ribosomal protein L21 pseudogene 16; ribosomal protein L21 pseudogene 53; ribosomal protein L21 pseudogene 120; ribosomal protein L21 pseudogene 37; ribosomal protein L21 pseudogene 93; ribosomal protein L21 pseudogene 39; ribosomal protein L21 pseudogene 29; ribosomal protein L21 pseudogene 28; ribosomal protein L21 pseudogene 14; ribosomal protein L21 pseudogene 98; ribosomal protein L21 pseudogene 105; ribosomal protein L21 pseudogene 87; ribosomal protein L21 pseudogene 128; ribosomal protein L21 pseudogene 69; ribosomal protein L21 pseudogene 97; ribosomal protein L21; ribosomal protein L21 pseudogene 119; ribosomal protein L21 pseudogene 125	Homo sapiens

728693	ribosomal protein L21 pseudogene 134; ribosomal protein L21 pseudogene 80; ribosomal protein L21 pseudogene 20; ribosomal protein L21 pseudogene 46; ribosomal protein L21 pseudogene 45; ribosomal protein L21 pseudogene 131; ribosomal protein L21 pseudogene 16; ribosomal protein L21 pseudogene 53; ribosomal protein L21 pseudogene 120; ribosomal protein L21 pseudogene 37; ribosomal protein L21 pseudogene 93; ribosomal protein L21 pseudogene 39; ribosomal protein L21 pseudogene 29; ribosomal protein L21 pseudogene 28; ribosomal protein L21 pseudogene 14; ribosomal protein L21 pseudogene 98; ribosomal protein L21 pseudogene 105; ribosomal protein L21 pseudogene 87; ribosomal protein L21 pseudogene 128; ribosomal protein L21 pseudogene 69; ribosomal protein L21 pseudogene 97; ribosomal protein L21; ribosomal protein L21 pseudogene 119; ribosomal protein L21 pseudogene 125	Homo sapiens
100129141	ribosomal protein L21 pseudogene 134; ribosomal protein L21 pseudogene 80; ribosomal protein L21 pseudogene 20; ribosomal protein L21 pseudogene 46; ribosomal protein L21 pseudogene 45; ribosomal protein L21 pseudogene 131; ribosomal protein L21 pseudogene 16; ribosomal protein L21 pseudogene 53; ribosomal protein L21 pseudogene 120; ribosomal protein L21 pseudogene 37; ribosomal protein L21 pseudogene 93; ribosomal protein L21 pseudogene 39; ribosomal protein L21 pseudogene 29; ribosomal protein L21 pseudogene 28; ribosomal protein L21 pseudogene 14; ribosomal protein L21 pseudogene 98; ribosomal protein L21 pseudogene 105; ribosomal protein L21 pseudogene 87; ribosomal protein L21 pseudogene 128; ribosomal protein L21 pseudogene 69; ribosomal protein L21 pseudogene 97; ribosomal protein L21; ribosomal protein L21 pseudogene 119; ribosomal protein L21 pseudogene 125	Homo sapiens
645174	ribosomal protein L21 pseudogene 134; ribosomal protein L21 pseudogene 80; ribosomal protein L21 pseudogene 20; ribosomal protein L21 pseudogene 46; ribosomal protein L21 pseudogene 45; ribosomal protein L21 pseudogene 131; ribosomal protein L21 pseudogene 16; ribosomal protein L21 pseudogene 53; ribosomal protein L21 pseudogene 120; ribosomal protein L21 pseudogene 37; ribosomal protein L21 pseudogene 93; ribosomal protein L21 pseudogene 39; ribosomal protein L21 pseudogene 29; ribosomal protein L21 pseudogene 28; ribosomal protein L21 pseudogene 14; ribosomal protein L21 pseudogene 98; ribosomal protein L21 pseudogene 105; ribosomal protein L21 pseudogene 87; ribosomal protein L21 pseudogene 128; ribosomal protein L21 pseudogene 69; ribosomal protein L21 pseudogene 97; ribosomal protein L21; ribosomal protein L21 pseudogene 119; ribosomal protein L21 pseudogene 125	Homo sapiens
440487	ribosomal protein L21 pseudogene 134; ribosomal protein L21 pseudogene 80; ribosomal protein L21 pseudogene 20; ribosomal protein L21 pseudogene 46; ribosomal protein L21 pseudogene 45; ribosomal protein L21 pseudogene 131; ribosomal protein L21 pseudogene 16; ribosomal protein L21 pseudogene 53; ribosomal protein L21 pseudogene 120; ribosomal protein L21 pseudogene 37; ribosomal protein L21 pseudogene 93; ribosomal protein L21 pseudogene 39; ribosomal protein L21 pseudogene 29; ribosomal protein L21 pseudogene 28; ribosomal protein L21 pseudogene 14; ribosomal protein L21 pseudogene 98; ribosomal protein L21 pseudogene 105; ribosomal protein L21 pseudogene 87; ribosomal protein L21 pseudogene 128; ribosomal protein L21 pseudogene 69; ribosomal protein L21 pseudogene 97; ribosomal protein L21; ribosomal protein L21 pseudogene 119; ribosomal protein L21 pseudogene 125	Homo sapiens
642738	ribosomal protein L21 pseudogene 134; ribosomal protein L21 pseudogene 80; ribosomal protein L21 pseudogene 20; ribosomal protein L21 pseudogene 46; ribosomal protein L21 pseudogene 45; ribosomal protein L21 pseudogene 131; ribosomal protein L21 pseudogene 16; ribosomal protein L21 pseudogene 53; ribosomal protein L21 pseudogene 120; ribosomal protein L21 pseudogene 37; ribosomal protein L21 pseudogene 93; ribosomal protein L21 pseudogene 39; ribosomal protein L21 pseudogene 29; ribosomal protein L21 pseudogene 28; ribosomal protein L21 pseudogene 14; ribosomal protein L21 pseudogene 98; ribosomal protein L21 pseudogene 105; ribosomal protein L21 pseudogene 87; ribosomal protein L21 pseudogene 128; ribosomal protein L21 pseudogene 69; ribosomal protein L21 pseudogene 97; ribosomal protein L21; ribosomal protein L21 pseudogene 119; ribosomal protein L21 pseudogene 125	Homo sapiens
113251	La ribonucleoprotein domain family, member 4	Homo sapiens
2824	glycoprotein M6B	Homo sapiens
6830	suppressor of Ty 6 homolog (S. cerevisiae)	Homo sapiens
26086	G-protein signaling modulator 1 (AGS3-like, C. elegans)	Homo sapiens
10600	ubiquitin specific peptidase 16	Homo sapiens
2177	Fanconi anemia, complementation group D2	Homo sapiens
26145	interferon regulatory factor 2 binding protein 1	Homo sapiens
23283	cleavage stimulation factor, 3' pre-RNA, subunit 2, 64kDa, tau variant	Homo sapiens
55707	NECAP endocytosis associated 2	Homo sapiens
7175	translocated promoter region (to activated MET oncogene)	Homo sapiens
64978	mitochondrial ribosomal protein L38	Homo sapiens
692224	FBXO22 opposite strand (non-protein coding); F-box protein 22	Homo sapiens
11218	DEAD (Asp-Glu-Ala-Asp) box polypeptide 20	Homo sapiens
649299	ribosomal protein L36a pseudogene 51; ribosomal protein L36a pseudogene 37; ribosomal protein L36a pseudogene 49; heterogeneous nuclear ribonucleoprotein H2 (H'); ribosomal protein L36a	Homo sapiens
729362	ribosomal protein L36a pseudogene 51; ribosomal protein L36a pseudogene 37; ribosomal protein L36a pseudogene 49; heterogeneous nuclear ribonucleoprotein H2 (H'); ribosomal protein L36a	Homo sapiens

284230	ribosomal protein L36a pseudogene 51; ribosomal protein L36a pseudogene 37; ribosomal protein L36a pseudogene 49; heterogeneous nuclear ribonucleoprotein H2 (H'); ribosomal protein L36a	Homo sapiens
6173	ribosomal protein L36a pseudogene 51; ribosomal protein L36a pseudogene 37; ribosomal protein L36a pseudogene 49; heterogeneous nuclear ribonucleoprotein H2 (H'); ribosomal protein L36a	Homo sapiens
23512	suppressor of zeste 12 homolog (Drosophila)	Homo sapiens
22992	lysine (K)-specific demethylase 2A	Homo sapiens
387597	immunoglobulin-like domain containing receptor 2	Homo sapiens
55034	molybdenum cofactor sulfurase	Homo sapiens
55787	chromosome X open reading frame 15	Homo sapiens
388552	biogenesis of lysosomal organelles complex-1, subunit 3	Homo sapiens
25904	CCR4-NOT transcription complex, subunit 10	Homo sapiens
9329	general transcription factor IIIC, polypeptide 4, 90kDa	Homo sapiens
9736	ubiquitin specific peptidase 34	Homo sapiens
2786	guanine nucleotide binding protein (G protein), gamma 4	Homo sapiens
9644	SH3 and PX domains 2A	Homo sapiens
10898	cleavage and polyadenylation specific factor 4, 30kDa	Homo sapiens
8816	WD repeat domain 22	Homo sapiens
9730	Vpr (HIV-1) binding protein	Homo sapiens
606551	ubiquitin-conjugating enzyme E2M (UBC12 homolog, yeast); ubiquitin-conjugating enzyme E2M pseudogene 1	Homo sapiens
27097	TAF5-like RNA polymerase II, p300/CBP-associated factor (PCAF)-associated factor, 65kDa	Homo sapiens
51668	heat shock protein family B (small), member 11	Homo sapiens
391370	ribosomal protein S12; ribosomal protein S12 pseudogene 4; ribosomal protein S12 pseudogene 11; ribosomal protein S12 pseudogene 9	Homo sapiens
727997	ribosomal protein S12; ribosomal protein S12 pseudogene 4; ribosomal protein S12 pseudogene 11; ribosomal protein S12 pseudogene 9	Homo sapiens
442270	ribosomal protein S12; ribosomal protein S12 pseudogene 4; ribosomal protein S12 pseudogene 11; ribosomal protein S12 pseudogene 9	Homo sapiens
11169	WD repeat and HMG-box DNA binding protein 1	Homo sapiens
642395	paraspeckle component 1; paraspeckle protein 1 pseudogene	Homo sapiens
4848	CCR4-NOT transcription complex, subunit 2	Homo sapiens
57553	microtubule associated monoxygenase, calponin and LIM domain containing 3	Homo sapiens
649	bone morphogenetic protein 1	Homo sapiens
221937	forkhead box K1	Homo sapiens
55958	kelch-like 9 (Drosophila)	Homo sapiens
1363	carboxypeptidase E	Homo sapiens
26577	procollagen C-endopeptidase enhancer 2	Homo sapiens
8318	CDC45 cell division cycle 45-like (S. cerevisiae)	Homo sapiens
23248	regulation of nuclear pre-mRNA domain containing 2	Homo sapiens
705	bystin-like	Homo sapiens
8722	cathepsin F	Homo sapiens
23543	RNA binding motif protein 9	Homo sapiens
54148	mitochondrial ribosomal protein L39	Homo sapiens
645175	mediator of cell motility 1; similar to mediator of cell motility 1	Homo sapiens
23299	bicaudal D homolog 2 (Drosophila)	Homo sapiens
54970	tetratricopeptide repeat domain 12	Homo sapiens
58496	lymphocyte antigen 6 complex, locus G5B; casein kinase 2, beta polypeptide	Homo sapiens
25819	CCR4 carbon catabolite repression 4-like (S. cerevisiae)	Homo sapiens
55086	chromosome X open reading frame 57	Homo sapiens
9470	eukaryotic translation initiation factor 4E family member 2	Homo sapiens
9825	spermatogenesis associated 2	Homo sapiens
133584	EGF-like, fibronectin type III and laminin G domains	Homo sapiens
64793	coiled-coil domain containing 21	Homo sapiens
5970	v-rel reticuloendotheliosis viral oncogene homolog A (avian)	Homo sapiens
374378	UDP-N-acetyl-alpha-D-galactosamine:polypeptide N-acetylgalactosaminyltransferase-like 4	Homo sapiens
9112	metastasis associated 1	Homo sapiens
115548	FCH domain only 2	Homo sapiens
23112	trinucleotide repeat containing 6B	Homo sapiens
83443	splicing factor 3b, subunit 5, 10kDa	Homo sapiens
6457	SH3-domain GRB2-like 3	Homo sapiens
1111	CHK1 checkpoint homolog (S. pombe)	Homo sapiens
9816	URB2 ribosome biogenesis 2 homolog (S. cerevisiae)	Homo sapiens
1063	centromere protein F, 350/400ka (mitosin)	Homo sapiens
29088	mitochondrial ribosomal protein L15	Homo sapiens
55132	La ribonucleoprotein domain family, member 1B	Homo sapiens
63894	chromosome 14 open reading frame 133	Homo sapiens
59286	ubiquitin-like 5	Homo sapiens
92105	integrator complex subunit 4	Homo sapiens
23351	KIAA0323	Homo sapiens
55291	SAPS domain family, member 3	Homo sapiens
55154	misato homolog 1 (Drosophila)	Homo sapiens
79724	zinc finger protein 768	Homo sapiens
23186	REST corepressor 1	Homo sapiens
10209	similar to eukaryotic translation initiation factor 1; eukaryotic translation initiation factor 1	Homo sapiens

730144	similar to eukaryotic translation initiation factor 1; eukaryotic translation initiation factor 1	Homo sapiens
60561	RAD50 interactor 1	Homo sapiens
79971	G protein-coupled receptor 177	Homo sapiens
9700	extra spindle pole bodies homolog 1 (S. cerevisiae)	Homo sapiens
6238	ribosome binding protein 1 homolog 180kDa (dog)	Homo sapiens
387680	family with sequence similarity 21, member B; family with sequence similarity 21, member A	Homo sapiens
55747	family with sequence similarity 21, member B; family with sequence similarity 21, member A	Homo sapiens
1852	dual specificity phosphatase 9	Homo sapiens
23173	methionyl aminopeptidase 1	Homo sapiens
64426	suppressor of defective silencing 3 homolog (S. cerevisiae)	Homo sapiens
23132	RAD54-like 2 (S. cerevisiae)	Homo sapiens
23511	nucleoporin 188kDa	Homo sapiens
727758	similar to Rho-associated, coiled-coil containing protein kinase 1; Rho-associated, coiled-coil containing protein kinase 1	Homo sapiens
1744	dihydrolipoamide S-succinyltransferase (E2 component of 2-oxo-glutarate complex); dihydrolipoamide S-succinyltransferase pseudogene (E2 component of 2-oxo-glutarate complex)	Homo sapiens
100129808	ribosomal protein L24; ribosomal protein L24 pseudogene 6	Homo sapiens
2197	Finkel-Biskis-Reilly murine sarcoma virus (FBR-MuSV) ubiquitously expressed	Homo sapiens
388556	ribosomal protein S9; ribosomal protein S9 pseudogene 4	Homo sapiens
100131735	similar to RNA binding motif protein, X-linked; similar to hCG2011544; RNA binding motif protein, X-linked	Homo sapiens
100129585	similar to RNA binding motif protein, X-linked; similar to hCG2011544; RNA binding motif protein, X-linked	Homo sapiens
388275	heterogeneous nuclear ribonucleoprotein A1-like 3; similar to heterogeneous nuclear ribonucleoprotein A1; heterogeneous nuclear ribonucleoprotein A1 pseudogene 2; heterogeneous nuclear ribonucleoprotein A1; heterogeneous nuclear ribonucleoprotein A1 pseudogene	Homo sapiens
100131609	heterogeneous nuclear ribonucleoprotein A1-like 3; similar to heterogeneous nuclear ribonucleoprotein A1; heterogeneous nuclear ribonucleoprotein A1 pseudogene 2; heterogeneous nuclear ribonucleoprotein A1; heterogeneous nuclear ribonucleoprotein A1 pseudogene	Homo sapiens
664709	heterogeneous nuclear ribonucleoprotein A1-like 3; similar to heterogeneous nuclear ribonucleoprotein A1; heterogeneous nuclear ribonucleoprotein A1 pseudogene 2; heterogeneous nuclear ribonucleoprotein A1; heterogeneous nuclear ribonucleoprotein A1 pseudogene	Homo sapiens
645691	heterogeneous nuclear ribonucleoprotein A1-like 3; similar to heterogeneous nuclear ribonucleoprotein A1; heterogeneous nuclear ribonucleoprotein A1 pseudogene 2; heterogeneous nuclear ribonucleoprotein A1; heterogeneous nuclear ribonucleoprotein A1 pseudogene	Homo sapiens
728643	heterogeneous nuclear ribonucleoprotein A1-like 3; similar to heterogeneous nuclear ribonucleoprotein A1; heterogeneous nuclear ribonucleoprotein A1 pseudogene 2; heterogeneous nuclear ribonucleoprotein A1; heterogeneous nuclear ribonucleoprotein A1 pseudogene	Homo sapiens
644037	heterogeneous nuclear ribonucleoprotein A1-like 3; similar to heterogeneous nuclear ribonucleoprotein A1; heterogeneous nuclear ribonucleoprotein A1 pseudogene 2; heterogeneous nuclear ribonucleoprotein A1; heterogeneous nuclear ribonucleoprotein A1 pseudogene	Homo sapiens
51322	WW domain containing adaptor with coiled-coil	Homo sapiens
727761	deoxythymidylate kinase (thymidylate kinase); similar to Deoxythymidylate kinase (thymidylate kinase)	Homo sapiens
4440	musashi homolog 1 (Drosophila)	Homo sapiens
170622	COMM domain containing 6	Homo sapiens
114885	oxysterol binding protein-like 11	Homo sapiens
96764	trimethylguanosine synthase homolog (S. cerevisiae)	Homo sapiens
4722	NADH dehydrogenase (ubiquinone) Fe-S protein 3, 30kDa (NADH-coenzyme Q reductase)	Homo sapiens
5436	polymerase (RNA) II (DNA directed) polypeptide G	Homo sapiens
10902	bromodomain containing 8	Homo sapiens
100131693	eukaryotic translation initiation factor 4E; similar to hCG1777996; similar to eukaryotic translation initiation factor 4E	Homo sapiens
100131565	eukaryotic translation initiation factor 4E; similar to hCG1777996; similar to eukaryotic translation initiation factor 4E	Homo sapiens
10534	Sjogren syndrome/scleroderma autoantigen 1	Homo sapiens
55699	isoleucyl-tRNA synthetase 2, mitochondrial	Homo sapiens
54497	HEAT repeat containing 5B	Homo sapiens
650638	similar to signal recognition particle 54kDa; signal recognition particle 54kDa	Homo sapiens
6419	SET domain and mariner transposase fusion gene	Homo sapiens
653879	similar to Complement C3 precursor; complement component 3; hypothetical protein LOC100133511	Homo sapiens
100133511	similar to Complement C3 precursor; complement component 3; hypothetical protein LOC100133511	Homo sapiens
9739	SET domain containing 1A	Homo sapiens
151579	basic leucine zipper and W2 domains 1 pseudogene 1; basic leucine zipper and W2 domains 1 like 1; basic leucine zipper and W2 domains 1	Homo sapiens

391592	basic leucine zipper and W2 domains 1 pseudogene 1; basic leucine zipper and W2 domains 1 like 1; basic leucine zipper and W2 domains 1	Homo sapiens
57572	dedicator of cytokinesis 6	Homo sapiens
28991	COMM domain containing 5	Homo sapiens
25847	anaphase promoting complex subunit 13	Homo sapiens
54480	chondroitin sulfate glucuronyltransferase	Homo sapiens
653450	family with sequence similarity 21, member D; family with sequence similarity 21, member C	Homo sapiens
253725	family with sequence similarity 21, member D; family with sequence similarity 21, member C	Homo sapiens
9910	RAB GTPase activating protein 1-like	Homo sapiens
1837	dystrobrevin, alpha	Homo sapiens
23215	BAT2 domain containing 1	Homo sapiens
22856	chondroitin sulfate synthase 1	Homo sapiens
55319	chromosome 4 open reading frame 43	Homo sapiens
23326	ubiquitin specific peptidase 22	Homo sapiens
84259	DCN1, defective in cullin neddylation 1, domain containing 5 (S. cerevisiae)	Homo sapiens
2131	exostoses (multiple) 1	Homo sapiens
64093	SPARC related modular calcium binding 1	Homo sapiens
389901	X-ray repair complementing defective repair in Chinese hamster cells 6; similar to ATP-dependent DNA helicase II, 70 kDa subunit	Homo sapiens
27344	proprotein convertase subtilisin/kexin type 1 inhibitor	Homo sapiens
50813	COP9 constitutive photomorphogenic homolog subunit 7A (Arabidopsis)	Homo sapiens
65109	UPF3 regulator of nonsense transcripts homolog B (yeast)	Homo sapiens
55174	integrator complex subunit 10	Homo sapiens
647030	eukaryotic translation elongation factor 1 beta 2; eukaryotic translation elongation factor 1 beta 2-like	Homo sapiens
27433	torsin family 2, member A	Homo sapiens
80328	UL16 binding protein 2	Homo sapiens
90957	DEAH (Asp-Glu-Ala-Asp/His) box polypeptide 57	Homo sapiens
8434	reversion-inducing-cysteine-rich protein with kazal motifs	Homo sapiens
723961	insulin-like growth factor 2 (somatomedin A); insulin; INS-IGF2 readthrough transcript	Homo sapiens
9474	ATG5 autophagy related 5 homolog (S. cerevisiae)	Homo sapiens
9444	quaking homolog, KH domain RNA binding (mouse)	Homo sapiens
79033	exoribonuclease 3	Homo sapiens
740	mitochondrial ribosomal protein L49	Homo sapiens
51299	neuritin 1	Homo sapiens
51366	similar to E3 ubiquitin protein ligase, HECT domain containing, 1; ubiquitin protein ligase E3 component n-recognin 5	Homo sapiens
730429	similar to E3 ubiquitin protein ligase, HECT domain containing, 1; ubiquitin protein ligase E3 component n-recognin 5	Homo sapiens
55432	YOD1 OTU deubiquinating enzyme 1 homolog (S. cerevisiae)	Homo sapiens
4885	neuronal pentraxin II	Homo sapiens
192669	eukaryotic translation initiation factor 2C, 3	Homo sapiens
100132779	serine/arginine repetitive matrix 2; hypothetical LOC100132779	Homo sapiens
176	aggrecan	Homo sapiens
221935	sidekick homolog 1, cell adhesion molecule (chicken); hypothetical LOC730351	Homo sapiens
730351	sidekick homolog 1, cell adhesion molecule (chicken); hypothetical LOC730351	Homo sapiens
10078	tumor suppressing subtransferable candidate 4	Homo sapiens
54914	KIAA1797	Homo sapiens
100129652	hypothetical protein LOC100129652; ezrin	Homo sapiens
81557	melanoma antigen family D, 4B; melanoma antigen family D, 4	Homo sapiens
728239	melanoma antigen family D, 4B; melanoma antigen family D, 4	Homo sapiens
51070	nitric oxide synthase interacting protein	Homo sapiens
84126	ATR interacting protein	Homo sapiens
7156	topoisomerase (DNA) III alpha	Homo sapiens
441089	similar to cofactor required for Sp1 transcriptional activation, subunit 8, 34kDa; mediator complex subunit 27; CRSP8 pseudogene	Homo sapiens
100134189	similar to cofactor required for Sp1 transcriptional activation, subunit 8, 34kDa; mediator complex subunit 27; CRSP8 pseudogene	Homo sapiens
9442	similar to cofactor required for Sp1 transcriptional activation, subunit 8, 34kDa; mediator complex subunit 27; CRSP8 pseudogene	Homo sapiens
10454	mitogen-activated protein kinase kinase kinase 7 interacting protein 1	Homo sapiens
699	budding uninhibited by benzimidazoles 1 homolog (yeast)	Homo sapiens
53917	RAB24, member RAS oncogene family	Homo sapiens
55197	regulation of nuclear pre-mRNA domain containing 1A	Homo sapiens
394	Rho GTPase activating protein 5	Homo sapiens
10196	protein arginine methyltransferase 3	Homo sapiens
6631	small nuclear ribonucleoprotein polypeptide C	Homo sapiens
84164	activating signal cointegrator 1 complex subunit 2	Homo sapiens
642954	hypothetical LOC642954; retinoblastoma binding protein 4	Homo sapiens
4236	microfibrillar-associated protein 1	Homo sapiens
10471	prefoldin subunit 6	Homo sapiens
51182	heat shock 70kDa protein 14	Homo sapiens
23405	dicer 1, ribonuclease type III	Homo sapiens
10460	transforming, acidic coiled-coil containing protein 3	Homo sapiens
4148	matrilin 3	Homo sapiens
84946	similar to putative protein STRF7; LTV1 homolog (S. cerevisiae)	Homo sapiens
100128319	similar to putative protein STRF7; LTV1 homolog (S. cerevisiae)	Homo sapiens

91775	family with sequence similarity 55, member C	Homo sapiens
7268	tetratricopeptide repeat domain 4	Homo sapiens
54496	protein arginine methyltransferase 7	Homo sapiens
55170	protein arginine methyltransferase 6	Homo sapiens
9993	DiGeorge syndrome critical region gene 2	Homo sapiens
7186	TNF receptor-associated factor 2	Homo sapiens
284252	potassium channel tetramerisation domain containing 1	Homo sapiens
8506	contactin associated protein 1	Homo sapiens
54963	uridine-cytidine kinase 1-like 1	Homo sapiens
2968	general transcription factor IIH, polypeptide 4, 52kDa	Homo sapiens
90390	mediator complex subunit 30	Homo sapiens
80013	chromosome 10 open reading frame 97	Homo sapiens
9969	mediator complex subunit 13	Homo sapiens
9562	multiple inositol polyphosphate histidine phosphatase, 1	Homo sapiens
26015	RNA polymerase II associated protein 1	Homo sapiens
8805	tripartite motif-containing 24	Homo sapiens
80279	CDK5 regulatory subunit associated protein 3	Homo sapiens
11222	mitochondrial ribosomal protein L3	Homo sapiens
10848	protein phosphatase 1, regulatory (inhibitor) subunit 13 like	Homo sapiens
64708	COP9 constitutive photomorphogenic homolog subunit 7B (Arabidopsis)	Homo sapiens
85364	zinc finger, CCHC domain containing 3	Homo sapiens
54505	DEAH (Asp-Glu-Ala-His) box polypeptide 29	Homo sapiens
9589	Wilms tumor 1 associated protein	Homo sapiens
9469	carbohydrate (chondroitin 6) sulfotransferase 3	Homo sapiens
11041	UDP-GlcNAc:betaGal beta-1,3-N-acetylglucosaminyltransferase 1; UDP-GlcNAc:betaGal beta-1,3-N-acetylglucosaminyltransferase 2	Homo sapiens
29066	zinc finger CCCH-type containing 7A	Homo sapiens
5191	peroxisomal biogenesis factor 7	Homo sapiens
54464	5'-3' exoribonuclease 1	Homo sapiens
84461	neuralized homolog 4 (Drosophila)	Homo sapiens
79699	zyg-11 homolog B (C. elegans)	Homo sapiens
79184	BRCA1/BRCA2-containing complex, subunit 3	Homo sapiens
22911	WD repeat domain 47	Homo sapiens
55723	ASF1 anti-silencing function 1 homolog B (S. cerevisiae)	Homo sapiens
653881	ribosomal protein L3; similar to 60S ribosomal protein L3 (L4)	Homo sapiens
100130561	high-mobility group box 1; high-mobility group box 1-like 10	Homo sapiens
5427	polymerase (DNA directed), epsilon 2 (p59 subunit)	Homo sapiens
10607	transducin (beta)-like 3	Homo sapiens
23367	La ribonucleoprotein domain family, member 1	Homo sapiens
64431	ARP6 actin-related protein 6 homolog (yeast)	Homo sapiens
64754	SET and MYND domain containing 3	Homo sapiens
57448	baculoviral IAP repeat-containing 6	Homo sapiens
11321	GPN-loop GTPase 1	Homo sapiens
10934	mortality factor 4; mortality factor 4 like 1	Homo sapiens
10933	mortality factor 4; mortality factor 4 like 1	Homo sapiens
338761	complement component 1, q subcomponent-like 4	Homo sapiens
646966	ribosomal protein L26 pseudogene 33; ribosomal protein L26; ribosomal protein L26 pseudogene 16; ribosomal protein L26 pseudogene 19; ribosomal protein L26 pseudogene 6	Homo sapiens
100131526	ribosomal protein L26 pseudogene 33; ribosomal protein L26; ribosomal protein L26 pseudogene 16; ribosomal protein L26 pseudogene 19; ribosomal protein L26 pseudogene 6	Homo sapiens
441073	ribosomal protein L26 pseudogene 33; ribosomal protein L26; ribosomal protein L26 pseudogene 16; ribosomal protein L26 pseudogene 19; ribosomal protein L26 pseudogene 6	Homo sapiens
100132547	ribosomal protein L26 pseudogene 33; ribosomal protein L26; ribosomal protein L26 pseudogene 16; ribosomal protein L26 pseudogene 19; ribosomal protein L26 pseudogene 6	Homo sapiens
400011	suppressor of Ty 16 homolog (S. cerevisiae); suppressor of Ty 16 homolog (S. cerevisiae) pseudogene	Homo sapiens
51371	proteasome maturation protein	Homo sapiens
8726	embryonic ectoderm development	Homo sapiens
8924	hect domain and RLD 2	Homo sapiens
4548	5-methyltetrahydrofolate-homocysteine methyltransferase	Homo sapiens
84364	ADP-ribosylation factor GTPase activating protein 2	Homo sapiens
54915	YTH domain family, member 1	Homo sapiens
10100	tetraspanin 2	Homo sapiens
10847	Snf2-related CREBBP activator protein	Homo sapiens
11230	PRA1 domain family, member 2	Homo sapiens
11340	exosome component 8	Homo sapiens
11135	CDC42 effector protein (Rho GTPase binding) 1	Homo sapiens
642590	spermine synthase; similar to spermine synthase	Homo sapiens
646347	spermine synthase; similar to spermine synthase	Homo sapiens
51126	N-acetyltransferase 5 (GCN5-related, putative)	Homo sapiens
1676	DNA fragmentation factor, 45kDa, alpha polypeptide	Homo sapiens
26523	eukaryotic translation initiation factor 2C, 1	Homo sapiens
25896	integrator complex subunit 7	Homo sapiens
440275	eukaryotic translation initiation factor 2 alpha kinase 4	Homo sapiens
222229	leucine-rich repeats and WD repeat domain containing 1	Homo sapiens
8106	poly(A) binding protein, nuclear 1	Homo sapiens

54665	round spermatid basic protein 1	Homo sapiens
100291837	similar to ribosomal protein S21	Homo sapiens
55112	WD repeat domain 60	Homo sapiens
8536	calcium/calmodulin-dependent protein kinase I	Homo sapiens
4790	nuclear factor of kappa light polypeptide gene enhancer in B-cells 1	Homo sapiens
8021	nucleoporin 214kDa	Homo sapiens
283237	tetratricopeptide repeat domain 9C	Homo sapiens
287	ankyrin 2, neuronal	Homo sapiens
27042	chromosome 1 open reading frame 107	Homo sapiens
2549	GRB2-associated binding protein 1	Homo sapiens
100037280	ubiquitin-conjugating enzyme E2D 3 (UBC4/5 homolog, yeast); ubiquitin-conjugating enzyme E2D 3 pseudogene	Homo sapiens
9815	G protein-coupled receptor kinase interacting ArfGAP 2	Homo sapiens
64146	peptide deformylase (mitochondrial); component of oligomeric golgi complex 8	Homo sapiens
84342	peptide deformylase (mitochondrial); component of oligomeric golgi complex 8	Homo sapiens
80222	threonyl-tRNA synthetase 2, mitochondrial (putative)	Homo sapiens
80227	proteasomal ATPase-associated factor 1	Homo sapiens
55054	ATG16 autophagy related 16-like 1 (S. cerevisiae)	Homo sapiens
653877	similar to Down-regulated in metastasis protein (Key-1A6 protein) (Novel nucleolar protein 73) (NNP73); UTP20, small subunit (SSU) processome component, homolog (yeast)	Homo sapiens
723972	hepatopoietin PCn127; acidic (leucine-rich) nuclear phosphoprotein 32 family, member A	Homo sapiens
22950	solute carrier family 4 (anion exchanger), member 1, adaptor protein	Homo sapiens
84135	UTP15, U3 small nucleolar ribonucleoprotein, homolog (S. cerevisiae)	Homo sapiens
6456	SH3-domain GRB2-like 2	Homo sapiens
93627	TBC domain-containing protein kinase-like	Homo sapiens
644063	heterogeneous nuclear ribonucleoprotein K; similar to heterogeneous nuclear ribonucleoprotein K	Homo sapiens
79786	kelch-like 36 (Drosophila)	Homo sapiens
23049	SMG1 homolog, phosphatidylinositol 3-kinase-related kinase (C. elegans)	Homo sapiens
651610	similar to Serine-protein kinase ATM (Ataxia telangiectasia mutated) (A-T, mutated); ataxia telangiectasia mutated	Homo sapiens
472	similar to Serine-protein kinase ATM (Ataxia telangiectasia mutated) (A-T, mutated); ataxia telangiectasia mutated	Homo sapiens
8019	bromodomain containing 3	Homo sapiens
4289	muskelin 1, intracellular mediator containing kelch motifs	Homo sapiens
728642	similar to cell division cycle 2-like 1 (PITSLRE proteins); cell division cycle 2-like 1 (PITSLRE proteins); cell division cycle 2-like 2 (PITSLRE proteins)	Homo sapiens
100133692	similar to cell division cycle 2-like 1 (PITSLRE proteins); cell division cycle 2-like 1 (PITSLRE proteins); cell division cycle 2-like 2 (PITSLRE proteins)	Homo sapiens
197370	non-SMC element 1 homolog (S. cerevisiae)	Homo sapiens
11080	DnaJ (Hsp40) homolog, subfamily B, member 4	Homo sapiens
9140	ATG12 autophagy related 12 homolog (S. cerevisiae)	Homo sapiens
727726	similar to poly (ADP-ribose) glycohydrolase; poly (ADP-ribose) glycohydrolase	Homo sapiens
8505	similar to poly (ADP-ribose) glycohydrolase; poly (ADP-ribose) glycohydrolase	Homo sapiens
79735	TBC1 domain family, member 17	Homo sapiens
1029	cyclin-dependent kinase inhibitor 2A (melanoma, p16, inhibits CDK4)	Homo sapiens
147700	kinesin light chain 3	Homo sapiens
5564	protein kinase, AMP-activated, beta 1 non-catalytic subunit	Homo sapiens
4524	5,10-methylenetetrahydrofolate reductase (NADPH)	Homo sapiens
55626	autophagy/beclin-1 regulator 1	Homo sapiens
55072	ring finger protein 31	Homo sapiens
57646	ubiquitin specific peptidase 28	Homo sapiens
100271831	hypothetical LOC100271831; mitogen-activated protein kinase 3	Homo sapiens
55142	HAUS augmin-like complex, subunit 2	Homo sapiens
79613	transmembrane and coiled-coil domains 7	Homo sapiens
3092	huntingtin interacting protein 1	Homo sapiens
5557	primase, DNA, polypeptide 1 (49kDa)	Homo sapiens
57707	KIAA1609	Homo sapiens
729494	similar to actin related protein 2/3 complex subunit 3; hypothetical LOC729841; actin related protein 2/3 complex, subunit 3, 21kDa	Homo sapiens
729841	similar to actin related protein 2/3 complex subunit 3; hypothetical LOC729841; actin related protein 2/3 complex, subunit 3, 21kDa	Homo sapiens
9360	peptidylprolyl isomerase G (cyclophilin G)	Homo sapiens
80306	mediator complex subunit 28	Homo sapiens
79726	WD repeat domain 59	Homo sapiens
51626	dynein, cytoplasmic 2, light intermediate chain 1	Homo sapiens
51133	potassium channel tetramerisation domain containing 3	Homo sapiens
731751	similar to protein kinase, DNA-activated, catalytic polypeptide; protein kinase, DNA-activated, catalytic polypeptide	Homo sapiens
79577	cell division cycle 73, Paf1/RNA polymerase II complex component, homolog (S. cerevisiae)	Homo sapiens
91369	ankyrin repeat domain 40	Homo sapiens
55746	nucleoporin 133kDa	Homo sapiens
92104	tetratricopeptide repeat domain 30A	Homo sapiens

85313	peptidylprolyl isomerase (cyclophilin)-like 4	Homo sapiens
11065	ubiquitin-conjugating enzyme E2C	Homo sapiens
80020	FAD-dependent oxidoreductase domain containing 2	Homo sapiens
51747	cisplatin resistance-associated overexpressed protein	Homo sapiens
57539	WD repeat domain 35	Homo sapiens
80304	chromosome 2 open reading frame 44	Homo sapiens
100131294	RAB13, member RAS oncogene family; similar to hCG24991	Homo sapiens
84950	PRP38 pre-mRNA processing factor 38 (yeast) domain containing A	Homo sapiens
474383	coagulation factor VIII-associated (intronic transcript) 2; coagulation factor VIII-associated (intronic transcript) 3; coagulation factor VIII-associated (intronic transcript) 1	Homo sapiens
8263	coagulation factor VIII-associated (intronic transcript) 2; coagulation factor VIII-associated (intronic transcript) 3; coagulation factor VIII-associated (intronic transcript) 1	Homo sapiens
474384	coagulation factor VIII-associated (intronic transcript) 2; coagulation factor VIII-associated (intronic transcript) 3; coagulation factor VIII-associated (intronic transcript) 1	Homo sapiens
10179	RNA binding motif protein 7	Homo sapiens
51742	AT rich interactive domain 4B (RBP1-like)	Homo sapiens
55759	WD repeat domain 12	Homo sapiens
83667	sestrin 2	Homo sapiens
51003	mediator complex subunit 31	Homo sapiens
55206	strawberry notch homolog 1 (Drosophila)	Homo sapiens
51693	trafficking protein particle complex 2-like	Homo sapiens
9456	homer homolog 1 (Drosophila)	Homo sapiens
1158	creatine kinase, muscle	Homo sapiens
2332	fragile X mental retardation 1	Homo sapiens
100130107	ribosomal protein S3A pseudogene 5; ribosomal protein S3a pseudogene 47; ribosomal protein S3a pseudogene 49; ribosomal protein S3A; hypothetical LOC100131699; hypothetical LOC100130107	Homo sapiens
146053	ribosomal protein S3A pseudogene 5; ribosomal protein S3a pseudogene 47; ribosomal protein S3a pseudogene 49; ribosomal protein S3A; hypothetical LOC100131699; hypothetical LOC100130107	Homo sapiens
100131699	ribosomal protein S3A pseudogene 5; ribosomal protein S3a pseudogene 47; ribosomal protein S3a pseudogene 49; ribosomal protein S3A; hypothetical LOC100131699; hypothetical LOC100130107	Homo sapiens
400652	ribosomal protein S3A pseudogene 5; ribosomal protein S3a pseudogene 47; ribosomal protein S3a pseudogene 49; ribosomal protein S3A; hypothetical LOC100131699; hypothetical LOC100130107	Homo sapiens
439992	ribosomal protein S3A pseudogene 5; ribosomal protein S3a pseudogene 47; ribosomal protein S3a pseudogene 49; ribosomal protein S3A; hypothetical LOC100131699; hypothetical LOC100130107	Homo sapiens
7092	tolloid-like 1	Homo sapiens
25842	ASF1 anti-silencing function 1 homolog A (S. cerevisiae)	Homo sapiens
23001	WD repeat and FYVE domain containing 3	Homo sapiens
7298	thymidylate synthetase	Homo sapiens
10371	sema domain, immunoglobulin domain (Ig), short basic domain, secreted, (semaphorin) 3A	Homo sapiens
731605	similar to Bcl-2-associated transcription factor 1 (Btf); BCL2-associated transcription factor 1	Homo sapiens
9774	similar to Bcl-2-associated transcription factor 1 (Btf); BCL2-associated transcription factor 1	Homo sapiens
3839	karyopherin alpha 3 (importin alpha 4)	Homo sapiens
55854	zinc finger CCCH-type containing 15	Homo sapiens
10914	poly(A) polymerase alpha	Homo sapiens
10799	ribonuclease P/MRP 40kDa subunit	Homo sapiens
100129882	ribosomal protein L31 pseudogene 49; ribosomal protein L31 pseudogene 17; ribosomal protein L31	Homo sapiens
653773	ribosomal protein L31 pseudogene 49; ribosomal protein L31 pseudogene 17; ribosomal protein L31	Homo sapiens
253959	GTPase activating Rap/RanGAP domain-like 1	Homo sapiens
54821	excision repair cross-complementing rodent repair deficiency, complementation group 6-like	Homo sapiens
9403	15 kDa selenoprotein	Homo sapiens
26019	UPF2 regulator of nonsense transcripts homolog (yeast)	Homo sapiens
57508	integrator complex subunit 2	Homo sapiens
79023	nucleoporin 37kDa	Homo sapiens
29079	mediator complex subunit 4	Homo sapiens
6198	ribosomal protein S6 kinase, 70kDa, polypeptide 1	Homo sapiens
79719	alpha- and gamma-adaptin-binding protein p34	Homo sapiens
55837	E2F-associated phosphoprotein	Homo sapiens
23074	UHRF1 binding protein 1-like	Homo sapiens
9873	FCH and double SH3 domains 2	Homo sapiens
55802	DCP1 decapping enzyme homolog A (S. cerevisiae)	Homo sapiens
23527	ArfGAP with coiled-coil, ankyrin repeat and PH domains 2	Homo sapiens
9026	huntingtin interacting protein 1 related	Homo sapiens
100132973	similar to elongin C; transcription elongation factor B (SIII), polypeptide 1 (15kDa, elongin C)	Homo sapiens
253714	chromosome 6 open reading frame 167	Homo sapiens
22828	RNA binding motif protein 16	Homo sapiens

6480	ST6 beta-galactosamide alpha-2,6-sialyltranferase 1	Homo sapiens
23431	adaptor-related protein complex 4, epsilon 1 subunit	Homo sapiens
9039	ubiquitin-like modifier activating enzyme 3	Homo sapiens
9779	TBC1 domain family, member 5	Homo sapiens
22980	transcription factor 25 (basic helix-loop-helix)	Homo sapiens
29888	striatin, calmodulin binding protein 4	Homo sapiens
79677	structural maintenance of chromosomes 6	Homo sapiens
441228	exportin, tRNA (nuclear export receptor for tRNAs); similar to Exportin-T (tRNA exportin) (Exportin(tRNA))	Homo sapiens
6604	SWI/SNF related, matrix associated, actin dependent regulator of chromatin, subfamily d, member 3	Homo sapiens
55127	HEAT repeat containing 1	Homo sapiens
344593	protein tyrosine phosphatase, non-receptor type 11; similar to protein tyrosine phosphatase, non-receptor type 11	Homo sapiens
442113	protein tyrosine phosphatase, non-receptor type 11; similar to protein tyrosine phosphatase, non-receptor type 11	Homo sapiens
26091	hect domain and RLD 4	Homo sapiens
56916	SWI/SNF-related, matrix-associated actin-dependent regulator of chromatin, subfamily a, containing DEAD/H box 1	Homo sapiens
374383	hypothetical protein DKFZp686024166	Homo sapiens
266722	heparan sulfate 6-O-sulfotransferase 3	Homo sapiens
5108	pericentriolar material 1	Homo sapiens
9064	mitogen-activated protein kinase kinase kinase 6	Homo sapiens
644101	similar to chromobox homolog 3; chromobox homolog 3 (HP1 gamma homolog, Drosophila)	Homo sapiens
391358	tRNA methyltransferase 11-2 homolog (S. cerevisiae); similar to CG12975	Homo sapiens
149951	COMM domain containing 7	Homo sapiens
11137	PWP1 homolog (S. cerevisiae)	Homo sapiens
89890	kelch repeat and BTB (POZ) domain containing 6	Homo sapiens
10241	calcium binding and coiled-coil domain 2	Homo sapiens
653888	actin related protein 2/3 complex, subunit 1B, 41kDa; similar to Actin-related protein 2/3 complex subunit 1B (ARP2/3 complex 41 kDa subunit) (p41-ARC)	Homo sapiens
140775	Smith-Magenis syndrome chromosome region, candidate 8	Homo sapiens
57122	nucleoporin 107kDa	Homo sapiens
9325	thyroid hormone receptor interactor 4	Homo sapiens
9374	palmitoyl-protein thioesterase 2	Homo sapiens
64132	xylosyltransferase II	Homo sapiens
646483	ribosomal protein L6 pseudogene 27; ribosomal protein L6 pseudogene 19; ribosomal protein L6; ribosomal protein L6 pseudogene 10	Homo sapiens
642828	ribosomal protein L6 pseudogene 27; ribosomal protein L6 pseudogene 19; ribosomal protein L6; ribosomal protein L6 pseudogene 10	Homo sapiens
645387	ribosomal protein L6 pseudogene 27; ribosomal protein L6 pseudogene 19; ribosomal protein L6; ribosomal protein L6 pseudogene 10	Homo sapiens
4884	neuronal pentraxin I	Homo sapiens
729046	ribosomal protein L17 pseudogene 22; ribosomal protein L17 pseudogene 36; ribosomal protein L17 pseudogene 20; similar to ribosomal protein L17; ribosomal protein L17 pseudogene 33; ribosomal protein L17 pseudogene 34; ribosomal protein L17 pseudogene 9; ribosomal protein L17; ribosomal protein L17 pseudogene 18; ribosomal protein L17 pseudogene 7; ribosomal protein L17 pseudogene 39	Homo sapiens
100129657	ribosomal protein L17 pseudogene 22; ribosomal protein L17 pseudogene 36; ribosomal protein L17 pseudogene 20; similar to ribosomal protein L17; ribosomal protein L17 pseudogene 33; ribosomal protein L17 pseudogene 34; ribosomal protein L17 pseudogene 9; ribosomal protein L17; ribosomal protein L17 pseudogene 18; ribosomal protein L17 pseudogene 7; ribosomal protein L17 pseudogene 39	Homo sapiens
643863	ribosomal protein L17 pseudogene 22; ribosomal protein L17 pseudogene 36; ribosomal protein L17 pseudogene 20; similar to ribosomal protein L17; ribosomal protein L17 pseudogene 33; ribosomal protein L17 pseudogene 34; ribosomal protein L17 pseudogene 9; ribosomal protein L17; ribosomal protein L17 pseudogene 18; ribosomal protein L17 pseudogene 7; ribosomal protein L17 pseudogene 39	Homo sapiens
100132742	ribosomal protein L17 pseudogene 22; ribosomal protein L17 pseudogene 36; ribosomal protein L17 pseudogene 20; similar to ribosomal protein L17; ribosomal protein L17 pseudogene 33; ribosomal protein L17 pseudogene 34; ribosomal protein L17 pseudogene 9; ribosomal protein L17; ribosomal protein L17 pseudogene 18; ribosomal protein L17 pseudogene 7; ribosomal protein L17 pseudogene 39	Homo sapiens
729301	ribosomal protein L17 pseudogene 22; ribosomal protein L17 pseudogene 36; ribosomal protein L17 pseudogene 20; similar to ribosomal protein L17; ribosomal protein L17 pseudogene 33; ribosomal protein L17 pseudogene 34; ribosomal protein L17 pseudogene 9; ribosomal protein L17; ribosomal protein L17 pseudogene 18; ribosomal protein L17 pseudogene 7; ribosomal protein L17 pseudogene 39	Homo sapiens

727984	ribosomal protein L17 pseudogene 22; ribosomal protein L17 pseudogene 36; ribosomal protein L17 pseudogene 20; similar to ribosomal protein L17; ribosomal protein L17 pseudogene 33; ribosomal protein L17 pseudogene 34; ribosomal protein L17 pseudogene 9; ribosomal protein L17; ribosomal protein L17 pseudogene 18; ribosomal protein L17 pseudogene 7; ribosomal protein L17 pseudogene 39	Homo sapiens
646909	ribosomal protein L17 pseudogene 22; ribosomal protein L17 pseudogene 36; ribosomal protein L17 pseudogene 20; similar to ribosomal protein L17; ribosomal protein L17 pseudogene 33; ribosomal protein L17 pseudogene 34; ribosomal protein L17 pseudogene 9; ribosomal protein L17; ribosomal protein L17 pseudogene 18; ribosomal protein L17 pseudogene 7; ribosomal protein L17 pseudogene 39	Homo sapiens
645296	ribosomal protein L17 pseudogene 22; ribosomal protein L17 pseudogene 36; ribosomal protein L17 pseudogene 20; similar to ribosomal protein L17; ribosomal protein L17 pseudogene 33; ribosomal protein L17 pseudogene 34; ribosomal protein L17 pseudogene 9; ribosomal protein L17; ribosomal protein L17 pseudogene 18; ribosomal protein L17 pseudogene 7; ribosomal protein L17 pseudogene 39	Homo sapiens
391655	ribosomal protein L17 pseudogene 22; ribosomal protein L17 pseudogene 36; ribosomal protein L17 pseudogene 20; similar to ribosomal protein L17; ribosomal protein L17 pseudogene 33; ribosomal protein L17 pseudogene 34; ribosomal protein L17 pseudogene 9; ribosomal protein L17; ribosomal protein L17 pseudogene 18; ribosomal protein L17 pseudogene 7; ribosomal protein L17 pseudogene 39	Homo sapiens
729340	ribosomal protein L17 pseudogene 22; ribosomal protein L17 pseudogene 36; ribosomal protein L17 pseudogene 20; similar to ribosomal protein L17; ribosomal protein L17 pseudogene 33; ribosomal protein L17 pseudogene 34; ribosomal protein L17 pseudogene 9; ribosomal protein L17; ribosomal protein L17 pseudogene 18; ribosomal protein L17 pseudogene 7; ribosomal protein L17 pseudogene 39	Homo sapiens
79586	chondroitin polymerizing factor	Homo sapiens
24140	FtsJ homolog 1 (E. coli)	Homo sapiens
64087	methylcrotonoyl-Coenzyme A carboxylase 2 (beta)	Homo sapiens
10403	NDC80 homolog, kinetochore complex component (S. cerevisiae)	Homo sapiens
1657	Dmx-like 1	Homo sapiens
6119	replication protein A3, 14kDa	Homo sapiens
5422	polymerase (DNA directed), alpha 1, catalytic subunit	Homo sapiens
9684	leucine rich repeat containing 14	Homo sapiens
55738	ADP-ribosylation factor GTPase activating protein 1	Homo sapiens
652346	promyelocytic leukemia; similar to promyelocytic leukemia protein isoform 1	Homo sapiens
5371	promyelocytic leukemia; similar to promyelocytic leukemia protein isoform 1	Homo sapiens
1105	chromodomain helicase DNA binding protein 1	Homo sapiens
728532	similar to dynein cytoplasmic 1 intermediate chain 2; dynein, cytoplasmic 1, intermediate chain 2	Homo sapiens
548645	DnaJ (Hsp40) homolog, subfamily C , member 25; guanine nucleotide binding protein (G protein), gamma 10; DNAJC25-GNG10 readthrough transcript	Homo sapiens
552891	DnaJ (Hsp40) homolog, subfamily C , member 25; guanine nucleotide binding protein (G protein), gamma 10; DNAJC25-GNG10 readthrough transcript	Homo sapiens
92675	histidyl-tRNA synthetase 2, mitochondrial (putative); D-tyrosyl-tRNA deacylase 1 homolog (S. cerevisiae)	Homo sapiens
55023	pleckstrin homology domain interacting protein	Homo sapiens
646791	similar to Acidic leucine-rich nuclear phosphoprotein 32 family member B (PHAPI2 protein) (Silver-stainable protein SSP29) (Acidic protein rich in leucines); acidic (leucine-rich) nuclear phosphoprotein 32 family, member B	Homo sapiens
22902	RUN and FYVE domain containing 3	Homo sapiens
79050	nucleolar complex associated 4 homolog (S. cerevisiae)	Homo sapiens
5393	exosome component 9	Homo sapiens
23063	wings apart-like homolog (Drosophila)	Homo sapiens
116966	WD repeat domain 17	Homo sapiens
390284	signal recognition particle 14kDa (homologous Alu RNA binding protein) pseudogene 1; signal recognition particle 14kDa (homologous Alu RNA binding protein)	Homo sapiens
8720	membrane-bound transcription factor peptidase, site 1	Homo sapiens
80760	inter-alpha (globulin) inhibitor H5	Homo sapiens
55317	chromosome 20 open reading frame 29	Homo sapiens
55596	zinc finger, CCHC domain containing 8	Homo sapiens
10296	macrophage erythroblast attacher	Homo sapiens
1340	cytochrome c oxidase subunit Vib polypeptide 1 (ubiquitous)	Homo sapiens
3267	ArfGAP with FG repeats 1	Homo sapiens
646766	ribosomal protein L35; ribosomal protein L35 pseudogene 1; ribosomal protein L35 pseudogene 2	Homo sapiens
440737	ribosomal protein L35; ribosomal protein L35 pseudogene 1; ribosomal protein L35 pseudogene 2	Homo sapiens
125950	ribonucleoprotein, PTB-binding 1	Homo sapiens
10947	adaptor-related protein complex 3, mu 2 subunit	Homo sapiens
27429	HtrA serine peptidase 2	Homo sapiens
55203	leucine-rich repeat LGI family, member 2	Homo sapiens
116461	tRNA splicing endonuclease 15 homolog (S. cerevisiae)	Homo sapiens
5929	retinoblastoma binding protein 5	Homo sapiens
390282	eukaryotic translation initiation factor 3, subunit F; similar to hCG2040283	Homo sapiens

26160	intraflagellar transport 172 homolog (Chlamydomonas)	Homo sapiens
81608	FIP1 like 1 (S. cerevisiae)	Homo sapiens
10671	dynactin 6	Homo sapiens
727826	ribosomal protein S11 pseudogene 5; ribosomal protein S11	Homo sapiens
286749	stonin 1; STON1-GTF2A1L readthrough transcript; general transcription factor IIA, 1-like	Homo sapiens
11037	stonin 1; STON1-GTF2A1L readthrough transcript; general transcription factor IIA, 1-like	Homo sapiens
11036	stonin 1; STON1-GTF2A1L readthrough transcript; general transcription factor IIA, 1-like	Homo sapiens
5875	Rab geranylgeranyltransferase, alpha subunit	Homo sapiens
51631	LUC7-like 2 (S. cerevisiae)	Homo sapiens
7328	ubiquitin-conjugating enzyme E2H (UBC8 homolog, yeast)	Homo sapiens
6791	aurora kinase A; aurora kinase A pseudogene 1	Homo sapiens
6790	aurora kinase A; aurora kinase A pseudogene 1	Homo sapiens
114884	oxysterol binding protein-like 10	Homo sapiens
5082	phosducin-like	Homo sapiens
10906	TRAF-type zinc finger domain containing 1	Homo sapiens
100130711	similar to hCG1778032; RAB35, member RAS oncogene family	Homo sapiens
79058	alveolar soft part sarcoma chromosome region, candidate 1	Homo sapiens
9913	suppressor of Ty 7 (S. cerevisiae)-like	Homo sapiens
83448	pseudouridylate synthase 7 homolog (S. cerevisiae)-like	Homo sapiens
4796	nuclear factor of kappa light polypeptide gene enhancer in B-cells inhibitor-like 2	Homo sapiens
54859	chromosome 3 open reading frame 75	Homo sapiens
51098	intraflagellar transport 52 homolog (Chlamydomonas)	Homo sapiens
9158	fibroblast growth factor (acidic) intracellular binding protein	Homo sapiens
732446	similar to growth arrest-specific 6; growth arrest-specific 6	Homo sapiens
84062	dystrobrevin binding protein 1	Homo sapiens
57452	UDP-N-acetyl-alpha-D-galactosamine:polypeptide N-acetylgalactosaminyltransferase-like 1	Homo sapiens
205428	chromosome 3 open reading frame 58	Homo sapiens
10283	serologically defined colon cancer antigen 10	Homo sapiens
9698	pumilio homolog 1 (Drosophila)	Homo sapiens
90161	heparan sulfate 6-O-sulfotransferase 2	Homo sapiens
643949	ribosomal protein, large, P2 pseudogene 3; ribosomal protein, large, P2	Homo sapiens
131870	nudix (nucleoside diphosphate linked moiety X)-type motif 16	Homo sapiens
341378	hypothetical LOC341378; thyroid hormone receptor interactor 11	Homo sapiens
29919	chromosome 18 open reading frame 8	Homo sapiens
55341	large subunit GTPase 1 homolog (S. cerevisiae)	Homo sapiens
64744	small ArfGAP2	Homo sapiens
25962	KIAA1429	Homo sapiens
64943	5'-nucleotidase domain containing 2	Homo sapiens
727896	cysteine and histidine-rich domain (CHORD)-containing 1; cysteine and histidine-rich domain (CHORD)-containing 1 pseudogene	Homo sapiens
25942	SIN3 homolog A, transcription regulator (yeast)	Homo sapiens
134430	WD repeat domain 36	Homo sapiens
392301	solute carrier family 25 (mitochondrial carrier; adenine nucleotide translocator), member 5; solute carrier family 25 (mitochondrial carrier; adenine nucleotide translocator), member 5 pseudogene 8	Homo sapiens
8905	adaptor-related protein complex 1, sigma 2 subunit pseudogene; adaptor-related protein complex 1, sigma 2 subunit	Homo sapiens
653653	adaptor-related protein complex 1, sigma 2 subunit pseudogene; adaptor-related protein complex 1, sigma 2 subunit	Homo sapiens
652826	similar to 26S protease regulatory subunit 6B (MIP224) (MB67-interacting protein) (TAT-binding protein 7) (TBP-7); proteasome (prosome, macropain) 26S subunit, ATPase, 4	Homo sapiens
55055	Zwilch, kinetochore associated, homolog (Drosophila)	Homo sapiens
7517	X-ray repair complementing defective repair in Chinese hamster cells 3	Homo sapiens
57464	family with sequence similarity 40, member B	Homo sapiens
646243	coxsackie virus and adenovirus receptor pseudogene 2; coxsackie virus and adenovirus receptor	Homo sapiens
643167	similar to RNA binding motif protein 39; RNA binding motif protein 39	Homo sapiens
751867	regulator of chromosome condensation 1; SNHG3-RCC1 readthrough transcript	Homo sapiens
6596	helicase-like transcription factor	Homo sapiens
58497	prune homolog (Drosophila)	Homo sapiens
23048	formin binding protein 1	Homo sapiens
9735	kinetochore associated 1	Homo sapiens
23595	origin recognition complex, subunit 3-like (yeast)	Homo sapiens
54552	guanine nucleotide binding protein-like 3 (nucleolar)-like	Homo sapiens
5347	polo-like kinase 1 (Drosophila)	Homo sapiens
25917	THUMP domain containing 3	Homo sapiens
5612	protein-kinase, interferon-inducible double stranded RNA dependent inhibitor, repressor of (P58 repressor)	Homo sapiens
55011	PIH1 domain containing 1	Homo sapiens
51808	phosphorylated adaptor for RNA export	Homo sapiens
25885	polymerase (RNA) I polypeptide A, 194kDa	Homo sapiens
6595	SWI/SNF related, matrix associated, actin dependent regulator of chromatin, subfamily a, member 2	Homo sapiens
283358	beta-1,4-N-acetyl-galactosaminyl transferase 3	Homo sapiens

54534	mitochondrial ribosomal protein L50	Homo sapiens
1478	cleavage stimulation factor, 3' pre-RNA, subunit 2, 64kDa	Homo sapiens
9746	calsyntenin 3	Homo sapiens
7555	CCHC-type zinc finger, nucleic acid binding protein	Homo sapiens
644131	similar to chaperonin containing TCP1, subunit 8 (theta); chaperonin containing TCP1, subunit 8 (theta)	Homo sapiens
5099	protocadherin 7	Homo sapiens
84916	cirrhosis, autosomal recessive 1A (cirhin)	Homo sapiens
83956	Rac GTPase activating protein 1 pseudogene; Rac GTPase activating protein 1	Homo sapiens
1838	dystrobrevin, beta	Homo sapiens
6827	suppressor of Ty 4 homolog 1 (S. cerevisiae)	Homo sapiens
100133770	hypothetical protein LOC100133770; vacuolar protein sorting 35 homolog (S. cerevisiae)	Homo sapiens
9683	NEDD4 binding protein 1	Homo sapiens
157	adrenergic, beta, receptor kinase 2	Homo sapiens
100293737	similar to protein phosphatase 1 regulatory subunit 14B	Homo sapiens
9882	TBC1 domain family, member 4	Homo sapiens
728860	karyopherin alpha 2 (RAG cohort 1, importin alpha 1); karyopherin alpha-2 subunit like	Homo sapiens
119358	small nuclear ribonucleoprotein D2 polypeptide 16.5kDa; similar to hCG2040270	Homo sapiens
148113	cartilage intermediate layer protein 2	Homo sapiens
79657	RNA polymerase II associated protein 3	Homo sapiens
8458	transcription termination factor, RNA polymerase II	Homo sapiens
93323	HAUS augmin-like complex, subunit 8	Homo sapiens
55179	Fas apoptotic inhibitory molecule	Homo sapiens
3832	kinesin family member 11	Homo sapiens
892	cyclin C	Homo sapiens
55818	lysine (K)-specific demethylase 3A	Homo sapiens
80018	chromosome 12 open reading frame 30	Homo sapiens
11319	ecdysoneless homolog (Drosophila)	Homo sapiens
79018	chromosome 17 open reading frame 39	Homo sapiens
9423	netrin 1	Homo sapiens
10885	WD repeat domain 3	Homo sapiens
23130	ATG2 autophagy related 2 homolog A (S. cerevisiae)	Homo sapiens
10570	dihydropyrimidinase-like 4	Homo sapiens
4745	NEL-like 1 (chicken)	Homo sapiens
55164	SHQ1 homolog (S. cerevisiae)	Homo sapiens
90293	kelch-like 13 (Drosophila)	Homo sapiens
642585	voltage-dependent anion channel 1; similar to voltage-dependent anion channel 1	Homo sapiens
90850	zinc finger protein 598	Homo sapiens
8148	TAF15 RNA polymerase II, TATA box binding protein (TBP)-associated factor, 68kDa	Homo sapiens
2962	general transcription factor IIF, polypeptide 1, 74kDa	Homo sapiens
11011	tousled-like kinase 2	Homo sapiens
79003	MIS12, MIND kinetochore complex component, homolog (S. pombe)	Homo sapiens
51164	dynactin 4 (p62)	Homo sapiens
91039	dipeptidyl-peptidase 9	Homo sapiens
57099	apoptosis, caspase activation inhibitor	Homo sapiens
1982	eukaryotic translation initiation factor 4 gamma, 2	Homo sapiens
645630	ribosomal protein S13 pseudogene 8; ribosomal protein S13; ribosomal protein S13 pseudogene 2	Homo sapiens
729236	ribosomal protein S13 pseudogene 8; ribosomal protein S13; ribosomal protein S13 pseudogene 2	Homo sapiens
122830	N-acetyltransferase 12 (GCN5-related, putative)	Homo sapiens
28998	mitochondrial ribosomal protein L13	Homo sapiens
55726	chromosome 12 open reading frame 11	Homo sapiens
9648	GRIP and coiled-coil domain containing 2	Homo sapiens
8120	adaptor-related protein complex 3, beta 2 subunit	Homo sapiens
124997	WD repeat domain 81	Homo sapiens
100134387	similar to DEAH (Asp-Glu-Ala-His) box polypeptide 40; DEAH (Asp-Glu-Ala-His) box polypeptide 40	Homo sapiens
79665	similar to DEAH (Asp-Glu-Ala-His) box polypeptide 40; DEAH (Asp-Glu-Ala-His) box polypeptide 40	Homo sapiens
79042	tRNA splicing endonuclease 34 homolog (S. cerevisiae)	Homo sapiens
54939	COMM domain containing 4	Homo sapiens
79047	potassium channel tetramerisation domain containing 15	Homo sapiens
9874	tousled-like kinase 1	Homo sapiens
389677	RNA binding motif protein 12B	Homo sapiens
79109	mitogen-activated protein kinase associated protein 1	Homo sapiens
9530	BCL2-associated athanogene 4	Homo sapiens
23085	ELKS/RAB6-interacting/CAST family member 1	Homo sapiens
374291	NADH dehydrogenase (ubiquinone) Fe-S protein 7, 20kDa (NADH-coenzyme Q reductase)	Homo sapiens
55114	Rho GTPase activating protein 17	Homo sapiens
650405	similar to hCG26659; immunoglobulin kappa constant; similar to Ig kappa chain V-I region HK102 precursor	Homo sapiens
652493	similar to hCG26659; immunoglobulin kappa constant; similar to Ig kappa chain V-I region HK102 precursor	Homo sapiens

10978	CLP1, cleavage and polyadenylation factor I subunit, homolog (S. cerevisiae)	Homo sapiens
51663	zinc finger RNA binding protein	Homo sapiens
79596	ring finger protein 219	Homo sapiens
79670	zinc finger, CCHC domain containing 6	Homo sapiens
23114	neurofascin homolog (chicken)	Homo sapiens
140459	ankyrin repeat and SOCS box-containing 6	Homo sapiens
5433	polymerase (RNA) II (DNA directed) polypeptide D	Homo sapiens
50717	WD repeat domain 42A	Homo sapiens
10523	calcium homeostasis endoplasmic reticulum protein	Homo sapiens
51256	TBC1 domain family, member 7	Homo sapiens
646819	ribosomal protein S15a pseudogene 17; ribosomal protein S15a pseudogene 19; ribosomal protein S15a pseudogene 12; ribosomal protein S15a pseudogene 24; ribosomal protein S15a pseudogene 11; ribosomal protein S15a	Homo sapiens
644790	ribosomal protein S15a pseudogene 17; ribosomal protein S15a pseudogene 19; ribosomal protein S15a pseudogene 12; ribosomal protein S15a pseudogene 24; ribosomal protein S15a pseudogene 11; ribosomal protein S15a	Homo sapiens
648729	ribosomal protein S15a pseudogene 17; ribosomal protein S15a pseudogene 19; ribosomal protein S15a pseudogene 12; ribosomal protein S15a pseudogene 24; ribosomal protein S15a pseudogene 11; ribosomal protein S15a	Homo sapiens
391656	ribosomal protein S15a pseudogene 17; ribosomal protein S15a pseudogene 19; ribosomal protein S15a pseudogene 12; ribosomal protein S15a pseudogene 24; ribosomal protein S15a pseudogene 11; ribosomal protein S15a	Homo sapiens
100129243	ribosomal protein S15a pseudogene 17; ribosomal protein S15a pseudogene 19; ribosomal protein S15a pseudogene 12; ribosomal protein S15a pseudogene 24; ribosomal protein S15a pseudogene 11; ribosomal protein S15a	Homo sapiens
6249	CAP-GLY domain containing linker protein 1	Homo sapiens
9551	ATP synthase, H+ transporting, mitochondrial F0 complex, subunit F2	Homo sapiens
160428	aldehyde dehydrogenase 1 family, member L2	Homo sapiens
6788	serine/threonine kinase 3 (STE20 homolog, yeast)	Homo sapiens
130507	ubiquitin protein ligase E3 component n-recognin 3 (putative)	Homo sapiens
253635	coiled-coil domain containing 75	Homo sapiens
83955	nascent-polypeptide-associated complex alpha polypeptide pseudogene 1	Homo sapiens
1287	collagen, type IV, alpha 5	Homo sapiens
9055	protein regulator of cytokinesis 1	Homo sapiens
51586	mediator complex subunit 15	Homo sapiens
9440	mediator complex subunit 17	Homo sapiens
54517	pseudouridylate synthase 7 homolog (S. cerevisiae)	Homo sapiens
51388	nuclear import 7 homolog (S. cerevisiae)	Homo sapiens
22920	kinesin-associated protein 3	Homo sapiens
5830	peroxisomal biogenesis factor 5	Homo sapiens
55636	chromodomain helicase DNA binding protein 7	Homo sapiens
9801	mitochondrial ribosomal protein L19	Homo sapiens
100133898	anaphase promoting complex subunit 1; similar to anaphase promoting complex subunit 1	Homo sapiens
100133982	anaphase promoting complex subunit 1; similar to anaphase promoting complex subunit 1	Homo sapiens
57728	WD repeat domain 19	Homo sapiens
643668	proteasome (prosome, macropain) 26S subunit, ATPase, 1; similar to protease (prosome, macropain) 26S subunit, ATPase 1	Homo sapiens
6046	bromodomain containing 2	Homo sapiens
83540	NUF2, NDC80 kinetochore complex component, homolog (S. cerevisiae)	Homo sapiens
23609	makorin ring finger protein 2	Homo sapiens
29959	nuclear receptor binding protein 1	Homo sapiens
653226	signal recognition particle 9-like 1; signal recognition particle 9kDa	Homo sapiens
2803	golgi autoantigen, golgin subfamily a, 4	Homo sapiens
51184	GPN-loop GTPase 3	Homo sapiens
112970	KTI12 homolog, chromatin associated (S. cerevisiae)	Homo sapiens
3988	lipase A, lysosomal acid, cholesterol esterase	Homo sapiens
219743	trypsin domain containing 1	Homo sapiens
59277	netrin 4	Homo sapiens
57570	TRM5 tRNA methyltransferase 5 homolog (S. cerevisiae)	Homo sapiens
25853	WD repeat domain 40A	Homo sapiens
7059	thrombospondin 3	Homo sapiens
58986	transmembrane protein 8A	Homo sapiens
85441	peroxisomal proliferator-activated receptor A interacting complex 285	Homo sapiens
10724	meningioma expressed antigen 5 (hyaluronidase)	Homo sapiens
692312	PPAN-P2RY11 readthrough transcript	Homo sapiens
51517	NCK interacting protein with SH3 domain	Homo sapiens
3550	similar to CG18005; IK cytokine, down-regulator of HLA II	Homo sapiens
644456	similar to CG18005; IK cytokine, down-regulator of HLA II	Homo sapiens
84321	similar to THO complex 3; THO complex 3	Homo sapiens
728554	similar to THO complex 3; THO complex 3	Homo sapiens
4928	nucleoporin 98kDa	Homo sapiens
6609	sphingomyelin phosphodiesterase 1, acid lysosomal	Homo sapiens
151162	mannosyl (alpha-1,6-)-glycoprotein beta-1,6-N-acetyl-glucosaminyltransferase; hypothetical LOC151162	Homo sapiens
26292	c-myc binding protein	Homo sapiens
51773	remodeling and spacing factor 1	Homo sapiens
56006	chromosome 19 open reading frame 61	Homo sapiens
9500	melanoma antigen family D, 1	Homo sapiens

9631	nucleoporin 155kDa	Homo sapiens
284403	WD repeat domain 62	Homo sapiens
10243	gephyrin	Homo sapiens
55827	IQ motif and WD repeats 1	Homo sapiens
79829	N-acetyltransferase 11 (GCN5-related, putative)	Homo sapiens
5886	RAD23 homolog A (S. cerevisiae)	Homo sapiens
23587	chromosome 17 open reading frame 81	Homo sapiens
94104	chromosome 21 open reading frame 66	Homo sapiens
114880	oxysterol binding protein-like 6	Homo sapiens
51605	tRNA methyltransferase 6 homolog (S. cerevisiae)	Homo sapiens
223117	sema domain, immunoglobulin domain (Ig), short basic domain, secreted, (semaphorin) 3D	Homo sapiens
55212	Bardet-Biedl syndrome 7	Homo sapiens
8409	ubiquitously-expressed transcript	Homo sapiens
2647	biogenesis of lysosomal organelles complex-1, subunit 1	Homo sapiens
80274	signal peptide, CUB domain, EGF-like 1	Homo sapiens
79989	tetratricopeptide repeat domain 26	Homo sapiens
51122	COMM domain containing 2	Homo sapiens
1643	damage-specific DNA binding protein 2, 48kDa	Homo sapiens
8725	chromosome 19 open reading frame 2	Homo sapiens
60682	small ArfGAP 1	Homo sapiens
9212	aurora kinase B	Homo sapiens
56339	methyltransferase like 3	Homo sapiens
8266	ubiquitin-like 4A	Homo sapiens
100131196	ribosomal protein S25 pseudogene 8; ribosomal protein S25	Homo sapiens
55658	ring finger protein 126	Homo sapiens
51260	chromosome X open reading frame 26	Homo sapiens
728953	ribosomal protein S19 pseudogene 3; ribosomal protein S19	Homo sapiens
26031	oxysterol binding protein-like 3	Homo sapiens
56342	peter pan homolog (Drosophila)	Homo sapiens
220134	chromosome 18 open reading frame 24	Homo sapiens
9855	FERM, RhoGEF and pleckstrin domain protein 2	Homo sapiens
29099	COMM domain containing 9	Homo sapiens
27075	tetraspanin 13	Homo sapiens
26035	glucuronic acid epimerase	Homo sapiens
8315	BRCA1 associated protein	Homo sapiens
129831	RNA binding motif protein 45	Homo sapiens
288	ankyrin 3, node of Ranvier (ankyrin G)	Homo sapiens
2508	ferritin, heavy polypeptide 1; ferritin, heavy polypeptide-like 16; similar to ferritin, heavy polypeptide 1; ferritin, heavy polypeptide-like 3 pseudogene	Homo sapiens
2498	ferritin, heavy polypeptide 1; ferritin, heavy polypeptide-like 16; similar to ferritin, heavy polypeptide 1; ferritin, heavy polypeptide-like 3 pseudogene	Homo sapiens
729009	ferritin, heavy polypeptide 1; ferritin, heavy polypeptide-like 16; similar to ferritin, heavy polypeptide 1; ferritin, heavy polypeptide-like 3 pseudogene	Homo sapiens
115752	DIS3 mitotic control homolog (S. cerevisiae)-like	Homo sapiens
2130	similar to Ewing sarcoma breakpoint region 1; Ewing sarcoma breakpoint region 1	Homo sapiens
284685	similar to Ewing sarcoma breakpoint region 1; Ewing sarcoma breakpoint region 1	Homo sapiens
286826	lin-9 homolog (C. elegans)	Homo sapiens
7024	transcription factor CP2	Homo sapiens
90480	growth arrest and DNA-damage-inducible, gamma interacting protein 1	Homo sapiens
