# Supplementary material for: Identification and Characterization of 293T Cell-Derived Exosomes by Profiling the Protein, mRNA and MicroRNA Components
Source: PLoS One. 2016 Sep 20;11(9):e0163043. doi: 10.1371/journal.pone.0163043 (PMC5029934; doi:10.1371/journal.pone.0163043)
Supplement: S4 Table — (PDF) [file pone.0163043.s005.pdf]

| ENTREZ_GENE_ID | Name | Species |
|---|---|---|
| 26057 | ankyrin repeat domain 17 | Homo sapiens |
| 29914 | UbiA prenyltransferase domain containing 1 | Homo sapiens |
| 10845 | ClpX caseinolytic peptidase X homolog (E. coli) | Homo sapiens |
| 84942 | WD repeat domain 73 | Homo sapiens |
| 10081 | programmed cell death 7 | Homo sapiens |
| 145389 | solute carrier family 38, member 6 | Homo sapiens |
| 79892 | chromosome 10 open reading frame 119 | Homo sapiens |
| 200894 | ADP-ribosylation factor-like 13B | Homo sapiens |
| 64895 | poly(A) polymerase gamma | Homo sapiens |
| 23394 | activity-dependent neuroprotector homeobox | Homo sapiens |
| 9282 | mediator complex subunit 14 | Homo sapiens |
| 5394 | exosome component 10 | Homo sapiens |
| 55252 | additional sex combs like 2 (Drosophila) | Homo sapiens |
| 100 | adenosine deaminase | Homo sapiens |
| 317749 | dehydrogenase/reductase (SDR family) member 4 like 2 | Homo sapiens |
| 9778 | KIAA0232 | Homo sapiens |
| 29094 | galectin-related protein | Homo sapiens |
| 340542 | brain expressed, X-linked 5 | Homo sapiens |
| 54776 | protein phosphatase 1, regulatory (inhibitor) subunit 12C | Homo sapiens |
| 9570 | golgi SNAP receptor complex member 2 | Homo sapiens |
| 79854 | non-protein coding RNA 115 | Homo sapiens |
| 26093 | coiled-coil domain containing 9 | Homo sapiens |
| 128866 | chromatin modifying protein 4B | Homo sapiens |
| 90121 | TSR2, 20S rRNA accumulation, homolog (S. cerevisiae) | Homo sapiens |
| 64215 | DnaJ (Hsp40) homolog, subfamily C, member 1 | Homo sapiens |
| 55151 | transmembrane protein 38B | Homo sapiens |
| 2734 | golgi apparatus protein 1 | Homo sapiens |
| 55012 | protein phosphatase 2 (formerly 2A), regulatory subunit B'', gamma | Homo sapiens |
| 440295 | similar to golgi autoantigen, golgin subfamily a-like; hypothetical protein LOC440295; hypothetical LOC642346; golgin subfamily A member 6-like protein 10-like | Homo sapiens |
| 79590 | mitochondrial ribosomal protein L24 | Homo sapiens |
| 5165 | pyruvate dehydrogenase kinase, isozyme 3 | Homo sapiens |
| 10529 | nebulette | Homo sapiens |
| 388 | ras homolog gene family, member B | Homo sapiens |
| 286144 | chromosome 8 open reading frame 83 | Homo sapiens |
| 100302285 | microRNA 1244 | Homo sapiens |
| 254225 | ring finger protein 169 | Homo sapiens |
| 80853 | jumonji C domain containing histone demethylase 1 homolog D (S. cerevisiae) | Homo sapiens |
| 84181 | chromodomain helicase DNA binding protein 6 | Homo sapiens |
| 79720 | vacuolar protein sorting 37 homolog B (S. cerevisiae) | Homo sapiens |
| 26065 | LSM14A, SCD6 homolog A (S. cerevisiae) | Homo sapiens |
| 10291 | splicing factor 3a, subunit 1, 120kDa | Homo sapiens |
| 55119 | PRP38 pre-mRNA processing factor 38 (yeast) domain containing B | Homo sapiens |
| 154743 | chromosome 7 open reading frame 60 | Homo sapiens |
| 375190 | chromosome 2 open reading frame 84; hypothetical protein LOC375190 | Homo sapiens |
| 55327 | lin-7 homolog C (C. elegans) | Homo sapiens |
| 23658 | LSM5 homolog, U6 small nuclear RNA associated (S. cerevisiae) | Homo sapiens |
| 57185 | NIPA-like domain containing 3 | Homo sapiens |
| 27333 | golgi integral membrane protein 4 | Homo sapiens |
| 8050 | pyruvate dehydrogenase complex, component X | Homo sapiens |
| 22836 | Rho-related BTB domain containing 3 | Homo sapiens |
| 26286 | ADP-ribosylation factor GTPase activating protein 3 | Homo sapiens |
| 10936 | G protein-coupled receptor 75 | Homo sapiens |
| 2992 | glycogenin 1 | Homo sapiens |
| 25980 | chromosome 20 open reading frame 4 | Homo sapiens |
| 89958 | chromosome 9 open reading frame 140 | Homo sapiens |
| 79823 | chromosome 2 open reading frame 34 | Homo sapiens |
| 6936 | chromosome 2 open reading frame 3 | Homo sapiens |
| 8867 | synaptojanin 1 | Homo sapiens |
| 51031 | glyoxalase domain containing 4 | Homo sapiens |
| 57215 | THAP domain containing 11 | Homo sapiens |
| 10449 | hypothetical LOC648603; acetyl-Coenzyme A acyltransferase 2 | Homo sapiens |
| 55810 | forkhead box J2 | Homo sapiens |
| 6940 | zinc finger protein 354A | Homo sapiens |
| 9480 | one cut homeobox 2 | Homo sapiens |
| 26115 | tetratricopeptide repeat, ankyrin repeat and coiled-coil containing 2 | Homo sapiens |
| 84282 | ring finger protein 135 | Homo sapiens |
| 50515 | carbohydrate (chondroitin 4) sulfotransferase 11 | Homo sapiens |
| 100133660 | hypothetical LOC100133660 | Homo sapiens |
| 3799 | kinesin family member 5B | Homo sapiens |
| 55812 | spermatogenesis associated 7 | Homo sapiens |
| 5495 | protein phosphatase 1B (formerly 2C), magnesium-dependent, beta isoform | Homo sapiens |
| 9349 | ribosomal protein L23 pseudogene 6; ribosomal protein L23 | Homo sapiens |
| 26999 | cytoplasmic FMR1 interacting protein 2 | Homo sapiens |
| 440248 | hect domain and RLD 2 pseudogene | Homo sapiens |
| 10641 | tumor suppressor candidate 4 | Homo sapiens |

| | | |
|---|---|---|
| 4898 | nardilysin (N-arginine dibasic convertase) | Homo sapiens |
| 10905 | mannosidase, alpha, class 1A, member 2 | Homo sapiens |
| 84305 | within bgcn homolog (Drosophila) | Homo sapiens |
| 114785 | methyl-CpG binding domain protein 6 | Homo sapiens |
| 9929 | Josephin domain containing 1 | Homo sapiens |
| 169200 | transmembrane protein 64 | Homo sapiens |
| 64151 | non-SMC condensin I complex, subunit G | Homo sapiens |
| 10527 | importin 7 | Homo sapiens |
| 528 | ATPase, H+ transporting, lysosomal 42kDa, V1 subunit C1 | Homo sapiens |
| 79672 | fructosamine 3 kinase related protein | Homo sapiens |
| 7752 | zinc finger protein 200 | Homo sapiens |
| 9364 | RAB28, member RAS oncogene family | Homo sapiens |
| 6130 | ribosomal protein L7a pseudogene 70; ribosomal protein L7a; ribosomal protein L7a pseudogene 30; ribosomal protein L7a pseudogene 66; ribosomal protein L7a pseudogene 27; ribosomal protein L7a pseudogene 11; ribosomal protein L7a pseudogene 62 | Homo sapiens |
| 25939 | SAM domain and HD domain 1 | Homo sapiens |
| 10635 | RAD51 associated protein 1 | Homo sapiens |
| 100292863 | similar to erlectin | Homo sapiens |
| 7353 | ubiquitin fusion degradation 1 like (yeast) | Homo sapiens |
| 339448 | chromosome 1 open reading frame 174 | Homo sapiens |
| 7528 | YY1 transcription factor | Homo sapiens |
| 5908 | RAP1B, member of RAS oncogene family | Homo sapiens |
| 153396 | transmembrane protein 161B | Homo sapiens |
| 57602 | ubiquitin specific peptidase 36 | Homo sapiens |
| 7392 | upstream transcription factor 2, c-fos interacting | Homo sapiens |
| 57019 | cytokine induced apoptosis inhibitor 1; cytokine induced apoptosis inhibitor 1 pseudogene | Homo sapiens |
| 10197 | proteasome (prosome, macropain) activator subunit 3 (PA28 gamma; Ki) | Homo sapiens |
| 79183 | tocopherol (alpha) transfer protein-like | Homo sapiens |
| 201475 | RAB12, member RAS oncogene family | Homo sapiens |
| 55744 | chromosome 7 open reading frame 44 | Homo sapiens |
| 7342 | upstream binding protein 1 (LBP-1a) | Homo sapiens |
| 11097 | nucleoporin like 2 | Homo sapiens |
| 401166 | FLJ44896 protein | Homo sapiens |
| 7692 | zinc finger protein 133 | Homo sapiens |
| 3915 | laminin, gamma 1 (formerly LAMB2) | Homo sapiens |
| 29107 | NTF2-like export factor 1 | Homo sapiens |
| 6342 | sterol carrier protein 2 | Homo sapiens |
| 56970 | ataxin 7-like 3 | Homo sapiens |
| 4899 | nuclear respiratory factor 1 | Homo sapiens |
| 1152 | creatine kinase, brain | Homo sapiens |
| 7536 | splicing factor 1 | Homo sapiens |
| 118460 | exosome component 6 | Homo sapiens |
| 7919 | HLA-B associated transcript 1 | Homo sapiens |
| 11127 | kinesin family member 3A | Homo sapiens |
| 51001 | MTERF domain containing 1 | Homo sapiens |
| 100288152 | hypothetical protein LOC100288152 | Homo sapiens |
| 90639 | COX19 cytochrome c oxidase assembly homolog (S. cerevisiae) | Homo sapiens |
| 11101 | arginyltransferase 1 | Homo sapiens |
| 54948 | mitochondrial ribosomal protein L16 | Homo sapiens |
| 5496 | protein phosphatase 1G (formerly 2C), magnesium-dependent, gamma isoform | Homo sapiens |
| 79701 | chromosome 17 open reading frame 101 | Homo sapiens |
| 55105 | G patch domain containing 2 | Homo sapiens |
| 55973 | B-cell receptor-associated protein 29 | Homo sapiens |
| 284098 | phosphatidylinositol glycan anchor biosynthesis, class W | Homo sapiens |
| 2733 | GLE1 RNA export mediator homolog (yeast) | Homo sapiens |
| 55181 | chromosome 17 open reading frame 71 | Homo sapiens |
| 10672 | guanine nucleotide binding protein (G protein), alpha 13 | Homo sapiens |
| 84964 | alkB, alkylation repair homolog 6 (E. coli) | Homo sapiens |
| 57291 | KIAA0114 | Homo sapiens |
| 7095 | SEC62 homolog (S. cerevisiae) | Homo sapiens |
| 1195 | CDC-like kinase 1 | Homo sapiens |
| 29883 | CCR4-NOT transcription complex, subunit 7 | Homo sapiens |
| 25996 | REX2, RNA exonuclease 2 homolog (S. cerevisiae) | Homo sapiens |
| 23210 | jumonji domain containing 6 | Homo sapiens |
| 53335 | B-cell CLL/lymphoma 11A (zinc finger protein) | Homo sapiens |
| 7748 | zinc finger protein 195 | Homo sapiens |
| 831 | calpastatin | Homo sapiens |
| 23381 | Smg-5 homolog, nonsense mediated mRNA decay factor (C. elegans) | Homo sapiens |
| 115123 | membrane-associated ring finger (C3HC4) 3 | Homo sapiens |
| 5096 | propionyl Coenzyme A carboxylase, beta polypeptide | Homo sapiens |
| 26073 | polymerase (DNA-directed), delta interacting protein 2 | Homo sapiens |
| 129401 | nucleoporin 35kDa | Homo sapiens |
| 6650 | small optic lobes homolog (Drosophila) | Homo sapiens |
| 84844 | PHD finger protein 5A | Homo sapiens |
| 148223 | chromosome 19 open reading frame 25 | Homo sapiens |
| 1911 | polyhomeotic homolog 1B (Drosophila); polyhomeotic homolog 1 (Drosophila) | Homo sapiens |

| | | |
|---|---|---|
| 65056 | GC-rich promoter binding protein 1 | Homo sapiens |
| 54545 | myotubularin related protein 12 | Homo sapiens |
| 55898 | unc-45 homolog A (C. elegans) | Homo sapiens |
| 26260 | F-box protein 25 | Homo sapiens |
| 8760 | CDP-diacylglycerol synthase (phosphatidate cytidylyltransferase) 2 | Homo sapiens |
| 3212 | homeobox B2 | Homo sapiens |
| 9441 | mediator complex subunit 26 | Homo sapiens |
| 9371 | kinesin family member 3B | Homo sapiens |
| 54840 | aprataxin | Homo sapiens |
| 7750 | zinc finger, MYM-type 2 | Homo sapiens |
| 51657 | serine/threonine/tyrosine interacting-like 1 | Homo sapiens |
| 10274 | stromal antigen 1 | Homo sapiens |
| 137492 | vacuolar protein sorting 37 homolog A (S. cerevisiae) | Homo sapiens |
| 153579 | butyrophilin-like 9 | Homo sapiens |
| 4149 | MYC associated factor X | Homo sapiens |
| 8813 | dolichyl-phosphate mannosyltransferase polypeptide 1, catalytic subunit | Homo sapiens |
| 57482 | KIAA1211 | Homo sapiens |
| 2020 | engrailed homeobox 2 | Homo sapiens |
| 90933 | tripartite motif-containing 41 | Homo sapiens |
| 22903 | BTB (POZ) domain containing 3 | Homo sapiens |
| 374986 | family with sequence similarity 73, member A | Homo sapiens |
| 5001 | origin recognition complex, subunit 5-like (yeast) | Homo sapiens |
| 706 | translocator protein (18kDa) | Homo sapiens |
| 84307 | zinc finger protein 397 | Homo sapiens |
| 1124 | chimerin (chimaerin) 2 | Homo sapiens |
| 6897 | threonyl-tRNA synthetase | Homo sapiens |
| 142678 | mindbomb homolog 2 (Drosophila) | Homo sapiens |
| 51422 | protein kinase, AMP-activated, gamma 2 non-catalytic subunit | Homo sapiens |
| 51095 | tRNA nucleotidyl transferase, CCA-adding, 1 | Homo sapiens |
| 54471 | Smith-Magenis syndrome chromosome region, candidate 7-like | Homo sapiens |
| 613037 | nuclear pore complex interacting protein pseudogene | Homo sapiens |
| 348995 | nucleoporin 43kDa | Homo sapiens |
| 55177 | family with sequence similarity 82, member A2 | Homo sapiens |
| 192683 | secretory carrier membrane protein 5 | Homo sapiens |
| 84284 | chromosome 1 open reading frame 57 | Homo sapiens |
| 203427 | solute carrier family 25, member 43 | Homo sapiens |
| 26960 | neurobeachin | Homo sapiens |
| 81563 | chromosome 1 open reading frame 21 | Homo sapiens |
| 7923 | hydroxysteroid (17-beta) dehydrogenase 8 | Homo sapiens |
| 54470 | similar to armadillo repeat containing, X-linked 6; armadillo repeat containing, X-linked 6 | Homo sapiens |
| 9819 | TSC22 domain family, member 2 | Homo sapiens |
| 10907 | thioredoxin-like 4A | Homo sapiens |
| 5903 | RAN binding protein 2 | Homo sapiens |
| 23560 | GTP binding protein 4 | Homo sapiens |
| 3043 | hemoglobin, beta | Homo sapiens |
| 23376 | KIAA0776 | Homo sapiens |
| 1633 | deoxycytidine kinase | Homo sapiens |
| 55780 | chromosome 6 open reading frame 70 | Homo sapiens |
| 5937 | RNA binding motif, single stranded interacting protein 1 | Homo sapiens |
| 7343 | upstream binding transcription factor, RNA polymerase I | Homo sapiens |
| 54431 | DnaJ (Hsp40) homolog, subfamily C, member 10 | Homo sapiens |
| 5223 | phosphoglycerate mutase 1 (brain) | Homo sapiens |
| 9520 | hypothetical protein FLJ11822; aminopeptidase puromycin sensitive | Homo sapiens |
| 25956 | SEC31 homolog B (S. cerevisiae) | Homo sapiens |
| 57688 | zinc finger, SWIM-type containing 6 | Homo sapiens |
| 9184 | budding uninhibited by benzimidazoles 3 homolog (yeast) | Homo sapiens |
| 80264 | zinc finger protein 430 | Homo sapiens |
| 51805 | coenzyme Q3 homolog, methyltransferase (S. cerevisiae) | Homo sapiens |
| 967 | CD63 molecule | Homo sapiens |
| 126789 | pseudouridylate synthase-like 1 | Homo sapiens |
| 8896 | BUD31 homolog (S. cerevisiae) | Homo sapiens |
| 284184 | chromosome 17 open reading frame 89 | Homo sapiens |
| 1540 | cylindromatosis (turban tumor syndrome) | Homo sapiens |
| 30851 | Tax1 (human T-cell leukemia virus type I) binding protein 3 | Homo sapiens |
| 535 | ATPase, H+ transporting, lysosomal V0 subunit a1 | Homo sapiens |
| 10664 | CCCTC-binding factor (zinc finger protein) | Homo sapiens |
| 54205 | cytochrome c, somatic | Homo sapiens |
| 7988 | zinc finger protein 212 | Homo sapiens |
| 57610 | RAN binding protein 10 | Homo sapiens |
| 51094 | adiponectin receptor 1 | Homo sapiens |
| 23475 | quinolinate phosphoribosyltransferase | Homo sapiens |
| 84967 | LSM10, U7 small nuclear RNA associated | Homo sapiens |
| 51440 | hippocalcin like 4 | Homo sapiens |
| 5527 | protein phosphatase 2, regulatory subunit B', gamma isoform | Homo sapiens |
| 58485 | trafficking protein particle complex 1 | Homo sapiens |
| 79680 | chromosome 22 open reading frame 29 | Homo sapiens |

| | | |
|---|---|---|
| 7013 | similar to telomeric repeat binding factor (NIMA-interacting) 1; telomeric repeat binding factor (NIMA-interacting) 1; telomeric repeat binding factor (NIMA-interacting) 1 pseudogene | Homo sapiens |
| 388969 | chromosome 2 open reading frame 68 | Homo sapiens |
| 7332 | ubiquitin-conjugating enzyme E2L 3 | Homo sapiens |
| 83742 | hypothetical LOC100270710; MARVEL domain containing 1 | Homo sapiens |
| 133015 | PARK2 co-regulated-like | Homo sapiens |
| 55625 | zinc finger, DHHC-type containing 7 | Homo sapiens |
| 55773 | TBC1 domain family, member 23 | Homo sapiens |
| 283209 | phosphoglucomutase 2-like 1 | Homo sapiens |
| 26503 | solute carrier family 17 (anion/sugar transporter), member 5 | Homo sapiens |
| 9703 | KIAA0100 | Homo sapiens |
| 1979 | eukaryotic translation initiation factor 4E binding protein 2 | Homo sapiens |
| 8777 | multiple PDZ domain protein | Homo sapiens |
| 148423 | chromosome 1 open reading frame 52 | Homo sapiens |
| 100134361 | similar to hCG1811002 | Homo sapiens |
| 122769 | peptidylprolyl isomerase (cyclophilin)-like 5 | Homo sapiens |
| 9326 | zinc finger, HIT type 3 | Homo sapiens |
| 54971 | BTG3 associated nuclear protein | Homo sapiens |
| 66005 | chitinase domain containing 1 | Homo sapiens |
| 6147 | ribosomal protein L23a pseudogene 63; ribosomal protein L23a pseudogene 75; ribosomal protein L23a pseudogene 37; ribosomal protein L23a pseudogene 65; ribosomal protein L23a pseudogene 43; ribosomal protein L23a pseudogene 44; ribosomal protein L23a | Homo sapiens |
| 26056 | RAB11 family interacting protein 5 (class I) | Homo sapiens |
| 4116 | mago-nashi homolog, proliferation-associated (Drosophila) | Homo sapiens |
| 117177 | RAB3A interacting protein (rabin3) | Homo sapiens |
| 152217 | hypothetical LOC152217 | Homo sapiens |
| 327 | N-acylaminoacyl-peptide hydrolase | Homo sapiens |
| 6594 | SWI/SNF related, matrix associated, actin dependent regulator of chromatin, subfamily a, member 1 | Homo sapiens |
| 3843 | importin 5 | Homo sapiens |
| 256586 | LysM, putative peptidoglycan-binding, domain containing 2 | Homo sapiens |
| 6533 | solute carrier family 6 (neurotransmitter transporter, taurine), member 6 | Homo sapiens |
| 11160 | ER lipid raft associated 2 | Homo sapiens |
| 6651 | SON DNA binding protein | Homo sapiens |
| 643253 | chaperonin containing TCP1, subunit 6 (zeta) pseudogene 1 | Homo sapiens |
| 23235 | salt-inducible kinase 2 | Homo sapiens |
| 144203 | ovostatin; ovostatin 2 | Homo sapiens |
| 7086 | transketolase | Homo sapiens |
| 9797 | TatD DNase domain containing 2 | Homo sapiens |
| 2631 | glioblastoma amplified sequence | Homo sapiens |
| 64897 | chromosome 12 open reading frame 43 | Homo sapiens |
| 54149 | chromosome 21 open reading frame 91 | Homo sapiens |
| 150590 | chromosome 2 open reading frame 15 | Homo sapiens |
| 23287 | ATP/GTP binding protein 1 | Homo sapiens |
| 293 | solute carrier family 25 (mitochondrial carrier; adenine nucleotide translocator), member 6 | Homo sapiens |
| 57575 | protocadherin 10 | Homo sapiens |
| 643155 | chromosome 5 open reading frame 43 | Homo sapiens |
| 51125 | golgi autoantigen, golgin subfamily a, 7 | Homo sapiens |
| 54187 | N-acetylneuraminic acid synthase | Homo sapiens |
| 8888 | minichromosome maintenance complex component 3 associated protein | Homo sapiens |
| 27330 | ribosomal protein S6 kinase, 90kDa, polypeptide 6 | Homo sapiens |
| 81539 | solute carrier family 38, member 1 | Homo sapiens |
| 9117 | SEC22 vesicle trafficking protein homolog C (S. cerevisiae) | Homo sapiens |
| 84789 | hypothetical protein MGC2889 | Homo sapiens |
| 83544 | dynein, axonemal, light chain 1 | Homo sapiens |
| 26128 | KIAA1279 | Homo sapiens |
| 11144 | DMC1 dosage suppressor of mck1 homolog, meiosis-specific homologous recombination (yeast) | Homo sapiens |
| 50848 | F11 receptor | Homo sapiens |
| 220988 | heterogeneous nuclear ribonucleoprotein A3 | Homo sapiens |
| 25900 | intermediate filament family orphan 1 | Homo sapiens |
| 140461 | ankyrin repeat and SOCS box-containing 8 | Homo sapiens |
| 5311 | polycystic kidney disease 2 (autosomal dominant) | Homo sapiens |
| 195827 | chromosome 9 open reading frame 21 | Homo sapiens |
| 767811 | H2B histone family, member X, pseudogene | Homo sapiens |
| 121260 | solute carrier family 15, member 4 | Homo sapiens |
| 51496 | CTD (carboxy-terminal domain, RNA polymerase II, polypeptide A) small phosphatase like 2 | Homo sapiens |
| 51389 | RWD domain containing 1-like 1; RWD domain containing 1 | Homo sapiens |
| 80346 | receptor accessory protein 4 | Homo sapiens |
| 79469 | deleted in lymphocytic leukemia 2 (non-protein coding); deleted in lymphocytic leukemia 2-like | Homo sapiens |
| 8847 | deleted in lymphocytic leukemia 2 (non-protein coding); deleted in lymphocytic leukemia 2-like | Homo sapiens |
| 91801 | alkB, alkylation repair homolog 8 (E. coli) | Homo sapiens |
| 400999 | hypothetical gene supported by AK124342 | Homo sapiens |

| | | |
|---|---|---|
| 5464 | pyrophosphatase (inorganic) 1 | Homo sapiens |
| 9732 | dedicator of cytokinesis 4 | Homo sapiens |
| 54995 | 3-oxoacyl-ACP synthase, mitochondrial | Homo sapiens |
| 92370 | acid phosphatase-like 2 | Homo sapiens |
| 253827 | methionine sulfoxide reductase B3 | Homo sapiens |
| 84617 | tubulin, beta 6 | Homo sapiens |
| 23190 | UBX domain protein 4 | Homo sapiens |
| 6722 | serum response factor (c-fos serum response element-binding transcription factor) | Homo sapiens |
| 7107 | G protein-coupled receptor 137B | Homo sapiens |
| 23588 | kelch domain containing 2 | Homo sapiens |
| 80308 | FAD1 flavin adenine dinucleotide synthetase homolog (S. cerevisiae) | Homo sapiens |
| 9658 | zinc finger protein 516 | Homo sapiens |
| 55610 | coiled-coil domain containing 132 | Homo sapiens |
| 6135 | ribosomal protein L11 | Homo sapiens |
| 55278 | glutaminyl-tRNA synthase (glutamine-hydrolyzing)-like 1 | Homo sapiens |
| 51193 | zinc finger protein 639 | Homo sapiens |
| 2081 | endoplasmic reticulum to nucleus signaling 1 | Homo sapiens |
| 10915 | transcription elongation regulator 1 | Homo sapiens |
| 55135 | WD repeat containing, antisense to TP53 | Homo sapiens |
| 51172 | N-acetylglucosamine-1-phosphodiester alpha-N-acetylglucosaminidase | Homo sapiens |
| 64784 | CREB regulated transcription coactivator 3 | Homo sapiens |
| 26149 | zinc finger protein 658 | Homo sapiens |
| 138716 | chromosome 9 open reading frame 23 | Homo sapiens |
| 5270 | serpin peptidase inhibitor, clade E (nexin, plasminogen activator inhibitor type 1), member 2 | Homo sapiens |
| 51006 | solute carrier family 35, member C2 | Homo sapiens |
| 493754 | RAB guanine nucleotide exchange factor (GEF) 1 pseudogene | Homo sapiens |
| 92259 | mitochondrial ribosomal protein S36 | Homo sapiens |
| 114932 | Morf4 family associated protein 1-like 1 | Homo sapiens |
| 645460 | hypothetical LOC645460 | Homo sapiens |
| 64110 | melanoma antigen family F, 1 | Homo sapiens |
| 7227 | trichorhinophalangeal syndrome I | Homo sapiens |
| 57082 | cancer susceptibility candidate 5 | Homo sapiens |
| 23767 | fibronectin leucine rich transmembrane protein 3 | Homo sapiens |
| 9253 | numb homolog (Drosophila)-like | Homo sapiens |
| 55454 | chondroitin sulfate N-acetylgalactosaminyltransferase 2; novel protein similar to chondroitin sulfate GalNAcT-2 (GALNACT-2) | Homo sapiens |
| 23091 | zinc finger CCCH-type containing 13 | Homo sapiens |
| 56650 | claudin domain containing 1 | Homo sapiens |
| 10399 | guanine nucleotide binding protein (G protein), beta polypeptide 2-like 1 | Homo sapiens |
| 9639 | Rho guanine nucleotide exchange factor (GEF) 10 | Homo sapiens |
| 22843 | protein phosphatase 1E (PP2C domain containing) | Homo sapiens |
| 5717 | proteasome (prosome, macropain) 26S subunit, non-ATPase, 11 | Homo sapiens |
| 23710 | GABA(A) receptors associated protein like 3 (pseudogene); GABA(A) receptor-associated protein like 1 | Homo sapiens |
| 23766 | GABA(A) receptors associated protein like 3 (pseudogene); GABA(A) receptor-associated protein like 1 | Homo sapiens |
| 54815 | GATA zinc finger domain containing 2A | Homo sapiens |
| 51124 | immediate early response 3 interacting protein 1 | Homo sapiens |
| 5782 | protein tyrosine phosphatase, non-receptor type 12 | Homo sapiens |
| 55869 | histone deacetylase 8 | Homo sapiens |
| 2122 | ecotropic viral integration site 1 | Homo sapiens |
| 140890 | splicing factor, arginine/serine-rich 12 | Homo sapiens |
| 55068 | ecto-NOX disulfide-thiol exchanger 1 | Homo sapiens |
| 1727 | cytochrome b5 reductase 3 | Homo sapiens |
| 2653 | similar to Glycine cleavage system H protein, mitochondrial precursor; glycine cleavage system protein H (aminomethyl carrier); similar to Glycine cleavage system H protein, mitochondrial | Homo sapiens |
| 26275 | 3-hydroxyisobutyryl-Coenzyme A hydrolase | Homo sapiens |
| 405 | aryl hydrocarbon receptor nuclear translocator | Homo sapiens |
| 9612 | nuclear receptor co-repressor 2 | Homo sapiens |
| 10642 | insulin-like growth factor 2 mRNA binding protein 1 | Homo sapiens |
| 253260 | RPTOR independent companion of MTOR, complex 2 | Homo sapiens |
| 3312 | heat shock 70kDa protein 8 | Homo sapiens |
| 3417 | isocitrate dehydrogenase 1 (NADP+), soluble | Homo sapiens |
| 9352 | thioredoxin-like 1 | Homo sapiens |
| 6767 | similar to heat shock 70kD protein binding protein; suppression of tumorigenicity 13 (colon carcinoma) (Hsp70 interacting protein) | Homo sapiens |
| 4869 | nucleophosmin 1 (nucleolar phosphoprotein B23, numatrin) pseudogene 21; hypothetical LOC100131044; similar to nucleophosmin 1; nucleophosmin (nucleolar phosphoprotein B23, numatrin) | Homo sapiens |
| 57634 | E1A binding protein p400 | Homo sapiens |
| 317762 | coiled-coil domain containing 85C | Homo sapiens |
| 79818 | zinc finger protein 552 | Homo sapiens |
| 5955 | reticulocalbin 2, EF-hand calcium binding domain | Homo sapiens |
| 117583 | par-3 partitioning defective 3 homolog B (C. elegans) | Homo sapiens |
| 51111 | suppressor of variegation 4-20 homolog 1 (Drosophila) | Homo sapiens |

| | | |
|---|---|---|
| 23131 | G patch domain containing 8 | Homo sapiens |
| 6591 | snail homolog 2 (Drosophila) | Homo sapiens |
| 84262 | proteasome (prosome, macropain) assembly chaperone 3 | Homo sapiens |
| 93487 | mitogen-activated protein kinase 1 interacting protein 1-like | Homo sapiens |
| 6159 | ribosomal protein L29 pseudogene 9; ribosomal protein L29 pseudogene 12; ribosomal protein L29 pseudogene 11; ribosomal protein L29; ribosomal protein L29 pseudogene 26 | Homo sapiens |
| 79583 | hypothetical protein FLJ22167 | Homo sapiens |
| 5357 | plastin 1 (I isoform) | Homo sapiens |
| 3764 | potassium inwardly-rectifying channel, subfamily J, member 8 | Homo sapiens |
| 79902 | nucleoporin 85kDa | Homo sapiens |
| 5681 | protein serine kinase H1 | Homo sapiens |
| 1595 | cytochrome P450, family 51, subfamily A, polypeptide 1 | Homo sapiens |
| 10440 | translocase of inner mitochondrial membrane 17 homolog A (yeast) | Homo sapiens |
| 401152 | chromosome 4 open reading frame 3 | Homo sapiens |
| 79065 | ATG9 autophagy related 9 homolog A (S. cerevisiae) | Homo sapiens |
| 51304 | zinc finger, DHHC-type containing 3 | Homo sapiens |
| 10244 | Rab9 effector protein with kelch motifs | Homo sapiens |
| 64841 | glucosamine-phosphate N-acetyltransferase 1 | Homo sapiens |
| 196527 | anoctamin 6 | Homo sapiens |
| 79676 | 2-oxoglutarate and iron-dependent oxygenase domain containing 2 | Homo sapiens |
| 55793 | family with sequence similarity 63, member A | Homo sapiens |
| 9690 | ubiquitin protein ligase E3C | Homo sapiens |
| 51282 | SCAN domain containing 1 | Homo sapiens |
| 65993 | mitochondrial ribosomal protein S34 | Homo sapiens |
| 1499 | catenin (cadherin-associated protein), beta 1, 88kDa | Homo sapiens |
| 131118 | similar to translocase of the inner mitochondrial membrane 14; DnaJ (Hsp40) homolog, subfamily C, member 19 | Homo sapiens |
| 57186 | chromosome 20 open reading frame 74 | Homo sapiens |
| 10294 | DnaJ (Hsp40) homolog, subfamily A, member 2 | Homo sapiens |
| 58528 | Ras-related GTP binding D | Homo sapiens |
| 79632 | family with sequence similarity 184, member A | Homo sapiens |
| 138199 | chromosome 9 open reading frame 41 | Homo sapiens |
| 149371 | exocyst complex component 8 | Homo sapiens |
| 25907 | transmembrane protein 158 | Homo sapiens |
| 5932 | retinoblastoma binding protein 8 | Homo sapiens |
| 8675 | syntaxin 16 | Homo sapiens |
| 3161 | hyaluronan-mediated motility receptor (RHAMM) | Homo sapiens |
| 79161 | chromosome 7 open reading frame 23 | Homo sapiens |
| 2273 | four and a half LIM domains 1 | Homo sapiens |
| 5432 | polymerase (RNA) II (DNA directed) polypeptide C, 33kDa | Homo sapiens |
| 65998 | hypothetical protein LOC65998 | Homo sapiens |
| 10042 | HMG box domain containing 4 | Homo sapiens |
| 5786 | protein tyrosine phosphatase, receptor type, A | Homo sapiens |
| 3280 | hairy and enhancer of split 1, (Drosophila) | Homo sapiens |
| 9793 | cytoskeleton associated protein 5 | Homo sapiens |
| 727751 | hypothetical LOC727751; hypothetical LOC729353 | Homo sapiens |
| 4208 | myocyte enhancer factor 2C | Homo sapiens |
| 2017 | cortactin | Homo sapiens |
| 1746 | distal-less homeobox 2 | Homo sapiens |
| 5454 | POU class 3 homeobox 2 | Homo sapiens |
| 80184 | centrosomal protein 290kDa | Homo sapiens |
| 79717 | phosphopantothenoylcysteine synthetase | Homo sapiens |
| 9653 | heparan sulfate 2-O-sulfotransferase 1 | Homo sapiens |
| 129642 | membrane bound O-acyltransferase domain containing 2 | Homo sapiens |
| 10051 | structural maintenance of chromosomes 4 | Homo sapiens |
| 3300 | DnaJ (Hsp40) homolog, subfamily B, member 2 | Homo sapiens |
| 10890 | RAB10, member RAS oncogene family | Homo sapiens |
| 84947 | serine active site containing 1 | Homo sapiens |
| 55704 | coiled-coil domain containing 88A | Homo sapiens |
| 522 | ATP synthase, H+ transporting, mitochondrial F0 complex, subunit F6 | Homo sapiens |
| 55090 | mediator complex subunit 9 | Homo sapiens |
| 8398 | phospholipase A2, group VI (cytosolic, calcium-independent) | Homo sapiens |
| 26098 | chromosome 10 open reading frame 137 | Homo sapiens |
| 10490 | vesicle transport through interaction with t-SNAREs homolog 1B (yeast) | Homo sapiens |
| 51029 | PPPDE peptidase domain containing 1 | Homo sapiens |
| 9455 | homer homolog 2 (Drosophila) | Homo sapiens |
| 2744 | glutaminase | Homo sapiens |
| 55722 | centrosomal protein 72kDa | Homo sapiens |
| 2512 | similar to ferritin, light polypeptide; ferritin, light polypeptide | Homo sapiens |
| 4921 | discoidin domain receptor tyrosine kinase 2 | Homo sapiens |
| 64421 | DNA cross-link repair 1C (PSO2 homolog, S. cerevisiae) | Homo sapiens |
| 7837 | peroxidasin homolog (Drosophila) | Homo sapiens |
| 7587 | zinc finger protein 37A | Homo sapiens |
| 80110 | zinc finger protein 614 | Homo sapiens |
| 11100 | heterogeneous nuclear ribonucleoprotein U-like 1 | Homo sapiens |
| 118987 | PDZ domain containing 8 | Homo sapiens |
| 9324 | high mobility group nucleosomal binding domain 3 | Homo sapiens |

| | | |
|---|---|---|
| 25864 | abhydrolase domain containing 14A | Homo sapiens |
| 84766 | EF-hand calcium binding domain 4B | Homo sapiens |
| 54726 | OTU domain containing 4 | Homo sapiens |
| 26156 | ribosomal L1 domain containing 1 | Homo sapiens |
| 51520 | leucyl-tRNA synthetase | Homo sapiens |
| 85476 | G elongation factor, mitochondrial 1 | Homo sapiens |
| 83693 | hydroxysteroid dehydrogenase like 1 | Homo sapiens |
| 201283 | hypothetical protein FLJ32065 | Homo sapiens |
| 57560 | intraflagellar transport 80 homolog (Chlamydomonas) | Homo sapiens |
| 6741 | Sjogren syndrome antigen B (autoantigen La) | Homo sapiens |
| 135295 | serine-arginine repressor protein (35 kDa) | Homo sapiens |
| 64324 | nuclear receptor binding SET domain protein 1 | Homo sapiens |
| 641638 | small nucleolar RNA host gene 6 (non-protein coding) | Homo sapiens |
| 100288618 | hypothetical protein LOC100288618 | Homo sapiens |
| 80228 | ORAI calcium release-activated calcium modulator 2 | Homo sapiens |
| 51134 | coiled-coil domain containing 41 | Homo sapiens |
| 349075 | zinc finger protein 713 | Homo sapiens |
| 116988 | ArfGAP with GTPase domain, ankyrin repeat and PH domain 3 | Homo sapiens |
| 100129405 | misato homolog 2 pseudogene | Homo sapiens |
| 6924 | transcription elongation factor B (SIII), polypeptide 3 (110kDa, elongin A) | Homo sapiens |
| 54059 | chromosome 21 open reading frame 57 | Homo sapiens |
| 351 | amyloid beta (A4) precursor protein | Homo sapiens |
| 10548 | transmembrane 9 superfamily member 1 | Homo sapiens |
| 375484 | chromosome 5 open reading frame 25 | Homo sapiens |
| 3925 | stathmin 1 | Homo sapiens |
| 10150 | muscleblind-like 2 (Drosophila) | Homo sapiens |
| 81831 | neuropilin (NRP) and tolloid (TLL)-like 2 | Homo sapiens |
| 3217 | homeobox B7 | Homo sapiens |
| 10926 | DBF4 homolog (S. cerevisiae) | Homo sapiens |
| 123263 | mitochondrial methionyl-tRNA formyltransferase | Homo sapiens |
| 283232 | transmembrane protein 80 | Homo sapiens |
| 222389 | BEN domain containing 7 | Homo sapiens |
| 8554 | protein inhibitor of activated STAT, 1 | Homo sapiens |
| 10054 | ubiquitin-like modifier activating enzyme 2 | Homo sapiens |
| 80273 | GrpE-like 1, mitochondrial (E. coli) | Homo sapiens |
| 55100 | WD repeat domain 70 | Homo sapiens |
| 6917 | transcription elongation factor A (SII), 1 pseudogene 2; transcription elongation factor A (SII), 1 | Homo sapiens |
| 9802 | DAZ associated protein 2 | Homo sapiens |
| 79760 | gem (nuclear organelle) associated protein 7 | Homo sapiens |
| 1738 | dihydrolipoamide dehydrogenase | Homo sapiens |
| 4800 | nuclear transcription factor Y, alpha | Homo sapiens |
| 285590 | SH3 and PX domains 2B | Homo sapiens |
| 5902 | similar to RAN binding protein 1; RAN binding protein 1 | Homo sapiens |
| 10526 | importin 8 | Homo sapiens |
| 84312 | breast cancer metastasis-suppressor 1-like | Homo sapiens |
| 6683 | spastin | Homo sapiens |
| 55316 | radical S-adenosyl methionine domain containing 1 | Homo sapiens |
| 55892 | myoneurin | Homo sapiens |
| 1267 | 2',3'-cyclic nucleotide 3' phosphodiesterase | Homo sapiens |
| 56288 | par-3 partitioning defective 3 homolog (C. elegans) | Homo sapiens |
| 85025 | transmembrane protein 60 | Homo sapiens |
| 54955 | chromosome 1 open reading frame 109 | Homo sapiens |
| 1155 | tubulin folding cofactor B | Homo sapiens |
| 286148 | dpy-19-like 4 (C. elegans) | Homo sapiens |
| 4953 | ornithine decarboxylase 1 | Homo sapiens |
| 10497 | unc-13 homolog B (C. elegans) | Homo sapiens |
| 5708 | proteasome (prosome, macropain) 26S subunit, non-ATPase, 2 | Homo sapiens |
| 5411 | pinin, desmosome associated protein | Homo sapiens |
| 79595 | Sin3A-associated protein, 130kDa | Homo sapiens |
| 55031 | ubiquitin specific peptidase 47 | Homo sapiens |
| 10330 | canopy 2 homolog (zebrafish) | Homo sapiens |
| 64783 | RNA binding motif protein 15 | Homo sapiens |
| 2517 | fucosidase, alpha-L- 1, tissue | Homo sapiens |
| 57001 | ACN9 homolog (S. cerevisiae) | Homo sapiens |
| 7290 | HIR histone cell cycle regulation defective homolog A (S. cerevisiae) | Homo sapiens |
| 8519 | interferon induced transmembrane protein 1 (9-27) | Homo sapiens |
| 729967 | MORN repeat containing 2 | Homo sapiens |
| 55250 | elongation protein 2 homolog (S. cerevisiae) | Homo sapiens |
| 51809 | UDP-N-acetyl-alpha-D-galactosamine:polypeptide N-acetylgalactosaminyltransferase 7 (GalNAc-T7) | Homo sapiens |
| 90459 | exoribonuclease 1 | Homo sapiens |
| 1040 | CDP-diacylglycerol synthase (phosphatidate cytidylyltransferase) 1 | Homo sapiens |
| 90416 | chromosome 15 open reading frame 57 | Homo sapiens |
| 4929 | nuclear receptor subfamily 4, group A, member 2 | Homo sapiens |
| 83606 | chromosome 22 open reading frame 13 | Homo sapiens |
| 9443 | mediator complex subunit 7 | Homo sapiens |

| | | |
|---|---|---|
| 7917 | HLA-B associated transcript 3 | Homo sapiens |
| 5576 | protein kinase, cAMP-dependent, regulatory, type II, alpha | Homo sapiens |
| 81566 | cysteine-serine-rich nuclear protein 2 | Homo sapiens |
| 6670 | Sp3 transcription factor | Homo sapiens |
| 3069 | high density lipoprotein binding protein | Homo sapiens |
| 57600 | folliculin interacting protein 2 | Homo sapiens |
| 1051 | CCAAT/enhancer binding protein (C/EBP), beta | Homo sapiens |
| 51170 | hydroxysteroid (17-beta) dehydrogenase 11 | Homo sapiens |
| 83941 | TM2 domain containing 1 | Homo sapiens |
| 79091 | chromosome 16 open reading frame 68 | Homo sapiens |
| 123920 | CKLF-like MARVEL transmembrane domain containing 3 | Homo sapiens |
| 5074 | PRKC, apoptosis, WT1, regulator | Homo sapiens |
| 579 | NK3 homeobox 2 | Homo sapiens |
| 1602 | dachshund homolog 1 (Drosophila) | Homo sapiens |
| 9491 | proteasome (prosome, macropain) inhibitor subunit 1 (PI31) | Homo sapiens |
| 54996 | MOCO sulphurase C-terminal domain containing 2 | Homo sapiens |
| 4712 | NADH dehydrogenase (ubiquinone) 1 beta subcomplex, 6, 17kDa | Homo sapiens |
| 23259 | DDHD domain containing 2 | Homo sapiens |
| 51706 | cytochrome b5 reductase 1 | Homo sapiens |
| 400949 | FKSG49 | Homo sapiens |
| 10059 | dynamin 1-like | Homo sapiens |
| 9213 | xenotropic and polytropic retrovirus receptor | Homo sapiens |
| 169714 | quiescin Q6 sulfhydryl oxidase 2 | Homo sapiens |
| 1938 | eukaryotic translation elongation factor 2 | Homo sapiens |
| 2109 | electron-transfer-flavoprotein, beta polypeptide | Homo sapiens |
| 54797 | mediator complex subunit 18 | Homo sapiens |
| 27095 | trafficking protein particle complex 3 | Homo sapiens |
| 25938 | HEAT repeat containing 5A | Homo sapiens |
| 139596 | uracil phosphoribosyltransferase (FUR1) homolog (S. cerevisiae) | Homo sapiens |
| 57466 | splicing factor, arginine/serine-rich 15 | Homo sapiens |
| 56919 | DEAH (Asp-Glu-Ala-His) box polypeptide 33 | Homo sapiens |
| 23102 | TBC1 domain family, member 2B | Homo sapiens |
| 51592 | tripartite motif-containing 33 | Homo sapiens |
| 10165 | solute carrier family 25, member 13 (citrin) | Homo sapiens |
| 6541 | solute carrier family 7 (cationic amino acid transporter, y+ system), member 1 | Homo sapiens |
| 3840 | karyopherin alpha 4 (importin alpha 3) | Homo sapiens |
| 83608 | chromosome 18 open reading frame 21 | Homo sapiens |
| 51121 | ribosomal protein L26-like 1 | Homo sapiens |
| 151230 | kelch-like 23 (Drosophila) | Homo sapiens |
| 10229 | coenzyme Q7 homolog, ubiquinone (yeast) | Homo sapiens |
| 10565 | ADP-ribosylation factor guanine nucleotide-exchange factor 1(brefeldin A-inhibited) | Homo sapiens |
| 3074 | hexosaminidase B (beta polypeptide) | Homo sapiens |
| 441366 | hypothetical gene supported by BX538329 | Homo sapiens |
| 4012 | leucyl/cystinyl aminopeptidase | Homo sapiens |
| 3609 | interleukin enhancer binding factor 3, 90kDa | Homo sapiens |
| 6636 | small nuclear ribonucleoprotein polypeptide F | Homo sapiens |
| 3622 | inhibitor of growth family, member 2 | Homo sapiens |
| 81853 | transmembrane protein 14D; transmembrane protein 14B | Homo sapiens |
| 79134 | transmembrane protein 185B (pseudogene) | Homo sapiens |
| 57606 | SLAIN motif family, member 2 | Homo sapiens |
| 5208 | 6-phosphofructo-2-kinase/fructose-2,6-biphosphatase 2 | Homo sapiens |
| 55740 | enabled homolog (Drosophila) | Homo sapiens |
| 143888 | KDEL (Lys-Asp-Glu-Leu) containing 2 | Homo sapiens |
| 29082 | chromatin modifying protein 4A | Homo sapiens |
| 644246 | hypothetical protein LOC644246 | Homo sapiens |
| 286097 | EF-hand domain family, member A2 | Homo sapiens |
| 26207 | phosphatidylinositol transfer protein, cytoplasmic 1 | Homo sapiens |
| 5202 | prefoldin subunit 2 | Homo sapiens |
| 149832 | hypothetical protein LOC149832 | Homo sapiens |
| 9208 | leucine rich repeat (in FLII) interacting protein 1 | Homo sapiens |
| 8690 | jerky homolog-like (mouse) | Homo sapiens |
| 55669 | mitofusin 1 | Homo sapiens |
| 81611 | acidic (leucine-rich) nuclear phosphoprotein 32 family, member E | Homo sapiens |
| 92482 | non-protein coding RNA 81 | Homo sapiens |
| 57118 | calcium/calmodulin-dependent protein kinase ID | Homo sapiens |
| 644511 | ribosomal protein L13a pseudogene 6 | Homo sapiens |
| 9824 | Rho GTPase activating protein 11B; Rho GTPase activating protein 11A | Homo sapiens |
| 7072 | TIA1 cytotoxic granule-associated RNA binding protein | Homo sapiens |
| 85440 | dedicator of cytokinesis 7 | Homo sapiens |
| 84084 | RAB6C, member RAS oncogene family; RAB6A, member RAS oncogene family; hypothetical LOC100130819; RAB6C-like | Homo sapiens |
| 5870 | RAB6C, member RAS oncogene family; RAB6A, member RAS oncogene family; hypothetical LOC100130819; RAB6C-like | Homo sapiens |
| 5877 | RAB interacting factor | Homo sapiens |
| 135293 | peptidase M20 domain containing 2 | Homo sapiens |
| 118429 | anthrax toxin receptor 2 | Homo sapiens |
| 80255 | solute carrier family 35, member F5 | Homo sapiens |

| | | |
|---|---|---|
| 6144 | ribosomal protein L21 pseudogene 134; ribosomal protein L21 pseudogene 80; ribosomal protein L21 pseudogene 20; ribosomal protein L21 pseudogene 46; ribosomal protein L21 pseudogene 45; ribosomal protein L21 pseudogene 131; ribosomal protein L21 pseudogene 16; ribosomal protein L21 pseudogene 53; ribosomal protein L21 pseudogene 120; ribosomal protein L21 pseudogene 37; ribosomal protein L21 pseudogene 93; ribosomal protein L21 pseudogene 39; ribosomal protein L21 pseudogene 29; ribosomal protein L21 pseudogene 28; ribosomal protein L21 pseudogene 14; ribosomal protein L21 pseudogene 98; ribosomal protein L21 pseudogene 105; ribosomal protein L21 pseudogene 87; ribosomal protein L21 pseudogene 128; ribosomal protein L21 pseudogene 69; ribosomal protein L21 pseudogene 97; ribosomal protein L21; ribosomal protein L21 pseudogene 119; ribosomal protein L21 pseudogene 125 | Homo sapiens |
| 84725 | pleckstrin homology domain containing, family A (phosphoinositide binding specific) member 8 | Homo sapiens |
| 113251 | La ribonucleoprotein domain family, member 4 | Homo sapiens |
| 266655 | non-protein coding RNA 94 | Homo sapiens |
| 79872 | Cas-Br-M (murine) ecotropic retroviral transforming sequence-like 1 | Homo sapiens |
| 3998 | lectin, mannose-binding, 1 | Homo sapiens |
| 23170 | tubulin tyrosine ligase-like family, member 12 | Homo sapiens |
| 10550 | ADP-ribosylation-like factor 6 interacting protein 5 | Homo sapiens |
| 138311 | family with sequence similarity 69, member B | Homo sapiens |
| 7678 | zinc finger protein 124 | Homo sapiens |
| 1958 | early growth response 1 | Homo sapiens |
| 54902 | tetratricopeptide repeat domain 19 | Homo sapiens |
| 51643 | transmembrane BAX inhibitor motif containing 4 | Homo sapiens |
| 163259 | DENN/MADD domain containing 2C | Homo sapiens |
| 6166 | ribosomal protein L36a-like | Homo sapiens |
| 51734 | selenoprotein X, 1 | Homo sapiens |
| 90268 | family with sequence similarity 105, member B | Homo sapiens |
| 57474 | zinc finger protein 490 | Homo sapiens |
| 51201 | zinc finger, DHHC-type containing 2 | Homo sapiens |
| 221785 | zinc finger protein 498 | Homo sapiens |
| 9126 | structural maintenance of chromosomes 3 | Homo sapiens |
| 9810 | ring finger protein 40 | Homo sapiens |
| 84986 | Rho GTPase activating protein 19 | Homo sapiens |
| 9675 | KIAA0406 | Homo sapiens |
| 7569 | zinc finger protein 182 | Homo sapiens |
| 133522 | peroxisome proliferator-activated receptor gamma, coactivator 1 beta | Homo sapiens |
| 2926 | G-rich RNA sequence binding factor 1 | Homo sapiens |
| 9926 | lysophosphatidylglycerol acyltransferase 1 | Homo sapiens |
| 51535 | periphilin 1 | Homo sapiens |
| 9730 | Vpr (HIV-1) binding protein | Homo sapiens |
| 100101467 | zinc finger protein 397 opposite strand | Homo sapiens |
| 7553 | zinc finger protein 7 | Homo sapiens |
| 51668 | heat shock protein family B (small), member 11 | Homo sapiens |
| 6418 | SET nuclear oncogene; similar to SET translocation | Homo sapiens |
| 9863 | membrane associated guanylate kinase, WW and PDZ domain containing 2 | Homo sapiens |
| 51633 | OTU domain containing 6B | Homo sapiens |
| 2117 | ets variant 3 | Homo sapiens |
| 80755 | alanyl-tRNA synthetase domain containing 1 | Homo sapiens |
| 51072 | mediator of cell motility 1; similar to mediator of cell motility 1 | Homo sapiens |
| 54148 | mitochondrial ribosomal protein L39 | Homo sapiens |
| 83549 | uridine-cytidine kinase 1 | Homo sapiens |
| 1200 | tripeptidyl peptidase I | Homo sapiens |
| 196047 | EMX2 opposite strand (non-protein coding) | Homo sapiens |
| 9470 | eukaryotic translation initiation factor 4E family member 2 | Homo sapiens |
| 10252 | sprouty homolog 1, antagonist of FGF signaling (Drosophila) | Homo sapiens |
| 83451 | abhydrolase domain containing 11 | Homo sapiens |
| 54585 | leucine zipper transcription factor-like 1 | Homo sapiens |
| 285958 | chromosome 7 open reading frame 40 | Homo sapiens |
| 11266 | dual specificity phosphatase 12 | Homo sapiens |
| 79706 | PRKR interacting protein 1 (IL11 inducible) | Homo sapiens |
| 5046 | proprotein convertase subtilisin/kexin type 6 | Homo sapiens |
| 10360 | nucleophosmin/nucleoplasmin, 3 | Homo sapiens |
| 7422 | vascular endothelial growth factor A | Homo sapiens |
| 1063 | centromere protein F, 350/400ka (mitosin) | Homo sapiens |
| 10329 | transmembrane protein 5 | Homo sapiens |
| 5832 | aldehyde dehydrogenase 18 family, member A1 | Homo sapiens |
| 10733 | polo-like kinase 4 (Drosophila) | Homo sapiens |
| 84132 | ubiquitin specific peptidase 42 | Homo sapiens |
| 285672 | SFRS12-interacting protein 1; family with sequence similarity 159, member B | Homo sapiens |
| 160897 | G protein-coupled receptor 180 | Homo sapiens |
| 27130 | inversin | Homo sapiens |
| 9454 | homer homolog 3 (Drosophila) | Homo sapiens |
| 55154 | misato homolog 1 (Drosophila) | Homo sapiens |
| 134147 | carboxymethylenebutenolidase homolog (Pseudomonas) | Homo sapiens |
| 23186 | REST corepressor 1 | Homo sapiens |
| 7804 | low density lipoprotein receptor-related protein 8, apolipoprotein e receptor | Homo sapiens |

| | | |
|---|---|---|
| 10695 | canopy 3 homolog (zebrafish) | Homo sapiens |
| 875 | cystathionine-beta-synthase | Homo sapiens |
| 51727 | cytidine monophosphate (UMP-CMP) kinase 1, cytosolic | Homo sapiens |
| 6745 | signal sequence receptor, alpha | Homo sapiens |
| 1743 | dihydrolipoamide S-succinyltransferase (E2 component of 2-oxo-glutarate complex); dihydrolipoamide S-succinyltransferase pseudogene (E2 component of 2-oxo-glutarate complex) | Homo sapiens |
| 146223 | CKLF-like MARVEL transmembrane domain containing 4 | Homo sapiens |
| 26271 | F-box protein 5 | Homo sapiens |
| 7157 | tumor protein p53 | Homo sapiens |
| 728643 | heterogeneous nuclear ribonucleoprotein A1-like 3; similar to heterogeneous nuclear ribonucleoprotein A1; heterogeneous nuclear ribonucleoprotein A1 pseudogene 2; heterogeneous nuclear ribonucleoprotein A1; heterogeneous nuclear ribonucleoprotein A1 pseudogene | Homo sapiens |
| 664709 | heterogeneous nuclear ribonucleoprotein A1-like 3; similar to heterogeneous nuclear ribonucleoprotein A1; heterogeneous nuclear ribonucleoprotein A1 pseudogene 2; heterogeneous nuclear ribonucleoprotein A1; heterogeneous nuclear ribonucleoprotein A1 pseudogene | Homo sapiens |
| 3178 | heterogeneous nuclear ribonucleoprotein A1-like 3; similar to heterogeneous nuclear ribonucleoprotein A1; heterogeneous nuclear ribonucleoprotein A1 pseudogene 2; heterogeneous nuclear ribonucleoprotein A1; heterogeneous nuclear ribonucleoprotein A1 pseudogene | Homo sapiens |
| 133619 | proline-rich coiled-coil 1 | Homo sapiens |
| 146542 | zinc finger protein 688; zinc finger protein 785 | Homo sapiens |
| 3420 | isocitrate dehydrogenase 3 (NAD+) beta | Homo sapiens |
| 1841 | deoxythymidylate kinase (thymidylate kinase); similar to Deoxythymidylate kinase (thymidylate kinase) | Homo sapiens |
| 400410 | suppressor of tumorigenicity 20 | Homo sapiens |
| 11030 | RNA binding protein with multiple splicing | Homo sapiens |
| 347734 | solute carrier family 35, member B2 | Homo sapiens |
| 847 | catalase | Homo sapiens |
| 96764 | trimethylguanosine synthase homolog (S. cerevisiae) | Homo sapiens |
| 4722 | NADH dehydrogenase (ubiquinone) Fe-S protein 3, 30kDa (NADH-coenzyme Q reductase) | Homo sapiens |
| 10768 | adenosylhomocysteinase-like 1 | Homo sapiens |
| 23657 | solute carrier family 7, (cationic amino acid transporter, y+ system) member 11 | Homo sapiens |
| 9939 | RNA binding motif protein 8A | Homo sapiens |
| 10126 | dynein, axonemal, light chain 4 | Homo sapiens |
| 6233 | ribosomal protein S27a pseudogene 12; ribosomal protein S27a; ribosomal protein S27a pseudogene 11; ribosomal protein S27a pseudogene 16 | Homo sapiens |
| 25909 | AT hook containing transcription factor 1; AT hook containing transcription factor 1 pseudogene | Homo sapiens |
| 5457 | POU class 4 homeobox 1 | Homo sapiens |
| 1557 | cytochrome P450, family 2, subfamily C, polypeptide 19 | Homo sapiens |
| 50810 | hepatoma-derived growth factor, related protein 3 | Homo sapiens |
| 5638 | proline rich Gla (G-carboxyglutamic acid) 1 | Homo sapiens |
| 27249 | methylmalonic aciduria (cobalamin deficiency) cblD type, with homocystinuria | Homo sapiens |
| 257396 | hypothetical protein LOC257396 | Homo sapiens |
| 7596 | zinc finger protein 45 | Homo sapiens |
| 54014 | bromodomain and WD repeat domain containing 1 | Homo sapiens |
| 84933 | chromosome 8 open reading frame 76 | Homo sapiens |
| 29889 | guanine nucleotide binding protein-like 2 (nucleolar) | Homo sapiens |
| 4783 | nuclear factor, interleukin 3 regulated | Homo sapiens |
| 8295 | transformation/transcription domain-associated protein | Homo sapiens |
| 55071 | chromosome 9 open reading frame 40 | Homo sapiens |
| 64207 | chromosome 14 open reading frame 4 | Homo sapiens |
| 8526 | diacylglycerol kinase, epsilon 64kDa | Homo sapiens |
| 10298 | p21 protein (Cdc42/Rac)-activated kinase 4 | Homo sapiens |
| 4199 | malic enzyme 1, NADP(+)-dependent, cytosolic | Homo sapiens |
| 654342 | lymphocyte-specific protein 1 pseudogene | Homo sapiens |
| 645166 | lymphocyte-specific protein 1 pseudogene | Homo sapiens |
| 2149 | coagulation factor II (thrombin) receptor | Homo sapiens |
| 1837 | dystrobrevin, alpha | Homo sapiens |
| 11052 | cleavage and polyadenylation specific factor 6, 68kDa | Homo sapiens |
| 22856 | chondroitin sulfate synthase 1 | Homo sapiens |
| 5141 | phosphodiesterase 4A, cAMP-specific (phosphodiesterase E2 dunce homolog, Drosophila) | Homo sapiens |
| 51060 | thioredoxin domain containing 12 (endoplasmic reticulum) | Homo sapiens |
| 91404 | SEC14 and spectrin domains 1 | Homo sapiens |
| 84259 | DCN1, defective in cullin neddylation 1, domain containing 5 (S. cerevisiae) | Homo sapiens |
| 2547 | X-ray repair complementing defective repair in Chinese hamster cells 6; similar to ATP-dependent DNA helicase II, 70 kDa subunit | Homo sapiens |
| 8502 | plakophilin 4 | Homo sapiens |
| 8503 | phosphoinositide-3-kinase, regulatory subunit 3 (gamma) | Homo sapiens |
| 59338 | pleckstrin homology domain containing, family A (phosphoinositide binding specific) member 1 | Homo sapiens |
| 54811 | zinc finger protein 562 | Homo sapiens |
| 84897 | transforming growth factor beta regulator 1 | Homo sapiens |
| 245812 | canopy 4 homolog (zebrafish) | Homo sapiens |

| | | |
|---|---|---|
| 23594 | origin recognition complex, subunit 6 like (yeast) | Homo sapiens |
| 84901 | nuclear factor of activated T-cells, cytoplasmic, calcineurin-dependent 2 interacting protein | Homo sapiens |
| 4507 | methylthioadenosine phosphorylase | Homo sapiens |
| 6224 | ribosomal protein S20 | Homo sapiens |
| 8480 | RAE1 RNA export 1 homolog (S. pombe) | Homo sapiens |
| 440026 | transmembrane protein 41B | Homo sapiens |
| 128439 | small nucleolar RNA host gene 11 (non-protein coding) | Homo sapiens |
| 26100 | WD repeat domain, phosphoinositide interacting 2 | Homo sapiens |
| 2990 | glucuronidase, beta | Homo sapiens |
| 6642 | sorting nexin 1 | Homo sapiens |
| 51104 | family with sequence similarity 108, member B1 | Homo sapiens |
| 23306 | transmembrane protein 194A | Homo sapiens |
| 2161 | coagulation factor XII (Hageman factor) | Homo sapiens |
| 7111 | tropomodulin 1 | Homo sapiens |
| 55432 | YOD1 OTU deubiquinating enzyme 1 homolog (S. cerevisiae) | Homo sapiens |
| 10614 | hexamethylene bis-acetamide inducible 1 | Homo sapiens |
| 79031 | phosducin-like 3 pseudogene; phosducin-like 3 | Homo sapiens |
| 285359 | phosducin-like 3 pseudogene; phosducin-like 3 | Homo sapiens |
| 64282 | PAP associated domain containing 5 | Homo sapiens |
| 54914 | KIAA1797 | Homo sapiens |
| 57102 | chromosome 12 open reading frame 40; chromosome 12 open reading frame 4 | Homo sapiens |
| 255758 | Tctex1 domain containing 2 | Homo sapiens |
| 56521 | DnaJ (Hsp40) homolog, subfamily C, member 12 | Homo sapiens |
| 2869 | G protein-coupled receptor kinase 5 | Homo sapiens |
| 120103 | solute carrier family 36 (proton/amino acid symporter), member 4 | Homo sapiens |
| 10018 | BCL2-like 11 (apoptosis facilitator) | Homo sapiens |
| 10400 | phosphatidylethanolamine N-methyltransferase | Homo sapiens |
| 53917 | RAB24, member RAS oncogene family | Homo sapiens |
| 55850 | unconventional SNARE in the ER 1 homolog (S. cerevisiae) | Homo sapiens |
| 29925 | GDP-mannose pyrophosphorylase B | Homo sapiens |
| 6443 | sarcoglycan, beta (43kDa dystrophin-associated glycoprotein) | Homo sapiens |
| 4835 | NAD(P)H dehydrogenase, quinone 2 | Homo sapiens |
| 57179 | KIAA1191 | Homo sapiens |
| 26468 | LIM homeobox 6 | Homo sapiens |
| 84656 | cytokine-like nuclear factor n-pac | Homo sapiens |
| 112495 | general transcription factor IIIC, polypeptide 6, alpha 35kDa | Homo sapiens |
| 51204 | coiled-coil domain containing 44 | Homo sapiens |
| 79736 | chromosome 17 open reading frame 42 | Homo sapiens |
| 5928 | hypothetical LOC642954; retinoblastoma binding protein 4 | Homo sapiens |
| 64395 | germ cell-less homolog 1 (Drosophila)-like; germ cell-less homolog 1 (Drosophila) | Homo sapiens |
| 64396 | germ cell-less homolog 1 (Drosophila)-like; germ cell-less homolog 1 (Drosophila) | Homo sapiens |
| 54499 | transmembrane and coiled-coil domains 1 | Homo sapiens |
| 4701 | NADH dehydrogenase (ubiquinone) 1 alpha subcomplex, 7, 14.5kDa | Homo sapiens |
| 192670 | eukaryotic translation initiation factor 2C, 4 | Homo sapiens |
| 9191 | death effector domain containing | Homo sapiens |
| 599 | BCL2-like 2 | Homo sapiens |
| 6749 | structure specific recognition protein 1 | Homo sapiens |
| 833 | cysteinyl-tRNA synthetase | Homo sapiens |
| 57017 | coenzyme Q9 homolog (S. cerevisiae) | Homo sapiens |
| 26064 | retinoic acid induced 14 | Homo sapiens |
| 54557 | small glutamine-rich tetratricopeptide repeat (TPR)-containing, beta | Homo sapiens |
| 5867 | RAB4A, member RAS oncogene family | Homo sapiens |
| 7458 | eukaryotic translation initiation factor 4H | Homo sapiens |
| 2037 | erythrocyte membrane protein band 4.1-like 2 | Homo sapiens |
| 10052 | gap junction protein, gamma 1, 45kDa | Homo sapiens |
| 11138 | TBC1 domain family, member 8 (with GRAM domain) | Homo sapiens |
| 90390 | mediator complex subunit 30 | Homo sapiens |
| 51128 | SAR1 homolog B (S. cerevisiae) | Homo sapiens |
| 9969 | mediator complex subunit 13 | Homo sapiens |
| 353376 | transmembrane emp24 protein transport domain containing 7; toll-like receptor adaptor molecule 2 | Homo sapiens |
| 51014 | transmembrane emp24 protein transport domain containing 7; toll-like receptor adaptor molecule 2 | Homo sapiens |
| 6500 | S-phase kinase-associated protein 1 | Homo sapiens |
| 4150 | MYC-associated zinc finger protein (purine-binding transcription factor) | Homo sapiens |
| 27005 | ubiquitin specific peptidase 21 | Homo sapiens |
| 10040 | target of myb1 (chicken)-like 1 | Homo sapiens |
| 440944 | hypothetical LOC440944 | Homo sapiens |
| 5191 | peroxisomal biogenesis factor 7 | Homo sapiens |
| 54464 | 5'-3' exoribonuclease 1 | Homo sapiens |
| 4735 | septin 2 | Homo sapiens |
| 2745 | glutaredoxin (thioltransferase) | Homo sapiens |
| 9794 | mastermind-like 1 (Drosophila) | Homo sapiens |
| 2947 | glutathione S-transferase mu 3 (brain) | Homo sapiens |
| 57026 | pyridoxal (pyridoxine, vitamin B6) phosphatase | Homo sapiens |
| 54332 | ganglioside-induced differentiation-associated protein 1 | Homo sapiens |

| | | |
|---|---|---|
| 51726 | DnaJ (Hsp40) homolog, subfamily B, member 11 | Homo sapiens |
| 27236 | ADP-ribosylation factor interacting protein 1 | Homo sapiens |
| 64225 | atlastin GTPase 2 | Homo sapiens |
| 54434 | slingshot homolog 1 (Drosophila) | Homo sapiens |
| 8237 | ubiquitin specific peptidase 11 | Homo sapiens |
| 6902 | tubulin folding cofactor A | Homo sapiens |
| 540 | ATPase, Cu++ transporting, beta polypeptide | Homo sapiens |
| 8602 | NOP14 nucleolar protein homolog (yeast) | Homo sapiens |
| 57727 | nuclear receptor coactivator 5 | Homo sapiens |
| 4733 | developmentally regulated GTP binding protein 1 | Homo sapiens |
| 123606 | non imprinted in Prader-Willi/Angelman syndrome 1 | Homo sapiens |
| 23288 | IQ motif containing E | Homo sapiens |
| 51660 | brain protein 44-like | Homo sapiens |
| 4548 | 5-methyltetrahydrofolate-homocysteine methyltransferase | Homo sapiens |
| 55871 | COBW domain containing 6; COBW domain containing 1 | Homo sapiens |
| 644019 | COBW domain containing 6; COBW domain containing 1 | Homo sapiens |
| 10969 | EBNA1 binding protein 2 | Homo sapiens |
| 29123 | ankyrin repeat domain 11; hypothetical protein LOC100128265 | Homo sapiens |
| 8674 | vesicle-associated membrane protein 4 | Homo sapiens |
| 54913 | ribonuclease P/MRP 25kDa subunit | Homo sapiens |
| 11340 | exosome component 8 | Homo sapiens |
| 10248 | processing of precursor 7, ribonuclease P/MRP subunit (S. cerevisiae) | Homo sapiens |
| 9859 | centrosomal protein 170kDa | Homo sapiens |
| 129787 | transmembrane protein 18 | Homo sapiens |
| 25896 | integrator complex subunit 7 | Homo sapiens |
| 284161 | glycerophosphodiester phosphodiesterase domain containing 1 | Homo sapiens |
| 23295 | mahogunin, ring finger 1 | Homo sapiens |
| 5894 | v-raf-1 murine leukemia viral oncogene homolog 1 | Homo sapiens |
| 8851 | cyclin-dependent kinase 5, regulatory subunit 1 (p35) | Homo sapiens |
| 25814 | ataxin 10 | Homo sapiens |
| 200185 | keratinocyte associated protein 2 | Homo sapiens |
| 148304 | chromosome 1 open reading frame 74 | Homo sapiens |
| 26578 | osteoclast stimulating factor 1 | Homo sapiens |
| 51341 | zinc finger and BTB domain containing 7A | Homo sapiens |
| 57415 | chromosome 3 open reading frame 14 | Homo sapiens |
| 79157 | major facilitator superfamily domain containing 11 | Homo sapiens |
| 9815 | G protein-coupled receptor kinase interacting ArfGAP 2 | Homo sapiens |
| 84545 | mitochondrial ribosomal protein L43 | Homo sapiens |
| 27020 | neuroplastin | Homo sapiens |
| 1482 | NK2 transcription factor related, locus 5 (Drosophila) | Homo sapiens |
| 253738 | early B-cell factor 3 | Homo sapiens |
| 222255 | ataxin 7-like 1 | Homo sapiens |
| 255488 | ring finger protein 144B | Homo sapiens |
| 7516 | X-ray repair complementing defective repair in Chinese hamster cells 2 | Homo sapiens |
| 55692 | LUC7-like (S. cerevisiae) | Homo sapiens |
| 8799 | peroxisomal biogenesis factor 11 beta | Homo sapiens |
| 80224 | nucleotide binding protein-like | Homo sapiens |
| 4430 | myosin IB | Homo sapiens |
| 10623 | polymerase (RNA) III (DNA directed) polypeptide C (62kD) | Homo sapiens |
| 2958 | general transcription factor IIA, 2, 12kDa | Homo sapiens |
| 54542 | ring finger and CCCH-type zinc finger domains 2 | Homo sapiens |
| 3708 | inositol 1,4,5-triphosphate receptor, type 1 | Homo sapiens |
| 1029 | cyclin-dependent kinase inhibitor 2A (melanoma, p16, inhibits CDK4) | Homo sapiens |
| 152815 | THAP domain containing 6 | Homo sapiens |
| 7580 | zinc finger protein 32 | Homo sapiens |
| 8804 | cellular repressor of E1A-stimulated genes 1 | Homo sapiens |
| 81706 | protein phosphatase 1, regulatory (inhibitor) subunit 14C | Homo sapiens |
| 4524 | 5,10-methylenetetrahydrofolate reductase (NADPH) | Homo sapiens |
| 10112 | kinesin family member 20A | Homo sapiens |
| 1854 | deoxyuridine triphosphatase | Homo sapiens |
| 23234 | DnaJ (Hsp40) homolog, subfamily C, member 9 | Homo sapiens |
| 253558 | lysocardiolipin acyltransferase 1 | Homo sapiens |
| 2717 | galactosidase, alpha | Homo sapiens |
| 23309 | SIN3 homolog B, transcription regulator (yeast) | Homo sapiens |
| 5719 | proteasome (prosome, macropain) 26S subunit, non-ATPase, 13 | Homo sapiens |
| 9994 | caspase 8 associated protein 2 | Homo sapiens |
| 9759 | histone deacetylase 4 | Homo sapiens |
| 4601 | MAX interactor 1 | Homo sapiens |
| 51747 | cisplatin resistance-associated overexpressed protein | Homo sapiens |
| 1387 | CREB binding protein | Homo sapiens |
| 5872 | RAB13, member RAS oncogene family; similar to hCG24991 | Homo sapiens |
| 84302 | chromosome 9 open reading frame 125 | Homo sapiens |
| 55161 | transmembrane protein 33 | Homo sapiens |
| 83667 | sestrin 2 | Homo sapiens |
| 440348 | nuclear pore complex interacting protein-like 2 | Homo sapiens |
| 23067 | SET domain containing 1B | Homo sapiens |
| 55280 | CWF19-like 1, cell cycle control (S. pombe) | Homo sapiens |

| | | |
|---|---|---|
| 122809 | suppressor of cytokine signaling 4 | Homo sapiens |
| 159371 | transmembrane protein 20 | Homo sapiens |
| 7337 | ubiquitin protein ligase E3A | Homo sapiens |
| 27077 | B9 protein domain 1 | Homo sapiens |
| 9774 | similar to Bcl-2-associated transcription factor 1 (Btf); BCL2-associated transcription factor 1 | Homo sapiens |
| 139322 | apolipoprotein O-like | Homo sapiens |
| 9741 | lysosomal protein transmembrane 4 alpha | Homo sapiens |
| 9858 | KIAA0649 | Homo sapiens |
| 84858 | zinc finger protein 503 | Homo sapiens |
| 376693 | ribosomal protein S10; ribosomal protein S10 pseudogene 4; ribosomal protein S10 pseudogene 11; ribosomal protein S10 pseudogene 22; ribosomal protein S10 pseudogene 7; ribosomal protein S10 pseudogene 13 | Homo sapiens |
| 6204 | ribosomal protein S10; ribosomal protein S10 pseudogene 4; ribosomal protein S10 pseudogene 11; ribosomal protein S10 pseudogene 22; ribosomal protein S10 pseudogene 7; ribosomal protein S10 pseudogene 13 | Homo sapiens |
| 2057 | erythropoietin receptor | Homo sapiens |
| 11165 | nudix (nucleoside diphosphate linked moiety X)-type motif 3 | Homo sapiens |
| 10914 | poly(A) polymerase alpha | Homo sapiens |
| 642869 | SET translocation (myeloid leukemia-associated) pseudogene | Homo sapiens |
| 387263 | chromosome 6 open reading frame 120 | Homo sapiens |
| 84456 | l(3)mbt-like 3 (Drosophila) | Homo sapiens |
| 253959 | GTPase activating Rap/RanGAP domain-like 1 | Homo sapiens |
| 9403 | 15 kDa selenoprotein | Homo sapiens |
| 80349 | WD repeat domain 61 | Homo sapiens |
| 57508 | integrator complex subunit 2 | Homo sapiens |
| 781 | calcium channel, voltage-dependent, alpha 2/delta subunit 1 | Homo sapiens |
| 26003 | golgi reassembly stacking protein 2, 55kDa | Homo sapiens |
| 26049 | family with sequence similarity 169, member A | Homo sapiens |
| 79719 | alpha- and gamma-adaptin-binding protein p34 | Homo sapiens |
| 5428 | polymerase (DNA directed), gamma | Homo sapiens |
| 148362 | chromosome 1 open reading frame 58 | Homo sapiens |
| 79086 | chromosome 19 open reading frame 42 | Homo sapiens |
| 55632 | G2/M-phase specific E3 ubiquitin ligase | Homo sapiens |
| 55245 | ubiquinol-cytochrome c reductase complex chaperone | Homo sapiens |
| 5911 | RAP2A, member of RAS oncogene family | Homo sapiens |
| 7064 | thimet oligopeptidase 1 | Homo sapiens |
| 221154 | EF-hand domain family, member A1 | Homo sapiens |
| 3329 | heat shock 60kDa protein 1 (chaperonin) pseudogene 5; heat shock 60kDa protein 1 (chaperonin) pseudogene 6; heat shock 60kDa protein 1 (chaperonin) pseudogene 1; heat shock 60kDa protein 1 (chaperonin) pseudogene 4; heat shock 60kDa protein 1 (chaperonin) | Homo sapiens |
| 11057 | abhydrolase domain containing 2 | Homo sapiens |
| 55143 | cell division cycle associated 8 | Homo sapiens |
| 22980 | transcription factor 25 (basic helix-loop-helix) | Homo sapiens |
| 643836 | zinc finger protein 62 homolog (mouse) | Homo sapiens |
| 5610 | eukaryotic translation initiation factor 2-alpha kinase 2 | Homo sapiens |
| 6150 | mitochondrial ribosomal protein L23 | Homo sapiens |
| 7991 | tumor suppressor candidate 3 | Homo sapiens |
| 148789 | beta-1,3-N-acetylgalactosaminyltransferase 2 | Homo sapiens |
| 27106 | arrestin domain containing 2 | Homo sapiens |
| 9737 | G protein-coupled receptor associated sorting protein 1 | Homo sapiens |
| 11260 | exportin, tRNA (nuclear export receptor for tRNAs); similar to Exportin-T (tRNA exportin) (Exportin(tRNA)) | Homo sapiens |
| 29928 | translocase of inner mitochondrial membrane 22 homolog (yeast) | Homo sapiens |
| 3133 | major histocompatibility complex, class I, E | Homo sapiens |
| 79896 | threonine synthase-like 1 (S. cerevisiae) | Homo sapiens |
| 143187 | vesicle transport through interaction with t-SNAREs homolog 1A (yeast) | Homo sapiens |
| 10096 | ARP3 actin-related protein 3 homolog (yeast) | Homo sapiens |
| 824 | calpain 2, (m/II) large subunit | Homo sapiens |
| 54704 | pyruvate dehyrogenase phosphatase catalytic subunit 1 | Homo sapiens |
| 80179 | myosin XIX | Homo sapiens |
| 23483 | TDP-glucose 4,6-dehydratase | Homo sapiens |
| 56967 | chromosome 14 open reading frame 132 | Homo sapiens |
| 6429 | splicing factor, arginine/serine-rich 4 | Homo sapiens |
| 2683 | UDP-Gal:betaGlcNAc beta 1,4- galactosyltransferase, polypeptide 1 | Homo sapiens |
| 5609 | mitogen-activated protein kinase kinase 7 | Homo sapiens |
| 9592 | immediate early response 2 | Homo sapiens |
| 9821 | RB1-inducible coiled-coil 1 | Homo sapiens |
| 6119 | replication protein A3, 14kDa | Homo sapiens |
| 55323 | La ribonucleoprotein domain family, member 6 | Homo sapiens |
| 84520 | chromosome 14 open reading frame 142 | Homo sapiens |
| 8895 | copine III | Homo sapiens |
| 158506 | zinc finger protein 645 | Homo sapiens |
| 7009 | transmembrane BAX inhibitor motif containing 6 | Homo sapiens |
| 10479 | solute carrier family 9 (sodium/hydrogen exchanger), member 6 | Homo sapiens |
| 118924 | chromosome 10 open reading frame 4 | Homo sapiens |

| | | |
|---|---|---|
| 10541 | similar to Acidic leucine-rich nuclear phosphoprotein 32 family member B (PHAPI2 protein) (Silver-stainable protein SSP29) (Acidic protein rich in leucines); acidic (leucine-rich) nuclear phosphoprotein 32 family, member B | Homo sapiens |
| 54799 | mbt domain containing 1 | Homo sapiens |
| 84311 | mitochondrial ribosomal protein L45 | Homo sapiens |
| 7027 | transcription factor Dp-1 | Homo sapiens |
| 152100 | COX assembly mitochondrial protein homolog (S. cerevisiae) | Homo sapiens |
| 8720 | membrane-bound transcription factor peptidase, site 1 | Homo sapiens |
| 4351 | mannose phosphate isomerase | Homo sapiens |
| 51118 | UTP11-like, U3 small nucleolar ribonucleoprotein, (yeast) | Homo sapiens |
| 1212 | clathrin, light chain (Lcb) | Homo sapiens |
| 3418 | isocitrate dehydrogenase 2 (NADP+), mitochondrial | Homo sapiens |
| 56922 | methylcrotonoyl-Coenzyme A carboxylase 1 (alpha) | Homo sapiens |
| 57515 | serine incorporator 1 | Homo sapiens |
| 54553 | hypothetical protein DKFZP434I0714 | Homo sapiens |
| 27429 | HtrA serine peptidase 2 | Homo sapiens |
| 387921 | NHL repeat containing 3 | Homo sapiens |
| 23390 | zinc finger, DHHC-type containing 17 | Homo sapiens |
| 26097 | chromosome 1 open reading frame 77 | Homo sapiens |
| 64422 | ATG3 autophagy related 3 homolog (S. cerevisiae) | Homo sapiens |
| 6744 | sperm specific antigen 2 | Homo sapiens |
| 7444 | vaccinia related kinase 2 | Homo sapiens |
| 25798 | brain protein I3; brain protein I3 pseudogene 1 | Homo sapiens |
| 113174 | serum amyloid A-like 1 | Homo sapiens |
| 115209 | OMA1 homolog, zinc metallopeptidase (S. cerevisiae) | Homo sapiens |
| 3201 | homeobox A4 | Homo sapiens |
| 84068 | solute carrier family 10 (sodium/bile acid cotransporter family), member 7 | Homo sapiens |
| 10671 | dynactin 6 | Homo sapiens |
| 116154 | phosphatase and actin regulator 3 | Homo sapiens |
| 9738 | CP110 protein | Homo sapiens |
| 23185 | La ribonucleoprotein domain family, member 4B | Homo sapiens |
| 6790 | aurora kinase A; aurora kinase A pseudogene 1 | Homo sapiens |
| 554282 | family with sequence similarity 72, member C | Homo sapiens |
| 34 | acyl-Coenzyme A dehydrogenase, C-4 to C-12 straight chain | Homo sapiens |
| 84838 | zinc finger protein 496 | Homo sapiens |
| 54906 | chromosome 10 open reading frame 18 | Homo sapiens |
| 10175 | cornichon homolog (Drosophila) | Homo sapiens |
| 6653 | sortilin-related receptor, L(DLR class) A repeats-containing | Homo sapiens |
| 9088 | protein kinase, membrane associated tyrosine/threonine 1 | Homo sapiens |
| 79982 | DnaJ (Hsp40) homolog, subfamily B, member 14 | Homo sapiens |
| 10283 | serologically defined colon cancer antigen 10 | Homo sapiens |
| 5570 | protein kinase (cAMP-dependent, catalytic) inhibitor beta | Homo sapiens |
| 5412 | ubiquitin-like 3 | Homo sapiens |
| 84859 | leucine-rich repeats and calponin homology (CH) domain containing 3 | Homo sapiens |
| 57555 | neuroligin 2 | Homo sapiens |
| 90161 | heparan sulfate 6-O-sulfotransferase 2 | Homo sapiens |
| 51621 | Kruppel-like factor 13 | Homo sapiens |
| 27069 | growth hormone inducible transmembrane protein | Homo sapiens |
| 22897 | centrosomal protein 164kDa | Homo sapiens |
| 27112 | family with sequence similarity 155, member B | Homo sapiens |
| 51155 | hematological and neurological expressed 1 | Homo sapiens |
| 116442 | RAB39B, member RAS oncogene family | Homo sapiens |
| 7169 | tropomyosin 2 (beta) | Homo sapiens |
| 3094 | histidine triad nucleotide binding protein 1 | Homo sapiens |
| 113278 | chromosome 20 open reading frame 54 | Homo sapiens |
| 54916 | chromosome 14 open reading frame 101 | Homo sapiens |
| 26046 | ring finger protein 160 | Homo sapiens |
| 2764 | glia maturation factor, beta | Homo sapiens |
| 221150 | chromosome 13 open reading frame 3 | Homo sapiens |
| 4831 | non-metastatic cells 1, protein (NM23A) expressed in; NME1-NME2 readthrough transcript; non-metastatic cells 2, protein (NM23B) expressed in | Homo sapiens |
| 4830 | non-metastatic cells 1, protein (NM23A) expressed in; NME1-NME2 readthrough transcript; non-metastatic cells 2, protein (NM23B) expressed in | Homo sapiens |
| 654364 | non-metastatic cells 1, protein (NM23A) expressed in; NME1-NME2 readthrough transcript; non-metastatic cells 2, protein (NM23B) expressed in | Homo sapiens |
| 55803 | ArfGAP with dual PH domains 2 | Homo sapiens |
| 727936 | glycosyltransferase 8 domain containing 4 | Homo sapiens |
| 11161 | chromosome 14 open reading frame 1 | Homo sapiens |
| 10121 | ARP1 actin-related protein 1 homolog A, centractin alpha (yeast) | Homo sapiens |
| 9860 | leucine-rich repeats and immunoglobulin-like domains 2 | Homo sapiens |
| 26297 | secretion regulating guanine nucleotide exchange factor | Homo sapiens |
| 57541 | zinc finger protein 398 | Homo sapiens |
| 8613 | phosphatidic acid phosphatase type 2B | Homo sapiens |
| 116984 | ArfGAP with RhoGAP domain, ankyrin repeat and PH domain 2 | Homo sapiens |
| 10006 | abl-interactor 1 | Homo sapiens |
| 10652 | YKT6 v-SNARE homolog (S. cerevisiae) | Homo sapiens |
| 401466 | chromosome 8 open reading frame 59 | Homo sapiens |

| | | |
|---|---|---|
| 23117 | nuclear pore complex interacting protein-like 3; similar to Uncharacterized protein KIAA0220 | Homo sapiens |
| 100132247 | nuclear pore complex interacting protein-like 3; similar to Uncharacterized protein KIAA0220 | Homo sapiens |
| 6772 | signal transducer and activator of transcription 1, 91kDa | Homo sapiens |
| 26267 | F-box protein 10 | Homo sapiens |
| 8454 | cullin 1 | Homo sapiens |
| 201895 | chromosome 4 open reading frame 34 | Homo sapiens |
| 100127983 | hypothetical protein LOC100127983 | Homo sapiens |
| 51503 | CWC15 spliceosome-associated protein homolog (S. cerevisiae) | Homo sapiens |
| 95681 | testis specific, 14 | Homo sapiens |
| 8624 | proteasome (prosome, macropain) assembly chaperone 1 | Homo sapiens |
| 342371 | ataxin 1-like | Homo sapiens |
| 64762 | hypothetical protein LOC100130616; family with sequence similarity 59, member A | Homo sapiens |
| 29101 | SSU72 RNA polymerase II CTD phosphatase homolog (S. cerevisiae) | Homo sapiens |
| 29058 | hypothetical LOC642975; chromosome 20 open reading frame 30 | Homo sapiens |
| 1545 | cytochrome P450, family 1, subfamily B, polypeptide 1 | Homo sapiens |
| 10057 | ATP-binding cassette, sub-family C (CFTR/MRP), member 5 | Homo sapiens |
| 64844 | membrane-associated ring finger (C3HC4) 7 | Homo sapiens |
| 10598 | AHA1, activator of heat shock 90kDa protein ATPase homolog 1 (yeast) | Homo sapiens |
| 8411 | early endosome antigen 1 | Homo sapiens |
| 23406 | coactosin-like 1 (Dictyostelium) | Homo sapiens |
| 864 | runt-related transcription factor 3 | Homo sapiens |
| 64417 | chromosome 5 open reading frame 28 | Homo sapiens |
| 84925 | disrupted in renal carcinoma 2 | Homo sapiens |
| 4774 | nuclear factor I/A | Homo sapiens |
| 1650 | dolichyl-diphosphooligosaccharide-protein glycosyltransferase | Homo sapiens |
| 6256 | retinoid X receptor, alpha | Homo sapiens |
| 6675 | UDP-N-acteylglucosamine pyrophosphorylase 1 | Homo sapiens |
| 64781 | ceramide kinase | Homo sapiens |
| 9921 | ring finger protein 10 | Homo sapiens |
| 25851 | tectonin beta-propeller repeat containing 1 | Homo sapiens |
| 388650 | family with sequence similarity 69, member A | Homo sapiens |
| 6253 | reticulon 2 | Homo sapiens |
| 51575 | similar to ABT1-associated protein; ESF1, nucleolar pre-rRNA processing protein, homolog (S. cerevisiae) | Homo sapiens |
| 51278 | immediate early response 5 | Homo sapiens |
| 160857 | coiled-coil domain containing 122 | Homo sapiens |
| 54543 | translocase of outer mitochondrial membrane 7 homolog (yeast) | Homo sapiens |
| 53354 | pantothenate kinase 1 | Homo sapiens |
| 1072 | cofilin 1 (non-muscle) | Homo sapiens |
| 80018 | chromosome 12 open reading frame 30 | Homo sapiens |
| 10745 | putative homeodomain transcription factor 1 | Homo sapiens |
| 367 | androgen receptor | Homo sapiens |
| 8218 | clathrin, heavy chain-like 1 | Homo sapiens |
| 79018 | chromosome 17 open reading frame 39 | Homo sapiens |
| 54556 | inhibitor of growth family, member 3 | Homo sapiens |
| 4697 | NADH dehydrogenase (ubiquinone) 1 alpha subcomplex, 4, 9kDa | Homo sapiens |
| 23315 | solute carrier family 9 (sodium/hydrogen exchanger), member 8 | Homo sapiens |
| 23347 | structural maintenance of chromosomes flexible hinge domain containing 1 | Homo sapiens |
| 84661 | dpy-30 homolog (C. elegans) | Homo sapiens |
| 51406 | nucleolar protein 7, 27kDa | Homo sapiens |
| 260294 | NOL1/NOP2/Sun domain family, member 5C | Homo sapiens |
| 11011 | tousled-like kinase 2 | Homo sapiens |
| 84210 | hCG2042718; chromosome 21 open reading frame 81; ankyrin repeat domain 20 family, member A1; ankyrin repeat domain 20 family, member A3; ankyrin repeat domain 20 family, member A2 | Homo sapiens |
| 441430 | hCG2042718; chromosome 21 open reading frame 81; ankyrin repeat domain 20 family, member A1; ankyrin repeat domain 20 family, member A3; ankyrin repeat domain 20 family, member A2 | Homo sapiens |
| 441425 | hCG2042718; chromosome 21 open reading frame 81; ankyrin repeat domain 20 family, member A1; ankyrin repeat domain 20 family, member A3; ankyrin repeat domain 20 family, member A2 | Homo sapiens |
| 391267 | hCG2042718; chromosome 21 open reading frame 81; ankyrin repeat domain 20 family, member A1; ankyrin repeat domain 20 family, member A3; ankyrin repeat domain 20 family, member A2 | Homo sapiens |
| 6397 | SEC14-like 1 (S. cerevisiae); SEC14-like 1 pseudogene | Homo sapiens |
| 1982 | eukaryotic translation initiation factor 4 gamma, 2 | Homo sapiens |
| 122830 | N-acetyltransferase 12 (GCN5-related, putative) | Homo sapiens |
| 28998 | mitochondrial ribosomal protein L13 | Homo sapiens |
| 8535 | chromobox homolog 4 (Pc class homolog, Drosophila) | Homo sapiens |
| 382 | ADP-ribosylation factor 6 | Homo sapiens |
| 7494 | X-box binding protein 1 | Homo sapiens |
| 79649 | MAP7 domain containing 3 | Homo sapiens |
| 23122 | cytoplasmic linker associated protein 2 | Homo sapiens |
| 10475 | tripartite motif-containing 38 | Homo sapiens |
| 8892 | eukaryotic translation initiation factor 2B, subunit 2 beta, 39kDa | Homo sapiens |

| | | |
|---|---|---|
| 389677 | RNA binding motif protein 12B | Homo sapiens |
| 79109 | mitogen-activated protein kinase associated protein 1 | Homo sapiens |
| 55450 | calcium/calmodulin-dependent protein kinase II inhibitor 1 | Homo sapiens |
| 729453 | PMS2 postmeiotic segregation increased 2 (S. cerevisiae)-like | Homo sapiens |
| 488 | ATPase, Ca++ transporting, cardiac muscle, slow twitch 2 | Homo sapiens |
| 29780 | parvin, beta | Homo sapiens |
| 100049716 | hypothetical protein LOC100049716 | Homo sapiens |
| 64777 | required for meiotic nuclear division 5 homolog B (S. cerevisiae) | Homo sapiens |
| 1445 | c-src tyrosine kinase | Homo sapiens |
| 79932 | KIAA0319-like | Homo sapiens |
| 55544 | RNA binding motif protein 38 | Homo sapiens |
| 4094 | v-maf musculoaponeurotic fibrosarcoma oncogene homolog (avian) | Homo sapiens |
| 22827 | poly-U binding splicing factor 60KDa | Homo sapiens |
| 114882 | oxysterol binding protein-like 8 | Homo sapiens |
| 143903 | layilin | Homo sapiens |
| 54808 | dymeclin | Homo sapiens |
| 56478 | eukaryotic translation initiation factor 4E nuclear import factor 1 | Homo sapiens |
| 51663 | zinc finger RNA binding protein | Homo sapiens |
| 79596 | ring finger protein 219 | Homo sapiens |
| 11179 | zinc finger protein 277 | Homo sapiens |
| 391356 | chromosome 2 open reading frame 79 | Homo sapiens |
| 91746 | YTH domain containing 1 | Homo sapiens |
| 339803 | hypothetical protein LOC339803 | Homo sapiens |
| 140690 | CCCTC-binding factor (zinc finger protein)-like | Homo sapiens |
| 323 | amyloid beta (A4) precursor protein-binding, family B, member 2 | Homo sapiens |
| 4747 | neurofilament, light polypeptide | Homo sapiens |
| 51720 | ubiquitin interaction motif containing 1 | Homo sapiens |
| 7520 | X-ray repair complementing defective repair in Chinese hamster cells 5 (double-strand-break rejoining) | Homo sapiens |
| 677778 | small Cajal body-specific RNA 15 | Homo sapiens |
| 10098 | tetraspanin 5 | Homo sapiens |
| 6788 | serine/threonine kinase 3 (STE20 homolog, yeast) | Homo sapiens |
| 83593 | Ras association (RalGDS/AF-6) domain family member 5 | Homo sapiens |
| 22861 | NLR family, pyrin domain containing 1 | Homo sapiens |
| 900 | cyclin G1 | Homo sapiens |
| 9949 | Alport syndrome, mental retardation, midface hypoplasia and elliptocytosis chromosomal region gene 1 | Homo sapiens |
| 10972 | transmembrane emp24-like trafficking protein 10 (yeast) | Homo sapiens |
| 83737 | itchy E3 ubiquitin protein ligase homolog (mouse) | Homo sapiens |
| 10048 | RAN binding protein 9 | Homo sapiens |
| 8225 | GTP binding protein 6 (putative) | Homo sapiens |
| 83540 | NUF2, NDC80 kinetochore complex component, homolog (S. cerevisiae) | Homo sapiens |
| 404266 | hypothetical LOC404266 | Homo sapiens |
| 26502 | nuclear prelamin A recognition factor | Homo sapiens |
| 7574 | zinc finger protein 26 | Homo sapiens |
| 10983 | cyclin I | Homo sapiens |
| 23534 | transportin 3 | Homo sapiens |
| 3988 | lipase A, lysosomal acid, cholesterol esterase | Homo sapiens |
| 57570 | TRM5 tRNA methyltransferase 5 homolog (S. cerevisiae) | Homo sapiens |
| 25853 | WD repeat domain 40A | Homo sapiens |
| 79866 | chromosome 13 open reading frame 34 | Homo sapiens |
| 2778 | GNAS complex locus | Homo sapiens |
| 81545 | F-box protein 38 | Homo sapiens |
| 128308 | mitochondrial ribosomal protein L55 | Homo sapiens |
| 10975 | ubiquinol-cytochrome c reductase, 6.4kDa subunit | Homo sapiens |
| 2619 | growth arrest-specific 1 | Homo sapiens |
| 57609 | DIP2 disco-interacting protein 2 homolog B (Drosophila) | Homo sapiens |
| 29083 | GTP-binding protein 8 (putative) | Homo sapiens |
| 7572 | zinc finger protein 24 | Homo sapiens |
| 51637 | chromosome 14 open reading frame 166 | Homo sapiens |
| 51517 | NCK interacting protein with SH3 domain | Homo sapiens |
| 63941 | N-terminal EF-hand calcium binding protein 3 | Homo sapiens |
| 2770 | guanine nucleotide binding protein (G protein), alpha inhibiting activity polypeptide 1 | Homo sapiens |
| 6526 | solute carrier family 5 (sodium/myo-inositol cotransporter), member 3 | Homo sapiens |
| 84321 | similar to THO complex 3; THO complex 3 | Homo sapiens |
| 728554 | similar to THO complex 3; THO complex 3 | Homo sapiens |
| 10396 | ATPase, aminophospholipid transporter (APLT), class I, type 8A, member 1 | Homo sapiens |
| 3215 | homeobox B5 | Homo sapiens |
| 2644 | GTP cyclohydrolase I feedback regulator | Homo sapiens |
| 11039 | glucuronidase, beta pseudogene | Homo sapiens |
| 3021 | H3 histone, family 3B (H3.3B); H3 histone, family 3A pseudogene; H3 histone, family 3A; similar to H3 histone, family 3B; similar to histone H3.3B | Homo sapiens |
| 440926 | H3 histone, family 3B (H3.3B); H3 histone, family 3A pseudogene; H3 histone, family 3A; similar to H3 histone, family 3B; similar to histone H3.3B | Homo sapiens |
| 3020 | H3 histone, family 3B (H3.3B); H3 histone, family 3A pseudogene; H3 histone, family 3A; similar to H3 histone, family 3B; similar to histone H3.3B | Homo sapiens |
| 738 | chromosome 11 open reading frame2 | Homo sapiens |

| | | |
|---|---|---|
| 583 | Bardet-Biedl syndrome 2 | Homo sapiens |
| 29934 | sorting nexin 12 | Homo sapiens |
| 143884 | CWF19-like 2, cell cycle control (S. pombe) | Homo sapiens |
| 55212 | Bardet-Biedl syndrome 7 | Homo sapiens |
| 132321 | chromosome 4 open reading frame 33 | Homo sapiens |
| 5879 | ras-related C3 botulinum toxin substrate 1 (rho family, small GTP binding protein Rac1) | Homo sapiens |
| 5862 | RAB2A, member RAS oncogene family | Homo sapiens |
| 57763 | ankyrin repeat, family A (RFXANK-like), 2 | Homo sapiens |
| 147184 | transmembrane protein 99 | Homo sapiens |
| 6446 | serum/glucocorticoid regulated kinase 1 | Homo sapiens |
| 140460 | ankyrin repeat and SOCS box-containing 7 | Homo sapiens |
| 26136 | testis derived transcript (3 LIM domains) | Homo sapiens |
| 23760 | phosphatidylinositol transfer protein, beta | Homo sapiens |
| 118 | adducin 1 (alpha) | Homo sapiens |
| 165324 | UBX domain protein 2A | Homo sapiens |
| 22937 | SREBF chaperone | Homo sapiens |
| 6230 | ribosomal protein S25 pseudogene 8; ribosomal protein S25 | Homo sapiens |
| 23093 | tubulin tyrosine ligase-like family, member 5 | Homo sapiens |
| 23261 | calmodulin binding transcription activator 1 | Homo sapiens |
| 3014 | H2A histone family, member X | Homo sapiens |
| 1937 | eukaryotic translation elongation factor 1 gamma | Homo sapiens |
| 4001 | lamin B1 | Homo sapiens |
| 7855 | frizzled homolog 5 (Drosophila) | Homo sapiens |
| 2296 | forkhead box C1 | Homo sapiens |
| 2495 | ferritin, heavy polypeptide 1; ferritin, heavy polypeptide-like 16; similar to ferritin, heavy polypeptide 1; ferritin, heavy polypeptide-like 3 pseudogene | Homo sapiens |
| 81929 | SEH1-like (S. cerevisiae) | Homo sapiens |
| 54830 | nucleoporin 62kDa C-terminal like | Homo sapiens |
| 11345 | GABA(A) receptor-associated protein-like 2 | Homo sapiens |
| 23633 | karyopherin alpha 6 (importin alpha 7) | Homo sapiens |
| 8204 | nuclear receptor interacting protein 1 | Homo sapiens |
| 4716 | NADH dehydrogenase (ubiquinone) 1 beta subcomplex, 10, 22kDa | Homo sapiens |
| 253143 | chromosome 22 open reading frame 30 | Homo sapiens |
| 23590 | prenyl (decaprenyl) diphosphate synthase, subunit 1 | Homo sapiens |
| 92342 | chromosome 1 open reading frame 156 | Homo sapiens |
| 205251 | non-protein coding RNA 116 | Homo sapiens |
| 51390 | androgen-induced 1 | Homo sapiens |
| 29070 | coiled-coil domain containing 113 | Homo sapiens |
| 91663 | myeloid-associated differentiation marker | Homo sapiens |
| 8428 | serine/threonine kinase 24 (STE20 homolog, yeast) | Homo sapiens |
| 659 | bone morphogenetic protein receptor, type II (serine/threonine kinase) | Homo sapiens |
| 890 | cyclin A2 | Homo sapiens |
| 2002 | ELK1, member of ETS oncogene family | Homo sapiens |
| 662 | BCL2/adenovirus E1B 19kDa interacting protein 1 | Homo sapiens |
| 51119 | Shwachman-Bodian-Diamond syndrome pseudogene; Shwachman-Bodian-Diamond syndrome | Homo sapiens |
| 155370 | Shwachman-Bodian-Diamond syndrome pseudogene; Shwachman-Bodian-Diamond syndrome | Homo sapiens |
| 728658 | ribosomal protein L13a pseudogene 7; ribosomal protein L13a pseudogene 5; ribosomal protein L13a pseudogene 16; ribosomal protein L13a; ribosomal protein L13a pseudogene 18 | Homo sapiens |
| 23521 | ribosomal protein L13a pseudogene 7; ribosomal protein L13a pseudogene 5; ribosomal protein L13a pseudogene 16; ribosomal protein L13a; ribosomal protein L13a pseudogene 18 | Homo sapiens |
| 51202 | DEAD (Asp-Glu-Ala-Asp) box polypeptide 47 | Homo sapiens |
| 9905 | small G protein signaling modulator 2 | Homo sapiens |
| 11049 | YDD19 protein | Homo sapiens |
| 1836 | solute carrier family 26 (sulfate transporter), member 2 | Homo sapiens |
| 144577 | chromosome 12 open reading frame 66 | Homo sapiens |
| 5584 | protein kinase C, iota | Homo sapiens |
| 5705 | proteasome (prosome, macropain) 26S subunit, ATPase, 5 | Homo sapiens |
| 51264 | mitochondrial ribosomal protein L27 | Homo sapiens |
| 587 | branched chain aminotransferase 2, mitochondrial | Homo sapiens |
| 127018 | lysophospholipase-like 1 | Homo sapiens |
| 65999 | leucine rich repeat containing 61 | Homo sapiens |
| 6133 | ribosomal protein L9; ribosomal protein L9 pseudogene 25 | Homo sapiens |
| 8031 | nuclear receptor coactivator 4 | Homo sapiens |
| 84938 | ATG4 autophagy related 4 homolog C (S. cerevisiae) | Homo sapiens |
| 8861 | LIM domain binding 1 | Homo sapiens |
| 9841 | zinc finger and BTB domain containing 24 | Homo sapiens |
| 3628 | inositol polyphosphate-1-phosphatase | Homo sapiens |
| 56954 | nitrilase family, member 2 | Homo sapiens |
| 8872 | cell division cycle 123 homolog (S. cerevisiae) | Homo sapiens |
| 5125 | proprotein convertase subtilisin/kexin type 5 | Homo sapiens |
| 85377 | MICAL-like 1 | Homo sapiens |
| 84923 | family with sequence similarity 104, member A | Homo sapiens |
| 221294 | 5'-nucleotidase domain containing 1 | Homo sapiens |
| 9202 | zinc finger, MYM-type 4 | Homo sapiens |
| 162681 | chromosome 18 open reading frame 54 | Homo sapiens |
| 284058 | KIAA1267 | Homo sapiens |
| 25901 | coiled-coil domain containing 28A | Homo sapiens |

| | | |
|---|---|---|
| 23362 | pleckstrin and Sec7 domain containing 3 | Homo sapiens |
| 55863 | transmembrane protein 126B | Homo sapiens |
| 6925 | transcription factor 4 | Homo sapiens |
| 145748 | LysM, putative peptidoglycan-binding, domain containing 4 | Homo sapiens |
| 163126 | EP300 interacting inhibitor of differentiation 2 | Homo sapiens |
| 10421 | CD2 (cytoplasmic tail) binding protein 2 | Homo sapiens |
| 6782 | heat shock protein 70kDa family, member 13 | Homo sapiens |
| 54751 | filamin binding LIM protein 1 | Homo sapiens |
| 81579 | phospholipase A2, group XIIA | Homo sapiens |
| 51326 | ADP-ribosylation factor-like 17 pseudogene 1; ADP-ribosylation factor-like 17 | Homo sapiens |
| 10428 | craniofacial development protein 1 | Homo sapiens |
| 4756 | neogenin homolog 1 (chicken) | Homo sapiens |
| 10749 | kinesin family member 1C | Homo sapiens |
| 201164 | phospholipase D family, member 6 | Homo sapiens |
| 3954 | leucine zipper-EF-hand containing transmembrane protein 1 | Homo sapiens |
| 9978 | ring-box 1 | Homo sapiens |
| 10961 | endoplasmic reticulum protein 29 | Homo sapiens |
| 6929 | transcription factor 3 (E2A immunoglobulin enhancer binding factors E12/E47) | Homo sapiens |
| 9670 | importin 13 | Homo sapiens |
| 79602 | adiponectin receptor 2 | Homo sapiens |
| 9668 | zinc finger protein 432 | Homo sapiens |
| 9647 | protein phosphatase 1F (PP2C domain containing) | Homo sapiens |
| 100132356 | hypothetical protein LOC100132356 | Homo sapiens |
| 7182 | nuclear receptor subfamily 2, group C, member 2 | Homo sapiens |
| 389765 | similar to KIF27C | Homo sapiens |
| 5783 | protein tyrosine phosphatase, non-receptor type 13 (APO-1/CD95 (Fas)-associated phosphatase) | Homo sapiens |
| 254170 | F-box protein 33 | Homo sapiens |
| 26175 | chromosome 14 open reading frame 109 | Homo sapiens |
| 27250 | programmed cell death 4 (neoplastic transformation inhibitor) | Homo sapiens |
| 23678 | serum/glucocorticoid regulated kinase family, member 3 | Homo sapiens |
| 92806 | hypothetical protein MGC16385 | Homo sapiens |
| 55700 | MAP7 domain containing 1 | Homo sapiens |
| 6322 | sex comb on midleg-like 1 (Drosophila) | Homo sapiens |
| 728661 | similar to solute carrier family 35, member E2 | Homo sapiens |
| 94103 | ORM1-like 3 (S. cerevisiae) | Homo sapiens |
| 26001 | ring finger protein 167 | Homo sapiens |
| 55527 | fem-1 homolog a (C. elegans); similar to fem-1 homolog a (C.elegans); similar to fem-1 homolog a | Homo sapiens |
| 8504 | peroxisomal biogenesis factor 3 | Homo sapiens |
| 5795 | protein tyrosine phosphatase, receptor type, J | Homo sapiens |
| 5015 | orthodenticle homeobox 2 | Homo sapiens |
| 3198 | homeobox A1 | Homo sapiens |
| 200576 | phosphoinositide kinase, FYVE finger containing | Homo sapiens |
| 4361 | MRE11 meiotic recombination 11 homolog A (S. cerevisiae) | Homo sapiens |
| 25994 | similar to HIG1 domain family, member 1A; HIG1 hypoxia inducible domain family, member 1A; HIG1 hypoxia inducible domain family, member 1D | Homo sapiens |
| 23176 | septin 8 | Homo sapiens |
| 23286 | WW and C2 domain containing 1 | Homo sapiens |
| 22894 | DIS3 mitotic control homolog (S. cerevisiae) | Homo sapiens |
| 253782 | LAG1 homolog, ceramide synthase 6 | Homo sapiens |
| 79828 | methyltransferase like 8 | Homo sapiens |
| 221830 | TWIST neighbor | Homo sapiens |
| 8034 | solute carrier family 25 (mitochondrial carrier; Graves disease autoantigen), member 16 | Homo sapiens |
| 7705 | zinc finger protein 146 | Homo sapiens |
| 22929 | selenophosphate synthetase 1; similar to selenophosphate synthetase 1 | Homo sapiens |
| 7170 | tropomyosin 3 | Homo sapiens |
| 117247 | solute carrier family 16, member 10 (aromatic amino acid transporter) | Homo sapiens |
| 7511 | X-prolyl aminopeptidase (aminopeptidase P) 1, soluble | Homo sapiens |
| 84081 | coiled-coil domain containing 55 | Homo sapiens |
| 83699 | SH3 domain binding glutamic acid-rich protein like 2 | Homo sapiens |
| 196294 | IMP1 inner mitochondrial membrane peptidase-like (S. cerevisiae) | Homo sapiens |
| 5501 | protein phosphatase 1, catalytic subunit, gamma isoform | Homo sapiens |
| 55116 | transmembrane protein 39B | Homo sapiens |
| 55783 | FtsJ methyltransferase domain containing 1 | Homo sapiens |
| 51100 | SH3-domain GRB2-like endophilin B1 | Homo sapiens |
| 3654 | interleukin-1 receptor-associated kinase 1 | Homo sapiens |
| 9812 | KIAA0141 | Homo sapiens |
| 6142 | ribosomal protein L18a pseudogene 6; ribosomal protein L18a | Homo sapiens |
| 842 | caspase 9, apoptosis-related cysteine peptidase | Homo sapiens |
| 10525 | hypoxia up-regulated 1 | Homo sapiens |
| 8487 | survival of motor neuron protein interacting protein 1 | Homo sapiens |
| 9667 | scaffold attachment factor B2 | Homo sapiens |
| 6934 | transcription factor 7-like 2 (T-cell specific, HMG-box) | Homo sapiens |
| 6416 | mitogen-activated protein kinase kinase 4 | Homo sapiens |
| 7728 | zinc finger protein 175 | Homo sapiens |
| 1912 | polyhomeotic homolog 2 (Drosophila) | Homo sapiens |

| | | |
|---|---|---|
| 203286 | ankyrin repeat and sterile alpha motif domain containing 6 | Homo sapiens |
| 400 | ADP-ribosylation factor-like 1 | Homo sapiens |
| 10723 | solute carrier family 12 (potassium/chloride transporters), member 7 | Homo sapiens |
| 57511 | component of oligomeric golgi complex 6 | Homo sapiens |
| 55165 | centrosomal protein 55kDa | Homo sapiens |
| 57862 | zinc finger protein 410 | Homo sapiens |
| 388722 | chromosome 1 open reading frame 53 | Homo sapiens |
| 6992 | protein phosphatase 1, regulatory (inhibitor) subunit 11 | Homo sapiens |
| 23463 | isoprenylcysteine carboxyl methyltransferase | Homo sapiens |
| 5599 | mitogen-activated protein kinase 8 | Homo sapiens |
| 2671 | growth factor, augmenter of liver regeneration | Homo sapiens |
| 5690 | proteasome (prosome, macropain) subunit, beta type, 2 | Homo sapiens |
| 10362 | high-mobility group 20B | Homo sapiens |
| 56950 | SET and MYND domain containing 2 | Homo sapiens |
| 6453 | intersectin 1 (SH3 domain protein) | Homo sapiens |
| 22976 | PAX interacting (with transcription-activation domain) protein 1 | Homo sapiens |
| 8819 | Sin3A-associated protein, 30kDa | Homo sapiens |
| 390535 | golgi autoantigen, golgin subfamily a, 8E | Homo sapiens |
| 285148 | isoamyl acetate-hydrolyzing esterase 1 homolog (S. cerevisiae) | Homo sapiens |
| 339983 | N-acetyltransferase 8-like (GCN5-related, putative) | Homo sapiens |
| 7626 | zinc finger protein 75D | Homo sapiens |
| 2045 | EPH receptor A7 | Homo sapiens |
| 254042 | methionine aminopeptidase 1D | Homo sapiens |
| 58490 | regulation of nuclear pre-mRNA domain containing 1B | Homo sapiens |
| 4189 | DnaJ (Hsp40) homolog, subfamily B, member 9 | Homo sapiens |
| 255252 | leucine rich repeat containing 57 | Homo sapiens |
| 79754 | ankyrin repeat and SOCS box-containing 13 | Homo sapiens |
| 137964 | 1-acylglycerol-3-phosphate O-acyltransferase 6 (lysophosphatidic acid acyltransferase, zeta) | Homo sapiens |
| 222068 | transmembrane emp24 protein transport domain containing 4 | Homo sapiens |
| 25888 | zinc finger protein 473 | Homo sapiens |
| 8407 | transgelin 2 | Homo sapiens |
| 54537 | family with sequence similarity 35, member A | Homo sapiens |
| 8463 | TEA domain family member 2 | Homo sapiens |
| 57222 | endoplasmic reticulum-golgi intermediate compartment (ERGIC) 1 | Homo sapiens |
| 50999 | transmembrane emp24 protein transport domain containing 5 | Homo sapiens |
| 79903 | N-acetyltransferase 15 (GCN5-related, putative) | Homo sapiens |
| 646903 | hypothetical LOC646903 | Homo sapiens |
| 10443 | NEDD4 binding protein 2-like 2 | Homo sapiens |
| 79877 | dephospho-CoA kinase domain containing | Homo sapiens |
| 51295 | ECSIT homolog (Drosophila) | Homo sapiens |
| 7782 | solute carrier family 30 (zinc transporter), member 4 | Homo sapiens |
| 22916 | nuclear cap binding protein subunit 2, 20kDa | Homo sapiens |
| 196513 | DCP1 decapping enzyme homolog B (S. cerevisiae) | Homo sapiens |
| 6926 | T-box 3 | Homo sapiens |
| 6612 | SMT3 suppressor of mif two 3 homolog 2 (S. cerevisiae) pseudogene; SMT3 suppressor of mif two 3 homolog 2 (S. cerevisiae); SMT3 suppressor of mif two 3 homolog 3 (S. cerevisiae) | Homo sapiens |
| 6613 | SMT3 suppressor of mif two 3 homolog 2 (S. cerevisiae) pseudogene; SMT3 suppressor of mif two 3 homolog 2 (S. cerevisiae); SMT3 suppressor of mif two 3 homolog 3 (S. cerevisiae) | Homo sapiens |
| 50854 | chromosome 6 open reading frame 48; small nucleolar RNA, C/D box 52 | Homo sapiens |
| 11328 | FK506 binding protein 9, 63 kDa | Homo sapiens |
| 7091 | transducin-like enhancer of split 4 (E(sp1) homolog, Drosophila) | Homo sapiens |
| 6314 | ataxin 7 | Homo sapiens |
| 23613 | zinc finger, MYND-type containing 8 | Homo sapiens |
| 162073 | inositol 1,4,5-triphosphate receptor interacting protein-like 2 | Homo sapiens |
| 55904 | myeloid/lymphoid or mixed-lineage leukemia 5 (trithorax homolog, Drosophila) | Homo sapiens |
| 7283 | tubulin, gamma 1; similar to Tubulin, gamma 1 | Homo sapiens |
| 10484 | Sec23 homolog A (S. cerevisiae) | Homo sapiens |
| 80342 | TRAF3 interacting protein 3 | Homo sapiens |
| 6777 | signal transducer and activator of transcription 5B | Homo sapiens |
| 84245 | methylthioribose-1-phosphate isomerase homolog (S. cerevisiae) | Homo sapiens |
| 54780 | non-SMC element 4 homolog A (S. cerevisiae) | Homo sapiens |
| 54708 | membrane-associated ring finger (C3HC4) 5 | Homo sapiens |
| 51315 | lysine-rich coiled-coil 1 | Homo sapiens |
| 79912 | pyridine nucleotide-disulphide oxidoreductase domain 1 | Homo sapiens |
| 92249 | hypothetical LOC92249 | Homo sapiens |
| 2627 | GATA binding protein 6 | Homo sapiens |
| 56848 | sphingosine kinase 2 | Homo sapiens |
| 57730 | similar to KIAA1641; similar to ankyrin repeat domain 26; ankyrin repeat domain 36B | Homo sapiens |
| 1973 | similar to eukaryotic translation initiation factor 4A; small nucleolar RNA, H/ACA box 67; eukaryotic translation initiation factor 4A, isoform 1 | Homo sapiens |
| 140606 | selenoprotein M | Homo sapiens |
| 83855 | Kruppel-like factor 16 | Homo sapiens |
| 25792 | CDKN1A interacting zinc finger protein 1 | Homo sapiens |
| 10721 | polymerase (DNA directed), theta | Homo sapiens |

| | | |
|---|---|---|
| 56681 | SAR1 homolog A (S. cerevisiae) | Homo sapiens |
| 55146 | zinc finger, DHHC-type containing 4 | Homo sapiens |
| 338758 | hypothetical protein LOC338758 | Homo sapiens |
| 283683 | hypothetical protein LOC283683 | Homo sapiens |
| 51526 | chromosome 20 open reading frame 111 | Homo sapiens |
| 80205 | chromodomain helicase DNA binding protein 9 | Homo sapiens |
| 26519 | translocase of inner mitochondrial membrane 10 homolog (yeast) | Homo sapiens |
| 11118 | butyrophilin, subfamily 3, member A2 | Homo sapiens |
| 7866 | interferon-related developmental regulator 2 | Homo sapiens |
| 29843 | SUMO1/sentrin specific peptidase 1 | Homo sapiens |
| 25865 | protein kinase D2 | Homo sapiens |
| 4950 | occludin pseudogene; occludin | Homo sapiens |
| 6217 | ribosomal protein S16 pseudogene 1; ribosomal protein S16 pseudogene 10; ribosomal protein S16 | Homo sapiens |
| 4087 | SMAD family member 2 | Homo sapiens |
| 55969 | chromosome 20 open reading frame 24 | Homo sapiens |
| 83641 | family with sequence similarity 107, member B | Homo sapiens |
| 653519 | G protein-coupled receptor 89C; G protein-coupled receptor 89B; G protein-coupled receptor 89A | Homo sapiens |
| 728932 | G protein-coupled receptor 89C; G protein-coupled receptor 89B; G protein-coupled receptor 89A | Homo sapiens |
| 51463 | G protein-coupled receptor 89C; G protein-coupled receptor 89B; G protein-coupled receptor 89A | Homo sapiens |
| 10691 | glucocorticoid modulatory element binding protein 1 | Homo sapiens |
| 163049 | zinc finger protein 791 | Homo sapiens |
| 152926 | protein phosphatase 1K (PP2C domain containing) | Homo sapiens |
| 116540 | mitochondrial ribosomal protein L53 | Homo sapiens |
| 109 | adenylate cyclase 3 | Homo sapiens |
| 8985 | procollagen-lysine, 2-oxoglutarate 5-dioxygenase 3 | Homo sapiens |
| 6129 | ribosomal protein L7 pseudogene 26; ribosomal protein L7 pseudogene 16; ribosomal protein L7; ribosomal protein L7 pseudogene 32; ribosomal protein L7 pseudogene 23; ribosomal protein L7 pseudogene 24; ribosomal protein L7 pseudogene 20 | Homo sapiens |
| 8848 | TSC22 domain family, member 1 | Homo sapiens |
| 134549 | shroom family member 1 | Homo sapiens |
| 4731 | NADH dehydrogenase (ubiquinone) flavoprotein 3, 10kDa | Homo sapiens |
| 387338 | NOL1/NOP2/Sun domain family, member 4 | Homo sapiens |
| 83658 | dynein, light chain, roadblock-type 1 | Homo sapiens |
| 64222 | torsin family 3, member A | Homo sapiens |
| 254295 | phytanoyl-CoA dioxygenase domain containing 1 | Homo sapiens |
| 29062 | WD repeat domain 91 | Homo sapiens |
| 197335 | WD repeat domain 90 | Homo sapiens |
| 9643 | mortality factor 4 like 2 | Homo sapiens |
| 23616 | SH3-domain binding protein 1 | Homo sapiens |
| 158293 | family with sequence similarity 120A opposite strand | Homo sapiens |
| 144699 | F-box and leucine-rich repeat protein 14 | Homo sapiens |
| 23612 | pleckstrin homology-like domain, family A, member 3 | Homo sapiens |
| 9315 | chromosome 5 open reading frame 13 | Homo sapiens |
| 117143 | transcriptional adaptor 1 (HFI1 homolog, yeast)-like | Homo sapiens |
| 4678 | nuclear autoantigenic sperm protein (histone-binding) | Homo sapiens |
| 8574 | aldo-keto reductase family 7, member A2 (aflatoxin aldehyde reductase) | Homo sapiens |
| 84516 | dynactin 5 (p25) | Homo sapiens |
| 414777 | HLA complex group 18 | Homo sapiens |
| 55683 | KIAA1310 | Homo sapiens |
| 6832 | suppressor of var1, 3-like 1 (S. cerevisiae) | Homo sapiens |
| 81555 | Yip1 domain family, member 5 | Homo sapiens |
| 54856 | YY1 associated protein 1; gon-4-like (C. elegans) | Homo sapiens |
| 55249 | YY1 associated protein 1; gon-4-like (C. elegans) | Homo sapiens |
| 84274 | coenzyme Q5 homolog, methyltransferase (S. cerevisiae) | Homo sapiens |
| 8192 | ClpP caseinolytic peptidase, ATP-dependent, proteolytic subunit homolog (E. coli) | Homo sapiens |
| 63916 | engulfment and cell motility 2 | Homo sapiens |
| 6662 | SRY (sex determining region Y)-box 9 | Homo sapiens |
| 9972 | nucleoporin 153kDa | Homo sapiens |
| 11073 | topoisomerase (DNA) II binding protein 1 | Homo sapiens |
| 54741 | leptin receptor overlapping transcript | Homo sapiens |
| 78988 | mitochondrial ribosomal protein 63 | Homo sapiens |
| 1869 | E2F transcription factor 1 | Homo sapiens |
| 80824 | dual specificity phosphatase 16 | Homo sapiens |
| 9069 | claudin 12 | Homo sapiens |
| 9128 | PRP4 pre-mRNA processing factor 4 homolog (yeast) | Homo sapiens |
| 663 | BCL2/adenovirus E1B 19kDa interacting protein 2 | Homo sapiens |
| 51652 | vacuolar protein sorting 24 homolog (S. cerevisiae); ring finger protein 103 | Homo sapiens |
| 7844 | vacuolar protein sorting 24 homolog (S. cerevisiae); ring finger protein 103 | Homo sapiens |
| 121441 | neural precursor cell expressed, developmentally down-regulated 1 | Homo sapiens |
| 9685 | clathrin interactor 1 | Homo sapiens |
| 150274 | HscB iron-sulfur cluster co-chaperone homolog (E. coli) | Homo sapiens |
| 9955 | heparan sulfate (glucosamine) 3-O-sulfotransferase 3A1 | Homo sapiens |
| 28977 | mitochondrial ribosomal protein L42 | Homo sapiens |

| | | |
|---|---|---|
| 11034 | destrin (actin depolymerizing factor) | Homo sapiens |
| 11176 | bromodomain adjacent to zinc finger domain, 2A | Homo sapiens |
| 128869 | phosphatidylinositol glycan anchor biosynthesis, class U | Homo sapiens |
| 8609 | Kruppel-like factor 7 (ubiquitous) | Homo sapiens |
| 27440 | cat eye syndrome chromosome region, candidate 5 | Homo sapiens |
| 7321 | ubiquitin-conjugating enzyme E2D 1 (UBC4/5 homolog, yeast) | Homo sapiens |
| 253461 | zinc finger and BTB domain containing 38 | Homo sapiens |
| 9748 | STE20-like kinase (yeast) | Homo sapiens |
| 5111 | proliferating cell nuclear antigen | Homo sapiens |
| 23528 | zinc finger protein 281 | Homo sapiens |
| 64743 | WD repeat domain 13 | Homo sapiens |
| 51649 | mitochondrial ribosomal protein S23 | Homo sapiens |
| 55900 | zinc finger protein 302 | Homo sapiens |
| 4247 | mannosyl (alpha-1,6-)-glycoprotein beta-1,2-N-acetylglucosaminyltransferase | Homo sapiens |
| 9692 | KIAA0391 | Homo sapiens |
| 3398 | inhibitor of DNA binding 2, dominant negative helix-loop-helix protein | Homo sapiens |
| 26058 | GRB10 interacting GYF protein 2 | Homo sapiens |
| 51654 | CDK5 regulatory subunit associated protein 1 | Homo sapiens |
| 11282 | mannosyl (alpha-1,3-)-glycoprotein beta-1,4-N-acetylglucosaminyltransferase, isozyme B | Homo sapiens |
| 26520 | translocase of inner mitochondrial membrane 9 homolog (yeast) | Homo sapiens |
| 353 | adenine phosphoribosyltransferase | Homo sapiens |
| 1611 | death-associated protein | Homo sapiens |
| 196 | aryl hydrocarbon receptor | Homo sapiens |
| 9711 | KIAA0226 | Homo sapiens |
| 9652 | tetratricopeptide repeat domain 37 | Homo sapiens |
| 52 | acid phosphatase 1, soluble | Homo sapiens |
| 9294 | sphingosine-1-phosphate receptor 2 | Homo sapiens |
| 153768 | PRELI domain containing 2 | Homo sapiens |
| 79145 | coiled-coil-helix-coiled-coil-helix domain containing 7 | Homo sapiens |
| 85013 | transmembrane protein 128 | Homo sapiens |
| 54458 | proline rich 13 | Homo sapiens |
| 23254 | kazrin | Homo sapiens |
| 64689 | golgi reassembly stacking protein 1, 65kDa | Homo sapiens |
| 157922 | calmodulin regulated spectrin-associated protein 1 | Homo sapiens |
| 27102 | eukaryotic translation initiation factor 2-alpha kinase 1 | Homo sapiens |
| 55680 | RUN and FYVE domain containing 2 | Homo sapiens |
| 998 | cell division cycle 42 (GTP binding protein, 25kDa); cell division cycle 42 pseudogene 2 | Homo sapiens |
| 677 | zinc finger protein 36, C3H type-like 1 | Homo sapiens |
| 4781 | nuclear factor I/B | Homo sapiens |
| 8507 | ectodermal-neural cortex (with BTB-like domain) | Homo sapiens |
| 3704 | inosine triphosphatase (nucleoside triphosphate pyrophosphatase) | Homo sapiens |
| 84271 | polymerase (DNA-directed), delta interacting protein 3 | Homo sapiens |
| 1802 | DPH2 homolog (S. cerevisiae) | Homo sapiens |
| 115201 | ATG4 autophagy related 4 homolog A (S. cerevisiae) | Homo sapiens |
| 113179 | adenosine deaminase, tRNA-specific 3, TAD3 homolog (S. cerevisiae) | Homo sapiens |
| 9451 | eukaryotic translation initiation factor 2-alpha kinase 3 | Homo sapiens |
| 50861 | stathmin-like 3 | Homo sapiens |
| 2632 | glucan (1,4-alpha-), branching enzyme 1 | Homo sapiens |
| 7483 | wingless-type MMTV integration site family, member 9A | Homo sapiens |
| 403 | ADP-ribosylation factor-like 3 | Homo sapiens |
| 163778 | small proline-rich protein 4 | Homo sapiens |
| 80745 | THUMP domain containing 2 | Homo sapiens |
| 4968 | 8-oxoguanine DNA glycosylase | Homo sapiens |
| 439949 | hypothetical protein LOC439949 | Homo sapiens |
| 6138 | ribosomal protein L15 pseudogene 22; ribosomal protein L15 pseudogene 18; ribosomal protein L15 pseudogene 17; ribosomal protein L15 pseudogene 3; ribosomal protein L15 pseudogene 7; ribosomal protein L15 | Homo sapiens |
| 6990 | dynein, light chain, Tctex-type 3 | Homo sapiens |
| 57003 | coiled-coil domain containing 47 | Homo sapiens |
| 94056 | synapse associated protein 1, SAP47 homolog (Drosophila) | Homo sapiens |
| 23788 | mitochondrial carrier homolog 2 (C. elegans) | Homo sapiens |
| 119559 | sideroflexin 4 | Homo sapiens |
| 54920 | dihydrouridine synthase 2-like, SMM1 homolog (S. cerevisiae) | Homo sapiens |
| 26268 | F-box protein 9 | Homo sapiens |
| 136647 | chromosome 7 open reading frame 11 | Homo sapiens |
| 55839 | centromere protein N | Homo sapiens |
| 9130 | family with sequence similarity 50, member A | Homo sapiens |
| 83461 | cell division cycle associated 3 | Homo sapiens |
| 388796 | hypothetical LOC388796 | Homo sapiens |
| 262 | adenosylmethionine decarboxylase 1 | Homo sapiens |
| 3239 | homeobox D13 | Homo sapiens |
| 157753 | transmembrane protein 74 | Homo sapiens |
| 9135 | rabaptin, RAB GTPase binding effector protein 1 | Homo sapiens |
| 5723 | phosphoserine phosphatase-like; phosphoserine phosphatase | Homo sapiens |
| 282991 | biogenesis of lysosomal organelles complex-1, subunit 2 | Homo sapiens |
| 84912 | solute carrier family 35, member B4 | Homo sapiens |

| | | |
|---|---|---|
| 2224 | farnesyl diphosphate synthase (farnesyl pyrophosphate synthetase, dimethylallyltranstransferase, geranyltranstransferase) | Homo sapiens |
| 124808 | coiled-coil domain containing 43 | Homo sapiens |
| 26580 | Bernardinelli-Seip congenital lipodystrophy 2 (seipin) | Homo sapiens |
| 132789 | glucosamine-6-phosphate deaminase 2 | Homo sapiens |
| 10437 | interferon, gamma-inducible protein 30 | Homo sapiens |
| 154661 | RUN domain containing 3B | Homo sapiens |
| 7172 | thiopurine S-methyltransferase | Homo sapiens |
| 5250 | solute carrier family 25 (mitochondrial carrier; phosphate carrier), member 3 | Homo sapiens |
| 1725 | deoxyhypusine synthase | Homo sapiens |
| 57579 | family with sequence similarity 135, member A | Homo sapiens |
| 23303 | kinesin family member 13B | Homo sapiens |
| 55631 | leucine rich repeat containing 40 | Homo sapiens |
| 22876 | inositol polyphosphate-5-phosphatase F | Homo sapiens |
| 84316 | LSM domain containing 1 | Homo sapiens |
| 10463 | solute carrier family 30 (zinc transporter), member 9 | Homo sapiens |
| 10424 | progesterone receptor membrane component 2 | Homo sapiens |
| 1716 | deoxyguanosine kinase | Homo sapiens |
| 23507 | leucine rich repeat containing 8 family, member B | Homo sapiens |
| 7162 | trophoblast glycoprotein | Homo sapiens |
| 9731 | KIAA0562 | Homo sapiens |
| 4983 | oligophrenin 1 | Homo sapiens |
| 23387 | serine/threonine-protein kinase QSK | Homo sapiens |
| 375704 | energy homeostasis associated | Homo sapiens |
| 60485 | salvador homolog 1 (Drosophila) | Homo sapiens |
| 7130 | tumor necrosis factor, alpha-induced protein 6 | Homo sapiens |
| 1058 | centromere protein A | Homo sapiens |
| 55425 | KIAA1704 | Homo sapiens |
| 28970 | chromosome 11 open reading frame 54 | Homo sapiens |
| 6120 | rcRPE; ribulose-5-phosphate-3-epimerase | Homo sapiens |
| 5034 | prolyl 4-hydroxylase, beta polypeptide | Homo sapiens |
| 118487 | coiled-coil-helix-coiled-coil-helix domain containing 1 | Homo sapiens |
| 23065 | KIAA0090 | Homo sapiens |
| 23054 | nuclear receptor coactivator 6 | Homo sapiens |
| 60343 | family with sequence similarity 3, member A | Homo sapiens |
| 8703 | UDP-Gal:betaGlcNAc beta 1,4- galactosyltransferase, polypeptide 3 | Homo sapiens |
| 55486 | presenilin associated, rhomboid-like | Homo sapiens |
| 10808 | heat shock 105kDa/110kDa protein 1 | Homo sapiens |
| 114825 | PWWP domain containing 2A | Homo sapiens |
| 23641 | leucine zipper, down-regulated in cancer 1 | Homo sapiens |
| 5252 | PHD finger protein 1 | Homo sapiens |
| 112616 | CKLF-like MARVEL transmembrane domain containing 7 | Homo sapiens |
| 201965 | RWD domain containing 4A | Homo sapiens |
| 5434 | polymerase (RNA) II (DNA directed) polypeptide E, 25kDa | Homo sapiens |
| 1070 | centrin, EF-hand protein, 3 (CDC31 homolog, yeast) | Homo sapiens |
| 6513 | solute carrier family 2 (facilitated glucose transporter), member 1 | Homo sapiens |
| 5203 | prefoldin subunit 4 | Homo sapiens |
| 2218 | fukutin | Homo sapiens |
| 51375 | sorting nexin 7 | Homo sapiens |
| 4508 | Cytochrome c oxidase subunit 3; ATP synthase subunit a; ATP synthase protein 8 | Homo sapiens |
| 30837 | suppressor of cytokine signaling 7 | Homo sapiens |
| 5725 | polypyrimidine tract binding protein 1 | Homo sapiens |
| 26354 | guanine nucleotide binding protein-like 3 (nucleolar) | Homo sapiens |
| 53981 | cleavage and polyadenylation specific factor 2, 100kDa | Homo sapiens |
| 5213 | phosphofructokinase, muscle | Homo sapiens |
| 9620 | cadherin, EGF LAG seven-pass G-type receptor 1 (flamingo homolog, Drosophila) | Homo sapiens |
| 8732 | RNA guanylyltransferase and 5'-phosphatase | Homo sapiens |
| 2957 | general transcription factor IIA, 1, 19/37kDa | Homo sapiens |
| 253868 | chromosome 20 open reading frame 200 | Homo sapiens |
| 23786 | BCL2-like 13 (apoptosis facilitator) | Homo sapiens |
| 80167 | chromosome 4 open reading frame 29 | Homo sapiens |
| 134429 | StAR-related lipid transfer (START) domain containing 4 | Homo sapiens |
| 157247 | hypothetical protein MGC27345 | Homo sapiens |
| 85459 | KIAA1731 | Homo sapiens |
| 8569 | MAP kinase interacting serine/threonine kinase 1 | Homo sapiens |
| 84299 | chromosome 17 open reading frame 37 | Homo sapiens |
| 57089 | ectonucleoside triphosphate diphosphohydrolase 7 | Homo sapiens |
| 23404 | exosome component 2 | Homo sapiens |
| 2649 | nuclear receptor subfamily 6, group A, member 1 | Homo sapiens |
| 155435 | RNA binding motif protein 33 | Homo sapiens |
| 28987 | NIN1/RPN12 binding protein 1 homolog (S. cerevisiae); hypothetical LOC100132364 | Homo sapiens |
| 9833 | maternal embryonic leucine zipper kinase | Homo sapiens |
| 29086 | chromosome 19 open reading frame 62 | Homo sapiens |
| 83989 | family with sequence similarity 172, member A | Homo sapiens |
| 5613 | protein kinase, X-linked | Homo sapiens |
| 23683 | protein kinase D3 | Homo sapiens |
| 126382 | nuclear receptor 2C2-associated protein | Homo sapiens |

| | | |
|---|---|---|
| 4670 | heterogeneous nuclear ribonucleoprotein M | Homo sapiens |
| 3226 | homeobox C10 | Homo sapiens |
| 1665 | DEAH (Asp-Glu-Ala-His) box polypeptide 15 | Homo sapiens |
| 131566 | discoidin, CUB and LCCL domain containing 2 | Homo sapiens |
| 1875 | E2F transcription factor 5, p130-binding | Homo sapiens |
| 128061 | chromosome 1 open reading frame 131 | Homo sapiens |
| 93973 | ARP8 actin-related protein 8 homolog (yeast) | Homo sapiens |
| 7248 | tuberous sclerosis 1 | Homo sapiens |
| 8836 | gamma-glutamyl hydrolase (conjugase, folylpolygammaglutamyl hydrolase) | Homo sapiens |
| 283742 | family with sequence similarity 98, member B | Homo sapiens |
| 23064 | senataxin | Homo sapiens |
| 3396 | immature colon carcinoma transcript 1 | Homo sapiens |
| 254048 | ubinuclein 2 | Homo sapiens |
| 9477 | mediator complex subunit 20 | Homo sapiens |
| 51398 | chromosome 19 open reading frame 56 | Homo sapiens |
| 11033 | ArfGAP with dual PH domains 1 | Homo sapiens |
| 9701 | SAPS domain family, member 2 | Homo sapiens |
| 3099 | hexokinase 2 pseudogene; hexokinase 2 | Homo sapiens |
| 162967 | zinc finger protein 320 | Homo sapiens |
| 5522 | protein phosphatase 2 (formerly 2A), regulatory subunit B, gamma isoform | Homo sapiens |
| 7314 | ubiquitin B | Homo sapiens |
| 55729 | activating transcription factor 7 interacting protein | Homo sapiens |
| 494143 | ChaC, cation transport regulator homolog 2 (E. coli) | Homo sapiens |
| 87 | actinin, alpha 1 | Homo sapiens |
| 54662 | TBC1 domain family, member 13 | Homo sapiens |
| 5577 | protein kinase, cAMP-dependent, regulatory, type II, beta | Homo sapiens |
| 55379 | leucine rich repeat containing 59 | Homo sapiens |
| 57679 | amyotrophic lateral sclerosis 2 (juvenile) | Homo sapiens |
| 908 | chaperonin containing TCP1, subunit 6A (zeta 1) | Homo sapiens |
| 22870 | SAPS domain family, member 1 | Homo sapiens |
| 28981 | intraflagellar transport 81 homolog (Chlamydomonas) | Homo sapiens |
| 114794 | extracellular leucine-rich repeat and fibronectin type III domain containing 2 | Homo sapiens |
| 84172 | polymerase (RNA) I polypeptide B, 128kDa | Homo sapiens |
| 79080 | coiled-coil domain containing 86 | Homo sapiens |
| 7465 | WEE1 homolog (S. pombe) | Homo sapiens |
| 114883 | oxysterol binding protein-like 9 | Homo sapiens |
| 140901 | serine/threonine kinase 35 | Homo sapiens |
| 80318 | G kinase anchoring protein 1 | Homo sapiens |
| 200942 | kelch domain containing 8B | Homo sapiens |
| 7485 | tryptophan rich basic protein | Homo sapiens |
| 203414 | chromosome X open reading frame 24 | Homo sapiens |
| 57209 | zinc finger protein 248 | Homo sapiens |
| 135112 | nuclear receptor coactivator 7 | Homo sapiens |
| 5130 | phosphate cytidylyltransferase 1, choline, alpha | Homo sapiens |
| 55035 | nucleolar protein 8 | Homo sapiens |
| 11059 | WW domain containing E3 ubiquitin protein ligase 1 | Homo sapiens |
| 11232 | polymerase (DNA directed), gamma 2, accessory subunit | Homo sapiens |
| 51479 | ankyrin repeat and FYVE domain containing 1 | Homo sapiens |
| 650794 | similar to FRAS1 related extracellular matrix protein 2 | Homo sapiens |
| 25978 | chromatin modifying protein 2B | Homo sapiens |
| 80351 | tankyrase, TRF1-interacting ankyrin-related ADP-ribose polymerase 2 | Homo sapiens |
| 5395 | PMS2 postmeiotic segregation increased 2 (S. cerevisiae) | Homo sapiens |
| 9380 | glyoxylate reductase/hydroxypyruvate reductase | Homo sapiens |
| 128387 | TatD DNase domain containing 3 | Homo sapiens |
| 10927 | spindlin 1 | Homo sapiens |
| 64976 | mitochondrial ribosomal protein L40 | Homo sapiens |
| 54802 | tRNA isopentenyltransferase 1 | Homo sapiens |
| 84187 | transmembrane protein 164 | Homo sapiens |
| 25804 | LSM4 homolog, U6 small nuclear RNA associated (S. cerevisiae) | Homo sapiens |
| 51205 | acid phosphatase 6, lysophosphatidic | Homo sapiens |
| 283455 | kinase suppressor of ras 2 | Homo sapiens |
| 54880 | BCL6 co-repressor | Homo sapiens |
| 224 | aldehyde dehydrogenase 3 family, member A2 | Homo sapiens |
| 387893 | SET domain containing (lysine methyltransferase) 8 | Homo sapiens |
| 29980 | downstream neighbor of SON | Homo sapiens |
| 4809 | NHP2 non-histone chromosome protein 2-like 1 (S. cerevisiae) | Homo sapiens |
| 3276 | protein arginine methyltransferase 1 | Homo sapiens |
| 51092 | SID1 transmembrane family, member 2 | Homo sapiens |
| 474170 | leucine rich repeat containing 37, member A2 | Homo sapiens |
| 51530 | zinc finger, C3HC-type containing 1 | Homo sapiens |
| 112939 | nucleus accumbens associated 1, BEN and BTB (POZ) domain containing | Homo sapiens |
| 391632 | olfactory receptor, family 7, subfamily E, member 35 pseudogene | Homo sapiens |
| 23411 | sirtuin (silent mating type information regulation 2 homolog) 1 (S. cerevisiae) | Homo sapiens |
| 8491 | mitogen-activated protein kinase kinase kinase kinase 3 | Homo sapiens |
| 116832 | ribosomal protein L39-like | Homo sapiens |
| 55814 | B double prime 1, subunit of RNA polymerase III transcription initiation factor IIIB | Homo sapiens |
| 1486 | chitobiase, di-N-acetyl- | Homo sapiens |

| | | |
|---|---|---|
| 6194 | ribosomal protein S6 pseudogene 25; ribosomal protein S6; ribosomal protein S6 pseudogene 1 | Homo sapiens |
| 51074 | APAF1 interacting protein; similar to APAF1 interacting protein | Homo sapiens |
| 10389 | sex comb on midleg-like 2 (Drosophila) | Homo sapiens |
| 51439 | family with sequence similarity 8, member A1 | Homo sapiens |
| 22820 | coatomer protein complex, subunit gamma | Homo sapiens |
| 81628 | TSC22 domain family, member 4 | Homo sapiens |
| 25886 | WD repeat domain 51A | Homo sapiens |
| 1121 | choroideremia (Rab escort protein 1) | Homo sapiens |
| 10069 | RWD domain containing 2B | Homo sapiens |
| 10465 | peptidylprolyl isomerase H (cyclophilin H) | Homo sapiens |
| 25801 | grancalcin, EF-hand calcium binding protein | Homo sapiens |
| 90410 | intraflagellar transport 20 homolog (Chlamydomonas) | Homo sapiens |
| 10935 | peroxiredoxin 3 | Homo sapiens |
| 9933 | KIAA0020 | Homo sapiens |
| 23266 | latrophilin 2 | Homo sapiens |
| 646762 | hypothetical LOC646762 | Homo sapiens |
| 80895 | integrin-linked kinase-associated serine/threonine phosphatase 2C | Homo sapiens |
| 55171 | TBCC domain containing 1 | Homo sapiens |
| 55156 | armadillo repeat containing 1 | Homo sapiens |
| 6432 | splicing factor, arginine/serine-rich 7, 35kDa | Homo sapiens |
| 9941 | endo/exonuclease (5'-3'), endonuclease G-like | Homo sapiens |
| 23272 | chromosome 3 open reading frame 63 | Homo sapiens |
| 354 | kallikrein-related peptidase 3 | Homo sapiens |
| 7082 | tight junction protein 1 (zona occludens 1) | Homo sapiens |
| 166815 | tigger transposable element derived 2 | Homo sapiens |
| 3607 | forkhead box K2 | Homo sapiens |
| 285830 | hypothetical LOC285830 | Homo sapiens |
| 8925 | hect (homologous to the E6-AP (UBE3A) carboxyl terminus) domain and RCC1 (CHC1)-like domain (RLD) 1 | Homo sapiens |
| 23020 | similar to U5 snRNP-specific protein, 200 kDa; small nuclear ribonucleoprotein 200kDa (U5) | Homo sapiens |
| 388327 | chromosome 17 open reading frame 100 | Homo sapiens |
| 387763 | hypothetical protein LOC387763 | Homo sapiens |
| 8682 | phosphoprotein enriched in astrocytes 15 | Homo sapiens |
| 4710 | hypothetical gene supported by AF044957; NM_004547; NADH dehydrogenase (ubiquinone) 1 beta subcomplex, 4, 15kDa | Homo sapiens |
| 84930 | microtubule associated serine/threonine kinase-like | Homo sapiens |
| 28958 | coiled-coil domain containing 56 | Homo sapiens |
| 4709 | NADH dehydrogenase (ubiquinone) 1 beta subcomplex, 3, 12kDa | Homo sapiens |
| 3235 | homeobox D9 | Homo sapiens |
| 1265 | calponin 2 | Homo sapiens |
| 10090 | uronyl-2-sulfotransferase | Homo sapiens |
| 396 | Rho GDP dissociation inhibitor (GDI) alpha | Homo sapiens |
| 9410 | small nuclear ribonucleoprotein 40kDa (U5) | Homo sapiens |
| 10687 | paraneoplastic antigen MA2 | Homo sapiens |
| 51253 | mitochondrial ribosomal protein L37 | Homo sapiens |
| 120 | adducin 3 (gamma) | Homo sapiens |
| 85451 | unkempt homolog (Drosophila) | Homo sapiens |
| 514 | ATP synthase, H+ transporting, mitochondrial F1 complex, epsilon subunit pseudogene 2; ATP synthase, H+ transporting, mitochondrial F1 complex, epsilon subunit | Homo sapiens |
| 2135 | exostoses (multiple)-like 2 | Homo sapiens |
| 55157 | aspartyl-tRNA synthetase 2, mitochondrial | Homo sapiens |
| 285282 | RAB, member of RAS oncogene family-like 3 | Homo sapiens |
| 6646 | sterol O-acyltransferase 1 | Homo sapiens |
| 284370 | zinc finger protein 615 | Homo sapiens |
| 444 | aspartate beta-hydroxylase | Homo sapiens |
| 55186 | solute carrier family 25, member 36 | Homo sapiens |
| 80145 | THO complex 7 homolog (Drosophila) | Homo sapiens |
| 55081 | intraflagellar transport 57 homolog (Chlamydomonas) | Homo sapiens |
| 152579 | sec1 family domain containing 2 | Homo sapiens |
| 3480 | insulin-like growth factor 1 receptor | Homo sapiens |
| 26009 | zinc finger, ZZ-type containing 3 | Homo sapiens |
| 26168 | SUMO1/sentrin/SMT3 specific peptidase 3 | Homo sapiens |
| 25870 | sulfatase modifying factor 2 | Homo sapiens |
| 5087 | pre-B-cell leukemia homeobox 1 | Homo sapiens |
| 55686 | melanoregulin | Homo sapiens |
| 493869 | glutathione peroxidase 8 (putative) | Homo sapiens |
| 163081 | zinc finger protein 567 | Homo sapiens |
| 25 | c-abl oncogene 1, receptor tyrosine kinase | Homo sapiens |
| 1741 | discs, large homolog 3 (Drosophila) | Homo sapiens |
| 117178 | synovial sarcoma, X breakpoint 2 interacting protein | Homo sapiens |
| 8209 | chromosome 21 open reading frame 33 | Homo sapiens |
| 283267 | hypothetical LOC283267 | Homo sapiens |
| 339175 | methyltransferase like 2A | Homo sapiens |
| 2782 | guanine nucleotide binding protein (G protein), beta polypeptide 1 | Homo sapiens |
| 57826 | RAP2C, member of RAS oncogene family | Homo sapiens |

| | | |
|---|---|---|
| 5984 | replication factor C (activator 1) 4, 37kDa | Homo sapiens |
| 10600 | ubiquitin specific peptidase 16 | Homo sapiens |
| 5569 | protein kinase (cAMP-dependent, catalytic) inhibitor alpha | Homo sapiens |
| 113201 | cancer susceptibility candidate 4 | Homo sapiens |
| 246243 | ribonuclease H1 | Homo sapiens |
| 23283 | cleavage stimulation factor, 3' pre-RNA, subunit 2, 64kDa, tau variant | Homo sapiens |
| 5589 | protein kinase C substrate 80K-H | Homo sapiens |
| 26088 | golgi associated, gamma adaptin ear containing, ARF binding protein 1 | Homo sapiens |
| 257397 | mitogen-activated protein kinase kinase kinase 7 interacting protein 3 | Homo sapiens |
| 80314 | enhancer of polycomb homolog 1 (Drosophila) | Homo sapiens |
| 3070 | helicase, lymphoid-specific | Homo sapiens |
| 158427 | chromosome 9 open reading frame 97 | Homo sapiens |
| 11218 | DEAD (Asp-Glu-Ala-Asp) box polypeptide 20 | Homo sapiens |
| 9373 | phospholipase A2-activating protein | Homo sapiens |
| 9991 | ROD1 regulator of differentiation 1 (S. pombe) | Homo sapiens |
| 29965 | chromosome 16 open reading frame 5 | Homo sapiens |
| 1203 | ceroid-lipofuscinosis, neuronal 5 | Homo sapiens |
| 441194 | PMS2 C-terminal like pseudogene | Homo sapiens |
| 51203 | nucleolar and spindle associated protein 1 | Homo sapiens |
| 79844 | zinc finger, DHHC-type containing 11 | Homo sapiens |
| 10105 | peptidylprolyl isomerase F | Homo sapiens |
| 55230 | ubiquitin specific peptidase 40 | Homo sapiens |
| 23358 | ubiquitin specific peptidase 24 | Homo sapiens |
| 9736 | ubiquitin specific peptidase 34 | Homo sapiens |
| 57223 | SMEK homolog 2, suppressor of mek1 (Dictyostelium) | Homo sapiens |
| 84273 | chromosome 4 open reading frame 14 | Homo sapiens |
| 163051 | zinc finger protein 709 | Homo sapiens |
| 51107 | anterior pharynx defective 1 homolog A (C. elegans) | Homo sapiens |
| 79172 | centromere protein O | Homo sapiens |
| 1600 | disabled homolog 1 (Drosophila) | Homo sapiens |
| 6468 | F-box and WD repeat domain containing 4 | Homo sapiens |
| 1788 | DNA (cytosine-5-)-methyltransferase 3 alpha | Homo sapiens |
| 8464 | suppressor of Ty 3 homolog (S. cerevisiae) | Homo sapiens |
| 493911 | phosphatase, orphan 2 | Homo sapiens |
| 58477 | signal recognition particle receptor, B subunit | Homo sapiens |
| 7534 | tyrosine 3-monooxygenase/tryptophan 5-monooxygenase activation protein, zeta polypeptide | Homo sapiens |
| 10300 | katanin p80 (WD repeat containing) subunit B 1 | Homo sapiens |
| 80097 | family with sequence similarity 128, member B | Homo sapiens |
| 123283 | threonyl-tRNA synthetase-like 2 | Homo sapiens |
| 55193 | polybromo 1 | Homo sapiens |
| 130589 | galactose mutarotase (aldose 1-epimerase) | Homo sapiens |
| 221937 | forkhead box K1 | Homo sapiens |
| 8462 | Kruppel-like factor 11 | Homo sapiens |
| 54893 | myotubularin related protein 10 | Homo sapiens |
| 5277 | phosphatidylinositol glycan anchor biosynthesis, class A | Homo sapiens |
| 26577 | procollagen C-endopeptidase enhancer 2 | Homo sapiens |
| 57338 | junctophilin 3 | Homo sapiens |
| 10945 | KDEL (Lys-Asp-Glu-Leu) endoplasmic reticulum protein retention receptor 1 | Homo sapiens |
| 50814 | NAD(P) dependent steroid dehydrogenase-like | Homo sapiens |
| 10654 | phosphomevalonate kinase | Homo sapiens |
| 5440 | polymerase (RNA) II (DNA directed) polypeptide K, 7.0kDa | Homo sapiens |
| 79874 | rabaptin, RAB GTPase binding effector protein 2 | Homo sapiens |
| 54832 | vacuolar protein sorting 13 homolog C (S. cerevisiae) | Homo sapiens |
| 23626 | SPO11 meiotic protein covalently bound to DSB homolog (S. cerevisiae) | Homo sapiens |
| 55578 | family with sequence similarity 48, member A | Homo sapiens |
| 5441 | polymerase (RNA) II (DNA directed) polypeptide L, 7.6kDa | Homo sapiens |
| 84294 | UTP23, small subunit (SSU) processome component, homolog (yeast) | Homo sapiens |
| 26022 | similar to transmembrane protein 98; transmembrane protein 98 | Homo sapiens |
| 138050 | heparan-alpha-glucosaminide N-acetyltransferase | Homo sapiens |
| 5049 | platelet-activating factor acetylhydrolase, isoform Ib, subunit 2 (30kDa) | Homo sapiens |
| 64065 | PERP, TP53 apoptosis effector | Homo sapiens |
| 55437 | STE20-related kinase adaptor beta | Homo sapiens |
| 283454 | hypothetical protein LOC283454 | Homo sapiens |
| 55086 | chromosome X open reading frame 57 | Homo sapiens |
| 91942 | NADH dehydrogenase (ubiquinone) 1 alpha subcomplex, assembly factor 2 | Homo sapiens |
| 55257 | chromosome 20 open reading frame 20 | Homo sapiens |
| 1537 | cytochrome c-1 | Homo sapiens |
| 30061 | solute carrier family 40 (iron-regulated transporter), member 1 | Homo sapiens |
| 5532 | protein phosphatase 3 (formerly 2B), catalytic subunit, beta isoform | Homo sapiens |
| 63826 | serine racemase | Homo sapiens |
| 10651 | metaxin 2 | Homo sapiens |
| 26224 | F-box and leucine-rich repeat protein 3 | Homo sapiens |
| 84865 | coiled-coil domain containing 142 | Homo sapiens |
| 116064 | leucine rich repeat containing 58 | Homo sapiens |
| 79364 | ZXD family zinc finger C | Homo sapiens |
| 7114 | thymosin-like 2 (pseudogene); thymosin-like 1 (pseudogene); thymosin beta 4, X-linked | Homo sapiens |
| 9753 | zinc finger and SCAN domain containing 12 | Homo sapiens |

| | | |
|---|---|---|
| 55291 | SAPS domain family, member 3 | Homo sapiens |
| 164 | adaptor-related protein complex 1, gamma 1 subunit | Homo sapiens |
| 57414 | rhomboid domain containing 2 | Homo sapiens |
| 144404 | transmembrane protein 120B | Homo sapiens |
| 6738 | TROVE domain family, member 2 | Homo sapiens |
| 9790 | BMS1 homolog, ribosome assembly protein (yeast) pseudogene; BMS1 homolog, ribosome assembly protein (yeast) | Homo sapiens |
| 112487 | chromosome 14 open reading frame 126 | Homo sapiens |
| 55846 | integrin alpha FG-GAP repeat containing 2 | Homo sapiens |
| 23363 | obscurin-like 1 | Homo sapiens |
| 91433 | RCC1 domain containing 1 | Homo sapiens |
| 473 | arginine-glutamic acid dipeptide (RE) repeats | Homo sapiens |
| 79085 | solute carrier family 25 (mitochondrial carrier; phosphate carrier), member 23 | Homo sapiens |
| 64981 | mitochondrial ribosomal protein L34 | Homo sapiens |
| 57643 | zinc finger, SWIM-type containing 5 | Homo sapiens |
| 6818 | sulfotransferase family, cytosolic, 1A, phenol-preferring, member 4; sulfotransferase family, cytosolic, 1A, phenol-preferring, member 3 | Homo sapiens |
| 445329 | sulfotransferase family, cytosolic, 1A, phenol-preferring, member 4; sulfotransferase family, cytosolic, 1A, phenol-preferring, member 3 | Homo sapiens |
| 118812 | MORN repeat containing 4 | Homo sapiens |
| 10616 | RanBP-type and C3HC4-type zinc finger containing 1 | Homo sapiens |
| 26278 | spastic ataxia of Charlevoix-Saguenay (sacsin) | Homo sapiens |
| 5537 | protein phosphatase 6, catalytic subunit | Homo sapiens |
| 57396 | CDC-like kinase 4 | Homo sapiens |
| 51186 | WW domain binding protein 5 | Homo sapiens |
| 9241 | noggin | Homo sapiens |
| 57821 | chromosome 1 open reading frame 114 | Homo sapiens |
| 84230 | leucine rich repeat containing 8 family, member C | Homo sapiens |
| 3187 | heterogeneous nuclear ribonucleoprotein H1 (H) | Homo sapiens |
| 114799 | establishment of cohesion 1 homolog 1 (S. cerevisiae) | Homo sapiens |
| 51460 | Scm-like with four mbt domains 1 | Homo sapiens |
| 59336 | PR domain containing 13 | Homo sapiens |
| 84791 | chromosome 1 open reading frame 97 | Homo sapiens |
| 989 | septin 7 | Homo sapiens |
| 55833 | ubiquitin associated protein 2 | Homo sapiens |
| 8754 | ADAM metallopeptidase domain 9 (meltrin gamma) | Homo sapiens |
| 11153 | FIC domain containing | Homo sapiens |
| 55531 | ELMO/CED-12 domain containing 1 | Homo sapiens |
| 4649 | myosin IXA | Homo sapiens |
| 147727 | hypothetical LOC147727 | Homo sapiens |
| 253725 | family with sequence similarity 21, member D; family with sequence similarity 21, member C | Homo sapiens |
| 653450 | family with sequence similarity 21, member D; family with sequence similarity 21, member C | Homo sapiens |
| 10783 | NIMA (never in mitosis gene a)-related kinase 6 | Homo sapiens |
| 9910 | RAB GTPase activating protein 1-like | Homo sapiens |
| 23215 | BAT2 domain containing 1 | Homo sapiens |
| 5562 | protein kinase, AMP-activated, alpha 1 catalytic subunit | Homo sapiens |
| 25874 | brain protein 44 | Homo sapiens |
| 79016 | DET1 and DDB1 associated 1 | Homo sapiens |
| 643911 | hCG1815491 | Homo sapiens |
| 84895 | family with sequence similarity 73, member B | Homo sapiens |
| 5709 | proteasome (prosome, macropain) 26S subunit, non-ATPase, 3 | Homo sapiens |
| 475 | ATX1 antioxidant protein 1 homolog (yeast) | Homo sapiens |
| 2737 | GLI family zinc finger 3 | Homo sapiens |
| 4902 | neurturin | Homo sapiens |
| 1431 | citrate synthase | Homo sapiens |
| 7586 | zinc finger with KRAB and SCAN domains 1 | Homo sapiens |
| 1174 | adaptor-related protein complex 1, sigma 1 subunit | Homo sapiens |
| 7390 | uroporphyrinogen III synthase | Homo sapiens |
| 7621 | zinc finger protein 70 | Homo sapiens |
| 54977 | solute carrier family 25, member 38 | Homo sapiens |
| 10302 | small nuclear RNA activating complex, polypeptide 5, 19kDa | Homo sapiens |
| 64429 | zinc finger, DHHC-type containing 6 | Homo sapiens |
| 3156 | 3-hydroxy-3-methylglutaryl-Coenzyme A reductase | Homo sapiens |
| 2923 | protein disulfide isomerase family A, member 3 | Homo sapiens |
| 27034 | acyl-Coenzyme A dehydrogenase family, member 8 | Homo sapiens |
| 55959 | sulfatase 2 | Homo sapiens |
| 55239 | 2-oxoglutarate and iron-dependent oxygenase domain containing 1 | Homo sapiens |
| 64983 | mitochondrial ribosomal protein L32 | Homo sapiens |
| 85462 | FH2 domain containing 1 | Homo sapiens |
| 29102 | ribonuclease type III, nuclear | Homo sapiens |
| 10286 | breast carcinoma amplified sequence 2 | Homo sapiens |
| 51366 | similar to E3 ubiquitin protein ligase, HECT domain containing, 1; ubiquitin protein ligase E3 component n-recognin 5 | Homo sapiens |
| 7181 | nuclear receptor subfamily 2, group C, member 1 | Homo sapiens |
| 79850 | family with sequence similarity 57, member A | Homo sapiens |

| | | |
|---|---|---|
| 51474 | LIM domain and actin binding 1 | Homo sapiens |
| 8854 | aldehyde dehydrogenase 1 family, member A2 | Homo sapiens |
| 139231 | chromosome X open reading frame 39 | Homo sapiens |
| 56180 | motile sperm domain containing 1 | Homo sapiens |
| 55705 | importin 9 | Homo sapiens |
| 4828 | neuromedin B | Homo sapiens |
| 55917 | CTTNBP2 N-terminal like | Homo sapiens |
| 114926 | chromosome 8 open reading frame 40 | Homo sapiens |
| 49856 | WD repeat domain 8 | Homo sapiens |
| 11258 | dynactin 3 (p22) | Homo sapiens |
| 92399 | mitochondrial ribosome recycling factor | Homo sapiens |
| 1810 | down-regulator of transcription 1, TBP-binding (negative cofactor 2) | Homo sapiens |
| 55197 | regulation of nuclear pre-mRNA domain containing 1A | Homo sapiens |
| 572558 | hypothetical locus LOC572558 | Homo sapiens |
| 57109 | REX4, RNA exonuclease 4 homolog (S. cerevisiae) | Homo sapiens |
| 394 | Rho GTPase activating protein 5 | Homo sapiens |
| 6631 | small nuclear ribonucleoprotein polypeptide C | Homo sapiens |
| 51182 | heat shock 70kDa protein 14 | Homo sapiens |
| 79177 | zinc finger protein 576 | Homo sapiens |
| 27309 | zinc finger protein 330 | Homo sapiens |
| 84890 | 2-aminoethanethiol (cysteamine) dioxygenase | Homo sapiens |
| 10267 | receptor (G protein-coupled) activity modifying protein 1 | Homo sapiens |
| 10806 | serologically defined colon cancer antigen 8 | Homo sapiens |
| 400073 | chromosome 12 open reading frame 76 | Homo sapiens |
| 23405 | dicer 1, ribonuclease type III | Homo sapiens |
| 166379 | Bardet-Biedl syndrome 12 | Homo sapiens |
| 51083 | galanin prepropeptide | Homo sapiens |
| 3910 | laminin, alpha 4 | Homo sapiens |
| 23673 | syntaxin 12 | Homo sapiens |
| 55666 | nuclear protein localization 4 homolog (S. cerevisiae) | Homo sapiens |
| 6421 | splicing factor proline/glutamine-rich (polypyrimidine tract binding protein associated) | Homo sapiens |
| 55170 | protein arginine methyltransferase 6 | Homo sapiens |
| 2885 | growth factor receptor-bound protein 2 | Homo sapiens |
| 348162 | hypothetical protein 348162 | Homo sapiens |
| 7549 | zinc finger protein 2 | Homo sapiens |
| 10151 | heterogeneous nuclear ribonucleoprotein A3 pseudogene 1 | Homo sapiens |
| 58508 | myeloid/lymphoid or mixed-lineage leukemia 3 | Homo sapiens |
| 5881 | ras-related C3 botulinum toxin substrate 3 (rho family, small GTP binding protein Rac3) | Homo sapiens |
| 55274 | PHD finger protein 10 | Homo sapiens |
| 29803 | replication initiator 1 | Homo sapiens |
| 51499 | TP53 regulated inhibitor of apoptosis 1 | Homo sapiens |
| 9553 | mitochondrial ribosomal protein L33 | Homo sapiens |
| 207 | v-akt murine thymoma viral oncogene homolog 1 | Homo sapiens |
| 90693 | coiled-coil domain containing 126 | Homo sapiens |
| 79699 | zyg-11 homolog B (C. elegans) | Homo sapiens |
| 79184 | BRCA1/BRCA2-containing complex, subunit 3 | Homo sapiens |
| 401504 | hypothetical gene supported by AK091718 | Homo sapiens |
| 6191 | ribosomal protein S4X pseudogene 6; ribosomal protein S4X pseudogene 13; ribosomal protein S4, X-linked | Homo sapiens |
| 5579 | protein kinase C, beta | Homo sapiens |
| 57678 | glycerol-3-phosphate acyltransferase, mitochondrial | Homo sapiens |
| 56994 | choline phosphotransferase 1 | Homo sapiens |
| 84749 | ubiquitin specific peptidase 30 | Homo sapiens |
| 55635 | DEP domain containing 1 | Homo sapiens |
| 6988 | T-cell leukemia translocation altered gene | Homo sapiens |
| 54784 | alkB, alkylation repair homolog 4 (E. coli) | Homo sapiens |
| 55283 | mucolipin 3 | Homo sapiens |
| 57448 | baculoviral IAP repeat-containing 6 | Homo sapiens |
| 142 | poly (ADP-ribose) polymerase 1 | Homo sapiens |
| 51371 | proteasome maturation protein | Homo sapiens |
| 112597 | hypothetical LOC541471; non-protein coding RNA 152 | Homo sapiens |
| 541471 | hypothetical LOC541471; non-protein coding RNA 152 | Homo sapiens |
| 3981 | ligase IV, DNA, ATP-dependent | Homo sapiens |
| 90362 | family with sequence similarity 110, member B | Homo sapiens |
| 11154 | adaptor-related protein complex 4, sigma 1 subunit | Homo sapiens |
| 10100 | tetraspanin 2 | Homo sapiens |
| 199692 | zinc finger protein 627 | Homo sapiens |
| 23366 | KIAA0895 | Homo sapiens |
| 6183 | mitochondrial ribosomal protein S12 | Homo sapiens |
| 256435 | ST6 (alpha-N-acetyl-neuraminyl-2,3-beta-galactosyl-1,3)-N-acetylgalactosaminide alpha-2,6-sialyltransferase 3 | Homo sapiens |
| 55844 | protein phosphatase 2, regulatory subunit B, delta isoform | Homo sapiens |
| 7368 | UDP glycosyltransferase 8 | Homo sapiens |
| 57695 | ubiquitin specific peptidase 37 | Homo sapiens |
| 23071 | endoplasmic reticulum protein 44 | Homo sapiens |
| 3336 | heat shock 10kDa protein 1 (chaperonin 10) | Homo sapiens |
| 23301 | EH domain binding protein 1 | Homo sapiens |

| | | |
|---|---|---|
| 8473 | O-linked N-acetylglucosamine (GlcNAc) transferase (UDP-N-acetylglucosamine:polypeptide-N-acetylglucosaminyl transferase) | Homo sapiens |
| 1415 | crystallin, beta B2 | Homo sapiens |
| 63971 | kinesin family member 13A | Homo sapiens |
| 51430 | chromosome 1 open reading frame 9 | Homo sapiens |
| 23660 | zinc finger with KRAB and SCAN domains 5 | Homo sapiens |
| 65980 | bromodomain containing 9 | Homo sapiens |
| 11332 | acyl-CoA thioesterase 7 | Homo sapiens |
| 121355 | gametocyte specific factor 1 | Homo sapiens |
| 10155 | tripartite motif-containing 28 | Homo sapiens |
| 11108 | PR domain containing 4 | Homo sapiens |
| 8543 | LIM domain only 4 | Homo sapiens |
| 406991 | microRNA 21 | Homo sapiens |
| 374969 | coiled-coil domain containing 23 | Homo sapiens |
| 115939 | chromosome 16 open reading frame 42 | Homo sapiens |
| 1452 | casein kinase 1, alpha 1 | Homo sapiens |
| 158586 | zinc finger, X-linked, duplicated B | Homo sapiens |
| 84342 | peptide deformylase (mitochondrial); component of oligomeric golgi complex 8 | Homo sapiens |
| 64146 | peptide deformylase (mitochondrial); component of oligomeric golgi complex 8 | Homo sapiens |
| 50484 | ribonucleotide reductase M2 B (TP53 inducible) | Homo sapiens |
| 29979 | ubiquilin 1 | Homo sapiens |
| 1870 | E2F transcription factor 2 | Homo sapiens |
| 122525 | chromosome 14 open reading frame 28 | Homo sapiens |
| 84750 | fucosyltransferase 10 (alpha (1,3) fucosyltransferase) | Homo sapiens |
| 8125 | hepatopoietin PCn127; acidic (leucine-rich) nuclear phosphoprotein 32 family, member A | Homo sapiens |
| 84135 | UTP15, U3 small nucleolar ribonucleoprotein, homolog (S. cerevisiae) | Homo sapiens |
| 4714 | NADH dehydrogenase (ubiquinone) 1 beta subcomplex, 8, 19kDa | Homo sapiens |
| 54733 | solute carrier family 35, member F2 | Homo sapiens |
| 56257 | methylphosphate capping enzyme | Homo sapiens |
| 10212 | DEAD (Asp-Glu-Ala-Asp) box polypeptide 39 | Homo sapiens |
| 23049 | SMG1 homolog, phosphatidylinositol 3-kinase-related kinase (C. elegans) | Homo sapiens |
| 729570 | hypothetical LOC729570 | Homo sapiens |
| 5707 | proteasome (prosome, macropain) 26S subunit, non-ATPase, 1 | Homo sapiens |
| 55761 | tetratricopeptide repeat domain 17 | Homo sapiens |
| 84146 | zinc finger protein 644 | Homo sapiens |
| 728642 | similar to cell division cycle 2-like 1 (PITSLRE proteins); cell division cycle 2-like 1 (PITSLRE proteins); cell division cycle 2-like 2 (PITSLRE proteins) | Homo sapiens |
| 55334 | solute carrier family 39 (zinc transporter), member 9 | Homo sapiens |
| 439911 | hypothetical gene supported by NM_194304 | Homo sapiens |
| 29767 | tropomodulin 2 (neuronal) | Homo sapiens |
| 6647 | superoxide dismutase 1, soluble | Homo sapiens |
| 25913 | POT1 protection of telomeres 1 homolog (S. pombe) | Homo sapiens |
| 9140 | ATG12 autophagy related 12 homolog (S. cerevisiae) | Homo sapiens |
| 84823 | lamin B2 | Homo sapiens |
| 5873 | RAB27A, member RAS oncogene family | Homo sapiens |
| 29927 | Sec61 alpha 1 subunit (S. cerevisiae) | Homo sapiens |
| 54438 | glucose-fructose oxidoreductase domain containing 1 | Homo sapiens |
| 10409 | brain abundant, membrane attached signal protein 1 | Homo sapiens |
| 7936 | RD RNA binding protein | Homo sapiens |
| 5564 | protein kinase, AMP-activated, beta 1 non-catalytic subunit | Homo sapiens |
| 79966 | stearoyl-CoA desaturase 5 | Homo sapiens |
| 10199 | M-phase phosphoprotein 10 (U3 small nucleolar ribonucleoprotein) | Homo sapiens |
| 4751 | NIMA (never in mitosis gene a)-related kinase 2 | Homo sapiens |
| 267004 | piggyBac transposable element derived 3 | Homo sapiens |
| 3092 | huntingtin interacting protein 1 | Homo sapiens |
| 9360 | peptidylprolyl isomerase G (cyclophilin G) | Homo sapiens |
| 90231 | KIAA2013; hypothetical LOC728138 | Homo sapiens |
| 51626 | dynein, cytoplasmic 2, light intermediate chain 1 | Homo sapiens |
| 51133 | potassium channel tetramerisation domain containing 3 | Homo sapiens |
| 5591 | similar to protein kinase, DNA-activated, catalytic polypeptide; protein kinase, DNA-activated, catalytic polypeptide | Homo sapiens |
| 79577 | cell division cycle 73, Paf1/RNA polymerase II complex component, homolog (S. cerevisiae) | Homo sapiens |
| 7852 | chemokine (C-X-C motif) receptor 4 | Homo sapiens |
| 91369 | ankyrin repeat domain 40 | Homo sapiens |
| 23244 | PDS5, regulator of cohesion maintenance, homolog A (S. cerevisiae) | Homo sapiens |
| 57539 | WD repeat domain 35 | Homo sapiens |
| 399491 | GPS, PLAT and transmembrane domain-containing protein | Homo sapiens |
| 57669 | erythrocyte membrane protein band 4.1 like 5 | Homo sapiens |
| 440354 | PI-3-kinase-related kinase SMG-1 pseudogene | Homo sapiens |
| 595101 | PI-3-kinase-related kinase SMG-1 pseudogene | Homo sapiens |
| 389 | ras homolog gene family, member C | Homo sapiens |
| 55920 | regulator of chromosome condensation 2 | Homo sapiens |
| 79135 | apolipoprotein O | Homo sapiens |
| 54664 | transmembrane protein 106B | Homo sapiens |
| 51742 | AT rich interactive domain 4B (RBP1-like) | Homo sapiens |
| 79621 | ribonuclease H2, subunit B | Homo sapiens |

| | | |
|---|---|---|
| 123016 | tetratricopeptide repeat domain 8 | Homo sapiens |
| 55975 | kelch-like 7 (Drosophila) | Homo sapiens |
| 4092 | SMAD family member 7 | Homo sapiens |
| 79794 | chromosome 12 open reading frame 49 | Homo sapiens |
| 9397 | N-myristoyltransferase 2 | Homo sapiens |
| 23229 | Cdc42 guanine nucleotide exchange factor (GEF) 9; hypothetical protein LOC100134381 | Homo sapiens |
| 23001 | WD repeat and FYVE domain containing 3 | Homo sapiens |
| 5306 | phosphatidylinositol transfer protein, alpha | Homo sapiens |
| 8930 | methyl-CpG binding domain protein 4 | Homo sapiens |
| 25902 | methylenetetrahydrofolate dehydrogenase (NADP+ dependent) 1-like | Homo sapiens |
| 23647 | ADP-ribosylation factor interacting protein 2 | Homo sapiens |
| 54583 | egl nine homolog 1 (C. elegans) | Homo sapiens |
| 57690 | trinucleotide repeat containing 6C | Homo sapiens |
| 3326 | heat shock protein 90kDa alpha (cytosolic), class B member 1 | Homo sapiens |
| 8897 | myotubularin related protein 3 | Homo sapiens |
| 142891 | sterile alpha motif domain containing 8 | Homo sapiens |
| 133308 | Na+/H+ exchanger domain containing 2 | Homo sapiens |
| 84908 | family with sequence similarity 136, member A | Homo sapiens |
| 4298 | myeloid/lymphoid or mixed-lineage leukemia (trithorax homolog, Drosophila); translocated to, 1 | Homo sapiens |
| 57798 | GATA zinc finger domain containing 1 | Homo sapiens |
| 84240 | zinc finger, CCHC domain containing 9 | Homo sapiens |
| 79023 | nucleoporin 37kDa | Homo sapiens |
| 79133 | chromosome 20 open reading frame 7 | Homo sapiens |
| 255967 | PAN3 poly(A) specific ribonuclease subunit homolog (S. cerevisiae) | Homo sapiens |
| 4067 | v-yes-1 Yamaguchi sarcoma viral related oncogene homolog | Homo sapiens |
| 6185 | ribophorin II | Homo sapiens |
| 79081 | chromosome 11 open reading frame 48 | Homo sapiens |
| 55435 | chromosome 4 open reading frame 16 | Homo sapiens |
| 6389 | succinate dehydrogenase complex, subunit A, flavoprotein (Fp) | Homo sapiens |
| 51493 | chromosome 22 open reading frame 28 | Homo sapiens |
| 65117 | arginine/serine-rich coiled-coil 2 | Homo sapiens |
| 4335 | MAX binding protein | Homo sapiens |
| 9552 | sperm associated antigen 7 | Homo sapiens |
| 4711 | NADH dehydrogenase (ubiquinone) 1 beta subcomplex, 5, 16kDa | Homo sapiens |
| 30000 | transportin 2 | Homo sapiens |
| 10061 | ATP-binding cassette, sub-family F (GCN20), member 2 | Homo sapiens |
| 1946 | ephrin-A5 | Homo sapiens |
| 64772 | endo-beta-N-acetylglucosaminidase | Homo sapiens |
| 5718 | proteasome (prosome, macropain) 26S subunit, non-ATPase, 12 | Homo sapiens |
| 178 | amylo-1, 6-glucosidase, 4-alpha-glucanotransferase | Homo sapiens |
| 132320 | sodium channel and clathrin linker 1 | Homo sapiens |
| 26127 | FGFR1 oncogene partner 2 | Homo sapiens |
| 6845 | vesicle-associated membrane protein 7 | Homo sapiens |
| 55148 | ubiquitin protein ligase E3 component n-recognin 7 (putative) | Homo sapiens |
| 10535 | ribonuclease H2, subunit A | Homo sapiens |
| 518 | ATP synthase, H+ transporting, mitochondrial F0 complex, subunit C3 (subunit 9) | Homo sapiens |
| 23029 | RNA binding motif protein 34 | Homo sapiens |
| 55002 | transmembrane and coiled-coil domains 3 | Homo sapiens |
| 5468 | peroxisome proliferator-activated receptor gamma | Homo sapiens |
| 375260 | WAS protein family homolog 3 pseudogene; WAS protein family homolog 2 pseudogene; WAS protein family homolog 1; WAS protein family homolog 5 pseudogene | Homo sapiens |
| 653635 | WAS protein family homolog 3 pseudogene; WAS protein family homolog 2 pseudogene; WAS protein family homolog 1; WAS protein family homolog 5 pseudogene | Homo sapiens |
| 374666 | WAS protein family homolog 3 pseudogene; WAS protein family homolog 2 pseudogene; WAS protein family homolog 1; WAS protein family homolog 5 pseudogene | Homo sapiens |
| 10390 | choline/ethanolamine phosphotransferase 1 | Homo sapiens |
| 8943 | adaptor-related protein complex 3, delta 1 subunit | Homo sapiens |
| 55893 | zinc finger protein 395 | Homo sapiens |
| 4947 | ornithine decarboxylase antizyme 2 | Homo sapiens |
| 9791 | phosphatidylserine synthase 1 | Homo sapiens |
| 23137 | structural maintenance of chromosomes 5 | Homo sapiens |
| 25936 | NSL1, MIND kinetochore complex component, homolog (S. cerevisiae) | Homo sapiens |
| 8481 | oral-facial-digital syndrome 1 | Homo sapiens |
| 64949 | mitochondrial ribosomal protein S26 | Homo sapiens |
| 84267 | chromosome 9 open reading frame 64 | Homo sapiens |
| 7247 | translin | Homo sapiens |
| 26092 | torsin A interacting protein 1 | Homo sapiens |
| 144108 | SPT2, Suppressor of Ty, domain containing 1 (S. cerevisiae) | Homo sapiens |
| 51504 | tRNA methyltransferase 11-2 homolog (S. cerevisiae); similar to CG12975 | Homo sapiens |
| 80312 | tet oncogene 1 | Homo sapiens |
| 55684 | chromosome 9 open reading frame 86 | Homo sapiens |
| 80135 | brix domain containing 5 | Homo sapiens |
| 51678 | membrane protein, palmitoylated 6 (MAGUK p55 subfamily member 6) | Homo sapiens |
| 140775 | Smith-Magenis syndrome chromosome region, candidate 8 | Homo sapiens |
| 57122 | nucleoporin 107kDa | Homo sapiens |
| 4676 | nucleosome assembly protein 1-like 4 | Homo sapiens |

| | | |
|---|---|---|
| 898 | cyclin E1 | Homo sapiens |
| 64397 | zinc finger protein 106 homolog (mouse) | Homo sapiens |
| 55109 | angiogenic factor with G patch and FHA domains 1 | Homo sapiens |
| 27031 | nephronophthisis 3 (adolescent); acyl-Coenzyme A dehydrogenase family, member 11 | Homo sapiens |
| 84129 | nephronophthisis 3 (adolescent); acyl-Coenzyme A dehydrogenase family, member 11 | Homo sapiens |
| 64924 | solute carrier family 30 (zinc transporter), member 5 | Homo sapiens |
| 23645 | protein phosphatase 1, regulatory (inhibitor) subunit 15A | Homo sapiens |
| 445571 | COBW domain containing 5; similar to COBW domain containing 1; COBW domain containing 7; COBW domain containing 3 | Homo sapiens |
| 728013 | COBW domain containing 5; similar to COBW domain containing 1; COBW domain containing 7; COBW domain containing 3 | Homo sapiens |
| 220869 | COBW domain containing 5; similar to COBW domain containing 1; COBW domain containing 7; COBW domain containing 3 | Homo sapiens |
| 57509 | mitochondrial tumor suppressor 1 | Homo sapiens |
| 27291 | chromosome 10 open reading frame 28 | Homo sapiens |
| 8704 | UDP-Gal:betaGlcNAc beta 1,4- galactosyltransferase, polypeptide 2 | Homo sapiens |
| 51512 | G-2 and S-phase expressed 1 | Homo sapiens |
| 28966 | sorting nexin 24 | Homo sapiens |
| 6944 | vacuolar protein sorting 72 homolog (S. cerevisiae) | Homo sapiens |
| 57700 | family with sequence similarity 160, member B1 | Homo sapiens |
| 9761 | malectin | Homo sapiens |
| 55317 | chromosome 20 open reading frame 29 | Homo sapiens |
| 84952 | cingulin-like 1 | Homo sapiens |
| 54998 | aurora kinase A interacting protein 1 | Homo sapiens |
| 205564 | SUMO1/sentrin specific peptidase 5 | Homo sapiens |
| 24149 | zinc finger protein 318 | Homo sapiens |
| 348093 | RNA binding protein with multiple splicing 2 | Homo sapiens |
| 85301 | collagen, type XXVII, alpha 1 | Homo sapiens |
| 2936 | glutathione reductase | Homo sapiens |
| 51272 | blocked early in transport 1 homolog (S. cerevisiae)-like | Homo sapiens |
| 6715 | steroid-5-alpha-reductase, alpha polypeptide 1 (3-oxo-5 alpha-steroid delta 4-dehydrogenase alpha 1) | Homo sapiens |
| 8666 | eukaryotic translation initiation factor 3, subunit G | Homo sapiens |
| 5987 | tripartite motif-containing 27 | Homo sapiens |
| 8846 | alkB, alkylation repair homolog 1 (E. coli) | Homo sapiens |
| 113246 | chromosome 12 open reading frame 57 | Homo sapiens |
| 9792 | SERTA domain containing 2 | Homo sapiens |
| 389072 | pleckstrin homology domain containing, family M, member 3 | Homo sapiens |
| 7328 | ubiquitin-conjugating enzyme E2H (UBC8 homolog, yeast) | Homo sapiens |
| 5082 | phosducin-like | Homo sapiens |
| 81620 | chromatin licensing and DNA replication factor 1 | Homo sapiens |
| 26146 | TNF receptor-associated factor 3 interacting protein 1 | Homo sapiens |
| 9158 | fibroblast growth factor (acidic) intracellular binding protein | Homo sapiens |
| 670 | biphenyl hydrolase-like (serine hydrolase) | Homo sapiens |
| 80789 | integrator complex subunit 5 | Homo sapiens |
| 22877 | MLX interacting protein | Homo sapiens |
| 131870 | nudix (nucleoside diphosphate linked moiety X)-type motif 16 | Homo sapiens |
| 23195 | MDN1, midasin homolog (yeast) | Homo sapiens |
| 134145 | family with sequence similarity 173, member B | Homo sapiens |
| 5702 | proteasome (prosome, macropain) 26S subunit, ATPase, 3 | Homo sapiens |
| 26524 | LATS, large tumor suppressor, homolog 2 (Drosophila) | Homo sapiens |
| 79056 | proline rich Gla (G-carboxyglutamic acid) 4 (transmembrane) | Homo sapiens |
| 51018 | ribosomal RNA processing 15 homolog (S. cerevisiae) | Homo sapiens |
| 55032 | solute carrier family 35, member A5 | Homo sapiens |
| 2959 | general transcription factor IIB | Homo sapiens |
| 5939 | RNA binding motif, single stranded interacting protein 2; RNA binding motif, single stranded interacting protein 2 pseudogene | Homo sapiens |
| 339123 | jumonji domain containing 8 | Homo sapiens |
| 56895 | 1-acylglycerol-3-phosphate O-acyltransferase 4 (lysophosphatidic acid acyltransferase, delta) | Homo sapiens |
| 7014 | telomeric repeat binding factor 2 | Homo sapiens |
| 4853 | Notch homolog 2 (Drosophila) | Homo sapiens |
| 64343 | 5-azacytidine induced 2 | Homo sapiens |
| 23566 | lysophosphatidic acid receptor 3 | Homo sapiens |
| 22954 | tripartite motif-containing 32 | Homo sapiens |
| 226 | aldolase A, fructose-bisphosphate | Homo sapiens |
| 4708 | NADH dehydrogenase (ubiquinone) 1 beta subcomplex, 2, 8kDa | Homo sapiens |
| 7410 | vav 2 guanine nucleotide exchange factor | Homo sapiens |
| 9584 | similar to RNA binding motif protein 39; RNA binding motif protein 39 | Homo sapiens |
| 29080 | coiled-coil domain containing 59 | Homo sapiens |
| 84458 | ligand dependent nuclear receptor corepressor | Homo sapiens |
| 100128731 | dolichyl-diphosphooligosaccharide--protein glycosyltransferase subunit 4 | Homo sapiens |
| 728888 | similar to acyl-CoA synthetase medium-chain family member 2 | Homo sapiens |
| 84285 | eukaryotic translation initiation factor 1A domain containing | Homo sapiens |
| 64418 | transmembrane protein 168 | Homo sapiens |
| 79038 | zinc finger, FYVE domain containing 21 | Homo sapiens |
| 55794 | DEAD (Asp-Glu-Ala-Asp) box polypeptide 28 | Homo sapiens |

| | | |
|---|---|---|
| 91252 | solute carrier family 39 (zinc transporter), member 13 | Homo sapiens |
| 7706 | tripartite motif-containing 25 | Homo sapiens |
| 389541 | chromosome 7 open reading frame 59 | Homo sapiens |
| 54926 | ubiquitin-conjugating enzyme E2R 2 | Homo sapiens |
| 116496 | family with sequence similarity 129, member A | Homo sapiens |
| 4686 | nuclear cap binding protein subunit 1, 80kDa | Homo sapiens |
| 23450 | splicing factor 3b, subunit 3, 130kDa | Homo sapiens |
| 8493 | protein phosphatase 1D magnesium-dependent, delta isoform | Homo sapiens |
| 10634 | growth arrest-specific 2 like 1 | Homo sapiens |
| 7874 | ubiquitin specific peptidase 7 (herpes virus-associated) | Homo sapiens |
| 84916 | cirrhosis, autosomal recessive 1A (cirhin) | Homo sapiens |
| 1978 | eukaryotic translation initiation factor 4E binding protein 1 | Homo sapiens |
| 85379 | KIAA1671 protein | Homo sapiens |
| 730094 | chromosome 16 open reading frame 52 | Homo sapiens |
| 7764 | zinc finger protein 217 | Homo sapiens |
| 689 | basic transcription factor 3; basic transcription factor 3, like 1 pseudogene | Homo sapiens |
| 80194 | transmembrane protein 134 | Homo sapiens |
| 6942 | transcription factor 20 (AR1) | Homo sapiens |
| 5533 | protein phosphatase 3 (formerly 2B), catalytic subunit, gamma isoform | Homo sapiens |
| 6049 | ring finger protein (C3H2C3 type) 6 | Homo sapiens |
| 55832 | cullin-associated and neddylation-dissociated 1 | Homo sapiens |
| 7015 | telomerase reverse transcriptase | Homo sapiens |
| 220594 | TL132 protein | Homo sapiens |
| 79751 | solute carrier family 25 (mitochondrial carrier: glutamate), member 22 | Homo sapiens |
| 54476 | ring finger protein 216 | Homo sapiens |
| 55818 | lysine (K)-specific demethylase 3A | Homo sapiens |
| 7905 | receptor accessory protein 5 | Homo sapiens |
| 11319 | ecdysoneless homolog (Drosophila) | Homo sapiens |
| 6169 | ribosomal protein L38 | Homo sapiens |
| 22841 | RAB11 family interacting protein 2 (class I) | Homo sapiens |
| 378938 | metastasis associated lung adenocarcinoma transcript 1 (non-protein coding) | Homo sapiens |
| 81488 | glutamate receptor, ionotropic, N-methyl D-aspartate-like 1B; glutamate receptor, ionotropic, N-methyl D-aspartate-like 1A; GRINL1A complex locus | Homo sapiens |
| 145781 | glutamate receptor, ionotropic, N-methyl D-aspartate-like 1B; glutamate receptor, ionotropic, N-methyl D-aspartate-like 1A; GRINL1A complex locus | Homo sapiens |
| 10570 | dihydropyrimidinase-like 4 | Homo sapiens |
| 11261 | calcium binding protein P22 | Homo sapiens |
| 60673 | chromosome 12 open reading frame 44 | Homo sapiens |
| 2962 | general transcription factor IIF, polypeptide 1, 74kDa | Homo sapiens |
| 2184 | fumarylacetoacetate hydrolase (fumarylacetoacetase) | Homo sapiens |
| 91461 | protein kinase-like protein SgK493 | Homo sapiens |
| 29081 | methyltransferase like 5 | Homo sapiens |
| 51042 | zinc finger protein 593 | Homo sapiens |
| 4174 | minichromosome maintenance complex component 5 | Homo sapiens |
| 6048 | ring finger protein 5; ring finger protein 5 pseudogene 1 | Homo sapiens |
| 80212 | coiled-coil domain containing 92 | Homo sapiens |
| 79665 | similar to DEAH (Asp-Glu-Ala-His) box polypeptide 40; DEAH (Asp-Glu-Ala-His) box polypeptide 40 | Homo sapiens |
| 10741 | retinoblastoma binding protein 9 | Homo sapiens |
| 79042 | tRNA splicing endonuclease 34 homolog (S. cerevisiae) | Homo sapiens |
| 78996 | chromosome 7 open reading frame 49 | Homo sapiens |
| 10597 | trafficking protein particle complex 2; trafficking protein particle complex 2 pseudogene 1 | Homo sapiens |
| 6399 | trafficking protein particle complex 2; trafficking protein particle complex 2 pseudogene 1 | Homo sapiens |
| 29957 | solute carrier family 25 (mitochondrial carrier; phosphate carrier), member 24 | Homo sapiens |
| 55608 | ankyrin repeat domain 10 | Homo sapiens |
| 79675 | FAST kinase domains 1 | Homo sapiens |
| 79191 | iroquois homeobox 3 | Homo sapiens |
| 3237 | homeobox D11 | Homo sapiens |
| 1201 | ceroid-lipofuscinosis, neuronal 3 | Homo sapiens |
| 22944 | KIN, antigenic determinant of recA protein homolog (mouse) | Homo sapiens |
| 29090 | chromosome 18 open reading frame 55 | Homo sapiens |
| 9355 | LIM homeobox 2 | Homo sapiens |
| 51593 | serrate RNA effector molecule homolog (Arabidopsis) | Homo sapiens |
| 5480 | peptidylprolyl isomerase C (cyclophilin C) | Homo sapiens |
| 51199 | ninein (GSK3B interacting protein) | Homo sapiens |
| 375346 | transmembrane protein 110; musculoskeletal, embryonic nuclear protein 1 | Homo sapiens |
| 9475 | Rho-associated, coiled-coil containing protein kinase 2 | Homo sapiens |
| 3028 | hydroxysteroid (17-beta) dehydrogenase 10 | Homo sapiens |
| 5516 | protein phosphatase 2 (formerly 2A), catalytic subunit, beta isoform | Homo sapiens |
| 81037 | CLPTM1-like | Homo sapiens |
| 3054 | host cell factor C1 (VP16-accessory protein) | Homo sapiens |
| 572 | BCL2-associated agonist of cell death | Homo sapiens |
| 130507 | ubiquitin protein ligase E3 component n-recognin 3 (putative) | Homo sapiens |
| 79817 | MOB1, Mps One Binder kinase activator-like 2B (yeast) | Homo sapiens |
| 25807 | rhomboid domain containing 3 | Homo sapiens |

| | | |
|---|---|---|
| 27 | v-abl Abelson murine leukemia viral oncogene homolog 2 (arg, Abelson-related gene) | Homo sapiens |
| 5292 | pim-1 oncogene | Homo sapiens |
| 3920 | lysosomal-associated membrane protein 2 | Homo sapiens |
| 3281 | heat shock factor binding protein 1 | Homo sapiens |
| 11191 | phosphatase and tensin homolog; phosphatase and tensin homolog pseudogene 1 | Homo sapiens |
| 5728 | phosphatase and tensin homolog; phosphatase and tensin homolog pseudogene 1 | Homo sapiens |
| 4752 | NIMA (never in mitosis gene a)-related kinase 3 | Homo sapiens |
| 9801 | mitochondrial ribosomal protein L19 | Homo sapiens |
| 8476 | CDC42 binding protein kinase alpha (DMPK-like) | Homo sapiens |
| 5700 | proteasome (prosome, macropain) 26S subunit, ATPase, 1; similar to protease (prosome, macropain) 26S subunit, ATPase 1 | Homo sapiens |
| 4718 | NADH dehydrogenase (ubiquinone) 1, subcomplex unknown, 2, 14.5kDa | Homo sapiens |
| 728825 | similar to SMT3 suppressor of mif two 3 homolog 2 pseudogene | Homo sapiens |
| 25914 | rotatin | Homo sapiens |
| 6726 | signal recognition particle 9-like 1; signal recognition particle 9kDa | Homo sapiens |
| 219743 | trypsin domain containing 1 | Homo sapiens |
| 64121 | Ras-related GTP binding C | Homo sapiens |
| 100101267 | POM121 membrane glycoprotein C; POM121 membrane glycoprotein B (pseudogene) | Homo sapiens |
| 503639 | double homeobox A pseudogene 10 | Homo sapiens |
| 2189 | Fanconi anemia, complementation group G | Homo sapiens |
| 26036 | zinc finger protein 451 | Homo sapiens |
| 54465 | Ewing tumor-associated antigen 1 | Homo sapiens |
| 23370 | Rho/Rac guanine nucleotide exchange factor (GEF) 18 | Homo sapiens |
| 10245 | translocase of inner mitochondrial membrane 17 homolog B (yeast) | Homo sapiens |
| 26119 | low density lipoprotein receptor adaptor protein 1 | Homo sapiens |
| 51560 | RAB6B, member RAS oncogene family | Homo sapiens |
| 6304 | SATB homeobox 1 | Homo sapiens |
| 5714 | proteasome (prosome, macropain) 26S subunit, non-ATPase, 8 | Homo sapiens |
| 55813 | UTP6, small subunit (SSU) processome component, homolog (yeast) | Homo sapiens |
| 26355 | family with sequence similarity 162, member A | Homo sapiens |
| 221002 | RasGEF domain family, member 1A | Homo sapiens |
| 53615 | methyl-CpG binding domain protein 3 | Homo sapiens |
| 400961 | poly(A) binding protein interacting protein 2B | Homo sapiens |
| 79884 | microtubule-associated protein 9 | Homo sapiens |
| 10466 | component of oligomeric golgi complex 5 | Homo sapiens |
| 51234 | transmembrane protein 85 | Homo sapiens |
| 83903 | germ cell associated 2 (haspin) | Homo sapiens |
| 7443 | vaccinia related kinase 1 | Homo sapiens |
| 51614 | ERGIC and golgi 3 | Homo sapiens |
| 4695 | NADH dehydrogenase (ubiquinone) 1 alpha subcomplex, 2, 8kDa | Homo sapiens |
| 80154 | hypothetical LOC80154 | Homo sapiens |
| 145553 | magnesium-dependent phosphatase 1 | Homo sapiens |
| 90809 | transmembrane protein 55B | Homo sapiens |
| 5889 | RAD51 homolog C (S. cerevisiae) | Homo sapiens |
| 8409 | ubiquitously-expressed transcript | Homo sapiens |
| 55603 | family with sequence similarity 46, member A | Homo sapiens |
| 51659 | GINS complex subunit 2 (Psf2 homolog) | Homo sapiens |
| 79989 | tetratricopeptide repeat domain 26 | Homo sapiens |
| 339500 | zinc finger protein 678 | Homo sapiens |
| 1643 | damage-specific DNA binding protein 2, 48kDa | Homo sapiens |
| 84336 | transmembrane protein 101 | Homo sapiens |
| 114805 | UDP-N-acetyl-alpha-D-galactosamine:polypeptide N-acetylgalactosaminyltransferase 13 (GalNAc-T13); UDP-N-acetyl-alpha-D-galactosamine:polypeptide N-acetylgalactosaminyltransferase 1 (GalNAc-T1) | Homo sapiens |
| 2589 | UDP-N-acetyl-alpha-D-galactosamine:polypeptide N-acetylgalactosaminyltransferase 13 (GalNAc-T13); UDP-N-acetyl-alpha-D-galactosamine:polypeptide N-acetylgalactosaminyltransferase 1 (GalNAc-T1) | Homo sapiens |
| 9212 | aurora kinase B | Homo sapiens |
| 9187 | solute carrier family 24 (sodium/potassium/calcium exchanger), member 1 | Homo sapiens |
| 5356 | pleiotropic regulator 1 (PRL1 homolog, Arabidopsis) | Homo sapiens |
| 100288939 | similar to hCG1987955 | Homo sapiens |
| 79894 | zinc finger protein 672; hypothetical LOC100130262 | Homo sapiens |
| 51260 | chromosome X open reading frame 26 | Homo sapiens |
| 548596 | creatine kinase, mitochondrial 1A; creatine kinase, mitochondrial 1B | Homo sapiens |
| 1159 | creatine kinase, mitochondrial 1A; creatine kinase, mitochondrial 1B | Homo sapiens |
| 4691 | nucleolin | Homo sapiens |
| 50937 | Cdon homolog (mouse) | Homo sapiens |
| 91316 | glucuronidase, beta/ immunoglobulin lambda-like polypeptide 1 pseudogene | Homo sapiens |
| 6299 | sal-like 1 (Drosophila) | Homo sapiens |
| 26636 | olfactory receptor, family 7, subfamily E, member 37 pseudogene | Homo sapiens |
| 7001 | peroxiredoxin 2 | Homo sapiens |
| 3652 | intracisternal A particle-promoted polypeptide | Homo sapiens |
| 6919 | transcription elongation factor A (SII), 2 | Homo sapiens |
| 129831 | RNA binding motif protein 45 | Homo sapiens |
| 56851 | chromosome 15 open reading frame 24 | Homo sapiens |
| 23184 | mesoderm development candidate 2 | Homo sapiens |
| 65988 | zinc finger protein 747 | Homo sapiens |

| | | |
|---|---|---|
| 29028 | ATPase family, AAA domain containing 2 | Homo sapiens |
| 637 | BH3 interacting domain death agonist | Homo sapiens |
| 100287482 | similar to hCG2038584 | Homo sapiens |
| 23204 | ADP-ribosylation factor-like 6 interacting protein 1 | Homo sapiens |
| 26986 | poly(A) binding protein, cytoplasmic pseudogene 5; poly(A) binding protein, cytoplasmic 1 | Homo sapiens |
| 11072 | dual specificity phosphatase 14 | Homo sapiens |
| 144717 | family with sequence similarity 109, member A | Homo sapiens |
| 51661 | FK506 binding protein 7 | Homo sapiens |
| 23167 | EFR3 homolog A (S. cerevisiae) | Homo sapiens |
| 353274 | zinc finger protein 445 | Homo sapiens |
| 55914 | erbb2 interacting protein | Homo sapiens |
| 4121 | mannosidase, alpha, class 1A, member 1 | Homo sapiens |
| 384 | arginase, type II | Homo sapiens |
| 23608 | makorin ring finger protein pseudogene 6; makorin ring finger protein 1 | Homo sapiens |
| 6760 | synovial sarcoma translocation, chromosome 18 | Homo sapiens |
| 115416 | chromosome 7 open reading frame 30 | Homo sapiens |
| 5515 | protein phosphatase 2 (formerly 2A), catalytic subunit, alpha isoform | Homo sapiens |
| 128 | alcohol dehydrogenase 5 (class III), chi polypeptide, pseudogene 4; alcohol dehydrogenase 5 (class III), chi polypeptide | Homo sapiens |
| 65977 | pleckstrin homology domain containing, family A (phosphoinositide binding specific) member 3 | Homo sapiens |
| 221687 | ring finger protein 182 | Homo sapiens |
| 8655 | dynein, light chain, LC8-type 1 | Homo sapiens |
| 7205 | thyroid hormone receptor interactor 6 | Homo sapiens |
| 818 | calcium/calmodulin-dependent protein kinase II gamma | Homo sapiens |
| 23567 | zinc finger protein 346 | Homo sapiens |
| 5685 | proteasome (prosome, macropain) subunit, alpha type, 4 | Homo sapiens |
| 63915 | thioredoxin domain containing 5 (endoplasmic reticulum); muted homolog (mouse) | Homo sapiens |
| 81567 | thioredoxin domain containing 5 (endoplasmic reticulum); muted homolog (mouse) | Homo sapiens |
| 283489 | zinc finger protein 828 | Homo sapiens |
| 25771 | TBC1 domain family, member 22A | Homo sapiens |
| 23464 | glycine C-acetyltransferase (2-amino-3-ketobutyrate coenzyme A ligase) | Homo sapiens |
| 80746 | tRNA splicing endonuclease 2 homolog (S. cerevisiae) | Homo sapiens |
| 55571 | chromosome 2 open reading frame 29 | Homo sapiens |
| 3035 | histidyl-tRNA synthetase | Homo sapiens |
| 51194 | importin 11 | Homo sapiens |
| 25912 | chromosome 1 open reading frame 43 | Homo sapiens |
| 197320 | zinc finger protein 778 | Homo sapiens |
| 7381 | similar to ubiquinol-cytochrome c reductase binding protein; ubiquinol-cytochrome c reductase binding protein pseudogene; ubiquinol-cytochrome c reductase binding protein | Homo sapiens |
| 9377 | cytochrome c oxidase subunit Va | Homo sapiens |
| 81552 | similar to EGFR-coamplified and overexpressed protein; EGFR-coamplified and overexpressed protein | Homo sapiens |
| 7982 | suppression of tumorigenicity 7 | Homo sapiens |
| 84939 | melanoma associated antigen (mutated) 1 | Homo sapiens |
| 1902 | lysophosphatidic acid receptor 1 | Homo sapiens |
| 552889 | hypothetical protein LOC552889 | Homo sapiens |
| 54921 | CTF8, chromosome transmission fidelity factor 8 homolog (S. cerevisiae) | Homo sapiens |
| 5965 | RecQ protein-like (DNA helicase Q1-like) | Homo sapiens |
| 139818 | dedicator of cytokinesis 11 | Homo sapiens |
| 1176 | adaptor-related protein complex 3, sigma 1 subunit | Homo sapiens |
| 54882 | ankyrin repeat and KH domain containing 1; ANKHD1-EIF4EBP3 readthrough transcript; eukaryotic translation initiation factor 4E binding protein 3 | Homo sapiens |
| 404734 | ankyrin repeat and KH domain containing 1; ANKHD1-EIF4EBP3 readthrough transcript; eukaryotic translation initiation factor 4E binding protein 3 | Homo sapiens |
| 84376 | hook homolog 3 (Drosophila) | Homo sapiens |
| 51529 | anaphase promoting complex subunit 11 | Homo sapiens |
| 4609 | v-myc myelocytomatosis viral oncogene homolog (avian) | Homo sapiens |
| 31 | acetyl-Coenzyme A carboxylase alpha | Homo sapiens |
| 55176 | Sec61 alpha 2 subunit (S. cerevisiae) | Homo sapiens |
| 115207 | potassium channel tetramerisation domain containing 12 | Homo sapiens |
| 116987 | ArfGAP with GTPase domain, ankyrin repeat and PH domain 1 | Homo sapiens |
| 10221 | tribbles homolog 1 (Drosophila) | Homo sapiens |
| 51015 | isochorismatase domain containing 1 | Homo sapiens |
| 51690 | LSM7 homolog, U6 small nuclear RNA associated (S. cerevisiae) | Homo sapiens |
| 124637 | cytochrome b5 domain containing 1 | Homo sapiens |
| 6392 | similar to succinate dehydrogenase complex, subunit D, integral membrane protein; succinate dehydrogenase complex, subunit D, integral membrane protein | Homo sapiens |
| 92106 | oxidoreductase NAD-binding domain containing 1 | Homo sapiens |
| 79068 | fat mass and obesity associated | Homo sapiens |
| 1612 | death-associated protein kinase 1 | Homo sapiens |
| 9830 | tripartite motif-containing 14 | Homo sapiens |
| 23197 | Fas associated factor family member 2 | Homo sapiens |
| 4512 | Cytochrome c oxidase subunit 1 | Homo sapiens |
| 80232 | WD repeat domain 26 | Homo sapiens |
| 64081 | phenazine biosynthesis-like protein domain containing | Homo sapiens |

| | | |
|---|---|---|
| 23636 | nucleoporin 62kDa | Homo sapiens |
| 6789 | serine/threonine kinase 4 | Homo sapiens |
| 154807 | vitamin K epoxide reductase complex, subunit 1-like 1 | Homo sapiens |
| 1892 | enoyl Coenzyme A hydratase, short chain, 1, mitochondrial | Homo sapiens |
| 51096 | UTP18, small subunit (SSU) processome component, homolog (yeast) | Homo sapiens |
| 9337 | CCR4-NOT transcription complex, subunit 8 | Homo sapiens |
| 57706 | DENN/MADD domain containing 1A | Homo sapiens |
| 2014 | epithelial membrane protein 3 | Homo sapiens |
| 56655 | polymerase (DNA-directed), epsilon 4 (p12 subunit) | Homo sapiens |
| 79959 | centrosomal protein 76kDa | Homo sapiens |
| 9807 | inositol hexakisphosphate kinase 1 | Homo sapiens |
| 1315 | coatomer protein complex, subunit beta 1 | Homo sapiens |
| 140739 | ubiquitin-conjugating enzyme E2F (putative) | Homo sapiens |
| 54468 | missing oocyte, meiosis regulator, homolog (Drosophila) | Homo sapiens |
| 10577 | Niemann-Pick disease, type C2 | Homo sapiens |
| 55153 | SDA1 domain containing 1 | Homo sapiens |
| 905 | cyclin T2 | Homo sapiens |
| 2767 | guanine nucleotide binding protein (G protein), alpha 11 (Gq class) | Homo sapiens |
| 84295 | PHD finger protein 6 | Homo sapiens |
| 159090 | family with sequence similarity 122B | Homo sapiens |
| 10736 | SIX homeobox 2 | Homo sapiens |
| 51611 | DPH5 homolog (S. cerevisiae) | Homo sapiens |
| 58526 | MID1 interacting protein 1 (gastrulation specific G12 homolog (zebrafish)) | Homo sapiens |
| 5605 | mitogen-activated protein kinase kinase 2 pseudogene; mitogen-activated protein kinase kinase 2 | Homo sapiens |
| 2961 | general transcription factor IIE, polypeptide 2, beta 34kDa | Homo sapiens |
| 6004 | regulator of G-protein signaling 16 | Homo sapiens |
| 55696 | RNA binding motif protein 22 | Homo sapiens |
| 4277 | MHC class I polypeptide-related sequence B | Homo sapiens |
| 100134445 | hypothetical LOC100134445 | Homo sapiens |
| 7718 | zinc finger protein 165 | Homo sapiens |
| 64753 | coiled-coil domain containing 136 | Homo sapiens |
| 131544 | beta-gamma crystallin domain containing 3 | Homo sapiens |
| 400236 | hypothetical LOC400236 | Homo sapiens |
| 3613 | inositol(myo)-1(or 4)-monophosphatase 2 | Homo sapiens |
| 402483 | hypothetical LOC402483; similar to capicua-like protein/double homeodomain 4 fusion protein | Homo sapiens |
| 100129502 | hypothetical protein LOC100129502 | Homo sapiens |
| 196528 | AT rich interactive domain 2 (ARID, RFX-like) | Homo sapiens |
| 57120 | golgi associated PDZ and coiled-coil motif containing | Homo sapiens |
| 2274 | four and a half LIM domains 2 | Homo sapiens |
| 25821 | mitochondrial translation optimization 1 homolog (S. cerevisiae) | Homo sapiens |
| 3032 | hydroxyacyl-Coenzyme A dehydrogenase/3-ketoacyl-Coenzyme A thiolase/enoyl-Coenzyme A hydratase (trifunctional protein), beta subunit | Homo sapiens |
| 84310 | chromosome 7 open reading frame 50 | Homo sapiens |
| 2072 | excision repair cross-complementing rodent repair deficiency, complementation group 4 | Homo sapiens |
| 5205 | ATPase, class I, type 8B, member 1 | Homo sapiens |
| 123879 | DCN1, defective in cullin neddylation 1, domain containing 3 (S. cerevisiae) | Homo sapiens |
| 10007 | glucosamine-6-phosphate deaminase 1 | Homo sapiens |
| 10602 | CDC42 effector protein (Rho GTPase binding) 3 | Homo sapiens |
| 221079 | ADP-ribosylation factor-like 5B | Homo sapiens |
| 811 | calreticulin | Homo sapiens |
| 1054 | CCAAT/enhancer binding protein (C/EBP), gamma | Homo sapiens |
| 92305 | transmembrane protein 129 | Homo sapiens |
| 7257 | translin-associated factor X | Homo sapiens |
| 1028 | cyclin-dependent kinase inhibitor 1C (p57, Kip2) | Homo sapiens |
| 79075 | defective in sister chromatid cohesion 1 homolog (S. cerevisiae) | Homo sapiens |
| 283659 | protogenin homolog (Gallus gallus) | Homo sapiens |
| 29115 | SAP30 binding protein | Homo sapiens |
| 3875 | keratin 18; keratin 18 pseudogene 26; keratin 18 pseudogene 19 | Homo sapiens |
| 2969 | general transcription factor II, i; general transcription factor II, i, pseudogene | Homo sapiens |
| 100093631 | general transcription factor II, i; general transcription factor II, i, pseudogene | Homo sapiens |
| 103 | adenosine deaminase, RNA-specific | Homo sapiens |
| 8508 | nipsnap homolog 1 (C. elegans) | Homo sapiens |
| 3183 | heterogeneous nuclear ribonucleoprotein C (C1/C2) | Homo sapiens |
| 9317 | phosphotriesterase related | Homo sapiens |
| 379025 | hypothetical LOC379025 | Homo sapiens |
| 57412 | arsenic (+3 oxidation state) methyltransferase | Homo sapiens |
| 23506 | KIAA0240 | Homo sapiens |
| 83857 | transmembrane and tetratricopeptide repeat containing 1 | Homo sapiens |
| 6746 | signal sequence receptor, beta (translocon-associated protein beta) | Homo sapiens |
| 10001 | mediator complex subunit 6 | Homo sapiens |
| 550643 | hypothetical LOC550643 | Homo sapiens |
| 119504 | chromosome 10 open reading frame 104 | Homo sapiens |
| 79918 | SET domain containing 6 | Homo sapiens |
| 387082 | SMT3 suppressor of mif two 3 homolog 4 (S. cerevisiae) | Homo sapiens |
| 80823 | basic helix-loop-helix domain containing, class B, 9 | Homo sapiens |

| | | |
|---|---|---|
| 8539 | API5-like 1; apoptosis inhibitor 5 | Homo sapiens |
| 1459 | casein kinase 2, alpha prime polypeptide | Homo sapiens |
| 1073 | cofilin 2 (muscle) | Homo sapiens |
| 6426 | splicing factor, arginine/serine-rich 1 | Homo sapiens |
| 23677 | SH3-domain binding protein 4 | Homo sapiens |
| 65979 | phosphatase and actin regulator 4 | Homo sapiens |
| 51495 | protein tyrosine phosphatase-like A domain containing 1 | Homo sapiens |
| 56260 | chromosome 8 open reading frame 44 | Homo sapiens |
| 8635 | ribonuclease T2 | Homo sapiens |
| 90861 | hematological and neurological expressed 1-like | Homo sapiens |
| 126003 | trafficking protein particle complex 5 | Homo sapiens |
| 57819 | LSM2 homolog, U6 small nuclear RNA associated (S. cerevisiae) | Homo sapiens |
| 55074 | oxidation resistance 1 | Homo sapiens |
| 55248 | transmembrane protein 206 | Homo sapiens |
| 6341 | SCO cytochrome oxidase deficient homolog 1 (yeast) | Homo sapiens |
| 5154 | platelet-derived growth factor alpha polypeptide | Homo sapiens |
| 10422 | UBA domain containing 1 | Homo sapiens |
| 91860 | calmodulin-like 4 | Homo sapiens |
| 329 | baculoviral IAP repeat-containing 2 | Homo sapiens |
| 79871 | RNA polymerase II associated protein 2 | Homo sapiens |
| 7150 | topoisomerase (DNA) I | Homo sapiens |
| 5204 | prefoldin subunit 5 | Homo sapiens |
| 7289 | tubby like protein 3 | Homo sapiens |
| 55860 | actin-related protein 10 homolog (S. cerevisiae) | Homo sapiens |
| 10606 | phosphoribosylaminoimidazole carboxylase, phosphoribosylaminoimidazole succinocarboxamide synthetase | Homo sapiens |
| 23252 | OTU domain containing 3 | Homo sapiens |
| 545 | ataxia telangiectasia and Rad3 related; similar to ataxia telangiectasia and Rad3 related protein | Homo sapiens |
| 6209 | ribosomal protein S15 pseudogene 5; ribosomal protein S15 | Homo sapiens |
| 55719 | family with sequence similarity 178, member A | Homo sapiens |
| 9666 | DAZ interacting protein 3, zinc finger | Homo sapiens |
| 22848 | AP2 associated kinase 1 | Homo sapiens |
| 728936 | neuroblastoma breakpoint family, member 16 | Homo sapiens |
| 79637 | armadillo repeat containing 7 | Homo sapiens |
| 5627 | protein S (alpha) | Homo sapiens |
| 54951 | COMM domain containing 8 | Homo sapiens |
| 9232 | pituitary tumor-transforming 1; pituitary tumor-transforming 2 | Homo sapiens |
| 64718 | unkempt homolog (Drosophila)-like | Homo sapiens |
| 64145 | zinc finger, FYVE domain containing 20 | Homo sapiens |
| 23214 | exportin 6 | Homo sapiens |
| 140609 | NIMA (never in mitosis gene a)-related kinase 7 | Homo sapiens |
| 115024 | 5'-nucleotidase, cytosolic III-like | Homo sapiens |
| 9274 | B-cell CLL/lymphoma 7C | Homo sapiens |
| 54602 | Nedd4 family interacting protein 2 | Homo sapiens |
| 79155 | TNFAIP3 interacting protein 2 | Homo sapiens |
| 27043 | proline, glutamate and leucine rich protein 1 | Homo sapiens |
| 152503 | SH3 domain containing 19 | Homo sapiens |
| 4681 | neuroblastoma, suppression of tumorigenicity 1 | Homo sapiens |
| 8664 | eukaryotic translation initiation factor 3, subunit D | Homo sapiens |
| 255231 | mucolipin 2 | Homo sapiens |
| 54888 | NOL1/NOP2/Sun domain family, member 2 | Homo sapiens |
| 30834 | zinc ribbon domain containing 1 | Homo sapiens |
| 3182 | heterogeneous nuclear ribonucleoprotein A/B | Homo sapiens |
| 23576 | dimethylarginine dimethylaminohydrolase 1 | Homo sapiens |
| 9950 | golgi autoantigen, golgin subfamily a, 5 | Homo sapiens |
| 645676 | hypothetical LOC645676 | Homo sapiens |
| 23383 | KIAA0892 | Homo sapiens |
| 57473 | zinc finger protein 512B | Homo sapiens |
| 253650 | ankyrin repeat domain 18A | Homo sapiens |
| 375056 | melanoma inhibitory activity family, member 3 | Homo sapiens |
| 7329 | ubiquitin-conjugating enzyme E2I (UBC9 homolog, yeast) | Homo sapiens |
| 3204 | homeobox A7 | Homo sapiens |
| 139341 | FUN14 domain containing 1 | Homo sapiens |
| 57787 | MAP/microtubule affinity-regulating kinase 4 | Homo sapiens |
| 126792 | UDP-Gal:betaGal beta 1,3-galactosyltransferase polypeptide 6 | Homo sapiens |
| 2186 | bromodomain PHD finger transcription factor | Homo sapiens |
| 92689 | family with sequence similarity 114, member A1 | Homo sapiens |
| 10576 | chaperonin containing TCP1, subunit 2 (beta) | Homo sapiens |
| 54861 | SNF related kinase | Homo sapiens |
| 84440 | RAB11 family interacting protein 4 (class II) | Homo sapiens |
| 23410 | sirtuin (silent mating type information regulation 2 homolog) 3 (S. cerevisiae) | Homo sapiens |
| 55204 | golgi phosphoprotein 3-like | Homo sapiens |
| 54890 | alkB, alkylation repair homolog 5 (E. coli) | Homo sapiens |
| 5479 | peptidylprolyl isomerase B (cyclophilin B) | Homo sapiens |
| 29970 | schwannomin interacting protein 1 | Homo sapiens |

| | | |
|---|---|---|
| 60481 | ELOVL family member 5, elongation of long chain fatty acids (FEN1/Elo2, SUR4/Elo3-like, yeast) | Homo sapiens |
| 80817 | KIAA1712 | Homo sapiens |
| 65084 | transmembrane protein 135 | Homo sapiens |
| 55901 | thrombospondin, type I, domain containing 1 | Homo sapiens |
| 112770 | chromosome 1 open reading frame 85 | Homo sapiens |
| 64147 | kinesin family member 9 | Homo sapiens |
| 90333 | zinc finger protein 468 | Homo sapiens |
| 54617 | INO80 homolog (S. cerevisiae) | Homo sapiens |
| 9014 | TATA box binding protein (TBP)-associated factor, RNA polymerase I, B, 63kDa | Homo sapiens |
| 54566 | erythrocyte membrane protein band 4.1 like 4B | Homo sapiens |
| 64398 | membrane protein, palmitoylated 5 (MAGUK p55 subfamily member 5) | Homo sapiens |
| 80007 | chromosome 10 open reading frame 88 | Homo sapiens |
| 997 | cell division cycle 34 homolog (S. cerevisiae) | Homo sapiens |
| 81669 | cyclin L2; chemokine (C-C motif) receptor 6 | Homo sapiens |
| 4048 | leukotriene A4 hydrolase | Homo sapiens |
| 8992 | ATPase, H+ transporting, lysosomal 9kDa, V0 subunit e1 | Homo sapiens |
| 55014 | syntaxin 17 | Homo sapiens |
| 9601 | protein disulfide isomerase family A, member 4 | Homo sapiens |
| 55657 | zinc finger protein 692 | Homo sapiens |
| 28512 | NFKB inhibitor interacting Ras-like 1 | Homo sapiens |
| 51123 | zinc finger protein 706 | Homo sapiens |
| 6880 | TAF9 RNA polymerase II, TATA box binding protein (TBP)-associated factor, 32kDa | Homo sapiens |
| 6431 | splicing factor, arginine/serine-rich 6; similar to arginine/serine-rich splicing factor 6 | Homo sapiens |
| 83480 | pseudouridylate synthase 3 | Homo sapiens |
| 23474 | ethylmalonic encephalopathy 1 | Homo sapiens |
| 150684 | copper metabolism (Murr1) domain containing 1 | Homo sapiens |
| 23774 | bromodomain containing 1 | Homo sapiens |
| 10767 | HBS1-like (S. cerevisiae) | Homo sapiens |
| 4595 | mutY homolog (E. coli) | Homo sapiens |
| 4801 | nuclear transcription factor Y, beta | Homo sapiens |
| 324 | adenomatous polyposis coli | Homo sapiens |
| 112936 | vacuolar protein sorting 26 homolog B (S. pombe) | Homo sapiens |
| 832 | capping protein (actin filament) muscle Z-line, beta | Homo sapiens |
| 55585 | ubiquitin-conjugating enzyme E2Q family member 1 | Homo sapiens |
| 56672 | chromosome 11 open reading frame 17 | Homo sapiens |
| 728498 | similar to Golgin subfamily A member 8-like protein 2; Golgin subfamily A member 8-like protein 1; Golgin subfamily A member 8-like protein 3; golgi autoantigen, golgin subfamily a, 9 pseudogene; similar to golgi autoantigen, golgin subfamily a, 8E | Homo sapiens |
| 401022 | hypothetical LOC401022 | Homo sapiens |
| 55285 | RNA binding motif protein 41 | Homo sapiens |
| 1455 | casein kinase 1, gamma 2 | Homo sapiens |
| 9255 | aminoacyl tRNA synthetase complex-interacting multifunctional protein 1 | Homo sapiens |
| 8611 | phosphatidic acid phosphatase type 2A | Homo sapiens |
| 23499 | microtubule-actin crosslinking factor 1 | Homo sapiens |
| 3909 | laminin, alpha 3 | Homo sapiens |
| 400506 | chromosome 16 open reading frame 88 | Homo sapiens |
| 7351 | uncoupling protein 2 (mitochondrial, proton carrier) | Homo sapiens |
| 50618 | intersectin 2 | Homo sapiens |
| 56987 | bobby sox homolog (Drosophila) | Homo sapiens |
| 80821 | DDHD domain containing 1 | Homo sapiens |
| 10000 | v-akt murine thymoma viral oncogene homolog 3 (protein kinase B, gamma) | Homo sapiens |
| 8936 | WAS protein family, member 1 | Homo sapiens |
| 84266 | alkB, alkylation repair homolog 7 (E. coli) | Homo sapiens |
| 54849 | differentially expressed in FDCP 8 homolog (mouse) | Homo sapiens |
| 55825 | peroxisomal trans-2-enoyl-CoA reductase | Homo sapiens |
| 6566 | solute carrier family 16, member 1 (monocarboxylic acid transporter 1) | Homo sapiens |
| 121642 | alkB, alkylation repair homolog 2 (E. coli) | Homo sapiens |
| 84196 | ubiquitin specific peptidase 48 | Homo sapiens |
| 1031 | cyclin-dependent kinase inhibitor 2C (p18, inhibits CDK4) | Homo sapiens |
| 9659 | hypothetical protein LOC100134230; similar to KIAA0454 protein; similar to phosphodiesterase 4D interacting protein isoform 2; phosphodiesterase 4D interacting protein | Homo sapiens |
| 123207 | chromosome 15 open reading frame 40 | Homo sapiens |
| 100132884 | hypothetical protein LOC100132884 | Homo sapiens |
| 8364 | histone cluster 1, H4l; histone cluster 1, H4k; histone cluster 4, H4; histone cluster 1, H4h; histone cluster 1, H4j; histone cluster 1, H4i; histone cluster 1, H4d; histone cluster 1, H4c; histone cluster 1, H4f; histone cluster 1, H4e; histone cluster 1, H4b; histone cluster 1, H4a; histone cluster 2, H4a; histone cluster 2, H4b | Homo sapiens |
| 10066 | secretory carrier membrane protein 2 | Homo sapiens |
| 10228 | syntaxin 6 | Homo sapiens |
| 6635 | small nuclear ribonucleoprotein polypeptide E-like 1; small nuclear ribonucleoprotein polypeptide E; similar to hCG23490 | Homo sapiens |
| 139285 | family with sequence similarity 123B | Homo sapiens |
| 283687 | chromosome 15 open reading frame 37 | Homo sapiens |
| 3572 | interleukin 6 signal transducer (gp130, oncostatin M receptor) | Homo sapiens |

| | | |
|---|---|---|
| 26122 | enhancer of polycomb homolog 2 (Drosophila) | Homo sapiens |
| 317671 | Rieske (Fe-S) domain containing | Homo sapiens |
| 152559 | progestin and adipoQ receptor family member III | Homo sapiens |
| 55113 | XK, Kell blood group complex subunit-related family, member 8 | Homo sapiens |
| 56907 | spire homolog 1 (Drosophila) | Homo sapiens |
| 5423 | polymerase (DNA directed), beta | Homo sapiens |
| 261729 | six transmembrane epithelial antigen of the prostate 2 | Homo sapiens |
| 10982 | microtubule-associated protein, RP/EB family, member 2 | Homo sapiens |
| 55768 | N-glycanase 1 | Homo sapiens |
| 84937 | zinc and ring finger 1 | Homo sapiens |
| 4723 | NADH dehydrogenase (ubiquinone) flavoprotein 1, 51kDa | Homo sapiens |
| 5080 | paired box 6 | Homo sapiens |
| 8880 | far upstream element (FUSE) binding protein 1 | Homo sapiens |
| 22926 | activating transcription factor 6 | Homo sapiens |
| 5236 | phosphoglucomutase 1 | Homo sapiens |
| 27346 | transmembrane protein 97 | Homo sapiens |
| 10166 | solute carrier family 25 (mitochondrial carrier; ornithine transporter) member 15 | Homo sapiens |
| 9656 | mediator of DNA-damage checkpoint 1 | Homo sapiens |
| 8233 | zinc finger (CCCH type), RNA-binding motif and serine/arginine rich 2 | Homo sapiens |
| 5694 | proteasome (prosome, macropain) subunit, beta type, 6 | Homo sapiens |
| 3030 | hydroxyacyl-Coenzyme A dehydrogenase/3-ketoacyl-Coenzyme A thiolase/enoyl-Coenzyme A hydratase (trifunctional protein), alpha subunit | Homo sapiens |
| 1112 | forkhead box N3 | Homo sapiens |
| 23028 | lysine (K)-specific demethylase 1 | Homo sapiens |
| 79002 | chromosome 19 open reading frame 43 | Homo sapiens |
| 84987 | chromosome 12 open reading frame 62 | Homo sapiens |
| 100286793 | hypothetical LOC100286793 | Homo sapiens |
| 2005 | ELK4, ETS-domain protein (SRF accessory protein 1) | Homo sapiens |
| 28992 | MACRO domain containing 1 | Homo sapiens |
| 283820 | NODAL modulator 3; NODAL modulator 1; NODAL modulator 2 | Homo sapiens |
| 23420 | NODAL modulator 3; NODAL modulator 1; NODAL modulator 2 | Homo sapiens |
| 408050 | NODAL modulator 3; NODAL modulator 1; NODAL modulator 2 | Homo sapiens |
| 10189 | THO complex 4 | Homo sapiens |
| 55559 | three prime repair exonuclease 2; HAUS augmin-like complex, subunit 7 | Homo sapiens |
| 11219 | three prime repair exonuclease 2; HAUS augmin-like complex, subunit 7 | Homo sapiens |
| 6232 | ribosomal protein S27 pseudogene 29; ribosomal protein S27 pseudogene 9; ribosomal protein S27 pseudogene 23; ribosomal protein S27 pseudogene 13; ribosomal protein S27; ribosomal protein S27 pseudogene 21; ribosomal protein S27 pseudogene 7; ribosomal protein S27 pseudogene 6; ribosomal protein S27 pseudogene 19 | Homo sapiens |
| 29945 | anaphase promoting complex subunit 4 | Homo sapiens |
| 55662 | hypoxia inducible factor 1, alpha subunit inhibitor | Homo sapiens |
| 11102 | ribonuclease P/MRP 14kDa subunit | Homo sapiens |
| 51209 | RAB9B, member RAS oncogene family | Homo sapiens |
| 644096 | succinate dehydrogenase complex assembly factor 1 | Homo sapiens |
| 7325 | ubiquitin-conjugating enzyme E2E 2 (UBC4/5 homolog, yeast) | Homo sapiens |
| 85463 | zinc finger CCCH-type containing 12C | Homo sapiens |
| 9870 | KIAA0317 | Homo sapiens |
| 28985 | malignant T cell amplified sequence 1 | Homo sapiens |
| 10531 | pitrilysin metallopeptidase 1 | Homo sapiens |
| 8443 | glyceronephosphate O-acyltransferase | Homo sapiens |
| 10147 | splicing factor, arginine/serine-rich 14 | Homo sapiens |
| 54801 | HAUS augmin-like complex, subunit 6 | Homo sapiens |
| 1398 | v-crk sarcoma virus CT10 oncogene homolog (avian) | Homo sapiens |
| 7849 | paired box 8 | Homo sapiens |
| 506 | ATP synthase, H+ transporting, mitochondrial F1 complex, beta polypeptide | Homo sapiens |
| 4860 | nucleoside phosphorylase | Homo sapiens |
| 27163 | N-acylethanolamine acid amidase | Homo sapiens |
| 9409 | peroxisomal biogenesis factor 16 | Homo sapiens |
| 54887 | UHRF1 binding protein 1 | Homo sapiens |
| 53838 | chromosome 11 open reading frame 24 | Homo sapiens |
| 114876 | oxysterol binding protein-like 1A | Homo sapiens |
| 80728 | KIAA1688 protein | Homo sapiens |
| 60560 | MAK10 homolog, amino-acid N-acetyltransferase subunit (S. cerevisiae) | Homo sapiens |
| 7378 | uridine phosphorylase 1 | Homo sapiens |
| 994 | cell division cycle 25 homolog B (S. pombe) | Homo sapiens |
| 1652 | D-dopachrome tautomerase | Homo sapiens |
| 29095 | ORM1-like 2 (S. cerevisiae) | Homo sapiens |
| 2799 | glucosamine (N-acetyl)-6-sulfatase | Homo sapiens |
| 79956 | endoplasmic reticulum metallopeptidase 1 | Homo sapiens |
| 27238 | G patch domain and KOW motifs | Homo sapiens |
| 55855 | family with sequence similarity 45, member B | Homo sapiens |
| 64863 | methyltransferase like 4 | Homo sapiens |
| 65996 | hypothetical LOC65996 | Homo sapiens |
| 57496 | MKL/myocardin-like 2 | Homo sapiens |
| 147179 | WAS/WASL interacting protein family, member 2 | Homo sapiens |
| 84289 | inhibitor of growth family, member 5 | Homo sapiens |
| 889 | KRIT1, ankyrin repeat containing | Homo sapiens |

| | | |
|---|---|---|
| 154215 | Na+/K+ transporting ATPase interacting 2 | Homo sapiens |
| 79979 | TRM2 tRNA methyltransferase 2 homolog B (S. cerevisiae) | Homo sapiens |
| 11183 | mitogen-activated protein kinase kinase kinase kinase 5 | Homo sapiens |
| 168455 | hypothetical protein FLJ36031 | Homo sapiens |
| 4076 | cell cycle associated protein 1 | Homo sapiens |
| 163486 | DENN/MADD domain containing 1B | Homo sapiens |
| 9482 | syntaxin 8 | Homo sapiens |
| 5936 | RNA binding motif protein 14; RNA binding motif protein 4 | Homo sapiens |
| 10432 | RNA binding motif protein 14; RNA binding motif protein 4 | Homo sapiens |
| 3482 | insulin-like growth factor 2 receptor | Homo sapiens |
| 60592 | short coiled-coil protein | Homo sapiens |
| 317 | apoptotic peptidase activating factor 1 | Homo sapiens |
| 27335 | eukaryotic translation initiation factor 3, subunit K | Homo sapiens |
| 79833 | gem (nuclear organelle) associated protein 6 | Homo sapiens |
| 55180 | lines homolog 1 (Drosophila) | Homo sapiens |
| 27430 | methionine adenosyltransferase II, beta | Homo sapiens |
| 23193 | glucosidase, alpha; neutral AB | Homo sapiens |
| 9203 | zinc finger, MYM-type 3 | Homo sapiens |
| 10011 | steroid receptor RNA activator 1 | Homo sapiens |
| 374354 | NHL repeat containing 2 | Homo sapiens |
| 647979 | hypothetical LOC647979 | Homo sapiens |
| 7541 | zinc finger protein 161 homolog (mouse) | Homo sapiens |
| 10498 | coactivator-associated arginine methyltransferase 1 | Homo sapiens |
| 54764 | zinc finger, RAN-binding domain containing 1 | Homo sapiens |
| 286827 | tripartite motif-containing 59 | Homo sapiens |
| 23157 | septin 6 | Homo sapiens |
| 389421 | lin-28 homolog B (C. elegans) | Homo sapiens |
| 9577 | brain and reproductive organ-expressed (TNFRSF1A modulator) | Homo sapiens |
| 728568 | chromosome 12 open reading frame 73 | Homo sapiens |
| 54793 | similar to Potassium channel tetramerisation domain containing 9; potassium channel tetramerisation domain containing 9; similar to potassium channel tetramerisation domain containing 9 | Homo sapiens |
| 9862 | mediator complex subunit 24 | Homo sapiens |
| 463 | zinc finger homeobox 3; hypothetical LOC100132068 | Homo sapiens |
| 51109 | retinol dehydrogenase 11 (all-trans/9-cis/11-cis) | Homo sapiens |
| 55352 | chromosome 17 open reading frame 79 | Homo sapiens |
| 3265 | v-Ha-ras Harvey rat sarcoma viral oncogene homolog | Homo sapiens |
| 19 | ATP-binding cassette, sub-family A (ABC1), member 1 | Homo sapiens |
| 390 | Rho family GTPase 3 | Homo sapiens |
| 5295 | phosphoinositide-3-kinase, regulatory subunit 1 (alpha) | Homo sapiens |
| 54877 | zinc finger, CCHC domain containing 2 | Homo sapiens |
| 9550 | ATPase, H+ transporting, lysosomal 13kDa, V1 subunit G1 | Homo sapiens |
| 770 | carbonic anhydrase XI | Homo sapiens |
| 221120 | alkB, alkylation repair homolog 3 (E. coli) | Homo sapiens |
| 2669 | GTP binding protein overexpressed in skeletal muscle | Homo sapiens |
| 54934 | chromosome 12 open reading frame 41 | Homo sapiens |
| 23 | ATP-binding cassette, sub-family F (GCN20), member 1 | Homo sapiens |
| 137682 | chromosome 8 open reading frame 38 | Homo sapiens |
| 27252 | kelch-like 20 (Drosophila) | Homo sapiens |
| 10163 | WAS protein family, member 2 | Homo sapiens |
| 8907 | adaptor-related protein complex 1, mu 1 subunit | Homo sapiens |
| 5806 | pentraxin-related gene, rapidly induced by IL-1 beta | Homo sapiens |
| 145508 | chromosome 14 open reading frame 145 | Homo sapiens |
| 79009 | DEAD (Asp-Glu-Ala-Asp) box polypeptide 50 | Homo sapiens |
| 553115 | penta-EF-hand domain containing 1 | Homo sapiens |
| 2009 | echinoderm microtubule associated protein like 1 | Homo sapiens |
| 112479 | exoribonuclease 2 | Homo sapiens |
| 3688 | integrin, beta 1 (fibronectin receptor, beta polypeptide, antigen CD29 includes MDF2, MSK12) | Homo sapiens |
| 399474 | transmembrane protein 200B | Homo sapiens |
| 2132 | exostoses (multiple) 2 | Homo sapiens |
| 10130 | protein disulfide isomerase family A, member 6 | Homo sapiens |
| 4125 | mannosidase, alpha, class 2B, member 1 | Homo sapiens |
| 1173 | adaptor-related protein complex 2, mu 1 subunit | Homo sapiens |
| 55743 | checkpoint with forkhead and ring finger domains | Homo sapiens |
| 1456 | casein kinase 1, gamma 3 | Homo sapiens |
| 6158 | ribosomal protein L28 | Homo sapiens |
| 4134 | microtubule-associated protein 4 | Homo sapiens |
| 51361 | hook homolog 1 (Drosophila) | Homo sapiens |
| 150275 | coiled-coil domain containing 117 | Homo sapiens |
| 10818 | fibroblast growth factor receptor substrate 2 | Homo sapiens |
| 138639 | protein tyrosine phosphatase domain containing 1 | Homo sapiens |
| 163590 | torsin A interacting protein 2 | Homo sapiens |
| 6815 | serine/threonine/tyrosine interacting protein | Homo sapiens |
| 84707 | brain expressed X-linked 2 | Homo sapiens |
| 10495 | ecto-NOX disulfide-thiol exchanger 2 | Homo sapiens |
| 10929 | splicing factor, arginine/serine-rich 2B | Homo sapiens |

| | | |
|---|---|---|
| 4350 | N-methylpurine-DNA glycosylase | Homo sapiens |
| 116143 | WD repeat domain 92 | Homo sapiens |
| 10499 | nuclear receptor coactivator 2 | Homo sapiens |
| 84668 | family with sequence similarity 126, member A | Homo sapiens |
| 54958 | transmembrane protein 160 | Homo sapiens |
| 91966 | chromosome X open reading frame 40A; chromosome X open reading frame 40B | Homo sapiens |
| 541578 | chromosome X open reading frame 40A; chromosome X open reading frame 40B | Homo sapiens |
| 8140 | solute carrier family 7 (cationic amino acid transporter, y+ system), member 5 | Homo sapiens |
| 144455 | E2F transcription factor 7 | Homo sapiens |
| 10788 | IQ motif containing GTPase activating protein 2 | Homo sapiens |
| 25937 | WW domain containing transcription regulator 1 | Homo sapiens |
| 10169 | chromosome 15 open reading frame 63; small EDRK-rich factor 2 | Homo sapiens |
| 25764 | chromosome 15 open reading frame 63; small EDRK-rich factor 2 | Homo sapiens |
| 6584 | solute carrier family 22 (organic cation/carnitine transporter), member 5 | Homo sapiens |
| 134553 | chromosome 5 open reading frame 24 | Homo sapiens |
| 57683 | zinc finger, DBF-type containing 2 | Homo sapiens |
| 84324 | SAP domain containing ribonucleoprotein | Homo sapiens |
| 9421 | heart and neural crest derivatives expressed 1 | Homo sapiens |
| 57148 | KIAA1219 | Homo sapiens |
| 81554 | RCC1-like G exchanging factor-like; Williams-Beuren syndrome chromosome region 16 | Homo sapiens |
| 23635 | single-stranded DNA binding protein 2 | Homo sapiens |
| 23213 | sulfatase 1 | Homo sapiens |
| 2874 | G protein pathway suppressor 2 | Homo sapiens |
| 54981 | chromosome 9 open reading frame 95 | Homo sapiens |
| 26472 | protein phosphatase 1, regulatory (inhibitor) subunit 14B | Homo sapiens |
| 55907 | cytidine monophosphate N-acetylneuraminic acid synthetase | Homo sapiens |
| 23255 | KIAA0802 | Homo sapiens |
| 2079 | enhancer of rudimentary homolog (Drosophila) | Homo sapiens |
| 439 | arsA arsenite transporter, ATP-binding, homolog 1 (bacterial) | Homo sapiens |
| 9818 | nucleoporin like 1 | Homo sapiens |
| 5635 | phosphoribosyl pyrophosphate synthetase-associated protein 1 | Homo sapiens |
| 4707 | NADH dehydrogenase (ubiquinone) 1 beta subcomplex, 1, 7kDa | Homo sapiens |
| 81544 | glycerophosphodiester phosphodiesterase domain containing 5 | Homo sapiens |
| 1432 | mitogen-activated protein kinase 14 | Homo sapiens |
| 390916 | nudix (nucleoside diphosphate linked moiety X)-type motif 19 | Homo sapiens |
| 950 | scavenger receptor class B, member 2 | Homo sapiens |
| 7270 | transcription termination factor, RNA polymerase I | Homo sapiens |
| 55954 | zinc finger, matrin type 5 | Homo sapiens |
| 81889 | fumarylacetoacetate hydrolase domain containing 1 | Homo sapiens |
| 79888 | lysophosphatidylcholine acyltransferase 1 | Homo sapiens |
| 868 | Cas-Br-M (murine) ecotropic retroviral transforming sequence b | Homo sapiens |
| 57142 | reticulon 4 | Homo sapiens |
| 64792 | RAB, member RAS oncogene family-like 5 | Homo sapiens |
| 728613 | programmed cell death 6 pseudogene | Homo sapiens |
| 57176 | valyl-tRNA synthetase 2, mitochondrial (putative) | Homo sapiens |
| 29978 | ubiquilin 2 | Homo sapiens |
| 26273 | F-box protein 3 | Homo sapiens |
| 63933 | coiled-coil domain containing 90A | Homo sapiens |
| 51108 | methyltransferase like 9 | Homo sapiens |
| 389856 | ubiquitin specific peptidase 27, X-linked | Homo sapiens |
| 51320 | mex-3 homolog C (C. elegans) | Homo sapiens |
| 100130837 | hypothetical LOC100130837 | Homo sapiens |
| 51372 | coiled-coil domain containing 72; hypothetical LOC729973; hypothetical LOC728416 | Homo sapiens |
| 58513 | epidermal growth factor receptor pathway substrate 15-like 1 | Homo sapiens |
| 220074 | leucine rich transmembrane and O-methyltransferase domain containing | Homo sapiens |
| 346689 | killer cell lectin-like receptor subfamily G, member 2 | Homo sapiens |
| 55027 | HEAT repeat containing 3 | Homo sapiens |
| 9522 | secretory carrier membrane protein 1 | Homo sapiens |
| 84798 | chromosome 19 open reading frame 48 | Homo sapiens |
| 113130 | cell division cycle associated 5 | Homo sapiens |
| 2063 | nuclear receptor subfamily 2, group F, member 6 | Homo sapiens |
| 7779 | solute carrier family 30 (zinc transporter), member 1 | Homo sapiens |
| 137695 | transmembrane protein 68 | Homo sapiens |
| 124512 | chromosome 17 open reading frame 95 | Homo sapiens |
| 9044 | BTAF1 RNA polymerase II, B-TFIID transcription factor-associated, 170kDa (Mot1 homolog, S. cerevisiae) | Homo sapiens |
| 4799 | nuclear transcription factor, X-box binding 1 | Homo sapiens |
| 29097 | cornichon homolog 4 (Drosophila) | Homo sapiens |
| 50865 | heme binding protein 1 | Homo sapiens |
| 644613 | hypothetical protein LOC644613 | Homo sapiens |
| 100287628 | hypothetical protein LOC100287628 | Homo sapiens |
| 151987 | protein phosphatase 4, regulatory subunit 2 | Homo sapiens |
| 140691 | tripartite motif-containing 69 | Homo sapiens |
| 56997 | chaperone, ABC1 activity of bc1 complex homolog (S. pombe) | Homo sapiens |
| 9657 | IQ motif containing B1 | Homo sapiens |
| 5106 | phosphoenolpyruvate carboxykinase 2 (mitochondrial) | Homo sapiens |
| 100287171 | similar to WAS protein family homolog 1 | Homo sapiens |

| | | |
|---|---|---|
| 4318 | matrix metallopeptidase 9 (gelatinase B, 92kDa gelatinase, 92kDa type IV collagenase) | Homo sapiens |
| 124411 | zinc finger protein 720 | Homo sapiens |
| 9775 | eukaryotic translation initiation factor 4A, isoform 3 | Homo sapiens |
| 55004 | chromosome 11 open reading frame 59 | Homo sapiens |
| 90007 | midnolin | Homo sapiens |
| 125150 | zinc finger, SWIM-type containing 7 | Homo sapiens |
| 23011 | RAB21, member RAS oncogene family | Homo sapiens |
| 9538 | etoposide induced 2.4 mRNA | Homo sapiens |
| 56948 | short chain dehydrogenase/reductase family 39U, member 1 | Homo sapiens |
| 10087 | collagen, type IV, alpha 3 (Goodpasture antigen) binding protein | Homo sapiens |
| 55222 | leucine rich repeat containing 20 | Homo sapiens |
| 63917 | UDP-N-acetyl-alpha-D-galactosamine:polypeptide N-acetylgalactosaminyltransferase 11 (GalNAc-T11) | Homo sapiens |
| 50640 | patatin-like phospholipase domain containing 8 | Homo sapiens |
| 157506 | retinol dehydrogenase 10 (all-trans) | Homo sapiens |
| 115106 | HAUS augmin-like complex, subunit 1 | Homo sapiens |
| 6047 | ring finger protein 4; hypothetical LOC644006 | Homo sapiens |
| 58517 | RNA binding motif protein 25 | Homo sapiens |
| 2519 | fucosidase, alpha-L- 2, plasma | Homo sapiens |
| 85403 | ELL associated factor 1 | Homo sapiens |
| 23505 | transmembrane protein 131 | Homo sapiens |
| 6648 | superoxide dismutase 2, mitochondrial | Homo sapiens |
| 5073 | poly(A)-specific ribonuclease (deadenylation nuclease) | Homo sapiens |
| 23310 | non-SMC condensin II complex, subunit D3 | Homo sapiens |
| 58488 | phosphatidylcholine transfer protein | Homo sapiens |
| 3930 | lamin B receptor | Homo sapiens |
| 55602 | CDKN2A interacting protein | Homo sapiens |
| 6874 | TAF4 RNA polymerase II, TATA box binding protein (TBP)-associated factor, 135kDa | Homo sapiens |
| 4775 | nuclear factor of activated T-cells, cytoplasmic, calcineurin-dependent 3 | Homo sapiens |
| 24137 | kinesin family member 4B; kinesin family member 4A | Homo sapiens |
| 22863 | KIAA0831 | Homo sapiens |
| 79991 | oligonucleotide/oligosaccharide-binding fold containing 1 | Homo sapiens |
| 83605 | cerebral cavernous malformation 2 | Homo sapiens |
| 8028 | myeloid/lymphoid or mixed-lineage leukemia (trithorax homolog, Drosophila); translocated to, 10 | Homo sapiens |
| 63976 | PR domain containing 16 | Homo sapiens |
| 64118 | dihydrouridine synthase 1-like (S. cerevisiae) | Homo sapiens |
| 4131 | microtubule-associated protein 1B | Homo sapiens |
| 9877 | zinc finger CCCH-type containing 11A | Homo sapiens |
| 55671 | SMEK homolog 1, suppressor of mek1 (Dictyostelium) | Homo sapiens |
| 9343 | elongation factor Tu GTP binding domain containing 2 | Homo sapiens |
| 79140 | coiled-coil domain containing 28B | Homo sapiens |
| 81688 | chromosome 6 open reading frame 62 | Homo sapiens |
| 39 | acetyl-Coenzyme A acetyltransferase 2 | Homo sapiens |
| 60386 | solute carrier family 25 (mitochondrial thiamine pyrophosphate carrier), member 19 | Homo sapiens |
| 22809 | activating transcription factor 5 | Homo sapiens |
| 23435 | TAR DNA binding protein | Homo sapiens |
| 9682 | lysine (K)-specific demethylase 4A | Homo sapiens |
| 9953 | heparan sulfate (glucosamine) 3-O-sulfotransferase 3B1 | Homo sapiens |
| 23518 | R3H domain containing 1 | Homo sapiens |
| 1964 | eukaryotic translation initiation factor 1A, X-linked | Homo sapiens |
| 23443 | solute carrier family 35 (UDP-N-acetylglucosamine (UDP-GlcNAc) transporter), member A3 | Homo sapiens |
| 3735 | lysyl-tRNA synthetase | Homo sapiens |
| 165055 | coiled-coil domain containing 138 | Homo sapiens |
| 3207 | homeobox A11 | Homo sapiens |
| 22880 | MORC family CW-type zinc finger 2 | Homo sapiens |
| 84527 | zinc finger protein 559 | Homo sapiens |
| 440400 | ribonuclease, RNase K | Homo sapiens |
| 346887 | similar to solute carrier family 16 (monocarboxylic acid transporters), member 14 | Homo sapiens |
| 80742 | proline rich 3 | Homo sapiens |
| 8879 | sphingosine-1-phosphate lyase 1 | Homo sapiens |
| 55168 | mitochondrial ribosomal protein S18A | Homo sapiens |
| 63931 | mitochondrial ribosomal protein S14 | Homo sapiens |
| 10778 | zinc finger protein 271 | Homo sapiens |
| 2976 | general transcription factor IIIC, polypeptide 2, beta 110kDa | Homo sapiens |
| 2091 | fibrillarin | Homo sapiens |
| 55661 | DEAD (Asp-Glu-Ala-Asp) box polypeptide 27 | Homo sapiens |
| 285381 | DPH3, KTI11 homolog (S. cerevisiae); DPH3B, KTI11 homolog B (S. cerevisiae) | Homo sapiens |
| 59343 | SUMO1/sentrin/SMT3 specific peptidase 2 | Homo sapiens |
| 63939 | chromosome 20 open reading frame 177 | Homo sapiens |
| 25946 | zinc finger protein 385A | Homo sapiens |
| 6100 | retinitis pigmentosa 9 (autosomal dominant) | Homo sapiens |
| 84168 | anthrax toxin receptor 1 | Homo sapiens |
| 285193 | dual specificity phosphatase 28 | Homo sapiens |
| 84650 | emopamil binding protein-like | Homo sapiens |
| 26099 | chromosome 1 open reading frame 144 | Homo sapiens |
| 204 | adenylate kinase 2 | Homo sapiens |

| | | |
|---|---|---|
| 891 | cyclin B1 | Homo sapiens |
| 2305 | forkhead box M1 | Homo sapiens |
| 11171 | serine/threonine kinase receptor associated protein | Homo sapiens |
| 90355 | chromosome 5 open reading frame 30 | Homo sapiens |
| 29072 | SET domain containing 2 | Homo sapiens |
| 25828 | thioredoxin 2 | Homo sapiens |
| 8898 | myotubularin related protein 2 | Homo sapiens |
| 80198 | MUS81 endonuclease homolog (S. cerevisiae) | Homo sapiens |
| 1027 | cyclin-dependent kinase inhibitor 1B (p27, Kip1) | Homo sapiens |
| 400579 | FLJ35934 protein | Homo sapiens |
| 10313 | reticulon 3 | Homo sapiens |
| 60488 | mitochondrial ribosomal protein S35 | Homo sapiens |
| 8677 | syntaxin 10 | Homo sapiens |
| 7799 | PR domain containing 2, with ZNF domain | Homo sapiens |
| 51699 | vacuolar protein sorting 29 homolog (S. cerevisiae) | Homo sapiens |
| 10201 | non-metastatic cells 6, protein expressed in (nucleoside-diphosphate kinase) | Homo sapiens |
| 9439 | mediator complex subunit 23 | Homo sapiens |
| 100131755 | similar to hCG1792883 | Homo sapiens |
| 55238 | solute carrier family 38, member 7 | Homo sapiens |
| 23451 | splicing factor 3b, subunit 1, 155kDa | Homo sapiens |
| 10826 | chromosome 5 open reading frame 4 | Homo sapiens |
| 3188 | ribosomal protein L36a pseudogene 51; ribosomal protein L36a pseudogene 37; ribosomal protein L36a pseudogene 49; heterogeneous nuclear ribonucleoprotein H2 (H'); ribosomal protein L36a | Homo sapiens |
| 6173 | ribosomal protein L36a pseudogene 51; ribosomal protein L36a pseudogene 37; ribosomal protein L36a pseudogene 49; heterogeneous nuclear ribonucleoprotein H2 (H'); ribosomal protein L36a | Homo sapiens |
| 79848 | centrosome and spindle pole associated protein 1 | Homo sapiens |
| 7812 | cold shock domain containing E1, RNA-binding | Homo sapiens |
| 22992 | lysine (K)-specific demethylase 2A | Homo sapiens |
| 80324 | pseudouridylate synthase 1 | Homo sapiens |
| 23398 | peptidylprolyl isomerase domain and WD repeat containing 1 | Homo sapiens |
| 9329 | general transcription factor IIIC, polypeptide 4, 90kDa | Homo sapiens |
| 4140 | MAP/microtubule affinity-regulating kinase 3 | Homo sapiens |
| 51582 | antizyme inhibitor 1 | Homo sapiens |
| 2193 | phenylalanyl-tRNA synthetase, alpha subunit | Homo sapiens |
| 9510 | ADAM metallopeptidase with thrombospondin type 1 motif, 1 | Homo sapiens |
| 339005 | similar to WAS protein homolog associated with actin, golgi membranes and microtubules; WAS protein homolog associated with actin, golgi membranes and microtubules-like 1 | Homo sapiens |
| 56063 | chromosome 1 open reading frame 91 | Homo sapiens |
| 5691 | proteasome (prosome, macropain) subunit, beta type, 3 | Homo sapiens |
| 129531 | MIT, microtubule interacting and transport, domain containing 1 | Homo sapiens |
| 7050 | TGFB-induced factor homeobox 1 | Homo sapiens |
| 9040 | ubiquitin-conjugating enzyme E2M (UBC12 homolog, yeast); ubiquitin-conjugating enzyme E2M pseudogene 1 | Homo sapiens |
| 10247 | heat-responsive protein 12 | Homo sapiens |
| 10073 | snurportin 1 | Homo sapiens |
| 151011 | septin 10 | Homo sapiens |
| 54961 | slingshot homolog 3 (Drosophila) | Homo sapiens |
| 54826 | gypsy retrotransposon integrase 1 | Homo sapiens |
| 1363 | carboxypeptidase E | Homo sapiens |
| 862 | runt-related transcription factor 1; translocated to, 1 (cyclin D-related) | Homo sapiens |
| 9375 | transmembrane 9 superfamily member 2 | Homo sapiens |
| 51076 | cutC copper transporter homolog (E. coli) | Homo sapiens |
| 5796 | protein tyrosine phosphatase, receptor type, K | Homo sapiens |
| 56731 | SLC2A4 regulator | Homo sapiens |
| 92092 | zinc finger CCCH-type, antiviral 1-like | Homo sapiens |
| 7068 | thyroid hormone receptor, beta (erythroblastic leukemia viral (v-erb-a) oncogene homolog 2, avian) | Homo sapiens |
| 65059 | Ras association (RalGDS/AF-6) and pleckstrin homology domains 1 | Homo sapiens |
| 10810 | WAS protein family, member 3 | Homo sapiens |
| 467 | activating transcription factor 3 | Homo sapiens |
| 88455 | ankyrin repeat domain 13A | Homo sapiens |
| 3394 | interferon regulatory factor 8 | Homo sapiens |
| 150678 | myeloma overexpressed 2 | Homo sapiens |
| 9318 | COP9 constitutive photomorphogenic homolog subunit 2 (Arabidopsis) | Homo sapiens |
| 339324 | zinc finger protein 260 | Homo sapiens |
| 132949 | aminoadipate-semialdehyde dehydrogenase | Homo sapiens |
| 53918 | pelota homolog (Drosophila) | Homo sapiens |
| 10827 | family with sequence similarity 114, member A2 | Homo sapiens |
| 25880 | transmembrane protein 186 | Homo sapiens |
| 7251 | tumor susceptibility gene 101 | Homo sapiens |
| 2290 | forkhead box G1 | Homo sapiens |
| 23078 | KIAA0564 | Homo sapiens |
| 54941 | ring finger protein 125 | Homo sapiens |
| 114804 | ring finger protein 157 | Homo sapiens |
| 441733 | protein kinase, X-linked, pseudogene 1 | Homo sapiens |

| | | |
|---|---|---|
| 79001 | vitamin K epoxide reductase complex, subunit 1 | Homo sapiens |
| 6879 | TAF7 RNA polymerase II, TATA box binding protein (TBP)-associated factor, 55kDa | Homo sapiens |
| 23545 | ATPase, H+ transporting, lysosomal V0 subunit a2 | Homo sapiens |
| 645566 | hypothetical LOC645566 | Homo sapiens |
| 9275 | B-cell CLL/lymphoma 7B | Homo sapiens |
| 79810 | pentatricopeptide repeat domain 2 | Homo sapiens |
| 55069 | chromosome 7 open reading frame 42 | Homo sapiens |
| 114088 | tripartite motif-containing 9 | Homo sapiens |
| 23446 | solute carrier family 44, member 1 | Homo sapiens |
| 1111 | CHK1 checkpoint homolog (S. pombe) | Homo sapiens |
| 8467 | SWI/SNF related, matrix associated, actin dependent regulator of chromatin, subfamily a, member 5 | Homo sapiens |
| 55132 | La ribonucleoprotein domain family, member 1B | Homo sapiens |
| 56261 | hypothetical protein KIAA1434 | Homo sapiens |
| 6495 | SIX homeobox 1 | Homo sapiens |
| 6627 | small nuclear ribonucleoprotein polypeptide A' | Homo sapiens |
| 29116 | myosin regulatory light chain interacting protein | Homo sapiens |
| 23351 | KIAA0323 | Homo sapiens |
| 27338 | ubiquitin-conjugating enzyme E2S | Homo sapiens |
| 5478 | similar to TRIMCyp; peptidylprolyl isomerase A (cyclophilin A); peptidylprolyl isomerase A (cyclophilin A)-like 3 | Homo sapiens |
| 26277 | TERF1 (TRF1)-interacting nuclear factor 2 | Homo sapiens |
| 113829 | solute carrier family 35, member A4 | Homo sapiens |
| 51497 | TH1-like (Drosophila) | Homo sapiens |
| 54677 | carnitine O-octanoyltransferase | Homo sapiens |
| 23548 | tetratricopeptide repeat domain 33 | Homo sapiens |
| 283508 | hypothetical protein LOC283508 | Homo sapiens |
| 84246 | mediator complex subunit 10 | Homo sapiens |
| 91687 | centromere protein L | Homo sapiens |
| 23173 | methionyl aminopeptidase 1 | Homo sapiens |
| 151009 | hypothetical LOC151009 | Homo sapiens |
| 8881 | cell division cycle 16 homolog (S. cerevisiae) | Homo sapiens |
| 1861 | torsin family 1, member A (torsin A) | Homo sapiens |
| 54443 | anillin, actin binding protein | Homo sapiens |
| 4520 | metal-regulatory transcription factor 1 | Homo sapiens |
| 3551 | inhibitor of kappa light polypeptide gene enhancer in B-cells, kinase beta | Homo sapiens |
| 126308 | MOB1, Mps One Binder kinase activator-like 2A (yeast) | Homo sapiens |
| 27235 | coenzyme Q2 homolog, prenyltransferase (yeast) | Homo sapiens |
| 387066 | small nucleolar RNA host gene 5 (non-protein coding) | Homo sapiens |
| 113000 | RNA pseudouridylate synthase domain containing 1 | Homo sapiens |
| 54753 | zinc finger protein 853 | Homo sapiens |
| 339229 | chromosome 17 open reading frame 90 | Homo sapiens |
| 9261 | mitogen-activated protein kinase-activated protein kinase 2 | Homo sapiens |
| 400916 | coiled-coil-helix-coiled-coil-helix domain containing 10 | Homo sapiens |
| 51030 | family with sequence similarity 18, member B2; family with sequence similarity 18, member B | Homo sapiens |
| 5436 | polymerase (RNA) II (DNA directed) polypeptide G | Homo sapiens |
| 22881 | ankyrin repeat domain 6 | Homo sapiens |
| 9887 | Smg-7 homolog, nonsense mediated mRNA decay factor (C. elegans) | Homo sapiens |
| 11212 | proline synthetase co-transcribed homolog (bacterial) | Homo sapiens |
| 1797 | dom-3 homolog Z (C. elegans) | Homo sapiens |
| 23471 | translocation associated membrane protein 1 | Homo sapiens |
| 7813 | ecotropic viral integration site 5 | Homo sapiens |
| 84293 | chromosome 10 open reading frame 58; chromosome 10 open reading frame 57 | Homo sapiens |
| 221895 | JAZF zinc finger 1 | Homo sapiens |
| 81025 | gap junction protein, alpha 9, 59kDa | Homo sapiens |
| 3213 | homeobox B3 | Homo sapiens |
| 16 | alanyl-tRNA synthetase | Homo sapiens |
| 28991 | COMM domain containing 5 | Homo sapiens |
| 23432 | G protein-coupled receptor 161 | Homo sapiens |
| 9716 | aquarius homolog (mouse) | Homo sapiens |
| 7514 | exportin 1 (CRM1 homolog, yeast) | Homo sapiens |
| 25847 | anaphase promoting complex subunit 13 | Homo sapiens |
| 199990 | chromosome 1 open reading frame 86 | Homo sapiens |
| 83940 | TatD DNase domain containing 1 | Homo sapiens |
| 9586 | cAMP responsive element binding protein 5 | Homo sapiens |
| 51271 | ubiquitin associated protein 1 | Homo sapiens |
| 51427 | zinc finger protein 107 | Homo sapiens |
| 79682 | MLF1 interacting protein | Homo sapiens |
| 1108 | chromodomain helicase DNA binding protein 4 | Homo sapiens |
| 1508 | cathepsin B | Homo sapiens |
| 79813 | euchromatic histone-lysine N-methyltransferase 1 | Homo sapiens |
| 10632 | ATP synthase, H+ transporting, mitochondrial F0 complex, subunit G | Homo sapiens |
| 79834 | NKF3 kinase family member | Homo sapiens |
| 23224 | spectrin repeat containing, nuclear envelope 2 | Homo sapiens |
| 23705 | cell adhesion molecule 1 | Homo sapiens |
| 8731 | RNA (guanine-7-) methyltransferase | Homo sapiens |

| | | |
|---|---|---|
| 740 | mitochondrial ribosomal protein L49 | Homo sapiens |
| 23433 | ras homolog gene family, member Q; similar to small GTP binding protein TC10 | Homo sapiens |
| 8036 | soc-2 suppressor of clear homolog (C. elegans) | Homo sapiens |
| 51569 | ubiquitin-fold modifier 1 | Homo sapiens |
| 9650 | mitochondrial fission regulator 1 | Homo sapiens |
| 7704 | zinc finger and BTB domain containing 16 | Homo sapiens |
| 8540 | alkylglycerone phosphate synthase | Homo sapiens |
| 9448 | mitogen-activated protein kinase kinase kinase kinase 4 | Homo sapiens |
| 54510 | protocadherin 18 | Homo sapiens |
| 79685 | SAP30-like | Homo sapiens |
| 51070 | nitric oxide synthase interacting protein | Homo sapiens |
| 84524 | zinc finger CCCH-type containing 8 | Homo sapiens |
| 79084 | WD repeat domain 77 | Homo sapiens |
| 5770 | protein tyrosine phosphatase, non-receptor type 1 | Homo sapiens |
| 5435 | polymerase (RNA) II (DNA directed) polypeptide F | Homo sapiens |
| 23140 | zinc finger, ZZ-type with EF-hand domain 1 | Homo sapiens |
| 54812 | aftiphilin | Homo sapiens |
| 79921 | transcription elongation factor A (SII)-like 4 | Homo sapiens |
| 1954 | multiple EGF-like-domains 8 | Homo sapiens |
| 3454 | interferon (alpha, beta and omega) receptor 1 | Homo sapiens |
| 11311 | vacuolar protein sorting 45 homolog (S. cerevisiae) | Homo sapiens |
| 80209 | chromosome 13 open reading frame 23 | Homo sapiens |
| 10026 | phosphatidylinositol glycan anchor biosynthesis, class K | Homo sapiens |
| 7073 | TIA1 cytotoxic granule-associated RNA binding protein-like 1 | Homo sapiens |
| 57799 | RAB40C, member RAS oncogene family | Homo sapiens |
| 55967 | NADH dehydrogenase (ubiquinone) 1 alpha subcomplex, 12 | Homo sapiens |
| 3992 | fatty acid desaturase 1 | Homo sapiens |
| 2788 | guanine nucleotide binding protein (G protein), gamma 7 | Homo sapiens |
| 8772 | Fas (TNFRSF6)-associated via death domain | Homo sapiens |
| 1849 | dual specificity phosphatase 7 | Homo sapiens |
| 10093 | tubulin tyrosine ligase-like family, member 3; actin related protein 2/3 complex, subunit 4, 20kDa | Homo sapiens |
| 3423 | iduronate 2-sulfatase | Homo sapiens |
| 9497 | solute carrier family 4, sodium bicarbonate cotransporter, member 7 | Homo sapiens |
| 9993 | DiGeorge syndrome critical region gene 2 | Homo sapiens |
| 284252 | potassium channel tetramerisation domain containing 1 | Homo sapiens |
| 55198 | adaptor protein, phosphotyrosine interaction, PH domain and leucine zipper containing 2 | Homo sapiens |
| 79101 | TATA box binding protein (TBP)-associated factor, RNA polymerase I, D, 41kDa; small nucleolar RNA, H/ACA box 32; small nucleolar RNA, H/ACA box 25 | Homo sapiens |
| 9562 | multiple inositol polyphosphate histidine phosphatase, 1 | Homo sapiens |
| 57103 | chromosome 12 open reading frame 5 | Homo sapiens |
| 567 | beta-2-microglobulin | Homo sapiens |
| 55339 | WD repeat domain 33 | Homo sapiens |
| 6117 | replication protein A1, 70kDa | Homo sapiens |
| 2720 | galactosidase, beta 1 | Homo sapiens |
| 1008 | cadherin 10, type 2 (T2-cadherin) | Homo sapiens |
| 7327 | ubiquitin-conjugating enzyme E2G 2 (UBC7 homolog, yeast) | Homo sapiens |
| 54505 | DEAH (Asp-Glu-Ala-His) box polypeptide 29 | Homo sapiens |
| 5682 | proteasome (prosome, macropain) subunit, alpha type, 1 | Homo sapiens |
| 27109 | ATP synthase, H+ transporting, mitochondrial F0 complex, subunit s (factor B) | Homo sapiens |
| 9469 | carbohydrate (chondroitin 6) sulfotransferase 3 | Homo sapiens |
| 56475 | reprimo, TP53 dependent G2 arrest mediator candidate | Homo sapiens |
| 54477 | pleckstrin homology domain containing, family A member 5 | Homo sapiens |
| 55735 | DnaJ (Hsp40) homolog, subfamily C, member 11 | Homo sapiens |
| 51360 | membrane-bound transcription factor peptidase, site 2 | Homo sapiens |
| 92609 | translocase of inner mitochondrial membrane 50 homolog (S. cerevisiae) | Homo sapiens |
| 5427 | polymerase (DNA directed), epsilon 2 (p59 subunit) | Homo sapiens |
| 8085 | myeloid/lymphoid or mixed-lineage leukemia 2 | Homo sapiens |
| 81572 | p53 and DNA-damage regulated 1 | Homo sapiens |
| 128486 | fat storage-inducing transmembrane protein 2 | Homo sapiens |
| 55627 | sphingomyelin phosphodiesterase 4, neutral membrane (neutral sphingomyelinase-3) | Homo sapiens |
| 1020 | cyclin-dependent kinase 5 | Homo sapiens |
| 221477 | chromosome 6 open reading frame 89 | Homo sapiens |
| 115426 | ubiquitin-like with PHD and ring finger domains 2 | Homo sapiens |
| 10884 | mitochondrial ribosomal protein S30 | Homo sapiens |
| 26276 | vacuolar protein sorting 33 homolog B (yeast) | Homo sapiens |
| 6154 | ribosomal protein L26 pseudogene 33; ribosomal protein L26; ribosomal protein L26 pseudogene 16; ribosomal protein L26 pseudogene 19; ribosomal protein L26 pseudogene 6 | Homo sapiens |
| 29098 | RAN guanine nucleotide release factor | Homo sapiens |
| 8726 | embryonic ectoderm development | Homo sapiens |
| 55695 | NOL1/NOP2/Sun domain family, member 5 | Homo sapiens |
| 10681 | guanine nucleotide binding protein (G protein), beta 5 | Homo sapiens |
| 8924 | hect domain and RLD 2 | Homo sapiens |
| 84364 | ADP-ribosylation factor GTPase activating protein 2 | Homo sapiens |
| 6558 | solute carrier family 12 (sodium/potassium/chloride transporters), member 2 | Homo sapiens |
| 6687 | spastic paraplegia 7 (pure and complicated autosomal recessive) | Homo sapiens |
| 51307 | family with sequence similarity 53, member C | Homo sapiens |

| | | |
|---|---|---|
| 3295 | hydroxysteroid (17-beta) dehydrogenase 4 | Homo sapiens |
| 284106 | CDGSH iron sulfur domain 3 | Homo sapiens |
| 51126 | N-acetyltransferase 5 (GCN5-related, putative) | Homo sapiens |
| 6611 | spermine synthase; similar to spermine synthase | Homo sapiens |
| 2044 | EPH receptor A5 | Homo sapiens |
| 27297 | CGRP receptor component | Homo sapiens |
| 8106 | poly(A) binding protein, nuclear 1 | Homo sapiens |
| 92014 | mitochondrial carrier triple repeat 1 | Homo sapiens |
| 619423 | family with sequence similarity 85, member A | Homo sapiens |
| 55137 | fidgetin | Homo sapiens |
| 154881 | potassium channel tetramerisation domain containing 7 | Homo sapiens |
| 55830 | glycosyltransferase 8 domain containing 1 | Homo sapiens |
| 58190 | CTD (carboxy-terminal domain, RNA polymerase II, polypeptide A) small phosphatase 1 | Homo sapiens |
| 54462 | KIAA1128 | Homo sapiens |
| 60684 | chromosome 4 open reading frame 41 | Homo sapiens |
| 6015 | ring finger protein 1 | Homo sapiens |
| 27153 | zinc finger protein 777 | Homo sapiens |
| 64771 | chromosome 6 open reading frame 106 | Homo sapiens |
| 53340 | sperm autoantigenic protein 17 | Homo sapiens |
| 574029 | dual specificity phosphatase 5 pseudogene | Homo sapiens |
| 80227 | proteasomal ATPase-associated factor 1 | Homo sapiens |
| 11334 | tumor suppressor candidate 2 | Homo sapiens |
| 56254 | ring finger protein 20 | Homo sapiens |
| 501 | aldehyde dehydrogenase 7 family, member A1 | Homo sapiens |
| 22950 | solute carrier family 4 (anion exchanger), member 1, adaptor protein | Homo sapiens |
| 10746 | mitogen-activated protein kinase kinase kinase 2 | Homo sapiens |
| 9053 | microtubule-associated protein 7 | Homo sapiens |
| 23336 | synemin, intermediate filament protein | Homo sapiens |
| 23596 | opsin 3 | Homo sapiens |
| 2618 | phosphoribosylglycinamide formyltransferase, phosphoribosylglycinamide synthetase, phosphoribosylaminoimidazole synthetase | Homo sapiens |
| 57037 | ankyrin repeat and MYND domain containing 2 | Homo sapiens |
| 55216 | chromosome 11 open reading frame 57 | Homo sapiens |
| 91368 | CDKN2A interacting protein N-terminal like | Homo sapiens |
| 8019 | bromodomain containing 3 | Homo sapiens |
| 3516 | recombination signal binding protein for immunoglobulin kappa J region | Homo sapiens |
| 4289 | muskelin 1, intracellular mediator containing kelch motifs | Homo sapiens |
| 113402 | SFT2 domain containing 1 | Homo sapiens |
| 22906 | trafficking protein, kinesin binding 1 | Homo sapiens |
| 9891 | NUAK family, SNF1-like kinase, 1 | Homo sapiens |
| 9787 | discs, large (Drosophila) homolog-associated protein 5 | Homo sapiens |
| 27347 | serine threonine kinase 39 (STE20/SPS1 homolog, yeast) | Homo sapiens |
| 9927 | mitofusin 2 | Homo sapiens |
| 440104 | hypothetical LOC440104 | Homo sapiens |
| 2997 | glycogen synthase 1 (muscle) | Homo sapiens |
| 85236 | histone cluster 1, H2bk | Homo sapiens |
| 7582 | zinc finger protein 33B | Homo sapiens |
| 7417 | voltage-dependent anion channel 2 | Homo sapiens |
| 55897 | mesoderm posterior 1 homolog (mouse) | Homo sapiens |
| 128637 | TBC1 domain family, member 20 | Homo sapiens |
| 9143 | synaptogyrin 3 | Homo sapiens |
| 585 | Bardet-Biedl syndrome 4 | Homo sapiens |
| 54549 | sidekick homolog 2 (chicken) | Homo sapiens |
| 2887 | growth factor receptor-bound protein 10 | Homo sapiens |
| 2534 | FYN oncogene related to SRC, FGR, YES | Homo sapiens |
| 1371 | coproporphyrinogen oxidase | Homo sapiens |
| 57707 | KIAA1609 | Homo sapiens |
| 7067 | thyroid hormone receptor, alpha (erythroblastic leukemia viral (v-erb-a) oncogene homolog, avian) | Homo sapiens |
| 25977 | NECAP endocytosis associated 1 | Homo sapiens |
| 57180 | ARP3 actin-related protein 3 homolog B (yeast) | Homo sapiens |
| 6905 | tubulin folding cofactor E | Homo sapiens |
| 64968 | mitochondrial ribosomal protein S6 | Homo sapiens |
| 84612 | par-6 partitioning defective 6 homolog beta (C. elegans) | Homo sapiens |
| 286052 | hypothetical protein LOC286052 | Homo sapiens |
| 112398 | egl nine homolog 2 (C. elegans) | Homo sapiens |
| 26953 | RAN binding protein 6 | Homo sapiens |
| 7737 | ring finger protein 113A | Homo sapiens |
| 55326 | 1-acylglycerol-3-phosphate O-acyltransferase 5 (lysophosphatidic acid acyltransferase, epsilon) | Homo sapiens |
| 4074 | mannose-6-phosphate receptor (cation dependent) | Homo sapiens |
| 1062 | centromere protein E, 312kDa | Homo sapiens |
| 84950 | PRP38 pre-mRNA processing factor 38 (yeast) domain containing A | Homo sapiens |
| 8263 | coagulation factor VIII-associated (intronic transcript) 2; coagulation factor VIII-associated (intronic transcript) 3; coagulation factor VIII-associated (intronic transcript) 1 | Homo sapiens |

| | | |
|---|---|---|
| 474383 | coagulation factor VIII-associated (intronic transcript) 2; coagulation factor VIII-associated (intronic transcript) 3; coagulation factor VIII-associated (intronic transcript) 1 | Homo sapiens |
| 474384 | coagulation factor VIII-associated (intronic transcript) 2; coagulation factor VIII-associated (intronic transcript) 3; coagulation factor VIII-associated (intronic transcript) 1 | Homo sapiens |
| 64776 | chromosome 11 open reading frame 1 | Homo sapiens |
| 91749 | KIAA1919 | Homo sapiens |
| 92815 | histone cluster 3, H2a | Homo sapiens |
| 56882 | CDC42 small effector 1 | Homo sapiens |
| 28957 | mitochondrial ribosomal protein S28 | Homo sapiens |
| 79930 | docking protein 3 | Homo sapiens |
| 10036 | chromatin assembly factor 1, subunit A (p150) | Homo sapiens |
| 60487 | tRNA methyltransferase 11 homolog (S. cerevisiae) | Homo sapiens |
| 667 | dystonin | Homo sapiens |
| 4050 | lymphotoxin beta (TNF superfamily, member 3) | Homo sapiens |
| 51693 | trafficking protein particle complex 2-like | Homo sapiens |
| 6189 | ribosomal protein S3A pseudogene 5; ribosomal protein S3a pseudogene 47; ribosomal protein S3a pseudogene 49; ribosomal protein S3A; hypothetical LOC100131699; hypothetical LOC100130107 | Homo sapiens |
| 7371 | uridine-cytidine kinase 2 | Homo sapiens |
| 79886 | chromosome 9 open reading frame 82 | Homo sapiens |
| 84919 | protein phosphatase 1, regulatory (inhibitor) subunit 15B | Homo sapiens |
| 84255 | solute carrier family 37 (glycerol-3-phosphate transporter), member 3 | Homo sapiens |
| 283635 | family with sequence similarity 177, member A1 | Homo sapiens |
| 84549 | MAK16 homolog (S. cerevisiae) | Homo sapiens |
| 440675 | neuroblastoma breakpoint family, member 15; neuroblastoma breakpoint family, member 14; neuroblastoma breakpoint family, member 9; neuroblastoma breakpoint family, member 11; neuroblastoma breakpoint family, member 11-like; neuroblastoma breakpoint family, member 10; neuroblastoma breakpoint family, member 12; neuroblastoma breakpoint family, member 20; neuroblastoma breakpoint family, member 1; KIAA1245; neuroblastoma breakpoint family, member 8 | Homo sapiens |
| 55672 | neuroblastoma breakpoint family, member 15; neuroblastoma breakpoint family, member 14; neuroblastoma breakpoint family, member 9; neuroblastoma breakpoint family, member 11; neuroblastoma breakpoint family, member 11-like; neuroblastoma breakpoint family, member 10; neuroblastoma breakpoint family, member 12; neuroblastoma breakpoint family, member 20; neuroblastoma breakpoint family, member 1; KIAA1245; neuroblastoma breakpoint family, member 8 | Homo sapiens |
| 284565 | neuroblastoma breakpoint family, member 15; neuroblastoma breakpoint family, member 14; neuroblastoma breakpoint family, member 9; neuroblastoma breakpoint family, member 11; neuroblastoma breakpoint family, member 11-like; neuroblastoma breakpoint family, member 10; neuroblastoma breakpoint family, member 12; neuroblastoma breakpoint family, member 20; neuroblastoma breakpoint family, member 1; KIAA1245; neuroblastoma breakpoint family, member 8 | Homo sapiens |
| 728841 | neuroblastoma breakpoint family, member 15; neuroblastoma breakpoint family, member 14; neuroblastoma breakpoint family, member 9; neuroblastoma breakpoint family, member 11; neuroblastoma breakpoint family, member 11-like; neuroblastoma breakpoint family, member 10; neuroblastoma breakpoint family, member 12; neuroblastoma breakpoint family, member 20; neuroblastoma breakpoint family, member 1; KIAA1245; neuroblastoma breakpoint family, member 8 | Homo sapiens |
| 200030 | neuroblastoma breakpoint family, member 15; neuroblastoma breakpoint family, member 14; neuroblastoma breakpoint family, member 9; neuroblastoma breakpoint family, member 11; neuroblastoma breakpoint family, member 11-like; neuroblastoma breakpoint family, member 10; neuroblastoma breakpoint family, member 12; neuroblastoma breakpoint family, member 20; neuroblastoma breakpoint family, member 1; KIAA1245; neuroblastoma breakpoint family, member 8 | Homo sapiens |
| 100132406 | neuroblastoma breakpoint family, member 15; neuroblastoma breakpoint family, member 14; neuroblastoma breakpoint family, member 9; neuroblastoma breakpoint family, member 11; neuroblastoma breakpoint family, member 11-like; neuroblastoma breakpoint family, member 10; neuroblastoma breakpoint family, member 12; neuroblastoma breakpoint family, member 20; neuroblastoma breakpoint family, member 1; KIAA1245; neuroblastoma breakpoint family, member 8 | Homo sapiens |
| 400818 | neuroblastoma breakpoint family, member 15; neuroblastoma breakpoint family, member 14; neuroblastoma breakpoint family, member 9; neuroblastoma breakpoint family, member 11; neuroblastoma breakpoint family, member 11-like; neuroblastoma breakpoint family, member 10; neuroblastoma breakpoint family, member 12; neuroblastoma breakpoint family, member 20; neuroblastoma breakpoint family, member 1; KIAA1245; neuroblastoma breakpoint family, member 8 | Homo sapiens |
| 728912 | neuroblastoma breakpoint family, member 15; neuroblastoma breakpoint family, member 14; neuroblastoma breakpoint family, member 9; neuroblastoma breakpoint family, member 11; neuroblastoma breakpoint family, member 11-like; neuroblastoma breakpoint family, member 10; neuroblastoma breakpoint family, member 12; neuroblastoma breakpoint family, member 20; neuroblastoma breakpoint family, member 1; KIAA1245; neuroblastoma breakpoint family, member 8 | Homo sapiens |

| | | |
|---|---|---|
| 25832 | neuroblastoma breakpoint family, member 15; neuroblastoma breakpoint family, member 14; neuroblastoma breakpoint family, member 9; neuroblastoma breakpoint family, member 11; neuroblastoma breakpoint family, member 11-like; neuroblastoma breakpoint family, member 10; neuroblastoma breakpoint family, member 12; neuroblastoma breakpoint family, member 20; neuroblastoma breakpoint family, member 1; KIAA1245; neuroblastoma breakpoint family, member 8 | Homo sapiens |
| 23271 | calmodulin regulated spectrin-associated protein 1-like 1 | Homo sapiens |
| 54821 | excision repair cross-complementing rodent repair deficiency, complementation group 6-like | Homo sapiens |
| 3097 | human immunodeficiency virus type I enhancer binding protein 2 | Homo sapiens |
| 26019 | UPF2 regulator of nonsense transcripts homolog (yeast) | Homo sapiens |
| 2967 | general transcription factor IIH, polypeptide 3, 34kDa | Homo sapiens |
| 91603 | zinc finger protein 830 | Homo sapiens |
| 129685 | TAF8 RNA polymerase II, TATA box binding protein (TBP)-associated factor, 43kDa | Homo sapiens |
| 2034 | endothelial PAS domain protein 1 | Homo sapiens |
| 55837 | E2F-associated phosphoprotein | Homo sapiens |
| 221960 | chromosome 7 open reading frame 28A; similar to Chromosome 7 open reading frame 28B; chromosome 7 open reading frame 28B | Homo sapiens |
| 51622 | chromosome 7 open reading frame 28A; similar to Chromosome 7 open reading frame 28B; chromosome 7 open reading frame 28B | Homo sapiens |
| 51056 | leucine aminopeptidase 3 | Homo sapiens |
| 25852 | armadillo repeat containing 8 | Homo sapiens |
| 3320 | heat shock protein 90kDa alpha (cytosolic), class A member 2; heat shock protein 90kDa alpha (cytosolic), class A member 1 | Homo sapiens |
| 51176 | lymphoid enhancer-binding factor 1 | Homo sapiens |
| 55272 | IMP3, U3 small nucleolar ribonucleoprotein, homolog (yeast) | Homo sapiens |
| 3984 | LIM domain kinase 1 | Homo sapiens |
| 375061 | family with sequence similarity 89, member A | Homo sapiens |
| 260425 | membrane associated guanylate kinase, WW and PDZ domain containing 3 | Homo sapiens |
| 9320 | thyroid hormone receptor interactor 12 | Homo sapiens |
| 51248 | PDZ domain containing 11 | Homo sapiens |
| 7345 | ubiquitin carboxyl-terminal esterase L1 (ubiquitin thiolesterase) | Homo sapiens |
| 51218 | glutaredoxin 5 | Homo sapiens |
| 7320 | ubiquitin-conjugating enzyme E2B (RAD6 homolog) | Homo sapiens |
| 8803 | succinate-CoA ligase, ADP-forming, beta subunit | Homo sapiens |
| 653061 | similar to Golgin subfamily A member 8-like protein 1 | Homo sapiens |
| 23265 | exocyst complex component 7 | Homo sapiens |
| 23510 | potassium channel tetramerisation domain containing 2 | Homo sapiens |
| 9039 | ubiquitin-like modifier activating enzyme 3 | Homo sapiens |
| 3705 | inositol 1,3,4-triphosphate 5/6 kinase | Homo sapiens |
| 79020 | chromosome 7 open reading frame 25 | Homo sapiens |
| 23034 | sterile alpha motif domain containing 4A | Homo sapiens |
| 100287676 | hypothetical protein LOC100287676 | Homo sapiens |
| 1 | alpha-1-B glycoprotein | Homo sapiens |
| 51601 | lipoyltransferase 1 | Homo sapiens |
| 9533 | polymerase (RNA) I polypeptide C, 30kDa | Homo sapiens |
| 55127 | HEAT repeat containing 1 | Homo sapiens |
| 728233 | phosphatidylinositol 4-kinase, catalytic, alpha pseudogene 1 | Homo sapiens |
| 25925 | zinc finger protein 521 | Homo sapiens |
| 79609 | chromosome 14 open reading frame 138 | Homo sapiens |
| 4603 | v-myb myeloblastosis viral oncogene homolog (avian)-like 1 | Homo sapiens |
| 2963 | general transcription factor IIF, polypeptide 2, 30kDa | Homo sapiens |
| 79582 | sperm associated antigen 16 | Homo sapiens |
| 57583 | transmembrane protein 181 | Homo sapiens |
| 6643 | sorting nexin 2 | Homo sapiens |
| 166378 | spermatogenesis associated 5 | Homo sapiens |
| 10487 | CAP, adenylate cyclase-associated protein 1 (yeast) | Homo sapiens |
| 6051 | arginyl aminopeptidase (aminopeptidase B) | Homo sapiens |
| 84133 | zinc and ring finger 3 | Homo sapiens |
| 56267 | cysteine conjugate-beta lyase 2 | Homo sapiens |
| 8604 | solute carrier family 25 (mitochondrial carrier, Aralar), member 12 | Homo sapiens |
| 3712 | isovaleryl Coenzyme A dehydrogenase | Homo sapiens |
| 9200 | protein tyrosine phosphatase-like (proline instead of catalytic arginine), member A | Homo sapiens |
| 441024 | methylenetetrahydrofolate dehydrogenase (NADP+ dependent) 2-like | Homo sapiens |
| 64087 | methylcrotonoyl-Coenzyme A carboxylase 2 (beta) | Homo sapiens |
| 10194 | teashirt zinc finger homeobox 1 | Homo sapiens |
| 83931 | serine/threonine kinase 40 | Homo sapiens |
| 285367 | RNA pseudouridylate synthase domain containing 3 | Homo sapiens |
| 4839 | NOP2 nucleolar protein homolog (yeast) | Homo sapiens |
| 1781 | similar to dynein cytoplasmic 1 intermediate chain 2; dynein, cytoplasmic 1, intermediate chain 2 | Homo sapiens |
| 11051 | nudix (nucleoside diphosphate linked moiety X)-type motif 21 | Homo sapiens |
| 57216 | vang-like 2 (van gogh, Drosophila) | Homo sapiens |
| 23554 | tetraspanin 12 | Homo sapiens |
| 6793 | serine/threonine kinase 10 | Homo sapiens |
| 746 | chromosome 11 open reading frame 10 | Homo sapiens |
| 5611 | DnaJ (Hsp40) homolog, subfamily C, member 3 | Homo sapiens |

| | | |
|---|---|---|
| 5774 | protein tyrosine phosphatase, non-receptor type 3 | Homo sapiens |
| 6747 | signal sequence receptor, gamma (translocon-associated protein gamma) | Homo sapiens |
| 27315 | post-GPI attachment to proteins 2 | Homo sapiens |
| 10296 | macrophage erythroblast attacher | Homo sapiens |
| 201973 | coiled-coil domain containing 111 | Homo sapiens |
| 120534 | chromosome 11 open reading frame 46 | Homo sapiens |
| 54453 | Ras and Rab interactor 2 | Homo sapiens |
| 56995 | tubby like protein 4 | Homo sapiens |
| 55353 | lysosomal protein transmembrane 4 beta | Homo sapiens |
| 8874 | Rho guanine nucleotide exchange factor (GEF) 7 | Homo sapiens |
| 5756 | twinfilin, actin-binding protein, homolog 1 (Drosophila) | Homo sapiens |
| 2787 | guanine nucleotide binding protein (G protein), gamma 5 | Homo sapiens |
| 23014 | F-box protein 21 | Homo sapiens |
| 5684 | proteasome (prosome, macropain) subunit, alpha type, 3 | Homo sapiens |
| 5784 | protein tyrosine phosphatase, non-receptor type 14 | Homo sapiens |
| 23365 | Rho guanine nucleotide exchange factor (GEF) 12 | Homo sapiens |
| 51542 | vacuolar protein sorting 54 homolog (S. cerevisiae) | Homo sapiens |
| 5929 | retinoblastoma binding protein 5 | Homo sapiens |
| 5621 | prion protein | Homo sapiens |
| 9673 | solute carrier family 25, member 44 | Homo sapiens |
| 58486 | zinc finger, BED-type containing 5 | Homo sapiens |
| 6205 | ribosomal protein S11 pseudogene 5; ribosomal protein S11 | Homo sapiens |
| 56889 | transmembrane 9 superfamily member 3 | Homo sapiens |
| 145270 | proline rich membrane anchor 1 | Homo sapiens |
| 54904 | Wolf-Hirschhorn syndrome candidate 1-like 1 | Homo sapiens |
| 6167 | ribosomal protein L37 | Homo sapiens |
| 51631 | LUC7-like 2 (S. cerevisiae) | Homo sapiens |
| 130916 | MTERF domain containing 2 | Homo sapiens |
| 653308 | N-acylsphingosine amidohydrolase (non-lysosomal ceramidase) 2B | Homo sapiens |
| 10906 | TRAF-type zinc finger domain containing 1 | Homo sapiens |
| 26750 | ribosomal protein S6 kinase, 52kDa, polypeptide 1 | Homo sapiens |
| 56270 | WDR45-like | Homo sapiens |
| 352909 | chromosome 19 open reading frame 51 | Homo sapiens |
| 7266 | DnaJ (Hsp40) homolog, subfamily C, member 7 | Homo sapiens |
| 5217 | profilin 2 | Homo sapiens |
| 5324 | pleiomorphic adenoma gene 1 | Homo sapiens |
| 100287015 | hypothetical protein LOC100287015 | Homo sapiens |
| 7071 | Kruppel-like factor 10 | Homo sapiens |
| 23008 | kelch domain containing 10 | Homo sapiens |
| 6218 | ribosomal protein S17 | Homo sapiens |
| 2483 | FSHD region gene 1 | Homo sapiens |
| 865 | core-binding factor, beta subunit | Homo sapiens |
| 7531 | similar to 14-3-3 protein epsilon (14-3-3E) (Mitochondrial import stimulation factor L subunit) (MSF L); tyrosine 3-monooxygenase/tryptophan 5-monooxygenase activation protein, epsilon polypeptide | Homo sapiens |
| 5588 | protein kinase C, theta | Homo sapiens |
| 788 | solute carrier family 25 (carnitine/acylcarnitine translocase), member 20 | Homo sapiens |
| 65991 | FUN14 domain containing 2 | Homo sapiens |
| 8073 | protein tyrosine phosphatase type IVA, member 2 | Homo sapiens |
| 1718 | 24-dehydrocholesterol reductase | Homo sapiens |
| 6996 | similar to G/T mismatch-specific thymine DNA glycosylase; thymine-DNA glycosylase | Homo sapiens |
| 3251 | hypoxanthine phosphoribosyltransferase 1 | Homo sapiens |
| 64943 | 5’-nucleotidase domain containing 2 | Homo sapiens |
| 10273 | STIP1 homology and U-box containing protein 1 | Homo sapiens |
| 2040 | stomatin | Homo sapiens |
| 9125 | RCD1 required for cell differentiation1 homolog (S. pombe) | Homo sapiens |
| 51277 | DnaJ (Hsp40) homolog, subfamily C, member 27 | Homo sapiens |
| 1825 | desmocollin 3 | Homo sapiens |
| 339344 | Myb-related transcription factor, partner of profilin | Homo sapiens |
| 93594 | WD repeat domain 67 | Homo sapiens |
| 159013 | chromosome X open reading frame 38 | Homo sapiens |
| 55796 | muscleblind-like 3 (Drosophila) | Homo sapiens |
| 27183 | vacuolar protein sorting 4 homolog A (S. cerevisiae) | Homo sapiens |
| 6836 | surfeit 4 | Homo sapiens |
| 55638 | Golgi-localized protein | Homo sapiens |
| 5050 | platelet-activating factor acetylhydrolase, isoform Ib, subunit 3 (29kDa) | Homo sapiens |
| 55819 | ring finger protein 130 | Homo sapiens |
| 100294402 | similar to single Ig IL-1R-related molecule | Homo sapiens |
| 2070 | eyes absent homolog 4 (Drosophila) | Homo sapiens |
| 10213 | proteasome (prosome, macropain) 26S subunit, non-ATPase, 14 | Homo sapiens |
| 23048 | formin binding protein 1 | Homo sapiens |
| 100131017 | zinc finger protein 316 | Homo sapiens |
| 146057 | tau tubulin kinase 2 | Homo sapiens |
| 5062 | p21 protein (Cdc42/Rac)-activated kinase 2 | Homo sapiens |
| 94239 | H2A histone family, member V | Homo sapiens |
| 9217 | VAMP (vesicle-associated membrane protein)-associated protein B and C | Homo sapiens |
| 7372 | uridine monophosphate synthetase | Homo sapiens |

| | | |
|---|---|---|
| 9827 | RGP1 retrograde golgi transport homolog (S. cerevisiae) | Homo sapiens |
| 10717 | adaptor-related protein complex 4, beta 1 subunit | Homo sapiens |
| 27248 | chromosome 2 open reading frame 30 | Homo sapiens |
| 7360 | UDP-glucose pyrophosphorylase 2 | Homo sapiens |
| 57532 | nuclear fragile X mental retardation protein interacting protein 2 | Homo sapiens |
| 131583 | family with sequence similarity 43, member A | Homo sapiens |
| 25917 | THUMP domain containing 3 | Homo sapiens |
| 55703 | polymerase (RNA) III (DNA directed) polypeptide B | Homo sapiens |
| 51808 | phosphorylated adaptor for RNA export | Homo sapiens |
| 1316 | Kruppel-like factor 6 | Homo sapiens |
| 55011 | PIH1 domain containing 1 | Homo sapiens |
| 6491 | SCL/TAL1 interrupting locus | Homo sapiens |
| 114049 | Williams Beuren syndrome chromosome region 22 | Homo sapiens |
| 9943 | oxidative-stress responsive 1 | Homo sapiens |
| 100288693 | hypothetical protein LOC100288693 | Homo sapiens |
| 6001 | regulator of G-protein signaling 10 | Homo sapiens |
| 2971 | general transcription factor IIIA | Homo sapiens |
| 51274 | Kruppel-like factor 3 (basic) | Homo sapiens |
| 9521 | eukaryotic translation elongation factor 1 epsilon 1 | Homo sapiens |
| 219771 | cyclin Y | Homo sapiens |
| 2314 | flightless I homolog (Drosophila) | Homo sapiens |
| 100093630 | small nucleolar RNA host gene 8 (non-protein coding) | Homo sapiens |
| 29923 | chromosome 7 open reading frame 68 | Homo sapiens |
| 10210 | topoisomerase I binding, arginine/serine-rich | Homo sapiens |
| 4636 | myosin, light chain 5, regulatory | Homo sapiens |
| 7168 | tropomyosin 1 (alpha) | Homo sapiens |
| 10694 | similar to chaperonin containing TCP1, subunit 8 (theta); chaperonin containing TCP1, subunit 8 (theta) | Homo sapiens |
| 10713 | ubiquitin specific peptidase 39 | Homo sapiens |
| 100131801 | similar to hCG2036585 | Homo sapiens |
| 79022 | transmembrane protein 106C | Homo sapiens |
| 155400 | NOL1/NOP2/Sun domain family, member 5B | Homo sapiens |
| 9918 | non-SMC condensin I complex, subunit D2 | Homo sapiens |
| 25871 | chromosome 3 open reading frame 17 | Homo sapiens |
| 55737 | hypothetical protein LOC100133770; vacuolar protein sorting 35 homolog (S. cerevisiae) | Homo sapiens |
| 221302 | zinc finger with UFM1-specific peptidase domain | Homo sapiens |
| 64864 | regulatory factor X, 7 | Homo sapiens |
| 5431 | polymerase (RNA) II (DNA directed) polypeptide B, 140kDa | Homo sapiens |
| 1632 | dodecenoyl-Coenzyme A delta isomerase (3,2 trans-enoyl-Coenzyme A isomerase) | Homo sapiens |
| 439921 | matrix-remodelling associated 7 | Homo sapiens |
| 7316 | ubiquitin C | Homo sapiens |
| 116068 | LysM, putative peptidoglycan-binding, domain containing 3 | Homo sapiens |
| 25825 | beta-site APP-cleaving enzyme 2 | Homo sapiens |
| 157 | adrenergic, beta, receptor kinase 2 | Homo sapiens |
| 28996 | homeodomain interacting protein kinase 2; similar to homeodomain interacting protein kinase 2 | Homo sapiens |
| 7695 | zinc finger protein 136 | Homo sapiens |
| 79657 | RNA polymerase II associated protein 3 | Homo sapiens |
| 54855 | family with sequence similarity 46, member C | Homo sapiens |
| 8458 | transcription termination factor, RNA polymerase II | Homo sapiens |
| 64208 | popeye domain containing 3 | Homo sapiens |
| 10807 | serologically defined colon cancer antigen 3; similar to Serologically defined colon cancer antigen 3 | Homo sapiens |
| 4700 | NADH dehydrogenase (ubiquinone) 1 alpha subcomplex, 6, 14kDa | Homo sapiens |
| 6405 | sema domain, immunoglobulin domain (Ig), short basic domain, secreted, (semaphorin) 3F | Homo sapiens |
| 440253 | WAS protein homolog associated with actin, golgi membranes and microtubules-like 2 (pseudogene) | Homo sapiens |
| 334 | amyloid beta (A4) precursor-like protein 2 | Homo sapiens |
| 10901 | dehydrogenase/reductase (SDR family) member 4 | Homo sapiens |
| 645745 | metallothionein 1 pseudogene 2 | Homo sapiens |
| 3987 | LIM and senescent cell antigen-like domains 1 | Homo sapiens |
| 150776 | sphingomyelin phosphodiesterase 4, neutral membrane pseudogene | Homo sapiens |
| 9240 | paraneoplastic antigen MA1 | Homo sapiens |
| 153527 | zinc finger, matrin type 2 | Homo sapiens |
| 8834 | transmembrane protein 11 | Homo sapiens |
| 64332 | nuclear factor of kappa light polypeptide gene enhancer in B-cells inhibitor, zeta | Homo sapiens |
| 57110 | HRAS-like suppressor | Homo sapiens |
| 55251 | protein-L-isoaspartate (D-aspartate) O-methyltransferase domain containing 2 | Homo sapiens |
| 9990 | solute carrier family 12 (potassium/chloride transporters), member 6 | Homo sapiens |
| 5499 | protein phosphatase 1, catalytic subunit, alpha isoform | Homo sapiens |
| 90293 | kelch-like 13 (Drosophila) | Homo sapiens |
| 10682 | emopamil binding protein (sterol isomerase) | Homo sapiens |
| 10327 | aldo-keto reductase family 1, member A1 (aldehyde reductase) | Homo sapiens |
| 5143 | phosphodiesterase 4C, cAMP-specific (phosphodiesterase E1 dunce homolog, Drosophila) | Homo sapiens |
| 1329 | cytochrome c oxidase subunit Vb | Homo sapiens |
| 8148 | TAF15 RNA polymerase II, TATA box binding protein (TBP)-associated factor, 68kDa | Homo sapiens |
| 1737 | dihydrolipoamide S-acetyltransferase | Homo sapiens |

| | | |
|---|---|---|
| 51068 | NMD3 homolog (S. cerevisiae) | Homo sapiens |
| 51028 | vacuolar protein sorting 36 homolog (S. cerevisiae) | Homo sapiens |
| 256987 | serine incorporator 5 | Homo sapiens |
| 51164 | dynactin 4 (p62) | Homo sapiens |
| 57099 | apoptosis, caspase activation inhibitor | Homo sapiens |
| 26084 | Src homology 3 domain-containing guanine nucleotide exchange factor | Homo sapiens |
| 51013 | exosome component 1 | Homo sapiens |
| 85465 | selenoprotein I | Homo sapiens |
| 388962 | similar to bolA-like 3; bolA homolog 3 (E. coli) | Homo sapiens |
| 54344 | dolichyl-phosphate mannosyltransferase polypeptide 3 | Homo sapiens |
| 5820 | Pvt1 oncogene (non-protein coding) | Homo sapiens |
| 64766 | S100P binding protein | Homo sapiens |
| 26574 | apoptosis antagonizing transcription factor | Homo sapiens |
| 55726 | chromosome 12 open reading frame 11 | Homo sapiens |
| 60496 | aminoadipate-semialdehyde dehydrogenase-phosphopantetheinyl transferase | Homo sapiens |
| 9648 | GRIP and coiled-coil domain containing 2 | Homo sapiens |
| 51701 | nemo-like kinase | Homo sapiens |
| 2648 | K(lysine) acetyltransferase 2A | Homo sapiens |
| 79571 | GRIP and coiled-coil domain containing 1 | Homo sapiens |
| 9221 | nucleolar and coiled-body phosphoprotein 1 | Homo sapiens |
| 10971 | tyrosine 3-monooxygenase/tryptophan 5-monooxygenase activation protein, theta polypeptide | Homo sapiens |
| 57494 | ribosomal modification protein rimK-like family member B | Homo sapiens |
| 162427 | family with sequence similarity 134, member C | Homo sapiens |
| 25782 | RAB3 GTPase activating protein subunit 2 (non-catalytic) | Homo sapiens |
| 151188 | ADP-ribosylation-like factor 6 interacting protein 6 | Homo sapiens |
| 23245 | astrotactin 2 | Homo sapiens |
| 402 | ADP-ribosylation factor-like 2 | Homo sapiens |
| 10024 | trophinin associated protein (tastin) | Homo sapiens |
| 9306 | suppressor of cytokine signaling 6 | Homo sapiens |
| 285074 | anaphase promoting complex subunit 1 pseudogene | Homo sapiens |
| 51236 | chromosome 8 open reading frame 30A | Homo sapiens |
| 51501 | chromosome 11 open reading frame 73 | Homo sapiens |
| 80148 | PQ loop repeat containing 1 | Homo sapiens |
| 201626 | phosphodiesterase 12 | Homo sapiens |
| 9517 | serine palmitoyltransferase, long chain base subunit 2 | Homo sapiens |
| 2195 | FAT tumor suppressor homolog 1 (Drosophila) | Homo sapiens |
| 164832 | LON peptidase N-terminal domain and ring finger 2 | Homo sapiens |
| 59342 | serine carboxypeptidase 1 | Homo sapiens |
| 10675 | chondroitin sulfate proteoglycan 5 (neuroglycan C) | Homo sapiens |
| 55852 | testis expressed 2 | Homo sapiens |
| 10978 | CLP1, cleavage and polyadenylation factor I subunit, homolog (S. cerevisiae) | Homo sapiens |
| 2526 | fucosyltransferase 4 (alpha (1,3) fucosyltransferase, myeloid-specific) | Homo sapiens |
| 23401 | frequently rearranged in advanced T-cell lymphomas 2 | Homo sapiens |
| 25797 | glutaminyl-peptide cyclotransferase | Homo sapiens |
| 2965 | general transcription factor IIH, polypeptide 1, 62kDa | Homo sapiens |
| 79142 | PHD finger protein 23 | Homo sapiens |
| 23582 | cyclin D-type binding-protein 1 | Homo sapiens |
| 1491 | cystathionase (cystathionine gamma-lyase) | Homo sapiens |
| 140459 | ankyrin repeat and SOCS box-containing 6 | Homo sapiens |
| 51256 | TBC1 domain family, member 7 | Homo sapiens |
| 127703 | chromosome 1 open reading frame 216 | Homo sapiens |
| 166968 | mesoderm induction early response 1, family member 3 | Homo sapiens |
| 57048 | phospholipid scramblase 3 | Homo sapiens |
| 83892 | potassium channel tetramerisation domain containing 10 | Homo sapiens |
| 51586 | mediator complex subunit 15 | Homo sapiens |
| 150223 | YdjC homolog (bacterial) | Homo sapiens |
| 92344 | golgin, RAB6-interacting | Homo sapiens |
| 54517 | pseudouridylate synthase 7 homolog (S. cerevisiae) | Homo sapiens |
| 4354 | membrane protein, palmitoylated 1, 55kDa | Homo sapiens |
| 100129792 | coiled-coil domain containing 152 | Homo sapiens |
| 10544 | protein C receptor, endothelial (EPCR) | Homo sapiens |
| 10142 | A kinase (PRKA) anchor protein (yotiao) 9 | Homo sapiens |
| 9019 | myelin protein zero-like 1 | Homo sapiens |
| 55847 | CDGSH iron sulfur domain 1 | Homo sapiens |
| 51184 | GPN-loop GTPase 3 | Homo sapiens |
| 112970 | KTI12 homolog, chromatin associated (S. cerevisiae) | Homo sapiens |
| 51097 | saccharopine dehydrogenase (putative) | Homo sapiens |
| 5546 | papillary renal cell carcinoma (translocation-associated) | Homo sapiens |
| 85461 | tetratricopeptide repeat, ankyrin repeat and coiled-coil containing 1 | Homo sapiens |
| 91661 | zinc finger protein 765 | Homo sapiens |
| 149603 | ring finger protein 187 | Homo sapiens |
| 127933 | U2AF homology motif (UHM) kinase 1 | Homo sapiens |
| 5792 | protein tyrosine phosphatase, receptor type, F | Homo sapiens |
| 8723 | sorting nexin 4 | Homo sapiens |
| 10726 | nuclear distribution gene C homolog (A. nidulans) | Homo sapiens |
| 4811 | nidogen 1 | Homo sapiens |

| | | |
|---|---|---|
| 1207 | chloride channel, nucleotide-sensitive, 1A | Homo sapiens |
| 11149 | blood vessel epicardial substance | Homo sapiens |
| 9768 | KIAA0101 | Homo sapiens |
| 90736 | family with sequence similarity 104, member B | Homo sapiens |
| 80012 | polyhomeotic homolog 3 (Drosophila) | Homo sapiens |
| 51773 | remodeling and spacing factor 1 | Homo sapiens |
| 10892 | mucosa associated lymphoid tissue lymphoma translocation gene 1 | Homo sapiens |
| 113419 | testis expressed 261 | Homo sapiens |
| 151246 | shugoshin-like 2 (S. pombe) | Homo sapiens |
| 27018 | nerve growth factor receptor (TNFRSF16) associated protein 1 | Homo sapiens |
| 7873 | mesencephalic astrocyte-derived neurotrophic factor | Homo sapiens |
| 8824 | carboxylesterase 2 (intestine, liver) | Homo sapiens |
| 115509 | zinc finger protein 689 | Homo sapiens |
| 54765 | tripartite motif-containing 44 | Homo sapiens |
| 94104 | chromosome 21 open reading frame 66 | Homo sapiens |
| 56888 | potassium channel modulatory factor 1 | Homo sapiens |
| 51605 | tRNA methyltransferase 6 homolog (S. cerevisiae) | Homo sapiens |
| 6497 | v-ski sarcoma viral oncogene homolog (avian) | Homo sapiens |
| 7690 | zinc finger protein 131 | Homo sapiens |
| 10419 | protein arginine methyltransferase 5 | Homo sapiens |
| 22931 | RAB18, member RAS oncogene family | Homo sapiens |
| 84992 | phosphatidylinositol glycan anchor biosynthesis, class Y | Homo sapiens |
| 54535 | coiled-coil alpha-helical rod protein 1 | Homo sapiens |
| 9655 | suppressor of cytokine signaling 5 | Homo sapiens |
| 10472 | zinc finger protein 238 | Homo sapiens |
| 533 | ATPase, H+ transporting, lysosomal 21kDa, V0 subunit b | Homo sapiens |
| 66036 | myotubularin related protein 9 | Homo sapiens |
| 2033 | E1A binding protein p300 | Homo sapiens |
| 64745 | methyltransferase 11 domain containing 1; similar to methyltransferase 11 domain containing 1 isoform 2 | Homo sapiens |
| 10284 | Sin3A-associated protein, 18kDa | Homo sapiens |
| 6787 | NIMA (never in mitosis gene a)-related kinase 4 | Homo sapiens |
| 283768 | golgi autoantigen, golgin subfamily a, 8D; golgi autoantigen, golgin subfamily a, 8C; similar to Golgin subfamily A member 8-like protein 1; golgi autoantigen, golgin subfamily a, 8F; golgi autoantigen, golgin subfamily a, 8G | Homo sapiens |
| 729786 | golgi autoantigen, golgin subfamily a, 8D; golgi autoantigen, golgin subfamily a, 8C; similar to Golgin subfamily A member 8-like protein 1; golgi autoantigen, golgin subfamily a, 8F; golgi autoantigen, golgin subfamily a, 8G | Homo sapiens |
| 100132979 | golgi autoantigen, golgin subfamily a, 8D; golgi autoantigen, golgin subfamily a, 8C; similar to Golgin subfamily A member 8-like protein 1; golgi autoantigen, golgin subfamily a, 8F; golgi autoantigen, golgin subfamily a, 8G | Homo sapiens |
| 23059 | clusterin associated protein 1 | Homo sapiens |
| 10640 | exocyst complex component 5 | Homo sapiens |
| 29099 | COMM domain containing 9 | Homo sapiens |
| 3177 | solute carrier family 29 (nucleoside transporters), member 2 | Homo sapiens |
| 10512 | sema domain, immunoglobulin domain (Ig), short basic domain, secreted, (semaphorin) 3C | Homo sapiens |
| 5933 | retinoblastoma-like 1 (p107) | Homo sapiens |
| 152485 | zinc finger protein 827 | Homo sapiens |
| 26035 | glucuronic acid epimerase | Homo sapiens |
| 4675 | nucleosome assembly protein 1-like 3 | Homo sapiens |
| 91875 | tetratricopeptide repeat domain 5 | Homo sapiens |
| 124044 | spermatogenesis associated 2-like | Homo sapiens |
| 729810 | hypothetical protein LOC729810 | Homo sapiens |
| 23414 | zinc finger protein, multitype 2 | Homo sapiens |
| 285598 | ADP-ribosylation factor-like 10 | Homo sapiens |
| 1122 | choroideremia-like (Rab escort protein 2) | Homo sapiens |
| 91419 | XRCC6 binding protein 1 | Homo sapiens |
| 10459 | MAD2 mitotic arrest deficient-like 2 (yeast) | Homo sapiens |
| 10949 | heterogeneous nuclear ribonucleoprotein A0 | Homo sapiens |
| 4753 | NEL-like 2 (chicken) | Homo sapiens |
| 65003 | mitochondrial ribosomal protein L11 | Homo sapiens |
| 9709 | homocysteine-inducible, endoplasmic reticulum stress-inducible, ubiquitin-like domain member 1 | Homo sapiens |
| 57405 | SPC25, NDC80 kinetochore complex component, homolog (S. cerevisiae) | Homo sapiens |
| 6868 | ADAM metallopeptidase domain 17 | Homo sapiens |
| 5253 | PHD finger protein 2 | Homo sapiens |
| 37 | acyl-Coenzyme A dehydrogenase, very long chain | Homo sapiens |
| 81610 | family with sequence similarity 83, member D | Homo sapiens |
| 64928 | mitochondrial ribosomal protein L14 | Homo sapiens |
| 2553 | GA binding protein transcription factor, beta subunit 1 | Homo sapiens |
| 25959 | KN motif and ankyrin repeat domains 2 | Homo sapiens |
| 90407 | transmembrane protein 41A | Homo sapiens |
| 529 | ATPase, H+ transporting, lysosomal 31kDa, V1 subunit E1 | Homo sapiens |
| 757 | transmembrane protein 50B | Homo sapiens |
| 29798 | hypothetical LOC642669; chromosome 2 open reading frame 27A; hypothetical LOC644525 | Homo sapiens |
| 55622 | tetratricopeptide repeat domain 27 | Homo sapiens |
| 55145 | THAP domain containing, apoptosis associated protein 1 | Homo sapiens |

| | | |
|---|---|---|
| 2043 | EPH receptor A4 | Homo sapiens |
| 8702 | UDP-Gal:betaGlcNAc beta 1,4- galactosyltransferase, polypeptide 4 | Homo sapiens |
| 283373 | ankyrin repeat domain 52 | Homo sapiens |
| 3419 | isocitrate dehydrogenase 3 (NAD+) alpha | Homo sapiens |
| 206358 | solute carrier family 36 (proton/amino acid symporter), member 1 | Homo sapiens |
| 55039 | tRNA methyltransferase 12 homolog (S. cerevisiae) | Homo sapiens |
| 285237 | chromosome 3 open reading frame 38 | Homo sapiens |
| 9637 | fasciculation and elongation protein zeta 2 (zygin II) | Homo sapiens |
| 10728 | prostaglandin E synthase 3 (cytosolic) | Homo sapiens |
| 200734 | sprouty-related, EVH1 domain containing 2 | Homo sapiens |
| 27336 | HIV-1 Tat specific factor 1 | Homo sapiens |
| 200728 | transmembrane protein 17 | Homo sapiens |
| 4594 | methylmalonyl Coenzyme A mutase | Homo sapiens |
| 56894 | 1-acylglycerol-3-phosphate O-acyltransferase 3 | Homo sapiens |
| 84280 | BTB (POZ) domain containing 10 | Homo sapiens |
| 6837 | mediator complex subunit 22 | Homo sapiens |
| 286205 | chromosome 9 open reading frame 126 | Homo sapiens |
| 22794 | cancer susceptibility candidate 3 | Homo sapiens |
| 143384 | chromosome 10 open reading frame 46 | Homo sapiens |
| 643837 | hypothetical LOC643837 | Homo sapiens |
| 55128 | tripartite motif-containing 68 | Homo sapiens |
| 7515 | X-ray repair complementing defective repair in Chinese hamster cells 1 | Homo sapiens |
| 64759 | tensin 3 | Homo sapiens |
| 4301 | similar to Afadin (Protein AF-6); myeloid/lymphoid or mixed-lineage leukemia (trithorax homolog, Drosophila); translocated to, 4 | Homo sapiens |
| 6164 | ribosomal protein L34 | Homo sapiens |
| 51490 | chromosome 9 open reading frame 114 | Homo sapiens |
| 3953 | leptin receptor | Homo sapiens |
| 64412 | GDNF-inducible zinc finger protein 1 | Homo sapiens |
| 134728 | interleukin-1 receptor-associated kinase 1 binding protein 1 | Homo sapiens |
| 284702 | chromosome 1 open reading frame 199 | Homo sapiens |
| 6666 | SRY (sex determining region Y)-box 12 | Homo sapiens |
| 220972 | membrane-associated ring finger (C3HC4) 8 | Homo sapiens |
| 1983 | eukaryotic translation initiation factor 5 | Homo sapiens |
| 55957 | lin-37 homolog (C. elegans) | Homo sapiens |
| 5524 | protein phosphatase 2A activator, regulatory subunit 4 | Homo sapiens |
| 10436 | EMG1 nucleolar protein homolog (S. cerevisiae) | Homo sapiens |
| 23513 | scribbled homolog (Drosophila) | Homo sapiens |
| 79443 | FYVE and coiled-coil domain containing 1 | Homo sapiens |
| 267 | autocrine motility factor receptor | Homo sapiens |
| 27086 | forkhead box P1 | Homo sapiens |
| 653464 | SLIT-ROBO Rho GTPase activating protein 2 pseudogene 1 | Homo sapiens |
| 59274 | mesoderm development candidate 1 | Homo sapiens |
| 55066 | pyruvate dehydrogenase phosphatase regulatory subunit | Homo sapiens |
| 9771 | Rap guanine nucleotide exchange factor (GEF) 5 | Homo sapiens |
| 11167 | follistatin-like 1 | Homo sapiens |
| 55870 | ash1 (absent, small, or homeotic)-like (Drosophila) | Homo sapiens |
| 80254 | centrosomal protein 63kDa | Homo sapiens |
| 116138 | kelch domain containing 3 | Homo sapiens |
| 79660 | protein phosphatase 1, regulatory (inhibitor) subunit 3B | Homo sapiens |
| 57698 | KIAA1598 | Homo sapiens |
| 830 | capping protein (actin filament) muscle Z-line, alpha 2 | Homo sapiens |
| 26521 | similar to translocase of inner mitochondrial membrane 8 homolog B; translocase of inner mitochondrial membrane 8 homolog B (yeast) | Homo sapiens |
| 7275 | tubby homolog (mouse) | Homo sapiens |
| 3631 | inositol polyphosphate-4-phosphatase, type I, 107kDa | Homo sapiens |
| 51167 | cytochrome b5 reductase 4 | Homo sapiens |
| 5906 | RAP1A, member of RAS oncogene family | Homo sapiens |
| 51250 | chromosome 6 open reading frame 203 | Homo sapiens |
| 92181 | ubiquitin domain containing 2 | Homo sapiens |
| 22982 | DIP2 disco-interacting protein 2 homolog C (Drosophila) | Homo sapiens |
| 27342 | RAB guanine nucleotide exchange factor (GEF) 1 | Homo sapiens |
| 85315 | progestin and adipoQ receptor family member VIII | Homo sapiens |
| 231 | aldo-keto reductase family 1, member B1 (aldose reductase) | Homo sapiens |
| 54495 | thioredoxin-related transmembrane protein 3 | Homo sapiens |
| 10152 | abl interactor 2 | Homo sapiens |
| 23397 | non-SMC condensin I complex, subunit H | Homo sapiens |
| 7707 | zinc finger protein 148 | Homo sapiens |
| 92912 | ubiquitin-conjugating enzyme E2Q family member 2 | Homo sapiens |
| 643988 | hypothetical LOC643988 | Homo sapiens |
| 115196 | zinc finger protein 554 | Homo sapiens |
| 56890 | Mdm1 nuclear protein homolog (mouse) | Homo sapiens |
| 11257 | TP53 target 1 (non-protein coding) | Homo sapiens |
| 78994 | proline rich 14 | Homo sapiens |
| 53339 | BTB (POZ) domain containing 1 | Homo sapiens |
| 7993 | UBX domain protein 8 | Homo sapiens |
| 6143 | ribosomal protein L19; ribosomal protein L19 pseudogene 12 | Homo sapiens |

| | | |
|---|---|---|
| 79733 | E2F transcription factor 8 | Homo sapiens |
| 23042 | pyridoxal-dependent decarboxylase domain containing 1 | Homo sapiens |
| 6202 | ribosomal protein S8; ribosomal protein S8 pseudogene 8; ribosomal protein S8 pseudogene 10 | Homo sapiens |
| 64795 | required for meiotic nuclear division 5 homolog A (S. cerevisiae) | Homo sapiens |
| 51642 | mitochondrial ribosomal protein L48 | Homo sapiens |
| 10513 | amyloid beta precursor protein (cytoplasmic tail) binding protein 2 | Homo sapiens |
| 1153 | cold inducible RNA binding protein | Homo sapiens |
| 54107 | polymerase (DNA directed), epsilon 3 (p17 subunit) | Homo sapiens |
| 79072 | FAST kinase domains 3 | Homo sapiens |
| 6655 | son of sevenless homolog 2 (Drosophila) | Homo sapiens |
| 56648 | eukaryotic translation initiation factor 5A2 | Homo sapiens |
| 100289210 | hypothetical protein LOC100289210 | Homo sapiens |
| 167153 | PAP associated domain containing 4 | Homo sapiens |
| 29994 | bromodomain adjacent to zinc finger domain, 2B | Homo sapiens |
| 84991 | RNA binding motif protein 17 | Homo sapiens |
| 9295 | splicing factor, arginine/serine-rich 11 | Homo sapiens |
| 5094 | poly(rC) binding protein 2 | Homo sapiens |
| 202 | absent in melanoma 1 | Homo sapiens |
| 54756 | interleukin 17 receptor D | Homo sapiens |
| 6146 | ribosomal protein L22 pseudogene 11; ribosomal protein L22 | Homo sapiens |
| 6478 | seven in absentia homolog 2 (Drosophila) | Homo sapiens |
| 7754 | zinc finger protein 204 pseudogene | Homo sapiens |
| 84677 | Down syndrome critical region gene 8 | Homo sapiens |
| 83607 | AMME chromosomal region gene 1-like | Homo sapiens |
| 7159 | tumor protein p53 binding protein, 2 | Homo sapiens |
| 307 | annexin A4 | Homo sapiens |
| 124944 | chromosome 17 open reading frame 49 | Homo sapiens |
| 80267 | ER degradation enhancer, mannosidase alpha-like 3 | Homo sapiens |
| 84296 | GINS complex subunit 4 (Sld5 homolog) | Homo sapiens |
| 80854 | SET domain containing (lysine methyltransferase) 7 | Homo sapiens |
| 28951 | tribbles homolog 2 (Drosophila) | Homo sapiens |
| 91754 | NIMA (never in mitosis gene a)- related kinase 9 | Homo sapiens |
| 29946 | SERTA domain containing 3 | Homo sapiens |
| 23108 | GTPase activating Rap/RanGAP domain-like 4 | Homo sapiens |
| 8882 | zinc finger protein 259 | Homo sapiens |
| 259266 | asp (abnormal spindle) homolog, microcephaly associated (Drosophila) | Homo sapiens |
| 10559 | solute carrier family 35 (CMP-sialic acid transporter), member A1 | Homo sapiens |
| 222546 | regulatory factor X, 6 | Homo sapiens |
| 84817 | thioredoxin domain containing 17 | Homo sapiens |
| 5565 | protein kinase, AMP-activated, beta 2 non-catalytic subunit | Homo sapiens |
| 441032 | eukaryotic translation elongation factor 1 alpha-like 7; eukaryotic translation elongation factor 1 alpha-like 3; similar to eukaryotic translation elongation factor 1 alpha 1; eukaryotic translation elongation factor 1 alpha 1 | Homo sapiens |
| 1915 | eukaryotic translation elongation factor 1 alpha-like 7; eukaryotic translation elongation factor 1 alpha-like 3; similar to eukaryotic translation elongation factor 1 alpha 1; eukaryotic translation elongation factor 1 alpha 1 | Homo sapiens |
| 10311 | Down syndrome critical region gene 3 | Homo sapiens |
| 5775 | protein tyrosine phosphatase, non-receptor type 4 (megakaryocyte) | Homo sapiens |
| 1407 | cryptochrome 1 (photolyase-like) | Homo sapiens |
| 2171 | fatty acid binding protein 5-like 2; fatty acid binding protein 5 (psoriasis-associated); fatty acid binding protein 5-like 8; fatty acid binding protein 5-like 7; fatty acid binding protein 5-like 9 | Homo sapiens |
| 57150 | chromosome 6 open reading frame 162 | Homo sapiens |
| 80045 | G protein-coupled receptor 157 | Homo sapiens |
| 51259 | transmembrane protein 216 | Homo sapiens |
| 8446 | dual specificity phosphatase 11 (RNA/RNP complex 1-interacting) | Homo sapiens |
| 3151 | hypothetical LOC729505; similar to hCG2040565; high-mobility group nucleosomal binding domain 2; similar to high-mobility group nucleosomal binding domain 2 | Homo sapiens |
| 1774 | deoxyribonuclease I-like 1 | Homo sapiens |
| 4536 | NADH-ubiquinone oxidoreductase chain 2 | Homo sapiens |
| 79053 | asparagine-linked glycosylation 8, alpha-1,3-glucosyltransferase homolog (S. cerevisiae) | Homo sapiens |
| 29761 | ubiquitin specific peptidase 25 | Homo sapiens |
| 158 | adenylosuccinate lyase | Homo sapiens |
| 9513 | fragile X mental retardation, autosomal homolog 2 | Homo sapiens |
| 10181 | RNA binding motif protein 5 | Homo sapiens |
| 171546 | chromosome 14 open reading frame 147 | Homo sapiens |
| 8451 | cullin 4A | Homo sapiens |
| 64400 | AKT interacting protein; similar to AKT interacting protein | Homo sapiens |
| 554203 | alanyl-tRNA synthetase domain containing 1 pseudogene | Homo sapiens |
| 131076 | coiled-coil domain containing 58 | Homo sapiens |
| 339263 | chromosome 17 open reading frame 51 | Homo sapiens |
| 51081 | mitochondrial ribosomal protein S7 | Homo sapiens |
| 84450 | zinc finger protein 512 | Homo sapiens |
| 116843 | chromosome 6 open reading frame 192 | Homo sapiens |
| 4296 | mitogen-activated protein kinase kinase kinase 11 | Homo sapiens |
| 23196 | family with sequence similarity 120A | Homo sapiens |

| | | |
|---|---|---|
| 375133 | phosphatidylinositol 4-kinase, catalytic, alpha pseudogene 2 | Homo sapiens |
| 11020 | RAB, member of RAS oncogene family-like 4 | Homo sapiens |
| 10762 | nucleoporin 50kDa | Homo sapiens |
| 58155 | polypyrimidine tract binding protein 2 | Homo sapiens |
| 51428 | DEAD (Asp-Glu-Ala-Asp) box polypeptide 41 | Homo sapiens |
| 71 | actin, gamma 1 | Homo sapiens |
| 65124 | ankyrin repeat domain 57 | Homo sapiens |
| 7164 | tumor protein D52-like 1 | Homo sapiens |
| 55711 | fatty acyl CoA reductase 2 | Homo sapiens |
| 28972 | signal peptidase complex subunit 1 homolog (S. cerevisiae) | Homo sapiens |
| 11124 | Fas (TNFRSF6) associated factor 1 | Homo sapiens |
| 22864 | R3H domain containing 2 | Homo sapiens |
| 79664 | NMDA receptor regulated 2 | Homo sapiens |
| 54454 | ATPase family, AAA domain containing 2B | Homo sapiens |
| 51132 | ring finger protein, LIM domain interacting; similar to ring finger protein (C3H2C3 type) 6 | Homo sapiens |
| 90196 | SYS1 Golgi-localized integral membrane protein homolog (S. cerevisiae) | Homo sapiens |
| 10787 | NCK-associated protein 1 | Homo sapiens |
| 1454 | casein kinase 1, epsilon | Homo sapiens |
| 51227 | phosphatidylinositol glycan anchor biosynthesis, class P | Homo sapiens |
| 25873 | ribosomal protein L36; ribosomal protein L36 pseudogene 14 | Homo sapiens |
| 23203 | peptidase (mitochondrial processing) alpha | Homo sapiens |
| 6877 | TAF5 RNA polymerase II, TATA box binding protein (TBP)-associated factor, 100kDa | Homo sapiens |
| 286319 | tumor suppressor candidate 1 | Homo sapiens |
| 84522 | jagunal homolog 1 (Drosophila) | Homo sapiens |
| 7003 | TEA domain family member 1 (SV40 transcriptional enhancer factor) | Homo sapiens |
| 27327 | trinucleotide repeat containing 6A | Homo sapiens |
| 728875 | hypothetical LOC728875 | Homo sapiens |
| 222658 | potassium channel tetramerisation domain containing 20 | Homo sapiens |
| 91012 | LAG1 homolog, ceramide synthase 5 | Homo sapiens |
| 5297 | phosphatidylinositol 4-kinase, catalytic, alpha | Homo sapiens |
| 6170 | ribosomal protein L39 pseudogene 10; ribosomal protein L39 pseudogene 20; ribosomal protein L39 pseudogene 27; ribosomal protein L39; ribosomal protein L39 pseudogene 13; ribosomal protein L39 pseudogene 32 | Homo sapiens |
| 9898 | ubiquitin associated protein 2-like | Homo sapiens |
| 23392 | KIAA0368 | Homo sapiens |
| 999 | cadherin 1, type 1, E-cadherin (epithelial) | Homo sapiens |
| 84002 | UDP-GlcNAc:betaGal beta-1,3-N-acetylglucosaminyltransferase 5 | Homo sapiens |
| 51303 | FK506 binding protein 11, 19 kDa | Homo sapiens |
| 1192 | chloride intracellular channel 1 | Homo sapiens |
| 5425 | polymerase (DNA directed), delta 2, regulatory subunit 50kDa | Homo sapiens |
| 56900 | transmembrane protein 167B | Homo sapiens |
| 55573 | CDV3 homolog (mouse) | Homo sapiens |
| 91768 | Cdk5 and Abl enzyme substrate 1 | Homo sapiens |
| 6908 | TATA box binding protein | Homo sapiens |
| 148203 | zinc finger protein 738 | Homo sapiens |
| 23480 | Sec61 gamma subunit | Homo sapiens |
| 166336 | prickle homolog 2 (Drosophila) | Homo sapiens |
| 9015 | TATA box binding protein (TBP)-associated factor, RNA polymerase I, A, 48kDa | Homo sapiens |
| 152137 | coiled-coil domain containing 50 | Homo sapiens |
| 23341 | DnaJ (Hsp40) homolog, subfamily C, member 16 | Homo sapiens |
| 55706 | transmembrane protein 48 | Homo sapiens |
| 8439 | neutral sphingomyelinase (N-SMase) activation associated factor | Homo sapiens |
| 5954 | reticulocalbin 1, EF-hand calcium binding domain | Homo sapiens |
| 51367 | processing of precursor 5, ribonuclease P/MRP subunit (S. cerevisiae) | Homo sapiens |
| 673 | v-raf murine sarcoma viral oncogene homolog B1 | Homo sapiens |
| 9270 | integrin beta 1 binding protein 1 | Homo sapiens |
| 51397 | COMM domain containing 10 | Homo sapiens |
| 55646 | Ly1 antibody reactive homolog (mouse) | Homo sapiens |
| 6059 | similar to ATP-binding cassette, sub-family E, member 1; ATP-binding cassette, sub-family E (OABP), member 1 | Homo sapiens |
| 6717 | sorcin | Homo sapiens |
| 6773 | signal transducer and activator of transcription 2, 113kDa | Homo sapiens |
| 10775 | processing of precursor 4, ribonuclease P/MRP subunit (S. cerevisiae) | Homo sapiens |
| 5469 | mediator complex subunit 1 | Homo sapiens |
| 154043 | membrane associated guanylate kinase, WW and PDZ domain containing 1; CNKSR family member 3 | Homo sapiens |
| 9223 | membrane associated guanylate kinase, WW and PDZ domain containing 1; CNKSR family member 3 | Homo sapiens |
| 26528 | DAZ associated protein 1 | Homo sapiens |
| 617 | BCS1-like (yeast) | Homo sapiens |
| 10395 | deleted in liver cancer 1 | Homo sapiens |
| 205717 | KIAA2018 | Homo sapiens |
| 285268 | zinc finger protein 621 | Homo sapiens |
| 4293 | mitogen-activated protein kinase kinase kinase 9 | Homo sapiens |
| 23515 | MORC family CW-type zinc finger 3 | Homo sapiens |
| 6293 | vacuolar protein sorting 52 homolog (S. cerevisiae) | Homo sapiens |

| | | |
|---|---|---|
| 257068 | pleckstrin homology-like domain, family B, member 2; phosphatidylinositol-specific phospholipase C, X domain containing 2 | Homo sapiens |
| 90102 | pleckstrin homology-like domain, family B, member 2; phosphatidylinositol-specific phospholipase C, X domain containing 2 | Homo sapiens |
| 23097 | cell division cycle 2-like 6 (CDK8-like) | Homo sapiens |
| 988 | CDC5 cell division cycle 5-like (S. pombe) | Homo sapiens |
| 65220 | NAD kinase | Homo sapiens |
| 26272 | F-box protein 4 | Homo sapiens |
| 55650 | phosphatidylinositol glycan anchor biosynthesis, class V | Homo sapiens |
| 83637 | zinc finger, MIZ-type containing 2 | Homo sapiens |
| 6546 | solute carrier family 8 (sodium/calcium exchanger), member 1 | Homo sapiens |
| 1948 | ephrin-B2 | Homo sapiens |
| 4738 | neural precursor cell expressed, developmentally down-regulated 8; similar to neural precursor cell expressed, developmentally down-regulated gene 8 | Homo sapiens |
| 6124 | ribosomal protein L4; ribosomal protein L4 pseudogene 5; ribosomal protein L4 pseudogene 4 | Homo sapiens |
| 5689 | proteasome (prosome, macropain) subunit, beta type, 1 | Homo sapiens |
| 96610 | BMS1 homolog, ribosome assembly protein (yeast) pseudogene | Homo sapiens |
| 340719 | nanos homolog 1 (Drosophila) | Homo sapiens |
| 79089 | transmembrane and ubiquitin-like domain containing 2 | Homo sapiens |
| 54968 | transmembrane protein 70 | Homo sapiens |
| 328 | APEX nuclease (multifunctional DNA repair enzyme) 1 | Homo sapiens |
| 5201 | prefoldin subunit 1 | Homo sapiens |
| 8239 | ubiquitin specific peptidase 9, X-linked | Homo sapiens |
| 100272147 | mature T-cell proliferation 1 neighbor; mature T-cell proliferation 1 | Homo sapiens |
| 30001 | ERO1-like (S. cerevisiae) | Homo sapiens |
| 4233 | met proto-oncogene (hepatocyte growth factor receptor) | Homo sapiens |
| 55749 | cell division cycle and apoptosis regulator 1 | Homo sapiens |
| 6667 | Sp1 transcription factor | Homo sapiens |
| 25974 | methylmalonic aciduria (cobalamin deficiency) cblC type, with homocystinuria | Homo sapiens |
| 57459 | GATA zinc finger domain containing 2B | Homo sapiens |
| 84064 | haloacid dehalogenase-like hydrolase domain containing 2 | Homo sapiens |
| 84971 | ATG4 autophagy related 4 homolog D (S. cerevisiae) | Homo sapiens |
| 51449 | prenylcysteine oxidase 1 | Homo sapiens |
| 79029 | spermatogenesis associated 5-like 1 | Homo sapiens |
| 4221 | multiple endocrine neoplasia I | Homo sapiens |
| 55009 | chromosome 19 open reading frame 24 | Homo sapiens |
| 22927 | hyaluronan binding protein 4 | Homo sapiens |
| 7178 | similar to tumor protein, translationally-controlled 1; tumor protein, translationally-controlled 1 | Homo sapiens |
| 57403 | RAB22A, member RAS oncogene family | Homo sapiens |
| 150864 | family with sequence similarity 117, member B | Homo sapiens |
| 25806 | ventral anterior homeobox 2 | Homo sapiens |
| 22878 | KIAA1012 | Homo sapiens |
| 222161 | hypothetical protein DKFZp586I1420 | Homo sapiens |
| 498 | ATP synthase, H+ transporting, mitochondrial F1 complex, alpha subunit 1, cardiac muscle | Homo sapiens |
| 5836 | phosphorylase, glycogen, liver | Homo sapiens |
| 60685 | zinc finger, AN1-type domain 3 | Homo sapiens |
| 10987 | COP9 constitutive photomorphogenic homolog subunit 5 (Arabidopsis) | Homo sapiens |
| 51114 | zinc finger, DHHC-type containing 9 | Homo sapiens |
| 1488 | C-terminal binding protein 2 | Homo sapiens |
| 223 | aldehyde dehydrogenase 9 family, member A1 | Homo sapiens |
| 7561 | zinc finger protein 14 | Homo sapiens |
| 51317 | PHD finger protein 21A | Homo sapiens |
| 91734 | isopentenyl-diphosphate delta isomerase 2 | Homo sapiens |
| 91057 | coiled-coil domain containing 34 | Homo sapiens |
| 23468 | chromobox homolog 5 (HP1 alpha homolog, Drosophila) | Homo sapiens |
| 1955 | multiple EGF-like-domains 9 | Homo sapiens |
| 125058 | TBC1 domain family, member 16 | Homo sapiens |
| 124801 | LSM12 homolog (S. cerevisiae) | Homo sapiens |
| 858 | caveolin 2 | Homo sapiens |
| 7307 | U2 small nuclear RNA auxiliary factor 1 | Homo sapiens |
| 56658 | tripartite motif-containing 39 | Homo sapiens |
| 7994 | MYST histone acetyltransferase (monocytic leukemia) 3 | Homo sapiens |
| 56061 | ubiquitin family domain containing 1 | Homo sapiens |
| 64747 | major facilitator superfamily domain containing 1 | Homo sapiens |
| 4702 | NADH dehydrogenase (ubiquinone) 1 alpha subcomplex, 8, 19kDa | Homo sapiens |
| 51258 | mitochondrial ribosomal protein L51 | Homo sapiens |
| 9782 | matrin 3 | Homo sapiens |
| 11031 | RAB31, member RAS oncogene family | Homo sapiens |
| 55075 | uveal autoantigen with coiled-coil domains and ankyrin repeats | Homo sapiens |
| 51651 | peptidyl-tRNA hydrolase 2 | Homo sapiens |
| 1500 | catenin (cadherin-associated protein), delta 1 | Homo sapiens |
| 6780 | staufen, RNA binding protein, homolog 1 (Drosophila) | Homo sapiens |
| 29766 | tropomodulin 3 (ubiquitous) | Homo sapiens |
| 6923 | transcription elongation factor B (SIII), polypeptide 2 (18kDa, elongin B) | Homo sapiens |
| 7074 | T-cell lymphoma invasion and metastasis 1 | Homo sapiens |

| | | |
|---|---|---|
| 64105 | centromere protein K | Homo sapiens |
| 10236 | heterogeneous nuclear ribonucleoprotein R | Homo sapiens |
| 80028 | F-box and leucine-rich repeat protein 18 | Homo sapiens |
| 150472 | COBW domain containing 2 | Homo sapiens |
| 1266 | calponin 3, acidic | Homo sapiens |
| 79776 | zinc finger homeobox 4 | Homo sapiens |
| 8815 | similar to barrier-to-autointegration factor; barrier to autointegration factor 1 | Homo sapiens |
| 134353 | LSM11, U7 small nuclear RNA associated | Homo sapiens |
| 10966 | RAB40B, member RAS oncogene family | Homo sapiens |
| 53371 | nucleoporin 54kDa | Homo sapiens |
| 84437 | KIAA1826 | Homo sapiens |
| 100131262 | hypothetical LOC100131262 | Homo sapiens |
| 55856 | acyl-CoA thioesterase 13 | Homo sapiens |
| 286077 | family with sequence similarity 83, member H | Homo sapiens |
| 8394 | phosphatidylinositol-4-phosphate 5-kinase, type I, alpha | Homo sapiens |
| 28978 | transmembrane protein 14A | Homo sapiens |
| 51646 | yippee-like 5 (Drosophila) | Homo sapiens |
| 9686 | vestigial like 4 (Drosophila) | Homo sapiens |
| 10714 | polymerase (DNA-directed), delta 3, accessory subunit | Homo sapiens |
| 5366 | phorbol-12-myristate-13-acetate-induced protein 1 | Homo sapiens |
| 11267 | SNF8, ESCRT-II complex subunit, homolog (S. cerevisiae) | Homo sapiens |
| 11162 | nudix (nucleoside diphosphate linked moiety X)-type motif 6 | Homo sapiens |
| 5926 | AT rich interactive domain 4A (RBP1-like) | Homo sapiens |
| 9883 | POM121 membrane glycoprotein (rat) | Homo sapiens |
| 672 | breast cancer 1, early onset | Homo sapiens |
| 654 | bone morphogenetic protein 6 | Homo sapiens |
| 6125 | ribosomal protein L5 pseudogene 34; ribosomal protein L5 pseudogene 1; ribosomal protein L5 | Homo sapiens |
| 84861 | kelch-like 22 (Drosophila) | Homo sapiens |
| 7503 | X (inactive)-specific transcript (non-protein coding) | Homo sapiens |
| 84328 | leucine zipper and CTNNBIP1 domain containing | Homo sapiens |
| 29068 | zinc finger and BTB domain containing 44 | Homo sapiens |
| 83479 | DEAD (Asp-Glu-Ala-Asp) box polypeptide 59 | Homo sapiens |
| 339231 | ADP-ribosylation factor-like 16 | Homo sapiens |
| 55226 | N-acetyltransferase 10 (GCN5-related) | Homo sapiens |
| 51603 | methyltransferase like 13 | Homo sapiens |
| 3221 | homeobox C4 | Homo sapiens |
| 79573 | tetratricopeptide repeat domain 13 | Homo sapiens |
| 79169 | chromosome 1 open reading frame 35 | Homo sapiens |
| 8739 | harakiri, BCL2 interacting protein (contains only BH3 domain) | Homo sapiens |
| 51374 | chromosome 2 open reading frame 28 | Homo sapiens |
| 605 | B-cell CLL/lymphoma 7A | Homo sapiens |
| 2247 | fibroblast growth factor 2 (basic) | Homo sapiens |
| 90338 | zinc finger protein 160 | Homo sapiens |
| 7163 | tumor protein D52 | Homo sapiens |
| 5376 | peripheral myelin protein 22 | Homo sapiens |
| 5500 | protein phosphatase 1, catalytic subunit, beta isoform; speedy homolog A (Xenopus laevis) | Homo sapiens |
| 158358 | KIAA2026 | Homo sapiens |
| 54942 | chromosome 9 open reading frame 6 | Homo sapiens |
| 25923 | atlastin GTPase 3 | Homo sapiens |
| 204851 | homeodomain interacting protein kinase 1 | Homo sapiens |
| 284361 | chromosome 19 open reading frame 63 | Homo sapiens |
| 6137 | ribosomal protein L13 pseudogene 12; ribosomal protein L13 | Homo sapiens |
| 8520 | histone acetyltransferase 1 | Homo sapiens |
| 10973 | activating signal cointegrator 1 complex subunit 3 | Homo sapiens |
| 9218 | VAMP (vesicle-associated membrane protein)-associated protein A, 33kDa | Homo sapiens |
| 27247 | NFU1 iron-sulfur cluster scaffold homolog (S. cerevisiae) | Homo sapiens |
| 51012 | slowmo homolog 2 (Drosophila) | Homo sapiens |
| 54925 | zinc finger protein 434 | Homo sapiens |
| 56893 | ubiquilin 4 | Homo sapiens |
| 286109 | hypothetical protein LOC286109 | Homo sapiens |
| 25970 | SH2B adaptor protein 1 | Homo sapiens |
| 54851 | ankyrin repeat domain 49 | Homo sapiens |
| 91137 | solute carrier family 25, member 46 | Homo sapiens |
| 9640 | zinc finger protein 592 | Homo sapiens |
| 302 | annexin A2 pseudogene 3; annexin A2; annexin A2 pseudogene 1 | Homo sapiens |
| 58527 | chromosome 6 open reading frame 115 | Homo sapiens |
| 8864 | period homolog 2 (Drosophila) | Homo sapiens |
| 9669 | eukaryotic translation initiation factor 5B | Homo sapiens |
| 7770 | zinc finger protein 227 | Homo sapiens |
| 29110 | TANK-binding kinase 1 | Homo sapiens |
| 90141 | chromosome 14 open reading frame 143 | Homo sapiens |
| 123803 | N-terminal asparagine amidase | Homo sapiens |
| 116228 | family with sequence similarity 36, member A | Homo sapiens |
| 55670 | peroxisomal biogenesis factor 26 | Homo sapiens |
| 4729 | NADH dehydrogenase (ubiquinone) flavoprotein 2, 24kDa | Homo sapiens |

| | | |
|---|---|---|
| 80124 | valosin containing protein (p97)/p47 complex interacting protein 1 | Homo sapiens |
| 58493 | chromosome 9 open reading frame 80 | Homo sapiens |
| 57680 | chromodomain helicase DNA binding protein 8 | Homo sapiens |
| 10282 | hypothetical protein LOC100128542; blocked early in transport 1 homolog (S. cerevisiae) | Homo sapiens |
| 57162 | pellino homolog 1 (Drosophila) | Homo sapiens |
| 89891 | WD repeat domain 34 | Homo sapiens |
| 2146 | enhancer of zeste homolog 2 (Drosophila) | Homo sapiens |
| 7220 | transient receptor potential cation channel, subfamily C, member 1 | Homo sapiens |
| 23360 | formin binding protein 4 | Homo sapiens |
| 170960 | zinc finger protein 721 | Homo sapiens |
| 8648 | nuclear receptor coactivator 1 | Homo sapiens |
| 494513 | deafness, autosomal recessive 59 | Homo sapiens |
| 6652 | sorbitol dehydrogenase | Homo sapiens |
| 84340 | G elongation factor, mitochondrial 2 | Homo sapiens |
| 22934 | ribose 5-phosphate isomerase A | Homo sapiens |
| 10144 | family with sequence similarity 13, member A | Homo sapiens |
| 728392 | hypothetical protein LOC728392 | Homo sapiens |
| 4698 | NADH dehydrogenase (ubiquinone) 1 alpha subcomplex, 5, 13kDa | Homo sapiens |
| 140823 | reactive oxygen species modulator 1 | Homo sapiens |
| 24139 | echinoderm microtubule associated protein like 2 | Homo sapiens |
| 29128 | ubiquitin-like with PHD and ring finger domains 1 | Homo sapiens |
| 8452 | cullin 3 | Homo sapiens |
| 8065 | cullin 5 | Homo sapiens |
| 476 | ATPase, Na+/K+ transporting, alpha 1 polypeptide | Homo sapiens |
| 25831 | HECT domain containing 1 | Homo sapiens |
| 2023 | enolase 1, (alpha) | Homo sapiens |
| 4513 | Cytochrome c oxidase subunit 2 | Homo sapiens |
| 7414 | vinculin | Homo sapiens |
| 10609 | synaptonemal complex protein SC65 | Homo sapiens |
| 23403 | F-box protein 46 | Homo sapiens |
| 10116 | fem-1 homolog b (C. elegans) | Homo sapiens |
| 9276 | coatomer protein complex, subunit beta 2 (beta prime) | Homo sapiens |
| 100287616 | hypothetical protein LOC100287616 | Homo sapiens |
| 5898 | v-ral simian leukemia viral oncogene homolog A (ras related) | Homo sapiens |
| 440983 | hypothetical gene supported by BC066916 | Homo sapiens |
| 4286 | microphthalmia-associated transcription factor | Homo sapiens |
| 1349 | cytochrome c oxidase subunit VIIb | Homo sapiens |
| 84333 | polycomb group ring finger 5 | Homo sapiens |
| 5471 | phosphoribosyl pyrophosphate amidotransferase | Homo sapiens |
| 5601 | mitogen-activated protein kinase 9 | Homo sapiens |
| 84056 | katanin p60 subunit A-like 1 | Homo sapiens |
| 9134 | cyclin E2 | Homo sapiens |
| 158135 | tubulin tyrosine ligase-like family, member 11 | Homo sapiens |
| 29890 | RNA binding motif protein 15B | Homo sapiens |
| 79589 | ring finger protein 128 | Homo sapiens |
| 83732 | RIO kinase 1 (yeast) | Homo sapiens |
| 92140 | metadherin | Homo sapiens |
| 154791 | chromosome 7 open reading frame 55 | Homo sapiens |
| 10423 | CDP-diacylglycerol--inositol 3-phosphatidyltransferase (phosphatidylinositol synthase) | Homo sapiens |
| 9786 | KIAA0586 | Homo sapiens |
| 120526 | DnaJ (Hsp40) homolog, subfamily C, member 24 | Homo sapiens |
| 440672 | nudix (nucleoside diphosphate linked moiety X)-type motif 4; nudix (nucleoside diphosphate linked moiety X)-type motif 4 pseudogene 1 | Homo sapiens |
| 11163 | nudix (nucleoside diphosphate linked moiety X)-type motif 4; nudix (nucleoside diphosphate linked moiety X)-type motif 4 pseudogene 1 | Homo sapiens |
| 23276 | kelch-like 18 (Drosophila) | Homo sapiens |
| 55331 | alkaline ceramidase 3 | Homo sapiens |
| 9847 | KIAA0528 | Homo sapiens |
| 10478 | solute carrier family 25 (mitochondrial carrier; peroxisomal membrane protein, 34kDa), member 17 | Homo sapiens |
| 642477 | hypothetical LOC642477; hypothetical LOC400879 | Homo sapiens |
| 55205 | similar to zinc finger protein 347; zinc finger protein 532 | Homo sapiens |
| 64946 | centromere protein H | Homo sapiens |
| 60509 | ATP/GTP binding protein-like 5 | Homo sapiens |
| 92906 | heterogeneous nuclear ribonucleoprotein L-like | Homo sapiens |
| 4200 | malic enzyme 2, NAD(+)-dependent, mitochondrial | Homo sapiens |
| 1351 | cytochrome c oxidase subunit 8A (ubiquitous) | Homo sapiens |
| 114793 | formin-like 2 | Homo sapiens |
| 7756 | zinc finger protein 207 | Homo sapiens |
| 5151 | phosphodiesterase 8A | Homo sapiens |
| 84320 | acyl-Coenzyme A binding domain containing 6 | Homo sapiens |
| 85437 | zinc finger CCHC-type and RNA binding motif 1 | Homo sapiens |
| 3985 | LIM domain kinase 2 | Homo sapiens |
| 157769 | family with sequence similarity 91, member A1 | Homo sapiens |
| 284232 | ankyrin repeat domain 20 family, member A2 pseudogene; ankyrin repeat domain 20B; ankyrin repeat domain 20 family, member A4 | Homo sapiens |

| | | |
|---|---|---|
| 728747 | ankyrin repeat domain 20 family, member A2 pseudogene; ankyrin repeat domain 20B; ankyrin repeat domain 20 family, member A4 | Homo sapiens |
| 23592 | LEM domain containing 3 | Homo sapiens |
| 91298 | chromosome 12 open reading frame 29 | Homo sapiens |
| 534 | ATPase, H+ transporting, lysosomal 13kDa, V1 subunit G2 | Homo sapiens |
| 23741 | EP300 interacting inhibitor of differentiation 1 | Homo sapiens |
| 4673 | nucleosome assembly protein 1-like 1 | Homo sapiens |
| 8406 | sushi-repeat-containing protein, X-linked | Homo sapiens |
| 128153 | spermatogenesis associated 17 | Homo sapiens |
| 10180 | RNA binding motif protein 6 | Homo sapiens |
| 8573 | calcium/calmodulin-dependent serine protein kinase (MAGUK family) | Homo sapiens |
| 7184 | heat shock protein 90kDa beta (Grp94), member 1 | Homo sapiens |
| 6016 | Ras-like without CAAX 1 | Homo sapiens |
| 1416 | crystallin, beta B2 pseudogene 1 | Homo sapiens |
| 56983 | KTEL (Lys-Tyr-Glu-Leu) containing 1 | Homo sapiens |
| 25998 | inhibitor of Bruton agammaglobulinemia tyrosine kinase | Homo sapiens |
| 10776 | cAMP-regulated phosphoprotein 19 pseudogene; cAMP-regulated phosphoprotein, 19kDa | Homo sapiens |
| 29893 | PSMC3 interacting protein | Homo sapiens |
| 8724 | sorting nexin 3 | Homo sapiens |
| 51319 | arginine/serine-rich coiled-coil 1 | Homo sapiens |
| 7088 | similar to transducin-like enhancer of split 1 (E(sp1) homolog, Drosophila); transducin-like enhancer of split 1 (E(sp1) homolog, Drosophila) | Homo sapiens |
| 641977 | septin 13 | Homo sapiens |
| 55848 | chromosome 9 open reading frame 46 | Homo sapiens |
| 221710 | hypothetical protein LOC221710 | Homo sapiens |
| 57704 | glucosidase, beta (bile acid) 2 | Homo sapiens |
| 4026 | LIM domain containing preferred translocation partner in lipoma | Homo sapiens |
| 29904 | eukaryotic elongation factor-2 kinase | Homo sapiens |
| 1939 | ligatin | Homo sapiens |
| 5228 | placental growth factor | Homo sapiens |
| 56005 | chromosome 19 open reading frame 10 | Homo sapiens |
| 81573 | ankyrin repeat domain 13C | Homo sapiens |
| 9097 | ubiquitin specific peptidase 14 (tRNA-guanine transglycosylase) | Homo sapiens |
| 55971 | BAI1-associated protein 2-like 1 | Homo sapiens |
| 51571 | family with sequence similarity 49, member B | Homo sapiens |
| 342357 | zinc finger with KRAB and SCAN domains 2 | Homo sapiens |
| 283578 | transmembrane emp24 protein transport domain containing 8 | Homo sapiens |
| 5298 | phosphatidylinositol 4-kinase, catalytic, beta | Homo sapiens |
| 513 | ATP synthase, H+ transporting, mitochondrial F1 complex, delta subunit | Homo sapiens |
| 5810 | RAD1 homolog (S. pombe) | Homo sapiens |
| 9909 | DENN/MADD domain containing 4B | Homo sapiens |
| 904 | cyclin T1 | Homo sapiens |
| 126626 | GA binding protein transcription factor, beta subunit 2 | Homo sapiens |
| 728340 | general transcription factor IIH, polypeptide 2, 44kDa; general transcription factor IIH, polypeptide 2C; general transcription factor IIH, polypeptide 2B; general transcription factor IIH, polypeptide 2D | Homo sapiens |
| 653238 | general transcription factor IIH, polypeptide 2, 44kDa; general transcription factor IIH, polypeptide 2C; general transcription factor IIH, polypeptide 2B; general transcription factor IIH, polypeptide 2D | Homo sapiens |
| 2966 | general transcription factor IIH, polypeptide 2, 44kDa; general transcription factor IIH, polypeptide 2C; general transcription factor IIH, polypeptide 2B; general transcription factor IIH, polypeptide 2D | Homo sapiens |
| 730394 | general transcription factor IIH, polypeptide 2, 44kDa; general transcription factor IIH, polypeptide 2C; general transcription factor IIH, polypeptide 2B; general transcription factor IIH, polypeptide 2D | Homo sapiens |
| 55076 | transmembrane protein 45A | Homo sapiens |
| 8546 | adaptor-related protein complex 3, beta 1 subunit | Homo sapiens |
| 23466 | chromobox homolog 6 | Homo sapiens |
| 55781 | RIO kinase 2 (yeast) | Homo sapiens |
| 539 | ATP synthase, H+ transporting, mitochondrial F1 complex, O subunit | Homo sapiens |
| 55190 | nudix (nucleoside diphosphate linked moiety X)-type motif 11 | Homo sapiens |
| 22846 | vasohibin 1 | Homo sapiens |
| 7019 | transcription factor A, mitochondrial | Homo sapiens |
| 4218 | RAB8A, member RAS oncogene family | Homo sapiens |
| 2562 | gamma-aminobutyric acid (GABA) A receptor, beta 3 | Homo sapiens |
| 9528 | transmembrane protein 59 | Homo sapiens |
| 26147 | PHD finger protein 19 | Homo sapiens |
| 2107 | eukaryotic translation termination factor 1 | Homo sapiens |
| 1314 | coatomer protein complex, subunit alpha | Homo sapiens |
| 90135 | BTB (POZ) domain containing 6 | Homo sapiens |
| 10200 | M-phase phosphoprotein 6 | Homo sapiens |
| 4191 | malate dehydrogenase 2, NAD (mitochondrial) | Homo sapiens |
| 23304 | ubiquitin protein ligase E3 component n-recognin 2 | Homo sapiens |
| 55862 | enoyl Coenzyme A hydratase domain containing 1 | Homo sapiens |
| 84681 | histidine triad nucleotide binding protein 2 | Homo sapiens |
| 64860 | armadillo repeat containing, X-linked 5 | Homo sapiens |
| 390980 | zinc finger protein 805 | Homo sapiens |

| | | |
|---|---|---|
| 55857 | non-protein coding RNA 153 | Homo sapiens |
| 134492 | NudC domain containing 2 | Homo sapiens |
| 26090 | abhydrolase domain containing 12 | Homo sapiens |
| 25921 | zinc finger, DHHC-type containing 5 | Homo sapiens |
| 8914 | timeless homolog (Drosophila) | Homo sapiens |
| 5303 | protein (peptidylprolyl cis/trans isomerase) NIMA-interacting, 4 (parvulin) | Homo sapiens |
| 6311 | ataxin 2 | Homo sapiens |
| 11112 | 3-hydroxyisobutyrate dehydrogenase | Homo sapiens |
| 55333 | synaptojanin 2 binding protein | Homo sapiens |
| 283431 | growth arrest-specific 2 like 3 | Homo sapiens |
| 11217 | A kinase (PRKA) anchor protein 2; paralemmin 2; PALM2-AKAP2 readthrough transcript | Homo sapiens |
| 445815 | A kinase (PRKA) anchor protein 2; paralemmin 2; PALM2-AKAP2 readthrough transcript | Homo sapiens |
| 65005 | mitochondrial ribosomal protein L9 | Homo sapiens |
| 51377 | ubiquitin carboxyl-terminal hydrolase L5 | Homo sapiens |
| 23192 | ATG4 autophagy related 4 homolog B (S. cerevisiae) | Homo sapiens |
| 84247 | leucine zipper, down-regulated in cancer 1-like | Homo sapiens |
| 55166 | centromere protein Q | Homo sapiens |
| 58516 | similar to hCG2020539; family with sequence similarity 60, member A; similar to family with sequence similarity 60, member A | Homo sapiens |
| 283927 | nudix (nucleoside diphosphate linked moiety X)-type motif 7 | Homo sapiens |
| 28973 | mitochondrial ribosomal protein S18B | Homo sapiens |
| 90874 | hypothetical LOC100130667; zinc finger protein 697 | Homo sapiens |
| 10605 | poly(A) binding protein interacting protein 1; similar to poly(A) binding protein interacting protein 1 | Homo sapiens |
| 57696 | DEAD (Asp-Glu-Ala-Asp) box polypeptide 55 | Homo sapiens |
| 65057 | adrenocortical dysplasia homolog (mouse) | Homo sapiens |
| 83394 | PITPNM family member 3 | Homo sapiens |
| 1513 | cathepsin K | Homo sapiens |
| 55607 | protein phosphatase 1, regulatory (inhibitor) subunit 9A | Homo sapiens |
| 6817 | sulfotransferase family, cytosolic, 1A, phenol-preferring, member 1 | Homo sapiens |
| 10928 | hypothetical LOC100129773; ralA binding protein 1 | Homo sapiens |
| 8301 | phosphatidylinositol binding clathrin assembly protein | Homo sapiens |
| 131474 | coiled-coil-helix-coiled-coil-helix domain containing 4 | Homo sapiens |
| 6385 | syndecan 4 | Homo sapiens |
| 2308 | forkhead box O1 | Homo sapiens |
| 23423 | transmembrane emp24 protein transport domain containing 3 | Homo sapiens |
| 29990 | paired immunoglobin-like type 2 receptor beta | Homo sapiens |
| 214 | hypothetical protein LOC100133690; activated leukocyte cell adhesion molecule | Homo sapiens |
| 6659 | SRY (sex determining region Y)-box 4 | Homo sapiens |
| 9284 | nuclear pore complex interacting protein | Homo sapiens |
| 159 | adenylosuccinate synthase | Homo sapiens |
| 22909 | myotubularin related protein 15 | Homo sapiens |
| 51143 | dynein, cytoplasmic 1, light intermediate chain 1 | Homo sapiens |
| 175 | aspartylglucosaminidase | Homo sapiens |
| 196394 | antagonist of mitotic exit network 1 homolog (S. cerevisiae) | Homo sapiens |
| 29 | active BCR-related gene | Homo sapiens |
| 6932 | transcription factor 7 (T-cell specific, HMG-box) | Homo sapiens |
| 57480 | pleckstrin homology domain containing, family G (with RhoGef domain) member 1 | Homo sapiens |
| 84888 | signal peptide peptidase-like 2A | Homo sapiens |
| 55739 | carbohydrate kinase domain containing | Homo sapiens |
| 3661 | interferon regulatory factor 3 | Homo sapiens |
| 158747 | motile sperm domain containing 2 | Homo sapiens |
| 157378 | transmembrane protein 65 | Homo sapiens |
| 100287932 | similar to translocase of inner mitochondrial membrane 23 (yeast) homolog | Homo sapiens |
| 3005 | H1 histone family, member 0 | Homo sapiens |
| 51804 | SIX homeobox 4 | Homo sapiens |
| 9931 | helicase with zinc finger | Homo sapiens |
| 9394 | heparan sulfate 6-O-sulfotransferase 1 | Homo sapiens |
| 55752 | septin 11 | Homo sapiens |
| 25934 | nipsnap homolog 3A (C. elegans) | Homo sapiens |
| 6734 | signal recognition particle receptor (docking protein) | Homo sapiens |
| 10645 | calcium/calmodulin-dependent protein kinase kinase 2, beta | Homo sapiens |
| 118980 | sideroflexin 2 | Homo sapiens |
| 23177 | centrosomal protein 68kDa | Homo sapiens |
| 8545 | CGG triplet repeat binding protein 1 | Homo sapiens |
| 8325 | frizzled homolog 8 (Drosophila) | Homo sapiens |
| 30968 | stomatin (EPB72)-like 2 | Homo sapiens |
| 26205 | glucocorticoid modulatory element binding protein 2 | Homo sapiens |
| 155061 | zinc finger protein 746 | Homo sapiens |
| 55734 | zinc finger protein 64 homolog (mouse) | Homo sapiens |
| 23456 | ATP-binding cassette, sub-family B (MDR/TAP), member 10 | Homo sapiens |
| 115294 | protein-L-isoaspartate (D-aspartate) O-methyltransferase domain containing 1 | Homo sapiens |
| 1534 | cytochrome b-561 | Homo sapiens |
| 2177 | Fanconi anemia, complementation group D2 | Homo sapiens |
| 10988 | methionyl aminopeptidase 2 | Homo sapiens |
| 6182 | mitochondrial ribosomal protein L12 | Homo sapiens |
| 53344 | cysteine-rich hydrophobic domain 1 | Homo sapiens |

| | | |
|---|---|---|
| 4267 | CD99 molecule | Homo sapiens |
| 6883 | TAF12 RNA polymerase II, TATA box binding protein (TBP)-associated factor, 20kDa | Homo sapiens |
| 7175 | translocated promoter region (to activated MET oncogene) | Homo sapiens |
| 11043 | midline 2 | Homo sapiens |
| 26048 | zinc finger protein 500 | Homo sapiens |
| 23512 | suppressor of zeste 12 homolog (Drosophila) | Homo sapiens |
| 10838 | zinc finger protein 275 | Homo sapiens |
| 28989 | methyltransferase like 11A | Homo sapiens |
| 55015 | PRP39 pre-mRNA processing factor 39 homolog (S. cerevisiae) | Homo sapiens |
| 23338 | PHD finger protein 15 | Homo sapiens |
| 2786 | guanine nucleotide binding protein (G protein), gamma 4 | Homo sapiens |
| 150928 | prothymosin, alpha pseudogene 5 | Homo sapiens |
| 6136 | ribosomal protein L12 pseudogene 2; ribosomal protein L12 pseudogene 32; ribosomal protein L12 pseudogene 35; ribosomal protein L12 pseudogene 19; ribosomal protein L12 pseudogene 6; ribosomal protein L12; ribosomal protein L12 pseudogene 14 | Homo sapiens |
| 5993 | regulatory factor X, 5 (influences HLA class II expression) | Homo sapiens |
| 10591 | chromosome 6 open reading frame 108 | Homo sapiens |
| 3216 | homeobox B6 | Homo sapiens |
| 29078 | NADH dehydrogenase (ubiquinone) 1 alpha subcomplex, assembly factor 4; similar to HSPC125 | Homo sapiens |
| 9183 | ZW10, kinetochore associated, homolog (Drosophila) | Homo sapiens |
| 4437 | mutS homolog 3 (E. coli) | Homo sapiens |
| 10128 | leucine-rich PPR-motif containing | Homo sapiens |
| 4719 | NADH dehydrogenase (ubiquinone) Fe-S protein 1, 75kDa (NADH-coenzyme Q reductase) | Homo sapiens |
| 80010 | RMI1, RecQ mediated genome instability 1, homolog (S. cerevisiae) | Homo sapiens |
| 259282 | biorientation of chromosomes in cell division 1-like | Homo sapiens |
| 219 | aldehyde dehydrogenase 1 family, member B1 | Homo sapiens |
| 389834 | ankyrin repeat domain 57 pseudogene | Homo sapiens |
| 2297 | forkhead box D1 | Homo sapiens |
| 6617 | small nuclear RNA activating complex, polypeptide 1, 43kDa | Homo sapiens |
| 10617 | STAM binding protein | Homo sapiens |
| 23189 | KN motif and ankyrin repeat domains 1; similar to ankyrin repeat domain protein 15 isoform b | Homo sapiens |
| 65095 | KRI1 homolog (S. cerevisiae) | Homo sapiens |
| 85015 | ubiquitin specific peptidase 45 | Homo sapiens |
| 29855 | ubinuclein 1 | Homo sapiens |
| 55958 | kelch-like 9 (Drosophila) | Homo sapiens |
| 94274 | protein phosphatase 1, regulatory (inhibitor) subunit 14A | Homo sapiens |
| 29789 | Obg-like ATPase 1 | Homo sapiens |
| 1808 | dihydropyrimidinase-like 2 | Homo sapiens |
| 3189 | heterogeneous nuclear ribonucleoprotein H3 (2H9) | Homo sapiens |
| 57721 | methyltransferase like 14 | Homo sapiens |
| 4820 | natural killer-tumor recognition sequence | Homo sapiens |
| 29902 | chromosome 12 open reading frame 24 | Homo sapiens |
| 5066 | peptidylglycine alpha-amidating monooxygenase | Homo sapiens |
| 79692 | zinc finger protein 322A | Homo sapiens |
| 93129 | ORAI calcium release-activated calcium modulator 3 | Homo sapiens |
| 55048 | vacuolar protein sorting 37 homolog C (S. cerevisiae) | Homo sapiens |
| 1871 | E2F transcription factor 3 | Homo sapiens |
| 51547 | sirtuin (silent mating type information regulation 2 homolog) 7 (S. cerevisiae) | Homo sapiens |
| 3927 | LIM and SH3 protein 1 | Homo sapiens |
| 10638 | S-phase response (cyclin related) | Homo sapiens |
| 79618 | homeobox containing 1 | Homo sapiens |
| 30815 | ST6 (alpha-N-acetyl-neuraminyl-2,3-beta-galactosyl-1,3)-N-acetylgalactosaminide alpha-2,6-sialyltransferase 6 | Homo sapiens |
| 11079 | RER1 retention in endoplasmic reticulum 1 homolog (S. cerevisiae) | Homo sapiens |
| 29035 | chromosome 16 open reading frame 72 | Homo sapiens |
| 10342 | TRK-fused gene | Homo sapiens |
| 5695 | proteasome (prosome, macropain) subunit, beta type, 7 | Homo sapiens |
| 4832 | non-metastatic cells 3, protein expressed in | Homo sapiens |
| 51514 | denticleless homolog (Drosophila) | Homo sapiens |
| 54108 | chromatin accessibility complex 1 | Homo sapiens |
| 11320 | mannosyl (alpha-1,3-)-glycoprotein beta-1,4-N-acetylglucosaminyltransferase, isozyme A | Homo sapiens |
| 6721 | sterol regulatory element binding transcription factor 2 | Homo sapiens |
| 253832 | zinc finger, DHHC-type containing 20 | Homo sapiens |
| 23522 | MYST histone acetyltransferase (monocytic leukemia) 4 | Homo sapiens |
| 211 | aminolevulinate, delta-, synthase 1 | Homo sapiens |
| 29088 | mitochondrial ribosomal protein L15 | Homo sapiens |
| 7627 | zinc finger protein 75a | Homo sapiens |
| 147991 | dpy-19-like 3 (C. elegans) | Homo sapiens |
| 51035 | UBX domain protein 1 | Homo sapiens |
| 6282 | S100 calcium binding protein A11; S100 calcium binding protein A11 pseudogene | Homo sapiens |
| 1512 | cathepsin H | Homo sapiens |
| 54962 | TIMELESS interacting protein | Homo sapiens |
| 1647 | growth arrest and DNA-damage-inducible, alpha | Homo sapiens |
| 9169 | splicing factor, arginine/serine-rich 2, interacting protein | Homo sapiens |
| 81559 | tripartite motif-containing 11 | Homo sapiens |

| | | |
|---|---|---|
| 10427 | SEC24 family, member B (S. cerevisiae) | Homo sapiens |
| 727910 | TLC domain containing 2 | Homo sapiens |
| 283131 | non-protein coding RNA 84 | Homo sapiens |
| 6238 | ribosome binding protein 1 homolog 180kDa (dog) | Homo sapiens |
| 55747 | family with sequence similarity 21, member B; family with sequence similarity 21, member A | Homo sapiens |
| 387680 | family with sequence similarity 21, member B; family with sequence similarity 21, member A | Homo sapiens |
| 64426 | suppressor of defective silencing 3 homolog (S. cerevisiae) | Homo sapiens |
| 1843 | dual specificity phosphatase 1 | Homo sapiens |
| 23132 | RAD54-like 2 (S. cerevisiae) | Homo sapiens |
| 23511 | nucleoporin 188kDa | Homo sapiens |
| 10558 | serine palmitoyltransferase, long chain base subunit 1 | Homo sapiens |
| 55750 | acylglycerol kinase | Homo sapiens |
| 23327 | neural precursor cell expressed, developmentally down-regulated 4-like | Homo sapiens |
| 94081 | sideroflexin 1 | Homo sapiens |
| 144245 | asparagine-linked glycosylation 10, alpha-1,2-glucosyltransferase homolog B (yeast) | Homo sapiens |
| 390066 | olfactory receptor, family 52, subfamily D, member 1 | Homo sapiens |
| 1390 | cAMP responsive element modulator | Homo sapiens |
| 9555 | H2A histone family, member Y | Homo sapiens |
| 10643 | insulin-like growth factor 2 mRNA binding protein 3 | Homo sapiens |
| 9917 | family with sequence similarity 20, member B | Homo sapiens |
| 26010 | spermatogenesis associated, serine-rich 2-like | Homo sapiens |
| 51079 | NADH dehydrogenase (ubiquinone) 1 alpha subcomplex, 13 | Homo sapiens |
| 10735 | stromal antigen 2 | Homo sapiens |
| 51280 | golgi membrane protein 1 | Homo sapiens |
| 4215 | mitogen-activated protein kinase kinase kinase 3 | Homo sapiens |
| 170622 | COMM domain containing 6 | Homo sapiens |
| 27107 | zinc finger and BTB domain containing 11 | Homo sapiens |
| 10038 | poly (ADP-ribose) polymerase 2 | Homo sapiens |
| 10412 | hypothetical gene supported by NM_014886; TGF beta-inducible nuclear protein 1; similar to TGF beta-inducible nuclear protein 1 | Homo sapiens |
| 54737 | M-phase phosphoprotein 8 | Homo sapiens |
| 90806 | angel homolog 2 (Drosophila) | Homo sapiens |
| 64750 | SMAD specific E3 ubiquitin protein ligase 2 | Homo sapiens |
| 7871 | sarcolemma associated protein | Homo sapiens |
| 1977 | eukaryotic translation initiation factor 4E; similar to hCG1777996; similar to eukaryotic translation initiation factor 4E | Homo sapiens |
| 2194 | fatty acid synthase | Homo sapiens |
| 4086 | SMAD family member 1 | Homo sapiens |
| 55699 | isoleucyl-tRNA synthetase 2, mitochondrial | Homo sapiens |
| 56947 | mitochondrial fission factor | Homo sapiens |
| 6729 | similar to signal recognition particle 54kDa; signal recognition particle 54kDa | Homo sapiens |
| 7155 | topoisomerase (DNA) II beta 180kDa | Homo sapiens |
| 57092 | PEST proteolytic signal containing nuclear protein | Homo sapiens |
| 1213 | clathrin, heavy chain (Hc) | Homo sapiens |
| 8891 | eukaryotic translation initiation factor 2B, subunit 3 gamma, 58kDa | Homo sapiens |
| 375 | ADP-ribosylation factor 1 | Homo sapiens |
| 84268 | RPA interacting protein | Homo sapiens |
| 23035 | PH domain and leucine rich repeat protein phosphatase 2 | Homo sapiens |
| 2131 | exostoses (multiple) 1 | Homo sapiens |
| 388695 | LysM, putative peptidoglycan-binding, domain containing 1 | Homo sapiens |
| 146909 | kinesin family member 18B | Homo sapiens |
| 23648 | single stranded DNA binding protein 3; hypothetical LOC100131851 | Homo sapiens |
| 54492 | neuralized homolog 1B (Drosophila) | Homo sapiens |
| 91647 | ATP synthase mitochondrial F1 complex assembly factor 2 | Homo sapiens |
| 7187 | TNF receptor-associated factor 3 | Homo sapiens |
| 100271836 | SMG1 homolog, phosphatidylinositol 3-kinase-related kinase pseudogene | Homo sapiens |
| 49854 | zinc finger protein 295 | Homo sapiens |
| 80218 | N-acetyltransferase 13 (GCN5-related) | Homo sapiens |
| 23478 | SEC11 homolog A (S. cerevisiae) | Homo sapiens |
| 91752 | zinc finger protein 804A | Homo sapiens |
| 10540 | dynactin 2 (p50) | Homo sapiens |
| 3831 | kinesin light chain 1 | Homo sapiens |
| 8434 | reversion-inducing-cysteine-rich protein with kazal motifs | Homo sapiens |
| 5686 | proteasome (prosome, macropain) subunit, alpha type, 5 | Homo sapiens |
| 55147 | RNA binding motif protein 23 | Homo sapiens |
| 10560 | solute carrier family 19 (thiamine transporter), member 2 | Homo sapiens |
| 8976 | Wiskott-Aldrich syndrome-like | Homo sapiens |
| 3313 | heat shock 70kDa protein 9 (mortalin) | Homo sapiens |
| 201266 | solute carrier family 39 (metal ion transporter), member 11 | Homo sapiens |
| 2665 | GDP dissociation inhibitor 2 | Homo sapiens |
| 9444 | quaking homolog, KH domain RNA binding (mouse) | Homo sapiens |
| 57507 | zinc finger protein 608 | Homo sapiens |
| 25961 | nudix (nucleoside diphosphate linked moiety X)-type motif 13 | Homo sapiens |
| 2261 | fibroblast growth factor receptor 3 | Homo sapiens |
| 221322 | chromosome 6 open reading frame 170 | Homo sapiens |

| | | |
|---|---|---|
| 100129361 | similar to mCG115122 | Homo sapiens |
| 54884 | retinol saturase (all-trans-retinol 13,14-reductase) | Homo sapiens |
| 23479 | iron-sulfur cluster scaffold homolog (E. coli) | Homo sapiens |
| 9197 | solute carrier family 33 (acetyl-CoA transporter), member 1 | Homo sapiens |
| 10957 | proline-rich nuclear receptor coactivator 1 | Homo sapiens |
| 64963 | mitochondrial ribosomal protein S11 | Homo sapiens |
| 3149 | similar to high mobility group box 3; high-mobility group box 3 | Homo sapiens |
| 9693 | Rap guanine nucleotide exchange factor (GEF) 2; similar to RAPGEF2 protein | Homo sapiens |
| 51299 | neuritin 1 | Homo sapiens |
| 23279 | nucleoporin 160kDa | Homo sapiens |
| 23787 | mitochondrial carrier homolog 1 (C. elegans) | Homo sapiens |
| 10956 | osteosarcoma amplified 9, endoplasmic reticulum associated protein | Homo sapiens |
| 79980 | DSN1, MIND kinetochore complex component, homolog (S. cerevisiae) | Homo sapiens |
| 5128 | PCTAIRE protein kinase 2 | Homo sapiens |
| 4093 | SMAD family member 9 | Homo sapiens |
| 650655 | ATP-binding cassette, sub-family A (ABC1), member 17 (pseudogene) | Homo sapiens |
| 55366 | leucine-rich repeat-containing G protein-coupled receptor 4 | Homo sapiens |
| 57617 | vacuolar protein sorting 18 homolog (S. cerevisiae) | Homo sapiens |
| 2850 | G protein-coupled receptor 27 | Homo sapiens |
| 119016 | ArfGAP with GTPase domain, ankyrin repeat and PH domain 4; ArfGAP with GTPase domain, ankyrin repeat and PH domain 6 | Homo sapiens |
| 26148 | chromosome 10 open reading frame 12 | Homo sapiens |
| 6711 | spectrin, beta, non-erythrocytic 1 | Homo sapiens |
| 4836 | N-myristoyltransferase 1 | Homo sapiens |
| 9231 | discs, large homolog 5 (Drosophila) | Homo sapiens |
| 55294 | F-box and WD repeat domain containing 7 | Homo sapiens |
| 55038 | cell division cycle associated 4 | Homo sapiens |
| 1677 | DNA fragmentation factor, 40kDa, beta polypeptide (caspase-activated DNase) | Homo sapiens |
| 84164 | activating signal cointegrator 1 complex subunit 2 | Homo sapiens |
| 4236 | microfibrillar-associated protein 1 | Homo sapiens |
| 11130 | ZW10 interactor | Homo sapiens |
| 401207 | hypothetical protein LOC401207 | Homo sapiens |
| 84138 | solute carrier family 7, member 6 opposite strand | Homo sapiens |
| 7402 | utrophin | Homo sapiens |
| 3033 | hydroxyacyl-Coenzyme A dehydrogenase | Homo sapiens |
| 100128737 | hypothetical LOC100128737 | Homo sapiens |
| 91775 | family with sequence similarity 55, member C | Homo sapiens |
| 83636 | chromosome 19 open reading frame 12 | Homo sapiens |
| 7268 | tetratricopeptide repeat domain 4 | Homo sapiens |
| 83642 | selenoprotein O | Homo sapiens |
| 2697 | gap junction protein, alpha 1, 43kDa | Homo sapiens |
| 10569 | SLU7 splicing factor homolog (S. cerevisiae) | Homo sapiens |
| 1660 | DEAH (Asp-Glu-Ala-His) box polypeptide 9 | Homo sapiens |
| 23386 | NudC domain containing 3 | Homo sapiens |
| 93624 | transcriptional adaptor 2 (ADA2 homolog, yeast)-beta | Homo sapiens |
| 6509 | solute carrier family 1 (glutamate/neutral amino acid transporter), member 4 | Homo sapiens |
| 65108 | MARCKS-like 1 | Homo sapiens |
| 3459 | interferon gamma receptor 1 | Homo sapiens |
| 57665 | retinol dehydrogenase 14 (all-trans/9-cis/11-cis); 5'-nucleotidase, cytosolic IB | Homo sapiens |
| 3455 | interferon (alpha, beta and omega) receptor 2 | Homo sapiens |
| 57335 | zinc finger protein 286A | Homo sapiens |
| 10193 | ring finger protein 41 | Homo sapiens |
| 23395 | leucyl-tRNA synthetase 2, mitochondrial | Homo sapiens |
| 84910 | transmembrane protein 87B | Homo sapiens |
| 29880 | asparagine-linked glycosylation 5, dolichyl-phosphate beta-glucosyltransferase homolog (S. cerevisiae) | Homo sapiens |
| 441094 | hypothetical LOC441094 | Homo sapiens |
| 23764 | v-maf musculoaponeurotic fibrosarcoma oncogene homolog F (avian) | Homo sapiens |
| 80174 | DBF4 homolog B (S. cerevisiae) | Homo sapiens |
| 9589 | Wilms tumor 1 associated protein | Homo sapiens |
| 11041 | UDP-GlcNAc:betaGal beta-1,3-N-acetylglucosaminyltransferase 1; UDP-GlcNAc:betaGal beta-1,3-N-acetylglucosaminyltransferase 2 | Homo sapiens |
| 3200 | homeobox A3 | Homo sapiens |
| 729680 | hypothetical protein LOC729680 | Homo sapiens |
| 345757 | family with sequence similarity 174, member A | Homo sapiens |
| 55120 | Fanconi anemia, complementation group L | Homo sapiens |
| 22911 | WD repeat domain 47 | Homo sapiens |
| 10140 | transducer of ERBB2, 1 | Homo sapiens |
| 1903 | sphingosine-1-phosphate receptor 3 | Homo sapiens |
| 56255 | thioredoxin-related transmembrane protein 4 | Homo sapiens |
| 51567 | TRAF and TNF receptor associated protein | Homo sapiens |
| 10482 | nuclear RNA export factor 1 | Homo sapiens |
| 81873 | actin related protein 2/3 complex, subunit 5-like | Homo sapiens |
| 1123 | chimerin (chimaerin) 1 | Homo sapiens |
| 254863 | chromosome 17 open reading frame 61 | Homo sapiens |
| 728689 | eukaryotic translation initiation factor 3, subunit C-like | Homo sapiens |
| 118424 | ubiquitin-conjugating enzyme E2, J2 (UBC6 homolog, yeast) | Homo sapiens |

| | | |
|---|---|---|
| 10933 | mortality factor 4; mortality factor 4 like 1 | Homo sapiens |
| 10934 | mortality factor 4; mortality factor 4 like 1 | Homo sapiens |
| 92856 | IMP4, U3 small nucleolar ribonucleoprotein, homolog (yeast) | Homo sapiens |
| 23484 | leptin receptor overlapping transcript-like 1 | Homo sapiens |
| 55884 | WD repeat and SOCS box-containing 2 | Homo sapiens |
| 4694 | NADH dehydrogenase (ubiquinone) 1 alpha subcomplex, 1, 7.5kDa | Homo sapiens |
| 27345 | potassium large conductance calcium-activated channel, subfamily M, beta member 4 | Homo sapiens |
| 4713 | NADH dehydrogenase (ubiquinone) 1 beta subcomplex, 7, 18kDa | Homo sapiens |
| 283450 | chromosome 12 open reading frame 51 | Homo sapiens |
| 51759 | chromosome 9 open reading frame 78 | Homo sapiens |
| 2868 | G protein-coupled receptor kinase 4 | Homo sapiens |
| 9543 | immunoglobulin superfamily, DCC subclass, member 3 | Homo sapiens |
| 100288778 | similar to WAS protein family homolog 1 | Homo sapiens |
| 2802 | golgi autoantigen, golgin subfamily a, 3 | Homo sapiens |
| 57654 | KIAA1530 | Homo sapiens |
| 340591 | carbonic anhydrase VB pseudogene | Homo sapiens |
| 1000 | cadherin 2, type 1, N-cadherin (neuronal) | Homo sapiens |
| 123811 | chromosome 16 open reading frame 63 | Homo sapiens |
| 1211 | clathrin, light chain (Lca) | Homo sapiens |
| 7267 | tetratricopeptide repeat domain 3; tetratricopeptide repeat domain 3-like | Homo sapiens |
| 54665 | round spermatid basic protein 1 | Homo sapiens |
| 56943 | enhancer of yellow 2 homolog (Drosophila) | Homo sapiens |
| 8658 | tankyrase, TRF1-interacting ankyrin-related ADP-ribose polymerase | Homo sapiens |
| 64770 | coiled-coil domain containing 14 | Homo sapiens |
| 283237 | tetratricopeptide repeat domain 9C | Homo sapiens |
| 27042 | chromosome 1 open reading frame 107 | Homo sapiens |
| 65055 | receptor accessory protein 1 | Homo sapiens |
| 91748 | chromosome 14 open reading frame 43 | Homo sapiens |
| 3632 | inositol polyphosphate-5-phosphatase, 40kDa | Homo sapiens |
| 84061 | magnesium transporter 1 | Homo sapiens |
| 5989 | regulatory factor X, 1 (influences HLA class II expression) | Homo sapiens |
| 221443 | chromosome 6 open reading frame 130 | Homo sapiens |
| 29087 | thymocyte nuclear protein 1 | Homo sapiens |
| 83985 | spinster homolog 1 (Drosophila) | Homo sapiens |
| 5901 | RAN, member RAS oncogene family | Homo sapiens |
| 79630 | chromosome 1 open reading frame 54 | Homo sapiens |
| 11157 | LSM6 homolog, U6 small nuclear RNA associated (S. cerevisiae) | Homo sapiens |
| 51181 | dicarbonyl/L-xylulose reductase | Homo sapiens |
| 56311 | ankyrin repeat domain 7 | Homo sapiens |
| 5930 | retinoblastoma binding protein 6 | Homo sapiens |
| 3190 | heterogeneous nuclear ribonucleoprotein K; similar to heterogeneous nuclear ribonucleoprotein K | Homo sapiens |
| 79770 | thioredoxin domain containing 15 | Homo sapiens |
| 51510 | chromatin modifying protein 5 | Homo sapiens |
| 8131 | chromosome 16 open reading frame 35 | Homo sapiens |
| 51645 | peptidylprolyl isomerase (cyclophilin)-like 1 | Homo sapiens |
| 4238 | microfibrillar-associated protein 3 | Homo sapiens |
| 374395 | transmembrane protein 179B | Homo sapiens |
| 55753 | oxoglutarate dehydrogenase-like | Homo sapiens |
| 9783 | regulating synaptic membrane exocytosis 3 | Homo sapiens |
| 5245 | prohibitin | Homo sapiens |
| 9948 | WD repeat domain 1 | Homo sapiens |
| 4628 | myosin, heavy chain 10, non-muscle | Homo sapiens |
| 9702 | centrosomal protein 57kDa | Homo sapiens |
| 7571 | zinc finger protein 23 (KOX 16) | Homo sapiens |
| 284273 | zinc binding alcohol dehydrogenase domain containing 2 | Homo sapiens |
| 25796 | 6-phosphogluconolactonase | Homo sapiens |
| 55681 | SCY1-like 2 (S. cerevisiae) | Homo sapiens |
| 653252 | translocase of inner mitochondrial membrane 23 homolog (yeast); translocase of inner mitochondrial membrane 23 homolog B (yeast) | Homo sapiens |
| 57646 | ubiquitin specific peptidase 28 | Homo sapiens |
| 23199 | KIAA0182 | Homo sapiens |
| 55142 | HAUS augmin-like complex, subunit 2 | Homo sapiens |
| 26121 | PRP31 pre-mRNA processing factor 31 homolog (S. cerevisiae) | Homo sapiens |
| 8562 | density-regulated protein | Homo sapiens |
| 348801 | leukemia NUP98 fusion partner 1 | Homo sapiens |
| 5557 | primase, DNA, polypeptide 1 (49kDa) | Homo sapiens |
| 10857 | progesterone receptor membrane component 1 | Homo sapiens |
| 6235 | ribosomal protein S29 pseudogene 11; ribosomal protein S29 pseudogene 16; ribosomal protein S29 pseudogene 17; ribosomal protein S29 pseudogene 3; ribosomal protein S29 pseudogene 9; ribosomal protein S29 | Homo sapiens |
| 57234 | family with sequence similarity 91, member A2 | Homo sapiens |
| 55791 | chromosome 1 open reading frame 103 | Homo sapiens |
| 29966 | striatin, calmodulin binding protein 3 | Homo sapiens |
| 122509 | interferon, alpha-inducible protein 27-like 1 | Homo sapiens |
| 1653 | DEAD (Asp-Glu-Ala-Asp) box polypeptide 1 | Homo sapiens |
| 51065 | ribosomal protein S27-like | Homo sapiens |

| | | |
|---|---|---|
| 51762 | RAB8B, member RAS oncogene family | Homo sapiens |
| 728411 | glucuronidase, beta-like 2; glucuronidase, beta pseudogene | Homo sapiens |
| 375513 | glucuronidase, beta-like 2; glucuronidase, beta pseudogene | Homo sapiens |
| 23361 | zinc finger protein 629 | Homo sapiens |
| 3720 | jumonji, AT rich interactive domain 2 | Homo sapiens |
| 113115 | family with sequence similarity 54, member A | Homo sapiens |
| 9168 | thymosin beta 10 | Homo sapiens |
| 2879 | glutathione peroxidase 4 (phospholipid hydroperoxidase) | Homo sapiens |
| 821 | calnexin | Homo sapiens |
| 25898 | ring finger and CHY zinc finger domain containing 1 | Homo sapiens |
| 10179 | RNA binding motif protein 7 | Homo sapiens |
| 7716 | vascular endothelial zinc finger 1 | Homo sapiens |
| 162 | adaptor-related protein complex 1, beta 1 subunit | Homo sapiens |
| 1017 | cyclin-dependent kinase 2 | Homo sapiens |
| 283377 | SPRY domain containing 4 | Homo sapiens |
| 26509 | myoferlin | Homo sapiens |
| 2332 | fragile X mental retardation 1 | Homo sapiens |
| 25777 | unc-84 homolog B (C. elegans) | Homo sapiens |
| 10097 | ARP2 actin-related protein 2 homolog (yeast) | Homo sapiens |
| 7546 | Zic family member 2 (odd-paired homolog, Drosophila) | Homo sapiens |
| 388677 | Notch homolog 2 (Drosophila) N-terminal like | Homo sapiens |
| 2677 | gamma-glutamyl carboxylase | Homo sapiens |
| 401505 | translocase of outer mitochondrial membrane 5 homolog (yeast) | Homo sapiens |
| 55108 | BSD domain containing 1 | Homo sapiens |
| 2254 | fibroblast growth factor 9 (glia-activating factor) | Homo sapiens |
| 221272 | hypothetical LOC221272 | Homo sapiens |
| 26258 | pallidin homolog (mouse) | Homo sapiens |
| 6198 | ribosomal protein S6 kinase, 70kDa, polypeptide 1 | Homo sapiens |
| 152006 | ring finger protein 38 | Homo sapiens |
| 1604 | CD55 molecule, decay accelerating factor for complement (Cromer blood group) | Homo sapiens |
| 90488 | chromosome 12 open reading frame 23 | Homo sapiens |
| 26517 | translocase of inner mitochondrial membrane 13 homolog (yeast) | Homo sapiens |
| 23111 | spastic paraplegia 20 (Troyer syndrome) | Homo sapiens |
| 29901 | SAC3 domain containing 1 | Homo sapiens |
| 51528 | chromosome 14 open reading frame 100 | Homo sapiens |
| 221061 | family with sequence similarity 171, member A1 | Homo sapiens |
| 22921 | methionine sulfoxide reductase B2 | Homo sapiens |
| 11275 | kelch-like 2, Mayven (Drosophila) | Homo sapiens |
| 377 | ADP-ribosylation factor 3 | Homo sapiens |
| 4162 | melanoma cell adhesion molecule | Homo sapiens |
| 84881 | RNA pseudouridylate synthase domain containing 4 | Homo sapiens |
| 10217 | CTD (carboxy-terminal domain, RNA polymerase II, polypeptide A) small phosphatase-like | Homo sapiens |
| 83460 | transmembrane protein 93 | Homo sapiens |
| 132720 | chromosome 4 open reading frame 32 | Homo sapiens |
| 9779 | TBC1 domain family, member 5 | Homo sapiens |
| 22823 | metal response element binding transcription factor 2 | Homo sapiens |
| 990 | cell division cycle 6 homolog (S. cerevisiae) | Homo sapiens |
| 9361 | lon peptidase 1, mitochondrial | Homo sapiens |
| 11329 | serine/threonine kinase 38 | Homo sapiens |
| 4927 | nucleoporin 88kDa | Homo sapiens |
| 5868 | RAB5A, member RAS oncogene family | Homo sapiens |
| 10208 | ubiquitin specific peptidase like 1 | Homo sapiens |
| 4000 | lamin A/C | Homo sapiens |
| 729288 | zinc finger protein 286B | Homo sapiens |
| 26091 | hect domain and RLD 4 | Homo sapiens |
| 64786 | TBC1 domain family, member 15 | Homo sapiens |
| 56916 | SWI/SNF-related, matrix-associated actin-dependent regulator of chromatin, subfamily a, containing DEAD/H box 1 | Homo sapiens |
| 5108 | pericentriolar material 1 | Homo sapiens |
| 6510 | solute carrier family 1 (neutral amino acid transporter), member 5 | Homo sapiens |
| 11335 | similar to chromobox homolog 3; chromobox homolog 3 (HP1 gamma homolog, Drosophila) | Homo sapiens |
| 9341 | vesicle-associated membrane protein 3 (cellubrevin) | Homo sapiens |
| 284926 | hypothetical protein LOC284926 | Homo sapiens |
| 89890 | kelch repeat and BTB (POZ) domain containing 6 | Homo sapiens |
| 2339 | farnesyltransferase, CAAX box, alpha | Homo sapiens |
| 6748 | signal sequence receptor, delta (translocon-associated protein delta) | Homo sapiens |
| 51251 | 5'-nucleotidase, cytosolic III | Homo sapiens |
| 10611 | PDZ and LIM domain 5 | Homo sapiens |
| 89796 | neuron navigator 1 | Homo sapiens |
| 25830 | sulfotransferase family 4A, member 1 | Homo sapiens |
| 85352 | KIAA1644 | Homo sapiens |
| 5597 | mitogen-activated protein kinase 6 | Homo sapiens |
| 7385 | ubiquinol-cytochrome c reductase core protein II | Homo sapiens |
| 10062 | nuclear receptor subfamily 1, group H, member 3 | Homo sapiens |
| 100130219 | similar to hCG2041987 | Homo sapiens |
| 2050 | EPH receptor B4 | Homo sapiens |
| 10793 | zinc finger protein 273 | Homo sapiens |

| | | |
|---|---|---|
| 6139 | ribosomal protein L17 pseudogene 22; ribosomal protein L17 pseudogene 36; ribosomal protein L17 pseudogene 20; similar to ribosomal protein L17; ribosomal protein L17 pseudogene 33; ribosomal protein L17 pseudogene 34; ribosomal protein L17 pseudogene 9; ribosomal protein L17; ribosomal protein L17 pseudogene 18; ribosomal protein L17 pseudogene 7; ribosomal protein L17 pseudogene 39 | Homo sapiens |
| 2222 | farnesyl-diphosphate farnesyltransferase 1 | Homo sapiens |
| 11159 | RAB, member of RAS oncogene family-like 2A | Homo sapiens |
| 64328 | exportin 4 | Homo sapiens |
| 57587 | KIAA1430 | Homo sapiens |
| 163859 | chromosome 1 open reading frame 55 | Homo sapiens |
| 5978 | RE1-silencing transcription factor | Homo sapiens |
| 100288092 | hypothetical protein LOC100288092 | Homo sapiens |
| 100129550 | hypothetical LOC100129550 | Homo sapiens |
| 368 | ATP-binding cassette, sub-family C, member 6 pseudogene 2; ATP-binding cassette, sub-family C (CFTR/MRP), member 6 | Homo sapiens |
| 55182 | ring finger protein 220 | Homo sapiens |
| 23165 | nucleoporin 205kDa | Homo sapiens |
| 6727 | signal recognition particle 14kDa (homologous Alu RNA binding protein) pseudogene 1; signal recognition particle 14kDa (homologous Alu RNA binding protein) | Homo sapiens |
| 84790 | tubulin, alpha 1c | Homo sapiens |
| 9695 | ER degradation enhancer, mannosidase alpha-like 1 | Homo sapiens |
| 11224 | ribosomal protein L35; ribosomal protein L35 pseudogene 1; ribosomal protein L35 pseudogene 2 | Homo sapiens |
| 55233 | MOB1, Mps One Binder kinase activator-like 1B (yeast) | Homo sapiens |
| 10904 | bladder cancer associated protein | Homo sapiens |
| 1827 | regulator of calcineurin 1 | Homo sapiens |
| 664 | BCL2/adenovirus E1B 19kDa interacting protein 3 | Homo sapiens |
| 10613 | ER lipid raft associated 1 | Homo sapiens |
| 9525 | vacuolar protein sorting 4 homolog B (S. cerevisiae) | Homo sapiens |
| 1184 | chloride channel 5 | Homo sapiens |
| 10146 | GTPase activating protein (SH3 domain) binding protein 1 | Homo sapiens |
| 112942 | coiled-coil domain containing 104 | Homo sapiens |
| 112858 | TP53 regulating kinase | Homo sapiens |
| 23313 | chromosome 22 open reading frame 9 | Homo sapiens |
| 1829 | desmoglein 2 | Homo sapiens |
| 6196 | ribosomal protein S6 kinase, 90kDa, polypeptide 2; hypothetical LOC100127984 | Homo sapiens |
| 84100 | ADP-ribosylation factor-like 6 | Homo sapiens |
| 10944 | chromosome 11 open reading frame 58 pseudogene; chromosome 11 open reading frame 58 | Homo sapiens |
| 51077 | FCF1 small subunit (SSU) processome component homolog (S. cerevisiae) | Homo sapiens |
| 8665 | eukaryotic translation initiation factor 3, subunit F; similar to hCG2040283 | Homo sapiens |
| 54813 | kelch-like 28 (Drosophila) | Homo sapiens |
| 7386 | ubiquinol-cytochrome c reductase, Rieske iron-sulfur polypeptide-like 1; ubiquinol-cytochrome c reductase, Rieske iron-sulfur polypeptide 1 | Homo sapiens |
| 54621 | hypothetical protein FLJ20674 | Homo sapiens |
| 1635 | dCMP deaminase | Homo sapiens |
| 84140 | family with sequence similarity 161, member A | Homo sapiens |
| 27253 | protocadherin 17 | Homo sapiens |
| 10308 | zinc finger protein 267 | Homo sapiens |
| 11037 | stonin 1; STON1-GTF2A1L readthrough transcript; general transcription factor IIA, 1-like | Homo sapiens |
| 54994 | chromosome 20 open reading frame 11 | Homo sapiens |
| 7020 | transcription factor AP-2 alpha (activating enhancer binding protein 2 alpha) | Homo sapiens |
| 10020 | glucosamine (UDP-N-acetyl)-2-epimerase/N-acetylmannosamine kinase | Homo sapiens |
| 100288911 | hypothetical protein LOC100288911 | Homo sapiens |
| 9831 | zinc finger protein 623 | Homo sapiens |
| 728489 | DNL-type zinc finger | Homo sapiens |
| 9913 | suppressor of Ty 7 (S. cerevisiae)-like | Homo sapiens |
| 79158 | N-acetylglucosamine-1-phosphate transferase, alpha and beta subunits | Homo sapiens |
| 154150 | hepatoma derived growth factor-like 1 | Homo sapiens |
| 134510 | ubiquitin-like domain containing CTD phosphatase 1 | Homo sapiens |
| 5660 | prosaposin | Homo sapiens |
| 7037 | transferrin receptor (p90, CD71) | Homo sapiens |
| 11193 | WW domain binding protein 4 (formin binding protein 21) | Homo sapiens |
| 56957 | OTU domain containing 7B | Homo sapiens |
| 55215 | Fanconi anemia, complementation group I | Homo sapiens |
| 9698 | pumilio homolog 1 (Drosophila) | Homo sapiens |
| 50488 | misshapen-like kinase 1 (zebrafish) | Homo sapiens |
| 170850 | potassium voltage-gated channel, subfamily G, member 3 | Homo sapiens |
| 6891 | transporter 2, ATP-binding cassette, sub-family B (MDR/TAP) | Homo sapiens |
| 8027 | signal transducing adaptor molecule (SH3 domain and ITAM motif) 1 | Homo sapiens |
| 100290215 | similar to actin-related protein 3-beta | Homo sapiens |
| 60436 | TGFB-induced factor homeobox 2 | Homo sapiens |
| 79188 | transmembrane protein 43 | Homo sapiens |
| 8349 | histone cluster 2, H2be | Homo sapiens |
| 55717 | bromodomain and WD repeat domain containing 2 | Homo sapiens |
| 817 | calcium/calmodulin-dependent protein kinase II delta | Homo sapiens |
| 387640 | chromosome 10 open reading frame 140 | Homo sapiens |
| 7918 | HLA-B associated transcript 4 | Homo sapiens |

| | | |
|---|---|---|
| 9131 | apoptosis-inducing factor, mitochondrion-associated, 1 | Homo sapiens |
| 2935 | G1 to S phase transition 1 | Homo sapiens |
| 5042 | poly(A) binding protein, cytoplasmic 3 | Homo sapiens |
| 90441 | zinc finger protein 622 | Homo sapiens |
| 7625 | zinc finger protein 74 | Homo sapiens |
| 25820 | ariadne homolog, ubiquitin-conjugating enzyme E2 binding protein, 1 (Drosophila) | Homo sapiens |
| 9412 | mediator complex subunit 21 | Homo sapiens |
| 27089 | ubiquinol-cytochrome c reductase, complex III subunit VII, 9.5kDa | Homo sapiens |
| 8099 | cyclin-dependent kinase 2 associated protein 1 | Homo sapiens |
| 147841 | SPC24, NDC80 kinetochore complex component, homolog (S. cerevisiae) | Homo sapiens |
| 4850 | CCR4-NOT transcription complex, subunit 4 | Homo sapiens |
| 148479 | PHD finger protein 13 | Homo sapiens |
| 8905 | adaptor-related protein complex 1, sigma 2 subunit pseudogene; adaptor-related protein complex 1, sigma 2 subunit | Homo sapiens |
| 387647 | patched domain containing 3 pseudogene | Homo sapiens |
| 79048 | SECIS binding protein 2 | Homo sapiens |
| 58497 | prune homolog (Drosophila) | Homo sapiens |
| 23595 | origin recognition complex, subunit 3-like (yeast) | Homo sapiens |
| 54552 | guanine nucleotide binding protein-like 3 (nucleolar)-like | Homo sapiens |
| 219333 | ubiquitin specific peptidase 12 | Homo sapiens |
| 3093 | ubiquitin-conjugating enzyme E2K (UBC1 homolog, yeast) | Homo sapiens |
| 11062 | dihydrouridine synthase 4-like (S. cerevisiae) | Homo sapiens |
| 645323 | hypothetical LOC645323; microRNA 9-2 | Homo sapiens |
| 5612 | protein-kinase, interferon-inducible double stranded RNA dependent inhibitor, repressor of (P58 repressor) | Homo sapiens |
| 23142 | DCN1, defective in cullin neddylation 1, domain containing 4 (S. cerevisiae) | Homo sapiens |
| 9849 | zinc finger protein 518A | Homo sapiens |
| 54386 | telomeric repeat binding factor 2, interacting protein | Homo sapiens |
| 10668 | cell growth regulator with ring finger domain 1 | Homo sapiens |
| 644068 | ribosomal protein S14 pseudogene 3 | Homo sapiens |
| 381 | ADP-ribosylation factor 5 | Homo sapiens |
| 6595 | SWI/SNF related, matrix associated, actin dependent regulator of chromatin, subfamily a, member 2 | Homo sapiens |
| 5693 | proteasome (prosome, macropain) subunit, beta type, 5 | Homo sapiens |
| 9556 | chromosome 14 open reading frame 2 | Homo sapiens |
| 9781 | ring finger protein 144A | Homo sapiens |
| 3015 | H2A histone family, member Z | Homo sapiens |
| 51306 | family with sequence similarity 13, member B | Homo sapiens |
| 93134 | zinc finger protein 561; zinc finger protein 812 | Homo sapiens |
| 57478 | ubiquitin specific peptidase 31 | Homo sapiens |
| 7976 | frizzled homolog 3 (Drosophila) | Homo sapiens |
| 2058 | glutamyl-prolyl-tRNA synthetase | Homo sapiens |
| 4706 | NADH dehydrogenase (ubiquinone) 1, alpha/beta subcomplex, 1, 8kDa | Homo sapiens |
| 5510 | protein phosphatase 1, regulatory (inhibitor) subunit 7 | Homo sapiens |
| 8727 | catenin (cadherin-associated protein), alpha-like 1 | Homo sapiens |
| 79447 | chromosome 16 open reading frame 53 | Homo sapiens |
| 84669 | similar to TBC1 domain family, member 3; ubiquitin specific peptidase 32 | Homo sapiens |
| 85358 | SH3 and multiple ankyrin repeat domains 3 | Homo sapiens |
| 22862 | fibronectin type III domain containing 3A | Homo sapiens |
| 6018 | rearranged L-myc fusion | Homo sapiens |
| 388591 | ring finger protein 207 | Homo sapiens |
| 1182 | chloride channel 3 | Homo sapiens |
| 57724 | KIAA1632 | Homo sapiens |
| 23002 | dishevelled associated activator of morphogenesis 1 | Homo sapiens |
| 54987 | chromosome 1 open reading frame 123 | Homo sapiens |
| 54931 | RNA (guanine-9-) methyltransferase domain containing 1 | Homo sapiens |
| 8607 | RuvB-like 1 (E. coli) | Homo sapiens |
| 222166 | chromosome 7 open reading frame 41 | Homo sapiens |
| 892 | cyclin C | Homo sapiens |
| 55835 | centromere protein J | Homo sapiens |
| 57761 | tribbles homolog 3 (Drosophila) | Homo sapiens |
| 636 | bicaudal D homolog 1 (Drosophila) | Homo sapiens |
| 25800 | solute carrier family 39 (zinc transporter), member 6 | Homo sapiens |
| 399948 | chromosome 11 open reading frame 92 | Homo sapiens |
| 161436 | echinoderm microtubule associated protein like 5 | Homo sapiens |
| 9031 | bromodomain adjacent to zinc finger domain, 1B | Homo sapiens |
| 29929 | asparagine-linked glycosylation 6, alpha-1,3-glucosyltransferase homolog (S. cerevisiae) | Homo sapiens |
| 10962 | myeloid/lymphoid or mixed-lineage leukemia (trithorax homolog, Drosophila); translocated to, 11 | Homo sapiens |
| 64768 | inositol 1,3,4,5,6-pentakisphosphate 2-kinase | Homo sapiens |
| 8630 | hydroxysteroid (17-beta) dehydrogenase 6 homolog (mouse) | Homo sapiens |
| 57458 | transmembrane and coiled-coil domain family 3 | Homo sapiens |
| 55164 | SHQ1 homolog (S. cerevisiae) | Homo sapiens |
| 9308 | CD83 molecule | Homo sapiens |
| 3225 | homeobox C9 | Homo sapiens |
| 4952 | oculocerebrorenal syndrome of Lowe | Homo sapiens |
| 25957 | splicing factor, arginine/serine-rich 18 | Homo sapiens |

| | | |
|---|---|---|
| 9141 | programmed cell death 5 | Homo sapiens |
| 797 | calcitonin-related polypeptide beta | Homo sapiens |
| 79003 | MIS12, MIND kinetochore complex component, homolog (S. pombe) | Homo sapiens |
| 79073 | transmembrane protein 109 | Homo sapiens |
| 1059 | centromere protein B, 80kDa | Homo sapiens |
| 6574 | solute carrier family 20 (phosphate transporter), member 1 | Homo sapiens |
| 84747 | unc-119 homolog B (C. elegans) | Homo sapiens |
| 51010 | exosome component 3 | Homo sapiens |
| 6207 | ribosomal protein S13 pseudogene 8; ribosomal protein S13; ribosomal protein S13 pseudogene 2 | Homo sapiens |
| 64854 | ubiquitin specific peptidase 46 | Homo sapiens |
| 157574 | F-box protein 16 | Homo sapiens |
| 10384 | butyrophilin, subfamily 3, member A3 | Homo sapiens |
| 653121 | zinc finger and BTB domain containing 8A | Homo sapiens |
| 23099 | zinc finger and BTB domain containing 43 | Homo sapiens |
| 54206 | ERBB receptor feedback inhibitor 1 | Homo sapiens |
| 81689 | iron-sulfur cluster assembly 1 homolog (S. cerevisiae) | Homo sapiens |
| 5192 | peroxisomal biogenesis factor 10 | Homo sapiens |
| 4171 | minichromosome maintenance complex component 2 | Homo sapiens |
| 728026 | hypothetical LOC728026; prothymosin, alpha; hypothetical gene supported by BC013859; prothymosin, alpha pseudogene 4 (gene sequence 112) | Homo sapiens |
| 441454 | hypothetical LOC728026; prothymosin, alpha; hypothetical gene supported by BC013859; prothymosin, alpha pseudogene 4 (gene sequence 112) | Homo sapiens |
| 5757 | hypothetical LOC728026; prothymosin, alpha; hypothetical gene supported by BC013859; prothymosin, alpha pseudogene 4 (gene sequence 112) | Homo sapiens |
| 57050 | UTP3, small subunit (SSU) processome component, homolog (S. cerevisiae) | Homo sapiens |
| 57585 | Crm, cramped-like (Drosophila) | Homo sapiens |
| 2201 | fibrillin 2 | Homo sapiens |
| 57801 | hairy and enhancer of split 4 (Drosophila) | Homo sapiens |
| 60493 | FAST kinase domains 5 | Homo sapiens |
| 79598 | centrosomal protein 97kDa | Homo sapiens |
| 55611 | OTU domain, ubiquitin aldehyde binding 1 | Homo sapiens |
| 6938 | transcription factor 12 | Homo sapiens |
| 57786 | hypothetical LOC389458; RB-associated KRAB zinc finger | Homo sapiens |
| 85415 | rhophilin, Rho GTPase binding protein 2; similar to rhophilin, Rho GTPase binding protein 2 | Homo sapiens |
| 150726 | F-box protein 41 | Homo sapiens |
| 8837 | CASP8 and FADD-like apoptosis regulator | Homo sapiens |
| 644353 | zinc finger, CCHC domain containing 18 | Homo sapiens |
| 10653 | serine peptidase inhibitor, Kunitz type, 2 | Homo sapiens |
| 81856 | zinc finger protein 611 | Homo sapiens |
| 10594 | PRP8 pre-mRNA processing factor 8 homolog (S. cerevisiae) | Homo sapiens |
| 257218 | SNF2 histone linker PHD RING helicase | Homo sapiens |
| 4946 | ornithine decarboxylase antizyme 1 | Homo sapiens |
| 91272 | biorientation of chromosomes in cell division 1 | Homo sapiens |
| 55114 | Rho GTPase activating protein 17 | Homo sapiens |
| 10015 | programmed cell death 6 interacting protein | Homo sapiens |
| 771 | carbonic anhydrase XII | Homo sapiens |
| 5358 | plastin 3 (T isoform) | Homo sapiens |
| 64172 | O-sialoglycoprotein endopeptidase-like 1 | Homo sapiens |
| 4726 | NADH dehydrogenase (ubiquinone) Fe-S protein 6, 13kDa (NADH-coenzyme Q reductase) | Homo sapiens |
| 84153 | ribonuclease H2, subunit C | Homo sapiens |
| 6599 | SWI/SNF related, matrix associated, actin dependent regulator of chromatin, subfamily c, member 1 | Homo sapiens |
| 26053 | autism susceptibility candidate 2 | Homo sapiens |
| 1981 | eukaryotic translation initiation factor 4 gamma, 1 | Homo sapiens |
| 81839 | vang-like 1 (van gogh, Drosophila) | Homo sapiens |
| 222236 | N-acyl phosphatidylethanolamine phospholipase D | Homo sapiens |
| 7543 | zinc finger protein, X-linked | Homo sapiens |
| 10092 | actin related protein 2/3 complex, subunit 5, 16kDa | Homo sapiens |
| 4522 | methylenetetrahydrofolate dehydrogenase (NADP+ dependent) 1, methenyltetrahydrofolate cyclohydrolase, formyltetrahydrofolate synthetase | Homo sapiens |
| 90271 | chromosome 10 open reading frame 75 | Homo sapiens |
| 5433 | polymerase (RNA) II (DNA directed) polypeptide D | Homo sapiens |
| 22834 | zinc finger protein 652 | Homo sapiens |
| 8527 | diacylglycerol kinase, delta 130kDa | Homo sapiens |
| 50717 | WD repeat domain 42A | Homo sapiens |
| 64979 | mitochondrial ribosomal protein L36 | Homo sapiens |
| 6210 | ribosomal protein S15a pseudogene 17; ribosomal protein S15a pseudogene 19; ribosomal protein S15a pseudogene 12; ribosomal protein S15a pseudogene 24; ribosomal protein S15a pseudogene 11; ribosomal protein S15a | Homo sapiens |
| 23262 | histidine acid phosphatase domain containing 1 | Homo sapiens |
| 5571 | protein kinase, AMP-activated, gamma 1 non-catalytic subunit | Homo sapiens |
| 160428 | aldehyde dehydrogenase 1 family, member L2 | Homo sapiens |
| 130535 | potassium channel tetramerisation domain containing 18 | Homo sapiens |
| 64927 | tetratricopeptide repeat domain 23 | Homo sapiens |
| 253635 | coiled-coil domain containing 75 | Homo sapiens |

| | | |
|---|---|---|
| 1287 | collagen, type IV, alpha 5 | Homo sapiens |
| 23379 | KIAA0947 | Homo sapiens |
| 56937 | prostate transmembrane protein, androgen induced 1 | Homo sapiens |
| 8883 | NEDD8 activating enzyme E1 subunit 1 | Homo sapiens |
| 154467 | chromosome 6 open reading frame 129 | Homo sapiens |
| 22920 | kinesin-associated protein 3 | Homo sapiens |
| 5830 | peroxisomal biogenesis factor 5 | Homo sapiens |
| 2319 | flotillin 2 | Homo sapiens |
| 5683 | proteasome (prosome, macropain) subunit, alpha type, 2 | Homo sapiens |
| 51292 | guanosine monophosphate reductase 2 | Homo sapiens |
| 79064 | transmembrane protein 223 | Homo sapiens |
| 7384 | ubiquinol-cytochrome c reductase core protein I | Homo sapiens |
| 836 | caspase 3, apoptosis-related cysteine peptidase | Homo sapiens |
| 2803 | golgi autoantigen, golgin subfamily a, 4 | Homo sapiens |
| 5226 | phosphogluconate dehydrogenase | Homo sapiens |
| 11235 | programmed cell death 10 | Homo sapiens |
| 55588 | mediator complex subunit 29 | Homo sapiens |
| 5931 | retinoblastoma binding protein 7 | Homo sapiens |
| 8323 | frizzled homolog 6 (Drosophila) | Homo sapiens |
| 79768 | chromosome 15 open reading frame 29 | Homo sapiens |
| 148189 | hypothetical LOC148189 | Homo sapiens |
| 8773 | synaptosomal-associated protein, 23kDa | Homo sapiens |
| 6634 | small nuclear ribonucleoprotein D3 polypeptide 18kDa | Homo sapiens |
| 10556 | ribonuclease P/MRP 30kDa subunit | Homo sapiens |
| 1642 | damage-specific DNA binding protein 1, 127kDa | Homo sapiens |
| 3134 | major histocompatibility complex, class I, F | Homo sapiens |
| 55026 | family with sequence similarity 70, member A | Homo sapiens |
| 57551 | TAO kinase 1 | Homo sapiens |
| 4904 | Y box binding protein 1 | Homo sapiens |
| 125144 | chromosome 17 open reading frame 45 | Homo sapiens |
| 63893 | ubiquitin-conjugating enzyme E2O | Homo sapiens |
| 4928 | nucleoporin 98kDa | Homo sapiens |
| 151162 | mannosyl (alpha-1,6-)-glycoprotein beta-1,6-N-acetyl-glucosaminyltransferase; hypothetical LOC151162 | Homo sapiens |
| 4249 | mannosyl (alpha-1,6-)-glycoprotein beta-1,6-N-acetyl-glucosaminyltransferase; hypothetical LOC151162 | Homo sapiens |
| 64782 | apoptosis enhancing nuclease | Homo sapiens |
| 4205 | myocyte enhancer factor 2A | Homo sapiens |
| 84327 | zinc finger, BED-type containing 3 | Homo sapiens |
| 3785 | potassium voltage-gated channel, KQT-like subfamily, member 2 | Homo sapiens |
| 3858 | keratin 10 | Homo sapiens |
| 10243 | gephyrin | Homo sapiens |
| 29887 | sorting nexin 10 | Homo sapiens |
| 30011 | SH3-domain kinase binding protein 1 | Homo sapiens |
| 56992 | kinesin family member 15 | Homo sapiens |
| 79829 | N-acetyltransferase 11 (GCN5-related, putative) | Homo sapiens |
| 5721 | proteasome (prosome, macropain) activator subunit 2 (PA28 beta) | Homo sapiens |
| 6882 | TAF11 RNA polymerase II, TATA box binding protein (TBP)-associated factor, 28kDa | Homo sapiens |
| 84809 | ciliary rootlet coiled-coil, rootletin-like 1 | Homo sapiens |
| 54463 | family with sequence similarity 134, member B | Homo sapiens |
| 147081 | chromosome 17 open reading frame 69 | Homo sapiens |
| 4717 | NADH dehydrogenase (ubiquinone) 1, subcomplex unknown, 1, 6kDa | Homo sapiens |
| 2590 | UDP-N-acetyl-alpha-D-galactosamine:polypeptide N-acetylgalactosaminyltransferase 2 (GalNAc-T2) | Homo sapiens |
| 51122 | COMM domain containing 2 | Homo sapiens |
| 100288142 | similar to neuroblastoma breakpoint family, member 10 | Homo sapiens |
| 6935 | zinc finger E-box binding homeobox 1 | Homo sapiens |
| 257364 | sorting nexin 33 | Homo sapiens |
| 22928 | selenophosphate synthetase 2 | Homo sapiens |
| 8667 | eukaryotic translation initiation factor 3, subunit H | Homo sapiens |
| 80339 | patatin-like phospholipase domain containing 3 | Homo sapiens |
| 23219 | F-box protein 28 | Homo sapiens |
| 79868 | asparagine-linked glycosylation 13 homolog (S. cerevisiae) | Homo sapiens |
| 4258 | microsomal glutathione S-transferase 2 | Homo sapiens |
| 8266 | ubiquitin-like 4A | Homo sapiens |
| 23015 | golgi autoantigen, golgin subfamily a, 8B; golgi autoantigen, golgin subfamily a, 8A | Homo sapiens |
| 192286 | HIG1 hypoxia inducible domain family, member 2A | Homo sapiens |
| 4864 | Niemann-Pick disease, type C1 | Homo sapiens |
| 768211 | RELT-like 1 | Homo sapiens |
| 25949 | SYF2 homolog, RNA splicing factor (S. cerevisiae) | Homo sapiens |
| 6155 | ribosomal protein L27 | Homo sapiens |
| 4173 | minichromosome maintenance complex component 4 | Homo sapiens |
| 64764 | cAMP responsive element binding protein 3-like 2 | Homo sapiens |
| 5710 | proteasome (prosome, macropain) 26S subunit, non-ATPase, 4 | Homo sapiens |
| 10916 | melanoma antigen family D, 2 | Homo sapiens |
| 3485 | insulin-like growth factor binding protein 2, 36kDa | Homo sapiens |
| 288 | ankyrin 3, node of Ranvier (ankyrin G) | Homo sapiens |

| | | |
|---|---|---|
| 3841 | karyopherin alpha 5 (importin alpha 6) | Homo sapiens |
| 56935 | hypothetical LOC728675; chromosome 11 open reading frame 75 | Homo sapiens |
| 10951 | chromobox homolog 1 (HP1 beta homolog Drosophila ) | Homo sapiens |
| 84959 | ubiquitin associated and SH3 domain containing, B | Homo sapiens |
| 894 | cyclin D2 | Homo sapiens |
| 4863 | nuclear protein, ataxia-telangiectasia locus | Homo sapiens |
| 7024 | transcription factor CP2 | Homo sapiens |
| 1951 | solute carrier family 26, member 6; cadherin, EGF LAG seven-pass G-type receptor 3 (flamingo homolog, Drosophila) | Homo sapiens |
| 130355 | chromosome 2 open reading frame 76 | Homo sapiens |
| 57129 | mitochondrial ribosomal protein L47 | Homo sapiens |
| 54800 | kelch-like 24 (Drosophila) | Homo sapiens |
| 23421 | integrin beta 3 binding protein (beta3-endonexin) | Homo sapiens |
| 5985 | replication factor C (activator 1) 5, 36.5kDa | Homo sapiens |
| 1130 | lysosomal trafficking regulator | Homo sapiens |
| 9923 | zinc finger and BTB domain containing 40 | Homo sapiens |
| 26589 | mitochondrial ribosomal protein L46 | Homo sapiens |
| 57510 | exportin 5 | Homo sapiens |
| 91151 | tigger transposable element derived 7 | Homo sapiens |
| 7584 | zinc finger protein 35 | Homo sapiens |
| 54820 | nudE nuclear distribution gene E homolog 1 (A. nidulans) | Homo sapiens |
| 93550 | AN1, ubiquitin-like, homolog (Xenopus laevis) | Homo sapiens |
| 9747 | family with sequence similarity 115, member A; family with sequence similarity 115, member B (pseudogene) | Homo sapiens |
| 6201 | ribosomal protein S7; ribosomal protein S7 pseudogene 11; ribosomal protein S7 pseudogene 4; ribosomal protein S7 pseudogene 10 | Homo sapiens |
| 5101 | protocadherin 9 | Homo sapiens |
| 6502 | S-phase kinase-associated protein 2 (p45) | Homo sapiens |
| 4176 | minichromosome maintenance complex component 7 | Homo sapiens |
| 255877 | B-cell CLL/lymphoma 6, member B (zinc finger protein) | Homo sapiens |
| 55968 | NSFL1 (p97) cofactor (p47) | Homo sapiens |
| 373156 | glutathione S-transferase kappa 1 | Homo sapiens |
| 7475 | wingless-type MMTV integration site family, member 6 | Homo sapiens |
| 147015 | dehydrogenase/reductase (SDR family) member 13 | Homo sapiens |
| 6091 | roundabout, axon guidance receptor, homolog 1 (Drosophila); similar to roundabout 1 isoform b | Homo sapiens |
| 285190 | RANBP2-like and GRIP domain containing 5; RANBP2-like and GRIP domain containing 4; RANBP2-like and GRIP domain containing 3; RANBP2-like and GRIP domain containing 8; RANBP2-like and GRIP domain containing 7; RANBP2-like and GRIP domain containing 6 | Homo sapiens |
| 729540 | RANBP2-like and GRIP domain containing 5; RANBP2-like and GRIP domain containing 4; RANBP2-like and GRIP domain containing 3; RANBP2-like and GRIP domain containing 8; RANBP2-like and GRIP domain containing 7; RANBP2-like and GRIP domain containing 6 | Homo sapiens |
| 727851 | RANBP2-like and GRIP domain containing 5; RANBP2-like and GRIP domain containing 4; RANBP2-like and GRIP domain containing 3; RANBP2-like and GRIP domain containing 8; RANBP2-like and GRIP domain containing 7; RANBP2-like and GRIP domain containing 6 | Homo sapiens |
| 84220 | RANBP2-like and GRIP domain containing 5; RANBP2-like and GRIP domain containing 4; RANBP2-like and GRIP domain containing 3; RANBP2-like and GRIP domain containing 8; RANBP2-like and GRIP domain containing 7; RANBP2-like and GRIP domain containing 6 | Homo sapiens |
| 387707 | coiled-coil and C2 domain containing 2B | Homo sapiens |
| 5927 | lysine (K)-specific demethylase 5A | Homo sapiens |
| 84236 | rhomboid domain containing 1 | Homo sapiens |
| 80775 | transmembrane protein 177 | Homo sapiens |
| 55916 | nuclear transport factor 2-like export factor 2 | Homo sapiens |
| 23300 | ATM interactor | Homo sapiens |
| 79078 | chromosome 1 open reading frame 50 | Homo sapiens |
| 25793 | F-box protein 7 | Homo sapiens |
| 133418 | embigin homolog (mouse) | Homo sapiens |
| 58478 | enolase-phosphatase 1 | Homo sapiens |
| 51283 | bifunctional apoptosis regulator | Homo sapiens |
| 8933 | family with sequence similarity 127, member A | Homo sapiens |
| 10955 | serine incorporator 3 | Homo sapiens |
| 966 | CD59 molecule, complement regulatory protein | Homo sapiens |
| 153241 | centrosomal protein 120kDa | Homo sapiens |
| 219654 | zinc finger, CCHC domain containing 24 | Homo sapiens |
| 23181 | DIP2 disco-interacting protein 2 homolog A (Drosophila) | Homo sapiens |
| 8763 | CD164 molecule, sialomucin | Homo sapiens |
| 9896 | FIG4 homolog (S. cerevisiae) | Homo sapiens |
| 79738 | Bardet-Biedl syndrome 10 | Homo sapiens |
| 100131067 | hypothetical protein LOC100131067 | Homo sapiens |
| 1831 | TSC22 domain family, member 3; GRAM domain containing 4 | Homo sapiens |
| 22998 | LIM and calponin homology domains 1 | Homo sapiens |
| 26234 | F-box and leucine-rich repeat protein 5 | Homo sapiens |
| 222484 | ligand of numb-protein X 2 | Homo sapiens |
| 3234 | homeobox D8 | Homo sapiens |
| 23642 | small nucleolar RNA host gene 1 (non-protein coding) | Homo sapiens |
| 200081 | taxilin alpha | Homo sapiens |
| 1629 | dihydrolipoamide branched chain transacylase E2 | Homo sapiens |

| | | |
|---|---|---|
| 63877 | chromosome 10 open reading frame 84 | Homo sapiens |
| 83439 | transcription factor 7-like 1 (T-cell specific, HMG-box) | Homo sapiens |
| 145567 | tetratricopeptide repeat domain 7B | Homo sapiens |
| 6867 | transforming, acidic coiled-coil containing protein 1 | Homo sapiens |
| 55102 | ATG2 autophagy related 2 homolog B (S. cerevisiae) | Homo sapiens |
| 5283 | phosphatidylinositol glycan anchor biosynthesis, class H | Homo sapiens |
| 22837 | COBL-like 1 | Homo sapiens |
| 4674 | nucleosome assembly protein 1-like 2 | Homo sapiens |
| 643224 | tubulin, beta 8 pseudogene | Homo sapiens |
| 401115 | chromosome 4 open reading frame 48 | Homo sapiens |
| 151963 | chromosome 3 open reading frame 59 | Homo sapiens |
| 3068 | hepatoma-derived growth factor (high-mobility group protein 1-like) | Homo sapiens |
| 56474 | CTP synthase II | Homo sapiens |
| 23198 | proteasome (prosome, macropain) activator subunit 4 | Homo sapiens |
| 203197 | chromosome 9 open reading frame 91 | Homo sapiens |
| 6171 | ribosomal protein L41 | Homo sapiens |
| 80763 | chromosome 12 open reading frame 39 | Homo sapiens |
| 284040 | CMT1A duplicated region transcript 4 | Homo sapiens |
| 87178 | polyribonucleotide nucleotidyltransferase 1 | Homo sapiens |
| 10667 | phenylalanyl-tRNA synthetase 2, mitochondrial | Homo sapiens |
| 26269 | F-box protein 8 | Homo sapiens |
| 119392 | chromosome 10 open reading frame 78 | Homo sapiens |
| 56172 | ankylosis, progressive homolog (mouse) | Homo sapiens |
| 57470 | leucine rich repeat containing 47 | Homo sapiens |
| 55005 | required for meiotic nuclear division 1 homolog (S. cerevisiae) | Homo sapiens |
| 23022 | palladin, cytoskeletal associated protein | Homo sapiens |
| 65062 | amyotrophic lateral sclerosis 2 (juvenile) chromosome region, candidate 4 | Homo sapiens |
| 51340 | crooked neck pre-mRNA splicing factor-like 1 (Drosophila) | Homo sapiens |
| 11147 | HERV-H LTR-associating 3 | Homo sapiens |
| 372 | archain 1 | Homo sapiens |
| 3292 | hydroxysteroid (17-beta) dehydrogenase 1 | Homo sapiens |
| 8575 | protein kinase, interferon-inducible double stranded RNA dependent activator | Homo sapiens |
| 7702 | zinc finger protein 143 | Homo sapiens |
| 57563 | kelch-like 8 (Drosophila) | Homo sapiens |
| 55687 | tRNA 5-methylaminomethyl-2-thiouridylate methyltransferase | Homo sapiens |
| 26993 | A kinase (PRKA) anchor protein 8-like | Homo sapiens |
| 79017 | gamma-glutamyl cyclotransferase | Homo sapiens |
| 57181 | solute carrier family 39 (zinc transporter), member 10 | Homo sapiens |
| 28962 | osteopetrosis associated transmembrane protein 1 | Homo sapiens |
| 84513 | phosphatidic acid phosphatase type 2 domain containing 1B | Homo sapiens |
| 6654 | son of sevenless homolog 1 (Drosophila) | Homo sapiens |
| 10477 | ubiquitin-conjugating enzyme E2E 3 (UBC4/5 homolog, yeast) | Homo sapiens |
| 6281 | S100 calcium binding protein A10 | Homo sapiens |
| 1876 | E2F transcription factor 6 | Homo sapiens |
| 515 | ATP synthase, H+ transporting, mitochondrial F0 complex, subunit B1 | Homo sapiens |
| 2597 | glyceraldehyde-3-phosphate dehydrogenase-like 6; hypothetical protein LOC100133042; glyceraldehyde-3-phosphate dehydrogenase | Homo sapiens |
| 54881 | testis expressed 10 | Homo sapiens |
| 26050 | SLIT and NTRK-like family, member 5 | Homo sapiens |
| 9836 | leucine carboxyl methyltransferase 2 | Homo sapiens |
| 6157 | ribosomal protein L27a | Homo sapiens |
| 3658 | iron-responsive element binding protein 2 | Homo sapiens |
| 92312 | mex-3 homolog A (C. elegans) | Homo sapiens |
| 22838 | ring finger protein 44 | Homo sapiens |
| 8802 | succinate-CoA ligase, alpha subunit | Homo sapiens |
| 9467 | SH3-domain binding protein 5 (BTK-associated) | Homo sapiens |
| 201627 | family with sequence similarity 116, member A | Homo sapiens |
| 63935 | PDX1 C-terminal inhibiting factor 1 | Homo sapiens |
| 7319 | ubiquitin-conjugating enzyme E2A (RAD6 homolog) | Homo sapiens |
| 9581 | prolyl endopeptidase-like | Homo sapiens |
| 2730 | glutamate-cysteine ligase, modifier subunit | Homo sapiens |
| 10016 | aryl-hydrocarbon receptor repressor; programmed cell death 6 | Homo sapiens |
| 57491 | aryl-hydrocarbon receptor repressor; programmed cell death 6 | Homo sapiens |
| 64853 | hypothetical LOC653631; hypothetical LOC646050; hypothetical LOC646890; axin interactor, dorsalization associated | Homo sapiens |
| 8313 | axin 2 | Homo sapiens |
| 53342 | interleukin 17D | Homo sapiens |
| 23378 | ribosomal RNA processing 8, methyltransferase, homolog (yeast) | Homo sapiens |
| 28971 | chromosome 11 open reading frame 67 | Homo sapiens |
| 4248 | mannosyl (beta-1,4-)-glycoprotein beta-1,4-N-acetylglucosaminyltransferase | Homo sapiens |
| 200916 | ribosomal protein L22-like 1 | Homo sapiens |
| 25816 | tumor necrosis factor, alpha-induced protein 8 | Homo sapiens |
| 550 | ancient ubiquitous protein 1 | Homo sapiens |
| 51466 | Enah/Vasp-like | Homo sapiens |
| 11325 | DEAD (Asp-Glu-Ala-Asp) box polypeptide 42 | Homo sapiens |
| 11120 | butyrophilin, subfamily 2, member A1 | Homo sapiens |
| 65243 | zinc finger protein 643 | Homo sapiens |

| | | |
|---|---|---|
| 54976 | chromosome 20 open reading frame 27 | Homo sapiens |
| 102 | ADAM metallopeptidase domain 10 | Homo sapiens |
| 84263 | hydroxysteroid dehydrogenase like 2 | Homo sapiens |
| 55030 | F-box protein 34 | Homo sapiens |
| 9181 | Rho/Rac guanine nucleotide exchange factor (GEF) 2 | Homo sapiens |
| 7374 | uracil-DNA glycosylase | Homo sapiens |
| 84306 | programmed cell death 2-like | Homo sapiens |
| 81618 | integral membrane protein 2C | Homo sapiens |
| 10494 | serine/threonine kinase 25 (STE20 homolog, yeast) | Homo sapiens |
| 7415 | valosin-containing protein | Homo sapiens |
| 23043 | TRAF2 and NCK interacting kinase | Homo sapiens |
| 57542 | kelch domain containing 5 | Homo sapiens |
| 51714 | selenoprotein T; similar to Selenoprotein T | Homo sapiens |
| 10250 | serine/arginine repetitive matrix 1 | Homo sapiens |
| 55173 | mitochondrial ribosomal protein S10 | Homo sapiens |
| 63977 | PR domain containing 15 | Homo sapiens |
| 200895 | dihydrofolate reductase-like 1 | Homo sapiens |
| 10952 | Sec61 beta subunit | Homo sapiens |
| 10564 | ADP-ribosylation factor guanine nucleotide-exchange factor 2 (brefeldin A-inhibited) | Homo sapiens |
| 55000 | taurine upregulated 1 (non-protein coding) | Homo sapiens |
| 4139 | MAP/microtubule affinity-regulating kinase 1 | Homo sapiens |
| 669 | 2,3-bisphosphoglycerate mutase | Homo sapiens |
| 81542 | thioredoxin-related transmembrane protein 1 | Homo sapiens |
| 26152 | zinc finger protein 337 | Homo sapiens |
| 140838 | N-acetylneuraminic acid phosphatase | Homo sapiens |
| 10621 | polymerase (RNA) III (DNA directed) polypeptide F, 39 kDa | Homo sapiens |
| 254887 | zinc finger, DHHC-type containing 23 | Homo sapiens |
| 11315 | Parkinson disease (autosomal recessive, early onset) 7 | Homo sapiens |
| 118433 | ribosomal protein L23a pseudogene 7 | Homo sapiens |
| 5692 | proteasome (prosome, macropain) subunit, beta type, 4 | Homo sapiens |
| 161823 | adenosine deaminase-like | Homo sapiens |
| 9401 | RecQ protein-like 4 | Homo sapiens |
| 145853 | chromosome 15 open reading frame 61 | Homo sapiens |
| 4696 | NADH dehydrogenase (ubiquinone) 1 alpha subcomplex, 3, 9kDa | Homo sapiens |
| 5563 | protein kinase, AMP-activated, alpha 2 catalytic subunit | Homo sapiens |
| 5127 | PCTAIRE protein kinase 1 | Homo sapiens |
| 157313 | cell division cycle associated 2 | Homo sapiens |
| 55628 | zinc finger protein 407 | Homo sapiens |
| 51293 | CD320 molecule | Homo sapiens |
| 975 | CD81 molecule | Homo sapiens |
| 10730 | YME1-like 1 (S. cerevisiae) | Homo sapiens |
| 122553 | trafficking protein particle complex 6B | Homo sapiens |
| 23532 | preferentially expressed antigen in melanoma | Homo sapiens |
| 9258 | malignant fibrous histiocytoma amplified sequence 1 | Homo sapiens |
| 23169 | solute carrier family 35 (UDP-glucuronic acid/UDP-N-acetylgalactosamine dual transporter), member D1 | Homo sapiens |
| 23180 | raftlin, lipid raft linker 1 | Homo sapiens |
| 4502 | metallothionein 2A | Homo sapiens |
| 84128 | WD repeat domain 75 | Homo sapiens |
| 390354 | ribosomal protein L18a pseudogene 3 | Homo sapiens |
| 56829 | zinc finger CCCH-type, antiviral 1 | Homo sapiens |
| 51174 | tubulin, delta 1 | Homo sapiens |
| 7525 | v-yes-1 Yamaguchi sarcoma viral oncogene homolog 1 | Homo sapiens |
| 51386 | eukaryotic translation initiation factor 3, subunit L | Homo sapiens |
| 9575 | clock homolog (mouse) | Homo sapiens |
| 55093 | WDYHV motif containing 1 | Homo sapiens |
| 3106 | major histocompatibility complex, class I, C; major histocompatibility complex, class I, B | Homo sapiens |
| 3107 | major histocompatibility complex, class I, C; major histocompatibility complex, class I, B | Homo sapiens |
| 55277 | FGGY carbohydrate kinase domain containing | Homo sapiens |
| 11004 | kinesin family member 2C | Homo sapiens |
| 1627 | drebrin 1 | Homo sapiens |
| 23312 | Dmx-like 2 | Homo sapiens |
| 79441 | HAUS augmin-like complex, subunit 3 | Homo sapiens |
| 65267 | WNK lysine deficient protein kinase 3 | Homo sapiens |
| 9817 | kelch-like ECH-associated protein 1 | Homo sapiens |
| 126298 | immunity-related GTPase family, Q | Homo sapiens |
| 51082 | polymerase (RNA) I polypeptide D, 16kDa | Homo sapiens |
| 1147 | conserved helix-loop-helix ubiquitous kinase | Homo sapiens |
| 10644 | insulin-like growth factor 2 mRNA binding protein 2 | Homo sapiens |
| 6941 | transcription factor 19 | Homo sapiens |
| 9616 | ring finger protein 7 | Homo sapiens |
| 51247 | poly(A) binding protein interacting protein 2 | Homo sapiens |
| 6878 | TAF6 RNA polymerase II, TATA box binding protein (TBP)-associated factor, 80kDa | Homo sapiens |
| 121457 | IKK interacting protein | Homo sapiens |
| 57168 | aspartate beta-hydroxylase domain containing 2 | Homo sapiens |

| | | |
|---|---|---|
| 51691 | LSM8 homolog, U6 small nuclear RNA associated (S. cerevisiae) | Homo sapiens |
| 55342 | spermatid perinuclear RNA binding protein | Homo sapiens |
| 9936 | CD302 molecule; lymphocyte antigen 75 | Homo sapiens |
| 7004 | TEA domain family member 4 | Homo sapiens |
| 2975 | general transcription factor IIIC, polypeptide 1, alpha 220kDa | Homo sapiens |
| 552900 | bolA homolog 2 (E. coli); bolA homolog 2B (E. coli) | Homo sapiens |
| 654483 | bolA homolog 2 (E. coli); bolA homolog 2B (E. coli) | Homo sapiens |
| 91833 | WD repeat domain 20 | Homo sapiens |
| 57590 | WD repeat and FYVE domain containing 1 | Homo sapiens |
| 10010 | TRAF family member-associated NFKB activator | Homo sapiens |
| 121665 | signal peptide peptidase 3 | Homo sapiens |
| 5170 | 3-phosphoinositide dependent protein kinase-1 | Homo sapiens |
| 9861 | proteasome (prosome, macropain) 26S subunit, non-ATPase, 6 | Homo sapiens |
| 57520 | HECT, C2 and WW domain containing E3 ubiquitin protein ligase 2 | Homo sapiens |
| 112885 | PHD finger protein 21B | Homo sapiens |
| 9672 | syndecan 3 | Homo sapiens |
| 51768 | transmembrane 7 superfamily member 3 | Homo sapiens |
| 5110 | protein-L-isoaspartate (D-aspartate) O-methyltransferase | Homo sapiens |
| 51105 | PHD finger protein 20-like 1 | Homo sapiens |
| 1798 | dolichyl-phosphate (UDP-N-acetylglucosamine) N-acetylglucosaminephosphotransferase 1 (GlcNAc-1-P transferase) | Homo sapiens |
| 5884 | RAD17 homolog (S. pombe) | Homo sapiens |
| 55049 | chromosome 19 open reading frame 60 | Homo sapiens |
| 9583 | ectonucleoside triphosphate diphosphohydrolase 4 | Homo sapiens |
| 11338 | U2 small nuclear RNA auxiliary factor 2 | Homo sapiens |
| 2823 | glycoprotein M6A | Homo sapiens |
| 10567 | Rab acceptor 1 (prenylated) | Homo sapiens |
| 5325 | pleiomorphic adenoma gene-like 1 | Homo sapiens |
| 55255 | WD repeat domain 41 | Homo sapiens |
| 80055 | post-GPI attachment to proteins 1 | Homo sapiens |
| 468 | activating transcription factor 4 (tax-responsive enhancer element B67); activating transcription factor 4C | Homo sapiens |
| 9706 | unc-51-like kinase 2 (C. elegans) | Homo sapiens |
| 373863 | dead end homolog 1 (zebrafish); similar to dead end homolog 1 | Homo sapiens |
| 6272 | sortilin 1 | Homo sapiens |
| 7699 | zinc finger protein 140 | Homo sapiens |
| 23119 | hypermethylated in cancer 2 | Homo sapiens |
| 64318 | nucleolar complex associated 3 homolog (S. cerevisiae) | Homo sapiens |
| 7311 | ubiquitin A-52 residue ribosomal protein fusion product 1 | Homo sapiens |
| 6835 | surfeit 2 | Homo sapiens |
| 79709 | glycosyltransferase 25 domain containing 1 | Homo sapiens |
| 57045 | twisted gastrulation homolog 1 (Drosophila) | Homo sapiens |
| 311 | annexin A11 | Homo sapiens |
| 1024 | cyclin-dependent kinase 8 | Homo sapiens |
| 25927 | cannabinoid receptor interacting protein 1 | Homo sapiens |
| 257152 | hypothetical protein LOC257152 | Homo sapiens |
| 84996 | chromosome 21 open reading frame 119 | Homo sapiens |
| 57178 | zinc finger, MIZ-type containing 1 | Homo sapiens |
| 147339 | chromosome 18 open reading frame 25 | Homo sapiens |
| 29100 | transmembrane protein 208 | Homo sapiens |
| 9107 | myotubularin related protein 6 | Homo sapiens |
| 9391 | cytosolic iron-sulfur protein assembly 1 homolog (S. cerevisiae) | Homo sapiens |
| 6565 | solute carrier family 15 (H+/peptide transporter), member 2 | Homo sapiens |
| 64327 | limb region 1 homolog (mouse) | Homo sapiens |
| 6132 | ribosomal protein L8; ribosomal protein L8 pseudogene 2 | Homo sapiens |
| 56683 | chromosome 21 open reading frame 59 | Homo sapiens |
| 7016 | testis-specific kinase 1 | Homo sapiens |
| 6451 | SH3 domain binding glutamic acid-rich protein like | Homo sapiens |
| 91750 | lin-52 homolog (C. elegans) | Homo sapiens |
| 10239 | adaptor-related protein complex 3, sigma 2 subunit | Homo sapiens |
| 80862 | non-protein coding RNA 171; HLA complex P5 pseudogene 10 | Homo sapiens |
| 26130 | GTPase activating protein and VPS9 domains 1 | Homo sapiens |
| 7469 | Wolf-Hirschhorn syndrome candidate 2 | Homo sapiens |
| 10572 | SIVA1, apoptosis-inducing factor | Homo sapiens |
| 57693 | zinc finger protein 317 | Homo sapiens |
| 389362 | proteasome (prosome, macropain) assembly chaperone 4 | Homo sapiens |
| 3646 | eukaryotic translation initiation factor 3, subunit E | Homo sapiens |
| 27161 | eukaryotic translation initiation factor 2C, 2 | Homo sapiens |
| 6809 | syntaxin 3 | Homo sapiens |
| 387882 | chromosome 12 open reading frame 75 | Homo sapiens |
| 29899 | G-protein signaling modulator 2 (AGS3-like, C. elegans) | Homo sapiens |
| 2820 | glycerol-3-phosphate dehydrogenase 2 (mitochondrial) | Homo sapiens |
| 1730 | diaphanous homolog 2 (Drosophila) | Homo sapiens |
| 1616 | death-domain associated protein | Homo sapiens |
| 5594 | mitogen-activated protein kinase 1 | Homo sapiens |
| 100289678 | similar to zinc finger protein 30 | Homo sapiens |
| 5264 | phytanoyl-CoA 2-hydroxylase | Homo sapiens |

| | | |
|---|---|---|
| 54516 | mitochondrial translational release factor 1-like | Homo sapiens |
| 284385 | hypothetical protein LOC284385 | Homo sapiens |
| 55209 | SET domain containing 5 | Homo sapiens |
| 25963 | transmembrane protein 87A | Homo sapiens |
| 10279 | protease, serine, 16 (thymus) | Homo sapiens |
| 51000 | solute carrier family 35, member B3 | Homo sapiens |
| 4501 | metallothionein 1X | Homo sapiens |
| 5052 | peroxiredoxin 1 | Homo sapiens |
| 10765 | similar to Jumonji, AT rich interactive domain 1B (RBP2-like); lysine (K)-specific demethylase 5B | Homo sapiens |
| 116541 | mitochondrial ribosomal protein L54 | Homo sapiens |
| 6834 | surfeit 1 | Homo sapiens |
| 5335 | phospholipase C, gamma 1 | Homo sapiens |
| 5216 | profilin 1 | Homo sapiens |
| 81602 | cytidine and dCMP deaminase domain containing 1 | Homo sapiens |
| 51019 | coiled-coil domain containing 53 | Homo sapiens |
| 359845 | hypothetical protein LOC100133632; family with sequence similarity 101, member B | Homo sapiens |
| 285527 | FRY-like | Homo sapiens |
| 80347 | Coenzyme A synthase | Homo sapiens |
| 84243 | zinc finger, DHHC-type containing 18 | Homo sapiens |
| 55284 | ubiquitin-conjugating enzyme E2W (putative) | Homo sapiens |
| 54442 | potassium channel tetramerisation domain containing 5 | Homo sapiens |
| 51099 | abhydrolase domain containing 5 | Homo sapiens |
| 64219 | praja ring finger 1 | Homo sapiens |
| 987 | LPS-responsive vesicle trafficking, beach and anchor containing | Homo sapiens |
| 58515 | selenoprotein K; similar to HSPC297 | Homo sapiens |
| 285753 | chromosome 6 open reading frame 182 | Homo sapiens |
| 2530 | fucosyltransferase 8 (alpha (1,6) fucosyltransferase) | Homo sapiens |
| 7280 | tubulin, beta 2A | Homo sapiens |
| 9319 | thyroid hormone receptor interactor 13 | Homo sapiens |
| 9330 | general transcription factor IIIC, polypeptide 3, 102kDa | Homo sapiens |
| 84317 | coiled-coil domain containing 115 | Homo sapiens |
| 54993 | zinc finger and SCAN domain containing 2 | Homo sapiens |
| 4690 | NCK adaptor protein 1 | Homo sapiens |
| 1993 | ELAV (embryonic lethal, abnormal vision, Drosophila)-like 2 (Hu antigen B) | Homo sapiens |
| 10989 | inner membrane protein, mitochondrial (mitofilin) | Homo sapiens |
| 55970 | guanine nucleotide binding protein (G protein), gamma 12 | Homo sapiens |
| 254428 | solute carrier family 41, member 1 | Homo sapiens |
| 3918 | laminin, gamma 2 | Homo sapiens |
| 9331 | UDP-Gal:betaGlcNAc beta 1,4- galactosyltransferase, polypeptide 6 | Homo sapiens |
| 23557 | SNAP-associated protein | Homo sapiens |
| 10393 | anaphase promoting complex subunit 10; anaphase promoting complex subunit 10 pseudogene | Homo sapiens |
| 56993 | translocase of outer mitochondrial membrane 22 homolog (yeast) | Homo sapiens |
| 9043 | sperm associated antigen 9 | Homo sapiens |
| 54965 | phosphatidylinositol glycan anchor biosynthesis, class X | Homo sapiens |
| 9189 | zinc finger, BED-type containing 1 | Homo sapiens |
| 51634 | RNA binding motif protein, X-linked 2 | Homo sapiens |
| 5727 | patched homolog 1 (Drosophila) | Homo sapiens |
| 92610 | TRAF-interacting protein with forkhead-associated domain | Homo sapiens |
| 84909 | chromosome 9 open reading frame 3 | Homo sapiens |
| 308 | annexin A5 | Homo sapiens |
| 51016 | family with sequence similarity 158, member A | Homo sapiens |
| 2932 | glycogen synthase kinase 3 beta | Homo sapiens |
| 23597 | acyl-CoA thioesterase 9 | Homo sapiens |
| 5587 | protein kinase D1 | Homo sapiens |
| 23161 | sorting nexin 13 | Homo sapiens |
| 138162 | chromosome 9 open reading frame 116 | Homo sapiens |
| 152518 | nuclear transcription factor, X-box binding-like 1 | Homo sapiens |
| 64756 | ATP synthase mitochondrial F1 complex assembly factor 1 | Homo sapiens |
| 64802 | nicotinamide nucleotide adenylyltransferase 1 | Homo sapiens |
| 200205 | chromosome 1 open reading frame 69 | Homo sapiens |
| 57380 | MRS2 magnesium homeostasis factor homolog (S. cerevisiae) | Homo sapiens |
| 9962 | solute carrier family 23 (nucleobase transporters), member 2 | Homo sapiens |
| 84657 | chromosome 3 open reading frame 42 | Homo sapiens |
| 57460 | protein phosphatase 1H (PP2C domain containing) | Homo sapiens |
| 6045 | ring finger protein 2 | Homo sapiens |
| 8859 | serine/threonine kinase 19 | Homo sapiens |
| 23150 | FERM domain containing 4B | Homo sapiens |
| 6597 | SWI/SNF related, matrix associated, actin dependent regulator of chromatin, subfamily a, member 4 | Homo sapiens |
| 152185 | coiled-coil domain containing 52 | Homo sapiens |
| 1317 | solute carrier family 31 (copper transporters), member 1 | Homo sapiens |
| 57406 | abhydrolase domain containing 6 | Homo sapiens |
| 9453 | geranylgeranyl diphosphate synthase 1 | Homo sapiens |
| 22955 | sex comb on midleg homolog 1 (Drosophila) | Homo sapiens |
| 90624 | Lyrm7 homolog (mouse) | Homo sapiens |
| 1477 | cleavage stimulation factor, 3' pre-RNA, subunit 1, 50kDa | Homo sapiens |

| | | |
|---|---|---|
| 9353 | slit homolog 2 (Drosophila) | Homo sapiens |
| 23389 | mediator complex subunit 13-like | Homo sapiens |
| 3912 | laminin, beta 1 | Homo sapiens |
| 51585 | PCF11, cleavage and polyadenylation factor subunit, homolog (S. cerevisiae) | Homo sapiens |
| 100131607 | similar to ATPase, H+ transporting, lysosomal 14kD, V1 subunit F | Homo sapiens |
| 6231 | ribosomal protein S26 pseudogene 38; ribosomal protein S26 pseudogene 39; ribosomal protein S26 pseudogene 35; ribosomal protein S26 pseudogene 31; ribosomal protein S26 pseudogene 20; ribosomal protein S26 pseudogene 54; ribosomal protein S26 pseudogene 2; ribosomal protein S26 pseudogene 53; ribosomal protein S26 pseudogene 25; ribosomal protein S26 pseudogene 50; ribosomal protein S26 pseudogene 6; ribosomal protein S26 pseudogene 8; ribosomal protein S26 | Homo sapiens |
| 54512 | exosome component 4 | Homo sapiens |
| 4144 | methionine adenosyltransferase II, alpha | Homo sapiens |
| 26230 | T-cell lymphoma invasion and metastasis 2 | Homo sapiens |
| 8501 | solute carrier family 43, member 1 | Homo sapiens |
| 389831 | hypothetical gene supported by AL713796 | Homo sapiens |
| 90120 | chromosome 9 open reading frame 69 | Homo sapiens |
| 8518 | inhibitor of kappa light polypeptide gene enhancer in B-cells, kinase complex-associated protein | Homo sapiens |
| 57169 | zinc finger, NFX1-type containing 1 | Homo sapiens |
| 51335 | neugrin, neurite outgrowth associated | Homo sapiens |
| 283951 | chromosome 16 open reading frame 91 | Homo sapiens |
| 6605 | SWI/SNF related, matrix associated, actin dependent regulator of chromatin, subfamily e, member 1 | Homo sapiens |
| 114991 | zinc finger protein 618 | Homo sapiens |
| 5910 | RAP1, GTP-GDP dissociation stimulator 1 | Homo sapiens |
| 84072 | HORMA domain containing 1 | Homo sapiens |
| 158960 | hypothetical protein BC009467 | Homo sapiens |
| 708 | complement component 1, q subcomponent binding protein | Homo sapiens |
| 388753 | chromosome 1 open reading frame 31 | Homo sapiens |
| 9446 | glutathione S-transferase omega 1 | Homo sapiens |
| 991 | cell division cycle 20 homolog (S. cerevisiae) | Homo sapiens |
| 10771 | zinc finger, MYND domain containing 11 | Homo sapiens |
| 8672 | eukaryotic translation initiation factor 4 gamma, 3 | Homo sapiens |
| 441259 | PMS2 postmeiotic segregation increased 2 (S. cerevisiae)-like | Homo sapiens |
| 1161 | excision repair cross-complementing rodent repair deficiency, complementation group 8 | Homo sapiens |
| 51021 | mitochondrial ribosomal protein S16 | Homo sapiens |
| 10276 | neuroepithelial cell transforming 1 | Homo sapiens |
| 9867 | praja ring finger 2 | Homo sapiens |
| 23429 | RING1 and YY1 binding protein | Homo sapiens |
| 1778 | dynein, cytoplasmic 1, heavy chain 1 | Homo sapiens |
| 996 | cell division cycle 27 homolog (S. cerevisiae) | Homo sapiens |
| 51647 | family with sequence similarity 96, member B | Homo sapiens |
| 6733 | SFRS protein kinase 2 | Homo sapiens |
| 57708 | mesoderm induction early response 1 homolog (Xenopus laevis) | Homo sapiens |
| 81790 | ring finger protein 170 | Homo sapiens |
| 27230 | stress-associated endoplasmic reticulum protein 1 | Homo sapiens |
| 27244 | sestrin 1 | Homo sapiens |
| 401522 | hypothetical LOC401522 | Homo sapiens |
| 79269 | WD repeat domain 32 | Homo sapiens |
| 92 | activin A receptor, type IIA | Homo sapiens |
| 5352 | procollagen-lysine, 2-oxoglutarate 5-dioxygenase 2 | Homo sapiens |
| 51665 | ankyrin repeat and SOCS box-containing 1 | Homo sapiens |
| 84641 | hippocampus abundant transcript-like 1 | Homo sapiens |
| 112950 | mediator complex subunit 8 | Homo sapiens |
| 51187 | ribosomal L24 domain containing 1; similar to ribosomal protein L24-like | Homo sapiens |
| 54989 | zinc finger protein 770 | Homo sapiens |
| 54953 | chromosome 1 open reading frame 27 | Homo sapiens |
| 51588 | protein inhibitor of activated STAT, 4 | Homo sapiens |
| 27352 | small G protein signaling modulator 3 | Homo sapiens |
| 6999 | tryptophan 2,3-dioxygenase | Homo sapiens |
| 258010 | small VCP/p97-interacting protein | Homo sapiens |
| 57149 | LYR motif containing 1 | Homo sapiens |
| 9100 | ubiquitin specific peptidase 10 | Homo sapiens |
| 22995 | centrosomal protein 152kDa | Homo sapiens |
| 8202 | nuclear receptor coactivator 3 | Homo sapiens |
| 203 | adenylate kinase 1 | Homo sapiens |
| 79691 | queuine tRNA-ribosyltransferase domain containing 1 | Homo sapiens |
| 54806 | Abelson helper integration site 1 | Homo sapiens |
| 2824 | glycoprotein M6B | Homo sapiens |
| 27349 | malonyl CoA:ACP acyltransferase (mitochondrial) | Homo sapiens |
| 91582 | ribosomal protein S19 binding protein 1 | Homo sapiens |
| 195828 | zinc finger protein 367 | Homo sapiens |
| 79745 | CAP-GLY domain containing linker protein family, member 4 | Homo sapiens |
| 84078 | kelch repeat and BTB (POZ) domain containing 7 | Homo sapiens |
| 114826 | SET and MYND domain containing 4 | Homo sapiens |
| 200845 | potassium channel tetramerisation domain containing 6 | Homo sapiens |

| | | |
|---|---|---|
| 285382 | chromosome 3 open reading frame 70 | Homo sapiens |
| 50808 | adenylate kinase 3 | Homo sapiens |
| 6197 | ribosomal protein S6 kinase, 90kDa, polypeptide 3 | Homo sapiens |
| 55247 | nei endonuclease VIII-like 3 (E. coli) | Homo sapiens |
| 205 | adenylate kinase 3-like 2; adenylate kinase 3-like 1 | Homo sapiens |
| 57616 | teashirt zinc finger homeobox 3 | Homo sapiens |
| 84313 | vacuolar protein sorting 25 homolog (S. cerevisiae) | Homo sapiens |
| 55034 | molybdenum cofactor sulfurase | Homo sapiens |
| 25941 | chromosome 18 open reading frame 10 | Homo sapiens |
| 9110 | myotubularin related protein 4 | Homo sapiens |
| 55227 | leucine rich repeat containing 1 | Homo sapiens |
| 286527 | thymosin beta 15a; thymosin beta 15B | Homo sapiens |
| 11013 | thymosin beta 15a; thymosin beta 15B | Homo sapiens |
| 727849 | similar to cis-Golgi matrix protein GM130 | Homo sapiens |
| 728855 | hypothetical LOC728855 | Homo sapiens |
| 11017 | small nuclear ribonucleoprotein 27kDa (U4/U6.U5) | Homo sapiens |
| 201292 | tripartite motif-containing 65 | Homo sapiens |
| 56951 | chromosome 5 open reading frame 15 | Homo sapiens |
| 3693 | integrin, beta 5 | Homo sapiens |
| 55269 | paraspeckle component 1; paraspeckle protein 1 pseudogene | Homo sapiens |
| 57553 | microtubule associated monoxygenase, calponin and LIM domain containing 3 | Homo sapiens |
| 22990 | pecanex homolog (Drosophila) | Homo sapiens |
| 2060 | epidermal growth factor receptor pathway substrate 15 | Homo sapiens |
| 642852 | hypothetical LOC642852 | Homo sapiens |
| 813 | calumenin | Homo sapiens |
| 60672 | migration and invasion inhibitory protein | Homo sapiens |
| 4891 | solute carrier family 11 (proton-coupled divalent metal ion transporters), member 2 | Homo sapiens |
| 442075 | hypothetical LOC442075 | Homo sapiens |
| 56704 | junctophilin 1 | Homo sapiens |
| 8932 | methyl-CpG binding domain protein 2 | Homo sapiens |
| 91612 | churchill domain containing 1 | Homo sapiens |
| 1460 | lymphocyte antigen 6 complex, locus G5B; casein kinase 2, beta polypeptide | Homo sapiens |
| 57472 | CCR4-NOT transcription complex, subunit 6 | Homo sapiens |
| 80757 | transmembrane protein 121 | Homo sapiens |
| 9638 | fasciculation and elongation protein zeta 1 (zygin I) | Homo sapiens |
| 4605 | v-myb myeloblastosis viral oncogene homolog (avian)-like 2 | Homo sapiens |
| 55974 | recombination activating gene 1 activating protein 1 | Homo sapiens |
| 25819 | CCR4 carbon catabolite repression 4-like (S. cerevisiae) | Homo sapiens |
| 84190 | chromosome 12 open reading frame 26 | Homo sapiens |
| 7322 | ubiquitin-conjugating enzyme E2D 2 (UBC4/5 homolog, yeast) | Homo sapiens |
| 374378 | UDP-N-acetyl-alpha-D-galactosamine:polypeptide N-acetylgalactosaminyltransferase-like 4 | Homo sapiens |
| 10376 | hypothetical gene supported by AF081484; NM_006082; tubulin, alpha 1b | Homo sapiens |
| 22981 | ninein-like | Homo sapiens |
| 93 | activin A receptor, type IIB | Homo sapiens |
| 5715 | proteasome (prosome, macropain) 26S subunit, non-ATPase, 9 | Homo sapiens |
| 9532 | BCL2-associated athanogene 2 | Homo sapiens |
| 84261 | F-box and WD repeat domain containing 9 | Homo sapiens |
| 79174 | cysteine-rich with EGF-like domains 2 | Homo sapiens |
| 84142 | family with sequence similarity 175, member A | Homo sapiens |
| 7468 | Wolf-Hirschhorn syndrome candidate 1 | Homo sapiens |
| 79724 | zinc finger protein 768 | Homo sapiens |
| 114915 | chromosome 5 open reading frame 26 | Homo sapiens |
| 149041 | ring finger and CCCH-type zinc finger domains 1 | Homo sapiens |
| 9700 | extra spindle pole bodies homolog 1 (S. cerevisiae) | Homo sapiens |
| 4141 | methionyl-tRNA synthetase | Homo sapiens |
| 64236 | PDZ and LIM domain 2 (mystique) | Homo sapiens |
| 51524 | transmembrane protein 138 | Homo sapiens |
| 51322 | WW domain containing adaptor with coiled-coil | Homo sapiens |
| 55760 | DEAH (Asp-Glu-Ala-His) box polypeptide 32 | Homo sapiens |
| 26996 | G protein-coupled receptor 160 | Homo sapiens |
| 80856 | KIAA1715 | Homo sapiens |
| 55745 | MU-2/AP1M2 domain containing, death-inducing | Homo sapiens |
| 114885 | oxysterol binding protein-like 11 | Homo sapiens |
| 1350 | cytochrome c oxidase subunit VIIc | Homo sapiens |
| 89781 | Hermansky-Pudlak syndrome 4 | Homo sapiens |
| 54680 | zinc finger, HIT type 6 | Homo sapiens |
| 64858 | DNA cross-link repair 1B (PSO2 homolog, S. cerevisiae) | Homo sapiens |
| 116841 | synaptosomal-associated protein, 47kDa | Homo sapiens |
| 23598 | POZ (BTB) and AT hook containing zinc finger 1 | Homo sapiens |
| 57648 | KIAA1522 | Homo sapiens |
| 128977 | chromosome 22 open reading frame 39 | Homo sapiens |
| 10106 | similar to hCG2013701; CTD (carboxy-terminal domain, RNA polymerase II, polypeptide A) small phosphatase 2 | Homo sapiens |
| 344558 | SH3 domain containing ring finger 3 | Homo sapiens |
| 26262 | tetraspanin 17 | Homo sapiens |
| 10285 | survival motor neuron domain containing 1 | Homo sapiens |
| 3192 | heterogeneous nuclear ribonucleoprotein U (scaffold attachment factor A) | Homo sapiens |

| | | |
|---|---|---|
| 79365 | basic helix-loop-helix family, member e41 | Homo sapiens |
| 55140 | elongation protein 3 homolog (S. cerevisiae) | Homo sapiens |
| 26118 | WD repeat and SOCS box-containing 1 | Homo sapiens |
| 83939 | eukaryotic translation initiation factor 2A, 65kDa | Homo sapiens |
| 59283 | calcium channel, voltage-dependent, gamma subunit 8 | Homo sapiens |
| 9804 | similar to translocase of outer mitochondrial membrane 20 homolog; similar to mitochondrial outer membrane protein 19; translocase of outer mitochondrial membrane 20 homolog (yeast) | Homo sapiens |
| 23326 | ubiquitin specific peptidase 22 | Homo sapiens |
| 246184 | cell division cycle 26 homolog (S. cerevisiae); cell division cycle 26 homolog (S. cerevisiae) pseudogene | Homo sapiens |
| 389741 | hypothetical LOC728903; hypothetical locus MGC21881 | Homo sapiens |
| 728903 | hypothetical LOC728903; hypothetical locus MGC21881 | Homo sapiens |
| 170506 | DEAH (Asp-Glu-Ala-His) box polypeptide 36 | Homo sapiens |
| 54487 | DiGeorge syndrome critical region gene 8 | Homo sapiens |
| 92285 | zinc finger protein 585B | Homo sapiens |
| 729082 | hypothetical protein LOC729082 | Homo sapiens |
| 252839 | transmembrane protein 9 | Homo sapiens |
| 64778 | fibronectin type III domain containing 3B | Homo sapiens |
| 56945 | mitochondrial ribosomal protein S22 | Homo sapiens |
| 54145 | H2B histone family, member S | Homo sapiens |
| 9474 | ATG5 autophagy related 5 homolog (S. cerevisiae) | Homo sapiens |
| 5218 | PFTAIRE protein kinase 1 | Homo sapiens |
| 25940 | family with sequence similarity 98, member A | Homo sapiens |
| 11278 | Kruppel-like factor 12 | Homo sapiens |
| 9911 | transmembrane and coiled-coil domain family 2 | Homo sapiens |
| 149175 | mannosidase, endo-alpha-like | Homo sapiens |
| 10618 | trans-golgi network protein 2 | Homo sapiens |
| 29117 | bromodomain containing 7; bromodomain containing 7 pseudogene 2 | Homo sapiens |
| 51300 | chromosome 3 open reading frame 1 | Homo sapiens |
| 11228 | Ras association (RalGDS/AF-6) domain family (N-terminal) member 8 | Homo sapiens |
| 8634 | RNA terminal phosphate cyclase domain 1 | Homo sapiens |
| 57002 | chromosome 7 open reading frame 36 | Homo sapiens |
| 47 | ATP citrate lyase | Homo sapiens |
| 5922 | RAS p21 protein activator 2 | Homo sapiens |
| 2747 | glutamate dehydrogenase 2 | Homo sapiens |
| 51136 | ring finger protein, transmembrane 1 | Homo sapiens |
| 11168 | PC4 and SFRS1 interacting protein 1 | Homo sapiens |
| 2013 | epithelial membrane protein 2 | Homo sapiens |
| 22796 | component of oligomeric golgi complex 2 | Homo sapiens |
| 8402 | solute carrier family 25 (mitochondrial carrier; oxoglutarate carrier), member 11 | Homo sapiens |
| 23168 | Rtf1, Paf1/RNA polymerase II complex component, homolog (S. cerevisiae) | Homo sapiens |
| 728239 | melanoma antigen family D, 4B; melanoma antigen family D, 4 | Homo sapiens |
| 81557 | melanoma antigen family D, 4B; melanoma antigen family D, 4 | Homo sapiens |
| 9645 | microtubule associated monoxygenase, calponin and LIM domain containing 2 | Homo sapiens |
| 3916 | lysosomal-associated membrane protein 1 | Homo sapiens |
| 7581 | zinc finger protein 33A | Homo sapiens |
| 114818 | kelch-like 29 (Drosophila) | Homo sapiens |
| 9557 | chromodomain helicase DNA binding protein 1-like | Homo sapiens |
| 699 | budding uninhibited by benzimidazoles 1 homolog (yeast) | Homo sapiens |
| 9425 | chromodomain protein, Y-like | Homo sapiens |
| 7165 | tumor protein D52-like 2 | Homo sapiens |
| 9422 | zinc finger protein 264 | Homo sapiens |
| 7138 | troponin T type 1 (skeletal, slow) | Homo sapiens |
| 56938 | aryl hydrocarbon receptor nuclear translocator-like 2 | Homo sapiens |
| 10196 | protein arginine methyltransferase 3 | Homo sapiens |
| 10522 | deformed epidermal autoregulatory factor 1 (Drosophila) | Homo sapiens |
| 138151 | NACC family member 2, BEN and BTB (POZ) domain containing | Homo sapiens |
| 5771 | protein tyrosine phosphatase, non-receptor type 2 | Homo sapiens |
| 3476 | chromosome 14 open reading frame 19; immunoglobulin (CD79A) binding protein 1 | Homo sapiens |
| 91452 | acyl-Coenzyme A binding domain containing 5 | Homo sapiens |
| 51385 | zinc finger protein 589 | Homo sapiens |
| 9411 | Rho GTPase activating protein 29 | Homo sapiens |
| 51088 | kelch-like 5 (Drosophila) | Homo sapiens |
| 23603 | coronin, actin binding protein, 1C | Homo sapiens |
| 1649 | DNA-damage-inducible transcript 3 | Homo sapiens |
| 124995 | mitochondrial ribosomal protein L10 | Homo sapiens |
| 7798 | leucine zipper protein 1 | Homo sapiens |
| 3340 | N-deacetylase/N-sulfotransferase (heparan glucosaminyl) 1 | Homo sapiens |
| 26515 | fracture callus 1 homolog (rat) | Homo sapiens |
| 374920 | chromosome 19 open reading frame 68 | Homo sapiens |
| 51527 | chromosome 14 open reading frame 129 | Homo sapiens |
| 8795 | tumor necrosis factor receptor superfamily, member 10b | Homo sapiens |
| 84914 | zinc finger protein 587 | Homo sapiens |
| 51071 | 2-deoxyribose-5-phosphate aldolase homolog (C. elegans) | Homo sapiens |
| 55006 | tRNA methyltransferase 61 homolog B (S. cerevisiae) | Homo sapiens |
| 10019 | SH2B adaptor protein 3 | Homo sapiens |

| | | |
|---|---|---|
| 11222 | mitochondrial ribosomal protein L3 | Homo sapiens |
| 134218 | DnaJ (Hsp40) homolog, subfamily C, member 21 | Homo sapiens |
| 29085 | phosphohistidine phosphatase 1 | Homo sapiens |
| 79644 | steroid 5 alpha-reductase 3 | Homo sapiens |
| 6122 | ribosomal protein L3; similar to 60S ribosomal protein L3 (L4) | Homo sapiens |
| 490 | ATPase, Ca++ transporting, plasma membrane 1 | Homo sapiens |
| 51228 | glycolipid transfer protein; glycolipid transfer protein pseudogene 1 | Homo sapiens |
| 55149 | mitochondrial poly(A) polymerase | Homo sapiens |
| 93974 | ATPase inhibitory factor 1 | Homo sapiens |
| 55617 | taspase, threonine aspartase, 1 | Homo sapiens |
| 5147 | phosphodiesterase 6D, cGMP-specific, rod, delta | Homo sapiens |
| 1175 | adaptor-related protein complex 2, sigma 1 subunit | Homo sapiens |
| 1678 | translocase of inner mitochondrial membrane 8 homolog A (yeast) | Homo sapiens |
| 285755 | peptidylprolyl isomerase (cyclophilin)-like 6 | Homo sapiens |
| 6875 | TAF4b RNA polymerase II, TATA box binding protein (TBP)-associated factor, 105kDa | Homo sapiens |
| 10397 | N-myc downstream regulated 1 | Homo sapiens |
| 11164 | nudix (nucleoside diphosphate linked moiety X)-type motif 5 | Homo sapiens |
| 6629 | small nuclear ribonucleoprotein polypeptide B'' | Homo sapiens |
| 97 | acylphosphatase 1, erythrocyte (common) type | Homo sapiens |
| 2804 | golgin B1, golgi integral membrane protein | Homo sapiens |
| 83930 | STARD3 N-terminal like | Homo sapiens |
| 54165 | DCN1, defective in cullin neddylation 1, domain containing 1 (S. cerevisiae) | Homo sapiens |
| 8833 | guanine monphosphate synthetase | Homo sapiens |
| 84899 | transmembrane and tetratricopeptide repeat containing 4 | Homo sapiens |
| 51287 | coiled-coil-helix-coiled-coil-helix domain containing 8 | Homo sapiens |
| 148252 | DIRAS family, GTP-binding RAS-like 1 | Homo sapiens |
| 57556 | sema domain, transmembrane domain (TM), and cytoplasmic domain, (semaphorin) 6A | Homo sapiens |
| 440275 | eukaryotic translation initiation factor 2 alpha kinase 4 | Homo sapiens |
| 286410 | ATPase, class VI, type 11C | Homo sapiens |
| 4495 | metallothionein 1G | Homo sapiens |
| 9776 | KIAA0652 | Homo sapiens |
| 23473 | calpain 7 | Homo sapiens |
| 56942 | chromosome 16 open reading frame 61 | Homo sapiens |
| 6241 | ribonucleotide reductase M2 polypeptide | Homo sapiens |
| 2549 | GRB2-associated binding protein 1 | Homo sapiens |
| 57531 | HECT domain and ankyrin repeat containing, E3 ubiquitin protein ligase 1 | Homo sapiens |
| 27090 | ST6 (alpha-N-acetyl-neuraminyl-2,3-beta-galactosyl-1,3)-N-acetylgalactosaminide alpha-2,6-sialyltransferase 4 | Homo sapiens |
| 1399 | v-crk sarcoma virus CT10 oncogene homolog (avian)-like | Homo sapiens |
| 6881 | TAF10 RNA polymerase II, TATA box binding protein (TBP)-associated factor, 30kDa | Homo sapiens |
| 79786 | kelch-like 36 (Drosophila) | Homo sapiens |
| 84418 | chromosome 5 open reading frame 32 | Homo sapiens |
| 767558 | myotrophin; leucine zipper protein 6 | Homo sapiens |
| 136319 | myotrophin; leucine zipper protein 6 | Homo sapiens |
| 84191 | family with sequence similarity 96, member A | Homo sapiens |
| 58487 | CREB/ATF bZIP transcription factor | Homo sapiens |
| 641371 | acyl-CoA thioesterase 1 | Homo sapiens |
| 286451 | Yip1 domain family, member 6 | Homo sapiens |
| 10055 | SUMO1 activating enzyme subunit 1 | Homo sapiens |
| 6628 | small nuclear ribonucleoprotein polypeptides B and B1 | Homo sapiens |
| 10626 | tripartite motif-containing 16 | Homo sapiens |
| 64951 | mitochondrial ribosomal protein S24 | Homo sapiens |
| 94241 | tumor protein p53 inducible nuclear protein 1 | Homo sapiens |
| 32 | acetyl-Coenzyme A carboxylase beta | Homo sapiens |
| 51024 | fission 1 (mitochondrial outer membrane) homolog (S. cerevisiae) | Homo sapiens |
| 112724 | retinol dehydrogenase 13 (all-trans/9-cis) | Homo sapiens |
| 1783 | dynein, cytoplasmic 1, light intermediate chain 2 | Homo sapiens |
| 8405 | speckle-type POZ protein | Homo sapiens |
| 10094 | similar to actin related protein 2/3 complex subunit 3; hypothetical LOC729841; actin related protein 2/3 complex, subunit 3, 21kDa | Homo sapiens |
| 55591 | vezatin, adherens junctions transmembrane protein | Homo sapiens |
| 80306 | mediator complex subunit 28 | Homo sapiens |
| 10923 | SUB1 homolog (S. cerevisiae) | Homo sapiens |
| 125988 | chromosome 19 open reading frame 70 | Homo sapiens |
| 3202 | homeobox A5 | Homo sapiens |
| 55746 | nucleoporin 133kDa | Homo sapiens |
| 85313 | peptidylprolyl isomerase (cyclophilin)-like 4 | Homo sapiens |
| 6606 | survival of motor neuron 1, telomeric; survival of motor neuron 2, centromeric | Homo sapiens |
| 6607 | survival of motor neuron 1, telomeric; survival of motor neuron 2, centromeric | Homo sapiens |
| 55017 | chromosome 14 open reading frame 119 | Homo sapiens |
| 1353 | COX11 homolog, cytochrome c oxidase assembly protein (yeast) | Homo sapiens |
| 94107 | transmembrane protein 203 | Homo sapiens |
| 8500 | protein tyrosine phosphatase, receptor type, f polypeptide (PTPRF), interacting protein (liprin), alpha 1 | Homo sapiens |
| 819 | calcium modulating ligand | Homo sapiens |
| 91893 | ferredoxin-fold anticodon binding domain containing 1 | Homo sapiens |
| 10985 | GCN1 general control of amino-acid synthesis 1-like 1 (yeast) | Homo sapiens |

| | | |
|---|---|---|
| 29960 | FtsJ homolog 2 (E. coli) | Homo sapiens |
| 23243 | ankyrin repeat domain 28 | Homo sapiens |
| 51003 | mediator complex subunit 31 | Homo sapiens |
| 2280 | FK506 binding protein 1A, 12kDa | Homo sapiens |
| 9314 | Kruppel-like factor 4 (gut) | Homo sapiens |
| 171023 | additional sex combs like 1 (Drosophila) | Homo sapiens |
| 4300 | myeloid/lymphoid or mixed-lineage leukemia (trithorax homolog, Drosophila); translocated to, 3 | Homo sapiens |
| 11215 | A kinase (PRKA) anchor protein 11 | Homo sapiens |
| 4750 | NIMA (never in mitosis gene a)-related kinase 1 | Homo sapiens |
| 51728 | polymerase (RNA) III (DNA directed) polypeptide K, 12.3 kDa | Homo sapiens |
| 93081 | chromosome 13 open reading frame 27 | Homo sapiens |
| 995 | cell division cycle 25 homolog C (S. pombe) | Homo sapiens |
| 516 | ATP synthase, H+ transporting, mitochondrial F0 complex, subunit C1 (subunit 9) | Homo sapiens |
| 1994 | ELAV (embryonic lethal, abnormal vision, Drosophila)-like 1 (Hu antigen R) | Homo sapiens |
| 124446 | transmembrane protein 219 | Homo sapiens |
| 29079 | mediator complex subunit 4 | Homo sapiens |
| 7026 | nuclear receptor subfamily 2, group F, member 2 | Homo sapiens |
| 7334 | ubiquitin-conjugating enzyme E2N (UBC13 homolog, yeast) | Homo sapiens |
| 116985 | ArfGAP with RhoGAP domain, ankyrin repeat and PH domain 1 | Homo sapiens |
| 2987 | guanylate kinase 1 | Homo sapiens |
| 23637 | RAB GTPase activating protein 1 | Homo sapiens |
| 9868 | translocase of outer mitochondrial membrane 70 homolog A (S. cerevisiae) | Homo sapiens |
| 100131997 | family with sequence similarity 27, member E3; family with sequence similarity 27, member E2; family with sequence similarity 27, member E1; similar to Protein FAM27E2 | Homo sapiens |
| 2908 | nuclear receptor subfamily 3, group C, member 1 (glucocorticoid receptor) | Homo sapiens |
| 144348 | zinc finger protein 664 | Homo sapiens |
| 100130360 | hypothetical LOC100130360 | Homo sapiens |
| 5781 | protein tyrosine phosphatase, non-receptor type 11; similar to protein tyrosine phosphatase, non-receptor type 11 | Homo sapiens |
| 55716 | limb region 1 homolog (mouse)-like | Homo sapiens |
| 641 | Bloom syndrome, RecQ helicase-like | Homo sapiens |
| 11137 | PWP1 homolog (S. cerevisiae) | Homo sapiens |
| 10425 | ariadne homolog 2 (Drosophila) | Homo sapiens |
| 199857 | asparagine-linked glycosylation 14 homolog (S. cerevisiae) | Homo sapiens |
| 23232 | TBC1 domain family, member 12 | Homo sapiens |
| 131408 | family with sequence similarity 131, member A | Homo sapiens |
| 9049 | aryl hydrocarbon receptor interacting protein | Homo sapiens |
| 4682 | nucleotide binding protein 1 (MinD homolog, E. coli) | Homo sapiens |
| 285033 | hypothetical protein LOC285033 | Homo sapiens |
| 2029 | endosulfine alpha | Homo sapiens |
| 548645 | DnaJ (Hsp40) homolog, subfamily C , member 25; guanine nucleotide binding protein (G protein), gamma 10; DNAJC25-GNG10 readthrough transcript | Homo sapiens |
| 2790 | DnaJ (Hsp40) homolog, subfamily C , member 25; guanine nucleotide binding protein (G protein), gamma 10; DNAJC25-GNG10 readthrough transcript | Homo sapiens |
| 27257 | LSM1 homolog, U6 small nuclear RNA associated (S. cerevisiae) | Homo sapiens |
| 7411 | von Hippel-Lindau binding protein 1 | Homo sapiens |
| 134266 | GrpE-like 2, mitochondrial (E. coli) | Homo sapiens |
| 374500 | thrombospondin, type I, domain containing 1 pseudogene | Homo sapiens |
| 1389 | cAMP responsive element binding protein-like 2 | Homo sapiens |
| 400043 | hypothetical LOC400043 | Homo sapiens |
| 10483 | Sec23 homolog B (S. cerevisiae) | Homo sapiens |
| 80762 | Nedd4 family interacting protein 1 | Homo sapiens |
| 55596 | zinc finger, CCHC domain containing 8 | Homo sapiens |
| 10159 | ATPase, H+ transporting, lysosomal accessory protein 2 | Homo sapiens |
| 3267 | ArfGAP with FG repeats 1 | Homo sapiens |
| 4257 | microsomal glutathione S-transferase 1 | Homo sapiens |
| 23013 | spen homolog, transcriptional regulator (Drosophila) | Homo sapiens |
| 23019 | CCR4-NOT transcription complex, subunit 1 | Homo sapiens |
| 80321 | centrosomal protein 70kDa | Homo sapiens |
| 160418 | transmembrane and tetratricopeptide repeat containing 3 | Homo sapiens |
| 9567 | GTP binding protein 1 | Homo sapiens |
| 116461 | tRNA splicing endonuclease 15 homolog (S. cerevisiae) | Homo sapiens |
| 147007 | transmembrane protein 199 | Homo sapiens |
| 152189 | CKLF-like MARVEL transmembrane domain containing 8 | Homo sapiens |
| 10965 | acyl-CoA thioesterase 2 | Homo sapiens |
| 23080 | AVL9 homolog (S. cerevisiase) | Homo sapiens |
| 7915 | aldehyde dehydrogenase 5 family, member A1 | Homo sapiens |
| 57533 | TBC1 domain family, member 14 | Homo sapiens |
| 27348 | torsin family 1, member B (torsin B) | Homo sapiens |
| 54700 | RRN3 RNA polymerase I transcription factor homolog (S. cerevisiae) | Homo sapiens |
| 4725 | similar to NADH dehydrogenase (ubiquinone) Fe-S protein 5, 15kDa (NADH-coenzyme Q reductase); NADH dehydrogenase (ubiquinone) Fe-S protein 5, 15kDa (NADH-coenzyme Q reductase) | Homo sapiens |
| 11016 | activating transcription factor 7 | Homo sapiens |
| 158158 | RAS and EF-hand domain containing | Homo sapiens |
| 3159 | hypothetical LOC100130009; high mobility group AT-hook 1 | Homo sapiens |

| | | |
|---|---|---|
| 54892 | non-SMC condensin II complex, subunit G2 | Homo sapiens |
| 84962 | jub, ajuba homolog (Xenopus laevis) | Homo sapiens |
| 6785 | elongation of very long chain fatty acids (FEN1/Elo2, SUR4/Elo3, yeast)-like 4 | Homo sapiens |
| 2286 | FK506 binding protein 2, 13kDa | Homo sapiens |
| 7105 | tetraspanin 6 | Homo sapiens |
| 84337 | elongation factor 1 homolog (S. cerevisiae) | Homo sapiens |
| 219988 | protein associated with topoisomerase II homolog 1 (yeast) | Homo sapiens |
| 81571 | microRNA 600; chromosome 9 open reading frame 45 | Homo sapiens |
| 285343 | chromosome 3 open reading frame 23 | Homo sapiens |
| 81502 | histocompatibility (minor) 13 | Homo sapiens |
| 2114 | v-ets erythroblastosis virus E26 oncogene homolog 2 (avian) | Homo sapiens |
| 5504 | protein phosphatase 1, regulatory (inhibitor) subunit 2 | Homo sapiens |
| 2950 | glutathione S-transferase pi 1 | Homo sapiens |
| 26973 | cysteine and histidine-rich domain (CHORD)-containing 1; cysteine and histidine-rich domain (CHORD)-containing 1 pseudogene | Homo sapiens |
| 284611 | family with sequence similarity 102, member B | Homo sapiens |
| 51562 | MAP3K12 binding inhibitory protein 1 | Homo sapiens |
| 2074 | excision repair cross-complementing rodent repair deficiency, complementation group 6 | Homo sapiens |
| 26002 | monooxygenase, DBH-like 1 | Homo sapiens |
| 653566 | signal peptidase complex subunit 2 homolog (S. cerevisiae); signal peptidase complex subunit 2 homolog pseudogene | Homo sapiens |
| 9789 | signal peptidase complex subunit 2 homolog (S. cerevisiae); signal peptidase complex subunit 2 homolog pseudogene | Homo sapiens |
| 8165 | A kinase (PRKA) anchor protein 1 | Homo sapiens |
| 6728 | signal recognition particle 19kDa | Homo sapiens |
| 200933 | F-box protein 45 | Homo sapiens |
| 81532 | HCCA2 protein | Homo sapiens |
| 11056 | DEAD (Asp-Glu-Ala-Asp) box polypeptide 52 | Homo sapiens |
| 23270 | TSPY-like 4 | Homo sapiens |
| 23174 | zinc finger, CCHC domain containing 14 | Homo sapiens |
| 835 | caspase 2, apoptosis-related cysteine peptidase | Homo sapiens |
| 7555 | CCHC-type zinc finger, nucleic acid binding protein | Homo sapiens |
| 81892 | chromosome 14 open reading frame 156 | Homo sapiens |
| 374650 | golgi autoantigen, golgin subfamily a-like pseudogene | Homo sapiens |
| 642361 | hypothetical LOC642361 | Homo sapiens |
| 79939 | solute carrier family 35, member E1 | Homo sapiens |
| 373 | tripartite motif-containing 23 | Homo sapiens |
| 56647 | BRCA2 and CDKN1A interacting protein | Homo sapiens |
| 9882 | TBC1 domain family, member 4 | Homo sapiens |
| 6633 | small nuclear ribonucleoprotein D2 polypeptide 16.5kDa; similar to hCG2040270 | Homo sapiens |
| 83987 | coiled-coil domain containing 8 | Homo sapiens |
| 79090 | trafficking protein particle complex 6A | Homo sapiens |
| 22948 | chaperonin containing TCP1, subunit 5 (epsilon) | Homo sapiens |
| 147965 | family with sequence similarity 98, member C | Homo sapiens |
| 1487 | C-terminal binding protein 1 | Homo sapiens |
| 140735 | dynein, light chain, LC8-type 2 | Homo sapiens |
| 7317 | ubiquitin-like modifier activating enzyme 1 | Homo sapiens |
| 1739 | discs, large homolog 1 (Drosophila) | Homo sapiens |
| 5465 | peroxisome proliferator-activated receptor alpha | Homo sapiens |
| 4259 | microsomal glutathione S-transferase 3 | Homo sapiens |
| 2624 | GATA binding protein 2 | Homo sapiens |
| 7533 | tyrosine 3-monooxygenase/tryptophan 5-monooxygenase activation protein, eta polypeptide | Homo sapiens |
| 401431 | hypothetical LOC401431 | Homo sapiens |
| 402055 | SRR1 domain containing | Homo sapiens |
| 11244 | zinc fingers and homeoboxes 1 | Homo sapiens |
| 84722 | proline/serine-rich coiled-coil 1 | Homo sapiens |
| 2150 | coagulation factor II (thrombin) receptor-like 1 | Homo sapiens |
| 55421 | chromosome 17 open reading frame 85 | Homo sapiens |
| 10658 | CUG triplet repeat, RNA binding protein 1 | Homo sapiens |
| 6228 | ribosomal protein S23 | Homo sapiens |
| 10782 | zinc finger protein 274 | Homo sapiens |
| 53407 | syntaxin 18 | Homo sapiens |
| 8826 | IQ motif containing GTPase activating protein 1 | Homo sapiens |
| 55651 | NHP2 ribonucleoprotein homolog (yeast) | Homo sapiens |
| 3113 | major histocompatibility complex, class II, DP alpha 1 | Homo sapiens |
| 54954 | family with sequence similarity 120C | Homo sapiens |
| 11042 | glucuronidase, beta pseudogene | Homo sapiens |
| 51399 | trafficking protein particle complex 4 | Homo sapiens |
| 7775 | zinc finger protein 232 | Homo sapiens |
| 282809 | WD repeat domain 51B | Homo sapiens |
| 54939 | COMM domain containing 4 | Homo sapiens |
| 79047 | potassium channel tetramerisation domain containing 15 | Homo sapiens |
| 220906 | hypothetical protein LOC220906 | Homo sapiens |
| 134548 | ankyrin repeat domain 43 | Homo sapiens |
| 9530 | BCL2-associated athanogene 4 | Homo sapiens |
| 3091 | hypoxia inducible factor 1, alpha subunit (basic helix-loop-helix transcription factor) | Homo sapiens |
| 23085 | ELKS/RAB6-interacting/CAST family member 1 | Homo sapiens |

| | | |
|---|---|---|
| 28511 | NFKB inhibitor interacting Ras-like 2 | Homo sapiens |
| 23353 | unc-84 homolog A (C. elegans) | Homo sapiens |
| 23062 | golgi associated, gamma adaptin ear containing, ARF binding protein 2 | Homo sapiens |
| 130399 | activin A receptor, type IC | Homo sapiens |
| 4817 | nitrilase 1 | Homo sapiens |
| 4739 | neural precursor cell expressed, developmentally down-regulated 9 | Homo sapiens |
| 149076 | zinc finger protein 362 | Homo sapiens |
| 7570 | zinc finger protein 22 (KOX 15) | Homo sapiens |
| 84319 | chromosome 3 open reading frame 26 | Homo sapiens |
| 51426 | polymerase (DNA directed) kappa | Homo sapiens |
| 10523 | calcium homeostasis endoplasmic reticulum protein | Homo sapiens |
| 6249 | CAP-GLY domain containing linker protein 1 | Homo sapiens |
| 3145 | hydroxymethylbilane synthase | Homo sapiens |
| 22900 | caspase recruitment domain family, member 8 | Homo sapiens |
| 80199 | fuzzy homolog (Drosophila) | Homo sapiens |
| 83955 | nascent-polypeptide-associated complex alpha polypeptide pseudogene 1 | Homo sapiens |
| 55178 | RNA methyltransferase like 1 | Homo sapiens |
| 23136 | erythrocyte membrane protein band 4.1-like 3 | Homo sapiens |
| 9055 | protein regulator of cytokinesis 1 | Homo sapiens |
| 51388 | nuclear import 7 homolog (S. cerevisiae) | Homo sapiens |
| 63943 | FK506 binding protein like | Homo sapiens |
| 51523 | CXXC finger 5 | Homo sapiens |
| 83877 | TM2 domain containing 2 | Homo sapiens |
| 6493 | single-minded homolog 2 (Drosophila) | Homo sapiens |
| 79811 | SAFB-like, transcription modulator | Homo sapiens |
| 55636 | chromodomain helicase DNA binding protein 7 | Homo sapiens |
| 653820 | family with sequence similarity 72, member B | Homo sapiens |
| 9919 | SEC16 homolog A (S. cerevisiae) | Homo sapiens |
| 6046 | bromodomain containing 2 | Homo sapiens |
| 23609 | makorin ring finger protein 2 | Homo sapiens |
| 6319 | stearoyl-CoA desaturase (delta-9-desaturase) | Homo sapiens |
| 122704 | mitochondrial ribosomal protein L52 | Homo sapiens |
| 26235 | F-box and leucine-rich repeat protein 4 | Homo sapiens |
| 387723 | similar to hCG1648656 | Homo sapiens |
| 92597 | MOB1, Mps One Binder kinase activator-like 1A (yeast) | Homo sapiens |
| 91975 | zinc finger protein 300 | Homo sapiens |
| 54796 | basonuclin 2 | Homo sapiens |
| 10724 | meningioma expressed antigen 5 (hyaluronidase) | Homo sapiens |
| 153129 | solute carrier family 38, member 9 | Homo sapiens |
| 4152 | methyl-CpG binding domain protein 1 | Homo sapiens |
| 9063 | protein inhibitor of activated STAT, 2 | Homo sapiens |
| 285440 | cytochrome P450, family 4, subfamily V, polypeptide 2 | Homo sapiens |
| 7428 | von Hippel-Lindau tumor suppressor | Homo sapiens |
| 3550 | similar to CG18005; IK cytokine, down-regulator of HLA II | Homo sapiens |
| 6742 | single-stranded DNA binding protein 1 | Homo sapiens |
| 220002 | cytochrome b, ascorbate dependent 3 | Homo sapiens |
| 4833 | non-metastatic cells 4, protein expressed in | Homo sapiens |
| 10575 | chaperonin containing TCP1, subunit 4 (delta) | Homo sapiens |
| 4193 | Mdm2 p53 binding protein homolog (mouse) | Homo sapiens |
| 117145 | thioesterase superfamily member 4 | Homo sapiens |
| 493 | ATPase, Ca++ transporting, plasma membrane 4 | Homo sapiens |
| 29089 | ubiquitin-conjugating enzyme E2T (putative) | Homo sapiens |
| 5886 | RAD23 homolog A (S. cerevisiae) | Homo sapiens |
| 100289635 | similar to ZNF605 protein | Homo sapiens |
| 147699 | protein phosphatase 1B-like | Homo sapiens |
| 23587 | chromosome 17 open reading frame 81 | Homo sapiens |
| 6602 | SWI/SNF related, matrix associated, actin dependent regulator of chromatin, subfamily d, member 1 | Homo sapiens |
| 144233 | BCDIN3 domain containing | Homo sapiens |
| 6184 | ribophorin I | Homo sapiens |
| 94101 | ORM1-like 1 (S. cerevisiae) | Homo sapiens |
| 1984 | eukaryotic translation initiation factor 5A; eukaryotic translation initiation factor 5A-like 1 | Homo sapiens |
| 83933 | histone deacetylase 10 | Homo sapiens |
| 8725 | chromosome 19 open reading frame 2 | Homo sapiens |
| 3422 | isopentenyl-diphosphate delta isomerase 1 | Homo sapiens |
| 729218 | hypothetical LOC729218 | Homo sapiens |
| 5167 | ectonucleotide pyrophosphatase/phosphodiesterase 1 | Homo sapiens |
| 6857 | synaptotagmin I | Homo sapiens |
| 11234 | Hermansky-Pudlak syndrome 5 | Homo sapiens |
| 151648 | shugoshin-like 1 (S. pombe) | Homo sapiens |
| 51078 | THAP domain containing 4 | Homo sapiens |
| 221833 | Sp8 transcription factor | Homo sapiens |
| 8315 | BRCA1 associated protein | Homo sapiens |
| 51455 | REV1 homolog (S. cerevisiae) | Homo sapiens |
| 10133 | optineurin | Homo sapiens |
| 50862 | ring finger protein 141 | Homo sapiens |

| | | |
|---|---|---|
| 55720 | TSR1, 20S rRNA accumulation, homolog (S. cerevisiae) | Homo sapiens |
| 7284 | Tu translation elongation factor, mitochondrial | Homo sapiens |
| 5451 | POU class 2 homeobox 1 | Homo sapiens |
| 79071 | ELOVL family member 6, elongation of long chain fatty acids (FEN1/Elo2, SUR4/Elo3-like, yeast) | Homo sapiens |
| 54663 | WD repeat domain 74 | Homo sapiens |
| 6386 | syndecan binding protein (syntenin) | Homo sapiens |
| 4081 | mab-21-like 1 (C. elegans) | Homo sapiens |
| 142940 | TruB pseudouridine (psi) synthase homolog 1 (E. coli) | Homo sapiens |
| 653784 | family with sequence similarity 128, member A | Homo sapiens |
| 5229 | protein geranylgeranyltransferase type I, beta subunit | Homo sapiens |
| 8438 | RAD54-like (S. cerevisiae) | Homo sapiens |
| 9445 | integral membrane protein 2B | Homo sapiens |
| 84188 | fatty acyl CoA reductase 1 | Homo sapiens |
| 644873 | hypothetical LOC644873 | Homo sapiens |
| 285855 | ribosomal protein L7-like 1; ribosomal protein L7 pseudogene 14; ribosomal protein L7 pseudogene 21; ribosomal protein L7 pseudogene 22; ribosomal protein L7 pseudogene 46 | Homo sapiens |
| 3184 | heterogeneous nuclear ribonucleoprotein D (AU-rich element RNA binding protein 1, 37kDa) | Homo sapiens |
| 84193 | SET domain containing 3 | Homo sapiens |
| 10553 | HIV-1 Tat interactive protein 2, 30kDa | Homo sapiens |
| 26043 | UBX domain protein 7 | Homo sapiens |
| 23256 | sec1 family domain containing 1 | Homo sapiens |
| 517 | ATP synthase, H+ transporting, mitochondrial F0 complex, subunit C2 (subunit 9) | Homo sapiens |
| 829 | capping protein (actin filament) muscle Z-line, alpha 1 | Homo sapiens |
| 55851 | presenilin enhancer 2 homolog (C. elegans) | Homo sapiens |
| 5033 | prolyl 4-hydroxylase, alpha polypeptide I | Homo sapiens |
| 9798 | similar to CG10103; KIAA0174 | Homo sapiens |
| 25976 | TCDD-inducible poly(ADP-ribose) polymerase | Homo sapiens |
| 1910 | endothelin receptor type B | Homo sapiens |
| 23476 | bromodomain containing 4 | Homo sapiens |
| 10135 | nicotinamide phosphoribosyltransferase | Homo sapiens |
| 85007 | alanine-glyoxylate aminotransferase 2-like 2 | Homo sapiens |
| 6134 | ribosomal protein L10; ribosomal protein L10 pseudogene 15; ribosomal protein L10 pseudogene 6; ribosomal protein L10 pseudogene 16; ribosomal protein L10 pseudogene 9 | Homo sapiens |
| 85027 | MSTP150 | Homo sapiens |
| 6239 | ras responsive element binding protein 1 | Homo sapiens |
| 51735 | Rap guanine nucleotide exchange factor (GEF) 6 | Homo sapiens |
| 8260 | ARD1 homolog A, N-acetyltransferase (S. cerevisiae) | Homo sapiens |
| 6310 | ataxin 1 | Homo sapiens |
| 129563 | DIS3 mitotic control homolog (S. cerevisiae)-like 2 | Homo sapiens |
| 2289 | FK506 binding protein 5 | Homo sapiens |
| 54478 | family with sequence similarity 64, member A | Homo sapiens |
| 9766 | KIAA0247 | Homo sapiens |
| 59345 | guanine nucleotide binding protein (G protein), beta polypeptide 4 | Homo sapiens |
| 6464 | SHC (Src homology 2 domain containing) transforming protein 1 | Homo sapiens |
| 10156 | RAS p21 protein activator 4; RAS p21 protein activator 4 pseudogene | Homo sapiens |
| 3135 | major histocompatibility complex, class I, G | Homo sapiens |
| 9603 | nuclear factor (erythroid-derived 2)-like 3 | Homo sapiens |
| 1666 | 2,4-dienoyl CoA reductase 1, mitochondrial | Homo sapiens |
| 7046 | transforming growth factor, beta receptor 1 | Homo sapiens |
| 55159 | ring finger and WD repeat domain 3 | Homo sapiens |
| 4254 | KIT ligand | Homo sapiens |
| 51025 | mitochondria-associated protein involved in granulocyte-macrophage colony-stimulating factor signal transduction | Homo sapiens |
| 1457 | casein kinase 2, alpha 1 polypeptide pseudogene; casein kinase 2, alpha 1 polypeptide | Homo sapiens |
| 84969 | TOX high mobility group box family member 2 | Homo sapiens |
| 100131354 | hypothetical LOC100131354 | Homo sapiens |
| 8030 | coiled-coil domain containing 6 | Homo sapiens |
| 1164 | CDC28 protein kinase regulatory subunit 2 | Homo sapiens |
| 5214 | phosphofructokinase, platelet | Homo sapiens |
| 9857 | centrosomal protein 350kDa | Homo sapiens |
| 3692 | eukaryotic translation initiation factor 6 | Homo sapiens |
| 5997 | regulator of G-protein signaling 2, 24kDa | Homo sapiens |
| 200424 | tet oncogene family member 3 | Homo sapiens |
| 158219 | tetratricopeptide repeat domain 39B | Homo sapiens |
| 6208 | ribosomal protein S14 | Homo sapiens |
| 60626 | resistance to inhibitors of cholinesterase 8 homolog A (C. elegans) | Homo sapiens |
| 9988 | cyclin D binding myb-like transcription factor 1 | Homo sapiens |
| 79572 | ATPase type 13A3 | Homo sapiens |
| 84298 | LLP homolog, long-term synaptic facilitation (Aplysia); similar to LLP homolog; similar to hCG2013595 | Homo sapiens |
| 54883 | coiled-coil domain containing 49 | Homo sapiens |
| 64710 | nuclear casein kinase and cyclin-dependent kinase substrate 1 | Homo sapiens |
| 22873 | DAZ interacting protein 1 | Homo sapiens |
| 53827 | FXYD domain containing ion transport regulator 5 | Homo sapiens |
| 10772 | FUS interacting protein (serine/arginine-rich) 1; similar to FUS interacting protein (serine-arginine rich) 1 | Homo sapiens |

| | | |
|---|---|---|
| 902 | cyclin H | Homo sapiens |
| 107 | adenylate cyclase 1 (brain) | Homo sapiens |
| 800 | caldesmon 1 | Homo sapiens |
| 3150 | high-mobility group nucleosome binding domain 1 | Homo sapiens |
| 9767 | PHD finger protein 16 | Homo sapiens |
| 80727 | tweety homolog 3 (Drosophila) | Homo sapiens |
| 54918 | CKLF-like MARVEL transmembrane domain containing 6 | Homo sapiens |
| 5780 | protein tyrosine phosphatase, non-receptor type 9 | Homo sapiens |
| 10554 | 1-acylglycerol-3-phosphate O-acyltransferase 1 (lysophosphatidic acid acyltransferase, alpha) | Homo sapiens |
| 26155 | nucleolar complex associated 2 homolog (S. cerevisiae) | Homo sapiens |
| 729515 | chromosome 6 open reading frame 35; hCG1820764; tetratricopeptide repeat domain 28 | Homo sapiens |
| 23331 | chromosome 6 open reading frame 35; hCG1820764; tetratricopeptide repeat domain 28 | Homo sapiens |
| 100289098 | hypothetical protein LOC100289098 | Homo sapiens |
| 23531 | monocyte to macrophage differentiation-associated | Homo sapiens |
| 613 | breakpoint cluster region | Homo sapiens |
| 30836 | deoxynucleotidyltransferase, terminal, interacting protein 2 | Homo sapiens |
| 4782 | nuclear factor I/C (CCAAT-binding transcription factor) | Homo sapiens |
| 23291 | F-box and WD repeat domain containing 11 | Homo sapiens |
| 8324 | frizzled homolog 7 (Drosophila) | Homo sapiens |
| 4591 | tripartite motif-containing 37 | Homo sapiens |
| 6118 | replication protein A2, 32kDa | Homo sapiens |
| 55872 | PDZ binding kinase | Homo sapiens |
| 1355 | COX15 homolog, cytochrome c oxidase assembly protein (yeast) | Homo sapiens |
| 11044 | polymerase (DNA directed) sigma | Homo sapiens |
| 5162 | pyruvate dehydrogenase (lipoamide) beta | Homo sapiens |
| 1974 | similar to eukaryotic translation initiation factor 4A2; eukaryotic translation initiation factor 4A, isoform 2 | Homo sapiens |
| 10455 | peroxisomal D3,D2-enoyl-CoA isomerase | Homo sapiens |
| 7803 | protein tyrosine phosphatase type IVA, member 1 | Homo sapiens |
| 100287515 | similar to zinc finger protein 26 (KOX 20) | Homo sapiens |
| 6439 | surfactant protein B | Homo sapiens |
| 81575 | apolipoprotein L domain containing 1 | Homo sapiens |
| 8195 | McKusick-Kaufman syndrome | Homo sapiens |
| 3344 | forkhead box N2 | Homo sapiens |
| 7112 | thymopoietin | Homo sapiens |
| 400322 | hect domain and RLD 2 pseudogene 2; hect domain and RLD 2 pseudogene 3 | Homo sapiens |
| 63905 | mannosidase, beta A, lysosomal-like; similar to mannosidase, beta A, lysosomal-like | Homo sapiens |
| 4214 | mitogen-activated protein kinase kinase kinase 1 | Homo sapiens |
| 571 | BTB and CNC homology 1, basic leucine zipper transcription factor 1 | Homo sapiens |
| 10980 | COP9 constitutive photomorphogenic homolog subunit 6 (Arabidopsis) | Homo sapiens |
| 440423 | suppressor of zeste 12 homolog pseudogene | Homo sapiens |
| 84314 | transmembrane protein 107 | Homo sapiens |
| 9663 | lipin 2 | Homo sapiens |
| 84973 | small nucleolar RNA host gene 7 (non-protein coding) | Homo sapiens |
| 134957 | syntaxin binding protein 5 (tomosyn) | Homo sapiens |
| 22847 | zinc finger protein 507 | Homo sapiens |
| 60491 | NIF3 NGG1 interacting factor 3-like 1 (S. pombe) | Homo sapiens |
| 483 | ATPase, Na+/K+ transporting, beta 3 polypeptide | Homo sapiens |
| 66008 | trafficking protein, kinesin binding 2 | Homo sapiens |
| 6300 | mitogen-activated protein kinase 12 | Homo sapiens |
| 81627 | chromosome 1 open reading frame 25 | Homo sapiens |
| 57182 | ankyrin repeat domain 50 | Homo sapiens |
| 84928 | transmembrane protein 209 | Homo sapiens |
| 8555 | CDC14 cell division cycle 14 homolog B (S. cerevisiae) | Homo sapiens |
| 7644 | zinc finger protein 91 | Homo sapiens |
| 125228 | chromosome 18 open reading frame 19 | Homo sapiens |
| 10056 | phenylalanyl-tRNA synthetase, beta subunit | Homo sapiens |
| 29969 | MyoD family inhibitor domain containing | Homo sapiens |
| 83786 | FERM domain containing 8 | Homo sapiens |
| 2937 | glutathione synthetase | Homo sapiens |
| 54460 | mitochondrial ribosomal protein S21 | Homo sapiens |
| 4644 | myosin VA (heavy chain 12, myoxin) | Homo sapiens |
| 23075 | SWAP switching B-cell complex 70kDa subunit | Homo sapiens |
| 98 | acylphosphatase 2, muscle type | Homo sapiens |
| 114971 | protein tyrosine phosphatase, mitochondrial 1 | Homo sapiens |
| 29937 | neuron derived neurotrophic factor | Homo sapiens |
| 60674 | growth arrest-specific 5 (non-protein coding) | Homo sapiens |
| 127262 | tumor protein p63 regulated 1-like | Homo sapiens |
| 526 | ATPase, H+ transporting, lysosomal 56/58kDa, V1 subunit B2 | Homo sapiens |
| 6461 | Src homology 2 domain containing adaptor protein B | Homo sapiens |
| 7296 | thioredoxin reductase 1; hypothetical LOC100130902 | Homo sapiens |
| 2145 | enhancer of zeste homolog 1 (Drosophila) | Homo sapiens |
| 10237 | solute carrier family 35, member B1 | Homo sapiens |
| 648 | BMI1 polycomb ring finger oncogene | Homo sapiens |
| 122961 | iron-sulfur cluster assembly 2 homolog (S. cerevisiae) | Homo sapiens |
| 220963 | solute carrier family 16, member 9 (monocarboxylic acid transporter 9) | Homo sapiens |

| | | |
|---|---|---|
| 527 | ATPase, H+ transporting, lysosomal 16kDa, V0 subunit c | Homo sapiens |
| 129285 | KLRAQ motif containing 1 | Homo sapiens |
| 6856 | synaptophysin-like 1 | Homo sapiens |
| 55240 | STEAP family member 3 | Homo sapiens |
| 64848 | YTH domain containing 2 | Homo sapiens |
| 100287525 | hypothetical protein LOC100287525 | Homo sapiens |
| 3301 | DnaJ (Hsp40) homolog, subfamily A, member 1 | Homo sapiens |
| 23317 | DnaJ (Hsp40) homolog, subfamily C, member 13 | Homo sapiens |
| 10413 | Yes-associated protein 1, 65kDa | Homo sapiens |
| 347733 | tubulin, beta 2B | Homo sapiens |
| 126328 | NADH dehydrogenase (ubiquinone) 1 alpha subcomplex, 11, 14.7kDa | Homo sapiens |
| 51692 | cleavage and polyadenylation specific factor 3, 73kDa | Homo sapiens |
| 8678 | beclin 1, autophagy related | Homo sapiens |
| 23384 | cytospin A | Homo sapiens |
| 27246 | ring finger protein 115 | Homo sapiens |
| 2794 | guanine nucleotide binding protein-like 1 | Homo sapiens |
| 64425 | polymerase (RNA) I polypeptide E, 53kDa | Homo sapiens |
| 22872 | SEC31 homolog A (S. cerevisiae) | Homo sapiens |
| 7295 | thioredoxin | Homo sapiens |
| 6229 | ribosomal protein S24 | Homo sapiens |
| 4976 | optic atrophy 1 (autosomal dominant) | Homo sapiens |
| 123 | adipose differentiation-related protein | Homo sapiens |
| 55664 | cell division cycle 37 homolog (S. cerevisiae)-like 1 | Homo sapiens |
| 10153 | CCAAT/enhancer binding protein (C/EBP), zeta | Homo sapiens |
| 5631 | phosphoribosyl pyrophosphate synthetase 1; phosphoribosyl pyrophosphate synthetase 1-like 1 | Homo sapiens |
| 84816 | reticulon 4 interacting protein 1 | Homo sapiens |
| 64112 | modulator of apoptosis 1 | Homo sapiens |
| 9093 | DnaJ (Hsp40) homolog, subfamily A, member 3 | Homo sapiens |
| 375757 | chromosome 9 open reading frame 119 | Homo sapiens |
| 116150 | nuclear undecaprenyl pyrophosphate synthase 1 pseudogene; nuclear undecaprenyl pyrophosphate synthase 1 homolog (S. cerevisiae) | Homo sapiens |
| 25813 | sorting and assembly machinery component 50 homolog (S. cerevisiae) | Homo sapiens |
| 51608 | chromosome 7 open reading frame 20 | Homo sapiens |
| 51506 | ubiquitin-fold modifier conjugating enzyme 1 | Homo sapiens |
| 140465 | myosin, light chain 6B, alkali, smooth muscle and non-muscle | Homo sapiens |
| 23052 | endonuclease domain containing 1 | Homo sapiens |
| 64359 | nucleoredoxin | Homo sapiens |
| 10574 | chaperonin containing TCP1, subunit 7 (eta) | Homo sapiens |
| 8697 | cell division cycle 23 homolog (S. cerevisiae) | Homo sapiens |
| 55297 | coiled-coil domain containing 91 | Homo sapiens |
| 55122 | akirin 2 | Homo sapiens |
| 1535 | cytochrome b-245, alpha polypeptide | Homo sapiens |
| 84154 | brix domain containing 1 pseudogene; brix domain containing 1 | Homo sapiens |
| 57584 | Rho GTPase activating protein 21 | Homo sapiens |
| 151195 | cyclin Y-like 1 | Homo sapiens |
| 729614 | hypothetical LOC729614 | Homo sapiens |
| 197131 | ubiquitin protein ligase E3 component n-recognin 1 | Homo sapiens |
| 6019 | relaxin 2 | Homo sapiens |
| 57190 | selenoprotein N, 1 | Homo sapiens |
| 63892 | thyroid adenoma associated | Homo sapiens |
| 85865 | GTP-binding protein 10 (putative) | Homo sapiens |
| 85460 | zinc finger protein 518B | Homo sapiens |
| 1662 | DEAD (Asp-Glu-Ala-Asp) box polypeptide 10 | Homo sapiens |
| 5193 | peroxisomal biogenesis factor 12 | Homo sapiens |
| 56259 | catenin, beta like 1 | Homo sapiens |
| 55256 | acireductone dioxygenase 1 | Homo sapiens |
| 9967 | thyroid hormone receptor associated protein 3 | Homo sapiens |
| 22800 | related RAS viral (r-ras) oncogene homolog 2; similar to related RAS viral (r-ras) oncogene homolog 2 | Homo sapiens |
| 5912 | RAP2B, member of RAS oncogene family | Homo sapiens |
| 642 | bleomycin hydrolase | Homo sapiens |
| 8774 | N-ethylmaleimide-sensitive factor attachment protein, gamma | Homo sapiens |
| 55667 | DENN/MADD domain containing 4C | Homo sapiens |
| 6095 | RAR-related orphan receptor A | Homo sapiens |
| 1952 | cadherin, EGF LAG seven-pass G-type receptor 2 (flamingo homolog, Drosophila) | Homo sapiens |
| 79139 | Der1-like domain family, member 1 | Homo sapiens |
| 121274 | zinc finger protein 641 | Homo sapiens |
| 81603 | tripartite motif-containing 8 | Homo sapiens |
| 140707 | BRI3 binding protein | Homo sapiens |
| 7551 | zinc finger protein 3 | Homo sapiens |
| 79647 | akirin 1 | Homo sapiens |
| 471 | 5-aminoimidazole-4-carboxamide ribonucleotide formyltransferase/IMP cyclohydrolase | Homo sapiens |
| 8550 | mitogen-activated protein kinase-activated protein kinase 5 | Homo sapiens |
| 55183 | RAP1 interacting factor homolog (yeast) | Homo sapiens |
| 151903 | coiled-coil domain containing 12 | Homo sapiens |
| 23139 | microtubule associated serine/threonine kinase 2 | Homo sapiens |

| | | |
|---|---|---|
| 10592 | structural maintenance of chromosomes 2 | Homo sapiens |
| 36 | acyl-Coenzyme A dehydrogenase, short/branched chain | Homo sapiens |
| 10785 | WD repeat domain 4 | Homo sapiens |
| 80223 | RAB11 family interacting protein 1 (class I) | Homo sapiens |
| 6188 | ribosomal protein S3 pseudogene 3; ribosomal protein S3 | Homo sapiens |
| 30844 | EH-domain containing 4 | Homo sapiens |
| 10190 | thioredoxin domain containing 9 | Homo sapiens |
| 23032 | ubiquitin specific peptidase 33 | Homo sapiens |
| 9585 | kinesin family member 20B | Homo sapiens |
| 57486 | neurolysin (metallopeptidase M3 family) | Homo sapiens |
| 80219 | coenzyme Q10 homolog B (S. cerevisiae) | Homo sapiens |
| 55236 | ubiquitin-like modifier activating enzyme 6 | Homo sapiens |
| 284613 | cytochrome b-561 domain containing 1 | Homo sapiens |
| 51329 | ADP-ribosylation-like factor 6 interacting protein 4 | Homo sapiens |
| 4281 | midline 1 (Opitz/BBB syndrome) | Homo sapiens |
| 57561 | arrestin domain containing 3 | Homo sapiens |
| 23171 | glycerol-3-phosphate dehydrogenase 1-like | Homo sapiens |
| 51026 | golgi transport 1 homolog B (S. cerevisiae) | Homo sapiens |
| 5238 | phosphoglucomutase 3 | Homo sapiens |
| 28974 | chromosome 19 open reading frame 53 | Homo sapiens |
| 55605 | kinesin family member 21A | Homo sapiens |
| 64097 | erythrocyte membrane protein band 4.1 like 4A | Homo sapiens |
| 2521 | fusion (involved in t(12;16) in malignant liposarcoma) | Homo sapiens |
| 92703 | transmembrane protein 183A; transmembrane protein 183B | Homo sapiens |
| 653659 | transmembrane protein 183A; transmembrane protein 183B | Homo sapiens |
| 80204 | F-box protein 11 | Homo sapiens |
| 2059 | epidermal growth factor receptor pathway substrate 8 | Homo sapiens |
| 79006 | meteorin, glial cell differentiation regulator | Homo sapiens |
| 6156 | ribosomal protein L30 | Homo sapiens |
| 23268 | dynamin binding protein | Homo sapiens |
| 283070 | hypothetical LOC283070 | Homo sapiens |
| 57325 | CSRP2 binding protein | Homo sapiens |
| 79007 | dysbindin (dystrobrevin binding protein 1) domain containing 1 | Homo sapiens |
| 4282 | macrophage migration inhibitory factor (glycosylation-inhibiting factor) | Homo sapiens |
| 55599 | RNA-binding region (RNP1, RRM) containing 3 | Homo sapiens |
| 114908 | transmembrane protein 123 | Homo sapiens |
| 65265 | chromosome 8 open reading frame 33 | Homo sapiens |
| 132430 | poly(A) binding protein, cytoplasmic 4-like | Homo sapiens |
| 22882 | zinc fingers and homeoboxes 2 | Homo sapiens |
| 9922 | IQ motif and Sec7 domain 1 | Homo sapiens |
| 388135 | chromosome 15 open reading frame 59 | Homo sapiens |
| 79836 | LON peptidase N-terminal domain and ring finger 3 | Homo sapiens |
| 22822 | pleckstrin homology-like domain, family A, member 1 | Homo sapiens |
| 6731 | signal recognition particle 72kDa | Homo sapiens |
| 3052 | holocytochrome c synthase (cytochrome c heme-lyase) | Homo sapiens |
| 79885 | histone deacetylase 11 | Homo sapiens |
| 26005 | C2 calcium-dependent domain containing 3 | Homo sapiens |
| 4487 | msh homeobox 1 | Homo sapiens |
| 51130 | ankyrin repeat and SOCS box-containing 3 | Homo sapiens |
| 51106 | transcription factor B1, mitochondrial | Homo sapiens |
| 8577 | transmembrane protein with EGF-like and two follistatin-like domains 1; chromosome 9 open reading frame 30; hypothetical LOC729538 | Homo sapiens |
| 91283 | transmembrane protein with EGF-like and two follistatin-like domains 1; chromosome 9 open reading frame 30; hypothetical LOC729538 | Homo sapiens |
| 2956 | mutS homolog 6 (E. coli) | Homo sapiens |
| 23040 | myelin transcription factor 1-like | Homo sapiens |
| 9145 | synaptogyrin 1 | Homo sapiens |
| 51255 | ring finger protein 181 | Homo sapiens |
| 138241 | chromosome 9 open reading frame 85 | Homo sapiens |
| 7532 | tyrosine 3-monooxygenase/tryptophan 5-monooxygenase activation protein, gamma polypeptide | Homo sapiens |
| 55290 | BRF2, subunit of RNA polymerase III transcription initiation factor, BRF1-like | Homo sapiens |
| 6206 | ribosomal protein S12; ribosomal protein S12 pseudogene 4; ribosomal protein S12 pseudogene 11; ribosomal protein S12 pseudogene 9 | Homo sapiens |
| 6622 | synuclein, alpha (non A4 component of amyloid precursor) | Homo sapiens |
| 79178 | thiamine triphosphatase | Homo sapiens |
| 51053 | geminin, DNA replication inhibitor | Homo sapiens |
| 23248 | regulation of nuclear pre-mRNA domain containing 2 | Homo sapiens |
| 399821 | FLJ37035 protein | Homo sapiens |
| 10082 | glypican 6 | Homo sapiens |
| 55253 | tRNA-yW synthesizing protein 1 homolog (S. cerevisiae) | Homo sapiens |
| 10171 | RNA terminal phosphate cyclase-like 1 | Homo sapiens |
| 63908 | N-ethylmaleimide-sensitive factor attachment protein, beta | Homo sapiens |
| 23543 | RNA binding motif protein 9 | Homo sapiens |
| 1965 | eukaryotic translation initiation factor 2, subunit 1 alpha, 35kDa | Homo sapiens |
| 1345 | cytochrome c oxidase subunit VIc | Homo sapiens |
| 51363 | carbohydrate (N-acetylgalactosamine 4-sulfate 6-O) sulfotransferase 15 | Homo sapiens |

| | | |
|---|---|---|
| 2776 | guanine nucleotide binding protein (G protein), q polypeptide | Homo sapiens |
| 54520 | coiled-coil domain containing 93 | Homo sapiens |
| 65985 | acetoacetyl-CoA synthetase | Homo sapiens |
| 10381 | tubulin, beta 3; melanocortin 1 receptor (alpha melanocyte stimulating hormone receptor) | Homo sapiens |
| 57085 | angiotensin II receptor-associated protein | Homo sapiens |
| 161 | adaptor-related protein complex 2, alpha 2 subunit | Homo sapiens |
| 9112 | metastasis associated 1 | Homo sapiens |
| 78990 | OTU domain, ubiquitin aldehyde binding 2 | Homo sapiens |
| 55751 | transmembrane protein 184C | Homo sapiens |
| 79725 | THAP domain containing 9 | Homo sapiens |
| 353116 | Rab interacting lysosomal protein-like 1 | Homo sapiens |
| 729013 | hypothetical protein LOC729013 | Homo sapiens |
| 5316 | PBX/knotted 1 homeobox 1 | Homo sapiens |
| 256302 | chromosome 17 open reading frame 103 | Homo sapiens |
| 10940 | processing of precursor 1, ribonuclease P/MRP subunit (S. cerevisiae) | Homo sapiens |
| 54455 | F-box protein 42 | Homo sapiens |
| 93663 | Rho GTPase activating protein 18 | Homo sapiens |
| 6152 | ribosomal protein L24; ribosomal protein L24 pseudogene 6 | Homo sapiens |
| 22919 | microtubule-associated protein, RP/EB family, member 1 | Homo sapiens |
| 55748 | CNDP dipeptidase 2 (metallopeptidase M20 family) | Homo sapiens |
| 27316 | similar to RNA binding motif protein, X-linked; similar to hCG2011544; RNA binding motif protein, X-linked | Homo sapiens |
| 5194 | peroxisomal biogenesis factor 13 | Homo sapiens |
| 22974 | TPX2, microtubule-associated, homolog (Xenopus laevis) | Homo sapiens |
| 5530 | protein phosphatase 3 (formerly 2B), catalytic subunit, alpha isoform | Homo sapiens |
| 157693 | similar to hCG1735895; family with sequence similarity 87, member B; family with sequence similarity 87, member A | Homo sapiens |
| 54497 | HEAT repeat containing 5B | Homo sapiens |
| 286272 | hypothetical protein LOC286272 | Homo sapiens |
| 26512 | integrator complex subunit 6 | Homo sapiens |
| 84079 | ankyrin repeat domain 27 (VPS9 domain) | Homo sapiens |
| 116448 | oligodendrocyte transcription factor 1 | Homo sapiens |
| 51447 | inositol hexakisphosphate kinase 2 | Homo sapiens |
| 29952 | dipeptidyl-peptidase 7 | Homo sapiens |
| 57226 | LYR motif containing 2 | Homo sapiens |
| 51667 | negative regulator of ubiquitin-like proteins 1 | Homo sapiens |
| 84836 | abhydrolase domain containing 14B | Homo sapiens |
| 57407 | NmrA-like family domain containing 1 | Homo sapiens |
| 27344 | proprotein convertase subtilisin/kexin type 1 inhibitor | Homo sapiens |
| 389170 | leucine, glutamate and lysine rich 1 | Homo sapiens |
| 5550 | prolyl endopeptidase | Homo sapiens |
| 11015 | KDEL (Lys-Asp-Glu-Leu) endoplasmic reticulum protein retention receptor 3 | Homo sapiens |
| 993 | cell division cycle 25 homolog A (S. pombe) | Homo sapiens |
| 84552 | par-6 partitioning defective 6 homolog gamma (C. elegans) | Homo sapiens |
| 3236 | homeobox D10 | Homo sapiens |
| 84248 | forty-two-three domain containing 1 | Homo sapiens |
| 9416 | DEAD (Asp-Glu-Ala-Asp) box polypeptide 23 | Homo sapiens |
| 117854 | TRIM6-TRIM34 readthrough transcript; tripartite motif-containing 6; tripartite motif-containing 34 | Homo sapiens |
| 1745 | distal-less homeobox 1 | Homo sapiens |
| 127253 | tRNA-yW synthesizing protein 3 homolog (S. cerevisiae) | Homo sapiens |
| 79005 | sodium channel modifier 1 | Homo sapiens |
| 8683 | splicing factor, arginine/serine-rich 9 | Homo sapiens |
| 51454 | GULP, engulfment adaptor PTB domain containing 1 | Homo sapiens |
| 56898 | 3-hydroxybutyrate dehydrogenase, type 2 | Homo sapiens |
| 23524 | serine/arginine repetitive matrix 2; hypothetical LOC100132779 | Homo sapiens |
| 162972 | zinc finger protein 550 | Homo sapiens |
| 7430 | hypothetical protein LOC100129652; ezrin | Homo sapiens |
| 54969 | chromosome 4 open reading frame 27 | Homo sapiens |
| 9442 | similar to cofactor required for Sp1 transcriptional activation, subunit 8, 34kDa; mediator complex subunit 27; CRSP8 pseudogene | Homo sapiens |
| 133746 | junction mediating and regulatory protein, p53 cofactor | Homo sapiens |
| 9545 | RAB3D, member RAS oncogene family | Homo sapiens |
| 23125 | calmodulin binding transcription activator 2 | Homo sapiens |
| 56905 | chromosome 15 open reading frame 39 | Homo sapiens |
| 51185 | cereblon | Homo sapiens |
| 5438 | polymerase (RNA) II (DNA directed) polypeptide I, 14.5kDa | Homo sapiens |
| 1736 | dyskeratosis congenita 1, dyskerin | Homo sapiens |
| 339804 | chromosome 2 open reading frame 74 | Homo sapiens |
| 80335 | WD repeat domain 82 | Homo sapiens |
| 5387 | postmeiotic segregation increased 2-like 3; zinc finger protein 12 | Homo sapiens |
| 7559 | postmeiotic segregation increased 2-like 3; zinc finger protein 12 | Homo sapiens |
| 439938 | hypothetical gene supported by NM_178534 | Homo sapiens |
| 129138 | ankyrin repeat domain 54 | Homo sapiens |
| 182 | jagged 1 (Alagille syndrome) | Homo sapiens |
| 114803 | Myb-like, SWIRM and MPN domains 1 | Homo sapiens |
| 3376 | isoleucyl-tRNA synthetase | Homo sapiens |

| | | |
|---|---|---|
| 10301 | deleted in lymphocytic leukemia 1 (non-protein coding) | Homo sapiens |
| 9334 | UDP-Gal:betaGlcNAc beta 1,4- galactosyltransferase, polypeptide 5 | Homo sapiens |
| 83695 | chromosome 12 open reading frame 32 | Homo sapiens |
| 10549 | peroxiredoxin 4 | Homo sapiens |
| 80013 | chromosome 10 open reading frame 97 | Homo sapiens |
| 166785 | methylmalonic aciduria (cobalamin deficiency) cblA type | Homo sapiens |
| 146050 | zinc finger and SCAN domain containing 29 | Homo sapiens |
| 29066 | zinc finger CCCH-type containing 7A | Homo sapiens |
| 26985 | adaptor-related protein complex 3, mu 1 subunit | Homo sapiens |
| 55266 | transmembrane protein 19 | Homo sapiens |
| 51616 | TAF9B RNA polymerase II, TATA box binding protein (TBP)-associated factor, 31kDa | Homo sapiens |
| 1462 | versican | Homo sapiens |
| 4085 | MAD2 mitotic arrest deficient-like 1 (yeast) | Homo sapiens |
| 3146 | high-mobility group box 1; high-mobility group box 1-like 10 | Homo sapiens |
| 57117 | integrator complex subunit 12 | Homo sapiens |
| 4154 | muscleblind-like (Drosophila) | Homo sapiens |
| 2230 | ferredoxin 1 | Homo sapiens |
| 3309 | hypothetical gene supported by AF216292; NM_005347; heat shock 70kDa protein 5 (glucose-regulated protein, 78kDa) | Homo sapiens |
| 64432 | mitochondrial ribosomal protein S25 | Homo sapiens |
| 27341 | ribosomal RNA processing 7 homolog B (S. cerevisiae); ribosomal RNA processing 7 homolog A (S. cerevisiae) | Homo sapiens |
| 162239 | zinc finger protein 1 homolog (mouse) | Homo sapiens |
| 4297 | myeloid/lymphoid or mixed-lineage leukemia (trithorax homolog, Drosophila) | Homo sapiens |
| 64434 | nucleolar protein with MIF4G domain 1 | Homo sapiens |
| 57106 | N-acetyltransferase 14 (GCN5-related, putative) | Homo sapiens |
| 79567 | family with sequence similarity 65, member A | Homo sapiens |
| 10127 | zinc finger protein 263 | Homo sapiens |
| 51232 | cysteine rich transmembrane BMP regulator 1 (chordin-like) | Homo sapiens |
| 8790 | TNNI3 interacting kinase; fucose-1-phosphate guanylyltransferase | Homo sapiens |
| 1676 | DNA fragmentation factor, 45kDa, alpha polypeptide | Homo sapiens |
| 79731 | asparaginyl-tRNA synthetase 2, mitochondrial (putative) | Homo sapiens |
| 79711 | importin 4 | Homo sapiens |
| 54529 | asparagine synthetase domain containing 1 | Homo sapiens |
| 360023 | zinc finger and BTB domain containing 41 | Homo sapiens |
| 57720 | G protein-coupled receptor 107 | Homo sapiens |
| 287 | ankyrin 2, neuronal | Homo sapiens |
| 10109 | actin related protein 2/3 complex, subunit 2, 34kDa | Homo sapiens |
| 8445 | dual-specificity tyrosine-(Y)-phosphorylation regulated kinase 2 | Homo sapiens |
| 79066 | methyltransferase 10 domain containing | Homo sapiens |
| 59349 | kelch-like 12 (Drosophila) | Homo sapiens |
| 8544 | pirin (iron-binding nuclear protein) | Homo sapiens |
| 5981 | replication factor C (activator 1) 1, 145kDa | Homo sapiens |
| 644596 | non-protein coding RNA 86; non-protein coding RNA 87 | Homo sapiens |
| 399668 | non-protein coding RNA 86; non-protein coding RNA 87 | Homo sapiens |
| 472 | similar to Serine-protein kinase ATM (Ataxia telangiectasia mutated) (A-T, mutated); ataxia telangiectasia mutated | Homo sapiens |
| 80315 | cytoplasmic polyadenylation element binding protein 4 | Homo sapiens |
| 11007 | coiled-coil domain containing 85B | Homo sapiens |
| 57154 | SMAD specific E3 ubiquitin protein ligase 1 | Homo sapiens |
| 8934 | RAB7, member RAS oncogene family-like 1 | Homo sapiens |
| 3836 | karyopherin alpha 1 (importin alpha 5) | Homo sapiens |
| 57711 | zinc finger protein 529 | Homo sapiens |
| 51710 | zinc finger protein 44 | Homo sapiens |
| 27436 | echinoderm microtubule associated protein like 4 | Homo sapiens |
| 8668 | eukaryotic translation initiation factor 3, subunit I | Homo sapiens |
| 80067 | chromosome 2 open reading frame 37 | Homo sapiens |
| 128338 | DNA-damage regulated autophagy modulator 2 | Homo sapiens |
| 8793 | tumor necrosis factor receptor superfamily, member 10d, decoy with truncated death domain | Homo sapiens |
| 284244 | hypothetical protein LOC284244 | Homo sapiens |
| 54819 | zinc finger, CCHC domain containing 10 | Homo sapiens |
| 8780 | RIO kinase 3 (yeast) | Homo sapiens |
| 51275 | chromosome 12 open reading frame 47 | Homo sapiens |
| 81631 | microtubule-associated protein 1 light chain 3 beta | Homo sapiens |
| 5688 | proteasome (prosome, macropain) subunit, alpha type, 7 | Homo sapiens |
| 51115 | family with sequence similarity 82, member B | Homo sapiens |
| 22887 | forkhead box J3 | Homo sapiens |
| 56977 | storkhead box 2 | Homo sapiens |
| 25842 | ASF1 anti-silencing function 1 homolog A (S. cerevisiae) | Homo sapiens |
| 166647 | G protein-coupled receptor 125 | Homo sapiens |
| 55070 | de-etiolated homolog 1 (Arabidopsis) | Homo sapiens |
| 3597 | interleukin 13 receptor, alpha 1 | Homo sapiens |
| 3219 | homeobox B9 | Homo sapiens |
| 83468 | glycosyltransferase 8 domain containing 2 | Homo sapiens |
| 10799 | ribonuclease P/MRP 40kDa subunit | Homo sapiens |
| 9527 | golgi SNAP receptor complex member 1 | Homo sapiens |

| | | |
|---|---|---|
| 84525 | HOP homeobox | Homo sapiens |
| 162989 | death effector domain containing 2 | Homo sapiens |
| 54929 | transmembrane protein 161A | Homo sapiens |
| 26225 | ADP-ribosylation factor-like 5A | Homo sapiens |
| 5887 | RAD23 homolog B (S. cerevisiae) | Homo sapiens |
| 23527 | ArfGAP with coiled-coil, ankyrin repeat and PH domains 2 | Homo sapiens |
| 4172 | minichromosome maintenance complex component 3 | Homo sapiens |
| 55858 | transmembrane protein 165 | Homo sapiens |
| 5876 | Rab geranylgeranyltransferase, beta subunit | Homo sapiens |
| 23431 | adaptor-related protein complex 4, epsilon 1 subunit | Homo sapiens |
| 55260 | transmembrane protein 143 | Homo sapiens |
| 29093 | mitochondrial ribosomal protein L22 | Homo sapiens |
| 51715 | RAB23, member RAS oncogene family | Homo sapiens |
| 9632 | SEC24 family, member C (S. cerevisiae) | Homo sapiens |
| 2878 | glutathione peroxidase 3 (plasma) | Homo sapiens |
| 8890 | eukaryotic translation initiation factor 2B, subunit 4 delta, 67kDa | Homo sapiens |
| 619279 | zinc finger protein 704 | Homo sapiens |
| 9519 | TBP-like 1 | Homo sapiens |
| 55346 | t-complex 11 (mouse)-like 1 | Homo sapiens |
| 441212 | retinitis pigmentosa 9 pseudogene | Homo sapiens |
| 22889 | KIAA0907 | Homo sapiens |
| 10657 | KH domain containing, RNA binding, signal transduction associated 1 | Homo sapiens |
| 284593 | family with sequence similarity 41, member C | Homo sapiens |
| 5422 | polymerase (DNA directed), alpha 1, catalytic subunit | Homo sapiens |
| 29105 | chromosome 16 open reading frame 80 | Homo sapiens |
| 1105 | chromodomain helicase DNA binding protein 1 | Homo sapiens |
| 2260 | fibroblast growth factor receptor 1 | Homo sapiens |
| 92675 | histidyl-tRNA synthetase 2, mitochondrial (putative); D-tyrosyl-tRNA deacylase 1 homolog (S. cerevisiae) | Homo sapiens |
| 11177 | bromodomain adjacent to zinc finger domain, 1A | Homo sapiens |
| 25932 | chloride intracellular channel 4 | Homo sapiens |
| 4637 | myosin, light chain 6, alkali, smooth muscle and non-muscle | Homo sapiens |
| 85021 | RALBP1 associated Eps domain containing 1 | Homo sapiens |
| 404672 | general transcription factor IIH, polypeptide 5 | Homo sapiens |
| 3206 | homeobox A10 | Homo sapiens |
| 93099 | dermokine | Homo sapiens |
| 1033 | cyclin-dependent kinase inhibitor 3 | Homo sapiens |
| 124540 | musashi homolog 2 (Drosophila) | Homo sapiens |
| 157567 | ankyrin repeat domain 46 | Homo sapiens |
| 2941 | glutathione S-transferase alpha 4 | Homo sapiens |
| 51154 | mRNA turnover 4 homolog (S. cerevisiae) | Homo sapiens |
| 9975 | nuclear receptor subfamily 1, group D, member 2 | Homo sapiens |
| 284942 | ribosomal protein L23a pseudogene 25; ribosomal protein L23a pseudogene 82 | Homo sapiens |
| 949 | scavenger receptor class B, member 1 | Homo sapiens |
| 4728 | NADH dehydrogenase (ubiquinone) Fe-S protein 8, 23kDa (NADH-coenzyme Q reductase) | Homo sapiens |
| 10314 | LanC lantibiotic synthetase component C-like 1 (bacterial) | Homo sapiens |
| 54859 | chromosome 3 open reading frame 75 | Homo sapiens |
| 79954 | nucleolar protein 10 | Homo sapiens |
| 4659 | protein phosphatase 1, regulatory (inhibitor) subunit 12A | Homo sapiens |
| 9908 | GTPase activating protein (SH3 domain) binding protein 2 | Homo sapiens |
| 23095 | kinesin family member 1B | Homo sapiens |
| 7117 | thymosin-like 3 | Homo sapiens |
| 9749 | phosphatase and actin regulator 2 | Homo sapiens |
| 9124 | PDZ and LIM domain 1 | Homo sapiens |
| 203068 | tubulin, beta; similar to tubulin, beta 5; tubulin, beta pseudogene 2; tubulin, beta pseudogene 1 | Homo sapiens |
| 4082 | myristoylated alanine-rich protein kinase C substrate | Homo sapiens |
| 2752 | glutamate-ammonia ligase (glutamine synthetase) | Homo sapiens |
| 54433 | GAR1 ribonucleoprotein homolog (yeast) | Homo sapiens |
| 64839 | F-box and leucine-rich repeat protein 17 | Homo sapiens |
| 79147 | fukutin related protein | Homo sapiens |
| 7355 | solute carrier family 35 (UDP-galactose transporter), member A2 | Homo sapiens |
| 26061 | 2-hydroxyacyl-CoA lyase 1 | Homo sapiens |
| 55776 | chromosome 6 open reading frame 64 | Homo sapiens |
| 2821 | glucose phosphate isomerase | Homo sapiens |
| 55055 | Zwilch, kinetochore associated, homolog (Drosophila) | Homo sapiens |
| 11158 | RAB, member of RAS oncogene family-like 2B | Homo sapiens |
| 10206 | tripartite motif-containing 13 | Homo sapiens |
| 283459 | glutamyl-tRNA(Gln) amidotransferase, subunit C homolog (bacterial) | Homo sapiens |
| 116092 | deoxynucleotidyltransferase, terminal, interacting protein 1 | Homo sapiens |
| 81926 | family with sequence similarity 108, member A4; family with sequence similarity 108, member A5; family with sequence similarity 108, member A1 | Homo sapiens |
| 147694 | zinc finger protein 548 | Homo sapiens |
| 1894 | epithelial cell transforming sequence 2 oncogene | Homo sapiens |
| 8870 | immediate early response 3 | Homo sapiens |
| 65981 | caprin family member 2 | Homo sapiens |
| 205327 | chromosome 2 open reading frame 69 | Homo sapiens |

| | | |
|---|---|---|
| 54788 | DnaJ (Hsp40) homolog, subfamily B, member 12 | Homo sapiens |
| 30845 | EH-domain containing 3 | Homo sapiens |
| 84678 | lysine (K)-specific demethylase 2B | Homo sapiens |
| 23623 | RUN and SH3 domain containing 1 | Homo sapiens |
| 744 | metallophosphoesterase domain containing 2 | Homo sapiens |
| 80311 | kelch-like 15 (Drosophila) | Homo sapiens |
| 596 | B-cell CLL/lymphoma 2 | Homo sapiens |
| 4488 | msh homeobox 2 | Homo sapiens |
| 983 | cell division cycle 2, G1 to S and G2 to M | Homo sapiens |
| 10963 | stress-induced-phosphoprotein 1 | Homo sapiens |
| 7913 | DEK oncogene | Homo sapiens |
| 509 | ATP synthase, H+ transporting, mitochondrial F1 complex, gamma polypeptide 1 | Homo sapiens |
| 3638 | insulin induced gene 1 | Homo sapiens |
| 57475 | pleckstrin homology domain containing, family H (with MyTH4 domain) member 1 | Homo sapiens |
| 23172 | family with sequence similarity 175, member B | Homo sapiens |
| 2348 | folate receptor 1 (adult) | Homo sapiens |
| 387509 | G protein-coupled receptor 153 | Homo sapiens |
| 81609 | sorting nexin family member 27 | Homo sapiens |
| 4291 | myeloid leukemia factor 1 | Homo sapiens |
| 113763 | chromosome 7 open reading frame 29 | Homo sapiens |
| 85360 | synapse defective 1, Rho GTPase, homolog 1 (C. elegans) | Homo sapiens |
| 271 | adenosine monophosphate deaminase 2 (isoform L) | Homo sapiens |
| 374291 | NADH dehydrogenase (ubiquinone) Fe-S protein 7, 20kDa (NADH-coenzyme Q reductase) | Homo sapiens |
| 5198 | phosphoribosylformylglycinamidine synthase | Homo sapiens |
| 23318 | zinc finger, CCHC domain containing 11 | Homo sapiens |
| 256471 | major facilitator superfamily domain containing 8 | Homo sapiens |
| 285761 | discoidin, CUB and LCCL domain containing 1 | Homo sapiens |
| 3643 | insulin receptor | Homo sapiens |
| 4124 | mannosidase, alpha, class 2A, member 1 | Homo sapiens |
| 1479 | cleavage stimulation factor, 3' pre-RNA, subunit 3, 77kDa | Homo sapiens |
| 54927 | coiled-coil-helix-coiled-coil-helix domain containing 3 | Homo sapiens |
| 3977 | leukemia inhibitory factor receptor alpha | Homo sapiens |
| 10846 | phosphodiesterase 10A | Homo sapiens |
| 9175 | mitogen-activated protein kinase kinase kinase 13 | Homo sapiens |
| 120227 | cytochrome P450, family 2, subfamily R, polypeptide 1 | Homo sapiens |
| 658 | bone morphogenetic protein receptor, type IB | Homo sapiens |
| 26994 | ring finger protein 11 | Homo sapiens |
| 9636 | ISG15 ubiquitin-like modifier | Homo sapiens |
| 729092 | ArfGAP with GTPase domain, ankyrin repeat and PH domain 5; ArfGAP with GTPase domain, ankyrin repeat and PH domain 9; ArfGAP with GTPase domain, ankyrin repeat and PH domain 10; centaurin, gamma-like family, member 11 pseudogene | Homo sapiens |
| 728127 | ArfGAP with GTPase domain, ankyrin repeat and PH domain 5; ArfGAP with GTPase domain, ankyrin repeat and PH domain 9; ArfGAP with GTPase domain, ankyrin repeat and PH domain 10; centaurin, gamma-like family, member 11 pseudogene | Homo sapiens |
| 642517 | ArfGAP with GTPase domain, ankyrin repeat and PH domain 5; ArfGAP with GTPase domain, ankyrin repeat and PH domain 9; ArfGAP with GTPase domain, ankyrin repeat and PH domain 10; centaurin, gamma-like family, member 11 pseudogene | Homo sapiens |
| 51073 | mitochondrial ribosomal protein L4 | Homo sapiens |
| 26586 | cytoskeleton associated protein 2 | Homo sapiens |
| 57175 | coronin, actin binding protein, 1B | Homo sapiens |
| 9724 | UTP14, U3 small nucleolar ribonucleoprotein, homolog C (yeast); asparagine-linked glycosylation 11, alpha-1,2-mannosyltransferase homolog (yeast) | Homo sapiens |
| 84085 | F-box protein 30 | Homo sapiens |
| 10438 | C1D nuclear receptor co-repressor; similar to nuclear DNA-binding protein; similar to hCG1791993 | Homo sapiens |
| 54978 | chromosome 2 open reading frame 18 | Homo sapiens |
| 754 | pituitary tumor-transforming 1 interacting protein | Homo sapiens |
| 27332 | zinc finger protein 638 | Homo sapiens |
| 9805 | secernin 1 | Homo sapiens |
| 9848 | microfibrillar-associated protein 3-like | Homo sapiens |
| 9101 | ubiquitin specific peptidase 8 | Homo sapiens |
| 51230 | PHD finger protein 20 | Homo sapiens |
| 7398 | ubiquitin specific peptidase 1 | Homo sapiens |
| 257407 | chromosome 2 open reading frame 72 | Homo sapiens |
| 283464 | glycosyltransferase 8 domain containing 3 | Homo sapiens |
| 112840 | WD repeat domain 89 | Homo sapiens |
| 4478 | moesin | Homo sapiens |
| 83604 | transmembrane protein 47 | Homo sapiens |
| 51312 | solute carrier family 25, member 37 | Homo sapiens |
| 85458 | DIX domain containing 1 | Homo sapiens |
| 60558 | GUF1 GTPase homolog (S. cerevisiae) | Homo sapiens |
| 60682 | small ArfGAP 1 | Homo sapiens |
| 27292 | DIM1 dimethyladenosine transferase 1-like (S. cerevisiae) | Homo sapiens |
| 51429 | sorting nexin 9 | Homo sapiens |
| 22850 | ADNP homeobox 2 | Homo sapiens |
| 57404 | cytochrome P450, family 20, subfamily A, polypeptide 1 | Homo sapiens |
| 6223 | ribosomal protein S19 pseudogene 3; ribosomal protein S19 | Homo sapiens |

| | | |
|---|---|---|
| 222223 | KIAA1324-like | Homo sapiens |
| 64975 | mitochondrial ribosomal protein L41 | Homo sapiens |
| 84984 | chromosome 3 open reading frame 34 | Homo sapiens |
| 5701 | proteasome (prosome, macropain) 26S subunit, ATPase, 2 | Homo sapiens |
| 27075 | tetraspanin 13 | Homo sapiens |
| 29796 | ubiquinol-cytochrome c reductase complex (7.2 kD) | Homo sapiens |
| 115752 | DIS3 mitotic control homolog (S. cerevisiae)-like | Homo sapiens |
| 51067 | tyrosyl-tRNA synthetase 2, mitochondrial | Homo sapiens |
| 90480 | growth arrest and DNA-damage-inducible, gamma interacting protein 1 | Homo sapiens |
| 91 | activin A receptor, type IB | Homo sapiens |
| 54732 | transmembrane emp24 protein transport domain containing 9 | Homo sapiens |
| 29940 | dermatan sulfate epimerase | Homo sapiens |
| 93622 | hypothetical LOC93622 | Homo sapiens |
| 54707 | GPN-loop GTPase 2 | Homo sapiens |
| 81537 | sphingosine-1-phosphate phosphatase 1 | Homo sapiens |
| 51324 | spastic paraplegia 21 (autosomal recessive, Mast syndrome) | Homo sapiens |
| 84057 | meiotic nuclear divisions 1 homolog (S. cerevisiae) | Homo sapiens |
| 23051 | zinc fingers and homeoboxes 3 | Homo sapiens |
| 57224 | NHS-like 1 | Homo sapiens |
| 23424 | tudor domain containing 7 | Homo sapiens |
| 53944 | casein kinase 1, gamma 1 | Homo sapiens |
| 22826 | DnaJ (Hsp40) homolog, subfamily C, member 8 | Homo sapiens |
| 7153 | topoisomerase (DNA) II alpha 170kDa | Homo sapiens |
| 3066 | histone deacetylase 2 | Homo sapiens |
| 6284 | S100 calcium binding protein A13 | Homo sapiens |
| 57157 | putative homeodomain transcription factor 2 | Homo sapiens |
| 84769 | MPV17 mitochondrial membrane protein-like 2 | Homo sapiens |
| 79634 | secernin 3 | Homo sapiens |
| 57161 | pellino homolog 2 (Drosophila) | Homo sapiens |
| 521 | ATP synthase, H+ transporting, mitochondrial F0 complex, subunit E | Homo sapiens |
| 84131 | centrosomal protein 78kDa | Homo sapiens |
| 9328 | general transcription factor IIIC, polypeptide 5, 63kDa | Homo sapiens |
| 8100 | intraflagellar transport 88 homolog (Chlamydomonas) | Homo sapiens |
| 8614 | stanniocalcin 2 | Homo sapiens |
| 11216 | A kinase (PRKA) anchor protein 10 | Homo sapiens |
| 10079 | ATPase, class II, type 9A | Homo sapiens |
| 27000 | DnaJ (Hsp40) homolog, subfamily C, member 2 | Homo sapiens |
| 112752 | chromosome 14 open reading frame 179 | Homo sapiens |
| 9928 | kinesin family member 14 | Homo sapiens |
| 84287 | zinc finger, DHHC-type containing 16 | Homo sapiens |
| 23082 | peroxisome proliferator-activated receptor gamma, coactivator-related 1 | Homo sapiens |
| 60528 | elaC homolog 2 (E. coli) | Homo sapiens |
| 54874 | formin binding protein 1-like | Homo sapiens |
| 79077 | dCTP pyrophosphatase 1 | Homo sapiens |
| 54414 | sialic acid acetylesterase | Homo sapiens |
| 54778 | ring finger protein 111 | Homo sapiens |
| 9016 | solute carrier family 25 (mitochondrial carrier, brain), member 14 | Homo sapiens |
| 65986 | zinc finger and BTB domain containing 10 | Homo sapiens |
| 84960 | KIAA1984; transmembrane protein 141 | Homo sapiens |
| 9025 | ring finger protein 8 | Homo sapiens |
| 57684 | zinc finger and BTB domain containing 26 | Homo sapiens |
| 6730 | signal recognition particle 68kDa | Homo sapiens |
| 8563 | THO complex 5 | Homo sapiens |
| 63899 | NOL1/NOP2/Sun domain family, member 3 | Homo sapiens |
| 23585 | transmembrane protein 50A | Homo sapiens |
| 55131 | RNA binding motif protein 28 | Homo sapiens |
| 9925 | zinc finger and BTB domain containing 5 | Homo sapiens |
| 2263 | fibroblast growth factor receptor 2 | Homo sapiens |
| 586 | branched chain aminotransferase 1, cytosolic | Homo sapiens |
| 51606 | ATPase, H+ transporting, lysosomal 50/57kDa, V1 subunit H | Homo sapiens |
| 79893 | gametogenetin binding protein 2 | Homo sapiens |
| 10383 | tubulin, beta 2C | Homo sapiens |
| 692205 | small nucleolar RNA, C/D box 89 | Homo sapiens |
| 55660 | PRP40 pre-mRNA processing factor 40 homolog A (S. cerevisiae) | Homo sapiens |
| 51602 | NOP58 ribonucleoprotein homolog (yeast) | Homo sapiens |
| 339044 | polycystic kidney disease 1 (autosomal dominant) pseudogene 1 | Homo sapiens |
| 55623 | THUMP domain containing 1 | Homo sapiens |
| 4088 | SMAD family member 3 | Homo sapiens |
| 7335 | ubiquitin-conjugating enzyme E2 variant 1; ubiquitin-conjugating enzyme E2 variant 1 pseudogene 2; transmembrane protein 189; TMEM189-UBE2V1 readthrough transcript | Homo sapiens |
| 387522 | ubiquitin-conjugating enzyme E2 variant 1; ubiquitin-conjugating enzyme E2 variant 1 pseudogene 2; transmembrane protein 189; TMEM189-UBE2V1 readthrough transcript | Homo sapiens |
| 387521 | ubiquitin-conjugating enzyme E2 variant 1; ubiquitin-conjugating enzyme E2 variant 1 pseudogene 2; transmembrane protein 189; TMEM189-UBE2V1 readthrough transcript | Homo sapiens |
| 392 | Rho GTPase activating protein 1 | Homo sapiens |
| 5359 | phospholipid scramblase 1 | Homo sapiens |
| 10318 | TNFAIP3 interacting protein 1 | Homo sapiens |

| | | |
|---|---|---|
| 91574 | chromosome 12 open reading frame 65 | Homo sapiens |
| 8662 | eukaryotic translation initiation factor 3, subunit B | Homo sapiens |
| 79180 | EF-hand domain family, member D2 | Homo sapiens |
| 10186 | lipoma HMGIC fusion partner | Homo sapiens |
| 26190 | F-box and WD repeat domain containing 2 | Homo sapiens |
| 401264 | hypothetical gene supported by AK095117 | Homo sapiens |
| 6009 | Ras homolog enriched in brain | Homo sapiens |
| 6161 | small nucleolar RNA, H/ACA box 7A; small nucleolar RNA, H/ACA box 7B; ribosomal protein L32 | Homo sapiens |
| 148327 | cAMP responsive element binding protein 3-like 4 | Homo sapiens |
| 9508 | ADAM metallopeptidase with thrombospondin type 1 motif, 3 | Homo sapiens |
| 5289 | phosphoinositide-3-kinase, class 3 | Homo sapiens |
| 9920 | kelch repeat and BTB (POZ) domain containing 11 | Homo sapiens |
| 201161 | centromere protein V | Homo sapiens |
| 5935 | RNA binding motif (RNP1, RRM) protein 3 | Homo sapiens |
| 147166 | tripartite motif-containing 16-like | Homo sapiens |
| 7272 | TTK protein kinase | Homo sapiens |
| 10452 | translocase of outer mitochondrial membrane 40 homolog (yeast) | Homo sapiens |
| 152877 | family with sequence similarity 53, member A | Homo sapiens |
| 51669 | transmembrane protein 66 | Homo sapiens |
| 7529 | tyrosine 3-monooxygenase/tryptophan 5-monooxygenase activation protein, beta polypeptide | Homo sapiens |
| 113263 | glucocorticoid induced transcript 1 | Homo sapiens |
| 85002 | family with sequence similarity 86, member B2; family with sequence similarity 86, member A; family with sequence similarity 86, member B1 | Homo sapiens |
| 399664 | mex-3 homolog D (C. elegans) | Homo sapiens |
| 7884 | stem-loop binding protein | Homo sapiens |
| 4537 | NADH-ubiquinone oxidoreductase chain 3 | Homo sapiens |
| 2960 | general transcription factor IIE, polypeptide 1, alpha 56kDa | Homo sapiens |
| 112574 | sorting nexin 18 | Homo sapiens |
| 6601 | SWI/SNF related, matrix associated, actin dependent regulator of chromatin, subfamily c, member 2 | Homo sapiens |
| 10049 | DnaJ (Hsp40) homolog, subfamily B, member 6 | Homo sapiens |
| 11116 | FGFR1 oncogene partner | Homo sapiens |
| 286046 | XK, Kell blood group complex subunit-related family, member 6 | Homo sapiens |
| 5520 | protein phosphatase 2 (formerly 2A), regulatory subunit B, alpha isoform | Homo sapiens |
| 7965 | aminoacyl tRNA synthetase complex-interacting multifunctional protein 2; stromal antigen 3-like 3 | Homo sapiens |
| 10113 | prolactin regulatory element binding | Homo sapiens |
| 55505 | NOP10 ribonucleoprotein homolog (yeast) | Homo sapiens |
| 196441 | zinc finger, C3H1-type containing | Homo sapiens |
| 27101 | similar to calcyclin binding protein; calcyclin binding protein | Homo sapiens |
| 29896 | transformer 2 alpha homolog (Drosophila) | Homo sapiens |
| 54467 | ankyrin repeat and IBR domain containing 1 | Homo sapiens |
| 81606 | limb bud and heart development homolog (mouse) | Homo sapiens |
| 55536 | cell division cycle associated 7-like | Homo sapiens |
| 10959 | transmembrane emp24 domain trafficking protein 2 | Homo sapiens |
| 79591 | chromosome 10 open reading frame 76 | Homo sapiens |
| 2580 | cyclin G associated kinase | Homo sapiens |
| 83734 | ATG10 autophagy related 10 homolog (S. cerevisiae) | Homo sapiens |
| 3337 | DnaJ (Hsp40) homolog, subfamily B, member 1 | Homo sapiens |
| 92070 | chromosome 4 open reading frame 42 | Homo sapiens |
| 51141 | insulin induced gene 2 | Homo sapiens |
| 2531 | 3-ketodihydrosphingosine reductase | Homo sapiens |
| 148418 | sterile alpha motif domain containing 13 | Homo sapiens |
| 25836 | Nipped-B homolog (Drosophila) | Homo sapiens |
| 137392 | family with sequence similarity 92, member A2; family with sequence similarity 92, member A1 | Homo sapiens |
| 285550 | hypothetical protein LOC285550 | Homo sapiens |
| 7326 | ubiquitin-conjugating enzyme E2G 1 (UBC7 homolog, yeast) | Homo sapiens |
| 55234 | smu-1 suppressor of mec-8 and unc-52 homolog (C. elegans) | Homo sapiens |
| 23061 | TBC1 domain family, member 9B (with GRAM domain) | Homo sapiens |
| 1523 | cut-like homeobox 1 | Homo sapiens |
| 9727 | RAB11 family interacting protein 3 (class II) | Homo sapiens |
| 53938 | peptidylprolyl isomerase (cyclophilin)-like 3 | Homo sapiens |
| 57484 | ring finger protein 150 | Homo sapiens |
| 58480 | ras homolog gene family, member U | Homo sapiens |
| 901 | cyclin G2 | Homo sapiens |
| 55718 | polymerase (RNA) III (DNA directed) polypeptide E (80kD) | Homo sapiens |
| 151613 | tetratricopeptide repeat domain 14 | Homo sapiens |
| 57534 | mindbomb homolog 1 (Drosophila) | Homo sapiens |
| 55013 | coiled-coil domain containing 109B | Homo sapiens |
| 53373 | two pore segment channel 1 | Homo sapiens |
| 150465 | tubulin tyrosine ligase | Homo sapiens |
| 4705 | NADH dehydrogenase (ubiquinone) 1 alpha subcomplex, 10, 42kDa | Homo sapiens |
| 4338 | molybdenum cofactor synthesis 2 | Homo sapiens |
| 93621 | Mof4 family associated protein 1 | Homo sapiens |
| 57187 | THO complex 2 | Homo sapiens |

| | | |
|---|---|---|
| 11337 | GABA(A) receptor-associated protein | Homo sapiens |
| 23076 | ribosomal RNA processing 1 homolog B (S. cerevisiae) | Homo sapiens |
| 8894 | eukaryotic translation initiation factor 2, subunit 2 beta, 38kDa | Homo sapiens |
| 8869 | ST3 beta-galactoside alpha-2,3-sialyltransferase 5 | Homo sapiens |
| 100188893 | translocase of outer mitochondrial membrane 6 homolog (yeast) | Homo sapiens |
| 9813 | KIAA0494 | Homo sapiens |
| 26039 | synovial sarcoma translocation gene on chromosome 18-like 1 | Homo sapiens |
| 2760 | GM2 ganglioside activator | Homo sapiens |
| 826 | calpain, small subunit 1 | Homo sapiens |
| 65080 | mitochondrial ribosomal protein L44 | Homo sapiens |
| 149420 | PDLIM1 interacting kinase 1 like | Homo sapiens |
| 1019 | cyclin-dependent kinase 4 | Homo sapiens |
| 150771 | inositol 1,4,5-triphosphate receptor interacting protein-like 1 | Homo sapiens |
| 10813 | UTP14, U3 small nucleolar ribonucleoprotein, homolog A (yeast) | Homo sapiens |
| 10910 | SGT1, suppressor of G2 allele of SKP1 (S. cerevisiae) | Homo sapiens |
| 10336 | polycomb group ring finger 3 | Homo sapiens |
| 4216 | mitogen-activated protein kinase kinase kinase 4 | Homo sapiens |
| 8161 | coilin | Homo sapiens |
| 9296 | ATPase, H+ transporting, lysosomal 14kDa, V1 subunit F | Homo sapiens |
| 9114 | ATPase, H+ transporting, lysosomal 38kDa, V0 subunit d1 | Homo sapiens |
| 56990 | CDC42 small effector 2 | Homo sapiens |
| 10528 | NOP56 ribonucleoprotein homolog (yeast) | Homo sapiens |
| 283991 | family with sequence similarity 100, member B | Homo sapiens |
| 65110 | UPF3 regulator of nonsense transcripts homolog A (yeast) | Homo sapiens |
| 2773 | guanine nucleotide binding protein (G protein), alpha inhibiting activity polypeptide 3 | Homo sapiens |
| 10625 | influenza virus NS1A binding protein | Homo sapiens |
| 100128822 | hypothetical LOC100128822 | Homo sapiens |
| 440574 | chromosome 1 open reading frame 151 | Homo sapiens |
| 54619 | cyclin J | Homo sapiens |
| 221883 | HOXA11 antisense RNA (non-protein coding) | Homo sapiens |
| 51433 | anaphase promoting complex subunit 5 | Homo sapiens |
| 50807 | ArfGAP with SH3 domain, ankyrin repeat and PH domain 1 | Homo sapiens |
| 55789 | DEP domain containing 1B | Homo sapiens |
| 283899 | INO80 complex subunit E | Homo sapiens |
| 84804 | major facilitator superfamily domain containing 9 | Homo sapiens |
| 8189 | symplekin | Homo sapiens |
| 80153 | enhancer of mRNA decapping 3 homolog (S. cerevisiae) | Homo sapiens |
| 5828 | peroxisomal membrane protein 3, 35kDa | Homo sapiens |
| 84515 | minichromosome maintenance complex component 8 | Homo sapiens |
| 84134 | translocase of outer mitochondrial membrane 40 homolog (yeast)-like | Homo sapiens |
| 255520 | ELMO/CED-12 domain containing 2 | Homo sapiens |
| 10732 | transcription factor-like 5 (basic helix-loop-helix) | Homo sapiens |
| 89910 | ubiquitin protein ligase E3B | Homo sapiens |
| 10670 | Ras-related GTP binding A | Homo sapiens |
| 55829 | selenoprotein S | Homo sapiens |
| 694 | B-cell translocation gene 1, anti-proliferative | Homo sapiens |
| 6885 | mitogen-activated protein kinase kinase kinase 7 | Homo sapiens |
| 285636 | chromosome 5 open reading frame 51 | Homo sapiens |
| 25879 | WD repeats and SOF1 domain containing | Homo sapiens |
| 51552 | RAB14, member RAS oncogene family | Homo sapiens |
| 55101 | ATP5S-like | Homo sapiens |
| 5230 | phosphoglycerate kinase 1 | Homo sapiens |
| 5825 | ATP-binding cassette, sub-family D (ALD), member 3 | Homo sapiens |
| 2700 | gap junction protein, alpha 3, 46kDa | Homo sapiens |
| 203547 | VMA21 vacuolar H+-ATPase homolog (S. cerevisiae) | Homo sapiens |
| 5305 | phosphatidylinositol-5-phosphate 4-kinase, type II, alpha | Homo sapiens |
| 6873 | TAF2 RNA polymerase II, TATA box binding protein (TBP)-associated factor, 150kDa | Homo sapiens |
| 4528 | mitochondrial translational initiation factor 2 | Homo sapiens |
| 7347 | ubiquitin carboxyl-terminal esterase L3 (ubiquitin thiolesterase) | Homo sapiens |
| 1654 | DEAD (Asp-Glu-Ala-Asp) box polypeptide 3, X-linked | Homo sapiens |
| 84365 | MKI67 (FHA domain) interacting nucleolar phosphoprotein | Homo sapiens |
| 51022 | glutaredoxin 2 | Homo sapiens |
| 9611 | nuclear receptor co-repressor 1 | Homo sapiens |
| 9989 | protein phosphatase 4, regulatory subunit 1 | Homo sapiens |
| 25897 | ring finger protein 19A | Homo sapiens |
| 84553 | chromosome 6 open reading frame 168 | Homo sapiens |
| 171568 | polymerase (RNA) III (DNA directed) polypeptide H (22.9kD) | Homo sapiens |
| 60 | actin, beta | Homo sapiens |
| 5994 | regulatory factor X-associated protein | Homo sapiens |
| 8515 | integrin, alpha 10 | Homo sapiens |
| 5616 | protein kinase, Y-linked | Homo sapiens |
| 23041 | MON2 homolog (S. cerevisiae) | Homo sapiens |
| 54928 | inositol monophosphatase domain containing 1 | Homo sapiens |
| 4810 | Nance-Horan syndrome (congenital cataracts and dental anomalies) | Homo sapiens |
| 728066 | hypothetical protein MGC40405 pseudogene | Homo sapiens |
| 400027 | hypothetical protein LOC400027 | Homo sapiens |
| 6608 | smoothened homolog (Drosophila) | Homo sapiens |

| | | |
|---|---|---|
| 6041 | ribonuclease L (2',5'-oligoisoadenylate synthetase-dependent) | Homo sapiens |
| 84792 | chromosome 7 open reading frame 70 | Homo sapiens |
| 27185 | disrupted in schizophrenia 1 | Homo sapiens |
| 10552 | actin related protein 2/3 complex, subunit 1A, 41kDa | Homo sapiens |
| 7358 | UDP-glucose dehydrogenase | Homo sapiens |
| 23593 | heme binding protein 2 | Homo sapiens |
| 4163 | mutated in colorectal cancers | Homo sapiens |
| 80816 | additional sex combs like 3 (Drosophila) | Homo sapiens |
| 11104 | katanin p60 (ATPase-containing) subunit A 1 | Homo sapiens |
| 6400 | sel-1 suppressor of lin-12-like (C. elegans) | Homo sapiens |
| 8560 | degenerative spermatocyte homolog 1, lipid desaturase (Drosophila) | Homo sapiens |
| 57130 | ATPase type 13A1 | Homo sapiens |
| 55037 | Pentatricopeptide repeat domain 3 | Homo sapiens |
| 55727 | BTB (POZ) domain containing 7 | Homo sapiens |
| 10238 | WD repeat domain 68 | Homo sapiens |
| 5281 | phosphatidylinositol glycan anchor biosynthesis, class F | Homo sapiens |
| 284309 | zinc finger protein 776 | Homo sapiens |
| 208 | v-akt murine thymoma viral oncogene homolog 2 | Homo sapiens |
| 401397 | hypothetical LOC401397 | Homo sapiens |
| 3727 | jun D proto-oncogene | Homo sapiens |
| 2970 | general transcription factor II, i, pseudogene 1 | Homo sapiens |
| 285362 | sulfatase modifying factor 1 | Homo sapiens |
| 10447 | family with sequence similarity 3, member C | Homo sapiens |
| 169981 | spindlin family, member 3 | Homo sapiens |
| 8314 | BRCA1 associated protein-1 (ubiquitin carboxy-terminal hydrolase) | Homo sapiens |
| 388685 | hypothetical FLJ39739 | Homo sapiens |
| 9886 | Rho-related BTB domain containing 1 | Homo sapiens |
| 79627 | opioid growth factor receptor-like 1 | Homo sapiens |
| 55676 | solute carrier family 30 (zinc transporter), member 6 | Homo sapiens |
| 58533 | sorting nexin 6 | Homo sapiens |
| 6307 | sterol-C4-methyl oxidase-like | Homo sapiens |
| 3181 | heterogeneous nuclear ribonucleoprotein A2/B1 | Homo sapiens |
| 55322 | chromosome 5 open reading frame 22 | Homo sapiens |
| 6637 | hypothetical protein LOC100132425; similar to small nuclear ribonucleoprotein polypeptide G; small nuclear ribonucleoprotein polypeptide G; small nuclear ribonucleoprotein G-like protein | Homo sapiens |
| 440288 | similar to FLJ16518 protein | Homo sapiens |
| 56605 | ERO1-like beta (S. cerevisiae) | Homo sapiens |
| 8945 | beta-transducin repeat containing | Homo sapiens |
| 79622 | small nuclear ribonucleoprotein 25kDa (U11/U12) | Homo sapiens |
| 84058 | WD repeat domain 54 | Homo sapiens |
| 116447 | topoisomerase (DNA) I, mitochondrial | Homo sapiens |
| 10137 | RNA binding motif protein 12; copine I | Homo sapiens |
| 6949 | Treacher Collins-Franceschetti syndrome 1 | Homo sapiens |
| 5962 | radixin | Homo sapiens |
| 201163 | folliculin | Homo sapiens |
| 1663 | DEAD/H (Asp-Glu-Ala-Asp/His) box polypeptide 11 (CHL1-like helicase homolog, S. cerevisiae) | Homo sapiens |
| 10899 | jumping translocation breakpoint | Homo sapiens |
| 9156 | exonuclease 1 | Homo sapiens |
| 92595 | zinc finger protein 764 | Homo sapiens |
| 64843 | ISL LIM homeobox 2 | Homo sapiens |
| 10009 | zinc finger and BTB domain containing 33 | Homo sapiens |
| 6222 | ribosomal protein S18 pseudogene 12; ribosomal protein S18 pseudogene 5; ribosomal protein S18 | Homo sapiens |
| 4666 | nascent polypeptide-associated complex alpha subunit | Homo sapiens |
| 10211 | flotillin 1 | Homo sapiens |
| 51596 | cutA divalent cation tolerance homolog (E. coli) | Homo sapiens |
| 5861 | RAB1A, member RAS oncogene family | Homo sapiens |
| 51441 | YTH domain family, member 2 | Homo sapiens |
| 7453 | tryptophanyl-tRNA synthetase | Homo sapiens |
| 79102 | ring finger protein 26 | Homo sapiens |
| 25875 | LETM1 domain containing 1 | Homo sapiens |
| 4736 | ribosomal protein L10a pseudogene 6; ribosomal protein L10a; ribosomal protein L10a pseudogene 9 | Homo sapiens |
| 9060 | 3'-phosphoadenosine 5'-phosphosulfate synthase 2 | Homo sapiens |
| 29954 | protein-O-mannosyltransferase 2 | Homo sapiens |
| 11123 | RCAN family member 3 | Homo sapiens |
| 9554 | SEC22 vesicle trafficking protein homolog B (S. cerevisiae) | Homo sapiens |
| 729852 | hypothetical protein LOC729852 | Homo sapiens |
| 9880 | zinc finger and BTB domain containing 39 | Homo sapiens |
| 60680 | bruno-like 5, RNA binding protein (Drosophila) | Homo sapiens |
| 6187 | ribosomal protein S2 pseudogene 8; ribosomal protein S2 pseudogene 11; ribosomal protein S2 pseudogene 5; ribosomal protein S2 pseudogene 12; ribosomal protein S2 pseudogene 51; ribosomal protein S2 pseudogene 17; ribosomal protein S2 pseudogene 55; ribosomal protein S2 pseudogene 20; ribosomal protein S2 | Homo sapiens |
| 123775 | chromosome 16 open reading frame 46 | Homo sapiens |

| | | |
|---|---|---|
| 64785 | GINS complex subunit 3 (Psf3 homolog) | Homo sapiens |
| 6259 | RYK receptor-like tyrosine kinase | Homo sapiens |
| 81533 | integrin alpha FG-GAP repeat containing 1 | Homo sapiens |
| 23753 | stromal cell-derived factor 2-like 1 | Homo sapiens |
| 5921 | RAS p21 protein activator (GTPase activating protein) 1 | Homo sapiens |
| 284889 | hypothetical protein LOC284889 | Homo sapiens |
| 51522 | transmembrane protein 14C | Homo sapiens |
| 57212 | KIAA0495 | Homo sapiens |
| 285464 | cysteine-rich PAK1 inhibitor | Homo sapiens |
| 22879 | MON1 homolog B (yeast) | Homo sapiens |
| 158471 | prune homolog 2 (Drosophila) | Homo sapiens |
| 51008 | activating signal cointegrator 1 complex subunit 1 | Homo sapiens |
| 3148 | high-mobility group box 2 | Homo sapiens |
| 403313 | phosphatidic acid phosphatase type 2 domain containing 2 | Homo sapiens |
| 4179 | CD46 molecule, complement regulatory protein | Homo sapiens |
| 2067 | excision repair cross-complementing rodent repair deficiency, complementation group 1 (includes overlapping antisense sequence) | Homo sapiens |
| 152573 | shisa homolog 3 (Xenopus laevis) | Homo sapiens |
| 57621 | zinc finger and BTB domain containing 2 | Homo sapiens |
| 55567 | dynein, axonemal, heavy chain 3 | Homo sapiens |
| 202781 | hypothetical protein LOC202781 | Homo sapiens |
| 80298 | MTERF domain containing 3 | Homo sapiens |
| 23036 | zinc finger protein 292 | Homo sapiens |
| 79694 | mannosidase, endo-alpha | Homo sapiens |
| 7109 | trafficking protein particle complex 10 | Homo sapiens |
| 83787 | armadillo repeat containing 10 | Homo sapiens |
| 60468 | BTB and CNC homology 1, basic leucine zipper transcription factor 2 | Homo sapiens |
| 253512 | solute carrier family 25, member 30 | Homo sapiens |
| 136051 | zinc finger protein 786 | Homo sapiens |
| 152007 | GLI pathogenesis-related 2 | Homo sapiens |
| 64746 | acyl-Coenzyme A binding domain containing 3 | Homo sapiens |
| 149986 | LSM14B, SCD6 homolog B (S. cerevisiae) | Homo sapiens |
| 5607 | mitogen-activated protein kinase kinase 5 | Homo sapiens |
| 497661 | chromosome 18 open reading frame 32 | Homo sapiens |
| 10277 | ubiquitination factor E4B (UFD2 homolog, yeast) | Homo sapiens |
| 8636 | Sjogren syndrome nuclear autoantigen 1 | Homo sapiens |
| 8853 | ArfGAP with SH3 domain, ankyrin repeat and PH domain 2 | Homo sapiens |
| 51382 | ATPase, H+ transporting, lysosomal 34kDa, V1 subunit D | Homo sapiens |
| 57456 | KIAA1143 | Homo sapiens |
| 728153 | family with sequence similarity 133, member B pseudogene; similar to FAM133B protein; family with sequence similarity 133, member B | Homo sapiens |
| 257415 | family with sequence similarity 133, member B pseudogene; similar to FAM133B protein; family with sequence similarity 133, member B | Homo sapiens |
| 2271 | fumarate hydratase | Homo sapiens |
| 8303 | stannin | Homo sapiens |
| 2176 | Fanconi anemia, complementation group C | Homo sapiens |
| 84640 | ubiquitin specific peptidase 38 | Homo sapiens |
| 217 | aldehyde dehydrogenase 2 family (mitochondrial) | Homo sapiens |
| 5116 | pericentrin | Homo sapiens |
| 1106 | chromodomain helicase DNA binding protein 2 | Homo sapiens |
| 80143 | suppressor of IKK epsilon | Homo sapiens |
| 9354 | ubiquitination factor E4A (UFD2 homolog, yeast) | Homo sapiens |
| 100288525 | hypothetical protein LOC100288525 | Homo sapiens |
| 50809 | heterochromatin protein 1, binding protein 3 | Homo sapiens |
| 723790 | histone cluster 2, H2aa3; histone cluster 2, H2aa4 | Homo sapiens |
| 8337 | histone cluster 2, H2aa3; histone cluster 2, H2aa4 | Homo sapiens |
| 3218 | homeobox B8 | Homo sapiens |
| 57599 | WD repeat domain 48 | Homo sapiens |
| 7110 | TATA element modulatory factor 1 | Homo sapiens |
| 5654 | HtrA serine peptidase 1 | Homo sapiens |
| 7879 | RAB7A, member RAS oncogene family | Homo sapiens |
| 8533 | COP9 constitutive photomorphogenic homolog subunit 3 (Arabidopsis) | Homo sapiens |
| 6303 | spermidine/spermine N1-acetyltransferase 1 | Homo sapiens |
| 116224 | family with sequence similarity 122A | Homo sapiens |
| 168451 | THAP domain containing 5 | Homo sapiens |
| 81624 | diaphanous homolog 3 (Drosophila) | Homo sapiens |
| 9678 | PHD finger protein 14 | Homo sapiens |
| 57673 | BEN domain containing 3 | Homo sapiens |
| 54876 | chromosome 4 open reading frame 30 | Homo sapiens |
| 161424 | chromosome 14 open reading frame 21 | Homo sapiens |
| 83999 | kringle containing transmembrane protein 1 | Homo sapiens |
| 6945 | MAX-like protein X | Homo sapiens |
| 119032 | chromosome 10 open reading frame 32 | Homo sapiens |
| 10766 | transducer of ERBB2, 2 | Homo sapiens |
| 11143 | MYST histone acetyltransferase 2; similar to MYST histone acetyltransferase 2 | Homo sapiens |
| 83932 | chromosome 1 open reading frame 124 | Homo sapiens |
| 5899 | v-ral simian leukemia viral oncogene homolog B (ras related; GTP binding protein) | Homo sapiens |

| | | |
|---|---|---|
| 127544 | ring finger protein 19B | Homo sapiens |
| 79876 | ubiquitin-like modifier activating enzyme 5 | Homo sapiens |
| 4306 | nuclear receptor subfamily 3, group C, member 2 | Homo sapiens |
| 80071 | coiled-coil domain containing 15 | Homo sapiens |
| 100134937 | hypothetical LOC100134937 | Homo sapiens |
| 114659 | similar to hCG1991475; leucine rich repeat containing 37B | Homo sapiens |
| 9266 | cytohesin 2 | Homo sapiens |
| 64859 | oligonucleotide/oligosaccharide-binding fold containing 2A | Homo sapiens |
| 4299 | AF4/FMR2 family, member 1 | Homo sapiens |
| 492311 | chromosome 5 open reading frame 53 | Homo sapiens |
| 378708 | cortistatin; apoptosis-inducing, TAF9-like domain 1 | Homo sapiens |
| 79718 | transducin (beta)-like 1 X-linked receptor 1 | Homo sapiens |
| 10450 | peptidylprolyl isomerase E (cyclophilin E) | Homo sapiens |
| 2729 | glutamate-cysteine ligase, catalytic subunit | Homo sapiens |
| 55213 | regulator of chromosome condensation (RCC1) and BTB (POZ) domain containing protein 1 | Homo sapiens |
| 283933 | zinc finger protein 843 | Homo sapiens |
| 6434 | transformer 2 beta homolog (Drosophila) | Homo sapiens |
| 729683 | hypothetical protein LOC729683 | Homo sapiens |
| 400569 | mediator complex subunit 11 | Homo sapiens |
| 1453 | casein kinase 1, delta | Homo sapiens |
| 160760 | PTC7 protein phosphatase homolog (S. cerevisiae) | Homo sapiens |
| 54930 | HAUS augmin-like complex, subunit 4 | Homo sapiens |
| 389206 | BEN domain containing 4 | Homo sapiens |
| 112812 | ferredoxin 1-like | Homo sapiens |
| 23307 | FK506 binding protein 15, 133kDa | Homo sapiens |
| 6515 | solute carrier family 2 (facilitated glucose transporter), member 3 | Homo sapiens |
| 10897 | Yip1 interacting factor homolog A (S. cerevisiae) | Homo sapiens |
| 595 | cyclin D1 | Homo sapiens |
| 140730 | regulating synaptic membrane exocytosis 4 | Homo sapiens |
| 51434 | anaphase promoting complex subunit 7 | Homo sapiens |
| 6576 | solute carrier family 25 (mitochondrial carrier; citrate transporter), member 1 | Homo sapiens |
| 6294 | scaffold attachment factor B | Homo sapiens |
| 27131 | sorting nexin 5 | Homo sapiens |
| 63929 | X-prolyl aminopeptidase (aminopeptidase P) 3, putative | Homo sapiens |
| 22808 | muscle RAS oncogene homolog | Homo sapiens |
| 252969 | nei like 2 (E. coli) | Homo sapiens |
| 79027 | zinc finger protein 655 | Homo sapiens |
| 22985 | apoptotic chromatin condensation inducer 1 | Homo sapiens |
| 49855 | S-phase cyclin A-associated protein in the ER | Homo sapiens |
| 387 | ras homolog gene family, member A | Homo sapiens |
| 26151 | N-acetyltransferase 9 (GCN5-related, putative) | Homo sapiens |
| 4841 | non-POU domain containing, octamer-binding | Homo sapiens |
| 3298 | heat shock transcription factor 2 | Homo sapiens |
| 122416 | ankyrin repeat domain 9 | Homo sapiens |
| 91869 | RFT1 homolog (S. cerevisiae) | Homo sapiens |
| 3460 | interferon gamma receptor 2 (interferon gamma transducer 1) | Homo sapiens |
| 5514 | protein phosphatase 1, regulatory (inhibitor) subunit 10 | Homo sapiens |
| 283352 | hypothetical protein LOC283352 | Homo sapiens |
| 1385 | cAMP responsive element binding protein 1 | Homo sapiens |
| 6168 | ribosomal protein L37a | Homo sapiens |
| 27125 | AF4/FMR2 family, member 4 | Homo sapiens |
| 10725 | nuclear factor of activated T-cells 5, tonicity-responsive | Homo sapiens |
| 5519 | protein phosphatase 2 (formerly 2A), regulatory subunit A, beta isoform | Homo sapiens |
| 348235 | family with sequence similarity 33, member A; similar to Spindle and kinetochore-associated protein 2 | Homo sapiens |
| 60313 | GC-rich promoter binding protein 1-like 1 | Homo sapiens |
| 26086 | G-protein signaling modulator 1 (AGS3-like, C. elegans) | Homo sapiens |
| 26575 | regulator of G-protein signaling 17 | Homo sapiens |
| 90411 | multiple coagulation factor deficiency 2 | Homo sapiens |
| 3746 | potassium voltage-gated channel, Shaw-related subfamily, member 1 | Homo sapiens |
| 91694 | LON peptidase N-terminal domain and ring finger 1 | Homo sapiens |
| 1373 | carbamoyl-phosphate synthetase 1, mitochondrial | Homo sapiens |
| 10542 | hepatitis B virus x interacting protein | Homo sapiens |
| 645644 | hypothetical LOC645644 | Homo sapiens |
| 2746 | glutamate dehydrogenase 1 | Homo sapiens |
| 7431 | vimentin | Homo sapiens |
| 8553 | basic helix-loop-helix family, member e40 | Homo sapiens |
| 8776 | myotubularin related protein 1 | Homo sapiens |
| 8766 | RAB11A, member RAS oncogene family | Homo sapiens |
| 84124 | zinc finger protein 394 | Homo sapiens |
| 7126 | tumor necrosis factor, alpha-induced protein 1 (endothelial) | Homo sapiens |
| 57095 | chromosome 1 open reading frame 128 | Homo sapiens |
| 10801 | septin 9 | Homo sapiens |
| 165215 | family with sequence similarity 171, member B | Homo sapiens |
| 342892 | zinc finger protein 850 pseudogene | Homo sapiens |
| 1347 | cytochrome c oxidase subunit VIIa polypeptide 2 (liver) | Homo sapiens |
| 6723 | spermidine synthase | Homo sapiens |

| | | |
|---|---|---|
| 55288 | ras homolog gene family, member T1 | Homo sapiens |
| 9644 | SH3 and PX domains 2A | Homo sapiens |
| 2889 | Rap guanine nucleotide exchange factor (GEF) 1 | Homo sapiens |
| 54069 | chromosome 21 open reading frame 45 | Homo sapiens |
| 6657 | SRY (sex determining region Y)-box 2 | Homo sapiens |
| 2000 | E74-like factor 4 (ets domain transcription factor) | Homo sapiens |
| 4779 | nuclear factor (erythroid-derived 2)-like 1 | Homo sapiens |
| 10084 | polyglutamine binding protein 1 | Homo sapiens |
| 57409 | MIF4G domain containing | Homo sapiens |
| 4097 | v-maf musculoaponeurotic fibrosarcoma oncogene homolog G (avian) | Homo sapiens |
| 10627 | myosin, light chain 12A, regulatory, non-sarcomeric | Homo sapiens |
| 4848 | CCR4-NOT transcription complex, subunit 2 | Homo sapiens |
| 22803 | 5'-3' exoribonuclease 2 | Homo sapiens |
| 4580 | metaxin 1 | Homo sapiens |
| 55207 | ADP-ribosylation factor-like 8B | Homo sapiens |
| 6165 | ribosomal protein L35a | Homo sapiens |
| 728723 | hypothetical LOC728723 | Homo sapiens |
| 146956 | essential meiotic endonuclease 1 homolog 1 (S. pombe) | Homo sapiens |
| 55275 | vacuolar protein sorting 53 homolog (S. cerevisiae) | Homo sapiens |
| 219402 | mitochondrial translational initiation factor 3 | Homo sapiens |
| 26054 | SUMO1/sentrin specific peptidase 6 | Homo sapiens |
| 51573 | glycerophosphodiester phosphodiesterase 1 | Homo sapiens |
| 57213 | chromosome 13 open reading frame 1 | Homo sapiens |
| 55754 | transmembrane protein 30A | Homo sapiens |
| 11103 | KRR1, small subunit (SSU) processome component, homolog (yeast) | Homo sapiens |
| 60559 | signal peptidase complex subunit 3 homolog (S. cerevisiae) | Homo sapiens |
| 23112 | trinucleotide repeat containing 6B | Homo sapiens |
| 51650 | mitochondrial ribosomal protein S33 | Homo sapiens |
| 5869 | RAB5B, member RAS oncogene family | Homo sapiens |
| 9512 | peptidase (mitochondrial processing) beta | Homo sapiens |
| 143279 | HECT domain containing 2 | Homo sapiens |
| 79568 | chromosome 2 open reading frame 47 | Homo sapiens |
| 3105 | major histocompatibility complex, class I, A | Homo sapiens |
| 375743 | protein prenyltransferase alpha subunit repeat containing 1 | Homo sapiens |
| 5567 | protein kinase, cAMP-dependent, catalytic, beta | Homo sapiens |
| 79971 | G protein-coupled receptor 177 | Homo sapiens |
| 55629 | proline-rich nuclear receptor coactivator 2; similar to hCG1728885 | Homo sapiens |
| 2237 | flap structure-specific endonuclease 1 | Homo sapiens |
| 4677 | asparaginyl-tRNA synthetase | Homo sapiens |
| 6093 | similar to Rho-associated, coiled-coil containing protein kinase 1; Rho-associated, coiled-coil containing protein kinase 1 | Homo sapiens |
| 10950 | BTG family, member 3 | Homo sapiens |
| 80213 | TM2 domain containing 3 | Homo sapiens |
| 5494 | protein phosphatase 1A (formerly 2C), magnesium-dependent, alpha isoform | Homo sapiens |
| 1789 | DNA (cytosine-5-)-methyltransferase 3 beta | Homo sapiens |
| 6203 | ribosomal protein S9; ribosomal protein S9 pseudogene 4 | Homo sapiens |
| 54439 | RNA binding motif protein 27 | Homo sapiens |
| 10445 | microspherule protein 1 | Homo sapiens |
| 5813 | purine-rich element binding protein A | Homo sapiens |
| 84717 | hepatoma-derived growth factor-related protein 2 | Homo sapiens |
| 3845 | v-Ki-ras2 Kirsten rat sarcoma viral oncogene homolog | Homo sapiens |
| 26608 | transducin (beta)-like 2 | Homo sapiens |
| 100131827 | zinc finger protein 717 | Homo sapiens |
| 10430 | transmembrane protein 147 | Homo sapiens |
| 127687 | chromosome 1 open reading frame 122 | Homo sapiens |
| 51075 | thioredoxin-related transmembrane protein 2 | Homo sapiens |
| 84108 | polycomb group ring finger 6 | Homo sapiens |
| 4763 | neurofibromin 1 | Homo sapiens |
| 7341 | SMT3 suppressor of mif two 3 homolog 1 (S. cerevisiae); SUMO1 pseudogene 3 | Homo sapiens |
| 5608 | mitogen-activated protein kinase kinase 6 | Homo sapiens |
| 10953 | translocase of outer mitochondrial membrane 34 | Homo sapiens |
| 27166 | PRELI domain containing 1; similar to Px19-like protein (25 kDa protein of relevant evolutionary and lymphoid interest) (PRELI) | Homo sapiens |
| 81875 | interferon stimulated exonuclease gene 20kDa-like 2 | Homo sapiens |
| 56941 | chromosome 3 open reading frame 37 | Homo sapiens |
| 9689 | basic leucine zipper and W2 domains 1 pseudogene 1; basic leucine zipper and W2 domains 1 like 1; basic leucine zipper and W2 domains 1 | Homo sapiens |
| 3775 | potassium channel, subfamily K, member 1 | Homo sapiens |
| 26017 | family with sequence similarity 32, member A | Homo sapiens |
| 3227 | homeobox C11 | Homo sapiens |
| 55084 | sine oculis binding protein homolog (Drosophila) | Homo sapiens |
| 54480 | chondroitin sulfate glucuronyltransferase | Homo sapiens |
| 401068 | hypothetical LOC401068 | Homo sapiens |
| 2202 | EGF-containing fibulin-like extracellular matrix protein 1 | Homo sapiens |
| 8812 | cyclin K | Homo sapiens |
| 8615 | USO1 homolog, vesicle docking protein (yeast) | Homo sapiens |
| 93643 | tight junction associated protein 1 (peripheral) | Homo sapiens |

| | | |
|---|---|---|
| 728833 | family with sequence similarity 72, member D; family with sequence similarity 72, member A | Homo sapiens |
| 729533 | family with sequence similarity 72, member D; family with sequence similarity 72, member A | Homo sapiens |
| 81577 | glucose-fructose oxidoreductase domain containing 2 | Homo sapiens |
| 10856 | RuvB-like 2 (E. coli) | Homo sapiens |
| 8509 | N-deacetylase/N-sulfotransferase (heparan glucosaminyl) 2 | Homo sapiens |
| 115560 | zinc finger protein 501 | Homo sapiens |
| 50813 | COP9 constitutive photomorphogenic homolog subunit 7A (Arabidopsis) | Homo sapiens |
| 65109 | UPF3 regulator of nonsense transcripts homolog B (yeast) | Homo sapiens |
| 6625 | small nuclear ribonucleoprotein 70kDa (U1) | Homo sapiens |
| 9338 | transcription elongation factor A (SII)-like 1 | Homo sapiens |
| 90957 | DEAH (Asp-Glu-Ala-Asp/His) box polypeptide 57 | Homo sapiens |
| 57666 | fibrosin-like 1 | Homo sapiens |
| 310 | annexin A7 | Homo sapiens |
| 221908 | chromosome 7 open reading frame 47 | Homo sapiens |
| 80331 | DnaJ (Hsp40) homolog, subfamily C, member 5 | Homo sapiens |
| 11231 | SEC63 homolog (S. cerevisiae) | Homo sapiens |
| 28969 | basic leucine zipper and W2 domains 2 | Homo sapiens |
| 51538 | zinc finger, CCHC domain containing 17 | Homo sapiens |
| 5573 | protein kinase, cAMP-dependent, regulatory, type I, alpha (tissue specific extinguisher 1) | Homo sapiens |
| 6888 | transaldolase 1 | Homo sapiens |
| 9342 | synaptosomal-associated protein, 29kDa | Homo sapiens |
| 389203 | hypothetical protein LOC389203 | Homo sapiens |
| 192669 | eukaryotic translation initiation factor 2C, 3 | Homo sapiens |
| 729970 | similar to hCG2028352 | Homo sapiens |
| 134359 | chromosome 5 open reading frame 37 | Homo sapiens |
| 1832 | desmoplakin | Homo sapiens |
| 219699 | unc-5 homolog B (C. elegans) | Homo sapiens |
| 8570 | KH-type splicing regulatory protein | Homo sapiens |
| 84918 | low density lipoprotein receptor-related protein 11 | Homo sapiens |
| 6774 | signal transducer and activator of transcription 3 (acute-phase response factor) | Homo sapiens |
| 6626 | small nuclear ribonucleoprotein polypeptide A | Homo sapiens |
| 4043 | low density lipoprotein receptor-related protein associated protein 1 | Homo sapiens |
| 5286 | phosphoinositide-3-kinase, class 2, alpha polypeptide | Homo sapiens |
| 81614 | non imprinted in Prader-Willi/Angelman syndrome 2 | Homo sapiens |
| 1466 | cysteine and glycine-rich protein 2 | Homo sapiens |
| 55052 | similar to mitochondrial ribosomal protein L20; mitochondrial ribosomal protein L20 | Homo sapiens |
| 10402 | ST3 beta-galactoside alpha-2,3-sialyltransferase 6 | Homo sapiens |
| 9662 | centrosomal protein 135kDa | Homo sapiens |
| 84946 | similar to putative protein STRF7; LTV1 homolog (S. cerevisiae) | Homo sapiens |
| 9837 | GINS complex subunit 1 (Psf1 homolog) | Homo sapiens |
| 23107 | mitochondrial ribosomal protein S27 | Homo sapiens |
| 3983 | actin binding LIM protein 1 | Homo sapiens |
| 9588 | peroxiredoxin 6 | Homo sapiens |
| 8971 | H1 histone family, member X | Homo sapiens |
| 280636 | chromosome 11 open reading frame 31 | Homo sapiens |
| 332 | baculoviral IAP repeat-containing 5 | Homo sapiens |
| 10628 | thioredoxin interacting protein | Homo sapiens |
| 64960 | mitochondrial ribosomal protein S15 | Homo sapiens |
| 84532 | acyl-CoA synthetase short-chain family member 1 | Homo sapiens |
| 84233 | transmembrane protein 126A | Homo sapiens |
| 5934 | retinoblastoma-like 2 (p130) | Homo sapiens |
| 80279 | CDK5 regulatory subunit associated protein 3 | Homo sapiens |
| 80262 | chromosome 16 open reading frame 70 | Homo sapiens |
| 64708 | COP9 constitutive photomorphogenic homolog subunit 7B (Arabidopsis) | Homo sapiens |
| 150381 | hypothetical LOC150381 | Homo sapiens |
| 201931 | transmembrane protein 192 | Homo sapiens |
| 23158 | TBC1 domain family, member 9 (with GRAM domain) | Homo sapiens |
| 23367 | La ribonucleoprotein domain family, member 1 | Homo sapiens |
| 23258 | DENN/MADD domain containing 5A | Homo sapiens |
| 10240 | mitochondrial ribosomal protein S31 | Homo sapiens |
| 84811 | BUD13 homolog (S. cerevisiae) | Homo sapiens |
| 55254 | transmembrane protein 39A | Homo sapiens |
| 11198 | suppressor of Ty 16 homolog (S. cerevisiae); suppressor of Ty 16 homolog (S. cerevisiae) pseudogene | Homo sapiens |
| 56902 | partner of NOB1 homolog (S. cerevisiae) | Homo sapiens |
| 10588 | 5,10-methenyltetrahydrofolate synthetase (5-formyltetrahydrofolate cyclo-ligase) | Homo sapiens |
| 54915 | YTH domain family, member 1 | Homo sapiens |
| 7204 | triple functional domain (PTPRF interacting) | Homo sapiens |
| 2317 | filamin B, beta (actin binding protein 278) | Homo sapiens |
| 79960 | PHD finger protein 17 | Homo sapiens |
| 148413 | hypothetical LOC148413 | Homo sapiens |
| 10802 | SEC24 family, member A (S. cerevisiae) | Homo sapiens |
| 100133121 | family with sequence similarity 27, member C; family with sequence similarity 27, member A; family with sequence similarity 27, member B | Homo sapiens |

| | | |
|---|---|---|
| 548321 | family with sequence similarity 27, member C; family with sequence similarity 27, member A; family with sequence similarity 27, member B | Homo sapiens |
| 100132948 | family with sequence similarity 27, member C; family with sequence similarity 27, member A; family with sequence similarity 27, member B | Homo sapiens |
| 493856 | CDGSH iron sulfur domain 2 | Homo sapiens |
| 26523 | eukaryotic translation initiation factor 2C, 1 | Homo sapiens |
| 23164 | myosin phosphatase Rho interacting protein; similar to Myosin phosphatase Rho-interacting protein (Rho-interacting protein 3) (M-RIP) (RIP3) (p116Rip) | Homo sapiens |
| 7357 | UDP-glucose ceramide glucosyltransferase | Homo sapiens |
| 5093 | poly(rC) binding protein 1 | Homo sapiens |
| 113457 | tubulin, alpha 3d; tubulin, alpha 3c | Homo sapiens |
| 10220 | growth differentiation factor 11 | Homo sapiens |
| 51537 | mitochondrial protein 18 kDa | Homo sapiens |
| 92255 | LMBR1 domain containing 2 | Homo sapiens |
| 7323 | ubiquitin-conjugating enzyme E2D 3 (UBC4/5 homolog, yeast); ubiquitin-conjugating enzyme E2D 3 pseudogene | Homo sapiens |
| 10124 | ADP-ribosylation factor-like 4A | Homo sapiens |
| 3308 | heat shock 70kDa protein 4 | Homo sapiens |
| 4538 | NADH-ubiquinone oxidoreductase chain 4L; NADH-ubiquinone oxidoreductase chain 4 | Homo sapiens |
| 23060 | zinc finger protein 609 | Homo sapiens |
| 84886 | chromosome 1 open reading frame 198 | Homo sapiens |
| 10289 | eukaryotic translation initiation factor 1B | Homo sapiens |
| 7975 | v-maf musculoaponeurotic fibrosarcoma oncogene homolog K (avian) | Homo sapiens |
| 388610 | TMF1-regulated nuclear protein 1 | Homo sapiens |
| 63979 | fidgetin-like 1 | Homo sapiens |
| 655 | bone morphogenetic protein 7 | Homo sapiens |
| 55788 | LMBR1 domain containing 1 | Homo sapiens |
| 197370 | non-SMC element 1 homolog (S. cerevisiae) | Homo sapiens |
| 79624 | chromosome 6 open reading frame 211 | Homo sapiens |
| 84988 | protein phosphatase 1, regulatory (inhibitor) subunit 16A | Homo sapiens |
| 857 | caveolin 1, caveolae protein, 22kDa | Homo sapiens |
| 25953 | paroxysmal nonkinesigenic dyskinesia | Homo sapiens |
| 64116 | solute carrier family 39 (zinc transporter), member 8 | Homo sapiens |
| 84231 | TNF receptor-associated factor 7 | Homo sapiens |
| 145173 | beta 1,3-galactosyltransferase-like | Homo sapiens |
| 573 | BCL2-associated athanogene | Homo sapiens |
| 4123 | mannosidase, alpha, class 2C, member 1 | Homo sapiens |
| 9559 | vacuolar protein sorting 26 homolog A (S. pombe) | Homo sapiens |
| 27044 | staphylococcal nuclease and tudor domain containing 1 | Homo sapiens |
| 79607 | family with sequence similarity 118, member B | Homo sapiens |
| 1615 | aspartyl-tRNA synthetase | Homo sapiens |
| 219854 | transmembrane protein 218 | Homo sapiens |
| 5713 | proteasome (prosome, macropain) 26S subunit, non-ATPase, 7 | Homo sapiens |
| 51379 | cytokine receptor-like factor 3 | Homo sapiens |
| 440145 | chromosome 13 open reading frame 37 | Homo sapiens |
| 80131 | leucine rich repeat containing 8 family, member E | Homo sapiens |
| 5634 | phosphoribosyl pyrophosphate synthetase 2 | Homo sapiens |
| 55312 | riboflavin kinase | Homo sapiens |
| 9201 | doublecortin-like kinase 1 | Homo sapiens |
| 3837 | karyopherin (importin) beta 1 | Homo sapiens |
| 84334 | chromosome 14 open reading frame 153 | Homo sapiens |
| 11142 | protein kinase (cAMP-dependent, catalytic) inhibitor gamma | Homo sapiens |
| 130074 | family with sequence similarity 168, member B | Homo sapiens |
| 23536 | adenosine deaminase, tRNA-specific 1 | Homo sapiens |
| 57630 | SH3 domain containing ring finger 1 | Homo sapiens |
| 8721 | endothelial differentiation-related factor 1 | Homo sapiens |
| 730092 | RRN3 RNA polymerase I transcription factor homolog (S. cerevisiae) pseudogene | Homo sapiens |
| 2295 | forkhead box F2 | Homo sapiens |
| 6535 | solute carrier family 6 (neurotransmitter transporter, creatine), member 8 | Homo sapiens |
| 7818 | death associated protein 3 | Homo sapiens |
| 7298 | thymidylate synthetase | Homo sapiens |
| 51142 | coiled-coil-helix-coiled-coil-helix domain containing 2; similar to coiled-coil-helix-coiled-coil-helix domain containing 2 | Homo sapiens |
| 10979 | fermitin family homolog 2 (Drosophila) | Homo sapiens |
| 2872 | MAP kinase interacting serine/threonine kinase 2 | Homo sapiens |
| 9688 | nucleoporin 93kDa | Homo sapiens |
| 282996 | RNA binding motif protein 20 | Homo sapiens |
| 165918 | ring finger protein 168 | Homo sapiens |
| 80777 | cytochrome b5 type B (outer mitochondrial membrane) | Homo sapiens |
| 116225 | zinc finger, MYND-type containing 19 | Homo sapiens |
| 2395 | frataxin | Homo sapiens |
| 28956 | roadblock domain containing 3 | Homo sapiens |
| 114789 | solute carrier family 25 (mitochondrial carrier; phosphate carrier), member 25 | Homo sapiens |
| 79666 | pleckstrin homology domain containing, family F (with FYVE domain) member 2 | Homo sapiens |
| 51002 | TP53RK binding protein | Homo sapiens |
| 29920 | pyrroline-5-carboxylate reductase family, member 2 | Homo sapiens |
| 9045 | ribosomal protein L14 | Homo sapiens |

| | | |
|---|---|---|
| 9563 | hexose-6-phosphate dehydrogenase (glucose 1-dehydrogenase) | Homo sapiens |
| 23517 | superkiller viralicidic activity 2-like 2 (S. cerevisiae) | Homo sapiens |
| 4212 | Meis homeobox 2 | Homo sapiens |
| 51302 | cytochrome P450, family 39, subfamily A, polypeptide 1 | Homo sapiens |
| 55802 | DCP1 decapping enzyme homolog A (S. cerevisiae) | Homo sapiens |
| 5378 | PMS1 postmeiotic segregation increased 1 (S. cerevisiae) | Homo sapiens |
| 54539 | NADH dehydrogenase (ubiquinone) 1 beta subcomplex, 11, 17.3kDa | Homo sapiens |
| 84869 | carbonyl reductase 4 | Homo sapiens |
| 92002 | family with sequence similarity 58, member A; hypothetical gene supported by NM_152274 | Homo sapiens |
| 4780 | nuclear factor (erythroid-derived 2)-like 2 | Homo sapiens |
| 10519 | calcium and integrin binding 1 (calmyrin) | Homo sapiens |
| 93986 | forkhead box P2 | Homo sapiens |
| 51534 | Vps20-associated 1 homolog (S. cerevisiae) | Homo sapiens |
| 51020 | HD domain containing 2 | Homo sapiens |
| 79677 | structural maintenance of chromosomes 6 | Homo sapiens |
| 5663 | presenilin 1 | Homo sapiens |
| 3098 | hexokinase 1 | Homo sapiens |
| 55033 | FK506 binding protein 14, 22 kDa | Homo sapiens |
| 203069 | R3H domain and coiled-coil containing 1 | Homo sapiens |
| 57545 | coiled-coil and C2 domain containing 2A | Homo sapiens |
| 57645 | pogo transposable element with KRAB domain | Homo sapiens |
| 2281 | FK506 binding protein 1B, 12.6 kDa | Homo sapiens |
| 5826 | ATP-binding cassette, sub-family D (ALD), member 4 | Homo sapiens |
| 2288 | FK506 binding protein 4, 59kDa | Homo sapiens |
| 10241 | calcium binding and coiled-coil domain 2 | Homo sapiens |
| 5586 | protein kinase N2 | Homo sapiens |
| 4194 | Mdm4 p53 binding protein homolog (mouse) | Homo sapiens |
| 56852 | RAD18 homolog (S. cerevisiae) | Homo sapiens |
| 8939 | far upstream element (FUSE) binding protein 3 | Homo sapiens |
| 23275 | protein O-fucosyltransferase 2 | Homo sapiens |
| 24140 | FtsJ homolog 1 (E. coli) | Homo sapiens |
| 56252 | YLP motif containing 1 | Homo sapiens |
| 253264 | hypothetical protein LOC253264 | Homo sapiens |
| 8673 | vesicle-associated membrane protein 8 (endobrevin) | Homo sapiens |
| 4802 | nuclear transcription factor Y, gamma | Homo sapiens |
| 6899 | T-box 1 | Homo sapiens |
| 9383 | XIST antisense RNA (non-protein coding) | Homo sapiens |
| 55023 | pleckstrin homology domain interacting protein | Homo sapiens |
| 65260 | chromosome 1 open reading frame 163 | Homo sapiens |
| 8450 | cullin 4B | Homo sapiens |
| 404636 | family with sequence similarity 45, member A | Homo sapiens |
| 23063 | wings apart-like homolog (Drosophila) | Homo sapiens |
| 339287 | male-specific lethal 1 homolog (Drosophila) | Homo sapiens |
| 84874 | zinc finger protein 514 | Homo sapiens |
| 51478 | hydroxysteroid (17-beta) dehydrogenase 7 | Homo sapiens |
| 1340 | cytochrome c oxidase subunit Vib polypeptide 1 (ubiquitous) | Homo sapiens |
| 441263 | postmeiotic segregation increased 2-like 11 pseudogene | Homo sapiens |
| 9889 | zinc finger, BED-type containing 4 | Homo sapiens |
| 91300 | chromosome 19 open reading frame 22 | Homo sapiens |
| 23163 | golgi associated, gamma adaptin ear containing, ARF binding protein 3 | Homo sapiens |
| 1075 | cathepsin C | Homo sapiens |
| 619208 | chromosome 6 open reading frame 225 | Homo sapiens |
| 10363 | high-mobility group 20A | Homo sapiens |
| 143686 | sestrin 3 | Homo sapiens |
| 642533 | hypothetical LOC642533 | Homo sapiens |
| 9406 | zinc finger, RAN-binding domain containing 2 | Homo sapiens |
| 128077 | Lix1 homolog (mouse)-like | Homo sapiens |
| 23350 | U2-associated SR140 protein | Homo sapiens |
| 254065 | bromodomain and WD repeat domain containing 3 | Homo sapiens |
| 10198 | M-phase phosphoprotein 9 | Homo sapiens |
| 84277 | DnaJ (Hsp40) homolog, subfamily C, member 30 | Homo sapiens |
| 11021 | similar to hCG1778032; RAB35, member RAS oncogene family | Homo sapiens |
| 163 | adaptor-related protein complex 2, beta 1 subunit | Homo sapiens |
| 84062 | dystrobrevin binding protein 1 | Homo sapiens |
| 399818 | methyltransferase like 10 | Homo sapiens |
| 144438 | hypothetical LOC144438 | Homo sapiens |
| 8800 | peroxisomal biogenesis factor 11 alpha | Homo sapiens |
| 6640 | syntrophin, alpha 1 (dystrophin-associated protein A1, 59kDa, acidic component) | Homo sapiens |
| 958 | CD40 molecule, TNF receptor superfamily member 5 | Homo sapiens |
| 53343 | nudix (nucleoside diphosphate linked moiety X)-type motif 9 | Homo sapiens |
| 5134 | programmed cell death 2 | Homo sapiens |
| 25945 | poliovirus receptor-related 3 | Homo sapiens |
| 11188 | nischarin | Homo sapiens |
| 25962 | KIAA1429 | Homo sapiens |
| 51444 | ring finger protein 138 | Homo sapiens |
| 64921 | CAS1 domain containing 1 | Homo sapiens |
| 25942 | SIN3 homolog A, transcription regulator (yeast) | Homo sapiens |

| | | |
|---|---|---|
| 3725 | jun oncogene | Homo sapiens |
| 6645 | syntrophin, beta 2 (dystrophin-associated protein A1, 59kDa, basic component 2) | Homo sapiens |
| 51635 | dehydrogenase/reductase (SDR family) member 7 | Homo sapiens |
| 1514 | cathepsin L1 | Homo sapiens |
| 5706 | proteasome (prosome, macropain) 26S subunit, ATPase, 6 | Homo sapiens |
| 391 | ras homolog gene family, member G (rho G) | Homo sapiens |
| 3209 | homeobox A13 | Homo sapiens |
| 144983 | heterogeneous nuclear ribonucleoprotein A1-like 2 | Homo sapiens |
| 10920 | COP9 constitutive photomorphogenic homolog subunit 8 (Arabidopsis) | Homo sapiens |
| 90843 | transcription elongation factor A (SII)-like 8 | Homo sapiens |
| 64645 | hippocampus abundant transcript 1 | Homo sapiens |
| 359948 | interferon regulatory factor 2 binding protein 2 | Homo sapiens |
| 51619 | ubiquitin-conjugating enzyme E2D 4 (putative) | Homo sapiens |
| 5189 | peroxisomal biogenesis factor 1 | Homo sapiens |
| 84265 | polymerase (RNA) III (DNA directed) polypeptide G (32kD)-like | Homo sapiens |
| 5007 | oxysterol binding protein | Homo sapiens |
| 4356 | membrane protein, palmitoylated 3 (MAGUK p55 subfamily member 3) | Homo sapiens |
| 54534 | mitochondrial ribosomal protein L50 | Homo sapiens |
| 2035 | erythrocyte membrane protein band 4.1 (elliptocytosis 1, RH-linked) | Homo sapiens |
| 6193 | ribosomal protein S5 | Homo sapiens |
| 9146 | hepatocyte growth factor-regulated tyrosine kinase substrate | Homo sapiens |
| 6827 | suppressor of Ty 4 homolog 1 (S. cerevisiae) | Homo sapiens |
| 23047 | PDS5, regulator of cohesion maintenance, homolog B (S. cerevisiae) | Homo sapiens |
| 8091 | high mobility group AT-hook 2 | Homo sapiens |
| 5089 | pre-B-cell leukemia homeobox 2 | Homo sapiens |
| 10970 | cytoskeleton-associated protein 4 | Homo sapiens |
| 59307 | single immunoglobulin and toll-interleukin 1 receptor (TIR) domain | Homo sapiens |
| 401548 | sorting nexin family member 30 | Homo sapiens |
| 3838 | karyopherin alpha 2 (RAG cohort 1, importin alpha 1); karyopherin alpha-2 subunit like | Homo sapiens |
| 29956 | LAG1 homolog, ceramide synthase 2 | Homo sapiens |
| 27067 | staufen, RNA binding protein, homolog 2 (Drosophila) | Homo sapiens |
| 79723 | suppressor of variegation 3-9 homolog 2 (Drosophila) | Homo sapiens |
| 137886 | UBX domain protein 2B | Homo sapiens |
| 9529 | BCL2-associated athanogene 5 | Homo sapiens |
| 55449 | chromosome 14 open reading frame 167 | Homo sapiens |
| 23413 | frequenin homolog (Drosophila) | Homo sapiens |
| 28955 | dexamethasone-induced transcript | Homo sapiens |
| 54407 | solute carrier family 38, member 2 | Homo sapiens |
| 3275 | protein arginine methyltransferase 2 | Homo sapiens |
| 55677 | IWS1 homolog (S. cerevisiae) | Homo sapiens |
| 79659 | dynein, cytoplasmic 2, heavy chain 1 | Homo sapiens |
| 928 | CD9 molecule | Homo sapiens |
| 51729 | WW domain binding protein 11 | Homo sapiens |
| 5529 | protein phosphatase 2, regulatory subunit B', epsilon isoform | Homo sapiens |
| 54841 | basic, immunoglobulin-like variable motif containing | Homo sapiens |
| 26012 | nasal embryonic LHRH factor | Homo sapiens |
| 7416 | voltage-dependent anion channel 1; similar to voltage-dependent anion channel 1 | Homo sapiens |
| 4137 | microtubule-associated protein tau | Homo sapiens |
| 22818 | coatomer protein complex, subunit zeta 1 | Homo sapiens |
| 8878 | sequestosome 1 | Homo sapiens |
| 85365 | asparagine-linked glycosylation 2, alpha-1,3-mannosyltransferase homolog (S. cerevisiae) | Homo sapiens |
| 466 | activating transcription factor 1 | Homo sapiens |
| 23731 | chromosome 9 open reading frame 5 | Homo sapiens |
| 81565 | nudE nuclear distribution gene E homolog (A. nidulans)-like 1 | Homo sapiens |
| 10480 | eukaryotic translation initiation factor 3, subunit M | Homo sapiens |
| 54908 | coiled-coil domain containing 99 | Homo sapiens |
| 1021 | cyclin-dependent kinase 6 | Homo sapiens |
| 84224 | neuroblastoma breakpoint family, member 3 | Homo sapiens |
| 93380 | membrane magnesium transporter 1 | Homo sapiens |
| 4602 | v-myb myeloblastosis viral oncogene homolog (avian) | Homo sapiens |
| 7324 | ubiquitin-conjugating enzyme E2E 1 (UBC4/5 homolog, yeast) | Homo sapiens |
| 203259 | chromosome 9 open reading frame 25 | Homo sapiens |
| 79882 | zinc finger CCCH-type containing 14 | Homo sapiens |
| 23116 | family with sequence similarity 179, member B | Homo sapiens |
| 400793 | chromosome 1 open reading frame 226 | Homo sapiens |
| 64788 | lipase maturation factor 1 | Homo sapiens |
| 222553 | solute carrier family 35, member F1 | Homo sapiens |
| 7077 | TIMP metallopeptidase inhibitor 2 | Homo sapiens |
| 84733 | chromobox homolog 2 (Pc class homolog, Drosophila) | Homo sapiens |
| 9061 | 3'-phosphoadenosine 5'-phosphosulfate synthase 1 | Homo sapiens |
| 23023 | transmembrane and coiled-coil domain family 1 | Homo sapiens |
| 27032 | ATPase, Ca++ transporting, type 2C, member 1 | Homo sapiens |
| 56984 | proteasome (prosome, macropain) assembly chaperone 2 | Homo sapiens |
| 64083 | golgi phosphoprotein 3 (coat-protein) | Homo sapiens |
| 9133 | cyclin B2 | Homo sapiens |
| 23269 | MAX gene associated | Homo sapiens |
| 388272 | chromosome 16 open reading frame 87 | Homo sapiens |

| | | |
|---|---|---|
| 5860 | quinoid dihydropteridine reductase | Homo sapiens |
| 143458 | low density lipoprotein receptor class A domain containing 3 | Homo sapiens |
| 1729 | diaphanous homolog 1 (Drosophila) | Homo sapiens |
| 5538 | palmitoyl-protein thioesterase 1 | Homo sapiens |
| 4646 | myosin VI | Homo sapiens |
| 5000 | origin recognition complex, subunit 4-like (yeast) | Homo sapiens |
| 22853 | lemur tyrosine kinase 2 | Homo sapiens |
| 7703 | polycomb group ring finger 2 | Homo sapiens |
| 51 | acyl-Coenzyme A oxidase 1, palmitoyl | Homo sapiens |
| 580 | BRCA1 associated RING domain 1 | Homo sapiens |
| 400684 | hypothetical gene supported by BC000922 | Homo sapiens |
| 84163 | GTF2I repeat domain containing 2 | Homo sapiens |
| 11091 | WD repeat domain 5 | Homo sapiens |
| 11083 | death inducer-obliterator 1 | Homo sapiens |
| 3703 | STT3, subunit of the oligosaccharyltransferase complex, homolog A (S. cerevisiae) | Homo sapiens |
| 202018 | transmembrane anterior posterior transformation 1 | Homo sapiens |
| 64682 | anaphase promoting complex subunit 1; similar to anaphase promoting complex subunit 1 | Homo sapiens |
| 201725 | hypothetical LOC100129478; chromosome 4 open reading frame 46 | Homo sapiens |
| 26249 | kelch-like 3 (Drosophila) | Homo sapiens |
| 54949 | chromosome 11 open reading frame 79 | Homo sapiens |
| 8935 | src kinase associated phosphoprotein 2 | Homo sapiens |
| 4638 | myosin light chain kinase | Homo sapiens |
| 146198 | zinc finger protein 90 homolog (mouse) | Homo sapiens |
| 1756 | dystrophin | Homo sapiens |
| 55167 | male-specific lethal 2 homolog (Drosophila) | Homo sapiens |
| 5445 | paraoxonase 2 | Homo sapiens |
| 81847 | ring finger protein 146 | Homo sapiens |
| 8841 | histone deacetylase 3 | Homo sapiens |
| 7167 | TPI1 pseudogene; triosephosphate isomerase 1 | Homo sapiens |
| 23016 | exosome component 7 | Homo sapiens |
| 51629 | solute carrier family 25, member 39 | Homo sapiens |
| 7286 | tuftelin 1 | Homo sapiens |
| 58986 | transmembrane protein 8A | Homo sapiens |
| 2768 | guanine nucleotide binding protein (G protein) alpha 12 | Homo sapiens |
| 22978 | 5'-nucleotidase, cytosolic II | Homo sapiens |
| 523 | ATPase, H+ transporting, lysosomal 70kDa, V1 subunit A | Homo sapiens |
| 1936 | eukaryotic translation elongation factor 1 delta (guanine nucleotide exchange protein) | Homo sapiens |
| 63924 | cell death-inducing DFFA-like effector c | Homo sapiens |
| 3714 | jagged 2 | Homo sapiens |
| 84083 | zinc finger, RAN-binding domain containing 3 | Homo sapiens |
| 51116 | mitochondrial ribosomal protein S2 | Homo sapiens |
| 85464 | slingshot homolog 2 (Drosophila) | Homo sapiens |
| 112483 | spermidine/spermine N1-acetyltransferase family member 2 | Homo sapiens |
| 9500 | melanoma antigen family D, 1 | Homo sapiens |
| 84250 | ankyrin repeat domain 32 | Homo sapiens |
| 5037 | phosphatidylethanolamine binding protein 1 | Homo sapiens |
| 153830 | ring finger protein 145 | Homo sapiens |
| 55827 | IQ motif and WD repeats 1 | Homo sapiens |
| 23228 | phospholipase C-like 2 | Homo sapiens |
| 790955 | chromosome 11 open reading frame 83 | Homo sapiens |
| 645722 | hypothetical LOC645722 | Homo sapiens |
| 51241 | COX16 cytochrome c oxidase assembly homolog (S. cerevisiae) | Homo sapiens |
| 23294 | ankyrin repeat and sterile alpha motif domain containing 1A | Homo sapiens |
| 10491 | cartilage associated protein | Homo sapiens |
| 285704 | RGM domain family, member B | Homo sapiens |
| 55728 | NEDD4 binding protein 2 | Homo sapiens |
| 5511 | protein phosphatase 1, regulatory (inhibitor) subunit 8 | Homo sapiens |
| 2647 | biogenesis of lysosomal organelles complex-1, subunit 1 | Homo sapiens |
| 4170 | myeloid cell leukemia sequence 1 (BCL2-related) | Homo sapiens |
| 25911 | deleted in a mouse model of primary ciliary dyskinesia | Homo sapiens |
| 57136 | chromosome 20 open reading frame 3 | Homo sapiens |
| 1639 | dynactin 1 (p150, glued homolog, Drosophila) | Homo sapiens |
| 90637 | zinc finger, AN1-type domain 2A | Homo sapiens |
| 139221 | melanoma associated antigen (mutated) 1-like 1 | Homo sapiens |
| 10795 | zinc finger protein 268 | Homo sapiens |
| 9531 | BCL2-associated athanogene 3 | Homo sapiens |
| 10946 | splicing factor 3a, subunit 3, 60kDa | Homo sapiens |
| 80139 | zinc finger protein 703 | Homo sapiens |
| 25805 | hypothetical LOC729590; BMP and activin membrane-bound inhibitor homolog (Xenopus laevis) | Homo sapiens |
| 92935 | methionyl-tRNA synthetase 2, mitochondrial | Homo sapiens |
| 79646 | pantothenate kinase 3 | Homo sapiens |
| 2130 | similar to Ewing sarcoma breakpoint region 1; Ewing sarcoma breakpoint region 1 | Homo sapiens |
| 167227 | DCP2 decapping enzyme homolog (S. cerevisiae) | Homo sapiens |
| 29842 | transcription factor CP2-like 1 | Homo sapiens |
| 135228 | CD109 molecule | Homo sapiens |
| 3163 | heme oxygenase (decycling) 2 | Homo sapiens |

| | | |
|---|---|---|
| 1069 | centrin, EF-hand protein, 2 | Homo sapiens |
| 84186 | zinc finger, CCHC domain containing 7 | Homo sapiens |
| 345778 | metaxin 3 | Homo sapiens |
| 94032 | calcium/calmodulin-dependent protein kinase II inhibitor 2 | Homo sapiens |
| 8661 | eukaryotic translation initiation factor 3, subunit A | Homo sapiens |
| 54838 | chromosome 10 open reading frame 26 | Homo sapiens |
| 643180 | chaperonin containing TCP1, subunit 6A pseudogene | Homo sapiens |
| 201254 | stimulated by retinoic acid 13 homolog (mouse) | Homo sapiens |
| 84892 | chromosome 3 open reading frame 39 | Homo sapiens |
| 23200 | ATPase, class VI, type 11B | Homo sapiens |
| 4664 | NGFI-A binding protein 1 (EGR1 binding protein 1) | Homo sapiens |
| 84301 | DDI1, DNA-damage inducible 1, homolog 2 (S. cerevisiae) | Homo sapiens |
| 26227 | phosphoglycerate dehydrogenase | Homo sapiens |
| 5036 | proliferation-associated 2G4, 38kDa; proliferation-associated 2G4 pseudogene 4 | Homo sapiens |
| 5379 | postmeiotic segregation increased 2-like 1 pseudogene | Homo sapiens |
| 8621 | cell division cycle 2-like 5 (cholinesterase-related cell division controller) | Homo sapiens |
| 7259 | TSPY-like 1 | Homo sapiens |
| 55228 | PNMA-like 1 | Homo sapiens |
| 84451 | mixed lineage kinase 4 | Homo sapiens |
| 23352 | ubiquitin protein ligase E3 component n-recognin 4 | Homo sapiens |
| 7979 | split hand/foot malformation (ectrodactyly) type 1 | Homo sapiens |
| 653390 | RRN3 RNA polymerase I transcription factor homolog (S. cerevisiae) pseudogene | Homo sapiens |
| 9894 | TEL2, telomere maintenance 2, homolog (S. cerevisiae) | Homo sapiens |
| 121053 | chromosome 12 open reading frame 45 | Homo sapiens |
| 6427 | splicing factor, arginine/serine-rich 2 | Homo sapiens |
| 23118 | mitogen-activated protein kinase kinase kinase 7 interacting protein 2 | Homo sapiens |
| 388789 | hypothetical LOC388789 | Homo sapiens |
| 27351 | PPPDE peptidase domain containing 2 | Homo sapiens |
| 6301 | seryl-tRNA synthetase | Homo sapiens |
| 124491 | transmembrane protein 170A | Homo sapiens |
| 54984 | PIN2-interacting protein 1 | Homo sapiens |
| 3400 | inhibitor of DNA binding 4, dominant negative helix-loop-helix protein | Homo sapiens |
| 2617 | glycyl-tRNA synthetase | Homo sapiens |
| 100129482 | zinc finger protein 37B (pseudogene) | Homo sapiens |
| 255919 | transmembrane protein 188; similar to TMEM188 protein | Homo sapiens |
| 2287 | FK506 binding protein 3, 25kDa | Homo sapiens |
| 151473 | solute carrier family 16, member 14 (monocarboxylic acid transporter 14) | Homo sapiens |
| 440465 | hypothetical LOC440465 | Homo sapiens |
| 4436 | mutS homolog 2, colon cancer, nonpolyposis type 1 (E. coli) | Homo sapiens |
| 23155 | chloride channel CLIC-like 1 | Homo sapiens |
| 3796 | kinesin heavy chain member 2A | Homo sapiens |
| 10434 | lysophospholipase I | Homo sapiens |
| 54940 | OCIA domain containing 1 | Homo sapiens |
| 7419 | voltage-dependent anion channel 3 | Homo sapiens |
| 6801 | striatin, calmodulin binding protein | Homo sapiens |
| 64393 | zinc finger, matrin type 3 | Homo sapiens |
| 23333 | dpy-19-like 1 (C. elegans); similar to hCG1645499 | Homo sapiens |
| 9665 | KIAA0430 | Homo sapiens |
| 51263 | mitochondrial ribosomal protein L30 | Homo sapiens |
| 4145 | megakaryocyte-associated tyrosine kinase | Homo sapiens |
| 29985 | solute carrier family 39 (zinc transporter), member 3 | Homo sapiens |
| 139886 | spindlin family, member 4 | Homo sapiens |
| 154796 | angiomotin | Homo sapiens |
| 25972 | unc-50 homolog (C. elegans) | Homo sapiens |
| 8531 | cold shock domain protein A; cold shock domain protein A pseudogene 1 | Homo sapiens |
| 29057 | family with sequence similarity 156, member A; family with sequence similarity 156, member B | Homo sapiens |
| 727866 | family with sequence similarity 156, member A; family with sequence similarity 156, member B | Homo sapiens |
| 4641 | myosin IC | Homo sapiens |
| 55845 | chromosome 3 open reading frame 10 | Homo sapiens |
| 80011 | NEFA-interacting nuclear protein NIP30 | Homo sapiens |
| 7763 | similar to zinc finger, AN1-type domain 5; zinc finger, AN1-type domain 5 | Homo sapiens |
| 60314 | chromosome 12 open reading frame 10 | Homo sapiens |
| 285172 | family with sequence similarity 126, member B | Homo sapiens |
| 259217 | heat shock 70kDa protein 12A | Homo sapiens |
| 64376 | IKAROS family zinc finger 5 (Pegasus) | Homo sapiens |
| 54482 | coiled-coil domain containing 76 | Homo sapiens |
| 27258 | LSM3 homolog, U6 small nuclear RNA associated (S. cerevisiae); similar to Lsm3 protein | Homo sapiens |
| 10622 | polymerase (RNA) III (DNA directed) polypeptide G (32kD) | Homo sapiens |
| 1362 | carboxypeptidase D | Homo sapiens |
| 51188 | synovial sarcoma translocation gene on chromosome 18-like 2 | Homo sapiens |
| 153642 | arylsulfatase family, member K | Homo sapiens |
| 7743 | zinc finger protein 189 | Homo sapiens |
| 38 | acetyl-Coenzyme A acetyltransferase 1 | Homo sapiens |
| 23583 | single-strand-selective monofunctional uracil-DNA glycosylase 1 | Homo sapiens |
| 23516 | solute carrier family 39 (zinc transporter), member 14 | Homo sapiens |

| | | |
|---|---|---|
| 9712 | USP6 N-terminal like | Homo sapiens |
| 51780 | lysine (K)-specific demethylase 3B | Homo sapiens |
| 2098 | esterase D/formylglutathione hydrolase | Homo sapiens |
| 11236 | ring finger protein 139 | Homo sapiens |
| 8669 | eukaryotic translation initiation factor 3, subunit J | Homo sapiens |
| 493753 | chromosome 2 open reading frame 64 | Homo sapiens |
| 29074 | mitochondrial ribosomal protein L18 | Homo sapiens |
| 8566 | pyridoxal (pyridoxine, vitamin B6) kinase | Homo sapiens |
| 57504 | metastasis associated 1 family, member 3 | Homo sapiens |
| 7552 | zinc finger protein 711 | Homo sapiens |
| 114327 | EF-hand domain (C-terminal) containing 1 | Homo sapiens |
| 83640 | family with sequence similarity 103, member A1 | Homo sapiens |
| 441478 | NOTCH-regulated ankyrin repeat protein | Homo sapiens |
| 6839 | suppressor of variegation 3-9 homolog 1 (Drosophila) | Homo sapiens |
| 79970 | zinc finger family member 767 | Homo sapiens |
| 23332 | cytoplasmic linker associated protein 1 | Homo sapiens |
| 126282 | tumor necrosis factor, alpha-induced protein 8-like 1 | Homo sapiens |
| 5983 | replication factor C (activator 1) 3, 38kDa | Homo sapiens |
| 6483 | ST3 beta-galactoside alpha-2,3-sialyltransferase 2 | Homo sapiens |
| 283337 | zinc finger protein 740 | Homo sapiens |
| 645 | biliverdin reductase B (flavin reductase (NADPH)) | Homo sapiens |
| 4288 | antigen identified by monoclonal antibody Ki-67 | Homo sapiens |
| 403341 | zinc finger and BTB domain containing 34 | Homo sapiens |
| 5279 | phosphatidylinositol glycan anchor biosynthesis, class C | Homo sapiens |
| 1622 | diazepam binding inhibitor (GABA receptor modulator, acyl-Coenzyme A binding protein) | Homo sapiens |
| 83543 | allograft inflammatory factor 1-like | Homo sapiens |
| 23357 | angel homolog 1 (Drosophila) | Homo sapiens |
| 22890 | zinc finger and BTB domain containing 1 | Homo sapiens |
| 340544 | hypothetical protein LOC340544 | Homo sapiens |
| 23160 | WD repeat domain 43 | Homo sapiens |
| 4925 | nucleobindin 2 | Homo sapiens |
| 6671 | Sp4 transcription factor | Homo sapiens |
| 481 | ATPase, Na+/K+ transporting, beta 1 polypeptide | Homo sapiens |
| 27068 | pyrophosphatase (inorganic) 2 | Homo sapiens |
| 79004 | CUE domain containing 2 | Homo sapiens |
| 22 | ATP-binding cassette, sub-family B (MDR/TAP), member 7 | Homo sapiens |
| 57184 | chromosome 15 open reading frame 17 | Homo sapiens |
| 22938 | SNW domain containing 1 | Homo sapiens |
| 28976 | acyl-Coenzyme A dehydrogenase family, member 9 | Homo sapiens |
| 121536 | AE binding protein 2 | Homo sapiens |
| 22859 | latrophilin 1 | Homo sapiens |
| 219927 | mitochondrial ribosomal protein L21 | Homo sapiens |
| 51550 | cyclin-dependent kinase 2-interacting protein | Homo sapiens |
| 133383 | chromosome 5 open reading frame 35 | Homo sapiens |
| 10954 | protein disulfide isomerase family A, member 5 | Homo sapiens |
| 7090 | transducin-like enhancer of split 3 (E(sp1) homolog, Drosophila) | Homo sapiens |
| 441951 | chromosome 20 open reading frame 199 | Homo sapiens |
| 2509 | ferritin, heavy polypeptide pseudogene 1 | Homo sapiens |
| 57820 | cyclin B1 interacting protein 1 | Homo sapiens |
| 146923 | RUN domain containing 1 | Homo sapiens |
| 84726 | HLA-B associated transcript 2-like | Homo sapiens |
| 103910 | myosin, light chain 12B, regulatory | Homo sapiens |
| 134637 | adenosine deaminase, tRNA-specific 2, TAD2 homolog (S. cerevisiae) | Homo sapiens |
| 9885 | oxysterol binding protein-like 2 | Homo sapiens |
| 5738 | prostaglandin F2 receptor negative regulator | Homo sapiens |
| 9604 | ring finger protein 14 | Homo sapiens |
| 1528 | cytochrome b5 type A (microsomal) | Homo sapiens |
| 51507 | chromosome 20 open reading frame 43 | Homo sapiens |
| 22907 | DEAH (Asp-Glu-Ala-His) box polypeptide 30 | Homo sapiens |
| 2181 | acyl-CoA synthetase long-chain family member 3 | Homo sapiens |
| 80025 | pantothenate kinase 2 | Homo sapiens |
| 8915 | B-cell CLL/lymphoma 10; hypothetical LOC646626 | Homo sapiens |
| 133686 | chromosome 5 open reading frame 33 | Homo sapiens |
| 11342 | ring finger protein 13 | Homo sapiens |
| 10063 | COX17 cytochrome c oxidase assembly homolog (S. cerevisiae) | Homo sapiens |
| 27013 | chromosome 2 open reading frame 24 | Homo sapiens |
| 27065 | DNA segment on chromosome 4 (unique) 234 expressed sequence | Homo sapiens |
| 546 | alpha thalassemia/mental retardation syndrome X-linked (RAD54 homolog, S. cerevisiae) | Homo sapiens |
| 23230 | vacuolar protein sorting 13 homolog A (S. cerevisiae) | Homo sapiens |
| 57198 | ATPase, class I, type 8B, member 2 | Homo sapiens |
| 25915 | NADH dehydrogenase (ubiquinone) 1 alpha subcomplex, assembly factor 3 | Homo sapiens |
| 23503 | zinc finger, FYVE domain containing 26 | Homo sapiens |
| 7867 | mitogen-activated protein kinase-activated protein kinase 3 | Homo sapiens |
| 11196 | SEC23 interacting protein | Homo sapiens |
| 89970 | ring finger and SPRY domain containing 1 | Homo sapiens |
| 89122 | tripartite motif-containing 4 | Homo sapiens |
| 5606 | mitogen-activated protein kinase kinase 3 | Homo sapiens |

| | | |
|---|---|---|
| 56952 | phosphoribosyl transferase domain containing 1 | Homo sapiens |
| 431707 | LIM homeobox 8 | Homo sapiens |
| 54494 | chromosome 11 open reading frame 71 | Homo sapiens |
| 79035 | oligonucleotide/oligosaccharide-binding fold containing 2B | Homo sapiens |
| 56256 | SERTA domain containing 4 | Homo sapiens |
| 55364 | Impact homolog (mouse) | Homo sapiens |
| 84331 | chromosome 16 open reading frame 14 | Homo sapiens |
| 285313 | immunoglobulin superfamily, member 10 | Homo sapiens |
| 55270 | nudix (nucleoside diphosphate linked moiety X)-type motif 15 | Homo sapiens |
| 1476 | cystatin B (stefin B) | Homo sapiens |
| 10939 | AFG3 ATPase family gene 3-like 2 (yeast) | Homo sapiens |
| 9987 | heterogeneous nuclear ribonucleoprotein D-like | Homo sapiens |
| 85028 | small nucleolar RNA host gene 12 (non-protein coding) | Homo sapiens |
| 6305 | SET binding factor 1; SET binding factor 1 pseudogene 1 | Homo sapiens |
| 285888 | canopy 1 homolog (zebrafish) | Homo sapiens |
| 6234 | ribosomal protein S28 pseudogene 6; ribosomal protein S28 pseudogene 9; ribosomal protein S28 | Homo sapiens |
| 118472 | zinc finger protein 511 | Homo sapiens |
| 4204 | methyl CpG binding protein 2 (Rett syndrome) | Homo sapiens |
| 65123 | integrator complex subunit 3 | Homo sapiens |
| 4684 | neural cell adhesion molecule 1 | Homo sapiens |
| 84162 | KIAA1109 | Homo sapiens |
| 3716 | Janus kinase 1 | Homo sapiens |
| 88745 | chromosome 6 open reading frame 153 | Homo sapiens |
| 2491 | centromere protein I | Homo sapiens |
| 701 | budding uninhibited by benzimidazoles 1 homolog beta (yeast) | Homo sapiens |
| 80144 | Fraser syndrome 1 | Homo sapiens |
| 3109 | major histocompatibility complex, class II, DM beta | Homo sapiens |
| 5528 | protein phosphatase 2, regulatory subunit B', delta isoform | Homo sapiens |
| 25929 | gem (nuclear organelle) associated protein 5 | Homo sapiens |
| 51400 | protein phosphatase methylesterase 1 | Homo sapiens |
| 3065 | histone deacetylase 1 | Homo sapiens |
| 55246 | coiled-coil domain containing 25 | Homo sapiens |
| 29997 | glioma tumor suppressor candidate region gene 2; glioma tumor suppressor candidate region gene 2 pseudogene | Homo sapiens |
| 4302 | myeloid/lymphoid or mixed-lineage leukemia (trithorax homolog, Drosophila); translocated to, 6 | Homo sapiens |
| 11270 | nurim (nuclear envelope membrane protein) | Homo sapiens |
| 10797 | methylenetetrahydrofolate dehydrogenase (NADP+ dependent) 2, methenyltetrahydrofolate cyclohydrolase | Homo sapiens |
| 57038 | arginyl-tRNA synthetase 2, mitochondrial | Homo sapiens |
| 9743 | Rho GTPase-activating protein | Homo sapiens |
| 3205 | homeobox A9 | Homo sapiens |
| 23314 | SATB homeobox 2 | Homo sapiens |
| 9147 | serologically defined colon cancer antigen 1 | Homo sapiens |
| 54878 | dipeptidyl-peptidase 8 | Homo sapiens |
| 440 | asparagine synthetase | Homo sapiens |
| 90321 | zinc finger protein 766 | Homo sapiens |
| 79629 | occludin/ELL domain containing 1 | Homo sapiens |
| 687 | Kruppel-like factor 9 | Homo sapiens |
| 79796 | asparagine-linked glycosylation 9, alpha-1,2-mannosyltransferase homolog (S. cerevisiae) | Homo sapiens |
| 1327 | cytochrome c oxidase subunit IV isoform 1 | Homo sapiens |
| 4052 | latent transforming growth factor beta binding protein 1 | Homo sapiens |
| 5437 | polymerase (RNA) II (DNA directed) polypeptide H | Homo sapiens |
| 9252 | ribosomal protein S6 kinase, 90kDa, polypeptide 5 | Homo sapiens |
| 54606 | DEAD (Asp-Glu-Ala-Asp) box polypeptide 56 | Homo sapiens |
| 8565 | tyrosyl-tRNA synthetase | Homo sapiens |
| 83879 | cell division cycle associated 7 | Homo sapiens |
| 734 | oxidative stress induced growth inhibitor family member 2 | Homo sapiens |
| 136 | hypothetical LOC100131909; adenosine A2b receptor | Homo sapiens |
| 25926 | nucleolar protein 11 | Homo sapiens |
| 55972 | solute carrier family 25, member 40 | Homo sapiens |
| 153222 | chromosome 5 open reading frame 41 | Homo sapiens |
| 51639 | splicing factor 3B, 14 kDa subunit | Homo sapiens |
| 25843 | MOB1, Mps One Binder kinase activator-like 3 (yeast) | Homo sapiens |
| 760 | carbonic anhydrase II | Homo sapiens |
| 79074 | chromosome 2 open reading frame 49 | Homo sapiens |
| 256714 | MAP7 domain containing 2 | Homo sapiens |
| 4175 | minichromosome maintenance complex component 6 | Homo sapiens |
| 54919 | HEAT repeat containing 2 | Homo sapiens |
| 8801 | similar to sucb; succinate-CoA ligase, GDP-forming, beta subunit | Homo sapiens |
| 728485 | hypothetical protein LOC728485 | Homo sapiens |
| 132 | adenosine kinase | Homo sapiens |
| 6390 | succinate dehydrogenase complex, subunit B, iron sulfur (Ip) | Homo sapiens |
| 246175 | CCR4-NOT transcription complex, subunit 6-like | Homo sapiens |
| 115 | adenylate cyclase 9 | Homo sapiens |
| 22936 | elongation factor, RNA polymerase II, 2 | Homo sapiens |

| | | |
|---|---|---|
| 347862 | Parkinson disease 7 domain containing 1 | Homo sapiens |
| 246721 | polymerase (RNA) II (DNA directed) polypeptide J3; polymerase (RNA) II (DNA directed) polypeptide J2 | Homo sapiens |
| 84934 | chromosome 12 open reading frame 52 | Homo sapiens |
| 5747 | PTK2 protein tyrosine kinase 2 | Homo sapiens |
| 6297 | sal-like 2 (Drosophila) | Homo sapiens |
| 56034 | platelet derived growth factor C | Homo sapiens |
| 10539 | glutaredoxin 3 | Homo sapiens |
| 57498 | kinase D-interacting substrate, 220kDa | Homo sapiens |
| 79832 | glutamine and serine rich 1 | Homo sapiens |
| 8243 | structural maintenance of chromosomes 1A | Homo sapiens |
| 8216 | leucine-zipper-like transcription regulator 1 | Homo sapiens |
| 79000 | chromosome 1 open reading frame 135 | Homo sapiens |
| 9745 | zinc finger protein 536 | Homo sapiens |
| 57128 | LYR motif containing 4 | Homo sapiens |
| 3640 | insulin-like 3 (Leydig cell) | Homo sapiens |
| 4942 | ornithine aminotransferase (gyrate atrophy) | Homo sapiens |
| 22908 | SAC1 suppressor of actin mutations 1-like (yeast) | Homo sapiens |
| 2140 | eyes absent homolog 3 (Drosophila) | Homo sapiens |
| 6176 | ribosomal protein, large, P1 | Homo sapiens |
| 309 | annexin A6 | Homo sapiens |
| 653687 | chromosome X open reading frame 50B; chromosome X open reading frame 50 | Homo sapiens |
| 26123 | tectonic family member 3 | Homo sapiens |
| 100132815 | hypothetical protein LOC100132815 | Homo sapiens |
| 493812 | HLA complex group 11 | Homo sapiens |
| 79036 | chromosome 19 open reading frame 50 | Homo sapiens |
| 55311 | zinc finger protein 444 | Homo sapiens |
| 140809 | sulfiredoxin 1 homolog (S. cerevisiae) | Homo sapiens |
| 6950 | hypothetical gene supported by BC000665; t-complex 1 | Homo sapiens |
| 10456 | HCLS1 associated protein X-1 | Homo sapiens |
| 55526 | dehydrogenase E1 and transketolase domain containing 1 | Homo sapiens |
| 51451 | leucine carboxyl methyltransferase 1 | Homo sapiens |
| 4683 | nibrin | Homo sapiens |
| 51719 | calcium binding protein 39 | Homo sapiens |
| 56886 | UDP-glucose ceramide glucosyltransferase-like 1 | Homo sapiens |
| 127281 | chromosome 1 open reading frame 93 | Homo sapiens |
| 55615 | Rho GTPase activating protein 8; proline rich 5 (renal); PRR5-ARHGAP8 fusion | Homo sapiens |
| 23779 | Rho GTPase activating protein 8; proline rich 5 (renal); PRR5-ARHGAP8 fusion | Homo sapiens |
| 553158 | Rho GTPase activating protein 8; proline rich 5 (renal); PRR5-ARHGAP8 fusion | Homo sapiens |
| 283871 | phosphoglycolate phosphatase | Homo sapiens |
| 57722 | immunoglobulin superfamily, DCC subclass, member 4 | Homo sapiens |
| 3945 | lactate dehydrogenase B | Homo sapiens |
| 7474 | wingless-type MMTV integration site family, member 5A | Homo sapiens |
| 140883 | zinc finger protein 280B | Homo sapiens |
| 11333 | PDGFA associated protein 1; similar to PDGFA associated protein 1 | Homo sapiens |
| 161779 | piggyBac transposable element derived 4 | Homo sapiens |
| 286053 | non-SMC element 2, MMS21 homolog (S. cerevisiae) | Homo sapiens |
| 9070 | ash2 (absent, small, or homeotic)-like (Drosophila) | Homo sapiens |
| 64769 | chromosome 1 open reading frame 149 | Homo sapiens |
| 143684 | family with sequence similarity 76, member B | Homo sapiens |
| 80146 | UDP-glucuronate decarboxylase 1 | Homo sapiens |
| 48 | aconitase 1, soluble | Homo sapiens |
| 10287 | regulator of G-protein signaling 19 | Homo sapiens |
| 65244 | spermatogenesis associated, serine-rich 2 | Homo sapiens |
| 1337 | cytochrome c oxidase subunit VIa polypeptide 1 | Homo sapiens |
| 50804 | myelin expression factor 2 | Homo sapiens |
| 119 | adducin 2 (beta) | Homo sapiens |
| 5160 | pyruvate dehydrogenase (lipoamide) alpha 1 | Homo sapiens |
| 92335 | STE20-related kinase adaptor alpha | Homo sapiens |
| 91689 | chromosome 22 open reading frame 32 | Homo sapiens |
| 80764 | THAP domain containing 7 | Homo sapiens |
| 55553 | SRY (sex determining region Y)-box 6 | Homo sapiens |
| 6993 | dynein, light chain, Tctex-type 1 | Homo sapiens |
| 261726 | TIP41, TOR signaling pathway regulator-like (S. cerevisiae) | Homo sapiens |
| 81671 | transmembrane protein 49 | Homo sapiens |
| 79830 | zinc finger, MYM-type 1 | Homo sapiens |
| 221955 | diacylglycerol lipase, beta | Homo sapiens |
| 11152 | WD repeat domain 45 | Homo sapiens |
| 388403 | yippee-like 2 (Drosophila) | Homo sapiens |
| 9167 | cytochrome c oxidase subunit VIIa polypeptide 2 like | Homo sapiens |
| 253943 | YTH domain family, member 3 | Homo sapiens |
| 51069 | mitochondrial ribosomal protein L2 | Homo sapiens |
| 5636 | phosphoribosyl pyrophosphate synthetase-associated protein 2 | Homo sapiens |
| 220042 | chromosome 11 open reading frame 82 | Homo sapiens |
| 10280 | sigma non-opioid intracellular receptor 1 | Homo sapiens |
| 5163 | pyruvate dehydrogenase kinase, isozyme 1 | Homo sapiens |
| 9263 | serine/threonine kinase 17a | Homo sapiens |

| | | |
|---|---|---|
| 8420 | small nucleolar RNA host gene 3 (non-protein coding) | Homo sapiens |
| 23649 | polymerase (DNA directed), alpha 2 (70kD subunit) | Homo sapiens |
| 8975 | ubiquitin specific peptidase 13 (isopeptidase T-3) | Homo sapiens |
| 26747 | nuclear fragile X mental retardation protein interacting protein 1 | Homo sapiens |
| 9878 | TOX high mobility group box family member 4 | Homo sapiens |
| 151176 | family with sequence similarity 132, member B | Homo sapiens |
| 2762 | GDP-mannose 4,6-dehydratase | Homo sapiens |
| 55103 | Ral GEF with PH domain and SH3 binding motif 2 | Homo sapiens |
| 10204 | nuclear transport factor 2 | Homo sapiens |
| 25829 | transmembrane protein 184B | Homo sapiens |
| 6433 | splicing factor, arginine/serine-rich 8 (suppressor-of-white-apricot homolog, Drosophila) | Homo sapiens |
| 5716 | proteasome (prosome, macropain) 26S subunit, non-ATPase, 10 | Homo sapiens |
| 378 | ADP-ribosylation factor 4 | Homo sapiens |
| 83700 | junctional adhesion molecule 3 | Homo sapiens |
| 84498 | family with sequence similarity 120B | Homo sapiens |
| 55010 | chromosome 12 open reading frame 48 | Homo sapiens |
| 29916 | sorting nexin 11 | Homo sapiens |
| 5291 | phosphoinositide-3-kinase, catalytic, beta polypeptide | Homo sapiens |
| 1656 | DEAD (Asp-Glu-Ala-Asp) box polypeptide 6 | Homo sapiens |
| 3191 | similar to heterogeneous nuclear ribonucleoprotein L-like; heterogeneous nuclear ribonucleoprotein L | Homo sapiens |
| 54865 | G patch domain containing 4 | Homo sapiens |
| 4724 | NADH dehydrogenase (ubiquinone) Fe-S protein 4, 18kDa (NADH-coenzyme Q reductase) | Homo sapiens |
| 254394 | minichromosome maintenance complex component 9 | Homo sapiens |
| 5077 | paired box 3 | Homo sapiens |
| 5525 | protein phosphatase 2, regulatory subunit B', alpha isoform | Homo sapiens |
| 11040 | pim-2 oncogene | Homo sapiens |
| 25776 | chibby homolog 1 (Drosophila) | Homo sapiens |
| 55905 | ring finger protein 114 | Homo sapiens |
| 729021 | hypothetical protein LOC729021 | Homo sapiens |
| 5980 | REV3-like, catalytic subunit of DNA polymerase zeta (yeast) | Homo sapiens |
| 10114 | homeodomain interacting protein kinase 3 | Homo sapiens |
| 6391 | succinate dehydrogenase complex, subunit C, integral membrane protein, 15kDa | Homo sapiens |
| 6713 | squalene epoxidase | Homo sapiens |
| 56940 | similar to mitogen-activated protein kinase phosphatase x; dual specificity phosphatase 22 | Homo sapiens |
| 57647 | DEAH (Asp-Glu-Ala-His) box polypeptide 37 | Homo sapiens |
| 80114 | bicaudal C homolog 1 (Drosophila) | Homo sapiens |
| 642236 | similar to FRG1 protein (FSHD region gene 1 protein) | Homo sapiens |
| 5439 | polymerase (RNA) II (DNA directed) polypeptide J, 13.3kDa | Homo sapiens |
| 9262 | serine/threonine kinase 17b | Homo sapiens |
| 401317 | hypothetical LOC401317 | Homo sapiens |
| 84864 | MYC induced nuclear antigen | Homo sapiens |
| 2054 | syntaxin 2 | Homo sapiens |
| 51566 | armadillo repeat containing, X-linked 3 | Homo sapiens |
| 9733 | squamous cell carcinoma antigen recognized by T cells 3 | Homo sapiens |
| 64062 | RNA binding motif protein 26 | Homo sapiens |
| 65125 | WNK lysine deficient protein kinase 1; hypothetical LOC100132369 | Homo sapiens |
| 57655 | GRAM domain containing 1A | Homo sapiens |
| 57685 | cache domain containing 1 | Homo sapiens |
| 1719 | dihydrofolate reductase | Homo sapiens |
| 56965 | poly (ADP-ribose) polymerase family, member 6 | Homo sapiens |
| 26135 | SERPINE1 mRNA binding protein 1 | Homo sapiens |
| 23236 | phospholipase C, beta 1 (phosphoinositide-specific) | Homo sapiens |
| 90871 | chromosome 9 open reading frame 123 | Homo sapiens |
| 4704 | NADH dehydrogenase (ubiquinone) 1 alpha subcomplex, 9, 39kDa | Homo sapiens |
| 92400 | RNA binding motif protein 18 | Homo sapiens |
| 4355 | membrane protein, palmitoylated 2 (MAGUK p55 subfamily member 2) | Homo sapiens |
| 57488 | family with sequence similarity 62 (C2 domain containing), member B | Homo sapiens |
| 58505 | oligosaccharyltransferase complex subunit; similar to DC2 protein | Homo sapiens |
| 661 | polymerase (RNA) III (DNA directed) polypeptide D, 44kDa | Homo sapiens |
| 29968 | chromosome 8 open reading frame 62; phosphoserine aminotransferase 1 | Homo sapiens |
| 2801 | golgi autoantigen, golgin subfamily a, 2 | Homo sapiens |
| 2241 | fer (fps/fes related) tyrosine kinase | Homo sapiens |
| 65264 | ubiquitin-conjugating enzyme E2Z | Homo sapiens |
| 55656 | integrator complex subunit 8 | Homo sapiens |
| 80829 | zinc finger protein 91 homolog (mouse); ZFP91-CNTF readthrough transcript; ciliary neurotrophic factor | Homo sapiens |
| 4905 | N-ethylmaleimide-sensitive factor | Homo sapiens |
| 85359 | DiGeorge syndrome critical region gene 6-like | Homo sapiens |
| 9646 | Ctr9, Paf1/RNA polymerase II complex component, homolog (S. cerevisiae) | Homo sapiens |
| 115286 | solute carrier family 25, member 26 | Homo sapiens |
| 9587 | MAD2L1 binding protein | Homo sapiens |
| 126272 | EP300 interacting inhibitor of differentiation 2B | Homo sapiens |
| 114044 | MCM3AP antisense RNA (non-protein coding) | Homo sapiens |
| 51465 | ubiquitin-conjugating enzyme E2, J1 (UBC6 homolog, yeast) | Homo sapiens |

| | | |
|---|---|---|
| 51491 | NOP16 nucleolar protein homolog (yeast) | Homo sapiens |
| 27243 | chromatin modifying protein 2A | Homo sapiens |
| 6251 | Ras suppressor protein 1 | Homo sapiens |
| 51138 | COP9 constitutive photomorphogenic homolog subunit 4 (Arabidopsis) | Homo sapiens |
| 10921 | similar to ribonucleic acid binding protein S1; RNA binding protein S1, serine-rich domain | Homo sapiens |
| 8079 | myeloid leukemia factor 2 | Homo sapiens |
| 6598 | SWI/SNF related, matrix associated, actin dependent regulator of chromatin, subfamily b, member 1 | Homo sapiens |
| 255783 | hypothetical protein LOC255783 | Homo sapiens |
| 2870 | G protein-coupled receptor kinase 6 | Homo sapiens |
| 8715 | nucleolar protein 4 | Homo sapiens |
| 91408 | basic transcription factor 3-like 4; similar to hCG2008008 | Homo sapiens |
| 128240 | apolipoprotein A-I binding protein | Homo sapiens |
| 6732 | SFRS protein kinase 1 | Homo sapiens |
| 4363 | ATP-binding cassette, sub-family C (CFTR/MRP), member 1 | Homo sapiens |
| 3949 | low density lipoprotein receptor | Homo sapiens |
| 10473 | high mobility group nucleosomal binding domain 4 | Homo sapiens |
| 3157 | 3-hydroxy-3-methylglutaryl-Coenzyme A synthase 1 (soluble) | Homo sapiens |
| 155066 | ATPase, H+ transporting V0 subunit e2 | Homo sapiens |
| 282969 | chromosome 10 open reading frame 125 | Homo sapiens |
| 170685 | nudix (nucleoside diphosphate linked moiety X)-type motif 10 | Homo sapiens |
| 126731 | chromosome 1 open reading frame 96 | Homo sapiens |
| 55500 | ethanolamine kinase 1 | Homo sapiens |
| 116028 | chromosome 16 open reading frame 75 | Homo sapiens |
| 79801 | SHC SH2-domain binding protein 1 | Homo sapiens |
| 201562 | protein tyrosine phosphatase-like (proline instead of catalytic arginine), member b | Homo sapiens |
| 3939 | lactate dehydrogenase A | Homo sapiens |
| 151194 | family with sequence similarity 119, member A | Homo sapiens |
| 51192 | chemokine-like factor | Homo sapiens |
| 6472 | serine hydroxymethyltransferase 2 (mitochondrial) | Homo sapiens |
| 9209 | leucine rich repeat (in FLII) interacting protein 2 | Homo sapiens |
| 151742 | protein phosphatase 1 (formerly 2C)-like | Homo sapiens |
| 5152 | phosphodiesterase 9A | Homo sapiens |
| 1762 | dystrophia myotonica, WD repeat containing | Homo sapiens |
| 6227 | ribosomal protein S21 | Homo sapiens |
| 5827 | hypothetical LOC100129532; peroxisomal membrane protein 2, 22kDa | Homo sapiens |
| 5018 | oxidase (cytochrome c) assembly 1-like | Homo sapiens |
| 2739 | glyoxalase I | Homo sapiens |
| 3842 | transportin 1 | Homo sapiens |
| 154810 | angiomotin like 1 | Homo sapiens |
| 55172 | chromosome 14 open reading frame 104 | Homo sapiens |
| 285521 | COX18 cytochrome c oxidase assembly homolog (S. cerevisiae) | Homo sapiens |
| 6430 | splicing factor, arginine/serine-rich 5 | Homo sapiens |
| 4090 | SMAD family member 5 | Homo sapiens |
| 387103 | chromosome 6 open reading frame 173 | Homo sapiens |
| 201595 | STT3, subunit of the oligosaccharyltransferase complex, homolog B (S. cerevisiae) | Homo sapiens |
| 8568 | ribosomal RNA processing 1 homolog (S. cerevisiae) | Homo sapiens |
| 9166 | estrogen receptor binding site associated, antigen, 9 | Homo sapiens |
| 6383 | syndecan 2 | Homo sapiens |
| 26263 | FBXO22 opposite strand (non-protein coding); F-box protein 22 | Homo sapiens |
| 23469 | PHD finger protein 3 | Homo sapiens |
| 55771 | proline rich 11 | Homo sapiens |
| 415116 | pim-3 oncogene | Homo sapiens |
| 80150 | asparaginase like 1 | Homo sapiens |
| 8087 | fragile X mental retardation, autosomal homolog 1 | Homo sapiens |
| 55787 | chromosome X open reading frame 15 | Homo sapiens |
| 55088 | chromosome 10 open reading frame 118 | Homo sapiens |
| 54541 | DNA-damage-inducible transcript 4 | Homo sapiens |
| 55915 | LanC lantibiotic synthetase component C-like 2 (bacterial) | Homo sapiens |
| 140688 | chromosome 20 open reading frame 112 | Homo sapiens |
| 255812 | succinate dehydrogenase complex, subunit A, flavoprotein pseudogene 1 | Homo sapiens |
| 51110 | lactamase, beta 2 | Homo sapiens |
| 10493 | vesicle amine transport protein 1 homolog (T. californica) | Homo sapiens |
| 4036 | low density lipoprotein-related protein 2 | Homo sapiens |
| 90701 | SEC11 homolog C (S. cerevisiae) | Homo sapiens |
| 81034 | solute carrier family 25, member 32 | Homo sapiens |
| 1312 | catechol-O-methyltransferase | Homo sapiens |
| 6428 | splicing factor, arginine/serine-rich 3 | Homo sapiens |
| 27097 | TAF5-like RNA polymerase II, p300/CBP-associated factor (PCAF)-associated factor, 65kDa | Homo sapiens |
| 100132999 | hypothetical protein LOC100132999 | Homo sapiens |
| 8417 | syntaxin 7 | Homo sapiens |
| 3110 | motor neuron and pancreas homeobox 1 | Homo sapiens |
| 7203 | chaperonin containing TCP1, subunit 3 (gamma) | Homo sapiens |
| 84326 | chromosome 16 open reading frame 13 | Homo sapiens |
| 65008 | mitochondrial ribosomal protein L1 | Homo sapiens |
| 5326 | pleiomorphic adenoma gene-like 2; similar to pleiomorphic adenoma gene-like 2 | Homo sapiens |

| | | |
|---|---|---|
| 6396 | SEC13 homolog (S. cerevisiae) | Homo sapiens |
| 10492 | synaptotagmin binding, cytoplasmic RNA interacting protein | Homo sapiens |
| 11285 | xylosylprotein beta 1,4-galactosyltransferase, polypeptide 7 (galactosyltransferase I) | Homo sapiens |
| 116151 | chromosome 20 open reading frame 108 | Homo sapiens |
| 23299 | bicaudal D homolog 2 (Drosophila) | Homo sapiens |
| 5578 | protein kinase C, alpha | Homo sapiens |
| 84833 | up-regulated during skeletal muscle growth 5 homolog (mouse) | Homo sapiens |
| 9825 | spermatogenesis associated 2 | Homo sapiens |
| 23012 | serine/threonine kinase 38 like | Homo sapiens |
| 56479 | potassium voltage-gated channel, KQT-like subfamily, member 5 | Homo sapiens |
| 5257 | phosphorylase kinase, beta | Homo sapiens |
| 9092 | squamous cell carcinoma antigen recognized by T cells | Homo sapiens |
| 51696 | headcase homolog (Drosophila) | Homo sapiens |
| 84159 | AT rich interactive domain 5B (MRF1-like) | Homo sapiens |
| 115548 | FCH domain only 2 | Homo sapiens |
| 5604 | mitogen-activated protein kinase kinase 1 | Homo sapiens |
| 83443 | splicing factor 3b, subunit 5, 10kDa | Homo sapiens |
| 6457 | SH3-domain GRB2-like 3 | Homo sapiens |
| 10269 | zinc metallopeptidase (STE24 homolog, S. cerevisiae) | Homo sapiens |
| 23211 | zinc finger CCCH-type containing 4 | Homo sapiens |
| 81605 | ubiquitin related modifier 1 homolog (S. cerevisiae) | Homo sapiens |
| 375444 | chromosome 5 open reading frame 34 | Homo sapiens |
| 10505 | sema domain, immunoglobulin domain (Ig), transmembrane domain (TM) and short cytoplasmic domain, (semaphorin) 4F | Homo sapiens |
| 23191 | cytoplasmic FMR1 interacting protein 1 | Homo sapiens |
| 4089 | SMAD family member 4 | Homo sapiens |
| 54850 | F-box and leucine-rich repeat protein 12 | Homo sapiens |
| 79710 | MORC family CW-type zinc finger 4 | Homo sapiens |
| 23141 | ankyrin repeat and LEM domain containing 2 | Homo sapiens |
| 644 | biliverdin reductase A | Homo sapiens |
| 10209 | similar to eukaryotic translation initiation factor 1; eukaryotic translation initiation factor 1 | Homo sapiens |
| 160518 | DENN/MADD domain containing 5B | Homo sapiens |
| 25778 | dual serine/threonine and tyrosine protein kinase | Homo sapiens |
| 57659 | zinc finger and BTB domain containing 4 | Homo sapiens |
| 100132733 | similar to FLJ00310 protein | Homo sapiens |
| 219541 | mediator complex subunit 19 | Homo sapiens |
| 5209 | 6-phosphofructo-2-kinase/fructose-2,6-biphosphatase 3 | Homo sapiens |
| 100129637 | hypothetical LOC100129637 | Homo sapiens |
| 91947 | arrestin domain containing 4 | Homo sapiens |
| 4357 | mercaptopyruvate sulfurtransferase | Homo sapiens |
| 8994 | LIM domains containing 1 | Homo sapiens |
| 2197 | Finkel-Biskis-Reilly murine sarcoma virus (FBR-MuSV) ubiquitously expressed | Homo sapiens |
| 79707 | nucleolar protein 9 | Homo sapiens |
| 23212 | RRS1 ribosome biogenesis regulator homolog (S. cerevisiae) | Homo sapiens |
| 84896 | ATPase family, AAA domain containing 1 | Homo sapiens |
| 79693 | yrdC domain containing (E. coli) | Homo sapiens |
| 55388 | minichromosome maintenance complex component 10 | Homo sapiens |
| 2010 | emerin | Homo sapiens |
| 9770 | Ras association (RalGDS/AF-6) domain family member 2 | Homo sapiens |
| 26060 | adaptor protein, phosphotyrosine interaction, PH domain and leucine zipper containing 1 | Homo sapiens |
| 2055 | ceroid-lipofuscinosis, neuronal 8 (epilepsy, progressive with mental retardation) | Homo sapiens |
| 387923 | stress-associated endoplasmic reticulum protein family member 2 | Homo sapiens |
| 427 | N-acylsphingosine amidohydrolase (acid ceramidase) 1 | Homo sapiens |
| 2108 | electron-transfer-flavoprotein, alpha polypeptide | Homo sapiens |
| 387119 | chromosome 6 open reading frame 204 | Homo sapiens |
| 4077 | neighbor of BRCA1 gene 1 | Homo sapiens |
| 83990 | BRCA1 interacting protein C-terminal helicase 1 | Homo sapiens |
| 84455 | EF-hand calcium binding domain 7 | Homo sapiens |
| 166929 | sphingomyelin synthase 2 | Homo sapiens |
| 64223 | MTOR associated protein, LST8 homolog (S. cerevisiae) | Homo sapiens |
| 6603 | SWI/SNF related, matrix associated, actin dependent regulator of chromatin, subfamily d, member 2 | Homo sapiens |
| 10902 | bromodomain containing 8 | Homo sapiens |
| 9590 | A kinase (PRKA) anchor protein 12 | Homo sapiens |
| 64779 | methenyltetrahydrofolate synthetase domain containing | Homo sapiens |
| 3856 | keratin 8 pseudogene 9; similar to keratin 8; keratin 8 | Homo sapiens |
| 10467 | zinc finger, HIT type 1 | Homo sapiens |
| 6812 | syntaxin binding protein 1 | Homo sapiens |
| 79714 | coiled-coil domain containing 51 | Homo sapiens |
| 85457 | KIAA1737 | Homo sapiens |
| 10998 | solute carrier family 27 (fatty acid transporter), member 5 | Homo sapiens |
| 2101 | estrogen-related receptor alpha | Homo sapiens |
| 1998 | E74-like factor 2 (ets domain transcription factor) | Homo sapiens |
| 57713 | Scm-like with four mbt domains 2 | Homo sapiens |
| 1820 | AT rich interactive domain 3A (BRIGHT-like) | Homo sapiens |
| 7076 | TIMP metallopeptidase inhibitor 1 | Homo sapiens |

| | | |
|---|---|---|
| 27229 | tubulin, gamma complex associated protein 4 | Homo sapiens |
| 3223 | homeobox C6 | Homo sapiens |
| 64326 | ring finger and WD repeat domain 2 | Homo sapiens |
| 10521 | DEAD (Asp-Glu-Ala-Asp) box polypeptide 17 | Homo sapiens |
| 3921 | ribosomal protein SA pseudogene 9; ribosomal protein SA pseudogene 8; ribosomal protein SA pseudogene 58; ribosomal protein SA pseudogene 19; ribosomal protein SA pseudogene 18; ribosomal protein SA; ribosomal protein SA pseudogene 15; ribosomal protein SA pseudogene 61; ribosomal protein SA pseudogene 29; ribosomal protein SA pseudogene 12 | Homo sapiens |
| 11339 | Opa interacting protein 5 | Homo sapiens |
| 2625 | GATA binding protein 3 | Homo sapiens |
| 5977 | D4, zinc and double PHD fingers family 2 | Homo sapiens |
| 6175 | ribosomal protein, large, P0 pseudogene 2; ribosomal protein, large, P0 pseudogene 3; ribosomal protein, large, P0 pseudogene 6; ribosomal protein, large, P0 | Homo sapiens |
| 220717 | ribosomal protein, large, P0 pseudogene 2; ribosomal protein, large, P0 pseudogene 3; ribosomal protein, large, P0 pseudogene 6; ribosomal protein, large, P0 | Homo sapiens |
| 10207 | InaD-like (Drosophila) | Homo sapiens |
| 4893 | neuroblastoma RAS viral (v-ras) oncogene homolog | Homo sapiens |
| 114112 | thioredoxin reductase 3 | Homo sapiens |
| 64216 | transcription factor B2, mitochondrial | Homo sapiens |
| 221035 | receptor accessory protein 3 | Homo sapiens |
| 4287 | ataxin 3 | Homo sapiens |
| 55174 | integrator complex subunit 10 | Homo sapiens |
| 1933 | eukaryotic translation elongation factor 1 beta 2; eukaryotic translation elongation factor 1 beta 2-like | Homo sapiens |
| 157697 | glutamate-rich 1 | Homo sapiens |
| 647070 | hypothetical LOC647070 | Homo sapiens |
| 57107 | prenyl (decaprenyl) diphosphate synthase, subunit 2 | Homo sapiens |
| 10023 | frequently rearranged in advanced T-cell lymphomas | Homo sapiens |
| 284996 | ring finger protein 149 | Homo sapiens |
| 11047 | adhesion regulating molecule 1 | Homo sapiens |
| 26610 | elongation protein 4 homolog (S. cerevisiae) | Homo sapiens |
| 51321 | archaelysin family metallopeptidase 2 | Homo sapiens |
| 25840 | methyltransferase like 7A | Homo sapiens |
| 55133 | S1 RNA binding domain 1 | Homo sapiens |
| 8293 | small EDRK-rich factor 1A (telomeric); small EDRK-rich factor 1B (centromeric) | Homo sapiens |
| 728492 | small EDRK-rich factor 1A (telomeric); small EDRK-rich factor 1B (centromeric) | Homo sapiens |
| 578 | BCL2-antagonist/killer 1; BCL2-like 7 pseudogene 1 | Homo sapiens |
| 27241 | Bardet-Biedl syndrome 9 | Homo sapiens |
| 5885 | RAD21 homolog (S. pombe) | Homo sapiens |
| 1975 | similar to eukaryotic translation initiation factor 4H; eukaryotic translation initiation factor 4B | Homo sapiens |
| 23328 | SAM and SH3 domain containing 1 | Homo sapiens |
| 2909 | glucocorticoid receptor DNA binding factor 1 | Homo sapiens |
| 63932 | chromosome X open reading frame 56 | Homo sapiens |
| 1386 | activating transcription factor 2 | Homo sapiens |
| 148867 | solute carrier family 30 (zinc transporter), member 7 | Homo sapiens |
| 9960 | ubiquitin specific peptidase 3 | Homo sapiens |
| 8761 | poly(A) binding protein, cytoplasmic 4 (inducible form) | Homo sapiens |
| 665 | BCL2/adenovirus E1B 19kDa interacting protein 3-like | Homo sapiens |
| 400960 | hypothetical gene supported by BC040598 | Homo sapiens |
| 8496 | PTPRF interacting protein, binding protein 1 (liprin beta 1) | Homo sapiens |
| 57506 | mitochondrial antiviral signaling protein | Homo sapiens |
| 29922 | non-metastatic cells 7, protein expressed in (nucleoside-diphosphate kinase) | Homo sapiens |
| 51150 | stromal cell derived factor 4 | Homo sapiens |
| 7840 | Alstrom syndrome 1 | Homo sapiens |
| 168374 | zinc finger protein 92 | Homo sapiens |
| 5481 | peptidylprolyl isomerase D | Homo sapiens |
| 4211 | Meis homeobox 1 | Homo sapiens |
| 6141 | ribosomal protein L18 | Homo sapiens |
| 191 | adenosylhomocysteinase | Homo sapiens |
| 83871 | RAB34, member RAS oncogene family | Homo sapiens |
| 51755 | Cdc2-related kinase, arginine/serine-rich | Homo sapiens |
| 8226 | haloacid dehalogenase-like hydrolase domain containing 1A | Homo sapiens |
| 55163 | pyridoxamine 5'-phosphate oxidase | Homo sapiens |
| 4771 | neurofibromin 2 (merlin) | Homo sapiens |
| 7336 | ubiquitin-conjugating enzyme E2 variant 2 | Homo sapiens |
| 8805 | tripartite motif-containing 24 | Homo sapiens |
| 85364 | zinc finger, CCHC domain containing 3 | Homo sapiens |
| 8908 | glycogenin 2 | Homo sapiens |
| 55798 | methyltransferase like 2B | Homo sapiens |
| 22930 | RAB3 GTPase activating protein subunit 1 (catalytic) | Homo sapiens |
| 1163 | CDC28 protein kinase regulatory subunit 1B | Homo sapiens |
| 122786 | FERM domain containing 6 | Homo sapiens |
| 131965 | methyltransferase like 6 | Homo sapiens |
| 284900 | hypothetical LOC284900 | Homo sapiens |
| 58489 | family with sequence similarity 108, member C1 | Homo sapiens |
| 56776 | formin 2 | Homo sapiens |

| | | |
|---|---|---|
| 64431 | ARP6 actin-related protein 6 homolog (yeast) | Homo sapiens |
| 1434 | CSE1 chromosome segregation 1-like (yeast) | Homo sapiens |
| 26511 | cysteine-rich hydrophobic domain 2 | Homo sapiens |
| 80344 | WD repeat domain 23 | Homo sapiens |
| 801 | calmodulin 3 (phosphorylase kinase, delta); calmodulin 2 (phosphorylase kinase, delta); calmodulin 1 (phosphorylase kinase, delta) | Homo sapiens |
| 808 | calmodulin 3 (phosphorylase kinase, delta); calmodulin 2 (phosphorylase kinase, delta); calmodulin 1 (phosphorylase kinase, delta) | Homo sapiens |
| 805 | calmodulin 3 (phosphorylase kinase, delta); calmodulin 2 (phosphorylase kinase, delta); calmodulin 1 (phosphorylase kinase, delta) | Homo sapiens |
| 56675 | nuclear receptor interacting protein 3 | Homo sapiens |
| 7405 | UV radiation resistance associated gene | Homo sapiens |
| 10620 | AT rich interactive domain 3B (BRIGHT-like) | Homo sapiens |
| 57461 | ISY1 splicing factor homolog (S. cerevisiae) | Homo sapiens |
| 55763 | exocyst complex component 1 | Homo sapiens |
| 1503 | CTP synthase | Homo sapiens |
| 7846 | tubulin, alpha 1a | Homo sapiens |
| 7171 | tropomyosin 4 | Homo sapiens |
| 3612 | inositol(myo)-1(or 4)-monophosphatase 1 | Homo sapiens |
| 8270 | L antigen family, member 3 | Homo sapiens |
| 7025 | nuclear receptor subfamily 2, group F, member 1 | Homo sapiens |
| 23549 | aspartyl aminopeptidase | Homo sapiens |
| 172 | AFG3 ATPase family gene 3-like 1 (S. cerevisiae) | Homo sapiens |
| 113444 | chromosome 1 open reading frame 212 | Homo sapiens |
| 6452 | SH3-domain binding protein 2 | Homo sapiens |
| 202052 | DnaJ (Hsp40) homolog, subfamily C, member 18 | Homo sapiens |
| 219902 | transmembrane protein 136 | Homo sapiens |
| 6907 | transducin (beta)-like 1X-linked | Homo sapiens |
| 80196 | ring finger protein 34 | Homo sapiens |
| 79752 | zinc finger, AN1-type domain 1 | Homo sapiens |
| 54839 | leucine rich repeat containing 49; similar to leucine rich repeat containing 49 | Homo sapiens |
| 867 | Cas-Br-M (murine) ecotropic retroviral transforming sequence | Homo sapiens |
| 5805 | 6-pyruvoyltetrahydropterin synthase | Homo sapiens |
| 79137 | family with sequence similarity 134, member A | Homo sapiens |
| 5687 | proteasome (prosome, macropain) subunit, alpha type, 6 | Homo sapiens |
| 2781 | guanine nucleotide binding protein (G protein), alpha z polypeptide | Homo sapiens |
| 2309 | forkhead box O3; forkhead box O3B pseudogene | Homo sapiens |
| 2310 | forkhead box O3; forkhead box O3B pseudogene | Homo sapiens |
| 93627 | TBC domain-containing protein kinase-like | Homo sapiens |
| 120114 | FAT tumor suppressor homolog 3 (Drosophila) | Homo sapiens |
| 387328 | zinc finger protein 322B | Homo sapiens |
| 641298 | SMG1 homolog, phosphatidylinositol 3-kinase-related kinase pseudogene | Homo sapiens |
| 6632 | small nuclear ribonucleoprotein D1 polypeptide 16kDa; hypothetical protein LOC100129492 | Homo sapiens |
| 90956 | aarF domain containing kinase 2 | Homo sapiens |
| 51318 | mitochondrial ribosomal protein L35 | Homo sapiens |
| 8220 | DiGeorge syndrome critical region gene 14 | Homo sapiens |
| 4201 | male-enhanced antigen 1 | Homo sapiens |
| 78999 | leucine rich repeat and fibronectin type III domain containing 4 | Homo sapiens |
| 51290 | ERGIC and golgi 2 | Homo sapiens |
| 11080 | DnaJ (Hsp40) homolog, subfamily B, member 4 | Homo sapiens |
| 28982 | feline leukemia virus subgroup C cellular receptor 1 | Homo sapiens |
| 55347 | abhydrolase domain containing 10 | Homo sapiens |
| 8792 | tumor necrosis factor receptor superfamily, member 11a, NFKB activator | Homo sapiens |
| 54469 | zinc finger, AN1-type domain 6 | Homo sapiens |
| 389906 | hypothetical protein LOC441528; similar to hCG1981372; similar to Serine/threonine-protein kinase PRKX (Protein kinase PKX1) | Homo sapiens |
| 8214 | DiGeorge syndrome critical region gene 6 | Homo sapiens |
| 10075 | HECT, UBA and WWE domain containing 1 | Homo sapiens |
| 11245 | G protein-coupled receptor 176 | Homo sapiens |
| 341 | apolipoprotein C-I | Homo sapiens |
| 135114 | histidine triad nucleotide binding protein 3 | Homo sapiens |
| 51144 | hydroxysteroid (17-beta) dehydrogenase 12 | Homo sapiens |
| 84961 | F-box and leucine-rich repeat protein 20 | Homo sapiens |
| 84705 | GTP binding protein 3 (mitochondrial) | Homo sapiens |
| 51540 | selenocysteine lyase | Homo sapiens |
| 9392 | transforming growth factor, beta receptor associated protein 1 | Homo sapiens |
| 11065 | ubiquitin-conjugating enzyme E2C | Homo sapiens |
| 80020 | FAD-dependent oxidoreductase domain containing 2 | Homo sapiens |
| 79762 | chromosome 1 open reading frame 115 | Homo sapiens |
| 124045 | chromosome 16 open reading frame 55 | Homo sapiens |
| 3475 | interferon-related developmental regulator 1 | Homo sapiens |
| 55759 | WD repeat domain 12 | Homo sapiens |
| 22913 | RNA binding protein, autoantigenic (hnRNP-associated with lethal yellow homolog (mouse)) | Homo sapiens |
| 55206 | strawberry notch homolog 1 (Drosophila) | Homo sapiens |
| 9456 | homer homolog 1 (Drosophila) | Homo sapiens |
| 7029 | transcription factor Dp-2 (E2F dimerization partner 2) | Homo sapiens |
| 54620 | F-box and leucine-rich repeat protein 19 | Homo sapiens |

| | | |
|---|---|---|
| 4552 | 5-methyltetrahydrofolate-homocysteine methyltransferase reductase | Homo sapiens |
| 84148 | MYST histone acetyltransferase 1 | Homo sapiens |
| 3839 | karyopherin alpha 3 (importin alpha 4) | Homo sapiens |
| 1748 | distal-less homeobox 4 | Homo sapiens |
| 55854 | zinc finger CCCH-type containing 15 | Homo sapiens |
| 10683 | delta-like 3 (Drosophila) | Homo sapiens |
| 144363 | LYR motif containing 5 | Homo sapiens |
| 130827 | transmembrane protein 182 | Homo sapiens |
| 6160 | ribosomal protein L31 pseudogene 49; ribosomal protein L31 pseudogene 17; ribosomal protein L31 | Homo sapiens |
| 79095 | chromosome 9 open reading frame 16 | Homo sapiens |
| 146059 | congenital dyserythropoietic anemia, type I | Homo sapiens |
| 51574 | La ribonucleoprotein domain family, member 7 | Homo sapiens |
| 4715 | NADH dehydrogenase (ubiquinone) 1 beta subcomplex, 9, 22kDa | Homo sapiens |
| 3364 | HUS1 checkpoint homolog (S. pombe) | Homo sapiens |
| 64224 | HERPUD family member 2 | Homo sapiens |
| 1968 | eukaryotic translation initiation factor 2, subunit 3 gamma, 52kDa | Homo sapiens |
| 9057 | solute carrier family 7 (cationic amino acid transporter, y+ system), member 6 | Homo sapiens |
| 3203 | homeobox A6 | Homo sapiens |
| 400657 | hypothetical LOC400657 | Homo sapiens |
| 157680 | vacuolar protein sorting 13 homolog B (yeast) | Homo sapiens |
| 253842 | hypothetical protein LOC253842 | Homo sapiens |
| 339487 | zinc finger and BTB domain containing 8 opposite strand; similar to hCG1645309; similar to zinc finger and BTB domain containing 8 opposite strand | Homo sapiens |
| 56616 | diablo homolog (Drosophila) | Homo sapiens |
| 6921 | similar to elongin C; transcription elongation factor B (SIII), polypeptide 1 (15kDa, elongin C) | Homo sapiens |
| 22828 | RNA binding motif protein 16 | Homo sapiens |
| 80155 | NMDA receptor regulated 1 | Homo sapiens |
| 57189 | KIAA1147 | Homo sapiens |
| 157638 | family with sequence similarity 84, member B | Homo sapiens |
| 23607 | CD2-associated protein | Homo sapiens |
| 9946 | crystallin, zeta (quinone reductase)-like 1 | Homo sapiens |
| 7566 | zinc finger protein 18 | Homo sapiens |
| 285812 | hypothetical protein LOC285812 | Homo sapiens |
| 9694 | tetratricopeptide repeat domain 35 | Homo sapiens |
| 84300 | chromosome 6 open reading frame 125 | Homo sapiens |
| 254531 | lysophosphatidylcholine acyltransferase 4 | Homo sapiens |
| 10518 | calcium and integrin binding family member 2 | Homo sapiens |
| 8886 | DEAD (Asp-Glu-Ala-Asp) box polypeptide 18 | Homo sapiens |
| 23321 | tripartite motif-containing 2 | Homo sapiens |
| 56271 | brain expressed, X-linked 4 | Homo sapiens |
| 84541 | kelch repeat and BTB (POZ) domain containing 8 | Homo sapiens |
| 9188 | DEAD (Asp-Glu-Ala-Asp) box polypeptide 21 | Homo sapiens |
| 84269 | coiled-coil-helix-coiled-coil-helix domain containing 5 | Homo sapiens |
| 3895 | kinectin 1 (kinesin receptor) | Homo sapiens |
| 57062 | DEAD (Asp-Glu-Ala-Asp) box polypeptide 24 | Homo sapiens |
| 149951 | COMM domain containing 7 | Homo sapiens |
| 10095 | actin related protein 2/3 complex, subunit 1B, 41kDa; similar to Actin-related protein 2/3 complex subunit 1B (ARP2/3 complex 41 kDa subunit) (p41-ARC) | Homo sapiens |
| 10476 | ATP synthase, H+ transporting, mitochondrial F0 complex, subunit d | Homo sapiens |
| 3768 | similar to hkir2.2x; similar to inward rectifying K+ channel negative regulator Kir2.2v; potassium inwardly-rectifying channel, subfamily J, member 12 | Homo sapiens |
| 54675 | cardiolipin synthase 1 | Homo sapiens |
| 6128 | ribosomal protein L6 pseudogene 27; ribosomal protein L6 pseudogene 19; ribosomal protein L6; ribosomal protein L6 pseudogene 10 | Homo sapiens |
| 127602 | dynein, axonemal, heavy chain 14 | Homo sapiens |
| 23399 | dullard homolog (Xenopus laevis) | Homo sapiens |
| 647087 | PL-5283 protein | Homo sapiens |
| 1845 | dual specificity phosphatase 3 | Homo sapiens |
| 9684 | leucine rich repeat containing 14 | Homo sapiens |
| 104 | adenosine deaminase, RNA-specific, B1 (RED1 homolog rat) | Homo sapiens |
| 6477 | seven in absentia homolog 1 (Drosophila) | Homo sapiens |
| 10943 | male-specific lethal 3 homolog (Drosophila) | Homo sapiens |
| 10299 | membrane-associated ring finger (C3HC4) 6 | Homo sapiens |
| 64965 | mitochondrial ribosomal protein S9 | Homo sapiens |
| 54623 | Paf1, RNA polymerase II associated factor, homolog (S. cerevisiae) | Homo sapiens |
| 25845 | hypothetical LOC25845 | Homo sapiens |
| 645513 | hypothetical LOC645513 | Homo sapiens |
| 3608 | interleukin enhancer binding factor 2, 45kDa | Homo sapiens |
| 55028 | chromosome 17 open reading frame 80 | Homo sapiens |
| 3241 | hippocalcin-like 1 | Homo sapiens |
| 5058 | p21 protein (Cdc42/Rac)-activated kinase 1 | Homo sapiens |
| 29964 | prickle homolog 4 (Drosophila) | Homo sapiens |
| 51117 | coenzyme Q4 homolog (S. cerevisiae) | Homo sapiens |
| 284018 | chromosome 17 open reading frame 58 | Homo sapiens |
| 11140 | cell division cycle 37 homolog (S. cerevisiae) | Homo sapiens |

| | | |
|---|---|---|
| 153443 | serum response factor binding protein 1 | Homo sapiens |
| 25943 | chromosome 20 open reading frame 194 | Homo sapiens |
| 5380 | postmeiotic segregation increased 2-like 5; postmeiotic segregation increased 2-like 5-like; similar to postmeiotic segregation increased 2-like 2; postmeiotic segregation increased 2-like 2 pseudogene | Homo sapiens |
| 5383 | postmeiotic segregation increased 2-like 5; postmeiotic segregation increased 2-like 5-like; similar to postmeiotic segregation increased 2-like 2; postmeiotic segregation increased 2-like 2 pseudogene | Homo sapiens |
| 339745 | speckle-type POZ protein-like | Homo sapiens |
| 2036 | erythrocyte membrane protein band 4.1-like 1 | Homo sapiens |
| 7776 | zinc finger protein 236 | Homo sapiens |
| 23369 | pumilio homolog 2 (Drosophila) | Homo sapiens |
| 93587 | RNA (guanine-9-) methyltransferase domain containing 2 | Homo sapiens |
| 1859 | dual-specificity tyrosine-(Y)-phosphorylation regulated kinase 1A | Homo sapiens |
| 4047 | lanosterol synthase (2,3-oxidosqualene-lanosterol cyclase) | Homo sapiens |
| 55634 | zinc finger family member 673 | Homo sapiens |
| 3421 | isocitrate dehydrogenase 3 (NAD+) gamma | Homo sapiens |
| 7388 | ubiquinol-cytochrome c reductase hinge protein-like; ubiquinol-cytochrome c reductase hinge protein | Homo sapiens |
| 2673 | glutamine-fructose-6-phosphate transaminase 1 | Homo sapiens |
| 5814 | purine-rich element binding protein B | Homo sapiens |
| 51098 | intraflagellar transport 52 homolog (Chlamydomonas) | Homo sapiens |
| 1655 | DEAD (Asp-Glu-Ala-Asp) box polypeptide 5 | Homo sapiens |
| 113612 | cytochrome P450, family 2, subfamily U, polypeptide 1 | Homo sapiens |
| 22839 | discs, large (Drosophila) homolog-associated protein 4 | Homo sapiens |
| 678 | zinc finger protein 36, C3H type-like 2 | Homo sapiens |
| 6181 | ribosomal protein, large, P2 pseudogene 3; ribosomal protein, large, P2 | Homo sapiens |
| 1495 | catenin (cadherin-associated protein), alpha 1, 102kDa | Homo sapiens |
| 8663 | eukaryotic translation initiation factor 3, subunit C | Homo sapiens |
| 1690 | coagulation factor C homolog, cochlin (Limulus polyphemus) | Homo sapiens |
| 4245 | mannosyl (alpha-1,3-)-glycoprotein beta-1,2-N-acetylglucosaminyltransferase | Homo sapiens |
| 55341 | large subunit GTPase 1 homolog (S. cerevisiae) | Homo sapiens |
| 10781 | zinc finger protein 266 | Homo sapiens |
| 8899 | similar to hCG1820375; PRP4 pre-mRNA processing factor 4 homolog B (yeast) | Homo sapiens |
| 51102 | mitochondrial trans-2-enoyl-CoA reductase | Homo sapiens |
| 284695 | zinc finger protein 326 | Homo sapiens |
| 80031 | sema domain, transmembrane domain (TM), and cytoplasmic domain, (semaphorin) 6D | Homo sapiens |
| 79612 | NMDA receptor regulated 1-like | Homo sapiens |
| 2643 | GTP cyclohydrolase 1 | Homo sapiens |
| 5090 | pre-B-cell leukemia homeobox 3 | Homo sapiens |
| 8317 | cell division cycle 7 homolog (S. cerevisiae) | Homo sapiens |
| 79805 | vasohibin 2 | Homo sapiens |
| 134430 | WD repeat domain 36 | Homo sapiens |
| 292 | solute carrier family 25 (mitochondrial carrier; adenine nucleotide translocator), member 5; solute carrier family 25 (mitochondrial carrier; adenine nucleotide translocator), member 5 pseudogene 8 | Homo sapiens |
| 100124700 | hox transcript antisense RNA (non-protein coding) | Homo sapiens |
| 125488 | tetratricopeptide repeat domain 39C | Homo sapiens |
| 56674 | TMEM9 domain family, member B | Homo sapiens |
| 92667 | chromosome 20 open reading frame 72 | Homo sapiens |
| 1525 | coxsackie virus and adenovirus receptor pseudogene 2; coxsackie virus and adenovirus receptor | Homo sapiens |
| 1104 | regulator of chromosome condensation 1; SNHG3-RCC1 readthrough transcript | Homo sapiens |
| 6596 | helicase-like transcription factor | Homo sapiens |
| 56910 | StAR-related lipid transfer (START) domain containing 7 | Homo sapiens |
| 8835 | suppressor of cytokine signaling 2 | Homo sapiens |
| 1856 | dishevelled, dsh homolog 2 (Drosophila) | Homo sapiens |
| 5859 | glutaminyl-tRNA synthetase | Homo sapiens |
| 6240 | ribonucleotide reductase M1 | Homo sapiens |
| 55144 | leucine rich repeat containing 8 family, member D | Homo sapiens |
| 4647 | myosin VIIA | Homo sapiens |
| 9419 | cysteine-rich PDZ-binding protein | Homo sapiens |
| 374977 | chromosome 1 open reading frame 175 | Homo sapiens |
| 11331 | prohibitin 2 | Homo sapiens |
| 84820 | polymerase (RNA) II (DNA directed) polypeptide J4, pseudogene | Homo sapiens |
| 5099 | protocadherin 7 | Homo sapiens |
| 29127 | Rac GTPase activating protein 1 pseudogene; Rac GTPase activating protein 1 | Homo sapiens |
| 10874 | neuromedin U | Homo sapiens |
| 23462 | hairy/enhancer-of-split related with YRPW motif 1 | Homo sapiens |
| 54891 | INO80 complex subunit D | Homo sapiens |
| 23077 | MYC binding protein 2 | Homo sapiens |
| 55556 | enolase superfamily member 1 | Homo sapiens |
| 10469 | translocase of inner mitochondrial membrane 44 homolog (yeast) | Homo sapiens |
| 2806 | glutamic-oxaloacetic transaminase 2, mitochondrial (aspartate aminotransferase 2) | Homo sapiens |
| 116254 | chromosome 6 open reading frame 72 | Homo sapiens |
| 10131 | TNF receptor-associated protein 1 | Homo sapiens |
| 9683 | NEDD4 binding protein 1 | Homo sapiens |

| | | |
|---|---|---|
| 4303 | forkhead box O4 | Homo sapiens |
| 3416 | insulin-degrading enzyme | Homo sapiens |
| 8649 | MAPK scaffold protein 1 | Homo sapiens |
| 26508 | hairy/enhancer-of-split related with YRPW motif-like | Homo sapiens |
| 51160 | vacuolar protein sorting 28 homolog (S. cerevisiae) | Homo sapiens |
| 5150 | phosphodiesterase 7A | Homo sapiens |
| 23178 | PAS domain containing serine/threonine kinase | Homo sapiens |
| 8559 | PRP18 pre-mRNA processing factor 18 homolog (S. cerevisiae) | Homo sapiens |
| 55179 | Fas apoptotic inhibitory molecule | Homo sapiens |
| 54532 | ubiquitin specific peptidase 53 | Homo sapiens |
| 84275 | solute carrier family 25, member 33 | Homo sapiens |
| 353322 | ankyrin repeat domain 37 | Homo sapiens |
| 11092 | chromosome 9 open reading frame 9 | Homo sapiens |
| 3615 | IMP (inosine monophosphate) dehydrogenase 2 | Homo sapiens |
| 25948 | kelch repeat and BTB (POZ) domain containing 2 | Homo sapiens |
| 124454 | glutamyl-tRNA synthetase 2, mitochondrial (putative) | Homo sapiens |
| 10123 | ADP-ribosylation factor-like 4C | Homo sapiens |
| 10885 | WD repeat domain 3 | Homo sapiens |
| 839 | caspase 6, apoptosis-related cysteine peptidase | Homo sapiens |
| 10099 | tetraspanin 3 | Homo sapiens |
| 64801 | ARV1 homolog (S. cerevisiae) | Homo sapiens |
| 3303 | heat shock 70kDa protein 1A; heat shock 70kDa protein 1B | Homo sapiens |
| 3304 | heat shock 70kDa protein 1A; heat shock 70kDa protein 1B | Homo sapiens |
| 2120 | ets variant 6 | Homo sapiens |
| 25983 | neuroguidin, EIF4E binding protein | Homo sapiens |
| 9516 | lipopolysaccharide-induced TNF factor | Homo sapiens |
| 55831 | transmembrane protein 111 | Homo sapiens |
| 7049 | transforming growth factor, beta receptor III | Homo sapiens |
| 80208 | spastic paraplegia 11 (autosomal recessive) | Homo sapiens |
| 9367 | RAB9A, member RAS oncogene family | Homo sapiens |
| 4190 | malate dehydrogenase 1, NAD (soluble) | Homo sapiens |
| 283357 | hypothetical protein LOC283357 | Homo sapiens |
| 51776 | sterile alpha motif and leucine zipper containing kinase AZK | Homo sapiens |
| 64969 | mitochondrial ribosomal protein S5 | Homo sapiens |
| 961 | CD47 molecule | Homo sapiens |
| 57544 | thioredoxin domain containing 16 | Homo sapiens |
| 9874 | tousled-like kinase 1 | Homo sapiens |
| 51741 | WW domain containing oxidoreductase | Homo sapiens |
| 23325 | KIAA1033 | Homo sapiens |
| 8887 | Tax1 (human T-cell leukemia virus type I) binding protein 1 | Homo sapiens |
| 8629 | jerky homolog (mouse) | Homo sapiens |
| 8991 | selenium binding protein 1 | Homo sapiens |
| 153339 | similar to transmembrane protein 167A; transmembrane protein 167A | Homo sapiens |
| 23625 | family with sequence similarity 89, member B | Homo sapiens |
| 2004 | ELK3, ETS-domain protein (SRF accessory protein 2) | Homo sapiens |
| 51765 | serine/threonine protein kinase MST4 | Homo sapiens |
| 79670 | zinc finger, CCHC domain containing 6 | Homo sapiens |
| 117584 | ring finger and FYVE-like domain containing 1 | Homo sapiens |
| 132864 | cytoplasmic polyadenylation element binding protein 2 | Homo sapiens |
| 9551 | ATP synthase, H+ transporting, mitochondrial F0 complex, subunit F2 | Homo sapiens |
| 7078 | TIMP metallopeptidase inhibitor 3 | Homo sapiens |
| 23412 | COMM domain containing 3 | Homo sapiens |
| 60492 | coiled-coil domain containing 90B | Homo sapiens |
| 131616 | transmembrane protein 42 | Homo sapiens |
| 3682 | integrin, alpha E (antigen CD103, human mucosal lymphocyte antigen 1; alpha polypeptide) | Homo sapiens |
| 5534 | protein phosphatase 3 (formerly 2B), regulatory subunit B, alpha isoform | Homo sapiens |
| 4040 | low density lipoprotein receptor-related protein 6 | Homo sapiens |
| 6050 | ribonuclease/angiogenin inhibitor 1 | Homo sapiens |
| 57728 | WD repeat domain 19 | Homo sapiens |
| 51246 | shisa homolog 5 (Xenopus laevis) | Homo sapiens |
| 161742 | sprouty-related, EVH1 domain containing 1 | Homo sapiens |
| 113452 | transmembrane protein 54 | Homo sapiens |
| 22884 | WD repeat domain 37 | Homo sapiens |
| 376267 | RAB15, member RAS onocogene family | Homo sapiens |
| 55041 | pleckstrin homology domain containing, family B (evectins) member 2 | Homo sapiens |
| 114928 | G protein-coupled receptor associated sorting protein 2 | Homo sapiens |
| 4814 | ninjurin 1 | Homo sapiens |
| 30 | acetyl-Coenzyme A acyltransferase 1 | Homo sapiens |
| 100113407 | transmembrane protein 170B | Homo sapiens |
| 25824 | peroxiredoxin 5 | Homo sapiens |
| 331 | X-linked inhibitor of apoptosis | Homo sapiens |
| 55117 | solute carrier family 6 (neutral amino acid transporter), member 15 | Homo sapiens |
| 7264 | tissue specific transplantation antigen P35B | Homo sapiens |
| 84851 | tripartite motif-containing 52 | Homo sapiens |
| 10328 | COX4 neighbor | Homo sapiens |
| 26292 | c-myc binding protein | Homo sapiens |
| 7375 | ubiquitin specific peptidase 4 (proto-oncogene) | Homo sapiens |

| | | |
|---|---|---|
| 1429 | crystallin, zeta (quinone reductase) | Homo sapiens |
| 85019 | chromosome 18 open reading frame 45 | Homo sapiens |
| 2876 | glutathione peroxidase 1 | Homo sapiens |
| 51175 | tubulin, epsilon 1 | Homo sapiens |
| 23201 | family with sequence similarity 168, member A | Homo sapiens |
| 114880 | oxysterol binding protein-like 6 | Homo sapiens |
| 10160 | FERM, RhoGEF (ARHGEF) and pleckstrin domain protein 1 (chondrocyte-derived) | Homo sapiens |
| 10352 | tryptophanyl tRNA synthetase 2, mitochondrial | Homo sapiens |
| 6309 | sterol-C5-desaturase (ERG3 delta-5-desaturase homolog, S. cerevisiae)-like | Homo sapiens |
| 56339 | methyltransferase like 3 | Homo sapiens |
| 56652 | chromosome 10 open reading frame 2 | Homo sapiens |
| 11014 | KDEL (Lys-Asp-Glu-Leu) endoplasmic reticulum protein retention receptor 2 | Homo sapiens |
| 7389 | uroporphyrinogen decarboxylase | Homo sapiens |
| 23175 | lipin 1 | Homo sapiens |
| 5048 | platelet-activating factor acetylhydrolase, isoform Ib, subunit 1 (45kDa) | Homo sapiens |
| 5878 | RAB5C, member RAS oncogene family | Homo sapiens |
| 1603 | defender against cell death 1 | Homo sapiens |
| 9942 | xylulokinase homolog (H. influenzae) | Homo sapiens |
| 51009 | Der1-like domain family, member 2 | Homo sapiens |
| 51373 | mitochondrial ribosomal protein S17 | Homo sapiens |
| 10743 | retinoic acid induced 1 | Homo sapiens |
| 10712 | chromosome 1 open reading frame 2 | Homo sapiens |
| 11252 | protein kinase C and casein kinase substrate in neurons 2 | Homo sapiens |
| 1787 | tRNA aspartic acid methyltransferase 1 | Homo sapiens |
| 317649 | eukaryotic translation initiation factor 4E family member 3 | Homo sapiens |
| 284325 | chromosome 19 open reading frame 54 | Homo sapiens |
| 6415 | selenoprotein W, 1 | Homo sapiens |
| 100131199 | hypothetical LOC100131199 | Homo sapiens |
