# Supplementary material for: Identification and Characterization of 293T Cell-Derived Exosomes by Profiling the Protein, mRNA and MicroRNA Components
Source: PLoS One. 2016 Sep 20;11(9):e0163043. doi: 10.1371/journal.pone.0163043 (PMC5029934; doi:10.1371/journal.pone.0163043)
Supplement: S5 Table — (PDF) [file pone.0163043.s006.pdf]

	Relative expression level		
miRBase20_name	1	2	3
hsa-miR-3960	13.96115	13.05392	13.97293
hsa-miR-1469	13.43818	13.22595	13.54249
hsa-miR-4497	13.29071	13.35728	13.48892
hsa-miR-3665	13.64416	12.71177	13.63597
hsa-miR-4787-5p	13.56284	12.52972	13.50022
hsa-miR-663a	12.73929	12.69926	12.83888
hsa-miR-1915-3p	12.87917	11.96779	12.80827
hsa-miR-4466	12.83579	11.9691	12.83579
hsa-miR-638	12.73572	12.02388	12.67049
hsa-miR-3196	12.62125	11.56404	12.63003
hsa-miR-4443	12.00162	12.82416	11.95237
hsa-miR-4508	12.59828	11.41813	12.67851
hsa-miR-4530	12.58204	10.95802	12.47933
hsa-miR-1908-5p	12.04599	11.75771	12.20872
hsa-miR-4488	12.2456	11.36164	12.28999
hsa-miR-2861	12.3685	11.1475	12.31352
hsa-miR-762	11.84846	11.36505	11.7676
hsa-miR-4454	11.4229	12.184	11.20871
hsa-miR-4516	12.11074	10.53879	12.07627
hsa-miR-149-3p	11.64538	11.12281	11.64366
hsa-miR-3656	11.82006	10.60267	11.88987
hsa-miR-4459	11.0909	10.80169	11.41867
hsa-miR-4687-3p	11.43802	10.30259	11.28878
hsa-miR-4745-5p	10.61089	10.56661	10.83366
hsa-miR-3138	10.53921	11.16815	10.09984
hsa-miR-1228-5p	10.7918	10.05949	10.9013
hsa-miR-3940-5p	10.77789	9.59353	10.91825
hsa-miR-17-5p	10.40851	10.66906	10.06982
hsa-miR-106a-5p	10.25098	10.56575	9.997275
hsa-miR-4463	10.36979	10.00329	10.36979
hsa-miR-4739	10.32582	9.887145	10.43773
hsa-miR-4763-3p	10.60375	9.372374	10.63761
hsa-miR-4492	10.70079	8.819457	10.85812
hsa-miR-1275	10.06093	10.33434	9.71038
hsa-miR-221-3p	9.909447	9.981245	10.17293
hsa-miR-4707-5p	10.1075	9.483634	10.4425
hsa-miR-20a-5p	9.871557	10.25596	9.607064
hsa-miR-4532	10.27063	8.996542	10.11195
hsa-miR-4467	10.25619	8.610929	10.47193
hsa-miR-4281	10.04021	9.023405	10.23188
hsa-miR-103a-3p	10.00755	9.924988	9.014766
hsa-miR-222-3p	9.56613	9.536891	9.81406
hsa-miR-1246	9.061166	9.827256	9.87962
hsa-miR-4505	10.27237	8.356071	10.13457
hsa-miR-4651	9.817745	8.691956	10.24528
hsa-miR-4734	9.851258	8.54664	10.05148
hsa-miR-92a-3p	9.469182	9.619136	9.320517
hsa-miR-4484	9.180144	9.235891	9.883215
hsa-miR-4507	10.03173	7.974578	10.07106
hsa-miR-4689	9.076094	9.18233	9.27187
hsa-miR-4270	9.193708	8.71347	9.598004
hsa-miR-3178	9.052498	8.881952	9.455199

hsa-miR-4695-5p	8.87345	9.007684	9.50814
hsa-miR-320a	9.478515	8.896557	9.012273
hsa-miR-93-5p	9.183878	9.066558	8.976719
hsa-miR-320b	9.307963	8.832175	8.963496
hsa-miR-25-5p	8.678685	9.503702	8.881952
hsa-miR-191-5p	8.930536	9.027046	9.062786
hsa-miR-3135b	8.474033	8.948259	9.567527
hsa-miR-107	9.162829	9.349047	8.403541
hsa-miR-92b-5p	8.798225	8.842107	9.247392
hsa-miR-4674	8.924302	8.283373	9.362763
hsa-miR-320c	9.050323	8.665651	8.689742
hsa-miR-16-5p	8.687433	8.992015	8.597623
hsa-miR-3185	9.102942	7.543605	9.430398
hsa-miR-4741	8.714156	7.965284	9.368228
hsa-miR-1207-5p	8.596853	8.552185	8.831407
hsa-miR-1268a	8.459597	8.290908	8.872475
hsa-miR-18a-5p	8.780321	8.789124	8.028869
hsa-miR-494-3p	8.969169	7.608939	8.855294
hsa-miR-4665-5p	8.1557	8.589789	8.577621
hsa-miR-3621	8.411707	7.704153	8.665652
hsa-miR-4749-5p	8.353823	7.877018	8.434521
hsa-miR-1909-3p	8.067466	8.279848	8.155849
hsa-miR-1268b	8.229382	7.958693	8.26941
hsa-miR-3187-3p	7.790599	8.499743	8.112565
hsa-miR-4433a-3p	8.270794	7.847835	8.283432
hsa-miR-361-5p	8.320006	8.109115	7.883978
hsa-miR-4298	8.145964	8.096842	7.976455
hsa-miR-4758-5p	8.312499	7.617709	8.215496
hsa-miR-4668-5p	8.029972	8.413519	7.70105
hsa-miR-4634	7.877585	8.018074	8.241407
hsa-miR-3141	8.174576	7.757066	8.128119
