# Supplementary material for: Identification and Characterization of 293T Cell-Derived Exosomes by Profiling the Protein, mRNA and MicroRNA Components
Source: PLoS One. 2016 Sep 20;11(9):e0163043. doi: 10.1371/journal.pone.0163043 (PMC5029934; doi:10.1371/journal.pone.0163043)
Supplement: S6 Table — (PDF) [file pone.0163043.s007.pdf]

ENTREZ_GENE_ID	Name	Species
63897	HEAT repeat containing 6	Homo sapiens
26057	ankyrin repeat domain 17	Homo sapiens
29914	UbiA prenyltransferase domain containing 1	Homo sapiens
55295	kelch-like 26 (Drosophila)	Homo sapiens
6752	somatostatin receptor 2	Homo sapiens
84942	WD repeat domain 73	Homo sapiens
7011	telomerase-associated protein 1	Homo sapiens
51330	tumor necrosis factor receptor superfamily, member 12A	Homo sapiens
79809	tetratricopeptide repeat domain 21B	Homo sapiens
64895	poly(A) polymerase gamma	Homo sapiens
1130	lysosomal trafficking regulator	Homo sapiens
94134	Rho GTPase activating protein 12	Homo sapiens
8863	period homolog 3 (Drosophila)	Homo sapiens
5394	exosome component 10	Homo sapiens
57510	exportin 5	Homo sapiens
55252	additional sex combs like 2 (Drosophila)	Homo sapiens
9778	KIAA0232	Homo sapiens
54776	protein phosphatase 1, regulatory (inhibitor) subunit 12C	Homo sapiens
7584	zinc finger protein 35	Homo sapiens
3750	potassium voltage-gated channel, Shal-related subfamily, member 1	Homo sapiens
54820	nudE nuclear distribution gene E homolog 1 (A. nidulans)	Homo sapiens
259230	sphingomyelin synthase 1	Homo sapiens
4071	transmembrane 4 L six family member 1	Homo sapiens
133	adrenomedullin	Homo sapiens
128866	chromatin modifying protein 4B	Homo sapiens
2843	G protein-coupled receptor 20	Homo sapiens
64215	DnaJ (Hsp40) homolog, subfamily C, member 1	Homo sapiens
2734	golgi apparatus protein 1	Homo sapiens
10533	ATG7 autophagy related 7 homolog (S. cerevisiae)	Homo sapiens
146862	unc-45 homolog B (C. elegans)	Homo sapiens
5734	prostaglandin E receptor 4 (subtype EP4)	Homo sapiens
27303	RNA binding motif, single stranded interacting protein	Homo sapiens
642346	similar to golgi autoantigen, golgin subfamily a-like; hypothetical protein LOC440295; hypothetical LOC642346; golgin subfamily A member 6-like protein 10-like	Homo sapiens
440295	similar to golgi autoantigen, golgin subfamily a-like; hypothetical protein LOC440295; hypothetical LOC642346; golgin subfamily A member 6-like protein 10-like	Homo sapiens
728636	similar to golgi autoantigen, golgin subfamily a-like; hypothetical protein LOC440295; hypothetical LOC642346; golgin subfamily A member 6-like protein 10-like	Homo sapiens
647042	similar to golgi autoantigen, golgin subfamily a-like; hypothetical protein LOC440295; hypothetical LOC642346; golgin subfamily A member 6-like protein 10-like	Homo sapiens
27136	MORC family CW-type zinc finger 1	Homo sapiens
201799	transmembrane protein 154	Homo sapiens
4176	minichromosome maintenance complex component 7	Homo sapiens
55968	NSFL1 (p97) cofactor (p47)	Homo sapiens
388	ras homolog gene family, member B	Homo sapiens
22801	integrin, alpha 11	Homo sapiens
91608	RAS-like, family 10, member B	Homo sapiens
6091	roundabout, axon guidance receptor, homolog 1 (Drosophila); similar to roundabout 1 isoform b	Homo sapiens
642132	roundabout, axon guidance receptor, homolog 1 (Drosophila); similar to roundabout 1 isoform b	Homo sapiens
11238	inactivation escape 2 (non-protein coding); carbonic anhydrase VB, mitochondrial	Homo sapiens
8551	inactivation escape 2 (non-protein coding); carbonic anhydrase VB, mitochondrial	Homo sapiens
56138	protocadherin alpha 11	Homo sapiens
7542	zinc finger protein-like 1	Homo sapiens
10291	splicing factor 3a, subunit 1, 120kDa	Homo sapiens
26065	LSM14A, SCD6 homolog A (S. cerevisiae)	Homo sapiens
3162	heme oxygenase (decycling) 1	Homo sapiens
154743	chromosome 7 open reading frame 60	Homo sapiens
84818	interleukin 17 receptor C	Homo sapiens
55327	lin-7 homolog C (C. elegans)	Homo sapiens
23658	LSM5 homolog, U6 small nuclear RNA associated (S. cerevisiae)	Homo sapiens
55916	nuclear transport factor 2-like export factor 2	Homo sapiens
7421	vitamin D (1,25- dihydroxyvitamin D3) receptor	Homo sapiens
9493	kinesin family member 23	Homo sapiens
79078	chromosome 1 open reading frame 50	Homo sapiens
27333	golgi integral membrane protein 4	Homo sapiens
317754	POTE ankyrin domain family, member D; POTE ankyrin domain family, member C; POTE ankyrin domain family, member B	Homo sapiens
339010	POTE ankyrin domain family, member D; POTE ankyrin domain family, member C; POTE ankyrin domain family, member B	Homo sapiens
388468	POTE ankyrin domain family, member D; POTE ankyrin domain family, member C; POTE ankyrin domain family, member B	Homo sapiens
133418	embigin homolog (mouse)	Homo sapiens
8050	pyruvate dehydrogenase complex, component X	Homo sapiens
22836	Rho-related BTB domain containing 3	Homo sapiens
5339	similar to Plectin 1 (PLTN) (PCN) (Hemidesmosomal protein 1) (HD1); plectin 1, intermediate filament binding protein 500kDa	Homo sapiens

652460	similar to Plectin 1 (PLTN) (PCN) (Hemidesmosomal protein 1) (HD1); plectin 1, intermediate filament binding protein 500kDa	Homo sapiens
10936	G protein-coupled receptor 75	Homo sapiens
58478	enolase-phosphatase 1	Homo sapiens
51283	bifunctional apoptosis regulator	Homo sapiens
8933	family with sequence similarity 127, member A	Homo sapiens
140831	zinc finger, SWIM-type containing 3	Homo sapiens
8867	synaptojanin 1	Homo sapiens
51031	glyoxalase domain containing 4	Homo sapiens
55810	forkhead box J2	Homo sapiens
10449	hypothetical LOC648603; acetyl-Coenzyme A acyltransferase 2	Homo sapiens
648603	hypothetical LOC648603; acetyl-Coenzyme A acyltransferase 2	Homo sapiens
9480	one cut homeobox 2	Homo sapiens
9230	RAB11B, member RAS oncogene family	Homo sapiens
4489	metallothionein 1A	Homo sapiens
55795	PCI domain containing 2	Homo sapiens
966	CD59 molecule, complement regulatory protein	Homo sapiens
153241	centrosomal protein 120kDa	Homo sapiens
219654	zinc finger, CCHC domain containing 24	Homo sapiens
3488	insulin-like growth factor binding protein 5	Homo sapiens
2538	glucose-6-phosphatase, catalytic subunit	Homo sapiens
23241	phosphofurin acidic cluster sorting protein 2	Homo sapiens
50515	carbohydrate (chondroitin 4) sulfotransferase 11	Homo sapiens
3799	kinesin family member 5B	Homo sapiens
1613	death-associated protein kinase 3	Homo sapiens
9896	FIG4 homolog (S. cerevisiae)	Homo sapiens
23151	TSC22 domain family, member 3; GRAM domain containing 4	Homo sapiens
1831	TSC22 domain family, member 3; GRAM domain containing 4	Homo sapiens
1052	CCAAT/enhancer binding protein (C/EBP), delta	Homo sapiens
2558	gamma-aminobutyric acid (GABA) A receptor, alpha 5	Homo sapiens
118738	zinc finger protein 488	Homo sapiens
26234	F-box and leucine-rich repeat protein 5	Homo sapiens
83546	retbindin	Homo sapiens
53833	interleukin 20 receptor beta	Homo sapiens
155060	zinc finger family member 783	Homo sapiens
114785	methyl-CpG binding domain protein 6	Homo sapiens
64375	IKAROS family zinc finger 4 (Eos)	Homo sapiens
7482	wingless-type MMTV integration site family, member 2B	Homo sapiens
169200	transmembrane protein 64	Homo sapiens
9929	Josephin domain containing 1	Homo sapiens
64151	non-SMC condensin I complex, subunit G	Homo sapiens
10527	importin 7	Homo sapiens
200958	mucin 20, cell surface associated	Homo sapiens
140680	chromosome 20 open reading frame 96	Homo sapiens
200081	taxilin alpha	Homo sapiens
1629	dihydrolipoamide branched chain transacylase E2	Homo sapiens
5888	RAD51 homolog (RecA homolog, E. coli) (S. cerevisiae)	Homo sapiens
79672	fructosamine 3 kinase related protein	Homo sapiens
6867	transforming, acidic coiled-coil containing protein 1	Homo sapiens
441034	ribosomal protein L7a pseudogene 70; ribosomal protein L7a; ribosomal protein L7a pseudogene 30; ribosomal protein L7a pseudogene 66; ribosomal protein L7a pseudogene 27; ribosomal protein L7a pseudogene 11; ribosomal protein L7a pseudogene 62	Homo sapiens
728992	ribosomal protein L7a pseudogene 70; ribosomal protein L7a; ribosomal protein L7a pseudogene 30; ribosomal protein L7a pseudogene 66; ribosomal protein L7a pseudogene 27; ribosomal protein L7a pseudogene 11; ribosomal protein L7a pseudogene 62	Homo sapiens
6130	ribosomal protein L7a pseudogene 70; ribosomal protein L7a; ribosomal protein L7a pseudogene 30; ribosomal protein L7a pseudogene 66; ribosomal protein L7a pseudogene 27; ribosomal protein L7a pseudogene 11; ribosomal protein L7a pseudogene 62	Homo sapiens
388474	ribosomal protein L7a pseudogene 70; ribosomal protein L7a; ribosomal protein L7a pseudogene 30; ribosomal protein L7a pseudogene 66; ribosomal protein L7a pseudogene 27; ribosomal protein L7a pseudogene 11; ribosomal protein L7a pseudogene 62	Homo sapiens
728139	ribosomal protein L7a pseudogene 70; ribosomal protein L7a; ribosomal protein L7a pseudogene 30; ribosomal protein L7a pseudogene 66; ribosomal protein L7a pseudogene 27; ribosomal protein L7a pseudogene 11; ribosomal protein L7a pseudogene 62	Homo sapiens
644029	ribosomal protein L7a pseudogene 70; ribosomal protein L7a; ribosomal protein L7a pseudogene 30; ribosomal protein L7a pseudogene 66; ribosomal protein L7a pseudogene 27; ribosomal protein L7a pseudogene 11; ribosomal protein L7a pseudogene 62	Homo sapiens
152663	ribosomal protein L7a pseudogene 70; ribosomal protein L7a; ribosomal protein L7a pseudogene 30; ribosomal protein L7a pseudogene 66; ribosomal protein L7a pseudogene 27; ribosomal protein L7a pseudogene 11; ribosomal protein L7a pseudogene 62	Homo sapiens
55102	ATG2 autophagy related 2 homolog B (S. cerevisiae)	Homo sapiens
10635	RAD51 associated protein 1	Homo sapiens
339448	chromosome 1 open reading frame 174	Homo sapiens
653319	KIAA0895-like	Homo sapiens
7528	YY1 transcription factor	Homo sapiens
5908	RAP1B, member of RAS oncogene family	Homo sapiens
53834	fibroblast growth factor receptor-like 1	Homo sapiens
153396	transmembrane protein 161B	Homo sapiens
57602	ubiquitin specific peptidase 36	Homo sapiens

7392	upstream transcription factor 2, c-fos interacting	Homo sapiens
728599	cytokine induced apoptosis inhibitor 1; cytokine induced apoptosis inhibitor 1 pseudogene	Homo sapiens
57019	cytokine induced apoptosis inhibitor 1; cytokine induced apoptosis inhibitor 1 pseudogene	Homo sapiens
3068	hepatoma-derived growth factor (high-mobility group protein 1-like)	Homo sapiens
10197	proteasome (prosome, macropain) activator subunit 3 (PA28 gamma; Ki)	Homo sapiens
201475	RAB12, member RAS oncogene family	Homo sapiens
79183	tocopherol (alpha) transfer protein-like	Homo sapiens
23198	proteasome (prosome, macropain) activator subunit 4	Homo sapiens
55208	DCN1, defective in cullin neddylation 1, domain containing 2 (S. cerevisiae)	Homo sapiens
79869	cleavage and polyadenylation specific factor 7, 59kDa	Homo sapiens
5917	arginyl-tRNA synthetase	Homo sapiens
3915	laminin, gamma 1 (formerly LAMB2)	Homo sapiens
29107	NTF2-like export factor 1	Homo sapiens
11054	opioid growth factor receptor	Homo sapiens
56970	ataxin 7-like 3	Homo sapiens
140	adenosine A3 receptor	Homo sapiens
641649	transmembrane protein 91	Homo sapiens
4899	nuclear respiratory factor 1	Homo sapiens
1152	creatine kinase, brain	Homo sapiens
81931	zinc finger protein 93	Homo sapiens
7536	splicing factor 1	Homo sapiens
87178	polyribonucleotide nucleotidyltransferase 1	Homo sapiens
114609	toll-interleukin 1 receptor (TIR) domain containing adaptor protein	Homo sapiens
7486	similar to Werner syndrome protein; Werner syndrome, RecQ helicase-like	Homo sapiens
652522	similar to Werner syndrome protein; Werner syndrome, RecQ helicase-like	Homo sapiens
84876	ORAI calcium release-activated calcium modulator 1	Homo sapiens
56172	ankylosis, progressive homolog (mouse)	Homo sapiens
57470	leucine rich repeat containing 47	Homo sapiens
55005	required for meiotic nuclear division 1 homolog (S. cerevisiae)	Homo sapiens
11213	interleukin-1 receptor-associated kinase 3	Homo sapiens
10867	tetraspanin 9	Homo sapiens
3743	potassium voltage-gated channel, shaker-related subfamily, member 7	Homo sapiens
90639	COX19 cytochrome c oxidase assembly homolog (S. cerevisiae)	Homo sapiens
55105	G patch domain containing 2	Homo sapiens
171586	abhydrolase domain containing 3	Homo sapiens
8641	protocadherin gamma subfamily B, 4	Homo sapiens
372	archain 1	Homo sapiens
84964	alkB, alkylation repair homolog 6 (E. coli)	Homo sapiens
651746	ankyrin repeat domain 33B	Homo sapiens
4973	oxidized low density lipoprotein (lectin-like) receptor 1	Homo sapiens
29883	CCR4-NOT transcription complex, subunit 7	Homo sapiens
7702	zinc finger protein 143	Homo sapiens
25996	REX2, RNA exonuclease 2 homolog (S. cerevisiae)	Homo sapiens
56924	p21 protein (Cdc42/Rac)-activated kinase 6	Homo sapiens
23210	jumonji domain containing 6	Homo sapiens
57563	kelch-like 8 (Drosophila)	Homo sapiens
810	calmodulin-like 3	Homo sapiens
57181	solute carrier family 39 (zinc transporter), member 10	Homo sapiens
51239	ankyrin repeat domain 39	Homo sapiens
79944	L-2-hydroxyglutarate dehydrogenase	Homo sapiens
831	calpastatin	Homo sapiens
28962	osteopetrosis associated transmembrane protein 1	Homo sapiens
26073	polymerase (DNA-directed), delta interacting protein 2	Homo sapiens
200316	apolipoprotein B mRNA editing enzyme, catalytic polypeptide-like 3F	Homo sapiens
129401	nucleoporin 35kDa	Homo sapiens
6650	small optic lobes homolog (Drosophila)	Homo sapiens
10693	chaperonin containing TCP1, subunit 6B (zeta 2)	Homo sapiens
653441	polyhomeotic homolog 1B (Drosophila); polyhomeotic homolog 1 (Drosophila)	Homo sapiens
1911	polyhomeotic homolog 1B (Drosophila); polyhomeotic homolog 1 (Drosophila)	Homo sapiens
100133042	glyceraldehyde-3-phosphate dehydrogenase-like 6; hypothetical protein LOC100133042; glyceraldehyde-3-phosphate dehydrogenase	Homo sapiens
729403	glyceraldehyde-3-phosphate dehydrogenase-like 6; hypothetical protein LOC100133042; glyceraldehyde-3-phosphate dehydrogenase	Homo sapiens
2597	glyceraldehyde-3-phosphate dehydrogenase-like 6; hypothetical protein LOC100133042; glyceraldehyde-3-phosphate dehydrogenase	Homo sapiens
65056	GC-rich promoter binding protein 1	Homo sapiens
170959	zinc finger protein 431	Homo sapiens
343702	XK, Kell blood group complex subunit-related family, member 7	Homo sapiens
54881	testis expressed 10	Homo sapiens
26050	SLIT and NTRK-like family, member 5	Homo sapiens
1233	chemokine (C-C motif) receptor 4	Homo sapiens
55898	unc-45 homolog A (C. elegans)	Homo sapiens
6157	ribosomal protein L27a	Homo sapiens
8760	CDP-diacylglycerol synthase (phosphatidate cytidylyltransferase) 2	Homo sapiens
3658	iron-responsive element binding protein 2	Homo sapiens
94234	forkhead box Q1	Homo sapiens
2356	folylpolyglutamate synthase	Homo sapiens

9441	mediator complex subunit 26	Homo sapiens
79842	zinc finger and BTB domain containing 3	Homo sapiens
9371	kinesin family member 3B	Homo sapiens
92312	mex-3 homolog A (C. elegans)	Homo sapiens
22838	ring finger protein 44	Homo sapiens
8802	succinate-CoA ligase, alpha subunit	Homo sapiens
10274	stromal antigen 1	Homo sapiens
137492	vacuolar protein sorting 37 homolog A (S. cerevisiae)	Homo sapiens
4149	MYC associated factor X	Homo sapiens
1186	chloride channel 7	Homo sapiens
2020	engrailed homeobox 2	Homo sapiens
9467	SH3-domain binding protein 5 (BTK-associated)	Homo sapiens
22832	KIAA1009	Homo sapiens
374986	family with sequence similarity 73, member A	Homo sapiens
3575	interleukin 7 receptor	Homo sapiens
6288	serum amyloid A1	Homo sapiens
142678	mindbomb homolog 2 (Drosophila)	Homo sapiens
56125	protocadherin beta 11	Homo sapiens
10016	aryl-hydrocarbon receptor repressor; programmed cell death 6	Homo sapiens
57491	aryl-hydrocarbon receptor repressor; programmed cell death 6	Homo sapiens
8313	axin 2	Homo sapiens
646050	hypothetical LOC653631; hypothetical LOC646050; hypothetical LOC646890; axin interactor, dorsalization associated	Homo sapiens
64853	hypothetical LOC653631; hypothetical LOC646050; hypothetical LOC646890; axin interactor, dorsalization associated	Homo sapiens
653631	hypothetical LOC653631; hypothetical LOC646050; hypothetical LOC646890; axin interactor, dorsalization associated	Homo sapiens
646890	hypothetical LOC653631; hypothetical LOC646050; hypothetical LOC646890; axin interactor, dorsalization associated	Homo sapiens
348995	nucleoporin 43kDa	Homo sapiens
54509	ras homolog gene family, member F (in filopodia)	Homo sapiens
192683	secretory carrier membrane protein 5	Homo sapiens
26960	neurobeachin	Homo sapiens
219285	sterile alpha motif domain containing 9-like	Homo sapiens
23378	ribosomal RNA processing 8, methyltransferase, homolog (yeast)	Homo sapiens
81563	chromosome 1 open reading frame 21	Homo sapiens
200916	ribosomal protein L22-like 1	Homo sapiens
7923	hydroxysteroid (17-beta) dehydrogenase 8	Homo sapiens
6455	SH3-domain GRB2-like 1	Homo sapiens
550	ancient ubiquitous protein 1	Homo sapiens
3018	histone cluster 1, H2bb	Homo sapiens
51466	Enah/Vasp-like	Homo sapiens
9819	TSC22 domain family, member 2	Homo sapiens
5903	RAN binding protein 2	Homo sapiens
23560	GTP binding protein 4	Homo sapiens
3043	hemoglobin, beta	Homo sapiens
23302	WSC domain containing 1	Homo sapiens
54976	chromosome 20 open reading frame 27	Homo sapiens
102	ADAM metallopeptidase domain 10	Homo sapiens
84263	hydroxysteroid dehydrogenase like 2	Homo sapiens
7343	upstream binding transcription factor, RNA polymerase I	Homo sapiens
9181	Rho/Rac guanine nucleotide exchange factor (GEF) 2	Homo sapiens
54431	DnaJ (Hsp40) homolog, subfamily C, member 10	Homo sapiens
5223	phosphoglycerate mutase 1 (brain)	Homo sapiens
81618	integral membrane protein 2C	Homo sapiens
10494	serine/threonine kinase 25 (STE20 homolog, yeast)	Homo sapiens
440434	hypothetical protein FLJ11822; aminopeptidase puromycin sensitive	Homo sapiens
9520	hypothetical protein FLJ11822; aminopeptidase puromycin sensitive	Homo sapiens
145195	chromosome 14 open reading frame 144	Homo sapiens
57688	zinc finger, SWIM-type containing 6	Homo sapiens
80264	zinc finger protein 430	Homo sapiens
9184	budding uninhibited by benzimidazoles 3 homolog (yeast)	Homo sapiens
51805	coenzyme Q3 homolog, methyltransferase (S. cerevisiae)	Homo sapiens
7415	valosin-containing protein	Homo sapiens
8729	golgi-specific brefeldin A resistant guanine nucleotide exchange factor 1	Homo sapiens
100133398	selenoprotein T; similar to Selenoprotein T	Homo sapiens
51714	selenoprotein T; similar to Selenoprotein T	Homo sapiens
1540	cylindromatosis (turban tumor syndrome)	Homo sapiens
100134938	uroplakin-like protein	Homo sapiens
10922	Fas-activated serine/threonine kinase	Homo sapiens
139411	patched domain containing 1	Homo sapiens
55173	mitochondrial ribosomal protein S10	Homo sapiens
30851	Tax1 (human T-cell leukemia virus type I) binding protein 3	Homo sapiens
63977	PR domain containing 15	Homo sapiens
126374	Wilms tumor 1 interacting protein	Homo sapiens
54205	cytochrome c, somatic	Homo sapiens
56925	latexin	Homo sapiens
10564	ADP-ribosylation factor guanine nucleotide-exchange factor 2 (brefeldin A-inhibited)	Homo sapiens
57610	RAN binding protein 10	Homo sapiens

57622	leucine rich repeat and fibronectin type III domain containing 1	Homo sapiens
84967	LSM10, U7 small nuclear RNA associated	Homo sapiens
134288	transmembrane protein 174	Homo sapiens
9785	DEAH (Asp-Glu-Ala-His) box polypeptide 38	Homo sapiens
5527	protein phosphatase 2, regulatory subunit B', gamma isoform	Homo sapiens
93100	nicotinate phosphoribosyltransferase domain containing 1	Homo sapiens
58485	trafficking protein particle complex 1	Homo sapiens
167410	Lix1 homolog (chicken)	Homo sapiens
4913	nth endonuclease III-like 1 (E. coli)	Homo sapiens
79680	chromosome 22 open reading frame 29	Homo sapiens
10621	polymerase (RNA) III (DNA directed) polypeptide F, 39 kDa	Homo sapiens
645978	similar to alkaline ceramidase 2; alkaline ceramidase 2	Homo sapiens
340485	similar to alkaline ceramidase 2; alkaline ceramidase 2	Homo sapiens
57649	PHD finger protein 12	Homo sapiens
11315	Parkinson disease (autosomal recessive, early onset) 7	Homo sapiens
7332	ubiquitin-conjugating enzyme E2L 3	Homo sapiens
100270710	hypothetical LOC100270710; MARVEL domain containing 1	Homo sapiens
83742	hypothetical LOC100270710; MARVEL domain containing 1	Homo sapiens
9628	regulator of G-protein signaling 6	Homo sapiens
7757	zinc finger protein 208	Homo sapiens
4696	NADH dehydrogenase (ubiquinone) 1 alpha subcomplex, 3, 9kDa	Homo sapiens
5914	retinoic acid receptor, alpha	Homo sapiens
374879	zinc finger protein 699	Homo sapiens
5563	protein kinase, AMP-activated, alpha 2 catalytic subunit	Homo sapiens
5127	PCTAIRE protein kinase 1	Homo sapiens
283209	phosphoglucomutase 2-like 1	Homo sapiens
9703	KIAA0100	Homo sapiens
1979	eukaryotic translation initiation factor 4E binding protein 2	Homo sapiens
10730	YME1-like 1 (S. cerevisiae)	Homo sapiens
1995	ELAV (embryonic lethal, abnormal vision, Drosophila)-like 3 (Hu antigen C)	Homo sapiens
23498	3-hydroxyanthranilate 3,4-dioxygenase	Homo sapiens
148423	chromosome 1 open reading frame 52	Homo sapiens
2048	EPH receptor B2	Homo sapiens
219899	tubulin folding cofactor E-like	Homo sapiens
9258	malignant fibrous histiocytoma amplified sequence 1	Homo sapiens
338321	NLR family, pyrin domain containing 9	Homo sapiens
6102	retinitis pigmentosa 2 (X-linked recessive)	Homo sapiens
117177	RAB3A interacting protein (rabin3)	Homo sapiens
3843	importin 5	Homo sapiens
537	ATPase, H+ transporting, lysosomal accessory protein 1	Homo sapiens
84128	WD repeat domain 75	Homo sapiens
56829	zinc finger CCCH-type, antiviral 1	Homo sapiens
7412	vascular cell adhesion molecule 1	Homo sapiens
7525	v-yes-1 Yamaguchi sarcoma viral oncogene homolog 1	Homo sapiens
51386	eukaryotic translation initiation factor 3, subunit L	Homo sapiens
9575	clock homolog (mouse)	Homo sapiens
51301	glucosaminyl (N-acetyl) transferase 4, core 2 (beta-1,6-N-acetylglucosaminyltransferase)	Homo sapiens
79041	transmembrane protein 38A	Homo sapiens
6651	SON DNA binding protein	Homo sapiens
11160	ER lipid raft associated 2	Homo sapiens
7294	TXK tyrosine kinase	Homo sapiens
23235	salt-inducible kinase 2	Homo sapiens
5743	prostaglandin-endoperoxide synthase 2 (prostaglandin G/H synthase and cyclooxygenase)	Homo sapiens
11004	kinesin family member 2C	Homo sapiens
83714	nuclear receptor interacting protein 2	Homo sapiens
7021	transcription factor AP-2 beta (activating enhancer binding protein 2 beta)	Homo sapiens
7086	transketolase	Homo sapiens
9797	TatD DNase domain containing 2	Homo sapiens
283985	fatty acid desaturase domain family, member 6	Homo sapiens
2631	glioblastoma amplified sequence	Homo sapiens
65267	WNK lysine deficient protein kinase 3	Homo sapiens
283130	solute carrier family 25, member 45	Homo sapiens
79441	HAUS augmin-like complex, subunit 3	Homo sapiens
54149	chromosome 21 open reading frame 91	Homo sapiens
51090	plasma membrane proteolipid (plasmolipin)	Homo sapiens
126298	immunity-related GTPase family, Q	Homo sapiens
23287	ATP/GTP binding protein 1	Homo sapiens
293	solute carrier family 25 (mitochondrial carrier; adenine nucleotide translocator), member 6	Homo sapiens
10794	zinc finger protein 460	Homo sapiens
10644	insulin-like growth factor 2 mRNA binding protein 2	Homo sapiens
6664	SRY (sex determining region Y)-box 11	Homo sapiens
79576	NFKB activating protein	Homo sapiens
1209	cleft lip and palate associated transmembrane protein 1	Homo sapiens
51247	poly(A) binding protein interacting protein 2	Homo sapiens
51125	golgi autoantigen, golgin subfamily a, 7	Homo sapiens
6878	TAF6 RNA polymerase II, TATA box binding protein (TBP)-associated factor, 80kDa	Homo sapiens
283514	seven in absentia homolog 3 (Drosophila)	Homo sapiens
716	complement component 1, s subcomponent	Homo sapiens

121457	IKK interacting protein	Homo sapiens
8888	minichromosome maintenance complex component 3 associated protein	Homo sapiens
9215	like-glycosyltransferase	Homo sapiens
10615	sperm associated antigen 5	Homo sapiens
9117	SEC22 vesicle trafficking protein homolog C (S. cerevisiae)	Homo sapiens
560	azoospermia factor 1	Homo sapiens
83544	dynein, axonemal, light chain 1	Homo sapiens
26128	KIAA1279	Homo sapiens
254228	family with sequence similarity 26, member E	Homo sapiens
2975	general transcription factor IIIC, polypeptide 1, alpha 220kDa	Homo sapiens
57590	WD repeat and FYVE domain containing 1	Homo sapiens
8884	solute carrier family 5 (sodium-dependent vitamin transporter), member 6	Homo sapiens
5311	polycystic kidney disease 2 (autosomal dominant)	Homo sapiens
8740	tumor necrosis factor (ligand) superfamily, member 14	Homo sapiens
10769	polo-like kinase 2 (Drosophila)	Homo sapiens
5170	3-phosphoinositide dependent protein kinase-1	Homo sapiens
3383	intercellular adhesion molecule 1	Homo sapiens
2232	ferredoxin reductase	Homo sapiens
112885	PHD finger protein 21B	Homo sapiens
284434	NACHT and WD repeat domain containing 1	Homo sapiens
5464	pyrophosphatase (inorganic) 1	Homo sapiens
92370	acid phosphatase-like 2	Homo sapiens
23348	dedicator of cytokinesis 9	Homo sapiens
51768	transmembrane 7 superfamily member 3	Homo sapiens
6722	serum response factor (c-fos serum response element-binding transcription factor)	Homo sapiens
5110	protein-L-isoaspartate (D-aspartate) O-methyltransferase	Homo sapiens
57619	shroom family member 3	Homo sapiens
90780	pygopus homolog 2 (Drosophila)	Homo sapiens
23588	kelch domain containing 2	Homo sapiens
7107	G protein-coupled receptor 137B	Homo sapiens
80308	FAD1 flavin adenine dinucleotide synthetase homolog (S. cerevisiae)	Homo sapiens
9583	ectonucleoside triphosphate diphosphohydrolase 4	Homo sapiens
55610	coiled-coil domain containing 132	Homo sapiens
11338	U2 small nuclear RNA auxiliary factor 2	Homo sapiens
55278	glutaminyl-tRNA synthase (glutamine-hydrolyzing)-like 1	Homo sapiens
6135	ribosomal protein L11	Homo sapiens
541565	chromosome 8 open reading frame 58	Homo sapiens
2823	glycoprotein M6A	Homo sapiens
10567	Rab acceptor 1 (prenylated)	Homo sapiens
80778	zinc finger protein 34	Homo sapiens
2081	endoplasmic reticulum to nucleus signaling 1	Homo sapiens
55255	WD repeat domain 41	Homo sapiens
9758	FERM and PDZ domain containing 4	Homo sapiens
64784	CREB regulated transcription coactivator 3	Homo sapiens
65983	GRAM domain containing 3	Homo sapiens
6272	sortilin 1	Homo sapiens
23119	hypermethylated in cancer 2	Homo sapiens
64318	nucleolar complex associated 3 homolog (S. cerevisiae)	Homo sapiens
7311	ubiquitin A-52 residue ribosomal protein fusion product 1	Homo sapiens
5270	serpin peptidase inhibitor, clade E (nexin, plasminogen activator inhibitor type 1), member 2	Homo sapiens
8334	histone cluster 1, H2ac	Homo sapiens
51006	solute carrier family 35, member C2	Homo sapiens
6520	solute carrier family 3 (activators of dibasic and neutral amino acid transport), member 2	Homo sapiens
132989	chromosome 4 open reading frame 36	Homo sapiens
9271	piwi-like 1 (Drosophila)	Homo sapiens
7363	UDP glucuronosyltransferase 2 family, polypeptide B4	Homo sapiens
29924	epsin 1	Homo sapiens
311	annexin A11	Homo sapiens
5126	proprotein convertase subtilisin/kexin type 2	Homo sapiens
25927	cannabinoid receptor interacting protein 1	Homo sapiens
64110	melanoma antigen family F, 1	Homo sapiens
7227	trichorhinophalangeal syndrome I	Homo sapiens
9278	zinc finger and BTB domain containing 22	Homo sapiens
8241	RNA binding motif protein 10	Homo sapiens
57082	cancer susceptibility candidate 5	Homo sapiens
23194	F-box and leucine-rich repeat protein 7	Homo sapiens
644815	family with sequence similarity 83, member G	Homo sapiens
55296	TBC1 domain family, member 19	Homo sapiens
1770	dynein, axonemal, heavy chain 9	Homo sapiens
159091	family with sequence similarity 122C	Homo sapiens
57701	KIAA1602	Homo sapiens
8942	kynureninase (L-kynurenine hydrolase)	Homo sapiens
165829	G protein-coupled receptor 156	Homo sapiens
57178	zinc finger, MIZ-type containing 1	Homo sapiens
56650	claudin domain containing 1	Homo sapiens
10399	guanine nucleotide binding protein (G protein), beta polypeptide 2-like 1	Homo sapiens
284339	transmembrane protein 145	Homo sapiens

79853	transmembrane 4 L six family member 20	Homo sapiens
5296	phosphoinositide-3-kinase, regulatory subunit 2 (beta)	Homo sapiens
147339	chromosome 18 open reading frame 25	Homo sapiens
79862	zinc finger protein 669	Homo sapiens
8482	semaphorin 7A, GPI membrane anchor (John Milton Hagen blood group)	Homo sapiens
5717	proteasome (prosome, macropain) 26S subunit, non-ATPase, 11	Homo sapiens
4881	natriuretic peptide receptor A/guanylate cyclase A (atrionatriuretic peptide receptor A)	Homo sapiens
23766	GABA(A) receptors associated protein like 3 (pseudogene); GABA(A) receptor-associated protein like 1	Homo sapiens
23710	GABA(A) receptors associated protein like 3 (pseudogene); GABA(A) receptor-associated protein like 1	Homo sapiens
9391	cytosolic iron-sulfur protein assembly 1 homolog (S. cerevisiae)	Homo sapiens
54815	GATA zinc finger domain containing 2A	Homo sapiens
8993	peptidoglycan recognition protein 1	Homo sapiens
51124	immediate early response 3 interacting protein 1	Homo sapiens
6132	ribosomal protein L8; ribosomal protein L8 pseudogene 2	Homo sapiens
100128766	ribosomal protein L8; ribosomal protein L8 pseudogene 2	Homo sapiens
5782	protein tyrosine phosphatase, non-receptor type 12	Homo sapiens
9370	adiponectin, C1Q and collagen domain containing	Homo sapiens
100130757	chromosome 10 open reading frame 71; similar to hCG1996658	Homo sapiens
118461	chromosome 10 open reading frame 71; similar to hCG1996658	Homo sapiens
1727	cytochrome b5 reductase 3	Homo sapiens
10239	adaptor-related protein complex 3, sigma 2 subunit	Homo sapiens
375298	ceramide kinase-like	Homo sapiens
54858	pyroglutamyl-peptidase I	Homo sapiens
26130	GTPase activating protein and VPS9 domains 1	Homo sapiens
25809	tubulin tyrosine ligase-like family, member 1	Homo sapiens
405	aryl hydrocarbon receptor nuclear translocator	Homo sapiens
9612	nuclear receptor co-repressor 2	Homo sapiens
253260	RPTOR independent companion of MTOR, complex 2	Homo sapiens
3312	heat shock 70kDa protein 8	Homo sapiens
57693	zinc finger protein 317	Homo sapiens
9352	thioredoxin-like 1	Homo sapiens
3646	eukaryotic translation initiation factor 3, subunit E	Homo sapiens
399804	nucleophosmin 1 (nucleolar phosphoprotein B23, numatrin) pseudogene 21; hypothetical LOC100131044; similar to nucleophosmin 1; nucleophosmin (nucleolar phosphoprotein B23, numatrin)	Homo sapiens
4869	nucleophosmin 1 (nucleolar phosphoprotein B23, numatrin) pseudogene 21; hypothetical LOC100131044; similar to nucleophosmin 1; nucleophosmin (nucleolar phosphoprotein B23, numatrin)	Homo sapiens
100131044	nucleophosmin 1 (nucleolar phosphoprotein B23, numatrin) pseudogene 21; hypothetical LOC100131044; similar to nucleophosmin 1; nucleophosmin (nucleolar phosphoprotein B23, numatrin)	Homo sapiens
729342	nucleophosmin 1 (nucleolar phosphoprotein B23, numatrin) pseudogene 21; hypothetical LOC100131044; similar to nucleophosmin 1; nucleophosmin (nucleolar phosphoprotein B23, numatrin)	Homo sapiens
440577	nucleophosmin 1 (nucleolar phosphoprotein B23, numatrin) pseudogene 21; hypothetical LOC100131044; similar to nucleophosmin 1; nucleophosmin (nucleolar phosphoprotein B23, numatrin)	Homo sapiens
100129237	nucleophosmin 1 (nucleolar phosphoprotein B23, numatrin) pseudogene 21; hypothetical LOC100131044; similar to nucleophosmin 1; nucleophosmin (nucleolar phosphoprotein B23, numatrin)	Homo sapiens
729686	nucleophosmin 1 (nucleolar phosphoprotein B23, numatrin) pseudogene 21; hypothetical LOC100131044; similar to nucleophosmin 1; nucleophosmin (nucleolar phosphoprotein B23, numatrin)	Homo sapiens
220359	tigger transposable element derived 3	Homo sapiens
57634	E1A binding protein p400	Homo sapiens
10844	tubulin, gamma complex associated protein 2	Homo sapiens
51111	suppressor of variegation 4-20 homolog 1 (Drosophila)	Homo sapiens
1730	diaphanous homolog 2 (Drosophila)	Homo sapiens
23131	G patch domain containing 8	Homo sapiens
3996	lethal giant larvae homolog 1 (Drosophila)	Homo sapiens
5531	protein phosphatase 4 (formerly X), catalytic subunit	Homo sapiens
2561	gamma-aminobutyric acid (GABA) A receptor, beta 2	Homo sapiens
161931	adenosine deaminase domain containing 2	Homo sapiens
1493	cytotoxic T-lymphocyte-associated protein 4	Homo sapiens
2629	glucosidase, beta; acid (includes glucosylceramidase)	Homo sapiens
1616	death-domain associated protein	Homo sapiens
3788	potassium voltage-gated channel, delayed-rectifier, subfamily S, member 2	Homo sapiens
84262	proteasome (prosome, macropain) assembly chaperone 3	Homo sapiens
93487	mitogen-activated protein kinase 1 interacting protein 1-like	Homo sapiens
641339	zinc finger family member 674	Homo sapiens
5594	mitogen-activated protein kinase 1	Homo sapiens
5357	plastin 1 (I isoform)	Homo sapiens
3764	potassium inwardly-rectifying channel, subfamily J, member 8	Homo sapiens
9249	dehydrogenase/reductase (SDR family) member 3	Homo sapiens
5264	phytanoyl-CoA 2-hydroxylase	Homo sapiens
339855	kyphoscoliosis peptidase	Homo sapiens
5681	protein serine kinase H1	Homo sapiens

1595	cytochrome P450, family 51, subfamily A, polypeptide 1	Homo sapiens
10440	translocase of inner mitochondrial membrane 17 homolog A (yeast)	Homo sapiens
55209	SET domain containing 5	Homo sapiens
53346	transmembrane 6 superfamily member 1	Homo sapiens
64167	endoplasmic reticulum aminopeptidase 2	Homo sapiens
25963	transmembrane protein 87A	Homo sapiens
5450	POU class 2 associating factor 1	Homo sapiens
55195	chromosome 14 open reading frame 105	Homo sapiens
2907	glutamate receptor, ionotropic, N-methyl D-aspartate-associated protein 1 (glutamate binding)	Homo sapiens
2785	guanine nucleotide binding protein (G protein), gamma 3	Homo sapiens
10279	protease, serine, 16 (thymus)	Homo sapiens
79065	ATG9 autophagy related 9 homolog A (S. cerevisiae)	Homo sapiens
51000	solute carrier family 35, member B3	Homo sapiens
64841	glucosamine-phosphate N-acetyltransferase 1	Homo sapiens
55218	exonuclease 3'-5' domain containing 2	Homo sapiens
9945	glutamine-fructose-6-phosphate transaminase 2	Homo sapiens
100133760	similar to Jumonji, AT rich interactive domain 1B (RBP2-like); lysine (K)-specific demethylase 5B	Homo sapiens
10765	similar to Jumonji, AT rich interactive domain 1B (RBP2-like); lysine (K)-specific demethylase 5B	Homo sapiens
55793	family with sequence similarity 63, member A	Homo sapiens
653882	similar to Mast/stem cell growth factor receptor precursor (SCFR) (Proto-oncogene tyrosine-protein kinase Kit) (c-kit) (CD117 antigen); v-kit Hardy-Zuckerman 4 feline sarcoma viral oncogene homolog	Homo sapiens
652799	similar to Mast/stem cell growth factor receptor precursor (SCFR) (Proto-oncogene tyrosine-protein kinase Kit) (c-kit) (CD117 antigen); v-kit Hardy-Zuckerman 4 feline sarcoma viral oncogene homolog	Homo sapiens
3815	similar to Mast/stem cell growth factor receptor precursor (SCFR) (Proto-oncogene tyrosine-protein kinase Kit) (c-kit) (CD117 antigen); v-kit Hardy-Zuckerman 4 feline sarcoma viral oncogene homolog	Homo sapiens
9690	ubiquitin protein ligase E3C	Homo sapiens
8193	D4, zinc and double PHD fingers family 1	Homo sapiens
1499	catenin (cadherin-associated protein), beta 1, 88kDa	Homo sapiens
5335	phospholipase C, gamma 1	Homo sapiens
5216	profilin 1	Homo sapiens
81602	cytidine and dCMP deaminase domain containing 1	Homo sapiens
10294	DnaJ (Hsp40) homolog, subfamily A, member 2	Homo sapiens
58528	Ras-related GTP binding D	Homo sapiens
7127	tumor necrosis factor, alpha-induced protein 2	Homo sapiens
3995	fatty acid desaturase 3	Homo sapiens
138199	chromosome 9 open reading frame 41	Homo sapiens
149371	exocyst complex component 8	Homo sapiens
3090	hypermethylated in cancer 1	Homo sapiens
8675	syntaxin 16	Homo sapiens
5932	retinoblastoma binding protein 8	Homo sapiens
64857	pleckstrin homology domain containing, family G (with RhoGef domain) member 2	Homo sapiens
10042	HMG box domain containing 4	Homo sapiens
9793	cytoskeleton associated protein 5	Homo sapiens
3280	hairy and enhancer of split 1, (Drosophila)	Homo sapiens
129446	xin actin-binding repeat containing 2	Homo sapiens
285527	FRY-like	Homo sapiens
4208	myocyte enhancer factor 2C	Homo sapiens
1746	distal-less homeobox 2	Homo sapiens
2017	cortactin	Homo sapiens
4967	oxoglutarate (alpha-ketoglutarate) dehydrogenase (lipoamide)	Homo sapiens
5454	POU class 3 homeobox 2	Homo sapiens
79717	phosphopantothenoylcysteine synthetase	Homo sapiens
55284	ubiquitin-conjugating enzyme E2W (putative)	Homo sapiens
9653	heparan sulfate 2-O-sulfotransferase 1	Homo sapiens
10051	structural maintenance of chromosomes 4	Homo sapiens
10890	RAB10, member RAS oncogene family	Homo sapiens
84947	serine active site containing 1	Homo sapiens
10085	EGF-like repeats and discoidin I-like domains 3	Homo sapiens
522	ATP synthase, H+ transporting, mitochondrial F0 complex, subunit F6	Homo sapiens
7280	tubulin, beta 2A	Homo sapiens
10490	vesicle transport through interaction with t-SNAREs homolog 1B (yeast)	Homo sapiens
9319	thyroid hormone receptor interactor 13	Homo sapiens
9330	general transcription factor IIIC, polypeptide 3, 102kDa	Homo sapiens
7249	tuberous sclerosis 2	Homo sapiens
392437	similar to ferritin, light polypeptide; ferritin, light polypeptide	Homo sapiens
2512	similar to ferritin, light polypeptide; ferritin, light polypeptide	Homo sapiens
2068	excision repair cross-complementing rodent repair deficiency, complementation group 2	Homo sapiens
55722	centrosomal protein 72kDa	Homo sapiens
22835	zinc finger protein 30 homolog (mouse)	Homo sapiens
1993	ELAV (embryonic lethal, abnormal vision, Drosophila)-like 2 (Hu antigen B)	Homo sapiens
55970	guanine nucleotide binding protein (G protein), gamma 12	Homo sapiens
2152	coagulation factor III (thromboplastin, tissue factor)	Homo sapiens
118987	PDZ domain containing 8	Homo sapiens

11100	heterogeneous nuclear ribonucleoprotein U-like 1	Homo sapiens
7265	tetratricopeptide repeat domain 1	Homo sapiens
23557	SNAP-associated protein	Homo sapiens
1435	colony stimulating factor 1 (macrophage)	Homo sapiens
54726	OTU domain containing 4	Homo sapiens
5321	phospholipase A2, group IVA (cytosolic, calcium-dependent)	Homo sapiens
26156	ribosomal L1 domain containing 1	Homo sapiens
79658	Rho GTPase activating protein 10	Homo sapiens
51520	leucyl-tRNA synthetase	Homo sapiens
9189	zinc finger, BED-type containing 1	Homo sapiens
85476	G elongation factor, mitochondrial 1	Homo sapiens
5727	patched homolog 1 (Drosophila)	Homo sapiens
84909	chromosome 9 open reading frame 3	Homo sapiens
112937	galactosidase, beta 1-like 3	Homo sapiens
342615	schlafen family member 12-like	Homo sapiens
23409	sirtuin (silent mating type information regulation 2 homolog) 4 (S. cerevisiae)	Homo sapiens
64324	nuclear receptor binding SET domain protein 1	Homo sapiens
2932	glycogen synthase kinase 3 beta	Homo sapiens
23597	acyl-CoA thioesterase 9	Homo sapiens
140801	ribosomal protein L10-like	Homo sapiens
1278	collagen, type I, alpha 2	Homo sapiens
80228	ORAI calcium release-activated calcium modulator 2	Homo sapiens
349075	zinc finger protein 713	Homo sapiens
2873	G protein pathway suppressor 1	Homo sapiens
5076	paired box 2	Homo sapiens
6924	transcription elongation factor B (SIII), polypeptide 3 (110kDa, elongin A)	Homo sapiens
10370	Cbp/p300-interacting transactivator, with Glu/Asp-rich carboxy-terminal domain, 2	Homo sapiens
64802	nicotinamide nucleotide adenylyltransferase 1	Homo sapiens
5453	POU class 3 homeobox 1	Homo sapiens
351	amyloid beta (A4) precursor protein	Homo sapiens
27443	cat eye syndrome chromosome region, candidate 2	Homo sapiens
100130633	hypothetical LOC100130633; zinc finger, MYM-type 6	Homo sapiens
9204	hypothetical LOC100130633; zinc finger, MYM-type 6	Homo sapiens
57380	MRS2 magnesium homeostasis factor homolog (S. cerevisiae)	Homo sapiens
10548	transmembrane 9 superfamily member 1	Homo sapiens
9962	solute carrier family 23 (nucleobase transporters), member 2	Homo sapiens
3925	stathmin 1	Homo sapiens
81831	neuropilin (NRP) and tolloid (TLL)-like 2	Homo sapiens
123263	mitochondrial methionyl-tRNA formyltransferase	Homo sapiens
57460	protein phosphatase 1H (PP2C domain containing)	Homo sapiens
6045	ring finger protein 2	Homo sapiens
6597	SWI/SNF related, matrix associated, actin dependent regulator of chromatin, subfamily a, member 4	Homo sapiens
84868	hepatitis A virus cellular receptor 2	Homo sapiens
55643	BTB (POZ) domain containing 2	Homo sapiens
399511	transcription elongation factor A (SII), 1 pseudogene 2; transcription elongation factor A (SII), 1	Homo sapiens
6917	transcription elongation factor A (SII), 1 pseudogene 2; transcription elongation factor A (SII), 1	Homo sapiens
27164	sal-like 3 (Drosophila)	Homo sapiens
9802	DAZ associated protein 2	Homo sapiens
1738	dihydrolipoamide dehydrogenase	Homo sapiens
4800	nuclear transcription factor Y, alpha	Homo sapiens
9839	zinc finger E-box binding homeobox 2	Homo sapiens
84312	breast cancer metastasis-suppressor 1-like	Homo sapiens
1003	cadherin 5, type 2 (vascular endothelium)	Homo sapiens
6683	spastin	Homo sapiens
121391	keratin 74	Homo sapiens
1477	cleavage stimulation factor, 3' pre-RNA, subunit 1, 50kDa	Homo sapiens
1267	2',3'-cyclic nucleotide 3' phosphodiesterase	Homo sapiens
56288	par-3 partitioning defective 3 homolog (C. elegans)	Homo sapiens
23389	mediator complex subunit 13-like	Homo sapiens
3912	laminin, beta 1	Homo sapiens
54955	chromosome 1 open reading frame 109	Homo sapiens
286148	dpy-19-like 4 (C. elegans)	Homo sapiens
10497	unc-13 homolog B (C. elegans)	Homo sapiens
5411	pinin, desmosome associated protein	Homo sapiens
5708	proteasome (prosome, macropain) 26S subunit, non-ATPase, 2	Homo sapiens
6775098	NADH-ubiquinone oxidoreductase chain 1	Homo sapiens
4535	NADH-ubiquinone oxidoreductase chain 1	Homo sapiens
92999	zinc finger and BTB domain containing 47	Homo sapiens
4144	methionine adenosyltransferase II, alpha	Homo sapiens
27158	NADPH dependent diflavin oxidoreductase 1	Homo sapiens
81550	tudor domain containing 3	Homo sapiens
54879	suppression of tumorigenicity 7 like	Homo sapiens
90120	chromosome 9 open reading frame 69	Homo sapiens
55250	elongation protein 2 homolog (S. cerevisiae)	Homo sapiens
8518	inhibitor of kappa light polypeptide gene enhancer in B-cells, kinase complex-associated protein	Homo sapiens

51809	UDP-N-acetyl-alpha-D-galactosamine:polypeptide N-acetylgalactosaminyltransferase 7 (GalNAc-T7)	Homo sapiens
57169	zinc finger, NFX1-type containing 1	Homo sapiens
51335	neugrin, neurite outgrowth associated	Homo sapiens
6605	SWI/SNF related, matrix associated, actin dependent regulator of chromatin, subfamily e, member 1	Homo sapiens
2006	elastin	Homo sapiens
286	ankyrin 1, erythrocytic	Homo sapiens
5910	RAP1, GTP-GDP dissociation stimulator 1	Homo sapiens
57552	arylacetamide deacetylase-like 1	Homo sapiens
9443	mediator complex subunit 7	Homo sapiens
57469	PNMA-like 2	Homo sapiens
5576	protein kinase, cAMP-dependent, regulatory, type II, alpha	Homo sapiens
3083	HGF activator	Homo sapiens
3069	high density lipoprotein binding protein	Homo sapiens
54943	DnaJ (Hsp40) homolog, subfamily C, member 28	Homo sapiens
991	cell division cycle 20 homolog (S. cerevisiae)	Homo sapiens
1051	CCAAT/enhancer binding protein (C/EBP), beta	Homo sapiens
5074	PRKC, apoptosis, WT1, regulator	Homo sapiens
10771	zinc finger, MYND domain containing 11	Homo sapiens
9491	proteasome (prosome, macropain) inhibitor subunit 1 (PI31)	Homo sapiens
8672	eukaryotic translation initiation factor 4 gamma, 3	Homo sapiens
51021	mitochondrial ribosomal protein S16	Homo sapiens
10276	neuroepithelial cell transforming 1	Homo sapiens
23429	RING1 and YY1 binding protein	Homo sapiens
1778	dynein, cytoplasmic 1, heavy chain 1	Homo sapiens
996	cell division cycle 27 homolog (S. cerevisiae)	Homo sapiens
51647	family with sequence similarity 96, member B	Homo sapiens
10059	dynamin 1-like	Homo sapiens
169714	quiescin Q6 sulfhydryl oxidase 2	Homo sapiens
9213	xenotropic and polytropic retrovirus receptor	Homo sapiens
441531	phosphoglycerate mutase family member 4	Homo sapiens
57708	mesoderm induction early response 1 homolog (Xenopus laevis)	Homo sapiens
1938	eukaryotic translation elongation factor 2	Homo sapiens
81790	ring finger protein 170	Homo sapiens
2591	UDP-N-acetyl-alpha-D-galactosamine:polypeptide N-acetylgalactosaminyltransferase 3 (GalNAc-T3)	Homo sapiens
54797	mediator complex subunit 18	Homo sapiens
23102	TBC1 domain family, member 2B	Homo sapiens
56919	DEAH (Asp-Glu-Ala-His) box polypeptide 33	Homo sapiens
6541	solute carrier family 7 (cationic amino acid transporter, y+ system), member 1	Homo sapiens
8643	patched homolog 2 (Drosophila)	Homo sapiens
79269	WD repeat domain 32	Homo sapiens
92	activin A receptor, type IIA	Homo sapiens
10316	neuromedin U receptor 1	Homo sapiens
4012	leucyl/cystinyl aminopeptidase	Homo sapiens
5352	procollagen-lysine, 2-oxoglutarate 5-dioxygenase 2	Homo sapiens
3609	interleukin enhancer binding factor 3, 90kDa	Homo sapiens
51665	ankyrin repeat and SOCS box-containing 1	Homo sapiens
84641	hippocampus abundant transcript-like 1	Homo sapiens
57606	SLAIN motif family, member 2	Homo sapiens
79134	transmembrane protein 185B (pseudogene)	Homo sapiens
5208	6-phosphofructo-2-kinase/fructose-2,6-biphosphatase 2	Homo sapiens
55740	enabled homolog (Drosophila)	Homo sapiens
54989	zinc finger protein 770	Homo sapiens
8625	regulatory factor X-associated ankyrin-containing protein	Homo sapiens
51588	protein inhibitor of activated STAT, 4	Homo sapiens
26207	phosphatidylinositol transfer protein, cytoplasmic 1	Homo sapiens
342667	SH3 and cysteine rich domain 2	Homo sapiens
258010	small VCP/p97-interacting protein	Homo sapiens
26504	cyclin M4	Homo sapiens
5144	phosphodiesterase 4D, cAMP-specific (phosphodiesterase E3 dunce homolog, Drosophila)	Homo sapiens
9100	ubiquitin specific peptidase 10	Homo sapiens
55669	mitofusin 1	Homo sapiens
81611	acidic (leucine-rich) nuclear phosphoprotein 32 family, member E	Homo sapiens
8202	nuclear receptor coactivator 3	Homo sapiens
389151	UPF0572 protein ENSP00000372650; hypothetical protein LOC389151	Homo sapiens
729627	UPF0572 protein ENSP00000372650; hypothetical protein LOC389151	Homo sapiens
7072	TIA1 cytotoxic granule-associated RNA binding protein	Homo sapiens
79691	queuine tRNA-ribosyltransferase domain containing 1	Homo sapiens
85440	dedicator of cytokinesis 7	Homo sapiens
5870	RAB6C, member RAS oncogene family; RAB6A, member RAS oncogene family; hypothetical LOC100130819; RAB6C-like	Homo sapiens
84084	RAB6C, member RAS oncogene family; RAB6A, member RAS oncogene family; hypothetical LOC100130819; RAB6C-like	Homo sapiens
150786	RAB6C, member RAS oncogene family; RAB6A, member RAS oncogene family; hypothetical LOC100130819; RAB6C-like	Homo sapiens
100130819	RAB6C, member RAS oncogene family; RAB6A, member RAS oncogene family; hypothetical LOC100130819; RAB6C-like	Homo sapiens

1659	DEAH (Asp-Glu-Ala-His) box polypeptide 8	Homo sapiens
54806	Abelson helper integration site 1	Homo sapiens
84318	coiled-coil domain containing 77	Homo sapiens
84444	DOT1-like, histone H3 methyltransferase (S. cerevisiae)	Homo sapiens
642738	ribosomal protein L21 pseudogene 134; ribosomal protein L21 pseudogene 80; ribosomal protein L21 pseudogene 20; ribosomal protein L21 pseudogene 46; ribosomal protein L21 pseudogene 45; ribosomal protein L21 pseudogene 131; ribosomal protein L21 pseudogene 16; ribosomal protein L21 pseudogene 53; ribosomal protein L21 pseudogene 120; ribosomal protein L21 pseudogene 37; ribosomal protein L21 pseudogene 93; ribosomal protein L21 pseudogene 39; ribosomal protein L21 pseudogene 29; ribosomal protein L21 pseudogene 28; ribosomal protein L21 pseudogene 14; ribosomal protein L21 pseudogene 98; ribosomal protein L21 pseudogene 105; ribosomal protein L21 pseudogene 87; ribosomal protein L21 pseudogene 128; ribosomal protein L21 pseudogene 69; ribosomal protein L21 pseudogene 97; ribosomal protein L21; ribosomal protein L21 pseudogene 119; ribosomal protein L21 pseudogene 125	Homo sapiens
729208	ribosomal protein L21 pseudogene 134; ribosomal protein L21 pseudogene 80; ribosomal protein L21 pseudogene 20; ribosomal protein L21 pseudogene 46; ribosomal protein L21 pseudogene 45; ribosomal protein L21 pseudogene 131; ribosomal protein L21 pseudogene 16; ribosomal protein L21 pseudogene 53; ribosomal protein L21 pseudogene 120; ribosomal protein L21 pseudogene 37; ribosomal protein L21 pseudogene 93; ribosomal protein L21 pseudogene 39; ribosomal protein L21 pseudogene 29; ribosomal protein L21 pseudogene 28; ribosomal protein L21 pseudogene 14; ribosomal protein L21 pseudogene 98; ribosomal protein L21 pseudogene 105; ribosomal protein L21 pseudogene 87; ribosomal protein L21 pseudogene 128; ribosomal protein L21 pseudogene 69; ribosomal protein L21 pseudogene 97; ribosomal protein L21; ribosomal protein L21 pseudogene 119; ribosomal protein L21 pseudogene 125	Homo sapiens
653156	ribosomal protein L21 pseudogene 134; ribosomal protein L21 pseudogene 80; ribosomal protein L21 pseudogene 20; ribosomal protein L21 pseudogene 46; ribosomal protein L21 pseudogene 45; ribosomal protein L21 pseudogene 131; ribosomal protein L21 pseudogene 16; ribosomal protein L21 pseudogene 53; ribosomal protein L21 pseudogene 120; ribosomal protein L21 pseudogene 37; ribosomal protein L21 pseudogene 93; ribosomal protein L21 pseudogene 39; ribosomal protein L21 pseudogene 29; ribosomal protein L21 pseudogene 28; ribosomal protein L21 pseudogene 14; ribosomal protein L21 pseudogene 98; ribosomal protein L21 pseudogene 105; ribosomal protein L21 pseudogene 87; ribosomal protein L21 pseudogene 128; ribosomal protein L21 pseudogene 69; ribosomal protein L21 pseudogene 97; ribosomal protein L21; ribosomal protein L21 pseudogene 119; ribosomal protein L21 pseudogene 125	Homo sapiens
645174	ribosomal protein L21 pseudogene 134; ribosomal protein L21 pseudogene 80; ribosomal protein L21 pseudogene 20; ribosomal protein L21 pseudogene 46; ribosomal protein L21 pseudogene 45; ribosomal protein L21 pseudogene 131; ribosomal protein L21 pseudogene 16; ribosomal protein L21 pseudogene 53; ribosomal protein L21 pseudogene 120; ribosomal protein L21 pseudogene 37; ribosomal protein L21 pseudogene 93; ribosomal protein L21 pseudogene 39; ribosomal protein L21 pseudogene 29; ribosomal protein L21 pseudogene 28; ribosomal protein L21 pseudogene 14; ribosomal protein L21 pseudogene 98; ribosomal protein L21 pseudogene 105; ribosomal protein L21 pseudogene 87; ribosomal protein L21 pseudogene 128; ribosomal protein L21 pseudogene 69; ribosomal protein L21 pseudogene 97; ribosomal protein L21; ribosomal protein L21 pseudogene 119; ribosomal protein L21 pseudogene 125	Homo sapiens
728693	ribosomal protein L21 pseudogene 134; ribosomal protein L21 pseudogene 80; ribosomal protein L21 pseudogene 20; ribosomal protein L21 pseudogene 46; ribosomal protein L21 pseudogene 45; ribosomal protein L21 pseudogene 131; ribosomal protein L21 pseudogene 16; ribosomal protein L21 pseudogene 53; ribosomal protein L21 pseudogene 120; ribosomal protein L21 pseudogene 37; ribosomal protein L21 pseudogene 93; ribosomal protein L21 pseudogene 39; ribosomal protein L21 pseudogene 29; ribosomal protein L21 pseudogene 28; ribosomal protein L21 pseudogene 14; ribosomal protein L21 pseudogene 98; ribosomal protein L21 pseudogene 105; ribosomal protein L21 pseudogene 87; ribosomal protein L21 pseudogene 128; ribosomal protein L21 pseudogene 69; ribosomal protein L21 pseudogene 97; ribosomal protein L21; ribosomal protein L21 pseudogene 119; ribosomal protein L21 pseudogene 125	Homo sapiens
100129685	ribosomal protein L21 pseudogene 134; ribosomal protein L21 pseudogene 80; ribosomal protein L21 pseudogene 20; ribosomal protein L21 pseudogene 46; ribosomal protein L21 pseudogene 45; ribosomal protein L21 pseudogene 131; ribosomal protein L21 pseudogene 16; ribosomal protein L21 pseudogene 53; ribosomal protein L21 pseudogene 120; ribosomal protein L21 pseudogene 37; ribosomal protein L21 pseudogene 93; ribosomal protein L21 pseudogene 39; ribosomal protein L21 pseudogene 29; ribosomal protein L21 pseudogene 28; ribosomal protein L21 pseudogene 14; ribosomal protein L21 pseudogene 98; ribosomal protein L21 pseudogene 105; ribosomal protein L21 pseudogene 87; ribosomal protein L21 pseudogene 128; ribosomal protein L21 pseudogene 69; ribosomal protein L21 pseudogene 97; ribosomal protein L21; ribosomal protein L21 pseudogene 119; ribosomal protein L21 pseudogene 125	Homo sapiens

100129141	ribosomal protein L21 pseudogene 134; ribosomal protein L21 pseudogene 80; ribosomal protein L21 pseudogene 20; ribosomal protein L21 pseudogene 46; ribosomal protein L21 pseudogene 45; ribosomal protein L21 pseudogene 131; ribosomal protein L21 pseudogene 16; ribosomal protein L21 pseudogene 53; ribosomal protein L21 pseudogene 120; ribosomal protein L21 pseudogene 37; ribosomal protein L21 pseudogene 93; ribosomal protein L21 pseudogene 39; ribosomal protein L21 pseudogene 29; ribosomal protein L21 pseudogene 28; ribosomal protein L21 pseudogene 14; ribosomal protein L21 pseudogene 98; ribosomal protein L21 pseudogene 105; ribosomal protein L21 pseudogene 87; ribosomal protein L21 pseudogene 128; ribosomal protein L21 pseudogene 69; ribosomal protein L21 pseudogene 97; ribosomal protein L21; ribosomal protein L21 pseudogene 119; ribosomal protein L21 pseudogene 125	Homo sapiens
653079	ribosomal protein L21 pseudogene 134; ribosomal protein L21 pseudogene 80; ribosomal protein L21 pseudogene 20; ribosomal protein L21 pseudogene 46; ribosomal protein L21 pseudogene 45; ribosomal protein L21 pseudogene 131; ribosomal protein L21 pseudogene 16; ribosomal protein L21 pseudogene 53; ribosomal protein L21 pseudogene 120; ribosomal protein L21 pseudogene 37; ribosomal protein L21 pseudogene 93; ribosomal protein L21 pseudogene 39; ribosomal protein L21 pseudogene 29; ribosomal protein L21 pseudogene 28; ribosomal protein L21 pseudogene 14; ribosomal protein L21 pseudogene 98; ribosomal protein L21 pseudogene 105; ribosomal protein L21 pseudogene 87; ribosomal protein L21 pseudogene 128; ribosomal protein L21 pseudogene 69; ribosomal protein L21 pseudogene 97; ribosomal protein L21; ribosomal protein L21 pseudogene 119; ribosomal protein L21 pseudogene 125	Homo sapiens
6144	ribosomal protein L21 pseudogene 134; ribosomal protein L21 pseudogene 80; ribosomal protein L21 pseudogene 20; ribosomal protein L21 pseudogene 46; ribosomal protein L21 pseudogene 45; ribosomal protein L21 pseudogene 131; ribosomal protein L21 pseudogene 16; ribosomal protein L21 pseudogene 53; ribosomal protein L21 pseudogene 120; ribosomal protein L21 pseudogene 37; ribosomal protein L21 pseudogene 93; ribosomal protein L21 pseudogene 39; ribosomal protein L21 pseudogene 29; ribosomal protein L21 pseudogene 28; ribosomal protein L21 pseudogene 14; ribosomal protein L21 pseudogene 98; ribosomal protein L21 pseudogene 105; ribosomal protein L21 pseudogene 87; ribosomal protein L21 pseudogene 128; ribosomal protein L21 pseudogene 69; ribosomal protein L21 pseudogene 97; ribosomal protein L21; ribosomal protein L21 pseudogene 119; ribosomal protein L21 pseudogene 125	Homo sapiens
729402	ribosomal protein L21 pseudogene 134; ribosomal protein L21 pseudogene 80; ribosomal protein L21 pseudogene 20; ribosomal protein L21 pseudogene 46; ribosomal protein L21 pseudogene 45; ribosomal protein L21 pseudogene 131; ribosomal protein L21 pseudogene 16; ribosomal protein L21 pseudogene 53; ribosomal protein L21 pseudogene 120; ribosomal protein L21 pseudogene 37; ribosomal protein L21 pseudogene 93; ribosomal protein L21 pseudogene 39; ribosomal protein L21 pseudogene 29; ribosomal protein L21 pseudogene 28; ribosomal protein L21 pseudogene 14; ribosomal protein L21 pseudogene 98; ribosomal protein L21 pseudogene 105; ribosomal protein L21 pseudogene 87; ribosomal protein L21 pseudogene 128; ribosomal protein L21 pseudogene 69; ribosomal protein L21 pseudogene 97; ribosomal protein L21; ribosomal protein L21 pseudogene 119; ribosomal protein L21 pseudogene 125	Homo sapiens
440487	ribosomal protein L21 pseudogene 134; ribosomal protein L21 pseudogene 80; ribosomal protein L21 pseudogene 20; ribosomal protein L21 pseudogene 46; ribosomal protein L21 pseudogene 45; ribosomal protein L21 pseudogene 131; ribosomal protein L21 pseudogene 16; ribosomal protein L21 pseudogene 53; ribosomal protein L21 pseudogene 120; ribosomal protein L21 pseudogene 37; ribosomal protein L21 pseudogene 93; ribosomal protein L21 pseudogene 39; ribosomal protein L21 pseudogene 29; ribosomal protein L21 pseudogene 28; ribosomal protein L21 pseudogene 14; ribosomal protein L21 pseudogene 98; ribosomal protein L21 pseudogene 105; ribosomal protein L21 pseudogene 87; ribosomal protein L21 pseudogene 128; ribosomal protein L21 pseudogene 69; ribosomal protein L21 pseudogene 97; ribosomal protein L21; ribosomal protein L21 pseudogene 119; ribosomal protein L21 pseudogene 125	Homo sapiens
653665	ribosomal protein L21 pseudogene 134; ribosomal protein L21 pseudogene 80; ribosomal protein L21 pseudogene 20; ribosomal protein L21 pseudogene 46; ribosomal protein L21 pseudogene 45; ribosomal protein L21 pseudogene 131; ribosomal protein L21 pseudogene 16; ribosomal protein L21 pseudogene 53; ribosomal protein L21 pseudogene 120; ribosomal protein L21 pseudogene 37; ribosomal protein L21 pseudogene 93; ribosomal protein L21 pseudogene 39; ribosomal protein L21 pseudogene 29; ribosomal protein L21 pseudogene 28; ribosomal protein L21 pseudogene 14; ribosomal protein L21 pseudogene 98; ribosomal protein L21 pseudogene 105; ribosomal protein L21 pseudogene 87; ribosomal protein L21 pseudogene 128; ribosomal protein L21 pseudogene 69; ribosomal protein L21 pseudogene 97; ribosomal protein L21; ribosomal protein L21 pseudogene 119; ribosomal protein L21 pseudogene 125	Homo sapiens
389156	ribosomal protein L21 pseudogene 134; ribosomal protein L21 pseudogene 80; ribosomal protein L21 pseudogene 20; ribosomal protein L21 pseudogene 46; ribosomal protein L21 pseudogene 45; ribosomal protein L21 pseudogene 131; ribosomal protein L21 pseudogene 16; ribosomal protein L21 pseudogene 53; ribosomal protein L21 pseudogene 120; ribosomal protein L21 pseudogene 37; ribosomal protein L21 pseudogene 93; ribosomal protein L21 pseudogene 39; ribosomal protein L21 pseudogene 29; ribosomal protein L21 pseudogene 28; ribosomal protein L21 pseudogene 14; ribosomal protein L21 pseudogene 98; ribosomal protein L21 pseudogene 105; ribosomal protein L21 pseudogene 87; ribosomal protein L21 pseudogene 128; ribosomal protein L21 pseudogene 69; ribosomal protein L21 pseudogene 97; ribosomal protein L21; ribosomal protein L21 pseudogene 119; ribosomal protein L21 pseudogene 125	Homo sapiens

440575	ribosomal protein L21 pseudogene 134; ribosomal protein L21 pseudogene 80; ribosomal protein L21 pseudogene 20; ribosomal protein L21 pseudogene 46; ribosomal protein L21 pseudogene 45; ribosomal protein L21 pseudogene 131; ribosomal protein L21 pseudogene 16; ribosomal protein L21 pseudogene 53; ribosomal protein L21 pseudogene 120; ribosomal protein L21 pseudogene 37; ribosomal protein L21 pseudogene 93; ribosomal protein L21 pseudogene 39; ribosomal protein L21 pseudogene 29; ribosomal protein L21 pseudogene 28; ribosomal protein L21 pseudogene 14; ribosomal protein L21 pseudogene 98; ribosomal protein L21 pseudogene 105; ribosomal protein L21 pseudogene 87; ribosomal protein L21 pseudogene 128; ribosomal protein L21 pseudogene 69; ribosomal protein L21 pseudogene 97; ribosomal protein L21; ribosomal protein L21 pseudogene 119; ribosomal protein L21 pseudogene 125	Homo sapiens
727821	ribosomal protein L21 pseudogene 134; ribosomal protein L21 pseudogene 80; ribosomal protein L21 pseudogene 20; ribosomal protein L21 pseudogene 46; ribosomal protein L21 pseudogene 45; ribosomal protein L21 pseudogene 131; ribosomal protein L21 pseudogene 16; ribosomal protein L21 pseudogene 53; ribosomal protein L21 pseudogene 120; ribosomal protein L21 pseudogene 37; ribosomal protein L21 pseudogene 93; ribosomal protein L21 pseudogene 39; ribosomal protein L21 pseudogene 29; ribosomal protein L21 pseudogene 28; ribosomal protein L21 pseudogene 14; ribosomal protein L21 pseudogene 98; ribosomal protein L21 pseudogene 105; ribosomal protein L21 pseudogene 87; ribosomal protein L21 pseudogene 128; ribosomal protein L21 pseudogene 69; ribosomal protein L21 pseudogene 97; ribosomal protein L21; ribosomal protein L21 pseudogene 119; ribosomal protein L21 pseudogene 125	Homo sapiens
387753	ribosomal protein L21 pseudogene 134; ribosomal protein L21 pseudogene 80; ribosomal protein L21 pseudogene 20; ribosomal protein L21 pseudogene 46; ribosomal protein L21 pseudogene 45; ribosomal protein L21 pseudogene 131; ribosomal protein L21 pseudogene 16; ribosomal protein L21 pseudogene 53; ribosomal protein L21 pseudogene 120; ribosomal protein L21 pseudogene 37; ribosomal protein L21 pseudogene 93; ribosomal protein L21 pseudogene 39; ribosomal protein L21 pseudogene 29; ribosomal protein L21 pseudogene 28; ribosomal protein L21 pseudogene 14; ribosomal protein L21 pseudogene 98; ribosomal protein L21 pseudogene 105; ribosomal protein L21 pseudogene 87; ribosomal protein L21 pseudogene 128; ribosomal protein L21 pseudogene 69; ribosomal protein L21 pseudogene 97; ribosomal protein L21; ribosomal protein L21 pseudogene 119; ribosomal protein L21 pseudogene 125	Homo sapiens
388621	ribosomal protein L21 pseudogene 134; ribosomal protein L21 pseudogene 80; ribosomal protein L21 pseudogene 20; ribosomal protein L21 pseudogene 46; ribosomal protein L21 pseudogene 45; ribosomal protein L21 pseudogene 131; ribosomal protein L21 pseudogene 16; ribosomal protein L21 pseudogene 53; ribosomal protein L21 pseudogene 120; ribosomal protein L21 pseudogene 37; ribosomal protein L21 pseudogene 93; ribosomal protein L21 pseudogene 39; ribosomal protein L21 pseudogene 29; ribosomal protein L21 pseudogene 28; ribosomal protein L21 pseudogene 14; ribosomal protein L21 pseudogene 98; ribosomal protein L21 pseudogene 105; ribosomal protein L21 pseudogene 87; ribosomal protein L21 pseudogene 128; ribosomal protein L21 pseudogene 69; ribosomal protein L21 pseudogene 97; ribosomal protein L21; ribosomal protein L21 pseudogene 119; ribosomal protein L21 pseudogene 125	Homo sapiens
731688	ribosomal protein L21 pseudogene 134; ribosomal protein L21 pseudogene 80; ribosomal protein L21 pseudogene 20; ribosomal protein L21 pseudogene 46; ribosomal protein L21 pseudogene 45; ribosomal protein L21 pseudogene 131; ribosomal protein L21 pseudogene 16; ribosomal protein L21 pseudogene 53; ribosomal protein L21 pseudogene 120; ribosomal protein L21 pseudogene 37; ribosomal protein L21 pseudogene 93; ribosomal protein L21 pseudogene 39; ribosomal protein L21 pseudogene 29; ribosomal protein L21 pseudogene 28; ribosomal protein L21 pseudogene 14; ribosomal protein L21 pseudogene 98; ribosomal protein L21 pseudogene 105; ribosomal protein L21 pseudogene 87; ribosomal protein L21 pseudogene 128; ribosomal protein L21 pseudogene 69; ribosomal protein L21 pseudogene 97; ribosomal protein L21; ribosomal protein L21 pseudogene 119; ribosomal protein L21 pseudogene 125	Homo sapiens
728782	ribosomal protein L21 pseudogene 134; ribosomal protein L21 pseudogene 80; ribosomal protein L21 pseudogene 20; ribosomal protein L21 pseudogene 46; ribosomal protein L21 pseudogene 45; ribosomal protein L21 pseudogene 131; ribosomal protein L21 pseudogene 16; ribosomal protein L21 pseudogene 53; ribosomal protein L21 pseudogene 120; ribosomal protein L21 pseudogene 37; ribosomal protein L21 pseudogene 93; ribosomal protein L21 pseudogene 39; ribosomal protein L21 pseudogene 29; ribosomal protein L21 pseudogene 28; ribosomal protein L21 pseudogene 14; ribosomal protein L21 pseudogene 98; ribosomal protein L21 pseudogene 105; ribosomal protein L21 pseudogene 87; ribosomal protein L21 pseudogene 128; ribosomal protein L21 pseudogene 69; ribosomal protein L21 pseudogene 97; ribosomal protein L21; ribosomal protein L21 pseudogene 119; ribosomal protein L21 pseudogene 125	Homo sapiens
653737	ribosomal protein L21 pseudogene 134; ribosomal protein L21 pseudogene 80; ribosomal protein L21 pseudogene 20; ribosomal protein L21 pseudogene 46; ribosomal protein L21 pseudogene 45; ribosomal protein L21 pseudogene 131; ribosomal protein L21 pseudogene 16; ribosomal protein L21 pseudogene 53; ribosomal protein L21 pseudogene 120; ribosomal protein L21 pseudogene 37; ribosomal protein L21 pseudogene 93; ribosomal protein L21 pseudogene 39; ribosomal protein L21 pseudogene 29; ribosomal protein L21 pseudogene 28; ribosomal protein L21 pseudogene 14; ribosomal protein L21 pseudogene 98; ribosomal protein L21 pseudogene 105; ribosomal protein L21 pseudogene 87; ribosomal protein L21 pseudogene 128; ribosomal protein L21 pseudogene 69; ribosomal protein L21 pseudogene 97; ribosomal protein L21; ribosomal protein L21 pseudogene 119; ribosomal protein L21 pseudogene 125	Homo sapiens

388532	ribosomal protein L21 pseudogene 134; ribosomal protein L21 pseudogene 80; ribosomal protein L21 pseudogene 20; ribosomal protein L21 pseudogene 46; ribosomal protein L21 pseudogene 45; ribosomal protein L21 pseudogene 131; ribosomal protein L21 pseudogene 16; ribosomal protein L21 pseudogene 53; ribosomal protein L21 pseudogene 120; ribosomal protein L21 pseudogene 37; ribosomal protein L21 pseudogene 93; ribosomal protein L21 pseudogene 39; ribosomal protein L21 pseudogene 29; ribosomal protein L21 pseudogene 28; ribosomal protein L21 pseudogene 14; ribosomal protein L21 pseudogene 98; ribosomal protein L21 pseudogene 105; ribosomal protein L21 pseudogene 87; ribosomal protein L21 pseudogene 128; ribosomal protein L21 pseudogene 69; ribosomal protein L21 pseudogene 97; ribosomal protein L21; ribosomal protein L21 pseudogene 119; ribosomal protein L21 pseudogene 125	Homo sapiens
100131672	ribosomal protein L21 pseudogene 134; ribosomal protein L21 pseudogene 80; ribosomal protein L21 pseudogene 20; ribosomal protein L21 pseudogene 46; ribosomal protein L21 pseudogene 45; ribosomal protein L21 pseudogene 131; ribosomal protein L21 pseudogene 16; ribosomal protein L21 pseudogene 53; ribosomal protein L21 pseudogene 120; ribosomal protein L21 pseudogene 37; ribosomal protein L21 pseudogene 93; ribosomal protein L21 pseudogene 39; ribosomal protein L21 pseudogene 29; ribosomal protein L21 pseudogene 28; ribosomal protein L21 pseudogene 14; ribosomal protein L21 pseudogene 98; ribosomal protein L21 pseudogene 105; ribosomal protein L21 pseudogene 87; ribosomal protein L21 pseudogene 128; ribosomal protein L21 pseudogene 69; ribosomal protein L21 pseudogene 97; ribosomal protein L21; ribosomal protein L21 pseudogene 119; ribosomal protein L21 pseudogene 125	Homo sapiens
100133273	ribosomal protein L21 pseudogene 134; ribosomal protein L21 pseudogene 80; ribosomal protein L21 pseudogene 20; ribosomal protein L21 pseudogene 46; ribosomal protein L21 pseudogene 45; ribosomal protein L21 pseudogene 131; ribosomal protein L21 pseudogene 16; ribosomal protein L21 pseudogene 53; ribosomal protein L21 pseudogene 120; ribosomal protein L21 pseudogene 37; ribosomal protein L21 pseudogene 93; ribosomal protein L21 pseudogene 39; ribosomal protein L21 pseudogene 29; ribosomal protein L21 pseudogene 28; ribosomal protein L21 pseudogene 14; ribosomal protein L21 pseudogene 98; ribosomal protein L21 pseudogene 105; ribosomal protein L21 pseudogene 87; ribosomal protein L21 pseudogene 128; ribosomal protein L21 pseudogene 69; ribosomal protein L21 pseudogene 97; ribosomal protein L21; ribosomal protein L21 pseudogene 119; ribosomal protein L21 pseudogene 125	Homo sapiens
100131205	ribosomal protein L21 pseudogene 134; ribosomal protein L21 pseudogene 80; ribosomal protein L21 pseudogene 20; ribosomal protein L21 pseudogene 46; ribosomal protein L21 pseudogene 45; ribosomal protein L21 pseudogene 131; ribosomal protein L21 pseudogene 16; ribosomal protein L21 pseudogene 53; ribosomal protein L21 pseudogene 120; ribosomal protein L21 pseudogene 37; ribosomal protein L21 pseudogene 93; ribosomal protein L21 pseudogene 39; ribosomal protein L21 pseudogene 29; ribosomal protein L21 pseudogene 28; ribosomal protein L21 pseudogene 14; ribosomal protein L21 pseudogene 98; ribosomal protein L21 pseudogene 105; ribosomal protein L21 pseudogene 87; ribosomal protein L21 pseudogene 128; ribosomal protein L21 pseudogene 69; ribosomal protein L21 pseudogene 97; ribosomal protein L21; ribosomal protein L21 pseudogene 119; ribosomal protein L21 pseudogene 125	Homo sapiens
80255	solute carrier family 35, member F5	Homo sapiens
84725	pleckstrin homology domain containing, family A (phosphoinositide binding specific) member 8	Homo sapiens
113251	La ribonucleoprotein domain family, member 4	Homo sapiens
2911	glutamate receptor, metabotropic 1	Homo sapiens
377677	carbonic anhydrase XIII	Homo sapiens
117285	defensin, beta 118	Homo sapiens
23170	tubulin tyrosine ligase-like family, member 12	Homo sapiens
54058	chromosome 21 open reading frame 58	Homo sapiens
7678	zinc finger protein 124	Homo sapiens
195828	zinc finger protein 367	Homo sapiens
79745	CAP-GLY domain containing linker protein family, member 4	Homo sapiens
26094	WD repeat domain 21A	Homo sapiens
5521	protein phosphatase 2 (formerly 2A), regulatory subunit B, beta isoform	Homo sapiens
29775	caspase recruitment domain family, member 10	Homo sapiens
51643	transmembrane BAX inhibitor motif containing 4	Homo sapiens
90268	family with sequence similarity 105, member B	Homo sapiens
57474	zinc finger protein 490	Homo sapiens
11069	Rap guanine nucleotide exchange factor (GEF) 4	Homo sapiens
8348	histone cluster 1, H2bo	Homo sapiens
94039	zinc finger protein 101	Homo sapiens
135250	retinoic acid early transcript 1E	Homo sapiens
6449	small glutamine-rich tetratricopeptide repeat (TPR)-containing, alpha	Homo sapiens
9810	ring finger protein 40	Homo sapiens
9126	structural maintenance of chromosomes 3	Homo sapiens
81490	phosphatidylserine synthase 2	Homo sapiens
91442	chromosome 19 open reading frame 40	Homo sapiens
2926	G-rich RNA sequence binding factor 1	Homo sapiens
9110	myotubularin related protein 4	Homo sapiens
8816	WD repeat domain 22	Homo sapiens
9926	lysophosphatidylglycerol acyltransferase 1	Homo sapiens
9730	Vpr (HIV-1) binding protein	Homo sapiens
201292	tripartite motif-containing 65	Homo sapiens
140564	apolipoprotein B mRNA editing enzyme, catalytic polypeptide-like 3D	Homo sapiens
7553	zinc finger protein 7	Homo sapiens

646817	SET nuclear oncogene; similar to SET translocation	Homo sapiens
6418	SET nuclear oncogene; similar to SET translocation	Homo sapiens
22990	pecanex homolog (Drosophila)	Homo sapiens
29109	formin homology 2 domain containing 1	Homo sapiens
90580	chromosome 19 open reading frame 52	Homo sapiens
813	calumenin	Homo sapiens
54982	ceroid-lipofuscinosis, neuronal 6, late infantile, variant	Homo sapiens
9091	phosphatidylinositol glycan anchor biosynthesis, class Q	Homo sapiens
363	aquaporin 6, kidney specific	Homo sapiens
2117	ets variant 3	Homo sapiens
140468	COX11 homolog, cytochrome c oxidase assembly protein (yeast) pseudogene	Homo sapiens
84948	tigger transposable element derived 5	Homo sapiens
51072	mediator of cell motility 1; similar to mediator of cell motility 1	Homo sapiens
645175	mediator of cell motility 1; similar to mediator of cell motility 1	Homo sapiens
4891	solute carrier family 11 (proton-coupled divalent metal ion transporters), member 2	Homo sapiens
8932	methyl-CpG binding domain protein 2	Homo sapiens
25794	fascin homolog 2, actin-bundling protein, retinal (Strongylocentrotus purpuratus)	Homo sapiens
83549	uridine-cytidine kinase 1	Homo sapiens
91612	churchill domain containing 1	Homo sapiens
57326	pre-B-cell leukemia homeobox interacting protein 1	Homo sapiens
58496	lymphocyte antigen 6 complex, locus G5B; casein kinase 2, beta polypeptide	Homo sapiens
1460	lymphocyte antigen 6 complex, locus G5B; casein kinase 2, beta polypeptide	Homo sapiens
7161	tumor protein p73	Homo sapiens
54475	notchless homolog 1 (Drosophila)	Homo sapiens
90139	tetraspanin 18	Homo sapiens
4605	v-myb myeloblastosis viral oncogene homolog (avian)-like 2	Homo sapiens
3835	kinesin family member 22	Homo sapiens
113878	deltex homolog 2 (Drosophila)	Homo sapiens
3800	kinesin family member 5C	Homo sapiens
83876	maestro	Homo sapiens
7322	ubiquitin-conjugating enzyme E2D 2 (UBC4/5 homolog, yeast)	Homo sapiens
643853	transmembrane protein with metallophosphoesterase domain	Homo sapiens
284541	cytochrome P450, family 4, subfamily A, polypeptide 22	Homo sapiens
10376	hypothetical gene supported by AF081484; NM_006082; tubulin, alpha 1b	Homo sapiens
399942	hypothetical gene supported by AF081484; NM_006082; tubulin, alpha 1b	Homo sapiens
5046	proprotein convertase subtilisin/kexin type 6	Homo sapiens
144165	prickle homolog 1 (Drosophila)	Homo sapiens
10360	nucleophosmin/nucleoplasmin, 3	Homo sapiens
7422	vascular endothelial growth factor A	Homo sapiens
27240	signaling threshold regulating transmembrane adaptor 1	Homo sapiens
1063	centromere protein F, 350/400ka (mitosin)	Homo sapiens
5832	aldehyde dehydrogenase 18 family, member A1	Homo sapiens
93	activin A receptor, type IIB	Homo sapiens
9532	BCL2-associated athanogene 2	Homo sapiens
84132	ubiquitin specific peptidase 42	Homo sapiens
160897	G protein-coupled receptor 180	Homo sapiens
4241	antigen p97 (melanoma associated) identified by monoclonal antibodies 133.2 and 96.5	Homo sapiens
27130	inversin	Homo sapiens
79174	cysteine-rich with EGF-like domains 2	Homo sapiens
129080	EMI domain containing 1	Homo sapiens
7468	Wolf-Hirschhorn syndrome candidate 1	Homo sapiens
23186	REST corepressor 1	Homo sapiens
10695	canopy 3 homolog (zebrafish)	Homo sapiens
3008	histone cluster 1, H1e	Homo sapiens
9700	extra spindle pole bodies homolog 1 (S. cerevisiae)	Homo sapiens
1852	dual specificity phosphatase 9	Homo sapiens
875	cystathionine-beta-synthase	Homo sapiens
79815	NIPA-like domain containing 2	Homo sapiens
55214	leprecan-like 1	Homo sapiens
51727	cytidine monophosphate (UMP-CMP) kinase 1, cytosolic	Homo sapiens
196743	polyamine oxidase (exo-N4-amino)	Homo sapiens
4141	methionyl-tRNA synthetase	Homo sapiens
6745	signal sequence receptor, alpha	Homo sapiens
1743	dihydrolipoamide S-succinyltransferase (E2 component of 2-oxo-glutarate complex); dihydrolipoamide S-succinyltransferase pseudogene (E2 component of 2-oxo-glutarate complex)	Homo sapiens
1744	dihydrolipoamide S-succinyltransferase (E2 component of 2-oxo-glutarate complex); dihydrolipoamide S-succinyltransferase pseudogene (E2 component of 2-oxo-glutarate complex)	Homo sapiens
89884	LIM homeobox 4	Homo sapiens
146223	CKLF-like MARVEL transmembrane domain containing 4	Homo sapiens
7157	tumor protein p53	Homo sapiens
79641	rogdi homolog (Drosophila)	Homo sapiens
51524	transmembrane protein 138	Homo sapiens
100131609	heterogeneous nuclear ribonucleoprotein A1-like 3; similar to heterogeneous nuclear ribonucleoprotein A1; heterogeneous nuclear ribonucleoprotein A1 pseudogene 2; heterogeneous nuclear ribonucleoprotein A1; heterogeneous nuclear ribonucleoprotein A1 pseudogene	Homo sapiens

728643	heterogeneous nuclear ribonucleoprotein A1-like 3; similar to heterogeneous nuclear ribonucleoprotein A1; heterogeneous nuclear ribonucleoprotein A1 pseudogene 2; heterogeneous nuclear ribonucleoprotein A1; heterogeneous nuclear ribonucleoprotein A1 pseudogene	Homo sapiens
388275	heterogeneous nuclear ribonucleoprotein A1-like 3; similar to heterogeneous nuclear ribonucleoprotein A1; heterogeneous nuclear ribonucleoprotein A1 pseudogene 2; heterogeneous nuclear ribonucleoprotein A1; heterogeneous nuclear ribonucleoprotein A1 pseudogene	Homo sapiens
664709	heterogeneous nuclear ribonucleoprotein A1-like 3; similar to heterogeneous nuclear ribonucleoprotein A1; heterogeneous nuclear ribonucleoprotein A1 pseudogene 2; heterogeneous nuclear ribonucleoprotein A1; heterogeneous nuclear ribonucleoprotein A1 pseudogene	Homo sapiens
3178	heterogeneous nuclear ribonucleoprotein A1-like 3; similar to heterogeneous nuclear ribonucleoprotein A1; heterogeneous nuclear ribonucleoprotein A1 pseudogene 2; heterogeneous nuclear ribonucleoprotein A1; heterogeneous nuclear ribonucleoprotein A1 pseudogene	Homo sapiens
645691	heterogeneous nuclear ribonucleoprotein A1-like 3; similar to heterogeneous nuclear ribonucleoprotein A1; heterogeneous nuclear ribonucleoprotein A1 pseudogene 2; heterogeneous nuclear ribonucleoprotein A1; heterogeneous nuclear ribonucleoprotein A1 pseudogene	Homo sapiens
644037	heterogeneous nuclear ribonucleoprotein A1-like 3; similar to heterogeneous nuclear ribonucleoprotein A1; heterogeneous nuclear ribonucleoprotein A1 pseudogene 2; heterogeneous nuclear ribonucleoprotein A1; heterogeneous nuclear ribonucleoprotein A1 pseudogene	Homo sapiens
51322	WW domain containing adaptor with coiled-coil	Homo sapiens
146542	zinc finger protein 688; zinc finger protein 785	Homo sapiens
146540	zinc finger protein 688; zinc finger protein 785	Homo sapiens
26959	HMG-box transcription factor 1	Homo sapiens
5166	pyruvate dehydrogenase kinase, isozyme 4	Homo sapiens
388698	filaggrin family member 2	Homo sapiens
80856	KIAA1715	Homo sapiens
4440	musashi homolog 1 (Drosophila)	Homo sapiens
3559	interleukin 2 receptor, alpha	Homo sapiens
347734	solute carrier family 35, member B2	Homo sapiens
64858	DNA cross-link repair 1B (PSO2 homolog, S. cerevisiae)	Homo sapiens
116841	synaptosomal-associated protein, 47kDa	Homo sapiens
79628	SH3 domain and tetratricopeptide repeats 2	Homo sapiens
219855	solute carrier family 37 (glycerol-3-phosphate transporter), member 2	Homo sapiens
10768	adenosylhomocysteinase-like 1	Homo sapiens
128977	chromosome 22 open reading frame 39	Homo sapiens
23657	solute carrier family 7, (cationic amino acid transporter, y+ system) member 11	Homo sapiens
9939	RNA binding motif protein 8A	Homo sapiens
100130446	ribosomal protein S27a pseudogene 12; ribosomal protein S27a; ribosomal protein S27a pseudogene 11; ribosomal protein S27a pseudogene 16	Homo sapiens
728590	ribosomal protein S27a pseudogene 12; ribosomal protein S27a; ribosomal protein S27a pseudogene 11; ribosomal protein S27a pseudogene 16	Homo sapiens
6233	ribosomal protein S27a pseudogene 12; ribosomal protein S27a; ribosomal protein S27a pseudogene 11; ribosomal protein S27a pseudogene 16	Homo sapiens
643358	ribosomal protein S27a pseudogene 12; ribosomal protein S27a; ribosomal protein S27a pseudogene 11; ribosomal protein S27a pseudogene 16	Homo sapiens
100128056	similar to hCG2013701; CTD (carboxy-terminal domain, RNA polymerase II, polypeptide A) small phosphatase 2	Homo sapiens
10106	similar to hCG2013701; CTD (carboxy-terminal domain, RNA polymerase II, polypeptide A) small phosphatase 2	Homo sapiens
1557	cytochrome P450, family 2, subfamily C, polypeptide 19	Homo sapiens
5509	protein phosphatase 1, regulatory (inhibitor) subunit 3D	Homo sapiens
5638	proline rich Gla (G-carboxyglutamic acid) 1	Homo sapiens
283349	Ras association (RalGDS/AF-6) domain family member 3	Homo sapiens
54933	rhomboid, veinlet-like 2 (Drosophila)	Homo sapiens
54014	bromodomain and WD repeat domain containing 1	Homo sapiens
9739	SET domain containing 1A	Homo sapiens
718	similar to Complement C3 precursor; complement component 3; hypothetical protein LOC100133511	Homo sapiens
653879	similar to Complement C3 precursor; complement component 3; hypothetical protein LOC100133511	Homo sapiens
100133511	similar to Complement C3 precursor; complement component 3; hypothetical protein LOC100133511	Homo sapiens
8295	transformation/transcription domain-associated protein	Homo sapiens
55071	chromosome 9 open reading frame 40	Homo sapiens
255057	chromosome 19 open reading frame 26	Homo sapiens
8526	diacylglycerol kinase, epsilon 64kDa	Homo sapiens
10666	CD226 molecule	Homo sapiens
3192	heterogeneous nuclear ribonucleoprotein U (scaffold attachment factor A)	Homo sapiens
10298	p21 protein (Cdc42/Rac)-activated kinase 4	Homo sapiens
57505	alanyl-tRNA synthetase 2, mitochondrial (putative)	Homo sapiens
55140	elongation protein 3 homolog (S. cerevisiae)	Homo sapiens
2149	coagulation factor II (thrombin) receptor	Homo sapiens
83939	eukaryotic translation initiation factor 2A, 65kDa	Homo sapiens
26118	WD repeat and SOCS box-containing 1	Homo sapiens

11052	cleavage and polyadenylation specific factor 6, 68kDa	Homo sapiens
59283	calcium channel, voltage-dependent, gamma subunit 8	Homo sapiens
22856	chondroitin sulfate synthase 1	Homo sapiens
54910	sema domain, immunoglobulin domain (Ig), transmembrane domain (TM) and short cytoplasmic domain, (semaphorin) 4C	Homo sapiens
23326	ubiquitin specific peptidase 22	Homo sapiens
100131463	similar to translocase of outer mitochondrial membrane 20 homolog; similar to mitochondrial outer membrane protein 19; translocase of outer mitochondrial membrane 20 homolog (yeast)	Homo sapiens
100129272	similar to translocase of outer mitochondrial membrane 20 homolog; similar to mitochondrial outer membrane protein 19; translocase of outer mitochondrial membrane 20 homolog (yeast)	Homo sapiens
9804	similar to translocase of outer mitochondrial membrane 20 homolog; similar to mitochondrial outer membrane protein 19; translocase of outer mitochondrial membrane 20 homolog (yeast)	Homo sapiens
170506	DEAH (Asp-Glu-Ala-His) box polypeptide 36	Homo sapiens
91404	SEC14 and spectrin domains 1	Homo sapiens
84259	DCN1, defective in cullin neddylation 1, domain containing 5 (S. cerevisiae)	Homo sapiens
64093	SPARC related modular calcium binding 1	Homo sapiens
51343	fizzy/cell division cycle 20 related 1 (Drosophila)	Homo sapiens
773	calcium channel, voltage-dependent, P/Q type, alpha 1A subunit	Homo sapiens
8061	FOS-like antigen 1	Homo sapiens
389901	X-ray repair complementing defective repair in Chinese hamster cells 6; similar to ATP-dependent DNA helicase II, 70 kDa subunit	Homo sapiens
2547	X-ray repair complementing defective repair in Chinese hamster cells 6; similar to ATP-dependent DNA helicase II, 70 kDa subunit	Homo sapiens
896	cyclin D3	Homo sapiens
144453	bestrophin 3	Homo sapiens
64760	family with sequence similarity 160, member B2	Homo sapiens
64321	SRY (sex determining region Y)-box 17	Homo sapiens
92285	zinc finger protein 585B	Homo sapiens
5602	mitogen-activated protein kinase 10	Homo sapiens
118788	phosphoinositide-3-kinase adaptor protein 1	Homo sapiens
339230	coiled-coil domain containing 137	Homo sapiens
166348	kelch domain containing 6	Homo sapiens
252839	transmembrane protein 9	Homo sapiens
5362	plexin A2	Homo sapiens
142679	dual specificity phosphatase 19	Homo sapiens
64778	fibronectin type III domain containing 3B	Homo sapiens
59338	pleckstrin homology domain containing, family A (phosphoinositide binding specific) member 1	Homo sapiens
54811	zinc finger protein 562	Homo sapiens
84897	transforming growth factor beta regulator 1	Homo sapiens
5218	PFTAIRE protein kinase 1	Homo sapiens
84901	nuclear factor of activated T-cells, cytoplasmic, calcineurin-dependent 2 interacting protein	Homo sapiens
25940	family with sequence similarity 98, member A	Homo sapiens
149175	mannosidase, endo-alpha-like	Homo sapiens
10618	trans-golgi network protein 2	Homo sapiens
170384	fucosyltransferase 11 (alpha (1,3) fucosyltransferase)	Homo sapiens
11185	indolethylamine N-methyltransferase	Homo sapiens
440026	transmembrane protein 41B	Homo sapiens
54328	G protein-coupled receptor 173	Homo sapiens
26100	WD repeat domain, phosphoinositide interacting 2	Homo sapiens
6642	sorting nexin 1	Homo sapiens
47	ATP citrate lyase	Homo sapiens
23306	transmembrane protein 194A	Homo sapiens
2013	epithelial membrane protein 2	Homo sapiens
115572	family with sequence similarity 46, member B	Homo sapiens
55432	YOD1 OTU deubiquinating enzyme 1 homolog (S. cerevisiae)	Homo sapiens
10614	hexamethylene bis-acetamide inducible 1	Homo sapiens
285359	phosducin-like 3 pseudogene; phosducin-like 3	Homo sapiens
79031	phosducin-like 3 pseudogene; phosducin-like 3	Homo sapiens
64282	PAP associated domain containing 5	Homo sapiens
346389	metastasis associated in colon cancer 1	Homo sapiens
9645	microtubule associated monoxygenase, calponin and LIM domain containing 2	Homo sapiens
2869	G protein-coupled receptor kinase 5	Homo sapiens
7581	zinc finger protein 33A	Homo sapiens
10454	mitogen-activated protein kinase kinase kinase 7 interacting protein 1	Homo sapiens
699	budding uninhibited by benzimidazoles 1 homolog (yeast)	Homo sapiens
10018	BCL2-like 11 (apoptosis facilitator)	Homo sapiens
84671	zinc finger protein 347	Homo sapiens
55558	plexin A3	Homo sapiens
135152	beta-1,3-glucuronyltransferase 2 (glucuronosyltransferase S)	Homo sapiens
4835	NAD(P)H dehydrogenase, quinone 2	Homo sapiens
57179	KIAA1191	Homo sapiens
23761	phosphatidylserine decarboxylase	Homo sapiens
9422	zinc finger protein 264	Homo sapiens
84656	cytokine-like nuclear factor n-pac	Homo sapiens

2354	FBJ murine osteosarcoma viral oncogene homolog B	Homo sapiens
112495	general transcription factor IIIC, polypeptide 6, alpha 35kDa	Homo sapiens
56938	aryl hydrocarbon receptor nuclear translocator-like 2	Homo sapiens
51204	coiled-coil domain containing 44	Homo sapiens
23439	ATPase, (Na+)/K+ transporting, beta 4 polypeptide	Homo sapiens
10196	protein arginine methyltransferase 3	Homo sapiens
138151	NACC family member 2, BEN and BTB (POZ) domain containing	Homo sapiens
203523	zinc finger protein 449	Homo sapiens
5928	hypothetical LOC642954; retinoblastoma binding protein 4	Homo sapiens
642954	hypothetical LOC642954; retinoblastoma binding protein 4	Homo sapiens
54499	transmembrane and coiled-coil domains 1	Homo sapiens
4701	NADH dehydrogenase (ubiquinone) 1 alpha subcomplex, 7, 14.5kDa	Homo sapiens
90990	kinesin family member C2	Homo sapiens
91452	acyl-Coenzyme A binding domain containing 5	Homo sapiens
8329	histone cluster 1, H2ag; histone cluster 1, H2ah; histone cluster 1, H2ai; histone cluster 1, H2ak; histone cluster 1, H2al; histone cluster 1, H2am	Homo sapiens
8330	histone cluster 1, H2ag; histone cluster 1, H2ah; histone cluster 1, H2ai; histone cluster 1, H2ak; histone cluster 1, H2al; histone cluster 1, H2am	Homo sapiens
8332	histone cluster 1, H2ag; histone cluster 1, H2ah; histone cluster 1, H2ai; histone cluster 1, H2ak; histone cluster 1, H2al; histone cluster 1, H2am	Homo sapiens
8336	histone cluster 1, H2ag; histone cluster 1, H2ah; histone cluster 1, H2ai; histone cluster 1, H2ak; histone cluster 1, H2al; histone cluster 1, H2am	Homo sapiens
8969	histone cluster 1, H2ag; histone cluster 1, H2ah; histone cluster 1, H2ai; histone cluster 1, H2ak; histone cluster 1, H2al; histone cluster 1, H2am	Homo sapiens
85235	histone cluster 1, H2ag; histone cluster 1, H2ah; histone cluster 1, H2ai; histone cluster 1, H2ak; histone cluster 1, H2al; histone cluster 1, H2am	Homo sapiens
599	BCL2-like 2	Homo sapiens
6749	structure specific recognition protein 1	Homo sapiens
80301	pleckstrin homology domain containing, family O member 2	Homo sapiens
833	cysteinyl-tRNA synthetase	Homo sapiens
124995	mitochondrial ribosomal protein L10	Homo sapiens
7490	Wilms tumor 1	Homo sapiens
54557	small glutamine-rich tetratricopeptide repeat (TPR)-containing, beta	Homo sapiens
5867	RAB4A, member RAS oncogene family	Homo sapiens
7798	leucine zipper protein 1	Homo sapiens
7458	eukaryotic translation initiation factor 4H	Homo sapiens
3340	N-deacetylase/N-sulfotransferase (heparan glucosaminyl) 1	Homo sapiens
6794	serine/threonine kinase 11	Homo sapiens
2037	erythrocyte membrane protein band 4.1-like 2	Homo sapiens
11138	TBC1 domain family, member 8 (with GRAM domain)	Homo sapiens
9464	heart and neural crest derivatives expressed 2	Homo sapiens
51128	SAR1 homolog B (S. cerevisiae)	Homo sapiens
8795	tumor necrosis factor receptor superfamily, member 10b	Homo sapiens
3717	Janus kinase 2	Homo sapiens
84439	HHIP-like 1	Homo sapiens
9969	mediator complex subunit 13	Homo sapiens
391104	von Hippel-Lindau tumor suppressor-like	Homo sapiens
84914	zinc finger protein 587	Homo sapiens
353376	transmembrane emp24 protein transport domain containing 7; toll-like receptor adaptor molecule 2	Homo sapiens
51014	transmembrane emp24 protein transport domain containing 7; toll-like receptor adaptor molecule 2	Homo sapiens
51071	2-deoxyribose-5-phosphate aldolase homolog (C. elegans)	Homo sapiens
493829	tripartite motif-containing 72	Homo sapiens
154141	membrane bound O-acyltransferase domain containing 1	Homo sapiens
10019	SH2B adaptor protein 3	Homo sapiens
4150	MYC-associated zinc finger protein (purine-binding transcription factor)	Homo sapiens
27005	ubiquitin specific peptidase 21	Homo sapiens
79744	zinc finger protein 419	Homo sapiens
11222	mitochondrial ribosomal protein L3	Homo sapiens
401409	RAB19, member RAS oncogene family	Homo sapiens
10040	target of myb1 (chicken)-like 1	Homo sapiens
55690	phosphofurin acidic cluster sorting protein 1	Homo sapiens
284391	zinc finger protein 844	Homo sapiens
6498	SKI-like oncogene	Homo sapiens
54464	5'-3' exoribonuclease 1	Homo sapiens
5191	peroxisomal biogenesis factor 7	Homo sapiens
2745	glutaredoxin (thioltransferase)	Homo sapiens
134218	DnaJ (Hsp40) homolog, subfamily C, member 21	Homo sapiens
653881	ribosomal protein L3; similar to 60S ribosomal protein L3 (L4)	Homo sapiens
6122	ribosomal protein L3; similar to 60S ribosomal protein L3 (L4)	Homo sapiens
490	ATPase, Ca++ transporting, plasma membrane 1	Homo sapiens
51228	glycolipid transfer protein; glycolipid transfer protein pseudogene 1	Homo sapiens
645312	glycolipid transfer protein; glycolipid transfer protein pseudogene 1	Homo sapiens
6404	selectin P ligand	Homo sapiens
55617	taspase, threonine aspartase, 1	Homo sapiens
55149	mitochondrial poly(A) polymerase	Homo sapiens
54332	ganglioside-induced differentiation-associated protein 1	Homo sapiens
65082	vacuolar protein sorting 33 homolog A (S. cerevisiae)	Homo sapiens

255027	MPV17 mitochondrial membrane protein-like	Homo sapiens
64225	atlastin GTPase 2	Homo sapiens
64061	TSPY-like 2	Homo sapiens
56140	protocadherin alpha 8; protocadherin alpha 6	Homo sapiens
56142	protocadherin alpha 8; protocadherin alpha 6	Homo sapiens
170961	ankyrin repeat domain 24	Homo sapiens
1678	translocase of inner mitochondrial membrane 8 homolog A (yeast)	Homo sapiens
540	ATPase, Cu++ transporting, beta polypeptide	Homo sapiens
8602	NOP14 nucleolar protein homolog (yeast)	Homo sapiens
23288	IQ motif containing E	Homo sapiens
123606	non imprinted in Prader-Willi/Angelman syndrome 1	Homo sapiens
55762	zinc finger protein 701	Homo sapiens
254251	ligand dependent nuclear receptor corepressor-like	Homo sapiens
55871	COBW domain containing 6; COBW domain containing 1	Homo sapiens
644019	COBW domain containing 6; COBW domain containing 1	Homo sapiens
10969	EBNA1 binding protein 2	Homo sapiens
150094	salt-inducible kinase 1	Homo sapiens
10509	sema domain, immunoglobulin domain (Ig), transmembrane domain (TM) and short cytoplasmic domain, (semaphorin) 4B	Homo sapiens
54913	ribonuclease P/MRP 25kDa subunit	Homo sapiens
9859	centrosomal protein 170kDa	Homo sapiens
8833	guanine monphosphate synthetase	Homo sapiens
84899	transmembrane and tetratricopeptide repeat containing 4	Homo sapiens
28960	decapping enzyme, scavenger	Homo sapiens
129787	transmembrane protein 18	Homo sapiens
284161	glycerophosphodiester phosphodiesterase domain containing 1	Homo sapiens
57556	sema domain, transmembrane domain (TM), and cytoplasmic domain, (semaphorin) 6A	Homo sapiens
25896	integrator complex subunit 7	Homo sapiens
23295	mahogunin, ring finger 1	Homo sapiens
8851	cyclin-dependent kinase 5, regulatory subunit 1 (p35)	Homo sapiens
7039	transforming growth factor, alpha	Homo sapiens
11276	AP1 gamma subunit binding protein 1	Homo sapiens
6920	transcription elongation factor A (SII), 3	Homo sapiens
5268	serpin peptidase inhibitor, clade B (ovalbumin), member 5	Homo sapiens
347688	tubulin, beta 8	Homo sapiens
51341	zinc finger and BTB domain containing 7A	Homo sapiens
1834	dentin sialophosphoprotein	Homo sapiens
6241	ribonucleotide reductase M2 polypeptide	Homo sapiens
57531	HECT domain and ankyrin repeat containing, E3 ubiquitin protein ligase 1	Homo sapiens
2549	GRB2-associated binding protein 1	Homo sapiens
9815	G protein-coupled receptor kinase interacting ArfGAP 2	Homo sapiens
1399	v-crk sarcoma virus CT10 oncogene homolog (avian)-like	Homo sapiens
27020	neuroplastin	Homo sapiens
91662	NLR family, pyrin domain containing 12	Homo sapiens
339416	ankyrin repeat domain 45	Homo sapiens
255488	ring finger protein 144B	Homo sapiens
79786	kelch-like 36 (Drosophila)	Homo sapiens
11237	ring finger protein 24	Homo sapiens
58487	CREB/ATF bZIP transcription factor	Homo sapiens
7516	X-ray repair complementing defective repair in Chinese hamster cells 2	Homo sapiens
286451	Yip1 domain family, member 6	Homo sapiens
55692	LUC7-like (S. cerevisiae)	Homo sapiens
8799	peroxisomal biogenesis factor 11 beta	Homo sapiens
10055	SUMO1 activating enzyme subunit 1	Homo sapiens
6628	small nuclear ribonucleoprotein polypeptides B and B1	Homo sapiens
4500	metallothionein 1L (gene/pseudogene); metallothionein 1E; metallothionein 1 pseudogene 3; metallothionein 1J (pseudogene)	Homo sapiens
4498	metallothionein 1L (gene/pseudogene); metallothionein 1E; metallothionein 1 pseudogene 3; metallothionein 1J (pseudogene)	Homo sapiens
4493	metallothionein 1L (gene/pseudogene); metallothionein 1E; metallothionein 1 pseudogene 3; metallothionein 1J (pseudogene)	Homo sapiens
140851	metallothionein 1L (gene/pseudogene); metallothionein 1E; metallothionein 1 pseudogene 3; metallothionein 1J (pseudogene)	Homo sapiens
94241	tumor protein p53 inducible nuclear protein 1	Homo sapiens
1029	cyclin-dependent kinase inhibitor 2A (melanoma, p16, inhibits CDK4)	Homo sapiens
3708	inositol 1,4,5-triphosphate receptor, type 1	Homo sapiens
152815	THAP domain containing 6	Homo sapiens
440590	zyg-11 homolog A (C. elegans)	Homo sapiens
81706	protein phosphatase 1, regulatory (inhibitor) subunit 14C	Homo sapiens
8804	cellular repressor of E1A-stimulated genes 1	Homo sapiens
4524	5,10-methylenetetrahydrofolate reductase (NADPH)	Homo sapiens
10112	kinesin family member 20A	Homo sapiens
202915	transmembrane protein 184A	Homo sapiens
1783	dynein, cytoplasmic 1, light intermediate chain 2	Homo sapiens
23234	DnaJ (Hsp40) homolog, subfamily C, member 9	Homo sapiens
55355	Holliday junction recognition protein	Homo sapiens
253558	lysocardiolipin acyltransferase 1	Homo sapiens
9238	transforming growth factor beta regulator 4	Homo sapiens
9725	transmembrane protein 63A	Homo sapiens

7056	thrombomodulin	Homo sapiens
2717	galactosidase, alpha	Homo sapiens
10094	similar to actin related protein 2/3 complex subunit 3; hypothetical LOC729841; actin related protein 2/3 complex, subunit 3, 21kDa	Homo sapiens
729841	similar to actin related protein 2/3 complex subunit 3; hypothetical LOC729841; actin related protein 2/3 complex, subunit 3, 21kDa	Homo sapiens
729494	similar to actin related protein 2/3 complex subunit 3; hypothetical LOC729841; actin related protein 2/3 complex, subunit 3, 21kDa	Homo sapiens
80306	mediator complex subunit 28	Homo sapiens
23309	SIN3 homolog B, transcription regulator (yeast)	Homo sapiens
55591	vezatin, adherens junctions transmembrane protein	Homo sapiens
440093	histone H3-like	Homo sapiens
10923	SUB1 homolog (S. cerevisiae)	Homo sapiens
3202	homeobox A5	Homo sapiens
10162	lysophosphatidylcholine acyltransferase 3	Homo sapiens
4601	MAX interactor 1	Homo sapiens
85313	peptidylprolyl isomerase (cyclophilin)-like 4	Homo sapiens
7565	zinc finger protein 17	Homo sapiens
51747	cisplatin resistance-associated overexpressed protein	Homo sapiens
55017	chromosome 14 open reading frame 119	Homo sapiens
10188	tyrosine kinase, non-receptor, 2	Homo sapiens
79875	thrombospondin, type I, domain containing 4	Homo sapiens
3547	immunoglobulin superfamily, member 1	Homo sapiens
56143	protocadherin alpha 5	Homo sapiens
57595	PDZ domain containing 4	Homo sapiens
9056	solute carrier family 7 (cationic amino acid transporter, y+ system), member 7	Homo sapiens
55161	transmembrane protein 33	Homo sapiens
8492	protease, serine, 12 (neurotrypsin, motopsin)	Homo sapiens
83667	sestrin 2	Homo sapiens
10985	GCN1 general control of amino-acid synthesis 1-like 1 (yeast)	Homo sapiens
23243	ankyrin repeat domain 28	Homo sapiens
140612	zinc finger protein 28 homolog (mouse)	Homo sapiens
55280	CWF19-like 1, cell cycle control (S. pombe)	Homo sapiens
23067	SET domain containing 1B	Homo sapiens
2280	FK506 binding protein 1A, 12kDa	Homo sapiens
9314	Kruppel-like factor 4 (gut)	Homo sapiens
171023	additional sex combs like 1 (Drosophila)	Homo sapiens
339834	coiled-coil domain containing 36	Homo sapiens
11215	A kinase (PRKA) anchor protein 11	Homo sapiens
51728	polymerase (RNA) III (DNA directed) polypeptide K, 12.3 kDa	Homo sapiens
64210	MMS19 nucleotide excision repair homolog (S. cerevisiae)	Homo sapiens
9741	lysosomal protein transmembrane 4 alpha	Homo sapiens
84858	zinc finger protein 503	Homo sapiens
2057	erythropoietin receptor	Homo sapiens
376693	ribosomal protein S10; ribosomal protein S10 pseudogene 4; ribosomal protein S10 pseudogene 11; ribosomal protein S10 pseudogene 22; ribosomal protein S10 pseudogene 7; ribosomal protein S10 pseudogene 13	Homo sapiens
401817	ribosomal protein S10; ribosomal protein S10 pseudogene 4; ribosomal protein S10 pseudogene 11; ribosomal protein S10 pseudogene 22; ribosomal protein S10 pseudogene 7; ribosomal protein S10 pseudogene 13	Homo sapiens
6204	ribosomal protein S10; ribosomal protein S10 pseudogene 4; ribosomal protein S10 pseudogene 11; ribosomal protein S10 pseudogene 22; ribosomal protein S10 pseudogene 7; ribosomal protein S10 pseudogene 13	Homo sapiens
728791	ribosomal protein S10; ribosomal protein S10 pseudogene 4; ribosomal protein S10 pseudogene 11; ribosomal protein S10 pseudogene 22; ribosomal protein S10 pseudogene 7; ribosomal protein S10 pseudogene 13	Homo sapiens
391833	ribosomal protein S10; ribosomal protein S10 pseudogene 4; ribosomal protein S10 pseudogene 11; ribosomal protein S10 pseudogene 22; ribosomal protein S10 pseudogene 7; ribosomal protein S10 pseudogene 13	Homo sapiens
646785	ribosomal protein S10; ribosomal protein S10 pseudogene 4; ribosomal protein S10 pseudogene 11; ribosomal protein S10 pseudogene 22; ribosomal protein S10 pseudogene 7; ribosomal protein S10 pseudogene 13	Homo sapiens
11165	nudix (nucleoside diphosphate linked moiety X)-type motif 3	Homo sapiens
10914	poly(A) polymerase alpha	Homo sapiens
387263	chromosome 6 open reading frame 120	Homo sapiens
1994	ELAV (embryonic lethal, abnormal vision, Drosophila)-like 1 (Hu antigen R)	Homo sapiens
253959	GTPase activating Rap/RanGAP domain-like 1	Homo sapiens
6792	cyclin-dependent kinase-like 5	Homo sapiens
79654	HECT domain containing 3	Homo sapiens
79728	partner and localizer of BRCA2	Homo sapiens
80349	WD repeat domain 61	Homo sapiens
29079	mediator complex subunit 4	Homo sapiens
26003	golgi reassembly stacking protein 2, 55kDa	Homo sapiens
781	calcium channel, voltage-dependent, alpha 2/delta subunit 1	Homo sapiens
79719	alpha- and gamma-adaptin-binding protein p34	Homo sapiens
5428	polymerase (DNA directed), gamma	Homo sapiens
55632	G2/M-phase specific E3 ubiquitin ligase	Homo sapiens
374875	hydroxysteroid (11-beta) dehydrogenase 1-like	Homo sapiens
7064	thimet oligopeptidase 1	Homo sapiens

7334	ubiquitin-conjugating enzyme E2N (UBC13 homolog, yeast)	Homo sapiens
9873	FCH and double SH3 domains 2	Homo sapiens
3833	kinesin family member C1	Homo sapiens
201255	leucine rich repeat containing 45	Homo sapiens
643300	heat shock 60kDa protein 1 (chaperonin) pseudogene 5; heat shock 60kDa protein 1 (chaperonin) pseudogene 6; heat shock 60kDa protein 1 (chaperonin) pseudogene 1; heat shock 60kDa protein 1 (chaperonin) pseudogene 4; heat shock 60kDa protein 1 (chaperonin)	Homo sapiens
3329	heat shock 60kDa protein 1 (chaperonin) pseudogene 5; heat shock 60kDa protein 1 (chaperonin) pseudogene 6; heat shock 60kDa protein 1 (chaperonin) pseudogene 1; heat shock 60kDa protein 1 (chaperonin) pseudogene 4; heat shock 60kDa protein 1 (chaperonin)	Homo sapiens
345041	heat shock 60kDa protein 1 (chaperonin) pseudogene 5; heat shock 60kDa protein 1 (chaperonin) pseudogene 6; heat shock 60kDa protein 1 (chaperonin) pseudogene 1; heat shock 60kDa protein 1 (chaperonin) pseudogene 4; heat shock 60kDa protein 1 (chaperonin)	Homo sapiens
645548	heat shock 60kDa protein 1 (chaperonin) pseudogene 5; heat shock 60kDa protein 1 (chaperonin) pseudogene 6; heat shock 60kDa protein 1 (chaperonin) pseudogene 1; heat shock 60kDa protein 1 (chaperonin) pseudogene 4; heat shock 60kDa protein 1 (chaperonin)	Homo sapiens
644745	heat shock 60kDa protein 1 (chaperonin) pseudogene 5; heat shock 60kDa protein 1 (chaperonin) pseudogene 6; heat shock 60kDa protein 1 (chaperonin) pseudogene 1; heat shock 60kDa protein 1 (chaperonin) pseudogene 4; heat shock 60kDa protein 1 (chaperonin)	Homo sapiens
710	serpin peptidase inhibitor, clade G (C1 inhibitor), member 1	Homo sapiens
23637	RAB GTPase activating protein 1	Homo sapiens
11057	abhydrolase domain containing 2	Homo sapiens
326625	methylmalonic aciduria (cobalamin deficiency) cblB type	Homo sapiens
4643	myosin IE	Homo sapiens
55143	cell division cycle associated 8	Homo sapiens
2069	epiregulin	Homo sapiens
9868	translocase of outer mitochondrial membrane 70 homolog A (S. cerevisiae)	Homo sapiens
5610	eukaryotic translation initiation factor 2-alpha kinase 2	Homo sapiens
643836	zinc finger protein 62 homolog (mouse)	Homo sapiens
58191	chemokine (C-X-C motif) ligand 16	Homo sapiens
148789	beta-1,3-N-acetylgalactosaminyltransferase 2	Homo sapiens
29928	translocase of inner mitochondrial membrane 22 homolog (yeast)	Homo sapiens
11260	exportin, tRNA (nuclear export receptor for tRNAs); similar to Exportin-T (tRNA exportin) (Exportin(tRNA))	Homo sapiens
441228	exportin, tRNA (nuclear export receptor for tRNAs); similar to Exportin-T (tRNA exportin) (Exportin(tRNA))	Homo sapiens
89874	solute carrier family 25 (mitochondrial oxodicarboxylate carrier), member 21	Homo sapiens
143187	vesicle transport through interaction with t-SNAREs homolog 1A (yeast)	Homo sapiens
442113	protein tyrosine phosphatase, non-receptor type 11; similar to protein tyrosine phosphatase, non-receptor type 11	Homo sapiens
5781	protein tyrosine phosphatase, non-receptor type 11; similar to protein tyrosine phosphatase, non-receptor type 11	Homo sapiens
344593	protein tyrosine phosphatase, non-receptor type 11; similar to protein tyrosine phosphatase, non-receptor type 11	Homo sapiens
55716	limb region 1 homolog (mouse)-like	Homo sapiens
9064	mitogen-activated protein kinase kinase kinase 6	Homo sapiens
54980	chromosome 2 open reading frame 42	Homo sapiens
80179	myosin XIX	Homo sapiens
10425	ariadne homolog 2 (Drosophila)	Homo sapiens
7188	TNF receptor-associated factor 5	Homo sapiens
56146	protocadherin alpha 2	Homo sapiens
170589	glycoprotein hormone alpha 2	Homo sapiens
23232	TBC1 domain family, member 12	Homo sapiens
199857	asparagine-linked glycosylation 14 homolog (S. cerevisiae)	Homo sapiens
55775	tyrosyl-DNA phosphodiesterase 1	Homo sapiens
9049	aryl hydrocarbon receptor interacting protein	Homo sapiens
2683	UDP-Gal:betaGlcNAc beta 1,4- galactosyltransferase, polypeptide 1	Homo sapiens
5609	mitogen-activated protein kinase kinase 7	Homo sapiens
9592	immediate early response 2	Homo sapiens
4884	neuronal pentraxin I	Homo sapiens
2029	endosulfine alpha	Homo sapiens
657	bone morphogenetic protein receptor, type IA; similar to ALK-3	Homo sapiens
643778	bone morphogenetic protein receptor, type IA; similar to ALK-3	Homo sapiens
84520	chromosome 14 open reading frame 142	Homo sapiens
55922	NFKB repressing factor	Homo sapiens
3897	L1 cell adhesion molecule	Homo sapiens
7009	transmembrane BAX inhibitor motif containing 6	Homo sapiens
7057	thrombospondin 1	Homo sapiens
10479	solute carrier family 9 (sodium/hydrogen exchanger), member 6	Homo sapiens
7411	von Hippel-Lindau binding protein 1	Homo sapiens
646791	similar to Acidic leucine-rich nuclear phosphoprotein 32 family member B (PHAPI2 protein) (Silver-stainable protein SSP29) (Acidic protein rich in leucines); acidic (leucine-rich) nuclear phosphoprotein 32 family, member B	Homo sapiens
10541	similar to Acidic leucine-rich nuclear phosphoprotein 32 family member B (PHAPI2 protein) (Silver-stainable protein SSP29) (Acidic protein rich in leucines); acidic (leucine-rich) nuclear phosphoprotein 32 family, member B	Homo sapiens
79050	nucleolar complex associated 4 homolog (S. cerevisiae)	Homo sapiens
64115	chromosome 10 open reading frame 54	Homo sapiens
84311	mitochondrial ribosomal protein L45	Homo sapiens

83445	germ cell associated 1	Homo sapiens
134266	GrpE-like 2, mitochondrial (E. coli)	Homo sapiens
7027	transcription factor Dp-1	Homo sapiens
1389	cAMP responsive element binding protein-like 2	Homo sapiens
5139	phosphodiesterase 3A, cGMP-inhibited	Homo sapiens
56033	BARX homeobox 1	Homo sapiens
4351	mannose phosphate isomerase	Homo sapiens
10483	Sec23 homolog B (S. cerevisiae)	Homo sapiens
7058	thrombospondin 2	Homo sapiens
55596	zinc finger, CCHC domain containing 8	Homo sapiens
3267	ArfGAP with FG repeats 1	Homo sapiens
3418	isocitrate dehydrogenase 2 (NADP+), mitochondrial	Homo sapiens
10947	adaptor-related protein complex 3, mu 2 subunit	Homo sapiens
23013	spen homolog, transcriptional regulator (Drosophila)	Homo sapiens
57515	serine incorporator 1	Homo sapiens
23019	CCR4-NOT transcription complex, subunit 1	Homo sapiens
79931	TNFAIP3 interacting protein 3	Homo sapiens
387921	NHL repeat containing 3	Homo sapiens
160418	transmembrane and tetratricopeptide repeat containing 3	Homo sapiens
9567	GTP binding protein 1	Homo sapiens
124923	uncharacterized serine/threonine-protein kinase SgK494	Homo sapiens
147007	transmembrane protein 199	Homo sapiens
10965	acyl-CoA thioesterase 2	Homo sapiens
26160	intraflagellar transport 172 homolog (Chlamydomonas)	Homo sapiens
23080	AVL9 homolog (S. cerevisiase)	Homo sapiens
57533	TBC1 domain family, member 14	Homo sapiens
283	angiogenin, ribonuclease, RNase A family, 5	Homo sapiens
27348	torsin family 1, member B (torsin B)	Homo sapiens
84068	solute carrier family 10 (sodium/bile acid cotransporter family), member 7	Homo sapiens
54700	RRN3 RNA polymerase I transcription factor homolog (S. cerevisiae)	Homo sapiens
115209	OMA1 homolog, zinc metallopeptidase (S. cerevisiae)	Homo sapiens
4725	similar to NADH dehydrogenase (ubiquinone) Fe-S protein 5, 15kDa (NADH-coenzyme Q reductase); NADH dehydrogenase (ubiquinone) Fe-S protein 5, 15kDa (NADH-coenzyme Q reductase)	Homo sapiens
100130794	similar to NADH dehydrogenase (ubiquinone) Fe-S protein 5, 15kDa (NADH-coenzyme Q reductase); NADH dehydrogenase (ubiquinone) Fe-S protein 5, 15kDa (NADH-coenzyme Q reductase)	Homo sapiens
10671	dynactin 6	Homo sapiens
7539	zinc finger protein 37 homolog (mouse)	Homo sapiens
221504	zinc finger and BTB domain containing 9	Homo sapiens
11016	activating transcription factor 7	Homo sapiens
158158	RAS and EF-hand domain containing	Homo sapiens
23185	La ribonucleoprotein domain family, member 4B	Homo sapiens
100130009	hypothetical LOC100130009; high mobility group AT-hook 1	Homo sapiens
3159	hypothetical LOC100130009; high mobility group AT-hook 1	Homo sapiens
84838	zinc finger protein 496	Homo sapiens
29763	protein kinase C and casein kinase substrate in neurons 3	Homo sapiens
84957	RELT tumor necrosis factor receptor	Homo sapiens
9754	StAR-related lipid transfer (START) domain containing 8	Homo sapiens
9088	protein kinase, membrane associated tyrosine/threonine 1	Homo sapiens
9032	transmembrane 4 L six family member 5	Homo sapiens
57479	proline rich 12	Homo sapiens
57555	neuroligin 2	Homo sapiens
84859	leucine-rich repeats and calponin homology (CH) domain containing 3	Homo sapiens
57691	KIAA1586	Homo sapiens
23129	plexin D1	Homo sapiens
7105	tetraspanin 6	Homo sapiens
51621	Kruppel-like factor 13	Homo sapiens
27069	growth hormone inducible transmembrane protein	Homo sapiens
9265	cytohesin 3	Homo sapiens
84337	elongation factor 1 homolog (S. cerevisiae)	Homo sapiens
27112	family with sequence similarity 155, member B	Homo sapiens
51155	hematological and neurological expressed 1	Homo sapiens
219988	protein associated with topoisomerase II homolog 1 (yeast)	Homo sapiens
345193	leucine-rich repeat, immunoglobulin-like and transmembrane domains 3	Homo sapiens
126259	transmembrane and immunoglobulin domain containing 2	Homo sapiens
3094	histidine triad nucleotide binding protein 1	Homo sapiens
7169	tropomyosin 2 (beta)	Homo sapiens
81502	histocompatibility (minor) 13	Homo sapiens
3017	histone cluster 1, H2bd	Homo sapiens
2114	v-ets erythroblastosis virus E26 oncogene homolog 2 (avian)	Homo sapiens
2950	glutathione S-transferase pi 1	Homo sapiens
9139	core-binding factor, runt domain, alpha subunit 2; translocated to, 2	Homo sapiens
9633	metallothionein-like 5, testis-specific (tesmin)	Homo sapiens
2764	glia maturation factor, beta	Homo sapiens
727896	cysteine and histidine-rich domain (CHORD)-containing 1; cysteine and histidine-rich domain (CHORD)-containing 1 pseudogene	Homo sapiens
26973	cysteine and histidine-rich domain (CHORD)-containing 1; cysteine and histidine-rich domain (CHORD)-containing 1 pseudogene	Homo sapiens

654364	non-metastatic cells 1, protein (NM23A) expressed in; NME1-NME2 readthrough transcript; non-metastatic cells 2, protein (NM23B) expressed in	Homo sapiens
4830	non-metastatic cells 1, protein (NM23A) expressed in; NME1-NME2 readthrough transcript; non-metastatic cells 2, protein (NM23B) expressed in	Homo sapiens
4831	non-metastatic cells 1, protein (NM23A) expressed in; NME1-NME2 readthrough transcript; non-metastatic cells 2, protein (NM23B) expressed in	Homo sapiens
2771	guanine nucleotide binding protein (G protein), alpha inhibiting activity polypeptide 2	Homo sapiens
727936	glycosyltransferase 8 domain containing 4	Homo sapiens
55577	N-acetylglucosamine kinase	Homo sapiens
388567	zinc finger protein 749	Homo sapiens
8938	BAI1-associated protein 3	Homo sapiens
11161	chromosome 14 open reading frame 1	Homo sapiens
9860	leucine-rich repeats and immunoglobulin-like domains 2	Homo sapiens
10121	ARP1 actin-related protein 1 homolog A, centractin alpha (yeast)	Homo sapiens
60401	ectodysplasin A2 receptor	Homo sapiens
57541	zinc finger protein 398	Homo sapiens
7517	X-ray repair complementing defective repair in Chinese hamster cells 3	Homo sapiens
8165	A kinase (PRKA) anchor protein 1	Homo sapiens
8613	phosphatidic acid phosphatase type 2B	Homo sapiens
23284	latrophilin 3	Homo sapiens
116984	ArfGAP with RhoGAP domain, ankyrin repeat and PH domain 2	Homo sapiens
6728	signal recognition particle 19kDa	Homo sapiens
23460	ATP-binding cassette, sub-family A (ABC1), member 6	Homo sapiens
84253	GTPase activating Rap/RanGAP domain-like 3	Homo sapiens
200933	F-box protein 45	Homo sapiens
10652	YKT6 v-SNARE homolog (S. cerevisiae)	Homo sapiens
26267	F-box protein 10	Homo sapiens
64599	GRB10 interacting GYF protein 1	Homo sapiens
11056	DEAD (Asp-Glu-Ala-Asp) box polypeptide 52	Homo sapiens
127670	transmembrane epididymal protein 1	Homo sapiens
342371	ataxin 1-like	Homo sapiens
8624	proteasome (prosome, macropain) assembly chaperone 1	Homo sapiens
51166	aminoadipate aminotransferase	Homo sapiens
29988	solute carrier family 2 (facilitated glucose transporter), member 8	Homo sapiens
29101	SSU72 RNA polymerase II CTD phosphatase homolog (S. cerevisiae)	Homo sapiens
4091	SMAD family member 6	Homo sapiens
1545	cytochrome P450, family 1, subfamily B, polypeptide 1	Homo sapiens
23174	zinc finger, CCHC domain containing 14	Homo sapiens
158511	chondrosarcoma associated gene 1	Homo sapiens
200312	ring finger protein 215	Homo sapiens
835	caspase 2, apoptosis-related cysteine peptidase	Homo sapiens
93611	F-box protein 44	Homo sapiens
7555	CCHC-type zinc finger, nucleic acid binding protein	Homo sapiens
23406	coactosin-like 1 (Dictyostelium)	Homo sapiens
8411	early endosome antigen 1	Homo sapiens
864	runt-related transcription factor 3	Homo sapiens
64417	chromosome 5 open reading frame 28	Homo sapiens
79939	solute carrier family 35, member E1	Homo sapiens
56647	BRCA2 and CDKN1A interacting protein	Homo sapiens
145482	prostaglandin reductase 2	Homo sapiens
84925	disrupted in renal carcinoma 2	Homo sapiens
51477	inositol-3-phosphate synthase 1	Homo sapiens
5802	protein tyrosine phosphatase, receptor type, S	Homo sapiens
23542	mitogen-activated protein kinase 8 interacting protein 2	Homo sapiens
4774	nuclear factor I/A	Homo sapiens
9882	TBC1 domain family, member 4	Homo sapiens
6256	retinoid X receptor, alpha	Homo sapiens
4858	neuro-oncological ventral antigen 2	Homo sapiens
7425	VGF nerve growth factor inducible	Homo sapiens
64781	ceramide kinase	Homo sapiens
93323	HAUS augmin-like complex, subunit 8	Homo sapiens
25851	tectonin beta-propeller repeat containing 1	Homo sapiens
9921	ring finger protein 10	Homo sapiens
3707	inositol 1,4,5-trisphosphate 3-kinase B	Homo sapiens
140735	dynein, light chain, LC8-type 2	Homo sapiens
7317	ubiquitin-like modifier activating enzyme 1	Homo sapiens
51575	similar to ABT1-associated protein; ESF1, nucleolar pre-rRNA processing protein, homolog (S. cerevisiae)	Homo sapiens
100131254	similar to ABT1-associated protein; ESF1, nucleolar pre-rRNA processing protein, homolog (S. cerevisiae)	Homo sapiens
6253	reticulon 2	Homo sapiens
160857	coiled-coil domain containing 122	Homo sapiens
35	acyl-Coenzyme A dehydrogenase, C-2 to C-3 short chain	Homo sapiens
53354	pantothenate kinase 1	Homo sapiens
80018	chromosome 12 open reading frame 30	Homo sapiens
367	androgen receptor	Homo sapiens
5465	peroxisome proliferator-activated receptor alpha	Homo sapiens
4259	microsomal glutathione S-transferase 3	Homo sapiens
9423	netrin 1	Homo sapiens

4697	NADH dehydrogenase (ubiquinone) 1 alpha subcomplex, 4, 9kDa	Homo sapiens
163351	guanylate binding protein family, member 6	Homo sapiens
8228	patatin-like phospholipase domain containing 4	Homo sapiens
7533	tyrosine 3-monooxygenase/tryptophan 5-monooxygenase activation protein, eta polypeptide	Homo sapiens
171389	NLR family, pyrin domain containing 6	Homo sapiens
84722	proline/serine-rich coiled-coil 1	Homo sapiens
2150	coagulation factor II (thrombin) receptor-like 1	Homo sapiens
55421	chromosome 17 open reading frame 85	Homo sapiens
6228	ribosomal protein S23	Homo sapiens
90850	zinc finger protein 598	Homo sapiens
9219	metastasis associated 1 family, member 2	Homo sapiens
4209	myocyte enhancer factor 2D	Homo sapiens
8826	IQ motif containing GTPase activating protein 1	Homo sapiens
8471	insulin receptor substrate 4	Homo sapiens
729799	SEC14-like 1 (S. cerevisiae); SEC14-like 1 pseudogene	Homo sapiens
6397	SEC14-like 1 (S. cerevisiae); SEC14-like 1 pseudogene	Homo sapiens
401136	similar to RIKEN cDNA 9930019B18 gene	Homo sapiens
1982	eukaryotic translation initiation factor 4 gamma, 2	Homo sapiens
54954	family with sequence similarity 120C	Homo sapiens
122830	N-acetyltransferase 12 (GCN5-related, putative)	Homo sapiens
8535	chromobox homolog 4 (Pc class homolog, Drosophila)	Homo sapiens
28998	mitochondrial ribosomal protein L13	Homo sapiens
9037	sema domain, seven thrombospondin repeats (type 1 and type 1-like), transmembrane domain (TM) and short cytoplasmic domain, (semaphorin) 5A	Homo sapiens
91445	ring finger protein 185	Homo sapiens
382	ADP-ribosylation factor 6	Homo sapiens
9052	G protein-coupled receptor, family C, group 5, member A	Homo sapiens
7494	X-box binding protein 1	Homo sapiens
338645	leucine zipper protein 2	Homo sapiens
94005	phosphatidylinositol glycan anchor biosynthesis, class S	Homo sapiens
54939	COMM domain containing 4	Homo sapiens
79047	potassium channel tetramerisation domain containing 15	Homo sapiens
8892	eukaryotic translation initiation factor 2B, subunit 2 beta, 39kDa	Homo sapiens
120935	coiled-coil domain containing 38	Homo sapiens
389677	RNA binding motif protein 12B	Homo sapiens
348654	Gen homolog 1, endonuclease (Drosophila)	Homo sapiens
51704	G protein-coupled receptor, family C, group 5, member B	Homo sapiens
440730	tripartite motif-containing 67	Homo sapiens
353132	late cornified envelope 1B	Homo sapiens
9530	BCL2-associated athanogene 4	Homo sapiens
3091	hypoxia inducible factor 1, alpha subunit (basic helix-loop-helix transcription factor)	Homo sapiens
55450	calcium/calmodulin-dependent protein kinase II inhibitor 1	Homo sapiens
2028	glutamyl aminopeptidase (aminopeptidase A)	Homo sapiens
23085	ELKS/RAB6-interacting/CAST family member 1	Homo sapiens
488	ATPase, Ca++ transporting, cardiac muscle, slow twitch 2	Homo sapiens
29780	parvin, beta	Homo sapiens
375323	lipoma HMGIC fusion partner-like 4	Homo sapiens
84735	carnosine dipeptidase 1 (metallopeptidase M20 family)	Homo sapiens
728505	WD repeat domain 82 pseudogene 1	Homo sapiens
64777	required for meiotic nuclear division 5 homolog B (S. cerevisiae)	Homo sapiens
55653	breast carcinoma amplified sequence 4	Homo sapiens
28511	NFKB inhibitor interacting Ras-like 2	Homo sapiens
23062	golgi associated, gamma adaptin ear containing, ARF binding protein 2	Homo sapiens
23353	unc-84 homolog A (C. elegans)	Homo sapiens
55544	RNA binding motif protein 38	Homo sapiens
4094	v-maf musculoaponeurotic fibrosarcoma oncogene homolog (avian)	Homo sapiens
11179	zinc finger protein 277	Homo sapiens
4739	neural precursor cell expressed, developmentally down-regulated 9	Homo sapiens
91746	YTH domain containing 1	Homo sapiens
284406	zinc finger protein 82 homolog (mouse)	Homo sapiens
116931	mediator complex subunit 12-like	Homo sapiens
323	amyloid beta (A4) precursor protein-binding, family B, member 2	Homo sapiens
10660	ladybird homeobox 1	Homo sapiens
10523	calcium homeostasis endoplasmic reticulum protein	Homo sapiens
23621	beta-site APP-cleaving enzyme 1	Homo sapiens
6249	CAP-GLY domain containing linker protein 1	Homo sapiens
7520	X-ray repair complementing defective repair in Chinese hamster cells 5 (double-strand-break rejoining)	Homo sapiens
11273	ataxin 2-like	Homo sapiens
51533	PHD finger protein 7	Homo sapiens
83593	Ras association (RalGDS/AF-6) domain family member 5	Homo sapiens
22900	caspase recruitment domain family, member 8	Homo sapiens
55178	RNA methyltransferase like 1	Homo sapiens
23136	erythrocyte membrane protein band 4.1-like 3	Homo sapiens
9055	protein regulator of cytokinesis 1	Homo sapiens
63943	FK506 binding protein like	Homo sapiens
55609	zinc finger protein 280C	Homo sapiens
55636	chromodomain helicase DNA binding protein 7	Homo sapiens

9949	Alport syndrome, mental retardation, midface hypoplasia and elliptocytosis chromosomal region gene 1	Homo sapiens
154214	ring finger protein 217	Homo sapiens
10580	sorbin and SH3 domain containing 1	Homo sapiens
2180	acyl-CoA synthetase long-chain family member 1	Homo sapiens
9919	SEC16 homolog A (S. cerevisiae)	Homo sapiens
83737	itchy E3 ubiquitin protein ligase homolog (mouse)	Homo sapiens
10972	transmembrane emp24-like trafficking protein 10 (yeast)	Homo sapiens
83540	NUF2, NDC80 kinetochore complex component, homolog (S. cerevisiae)	Homo sapiens
23609	makorin ring finger protein 2	Homo sapiens
6319	stearoyl-CoA desaturase (delta-9-desaturase)	Homo sapiens
1942	ephrin-A1	Homo sapiens
729438	GATS protein-like 2	Homo sapiens
26502	nuclear prelamin A recognition factor	Homo sapiens
10983	cyclin I	Homo sapiens
29959	nuclear receptor binding protein 1	Homo sapiens
23534	transportin 3	Homo sapiens
57570	TRM5 tRNA methyltransferase 5 homolog (S. cerevisiae)	Homo sapiens
124590	Usher syndrome 1G (autosomal recessive)	Homo sapiens
2778	GNAS complex locus	Homo sapiens
9242	musculin (activated B-cell factor-1)	Homo sapiens
54796	basonuclin 2	Homo sapiens
7772	zinc finger protein 229	Homo sapiens
10975	ubiquinol-cytochrome c reductase, 6.4kDa subunit	Homo sapiens
10724	meningioma expressed antigen 5 (hyaluronidase)	Homo sapiens
8289	AT rich interactive domain 1A (SWI-like)	Homo sapiens
602	B-cell CLL/lymphoma 3	Homo sapiens
81562	lectin, mannose-binding 2-like	Homo sapiens
153129	solute carrier family 38, member 9	Homo sapiens
29083	GTP-binding protein 8 (putative)	Homo sapiens
4152	methyl-CpG binding domain protein 1	Homo sapiens
55966	adherens junctions associated protein 1	Homo sapiens
7572	zinc finger protein 24	Homo sapiens
51517	NCK interacting protein with SH3 domain	Homo sapiens
285440	cytochrome P450, family 4, subfamily V, polypeptide 2	Homo sapiens
63941	N-terminal EF-hand calcium binding protein 3	Homo sapiens
6526	solute carrier family 5 (sodium/myo-inositol cotransporter), member 3	Homo sapiens
4343	Mov10, Moloney leukemia virus 10, homolog (mouse)	Homo sapiens
3550	similar to CG18005; IK cytokine, down-regulator of HLA II	Homo sapiens
644456	similar to CG18005; IK cytokine, down-regulator of HLA II	Homo sapiens
7739	zinc finger protein 185 (LIM domain)	Homo sapiens
23237	activity-regulated cytoskeleton-associated protein	Homo sapiens
55349	choline dehydrogenase	Homo sapiens
3215	homeobox B5	Homo sapiens
93349	SP140 nuclear body protein-like	Homo sapiens
117145	thioesterase superfamily member 4	Homo sapiens
2644	GTP cyclohydrolase I feedback regulator	Homo sapiens
4193	Mdm2 p53 binding protein homolog (mouse)	Homo sapiens
2475	mechanistic target of rapamycin (serine/threonine kinase)	Homo sapiens
493	ATPase, Ca++ transporting, plasma membrane 4	Homo sapiens
644914	H3 histone, family 3B (H3.3B); H3 histone, family 3A pseudogene; H3 histone, family 3A; similar to H3 histone, family 3B; similar to histone H3.3B	Homo sapiens
3020	H3 histone, family 3B (H3.3B); H3 histone, family 3A pseudogene; H3 histone, family 3A; similar to H3 histone, family 3B; similar to histone H3.3B	Homo sapiens
440926	H3 histone, family 3B (H3.3B); H3 histone, family 3A pseudogene; H3 histone, family 3A; similar to H3 histone, family 3B; similar to histone H3.3B	Homo sapiens
3021	H3 histone, family 3B (H3.3B); H3 histone, family 3A pseudogene; H3 histone, family 3A; similar to H3 histone, family 3B; similar to histone H3.3B	Homo sapiens
728914	H3 histone, family 3B (H3.3B); H3 histone, family 3A pseudogene; H3 histone, family 3A; similar to H3 histone, family 3B; similar to histone H3.3B	Homo sapiens
29934	sorting nexin 12	Homo sapiens
5886	RAD23 homolog A (S. cerevisiae)	Homo sapiens
6602	SWI/SNF related, matrix associated, actin dependent regulator of chromatin, subfamily d, member 1	Homo sapiens
5879	ras-related C3 botulinum toxin substrate 1 (rho family, small GTP binding protein Rac1)	Homo sapiens
54682	MANSC domain containing 1	Homo sapiens
11170	family with sequence similarity 107, member A	Homo sapiens
57677	zinc finger protein 14 homolog (mouse)	Homo sapiens
5862	RAB2A, member RAS oncogene family	Homo sapiens
6184	ribophorin I	Homo sapiens
9715	family with sequence similarity 131, member B	Homo sapiens
5447	P450 (cytochrome) oxidoreductase	Homo sapiens
143244	eukaryotic translation initiation factor 5A; eukaryotic translation initiation factor 5A-like 1	Homo sapiens
1984	eukaryotic translation initiation factor 5A; eukaryotic translation initiation factor 5A-like 1	Homo sapiens
6446	serum/glucocorticoid regulated kinase 1	Homo sapiens
79856	sorting nexin 22	Homo sapiens
26136	testis derived transcript (3 LIM domains)	Homo sapiens

118	adducin 1 (alpha)	Homo sapiens
165324	UBX domain protein 2A	Homo sapiens
55106	schlafen family member 12	Homo sapiens
6230	ribosomal protein S25 pseudogene 8; ribosomal protein S25	Homo sapiens
100131196	ribosomal protein S25 pseudogene 8; ribosomal protein S25	Homo sapiens
5167	ectonucleotide pyrophosphatase/phosphodiesterase 1	Homo sapiens
79847	transmembrane protein 180	Homo sapiens
5754	PTK7 protein tyrosine kinase 7	Homo sapiens
23093	tubulin tyrosine ligase-like family, member 5	Homo sapiens
255324	epithelial mitogen homolog (mouse)	Homo sapiens
23261	calmodulin binding transcription activator 1	Homo sapiens
151648	shugoshin-like 1 (S. pombe)	Homo sapiens
3014	H2A histone family, member X	Homo sapiens
5133	programmed cell death 1	Homo sapiens
1937	eukaryotic translation elongation factor 1 gamma	Homo sapiens
51078	THAP domain containing 4	Homo sapiens
4001	lamin B1	Homo sapiens
2296	forkhead box C1	Homo sapiens
148753	family with sequence similarity 163, member A	Homo sapiens
10133	optineurin	Homo sapiens
51455	REV1 homolog (S. cerevisiae)	Homo sapiens
729009	ferritin, heavy polypeptide 1; ferritin, heavy polypeptide-like 16; similar to ferritin, heavy polypeptide 1; ferritin, heavy polypeptide-like 3 pseudogene	Homo sapiens
2508	ferritin, heavy polypeptide 1; ferritin, heavy polypeptide-like 16; similar to ferritin, heavy polypeptide 1; ferritin, heavy polypeptide-like 3 pseudogene	Homo sapiens
2495	ferritin, heavy polypeptide 1; ferritin, heavy polypeptide-like 16; similar to ferritin, heavy polypeptide 1; ferritin, heavy polypeptide-like 3 pseudogene	Homo sapiens
2498	ferritin, heavy polypeptide 1; ferritin, heavy polypeptide-like 16; similar to ferritin, heavy polypeptide 1; ferritin, heavy polypeptide-like 3 pseudogene	Homo sapiens
81929	SEH1-like (S. cerevisiae)	Homo sapiens
2664	GDP dissociation inhibitor 1	Homo sapiens
55092	transmembrane protein 51	Homo sapiens
23633	karyopherin alpha 6 (importin alpha 7)	Homo sapiens
11345	GABA(A) receptor-associated protein-like 2	Homo sapiens
4143	methionine adenosyltransferase I, alpha	Homo sapiens
55720	TSR1, 20S rRNA accumulation, homolog (S. cerevisiae)	Homo sapiens
344787	zinc finger protein 860	Homo sapiens
4716	NADH dehydrogenase (ubiquinone) 1 beta subcomplex, 10, 22kDa	Homo sapiens
7284	Tu translation elongation factor, mitochondrial	Homo sapiens
1579	cytochrome P450, family 4, subfamily A, polypeptide 11	Homo sapiens
79071	ELOVL family member 6, elongation of long chain fatty acids (FEN1/Elo2, SUR4/Elo3-like, yeast)	Homo sapiens
6386	syndecan binding protein (syntenin)	Homo sapiens
24144	tuftelin interacting protein 11	Homo sapiens
29070	coiled-coil domain containing 113	Homo sapiens
64091	popeye domain containing 2	Homo sapiens
91663	myeloid-associated differentiation marker	Homo sapiens
142940	TruB pseudouridine (psi) synthase homolog 1 (E. coli)	Homo sapiens
659	bone morphogenetic protein receptor, type II (serine/threonine kinase)	Homo sapiens
2002	ELK1, member of ETS oncogene family	Homo sapiens
728658	ribosomal protein L13a pseudogene 7; ribosomal protein L13a pseudogene 5; ribosomal protein L13a pseudogene 16; ribosomal protein L13a; ribosomal protein L13a pseudogene 18	Homo sapiens
284821	ribosomal protein L13a pseudogene 7; ribosomal protein L13a pseudogene 5; ribosomal protein L13a pseudogene 16; ribosomal protein L13a; ribosomal protein L13a pseudogene 18	Homo sapiens
23521	ribosomal protein L13a pseudogene 7; ribosomal protein L13a pseudogene 5; ribosomal protein L13a pseudogene 16; ribosomal protein L13a; ribosomal protein L13a pseudogene 18	Homo sapiens
100129553	ribosomal protein L13a pseudogene 7; ribosomal protein L13a pseudogene 5; ribosomal protein L13a pseudogene 16; ribosomal protein L13a; ribosomal protein L13a pseudogene 18	Homo sapiens
402342	ribosomal protein L13a pseudogene 7; ribosomal protein L13a pseudogene 5; ribosomal protein L13a pseudogene 16; ribosomal protein L13a; ribosomal protein L13a pseudogene 18	Homo sapiens
29108	PYD and CARD domain containing	Homo sapiens
83552	membrane frizzled-related protein	Homo sapiens
5584	protein kinase C, iota	Homo sapiens
1836	solute carrier family 26 (sulfate transporter), member 2	Homo sapiens
9445	integral membrane protein 2B	Homo sapiens
84188	fatty acyl CoA reductase 1	Homo sapiens
3777	potassium channel, subfamily K, member 3	Homo sapiens
27286	sushi-repeat-containing protein, X-linked 2	Homo sapiens
654350	ribosomal protein L9; ribosomal protein L9 pseudogene 25	Homo sapiens
6133	ribosomal protein L9; ribosomal protein L9 pseudogene 25	Homo sapiens
8031	nuclear receptor coactivator 4	Homo sapiens
63891	ring finger protein 123	Homo sapiens
4629	myosin, heavy chain 11, smooth muscle	Homo sapiens
8861	LIM domain binding 1	Homo sapiens
56954	nitrilase family, member 2	Homo sapiens
8872	cell division cycle 123 homolog (S. cerevisiae)	Homo sapiens
146857	schlafen family member 13	Homo sapiens
100129743	ribosomal protein L7-like 1; ribosomal protein L7 pseudogene 14; ribosomal protein L7 pseudogene 21; ribosomal protein L7 pseudogene 22; ribosomal protein L7 pseudogene 46	Homo sapiens

642451	ribosomal protein L7-like 1; ribosomal protein L7 pseudogene 14; ribosomal protein L7 pseudogene 21; ribosomal protein L7 pseudogene 22; ribosomal protein L7 pseudogene 46	Homo sapiens
285855	ribosomal protein L7-like 1; ribosomal protein L7 pseudogene 14; ribosomal protein L7 pseudogene 21; ribosomal protein L7 pseudogene 22; ribosomal protein L7 pseudogene 46	Homo sapiens
729123	ribosomal protein L7-like 1; ribosomal protein L7 pseudogene 14; ribosomal protein L7 pseudogene 21; ribosomal protein L7 pseudogene 22; ribosomal protein L7 pseudogene 46	Homo sapiens
728620	ribosomal protein L7-like 1; ribosomal protein L7 pseudogene 14; ribosomal protein L7 pseudogene 21; ribosomal protein L7 pseudogene 22; ribosomal protein L7 pseudogene 46	Homo sapiens
84923	family with sequence similarity 104, member A	Homo sapiens
3184	heterogeneous nuclear ribonucleoprotein D (AU-rich element RNA binding protein 1, 37kDa)	Homo sapiens
57402	S100 calcium binding protein A14	Homo sapiens
221294	5'-nucleotidase domain containing 1	Homo sapiens
30062	retina and anterior neural fold homeobox	Homo sapiens
6925	transcription factor 4	Homo sapiens
23362	pleckstrin and Sec7 domain containing 3	Homo sapiens
4223	mesenchyme homeobox 2	Homo sapiens
26043	UBX domain protein 7	Homo sapiens
10421	CD2 (cytoplasmic tail) binding protein 2	Homo sapiens
54751	filamin binding LIM protein 1	Homo sapiens
8331	histone cluster 1, H2aj	Homo sapiens
81579	phospholipase A2, group XIIA	Homo sapiens
829	capping protein (actin filament) muscle Z-line, alpha 1	Homo sapiens
641522	ADP-ribosylation factor-like 17 pseudogene 1; ADP-ribosylation factor-like 17	Homo sapiens
51326	ADP-ribosylation factor-like 17 pseudogene 1; ADP-ribosylation factor-like 17	Homo sapiens
4756	neogenin homolog 1 (chicken)	Homo sapiens
92154	metastasis suppressor 1-like	Homo sapiens
29915	host cell factor C2	Homo sapiens
5033	prolyl 4-hydroxylase, alpha polypeptide I	Homo sapiens
10749	kinesin family member 1C	Homo sapiens
201164	phospholipase D family, member 6	Homo sapiens
3954	leucine zipper-EF-hand containing transmembrane protein 1	Homo sapiens
6810	syntaxin 4	Homo sapiens
9978	ring-box 1	Homo sapiens
6548	solute carrier family 9 (sodium/hydrogen exchanger), member 1	Homo sapiens
201294	unc-13 homolog D (C. elegans)	Homo sapiens
6929	transcription factor 3 (E2A immunoglobulin enhancer binding factors E12/E47)	Homo sapiens
25976	TCDD-inducible poly(ADP-ribose) polymerase	Homo sapiens
79924	adrenomedullin 2	Homo sapiens
9668	zinc finger protein 432	Homo sapiens
23476	bromodomain containing 4	Homo sapiens
10025	mediator complex subunit 16	Homo sapiens
9647	protein phosphatase 1F (PP2C domain containing)	Homo sapiens
10135	nicotinamide phosphoribosyltransferase	Homo sapiens
7182	nuclear receptor subfamily 2, group C, member 2	Homo sapiens
957	ectonucleoside triphosphate diphosphohydrolase 5	Homo sapiens
392335	similar to acetyl-Coenzyme A acyltransferase 2 (mitochondrial 3-oxoacyl-Coenzyme A thiolase); similar to KIAA1119 protein; myosin VB	Homo sapiens
441420	similar to acetyl-Coenzyme A acyltransferase 2 (mitochondrial 3-oxoacyl-Coenzyme A thiolase); similar to KIAA1119 protein; myosin VB	Homo sapiens
4645	similar to acetyl-Coenzyme A acyltransferase 2 (mitochondrial 3-oxoacyl-Coenzyme A thiolase); similar to KIAA1119 protein; myosin VB	Homo sapiens
27250	programmed cell death 4 (neoplastic transformation inhibitor)	Homo sapiens
389342	ribosomal protein L10; ribosomal protein L10 pseudogene 15; ribosomal protein L10 pseudogene 6; ribosomal protein L10 pseudogene 16; ribosomal protein L10 pseudogene 9	Homo sapiens
6134	ribosomal protein L10; ribosomal protein L10 pseudogene 15; ribosomal protein L10 pseudogene 6; ribosomal protein L10 pseudogene 16; ribosomal protein L10 pseudogene 9	Homo sapiens
100129975	ribosomal protein L10; ribosomal protein L10 pseudogene 15; ribosomal protein L10 pseudogene 6; ribosomal protein L10 pseudogene 16; ribosomal protein L10 pseudogene 9	Homo sapiens
284393	ribosomal protein L10; ribosomal protein L10 pseudogene 15; ribosomal protein L10 pseudogene 6; ribosomal protein L10 pseudogene 16; ribosomal protein L10 pseudogene 9	Homo sapiens
285176	ribosomal protein L10; ribosomal protein L10 pseudogene 15; ribosomal protein L10 pseudogene 6; ribosomal protein L10 pseudogene 16; ribosomal protein L10 pseudogene 9	Homo sapiens
151888	B and T lymphocyte associated	Homo sapiens
151354	hypothetical LOC653602; family with sequence similarity 84, member A	Homo sapiens
653602	hypothetical LOC653602; family with sequence similarity 84, member A	Homo sapiens
23678	serum/glucocorticoid regulated kinase family, member 3	Homo sapiens
55700	MAP7 domain containing 1	Homo sapiens
6239	ras responsive element binding protein 1	Homo sapiens
221178	spermatogenesis associated 13	Homo sapiens
55325	UFM1-specific peptidase 2	Homo sapiens
94103	ORM1-like 3 (S. cerevisiae)	Homo sapiens
2151	coagulation factor II (thrombin) receptor-like 2	Homo sapiens
652634	fem-1 homolog a (C. elegans); similar to fem-1 homolog a (C.elegans); similar to fem-1 homolog a	Homo sapiens
55527	fem-1 homolog a (C. elegans); similar to fem-1 homolog a (C.elegans); similar to fem-1 homolog a	Homo sapiens
390834	fem-1 homolog a (C. elegans); similar to fem-1 homolog a (C.elegans); similar to fem-1 homolog a	Homo sapiens
1850	dual specificity phosphatase 8	Homo sapiens

5795	protein tyrosine phosphatase, receptor type, J	Homo sapiens
79933	synaptopodin 2-like	Homo sapiens
8260	ARD1 homolog A, N-acetyltransferase (S. cerevisiae)	Homo sapiens
338328	glycosylphosphatidylinositol anchored high density lipoprotein binding protein 1	Homo sapiens
4361	MRE11 meiotic recombination 11 homolog A (S. cerevisiae)	Homo sapiens
100129086	similar to HIG1 domain family, member 1A; HIG1 hypoxia inducible domain family, member 1A; HIG1 hypoxia inducible domain family, member 1D	Homo sapiens
25994	similar to HIG1 domain family, member 1A; HIG1 hypoxia inducible domain family, member 1A; HIG1 hypoxia inducible domain family, member 1D	Homo sapiens
100130383	similar to HIG1 domain family, member 1A; HIG1 hypoxia inducible domain family, member 1A; HIG1 hypoxia inducible domain family, member 1D	Homo sapiens
29942	purine-rich element binding protein G	Homo sapiens
124245	zinc finger CCCH-type containing 18	Homo sapiens
23286	WW and C2 domain containing 1	Homo sapiens
79828	methyltransferase like 8	Homo sapiens
221830	TWIST neighbor	Homo sapiens
8034	solute carrier family 25 (mitochondrial carrier; Graves disease autoantigen), member 16	Homo sapiens
389873	selenophosphate synthetase 1; similar to selenophosphate synthetase 1	Homo sapiens
22929	selenophosphate synthetase 1; similar to selenophosphate synthetase 1	Homo sapiens
7170	tropomyosin 3	Homo sapiens
117247	solute carrier family 16, member 10 (aromatic amino acid transporter)	Homo sapiens
89882	tumor protein D52-like 3	Homo sapiens
6310	ataxin 1	Homo sapiens
2289	FK506 binding protein 5	Homo sapiens
57614	KIAA1468	Homo sapiens
5501	protein phosphatase 1, catalytic subunit, gamma isoform	Homo sapiens
9001	huntingtin-associated protein 1	Homo sapiens
55783	FtsJ methyltransferase domain containing 1	Homo sapiens
59345	guanine nucleotide binding protein (G protein), beta polypeptide 4	Homo sapiens
51100	SH3-domain GRB2-like endophilin B1	Homo sapiens
440503	lipid storage droplet protein 5	Homo sapiens
284459	GLI-Kruppel family member HKR1	Homo sapiens
3654	interleukin-1 receptor-associated kinase 1	Homo sapiens
285053	ribosomal protein L18a pseudogene 6; ribosomal protein L18a	Homo sapiens
6142	ribosomal protein L18a pseudogene 6; ribosomal protein L18a	Homo sapiens
1666	2,4-dienoyl CoA reductase 1, mitochondrial	Homo sapiens
10525	hypoxia up-regulated 1	Homo sapiens
6934	transcription factor 7-like 2 (T-cell specific, HMG-box)	Homo sapiens
7046	transforming growth factor, beta receptor 1	Homo sapiens
6416	mitogen-activated protein kinase kinase 4	Homo sapiens
51351	zinc finger protein 117	Homo sapiens
283106	casein kinase 2, alpha 1 polypeptide pseudogene; casein kinase 2, alpha 1 polypeptide	Homo sapiens
1457	casein kinase 2, alpha 1 polypeptide pseudogene; casein kinase 2, alpha 1 polypeptide	Homo sapiens
5168	ectonucleotide pyrophosphatase/phosphodiesterase 2	Homo sapiens
400	ADP-ribosylation factor-like 1	Homo sapiens
6273	S100 calcium binding protein A2	Homo sapiens
11119	butyrophilin, subfamily 3, member A1	Homo sapiens
8030	coiled-coil domain containing 6	Homo sapiens
1164	CDC28 protein kinase regulatory subunit 2	Homo sapiens
5214	phosphofructokinase, platelet	Homo sapiens
6542	solute carrier family 7 (cationic amino acid transporter, y+ system), member 2	Homo sapiens
55165	centrosomal protein 55kDa	Homo sapiens
23650	tripartite motif-containing 29	Homo sapiens
6992	protein phosphatase 1, regulatory (inhibitor) subunit 11	Homo sapiens
10398	myosin, light chain 9, regulatory	Homo sapiens
11128	polymerase (RNA) III (DNA directed) polypeptide A, 155kDa	Homo sapiens
728418	POM121 membrane glycoprotein-like 7 (rat); POM121 membrane glycoprotein-like 4 pseudogene (rat); POM121 membrane glycoprotein-like 3 (rat) pseudogene; POM121 membrane glycoprotein-like 10 (rat) pseudogene; similar to nuclear pore membrane protein 121	Homo sapiens
266697	POM121 membrane glycoprotein-like 7 (rat); POM121 membrane glycoprotein-like 4 pseudogene (rat); POM121 membrane glycoprotein-like 3 (rat) pseudogene; POM121 membrane glycoprotein-like 10 (rat) pseudogene; similar to nuclear pore membrane protein 121	Homo sapiens
727983	POM121 membrane glycoprotein-like 7 (rat); POM121 membrane glycoprotein-like 4 pseudogene (rat); POM121 membrane glycoprotein-like 3 (rat) pseudogene; POM121 membrane glycoprotein-like 10 (rat) pseudogene; similar to nuclear pore membrane protein 121	Homo sapiens
651452	POM121 membrane glycoprotein-like 7 (rat); POM121 membrane glycoprotein-like 4 pseudogene (rat); POM121 membrane glycoprotein-like 3 (rat) pseudogene; POM121 membrane glycoprotein-like 10 (rat) pseudogene; similar to nuclear pore membrane protein 121	Homo sapiens
646074	POM121 membrane glycoprotein-like 7 (rat); POM121 membrane glycoprotein-like 4 pseudogene (rat); POM121 membrane glycoprotein-like 3 (rat) pseudogene; POM121 membrane glycoprotein-like 10 (rat) pseudogene; similar to nuclear pore membrane protein 121	Homo sapiens
200424	tet oncogene family member 3	Homo sapiens
158219	tetratricopeptide repeat domain 39B	Homo sapiens
114984	FLYWCH family member 2	Homo sapiens
6208	ribosomal protein S14	Homo sapiens
23463	isoprenylcysteine carboxyl methyltransferase	Homo sapiens
58506	SR-related CTD-associated factor 1	Homo sapiens
9988	cyclin D binding myb-like transcription factor 1	Homo sapiens
2671	growth factor, augmenter of liver regeneration	Homo sapiens

57332	chromobox homolog 8 (Pc class homolog, Drosophila)	Homo sapiens
56950	SET and MYND domain containing 2	Homo sapiens
79572	ATPase type 13A3	Homo sapiens
8970	histone cluster 1, H2bj	Homo sapiens
4166	carbohydrate (N-acetylglucosamine 6-O) sulfotransferase 6	Homo sapiens
8819	Sin3A-associated protein, 30kDa	Homo sapiens
114034	target of EGR1, member 1 (nuclear)	Homo sapiens
339983	N-acetyltransferase 8-like (GCN5-related, putative)	Homo sapiens
84298	LLP homolog, long-term synaptic facilitation (Aplysia); similar to LLP homolog; similar to hCG2013595	Homo sapiens
100128533	LLP homolog, long-term synaptic facilitation (Aplysia); similar to LLP homolog; similar to hCG2013595	Homo sapiens
646644	LLP homolog, long-term synaptic facilitation (Aplysia); similar to LLP homolog; similar to hCG2013595	Homo sapiens
2045	EPH receptor A7	Homo sapiens
54883	coiled-coil domain containing 49	Homo sapiens
58490	regulation of nuclear pre-mRNA domain containing 1B	Homo sapiens
64710	nuclear casein kinase and cyclin-dependent kinase substrate 1	Homo sapiens
4189	DnaJ (Hsp40) homolog, subfamily B, member 9	Homo sapiens
255252	leucine rich repeat containing 57	Homo sapiens
137964	1-acylglycerol-3-phosphate O-acyltransferase 6 (lysophosphatidic acid acyltransferase, zeta)	Homo sapiens
8407	transgelin 2	Homo sapiens
2257	fibroblast growth factor 12	Homo sapiens
53827	FXYD domain containing ion transport regulator 5	Homo sapiens
54537	family with sequence similarity 35, member A	Homo sapiens
26032	sushi domain containing 5	Homo sapiens
57222	endoplasmic reticulum-golgi intermediate compartment (ERGIC) 1	Homo sapiens
388507	zinc finger family member 788	Homo sapiens
107	adenylate cyclase 1 (brain)	Homo sapiens
51135	interleukin-1 receptor-associated kinase 4	Homo sapiens
10443	NEDD4 binding protein 2-like 2	Homo sapiens
8153	Rho family GTPase 2	Homo sapiens
25822	DnaJ (Hsp40) homolog, subfamily B, member 5	Homo sapiens
23508	tetratricopeptide repeat domain 9	Homo sapiens
3150	high-mobility group nucleosome binding domain 1	Homo sapiens
7782	solute carrier family 30 (zinc transporter), member 4	Homo sapiens
126014	osteoclast associated, immunoglobulin-like receptor	Homo sapiens
11328	FK506 binding protein 9, 63 kDa	Homo sapiens
51752	endoplasmic reticulum aminopeptidase 1	Homo sapiens
5585	protein kinase N1	Homo sapiens
7091	transducin-like enhancer of split 4 (E(sp1) homolog, Drosophila)	Homo sapiens
6314	ataxin 7	Homo sapiens
23613	zinc finger, MYND-type containing 8	Homo sapiens
80727	tweety homolog 3 (Drosophila)	Homo sapiens
162073	inositol 1,4,5-triphosphate receptor interacting protein-like 2	Homo sapiens
54918	CKLF-like MARVEL transmembrane domain containing 6	Homo sapiens
5780	protein tyrosine phosphatase, non-receptor type 9	Homo sapiens
54973	cleavage and polyadenylation specific factor 3-like	Homo sapiens
10484	Sec23 homolog A (S. cerevisiae)	Homo sapiens
1804	dipeptidyl-peptidase 6	Homo sapiens
84245	methylthioribose-1-phosphate isomerase homolog (S. cerevisiae)	Homo sapiens
26155	nucleolar complex associated 2 homolog (S. cerevisiae)	Homo sapiens
3696	integrin, beta 8	Homo sapiens
2627	GATA binding protein 6	Homo sapiens
1973	similar to eukaryotic translation initiation factor 4A; small nucleolar RNA, H/ACA box 67; eukaryotic translation initiation factor 4A, isoform 1	Homo sapiens
728698	similar to eukaryotic translation initiation factor 4A; small nucleolar RNA, H/ACA box 67; eukaryotic translation initiation factor 4A, isoform 1	Homo sapiens
26781	similar to eukaryotic translation initiation factor 4A; small nucleolar RNA, H/ACA box 67; eukaryotic translation initiation factor 4A, isoform 1	Homo sapiens
130497	odd-skipped related 1 (Drosophila)	Homo sapiens
83855	Kruppel-like factor 16	Homo sapiens
25792	CDKN1A interacting zinc finger protein 1	Homo sapiens
10721	polymerase (DNA directed), theta	Homo sapiens
7637	zinc finger protein 84	Homo sapiens
7128	tumor necrosis factor, alpha-induced protein 3	Homo sapiens
56681	SAR1 homolog A (S. cerevisiae)	Homo sapiens
55146	zinc finger, DHHC-type containing 4	Homo sapiens
27113	BCL2 binding component 3	Homo sapiens
5976	UPF1 regulator of nonsense transcripts homolog (yeast)	Homo sapiens
613	breakpoint cluster region	Homo sapiens
80205	chromodomain helicase DNA binding protein 9	Homo sapiens
30836	deoxynucleotidyltransferase, terminal, interacting protein 2	Homo sapiens
4782	nuclear factor I/C (CCAAT-binding transcription factor)	Homo sapiens
8372	hyaluronoglucosaminidase 3	Homo sapiens
11118	butyrophilin, subfamily 3, member A2	Homo sapiens
7866	interferon-related developmental regulator 2	Homo sapiens
29843	SUMO1/sentrin specific peptidase 1	Homo sapiens

23291	F-box and WD repeat domain containing 11	Homo sapiens
646915	zinc finger protein 285B; zinc finger protein 285A; zinc finger protein 806	Homo sapiens
26974	zinc finger protein 285B; zinc finger protein 285A; zinc finger protein 806	Homo sapiens
147711	zinc finger protein 285B; zinc finger protein 285A; zinc finger protein 806	Homo sapiens
4591	tripartite motif-containing 37	Homo sapiens
6118	replication protein A2, 32kDa	Homo sapiens
4087	SMAD family member 2	Homo sapiens
55969	chromosome 20 open reading frame 24	Homo sapiens
4069	lysozyme (renal amyloidosis)	Homo sapiens
51463	G protein-coupled receptor 89C; G protein-coupled receptor 89B; G protein-coupled receptor 89A	Homo sapiens
653519	G protein-coupled receptor 89C; G protein-coupled receptor 89B; G protein-coupled receptor 89A	Homo sapiens
728932	G protein-coupled receptor 89C; G protein-coupled receptor 89B; G protein-coupled receptor 89A	Homo sapiens
6814	syntaxin binding protein 3	Homo sapiens
10691	glucocorticoid modulatory element binding protein 1	Homo sapiens
6195	ribosomal protein S6 kinase, 90kDa, polypeptide 1	Homo sapiens
3073	hexosaminidase A (alpha polypeptide)	Homo sapiens
163049	zinc finger protein 791	Homo sapiens
152926	protein phosphatase 1K (PP2C domain containing)	Homo sapiens
11044	polymerase (DNA directed) sigma	Homo sapiens
7365	UDP glucuronosyltransferase 2 family, polypeptide B10	Homo sapiens
348738	chromosome 2 open reading frame 48	Homo sapiens
8985	procollagen-lysine, 2-oxoglutarate 5-dioxygenase 3	Homo sapiens
729677	ribosomal protein L7 pseudogene 26; ribosomal protein L7 pseudogene 16; ribosomal protein L7; ribosomal protein L7 pseudogene 32; ribosomal protein L7 pseudogene 23; ribosomal protein L7 pseudogene 24; ribosomal protein L7 pseudogene 20	Homo sapiens
728843	ribosomal protein L7 pseudogene 26; ribosomal protein L7 pseudogene 16; ribosomal protein L7; ribosomal protein L7 pseudogene 32; ribosomal protein L7 pseudogene 23; ribosomal protein L7 pseudogene 24; ribosomal protein L7 pseudogene 20	Homo sapiens
648000	ribosomal protein L7 pseudogene 26; ribosomal protein L7 pseudogene 16; ribosomal protein L7; ribosomal protein L7 pseudogene 32; ribosomal protein L7 pseudogene 23; ribosomal protein L7 pseudogene 24; ribosomal protein L7 pseudogene 20	Homo sapiens
100127893	ribosomal protein L7 pseudogene 26; ribosomal protein L7 pseudogene 16; ribosomal protein L7; ribosomal protein L7 pseudogene 32; ribosomal protein L7 pseudogene 23; ribosomal protein L7 pseudogene 24; ribosomal protein L7 pseudogene 20	Homo sapiens
100130892	ribosomal protein L7 pseudogene 26; ribosomal protein L7 pseudogene 16; ribosomal protein L7; ribosomal protein L7 pseudogene 32; ribosomal protein L7 pseudogene 23; ribosomal protein L7 pseudogene 24; ribosomal protein L7 pseudogene 20	Homo sapiens
728380	ribosomal protein L7 pseudogene 26; ribosomal protein L7 pseudogene 16; ribosomal protein L7; ribosomal protein L7 pseudogene 32; ribosomal protein L7 pseudogene 23; ribosomal protein L7 pseudogene 24; ribosomal protein L7 pseudogene 20	Homo sapiens
6129	ribosomal protein L7 pseudogene 26; ribosomal protein L7 pseudogene 16; ribosomal protein L7; ribosomal protein L7 pseudogene 32; ribosomal protein L7 pseudogene 23; ribosomal protein L7 pseudogene 24; ribosomal protein L7 pseudogene 20	Homo sapiens
5162	pyruvate dehydrogenase (lipoamide) beta	Homo sapiens
286512	similar to eukaryotic translation initiation factor 4A2; eukaryotic translation initiation factor 4A, isoform 2	Homo sapiens
1974	similar to eukaryotic translation initiation factor 4A2; eukaryotic translation initiation factor 4A, isoform 2	Homo sapiens
83539	carbohydrate (N-acetylgalactosamine 4-0) sulfotransferase 9	Homo sapiens
8848	TSC22 domain family, member 1	Homo sapiens
7803	protein tyrosine phosphatase type IVA, member 1	Homo sapiens
4731	NADH dehydrogenase (ubiquinone) flavoprotein 3, 10kDa	Homo sapiens
10866	HLA complex P5	Homo sapiens
387338	NOL1/NOP2/Sun domain family, member 4	Homo sapiens
8479	HIRA interacting protein 3	Homo sapiens
6439	surfactant protein B	Homo sapiens
11343	monoglyceride lipase	Homo sapiens
29062	WD repeat domain 91	Homo sapiens
81575	apolipoprotein L domain containing 1	Homo sapiens
8195	McKusick-Kaufman syndrome	Homo sapiens
9643	mortality factor 4 like 2	Homo sapiens
3344	forkhead box N2	Homo sapiens
158293	family with sequence similarity 120A opposite strand	Homo sapiens
23612	pleckstrin homology-like domain, family A, member 3	Homo sapiens
477	ATPase, Na+/K+ transporting, alpha 2 (+) polypeptide	Homo sapiens
8574	aldo-keto reductase family 7, member A2 (aflatoxin aldehyde reductase)	Homo sapiens
84516	dynactin 5 (p25)	Homo sapiens
6832	suppressor of var1, 3-like 1 (S. cerevisiae)	Homo sapiens
7112	thymopoietin	Homo sapiens
57536	KIAA1328	Homo sapiens
81555	Yip1 domain family, member 5	Homo sapiens
26071	family with sequence similarity 127, member B	Homo sapiens
63916	engulfment and cell motility 2	Homo sapiens
339327	zinc finger protein 546	Homo sapiens
2693	growth hormone secretagogue receptor	Homo sapiens
149466	chromosome 1 open reading frame 210	Homo sapiens

9972	nucleoporin 153kDa	Homo sapiens
571	BTB and CNC homology 1, basic leucine zipper transcription factor 1	Homo sapiens
127829	ADP-ribosylation factor-like 8A	Homo sapiens
10980	COP9 constitutive photomorphogenic homolog subunit 6 (Arabidopsis)	Homo sapiens
54741	leptin receptor overlapping transcript	Homo sapiens
1869	E2F transcription factor 1	Homo sapiens
9069	claudin 12	Homo sapiens
9128	PRP4 pre-mRNA processing factor 4 homolog (yeast)	Homo sapiens
22847	zinc finger protein 507	Homo sapiens
663	BCL2/adenovirus E1B 19kDa interacting protein 2	Homo sapiens
51652	vacuolar protein sorting 24 homolog (S. cerevisiae); ring finger protein 103	Homo sapiens
7844	vacuolar protein sorting 24 homolog (S. cerevisiae); ring finger protein 103	Homo sapiens
85407	naked cuticle homolog 1 (Drosophila)	Homo sapiens
60491	NIF3 NGG1 interacting factor 3-like 1 (S. pombe)	Homo sapiens
483	ATPase, Na+/K+ transporting, beta 3 polypeptide	Homo sapiens
9066	synaptotagmin XVII; synaptotagmin VII	Homo sapiens
51760	synaptotagmin XVII; synaptotagmin VII	Homo sapiens
9955	heparan sulfate (glucosamine) 3-O-sulfotransferase 3A1	Homo sapiens
10251	sprouty homolog 3 (Drosophila)	Homo sapiens
11176	bromodomain adjacent to zinc finger domain, 2A	Homo sapiens
284723	solute carrier family 25, member 34	Homo sapiens
83998	regenerating islet-derived family, member 4	Homo sapiens
27440	cat eye syndrome chromosome region, candidate 5	Homo sapiens
253461	zinc finger and BTB domain containing 38	Homo sapiens
9748	STE20-like kinase (yeast)	Homo sapiens
1785	dynamin 2	Homo sapiens
5111	proliferating cell nuclear antigen	Homo sapiens
57182	ankyrin repeat domain 50	Homo sapiens
23528	zinc finger protein 281	Homo sapiens
84928	transmembrane protein 209	Homo sapiens
64743	WD repeat domain 13	Homo sapiens
51649	mitochondrial ribosomal protein S23	Homo sapiens
8555	CDC14 cell division cycle 14 homolog B (S. cerevisiae)	Homo sapiens
7644	zinc finger protein 91	Homo sapiens
9692	KIAA0391	Homo sapiens
10913	ectodysplasin A receptor	Homo sapiens
29886	sorting nexin 8	Homo sapiens
8797	tumor necrosis factor receptor superfamily, member 10a	Homo sapiens
51654	CDK5 regulatory subunit associated protein 1	Homo sapiens
11282	mannosyl (alpha-1,3-)-glycoprotein beta-1,4-N-acetylglucosaminyltransferase, isozyme B	Homo sapiens
196	aryl hydrocarbon receptor	Homo sapiens
5905	Ran GTPase activating protein 1	Homo sapiens
9711	KIAA0226	Homo sapiens
9652	tetratricopeptide repeat domain 37	Homo sapiens
54460	mitochondrial ribosomal protein S21	Homo sapiens
9294	sphingosine-1-phosphate receptor 2	Homo sapiens
4337	molybdenum cofactor synthesis 1	Homo sapiens
202134	family with sequence similarity 153, member B	Homo sapiens
51249	transmembrane protein 69	Homo sapiens
81844	tripartite motif-containing 56	Homo sapiens
54458	proline rich 13	Homo sapiens
4644	myosin VA (heavy chain 12, myoxin)	Homo sapiens
157922	calmodulin regulated spectrin-associated protein 1	Homo sapiens
115704	ecotropic viral integration site 5-like	Homo sapiens
27102	eukaryotic translation initiation factor 2-alpha kinase 1	Homo sapiens
23075	SWAP switching B-cell complex 70kDa subunit	Homo sapiens
55680	RUN and FYVE domain containing 2	Homo sapiens
9236	cell cycle progression 1	Homo sapiens
677	zinc finger protein 36, C3H type-like 1	Homo sapiens
4781	nuclear factor I/B	Homo sapiens
3704	inosine triphosphatase (nucleoside triphosphate pyrophosphatase)	Homo sapiens
96459	folliculin interacting protein 1	Homo sapiens
51196	phospholipase C, epsilon 1	Homo sapiens
84271	polymerase (DNA-directed), delta interacting protein 3	Homo sapiens
1802	DPH2 homolog (S. cerevisiae)	Homo sapiens
126017	zinc finger protein 813	Homo sapiens
127262	tumor protein p63 regulated 1-like	Homo sapiens
57834	cytochrome P450, family 4, subfamily F, polypeptide 11	Homo sapiens
54361	wingless-type MMTV integration site family, member 4	Homo sapiens
526	ATPase, H+ transporting, lysosomal 56/58kDa, V1 subunit B2	Homo sapiens
50861	stathmin-like 3	Homo sapiens
9372	zinc finger, FYVE domain containing 9	Homo sapiens
403	ADP-ribosylation factor-like 3	Homo sapiens
23558	WW domain binding protein 2	Homo sapiens
4598	mevalonate kinase	Homo sapiens
2145	enhancer of zeste homolog 1 (Drosophila)	Homo sapiens
728576	ribosomal protein L15 pseudogene 22; ribosomal protein L15 pseudogene 18; ribosomal protein L15 pseudogene 17; ribosomal protein L15 pseudogene 3; ribosomal protein L15 pseudogene 7; ribosomal protein L15	Homo sapiens

100130624	ribosomal protein L15 pseudogene 22; ribosomal protein L15 pseudogene 18; ribosomal protein L15 pseudogene 17; ribosomal protein L15 pseudogene 3; ribosomal protein L15 pseudogene 7; ribosomal protein L15	Homo sapiens
6138	ribosomal protein L15 pseudogene 22; ribosomal protein L15 pseudogene 18; ribosomal protein L15 pseudogene 17; ribosomal protein L15 pseudogene 3; ribosomal protein L15 pseudogene 7; ribosomal protein L15	Homo sapiens
728002	ribosomal protein L15 pseudogene 22; ribosomal protein L15 pseudogene 18; ribosomal protein L15 pseudogene 17; ribosomal protein L15 pseudogene 3; ribosomal protein L15 pseudogene 7; ribosomal protein L15	Homo sapiens
653232	ribosomal protein L15 pseudogene 22; ribosomal protein L15 pseudogene 18; ribosomal protein L15 pseudogene 17; ribosomal protein L15 pseudogene 3; ribosomal protein L15 pseudogene 7; ribosomal protein L15	Homo sapiens
728088	ribosomal protein L15 pseudogene 22; ribosomal protein L15 pseudogene 18; ribosomal protein L15 pseudogene 17; ribosomal protein L15 pseudogene 3; ribosomal protein L15 pseudogene 7; ribosomal protein L15	Homo sapiens
6990	dynein, light chain, Tctex-type 3	Homo sapiens
57003	coiled-coil domain containing 47	Homo sapiens
94056	synapse associated protein 1, SAP47 homolog (Drosophila)	Homo sapiens
54920	dihydrouridine synthase 2-like, SMM1 homolog (S. cerevisiae)	Homo sapiens
55839	centromere protein N	Homo sapiens
83461	cell division cycle associated 3	Homo sapiens
122961	iron-sulfur cluster assembly 2 homolog (S. cerevisiae)	Homo sapiens
23162	mitogen-activated protein kinase 8 interacting protein 3	Homo sapiens
9609	RAB36, member RAS oncogene family	Homo sapiens
57455	REX1, RNA exonuclease 1 homolog (S. cerevisiae)	Homo sapiens
262	adenosylmethionine decarboxylase 1	Homo sapiens
3239	homeobox D13	Homo sapiens
55273	transmembrane protein 100	Homo sapiens
220963	solute carrier family 16, member 9 (monocarboxylic acid transporter 9)	Homo sapiens
23005	mitogen-activated protein kinase binding protein 1	Homo sapiens
9135	rabaptin, RAB GTPase binding effector protein 1	Homo sapiens
5723	phosphoserine phosphatase-like; phosphoserine phosphatase	Homo sapiens
8781	phosphoserine phosphatase-like; phosphoserine phosphatase	Homo sapiens
1610	D-amino-acid oxidase	Homo sapiens
6856	synaptophysin-like 1	Homo sapiens
26580	Bernardinelli-Seip congenital lipodystrophy 2 (seipin)	Homo sapiens
154661	RUN domain containing 3B	Homo sapiens
55240	STEAP family member 3	Homo sapiens
5250	solute carrier family 25 (mitochondrial carrier; phosphate carrier), member 3	Homo sapiens
57579	family with sequence similarity 135, member A	Homo sapiens
22876	inositol polyphosphate-5-phosphatase F	Homo sapiens
3798	kinesin family member 5A	Homo sapiens
10424	progesterone receptor membrane component 2	Homo sapiens
8290	histone cluster 3, H3	Homo sapiens
23507	leucine rich repeat containing 8 family, member B	Homo sapiens
7162	trophoblast glycoprotein	Homo sapiens
10630	podoplanin	Homo sapiens
4983	oligophrenin 1	Homo sapiens
3301	DnaJ (Hsp40) homolog, subfamily A, member 1	Homo sapiens
23057	nicotinamide nucleotide adenylyltransferase 2	Homo sapiens
60485	salvador homolog 1 (Drosophila)	Homo sapiens
347733	tubulin, beta 2B	Homo sapiens
30818	Kv channel interacting protein 3, calsenilin	Homo sapiens
126328	NADH dehydrogenase (ubiquinone) 1 alpha subcomplex, 11, 14.7kDa	Homo sapiens
1058	centromere protein A	Homo sapiens
28970	chromosome 11 open reading frame 54	Homo sapiens
5034	prolyl 4-hydroxylase, beta polypeptide	Homo sapiens
255189	phospholipase A2, group IVF	Homo sapiens
23054	nuclear receptor coactivator 6	Homo sapiens
23780	apolipoprotein L, 2	Homo sapiens
60343	family with sequence similarity 3, member A	Homo sapiens
27246	ring finger protein 115	Homo sapiens
2794	guanine nucleotide binding protein-like 1	Homo sapiens
7620	zinc finger protein 69	Homo sapiens
7295	thioredoxin	Homo sapiens
6229	ribosomal protein S24	Homo sapiens
10808	heat shock 105kDa/110kDa protein 1	Homo sapiens
4976	optic atrophy 1 (autosomal dominant)	Homo sapiens
83881	Mix1 homeobox-like 1 (Xenopus laevis)	Homo sapiens
114825	PWWP domain containing 2A	Homo sapiens
607	B-cell CLL/lymphoma 9	Homo sapiens
22807	IKAROS family zinc finger 2 (Helios)	Homo sapiens
6892	TAP binding protein (tapasin)	Homo sapiens
55664	cell division cycle 37 homolog (S. cerevisiae)-like 1	Homo sapiens
84932	RAB2B, member RAS oncogene family	Homo sapiens
58494	junctional adhesion molecule 2	Homo sapiens
6513	solute carrier family 2 (facilitated glucose transporter), member 1	Homo sapiens
2218	fukutin	Homo sapiens
9180	oncostatin M receptor	Homo sapiens

4508	Cytochrome c oxidase subunit 3; ATP synthase subunit a; ATP synthase protein 8	Homo sapiens
4514	Cytochrome c oxidase subunit 3; ATP synthase subunit a; ATP synthase protein 8	Homo sapiens
4509	Cytochrome c oxidase subunit 3; ATP synthase subunit a; ATP synthase protein 8	Homo sapiens
6775074	Cytochrome c oxidase subunit 3; ATP synthase subunit a; ATP synthase protein 8	Homo sapiens
6775073	Cytochrome c oxidase subunit 3; ATP synthase subunit a; ATP synthase protein 8	Homo sapiens
6775077	Cytochrome c oxidase subunit 3; ATP synthase subunit a; ATP synthase protein 8	Homo sapiens
30837	suppressor of cytokine signaling 7	Homo sapiens
5725	polypyrimidine tract binding protein 1	Homo sapiens
10005	acyl-CoA thioesterase 8	Homo sapiens
55210	ATPase family, AAA domain containing 3A	Homo sapiens
53981	cleavage and polyadenylation specific factor 2, 100kDa	Homo sapiens
5213	phosphofructokinase, muscle	Homo sapiens
9620	cadherin, EGF LAG seven-pass G-type receptor 1 (flamingo homolog, Drosophila)	Homo sapiens
394263	mucin 21, cell surface associated	Homo sapiens
59084	ectonucleotide pyrophosphatase/phosphodiesterase 5 (putative function)	Homo sapiens
8732	RNA guanylyltransferase and 5'-phosphatase	Homo sapiens
221823	phosphoribosyl pyrophosphate synthetase 1; phosphoribosyl pyrophosphate synthetase 1-like 1	Homo sapiens
5631	phosphoribosyl pyrophosphate synthetase 1; phosphoribosyl pyrophosphate synthetase 1-like 1	Homo sapiens
2957	general transcription factor IIA, 1, 19/37kDa	Homo sapiens
222584	family with sequence similarity 83, member B	Homo sapiens
23786	BCL2-like 13 (apoptosis facilitator)	Homo sapiens
80167	chromosome 4 open reading frame 29	Homo sapiens
64112	modulator of apoptosis 1	Homo sapiens
1750	distal-less homeobox 6	Homo sapiens
2012	epithelial membrane protein 1	Homo sapiens
7189	TNF receptor-associated factor 6	Homo sapiens
9093	DnaJ (Hsp40) homolog, subfamily A, member 3	Homo sapiens
3673	integrin, alpha 2 (CD49B, alpha 2 subunit of VLA-2 receptor)	Homo sapiens
57089	ectonucleoside triphosphate diphosphohydrolase 7	Homo sapiens
23404	exosome component 2	Homo sapiens
729148	nuclear undecaprenyl pyrophosphate synthase 1 pseudogene; nuclear undecaprenyl pyrophosphate synthase 1 homolog (S. cerevisiae)	Homo sapiens
116150	nuclear undecaprenyl pyrophosphate synthase 1 pseudogene; nuclear undecaprenyl pyrophosphate synthase 1 homolog (S. cerevisiae)	Homo sapiens
2649	nuclear receptor subfamily 6, group A, member 1	Homo sapiens
155435	RNA binding motif protein 33	Homo sapiens
100132364	NIN1/RPN12 binding protein 1 homolog (S. cerevisiae); hypothetical LOC100132364	Homo sapiens
28987	NIN1/RPN12 binding protein 1 homolog (S. cerevisiae); hypothetical LOC100132364	Homo sapiens
9833	maternal embryonic leucine zipper kinase	Homo sapiens
920	CD4 molecule	Homo sapiens
51506	ubiquitin-fold modifier conjugating enzyme 1	Homo sapiens
23683	protein kinase D3	Homo sapiens
126382	nuclear receptor 2C2-associated protein	Homo sapiens
2329	flavin containing monooxygenase 4	Homo sapiens
3226	homeobox C10	Homo sapiens
4670	heterogeneous nuclear ribonucleoprotein M	Homo sapiens
1665	DEAH (Asp-Glu-Ala-His) box polypeptide 15	Homo sapiens
131566	discoidin, CUB and LCCL domain containing 2	Homo sapiens
1875	E2F transcription factor 5, p130-binding	Homo sapiens
356	Fas ligand (TNF superfamily, member 6)	Homo sapiens
3321	immunoglobulin superfamily, member 3	Homo sapiens
56896	dihydropyrimidinase-like 5	Homo sapiens
64359	nucleoredoxin	Homo sapiens
7248	tuberous sclerosis 1	Homo sapiens
3745	potassium voltage-gated channel, Shab-related subfamily, member 1	Homo sapiens
10574	chaperonin containing TCP1, subunit 7 (eta)	Homo sapiens
57481	KIAA1210	Homo sapiens
254048	ubinuclein 2	Homo sapiens
5251	phosphate regulating endopeptidase homolog, X-linked	Homo sapiens
1605	dystroglycan 1 (dystrophin-associated glycoprotein 1)	Homo sapiens
83743	glutamate-rich WD repeat containing 1	Homo sapiens
11033	ArfGAP with dual PH domains 1	Homo sapiens
8697	cell division cycle 23 homolog (S. cerevisiae)	Homo sapiens
57592	zinc finger protein 687	Homo sapiens
6346	chemokine (C-C motif) ligand 1	Homo sapiens
55729	activating transcription factor 7 interacting protein	Homo sapiens
87	actinin, alpha 1	Homo sapiens
54662	TBC1 domain family, member 13	Homo sapiens
908	chaperonin containing TCP1, subunit 6A (zeta 1)	Homo sapiens
11060	WW domain containing E3 ubiquitin protein ligase 2	Homo sapiens
1535	cytochrome b-245, alpha polypeptide	Homo sapiens
84154	brix domain containing 1 pseudogene; brix domain containing 1	Homo sapiens
729608	brix domain containing 1 pseudogene; brix domain containing 1	Homo sapiens
84172	polymerase (RNA) I polypeptide B, 128kDa	Homo sapiens
79080	coiled-coil domain containing 86	Homo sapiens
7465	WEE1 homolog (S. pombe)	Homo sapiens
140901	serine/threonine kinase 35	Homo sapiens

4943	TBC1 domain family, member 25	Homo sapiens
6367	chemokine (C-C motif) ligand 22	Homo sapiens
598	BCL2-like 1	Homo sapiens
2550	gamma-aminobutyric acid (GABA) B receptor, 1	Homo sapiens
2220	ficolin (collagen/fibrinogen domain containing lectin) 2 (hucolin)	Homo sapiens
197131	ubiquitin protein ligase E3 component n-recognin 1	Homo sapiens
5130	phosphate cytidylyltransferase 1, choline, alpha	Homo sapiens
139562	OTU domain containing 6A	Homo sapiens
353131	late cornified envelope 1A	Homo sapiens
51479	ankyrin repeat and FYVE domain containing 1	Homo sapiens
5193	peroxisomal biogenesis factor 12	Homo sapiens
56259	catenin, beta like 1	Homo sapiens
80351	tankyrase, TRF1-interacting ankyrin-related ADP-ribose polymerase 2	Homo sapiens
33	acyl-Coenzyme A dehydrogenase, long chain	Homo sapiens
9967	thyroid hormone receptor associated protein 3	Homo sapiens
22800	related RAS viral (r-ras) oncogene homolog 2; similar to related RAS viral (r-ras) oncogene homolog 2	Homo sapiens
100133211	related RAS viral (r-ras) oncogene homolog 2; similar to related RAS viral (r-ras) oncogene homolog 2	Homo sapiens
64976	mitochondrial ribosomal protein L40	Homo sapiens
5912	RAP2B, member of RAS oncogene family	Homo sapiens
84187	transmembrane protein 164	Homo sapiens
51205	acid phosphatase 6, lysophosphatidic	Homo sapiens
374308	patched domain containing 3	Homo sapiens
440193	coiled-coil domain containing 88C	Homo sapiens
642	bleomycin hydrolase	Homo sapiens
8774	N-ethylmaleimide-sensitive factor attachment protein, gamma	Homo sapiens
54880	BCL6 co-repressor	Homo sapiens
165631	poly (ADP-ribose) polymerase family, member 15	Homo sapiens
256130	transmembrane protein 196	Homo sapiens
6095	RAR-related orphan receptor A	Homo sapiens
387893	SET domain containing (lysine methyltransferase) 8	Homo sapiens
4809	NHP2 non-histone chromosome protein 2-like 1 (S. cerevisiae)	Homo sapiens
5336	phospholipase C, gamma 2 (phosphatidylinositol-specific)	Homo sapiens
121274	zinc finger protein 641	Homo sapiens
5476	cathepsin A	Homo sapiens
84460	zinc finger, matrin type 1	Homo sapiens
81603	tripartite motif-containing 8	Homo sapiens
140707	BRI3 binding protein	Homo sapiens
51092	SID1 transmembrane family, member 2	Homo sapiens
7551	zinc finger protein 3	Homo sapiens
474170	leucine rich repeat containing 37, member A2	Homo sapiens
112939	nucleus accumbens associated 1, BEN and BTB (POZ) domain containing	Homo sapiens
84679	solute carrier family 9 (sodium/hydrogen exchanger), member 7	Homo sapiens
55521	tripartite motif-containing 36	Homo sapiens
8550	mitogen-activated protein kinase-activated protein kinase 5	Homo sapiens
440086	ribosomal protein S6 pseudogene 25; ribosomal protein S6; ribosomal protein S6 pseudogene 1	Homo sapiens
6194	ribosomal protein S6 pseudogene 25; ribosomal protein S6; ribosomal protein S6 pseudogene 1	Homo sapiens
729389	ribosomal protein S6 pseudogene 25; ribosomal protein S6; ribosomal protein S6 pseudogene 1	Homo sapiens
55183	RAP1 interacting factor homolog (yeast)	Homo sapiens
64122	fructosamine 3 kinase	Homo sapiens
100128086	APAF1 interacting protein; similar to APAF1 interacting protein	Homo sapiens
51074	APAF1 interacting protein; similar to APAF1 interacting protein	Homo sapiens
7433	vasoactive intestinal peptide receptor 1	Homo sapiens
4988	opioid receptor, mu 1	Homo sapiens
5083	paired box 9	Homo sapiens
9054	NFS1 nitrogen fixation 1 homolog (S. cerevisiae)	Homo sapiens
126638	repetin	Homo sapiens
10389	sex comb on midleg-like 2 (Drosophila)	Homo sapiens
51439	family with sequence similarity 8, member A1	Homo sapiens
25886	WD repeat domain 51A	Homo sapiens
58512	discs, large (Drosophila) homolog-associated protein 3	Homo sapiens
10935	peroxiredoxin 3	Homo sapiens
23266	latrophilin 2	Homo sapiens
10592	structural maintenance of chromosomes 2	Homo sapiens
7222	transient receptor potential cation channel, subfamily C, member 3	Homo sapiens
8453	cullin 2	Homo sapiens
6829	suppressor of Ty 5 homolog (S. cerevisiae)	Homo sapiens
36	acyl-Coenzyme A dehydrogenase, short/branched chain	Homo sapiens
55171	TBCC domain containing 1	Homo sapiens
80223	RAB11 family interacting protein 1 (class I)	Homo sapiens
255101	coiled-coil domain containing 108	Homo sapiens
440991	ribosomal protein S3 pseudogene 3; ribosomal protein S3	Homo sapiens
6188	ribosomal protein S3 pseudogene 3; ribosomal protein S3	Homo sapiens
83943	IMP2 inner mitochondrial membrane peptidase-like (S. cerevisiae)	Homo sapiens
89797	neuron navigator 2	Homo sapiens

7082	tight junction protein 1 (zona occludens 1)	Homo sapiens
24147	four jointed box 1 (Drosophila)	Homo sapiens
3607	forkhead box K2	Homo sapiens
79837	phosphatidylinositol-5-phosphate 4-kinase, type II, gamma	Homo sapiens
8925	hect (homologous to the E6-AP (UBE3A) carboxyl terminus) domain and RCC1 (CHC1)-like domain (RLD) 1	Homo sapiens
57486	neurolysin (metallopeptidase M3 family)	Homo sapiens
4062	lymphocyte antigen 6 complex, locus H	Homo sapiens
26020	low density lipoprotein receptor-related protein 10	Homo sapiens
80219	coenzyme Q10 homolog B (S. cerevisiae)	Homo sapiens
8682	phosphoprotein enriched in astrocytes 15	Homo sapiens
285175	chromosome 2 open reading frame 21	Homo sapiens
84930	microtubule associated serine/threonine kinase-like	Homo sapiens
284613	cytochrome b-561 domain containing 1	Homo sapiens
83548	component of oligomeric golgi complex 3	Homo sapiens
5329	plasminogen activator, urokinase receptor	Homo sapiens
51329	ADP-ribosylation-like factor 6 interacting protein 4	Homo sapiens
3235	homeobox D9	Homo sapiens
4281	midline 1 (Opitz/BBB syndrome)	Homo sapiens
8653	DEAD (Asp-Glu-Ala-Asp) box polypeptide 3, Y-linked	Homo sapiens
10090	uronyl-2-sulfotransferase	Homo sapiens
57489	outer dense fiber of sperm tails 2-like	Homo sapiens
396	Rho GDP dissociation inhibitor (GDI) alpha	Homo sapiens
51026	golgi transport 1 homolog B (S. cerevisiae)	Homo sapiens
200407	cellular repressor of E1A-stimulated genes 2	Homo sapiens
8444	dual-specificity tyrosine-(Y)-phosphorylation regulated kinase 3	Homo sapiens
10687	paraneoplastic antigen MA2	Homo sapiens
2702	gap junction protein, alpha 5, 40kDa	Homo sapiens
28974	chromosome 19 open reading frame 53	Homo sapiens
85451	unkempt homolog (Drosophila)	Homo sapiens
55157	aspartyl-tRNA synthetase 2, mitochondrial	Homo sapiens
2521	fusion (involved in t(12;16) in malignant liposarcoma)	Homo sapiens
221458	kinesin family member 6	Homo sapiens
653659	transmembrane protein 183A; transmembrane protein 183B	Homo sapiens
92703	transmembrane protein 183A; transmembrane protein 183B	Homo sapiens
6646	sterol O-acyltransferase 1	Homo sapiens
444	aspartate beta-hydroxylase	Homo sapiens
64423	inverted formin, FH2 and WH2 domain containing	Homo sapiens
55186	solute carrier family 25, member 36	Homo sapiens
6156	ribosomal protein L30	Homo sapiens
23268	dynamin binding protein	Homo sapiens
388569	zinc finger protein 324B	Homo sapiens
3480	insulin-like growth factor 1 receptor	Homo sapiens
29121	C-type lectin domain family 2, member D	Homo sapiens
26168	SUMO1/sentrin/SMT3 specific peptidase 3	Homo sapiens
256356	glycerol kinase 5 (putative)	Homo sapiens
25870	sulfatase modifying factor 2	Homo sapiens
9852	EPM2A (laforin) interacting protein 1	Homo sapiens
9149	dual-specificity tyrosine-(Y)-phosphorylation regulated kinase 1B	Homo sapiens
6903	tubulin folding cofactor C	Homo sapiens
25	c-abl oncogene 1, receptor tyrosine kinase	Homo sapiens
64344	hypoxia inducible factor 3, alpha subunit	Homo sapiens
117178	synovial sarcoma, X breakpoint 2 interacting protein	Homo sapiens
4282	macrophage migration inhibitory factor (glycosylation-inhibiting factor)	Homo sapiens
133121	ectonucleotide pyrophosphatase/phosphodiesterase 6	Homo sapiens
114908	transmembrane protein 123	Homo sapiens
65265	chromosome 8 open reading frame 33	Homo sapiens
339175	methyltransferase like 2A	Homo sapiens
2782	guanine nucleotide binding protein (G protein), beta polypeptide 1	Homo sapiens
57826	RAP2C, member of RAS oncogene family	Homo sapiens
64855	family with sequence similarity 129, member B	Homo sapiens
10600	ubiquitin specific peptidase 16	Homo sapiens
9922	IQ motif and Sec7 domain 1	Homo sapiens
57097	poly (ADP-ribose) polymerase family, member 11	Homo sapiens
246243	ribonuclease H1	Homo sapiens
23283	cleavage stimulation factor, 3' pre-RNA, subunit 2, 64kDa, tau variant	Homo sapiens
79836	LON peptidase N-terminal domain and ring finger 3	Homo sapiens
26088	golgi associated, gamma adaptin ear containing, ARF binding protein 1	Homo sapiens
5589	protein kinase C substrate 80K-H	Homo sapiens
80314	enhancer of polycomb homolog 1 (Drosophila)	Homo sapiens
158427	chromosome 9 open reading frame 97	Homo sapiens
6731	signal recognition particle 72kDa	Homo sapiens
3052	holocytochrome c synthase (cytochrome c heme-lyase)	Homo sapiens
728927	similar to hCG40110	Homo sapiens
136259	Kruppel-like factor 14	Homo sapiens
10105	peptidylprolyl isomerase F	Homo sapiens
8645	potassium channel, subfamily K, member 5	Homo sapiens
2355	FOS-like antigen 2	Homo sapiens
51106	transcription factor B1, mitochondrial	Homo sapiens

51107	anterior pharynx defective 1 homolog A (C. elegans)	Homo sapiens
9145	synaptogyrin 1	Homo sapiens
197257	lactate dehydrogenase D	Homo sapiens
79172	centromere protein O	Homo sapiens
84687	protein phosphatase 1, regulatory (inhibitor) subunit 9B	Homo sapiens
8711	tyrosine kinase, non-receptor, 1	Homo sapiens
2903	glutamate receptor, ionotropic, N-methyl D-aspartate 2A	Homo sapiens
7532	tyrosine 3-monooxygenase/tryptophan 5-monooxygenase activation protein, gamma polypeptide	Homo sapiens
26040	SET binding protein 1	Homo sapiens
7534	tyrosine 3-monooxygenase/tryptophan 5-monooxygenase activation protein, zeta polypeptide	Homo sapiens
58477	signal recognition particle receptor, B subunit	Homo sapiens
727997	ribosomal protein S12; ribosomal protein S12 pseudogene 4; ribosomal protein S12 pseudogene 11; ribosomal protein S12 pseudogene 9	Homo sapiens
442270	ribosomal protein S12; ribosomal protein S12 pseudogene 4; ribosomal protein S12 pseudogene 11; ribosomal protein S12 pseudogene 9	Homo sapiens
6206	ribosomal protein S12; ribosomal protein S12 pseudogene 4; ribosomal protein S12 pseudogene 11; ribosomal protein S12 pseudogene 9	Homo sapiens
391370	ribosomal protein S12; ribosomal protein S12 pseudogene 4; ribosomal protein S12 pseudogene 11; ribosomal protein S12 pseudogene 9	Homo sapiens
130589	galactose mutarotase (aldose 1-epimerase)	Homo sapiens
55193	polybromo 1	Homo sapiens
80207	optic atrophy 3 (autosomal recessive, with chorea and spastic paraplegia)	Homo sapiens
221937	forkhead box K1	Homo sapiens
54893	myotubularin related protein 10	Homo sapiens
64919	B-cell CLL/lymphoma 11B (zinc finger protein)	Homo sapiens
10945	KDEL (Lys-Asp-Glu-Leu) endoplasmic reticulum protein retention receptor 1	Homo sapiens
5824	peroxisomal biogenesis factor 19	Homo sapiens
23248	regulation of nuclear pre-mRNA domain containing 2	Homo sapiens
54832	vacuolar protein sorting 13 homolog C (S. cerevisiae)	Homo sapiens
10082	glypican 6	Homo sapiens
5441	polymerase (RNA) II (DNA directed) polypeptide L, 7.6kDa	Homo sapiens
10171	RNA terminal phosphate cyclase-like 1	Homo sapiens
10195	asparagine-linked glycosylation 3, alpha-1,3- mannosyltransferase homolog (S. cerevisiae)	Homo sapiens
1965	eukaryotic translation initiation factor 2, subunit 1 alpha, 35kDa	Homo sapiens
56134	protocadherin alpha 13; protocadherin alpha 10; protocadherin alpha subfamily C, 1; protocadherin alpha subfamily C, 2	Homo sapiens
56139	protocadherin alpha 13; protocadherin alpha 10; protocadherin alpha subfamily C, 1; protocadherin alpha subfamily C, 2	Homo sapiens
56135	protocadherin alpha 13; protocadherin alpha 10; protocadherin alpha subfamily C, 1; protocadherin alpha subfamily C, 2	Homo sapiens
56136	protocadherin alpha 13; protocadherin alpha 10; protocadherin alpha subfamily C, 1; protocadherin alpha subfamily C, 2	Homo sapiens
5753	PTK6 protein tyrosine kinase 6	Homo sapiens
84294	UTP23, small subunit (SSU) processome component, homolog (yeast)	Homo sapiens
155368	Williams Beuren syndrome chromosome region 27	Homo sapiens
2776	guanine nucleotide binding protein (G protein), q polypeptide	Homo sapiens
55124	hydroxy-delta-5-steroid dehydrogenase, 3 beta- and steroid delta-isomerase 7; piwi-like 2 (Drosophila)	Homo sapiens
80270	hydroxy-delta-5-steroid dehydrogenase, 3 beta- and steroid delta-isomerase 7; piwi-like 2 (Drosophila)	Homo sapiens
440181	similar to transmembrane protein 98; transmembrane protein 98	Homo sapiens
26022	similar to transmembrane protein 98; transmembrane protein 98	Homo sapiens
63876	PBX/knotted 1 homeobox 2	Homo sapiens
65985	acetoacetyl-CoA synthetase	Homo sapiens
5049	platelet-activating factor acetylhydrolase, isoform Ib, subunit 2 (30kDa)	Homo sapiens
4326	matrix metallopeptidase 17 (membrane-inserted)	Homo sapiens
55890	G protein-coupled receptor, family C, group 5, member C	Homo sapiens
23467	neuronal pentraxin receptor	Homo sapiens
114987	WD repeat domain 31	Homo sapiens
4157	tubulin, beta 3; melanocortin 1 receptor (alpha melanocyte stimulating hormone receptor)	Homo sapiens
10381	tubulin, beta 3; melanocortin 1 receptor (alpha melanocyte stimulating hormone receptor)	Homo sapiens
57085	angiotensin II receptor-associated protein	Homo sapiens
9112	metastasis associated 1	Homo sapiens
25979	dehydrogenase/reductase (SDR family) member 7B	Homo sapiens
283149	B-cell CLL/lymphoma 9-like	Homo sapiens
116064	leucine rich repeat containing 58	Homo sapiens
84865	coiled-coil domain containing 142	Homo sapiens
26224	F-box and leucine-rich repeat protein 3	Homo sapiens
9816	URB2 ribosome biogenesis 2 homolog (S. cerevisiae)	Homo sapiens
3386	intercellular adhesion molecule 4 (Landsteiner-Wiener blood group)	Homo sapiens
91010	formin-like 3	Homo sapiens
55751	transmembrane protein 184C	Homo sapiens
9753	zinc finger and SCAN domain containing 12	Homo sapiens
59286	ubiquitin-like 5	Homo sapiens
2103	estrogen-related receptor beta	Homo sapiens
353116	Rab interacting lysosomal protein-like 1	Homo sapiens
9402	GRB2-related adaptor protein 2	Homo sapiens

83861	radial spoke 3 homolog (Chlamydomonas)	Homo sapiens
91050	coiled-coil domain containing 149	Homo sapiens
29933	G protein-coupled receptor 132	Homo sapiens
164	adaptor-related protein complex 1, gamma 1 subunit	Homo sapiens
10257	ATP-binding cassette, sub-family C (CFTR/MRP), member 4	Homo sapiens
144404	transmembrane protein 120B	Homo sapiens
57010	calcium binding protein 4	Homo sapiens
728611	BMS1 homolog, ribosome assembly protein (yeast) pseudogene; BMS1 homolog, ribosome assembly protein (yeast)	Homo sapiens
653557	BMS1 homolog, ribosome assembly protein (yeast) pseudogene; BMS1 homolog, ribosome assembly protein (yeast)	Homo sapiens
642311	BMS1 homolog, ribosome assembly protein (yeast) pseudogene; BMS1 homolog, ribosome assembly protein (yeast)	Homo sapiens
9790	BMS1 homolog, ribosome assembly protein (yeast) pseudogene; BMS1 homolog, ribosome assembly protein (yeast)	Homo sapiens
5316	PBX/knotted 1 homeobox 1	Homo sapiens
55846	integrin alpha FG-GAP repeat containing 2	Homo sapiens
10940	processing of precursor 1, ribonuclease P/MRP subunit (S. cerevisiae)	Homo sapiens
4213	Meis homeobox 3 pseudogene 1	Homo sapiens
93663	Rho GTPase activating protein 18	Homo sapiens
6152	ribosomal protein L24; ribosomal protein L24 pseudogene 6	Homo sapiens
100129808	ribosomal protein L24; ribosomal protein L24 pseudogene 6	Homo sapiens
95	aminoacylase 1	Homo sapiens
91433	RCC1 domain containing 1	Homo sapiens
473	arginine-glutamic acid dipeptide (RE) repeats	Homo sapiens
79901	cytochrome b reductase 1	Homo sapiens
64981	mitochondrial ribosomal protein L34	Homo sapiens
5194	peroxisomal biogenesis factor 13	Homo sapiens
118812	MORN repeat containing 4	Homo sapiens
1583	cytochrome P450, family 11, subfamily A, polypeptide 1	Homo sapiens
23087	tripartite motif-containing 35	Homo sapiens
26278	spastic ataxia of Charlevoix-Saguenay (sacsin)	Homo sapiens
5537	protein phosphatase 6, catalytic subunit	Homo sapiens
8798	dual-specificity tyrosine-(Y)-phosphorylation regulated kinase 4	Homo sapiens
81890	queuine tRNA-ribosyltransferase 1	Homo sapiens
23339	vacuolar protein sorting 39 homolog (S. cerevisiae)	Homo sapiens
11249	neurexophilin 2	Homo sapiens
3187	heterogeneous nuclear ribonucleoprotein H1 (H)	Homo sapiens
23247	KIAA0556	Homo sapiens
93474	zinc finger protein 670	Homo sapiens
63875	mitochondrial ribosomal protein L17	Homo sapiens
55586	myo-inositol oxygenase	Homo sapiens
11153	FIC domain containing	Homo sapiens
9910	RAB GTPase activating protein 1-like	Homo sapiens
1520	cathepsin S	Homo sapiens
57226	LYR motif containing 2	Homo sapiens
5562	protein kinase, AMP-activated, alpha 1 catalytic subunit	Homo sapiens
79016	DET1 and DDB1 associated 1	Homo sapiens
84836	abhydrolase domain containing 14B	Homo sapiens
51384	wingless-type MMTV integration site family, member 16	Homo sapiens
84895	family with sequence similarity 73, member B	Homo sapiens
1145	cholinergic receptor, nicotinic, epsilon	Homo sapiens
475	ATX1 antioxidant protein 1 homolog (yeast)	Homo sapiens
5709	proteasome (prosome, macropain) 26S subunit, non-ATPase, 3	Homo sapiens
177	advanced glycosylation end product-specific receptor	Homo sapiens
2737	GLI family zinc finger 3	Homo sapiens
1431	citrate synthase	Homo sapiens
993	cell division cycle 25 homolog A (S. pombe)	Homo sapiens
1174	adaptor-related protein complex 1, sigma 1 subunit	Homo sapiens
7621	zinc finger protein 70	Homo sapiens
6581	solute carrier family 22 (extraneuronal monoamine transporter), member 3	Homo sapiens
64429	zinc finger, DHHC-type containing 6	Homo sapiens
3156	3-hydroxy-3-methylglutaryl-Coenzyme A reductase	Homo sapiens
441631	tetraspanin 11	Homo sapiens
389434	iodotyrosine deiodinase	Homo sapiens
84248	forty-two-three domain containing 1	Homo sapiens
9416	DEAD (Asp-Glu-Ala-Asp) box polypeptide 23	Homo sapiens
23031	microtubule associated serine/threonine kinase 3	Homo sapiens
55239	2-oxoglutarate and iron-dependent oxygenase domain containing 1	Homo sapiens
64983	mitochondrial ribosomal protein L32	Homo sapiens
79005	sodium channel modifier 1	Homo sapiens
85462	FH2 domain containing 1	Homo sapiens
10286	breast carcinoma amplified sequence 2	Homo sapiens
283078	mohawk homeobox	Homo sapiens
730429	similar to E3 ubiquitin protein ligase, HECT domain containing, 1; ubiquitin protein ligase E3 component n-recognin 5	Homo sapiens
51366	similar to E3 ubiquitin protein ligase, HECT domain containing, 1; ubiquitin protein ligase E3 component n-recognin 5	Homo sapiens
100132779	serine/arginine repetitive matrix 2; hypothetical LOC100132779	Homo sapiens

23524	serine/arginine repetitive matrix 2; hypothetical LOC100132779	Homo sapiens
6517	solute carrier family 2 (facilitated glucose transporter), member 4	Homo sapiens
9340	glucagon-like peptide 2 receptor	Homo sapiens
100129652	hypothetical protein LOC100129652; ezrin	Homo sapiens
7430	hypothetical protein LOC100129652; ezrin	Homo sapiens
79850	family with sequence similarity 57, member A	Homo sapiens
51474	LIM domain and actin binding 1	Homo sapiens
9076	claudin 1	Homo sapiens
50940	phosphodiesterase 11A	Homo sapiens
54969	chromosome 4 open reading frame 27	Homo sapiens
133746	junction mediating and regulatory protein, p53 cofactor	Homo sapiens
9545	RAB3D, member RAS oncogene family	Homo sapiens
23125	calmodulin binding transcription activator 2	Homo sapiens
56905	chromosome 15 open reading frame 39	Homo sapiens
92399	mitochondrial ribosome recycling factor	Homo sapiens
643314	hypothetical LOC643314	Homo sapiens
1810	down-regulator of transcription 1, TBP-binding (negative cofactor 2)	Homo sapiens
55197	regulation of nuclear pre-mRNA domain containing 1A	Homo sapiens
51185	cereblon	Homo sapiens
64284	RAB17, member RAS oncogene family	Homo sapiens
6550	solute carrier family 9 (sodium/hydrogen exchanger), member 3	Homo sapiens
394	Rho GTPase activating protein 5	Homo sapiens
5438	polymerase (RNA) II (DNA directed) polypeptide I, 14.5kDa	Homo sapiens
6631	small nuclear ribonucleoprotein polypeptide C	Homo sapiens
389432	sterile alpha motif domain containing 5	Homo sapiens
51182	heat shock 70kDa protein 14	Homo sapiens
84890	2-aminoethanethiol (cysteamine) dioxygenase	Homo sapiens
1736	dyskeratosis congenita 1, dyskerin	Homo sapiens
339804	chromosome 2 open reading frame 74	Homo sapiens
23405	dicer 1, ribonuclease type III	Homo sapiens
80335	WD repeat domain 82	Homo sapiens
7559	postmeiotic segregation increased 2-like 3; zinc finger protein 12	Homo sapiens
5387	postmeiotic segregation increased 2-like 3; zinc finger protein 12	Homo sapiens
84542	KIAA1841	Homo sapiens
6769	SH3 and cysteine rich domain	Homo sapiens
83591	THAP domain containing, apoptosis associated protein 2	Homo sapiens
3910	laminin, alpha 4	Homo sapiens
388697	hornerin	Homo sapiens
23673	syntaxin 12	Homo sapiens
55666	nuclear protein localization 4 homolog (S. cerevisiae)	Homo sapiens
3376	isoleucyl-tRNA synthetase	Homo sapiens
2885	growth factor receptor-bound protein 2	Homo sapiens
11113	citron (rho-interacting, serine/threonine kinase 21)	Homo sapiens
55083	kinesin family member 26B	Homo sapiens
375287	RNA binding motif protein 43	Homo sapiens
10549	peroxiredoxin 4	Homo sapiens
5881	ras-related C3 botulinum toxin substrate 3 (rho family, small GTP binding protein Rac3)	Homo sapiens
170691	ADAM metallopeptidase with thrombospondin type 1 motif, 17	Homo sapiens
23523	calcineurin binding protein 1	Homo sapiens
26015	RNA polymerase II associated protein 1	Homo sapiens
6884	TAF13 RNA polymerase II, TATA box binding protein (TBP)-associated factor, 18kDa	Homo sapiens
29803	replication initiator 1	Homo sapiens
23207	pleckstrin homology domain containing, family M (with RUN domain) member 2	Homo sapiens
1404	hyaluronan and proteoglycan link protein 1	Homo sapiens
29986	solute carrier family 39 (zinc transporter), member 2	Homo sapiens
130574	LY6/PLAUR domain containing 6	Homo sapiens
57418	WD repeat domain 18	Homo sapiens
146050	zinc finger and SCAN domain containing 29	Homo sapiens
29066	zinc finger CCCH-type containing 7A	Homo sapiens
26985	adaptor-related protein complex 3, mu 1 subunit	Homo sapiens
55266	transmembrane protein 19	Homo sapiens
51616	TAF9B RNA polymerase II, TATA box binding protein (TBP)-associated factor, 31kDa	Homo sapiens
79699	zyg-11 homolog B (C. elegans)	Homo sapiens
1462	versican	Homo sapiens
79184	BRCA1/BRCA2-containing complex, subunit 3	Homo sapiens
23654	plexin B2	Homo sapiens
4085	MAD2 mitotic arrest deficient-like 1 (yeast)	Homo sapiens
5579	protein kinase C, beta	Homo sapiens
100130561	high-mobility group box 1; high-mobility group box 1-like 10	Homo sapiens
3146	high-mobility group box 1; high-mobility group box 1-like 10	Homo sapiens
57678	glycerol-3-phosphate acyltransferase, mitochondrial	Homo sapiens
6352	chemokine (C-C motif) ligand 5	Homo sapiens
56994	choline phosphotransferase 1	Homo sapiens
4154	muscleblind-like (Drosophila)	Homo sapiens
55635	DEP domain containing 1	Homo sapiens
10607	transducin (beta)-like 3	Homo sapiens
3309	hypothetical gene supported by AF216292; NM_005347; heat shock 70kDa protein 5 (glucose-regulated protein, 78kDa)	Homo sapiens

400750	hypothetical gene supported by AF216292; NM_005347; heat shock 70kDa protein 5 (glucose-regulated protein, 78kDa)	Homo sapiens
54784	alkB, alkylation repair homolog 4 (E. coli)	Homo sapiens
375035	SFT2 domain containing 2	Homo sapiens
64432	mitochondrial ribosomal protein S25	Homo sapiens
4008	LIM domain 7	Homo sapiens
11330	chymotrypsin C (caldecrin)	Homo sapiens
871	serpin peptidase inhibitor, clade H (heat shock protein 47), member 1, (collagen binding protein 1)	Homo sapiens
79739	tubulin tyrosine ligase-like family, member 7	Homo sapiens
27341	ribosomal RNA processing 7 homolog B (S. cerevisiae); ribosomal RNA processing 7 homolog A (S. cerevisiae)	Homo sapiens
91695	ribosomal RNA processing 7 homolog B (S. cerevisiae); ribosomal RNA processing 7 homolog A (S. cerevisiae)	Homo sapiens
162239	zinc finger protein 1 homolog (mouse)	Homo sapiens
1763	DNA replication helicase 2 homolog (yeast)	Homo sapiens
3981	ligase IV, DNA, ATP-dependent	Homo sapiens
7436	very low density lipoprotein receptor	Homo sapiens
64434	nucleolar protein with MIF4G domain 1	Homo sapiens
56897	Werner helicase interacting protein 1	Homo sapiens
5518	protein phosphatase 2 (formerly 2A), regulatory subunit A, alpha isoform	Homo sapiens
23366	KIAA0895	Homo sapiens
6590	secretory leukocyte peptidase inhibitor	Homo sapiens
57695	ubiquitin specific peptidase 37	Homo sapiens
56204	KIAA1370	Homo sapiens
7368	UDP glycosyltransferase 8	Homo sapiens
100133017	hypothetical LOC100132856; hypothetical LOC100128100; hypothetical LOC100133017; SNAP25-interacting protein	Homo sapiens
100132856	hypothetical LOC100132856; hypothetical LOC100128100; hypothetical LOC100133017; SNAP25-interacting protein	Homo sapiens
80725	hypothetical LOC100132856; hypothetical LOC100128100; hypothetical LOC100133017; SNAP25-interacting protein	Homo sapiens
100128100	hypothetical LOC100132856; hypothetical LOC100128100; hypothetical LOC100133017; SNAP25-interacting protein	Homo sapiens
23071	endoplasmic reticulum protein 44	Homo sapiens
1676	DNA fragmentation factor, 45kDa, alpha polypeptide	Homo sapiens
55299	brix domain containing 2	Homo sapiens
8473	O-linked N-acetylglucosamine (GlcNAc) transferase (UDP-N-acetylglucosamine:polypeptide-N-acetylglucosaminyl transferase)	Homo sapiens
414236	chromosome 10 open reading frame 55	Homo sapiens
222229	leucine-rich repeats and WD repeat domain containing 1	Homo sapiens
79731	asparaginyl-tRNA synthetase 2, mitochondrial (putative)	Homo sapiens
79711	importin 4	Homo sapiens
8744	tumor necrosis factor (ligand) superfamily, member 9	Homo sapiens
11108	PR domain containing 4	Homo sapiens
10155	tripartite motif-containing 28	Homo sapiens
360023	zinc finger and BTB domain containing 41	Homo sapiens
148206	zinc finger protein 714	Homo sapiens
57720	G protein-coupled receptor 107	Homo sapiens
81619	tetraspanin 14	Homo sapiens
23380	SLIT-ROBO Rho GTPase activating protein 2	Homo sapiens
1452	casein kinase 1, alpha 1	Homo sapiens
10109	actin related protein 2/3 complex, subunit 2, 34kDa	Homo sapiens
404785	POTE ankyrin domain family, member G	Homo sapiens
8445	dual-specificity tyrosine-(Y)-phosphorylation regulated kinase 2	Homo sapiens
59269	human immunodeficiency virus type I enhancer binding protein 3	Homo sapiens
158586	zinc finger, X-linked, duplicated B	Homo sapiens
84342	peptide deformylase (mitochondrial); component of oligomeric golgi complex 8	Homo sapiens
64146	peptide deformylase (mitochondrial); component of oligomeric golgi complex 8	Homo sapiens
915	CD3d molecule, delta (CD3-TCR complex)	Homo sapiens
59349	kelch-like 12 (Drosophila)	Homo sapiens
114294	lactamase, beta	Homo sapiens
1870	E2F transcription factor 2	Homo sapiens
122525	chromosome 14 open reading frame 28	Homo sapiens
84750	fucosyltransferase 10 (alpha (1,3) fucosyltransferase)	Homo sapiens
51816	cat eye syndrome chromosome region, candidate 1	Homo sapiens
27340	similar to Down-regulated in metastasis protein (Key-1A6 protein) (Novel nucleolar protein 73) (NNP73); UTP20, small subunit (SSU) processome component, homolog (yeast)	Homo sapiens
653877	similar to Down-regulated in metastasis protein (Key-1A6 protein) (Novel nucleolar protein 73) (NNP73); UTP20, small subunit (SSU) processome component, homolog (yeast)	Homo sapiens
84135	UTP15, U3 small nucleolar ribonucleoprotein, homolog (S. cerevisiae)	Homo sapiens
408	arrestin, beta 1	Homo sapiens
56257	methylphosphate capping enzyme	Homo sapiens
5981	replication factor C (activator 1) 1, 145kDa	Homo sapiens
23049	SMG1 homolog, phosphatidylinositol 3-kinase-related kinase (C. elegans)	Homo sapiens
84720	phosphatidylinositol glycan anchor biosynthesis, class O	Homo sapiens
472	similar to Serine-protein kinase ATM (Ataxia telangiectasia mutated) (A-T, mutated); ataxia telangiectasia mutated	Homo sapiens

651610	similar to Serine-protein kinase ATM (Ataxia telangiectasia mutated) (A-T, mutated); ataxia telangiectasia mutated	Homo sapiens
80315	cytoplasmic polyadenylation element binding protein 4	Homo sapiens
57154	SMAD specific E3 ubiquitin protein ligase 1	Homo sapiens
9322	thyroid hormone receptor interactor 10	Homo sapiens
283643	chromosome 14 open reading frame 80	Homo sapiens
84146	zinc finger protein 644	Homo sapiens
728642	similar to cell division cycle 2-like 1 (PITSLRE proteins); cell division cycle 2-like 1 (PITSLRE proteins); cell division cycle 2-like 2 (PITSLRE proteins)	Homo sapiens
984	similar to cell division cycle 2-like 1 (PITSLRE proteins); cell division cycle 2-like 1 (PITSLRE proteins); cell division cycle 2-like 2 (PITSLRE proteins)	Homo sapiens
100133692	similar to cell division cycle 2-like 1 (PITSLRE proteins); cell division cycle 2-like 1 (PITSLRE proteins); cell division cycle 2-like 2 (PITSLRE proteins)	Homo sapiens
55334	solute carrier family 39 (zinc transporter), member 9	Homo sapiens
29767	tropomodulin 2 (neuronal)	Homo sapiens
375057	chromosome 1 open reading frame 95	Homo sapiens
9140	ATG12 autophagy related 12 homolog (S. cerevisiae)	Homo sapiens
5158	phosphodiesterase 6B, cGMP-specific, rod, beta	Homo sapiens
84823	lamin B2	Homo sapiens
1368	carboxypeptidase M	Homo sapiens
3836	karyopherin alpha 1 (importin alpha 5)	Homo sapiens
5873	RAB27A, member RAS oncogene family	Homo sapiens
29927	Sec61 alpha 1 subunit (S. cerevisiae)	Homo sapiens
10409	brain abundant, membrane attached signal protein 1	Homo sapiens
27436	echinoderm microtubule associated protein like 4	Homo sapiens
79966	stearoyl-CoA desaturase 5	Homo sapiens
8668	eukaryotic translation initiation factor 3, subunit I	Homo sapiens
80067	chromosome 2 open reading frame 37	Homo sapiens
128338	DNA-damage regulated autophagy modulator 2	Homo sapiens
3092	huntingtin interacting protein 1	Homo sapiens
9360	peptidylprolyl isomerase G (cyclophilin G)	Homo sapiens
90231	KIAA2013; hypothetical LOC728138	Homo sapiens
728138	KIAA2013; hypothetical LOC728138	Homo sapiens
8780	RIO kinase 3 (yeast)	Homo sapiens
91369	ankyrin repeat domain 40	Homo sapiens
5549	proline/arginine-rich end leucine-rich repeat protein	Homo sapiens
221	aldehyde dehydrogenase 3 family, member B1	Homo sapiens
5155	platelet-derived growth factor beta polypeptide (simian sarcoma viral (v-sis) oncogene homolog)	Homo sapiens
81631	microtubule-associated protein 1 light chain 3 beta	Homo sapiens
389	ras homolog gene family, member C	Homo sapiens
55920	regulator of chromosome condensation 2	Homo sapiens
783	calcium channel, voltage-dependent, beta 2 subunit	Homo sapiens
5688	proteasome (prosome, macropain) subunit, alpha type, 7	Homo sapiens
54664	transmembrane protein 106B	Homo sapiens
51742	AT rich interactive domain 4B (RBP1-like)	Homo sapiens
79621	ribonuclease H2, subunit B	Homo sapiens
29943	peptidyl arginine deiminase, type I	Homo sapiens
1282	collagen, type IV, alpha 1	Homo sapiens
4092	SMAD family member 7	Homo sapiens
79794	chromosome 12 open reading frame 49	Homo sapiens
22887	forkhead box J3	Homo sapiens
6672	SP100 nuclear antigen	Homo sapiens
56977	storkhead box 2	Homo sapiens
25842	ASF1 anti-silencing function 1 homolog A (S. cerevisiae)	Homo sapiens
7092	tolloid-like 1	Homo sapiens
8930	methyl-CpG binding domain protein 4	Homo sapiens
5306	phosphatidylinositol transfer protein, alpha	Homo sapiens
6521	solute carrier family 4, anion exchanger, member 1 (erythrocyte membrane protein band 3, Diego blood group)	Homo sapiens
5915	retinoic acid receptor, beta	Homo sapiens
25902	methylenetetrahydrofolate dehydrogenase (NADP+ dependent) 1-like	Homo sapiens
401474	sterile alpha motif domain containing 12	Homo sapiens
7691	zinc finger protein 132	Homo sapiens
57690	trinucleotide repeat containing 6C	Homo sapiens
3326	heat shock protein 90kDa alpha (cytosolic), class B member 1	Homo sapiens
285676	zinc finger protein 454	Homo sapiens
7473	wingless-type MMTV integration site family, member 3	Homo sapiens
140453	mucin 17, cell surface associated	Homo sapiens
8897	myotubularin related protein 3	Homo sapiens
142891	sterile alpha motif domain containing 8	Homo sapiens
9527	golgi SNAP receptor complex member 1	Homo sapiens
84908	family with sequence similarity 136, member A	Homo sapiens
4298	myeloid/lymphoid or mixed-lineage leukemia (trithorax homolog, Drosophila); translocated to, 1	Homo sapiens
57798	GATA zinc finger domain containing 1	Homo sapiens
162989	death effector domain containing 2	Homo sapiens
6002	regulator of G-protein signaling 12	Homo sapiens
4067	v-yes-1 Yamaguchi sarcoma viral related oncogene homolog	Homo sapiens

57537	sortilin-related VPS10 domain containing receptor 2	Homo sapiens
8326	frizzled homolog 9 (Drosophila)	Homo sapiens
23670	transmembrane protein 2	Homo sapiens
23225	nucleoporin 210kDa	Homo sapiens
10111	RAD50 homolog (S. cerevisiae)	Homo sapiens
3611	integrin-linked kinase	Homo sapiens
150921	transcription factor 23	Homo sapiens
54929	transmembrane protein 161A	Homo sapiens
6389	succinate dehydrogenase complex, subunit A, flavoprotein (Fp)	Homo sapiens
5887	RAD23 homolog B (S. cerevisiae)	Homo sapiens
23527	ArfGAP with coiled-coil, ankyrin repeat and PH domains 2	Homo sapiens
83452	RAB33B, member RAS oncogene family	Homo sapiens
4335	MAX binding protein	Homo sapiens
4172	minichromosome maintenance complex component 3	Homo sapiens
55858	transmembrane protein 165	Homo sapiens
4711	NADH dehydrogenase (ubiquinone) 1 beta subcomplex, 5, 16kDa	Homo sapiens
10061	ATP-binding cassette, sub-family F (GCN20), member 2	Homo sapiens
30000	transportin 2	Homo sapiens
5876	Rab geranylgeranyltransferase, beta subunit	Homo sapiens
1946	ephrin-A5	Homo sapiens
153020	RasGEF domain family, member 1B	Homo sapiens
5718	proteasome (prosome, macropain) 26S subunit, non-ATPase, 12	Homo sapiens
26127	FGFR1 oncogene partner 2	Homo sapiens
6845	vesicle-associated membrane protein 7	Homo sapiens
55148	ubiquitin protein ligase E3 component n-recognin 7 (putative)	Homo sapiens
518	ATP synthase, H+ transporting, mitochondrial F0 complex, subunit C3 (subunit 9)	Homo sapiens
51715	RAB23, member RAS oncogene family	Homo sapiens
9632	SEC24 family, member C (S. cerevisiae)	Homo sapiens
2878	glutathione peroxidase 3 (plasma)	Homo sapiens
8943	adaptor-related protein complex 3, delta 1 subunit	Homo sapiens
57333	reticulocalbin 3, EF-hand calcium binding domain	Homo sapiens
51761	ATPase, aminophospholipid transporter-like, class I, type 8A, member 2	Homo sapiens
3096	human immunodeficiency virus type I enhancer binding protein 1	Homo sapiens
4947	ornithine decarboxylase antizyme 2	Homo sapiens
23137	structural maintenance of chromosomes 5	Homo sapiens
10317	UDP-Gal:betaGlcNAc beta 1,3-galactosyltransferase, polypeptide 5	Homo sapiens
7247	translin	Homo sapiens
84267	chromosome 9 open reading frame 64	Homo sapiens
619279	zinc finger protein 704	Homo sapiens
144108	SPT2, Suppressor of Ty, domain containing 1 (S. cerevisiae)	Homo sapiens
140775	Smith-Magenis syndrome chromosome region, candidate 8	Homo sapiens
9519	TBP-like 1	Homo sapiens
57119	WAP four-disulfide core domain 6; serine peptidase inhibitor-like, with Kunitz and WAP domains 1 (eppin)	Homo sapiens
140870	WAP four-disulfide core domain 6; serine peptidase inhibitor-like, with Kunitz and WAP domains 1 (eppin)	Homo sapiens
57122	nucleoporin 107kDa	Homo sapiens
898	cyclin E1	Homo sapiens
22889	KIAA0907	Homo sapiens
64132	xylosyltransferase II	Homo sapiens
10657	KH domain containing, RNA binding, signal transduction associated 1	Homo sapiens
27031	nephronophthisis 3 (adolescent); acyl-Coenzyme A dehydrogenase family, member 11	Homo sapiens
84129	nephronophthisis 3 (adolescent); acyl-Coenzyme A dehydrogenase family, member 11	Homo sapiens
64924	solute carrier family 30 (zinc transporter), member 5	Homo sapiens
388531	regulator of G protein signaling 9 binding protein	Homo sapiens
728013	COBW domain containing 5; similar to COBW domain containing 1; COBW domain containing 7; COBW domain containing 3	Homo sapiens
653510	COBW domain containing 5; similar to COBW domain containing 1; COBW domain containing 7; COBW domain containing 3	Homo sapiens
445571	COBW domain containing 5; similar to COBW domain containing 1; COBW domain containing 7; COBW domain containing 3	Homo sapiens
220869	COBW domain containing 5; similar to COBW domain containing 1; COBW domain containing 7; COBW domain containing 3	Homo sapiens
23645	protein phosphatase 1, regulatory (inhibitor) subunit 15A	Homo sapiens
57509	mitochondrial tumor suppressor 1	Homo sapiens
8704	UDP-Gal:betaGlcNAc beta 1,4- galactosyltransferase, polypeptide 2	Homo sapiens
121268	Ras homolog enriched in brain like 1	Homo sapiens
23438	histidyl-tRNA synthetase 2, mitochondrial (putative); D-tyrosyl-tRNA deacylase 1 homolog (S. cerevisiae)	Homo sapiens
92675	histidyl-tRNA synthetase 2, mitochondrial (putative); D-tyrosyl-tRNA deacylase 1 homolog (S. cerevisiae)	Homo sapiens
29907	sorting nexin 15	Homo sapiens
25932	chloride intracellular channel 4	Homo sapiens
25987	tsukushin	Homo sapiens
125111	gap junction protein, delta 3, 31.9kDa	Homo sapiens
57700	family with sequence similarity 160, member B1	Homo sapiens
28966	sorting nexin 24	Homo sapiens
155382	vacuolar protein sorting 37 homolog D (S. cerevisiae)	Homo sapiens
10263	cyclin-dependent kinase 2 associated protein 2	Homo sapiens

26051	protein phosphatase 1, regulatory (inhibitor) subunit 16B	Homo sapiens
9761	malectin	Homo sapiens
146434	zinc finger protein 597	Homo sapiens
205564	SUMO1/sentrin specific peptidase 5	Homo sapiens
125950	ribonucleoprotein, PTB-binding 1	Homo sapiens
6753	somatostatin receptor 3	Homo sapiens
1969	EPH receptor A2	Homo sapiens
3206	homeobox A10	Homo sapiens
2936	glutathione reductase	Homo sapiens
57716	periaxin	Homo sapiens
51272	blocked early in transport 1 homolog (S. cerevisiae)-like	Homo sapiens
93099	dermokine	Homo sapiens
5140	phosphodiesterase 3B, cGMP-inhibited	Homo sapiens
79088	zinc finger protein 426	Homo sapiens
1033	cyclin-dependent kinase inhibitor 3	Homo sapiens
8666	eukaryotic translation initiation factor 3, subunit G	Homo sapiens
390927	zinc finger protein 793	Homo sapiens
5871	mitogen-activated protein kinase kinase kinase kinase 2	Homo sapiens
9792	SERTA domain containing 2	Homo sapiens
9600	phosphatidylinositol transfer protein, membrane-associated 1	Homo sapiens
54676	GTP binding protein 2	Homo sapiens
11201	polymerase (DNA directed) iota	Homo sapiens
389072	pleckstrin homology domain containing, family M, member 3	Homo sapiens
5029	purinergic receptor P2Y, G-protein coupled, 2	Homo sapiens
7328	ubiquitin-conjugating enzyme E2H (UBC8 homolog, yeast)	Homo sapiens
9975	nuclear receptor subfamily 1, group D, member 2	Homo sapiens
861	runt-related transcription factor 1	Homo sapiens
7441	pre-B lymphocyte 1	Homo sapiens
114884	oxysterol binding protein-like 10	Homo sapiens
9980	dopey family member 2	Homo sapiens
56946	chromosome 11 open reading frame 30	Homo sapiens
163732	Cbp/p300-interacting transactivator, with Glu/Asp-rich carboxy-terminal domain, 4	Homo sapiens
949	scavenger receptor class B, member 1	Homo sapiens
64241	ATP-binding cassette, sub-family G (WHITE), member 8	Homo sapiens
10314	LanC lantibiotic synthetase component C-like 1 (bacterial)	Homo sapiens
26146	TNF receptor-associated factor 3 interacting protein 1	Homo sapiens
79954	nucleolar protein 10	Homo sapiens
4659	protein phosphatase 1, regulatory (inhibitor) subunit 12A	Homo sapiens
158405	KIAA1958	Homo sapiens
9908	GTPase activating protein (SH3 domain) binding protein 2	Homo sapiens
23095	kinesin family member 1B	Homo sapiens
9749	phosphatase and actin regulator 2	Homo sapiens
80789	integrator complex subunit 5	Homo sapiens
10261	immunoglobulin superfamily, member 6	Homo sapiens
22877	MLX interacting protein	Homo sapiens
5865	RAB3B, member RAS oncogene family	Homo sapiens
23195	MDN1, midasin homolog (yeast)	Homo sapiens
134145	family with sequence similarity 173, member B	Homo sapiens
9124	PDZ and LIM domain 1	Homo sapiens
92755	tubulin, beta; similar to tubulin, beta 5; tubulin, beta pseudogene 2; tubulin, beta pseudogene 1	Homo sapiens
647000	tubulin, beta; similar to tubulin, beta 5; tubulin, beta pseudogene 2; tubulin, beta pseudogene 1	Homo sapiens
442308	tubulin, beta; similar to tubulin, beta 5; tubulin, beta pseudogene 2; tubulin, beta pseudogene 1	Homo sapiens
203068	tubulin, beta; similar to tubulin, beta 5; tubulin, beta pseudogene 2; tubulin, beta pseudogene 1	Homo sapiens
4082	myristoylated alanine-rich protein kinase C substrate	Homo sapiens
152831	klotho beta	Homo sapiens
2752	glutamate-ammonia ligase (glutamine synthetase)	Homo sapiens
5702	proteasome (prosome, macropain) 26S subunit, ATPase, 3	Homo sapiens
26524	LATS, large tumor suppressor, homolog 2 (Drosophila)	Homo sapiens
79056	proline rich Gla (G-carboxyglutamic acid) 4 (transmembrane)	Homo sapiens
22895	rabphilin 3A homolog (mouse)	Homo sapiens
51018	ribosomal RNA processing 15 homolog (S. cerevisiae)	Homo sapiens
643427	RNA binding motif, single stranded interacting protein 2; RNA binding motif, single stranded interacting protein 2 pseudogene	Homo sapiens
5939	RNA binding motif, single stranded interacting protein 2; RNA binding motif, single stranded interacting protein 2 pseudogene	Homo sapiens
401494	protein tyrosine phosphatase-like A domain containing 2	Homo sapiens
9697	translocation associated membrane protein 2	Homo sapiens
130026	islet cell autoantigen 1,69kDa-like	Homo sapiens
7014	telomeric repeat binding factor 2	Homo sapiens
79147	fukutin related protein	Homo sapiens
10333	toll-like receptor 6	Homo sapiens
7355	solute carrier family 35 (UDP-galactose transporter), member A2	Homo sapiens
4853	Notch homolog 2 (Drosophila)	Homo sapiens
23025	unc-13 homolog A (C. elegans)	Homo sapiens
6494	signal-induced proliferation-associated 1	Homo sapiens

22954	tripartite motif-containing 32	Homo sapiens
226	aldolase A, fructose-bisphosphate	Homo sapiens
2821	glucose phosphate isomerase	Homo sapiens
7410	vav 2 guanine nucleotide exchange factor	Homo sapiens
29080	coiled-coil domain containing 59	Homo sapiens
643167	similar to RNA binding motif protein 39; RNA binding motif protein 39	Homo sapiens
9584	similar to RNA binding motif protein 39; RNA binding motif protein 39	Homo sapiens
84458	ligand dependent nuclear receptor corepressor	Homo sapiens
10206	tripartite motif-containing 13	Homo sapiens
9182	Ras association (RalGDS/AF-6) domain family (N-terminal) member 9	Homo sapiens
84529	chromosome 15 open reading frame 41	Homo sapiens
3588	interleukin 10 receptor, beta	Homo sapiens
9853	RUN and SH3 domain containing 2	Homo sapiens
84285	eukaryotic translation initiation factor 1A domain containing	Homo sapiens
64418	transmembrane protein 168	Homo sapiens
79038	zinc finger, FYVE domain containing 21	Homo sapiens
147694	zinc finger protein 548	Homo sapiens
10060	ATP-binding cassette, sub-family C (CFTR/MRP), member 9	Homo sapiens
116496	family with sequence similarity 129, member A	Homo sapiens
54926	ubiquitin-conjugating enzyme E2R 2	Homo sapiens
8870	immediate early response 3	Homo sapiens
23450	splicing factor 3b, subunit 3, 130kDa	Homo sapiens
65981	caprin family member 2	Homo sapiens
8493	protein phosphatase 1D magnesium-dependent, delta isoform	Homo sapiens
654429	leucine-rich repeats and transmembrane domains 2	Homo sapiens
205327	chromosome 2 open reading frame 69	Homo sapiens
7874	ubiquitin specific peptidase 7 (herpes virus-associated)	Homo sapiens
84916	cirrhosis, autosomal recessive 1A (cirhin)	Homo sapiens
54788	DnaJ (Hsp40) homolog, subfamily B, member 12	Homo sapiens
5187	period homolog 1 (Drosophila)	Homo sapiens
1978	eukaryotic translation initiation factor 4E binding protein 1	Homo sapiens
85379	KIAA1671 protein	Homo sapiens
689	basic transcription factor 3; basic transcription factor 3, like 1 pseudogene	Homo sapiens
690	basic transcription factor 3; basic transcription factor 3, like 1 pseudogene	Homo sapiens
7764	zinc finger protein 217	Homo sapiens
730094	chromosome 16 open reading frame 52	Homo sapiens
55220	kelch domain containing 8A	Homo sapiens
114781	BTB (POZ) domain containing 9	Homo sapiens
80194	transmembrane protein 134	Homo sapiens
6482	ST3 beta-galactoside alpha-2,3-sialyltransferase 1	Homo sapiens
80311	kelch-like 15 (Drosophila)	Homo sapiens
596	B-cell CLL/lymphoma 2	Homo sapiens
79751	solute carrier family 25 (mitochondrial carrier: glutamate), member 22	Homo sapiens
983	cell division cycle 2, G1 to S and G2 to M	Homo sapiens
54476	ring finger protein 216	Homo sapiens
10963	stress-induced-phosphoprotein 1	Homo sapiens
7905	receptor accessory protein 5	Homo sapiens
11319	ecdysoneless homolog (Drosophila)	Homo sapiens
3638	insulin induced gene 1	Homo sapiens
22841	RAB11 family interacting protein 2 (class I)	Homo sapiens
8647	ATP-binding cassette, sub-family B (MDR/TAP), member 11	Homo sapiens
57475	pleckstrin homology domain containing, family H (with MyTH4 domain) member 1	Homo sapiens
54822	transient receptor potential cation channel, subfamily M, member 7	Homo sapiens
157570	establishment of cohesion 1 homolog 2 (S. cerevisiae)	Homo sapiens
4900	neurogranin (protein kinase C substrate, RC3)	Homo sapiens
2962	general transcription factor IIF, polypeptide 1, 74kDa	Homo sapiens
2184	fumarylacetoacetate hydrolase (fumarylacetoacetase)	Homo sapiens
5834	phosphorylase, glycogen; brain	Homo sapiens
58500	zinc finger protein 250	Homo sapiens
729993	hypothetical protein LOC729993	Homo sapiens
2348	folate receptor 1 (adult)	Homo sapiens
4174	minichromosome maintenance complex component 5	Homo sapiens
207063	dehydrogenase/reductase (SDR family) X-linked	Homo sapiens
81609	sorting nexin family member 27	Homo sapiens
100134387	similar to DEAH (Asp-Glu-Ala-His) box polypeptide 40; DEAH (Asp-Glu-Ala-His) box polypeptide 40	Homo sapiens
79665	similar to DEAH (Asp-Glu-Ala-His) box polypeptide 40; DEAH (Asp-Glu-Ala-His) box polypeptide 40	Homo sapiens
6585	slit homolog 1 (Drosophila)	Homo sapiens
6399	trafficking protein particle complex 2; trafficking protein particle complex 2 pseudogene 1	Homo sapiens
10597	trafficking protein particle complex 2; trafficking protein particle complex 2 pseudogene 1	Homo sapiens
160364	C-type lectin domain family 12, member A; C-type lectin domain family 12, member B	Homo sapiens
387837	C-type lectin domain family 12, member A; C-type lectin domain family 12, member B	Homo sapiens
79675	FAST kinase domains 1	Homo sapiens
3662	interferon regulatory factor 4	Homo sapiens
23324	mannosidase, alpha, class 2B, member 2	Homo sapiens
3752	potassium voltage-gated channel, Shal-related subfamily, member 3	Homo sapiens

3237	homeobox D11	Homo sapiens
5198	phosphoribosylformylglycinamidine synthase	Homo sapiens
80704	solute carrier family 19, member 3	Homo sapiens
256471	major facilitator superfamily domain containing 8	Homo sapiens
4124	mannosidase, alpha, class 2A, member 1	Homo sapiens
22944	KIN, antigenic determinant of recA protein homolog (mouse)	Homo sapiens
4099	myelin associated glycoprotein	Homo sapiens
5310	polycystic kidney disease 1 (autosomal dominant)	Homo sapiens
51593	serrate RNA effector molecule homolog (Arabidopsis)	Homo sapiens
10849	CD3e molecule, epsilon associated protein	Homo sapiens
220213	OTU domain containing 1	Homo sapiens
51199	ninein (GSK3B interacting protein)	Homo sapiens
5480	peptidylprolyl isomerase C (cyclophilin C)	Homo sapiens
9175	mitogen-activated protein kinase kinase kinase 13	Homo sapiens
3028	hydroxysteroid (17-beta) dehydrogenase 10	Homo sapiens
23101	MCF.2 cell line derived transforming sequence-like 2	Homo sapiens
5982	replication factor C (activator 1) 2, 40kDa	Homo sapiens
3054	host cell factor C1 (VP16-accessory protein)	Homo sapiens
26994	ring finger protein 11	Homo sapiens
729839	similar to DTW domain containing 2; DTW domain containing 2	Homo sapiens
285605	similar to DTW domain containing 2; DTW domain containing 2	Homo sapiens
130507	ubiquitin protein ligase E3 component n-recognin 3 (putative)	Homo sapiens
728127	ArfGAP with GTPase domain, ankyrin repeat and PH domain 5; ArfGAP with GTPase domain, ankyrin repeat and PH domain 9; ArfGAP with GTPase domain, ankyrin repeat and PH domain 10; centaurin, gamma-like family, member 11 pseudogene	Homo sapiens
729092	ArfGAP with GTPase domain, ankyrin repeat and PH domain 5; ArfGAP with GTPase domain, ankyrin repeat and PH domain 9; ArfGAP with GTPase domain, ankyrin repeat and PH domain 10; centaurin, gamma-like family, member 11 pseudogene	Homo sapiens
653259	ArfGAP with GTPase domain, ankyrin repeat and PH domain 5; ArfGAP with GTPase domain, ankyrin repeat and PH domain 9; ArfGAP with GTPase domain, ankyrin repeat and PH domain 10; centaurin, gamma-like family, member 11 pseudogene	Homo sapiens
642517	ArfGAP with GTPase domain, ankyrin repeat and PH domain 5; ArfGAP with GTPase domain, ankyrin repeat and PH domain 9; ArfGAP with GTPase domain, ankyrin repeat and PH domain 10; centaurin, gamma-like family, member 11 pseudogene	Homo sapiens
27	v-abl Abelson murine leukemia viral oncogene homolog 2 (arg, Abelson-related gene)	Homo sapiens
5292	pim-1 oncogene	Homo sapiens
3920	lysosomal-associated membrane protein 2	Homo sapiens
5990	regulatory factor X, 2 (influences HLA class II expression)	Homo sapiens
5728	phosphatase and tensin homolog; phosphatase and tensin homolog pseudogene 1	Homo sapiens
11191	phosphatase and tensin homolog; phosphatase and tensin homolog pseudogene 1	Homo sapiens
91851	chordin-like 1	Homo sapiens
9801	mitochondrial ribosomal protein L19	Homo sapiens
26586	cytoskeleton associated protein 2	Homo sapiens
10981	RAB32, member RAS oncogene family	Homo sapiens
25914	rotatin	Homo sapiens
642538	C1D nuclear receptor co-repressor; similar to nuclear DNA-binding protein; similar to hCG1791993	Homo sapiens
642521	C1D nuclear receptor co-repressor; similar to nuclear DNA-binding protein; similar to hCG1791993	Homo sapiens
10438	C1D nuclear receptor co-repressor; similar to nuclear DNA-binding protein; similar to hCG1791993	Homo sapiens
219743	trypsin domain containing 1	Homo sapiens
100101267	POM121 membrane glycoprotein C; POM121 membrane glycoprotein B (pseudogene)	Homo sapiens
729316	POM121 membrane glycoprotein C; POM121 membrane glycoprotein B (pseudogene)	Homo sapiens
64121	Ras-related GTP binding C	Homo sapiens
26036	zinc finger protein 451	Homo sapiens
503582	arginine-fifty homeobox	Homo sapiens
23370	Rho/Rac guanine nucleotide exchange factor (GEF) 18	Homo sapiens
27332	zinc finger protein 638	Homo sapiens
26119	low density lipoprotein receptor adaptor protein 1	Homo sapiens
81848	sprouty homolog 4 (Drosophila)	Homo sapiens
7287	tubby like protein 1	Homo sapiens
10466	component of oligomeric golgi complex 5	Homo sapiens
9805	secernin 1	Homo sapiens
79366	nucleosomal binding protein 1	Homo sapiens
51149	chromosome 5 open reading frame 45	Homo sapiens
117246	FtsJ homolog 3 (E. coli)	Homo sapiens
55790	chondroitin sulfate N-acetylgalactosaminyltransferase 1	Homo sapiens
9101	ubiquitin specific peptidase 8	Homo sapiens
83903	germ cell associated 2 (haspin)	Homo sapiens
51230	PHD finger protein 20	Homo sapiens
94120	synaptotagmin-like 3	Homo sapiens
2831	neuropeptides B/W receptor 1	Homo sapiens
84239	ATPase type 13A4	Homo sapiens
283464	glycosyltransferase 8 domain containing 3	Homo sapiens
112840	WD repeat domain 89	Homo sapiens
4478	moesin	Homo sapiens
51312	solute carrier family 25, member 37	Homo sapiens
90809	transmembrane protein 55B	Homo sapiens

64852	terminal uridylyl transferase 1, U6 snRNA-specific	Homo sapiens
5889	RAD51 homolog C (S. cerevisiae)	Homo sapiens
85458	DIX domain containing 1	Homo sapiens
339500	zinc finger protein 678	Homo sapiens
60558	GUF1 GTPase homolog (S. cerevisiae)	Homo sapiens
6499	superkiller viralicidic activity 2-like (S. cerevisiae)	Homo sapiens
84336	transmembrane protein 101	Homo sapiens
9212	aurora kinase B	Homo sapiens
2589	UDP-N-acetyl-alpha-D-galactosamine:polypeptide N-acetylgalactosaminyltransferase 13 (GalNAc-T13); UDP-N-acetyl-alpha-D-galactosamine:polypeptide N-acetylgalactosaminyltransferase 1 (GalNAc-T1)	Homo sapiens
114805	UDP-N-acetyl-alpha-D-galactosamine:polypeptide N-acetylgalactosaminyltransferase 13 (GalNAc-T13); UDP-N-acetyl-alpha-D-galactosamine:polypeptide N-acetylgalactosaminyltransferase 1 (GalNAc-T1)	Homo sapiens
2019	engrailed homeobox 1	Homo sapiens
51429	sorting nexin 9	Homo sapiens
2444	fyn-related kinase	Homo sapiens
653598	peptidylprolyl isomerase A (cyclophilin A)-like 4E; peptidylprolyl isomerase A (cyclophilin A)-like 4G; peptidylprolyl isomerase A (cyclophilin A)-like 4F; peptidylprolyl isomerase A (cyclophilin A)-like 4A; peptidylprolyl isomerase A (cyclophilin A)-like 4C; peptidylprolyl isomerase A (cyclophilin A)-like 4B	Homo sapiens
730262	peptidylprolyl isomerase A (cyclophilin A)-like 4E; peptidylprolyl isomerase A (cyclophilin A)-like 4G; peptidylprolyl isomerase A (cyclophilin A)-like 4F; peptidylprolyl isomerase A (cyclophilin A)-like 4A; peptidylprolyl isomerase A (cyclophilin A)-like 4C; peptidylprolyl isomerase A (cyclophilin A)-like 4B	Homo sapiens
644591	peptidylprolyl isomerase A (cyclophilin A)-like 4E; peptidylprolyl isomerase A (cyclophilin A)-like 4G; peptidylprolyl isomerase A (cyclophilin A)-like 4F; peptidylprolyl isomerase A (cyclophilin A)-like 4A; peptidylprolyl isomerase A (cyclophilin A)-like 4C; peptidylprolyl isomerase A (cyclophilin A)-like 4B	Homo sapiens
653505	peptidylprolyl isomerase A (cyclophilin A)-like 4E; peptidylprolyl isomerase A (cyclophilin A)-like 4G; peptidylprolyl isomerase A (cyclophilin A)-like 4F; peptidylprolyl isomerase A (cyclophilin A)-like 4A; peptidylprolyl isomerase A (cyclophilin A)-like 4C; peptidylprolyl isomerase A (cyclophilin A)-like 4B	Homo sapiens
164022	peptidylprolyl isomerase A (cyclophilin A)-like 4E; peptidylprolyl isomerase A (cyclophilin A)-like 4G; peptidylprolyl isomerase A (cyclophilin A)-like 4F; peptidylprolyl isomerase A (cyclophilin A)-like 4A; peptidylprolyl isomerase A (cyclophilin A)-like 4C; peptidylprolyl isomerase A (cyclophilin A)-like 4B	Homo sapiens
728945	peptidylprolyl isomerase A (cyclophilin A)-like 4E; peptidylprolyl isomerase A (cyclophilin A)-like 4G; peptidylprolyl isomerase A (cyclophilin A)-like 4F; peptidylprolyl isomerase A (cyclophilin A)-like 4A; peptidylprolyl isomerase A (cyclophilin A)-like 4C; peptidylprolyl isomerase A (cyclophilin A)-like 4B	Homo sapiens
4064	CD180 molecule	Homo sapiens
5356	pleiotropic regulator 1 (PRL1 homolog, Arabidopsis)	Homo sapiens
57404	cytochrome P450, family 20, subfamily A, polypeptide 1	Homo sapiens
548596	creatine kinase, mitochondrial 1A; creatine kinase, mitochondrial 1B	Homo sapiens
1159	creatine kinase, mitochondrial 1A; creatine kinase, mitochondrial 1B	Homo sapiens
84034	elastin microfibril interfacer 2	Homo sapiens
54905	cytochrome P450, family 2, subfamily W, polypeptide 1	Homo sapiens
26031	oxysterol binding protein-like 3	Homo sapiens
50937	Cdon homolog (mouse)	Homo sapiens
4691	nucleolin	Homo sapiens
56342	peter pan homolog (Drosophila)	Homo sapiens
6299	sal-like 1 (Drosophila)	Homo sapiens
3652	intracisternal A particle-promoted polypeptide	Homo sapiens
5701	proteasome (prosome, macropain) 26S subunit, ATPase, 2	Homo sapiens
27075	tetraspanin 13	Homo sapiens
6919	transcription elongation factor A (SII), 2	Homo sapiens
256815	chromosome 10 open reading frame 67	Homo sapiens
29796	ubiquinol-cytochrome c reductase complex (7.2 kD)	Homo sapiens
2551	GA binding protein transcription factor, alpha subunit 60kDa	Homo sapiens
115752	DIS3 mitotic control homolog (S. cerevisiae)-like	Homo sapiens
23184	mesoderm development candidate 2	Homo sapiens
51067	tyrosyl-tRNA synthetase 2, mitochondrial	Homo sapiens
1289	collagen, type V, alpha 1	Homo sapiens
79990	pleckstrin homology domain containing, family H (with MyTH4 domain) member 3	Homo sapiens
54732	transmembrane emp24 protein transport domain containing 9	Homo sapiens
91	activin A receptor, type IB	Homo sapiens
1509	cathepsin D	Homo sapiens
29028	ATPase family, AAA domain containing 2	Homo sapiens
54707	GPN-loop GTPase 2	Homo sapiens
23204	ADP-ribosylation factor-like 6 interacting protein 1	Homo sapiens
637	BH3 interacting domain death agonist	Homo sapiens
81537	sphingosine-1-phosphate phosphatase 1	Homo sapiens
388336	FLJ45455 protein	Homo sapiens
11072	dual specificity phosphatase 14	Homo sapiens
51661	FK506 binding protein 7	Homo sapiens
7276	transthyretin	Homo sapiens
57559	STAM binding protein-like 1	Homo sapiens
2030	solute carrier family 29 (nucleoside transporters), member 1	Homo sapiens

57224	NHS-like 1	Homo sapiens
54900	lymphocyte transmembrane adaptor 1	Homo sapiens
23608	makorin ring finger protein pseudogene 6; makorin ring finger protein 1	Homo sapiens
400058	makorin ring finger protein pseudogene 6; makorin ring finger protein 1	Homo sapiens
79230	zinc finger protein 557	Homo sapiens
53944	casein kinase 1, gamma 1	Homo sapiens
55244	solute carrier family 47, member 1	Homo sapiens
6760	synovial sarcoma translocation, chromosome 18	Homo sapiens
22826	DnaJ (Hsp40) homolog, subfamily C, member 8	Homo sapiens
5789	protein tyrosine phosphatase, receptor type, D	Homo sapiens
57157	putative homeodomain transcription factor 2	Homo sapiens
65977	pleckstrin homology domain containing, family A (phosphoinositide binding specific) member 3	Homo sapiens
128	alcohol dehydrogenase 5 (class III), chi polypeptide, pseudogene 4; alcohol dehydrogenase 5 (class III), chi polypeptide	Homo sapiens
642443	alcohol dehydrogenase 5 (class III), chi polypeptide, pseudogene 4; alcohol dehydrogenase 5 (class III), chi polypeptide	Homo sapiens
5515	protein phosphatase 2 (formerly 2A), catalytic subunit, alpha isoform	Homo sapiens
30833	5', 3'-nucleotidase, cytosolic	Homo sapiens
221687	ring finger protein 182	Homo sapiens
9869	SET domain, bifurcated 1	Homo sapiens
8655	dynein, light chain, LC8-type 1	Homo sapiens
57161	pellino homolog 2 (Drosophila)	Homo sapiens
818	calcium/calmodulin-dependent protein kinase II gamma	Homo sapiens
83986	integrin alpha FG-GAP repeat containing 3	Homo sapiens
5685	proteasome (prosome, macropain) subunit, alpha type, 4	Homo sapiens
81567	thioredoxin domain containing 5 (endoplasmic reticulum); muted homolog (mouse)	Homo sapiens
63915	thioredoxin domain containing 5 (endoplasmic reticulum); muted homolog (mouse)	Homo sapiens
9328	general transcription factor IIIC, polypeptide 5, 63kDa	Homo sapiens
8614	stanniocalcin 2	Homo sapiens
9254	calcium channel, voltage-dependent, alpha 2/delta subunit 2	Homo sapiens
11216	A kinase (PRKA) anchor protein 10	Homo sapiens
81570	ClpB caseinolytic peptidase B homolog (E. coli)	Homo sapiens
3035	histidyl-tRNA synthetase	Homo sapiens
222194	round spermatid basic protein 1-like	Homo sapiens
27000	DnaJ (Hsp40) homolog, subfamily C, member 2	Homo sapiens
442454	similar to ubiquinol-cytochrome c reductase binding protein; ubiquinol-cytochrome c reductase binding protein pseudogene; ubiquinol-cytochrome c reductase binding protein	Homo sapiens
727947	similar to ubiquinol-cytochrome c reductase binding protein; ubiquinol-cytochrome c reductase binding protein pseudogene; ubiquinol-cytochrome c reductase binding protein	Homo sapiens
7381	similar to ubiquinol-cytochrome c reductase binding protein; ubiquinol-cytochrome c reductase binding protein pseudogene; ubiquinol-cytochrome c reductase binding protein	Homo sapiens
9928	kinesin family member 14	Homo sapiens
100128046	hypothetical LOC100128046	Homo sapiens
81552	similar to EGFR-coamplified and overexpressed protein; EGFR-coamplified and overexpressed protein	Homo sapiens
729086	similar to EGFR-coamplified and overexpressed protein; EGFR-coamplified and overexpressed protein	Homo sapiens
9377	cytochrome c oxidase subunit Va	Homo sapiens
84287	zinc finger, DHHC-type containing 16	Homo sapiens
23082	peroxisome proliferator-activated receptor gamma, coactivator-related 1	Homo sapiens
54874	formin binding protein 1-like	Homo sapiens
54921	CTF8, chromosome transmission fidelity factor 8 homolog (S. cerevisiae)	Homo sapiens
60528	elaC homolog 2 (E. coli)	Homo sapiens
51316	placenta-specific 8	Homo sapiens
5965	RecQ protein-like (DNA helicase Q1-like)	Homo sapiens
54778	ring finger protein 111	Homo sapiens
139818	dedicator of cytokinesis 11	Homo sapiens
5742	prostaglandin-endoperoxide synthase 1 (prostaglandin G/H synthase and cyclooxygenase)	Homo sapiens
284467	family with sequence similarity 19 (chemokine (C-C motif)-like), member A3	Homo sapiens
54882	ankyrin repeat and KH domain containing 1; ANKHD1-EIF4EBP3 readthrough transcript; eukaryotic translation initiation factor 4E binding protein 3	Homo sapiens
8637	ankyrin repeat and KH domain containing 1; ANKHD1-EIF4EBP3 readthrough transcript; eukaryotic translation initiation factor 4E binding protein 3	Homo sapiens
404734	ankyrin repeat and KH domain containing 1; ANKHD1-EIF4EBP3 readthrough transcript; eukaryotic translation initiation factor 4E binding protein 3	Homo sapiens
65986	zinc finger and BTB domain containing 10	Homo sapiens
84376	hook homolog 3 (Drosophila)	Homo sapiens
374655	zinc finger protein 710	Homo sapiens
4609	v-myc myelocytomatosis viral oncogene homolog (avian)	Homo sapiens
23126	pogo transposable element with ZNF domain	Homo sapiens
6730	signal recognition particle 68kDa	Homo sapiens
31	acetyl-Coenzyme A carboxylase alpha	Homo sapiens
26172	chromosome 3 open reading frame 41; hypothetical protein LOC729085	Homo sapiens
729085	chromosome 3 open reading frame 41; hypothetical protein LOC729085	Homo sapiens
8563	THO complex 5	Homo sapiens
116987	ArfGAP with GTPase domain, ankyrin repeat and PH domain 1	Homo sapiens
84926	SPRY domain containing 3	Homo sapiens
55131	RNA binding motif protein 28	Homo sapiens

51015	isochorismatase domain containing 1	Homo sapiens
654790	Purkinje cell protein 4 like 1	Homo sapiens
51690	LSM7 homolog, U6 small nuclear RNA associated (S. cerevisiae)	Homo sapiens
9925	zinc finger and BTB domain containing 5	Homo sapiens
586	branched chain aminotransferase 1, cytosolic	Homo sapiens
6624	fascin homolog 1, actin-bundling protein (Strongylocentrotus purpuratus)	Homo sapiens
154007	small nuclear ribonucleoprotein 48kDa (U11/U12)	Homo sapiens
132946	ADP-ribosylation factor-like 9	Homo sapiens
6392	similar to succinate dehydrogenase complex, subunit D, integral membrane protein; succinate dehydrogenase complex, subunit D, integral membrane protein	Homo sapiens
100130320	similar to succinate dehydrogenase complex, subunit D, integral membrane protein; succinate dehydrogenase complex, subunit D, integral membrane protein	Homo sapiens
8242	lysine (K)-specific demethylase 5C	Homo sapiens
79068	fat mass and obesity associated	Homo sapiens
55423	signal-regulatory protein gamma	Homo sapiens
2018	empty spiracles homeobox 2	Homo sapiens
9830	tripartite motif-containing 14	Homo sapiens
51602	NOP58 ribonucleoprotein homolog (yeast)	Homo sapiens
9902	mannose receptor, C type 2	Homo sapiens
171017	zinc finger protein 384	Homo sapiens
2774	guanine nucleotide binding protein (G protein), alpha activating activity polypeptide, olfactory type	Homo sapiens
4512	Cytochrome c oxidase subunit 1	Homo sapiens
6775083	Cytochrome c oxidase subunit 1	Homo sapiens
23197	Fas associated factor family member 2	Homo sapiens
80232	WD repeat domain 26	Homo sapiens
55623	THUMP domain containing 1	Homo sapiens
23636	nucleoporin 62kDa	Homo sapiens
4088	SMAD family member 3	Homo sapiens
6789	serine/threonine kinase 4	Homo sapiens
730052	ubiquitin-conjugating enzyme E2 variant 1; ubiquitin-conjugating enzyme E2 variant 1 pseudogene 2; transmembrane protein 189; TMEM189-UBE2V1 readthrough transcript	Homo sapiens
7335	ubiquitin-conjugating enzyme E2 variant 1; ubiquitin-conjugating enzyme E2 variant 1 pseudogene 2; transmembrane protein 189; TMEM189-UBE2V1 readthrough transcript	Homo sapiens
387521	ubiquitin-conjugating enzyme E2 variant 1; ubiquitin-conjugating enzyme E2 variant 1 pseudogene 2; transmembrane protein 189; TMEM189-UBE2V1 readthrough transcript	Homo sapiens
387522	ubiquitin-conjugating enzyme E2 variant 1; ubiquitin-conjugating enzyme E2 variant 1 pseudogene 2; transmembrane protein 189; TMEM189-UBE2V1 readthrough transcript	Homo sapiens
392	Rho GTPase activating protein 1	Homo sapiens
284323	zinc finger protein 780A	Homo sapiens
1742	discs, large homolog 4 (Drosophila)	Homo sapiens
56655	polymerase (DNA-directed), epsilon 4 (p12 subunit)	Homo sapiens
84879	major facilitator superfamily domain containing 2	Homo sapiens
91574	chromosome 12 open reading frame 65	Homo sapiens
10318	TNFAIP3 interacting protein 1	Homo sapiens
8662	eukaryotic translation initiation factor 3, subunit B	Homo sapiens
55621	TRM1 tRNA methyltransferase 1 homolog (S. cerevisiae)	Homo sapiens
9807	inositol hexakisphosphate kinase 1	Homo sapiens
79180	EF-hand domain family, member D2	Homo sapiens
57191	vomeronasal 1 receptor 1	Homo sapiens
26190	F-box and WD repeat domain containing 2	Homo sapiens
10577	Niemann-Pick disease, type C2	Homo sapiens
11180	WD repeat domain 6	Homo sapiens
10335	murine retrovirus integration site 1 homolog	Homo sapiens
6009	Ras homolog enriched in brain	Homo sapiens
905	cyclin T2	Homo sapiens
7008	thyrotrophic embryonic factor	Homo sapiens
345651	actin, beta-like 2	Homo sapiens
159090	family with sequence similarity 122B	Homo sapiens
84295	PHD finger protein 6	Homo sapiens
80173	intraflagellar transport 74 homolog (Chlamydomonas)	Homo sapiens
5426	polymerase (DNA directed), epsilon	Homo sapiens
58526	MID1 interacting protein 1 (gastrulation specific G12 homolog (zebrafish))	Homo sapiens
56137	protocadherin alpha 12	Homo sapiens
27180	sialic acid binding Ig-like lectin 9	Homo sapiens
863	core-binding factor, runt domain, alpha subunit 2; translocated to, 3	Homo sapiens
115908	collagen triple helix repeat containing 1	Homo sapiens
55696	RNA binding motif protein 22	Homo sapiens
6004	regulator of G-protein signaling 16	Homo sapiens
4277	MHC class I polypeptide-related sequence B	Homo sapiens
4958	osteomodulin	Homo sapiens
26034	interaction protein for cytohesin exchange factors 1	Homo sapiens
150350	ENTH domain containing 1	Homo sapiens
57132	chromatin modifying protein 1B	Homo sapiens
84883	apoptosis-inducing factor, mitochondrion-associated, 2	Homo sapiens
57710	KIAA1614	Homo sapiens
6895	TAR (HIV-1) RNA binding protein 2	Homo sapiens
10452	translocase of outer mitochondrial membrane 40 homolog (yeast)	Homo sapiens
57120	golgi associated PDZ and coiled-coil motif containing	Homo sapiens

2274	four and a half LIM domains 2	Homo sapiens
2252	hypothetical LOC100132771; fibroblast growth factor 7 (keratinocyte growth factor); fibroblast growth factor 7 pseudogene 2	Homo sapiens
100132771	hypothetical LOC100132771; fibroblast growth factor 7 (keratinocyte growth factor); fibroblast growth factor 7 pseudogene 2	Homo sapiens
394217	hypothetical LOC100132771; fibroblast growth factor 7 (keratinocyte growth factor); fibroblast growth factor 7 pseudogene 2	Homo sapiens
7529	tyrosine 3-monooxygenase/tryptophan 5-monooxygenase activation protein, beta polypeptide	Homo sapiens
9024	BR serine/threonine kinase 2	Homo sapiens
51669	transmembrane protein 66	Homo sapiens
9144	synaptogyrin 2	Homo sapiens
5205	ATPase, class I, type 8B, member 1	Homo sapiens
123879	DCN1, defective in cullin neddylation 1, domain containing 3 (S. cerevisiae)	Homo sapiens
2960	general transcription factor IIE, polypeptide 1, alpha 56kDa	Homo sapiens
221079	ADP-ribosylation factor-like 5B	Homo sapiens
6601	SWI/SNF related, matrix associated, actin dependent regulator of chromatin, subfamily c, member 2	Homo sapiens
1054	CCAAT/enhancer binding protein (C/EBP), gamma	Homo sapiens
811	calreticulin	Homo sapiens
11116	FGFR1 oncogene partner	Homo sapiens
10049	DnaJ (Hsp40) homolog, subfamily B, member 6	Homo sapiens
1028	cyclin-dependent kinase inhibitor 1C (p57, Kip2)	Homo sapiens
5520	protein phosphatase 2 (formerly 2A), regulatory subunit B, alpha isoform	Homo sapiens
29115	SAP30 binding protein	Homo sapiens
219931	two pore segment channel 2	Homo sapiens
376497	solute carrier family 27 (fatty acid transporter), member 1	Homo sapiens
9957	heparan sulfate (glucosamine) 3-O-sulfotransferase 1	Homo sapiens
55505	NOP10 ribonucleoprotein homolog (yeast)	Homo sapiens
103	adenosine deaminase, RNA-specific	Homo sapiens
196441	zinc finger, C3H1-type containing	Homo sapiens
161003	stomatin (EPB72)-like 3	Homo sapiens
8277	transketolase-like 1	Homo sapiens
83857	transmembrane and tetratricopeptide repeat containing 1	Homo sapiens
57412	arsenic (+3 oxidation state) methyltransferase	Homo sapiens
54467	ankyrin repeat and IBR domain containing 1	Homo sapiens
119504	chromosome 10 open reading frame 104	Homo sapiens
10959	transmembrane emp24 domain trafficking protein 2	Homo sapiens
79591	chromosome 10 open reading frame 76	Homo sapiens
57111	RAB25, member RAS oncogene family	Homo sapiens
2128	even-skipped homeobox 1	Homo sapiens
23308	inducible T-cell co-stimulator ligand	Homo sapiens
90649	zinc finger protein 486	Homo sapiens
2580	cyclin G associated kinase	Homo sapiens
3337	DnaJ (Hsp40) homolog, subfamily B, member 1	Homo sapiens
26284	Era G-protein-like 1 (E. coli)	Homo sapiens
1073	cofilin 2 (muscle)	Homo sapiens
23677	SH3-domain binding protein 4	Homo sapiens
65979	phosphatase and actin regulator 4	Homo sapiens
56260	chromosome 8 open reading frame 44	Homo sapiens
5071	Parkinson disease (autosomal recessive, juvenile) 2, parkin	Homo sapiens
55074	oxidation resistance 1	Homo sapiens
2531	3-ketodihydrosphingosine reductase	Homo sapiens
6341	SCO cytochrome oxidase deficient homolog 1 (yeast)	Homo sapiens
7023	transcription factor AP-4 (activating enhancer binding protein 4)	Homo sapiens
410	arylsulfatase A	Homo sapiens
6821	sulfite oxidase	Homo sapiens
2235	ferrochelatase (protoporphyria)	Homo sapiens
127833	synaptotagmin II	Homo sapiens
55860	actin-related protein 10 homolog (S. cerevisiae)	Homo sapiens
7326	ubiquitin-conjugating enzyme E2G 1 (UBC7 homolog, yeast)	Homo sapiens
23061	TBC1 domain family, member 9B (with GRAM domain)	Homo sapiens
355	Fas (TNF receptor superfamily, member 6)	Homo sapiens
55234	smu-1 suppressor of mec-8 and unc-52 homolog (C. elegans)	Homo sapiens
23252	OTU domain containing 3	Homo sapiens
10606	phosphoribosylaminoimidazole carboxylase, phosphoribosylaminoimidazole succinocarboxamide synthetase	Homo sapiens
729789	ribosomal protein S15 pseudogene 5; ribosomal protein S15	Homo sapiens
6209	ribosomal protein S15 pseudogene 5; ribosomal protein S15	Homo sapiens
1523	cut-like homeobox 1	Homo sapiens
9666	DAZ interacting protein 3, zinc finger	Homo sapiens
22848	AP2 associated kinase 1	Homo sapiens
10908	patatin-like phospholipase domain containing 6	Homo sapiens
9232	pituitary tumor-transforming 1; pituitary tumor-transforming 2	Homo sapiens
10744	pituitary tumor-transforming 1; pituitary tumor-transforming 2	Homo sapiens
64145	zinc finger, FYVE domain containing 20	Homo sapiens
64718	unkempt homolog (Drosophila)-like	Homo sapiens
8139	gigaxonin	Homo sapiens
901	cyclin G2	Homo sapiens
56203	leiomodin 3 (fetal)	Homo sapiens

23214	exportin 6	Homo sapiens
140609	NIMA (never in mitosis gene a)-related kinase 7	Homo sapiens
57534	mindbomb homolog 1 (Drosophila)	Homo sapiens
255738	proprotein convertase subtilisin/kexin type 9	Homo sapiens
21	ATP-binding cassette, sub-family A (ABC1), member 3	Homo sapiens
57158	junctophilin 2	Homo sapiens
150465	tubulin tyrosine ligase	Homo sapiens
8490	regulator of G-protein signaling 5	Homo sapiens
27043	proline, glutamate and leucine rich protein 1	Homo sapiens
645369	two transmembrane domain family member A	Homo sapiens
3006	histone cluster 1, H1c	Homo sapiens
4681	neuroblastoma, suppression of tumorigenicity 1	Homo sapiens
85376	RIMS binding protein 3B; RIMS binding protein 3C; RIMS binding protein 3	Homo sapiens
150221	RIMS binding protein 3B; RIMS binding protein 3C; RIMS binding protein 3	Homo sapiens
440804	RIMS binding protein 3B; RIMS binding protein 3C; RIMS binding protein 3	Homo sapiens
93621	Mof4 family associated protein 1	Homo sapiens
255231	mucolipin 2	Homo sapiens
11337	GABA(A) receptor-associated protein	Homo sapiens
54888	NOL1/NOP2/Sun domain family, member 2	Homo sapiens
100132308	solute carrier family 29 (nucleoside transporters), member 4; similar to solute carrier family 29 (nucleoside transporters), member 4	Homo sapiens
402509	solute carrier family 29 (nucleoside transporters), member 4; similar to solute carrier family 29 (nucleoside transporters), member 4	Homo sapiens
222962	solute carrier family 29 (nucleoside transporters), member 4; similar to solute carrier family 29 (nucleoside transporters), member 4	Homo sapiens
969	CD69 molecule	Homo sapiens
767	carbonic anhydrase VIII	Homo sapiens
79915	ATPase family, AAA domain containing 5	Homo sapiens
3182	heterogeneous nuclear ribonucleoprotein A/B	Homo sapiens
23576	dimethylarginine dimethylaminohydrolase 1	Homo sapiens
9950	golgi autoantigen, golgin subfamily a, 5	Homo sapiens
387254	SLC7A5 pseudogene	Homo sapiens
26039	synovial sarcoma translocation gene on chromosome 18-like 1	Homo sapiens
375056	melanoma inhibitory activity family, member 3	Homo sapiens
650	bone morphogenetic protein 2	Homo sapiens
2760	GM2 ganglioside activator	Homo sapiens
2186	bromodomain PHD finger transcription factor	Homo sapiens
826	calpain, small subunit 1	Homo sapiens
1019	cyclin-dependent kinase 4	Homo sapiens
84440	RAB11 family interacting protein 4 (class II)	Homo sapiens
149420	PDLIM1 interacting kinase 1 like	Homo sapiens
55204	golgi phosphoprotein 3-like	Homo sapiens
54890	alkB, alkylation repair homolog 5 (E. coli)	Homo sapiens
79814	agmatine ureohydrolase (agmatinase)	Homo sapiens
5479	peptidylprolyl isomerase B (cyclophilin B)	Homo sapiens
6536	solute carrier family 6 (neurotransmitter transporter, glycine), member 9	Homo sapiens
10813	UTP14, U3 small nucleolar ribonucleoprotein, homolog A (yeast)	Homo sapiens
60481	ELOVL family member 5, elongation of long chain fatty acids (FEN1/Elo2, SUR4/Elo3-like, yeast)	Homo sapiens
10336	polycomb group ring finger 3	Homo sapiens
10910	SGT1, suppressor of G2 allele of SKP1 (S. cerevisiae)	Homo sapiens
4216	mitogen-activated protein kinase kinase kinase 4	Homo sapiens
8338	histone cluster 2, H2ac	Homo sapiens
80142	prostaglandin E synthase 2	Homo sapiens
8161	coilin	Homo sapiens
65084	transmembrane protein 135	Homo sapiens
7100	toll-like receptor 5	Homo sapiens
7408	vasodilator-stimulated phosphoprotein	Homo sapiens
5290	phosphoinositide-3-kinase, catalytic, alpha polypeptide	Homo sapiens
56990	CDC42 small effector 2	Homo sapiens
54617	INO80 homolog (S. cerevisiae)	Homo sapiens
1877	E4F transcription factor 1	Homo sapiens
54566	erythrocyte membrane protein band 4.1 like 4B	Homo sapiens
10625	influenza virus NS1A binding protein	Homo sapiens
163227	zinc finger protein 100	Homo sapiens
22824	heat shock 70kDa protein 4-like	Homo sapiens
81669	cyclin L2; chemokine (C-C motif) receptor 6	Homo sapiens
1235	cyclin L2; chemokine (C-C motif) receptor 6	Homo sapiens
51433	anaphase promoting complex subunit 5	Homo sapiens
56904	SH3-domain GRB2-like endophilin B2	Homo sapiens
8992	ATPase, H+ transporting, lysosomal 9kDa, V0 subunit e1	Homo sapiens
6712	spectrin, beta, non-erythrocytic 2	Homo sapiens
55789	DEP domain containing 1B	Homo sapiens
9601	protein disulfide isomerase family A, member 4	Homo sapiens
2064	v-erb-b2 erythroblastic leukemia viral oncogene homolog 2, neuro/glioblastoma derived oncogene homolog (avian)	Homo sapiens
283899	INO80 complex subunit E	Homo sapiens
84069	pleckstrin homology domain containing, family N member 1	Homo sapiens
28512	NFKB inhibitor interacting Ras-like 1	Homo sapiens

6880	TAF9 RNA polymerase II, TATA box binding protein (TBP)-associated factor, 32kDa	Homo sapiens
126917	intermediate filament family orphan 2	Homo sapiens
80153	enhancer of mRNA decapping 3 homolog (S. cerevisiae)	Homo sapiens
84515	minichromosome maintenance complex component 8	Homo sapiens
1581	cytochrome P450, family 7, subfamily A, polypeptide 1	Homo sapiens
5828	peroxisomal membrane protein 3, 35kDa	Homo sapiens
64837	kinesin light chain 2	Homo sapiens
54626	hairy and enhancer of split 2 (Drosophila)	Homo sapiens
283554	G protein-coupled receptor 137C	Homo sapiens
85406	DnaJ (Hsp40) homolog, subfamily C, member 14	Homo sapiens
10732	transcription factor-like 5 (basic helix-loop-helix)	Homo sapiens
10767	HBS1-like (S. cerevisiae)	Homo sapiens
23774	bromodomain containing 1	Homo sapiens
4801	nuclear transcription factor Y, beta	Homo sapiens
832	capping protein (actin filament) muscle Z-line, beta	Homo sapiens
55585	ubiquitin-conjugating enzyme E2Q family member 1	Homo sapiens
89910	ubiquitin protein ligase E3B	Homo sapiens
128178	EDAR-associated death domain	Homo sapiens
6885	mitogen-activated protein kinase kinase kinase 7	Homo sapiens
1290	collagen, type V, alpha 2	Homo sapiens
285636	chromosome 5 open reading frame 51	Homo sapiens
6901	tafazzin	Homo sapiens
25879	WD repeats and SOF1 domain containing	Homo sapiens
55624	protein O-linked mannose beta1,2-N-acetylglucosaminyltransferase	Homo sapiens
55285	RNA binding motif protein 41	Homo sapiens
940	CD28 molecule	Homo sapiens
1455	casein kinase 1, gamma 2	Homo sapiens
9255	aminoacyl tRNA synthetase complex-interacting multifunctional protein 1	Homo sapiens
55101	ATP5S-like	Homo sapiens
5230	phosphoglycerate kinase 1	Homo sapiens
23499	microtubule-actin crosslinking factor 1	Homo sapiens
2011	MAP/microtubule affinity-regulating kinase 2	Homo sapiens
50618	intersectin 2	Homo sapiens
7351	uncoupling protein 2 (mitochondrial, proton carrier)	Homo sapiens
203547	VMA21 vacuolar H+-ATPase homolog (S. cerevisiae)	Homo sapiens
56987	bobby sox homolog (Drosophila)	Homo sapiens
5305	phosphatidylinositol-5-phosphate 4-kinase, type II, alpha	Homo sapiens
80821	DDHD domain containing 1	Homo sapiens
10000	v-akt murine thymoma viral oncogene homolog 3 (protein kinase B, gamma)	Homo sapiens
8650	numb homolog (Drosophila)	Homo sapiens
1654	DEAD (Asp-Glu-Ala-Asp) box polypeptide 3, X-linked	Homo sapiens
9369	neurexin 3	Homo sapiens
51022	glutaredoxin 2	Homo sapiens
1717	7-dehydrocholesterol reductase	Homo sapiens
23293	Smg-6 homolog, nonsense mediated mRNA decay factor (C. elegans)	Homo sapiens
7693	zinc finger protein 134	Homo sapiens
9796	phytanoyl-CoA 2-hydroxylase interacting protein	Homo sapiens
79465	UL16 binding protein 3	Homo sapiens
340156	myosin light chain kinase family, member 4	Homo sapiens
84196	ubiquitin specific peptidase 48	Homo sapiens
123207	chromosome 15 open reading frame 40	Homo sapiens
9659	hypothetical protein LOC100134230; similar to KIAA0454 protein; similar to phosphodiesterase 4D interacting protein isoform 2; phosphodiesterase 4D interacting protein	Homo sapiens
100134230	hypothetical protein LOC100134230; similar to KIAA0454 protein; similar to phosphodiesterase 4D interacting protein isoform 2; phosphodiesterase 4D interacting protein	Homo sapiens
727927	hypothetical protein LOC100134230; similar to KIAA0454 protein; similar to phosphodiesterase 4D interacting protein isoform 2; phosphodiesterase 4D interacting protein	Homo sapiens
652164	hypothetical protein LOC100134230; similar to KIAA0454 protein; similar to phosphodiesterase 4D interacting protein isoform 2; phosphodiesterase 4D interacting protein	Homo sapiens
150696	prominin 2	Homo sapiens
121504	histone cluster 1, H4l; histone cluster 1, H4k; histone cluster 4, H4; histone cluster 1, H4h; histone cluster 1, H4j; histone cluster 1, H4i; histone cluster 1, H4d; histone cluster 1, H4c; histone cluster 1, H4f; histone cluster 1, H4e; histone cluster 1, H4b; histone cluster 1, H4a; histone cluster 2, H4a; histone cluster 2, H4b	Homo sapiens
8364	histone cluster 1, H4l; histone cluster 1, H4k; histone cluster 4, H4; histone cluster 1, H4h; histone cluster 1, H4j; histone cluster 1, H4i; histone cluster 1, H4d; histone cluster 1, H4c; histone cluster 1, H4f; histone cluster 1, H4e; histone cluster 1, H4b; histone cluster 1, H4a; histone cluster 2, H4a; histone cluster 2, H4b	Homo sapiens
8365	histone cluster 1, H4l; histone cluster 1, H4k; histone cluster 4, H4; histone cluster 1, H4h; histone cluster 1, H4j; histone cluster 1, H4i; histone cluster 1, H4d; histone cluster 1, H4c; histone cluster 1, H4f; histone cluster 1, H4e; histone cluster 1, H4b; histone cluster 1, H4a; histone cluster 2, H4a; histone cluster 2, H4b	Homo sapiens

8368	histone cluster 1, H4l; histone cluster 1, H4k; histone cluster 4, H4; histone cluster 1, H4h; histone cluster 1, H4j; histone cluster 1, H4i; histone cluster 1, H4d; histone cluster 1, H4c; histone cluster 1, H4f; histone cluster 1, H4e; histone cluster 1, H4b; histone cluster 1, H4a; histone cluster 2, H4a; histone cluster 2, H4b	Homo sapiens
554313	histone cluster 1, H4l; histone cluster 1, H4k; histone cluster 4, H4; histone cluster 1, H4h; histone cluster 1, H4j; histone cluster 1, H4i; histone cluster 1, H4d; histone cluster 1, H4c; histone cluster 1, H4f; histone cluster 1, H4e; histone cluster 1, H4b; histone cluster 1, H4a; histone cluster 2, H4a; histone cluster 2, H4b	Homo sapiens
8359	histone cluster 1, H4l; histone cluster 1, H4k; histone cluster 4, H4; histone cluster 1, H4h; histone cluster 1, H4j; histone cluster 1, H4i; histone cluster 1, H4d; histone cluster 1, H4c; histone cluster 1, H4f; histone cluster 1, H4e; histone cluster 1, H4b; histone cluster 1, H4a; histone cluster 2, H4a; histone cluster 2, H4b	Homo sapiens
8366	histone cluster 1, H4l; histone cluster 1, H4k; histone cluster 4, H4; histone cluster 1, H4h; histone cluster 1, H4j; histone cluster 1, H4i; histone cluster 1, H4d; histone cluster 1, H4c; histone cluster 1, H4f; histone cluster 1, H4e; histone cluster 1, H4b; histone cluster 1, H4a; histone cluster 2, H4a; histone cluster 2, H4b	Homo sapiens
8361	histone cluster 1, H4l; histone cluster 1, H4k; histone cluster 4, H4; histone cluster 1, H4h; histone cluster 1, H4j; histone cluster 1, H4i; histone cluster 1, H4d; histone cluster 1, H4c; histone cluster 1, H4f; histone cluster 1, H4e; histone cluster 1, H4b; histone cluster 1, H4a; histone cluster 2, H4a; histone cluster 2, H4b	Homo sapiens
8294	histone cluster 1, H4l; histone cluster 1, H4k; histone cluster 4, H4; histone cluster 1, H4h; histone cluster 1, H4j; histone cluster 1, H4i; histone cluster 1, H4d; histone cluster 1, H4c; histone cluster 1, H4f; histone cluster 1, H4e; histone cluster 1, H4b; histone cluster 1, H4a; histone cluster 2, H4a; histone cluster 2, H4b	Homo sapiens
8367	histone cluster 1, H4l; histone cluster 1, H4k; histone cluster 4, H4; histone cluster 1, H4h; histone cluster 1, H4j; histone cluster 1, H4i; histone cluster 1, H4d; histone cluster 1, H4c; histone cluster 1, H4f; histone cluster 1, H4e; histone cluster 1, H4b; histone cluster 1, H4a; histone cluster 2, H4a; histone cluster 2, H4b	Homo sapiens
8360	histone cluster 1, H4l; histone cluster 1, H4k; histone cluster 4, H4; histone cluster 1, H4h; histone cluster 1, H4j; histone cluster 1, H4i; histone cluster 1, H4d; histone cluster 1, H4c; histone cluster 1, H4f; histone cluster 1, H4e; histone cluster 1, H4b; histone cluster 1, H4a; histone cluster 2, H4a; histone cluster 2, H4b	Homo sapiens
8370	histone cluster 1, H4l; histone cluster 1, H4k; histone cluster 4, H4; histone cluster 1, H4h; histone cluster 1, H4j; histone cluster 1, H4i; histone cluster 1, H4d; histone cluster 1, H4c; histone cluster 1, H4f; histone cluster 1, H4e; histone cluster 1, H4b; histone cluster 1, H4a; histone cluster 2, H4a; histone cluster 2, H4b	Homo sapiens
8363	histone cluster 1, H4l; histone cluster 1, H4k; histone cluster 4, H4; histone cluster 1, H4h; histone cluster 1, H4j; histone cluster 1, H4i; histone cluster 1, H4d; histone cluster 1, H4c; histone cluster 1, H4f; histone cluster 1, H4e; histone cluster 1, H4b; histone cluster 1, H4a; histone cluster 2, H4a; histone cluster 2, H4b	Homo sapiens
8362	histone cluster 1, H4l; histone cluster 1, H4k; histone cluster 4, H4; histone cluster 1, H4h; histone cluster 1, H4j; histone cluster 1, H4i; histone cluster 1, H4d; histone cluster 1, H4c; histone cluster 1, H4f; histone cluster 1, H4e; histone cluster 1, H4b; histone cluster 1, H4a; histone cluster 2, H4a; histone cluster 2, H4b	Homo sapiens
60	actin, beta	Homo sapiens
5994	regulatory factor X-associated protein	Homo sapiens
10228	syntaxin 6	Homo sapiens
10066	secretory carrier membrane protein 2	Homo sapiens
6635	small nuclear ribonucleoprotein polypeptide E-like 1; small nuclear ribonucleoprotein polypeptide E; similar to hCG23490	Homo sapiens
414153	small nuclear ribonucleoprotein polypeptide E-like 1; small nuclear ribonucleoprotein polypeptide E; similar to hCG23490	Homo sapiens
100130109	small nuclear ribonucleoprotein polypeptide E-like 1; small nuclear ribonucleoprotein polypeptide E; similar to hCG23490	Homo sapiens
7461	CAP-GLY domain containing linker protein 2	Homo sapiens
9123	solute carrier family 16, member 3 (monocarboxylic acid transporter 4)	Homo sapiens
6813	syntaxin binding protein 2	Homo sapiens
23240	KIAA0922	Homo sapiens
23644	enhancer of mRNA decapping 4	Homo sapiens
54928	inositol monophosphatase domain containing 1	Homo sapiens
80243	phosphatidylinositol-3,4,5-trisphosphate-dependent Rac exchange factor 2	Homo sapiens
4810	Nance-Horan syndrome (congenital cataracts and dental anomalies)	Homo sapiens
3572	interleukin 6 signal transducer (gp130, oncostatin M receptor)	Homo sapiens
115650	tumor necrosis factor receptor superfamily, member 13C	Homo sapiens
152559	progestin and adipoQ receptor family member III	Homo sapiens
9283	G protein-coupled receptor 37 like 1	Homo sapiens
6041	ribonuclease L (2',5'-oligoisoadenylate synthetase-dependent)	Homo sapiens
27185	disrupted in schizophrenia 1	Homo sapiens
3232	homeobox D3	Homo sapiens
5423	polymerase (DNA directed), beta	Homo sapiens
7358	UDP-glucose dehydrogenase	Homo sapiens
10982	microtubule-associated protein, RP/EB family, member 2	Homo sapiens
653437	aquaporin 12B	Homo sapiens
23593	heme binding protein 2	Homo sapiens
84937	zinc and ring finger 1	Homo sapiens
4163	mutated in colorectal cancers	Homo sapiens
22926	activating transcription factor 6	Homo sapiens
80816	additional sex combs like 3 (Drosophila)	Homo sapiens
8880	far upstream element (FUSE) binding protein 1	Homo sapiens

55679	LIM and senescent cell antigen-like domains 2	Homo sapiens
27346	transmembrane protein 97	Homo sapiens
80975	transmembrane protease, serine 5	Homo sapiens
89848	FCH and double SH3 domains 1	Homo sapiens
9656	mediator of DNA-damage checkpoint 1	Homo sapiens
5694	proteasome (prosome, macropain) subunit, beta type, 6	Homo sapiens
8560	degenerative spermatocyte homolog 1, lipid desaturase (Drosophila)	Homo sapiens
27152	inturned planar cell polarity effector homolog (Drosophila)	Homo sapiens
3030	hydroxyacyl-Coenzyme A dehydrogenase/3-ketoacyl-Coenzyme A thiolase/enoyl-Coenzyme A hydratase (trifunctional protein), alpha subunit	Homo sapiens
23135	lysine (K)-specific demethylase 6B	Homo sapiens
8343	histone cluster 1, H2bi; histone cluster 1, H2bg; histone cluster 1, H2be; histone cluster 1, H2bf; histone cluster 1, H2bc	Homo sapiens
8344	histone cluster 1, H2bi; histone cluster 1, H2bg; histone cluster 1, H2be; histone cluster 1, H2bf; histone cluster 1, H2bc	Homo sapiens
8347	histone cluster 1, H2bi; histone cluster 1, H2bg; histone cluster 1, H2be; histone cluster 1, H2bf; histone cluster 1, H2bc	Homo sapiens
8339	histone cluster 1, H2bi; histone cluster 1, H2bg; histone cluster 1, H2be; histone cluster 1, H2bf; histone cluster 1, H2bc	Homo sapiens
8346	histone cluster 1, H2bi; histone cluster 1, H2bg; histone cluster 1, H2be; histone cluster 1, H2bf; histone cluster 1, H2bc	Homo sapiens
29126	CD274 molecule	Homo sapiens
79002	chromosome 19 open reading frame 43	Homo sapiens
55727	BTB (POZ) domain containing 7	Homo sapiens
55037	Pentatricopeptide repeat domain 3	Homo sapiens
10238	WD repeat domain 68	Homo sapiens
8751	ADAM metallopeptidase domain 15	Homo sapiens
284309	zinc finger protein 776	Homo sapiens
2005	ELK4, ETS-domain protein (SRF accessory protein 1)	Homo sapiens
3727	jun D proto-oncogene	Homo sapiens
401124	FLJ16686 protein	Homo sapiens
7767	zinc finger protein 224	Homo sapiens
59340	histamine receptor H4	Homo sapiens
100131572	ribosomal protein S27 pseudogene 29; ribosomal protein S27 pseudogene 9; ribosomal protein S27 pseudogene 23; ribosomal protein S27 pseudogene 13; ribosomal protein S27; ribosomal protein S27 pseudogene 21; ribosomal protein S27 pseudogene 7; ribosomal protein S27 pseudogene 6; ribosomal protein S27 pseudogene 19	Homo sapiens
100131787	ribosomal protein S27 pseudogene 29; ribosomal protein S27 pseudogene 9; ribosomal protein S27 pseudogene 23; ribosomal protein S27 pseudogene 13; ribosomal protein S27; ribosomal protein S27 pseudogene 21; ribosomal protein S27 pseudogene 7; ribosomal protein S27 pseudogene 6; ribosomal protein S27 pseudogene 19	Homo sapiens
100129905	ribosomal protein S27 pseudogene 29; ribosomal protein S27 pseudogene 9; ribosomal protein S27 pseudogene 23; ribosomal protein S27 pseudogene 13; ribosomal protein S27; ribosomal protein S27 pseudogene 21; ribosomal protein S27 pseudogene 7; ribosomal protein S27 pseudogene 6; ribosomal protein S27 pseudogene 19	Homo sapiens
100132291	ribosomal protein S27 pseudogene 29; ribosomal protein S27 pseudogene 9; ribosomal protein S27 pseudogene 23; ribosomal protein S27 pseudogene 13; ribosomal protein S27; ribosomal protein S27 pseudogene 21; ribosomal protein S27 pseudogene 7; ribosomal protein S27 pseudogene 6; ribosomal protein S27 pseudogene 19	Homo sapiens
100130070	ribosomal protein S27 pseudogene 29; ribosomal protein S27 pseudogene 9; ribosomal protein S27 pseudogene 23; ribosomal protein S27 pseudogene 13; ribosomal protein S27; ribosomal protein S27 pseudogene 21; ribosomal protein S27 pseudogene 7; ribosomal protein S27 pseudogene 6; ribosomal protein S27 pseudogene 19	Homo sapiens
100130775	ribosomal protein S27 pseudogene 29; ribosomal protein S27 pseudogene 9; ribosomal protein S27 pseudogene 23; ribosomal protein S27 pseudogene 13; ribosomal protein S27; ribosomal protein S27 pseudogene 21; ribosomal protein S27 pseudogene 7; ribosomal protein S27 pseudogene 6; ribosomal protein S27 pseudogene 19	Homo sapiens
100131905	ribosomal protein S27 pseudogene 29; ribosomal protein S27 pseudogene 9; ribosomal protein S27 pseudogene 23; ribosomal protein S27 pseudogene 13; ribosomal protein S27; ribosomal protein S27 pseudogene 21; ribosomal protein S27 pseudogene 7; ribosomal protein S27 pseudogene 6; ribosomal protein S27 pseudogene 19	Homo sapiens
100132488	ribosomal protein S27 pseudogene 29; ribosomal protein S27 pseudogene 9; ribosomal protein S27 pseudogene 23; ribosomal protein S27 pseudogene 13; ribosomal protein S27; ribosomal protein S27 pseudogene 21; ribosomal protein S27 pseudogene 7; ribosomal protein S27 pseudogene 6; ribosomal protein S27 pseudogene 19	Homo sapiens
6232	ribosomal protein S27 pseudogene 29; ribosomal protein S27 pseudogene 9; ribosomal protein S27 pseudogene 23; ribosomal protein S27 pseudogene 13; ribosomal protein S27; ribosomal protein S27 pseudogene 21; ribosomal protein S27 pseudogene 7; ribosomal protein S27 pseudogene 6; ribosomal protein S27 pseudogene 19	Homo sapiens
55662	hypoxia inducible factor 1, alpha subunit inhibitor	Homo sapiens
11102	ribonuclease P/MRP 14kDa subunit	Homo sapiens
84929	fibrinogen C domain containing 1	Homo sapiens
10447	family with sequence similarity 3, member C	Homo sapiens
317772	histone cluster 2, H2ab	Homo sapiens
4604	myosin binding protein C, slow type	Homo sapiens
64149	chromosome 17 open reading frame 75	Homo sapiens
85463	zinc finger CCCH-type containing 12C	Homo sapiens

731035	IQ motif and Sec7 domain 3; similar to IQ motif and Sec7 domain-containing protein 3; similar to IQ motif and SEC7 domain-containing protein 3; similar to IQ motif and Sec7 domain 3	Homo sapiens
100134209	IQ motif and Sec7 domain 3; similar to IQ motif and Sec7 domain-containing protein 3; similar to IQ motif and SEC7 domain-containing protein 3; similar to IQ motif and Sec7 domain 3	Homo sapiens
440073	IQ motif and Sec7 domain 3; similar to IQ motif and Sec7 domain-containing protein 3; similar to IQ motif and SEC7 domain-containing protein 3; similar to IQ motif and Sec7 domain 3	Homo sapiens
728056	IQ motif and Sec7 domain 3; similar to IQ motif and Sec7 domain-containing protein 3; similar to IQ motif and SEC7 domain-containing protein 3; similar to IQ motif and Sec7 domain 3	Homo sapiens
28985	malignant T cell amplified sequence 1	Homo sapiens
10531	pitrilysin metallopeptidase 1	Homo sapiens
1398	v-crk sarcoma virus CT10 oncogene homolog (avian)	Homo sapiens
79627	opioid growth factor receptor-like 1	Homo sapiens
506	ATP synthase, H+ transporting, mitochondrial F1 complex, beta polypeptide	Homo sapiens
4860	nucleoside phosphorylase	Homo sapiens
342945	zinc finger and SCAN domain containing 22	Homo sapiens
4924	nucleobindin 1	Homo sapiens
53838	chromosome 11 open reading frame 24	Homo sapiens
54887	UHRF1 binding protein 1	Homo sapiens
3181	heterogeneous nuclear ribonucleoprotein A2/B1	Homo sapiens
80221	acyl-CoA synthetase family member 2	Homo sapiens
55322	chromosome 5 open reading frame 22	Homo sapiens
1652	D-dopachrome tautomerase	Homo sapiens
100132425	hypothetical protein LOC100132425; similar to small nuclear ribonucleoprotein polypeptide G; small nuclear ribonucleoprotein polypeptide G; small nuclear ribonucleoprotein G-like protein	Homo sapiens
100130003	hypothetical protein LOC100132425; similar to small nuclear ribonucleoprotein polypeptide G; small nuclear ribonucleoprotein polypeptide G; small nuclear ribonucleoprotein G-like protein	Homo sapiens
6637	hypothetical protein LOC100132425; similar to small nuclear ribonucleoprotein polypeptide G; small nuclear ribonucleoprotein polypeptide G; small nuclear ribonucleoprotein G-like protein	Homo sapiens
100130932	hypothetical protein LOC100132425; similar to small nuclear ribonucleoprotein polypeptide G; small nuclear ribonucleoprotein polypeptide G; small nuclear ribonucleoprotein G-like protein	Homo sapiens
100130289	hypothetical protein LOC100132425; similar to small nuclear ribonucleoprotein polypeptide G; small nuclear ribonucleoprotein polypeptide G; small nuclear ribonucleoprotein G-like protein	Homo sapiens
27314	RAB30, member RAS oncogene family	Homo sapiens
2799	glucosamine (N-acetyl)-6-sulfatase	Homo sapiens
29095	ORM1-like 2 (S. cerevisiae)	Homo sapiens
27238	G patch domain and KOW motifs	Homo sapiens
79791	F-box protein 31	Homo sapiens
8945	beta-transducin repeat containing	Homo sapiens
64089	sorting nexin 16	Homo sapiens
6262	ryanodine receptor 2 (cardiac)	Homo sapiens
10137	RNA binding motif protein 12; copine I	Homo sapiens
8904	RNA binding motif protein 12; copine I	Homo sapiens
6949	Treacher Collins-Franceschetti syndrome 1	Homo sapiens
147179	WAS/WASL interacting protein family, member 2	Homo sapiens
84289	inhibitor of growth family, member 5	Homo sapiens
201163	folliculin	Homo sapiens
10899	jumping translocation breakpoint	Homo sapiens
219972	macrophage expressed 1	Homo sapiens
4076	cell cycle associated protein 1	Homo sapiens
64843	ISL LIM homeobox 2	Homo sapiens
10009	zinc finger and BTB domain containing 33	Homo sapiens
5287	phosphoinositide-3-kinase, class 2, beta polypeptide	Homo sapiens
4666	nascent polypeptide-associated complex alpha subunit	Homo sapiens
10211	flotillin 1	Homo sapiens
170082	transcription elongation factor A (SII) N-terminal and central domain containing	Homo sapiens
60592	short coiled-coil protein	Homo sapiens
55693	lysine (K)-specific demethylase 4D	Homo sapiens
3064	huntingtin	Homo sapiens
5861	RAB1A, member RAS oncogene family	Homo sapiens
7453	tryptophanyl-tRNA synthetase	Homo sapiens
121227	leucine-rich repeats and immunoglobulin-like domains 3	Homo sapiens
2542	solute carrier family 37 (glucose-6-phosphate transporter), member 4	Homo sapiens
317	apoptotic peptidase activating factor 1	Homo sapiens
27335	eukaryotic translation initiation factor 3, subunit K	Homo sapiens
79833	gem (nuclear organelle) associated protein 6	Homo sapiens
23193	glucosidase, alpha; neutral AB	Homo sapiens
9203	zinc finger, MYM-type 3	Homo sapiens
25875	LETM1 domain containing 1	Homo sapiens
9060	3'-phosphoadenosine 5'-phosphosulfate synthase 2	Homo sapiens
11123	RCAN family member 3	Homo sapiens

55313	calcineurin-like phosphoesterase domain containing 1	Homo sapiens
10498	coactivator-associated arginine methyltransferase 1	Homo sapiens
54764	zinc finger, RAN-binding domain containing 1	Homo sapiens
286827	tripartite motif-containing 59	Homo sapiens
3741	potassium voltage-gated channel, shaker-related subfamily, member 5	Homo sapiens
9880	zinc finger and BTB domain containing 39	Homo sapiens
374872	chromosome 19 open reading frame 35	Homo sapiens
132671	spermatogenesis associated 18 homolog (rat)	Homo sapiens
4761	neurogenic differentiation 2	Homo sapiens
9862	mediator complex subunit 24	Homo sapiens
58499	zinc finger protein 462	Homo sapiens
463	zinc finger homeobox 3; hypothetical LOC100132068	Homo sapiens
100132068	zinc finger homeobox 3; hypothetical LOC100132068	Homo sapiens
51109	retinol dehydrogenase 11 (all-trans/9-cis/11-cis)	Homo sapiens
64866	CUB domain containing protein 1	Homo sapiens
6187	ribosomal protein S2 pseudogene 8; ribosomal protein S2 pseudogene 11; ribosomal protein S2 pseudogene 5; ribosomal protein S2 pseudogene 12; ribosomal protein S2 pseudogene 51; ribosomal protein S2 pseudogene 17; ribosomal protein S2 pseudogene 55; ribosomal protein S2 pseudogene 20; ribosomal protein S2	Homo sapiens
286444	ribosomal protein S2 pseudogene 8; ribosomal protein S2 pseudogene 11; ribosomal protein S2 pseudogene 5; ribosomal protein S2 pseudogene 12; ribosomal protein S2 pseudogene 51; ribosomal protein S2 pseudogene 17; ribosomal protein S2 pseudogene 55; ribosomal protein S2 pseudogene 20; ribosomal protein S2	Homo sapiens
100130562	ribosomal protein S2 pseudogene 8; ribosomal protein S2 pseudogene 11; ribosomal protein S2 pseudogene 5; ribosomal protein S2 pseudogene 12; ribosomal protein S2 pseudogene 51; ribosomal protein S2 pseudogene 17; ribosomal protein S2 pseudogene 55; ribosomal protein S2 pseudogene 20; ribosomal protein S2	Homo sapiens
650901	ribosomal protein S2 pseudogene 8; ribosomal protein S2 pseudogene 11; ribosomal protein S2 pseudogene 5; ribosomal protein S2 pseudogene 12; ribosomal protein S2 pseudogene 51; ribosomal protein S2 pseudogene 17; ribosomal protein S2 pseudogene 55; ribosomal protein S2 pseudogene 20; ribosomal protein S2	Homo sapiens
343184	ribosomal protein S2 pseudogene 8; ribosomal protein S2 pseudogene 11; ribosomal protein S2 pseudogene 5; ribosomal protein S2 pseudogene 12; ribosomal protein S2 pseudogene 51; ribosomal protein S2 pseudogene 17; ribosomal protein S2 pseudogene 55; ribosomal protein S2 pseudogene 20; ribosomal protein S2	Homo sapiens
440589	ribosomal protein S2 pseudogene 8; ribosomal protein S2 pseudogene 11; ribosomal protein S2 pseudogene 5; ribosomal protein S2 pseudogene 12; ribosomal protein S2 pseudogene 51; ribosomal protein S2 pseudogene 17; ribosomal protein S2 pseudogene 55; ribosomal protein S2 pseudogene 20; ribosomal protein S2	Homo sapiens
400963	ribosomal protein S2 pseudogene 8; ribosomal protein S2 pseudogene 11; ribosomal protein S2 pseudogene 5; ribosomal protein S2 pseudogene 12; ribosomal protein S2 pseudogene 51; ribosomal protein S2 pseudogene 17; ribosomal protein S2 pseudogene 55; ribosomal protein S2 pseudogene 20; ribosomal protein S2	Homo sapiens
729679	ribosomal protein S2 pseudogene 8; ribosomal protein S2 pseudogene 11; ribosomal protein S2 pseudogene 5; ribosomal protein S2 pseudogene 12; ribosomal protein S2 pseudogene 51; ribosomal protein S2 pseudogene 17; ribosomal protein S2 pseudogene 55; ribosomal protein S2 pseudogene 20; ribosomal protein S2	Homo sapiens
645018	ribosomal protein S2 pseudogene 8; ribosomal protein S2 pseudogene 11; ribosomal protein S2 pseudogene 5; ribosomal protein S2 pseudogene 12; ribosomal protein S2 pseudogene 51; ribosomal protein S2 pseudogene 17; ribosomal protein S2 pseudogene 55; ribosomal protein S2 pseudogene 20; ribosomal protein S2	Homo sapiens
19	ATP-binding cassette, sub-family A (ABC1), member 1	Homo sapiens
390	Rho family GTPase 3	Homo sapiens
5295	phosphoinositide-3-kinase, regulatory subunit 1 (alpha)	Homo sapiens
201514	zinc finger protein 584	Homo sapiens
23	ATP-binding cassette, sub-family F (GCN20), member 1	Homo sapiens
81796	solute carrier organic anion transporter family, member 5A1	Homo sapiens
5921	RAS p21 protein activator (GTPase activating protein) 1	Homo sapiens
27252	kelch-like 20 (Drosophila)	Homo sapiens
22806	IKAROS family zinc finger 3 (Aiolos)	Homo sapiens
8907	adaptor-related protein complex 1, mu 1 subunit	Homo sapiens
11019	lipoic acid synthetase	Homo sapiens
22879	MON1 homolog B (yeast)	Homo sapiens
256051	zinc finger protein 549	Homo sapiens
126231	zinc finger protein 573	Homo sapiens
11133	kaptin (actin binding protein)	Homo sapiens
158471	prune homolog 2 (Drosophila)	Homo sapiens
3688	integrin, beta 1 (fibronectin receptor, beta polypeptide, antigen CD29 includes MDF2, MSK12)	Homo sapiens
643904	ring finger protein 222	Homo sapiens
8578	scavenger receptor class F, member 1	Homo sapiens
51008	activating signal cointegrator 1 complex subunit 1	Homo sapiens
22875	ectonucleotide pyrophosphatase/phosphodiesterase 4 (putative function)	Homo sapiens
388112	Nanog homeobox pseudogene 8; Nanog homeobox	Homo sapiens
79923	Nanog homeobox pseudogene 8; Nanog homeobox	Homo sapiens
3148	high-mobility group box 2	Homo sapiens
2132	exostoses (multiple) 2	Homo sapiens
10130	protein disulfide isomerase family A, member 6	Homo sapiens
1173	adaptor-related protein complex 2, mu 1 subunit	Homo sapiens

1456	casein kinase 1, gamma 3	Homo sapiens
6158	ribosomal protein L28	Homo sapiens
4134	microtubule-associated protein 4	Homo sapiens
51361	hook homolog 1 (Drosophila)	Homo sapiens
150275	coiled-coil domain containing 117	Homo sapiens
6872	TAF1 RNA polymerase II, TATA box binding protein (TBP)-associated factor, 250kDa	Homo sapiens
4179	CD46 molecule, complement regulatory protein	Homo sapiens
7158	tumor protein p53 binding protein 1	Homo sapiens
10818	fibroblast growth factor receptor substrate 2	Homo sapiens
2067	excision repair cross-complementing rodent repair deficiency, complementation group 1 (includes overlapping antisense sequence)	Homo sapiens
138639	protein tyrosine phosphatase domain containing 1	Homo sapiens
163590	torsin A interacting protein 2	Homo sapiens
151887	coiled-coil domain containing 80	Homo sapiens
54742	lymphocyte antigen 6 complex, locus K	Homo sapiens
10495	ecto-NOX disulfide-thiol exchanger 2	Homo sapiens
65250	chromosome 5 open reading frame 42	Homo sapiens
57621	zinc finger and BTB domain containing 2	Homo sapiens
4350	N-methylpurine-DNA glycosylase	Homo sapiens
57493	HEG homolog 1 (zebrafish)	Homo sapiens
116143	WD repeat domain 92	Homo sapiens
54629	family with sequence similarity 63, member B	Homo sapiens
54958	transmembrane protein 160	Homo sapiens
29103	DnaJ (Hsp40) homolog, subfamily C, member 15	Homo sapiens
541578	chromosome X open reading frame 40A; chromosome X open reading frame 40B	Homo sapiens
91966	chromosome X open reading frame 40A; chromosome X open reading frame 40B	Homo sapiens
8140	solute carrier family 7 (cationic amino acid transporter, y+ system), member 5	Homo sapiens
388759	chromosome 1 open reading frame 229	Homo sapiens
144455	E2F transcription factor 7	Homo sapiens
25937	WW domain containing transcription regulator 1	Homo sapiens
10169	chromosome 15 open reading frame 63; small EDRK-rich factor 2	Homo sapiens
25764	chromosome 15 open reading frame 63; small EDRK-rich factor 2	Homo sapiens
134553	chromosome 5 open reading frame 24	Homo sapiens
7109	trafficking protein particle complex 10	Homo sapiens
60468	BTB and CNC homology 1, basic leucine zipper transcription factor 2	Homo sapiens
9421	heart and neural crest derivatives expressed 1	Homo sapiens
57148	KIAA1219	Homo sapiens
653375	RCC1-like G exchanging factor-like; Williams-Beuren syndrome chromosome region 16	Homo sapiens
81554	RCC1-like G exchanging factor-like; Williams-Beuren syndrome chromosome region 16	Homo sapiens
136051	zinc finger protein 786	Homo sapiens
23635	single-stranded DNA binding protein 2	Homo sapiens
26259	F-box and WD repeat domain containing 8	Homo sapiens
55907	cytidine monophosphate N-acetylneuraminic acid synthetase	Homo sapiens
2079	enhancer of rudimentary homolog (Drosophila)	Homo sapiens
148534	transmembrane protein 56	Homo sapiens
79012	CaM kinase-like vesicle-associated	Homo sapiens
81876	RAB1B, member RAS oncogene family	Homo sapiens
9818	nucleoporin like 1	Homo sapiens
5635	phosphoribosyl pyrophosphate synthetase-associated protein 1	Homo sapiens
26037	signal-induced proliferation-associated 1 like 1	Homo sapiens
3824	killer cell lectin-like receptor subfamily D, member 1	Homo sapiens
81544	glycerophosphodiester phosphodiesterase domain containing 5	Homo sapiens
1432	mitogen-activated protein kinase 14	Homo sapiens
390916	nudix (nucleoside diphosphate linked moiety X)-type motif 19	Homo sapiens
23743	betaine-homocysteine methyltransferase 2	Homo sapiens
497661	chromosome 18 open reading frame 32	Homo sapiens
950	scavenger receptor class B, member 2	Homo sapiens
55954	zinc finger, matrin type 5	Homo sapiens
81889	fumarylacetoacetate hydrolase domain containing 1	Homo sapiens
57142	reticulon 4	Homo sapiens
10277	ubiquitination factor E4B (UFD2 homolog, yeast)	Homo sapiens
1723	dihydroorotate dehydrogenase	Homo sapiens
9348	N-deacetylase/N-sulfotransferase (heparan glucosaminyl) 3	Homo sapiens
387914	shisa homolog 2 (Xenopus laevis)	Homo sapiens
64792	RAB, member RAS oncogene family-like 5	Homo sapiens
57456	KIAA1143	Homo sapiens
2972	BRF1 homolog, subunit of RNA polymerase III transcription initiation factor IIIB (S. cerevisiae)	Homo sapiens
26273	F-box protein 3	Homo sapiens
8303	stannin	Homo sapiens
2176	Fanconi anemia, complementation group C	Homo sapiens
217	aldehyde dehydrogenase 2 family (mitochondrial)	Homo sapiens
58513	epidermal growth factor receptor pathway substrate 15-like 1	Homo sapiens
5116	pericentrin	Homo sapiens
220074	leucine rich transmembrane and O-methyltransferase domain containing	Homo sapiens
80143	suppressor of IKK epsilon	Homo sapiens
55027	HEAT repeat containing 3	Homo sapiens
9354	ubiquitination factor E4A (UFD2 homolog, yeast)	Homo sapiens
1305	collagen, type XIII, alpha 1	Homo sapiens

9522	secretory carrier membrane protein 1	Homo sapiens
114134	solute carrier family 2 (facilitated glucose transporter), member 13	Homo sapiens
2063	nuclear receptor subfamily 2, group F, member 6	Homo sapiens
7779	solute carrier family 30 (zinc transporter), member 1	Homo sapiens
9044	BTAF1 RNA polymerase II, B-TFIID transcription factor-associated, 170kDa (Mot1 homolog, S. cerevisiae)	Homo sapiens
4799	nuclear transcription factor, X-box binding 1	Homo sapiens
723790	histone cluster 2, H2aa3; histone cluster 2, H2aa4	Homo sapiens
8337	histone cluster 2, H2aa3; histone cluster 2, H2aa4	Homo sapiens
10072	dipeptidyl-peptidase 3	Homo sapiens
10948	StAR-related lipid transfer (START) domain containing 3	Homo sapiens
3218	homeobox B8	Homo sapiens
10481	homeobox B13	Homo sapiens
7110	TATA element modulatory factor 1	Homo sapiens
5654	HtrA serine peptidase 1	Homo sapiens
5106	phosphoenolpyruvate carboxykinase 2 (mitochondrial)	Homo sapiens
5603	mitogen-activated protein kinase 13	Homo sapiens
7879	RAB7A, member RAS oncogene family	Homo sapiens
8533	COP9 constitutive photomorphogenic homolog subunit 3 (Arabidopsis)	Homo sapiens
6303	spermidine/spermine N1-acetyltransferase 1	Homo sapiens
116224	family with sequence similarity 122A	Homo sapiens
168451	THAP domain containing 5	Homo sapiens
23551	RASD family, member 2	Homo sapiens
9775	eukaryotic translation initiation factor 4A, isoform 3	Homo sapiens
54876	chromosome 4 open reading frame 30	Homo sapiens
90007	midnolin	Homo sapiens
83999	kringle containing transmembrane protein 1	Homo sapiens
7350	uncoupling protein 1 (mitochondrial, proton carrier)	Homo sapiens
125150	zinc finger, SWIM-type containing 7	Homo sapiens
3508	immunoglobulin mu binding protein 2	Homo sapiens
23011	RAB21, member RAS oncogene family	Homo sapiens
9538	etoposide induced 2.4 mRNA	Homo sapiens
83596	BCL2-like 12 (proline rich)	Homo sapiens
6945	MAX-like protein X	Homo sapiens
2022	endoglin	Homo sapiens
10087	collagen, type IV, alpha 3 (Goodpasture antigen) binding protein	Homo sapiens
10766	transducer of ERBB2, 2	Homo sapiens
55222	leucine rich repeat containing 20	Homo sapiens
127544	ring finger protein 19B	Homo sapiens
9113	LATS, large tumor suppressor, homolog 1 (Drosophila)	Homo sapiens
157506	retinol dehydrogenase 10 (all-trans)	Homo sapiens
51162	EGF-like-domain, multiple 7	Homo sapiens
115106	HAUS augmin-like complex, subunit 1	Homo sapiens
353500	bone morphogenetic protein 8a	Homo sapiens
6047	ring finger protein 4; hypothetical LOC644006	Homo sapiens
644006	ring finger protein 4; hypothetical LOC644006	Homo sapiens
2814	glycoprotein V (platelet)	Homo sapiens
5255	phosphorylase kinase, alpha 1 pseudogene 1; phosphorylase kinase, alpha 1 (muscle)	Homo sapiens
646780	phosphorylase kinase, alpha 1 pseudogene 1; phosphorylase kinase, alpha 1 (muscle)	Homo sapiens
9266	cytohesin 2	Homo sapiens
85403	ELL associated factor 1	Homo sapiens
84503	zinc finger protein 527	Homo sapiens
4299	AF4/FMR2 family, member 1	Homo sapiens
2110	electron-transferring-flavoprotein dehydrogenase	Homo sapiens
6648	superoxide dismutase 2, mitochondrial	Homo sapiens
55654	transmembrane protein 127	Homo sapiens
8850	K(lysine) acetyltransferase 2B	Homo sapiens
23310	non-SMC condensin II complex, subunit D3	Homo sapiens
79718	transducin (beta)-like 1 X-linked receptor 1	Homo sapiens
3930	lamin B receptor	Homo sapiens
8603	chromosome 4 open reading frame 8	Homo sapiens
24137	kinesin family member 4B; kinesin family member 4A	Homo sapiens
285643	kinesin family member 4B; kinesin family member 4A	Homo sapiens
343035	retinal degeneration 3	Homo sapiens
23250	ATPase, class VI, type 11A	Homo sapiens
10067	secretory carrier membrane protein 3	Homo sapiens
147372	collagen and calcium binding EGF domains 1	Homo sapiens
79991	oligonucleotide/oligosaccharide-binding fold containing 1	Homo sapiens
8026	DiGeorge syndrome chromosome region-2	Homo sapiens
283933	zinc finger protein 843	Homo sapiens
144501	keratin 80	Homo sapiens
6434	transformer 2 beta homolog (Drosophila)	Homo sapiens
4131	microtubule-associated protein 1B	Homo sapiens
9877	zinc finger CCCH-type containing 11A	Homo sapiens
400569	mediator complex subunit 11	Homo sapiens
11078	TRIO and F-actin binding protein	Homo sapiens
9343	elongation factor Tu GTP binding domain containing 2	Homo sapiens
160760	PTC7 protein phosphatase homolog (S. cerevisiae)	Homo sapiens
55262	chromosome 7 open reading frame 43	Homo sapiens

60625	DEAH (Asp-Glu-Ala-His) box polypeptide 35	Homo sapiens
389206	BEN domain containing 4	Homo sapiens
81688	chromosome 6 open reading frame 62	Homo sapiens
23307	FK506 binding protein 15, 133kDa	Homo sapiens
22809	activating transcription factor 5	Homo sapiens
6515	solute carrier family 2 (facilitated glucose transporter), member 3	Homo sapiens
9764	KIAA0513	Homo sapiens
595	cyclin D1	Homo sapiens
23435	TAR DNA binding protein	Homo sapiens
80851	SH3-binding domain protein 5-like	Homo sapiens
90522	Yip1 interacting factor homolog B (S. cerevisiae)	Homo sapiens
6294	scaffold attachment factor B	Homo sapiens
63929	X-prolyl aminopeptidase (aminopeptidase P) 3, putative	Homo sapiens
23518	R3H domain containing 1	Homo sapiens
1964	eukaryotic translation initiation factor 1A, X-linked	Homo sapiens
7711	zinc finger protein 155	Homo sapiens
4117	male germ cell-associated kinase	Homo sapiens
79027	zinc finger protein 655	Homo sapiens
22985	apoptotic chromatin condensation inducer 1	Homo sapiens
3207	homeobox A11	Homo sapiens
3884	keratin 33B	Homo sapiens
387	ras homolog gene family, member A	Homo sapiens
160	adaptor-related protein complex 2, alpha 1 subunit	Homo sapiens
84527	zinc finger protein 559	Homo sapiens
26151	N-acetyltransferase 9 (GCN5-related, putative)	Homo sapiens
440400	ribonuclease, RNase K	Homo sapiens
4841	non-POU domain containing, octamer-binding	Homo sapiens
80742	proline rich 3	Homo sapiens
6737	tripartite motif-containing 21	Homo sapiens
8879	sphingosine-1-phosphate lyase 1	Homo sapiens
63931	mitochondrial ribosomal protein S14	Homo sapiens
5430	polymerase (RNA) II (DNA directed) polypeptide A, 220kDa	Homo sapiens
63906	G patch domain containing 3	Homo sapiens
6247	retinoschisin 1	Homo sapiens
57468	solute carrier family 12 (potassium-chloride transporter), member 5	Homo sapiens
122416	ankyrin repeat domain 9	Homo sapiens
2976	general transcription factor IIIC, polypeptide 2, beta 110kDa	Homo sapiens
91869	RFT1 homolog (S. cerevisiae)	Homo sapiens
2091	fibrillarin	Homo sapiens
80313	leucine rich repeat containing 27	Homo sapiens
113146	AHNAK nucleoprotein 2	Homo sapiens
64420	sushi domain containing 1	Homo sapiens
3460	interferon gamma receptor 2 (interferon gamma transducer 1)	Homo sapiens
23154	neurochondrin	Homo sapiens
25946	zinc finger protein 385A	Homo sapiens
1385	cAMP responsive element binding protein 1	Homo sapiens
6168	ribosomal protein L37a	Homo sapiens
10489	leucine rich repeat containing 41	Homo sapiens
27125	AF4/FMR2 family, member 4	Homo sapiens
112399	egl nine homolog 3 (C. elegans)	Homo sapiens
645191	leucine rich repeat and Ig domain containing 3	Homo sapiens
10725	nuclear factor of activated T-cells 5, tonicity-responsive	Homo sapiens
285193	dual specificity phosphatase 28	Homo sapiens
5519	protein phosphatase 2 (formerly 2A), regulatory subunit A, beta isoform	Homo sapiens
84254	calcium/calmodulin-dependent protein kinase kinase 1, alpha	Homo sapiens
2357	formyl peptide receptor 1	Homo sapiens
115273	RAB42, member RAS oncogene family	Homo sapiens
204	adenylate kinase 2	Homo sapiens
124583	calcium activated nucleotidase 1	Homo sapiens
85416	Zic family member 5 (odd-paired homolog, Drosophila)	Homo sapiens
891	cyclin B1	Homo sapiens
11171	serine/threonine kinase receptor associated protein	Homo sapiens
90411	multiple coagulation factor deficiency 2	Homo sapiens
89927	chromosome 16 open reading frame 45	Homo sapiens
25828	thioredoxin 2	Homo sapiens
23568	ADP-ribosylation factor-like 2 binding protein	Homo sapiens
8832	CD84 molecule	Homo sapiens
91694	LON peptidase N-terminal domain and ring finger 1	Homo sapiens
1373	carbamoyl-phosphate synthetase 1, mitochondrial	Homo sapiens
1027	cyclin-dependent kinase inhibitor 1B (p27, Kip1)	Homo sapiens
10313	reticulon 3	Homo sapiens
60488	mitochondrial ribosomal protein S35	Homo sapiens
7799	PR domain containing 2, with ZNF domain	Homo sapiens
7431	vimentin	Homo sapiens
10201	non-metastatic cells 6, protein expressed in (nucleoside-diphosphate kinase)	Homo sapiens
9439	mediator complex subunit 23	Homo sapiens
55238	solute carrier family 38, member 7	Homo sapiens
23451	splicing factor 3b, subunit 1, 155kDa	Homo sapiens
8553	basic helix-loop-helix family, member e40	Homo sapiens

23096	IQ motif and Sec7 domain 2	Homo sapiens
8776	myotubularin related protein 1	Homo sapiens
7812	cold shock domain containing E1, RNA-binding	Homo sapiens
8766	RAB11A, member RAS oncogene family	Homo sapiens
84124	zinc finger protein 394	Homo sapiens
22992	lysine (K)-specific demethylase 2A	Homo sapiens
7126	tumor necrosis factor, alpha-induced protein 1 (endothelial)	Homo sapiens
152404	immunoglobulin superfamily, member 11	Homo sapiens
3024	histone cluster 1, H1a	Homo sapiens
916	CD3e molecule, epsilon (CD3-TCR complex)	Homo sapiens
2275	four and a half LIM domains 3	Homo sapiens
6723	spermidine synthase	Homo sapiens
9329	general transcription factor IIIC, polypeptide 4, 90kDa	Homo sapiens
55288	ras homolog gene family, member T1	Homo sapiens
51582	antizyme inhibitor 1	Homo sapiens
80111	chromosome 3 open reading frame 36	Homo sapiens
2193	phenylalanyl-tRNA synthetase, alpha subunit	Homo sapiens
9510	ADAM metallopeptidase with thrombospondin type 1 motif, 1	Homo sapiens
9644	SH3 and PX domains 2A	Homo sapiens
128312	histone cluster 3, H2bb	Homo sapiens
348793	WD repeat domain 53	Homo sapiens
64902	alanine-glyoxylate aminotransferase 2	Homo sapiens
9388	lipase, endothelial	Homo sapiens
9722	nitric oxide synthase 1 (neuronal) adaptor protein	Homo sapiens
7050	TGFB-induced factor homeobox 1	Homo sapiens
9040	ubiquitin-conjugating enzyme E2M (UBC12 homolog, yeast); ubiquitin-conjugating enzyme E2M pseudogene 1	Homo sapiens
606551	ubiquitin-conjugating enzyme E2M (UBC12 homolog, yeast); ubiquitin-conjugating enzyme E2M pseudogene 1	Homo sapiens
10247	heat-responsive protein 12	Homo sapiens
2000	E74-like factor 4 (ets domain transcription factor)	Homo sapiens
4779	nuclear factor (erythroid-derived 2)-like 1	Homo sapiens
5724	platelet-activating factor receptor	Homo sapiens
4097	v-maf musculoaponeurotic fibrosarcoma oncogene homolog G (avian)	Homo sapiens
6876	transgelin	Homo sapiens
10627	myosin, light chain 12A, regulatory, non-sarcomeric	Homo sapiens
4848	CCR4-NOT transcription complex, subunit 2	Homo sapiens
1363	carboxypeptidase E	Homo sapiens
862	runt-related transcription factor 1; translocated to, 1 (cyclin D-related)	Homo sapiens
9375	transmembrane 9 superfamily member 2	Homo sapiens
7094	talin 1	Homo sapiens
92092	zinc finger CCCH-type, antiviral 1-like	Homo sapiens
89858	sialic acid binding Ig-like lectin 12	Homo sapiens
3621	inhibitor of growth family, member 1	Homo sapiens
10810	WAS protein family, member 3	Homo sapiens
64063	protease, serine, 22	Homo sapiens
6165	ribosomal protein L35a	Homo sapiens
467	activating transcription factor 3	Homo sapiens
2158	coagulation factor IX	Homo sapiens
55275	vacuolar protein sorting 53 homolog (S. cerevisiae)	Homo sapiens
3570	interleukin 6 receptor	Homo sapiens
53918	pelota homolog (Drosophila)	Homo sapiens
25880	transmembrane protein 186	Homo sapiens
26054	SUMO1/sentrin specific peptidase 6	Homo sapiens
3257	Hermansky-Pudlak syndrome 1	Homo sapiens
26256	calcium binding tyrosine-(Y)-phosphorylation regulated	Homo sapiens
387032	zinc finger with KRAB and SCAN domains 4	Homo sapiens
7251	tumor susceptibility gene 101	Homo sapiens
51573	glycerophosphodiester phosphodiesterase 1	Homo sapiens
54941	ring finger protein 125	Homo sapiens
114804	ring finger protein 157	Homo sapiens
83759	RNA binding motif protein 4B	Homo sapiens
147657	zinc finger protein 480	Homo sapiens
79001	vitamin K epoxide reductase complex, subunit 1	Homo sapiens
23545	ATPase, H+ transporting, lysosomal V0 subunit a2	Homo sapiens
9275	B-cell CLL/lymphoma 7B	Homo sapiens
8856	nuclear receptor subfamily 1, group I, member 2	Homo sapiens
6522	solute carrier family 4, anion exchanger, member 2 (erythrocyte membrane protein band 3-like 1)	Homo sapiens
11103	KRR1, small subunit (SSU) processome component, homolog (yeast)	Homo sapiens
60559	signal peptidase complex subunit 3 homolog (S. cerevisiae)	Homo sapiens
23112	trinucleotide repeat containing 6B	Homo sapiens
23446	solute carrier family 44, member 1	Homo sapiens
1111	CHK1 checkpoint homolog (S. pombe)	Homo sapiens
8467	SWI/SNF related, matrix associated, actin dependent regulator of chromatin, subfamily a, member 5	Homo sapiens
648791	protein phosphatase 1, regulatory (inhibitor) subunit 3G	Homo sapiens
122060	SLAIN motif family, member 1	Homo sapiens
56261	hypothetical protein KIAA1434	Homo sapiens

3763	potassium inwardly-rectifying channel, subfamily J, member 6	Homo sapiens
5869	RAB5B, member RAS oncogene family	Homo sapiens
6627	small nuclear ribonucleoprotein polypeptide A'	Homo sapiens
29116	myosin regulatory light chain interacting protein	Homo sapiens
23351	KIAA0323	Homo sapiens
27338	ubiquitin-conjugating enzyme E2S	Homo sapiens
8013	nuclear receptor subfamily 4, group A, member 3	Homo sapiens
57699	copine V	Homo sapiens
7407	valyl-tRNA synthetase	Homo sapiens
143279	HECT domain containing 2	Homo sapiens
439953	similar to TRIMCyp; peptidylprolyl isomerase A (cyclophilin A); peptidylprolyl isomerase A (cyclophilin A)-like 3	Homo sapiens
653214	similar to TRIMCyp; peptidylprolyl isomerase A (cyclophilin A); peptidylprolyl isomerase A (cyclophilin A)-like 3	Homo sapiens
5478	similar to TRIMCyp; peptidylprolyl isomerase A (cyclophilin A); peptidylprolyl isomerase A (cyclophilin A)-like 3	Homo sapiens
79568	chromosome 2 open reading frame 47	Homo sapiens
113829	solute carrier family 35, member A4	Homo sapiens
554251	F-box protein 48	Homo sapiens
54677	carnitine O-octanoyltransferase	Homo sapiens
375743	protein prenyltransferase alpha subunit repeat containing 1	Homo sapiens
5567	protein kinase, cAMP-dependent, catalytic, beta	Homo sapiens
23704	potassium voltage-gated channel, Isk-related family, member 4	Homo sapiens
55629	proline-rich nuclear receptor coactivator 2; similar to hCG1728885	Homo sapiens
100131261	proline-rich nuclear receptor coactivator 2; similar to hCG1728885	Homo sapiens
126669	Src homology 2 domain containing E	Homo sapiens
4677	asparaginyl-tRNA synthetase	Homo sapiens
8881	cell division cycle 16 homolog (S. cerevisiae)	Homo sapiens
10950	BTG family, member 3	Homo sapiens
5494	protein phosphatase 1A (formerly 2C), magnesium-dependent, alpha isoform	Homo sapiens
1789	DNA (cytosine-5-)-methyltransferase 3 beta	Homo sapiens
54439	RNA binding motif protein 27	Homo sapiens
1861	torsin family 1, member A (torsin A)	Homo sapiens
10445	microspherule protein 1	Homo sapiens
5813	purine-rich element binding protein A	Homo sapiens
4520	metal-regulatory transcription factor 1	Homo sapiens
84109	pyroglutamylated RFamide peptide receptor	Homo sapiens
27235	coenzyme Q2 homolog, prenyltransferase (yeast)	Homo sapiens
54899	PX domain containing serine/threonine kinase	Homo sapiens
26608	transducin (beta)-like 2	Homo sapiens
2121	Ellis van Creveld syndrome	Homo sapiens
285429	WD repeat domain 21B	Homo sapiens
22883	calsyntenin 1	Homo sapiens
24148	similar to U5 snRNP-associated 102 kDa protein (U5-102 kDa protein); PRP6 pre-mRNA processing factor 6 homolog (S. cerevisiae)	Homo sapiens
653889	similar to U5 snRNP-associated 102 kDa protein (U5-102 kDa protein); PRP6 pre-mRNA processing factor 6 homolog (S. cerevisiae)	Homo sapiens
400916	coiled-coil-helix-coiled-coil-helix domain containing 10	Homo sapiens
474338	SMT3 suppressor of mif two 3 homolog 1 (S. cerevisiae); SUMO1 pseudogene 3	Homo sapiens
7341	SMT3 suppressor of mif two 3 homolog 1 (S. cerevisiae); SUMO1 pseudogene 3	Homo sapiens
23509	protein O-fucosyltransferase 1	Homo sapiens
57116	zinc finger protein 695	Homo sapiens
10953	translocase of outer mitochondrial membrane 34	Homo sapiens
5966	v-rel reticuloendotheliosis viral oncogene homolog (avian)	Homo sapiens
9887	Smg-7 homolog, nonsense mediated mRNA decay factor (C. elegans)	Homo sapiens
27166	PRELI domain containing 1; similar to Px19-like protein (25 kDa protein of relevant evolutionary and lymphoid interest) (PRELI)	Homo sapiens
728666	PRELI domain containing 1; similar to Px19-like protein (25 kDa protein of relevant evolutionary and lymphoid interest) (PRELI)	Homo sapiens
5740	prostaglandin I2 (prostacyclin) synthase	Homo sapiens
9806	sparc/osteonectin, cwcv and kazal-like domains proteoglycan (testican) 2	Homo sapiens
11212	proline synthetase co-transcribed homolog (bacterial)	Homo sapiens
23471	translocation associated membrane protein 1	Homo sapiens
7813	ecotropic viral integration site 5	Homo sapiens
81875	interferon stimulated exonuclease gene 20kDa-like 2	Homo sapiens
55501	carbohydrate (chondroitin 4) sulfotransferase 12	Homo sapiens
3213	homeobox B3	Homo sapiens
391592	basic leucine zipper and W2 domains 1 pseudogene 1; basic leucine zipper and W2 domains 1 like 1; basic leucine zipper and W2 domains 1	Homo sapiens
151579	basic leucine zipper and W2 domains 1 pseudogene 1; basic leucine zipper and W2 domains 1 like 1; basic leucine zipper and W2 domains 1	Homo sapiens
9689	basic leucine zipper and W2 domains 1 pseudogene 1; basic leucine zipper and W2 domains 1 like 1; basic leucine zipper and W2 domains 1	Homo sapiens
57572	dedicator of cytokinesis 6	Homo sapiens
28991	COMM domain containing 5	Homo sapiens
9716	aquarius homolog (mouse)	Homo sapiens
7514	exportin 1 (CRM1 homolog, yeast)	Homo sapiens
3227	homeobox C11	Homo sapiens
25847	anaphase promoting complex subunit 13	Homo sapiens

8676	syntaxin 11	Homo sapiens
80345	zinc finger and SCAN domain containing 16	Homo sapiens
51427	zinc finger protein 107	Homo sapiens
430	achaete-scute complex homolog 2 (Drosophila)	Homo sapiens
93643	tight junction associated protein 1 (peripheral)	Homo sapiens
10856	RuvB-like 2 (E. coli)	Homo sapiens
11182	solute carrier family 2 (facilitated glucose transporter), member 6	Homo sapiens
50813	COP9 constitutive photomorphogenic homolog subunit 7A (Arabidopsis)	Homo sapiens
6625	small nuclear ribonucleoprotein 70kDa (U1)	Homo sapiens
1508	cathepsin B	Homo sapiens
9338	transcription elongation factor A (SII)-like 1	Homo sapiens
63967	claspin homolog (Xenopus laevis)	Homo sapiens
6718	aldo-keto reductase family 1, member D1 (delta 4-3-ketosteroid-5-beta-reductase)	Homo sapiens
79039	DEAD (Asp-Glu-Ala-Asp) box polypeptide 54	Homo sapiens
23705	cell adhesion molecule 1	Homo sapiens
8731	RNA (guanine-7-) methyltransferase	Homo sapiens
9900	synaptic vesicle glycoprotein 2A	Homo sapiens
8036	soc-2 suppressor of clear homolog (C. elegans)	Homo sapiens
56141	protocadherin alpha 7	Homo sapiens
11231	SEC63 homolog (S. cerevisiae)	Homo sapiens
28969	basic leucine zipper and W2 domains 2	Homo sapiens
4153	mannose-binding lectin (protein C) 2, soluble (opsonic defect)	Homo sapiens
729747	similar to zinc finger protein 709	Homo sapiens
5573	protein kinase, cAMP-dependent, regulatory, type I, alpha (tissue specific extinguisher 1)	Homo sapiens
1950	epidermal growth factor (beta-urogastrone)	Homo sapiens
6888	transaldolase 1	Homo sapiens
7704	zinc finger and BTB domain containing 16	Homo sapiens
8540	alkylglycerone phosphate synthase	Homo sapiens
55824	phosphoprotein associated with glycosphingolipid microdomains 1	Homo sapiens
54514	DEAD (Asp-Glu-Ala-Asp) box polypeptide 4	Homo sapiens
79685	SAP30-like	Homo sapiens
84260	trichoplein, keratin filament binding	Homo sapiens
3034	histidine ammonia-lyase	Homo sapiens
79084	WD repeat domain 77	Homo sapiens
8570	KH-type splicing regulatory protein	Homo sapiens
5435	polymerase (RNA) II (DNA directed) polypeptide F	Homo sapiens
23140	zinc finger, ZZ-type with EF-hand domain 1	Homo sapiens
400720	zinc finger protein 772	Homo sapiens
6774	signal transducer and activator of transcription 3 (acute-phase response factor)	Homo sapiens
6626	small nuclear ribonucleoprotein polypeptide A	Homo sapiens
4043	low density lipoprotein receptor-related protein associated protein 1	Homo sapiens
5286	phosphoinositide-3-kinase, class 2, alpha polypeptide	Homo sapiens
1954	multiple EGF-like-domains 8	Homo sapiens
3454	interferon (alpha, beta and omega) receptor 1	Homo sapiens
80152	centromere protein T	Homo sapiens
3990	lipase, hepatic	Homo sapiens
11311	vacuolar protein sorting 45 homolog (S. cerevisiae)	Homo sapiens
56980	PR domain containing 10	Homo sapiens
642393	similar to mitochondrial ribosomal protein L20; mitochondrial ribosomal protein L20	Homo sapiens
55052	similar to mitochondrial ribosomal protein L20; mitochondrial ribosomal protein L20	Homo sapiens
126637	trichohyalin-like 1	Homo sapiens
7073	TIA1 cytotoxic granule-associated RNA binding protein-like 1	Homo sapiens
85480	thymic stromal lymphopoietin	Homo sapiens
57799	RAB40C, member RAS oncogene family	Homo sapiens
81789	tigger transposable element derived 6	Homo sapiens
57035	chromosome 1 open reading frame 63	Homo sapiens
3992	fatty acid desaturase 1	Homo sapiens
10471	prefoldin subunit 6	Homo sapiens
56975	family with sequence similarity 20, member C	Homo sapiens
2326	flavin containing monooxygenase 1	Homo sapiens
1849	dual specificity phosphatase 7	Homo sapiens
9832	Janus kinase and microtubule interacting protein 2	Homo sapiens
79788	zinc finger protein 665	Homo sapiens
23107	mitochondrial ribosomal protein S27	Homo sapiens
3983	actin binding LIM protein 1	Homo sapiens
948	CD36 molecule (thrombospondin receptor)	Homo sapiens
161291	transmembrane protein 30B	Homo sapiens
3423	iduronate 2-sulfatase	Homo sapiens
8971	H1 histone family, member X	Homo sapiens
280636	chromosome 11 open reading frame 31	Homo sapiens
9497	solute carrier family 4, sodium bicarbonate cotransporter, member 7	Homo sapiens
353189	solute carrier organic anion transporter family, member 4C1	Homo sapiens
332	baculoviral IAP repeat-containing 5	Homo sapiens
728621	hypothetical protein LOC728621	Homo sapiens
5470	protein phosphatase, EF-hand calcium binding domain 2	Homo sapiens
10628	thioredoxin interacting protein	Homo sapiens
64960	mitochondrial ribosomal protein S15	Homo sapiens
54558	spermatogenesis associated 6	Homo sapiens

1004	cadherin 6, type 2, K-cadherin (fetal kidney)	Homo sapiens
684959	TATA box binding protein (TBP)-associated factor, RNA polymerase I, D, 41kDa; small nucleolar RNA, H/ACA box 32; small nucleolar RNA, H/ACA box 25	Homo sapiens
79101	TATA box binding protein (TBP)-associated factor, RNA polymerase I, D, 41kDa; small nucleolar RNA, H/ACA box 32; small nucleolar RNA, H/ACA box 25	Homo sapiens
692063	TATA box binding protein (TBP)-associated factor, RNA polymerase I, D, 41kDa; small nucleolar RNA, H/ACA box 32; small nucleolar RNA, H/ACA box 25	Homo sapiens
84532	acyl-CoA synthetase short-chain family member 1	Homo sapiens
7733	zinc finger protein 180	Homo sapiens
567	beta-2-microglobulin	Homo sapiens
55339	WD repeat domain 33	Homo sapiens
167555	family with sequence similarity 151, member B	Homo sapiens
84233	transmembrane protein 126A	Homo sapiens
5934	retinoblastoma-like 2 (p130)	Homo sapiens
55374	transmembrane and coiled-coil domains 6	Homo sapiens
80262	chromosome 16 open reading frame 70	Homo sapiens
64708	COP9 constitutive photomorphogenic homolog subunit 7B (Arabidopsis)	Homo sapiens
9469	carbohydrate (chondroitin 6) sulfotransferase 3	Homo sapiens
56475	reprimo, TP53 dependent G2 arrest mediator candidate	Homo sapiens
7862	bromodomain and PHD finger containing, 1	Homo sapiens
199699	DAN domain family, member 5	Homo sapiens
6011	G protein-coupled receptor kinase 1	Homo sapiens
201931	transmembrane protein 192	Homo sapiens
81572	p53 and DNA-damage regulated 1	Homo sapiens
23367	La ribonucleoprotein domain family, member 1	Homo sapiens
128486	fat storage-inducing transmembrane protein 2	Homo sapiens
55627	sphingomyelin phosphodiesterase 4, neutral membrane (neutral sphingomyelinase-3)	Homo sapiens
221477	chromosome 6 open reading frame 89	Homo sapiens
378807	cation channel, sperm associated 4	Homo sapiens
115426	ubiquitin-like with PHD and ring finger domains 2	Homo sapiens
10240	mitochondrial ribosomal protein S31	Homo sapiens
54852	progestin and adipoQ receptor family member V	Homo sapiens
128876	family with sequence similarity 83, member C	Homo sapiens
26276	vacuolar protein sorting 33 homolog B (yeast)	Homo sapiens
285513	GPRIN family member 3	Homo sapiens
5334	phospholipase C-like 1	Homo sapiens
5442	polymerase (RNA) mitochondrial (DNA directed)	Homo sapiens
9472	A kinase (PRKA) anchor protein 6	Homo sapiens
400011	suppressor of Ty 16 homolog (S. cerevisiae); suppressor of Ty 16 homolog (S. cerevisiae) pseudogene	Homo sapiens
11198	suppressor of Ty 16 homolog (S. cerevisiae); suppressor of Ty 16 homolog (S. cerevisiae) pseudogene	Homo sapiens
80739	chromosome 6 open reading frame 25	Homo sapiens
55695	NOL1/NOP2/Sun domain family, member 5	Homo sapiens
56902	partner of NOB1 homolog (S. cerevisiae)	Homo sapiens
10681	guanine nucleotide binding protein (G protein), beta 5	Homo sapiens
7204	triple functional domain (PTPRF interacting)	Homo sapiens
23305	acyl-CoA synthetase long-chain family member 6	Homo sapiens
2317	filamin B, beta (actin binding protein 278)	Homo sapiens
1406	cone-rod homeobox	Homo sapiens
51307	family with sequence similarity 53, member C	Homo sapiens
10802	SEC24 family, member A (S. cerevisiae)	Homo sapiens
9750	family with sequence similarity 65, member B	Homo sapiens
55336	F-box and leucine-rich repeat protein 8	Homo sapiens
646347	spermine synthase; similar to spermine synthase	Homo sapiens
642590	spermine synthase; similar to spermine synthase	Homo sapiens
6611	spermine synthase; similar to spermine synthase	Homo sapiens
85477	scinderin	Homo sapiens
8322	frizzled homolog 4 (Drosophila)	Homo sapiens
171484	family with sequence similarity 9, member C	Homo sapiens
7083	thymidine kinase 1, soluble	Homo sapiens
54964	chromosome 1 open reading frame 56	Homo sapiens
27297	CGRP receptor component	Homo sapiens
27434	polymerase (DNA directed), mu	Homo sapiens
729143	myosin phosphatase Rho interacting protein; similar to Myosin phosphatase Rho-interacting protein (Rho-interacting protein 3) (M-RIP) (RIP3) (p116Rip)	Homo sapiens
23164	myosin phosphatase Rho interacting protein; similar to Myosin phosphatase Rho-interacting protein (Rho-interacting protein 3) (M-RIP) (RIP3) (p116Rip)	Homo sapiens
7357	UDP-glucose ceramide glucosyltransferase	Homo sapiens
114990	vasorin	Homo sapiens
8106	poly(A) binding protein, nuclear 1	Homo sapiens
154881	potassium channel tetramerisation domain containing 7	Homo sapiens
10220	growth differentiation factor 11	Homo sapiens
55830	glycosyltransferase 8 domain containing 1	Homo sapiens
4790	nuclear factor of kappa light polypeptide gene enhancer in B-cells 1	Homo sapiens
65078	reticulon 4 receptor	Homo sapiens
100037280	ubiquitin-conjugating enzyme E2D 3 (UBC4/5 homolog, yeast); ubiquitin-conjugating enzyme E2D 3 pseudogene	Homo sapiens

7323	ubiquitin-conjugating enzyme E2D 3 (UBC4/5 homolog, yeast); ubiquitin-conjugating enzyme E2D 3 pseudogene	Homo sapiens
25973	prolyl-tRNA synthetase 2, mitochondrial (putative)	Homo sapiens
3308	heat shock 70kDa protein 4	Homo sapiens
4539	NADH-ubiquinone oxidoreductase chain 4L; NADH-ubiquinone oxidoreductase chain 4	Homo sapiens
4538	NADH-ubiquinone oxidoreductase chain 4L; NADH-ubiquinone oxidoreductase chain 4	Homo sapiens
6775072	NADH-ubiquinone oxidoreductase chain 4L; NADH-ubiquinone oxidoreductase chain 4	Homo sapiens
6775071	NADH-ubiquinone oxidoreductase chain 4L; NADH-ubiquinone oxidoreductase chain 4	Homo sapiens
64771	chromosome 6 open reading frame 106	Homo sapiens
53340	sperm autoantigenic protein 17	Homo sapiens
11279	Kruppel-like factor 8	Homo sapiens
55054	ATG16 autophagy related 16-like 1 (S. cerevisiae)	Homo sapiens
116983	ArfGAP with coiled-coil, ankyrin repeat and PH domains 3	Homo sapiens
11334	tumor suppressor candidate 2	Homo sapiens
56254	ring finger protein 20	Homo sapiens
22950	solute carrier family 4 (anion exchanger), member 1, adaptor protein	Homo sapiens
9053	microtubule-associated protein 7	Homo sapiens
343578	chromosome 20 open reading frame 95	Homo sapiens
10746	mitogen-activated protein kinase kinase kinase 2	Homo sapiens
399979	sorting nexin 19	Homo sapiens
3037	hyaluronan synthase 2	Homo sapiens
7975	v-maf musculoaponeurotic fibrosarcoma oncogene homolog K (avian)	Homo sapiens
23336	synemin, intermediate filament protein	Homo sapiens
2618	phosphoribosylglycinamide formyltransferase, phosphoribosylglycinamide synthetase, phosphoribosylaminoimidazole synthetase	Homo sapiens
55216	chromosome 11 open reading frame 57	Homo sapiens
91368	CDKN2A interacting protein N-terminal like	Homo sapiens
3516	recombination signal binding protein for immunoglobulin kappa J region	Homo sapiens
63979	fidgetin-like 1	Homo sapiens
4289	muskelin 1, intracellular mediator containing kelch motifs	Homo sapiens
254272	TBC1 domain family, member 28	Homo sapiens
22906	trafficking protein, kinesin binding 1	Homo sapiens
10773	zinc finger and BTB domain containing 6	Homo sapiens
2736	GLI family zinc finger 2	Homo sapiens
857	caveolin 1, caveolae protein, 22kDa	Homo sapiens
79735	TBC1 domain family, member 17	Homo sapiens
9927	mitofusin 2	Homo sapiens
651	bone morphogenetic protein 3	Homo sapiens
85236	histone cluster 1, H2bk	Homo sapiens
6838	surfeit 6	Homo sapiens
1857	dishevelled, dsh homolog 3 (Drosophila)	Homo sapiens
7417	voltage-dependent anion channel 2	Homo sapiens
815	calcium/calmodulin-dependent protein kinase II alpha	Homo sapiens
128637	TBC1 domain family, member 20	Homo sapiens
100271831	hypothetical LOC100271831; mitogen-activated protein kinase 3	Homo sapiens
5595	hypothetical LOC100271831; mitogen-activated protein kinase 3	Homo sapiens
113189	carbohydrate (N-acetylgalactosamine 4-0) sulfotransferase 14	Homo sapiens
9559	vacuolar protein sorting 26 homolog A (S. pombe)	Homo sapiens
2887	growth factor receptor-bound protein 10	Homo sapiens
27044	staphylococcal nuclease and tudor domain containing 1	Homo sapiens
11190	centrosomal protein 250kDa	Homo sapiens
1371	coproporphyrinogen oxidase	Homo sapiens
7067	thyroid hormone receptor, alpha (erythroblastic leukemia viral (v-erb-a) oncogene homolog, avian)	Homo sapiens
1615	aspartyl-tRNA synthetase	Homo sapiens
150468	cytoskeleton associated protein 2-like	Homo sapiens
25977	NECAP endocytosis associated 1	Homo sapiens
55187	vacuolar protein sorting 13 homolog D (S. cerevisiae)	Homo sapiens
84270	chromosome 9 open reading frame 89	Homo sapiens
57180	ARP3 actin-related protein 3 homolog B (yeast)	Homo sapiens
84612	par-6 partitioning defective 6 homolog beta (C. elegans)	Homo sapiens
112398	egl nine homolog 2 (C. elegans)	Homo sapiens
1185	chloride channel 6	Homo sapiens
26953	RAN binding protein 6	Homo sapiens
85016	chromosome 11 open reading frame 70	Homo sapiens
51379	cytokine receptor-like factor 3	Homo sapiens
55326	1-acylglycerol-3-phosphate O-acyltransferase 5 (lysophosphatidic acid acyltransferase, epsilon)	Homo sapiens
4074	mannose-6-phosphate receptor (cation dependent)	Homo sapiens
6689	Spi-B transcription factor (Spi-1/PU.1 related)	Homo sapiens
55312	riboflavin kinase	Homo sapiens
474383	coagulation factor VIII-associated (intronic transcript) 2; coagulation factor VIII-associated (intronic transcript) 3; coagulation factor VIII-associated (intronic transcript) 1	Homo sapiens
474384	coagulation factor VIII-associated (intronic transcript) 2; coagulation factor VIII-associated (intronic transcript) 3; coagulation factor VIII-associated (intronic transcript) 1	Homo sapiens

8263	coagulation factor VIII-associated (intronic transcript) 2; coagulation factor VIII-associated (intronic transcript) 3; coagulation factor VIII-associated (intronic transcript) 1	Homo sapiens
3837	karyopherin (importin) beta 1	Homo sapiens
92815	histone cluster 3, H2a	Homo sapiens
91749	KIAA1919	Homo sapiens
56882	CDC42 small effector 1	Homo sapiens
10036	chromatin assembly factor 1, subunit A (p150)	Homo sapiens
57514	Cdc42 GTPase-activating protein	Homo sapiens
79930	docking protein 3	Homo sapiens
130074	family with sequence similarity 168, member B	Homo sapiens
83935	transmembrane protein 133	Homo sapiens
667	dystonin	Homo sapiens
57630	SH3 domain containing ring finger 1	Homo sapiens
5300	peptidylprolyl cis/trans isomerase, NIMA-interacting 1	Homo sapiens
6535	solute carrier family 6 (neurotransmitter transporter, creatine), member 8	Homo sapiens
7818	death associated protein 3	Homo sapiens
6189	ribosomal protein S3A pseudogene 5; ribosomal protein S3a pseudogene 47; ribosomal protein S3a pseudogene 49; ribosomal protein S3A; hypothetical LOC100131699; hypothetical LOC100130107	Homo sapiens
100130107	ribosomal protein S3A pseudogene 5; ribosomal protein S3a pseudogene 47; ribosomal protein S3a pseudogene 49; ribosomal protein S3A; hypothetical LOC100131699; hypothetical LOC100130107	Homo sapiens
400652	ribosomal protein S3A pseudogene 5; ribosomal protein S3a pseudogene 47; ribosomal protein S3a pseudogene 49; ribosomal protein S3A; hypothetical LOC100131699; hypothetical LOC100130107	Homo sapiens
146053	ribosomal protein S3A pseudogene 5; ribosomal protein S3a pseudogene 47; ribosomal protein S3a pseudogene 49; ribosomal protein S3A; hypothetical LOC100131699; hypothetical LOC100130107	Homo sapiens
439992	ribosomal protein S3A pseudogene 5; ribosomal protein S3a pseudogene 47; ribosomal protein S3a pseudogene 49; ribosomal protein S3A; hypothetical LOC100131699; hypothetical LOC100130107	Homo sapiens
100131699	ribosomal protein S3A pseudogene 5; ribosomal protein S3a pseudogene 47; ribosomal protein S3a pseudogene 49; ribosomal protein S3A; hypothetical LOC100131699; hypothetical LOC100130107	Homo sapiens
79742	chromosome X open reading frame 36	Homo sapiens
645317	coiled-coil-helix-coiled-coil-helix domain containing 2; similar to coiled-coil-helix-coiled-coil-helix domain containing 2	Homo sapiens
728324	coiled-coil-helix-coiled-coil-helix domain containing 2; similar to coiled-coil-helix-coiled-coil-helix domain containing 2	Homo sapiens
51142	coiled-coil-helix-coiled-coil-helix domain containing 2; similar to coiled-coil-helix-coiled-coil-helix domain containing 2	Homo sapiens
7371	uridine-cytidine kinase 2	Homo sapiens
84919	protein phosphatase 1, regulatory (inhibitor) subunit 15B	Homo sapiens
84255	solute carrier family 37 (glycerol-3-phosphate transporter), member 3	Homo sapiens
84549	MAK16 homolog (S. cerevisiae)	Homo sapiens
2872	MAP kinase interacting serine/threonine kinase 2	Homo sapiens
9688	nucleoporin 93kDa	Homo sapiens
282996	RNA binding motif protein 20	Homo sapiens
200030	neuroblastoma breakpoint family, member 15; neuroblastoma breakpoint family, member 14; neuroblastoma breakpoint family, member 9; neuroblastoma breakpoint family, member 11; neuroblastoma breakpoint family, member 11-like; neuroblastoma breakpoint family, member 10; neuroblastoma breakpoint family, member 12; neuroblastoma breakpoint family, member 20; neuroblastoma breakpoint family, member 1; KIAA1245; neuroblastoma breakpoint family, member 8	Homo sapiens
149013	neuroblastoma breakpoint family, member 15; neuroblastoma breakpoint family, member 14; neuroblastoma breakpoint family, member 9; neuroblastoma breakpoint family, member 11; neuroblastoma breakpoint family, member 11-like; neuroblastoma breakpoint family, member 10; neuroblastoma breakpoint family, member 12; neuroblastoma breakpoint family, member 20; neuroblastoma breakpoint family, member 1; KIAA1245; neuroblastoma breakpoint family, member 8	Homo sapiens
728841	neuroblastoma breakpoint family, member 15; neuroblastoma breakpoint family, member 14; neuroblastoma breakpoint family, member 9; neuroblastoma breakpoint family, member 11; neuroblastoma breakpoint family, member 11-like; neuroblastoma breakpoint family, member 10; neuroblastoma breakpoint family, member 12; neuroblastoma breakpoint family, member 20; neuroblastoma breakpoint family, member 1; KIAA1245; neuroblastoma breakpoint family, member 8	Homo sapiens
100132406	neuroblastoma breakpoint family, member 15; neuroblastoma breakpoint family, member 14; neuroblastoma breakpoint family, member 9; neuroblastoma breakpoint family, member 11; neuroblastoma breakpoint family, member 11-like; neuroblastoma breakpoint family, member 10; neuroblastoma breakpoint family, member 12; neuroblastoma breakpoint family, member 20; neuroblastoma breakpoint family, member 1; KIAA1245; neuroblastoma breakpoint family, member 8	Homo sapiens
400818	neuroblastoma breakpoint family, member 15; neuroblastoma breakpoint family, member 14; neuroblastoma breakpoint family, member 9; neuroblastoma breakpoint family, member 11; neuroblastoma breakpoint family, member 11-like; neuroblastoma breakpoint family, member 10; neuroblastoma breakpoint family, member 12; neuroblastoma breakpoint family, member 20; neuroblastoma breakpoint family, member 1; KIAA1245; neuroblastoma breakpoint family, member 8	Homo sapiens

440675	neuroblastoma breakpoint family, member 15; neuroblastoma breakpoint family, member 14; neuroblastoma breakpoint family, member 9; neuroblastoma breakpoint family, member 11; neuroblastoma breakpoint family, member 11-like; neuroblastoma breakpoint family, member 10; neuroblastoma breakpoint family, member 12; neuroblastoma breakpoint family, member 20; neuroblastoma breakpoint family, member 1; KIAA1245; neuroblastoma breakpoint family, member 8	Homo sapiens
55672	neuroblastoma breakpoint family, member 15; neuroblastoma breakpoint family, member 14; neuroblastoma breakpoint family, member 9; neuroblastoma breakpoint family, member 11; neuroblastoma breakpoint family, member 11-like; neuroblastoma breakpoint family, member 10; neuroblastoma breakpoint family, member 12; neuroblastoma breakpoint family, member 20; neuroblastoma breakpoint family, member 1; KIAA1245; neuroblastoma breakpoint family, member 8	Homo sapiens
25832	neuroblastoma breakpoint family, member 15; neuroblastoma breakpoint family, member 14; neuroblastoma breakpoint family, member 9; neuroblastoma breakpoint family, member 11; neuroblastoma breakpoint family, member 11-like; neuroblastoma breakpoint family, member 10; neuroblastoma breakpoint family, member 12; neuroblastoma breakpoint family, member 20; neuroblastoma breakpoint family, member 1; KIAA1245; neuroblastoma breakpoint family, member 8	Homo sapiens
728912	neuroblastoma breakpoint family, member 15; neuroblastoma breakpoint family, member 14; neuroblastoma breakpoint family, member 9; neuroblastoma breakpoint family, member 11; neuroblastoma breakpoint family, member 11-like; neuroblastoma breakpoint family, member 10; neuroblastoma breakpoint family, member 12; neuroblastoma breakpoint family, member 20; neuroblastoma breakpoint family, member 1; KIAA1245; neuroblastoma breakpoint family, member 8	Homo sapiens
440670	neuroblastoma breakpoint family, member 15; neuroblastoma breakpoint family, member 14; neuroblastoma breakpoint family, member 9; neuroblastoma breakpoint family, member 11; neuroblastoma breakpoint family, member 11-like; neuroblastoma breakpoint family, member 10; neuroblastoma breakpoint family, member 12; neuroblastoma breakpoint family, member 20; neuroblastoma breakpoint family, member 1; KIAA1245; neuroblastoma breakpoint family, member 8	Homo sapiens
284565	neuroblastoma breakpoint family, member 15; neuroblastoma breakpoint family, member 14; neuroblastoma breakpoint family, member 9; neuroblastoma breakpoint family, member 11; neuroblastoma breakpoint family, member 11-like; neuroblastoma breakpoint family, member 10; neuroblastoma breakpoint family, member 12; neuroblastoma breakpoint family, member 20; neuroblastoma breakpoint family, member 1; KIAA1245; neuroblastoma breakpoint family, member 8	Homo sapiens
165918	ring finger protein 168	Homo sapiens
54821	excision repair cross-complementing rodent repair deficiency, complementation group 6-like	Homo sapiens
26019	UPF2 regulator of nonsense transcripts homolog (yeast)	Homo sapiens
84286	transmembrane protein 175	Homo sapiens
2395	frataxin	Homo sapiens
129685	TAF8 RNA polymerase II, TATA box binding protein (TBP)-associated factor, 43kDa	Homo sapiens
113178	secretory carrier membrane protein 4	Homo sapiens
2967	general transcription factor IIH, polypeptide 3, 34kDa	Homo sapiens
55837	E2F-associated phosphoprotein	Homo sapiens
83590	transmembrane and ubiquitin-like domain containing 1	Homo sapiens
79666	pleckstrin homology domain containing, family F (with FYVE domain) member 2	Homo sapiens
130367	sphingosine-1-phosphate phosphotase 2	Homo sapiens
3324	heat shock protein 90kDa alpha (cytosolic), class A member 2; heat shock protein 90kDa alpha (cytosolic), class A member 1	Homo sapiens
3320	heat shock protein 90kDa alpha (cytosolic), class A member 2; heat shock protein 90kDa alpha (cytosolic), class A member 1	Homo sapiens
9045	ribosomal protein L14	Homo sapiens
5462	POU class 5 homeobox 1B	Homo sapiens
375061	family with sequence similarity 89, member A	Homo sapiens
246181	aldo-keto reductase family 7-like	Homo sapiens
260425	membrane associated guanylate kinase, WW and PDZ domain containing 3	Homo sapiens
23074	UHRF1 binding protein 1-like	Homo sapiens
23671	transmembrane protein with EGF-like and two follistatin-like domains 2	Homo sapiens
51248	PDZ domain containing 11	Homo sapiens
55802	DCP1 decapping enzyme homolog A (S. cerevisiae)	Homo sapiens
11269	DEAD (Asp-Glu-Ala-As) box polypeptide 19B	Homo sapiens
83716	cysteine-rich secretory protein LCCL domain containing 2	Homo sapiens
7345	ubiquitin carboxyl-terminal esterase L1 (ubiquitin thiolesterase)	Homo sapiens
90060	coiled-coil domain containing 120	Homo sapiens
55287	transmembrane protein 40	Homo sapiens
51218	glutaredoxin 5	Homo sapiens
7320	ubiquitin-conjugating enzyme E2B (RAD6 homolog)	Homo sapiens
200186	CREB regulated transcription coactivator 2	Homo sapiens
8803	succinate-CoA ligase, ADP-forming, beta subunit	Homo sapiens
23510	potassium channel tetramerisation domain containing 2	Homo sapiens
81	actinin, alpha 4	Homo sapiens
10519	calcium and integrin binding 1 (calmyrin)	Homo sapiens
5580	protein kinase C, delta	Homo sapiens
8821	inositol polyphosphate-4-phosphatase, type II, 105kDa	Homo sapiens
29888	striatin, calmodulin binding protein 4	Homo sapiens
23034	sterile alpha motif domain containing 4A	Homo sapiens
51020	HD domain containing 2	Homo sapiens

79677	structural maintenance of chromosomes 6	Homo sapiens
3098	hexokinase 1	Homo sapiens
9533	polymerase (RNA) I polypeptide C, 30kDa	Homo sapiens
51338	membrane-spanning 4-domains, subfamily A, member 4	Homo sapiens
9378	neurexin 1	Homo sapiens
55033	FK506 binding protein 14, 22 kDa	Homo sapiens
63027	solute carrier family 22, member 23	Homo sapiens
55127	HEAT repeat containing 1	Homo sapiens
57545	coiled-coil and C2 domain containing 2A	Homo sapiens
54554	WD repeat domain 5B	Homo sapiens
57645	pogo transposable element with KRAB domain	Homo sapiens
4603	v-myb myeloblastosis viral oncogene homolog (avian)-like 1	Homo sapiens
2288	FK506 binding protein 4, 59kDa	Homo sapiens
27072	vacuolar protein sorting 41 homolog (S. cerevisiae)	Homo sapiens
10241	calcium binding and coiled-coil domain 2	Homo sapiens
4194	Mdm4 p53 binding protein homolog (mouse)	Homo sapiens
10487	CAP, adenylate cyclase-associated protein 1 (yeast)	Homo sapiens
148808	major facilitator superfamily domain containing 4	Homo sapiens
26133	transient receptor potential cation channel, subfamily C, member 4 associated protein	Homo sapiens
56852	RAD18 homolog (S. cerevisiae)	Homo sapiens
84133	zinc and ring finger 3	Homo sapiens
114790	serine/threonine kinase 11 interacting protein	Homo sapiens
8604	solute carrier family 25 (mitochondrial carrier, Aralar), member 12	Homo sapiens
23275	protein O-fucosyltransferase 2	Homo sapiens
54498	spermine oxidase	Homo sapiens
24140	FtsJ homolog 1 (E. coli)	Homo sapiens
56252	YLP motif containing 1	Homo sapiens
1657	Dmx-like 1	Homo sapiens
83931	serine/threonine kinase 40	Homo sapiens
4802	nuclear transcription factor Y, gamma	Homo sapiens
4839	NOP2 nucleolar protein homolog (yeast)	Homo sapiens
11051	nudix (nucleoside diphosphate linked moiety X)-type motif 21	Homo sapiens
57216	vang-like 2 (van gogh, Drosophila)	Homo sapiens
57670	KIAA1549	Homo sapiens
55023	pleckstrin homology domain interacting protein	Homo sapiens
145957	neuregulin 4	Homo sapiens
3660	interferon regulatory factor 2	Homo sapiens
116966	WD repeat domain 17	Homo sapiens
339287	male-specific lethal 1 homolog (Drosophila)	Homo sapiens
10758	TRAF3 interacting protein 2	Homo sapiens
5774	protein tyrosine phosphatase, non-receptor type 3	Homo sapiens
64094	SPARC related modular calcium binding 2	Homo sapiens
1497	cystinosis, nephropathic	Homo sapiens
375341	chromosome 3 open reading frame 62	Homo sapiens
84874	zinc finger protein 514	Homo sapiens
6747	signal sequence receptor, gamma (translocon-associated protein gamma)	Homo sapiens
201973	coiled-coil domain containing 111	Homo sapiens
1340	cytochrome c oxidase subunit Vib polypeptide 1 (ubiquitous)	Homo sapiens
6257	retinoid X receptor, beta	Homo sapiens
23163	golgi associated, gamma adaptin ear containing, ARF binding protein 3	Homo sapiens
56995	tubby like protein 4	Homo sapiens
55353	lysosomal protein transmembrane 4 beta	Homo sapiens
8874	Rho guanine nucleotide exchange factor (GEF) 7	Homo sapiens
5756	twinfilin, actin-binding protein, homolog 1 (Drosophila)	Homo sapiens
84467	fibrillin 3	Homo sapiens
23014	F-box protein 21	Homo sapiens
5784	protein tyrosine phosphatase, non-receptor type 14	Homo sapiens
4837	nicotinamide N-methyltransferase	Homo sapiens
51542	vacuolar protein sorting 54 homolog (S. cerevisiae)	Homo sapiens
5621	prion protein	Homo sapiens
9673	solute carrier family 25, member 44	Homo sapiens
10363	high-mobility group 20A	Homo sapiens
143686	sestrin 3	Homo sapiens
7185	TNF receptor-associated factor 1	Homo sapiens
6167	ribosomal protein L37	Homo sapiens
9406	zinc finger, RAN-binding domain containing 2	Homo sapiens
653308	N-acylsphingosine amidohydrolase (non-lysosomal ceramidase) 2B	Homo sapiens
128077	Lix1 homolog (mouse)-like	Homo sapiens
84277	DnaJ (Hsp40) homolog, subfamily C, member 30	Homo sapiens
4773	nuclear factor of activated T-cells, cytoplasmic, calcineurin-dependent 2	Homo sapiens
7727	zinc finger protein 174	Homo sapiens
54960	gem (nuclear organelle) associated protein 8	Homo sapiens
163	adaptor-related protein complex 2, beta 1 subunit	Homo sapiens
9185	RALBP1 associated Eps domain containing 2	Homo sapiens
145645	chromosome 15 open reading frame 43	Homo sapiens
7379	uroplakin 2	Homo sapiens
5324	pleiomorphic adenoma gene 1	Homo sapiens
3010	histone cluster 1, H1t	Homo sapiens
7071	Kruppel-like factor 10	Homo sapiens

23008	kelch domain containing 10	Homo sapiens
6218	ribosomal protein S17	Homo sapiens
30008	EGF-containing fibulin-like extracellular matrix protein 2	Homo sapiens
7531	similar to 14-3-3 protein epsilon (14-3-3E) (Mitochondrial import stimulation factor L subunit) (MSF L); tyrosine 3-monooxygenase/tryptophan 5-monooxygenase activation protein, epsilon polypeptide	Homo sapiens
440917	similar to 14-3-3 protein epsilon (14-3-3E) (Mitochondrial import stimulation factor L subunit) (MSF L); tyrosine 3-monooxygenase/tryptophan 5-monooxygenase activation protein, epsilon polypeptide	Homo sapiens
6236	Ras-related associated with diabetes	Homo sapiens
8073	protein tyrosine phosphatase type IVA, member 2	Homo sapiens
11188	nischarin	Homo sapiens
1718	24-dehydrocholesterol reductase	Homo sapiens
3251	hypoxanthine phosphoribosyltransferase 1	Homo sapiens
64943	5'-nucleotidase domain containing 2	Homo sapiens
51444	ring finger protein 138	Homo sapiens
51277	DnaJ (Hsp40) homolog, subfamily C, member 27	Homo sapiens
254778	chromosome 8 open reading frame 46	Homo sapiens
64921	CAS1 domain containing 1	Homo sapiens
25942	SIN3 homolog A, transcription regulator (yeast)	Homo sapiens
159013	chromosome X open reading frame 38	Homo sapiens
57134	mannosidase, alpha, class 1C, member 1	Homo sapiens
3725	jun oncogene	Homo sapiens
6836	surfeit 4	Homo sapiens
27183	vacuolar protein sorting 4 homolog A (S. cerevisiae)	Homo sapiens
6645	syntrophin, beta 2 (dystrophin-associated protein A1, 59kDa, basic component 2)	Homo sapiens
85300	ataxia, cerebellar, Cayman type	Homo sapiens
57589	KIAA1432	Homo sapiens
58473	pleckstrin homology domain containing, family B (evectins) member 1	Homo sapiens
3164	nuclear receptor subfamily 4, group A, member 1	Homo sapiens
23048	formin binding protein 1	Homo sapiens
79784	myosin, heavy chain 14	Homo sapiens
140700	sterile alpha motif domain containing 10	Homo sapiens
3209	homeobox A13	Homo sapiens
5062	p21 protein (Cdc42/Rac)-activated kinase 2	Homo sapiens
7372	uridine monophosphate synthetase	Homo sapiens
9827	RGP1 retrograde golgi transport homolog (S. cerevisiae)	Homo sapiens
144983	heterogeneous nuclear ribonucleoprotein A1-like 2	Homo sapiens
7360	UDP-glucose pyrophosphorylase 2	Homo sapiens
57532	nuclear fragile X mental retardation protein interacting protein 2	Homo sapiens
5347	polo-like kinase 1 (Drosophila)	Homo sapiens
25917	THUMP domain containing 3	Homo sapiens
131583	family with sequence similarity 43, member A	Homo sapiens
359948	interferon regulatory factor 2 binding protein 2	Homo sapiens
114787	G protein regulated inducer of neurite outgrowth 1	Homo sapiens
1316	Kruppel-like factor 6	Homo sapiens
51808	phosphorylated adaptor for RNA export	Homo sapiens
51619	ubiquitin-conjugating enzyme E2D 4 (putative)	Homo sapiens
6491	SCL/TAL1 interrupting locus	Homo sapiens
5189	peroxisomal biogenesis factor 1	Homo sapiens
51274	Kruppel-like factor 3 (basic)	Homo sapiens
9521	eukaryotic translation elongation factor 1 epsilon 1	Homo sapiens
115361	guanylate binding protein 4	Homo sapiens
219771	cyclin Y	Homo sapiens
10210	topoisomerase I binding, arginine/serine-rich	Homo sapiens
1478	cleavage stimulation factor, 3' pre-RNA, subunit 2, 64kDa	Homo sapiens
2035	erythrocyte membrane protein band 4.1 (elliptocytosis 1, RH-linked)	Homo sapiens
6193	ribosomal protein S5	Homo sapiens
644131	similar to chaperonin containing TCP1, subunit 8 (theta); chaperonin containing TCP1, subunit 8 (theta)	Homo sapiens
10694	similar to chaperonin containing TCP1, subunit 8 (theta); chaperonin containing TCP1, subunit 8 (theta)	Homo sapiens
220047	coiled-coil domain containing 83	Homo sapiens
9918	non-SMC condensin I complex, subunit D2	Homo sapiens
100133770	hypothetical protein LOC100133770; vacuolar protein sorting 35 homolog (S. cerevisiae)	Homo sapiens
55737	hypothetical protein LOC100133770; vacuolar protein sorting 35 homolog (S. cerevisiae)	Homo sapiens
23047	PDS5, regulator of cohesion maintenance, homolog B (S. cerevisiae)	Homo sapiens
64864	regulatory factor X, 7	Homo sapiens
57597	BAH domain and coiled-coil containing 1	Homo sapiens
8091	high mobility group AT-hook 2	Homo sapiens
5089	pre-B-cell leukemia homeobox 2	Homo sapiens
7832	BTG family, member 2	Homo sapiens
439921	matrix-remodelling associated 7	Homo sapiens
10970	cytoskeleton-associated protein 4	Homo sapiens
8345	histone cluster 1, H2bh	Homo sapiens
7316	ubiquitin C	Homo sapiens
4276	MHC class I polypeptide-related sequence A	Homo sapiens
116068	LysM, putative peptidoglycan-binding, domain containing 3	Homo sapiens
157	adrenergic, beta, receptor kinase 2	Homo sapiens

28996	homeodomain interacting protein kinase 2; similar to homeodomain interacting protein kinase 2	Homo sapiens
653052	homeodomain interacting protein kinase 2; similar to homeodomain interacting protein kinase 2	Homo sapiens
3838	karyopherin alpha 2 (RAG cohort 1, importin alpha 1); karyopherin alpha-2 subunit like	Homo sapiens
728860	karyopherin alpha 2 (RAG cohort 1, importin alpha 1); karyopherin alpha-2 subunit like	Homo sapiens
10444	zer-1 homolog (C. elegans)	Homo sapiens
54855	family with sequence similarity 46, member C	Homo sapiens
8458	transcription termination factor, RNA polymerase II	Homo sapiens
2353	v-fos FBJ murine osteosarcoma viral oncogene homolog	Homo sapiens
137886	UBX domain protein 2B	Homo sapiens
6405	sema domain, immunoglobulin domain (Ig), short basic domain, secreted, (semaphorin) 3F	Homo sapiens
334	amyloid beta (A4) precursor-like protein 2	Homo sapiens
9529	BCL2-associated athanogene 5	Homo sapiens
23413	frequenin homolog (Drosophila)	Homo sapiens
3987	LIM and senescent cell antigen-like domains 1	Homo sapiens
23491	carboxylesterase 3	Homo sapiens
54407	solute carrier family 38, member 2	Homo sapiens
7423	vascular endothelial growth factor B	Homo sapiens
55677	IWS1 homolog (S. cerevisiae)	Homo sapiens
8834	transmembrane protein 11	Homo sapiens
23130	ATG2 autophagy related 2 homolog A (S. cerevisiae)	Homo sapiens
51729	WW domain binding protein 11	Homo sapiens
5499	protein phosphatase 1, catalytic subunit, alpha isoform	Homo sapiens
5529	protein phosphatase 2, regulatory subunit B', epsilon isoform	Homo sapiens
10682	emopamil binding protein (sterol isomerase)	Homo sapiens
7416	voltage-dependent anion channel 1; similar to voltage-dependent anion channel 1	Homo sapiens
642585	voltage-dependent anion channel 1; similar to voltage-dependent anion channel 1	Homo sapiens
5143	phosphodiesterase 4C, cAMP-specific (phosphodiesterase E1 dunce homolog, Drosophila)	Homo sapiens
8986	ribosomal protein S6 kinase, 90kDa, polypeptide 4	Homo sapiens
8148	TAF15 RNA polymerase II, TATA box binding protein (TBP)-associated factor, 68kDa	Homo sapiens
51068	NMD3 homolog (S. cerevisiae)	Homo sapiens
57018	cyclin L1	Homo sapiens
8878	sequestosome 1	Homo sapiens
85365	asparagine-linked glycosylation 2, alpha-1,3-mannosyltransferase homolog (S. cerevisiae)	Homo sapiens
256987	serine incorporator 5	Homo sapiens
284001	coiled-coil domain containing 57	Homo sapiens
51164	dynactin 4 (p62)	Homo sapiens
81565	nudE nuclear distribution gene E homolog (A. nidulans)-like 1	Homo sapiens
51013	exosome component 1	Homo sapiens
128553	teashirt zinc finger homeobox 2	Homo sapiens
85465	selenoprotein I	Homo sapiens
10480	eukaryotic translation initiation factor 3, subunit M	Homo sapiens
3619	inner centromere protein antigens 135/155kDa	Homo sapiens
60496	aminoadipate-semialdehyde dehydrogenase-phosphopantetheinyl transferase	Homo sapiens
8029	cubilin (intrinsic factor-cobalamin receptor)	Homo sapiens
2648	K(lysine) acetyltransferase 2A	Homo sapiens
79571	GRIP and coiled-coil domain containing 1	Homo sapiens
9221	nucleolar and coiled-body phosphoprotein 1	Homo sapiens
10971	tyrosine 3-monooxygenase/tryptophan 5-monooxygenase activation protein, theta polypeptide	Homo sapiens
1021	cyclin-dependent kinase 6	Homo sapiens
346007	eyes shut homolog (Drosophila)	Homo sapiens
9986	RCE1 homolog, prenyl protein peptidase (S. cerevisiae)	Homo sapiens
23245	astrotactin 2	Homo sapiens
402	ADP-ribosylation factor-like 2	Homo sapiens
6474	short stature homeobox 2	Homo sapiens
400793	chromosome 1 open reading frame 226	Homo sapiens
201626	phosphodiesterase 12	Homo sapiens
9517	serine palmitoyltransferase, long chain base subunit 2	Homo sapiens
538	ATPase, Cu++ transporting, alpha polypeptide	Homo sapiens
2195	FAT tumor suppressor homolog 1 (Drosophila)	Homo sapiens
84733	chromobox homolog 2 (Pc class homolog, Drosophila)	Homo sapiens
151	adrenergic, alpha-2B-, receptor	Homo sapiens
164832	LON peptidase N-terminal domain and ring finger 2	Homo sapiens
23023	transmembrane and coiled-coil domain family 1	Homo sapiens
10675	chondroitin sulfate proteoglycan 5 (neuroglycan C)	Homo sapiens
7753	zinc finger protein 202	Homo sapiens
2100	estrogen receptor 2 (ER beta)	Homo sapiens
2526	fucosyltransferase 4 (alpha (1,3) fucosyltransferase, myeloid-specific)	Homo sapiens
23401	frequently rearranged in advanced T-cell lymphomas 2	Homo sapiens
3185	heterogeneous nuclear ribonucleoprotein F	Homo sapiens
5860	quinoid dihydropteridine reductase	Homo sapiens
2965	general transcription factor IIH, polypeptide 1, 62kDa	Homo sapiens
1729	diaphanous homolog 1 (Drosophila)	Homo sapiens
23114	neurofascin homolog (chicken)	Homo sapiens
140459	ankyrin repeat and SOCS box-containing 6	Homo sapiens
5538	palmitoyl-protein thioesterase 1	Homo sapiens
79673	zinc finger protein 329	Homo sapiens

4646	myosin VI	Homo sapiens
682	basigin (Ok blood group)	Homo sapiens
3691	integrin, beta 4	Homo sapiens
51	acyl-Coenzyme A oxidase 1, palmitoyl	Homo sapiens
166968	mesoderm induction early response 1, family member 3	Homo sapiens
2104	estrogen-related receptor gamma	Homo sapiens
84163	GTF2I repeat domain containing 2	Homo sapiens
9440	mediator complex subunit 17	Homo sapiens
23354	HAUS augmin-like complex, subunit 5	Homo sapiens
9618	TNF receptor-associated factor 4	Homo sapiens
4354	membrane protein, palmitoylated 1, 55kDa	Homo sapiens
121296	transmembrane protein 132B; hypothetical LOC121296	Homo sapiens
114795	transmembrane protein 132B; hypothetical LOC121296	Homo sapiens
55508	solute carrier family 35, member E3	Homo sapiens
26249	kelch-like 3 (Drosophila)	Homo sapiens
54949	chromosome 11 open reading frame 79	Homo sapiens
1594	cytochrome P450, family 27, subfamily B, polypeptide 1	Homo sapiens
8935	src kinase associated phosphoprotein 2	Homo sapiens
11122	protein tyrosine phosphatase, receptor type, T	Homo sapiens
55847	CDGSH iron sulfur domain 1	Homo sapiens
5445	paraoxonase 2	Homo sapiens
81847	ring finger protein 146	Homo sapiens
729708	TPI1 pseudogene; triosephosphate isomerase 1	Homo sapiens
7167	TPI1 pseudogene; triosephosphate isomerase 1	Homo sapiens
85461	tetratricopeptide repeat, ankyrin repeat and coiled-coil containing 1	Homo sapiens
51629	solute carrier family 25, member 39	Homo sapiens
84924	zinc finger protein 566	Homo sapiens
7286	tuftelin 1	Homo sapiens
137075	claudin 23	Homo sapiens
54845	epithelial splicing regulatory protein 1	Homo sapiens
2768	guanine nucleotide binding protein (G protein) alpha 12	Homo sapiens
22978	5'-nucleotidase, cytosolic II	Homo sapiens
2166	fatty acid amide hydrolase	Homo sapiens
127933	U2AF homology motif (UHM) kinase 1	Homo sapiens
523	ATPase, H+ transporting, lysosomal 70kDa, V1 subunit A	Homo sapiens
8723	sorting nexin 4	Homo sapiens
5792	protein tyrosine phosphatase, receptor type, F	Homo sapiens
63924	cell death-inducing DFFA-like effector c	Homo sapiens
3714	jagged 2	Homo sapiens
51116	mitochondrial ribosomal protein S2	Homo sapiens
4811	nidogen 1	Homo sapiens
54834	ganglioside induced differentiation associated protein 2	Homo sapiens
85464	slingshot homolog 2 (Drosophila)	Homo sapiens
11149	blood vessel epicardial substance	Homo sapiens
1207	chloride channel, nucleotide-sensitive, 1A	Homo sapiens
9768	KIAA0101	Homo sapiens
80012	polyhomeotic homolog 3 (Drosophila)	Homo sapiens
84250	ankyrin repeat domain 32	Homo sapiens
83660	talin 2	Homo sapiens
5037	phosphatidylethanolamine binding protein 1	Homo sapiens
153830	ring finger protein 145	Homo sapiens
56052	asparagine-linked glycosylation 1, beta-1,4-mannosyltransferase homolog (S. cerevisiae)	Homo sapiens
27018	nerve growth factor receptor (TNFRSF16) associated protein 1	Homo sapiens
115019	solute carrier family 26, member 9	Homo sapiens
8824	carboxylesterase 2 (intestine, liver)	Homo sapiens
4542	myosin IF	Homo sapiens
55089	solute carrier family 38, member 4	Homo sapiens
790955	chromosome 11 open reading frame 83	Homo sapiens
342909	zinc finger protein 284	Homo sapiens
27242	tumor necrosis factor receptor superfamily, member 21	Homo sapiens
54765	tripartite motif-containing 44	Homo sapiens
115509	zinc finger protein 689	Homo sapiens
23294	ankyrin repeat and sterile alpha motif domain containing 1A	Homo sapiens
10491	cartilage associated protein	Homo sapiens
285704	RGM domain family, member B	Homo sapiens
56888	potassium channel modulatory factor 1	Homo sapiens
774	calcium channel, voltage-dependent, N type, alpha 1B subunit	Homo sapiens
6497	v-ski sarcoma viral oncogene homolog (avian)	Homo sapiens
51605	tRNA methyltransferase 6 homolog (S. cerevisiae)	Homo sapiens
55765	chromosome 1 open reading frame 106	Homo sapiens
7690	zinc finger protein 131	Homo sapiens
10419	protein arginine methyltransferase 5	Homo sapiens
4261	class II, major histocompatibility complex, transactivator	Homo sapiens
3911	laminin, alpha 5	Homo sapiens
4170	myeloid cell leukemia sequence 1 (BCL2-related)	Homo sapiens
9655	suppressor of cytokine signaling 5	Homo sapiens
91147	transmembrane protein 67	Homo sapiens
1639	dynactin 1 (p150, glued homolog, Drosophila)	Homo sapiens
533	ATPase, H+ transporting, lysosomal 21kDa, V0 subunit b	Homo sapiens

5355	proteolipid protein 2 (colonic epithelium-enriched)	Homo sapiens
554250	growth differentiation factor 5 opposite strand	Homo sapiens
66036	myotubularin related protein 9	Homo sapiens
2033	E1A binding protein p300	Homo sapiens
78992	Yip1 domain family, member 2	Homo sapiens
345274	solute carrier family 10 (sodium/bile acid cotransporter family), member 6	Homo sapiens
10284	Sin3A-associated protein, 18kDa	Homo sapiens
117608	zinc finger protein 354B	Homo sapiens
489	ATPase, Ca++ transporting, ubiquitous	Homo sapiens
6787	NIMA (never in mitosis gene a)-related kinase 4	Homo sapiens
23059	clusterin associated protein 1	Homo sapiens
10640	exocyst complex component 5	Homo sapiens
131405	tripartite motif-containing 71	Homo sapiens
3177	solute carrier family 29 (nucleoside transporters), member 2	Homo sapiens
11027	leukocyte immunoglobulin-like receptor, subfamily A (with TM domain), member 2	Homo sapiens
10946	splicing factor 3a, subunit 3, 60kDa	Homo sapiens
23152	capicua homolog (Drosophila)	Homo sapiens
80139	zinc finger protein 703	Homo sapiens
152485	zinc finger protein 827	Homo sapiens
10514	MYB binding protein (P160) 1a	Homo sapiens
91875	tetratricopeptide repeat domain 5	Homo sapiens
284390	zinc finger protein 763	Homo sapiens
2639	glutaryl-Coenzyme A dehydrogenase	Homo sapiens
10295	branched chain ketoacid dehydrogenase kinase	Homo sapiens
25805	hypothetical LOC729590; BMP and activin membrane-bound inhibitor homolog (Xenopus laevis)	Homo sapiens
729590	hypothetical LOC729590; BMP and activin membrane-bound inhibitor homolog (Xenopus laevis)	Homo sapiens
92935	methionyl-tRNA synthetase 2, mitochondrial	Homo sapiens
79646	pantothenate kinase 3	Homo sapiens
284685	similar to Ewing sarcoma breakpoint region 1; Ewing sarcoma breakpoint region 1	Homo sapiens
2130	similar to Ewing sarcoma breakpoint region 1; Ewing sarcoma breakpoint region 1	Homo sapiens
2800	golgi autoantigen, golgin subfamily a, 1	Homo sapiens
167227	DCP2 decapping enzyme homolog (S. cerevisiae)	Homo sapiens
10385	butyrophilin, subfamily 2, member A2	Homo sapiens
55082	arginine and glutamate rich 1	Homo sapiens
29842	transcription factor CP2-like 1	Homo sapiens
2635	guanylate binding protein 3	Homo sapiens
285598	ADP-ribosylation factor-like 10	Homo sapiens
5822	PWP2 periodic tryptophan protein homolog (yeast)	Homo sapiens
10949	heterogeneous nuclear ribonucleoprotein A0	Homo sapiens
4753	NEL-like 2 (chicken)	Homo sapiens
1069	centrin, EF-hand protein, 2	Homo sapiens
270	adenosine monophosphate deaminase 1 (isoform M)	Homo sapiens
100128526	hypothetical LOC100128526; target of myb1 (chicken)	Homo sapiens
10043	hypothetical LOC100128526; target of myb1 (chicken)	Homo sapiens
9709	homocysteine-inducible, endoplasmic reticulum stress-inducible, ubiquitin-like domain member 1	Homo sapiens
90204	zinc finger, SWIM-type containing 1	Homo sapiens
201191	sterile alpha motif domain containing 14	Homo sapiens
81610	family with sequence similarity 83, member D	Homo sapiens
2553	GA binding protein transcription factor, beta subunit 1	Homo sapiens
345778	metaxin 3	Homo sapiens
94032	calcium/calmodulin-dependent protein kinase II inhibitor 2	Homo sapiens
90407	transmembrane protein 41A	Homo sapiens
25959	KN motif and ankyrin repeat domains 2	Homo sapiens
346157	zinc finger protein 391	Homo sapiens
51148	cerebral endothelial cell adhesion molecule	Homo sapiens
8661	eukaryotic translation initiation factor 3, subunit A	Homo sapiens
529	ATPase, H+ transporting, lysosomal 31kDa, V1 subunit E1	Homo sapiens
5768	quiescin Q6 sulfhydryl oxidase 1	Homo sapiens
7403	lysine (K)-specific demethylase 6A	Homo sapiens
23200	ATPase, class VI, type 11B	Homo sapiens
55145	THAP domain containing, apoptosis associated protein 1	Homo sapiens
2043	EPH receptor A4	Homo sapiens
4664	NGFI-A binding protein 1 (EGR1 binding protein 1)	Homo sapiens
3419	isocitrate dehydrogenase 3 (NAD+) alpha	Homo sapiens
283373	ankyrin repeat domain 52	Homo sapiens
84301	DDI1, DNA-damage inducible 1, homolog 2 (S. cerevisiae)	Homo sapiens
206358	solute carrier family 36 (proton/amino acid symporter), member 1	Homo sapiens
27254	cold shock domain containing C2, RNA binding	Homo sapiens
26227	phosphoglycerate dehydrogenase	Homo sapiens
5036	proliferation-associated 2G4, 38kDa; proliferation-associated 2G4 pseudogene 4	Homo sapiens
647033	proliferation-associated 2G4, 38kDa; proliferation-associated 2G4 pseudogene 4	Homo sapiens
1555	cytochrome P450, family 2, subfamily B, polypeptide 6	Homo sapiens
7259	TSPY-like 1	Homo sapiens
51058	zinc finger protein 691	Homo sapiens
6720	sterol regulatory element binding transcription factor 1	Homo sapiens
80003	pecanex-like 2 (Drosophila)	Homo sapiens

6668	Sp2 transcription factor	Homo sapiens
285237	chromosome 3 open reading frame 38	Homo sapiens
10728	prostaglandin E synthase 3 (cytosolic)	Homo sapiens
9637	fasciculation and elongation protein zeta 2 (zygin II)	Homo sapiens
151449	growth differentiation factor 7	Homo sapiens
7045	transforming growth factor, beta-induced, 68kDa	Homo sapiens
220992	zinc finger protein 485	Homo sapiens
9894	TEL2, telomere maintenance 2, homolog (S. cerevisiae)	Homo sapiens
157927	chromosome 9 open reading frame 62	Homo sapiens
84276	nicolin 1	Homo sapiens
257194	neuronal growth regulator 1	Homo sapiens
84280	BTB (POZ) domain containing 10	Homo sapiens
23118	mitogen-activated protein kinase kinase kinase 7 interacting protein 2	Homo sapiens
57465	TBC1 domain family, member 24	Homo sapiens
6301	seryl-tRNA synthetase	Homo sapiens
124491	transmembrane protein 170A	Homo sapiens
22794	cancer susceptibility candidate 3	Homo sapiens
3400	inhibitor of DNA binding 4, dominant negative helix-loop-helix protein	Homo sapiens
92591	ankyrin repeat and SOCS box-containing 16	Homo sapiens
959	CD40 ligand	Homo sapiens
2617	glycyl-tRNA synthetase	Homo sapiens
29993	protein kinase C and casein kinase substrate in neurons 1	Homo sapiens
56929	fem-1 homolog c (C. elegans)	Homo sapiens
730031	similar to Afadin (Protein AF-6); myeloid/lymphoid or mixed-lineage leukemia (trithorax homolog, Drosophila); translocated to, 4	Homo sapiens
4301	similar to Afadin (Protein AF-6); myeloid/lymphoid or mixed-lineage leukemia (trithorax homolog, Drosophila); translocated to, 4	Homo sapiens
6164	ribosomal protein L34	Homo sapiens
51490	chromosome 9 open reading frame 114	Homo sapiens
4436	mutS homolog 2, colon cancer, nonpolyposis type 1 (E. coli)	Homo sapiens
134728	interleukin-1 receptor-associated kinase 1 binding protein 1	Homo sapiens
6666	SRY (sex determining region Y)-box 12	Homo sapiens
23155	chloride channel CLIC-like 1	Homo sapiens
2316	filamin A, alpha (actin binding protein 280)	Homo sapiens
1983	eukaryotic translation initiation factor 5	Homo sapiens
3796	kinesin heavy chain member 2A	Homo sapiens
389799	chromosome 9 open reading frame 171	Homo sapiens
54940	OCIA domain containing 1	Homo sapiens
64386	matrix metallopeptidase 25	Homo sapiens
79443	FYVE and coiled-coil domain containing 1	Homo sapiens
64393	zinc finger, matrin type 3	Homo sapiens
462	serpin peptidase inhibitor, clade C (antithrombin), member 1	Homo sapiens
84272	Yip1 domain family, member 4	Homo sapiens
27086	forkhead box P1	Homo sapiens
55095	sterile alpha motif domain containing 4B	Homo sapiens
139886	spindlin family, member 4	Homo sapiens
154796	angiomotin	Homo sapiens
26018	leucine-rich repeats and immunoglobulin-like domains 1	Homo sapiens
4641	myosin IC	Homo sapiens
55066	pyruvate dehydrogenase phosphatase regulatory subunit	Homo sapiens
2894	glutamate receptor, ionotropic, delta 1	Homo sapiens
81493	syncoilin, intermediate filament protein	Homo sapiens
7291	twist homolog 1 (Drosophila)	Homo sapiens
1847	dual specificity phosphatase 5	Homo sapiens
80254	centrosomal protein 63kDa	Homo sapiens
55870	ash1 (absent, small, or homeotic)-like (Drosophila)	Homo sapiens
7763	similar to zinc finger, AN1-type domain 5; zinc finger, AN1-type domain 5	Homo sapiens
732229	similar to zinc finger, AN1-type domain 5; zinc finger, AN1-type domain 5	Homo sapiens
79660	protein phosphatase 1, regulatory (inhibitor) subunit 3B	Homo sapiens
147495	adenomatosis polyposis coli down-regulated 1	Homo sapiens
285172	family with sequence similarity 126, member B	Homo sapiens
830	capping protein (actin filament) muscle Z-line, alpha 2	Homo sapiens
4084	MAX dimerization protein 1	Homo sapiens
26521	similar to translocase of inner mitochondrial membrane 8 homolog B; translocase of inner mitochondrial membrane 8 homolog B (yeast)	Homo sapiens
100131128	similar to translocase of inner mitochondrial membrane 8 homolog B; translocase of inner mitochondrial membrane 8 homolog B (yeast)	Homo sapiens
647302	LSM3 homolog, U6 small nuclear RNA associated (S. cerevisiae); similar to Lsm3 protein	Homo sapiens
27258	LSM3 homolog, U6 small nuclear RNA associated (S. cerevisiae); similar to Lsm3 protein	Homo sapiens
121214	short chain dehydrogenase/reductase family 9C, member 7	Homo sapiens
138009	WD repeat domain 21C	Homo sapiens
7275	tubby homolog (mouse)	Homo sapiens
10622	polymerase (RNA) III (DNA directed) polypeptide G (32kD)	Homo sapiens
5906	RAP1A, member of RAS oncogene family	Homo sapiens
51167	cytochrome b5 reductase 4	Homo sapiens
92181	ubiquitin domain containing 2	Homo sapiens
1362	carboxypeptidase D	Homo sapiens
64111	neuropeptide VF precursor	Homo sapiens
153642	arylsulfatase family, member K	Homo sapiens

54495	thioredoxin-related transmembrane protein 3	Homo sapiens
23583	single-strand-selective monofunctional uracil-DNA glycosylase 1	Homo sapiens
10152	abl interactor 2	Homo sapiens
29071	C1GALT1-specific chaperone 1	Homo sapiens
23516	solute carrier family 39 (zinc transporter), member 14	Homo sapiens
23397	non-SMC condensin I complex, subunit H	Homo sapiens
7707	zinc finger protein 148	Homo sapiens
9712	USP6 N-terminal like	Homo sapiens
51780	lysine (K)-specific demethylase 3B	Homo sapiens
4613	v-myc myelocytomatosis viral related oncogene, neuroblastoma derived (avian)	Homo sapiens
92912	ubiquitin-conjugating enzyme E2Q family member 2	Homo sapiens
2098	esterase D/formylglutathione hydrolase	Homo sapiens
115196	zinc finger protein 554	Homo sapiens
5307	paired-like homeodomain 1	Homo sapiens
8669	eukaryotic translation initiation factor 3, subunit J	Homo sapiens
23042	pyridoxal-dependent decarboxylase domain containing 1	Homo sapiens
10960	lectin, mannose-binding 2	Homo sapiens
8566	pyridoxal (pyridoxine, vitamin B6) kinase	Homo sapiens
57504	metastasis associated 1 family, member 3	Homo sapiens
6202	ribosomal protein S8; ribosomal protein S8 pseudogene 8; ribosomal protein S8 pseudogene 10	Homo sapiens
728553	ribosomal protein S8; ribosomal protein S8 pseudogene 8; ribosomal protein S8 pseudogene 10	Homo sapiens
388076	ribosomal protein S8; ribosomal protein S8 pseudogene 8; ribosomal protein S8 pseudogene 10	Homo sapiens
3166	H6 family homeobox 1	Homo sapiens
7552	zinc finger protein 711	Homo sapiens
114327	EF-hand domain (C-terminal) containing 1	Homo sapiens
2896	granulin	Homo sapiens
83640	family with sequence similarity 103, member A1	Homo sapiens
10513	amyloid beta precursor protein (cytoplasmic tail) binding protein 2	Homo sapiens
57492	AT rich interactive domain 1B (SWI1-like)	Homo sapiens
54107	polymerase (DNA directed), epsilon 3 (p17 subunit)	Homo sapiens
441478	NOTCH-regulated ankyrin repeat protein	Homo sapiens
6839	suppressor of variegation 3-9 homolog 1 (Drosophila)	Homo sapiens
56648	eukaryotic translation initiation factor 5A2	Homo sapiens
23332	cytoplasmic linker associated protein 1	Homo sapiens
27304	molybdenum cofactor synthesis 3	Homo sapiens
29994	bromodomain adjacent to zinc finger domain, 2B	Homo sapiens
126282	tumor necrosis factor, alpha-induced protein 8-like 1	Homo sapiens
284353	NTPase, KAP family P-loop domain containing 1	Homo sapiens
5983	replication factor C (activator 1) 3, 38kDa	Homo sapiens
5094	poly(rC) binding protein 2	Homo sapiens
283337	zinc finger protein 740	Homo sapiens
222235	F-box and leucine-rich repeat protein 13	Homo sapiens
4288	antigen identified by monoclonal antibody Ki-67	Homo sapiens
403341	zinc finger and BTB domain containing 34	Homo sapiens
4234	methyltransferase like 1	Homo sapiens
6146	ribosomal protein L22 pseudogene 11; ribosomal protein L22	Homo sapiens
402100	ribosomal protein L22 pseudogene 11; ribosomal protein L22	Homo sapiens
338707	beta-1,4-N-acetyl-galactosaminyl transferase 4	Homo sapiens
23217	zinc finger RNA binding protein 2	Homo sapiens
83607	AMME chromosomal region gene 1-like	Homo sapiens
7159	tumor protein p53 binding protein, 2	Homo sapiens
10226	mannose-6-phosphate receptor binding protein 1	Homo sapiens
23160	WD repeat domain 43	Homo sapiens
9154	solute carrier family 28 (sodium-coupled nucleoside transporter), member 1	Homo sapiens
57476	GRAM domain containing 1B	Homo sapiens
84296	GINS complex subunit 4 (Sld5 homolog)	Homo sapiens
23108	GTPase activating Rap/RanGAP domain-like 4	Homo sapiens
85319	B melanoma antigen family, member 2; B melanoma antigen family, member 3; B melanoma antigen family, member 4; B melanoma antigen family, member 5	Homo sapiens
85316	B melanoma antigen family, member 2; B melanoma antigen family, member 3; B melanoma antigen family, member 4; B melanoma antigen family, member 5	Homo sapiens
85318	B melanoma antigen family, member 2; B melanoma antigen family, member 3; B melanoma antigen family, member 4; B melanoma antigen family, member 5	Homo sapiens
85317	B melanoma antigen family, member 2; B melanoma antigen family, member 3; B melanoma antigen family, member 4; B melanoma antigen family, member 5	Homo sapiens
29946	SERTA domain containing 3	Homo sapiens
4990	SIX homeobox 6	Homo sapiens
6671	Sp4 transcription factor	Homo sapiens
118813	zinc finger, FYVE domain containing 27	Homo sapiens
5565	protein kinase, AMP-activated, beta 2 non-catalytic subunit	Homo sapiens
22	ATP-binding cassette, sub-family B (MDR/TAP), member 7	Homo sapiens
55003	PAK1 interacting protein 1	Homo sapiens
89780	wingless-type MMTV integration site family, member 3A	Homo sapiens
10311	Down syndrome critical region gene 3	Homo sapiens

441032	eukaryotic translation elongation factor 1 alpha-like 7; eukaryotic translation elongation factor 1 alpha-like 3; similar to eukaryotic translation elongation factor 1 alpha 1; eukaryotic translation elongation factor 1 alpha 1	Homo sapiens
158078	eukaryotic translation elongation factor 1 alpha-like 7; eukaryotic translation elongation factor 1 alpha-like 3; similar to eukaryotic translation elongation factor 1 alpha 1; eukaryotic translation elongation factor 1 alpha 1	Homo sapiens
645715	eukaryotic translation elongation factor 1 alpha-like 7; eukaryotic translation elongation factor 1 alpha-like 3; similar to eukaryotic translation elongation factor 1 alpha 1; eukaryotic translation elongation factor 1 alpha 1	Homo sapiens
1915	eukaryotic translation elongation factor 1 alpha-like 7; eukaryotic translation elongation factor 1 alpha-like 3; similar to eukaryotic translation elongation factor 1 alpha 1; eukaryotic translation elongation factor 1 alpha 1	Homo sapiens
5775	protein tyrosine phosphatase, non-receptor type 4 (megakaryocyte)	Homo sapiens
121536	AE binding protein 2	Homo sapiens
22859	latrophilin 1	Homo sapiens
80045	G protein-coupled receptor 157	Homo sapiens
51259	transmembrane protein 216	Homo sapiens
219927	mitochondrial ribosomal protein L21	Homo sapiens
51550	cyclin-dependent kinase 2-interacting protein	Homo sapiens
145282	mirror-image polydactyly 1	Homo sapiens
8446	dual specificity phosphatase 11 (RNA/RNP complex 1-interacting)	Homo sapiens
3151	hypothetical LOC729505; similar to hCG2040565; high-mobility group nucleosomal binding domain 2; similar to high-mobility group nucleosomal binding domain 2	Homo sapiens
648822	hypothetical LOC729505; similar to hCG2040565; high-mobility group nucleosomal binding domain 2; similar to high-mobility group nucleosomal binding domain 2	Homo sapiens
729505	hypothetical LOC729505; similar to hCG2040565; high-mobility group nucleosomal binding domain 2; similar to high-mobility group nucleosomal binding domain 2	Homo sapiens
729687	hypothetical LOC729505; similar to hCG2040565; high-mobility group nucleosomal binding domain 2; similar to high-mobility group nucleosomal binding domain 2	Homo sapiens
4536	NADH-ubiquinone oxidoreductase chain 2	Homo sapiens
6775094	NADH-ubiquinone oxidoreductase chain 2	Homo sapiens
79053	asparagine-linked glycosylation 8, alpha-1,3-glucosyltransferase homolog (S. cerevisiae)	Homo sapiens
23355	vacuolar protein sorting 8 homolog (S. cerevisiae)	Homo sapiens
4053	latent transforming growth factor beta binding protein 2	Homo sapiens
9513	fragile X mental retardation, autosomal homolog 2	Homo sapiens
7090	transducin-like enhancer of split 3 (E(sp1) homolog, Drosophila)	Homo sapiens
2182	acyl-CoA synthetase long-chain family member 4	Homo sapiens
10181	RNA binding motif protein 5	Homo sapiens
147463	ankyrin repeat domain 29	Homo sapiens
8451	cullin 4A	Homo sapiens
100130746	AKT interacting protein; similar to AKT interacting protein	Homo sapiens
64400	AKT interacting protein; similar to AKT interacting protein	Homo sapiens
131076	coiled-coil domain containing 58	Homo sapiens
653639	lysophospholipase II pseudogene 1; lysophospholipase II	Homo sapiens
11313	lysophospholipase II pseudogene 1; lysophospholipase II	Homo sapiens
339263	chromosome 17 open reading frame 51	Homo sapiens
8717	TNFRSF1A-associated via death domain	Homo sapiens
55568	UDP-N-acetyl-alpha-D-galactosamine:polypeptide N-acetylgalactosaminyltransferase 10 (GalNAc-T10)	Homo sapiens
146923	RUN domain containing 1	Homo sapiens
140803	transient receptor potential cation channel, subfamily M, member 6	Homo sapiens
10762	nucleoporin 50kDa	Homo sapiens
51428	DEAD (Asp-Glu-Ala-Asp) box polypeptide 41	Homo sapiens
71	actin, gamma 1	Homo sapiens
134637	adenosine deaminase, tRNA-specific 2, TAD2 homolog (S. cerevisiae)	Homo sapiens
7164	tumor protein D52-like 1	Homo sapiens
5738	prostaglandin F2 receptor negative regulator	Homo sapiens
9604	ring finger protein 14	Homo sapiens
5817	poliovirus receptor	Homo sapiens
1528	cytochrome b5 type A (microsomal)	Homo sapiens
22907	DEAH (Asp-Glu-Ala-His) box polypeptide 30	Homo sapiens
28972	signal peptidase complex subunit 1 homolog (S. cerevisiae)	Homo sapiens
3779	potassium large conductance calcium-activated channel, subfamily M, beta member 1	Homo sapiens
525	ATPase, H+ transporting, lysosomal 56/58kDa, V1 subunit B1	Homo sapiens
11124	Fas (TNFRSF6) associated factor 1	Homo sapiens
2181	acyl-CoA synthetase long-chain family member 3	Homo sapiens
1408	cryptochrome 2 (photolyase-like)	Homo sapiens
645453	ring finger protein, LIM domain interacting; similar to ring finger protein (C3H2C3 type) 6	Homo sapiens
51132	ring finger protein, LIM domain interacting; similar to ring finger protein (C3H2C3 type) 6	Homo sapiens
54429	taste receptor, type 2, member 5	Homo sapiens
23640	HSPA (heat shock 70kDa) binding protein, cytoplasmic cochaperone 1	Homo sapiens
51284	toll-like receptor 7	Homo sapiens
90196	SYS1 Golgi-localized integral membrane protein homolog (S. cerevisiae)	Homo sapiens
10787	NCK-associated protein 1	Homo sapiens
25873	ribosomal protein L36; ribosomal protein L36 pseudogene 14	Homo sapiens
347292	ribosomal protein L36; ribosomal protein L36 pseudogene 14	Homo sapiens
51227	phosphatidylinositol glycan anchor biosynthesis, class P	Homo sapiens

1454	casein kinase 1, epsilon	Homo sapiens
23203	peptidase (mitochondrial processing) alpha	Homo sapiens
80025	pantothenate kinase 2	Homo sapiens
286319	tumor suppressor candidate 1	Homo sapiens
7003	TEA domain family member 1 (SV40 transcriptional enhancer factor)	Homo sapiens
26995	TruB pseudouridine (psi) synthase homolog 2 (E. coli)	Homo sapiens
27327	trinucleotide repeat containing 6A	Homo sapiens
285016	family with sequence similarity 150, member B	Homo sapiens
960	CD44 molecule (Indian blood group)	Homo sapiens
222658	potassium channel tetramerisation domain containing 20	Homo sapiens
55300	phosphatidylinositol 4-kinase type 2 beta	Homo sapiens
5583	protein kinase C, eta	Homo sapiens
100131387	ribosomal protein L39 pseudogene 10; ribosomal protein L39 pseudogene 20; ribosomal protein L39 pseudogene 27; ribosomal protein L39; ribosomal protein L39 pseudogene 13; ribosomal protein L39 pseudogene 32	Homo sapiens
100130802	ribosomal protein L39 pseudogene 10; ribosomal protein L39 pseudogene 20; ribosomal protein L39 pseudogene 27; ribosomal protein L39; ribosomal protein L39 pseudogene 13; ribosomal protein L39 pseudogene 32	Homo sapiens
100133222	ribosomal protein L39 pseudogene 10; ribosomal protein L39 pseudogene 20; ribosomal protein L39 pseudogene 27; ribosomal protein L39; ribosomal protein L39 pseudogene 13; ribosomal protein L39 pseudogene 32	Homo sapiens
100131160	ribosomal protein L39 pseudogene 10; ribosomal protein L39 pseudogene 20; ribosomal protein L39 pseudogene 27; ribosomal protein L39; ribosomal protein L39 pseudogene 13; ribosomal protein L39 pseudogene 32	Homo sapiens
6170	ribosomal protein L39 pseudogene 10; ribosomal protein L39 pseudogene 20; ribosomal protein L39 pseudogene 27; ribosomal protein L39; ribosomal protein L39 pseudogene 13; ribosomal protein L39 pseudogene 32	Homo sapiens
100129952	ribosomal protein L39 pseudogene 10; ribosomal protein L39 pseudogene 20; ribosomal protein L39 pseudogene 27; ribosomal protein L39; ribosomal protein L39 pseudogene 13; ribosomal protein L39 pseudogene 32	Homo sapiens
54760	proprotein convertase subtilisin/kexin type 4	Homo sapiens
51531	chromosome 9 open reading frame 156	Homo sapiens
9898	ubiquitin associated protein 2-like	Homo sapiens
23105	follistatin-like 4	Homo sapiens
23392	KIAA0368	Homo sapiens
23503	zinc finger, FYVE domain containing 26	Homo sapiens
5425	polymerase (DNA directed), delta 2, regulatory subunit 50kDa	Homo sapiens
1192	chloride intracellular channel 1	Homo sapiens
55573	CDV3 homolog (mouse)	Homo sapiens
11196	SEC23 interacting protein	Homo sapiens
89970	ring finger and SPRY domain containing 1	Homo sapiens
89122	tripartite motif-containing 4	Homo sapiens
79642	arylsulfatase family, member J	Homo sapiens
91768	Cdk5 and Abl enzyme substrate 1	Homo sapiens
6908	TATA box binding protein	Homo sapiens
89790	sialic acid binding Ig-like lectin 10	Homo sapiens
114821	SCAN domain containing 3	Homo sapiens
166336	prickle homolog 2 (Drosophila)	Homo sapiens
5829	paxillin	Homo sapiens
9020	mitogen-activated protein kinase kinase kinase 14	Homo sapiens
56256	SERTA domain containing 4	Homo sapiens
5420	podocalyxin-like	Homo sapiens
54584	guanine nucleotide binding protein (G protein), beta polypeptide 1-like	Homo sapiens
130132	raftlin family member 2	Homo sapiens
84331	chromosome 16 open reading frame 14	Homo sapiens
8439	neutral sphingomyelinase (N-SMase) activation associated factor	Homo sapiens
147949	zinc finger protein 583	Homo sapiens
51557	lengsin, lens protein with glutamine synthetase domain	Homo sapiens
10939	AFG3 ATPase family gene 3-like 2 (yeast)	Homo sapiens
90249	unc-5 homolog A (C. elegans)	Homo sapiens
673	v-raf murine sarcoma viral oncogene homolog B1	Homo sapiens
51378	angiopoietin 4	Homo sapiens
6305	SET binding factor 1; SET binding factor 1 pseudogene 1	Homo sapiens
100133234	SET binding factor 1; SET binding factor 1 pseudogene 1	Homo sapiens
3710	inositol 1,4,5-triphosphate receptor, type 3	Homo sapiens
51397	COMM domain containing 10	Homo sapiens
728453	ribosomal protein S28 pseudogene 6; ribosomal protein S28 pseudogene 9; ribosomal protein S28	Homo sapiens
650788	ribosomal protein S28 pseudogene 6; ribosomal protein S28 pseudogene 9; ribosomal protein S28	Homo sapiens
6234	ribosomal protein S28 pseudogene 6; ribosomal protein S28 pseudogene 9; ribosomal protein S28	Homo sapiens
10775	processing of precursor 4, ribonuclease P/MRP subunit (S. cerevisiae)	Homo sapiens
4204	methyl CpG binding protein 2 (Rett syndrome)	Homo sapiens
6773	signal transducer and activator of transcription 2, 113kDa	Homo sapiens
6751	somatostatin receptor 1	Homo sapiens
65123	integrator complex subunit 3	Homo sapiens
154043	membrane associated guanylate kinase, WW and PDZ domain containing 1; CNKSR family member 3	Homo sapiens

9223	membrane associated guanylate kinase, WW and PDZ domain containing 1; CNKSR family member 3	Homo sapiens
26528	DAZ associated protein 1	Homo sapiens
128822	cystatin 9 (testatin)	Homo sapiens
617	BCS1-like (yeast)	Homo sapiens
84162	KIAA1109	Homo sapiens
205717	KIAA2018	Homo sapiens
3716	Janus kinase 1	Homo sapiens
10395	deleted in liver cancer 1	Homo sapiens
4293	mitogen-activated protein kinase kinase kinase 9	Homo sapiens
284119	polymerase I and transcript release factor	Homo sapiens
126393	heat shock protein, alpha-crystallin-related, B6	Homo sapiens
6293	vacuolar protein sorting 52 homolog (S. cerevisiae)	Homo sapiens
23515	MORC family CW-type zinc finger 3	Homo sapiens
8639	amine oxidase, copper containing 3 (vascular adhesion protein 1)	Homo sapiens
23097	cell division cycle 2-like 6 (CDK8-like)	Homo sapiens
257068	pleckstrin homology-like domain, family B, member 2; phosphatidylinositol-specific phospholipase C, X domain containing 2	Homo sapiens
90102	pleckstrin homology-like domain, family B, member 2; phosphatidylinositol-specific phospholipase C, X domain containing 2	Homo sapiens
25929	gem (nuclear organelle) associated protein 5	Homo sapiens
3065	histone deacetylase 1	Homo sapiens
202243	coiled-coil domain containing 125	Homo sapiens
392862	glutamate receptor, ionotropic, delta 2 (Grid2) interacting protein	Homo sapiens
988	CDC5 cell division cycle 5-like (S. pombe)	Homo sapiens
399665	family with sequence similarity 102, member A	Homo sapiens
65220	NAD kinase	Homo sapiens
440311	glioma tumor suppressor candidate region gene 2; glioma tumor suppressor candidate region gene 2 pseudogene	Homo sapiens
29997	glioma tumor suppressor candidate region gene 2; glioma tumor suppressor candidate region gene 2 pseudogene	Homo sapiens
8208	chromatin assembly factor 1, subunit B (p60)	Homo sapiens
4302	myeloid/lymphoid or mixed-lineage leukemia (trithorax homolog, Drosophila); translocated to, 6	Homo sapiens
83637	zinc finger, MIZ-type containing 2	Homo sapiens
90233	zinc finger protein 551	Homo sapiens
6546	solute carrier family 8 (sodium/calcium exchanger), member 1	Homo sapiens
10797	methylenetetrahydrofolate dehydrogenase (NADP+ dependent) 2, methenyltetrahydrofolate cyclohydrolase	Homo sapiens
1948	ephrin-B2	Homo sapiens
158345	ribosomal protein L4; ribosomal protein L4 pseudogene 5; ribosomal protein L4 pseudogene 4	Homo sapiens
647276	ribosomal protein L4; ribosomal protein L4 pseudogene 5; ribosomal protein L4 pseudogene 4	Homo sapiens
6124	ribosomal protein L4; ribosomal protein L4 pseudogene 5; ribosomal protein L4 pseudogene 4	Homo sapiens
5689	proteasome (prosome, macropain) subunit, beta type, 1	Homo sapiens
9743	Rho GTPase-activating protein	Homo sapiens
3205	homeobox A9	Homo sapiens
23314	SATB homeobox 2	Homo sapiens
54878	dipeptidyl-peptidase 8	Homo sapiens
440	asparagine synthetase	Homo sapiens
340719	nanos homolog 1 (Drosophila)	Homo sapiens
79089	transmembrane and ubiquitin-like domain containing 2	Homo sapiens
328	APEX nuclease (multifunctional DNA repair enzyme) 1	Homo sapiens
8239	ubiquitin specific peptidase 9, X-linked	Homo sapiens
7424	vascular endothelial growth factor C	Homo sapiens
230	aldolase C, fructose-bisphosphate	Homo sapiens
30001	ERO1-like (S. cerevisiae)	Homo sapiens
687	Kruppel-like factor 9	Homo sapiens
79796	asparagine-linked glycosylation 9, alpha-1,2-mannosyltransferase homolog (S. cerevisiae)	Homo sapiens
1327	cytochrome c oxidase subunit IV isoform 1	Homo sapiens
4052	latent transforming growth factor beta binding protein 1	Homo sapiens
92745	solute carrier family 38, member 5	Homo sapiens
4233	met proto-oncogene (hepatocyte growth factor receptor)	Homo sapiens
83882	tetraspanin 10	Homo sapiens
55749	cell division cycle and apoptosis regulator 1	Homo sapiens
441549	cerebral dopamine neurotrophic factor	Homo sapiens
6667	Sp1 transcription factor	Homo sapiens
9252	ribosomal protein S6 kinase, 90kDa, polypeptide 5	Homo sapiens
8565	tyrosyl-tRNA synthetase	Homo sapiens
57459	GATA zinc finger domain containing 2B	Homo sapiens
25926	nucleolar protein 11	Homo sapiens
445347	TCR gamma alternate reading frame protein; T cell receptor gamma variable 9; T cell receptor gamma constant 1	Homo sapiens
6966	TCR gamma alternate reading frame protein; T cell receptor gamma variable 9; T cell receptor gamma constant 1	Homo sapiens
6983	TCR gamma alternate reading frame protein; T cell receptor gamma variable 9; T cell receptor gamma constant 1	Homo sapiens

4221	multiple endocrine neoplasia I	Homo sapiens
84898	plexin domain containing 2	Homo sapiens
57403	RAB22A, member RAS oncogene family	Homo sapiens
389787	similar to tumor protein, translationally-controlled 1; tumor protein, translationally-controlled 1	Homo sapiens
7178	similar to tumor protein, translationally-controlled 1; tumor protein, translationally-controlled 1	Homo sapiens
285741	similar to tumor protein, translationally-controlled 1; tumor protein, translationally-controlled 1	Homo sapiens
150864	family with sequence similarity 117, member B	Homo sapiens
375612	lipoma HMGIC fusion partner-like 3	Homo sapiens
83937	Ras association (RalGDS/AF-6) domain family member 4	Homo sapiens
498	ATP synthase, H+ transporting, mitochondrial F1 complex, alpha subunit 1, cardiac muscle	Homo sapiens
5836	phosphorylase, glycogen, liver	Homo sapiens
23500	dishevelled associated activator of morphogenesis 2	Homo sapiens
54919	HEAT repeat containing 2	Homo sapiens
54790	tet oncogene family member 2	Homo sapiens
8632	dynein, axonemal, heavy chain 17	Homo sapiens
27134	tight junction protein 3 (zona occludens 3)	Homo sapiens
284086	NIMA (never in mitosis gene a)- related kinase 8	Homo sapiens
223	aldehyde dehydrogenase 9 family, member A1	Homo sapiens
132	adenosine kinase	Homo sapiens
51317	PHD finger protein 21A	Homo sapiens
9968	mediator complex subunit 12	Homo sapiens
2779	guanine nucleotide binding protein (G protein), alpha transducing activity polypeptide 1	Homo sapiens
8379	MAD1 mitotic arrest deficient-like 1 (yeast)	Homo sapiens
23468	chromobox homolog 5 (HP1 alpha homolog, Drosophila)	Homo sapiens
246175	CCR4-NOT transcription complex, subunit 6-like	Homo sapiens
124801	LSM12 homolog (S. cerevisiae)	Homo sapiens
858	caveolin 2	Homo sapiens
248	alkaline phosphatase, intestinal	Homo sapiens
115	adenylate cyclase 9	Homo sapiens
333932	histone cluster 1, H3j; histone cluster 1, H3i; histone cluster 1, H3h; histone cluster 1, H3g; histone cluster 1, H3f; histone cluster 1, H3e; histone cluster 1, H3d; histone cluster 1, H3c; histone cluster 1, H3b; histone cluster 1, H3a; histone cluster 1, H2ad; histone cluster 2, H3a; histone cluster 2, H3c; histone cluster 2, H3d	Homo sapiens
8356	histone cluster 1, H3j; histone cluster 1, H3i; histone cluster 1, H3h; histone cluster 1, H3g; histone cluster 1, H3f; histone cluster 1, H3e; histone cluster 1, H3d; histone cluster 1, H3c; histone cluster 1, H3b; histone cluster 1, H3a; histone cluster 1, H2ad; histone cluster 2, H3a; histone cluster 2, H3c; histone cluster 2, H3d	Homo sapiens
8355	histone cluster 1, H3j; histone cluster 1, H3i; histone cluster 1, H3h; histone cluster 1, H3g; histone cluster 1, H3f; histone cluster 1, H3e; histone cluster 1, H3d; histone cluster 1, H3c; histone cluster 1, H3b; histone cluster 1, H3a; histone cluster 1, H2ad; histone cluster 2, H3a; histone cluster 2, H3c; histone cluster 2, H3d	Homo sapiens
8352	histone cluster 1, H3j; histone cluster 1, H3i; histone cluster 1, H3h; histone cluster 1, H3g; histone cluster 1, H3f; histone cluster 1, H3e; histone cluster 1, H3d; histone cluster 1, H3c; histone cluster 1, H3b; histone cluster 1, H3a; histone cluster 1, H2ad; histone cluster 2, H3a; histone cluster 2, H3c; histone cluster 2, H3d	Homo sapiens
8350	histone cluster 1, H3j; histone cluster 1, H3i; histone cluster 1, H3h; histone cluster 1, H3g; histone cluster 1, H3f; histone cluster 1, H3e; histone cluster 1, H3d; histone cluster 1, H3c; histone cluster 1, H3b; histone cluster 1, H3a; histone cluster 1, H2ad; histone cluster 2, H3a; histone cluster 2, H3c; histone cluster 2, H3d	Homo sapiens
653604	histone cluster 1, H3j; histone cluster 1, H3i; histone cluster 1, H3h; histone cluster 1, H3g; histone cluster 1, H3f; histone cluster 1, H3e; histone cluster 1, H3d; histone cluster 1, H3c; histone cluster 1, H3b; histone cluster 1, H3a; histone cluster 1, H2ad; histone cluster 2, H3a; histone cluster 2, H3c; histone cluster 2, H3d	Homo sapiens
8968	histone cluster 1, H3j; histone cluster 1, H3i; histone cluster 1, H3h; histone cluster 1, H3g; histone cluster 1, H3f; histone cluster 1, H3e; histone cluster 1, H3d; histone cluster 1, H3c; histone cluster 1, H3b; histone cluster 1, H3a; histone cluster 1, H2ad; histone cluster 2, H3a; histone cluster 2, H3c; histone cluster 2, H3d	Homo sapiens
8358	histone cluster 1, H3j; histone cluster 1, H3i; histone cluster 1, H3h; histone cluster 1, H3g; histone cluster 1, H3f; histone cluster 1, H3e; histone cluster 1, H3d; histone cluster 1, H3c; histone cluster 1, H3b; histone cluster 1, H3a; histone cluster 1, H2ad; histone cluster 2, H3a; histone cluster 2, H3c; histone cluster 2, H3d	Homo sapiens
126961	histone cluster 1, H3j; histone cluster 1, H3i; histone cluster 1, H3h; histone cluster 1, H3g; histone cluster 1, H3f; histone cluster 1, H3e; histone cluster 1, H3d; histone cluster 1, H3c; histone cluster 1, H3b; histone cluster 1, H3a; histone cluster 1, H2ad; histone cluster 2, H3a; histone cluster 2, H3c; histone cluster 2, H3d	Homo sapiens
8357	histone cluster 1, H3j; histone cluster 1, H3i; histone cluster 1, H3h; histone cluster 1, H3g; histone cluster 1, H3f; histone cluster 1, H3e; histone cluster 1, H3d; histone cluster 1, H3c; histone cluster 1, H3b; histone cluster 1, H3a; histone cluster 1, H2ad; histone cluster 2, H3a; histone cluster 2, H3c; histone cluster 2, H3d	Homo sapiens
8353	histone cluster 1, H3j; histone cluster 1, H3i; histone cluster 1, H3h; histone cluster 1, H3g; histone cluster 1, H3f; histone cluster 1, H3e; histone cluster 1, H3d; histone cluster 1, H3c; histone cluster 1, H3b; histone cluster 1, H3a; histone cluster 1, H2ad; histone cluster 2, H3a; histone cluster 2, H3c; histone cluster 2, H3d	Homo sapiens

3013	histone cluster 1, H3j; histone cluster 1, H3i; histone cluster 1, H3h; histone cluster 1, H3g; histone cluster 1, H3f; histone cluster 1, H3e; histone cluster 1, H3d; histone cluster 1, H3c; histone cluster 1, H3b; histone cluster 1, H3a; histone cluster 1, H2ad; histone cluster 2, H3a; histone cluster 2, H3c; histone cluster 2, H3d	Homo sapiens
8354	histone cluster 1, H3j; histone cluster 1, H3i; histone cluster 1, H3h; histone cluster 1, H3g; histone cluster 1, H3f; histone cluster 1, H3e; histone cluster 1, H3d; histone cluster 1, H3c; histone cluster 1, H3b; histone cluster 1, H3a; histone cluster 1, H2ad; histone cluster 2, H3a; histone cluster 2, H3c; histone cluster 2, H3d	Homo sapiens
8351	histone cluster 1, H3j; histone cluster 1, H3i; histone cluster 1, H3h; histone cluster 1, H3g; histone cluster 1, H3f; histone cluster 1, H3e; histone cluster 1, H3d; histone cluster 1, H3c; histone cluster 1, H3b; histone cluster 1, H3a; histone cluster 1, H2ad; histone cluster 2, H3a; histone cluster 2, H3c; histone cluster 2, H3d	Homo sapiens
56061	ubiquitin family domain containing 1	Homo sapiens
6356	chemokine (C-C motif) ligand 11	Homo sapiens
57661	PHD and ring finger domains 1	Homo sapiens
64747	major facilitator superfamily domain containing 1	Homo sapiens
246721	polymerase (RNA) II (DNA directed) polypeptide J3; polymerase (RNA) II (DNA directed) polypeptide J2	Homo sapiens
548644	polymerase (RNA) II (DNA directed) polypeptide J3; polymerase (RNA) II (DNA directed) polypeptide J2	Homo sapiens
55663	zinc finger protein 446	Homo sapiens
3690	integrin, beta 3 (platelet glycoprotein IIIa, antigen CD61)	Homo sapiens
6496	SIX homeobox 3	Homo sapiens
56133	protocadherin beta 2	Homo sapiens
51258	mitochondrial ribosomal protein L51	Homo sapiens
9782	matrin 3	Homo sapiens
5558	primase, DNA, polypeptide 2 (58kDa)	Homo sapiens
5925	retinoblastoma 1	Homo sapiens
342184	formin 1	Homo sapiens
84934	chromosome 12 open reading frame 52	Homo sapiens
112476	proline-rich transmembrane protein 2	Homo sapiens
2016	empty spiracles homeobox 1	Homo sapiens
6297	sal-like 2 (Drosophila)	Homo sapiens
90379	chromosome 19 open reading frame 72	Homo sapiens
55075	uveal autoantigen with coiled-coil domains and ankyrin repeats	Homo sapiens
1500	catenin (cadherin-associated protein), delta 1	Homo sapiens
29766	tropomodulin 3 (ubiquitous)	Homo sapiens
6780	staufen, RNA binding protein, homolog 1 (Drosophila)	Homo sapiens
342527	smoothelin-like 2	Homo sapiens
6923	transcription elongation factor B (SIII), polypeptide 2 (18kDa, elongin B)	Homo sapiens
7074	T-cell lymphoma invasion and metastasis 1	Homo sapiens
57498	kinase D-interacting substrate, 220kDa	Homo sapiens
79832	glutamine and serine rich 1	Homo sapiens
80028	F-box and leucine-rich repeat protein 18	Homo sapiens
7040	transforming growth factor, beta 1	Homo sapiens
10236	heterogeneous nuclear ribonucleoprotein R	Homo sapiens
11145	phospholipase A2, group XVI	Homo sapiens
1266	calponin 3, acidic	Homo sapiens
79776	zinc finger homeobox 4	Homo sapiens
65975	serine/threonine kinase 33	Homo sapiens
2783	guanine nucleotide binding protein (G protein), beta polypeptide 2	Homo sapiens
134353	LSM11, U7 small nuclear RNA associated	Homo sapiens
2147	coagulation factor II (thrombin)	Homo sapiens
57128	LYR motif containing 4	Homo sapiens
8829	neuropilin 1	Homo sapiens
4660	protein phosphatase 1, regulatory (inhibitor) subunit 12B	Homo sapiens
10966	RAB40B, member RAS oncogene family	Homo sapiens
51043	zinc finger and BTB domain containing 7B	Homo sapiens
51308	receptor accessory protein 2	Homo sapiens
8838	WNT1 inducible signaling pathway protein 3	Homo sapiens
151556	G protein-coupled receptor 155	Homo sapiens
286077	family with sequence similarity 83, member H	Homo sapiens
7464	coronin, actin binding protein, 2A	Homo sapiens
8541	protein tyrosine phosphatase, receptor type, f polypeptide (PTPRF), interacting protein (liprin), alpha 3	Homo sapiens
22908	SAC1 suppressor of actin mutations 1-like (yeast)	Homo sapiens
8394	phosphatidylinositol-4-phosphate 5-kinase, type I, alpha	Homo sapiens
8427	zinc finger protein 282	Homo sapiens
56147	protocadherin alpha 1; protocadherin alpha 4	Homo sapiens
56144	protocadherin alpha 1; protocadherin alpha 4	Homo sapiens
6176	ribosomal protein, large, P1	Homo sapiens
23253	ankyrin repeat domain 12	Homo sapiens
10714	polymerase (DNA-directed), delta 3, accessory subunit	Homo sapiens
9686	vestigial like 4 (Drosophila)	Homo sapiens
5366	phorbol-12-myristate-13-acetate-induced protein 1	Homo sapiens
11267	SNF8, ESCRT-II complex subunit, homolog (S. cerevisiae)	Homo sapiens
9883	POM121 membrane glycoprotein (rat)	Homo sapiens
222537	heparan sulfate (glucosamine) 3-O-sulfotransferase 5	Homo sapiens

388907	ribosomal protein L5 pseudogene 34; ribosomal protein L5 pseudogene 1; ribosomal protein L5	Homo sapiens
6125	ribosomal protein L5 pseudogene 34; ribosomal protein L5 pseudogene 1; ribosomal protein L5	Homo sapiens
647436	ribosomal protein L5 pseudogene 34; ribosomal protein L5 pseudogene 1; ribosomal protein L5	Homo sapiens
2645	glucokinase (hexokinase 4)	Homo sapiens
3675	integrin, alpha 3 (antigen CD49C, alpha 3 subunit of VLA-3 receptor)	Homo sapiens
55311	zinc finger protein 444	Homo sapiens
84328	leucine zipper and CTNNBIP1 domain containing	Homo sapiens
899	cyclin F	Homo sapiens
6950	hypothetical gene supported by BC000665; t-complex 1	Homo sapiens
400013	hypothetical gene supported by BC000665; t-complex 1	Homo sapiens
55526	dehydrogenase E1 and transketolase domain containing 1	Homo sapiens
51719	calcium binding protein 39	Homo sapiens
123041	solute carrier family 24 (sodium/potassium/calcium exchanger), member 4	Homo sapiens
265	amelogenin (amelogenesis imperfecta 1, X-linked)	Homo sapiens
23765	interleukin 17 receptor A	Homo sapiens
56886	UDP-glucose ceramide glucosyltransferase-like 1	Homo sapiens
3221	homeobox C4	Homo sapiens
283871	phosphoglycolate phosphatase	Homo sapiens
79169	chromosome 1 open reading frame 35	Homo sapiens
60526	chromosome 2 open reading frame 43	Homo sapiens
3945	lactate dehydrogenase B	Homo sapiens
8739	harakiri, BCL2 interacting protein (contains only BH3 domain)	Homo sapiens
144097	chromosome 11 open reading frame 84	Homo sapiens
7474	wingless-type MMTV integration site family, member 5A	Homo sapiens
605	B-cell CLL/lymphoma 7A	Homo sapiens
140883	zinc finger protein 280B	Homo sapiens
645181	PDGFA associated protein 1; similar to PDGFA associated protein 1	Homo sapiens
11333	PDGFA associated protein 1; similar to PDGFA associated protein 1	Homo sapiens
2247	fibroblast growth factor 2 (basic)	Homo sapiens
161779	piggyBac transposable element derived 4	Homo sapiens
7163	tumor protein D52	Homo sapiens
441282	aldo-keto reductase family 1, member B10 (aldose reductase); aldo-keto reductase family 1, member B10-like	Homo sapiens
57016	aldo-keto reductase family 1, member B10 (aldose reductase); aldo-keto reductase family 1, member B10-like	Homo sapiens
1107	chromodomain helicase DNA binding protein 3	Homo sapiens
64769	chromosome 1 open reading frame 149	Homo sapiens
5500	protein phosphatase 1, catalytic subunit, beta isoform; speedy homolog A (Xenopus laevis)	Homo sapiens
245711	protein phosphatase 1, catalytic subunit, beta isoform; speedy homolog A (Xenopus laevis)	Homo sapiens
80146	UDP-glucuronate decarboxylase 1	Homo sapiens
204851	homeodomain interacting protein kinase 1	Homo sapiens
25923	atlastin GTPase 3	Homo sapiens
64399	hedgehog interacting protein	Homo sapiens
10287	regulator of G-protein signaling 19	Homo sapiens
65244	spermatogenesis associated, serine-rich 2	Homo sapiens
388344	ribosomal protein L13 pseudogene 12; ribosomal protein L13	Homo sapiens
6137	ribosomal protein L13 pseudogene 12; ribosomal protein L13	Homo sapiens
28232	solute carrier organic anion transporter family, member 3A1	Homo sapiens
8520	histone acetyltransferase 1	Homo sapiens
50804	myelin expression factor 2	Homo sapiens
119	adducin 2 (beta)	Homo sapiens
5160	pyruvate dehydrogenase (lipoamide) alpha 1	Homo sapiens
84872	zinc finger CCCH-type containing 10	Homo sapiens
80764	THAP domain containing 7	Homo sapiens
55553	SRY (sex determining region Y)-box 6	Homo sapiens
56893	ubiquilin 4	Homo sapiens
9136	ribosomal RNA processing 9, small subunit (SSU) processome component, homolog (yeast)	Homo sapiens
57156	transmembrane protein 63C	Homo sapiens
79830	zinc finger, MYM-type 1	Homo sapiens
25970	SH2B adaptor protein 1	Homo sapiens
161527	hypothetical protein LOC161527	Homo sapiens
4627	myosin, heavy chain 9, non-muscle	Homo sapiens
91137	solute carrier family 25, member 46	Homo sapiens
6007	Rh blood group, D antigen	Homo sapiens
6532	solute carrier family 6 (neurotransmitter transporter, serotonin), member 4	Homo sapiens
388403	yippee-like 2 (Drosophila)	Homo sapiens
8864	period homolog 2 (Drosophila)	Homo sapiens
156	adrenergic, beta, receptor kinase 1	Homo sapiens
55008	hect domain and RLD 6	Homo sapiens
51069	mitochondrial ribosomal protein L2	Homo sapiens
84501	spire homolog 2 (Drosophila)	Homo sapiens
3749	potassium voltage-gated channel, Shaw-related subfamily, member 4	Homo sapiens
80124	valosin containing protein (p97)/p47 complex interacting protein 1	Homo sapiens
55670	peroxisomal biogenesis factor 26	Homo sapiens

6915	thromboxane A2 receptor	Homo sapiens
57162	pellino homolog 1 (Drosophila)	Homo sapiens
89891	WD repeat domain 34	Homo sapiens
6775066	NADH-ubiquinone oxidoreductase chain 5	Homo sapiens
4540	NADH-ubiquinone oxidoreductase chain 5	Homo sapiens
162998	olfactory receptor, family 7, subfamily D, member 2	Homo sapiens
2146	enhancer of zeste homolog 2 (Drosophila)	Homo sapiens
170960	zinc finger protein 721	Homo sapiens
23360	formin binding protein 4	Homo sapiens
57088	phospholipid scramblase 4	Homo sapiens
54901	CDK5 regulatory subunit associated protein 1-like 1	Homo sapiens
54814	glutaminyl-peptide cyclotransferase-like	Homo sapiens
79867	tectonic family member 2	Homo sapiens
494513	deafness, autosomal recessive 59	Homo sapiens
84706	glutamic pyruvate transaminase (alanine aminotransferase) 2	Homo sapiens
8975	ubiquitin specific peptidase 13 (isopeptidase T-3)	Homo sapiens
116255	monoacylglycerol O-acyltransferase 1	Homo sapiens
9878	TOX high mobility group box family member 4	Homo sapiens
2762	GDP-mannose 4,6-dehydratase	Homo sapiens
1463	neurocan	Homo sapiens
25829	transmembrane protein 184B	Homo sapiens
5716	proteasome (prosome, macropain) 26S subunit, non-ATPase, 10	Homo sapiens
55593	OTU domain containing 5	Homo sapiens
4698	NADH dehydrogenase (ubiquinone) 1 alpha subcomplex, 5, 13kDa	Homo sapiens
338382	RAB7B, member RAS oncogene family	Homo sapiens
5455	POU class 3 homeobox 3	Homo sapiens
140823	reactive oxygen species modulator 1	Homo sapiens
2948	glutathione S-transferase mu 4	Homo sapiens
114786	XK, Kell blood group complex subunit-related family, member 4	Homo sapiens
29128	ubiquitin-like with PHD and ring finger domains 1	Homo sapiens
29916	sorting nexin 11	Homo sapiens
8065	cullin 5	Homo sapiens
8452	cullin 3	Homo sapiens
25831	HECT domain containing 1	Homo sapiens
10451	vav 3 guanine nucleotide exchange factor	Homo sapiens
476	ATPase, Na+/K+ transporting, alpha 1 polypeptide	Homo sapiens
1656	DEAD (Asp-Glu-Ala-Asp) box polypeptide 6	Homo sapiens
2023	enolase 1, (alpha)	Homo sapiens
6775079	Cytochrome c oxidase subunit 2	Homo sapiens
4513	Cytochrome c oxidase subunit 2	Homo sapiens
644390	similar to heterogeneous nuclear ribonucleoprotein L-like; heterogeneous nuclear ribonucleoprotein L	Homo sapiens
3191	similar to heterogeneous nuclear ribonucleoprotein L-like; heterogeneous nuclear ribonucleoprotein L	Homo sapiens
5129	PCTAIRE protein kinase 3	Homo sapiens
7414	vinculin	Homo sapiens
54865	G patch domain containing 4	Homo sapiens
10116	fem-1 homolog b (C. elegans)	Homo sapiens
93010	UDP-GlcNAc:betaGal beta-1,3-N-acetylglucosaminyltransferase 7	Homo sapiens
1349	cytochrome c oxidase subunit VIIb	Homo sapiens
1005	cadherin 7, type 2	Homo sapiens
953	ectonucleoside triphosphate diphosphohydrolase 1	Homo sapiens
84333	polycomb group ring finger 5	Homo sapiens
5601	mitogen-activated protein kinase 9	Homo sapiens
9134	cyclin E2	Homo sapiens
84056	katanin p60 subunit A-like 1	Homo sapiens
11040	pim-2 oncogene	Homo sapiens
10815	complexin 1	Homo sapiens
5980	REV3-like, catalytic subunit of DNA polymerase zeta (yeast)	Homo sapiens
10114	homeodomain interacting protein kinase 3	Homo sapiens
6391	succinate dehydrogenase complex, subunit C, integral membrane protein, 15kDa	Homo sapiens
79589	ring finger protein 128	Homo sapiens
29890	RNA binding motif protein 15B	Homo sapiens
92140	metadherin	Homo sapiens
83732	RIO kinase 1 (yeast)	Homo sapiens
5711	proteasome (prosome, macropain) 26S subunit, non-ATPase, 5	Homo sapiens
154791	chromosome 7 open reading frame 55	Homo sapiens
10423	CDP-diacylglycerol--inositol 3-phosphatidyltransferase (phosphatidylinositol synthase)	Homo sapiens
100134291	similar to mitogen-activated protein kinase phosphatase x; dual specificity phosphatase 22	Homo sapiens
56940	similar to mitogen-activated protein kinase phosphatase x; dual specificity phosphatase 22	Homo sapiens
57647	DEAH (Asp-Glu-Ala-His) box polypeptide 37	Homo sapiens
120526	DnaJ (Hsp40) homolog, subfamily C, member 24	Homo sapiens
9786	KIAA0586	Homo sapiens
81553	family with sequence similarity 49, member A	Homo sapiens
9495	A kinase (PRKA) anchor protein 5	Homo sapiens
11163	nudix (nucleoside diphosphate linked moiety X)-type motif 4; nudix (nucleoside diphosphate linked moiety X)-type motif 4 pseudogene 1	Homo sapiens

440672	nudix (nucleoside diphosphate linked moiety X)-type motif 4; nudix (nucleoside diphosphate linked moiety X)-type motif 4 pseudogene 1	Homo sapiens
728116	zinc finger and BTB domain containing 8B	Homo sapiens
23276	kelch-like 18 (Drosophila)	Homo sapiens
9262	serine/threonine kinase 17b	Homo sapiens
80034	cysteine-serine-rich nuclear protein 3	Homo sapiens
5284	polymeric immunoglobulin receptor	Homo sapiens
114904	C1q and tumor necrosis factor related protein 6	Homo sapiens
10478	solute carrier family 25 (mitochondrial carrier; peroxisomal membrane protein, 34kDa), member 17	Homo sapiens
84864	MYC induced nuclear antigen	Homo sapiens
442041	similar to zinc finger protein 347; zinc finger protein 532	Homo sapiens
55205	similar to zinc finger protein 347; zinc finger protein 532	Homo sapiens
60509	ATP/GTP binding protein-like 5	Homo sapiens
8408	unc-51-like kinase 1 (C. elegans)	Homo sapiens
51281	ankyrin repeat and MYND domain containing 1	Homo sapiens
4200	malic enzyme 2, NAD(+)-dependent, mitochondrial	Homo sapiens
114793	formin-like 2	Homo sapiens
7756	zinc finger protein 207	Homo sapiens
9733	squamous cell carcinoma antigen recognized by T cells 3	Homo sapiens
157769	family with sequence similarity 91, member A1	Homo sapiens
6506	solute carrier family 1 (glial high affinity glutamate transporter), member 2	Homo sapiens
4673	nucleosome assembly protein 1-like 1	Homo sapiens
100132369	WNK lysine deficient protein kinase 1; hypothetical LOC100132369	Homo sapiens
65125	WNK lysine deficient protein kinase 1; hypothetical LOC100132369	Homo sapiens
57655	GRAM domain containing 1A	Homo sapiens
10180	RNA binding motif protein 6	Homo sapiens
10516	fibulin 5	Homo sapiens
8573	calcium/calmodulin-dependent serine protein kinase (MAGUK family)	Homo sapiens
1284	collagen, type IV, alpha 2	Homo sapiens
7184	heat shock protein 90kDa beta (Grp94), member 1	Homo sapiens
57524	CASK interacting protein 1	Homo sapiens
3760	potassium inwardly-rectifying channel, subfamily J, member 3	Homo sapiens
1558	cytochrome P450, family 2, subfamily C, polypeptide 8	Homo sapiens
131890	G protein-coupled receptor kinase 7	Homo sapiens
26135	SERPINE1 mRNA binding protein 1	Homo sapiens
25998	inhibitor of Bruton agammaglobulinemia tyrosine kinase	Homo sapiens
220	aldehyde dehydrogenase 1 family, member A3	Homo sapiens
646227	cAMP-regulated phosphoprotein 19 pseudogene; cAMP-regulated phosphoprotein, 19kDa	Homo sapiens
10776	cAMP-regulated phosphoprotein 19 pseudogene; cAMP-regulated phosphoprotein, 19kDa	Homo sapiens
643896	cAMP-regulated phosphoprotein 19 pseudogene; cAMP-regulated phosphoprotein, 19kDa	Homo sapiens
57546	pyruvate dehyrogenase phosphatase catalytic subunit 2	Homo sapiens
51319	arginine/serine-rich coiled-coil 1	Homo sapiens
645381	similar to transducin-like enhancer of split 1 (E(sp1) homolog, Drosophila); transducin-like enhancer of split 1 (E(sp1) homolog, Drosophila)	Homo sapiens
7088	similar to transducin-like enhancer of split 1 (E(sp1) homolog, Drosophila); transducin-like enhancer of split 1 (E(sp1) homolog, Drosophila)	Homo sapiens
4704	NADH dehydrogenase (ubiquinone) 1 alpha subcomplex, 9, 39kDa	Homo sapiens
23218	neurobeachin-like 2	Homo sapiens
57488	family with sequence similarity 62 (C2 domain containing), member B	Homo sapiens
4026	LIM domain containing preferred translocation partner in lipoma	Homo sapiens
57704	glucosidase, beta (bile acid) 2	Homo sapiens
9924	PAN2 poly(A) specific ribonuclease subunit homolog (S. cerevisiae)	Homo sapiens
661	polymerase (RNA) III (DNA directed) polypeptide D, 44kDa	Homo sapiens
29904	eukaryotic elongation factor-2 kinase	Homo sapiens
84775	zinc finger protein 607	Homo sapiens
57565	kelch-like 14 (Drosophila)	Homo sapiens
51545	zinc finger protein 581	Homo sapiens
29968	chromosome 8 open reading frame 62; phosphoserine aminotransferase 1	Homo sapiens
137133	chromosome 8 open reading frame 62; phosphoserine aminotransferase 1	Homo sapiens
2801	golgi autoantigen, golgin subfamily a, 2	Homo sapiens
2241	fer (fps/fes related) tyrosine kinase	Homo sapiens
81573	ankyrin repeat domain 13C	Homo sapiens
10184	lipoma HMGIC fusion partner-like 2	Homo sapiens
65264	ubiquitin-conjugating enzyme E2Z	Homo sapiens
1830	desmoglein 3 (pemphigus vulgaris antigen)	Homo sapiens
386607	zinc finger protein 91 homolog (mouse); ZFP91-CNTF readthrough transcript; ciliary neurotrophic factor	Homo sapiens
1270	zinc finger protein 91 homolog (mouse); ZFP91-CNTF readthrough transcript; ciliary neurotrophic factor	Homo sapiens
80829	zinc finger protein 91 homolog (mouse); ZFP91-CNTF readthrough transcript; ciliary neurotrophic factor	Homo sapiens
4905	N-ethylmaleimide-sensitive factor	Homo sapiens
5298	phosphatidylinositol 4-kinase, catalytic, beta	Homo sapiens
83752	lon peptidase 2, peroxisomal	Homo sapiens
9646	Ctr9, Paf1/RNA polymerase II complex component, homolog (S. cerevisiae)	Homo sapiens
904	cyclin T1	Homo sapiens
9909	DENN/MADD domain containing 4B	Homo sapiens
547	kinesin family member 1A	Homo sapiens

5309	paired-like homeodomain 3	Homo sapiens
2966	general transcription factor IIH, polypeptide 2, 44kDa; general transcription factor IIH, polypeptide 2C; general transcription factor IIH, polypeptide 2B; general transcription factor IIH, polypeptide 2D	Homo sapiens
730394	general transcription factor IIH, polypeptide 2, 44kDa; general transcription factor IIH, polypeptide 2C; general transcription factor IIH, polypeptide 2B; general transcription factor IIH, polypeptide 2D	Homo sapiens
653238	general transcription factor IIH, polypeptide 2, 44kDa; general transcription factor IIH, polypeptide 2C; general transcription factor IIH, polypeptide 2B; general transcription factor IIH, polypeptide 2D	Homo sapiens
728340	general transcription factor IIH, polypeptide 2, 44kDa; general transcription factor IIH, polypeptide 2C; general transcription factor IIH, polypeptide 2B; general transcription factor IIH, polypeptide 2D	Homo sapiens
28988	drebrin-like	Homo sapiens
23466	chromobox homolog 6	Homo sapiens
22846	vasohibin 1	Homo sapiens
7019	transcription factor A, mitochondrial	Homo sapiens
126272	EP300 interacting inhibitor of differentiation 2B	Homo sapiens
4218	RAB8A, member RAS oncogene family	Homo sapiens
51465	ubiquitin-conjugating enzyme E2, J1 (UBC6 homolog, yeast)	Homo sapiens
9963	solute carrier family 23 (nucleobase transporters), member 1	Homo sapiens
50852	T cell receptor associated transmembrane adaptor 1	Homo sapiens
9528	transmembrane protein 59	Homo sapiens
1314	coatomer protein complex, subunit alpha	Homo sapiens
2107	eukaryotic translation termination factor 1	Homo sapiens
26147	PHD finger protein 19	Homo sapiens
4191	malate dehydrogenase 2, NAD (mitochondrial)	Homo sapiens
26469	protein tyrosine phosphatase, non-receptor type 18 (brain-derived)	Homo sapiens
55862	enoyl Coenzyme A hydratase domain containing 1	Homo sapiens
390980	zinc finger protein 805	Homo sapiens
1947	ephrin-B1	Homo sapiens
25921	zinc finger, DHHC-type containing 5	Homo sapiens
26090	abhydrolase domain containing 12	Homo sapiens
163786	spindle assembly 6 homolog (C. elegans)	Homo sapiens
8914	timeless homolog (Drosophila)	Homo sapiens
6311	ataxin 2	Homo sapiens
643446	similar to ribonucleic acid binding protein S1; RNA binding protein S1, serine-rich domain	Homo sapiens
10921	similar to ribonucleic acid binding protein S1; RNA binding protein S1, serine-rich domain	Homo sapiens
9958	ubiquitin specific peptidase 15	Homo sapiens
1890	thymidine phosphorylase	Homo sapiens
8079	myeloid leukemia factor 2	Homo sapiens
55333	synaptojanin 2 binding protein	Homo sapiens
55293	UEV and lactate/malate dehyrogenase domains	Homo sapiens
2870	G protein-coupled receptor kinase 6	Homo sapiens
445815	A kinase (PRKA) anchor protein 2; paralemmin 2; PALM2-AKAP2 readthrough transcript	Homo sapiens
11217	A kinase (PRKA) anchor protein 2; paralemmin 2; PALM2-AKAP2 readthrough transcript	Homo sapiens
114299	A kinase (PRKA) anchor protein 2; paralemmin 2; PALM2-AKAP2 readthrough transcript	Homo sapiens
100131401	basic transcription factor 3-like 4; similar to hCG2008008	Homo sapiens
91408	basic transcription factor 3-like 4; similar to hCG2008008	Homo sapiens
128240	apolipoprotein A-I binding protein	Homo sapiens
51377	ubiquitin carboxyl-terminal hydrolase L5	Homo sapiens
65005	mitochondrial ribosomal protein L9	Homo sapiens
346653	family with sequence similarity 71, member F2	Homo sapiens
55166	centromere protein Q	Homo sapiens
80830	apolipoprotein L, 6	Homo sapiens
6732	SFRS protein kinase 1	Homo sapiens
58516	similar to hCG2020539; family with sequence similarity 60, member A; similar to family with sequence similarity 60, member A	Homo sapiens
728115	similar to hCG2020539; family with sequence similarity 60, member A; similar to family with sequence similarity 60, member A	Homo sapiens
728592	similar to hCG2020539; family with sequence similarity 60, member A; similar to family with sequence similarity 60, member A	Homo sapiens
3431	SP110 nuclear body protein	Homo sapiens
4363	ATP-binding cassette, sub-family C (CFTR/MRP), member 1	Homo sapiens
28973	mitochondrial ribosomal protein S18B	Homo sapiens
3949	low density lipoprotein receptor	Homo sapiens
10605	poly(A) binding protein interacting protein 1; similar to poly(A) binding protein interacting protein 1	Homo sapiens
645139	poly(A) binding protein interacting protein 1; similar to poly(A) binding protein interacting protein 1	Homo sapiens
83394	PITPNM family member 3	Homo sapiens
3157	3-hydroxy-3-methylglutaryl-Coenzyme A synthase 1 (soluble)	Homo sapiens
7087	intercellular adhesion molecule 5, telencephalin	Homo sapiens
10138	YY1 associated factor 2	Homo sapiens
6385	syndecan 4	Homo sapiens
7597	zinc finger and BTB domain containing 25	Homo sapiens
84614	zinc finger and BTB domain containing 37	Homo sapiens

8086	achalasia, adrenocortical insufficiency, alacrimia (Allgrove, triple-A)	Homo sapiens
100130108	zinc finger protein 98 (F7175); keratin associated protein 1-1; zinc finger protein 492; zinc finger protein 849 pseudogene	Homo sapiens
57615	zinc finger protein 98 (F7175); keratin associated protein 1-1; zinc finger protein 492; zinc finger protein 849 pseudogene	Homo sapiens
81851	zinc finger protein 98 (F7175); keratin associated protein 1-1; zinc finger protein 492; zinc finger protein 849 pseudogene	Homo sapiens
148198	zinc finger protein 98 (F7175); keratin associated protein 1-1; zinc finger protein 492; zinc finger protein 849 pseudogene	Homo sapiens
6659	SRY (sex determining region Y)-box 4	Homo sapiens
56751	BarH-like homeobox 1	Homo sapiens
159	adenylosuccinate synthase	Homo sapiens
1777	deoxyribonuclease II, lysosomal	Homo sapiens
130502	tetratricopeptide repeat domain 32	Homo sapiens
192111	phosphoglycerate mutase family member 5	Homo sapiens
55500	ethanolamine kinase 1	Homo sapiens
7035	tissue factor pathway inhibitor (lipoprotein-associated coagulation inhibitor)	Homo sapiens
79801	SHC SH2-domain binding protein 1	Homo sapiens
201562	protein tyrosine phosphatase-like (proline instead of catalytic arginine), member b	Homo sapiens
29	active BCR-related gene	Homo sapiens
80317	zinc finger with KRAB and SCAN domains 3	Homo sapiens
55175	kelch-like 11 (Drosophila)	Homo sapiens
84888	signal peptide peptidase-like 2A	Homo sapiens
3939	lactate dehydrogenase A	Homo sapiens
116842	liver expressed antimicrobial peptide 2	Homo sapiens
79019	centromere protein M	Homo sapiens
3268	ArfGAP with FG repeats 2	Homo sapiens
157378	transmembrane protein 65	Homo sapiens
6472	serine hydroxymethyltransferase 2 (mitochondrial)	Homo sapiens
9209	leucine rich repeat (in FLII) interacting protein 2	Homo sapiens
151742	protein phosphatase 1 (formerly 2C)-like	Homo sapiens
20	ATP-binding cassette, sub-family A (ABC1), member 2	Homo sapiens
138428	peptidyl-tRNA hydrolase 1 homolog (S. cerevisiae)	Homo sapiens
10380	3'(2'), 5'-bisphosphate nucleotidase 1	Homo sapiens
9227	lecithin retinol acyltransferase (phosphatidylcholine--retinol O-acyltransferase)	Homo sapiens
3005	H1 histone family, member 0	Homo sapiens
25966	C2 calcium-dependent domain containing 2	Homo sapiens
51804	SIX homeobox 4	Homo sapiens
51655	RAS, dexamethasone-induced 1	Homo sapiens
1762	dystrophia myotonica, WD repeat containing	Homo sapiens
9931	helicase with zinc finger	Homo sapiens
6227	ribosomal protein S21	Homo sapiens
9394	heparan sulfate 6-O-sulfotransferase 1	Homo sapiens
8733	glycosylphosphatidylinositol anchor attachment protein 1 homolog (yeast)	Homo sapiens
2739	glyoxalase I	Homo sapiens
6734	signal recognition particle receptor (docking protein)	Homo sapiens
10645	calcium/calmodulin-dependent protein kinase kinase 2, beta	Homo sapiens
3842	transportin 1	Homo sapiens
154810	angiomotin like 1	Homo sapiens
9866	tripartite motif-containing 66	Homo sapiens
30968	stomatin (EPB72)-like 2	Homo sapiens
285521	COX18 cytochrome c oxidase assembly homolog (S. cerevisiae)	Homo sapiens
155061	zinc finger protein 746	Homo sapiens
4090	SMAD family member 5	Homo sapiens
284371	zinc finger protein 841	Homo sapiens
55734	zinc finger protein 64 homolog (mouse)	Homo sapiens
1534	cytochrome b-561	Homo sapiens
115294	protein-L-isoaspartate (D-aspartate) O-methyltransferase domain containing 1	Homo sapiens
1582	cytochrome P450, family 8, subfamily B, polypeptide 1	Homo sapiens
201595	STT3, subunit of the oligosaccharyltransferase complex, homolog B (S. cerevisiae)	Homo sapiens
10988	methionyl aminopeptidase 2	Homo sapiens
53344	cysteine-rich hydrophobic domain 1	Homo sapiens
6182	mitochondrial ribosomal protein L12	Homo sapiens
84807	nuclear factor of kappa light polypeptide gene enhancer in B-cells inhibitor, delta	Homo sapiens
6883	TAF12 RNA polymerase II, TATA box binding protein (TBP)-associated factor, 20kDa	Homo sapiens
22874	pleckstrin homology domain containing, family A member 6	Homo sapiens
9895	tectonin beta-propeller repeat containing 2	Homo sapiens
9899	synaptic vesicle glycoprotein 2B; hypothetical protein LOC100128403	Homo sapiens
100128403	synaptic vesicle glycoprotein 2B; hypothetical protein LOC100128403	Homo sapiens
29894	cleavage and polyadenylation specific factor 1, 160kDa	Homo sapiens
646019	chibby homolog 3 (Drosophila)	Homo sapiens
81894	solute carrier family 25, member 28	Homo sapiens
26048	zinc finger protein 500	Homo sapiens
30850	cerebellar degeneration-related protein 2-like	Homo sapiens
23469	PHD finger protein 3	Homo sapiens
108	adenylate cyclase 2 (brain)	Homo sapiens
2213	Fc fragment of IgG, low affinity IIb, receptor (CD32); Fc fragment of IgG, low affinity IIc, receptor for (CD32)	Homo sapiens

9103	Fc fragment of IgG, low affinity IIb, receptor (CD32); Fc fragment of IgG, low affinity IIc, receptor for (CD32)	Homo sapiens
23512	suppressor of zeste 12 homolog (Drosophila)	Homo sapiens
55771	proline rich 11	Homo sapiens
10838	zinc finger protein 275	Homo sapiens
55930	myosin VC	Homo sapiens
6549	solute carrier family 9 (sodium/hydrogen exchanger), member 2	Homo sapiens
388536	zinc finger protein 790	Homo sapiens
1374	carnitine palmitoyltransferase 1A (liver)	Homo sapiens
8893	eukaryotic translation initiation factor 2B, subunit 5 epsilon, 82kDa	Homo sapiens
23338	PHD finger protein 15	Homo sapiens
4237	microfibrillar-associated protein 2	Homo sapiens
8087	fragile X mental retardation, autosomal homolog 1	Homo sapiens
388552	biogenesis of lysosomal organelles complex-1, subunit 3	Homo sapiens
51279	complement component 1, r subcomponent-like	Homo sapiens
253639	zinc finger protein 620	Homo sapiens
2786	guanine nucleotide binding protein (G protein), gamma 4	Homo sapiens
55088	chromosome 10 open reading frame 118	Homo sapiens
400389	ribosomal protein L12 pseudogene 2; ribosomal protein L12 pseudogene 32; ribosomal protein L12 pseudogene 35; ribosomal protein L12 pseudogene 19; ribosomal protein L12 pseudogene 6; ribosomal protein L12; ribosomal protein L12 pseudogene 14	Homo sapiens
440176	ribosomal protein L12 pseudogene 2; ribosomal protein L12 pseudogene 32; ribosomal protein L12 pseudogene 35; ribosomal protein L12 pseudogene 19; ribosomal protein L12 pseudogene 6; ribosomal protein L12; ribosomal protein L12 pseudogene 14	Homo sapiens
6136	ribosomal protein L12 pseudogene 2; ribosomal protein L12 pseudogene 32; ribosomal protein L12 pseudogene 35; ribosomal protein L12 pseudogene 19; ribosomal protein L12 pseudogene 6; ribosomal protein L12; ribosomal protein L12 pseudogene 14	Homo sapiens
100129982	ribosomal protein L12 pseudogene 2; ribosomal protein L12 pseudogene 32; ribosomal protein L12 pseudogene 35; ribosomal protein L12 pseudogene 19; ribosomal protein L12 pseudogene 6; ribosomal protein L12; ribosomal protein L12 pseudogene 14	Homo sapiens
100132795	ribosomal protein L12 pseudogene 2; ribosomal protein L12 pseudogene 32; ribosomal protein L12 pseudogene 35; ribosomal protein L12 pseudogene 19; ribosomal protein L12 pseudogene 6; ribosomal protein L12; ribosomal protein L12 pseudogene 14	Homo sapiens
646875	ribosomal protein L12 pseudogene 2; ribosomal protein L12 pseudogene 32; ribosomal protein L12 pseudogene 35; ribosomal protein L12 pseudogene 19; ribosomal protein L12 pseudogene 6; ribosomal protein L12; ribosomal protein L12 pseudogene 14	Homo sapiens
729500	ribosomal protein L12 pseudogene 2; ribosomal protein L12 pseudogene 32; ribosomal protein L12 pseudogene 35; ribosomal protein L12 pseudogene 19; ribosomal protein L12 pseudogene 6; ribosomal protein L12; ribosomal protein L12 pseudogene 14	Homo sapiens
54541	DNA-damage-inducible transcript 4	Homo sapiens
3783	potassium intermediate/small conductance calcium-activated channel, subfamily N, member 4	Homo sapiens
140688	chromosome 20 open reading frame 112	Homo sapiens
27010	thiamin pyrophosphokinase 1	Homo sapiens
9183	ZW10, kinetochore associated, homolog (Drosophila)	Homo sapiens
100131149	NADH dehydrogenase (ubiquinone) 1 alpha subcomplex, assembly factor 4; similar to HSPC125	Homo sapiens
29078	NADH dehydrogenase (ubiquinone) 1 alpha subcomplex, assembly factor 4; similar to HSPC125	Homo sapiens
4437	mutS homolog 3 (E. coli)	Homo sapiens
255626	histone cluster 1, H2ba	Homo sapiens
4719	NADH dehydrogenase (ubiquinone) Fe-S protein 1, 75kDa (NADH-coenzyme Q reductase)	Homo sapiens
80021	transmembrane protein 62	Homo sapiens
10128	leucine-rich PPR-motif containing	Homo sapiens
219	aldehyde dehydrogenase 1 family, member B1	Homo sapiens
81034	solute carrier family 25, member 32	Homo sapiens
1312	catechol-O-methyltransferase	Homo sapiens
54867	transmembrane protein 214	Homo sapiens
10617	STAM binding protein	Homo sapiens
85015	ubiquitin specific peptidase 45	Homo sapiens
54805	cyclin M2	Homo sapiens
3110	motor neuron and pancreas homeobox 1	Homo sapiens
29855	ubinuclein 1	Homo sapiens
7203	chaperonin containing TCP1, subunit 3 (gamma)	Homo sapiens
94274	protein phosphatase 1, regulatory (inhibitor) subunit 14A	Homo sapiens
65008	mitochondrial ribosomal protein L1	Homo sapiens
29789	Obg-like ATPase 1	Homo sapiens
168850	zinc finger protein 800	Homo sapiens
51513	ets variant 7	Homo sapiens
1808	dihydropyrimidinase-like 2	Homo sapiens
223082	zinc and ring finger 2	Homo sapiens
124930	ankyrin repeat domain 13B	Homo sapiens
57721	methyltransferase like 14	Homo sapiens
9170	lysophosphatidic acid receptor 2	Homo sapiens
5326	pleiomorphic adenoma gene-like 2; similar to pleiomorphic adenoma gene-like 2	Homo sapiens
152845	pleiomorphic adenoma gene-like 2; similar to pleiomorphic adenoma gene-like 2	Homo sapiens
705	bystin-like	Homo sapiens
10492	synaptotagmin binding, cytoplasmic RNA interacting protein	Homo sapiens
400935	interleukin 17 receptor E-like	Homo sapiens

93129	ORAI calcium release-activated calcium modulator 3	Homo sapiens
11285	xylosylprotein beta 1,4-galactosyltransferase, polypeptide 7 (galactosyltransferase I)	Homo sapiens
55048	vacuolar protein sorting 37 homolog C (S. cerevisiae)	Homo sapiens
92558	coiled-coil domain containing 64	Homo sapiens
1871	E2F transcription factor 3	Homo sapiens
23299	bicaudal D homolog 2 (Drosophila)	Homo sapiens
5578	protein kinase C, alpha	Homo sapiens
3927	LIM and SH3 protein 1	Homo sapiens
79618	homeobox containing 1	Homo sapiens
79585	coronin 7	Homo sapiens
9823	armadillo repeat containing, X-linked 2	Homo sapiens
22993	HMG box domain containing 3	Homo sapiens
9825	spermatogenesis associated 2	Homo sapiens
10342	TRK-fused gene	Homo sapiens
29035	chromosome 16 open reading frame 72	Homo sapiens
5257	phosphorylase kinase, beta	Homo sapiens
2705	gap junction protein, beta 1, 32kDa	Homo sapiens
9092	squamous cell carcinoma antigen recognized by T cells	Homo sapiens
51696	headcase homolog (Drosophila)	Homo sapiens
51514	denticleless homolog (Drosophila)	Homo sapiens
84159	AT rich interactive domain 5B (MRF1-like)	Homo sapiens
115548	FCH domain only 2	Homo sapiens
11320	mannosyl (alpha-1,3-)-glycoprotein beta-1,4-N-acetylglucosaminyltransferase, isozyme A	Homo sapiens
129049	small G protein signaling modulator 1	Homo sapiens
6721	sterol regulatory element binding transcription factor 2	Homo sapiens
1844	dual specificity phosphatase 2	Homo sapiens
64410	kelch-like 25 (Drosophila)	Homo sapiens
253832	zinc finger, DHHC-type containing 20	Homo sapiens
7627	zinc finger protein 75a	Homo sapiens
55843	Rho GTPase activating protein 15	Homo sapiens
132660	lin-54 homolog (C. elegans)	Homo sapiens
54962	TIMELESS interacting protein	Homo sapiens
51409	HemK methyltransferase family member 1	Homo sapiens
9973	copper chaperone for superoxide dismutase	Homo sapiens
4089	SMAD family member 4	Homo sapiens
84665	myopalladin	Homo sapiens
79710	MORC family CW-type zinc finger 4	Homo sapiens
4059	basal cell adhesion molecule (Lutheran blood group)	Homo sapiens
23141	ankyrin repeat and LEM domain containing 2	Homo sapiens
644	biliverdin reductase A	Homo sapiens
730144	similar to eukaryotic translation initiation factor 1; eukaryotic translation initiation factor 1	Homo sapiens
10209	similar to eukaryotic translation initiation factor 1; eukaryotic translation initiation factor 1	Homo sapiens
81559	tripartite motif-containing 11	Homo sapiens
160518	DENN/MADD domain containing 5B	Homo sapiens
7262	pleckstrin homology-like domain, family A, member 2	Homo sapiens
388335	transmembrane protein 220	Homo sapiens
10427	SEC24 family, member B (S. cerevisiae)	Homo sapiens
25778	dual serine/threonine and tyrosine protein kinase	Homo sapiens
57659	zinc finger and BTB domain containing 4	Homo sapiens
340554	zinc finger CCCH-type containing 12B	Homo sapiens
79913	ARP5 actin-related protein 5 homolog (yeast)	Homo sapiens
219541	mediator complex subunit 19	Homo sapiens
5209	6-phosphofructo-2-kinase/fructose-2,6-biphosphatase 3	Homo sapiens
23132	RAD54-like 2 (S. cerevisiae)	Homo sapiens
23511	nucleoporin 188kDa	Homo sapiens
4357	mercaptopyruvate sulfurtransferase	Homo sapiens
10558	serine palmitoyltransferase, long chain base subunit 1	Homo sapiens
8994	LIM domains containing 1	Homo sapiens
8335	histone cluster 1, H2ae; histone cluster 1, H2ab	Homo sapiens
3012	histone cluster 1, H2ae; histone cluster 1, H2ab	Homo sapiens
55750	acylglycerol kinase	Homo sapiens
23327	neural precursor cell expressed, developmentally down-regulated 4-like	Homo sapiens
2197	Finkel-Biskis-Reilly murine sarcoma virus (FBR-MuSV) ubiquitously expressed	Homo sapiens
4642	myosin ID	Homo sapiens
79707	nucleolar protein 9	Homo sapiens
144245	asparagine-linked glycosylation 10, alpha-1,2-glucosyltransferase homolog B (yeast)	Homo sapiens
23212	RRS1 ribosome biogenesis regulator homolog (S. cerevisiae)	Homo sapiens
79693	yrdC domain containing (E. coli)	Homo sapiens
10942	protease, serine, 21 (testisin)	Homo sapiens
2010	emerin	Homo sapiens
9770	Ras association (RalGDS/AF-6) domain family member 2	Homo sapiens
26060	adaptor protein, phosphotyrosine interaction, PH domain and leucine zipper containing 1	Homo sapiens
10643	insulin-like growth factor 2 mRNA binding protein 3	Homo sapiens
2055	ceroid-lipofuscinosis, neuronal 8 (epilepsy, progressive with mental retardation)	Homo sapiens
401250	mitochondrial coiled-coil domain 1	Homo sapiens
765	carbonic anhydrase VI	Homo sapiens
57697	Fanconi anemia, complementation group M	Homo sapiens

427	N-acylsphingosine amidohydrolase (acid ceramidase) 1	Homo sapiens
26010	spermatogenesis associated, serine-rich 2-like	Homo sapiens
6518	solute carrier family 2 (facilitated glucose/fructose transporter), member 5	Homo sapiens
51280	golgi membrane protein 1	Homo sapiens
1295	collagen, type VIII, alpha 1	Homo sapiens
2108	electron-transfer-flavoprotein, alpha polypeptide	Homo sapiens
4215	mitogen-activated protein kinase kinase kinase 3	Homo sapiens
4077	neighbor of BRCA1 gene 1	Homo sapiens
10038	poly (ADP-ribose) polymerase 2	Homo sapiens
100132658	hypothetical gene supported by NM_014886; TGF beta-inducible nuclear protein 1; similar to TGF beta-inducible nuclear protein 1	Homo sapiens
10412	hypothetical gene supported by NM_014886; TGF beta-inducible nuclear protein 1; similar to TGF beta-inducible nuclear protein 1	Homo sapiens
400061	hypothetical gene supported by NM_014886; TGF beta-inducible nuclear protein 1; similar to TGF beta-inducible nuclear protein 1	Homo sapiens
54737	M-phase phosphoprotein 8	Homo sapiens
64750	SMAD specific E3 ubiquitin protein ligase 2	Homo sapiens
100131565	eukaryotic translation initiation factor 4E; similar to hCG1777996; similar to eukaryotic translation initiation factor 4E	Homo sapiens
1977	eukaryotic translation initiation factor 4E; similar to hCG1777996; similar to eukaryotic translation initiation factor 4E	Homo sapiens
100131693	eukaryotic translation initiation factor 4E; similar to hCG1777996; similar to eukaryotic translation initiation factor 4E	Homo sapiens
2194	fatty acid synthase	Homo sapiens
6927	HNF1 homeobox A	Homo sapiens
64779	methenyltetrahydrofolate synthetase domain containing	Homo sapiens
4784	nuclear factor I/X (CCAAT-binding transcription factor)	Homo sapiens
3856	keratin 8 pseudogene 9; similar to keratin 8; keratin 8	Homo sapiens
390601	keratin 8 pseudogene 9; similar to keratin 8; keratin 8	Homo sapiens
149501	keratin 8 pseudogene 9; similar to keratin 8; keratin 8	Homo sapiens
10534	Sjogren syndrome/scleroderma autoantigen 1	Homo sapiens
4086	SMAD family member 1	Homo sapiens
54769	DIRAS family, GTP-binding RAS-like 2	Homo sapiens
55699	isoleucyl-tRNA synthetase 2, mitochondrial	Homo sapiens
56947	mitochondrial fission factor	Homo sapiens
64794	DEAD (Asp-Glu-Ala-Asp) box polypeptide 31	Homo sapiens
55614	kinesin family member 16B	Homo sapiens
57713	Scm-like with four mbt domains 2	Homo sapiens
782	calcium channel, voltage-dependent, beta 1 subunit	Homo sapiens
57092	PEST proteolytic signal containing nuclear protein	Homo sapiens
1213	clathrin, heavy chain (Hc)	Homo sapiens
440603	BCL2-like 15	Homo sapiens
64901	RAN binding protein 17	Homo sapiens
64326	ring finger and WD repeat domain 2	Homo sapiens
10521	DEAD (Asp-Glu-Ala-Asp) box polypeptide 17	Homo sapiens
388524	ribosomal protein SA pseudogene 9; ribosomal protein SA pseudogene 8; ribosomal protein SA pseudogene 58; ribosomal protein SA pseudogene 19; ribosomal protein SA pseudogene 18; ribosomal protein SA; ribosomal protein SA pseudogene 15; ribosomal protein SA pseudogene 61; ribosomal protein SA pseudogene 29; ribosomal protein SA pseudogene 12	Homo sapiens
389141	ribosomal protein SA pseudogene 9; ribosomal protein SA pseudogene 8; ribosomal protein SA pseudogene 58; ribosomal protein SA pseudogene 19; ribosomal protein SA pseudogene 18; ribosomal protein SA; ribosomal protein SA pseudogene 15; ribosomal protein SA pseudogene 61; ribosomal protein SA pseudogene 29; ribosomal protein SA pseudogene 12	Homo sapiens
387867	ribosomal protein SA pseudogene 9; ribosomal protein SA pseudogene 8; ribosomal protein SA pseudogene 58; ribosomal protein SA pseudogene 19; ribosomal protein SA pseudogene 18; ribosomal protein SA; ribosomal protein SA pseudogene 15; ribosomal protein SA pseudogene 61; ribosomal protein SA pseudogene 29; ribosomal protein SA pseudogene 12	Homo sapiens
220885	ribosomal protein SA pseudogene 9; ribosomal protein SA pseudogene 8; ribosomal protein SA pseudogene 58; ribosomal protein SA pseudogene 19; ribosomal protein SA pseudogene 18; ribosomal protein SA; ribosomal protein SA pseudogene 15; ribosomal protein SA pseudogene 61; ribosomal protein SA pseudogene 29; ribosomal protein SA pseudogene 12	Homo sapiens
653162	ribosomal protein SA pseudogene 9; ribosomal protein SA pseudogene 8; ribosomal protein SA pseudogene 58; ribosomal protein SA pseudogene 19; ribosomal protein SA pseudogene 18; ribosomal protein SA; ribosomal protein SA pseudogene 15; ribosomal protein SA pseudogene 61; ribosomal protein SA pseudogene 29; ribosomal protein SA pseudogene 12	Homo sapiens
643617	ribosomal protein SA pseudogene 9; ribosomal protein SA pseudogene 8; ribosomal protein SA pseudogene 58; ribosomal protein SA pseudogene 19; ribosomal protein SA pseudogene 18; ribosomal protein SA; ribosomal protein SA pseudogene 15; ribosomal protein SA pseudogene 61; ribosomal protein SA pseudogene 29; ribosomal protein SA pseudogene 12	Homo sapiens
388707	ribosomal protein SA pseudogene 9; ribosomal protein SA pseudogene 8; ribosomal protein SA pseudogene 58; ribosomal protein SA pseudogene 19; ribosomal protein SA pseudogene 18; ribosomal protein SA; ribosomal protein SA pseudogene 15; ribosomal protein SA pseudogene 61; ribosomal protein SA pseudogene 29; ribosomal protein SA pseudogene 12	Homo sapiens
730029	ribosomal protein SA pseudogene 9; ribosomal protein SA pseudogene 8; ribosomal protein SA pseudogene 58; ribosomal protein SA pseudogene 19; ribosomal protein SA pseudogene 18; ribosomal protein SA; ribosomal protein SA pseudogene 15; ribosomal protein SA pseudogene 61; ribosomal protein SA pseudogene 29; ribosomal protein SA pseudogene 12	Homo sapiens

644464	ribosomal protein SA pseudogene 9; ribosomal protein SA pseudogene 8; ribosomal protein SA pseudogene 58; ribosomal protein SA pseudogene 19; ribosomal protein SA pseudogene 18; ribosomal protein SA; ribosomal protein SA pseudogene 15; ribosomal protein SA pseudogene 61; ribosomal protein SA pseudogene 29; ribosomal protein SA pseudogene 12	Homo sapiens
3921	ribosomal protein SA pseudogene 9; ribosomal protein SA pseudogene 8; ribosomal protein SA pseudogene 58; ribosomal protein SA pseudogene 19; ribosomal protein SA pseudogene 18; ribosomal protein SA; ribosomal protein SA pseudogene 15; ribosomal protein SA pseudogene 61; ribosomal protein SA pseudogene 29; ribosomal protein SA pseudogene 12	Homo sapiens
11339	Opa interacting protein 5	Homo sapiens
2625	GATA binding protein 3	Homo sapiens
8891	eukaryotic translation initiation factor 2B, subunit 3 gamma, 58kDa	Homo sapiens
375	ADP-ribosylation factor 1	Homo sapiens
3487	insulin-like growth factor binding protein 4	Homo sapiens
284186	transmembrane protein 105	Homo sapiens
84268	RPA interacting protein	Homo sapiens
2131	exostoses (multiple) 1	Homo sapiens
113157	ribosomal protein, large, P0 pseudogene 2; ribosomal protein, large, P0 pseudogene 3; ribosomal protein, large, P0 pseudogene 6; ribosomal protein, large, P0	Homo sapiens
220717	ribosomal protein, large, P0 pseudogene 2; ribosomal protein, large, P0 pseudogene 3; ribosomal protein, large, P0 pseudogene 6; ribosomal protein, large, P0	Homo sapiens
122589	ribosomal protein, large, P0 pseudogene 2; ribosomal protein, large, P0 pseudogene 3; ribosomal protein, large, P0 pseudogene 6; ribosomal protein, large, P0	Homo sapiens
6175	ribosomal protein, large, P0 pseudogene 2; ribosomal protein, large, P0 pseudogene 3; ribosomal protein, large, P0 pseudogene 6; ribosomal protein, large, P0	Homo sapiens
23035	PH domain and leucine rich repeat protein phosphatase 2	Homo sapiens
388695	LysM, putative peptidoglycan-binding, domain containing 1	Homo sapiens
146909	kinesin family member 18B	Homo sapiens
257240	kelch-like 34 (Drosophila)	Homo sapiens
4893	neuroblastoma RAS viral (v-ras) oncogene homolog	Homo sapiens
91647	ATP synthase mitochondrial F1 complex assembly factor 2	Homo sapiens
22868	FAST kinase domains 2	Homo sapiens
6573	solute carrier family 19 (folate transporter), member 1	Homo sapiens
10172	zinc finger protein 256	Homo sapiens
90594	zinc finger protein 439	Homo sapiens
221035	receptor accessory protein 3	Homo sapiens
4287	ataxin 3	Homo sapiens
80218	N-acetyltransferase 13 (GCN5-related)	Homo sapiens
132299	OCIA domain containing 2	Homo sapiens
80005	dedicator of cytokinesis 5	Homo sapiens
146664	mannosyl (alpha-1,6-)-glycoprotein beta-1,6-N-acetyl-glucosaminyltransferase, isozyme B	Homo sapiens
23478	SEC11 homolog A (S. cerevisiae)	Homo sapiens
10540	dynactin 2 (p50)	Homo sapiens
255743	nephronectin	Homo sapiens
147687	zinc finger protein 417	Homo sapiens
5686	proteasome (prosome, macropain) subunit, alpha type, 5	Homo sapiens
8434	reversion-inducing-cysteine-rich protein with kazal motifs	Homo sapiens
3630	insulin-like growth factor 2 (somatomedin A); insulin; INS-IGF2 readthrough transcript	Homo sapiens
723961	insulin-like growth factor 2 (somatomedin A); insulin; INS-IGF2 readthrough transcript	Homo sapiens
3481	insulin-like growth factor 2 (somatomedin A); insulin; INS-IGF2 readthrough transcript	Homo sapiens
55147	RNA binding motif protein 23	Homo sapiens
25790	coiled-coil domain containing 19	Homo sapiens
8976	Wiskott-Aldrich syndrome-like	Homo sapiens
3313	heat shock 70kDa protein 9 (mortalin)	Homo sapiens
5028	purinergic receptor P2Y, G-protein coupled, 1	Homo sapiens
201266	solute carrier family 39 (metal ion transporter), member 11	Homo sapiens
9444	quaking homolog, KH domain RNA binding (mouse)	Homo sapiens
57507	zinc finger protein 608	Homo sapiens
7639	zinc finger protein 85	Homo sapiens
3780	potassium intermediate/small conductance calcium-activated channel, subfamily N, member 1	Homo sapiens
284996	ring finger protein 149	Homo sapiens
79413	zinc finger, BED-type containing 2	Homo sapiens
54884	retinol saturase (all-trans-retinol 13,14-reductase)	Homo sapiens
11047	adhesion regulating molecule 1	Homo sapiens
10655	doublesex and mab-3 related transcription factor 2	Homo sapiens
10957	proline-rich nuclear receptor coactivator 1	Homo sapiens
116236	abhydrolase domain containing 15	Homo sapiens
5348	FXYD domain containing ion transport regulator 1	Homo sapiens
729595	similar to high mobility group box 3; high-mobility group box 3	Homo sapiens
729952	similar to high mobility group box 3; high-mobility group box 3	Homo sapiens
3149	similar to high mobility group box 3; high-mobility group box 3	Homo sapiens
646993	similar to high mobility group box 3; high-mobility group box 3	Homo sapiens
25840	methyltransferase like 7A	Homo sapiens
23279	nucleoporin 160kDa	Homo sapiens
23787	mitochondrial carrier homolog 1 (C. elegans)	Homo sapiens
8293	small EDRK-rich factor 1A (telomeric); small EDRK-rich factor 1B (centromeric)	Homo sapiens
728492	small EDRK-rich factor 1A (telomeric); small EDRK-rich factor 1B (centromeric)	Homo sapiens
79980	DSN1, MIND kinetochore complex component, homolog (S. cerevisiae)	Homo sapiens
5128	PCTAIRE protein kinase 2	Homo sapiens

578	BCL2-antagonist/killer 1; BCL2-like 7 pseudogene 1	Homo sapiens
600	BCL2-antagonist/killer 1; BCL2-like 7 pseudogene 1	Homo sapiens
4093	SMAD family member 9	Homo sapiens
57617	vacuolar protein sorting 18 homolog (S. cerevisiae)	Homo sapiens
27241	Bardet-Biedl syndrome 9	Homo sapiens
2850	G protein-coupled receptor 27	Homo sapiens
1260	cyclic nucleotide gated channel alpha 2	Homo sapiens
5885	RAD21 homolog (S. pombe)	Homo sapiens
6711	spectrin, beta, non-erythrocytic 1	Homo sapiens
643873	similar to eukaryotic translation initiation factor 4H; eukaryotic translation initiation factor 4B	Homo sapiens
1975	similar to eukaryotic translation initiation factor 4H; eukaryotic translation initiation factor 4B	Homo sapiens
64600	phospholipase A2, group IIF	Homo sapiens
9231	discs, large homolog 5 (Drosophila)	Homo sapiens
55294	F-box and WD repeat domain containing 7	Homo sapiens
63932	chromosome X open reading frame 56	Homo sapiens
55038	cell division cycle associated 4	Homo sapiens
148867	solute carrier family 30 (zinc transporter), member 7	Homo sapiens
9960	ubiquitin specific peptidase 3	Homo sapiens
94137	retinitis pigmentosa 1-like 1	Homo sapiens
8396	phosphatidylinositol-5-phosphate 4-kinase, type II, beta	Homo sapiens
8761	poly(A) binding protein, cytoplasmic 4 (inducible form)	Homo sapiens
84164	activating signal cointegrator 1 complex subunit 2	Homo sapiens
3914	laminin, beta 3	Homo sapiens
11130	ZW10 interactor	Homo sapiens
57506	mitochondrial antiviral signaling protein	Homo sapiens
10460	transforming, acidic coiled-coil containing protein 3	Homo sapiens
51150	stromal cell derived factor 4	Homo sapiens
124751	KRAB-A domain containing 2	Homo sapiens
168374	zinc finger protein 92	Homo sapiens
83636	chromosome 19 open reading frame 12	Homo sapiens
23109	dendrin	Homo sapiens
7186	TNF receptor-associated factor 2	Homo sapiens
51755	Cdc2-related kinase, arginine/serine-rich	Homo sapiens
23386	NudC domain containing 3	Homo sapiens
93624	transcriptional adaptor 2 (ADA2 homolog, yeast)-beta	Homo sapiens
6509	solute carrier family 1 (glutamate/neutral amino acid transporter), member 4	Homo sapiens
55163	pyridoxamine 5'-phosphate oxidase	Homo sapiens
7484	wingless-type MMTV integration site family, member 9B	Homo sapiens
4771	neurofibromin 2 (merlin)	Homo sapiens
10193	ring finger protein 41	Homo sapiens
3455	interferon (alpha, beta and omega) receptor 2	Homo sapiens
7336	ubiquitin-conjugating enzyme E2 variant 2	Homo sapiens
3224	homeobox C8	Homo sapiens
23764	v-maf musculoaponeurotic fibrosarcoma oncogene homolog F (avian)	Homo sapiens
85364	zinc finger, CCHC domain containing 3	Homo sapiens
3200	homeobox A3	Homo sapiens
10678	UDP-GlcNAc:betaGal beta-1,3-N-acetylglucosaminyltransferase 1; UDP-GlcNAc:betaGal beta-1,3-N-acetylglucosaminyltransferase 2	Homo sapiens
11041	UDP-GlcNAc:betaGal beta-1,3-N-acetylglucosaminyltransferase 1; UDP-GlcNAc:betaGal beta-1,3-N-acetylglucosaminyltransferase 2	Homo sapiens
1163	CDC28 protein kinase regulatory subunit 1B	Homo sapiens
374407	DnaJ (Hsp40) related, subfamily B, member 13	Homo sapiens
122786	FERM domain containing 6	Homo sapiens
169611	olfactomedin-like 2A	Homo sapiens
10140	transducer of ERBB2, 1	Homo sapiens
131965	methyltransferase like 6	Homo sapiens
26762	hepatitis A virus cellular receptor 1	Homo sapiens
56255	thioredoxin-related transmembrane protein 4	Homo sapiens
81873	actin related protein 2/3 complex, subunit 5-like	Homo sapiens
374900	zinc finger protein 568	Homo sapiens
1822	atrophin 1	Homo sapiens
1434	CSE1 chromosome segregation 1-like (yeast)	Homo sapiens
80344	WD repeat domain 23	Homo sapiens
11321	GPN-loop GTPase 1	Homo sapiens
808	calmodulin 3 (phosphorylase kinase, delta); calmodulin 2 (phosphorylase kinase, delta); calmodulin 1 (phosphorylase kinase, delta)	Homo sapiens
805	calmodulin 3 (phosphorylase kinase, delta); calmodulin 2 (phosphorylase kinase, delta); calmodulin 1 (phosphorylase kinase, delta)	Homo sapiens
801	calmodulin 3 (phosphorylase kinase, delta); calmodulin 2 (phosphorylase kinase, delta); calmodulin 1 (phosphorylase kinase, delta)	Homo sapiens
56675	nuclear receptor interacting protein 3	Homo sapiens
7405	UV radiation resistance associated gene	Homo sapiens
728689	eukaryotic translation initiation factor 3, subunit C-like	Homo sapiens
10934	mortality factor 4; mortality factor 4 like 1	Homo sapiens
10933	mortality factor 4; mortality factor 4 like 1	Homo sapiens
92856	IMP4, U3 small nucleolar ribonucleoprotein, homolog (yeast)	Homo sapiens
23484	leptin receptor overlapping transcript-like 1	Homo sapiens

57461	ISY1 splicing factor homolog (S. cerevisiae)	Homo sapiens
445	argininosuccinate synthetase 1	Homo sapiens
7171	tropomyosin 4	Homo sapiens
57522	SLIT-ROBO Rho GTPase activating protein 1	Homo sapiens
10847	Snf2-related CREBBP activator protein	Homo sapiens
1352	COX10 homolog, cytochrome c oxidase assembly protein, heme A: farnesyltransferase (yeast)	Homo sapiens
1760	dystrophia myotonica-protein kinase	Homo sapiens
23549	aspartyl aminopeptidase	Homo sapiens
51759	chromosome 9 open reading frame 78	Homo sapiens
2802	golgi autoantigen, golgin subfamily a, 3	Homo sapiens
6452	SH3-domain binding protein 2	Homo sapiens
6907	transducin (beta)-like 1X-linked	Homo sapiens
23092	Rho GTPase activating protein 26	Homo sapiens
80196	ring finger protein 34	Homo sapiens
1211	clathrin, light chain (Lca)	Homo sapiens
790	carbamoyl-phosphate synthetase 2, aspartate transcarbamylase, and dihydroorotase	Homo sapiens
7070	Thy-1 cell surface antigen	Homo sapiens
65249	zinc finger, SWIM-type containing 4	Homo sapiens
54665	round spermatid basic protein 1	Homo sapiens
1891	enoyl Coenzyme A hydratase 1, peroxisomal	Homo sapiens
56943	enhancer of yellow 2 homolog (Drosophila)	Homo sapiens
125919	zinc finger protein 543	Homo sapiens
867	Cas-Br-M (murine) ecotropic retroviral transforming sequence	Homo sapiens
200014	coiled-coil and C2 domain containing 1B	Homo sapiens
64770	coiled-coil domain containing 14	Homo sapiens
8021	nucleoporin 214kDa	Homo sapiens
162963	zinc finger protein 610	Homo sapiens
4862	neuronal PAS domain protein 2	Homo sapiens
283237	tetratricopeptide repeat domain 9C	Homo sapiens
1280	collagen, type II, alpha 1	Homo sapiens
27284	sulfotransferase family, cytosolic, 1B, member 1	Homo sapiens
5805	6-pyruvoyltetrahydropterin synthase	Homo sapiens
7098	toll-like receptor 3	Homo sapiens
79137	family with sequence similarity 134, member A	Homo sapiens
83985	spinster homolog 1 (Drosophila)	Homo sapiens
5901	RAN, member RAS oncogene family	Homo sapiens
120114	FAT tumor suppressor homolog 3 (Drosophila)	Homo sapiens
3959	lectin, galactoside-binding, soluble, 3 binding protein	Homo sapiens
5930	retinoblastoma binding protein 6	Homo sapiens
79770	thioredoxin domain containing 15	Homo sapiens
22821	RAS p21 protein activator 3	Homo sapiens
6632	small nuclear ribonucleoprotein D1 polypeptide 16kDa; hypothetical protein LOC100129492	Homo sapiens
100129492	small nuclear ribonucleoprotein D1 polypeptide 16kDa; hypothetical protein LOC100129492	Homo sapiens
57446	NDRG family member 3	Homo sapiens
51318	mitochondrial ribosomal protein L35	Homo sapiens
22924	microtubule-associated protein, RP/EB family, member 3	Homo sapiens
4201	male-enhanced antigen 1	Homo sapiens
51290	ERGIC and golgi 2	Homo sapiens
8131	chromosome 16 open reading frame 35	Homo sapiens
29974	APOBEC1 complementation factor	Homo sapiens
51645	peptidylprolyl isomerase (cyclophilin)-like 1	Homo sapiens
4238	microfibrillar-associated protein 3	Homo sapiens
11080	DnaJ (Hsp40) homolog, subfamily B, member 4	Homo sapiens
9783	regulating synaptic membrane exocytosis 3	Homo sapiens
29998	glioma tumor suppressor candidate region gene 1	Homo sapiens
55347	abhydrolase domain containing 10	Homo sapiens
9702	centrosomal protein 57kDa	Homo sapiens
9948	WD repeat domain 1	Homo sapiens
284273	zinc binding alcohol dehydrogenase domain containing 2	Homo sapiens
57646	ubiquitin specific peptidase 28	Homo sapiens
10075	HECT, UBA and WWE domain containing 1	Homo sapiens
55142	HAUS augmin-like complex, subunit 2	Homo sapiens
6242	rhotekin	Homo sapiens
8562	density-regulated protein	Homo sapiens
5557	primase, DNA, polypeptide 1 (49kDa)	Homo sapiens
51144	hydroxysteroid (17-beta) dehydrogenase 12	Homo sapiens
359	aquaporin 2 (collecting duct)	Homo sapiens
84961	F-box and leucine-rich repeat protein 20	Homo sapiens
55	acid phosphatase, prostate	Homo sapiens
51762	RAB8B, member RAS oncogene family	Homo sapiens
51540	selenocysteine lyase	Homo sapiens
11065	ubiquitin-conjugating enzyme E2C	Homo sapiens
80020	FAD-dependent oxidoreductase domain containing 2	Homo sapiens
3720	jumonji, AT rich interactive domain 2	Homo sapiens
3475	interferon-related developmental regulator 1	Homo sapiens
9168	thymosin beta 10	Homo sapiens
821	calnexin	Homo sapiens
54897	castor zinc finger 1	Homo sapiens

55759	WD repeat domain 12	Homo sapiens
162	adaptor-related protein complex 1, beta 1 subunit	Homo sapiens
7716	vascular endothelial zinc finger 1	Homo sapiens
22913	RNA binding protein, autoantigenic (hnRNP-associated with lethal yellow homolog (mouse))	Homo sapiens
55206	strawberry notch homolog 1 (Drosophila)	Homo sapiens
113510	helicase, POLQ-like	Homo sapiens
1017	cyclin-dependent kinase 2	Homo sapiens
7029	transcription factor Dp-2 (E2F dimerization partner 2)	Homo sapiens
283377	SPRY domain containing 4	Homo sapiens
54620	F-box and leucine-rich repeat protein 19	Homo sapiens
10365	Kruppel-like factor 2 (lung)	Homo sapiens
25777	unc-84 homolog B (C. elegans)	Homo sapiens
727940	Rhox homeobox family, member 2B	Homo sapiens
23089	paternally expressed 10	Homo sapiens
115330	G protein-coupled receptor 146	Homo sapiens
23205	acyl-CoA synthetase bubblegum family member 1	Homo sapiens
10097	ARP2 actin-related protein 2 homolog (yeast)	Homo sapiens
7546	Zic family member 2 (odd-paired homolog, Drosophila)	Homo sapiens
3839	karyopherin alpha 3 (importin alpha 4)	Homo sapiens
2677	gamma-glutamyl carboxylase	Homo sapiens
5467	peroxisome proliferator-activated receptor delta	Homo sapiens
3479	insulin-like growth factor 1 (somatomedin C)	Homo sapiens
656	bone morphogenetic protein 8b	Homo sapiens
144363	LYR motif containing 5	Homo sapiens
55108	BSD domain containing 1	Homo sapiens
375248	ankyrin repeat domain 36; similar to ankyrin repeat domain 36	Homo sapiens
652726	ankyrin repeat domain 36; similar to ankyrin repeat domain 36	Homo sapiens
1641	doublecortin	Homo sapiens
153090	DAB2 interacting protein	Homo sapiens
6160	ribosomal protein L31 pseudogene 49; ribosomal protein L31 pseudogene 17; ribosomal protein L31	Homo sapiens
100129882	ribosomal protein L31 pseudogene 49; ribosomal protein L31 pseudogene 17; ribosomal protein L31	Homo sapiens
653773	ribosomal protein L31 pseudogene 49; ribosomal protein L31 pseudogene 17; ribosomal protein L31	Homo sapiens
6198	ribosomal protein S6 kinase, 70kDa, polypeptide 1	Homo sapiens
4340	myelin oligodendrocyte glycoprotein	Homo sapiens
22849	cytoplasmic polyadenylation element binding protein 3	Homo sapiens
113235	solute carrier family 46 (folate transporter), member 1	Homo sapiens
152006	ring finger protein 38	Homo sapiens
1968	eukaryotic translation initiation factor 2, subunit 3 gamma, 52kDa	Homo sapiens
1604	CD55 molecule, decay accelerating factor for complement (Cromer blood group)	Homo sapiens
23111	spastic paraplegia 20 (Troyer syndrome)	Homo sapiens
26517	translocase of inner mitochondrial membrane 13 homolog (yeast)	Homo sapiens
23530	nicotinamide nucleotide transhydrogenase	Homo sapiens
51528	chromosome 14 open reading frame 100	Homo sapiens
221061	family with sequence similarity 171, member A1	Homo sapiens
348262	hypothetical protein LOC348262	Homo sapiens
6921	similar to elongin C; transcription elongation factor B (SIII), polypeptide 1 (15kDa, elongin C)	Homo sapiens
100132973	similar to elongin C; transcription elongation factor B (SIII), polypeptide 1 (15kDa, elongin C)	Homo sapiens
5195	peroxisomal biogenesis factor 14	Homo sapiens
9026	huntingtin interacting protein 1 related	Homo sapiens
80155	NMDA receptor regulated 1	Homo sapiens
253559	cell adhesion molecule 2	Homo sapiens
57189	KIAA1147	Homo sapiens
11221	dual specificity phosphatase 10	Homo sapiens
23607	CD2-associated protein	Homo sapiens
377	ADP-ribosylation factor 3	Homo sapiens
10217	CTD (carboxy-terminal domain, RNA polymerase II, polypeptide A) small phosphatase-like	Homo sapiens
84881	RNA pseudouridylate synthase domain containing 4	Homo sapiens
147040	potassium channel tetramerisation domain containing 11	Homo sapiens
3659	interferon regulatory factor 1	Homo sapiens
132720	chromosome 4 open reading frame 32	Homo sapiens
990	cell division cycle 6 homolog (S. cerevisiae)	Homo sapiens
350	apolipoprotein H (beta-2-glycoprotein I)	Homo sapiens
9361	lon peptidase 1, mitochondrial	Homo sapiens
11329	serine/threonine kinase 38	Homo sapiens
9420	cytochrome P450, family 7, subfamily B, polypeptide 1	Homo sapiens
149840	chromosome 20 open reading frame 196	Homo sapiens
148213	zinc finger protein 681	Homo sapiens
10208	ubiquitin specific peptidase like 1	Homo sapiens
9188	DEAD (Asp-Glu-Ala-Asp) box polypeptide 21	Homo sapiens
56916	SWI/SNF-related, matrix-associated actin-dependent regulator of chromatin, subfamily a, containing DEAD/H box 1	Homo sapiens
64786	TBC1 domain family, member 15	Homo sapiens
6510	solute carrier family 1 (neutral amino acid transporter), member 5	Homo sapiens
84269	coiled-coil-helix-coiled-coil-helix domain containing 5	Homo sapiens

89890	kelch repeat and BTB (POZ) domain containing 6	Homo sapiens
421	armadillo repeat gene deletes in velocardiofacial syndrome	Homo sapiens
2339	farnesyltransferase, CAAX box, alpha	Homo sapiens
10095	actin related protein 2/3 complex, subunit 1B, 41kDa; similar to Actin-related protein 2/3 complex subunit 1B (ARP2/3 complex 41 kDa subunit) (p41-ARC)	Homo sapiens
653888	actin related protein 2/3 complex, subunit 1B, 41kDa; similar to Actin-related protein 2/3 complex subunit 1B (ARP2/3 complex 41 kDa subunit) (p41-ARC)	Homo sapiens
10611	PDZ and LIM domain 5	Homo sapiens
89796	neuron navigator 1	Homo sapiens
150365	meiosis inhibitor 1	Homo sapiens
126069	zinc finger protein 491	Homo sapiens
100131509	similar to hkir2.2x; similar to inward rectifying K+ channel negative regulator Kir2.2v; potassium inwardly-rectifying channel, subfamily J, member 12	Homo sapiens
3768	similar to hkir2.2x; similar to inward rectifying K+ channel negative regulator Kir2.2v; potassium inwardly-rectifying channel, subfamily J, member 12	Homo sapiens
100134444	similar to hkir2.2x; similar to inward rectifying K+ channel negative regulator Kir2.2v; potassium inwardly-rectifying channel, subfamily J, member 12	Homo sapiens
257629	ankyrin repeat and sterile alpha motif domain containing 4B	Homo sapiens
2050	EPH receptor B4	Homo sapiens
10793	zinc finger protein 273	Homo sapiens
100132742	ribosomal protein L17 pseudogene 22; ribosomal protein L17 pseudogene 36; ribosomal protein L17 pseudogene 20; similar to ribosomal protein L17; ribosomal protein L17 pseudogene 33; ribosomal protein L17 pseudogene 34; ribosomal protein L17 pseudogene 9; ribosomal protein L17; ribosomal protein L17 pseudogene 18; ribosomal protein L17 pseudogene 7; ribosomal protein L17 pseudogene 39	Homo sapiens
391655	ribosomal protein L17 pseudogene 22; ribosomal protein L17 pseudogene 36; ribosomal protein L17 pseudogene 20; similar to ribosomal protein L17; ribosomal protein L17 pseudogene 33; ribosomal protein L17 pseudogene 34; ribosomal protein L17 pseudogene 9; ribosomal protein L17; ribosomal protein L17 pseudogene 18; ribosomal protein L17 pseudogene 7; ribosomal protein L17 pseudogene 39	Homo sapiens
727984	ribosomal protein L17 pseudogene 22; ribosomal protein L17 pseudogene 36; ribosomal protein L17 pseudogene 20; similar to ribosomal protein L17; ribosomal protein L17 pseudogene 33; ribosomal protein L17 pseudogene 34; ribosomal protein L17 pseudogene 9; ribosomal protein L17; ribosomal protein L17 pseudogene 18; ribosomal protein L17 pseudogene 7; ribosomal protein L17 pseudogene 39	Homo sapiens
729046	ribosomal protein L17 pseudogene 22; ribosomal protein L17 pseudogene 36; ribosomal protein L17 pseudogene 20; similar to ribosomal protein L17; ribosomal protein L17 pseudogene 33; ribosomal protein L17 pseudogene 34; ribosomal protein L17 pseudogene 9; ribosomal protein L17; ribosomal protein L17 pseudogene 18; ribosomal protein L17 pseudogene 7; ribosomal protein L17 pseudogene 39	Homo sapiens
729301	ribosomal protein L17 pseudogene 22; ribosomal protein L17 pseudogene 36; ribosomal protein L17 pseudogene 20; similar to ribosomal protein L17; ribosomal protein L17 pseudogene 33; ribosomal protein L17 pseudogene 34; ribosomal protein L17 pseudogene 9; ribosomal protein L17; ribosomal protein L17 pseudogene 18; ribosomal protein L17 pseudogene 7; ribosomal protein L17 pseudogene 39	Homo sapiens
729340	ribosomal protein L17 pseudogene 22; ribosomal protein L17 pseudogene 36; ribosomal protein L17 pseudogene 20; similar to ribosomal protein L17; ribosomal protein L17 pseudogene 33; ribosomal protein L17 pseudogene 34; ribosomal protein L17 pseudogene 9; ribosomal protein L17; ribosomal protein L17 pseudogene 18; ribosomal protein L17 pseudogene 7; ribosomal protein L17 pseudogene 39	Homo sapiens
646909	ribosomal protein L17 pseudogene 22; ribosomal protein L17 pseudogene 36; ribosomal protein L17 pseudogene 20; similar to ribosomal protein L17; ribosomal protein L17 pseudogene 33; ribosomal protein L17 pseudogene 34; ribosomal protein L17 pseudogene 9; ribosomal protein L17; ribosomal protein L17 pseudogene 18; ribosomal protein L17 pseudogene 7; ribosomal protein L17 pseudogene 39	Homo sapiens
645296	ribosomal protein L17 pseudogene 22; ribosomal protein L17 pseudogene 36; ribosomal protein L17 pseudogene 20; similar to ribosomal protein L17; ribosomal protein L17 pseudogene 33; ribosomal protein L17 pseudogene 34; ribosomal protein L17 pseudogene 9; ribosomal protein L17; ribosomal protein L17 pseudogene 18; ribosomal protein L17 pseudogene 7; ribosomal protein L17 pseudogene 39	Homo sapiens
6139	ribosomal protein L17 pseudogene 22; ribosomal protein L17 pseudogene 36; ribosomal protein L17 pseudogene 20; similar to ribosomal protein L17; ribosomal protein L17 pseudogene 33; ribosomal protein L17 pseudogene 34; ribosomal protein L17 pseudogene 9; ribosomal protein L17; ribosomal protein L17 pseudogene 18; ribosomal protein L17 pseudogene 7; ribosomal protein L17 pseudogene 39	Homo sapiens
100129657	ribosomal protein L17 pseudogene 22; ribosomal protein L17 pseudogene 36; ribosomal protein L17 pseudogene 20; similar to ribosomal protein L17; ribosomal protein L17 pseudogene 33; ribosomal protein L17 pseudogene 34; ribosomal protein L17 pseudogene 9; ribosomal protein L17; ribosomal protein L17 pseudogene 18; ribosomal protein L17 pseudogene 7; ribosomal protein L17 pseudogene 39	Homo sapiens
643863	ribosomal protein L17 pseudogene 22; ribosomal protein L17 pseudogene 36; ribosomal protein L17 pseudogene 20; similar to ribosomal protein L17; ribosomal protein L17 pseudogene 33; ribosomal protein L17 pseudogene 34; ribosomal protein L17 pseudogene 9; ribosomal protein L17; ribosomal protein L17 pseudogene 18; ribosomal protein L17 pseudogene 7; ribosomal protein L17 pseudogene 39	Homo sapiens
1845	dual specificity phosphatase 3	Homo sapiens
64328	exportin 4	Homo sapiens
104	adenosine deaminase, RNA-specific, B1 (RED1 homolog rat)	Homo sapiens
5978	RE1-silencing transcription factor	Homo sapiens

2560	gamma-aminobutyric acid (GABA) A receptor, beta 1	Homo sapiens
9424	potassium channel, subfamily K, member 6	Homo sapiens
10943	male-specific lethal 3 homolog (Drosophila)	Homo sapiens
25833	POU class 2 homeobox 3	Homo sapiens
7048	transforming growth factor, beta receptor II (70/80kDa)	Homo sapiens
730013	ATP-binding cassette, sub-family C, member 6 pseudogene 2; ATP-binding cassette, sub-family C (CFTR/MRP), member 6	Homo sapiens
368	ATP-binding cassette, sub-family C, member 6 pseudogene 2; ATP-binding cassette, sub-family C (CFTR/MRP), member 6	Homo sapiens
81793	toll-like receptor 10	Homo sapiens
55182	ring finger protein 220	Homo sapiens
23165	nucleoporin 205kDa	Homo sapiens
84790	tubulin, alpha 1c	Homo sapiens
9695	ER degradation enhancer, mannosidase alpha-like 1	Homo sapiens
7781	solute carrier family 30 (zinc transporter), member 3	Homo sapiens
3608	interleukin enhancer binding factor 2, 45kDa	Homo sapiens
55028	chromosome 17 open reading frame 80	Homo sapiens
1827	regulator of calcineurin 1	Homo sapiens
5058	p21 protein (Cdc42/Rac)-activated kinase 1	Homo sapiens
10613	ER lipid raft associated 1	Homo sapiens
29964	prickle homolog 4 (Drosophila)	Homo sapiens
51117	coenzyme Q4 homolog (S. cerevisiae)	Homo sapiens
1184	chloride channel 5	Homo sapiens
9525	vacuolar protein sorting 4 homolog B (S. cerevisiae)	Homo sapiens
79094	ChaC, cation transport regulator homolog 1 (E. coli)	Homo sapiens
10146	GTPase activating protein (SH3 domain) binding protein 1	Homo sapiens
11140	cell division cycle 37 homolog (S. cerevisiae)	Homo sapiens
23223	ribosomal RNA processing 12 homolog (S. cerevisiae)	Homo sapiens
1829	desmoglein 2	Homo sapiens
153443	serum response factor binding protein 1	Homo sapiens
645086	chromosome 11 open reading frame 58 pseudogene; chromosome 11 open reading frame 58	Homo sapiens
10944	chromosome 11 open reading frame 58 pseudogene; chromosome 11 open reading frame 58	Homo sapiens
162966	zinc finger protein 600	Homo sapiens
8665	eukaryotic translation initiation factor 3, subunit F; similar to hCG2040283	Homo sapiens
390282	eukaryotic translation initiation factor 3, subunit F; similar to hCG2040283	Homo sapiens
51077	FCF1 small subunit (SSU) processome component homolog (S. cerevisiae)	Homo sapiens
100128525	ubiquinol-cytochrome c reductase, Rieske iron-sulfur polypeptide-like 1; ubiquinol-cytochrome c reductase, Rieske iron-sulfur polypeptide 1	Homo sapiens
7386	ubiquinol-cytochrome c reductase, Rieske iron-sulfur polypeptide-like 1; ubiquinol-cytochrome c reductase, Rieske iron-sulfur polypeptide 1	Homo sapiens
54813	kelch-like 28 (Drosophila)	Homo sapiens
339745	speckle-type POZ protein-like	Homo sapiens
7776	zinc finger protein 236	Homo sapiens
4192	midkine (neurite growth-promoting factor 2)	Homo sapiens
91807	myosin light chain kinase 3	Homo sapiens
653689	glutathione S-transferase theta 2B (gene/pseudogene); glutathione S-transferase theta 2	Homo sapiens
2953	glutathione S-transferase theta 2B (gene/pseudogene); glutathione S-transferase theta 2	Homo sapiens
1635	dCMP deaminase	Homo sapiens
1326	mitogen-activated protein kinase kinase kinase 8	Homo sapiens
23369	pumilio homolog 2 (Drosophila)	Homo sapiens
10308	zinc finger protein 267	Homo sapiens
1859	dual-specificity tyrosine-(Y)-phosphorylation regulated kinase 1A	Homo sapiens
26505	cyclin M3	Homo sapiens
81887	LAS1-like (S. cerevisiae)	Homo sapiens
7020	transcription factor AP-2 alpha (activating enhancer binding protein 2 alpha)	Homo sapiens
10020	glucosamine (UDP-N-acetyl)-2-epimerase/N-acetylmannosamine kinase	Homo sapiens
2188	Fanconi anemia, complementation group F	Homo sapiens
728489	DNL-type zinc finger	Homo sapiens
144110	transmembrane protein 86A	Homo sapiens
79158	N-acetylglucosamine-1-phosphate transferase, alpha and beta subunits	Homo sapiens
3421	isocitrate dehydrogenase 3 (NAD+) gamma	Homo sapiens
7388	ubiquinol-cytochrome c reductase hinge protein-like; ubiquinol-cytochrome c reductase hinge protein	Homo sapiens
440567	ubiquinol-cytochrome c reductase hinge protein-like; ubiquinol-cytochrome c reductase hinge protein	Homo sapiens
2673	glutamine-fructose-6-phosphate transaminase 1	Homo sapiens
80095	zinc finger protein 606	Homo sapiens
5814	purine-rich element binding protein B	Homo sapiens
86	actin-like 6A	Homo sapiens
1655	DEAD (Asp-Glu-Ala-Asp) box polypeptide 5	Homo sapiens
166793	zinc finger protein 509	Homo sapiens
1944	ephrin-A3	Homo sapiens
134510	ubiquitin-like domain containing CTD phosphatase 1	Homo sapiens
5660	prosaposin	Homo sapiens
56957	OTU domain containing 7B	Homo sapiens
22839	discs, large (Drosophila) homolog-associated protein 4	Homo sapiens
1025	cyclin-dependent kinase 9	Homo sapiens
9698	pumilio homolog 1 (Drosophila)	Homo sapiens
8818	dolichyl-phosphate mannosyltransferase polypeptide 2, regulatory subunit	Homo sapiens

678	zinc finger protein 36, C3H type-like 2	Homo sapiens
246269	lactation elevated 1	Homo sapiens
50488	misshapen-like kinase 1 (zebrafish)	Homo sapiens
170850	potassium voltage-gated channel, subfamily G, member 3	Homo sapiens
389524	GTF2I repeat domain containing 2B	Homo sapiens
164656	transmembrane protease, serine 6	Homo sapiens
8663	eukaryotic translation initiation factor 3, subunit C	Homo sapiens
4804	nerve growth factor receptor (TNFR superfamily, member 16)	Homo sapiens
79188	transmembrane protein 43	Homo sapiens
55341	large subunit GTPase 1 homolog (S. cerevisiae)	Homo sapiens
23418	crumbs homolog 1 (Drosophila)	Homo sapiens
8349	histone cluster 2, H2be	Homo sapiens
7562	zinc finger protein 708	Homo sapiens
653155	similar to hCG1820375; PRP4 pre-mRNA processing factor 4 homolog B (yeast)	Homo sapiens
8899	similar to hCG1820375; PRP4 pre-mRNA processing factor 4 homolog B (yeast)	Homo sapiens
64744	small ArfGAP2	Homo sapiens
2935	G1 to S phase transition 1	Homo sapiens
90441	zinc finger protein 622	Homo sapiens
25820	ariadne homolog, ubiquitin-conjugating enzyme E2 binding protein, 1 (Drosophila)	Homo sapiens
9412	mediator complex subunit 21	Homo sapiens
2643	GTP cyclohydrolase 1	Homo sapiens
5090	pre-B-cell leukemia homeobox 3	Homo sapiens
27089	ubiquinol-cytochrome c reductase, complex III subunit VII, 9.5kDa	Homo sapiens
134430	WD repeat domain 36	Homo sapiens
5916	retinoic acid receptor, gamma	Homo sapiens
64388	gremlin 2, cysteine knot superfamily, homolog (Xenopus laevis)	Homo sapiens
391059	ferric-chelate reductase 1	Homo sapiens
25775	chromosome 22 open reading frame 24	Homo sapiens
147841	SPC24, NDC80 kinetochore complex component, homolog (S. cerevisiae)	Homo sapiens
340252	zinc finger protein 680	Homo sapiens
4850	CCR4-NOT transcription complex, subunit 4	Homo sapiens
292	solute carrier family 25 (mitochondrial carrier; adenine nucleotide translocator), member 5; solute carrier family 25 (mitochondrial carrier; adenine nucleotide translocator), member 5 pseudogene 8	Homo sapiens
392301	solute carrier family 25 (mitochondrial carrier; adenine nucleotide translocator), member 5; solute carrier family 25 (mitochondrial carrier; adenine nucleotide translocator), member 5 pseudogene 8	Homo sapiens
148479	PHD finger protein 13	Homo sapiens
8076	microfibrillar associated protein 5	Homo sapiens
652826	similar to 26S protease regulatory subunit 6B (MIP224) (MB67-interacting protein) (TAT-binding protein 7) (TBP-7); proteasome (prosome, macropain) 26S subunit, ATPase, 4	Homo sapiens
5704	similar to 26S protease regulatory subunit 6B (MIP224) (MB67-interacting protein) (TAT-binding protein 7) (TBP-7); proteasome (prosome, macropain) 26S subunit, ATPase, 4	Homo sapiens
56674	TMEM9 domain family, member B	Homo sapiens
23094	signal-induced proliferation-associated 1 like 3	Homo sapiens
9765	zinc finger, FYVE domain containing 16	Homo sapiens
8340	histone cluster 1, H2bl	Homo sapiens
751867	regulator of chromosome condensation 1; SNHG3-RCC1 readthrough transcript	Homo sapiens
1104	regulator of chromosome condensation 1; SNHG3-RCC1 readthrough transcript	Homo sapiens
3549	Indian hedgehog homolog (Drosophila)	Homo sapiens
56910	StAR-related lipid transfer (START) domain containing 7	Homo sapiens
1026	cyclin-dependent kinase inhibitor 1A (p21, Cip1)	Homo sapiens
8835	suppressor of cytokine signaling 2	Homo sapiens
93517	short chain dehydrogenase/reductase family 42E, member 1	Homo sapiens
54552	guanine nucleotide binding protein-like 3 (nucleolar)-like	Homo sapiens
5859	glutaminyl-tRNA synthetase	Homo sapiens
6240	ribonucleotide reductase M1	Homo sapiens
23142	DCN1, defective in cullin neddylation 1, domain containing 4 (S. cerevisiae)	Homo sapiens
54386	telomeric repeat binding factor 2, interacting protein	Homo sapiens
9419	cysteine-rich PDZ-binding protein	Homo sapiens
9556	chromosome 14 open reading frame 2	Homo sapiens
5693	proteasome (prosome, macropain) subunit, beta type, 5	Homo sapiens
64077	phospholysine phosphohistidine inorganic pyrophosphate phosphatase	Homo sapiens
5361	plexin A1	Homo sapiens
11331	prohibitin 2	Homo sapiens
9002	coagulation factor II (thrombin) receptor-like 3	Homo sapiens
8517	inhibitor of kappa light polypeptide gene enhancer in B-cells, kinase gamma	Homo sapiens
57478	ubiquitin specific peptidase 31	Homo sapiens
976	CD97 molecule	Homo sapiens
163033	zinc finger protein 579	Homo sapiens
5099	protocadherin 7	Homo sapiens
29127	Rac GTPase activating protein 1 pseudogene; Rac GTPase activating protein 1	Homo sapiens
83956	Rac GTPase activating protein 1 pseudogene; Rac GTPase activating protein 1	Homo sapiens
5510	protein phosphatase 1, regulatory (inhibitor) subunit 7	Homo sapiens
8727	catenin (cadherin-associated protein), alpha-like 1	Homo sapiens
54891	INO80 complex subunit D	Homo sapiens
23077	MYC binding protein 2	Homo sapiens
645822	similar to TBC1 domain family, member 3; ubiquitin specific peptidase 32	Homo sapiens
84669	similar to TBC1 domain family, member 3; ubiquitin specific peptidase 32	Homo sapiens

6018	rearranged L-myc fusion	Homo sapiens
2806	glutamic-oxaloacetic transaminase 2, mitochondrial (aspartate aminotransferase 2)	Homo sapiens
10131	TNF receptor-associated protein 1	Homo sapiens
9683	NEDD4 binding protein 1	Homo sapiens
1182	chloride channel 3	Homo sapiens
51393	transient receptor potential cation channel, subfamily V, member 2	Homo sapiens
3556	interleukin 1 receptor accessory protein	Homo sapiens
26508	hairy/enhancer-of-split related with YRPW motif-like	Homo sapiens
8449	DEAH (Asp-Glu-Ala-His) box polypeptide 16	Homo sapiens
7851	mal, T-cell differentiation protein-like	Homo sapiens
147660	zinc finger protein 578	Homo sapiens
150290	dual specificity phosphatase 18	Homo sapiens
9526	mannose-P-dolichol utilization defect 1	Homo sapiens
7710	zinc finger protein 154	Homo sapiens
23382	adenosylhomocysteinase-like 2	Homo sapiens
84275	solute carrier family 25, member 33	Homo sapiens
55835	centromere protein J	Homo sapiens
7791	zyxin	Homo sapiens
91607	schlafen family member 11	Homo sapiens
57761	tribbles homolog 3 (Drosophila)	Homo sapiens
3615	IMP (inosine monophosphate) dehydrogenase 2	Homo sapiens
25800	solute carrier family 39 (zinc transporter), member 6	Homo sapiens
124454	glutamyl-tRNA synthetase 2, mitochondrial (putative)	Homo sapiens
10123	ADP-ribosylation factor-like 4C	Homo sapiens
390874	one cut homeobox 3	Homo sapiens
100131098	hypothetical protein LOC100131098; calcium channel, voltage-dependent, L type, alpha 1C subunit	Homo sapiens
775	hypothetical protein LOC100131098; calcium channel, voltage-dependent, L type, alpha 1C subunit	Homo sapiens
10885	WD repeat domain 3	Homo sapiens
23746	aryl hydrocarbon receptor interacting protein-like 1	Homo sapiens
9031	bromodomain adjacent to zinc finger domain, 1B	Homo sapiens
10962	myeloid/lymphoid or mixed-lineage leukemia (trithorax homolog, Drosophila); translocated to, 11	Homo sapiens
126526	chromosome 19 open reading frame 47	Homo sapiens
64768	inositol 1,3,4,5,6-pentakisphosphate 2-kinase	Homo sapiens
57458	transmembrane and coiled-coil domain family 3	Homo sapiens
3304	heat shock 70kDa protein 1A; heat shock 70kDa protein 1B	Homo sapiens
3303	heat shock 70kDa protein 1A; heat shock 70kDa protein 1B	Homo sapiens
4952	oculocerebrorenal syndrome of Lowe	Homo sapiens
29967	low density lipoprotein-related protein 12	Homo sapiens
9516	lipopolysaccharide-induced TNF factor	Homo sapiens
145741	family with sequence similarity 148, member A	Homo sapiens
7049	transforming growth factor, beta receptor III	Homo sapiens
79003	MIS12, MIND kinetochore complex component, homolog (S. pombe)	Homo sapiens
9879	DEAD (Asp-Glu-Ala-Asp) box polypeptide 46	Homo sapiens
79073	transmembrane protein 109	Homo sapiens
6574	solute carrier family 20 (phosphate transporter), member 1	Homo sapiens
84747	unc-119 homolog B (C. elegans)	Homo sapiens
9367	RAB9A, member RAS oncogene family	Homo sapiens
64854	ubiquitin specific peptidase 46	Homo sapiens
389084	chromosome 2 open reading frame 82	Homo sapiens
10384	butyrophilin, subfamily 3, member A3	Homo sapiens
653121	zinc finger and BTB domain containing 8A	Homo sapiens
54206	ERBB receptor feedback inhibitor 1	Homo sapiens
56241	sushi domain containing 2	Homo sapiens
8120	adaptor-related protein complex 3, beta 2 subunit	Homo sapiens
124997	WD repeat domain 81	Homo sapiens
64969	mitochondrial ribosomal protein S5	Homo sapiens
57050	UTP3, small subunit (SSU) processome component, homolog (S. cerevisiae)	Homo sapiens
961	CD47 molecule	Homo sapiens
2201	fibrillin 2	Homo sapiens
57544	thioredoxin domain containing 16	Homo sapiens
9874	tousled-like kinase 1	Homo sapiens
7477	wingless-type MMTV integration site family, member 7B	Homo sapiens
57801	hairy and enhancer of split 4 (Drosophila)	Homo sapiens
80032	zinc finger protein 556	Homo sapiens
164091	progestin and adipoQ receptor family member VII	Homo sapiens
79598	centrosomal protein 97kDa	Homo sapiens
55611	OTU domain, ubiquitin aldehyde binding 1	Homo sapiens
3903	leukocyte-associated immunoglobulin-like receptor 1	Homo sapiens
64131	xylosyltransferase I	Homo sapiens
57105	cysteinyl leukotriene receptor 2	Homo sapiens
8542	apolipoprotein L, 1	Homo sapiens
5079	paired box 5	Homo sapiens
150726	F-box protein 41	Homo sapiens
8887	Tax1 (human T-cell leukemia virus type I) binding protein 1	Homo sapiens
91120	zinc finger protein 682	Homo sapiens
8991	selenium binding protein 1	Homo sapiens

153339	similar to transmembrane protein 167A; transmembrane protein 167A	Homo sapiens
100129118	similar to transmembrane protein 167A; transmembrane protein 167A	Homo sapiens
4946	ornithine decarboxylase antizyme 1	Homo sapiens
257218	SNF2 histone linker PHD RING helicase	Homo sapiens
10594	PRP8 pre-mRNA processing factor 8 homolog (S. cerevisiae)	Homo sapiens
55114	Rho GTPase activating protein 17	Homo sapiens
10015	programmed cell death 6 interacting protein	Homo sapiens
771	carbonic anhydrase XII	Homo sapiens
5358	plastin 3 (T isoform)	Homo sapiens
64172	O-sialoglycoprotein endopeptidase-like 1	Homo sapiens
26053	autism susceptibility candidate 2	Homo sapiens
81839	vang-like 1 (van gogh, Drosophila)	Homo sapiens
1981	eukaryotic translation initiation factor 4 gamma, 1	Homo sapiens
222236	N-acyl phosphatidylethanolamine phospholipase D	Homo sapiens
84105	pterin-4 alpha-carbinolamine dehydratase/dimerization cofactor of hepatocyte nuclear factor 1 alpha (TCF1) 2	Homo sapiens
117584	ring finger and FYVE-like domain containing 1	Homo sapiens
132864	cytoplasmic polyadenylation element binding protein 2	Homo sapiens
5433	polymerase (RNA) II (DNA directed) polypeptide D	Homo sapiens
22834	zinc finger protein 652	Homo sapiens
50717	WD repeat domain 42A	Homo sapiens
644790	ribosomal protein S15a pseudogene 17; ribosomal protein S15a pseudogene 19; ribosomal protein S15a pseudogene 12; ribosomal protein S15a pseudogene 24; ribosomal protein S15a pseudogene 11; ribosomal protein S15a	Homo sapiens
6210	ribosomal protein S15a pseudogene 17; ribosomal protein S15a pseudogene 19; ribosomal protein S15a pseudogene 12; ribosomal protein S15a pseudogene 24; ribosomal protein S15a pseudogene 11; ribosomal protein S15a	Homo sapiens
391656	ribosomal protein S15a pseudogene 17; ribosomal protein S15a pseudogene 19; ribosomal protein S15a pseudogene 12; ribosomal protein S15a pseudogene 24; ribosomal protein S15a pseudogene 11; ribosomal protein S15a	Homo sapiens
648729	ribosomal protein S15a pseudogene 17; ribosomal protein S15a pseudogene 19; ribosomal protein S15a pseudogene 12; ribosomal protein S15a pseudogene 24; ribosomal protein S15a pseudogene 11; ribosomal protein S15a	Homo sapiens
646819	ribosomal protein S15a pseudogene 17; ribosomal protein S15a pseudogene 19; ribosomal protein S15a pseudogene 12; ribosomal protein S15a pseudogene 24; ribosomal protein S15a pseudogene 11; ribosomal protein S15a	Homo sapiens
100129243	ribosomal protein S15a pseudogene 17; ribosomal protein S15a pseudogene 19; ribosomal protein S15a pseudogene 12; ribosomal protein S15a pseudogene 24; ribosomal protein S15a pseudogene 11; ribosomal protein S15a	Homo sapiens
64979	mitochondrial ribosomal protein L36	Homo sapiens
55502	hairy and enhancer of split 6 (Drosophila)	Homo sapiens
23262	histidine acid phosphatase domain containing 1	Homo sapiens
7078	TIMP metallopeptidase inhibitor 3	Homo sapiens
8312	axin 1	Homo sapiens
132203	sentan, cilia apical structure protein	Homo sapiens
23039	exportin 7	Homo sapiens
60492	coiled-coil domain containing 90B	Homo sapiens
121006	family with sequence similarity 186, member A	Homo sapiens
245908	defensin, beta 105A; defensin, beta 105B	Homo sapiens
504180	defensin, beta 105A; defensin, beta 105B	Homo sapiens
5534	protein phosphatase 3 (formerly 2B), regulatory subunit B, alpha isoform	Homo sapiens
2319	flotillin 2	Homo sapiens
4040	low density lipoprotein receptor-related protein 6	Homo sapiens
50628	gem (nuclear organelle) associated protein 4	Homo sapiens
6050	ribonuclease/angiogenin inhibitor 1	Homo sapiens
57728	WD repeat domain 19	Homo sapiens
5683	proteasome (prosome, macropain) subunit, alpha type, 2	Homo sapiens
51246	shisa homolog 5 (Xenopus laevis)	Homo sapiens
161742	sprouty-related, EVH1 domain containing 1	Homo sapiens
8876	vanin 1	Homo sapiens
22884	WD repeat domain 37	Homo sapiens
376267	RAB15, member RAS onocogene family	Homo sapiens
22888	U-box domain containing 5	Homo sapiens
55041	pleckstrin homology domain containing, family B (evectins) member 2	Homo sapiens
144402	copine VIII	Homo sapiens
4814	ninjurin 1	Homo sapiens
7384	ubiquinol-cytochrome c reductase core protein I	Homo sapiens
2803	golgi autoantigen, golgin subfamily a, 4	Homo sapiens
836	caspase 3, apoptosis-related cysteine peptidase	Homo sapiens
5226	phosphogluconate dehydrogenase	Homo sapiens
196403	deltex homolog 3 (Drosophila)	Homo sapiens
55588	mediator complex subunit 29	Homo sapiens
121599	Spi-C transcription factor (Spi-1/PU.1 related)	Homo sapiens
30	acetyl-Coenzyme A acyltransferase 1	Homo sapiens
5931	retinoblastoma binding protein 7	Homo sapiens
147923	zinc finger protein 420	Homo sapiens
8323	frizzled homolog 6 (Drosophila)	Homo sapiens
8773	synaptosomal-associated protein, 23kDa	Homo sapiens
10556	ribonuclease P/MRP 30kDa subunit	Homo sapiens

6634	small nuclear ribonucleoprotein D3 polypeptide 18kDa	Homo sapiens
331	X-linked inhibitor of apoptosis	Homo sapiens
51270	transcription factor Dp family, member 3	Homo sapiens
57551	TAO kinase 1	Homo sapiens
23396	phosphatidylinositol-4-phosphate 5-kinase, type I, gamma	Homo sapiens
4904	Y box binding protein 1	Homo sapiens
51276	zinc finger protein 571	Homo sapiens
132204	synaptoporin	Homo sapiens
58525	widely interspaced zinc finger motifs	Homo sapiens
3641	insulin-like 4 (placenta)	Homo sapiens
63893	ubiquitin-conjugating enzyme E2O	Homo sapiens
4928	nucleoporin 98kDa	Homo sapiens
56603	cytochrome P450, family 26, subfamily B, polypeptide 1	Homo sapiens
64782	apoptosis enhancing nuclease	Homo sapiens
4249	mannosyl (alpha-1,6-)-glycoprotein beta-1,6-N-acetyl-glucosaminyltransferase; hypothetical LOC151162	Homo sapiens
151162	mannosyl (alpha-1,6-)-glycoprotein beta-1,6-N-acetyl-glucosaminyltransferase; hypothetical LOC151162	Homo sapiens
26292	c-myc binding protein	Homo sapiens
84327	zinc finger, BED-type containing 3	Homo sapiens
9631	nucleoporin 155kDa	Homo sapiens
3858	keratin 10	Homo sapiens
3399	inhibitor of DNA binding 3, dominant negative helix-loop-helix protein	Homo sapiens
29887	sorting nexin 10	Homo sapiens
56154	testis expressed 15	Homo sapiens
2876	glutathione peroxidase 1	Homo sapiens
6886	T-cell acute lymphocytic leukemia 1	Homo sapiens
23201	family with sequence similarity 168, member A	Homo sapiens
677790	transmembrane protein 78	Homo sapiens
4017	lysyl oxidase-like 2	Homo sapiens
337867	UBA domain containing 2	Homo sapiens
2264	fibroblast growth factor receptor 4	Homo sapiens
257364	sorting nexin 33	Homo sapiens
256472	transmembrane protein 151A	Homo sapiens
23219	F-box protein 28	Homo sapiens
8266	ubiquitin-like 4A	Homo sapiens
440270	golgi autoantigen, golgin subfamily a, 8B; golgi autoantigen, golgin subfamily a, 8A	Homo sapiens
23015	golgi autoantigen, golgin subfamily a, 8B; golgi autoantigen, golgin subfamily a, 8A	Homo sapiens
7389	uroporphyrinogen decarboxylase	Homo sapiens
4037	low density lipoprotein receptor-related protein 3	Homo sapiens
433	asialoglycoprotein receptor 2	Homo sapiens
23175	lipin 1	Homo sapiens
25949	SYF2 homolog, RNA splicing factor (S. cerevisiae)	Homo sapiens
4173	minichromosome maintenance complex component 4	Homo sapiens
6155	ribosomal protein L27	Homo sapiens
64764	cAMP responsive element binding protein 3-like 2	Homo sapiens
5710	proteasome (prosome, macropain) 26S subunit, non-ATPase, 4	Homo sapiens
5048	platelet-activating factor acetylhydrolase, isoform Ib, subunit 1 (45kDa)	Homo sapiens
5878	RAB5C, member RAS oncogene family	Homo sapiens
1603	defender against cell death 1	Homo sapiens
55298	ring finger protein 121	Homo sapiens
51009	Der1-like domain family, member 2	Homo sapiens
751071	methyltransferase like 12	Homo sapiens
22984	programmed cell death 11	Homo sapiens
10743	retinoic acid induced 1	Homo sapiens
10712	chromosome 1 open reading frame 2	Homo sapiens
10951	chromobox homolog 1 (HP1 beta homolog Drosophila)	Homo sapiens
4863	nuclear protein, ataxia-telangiectasia locus	Homo sapiens
4061	lymphocyte antigen 6 complex, locus E	Homo sapiens
375790	agrin	Homo sapiens
894	cyclin D2	Homo sapiens
10461	c-mer proto-oncogene tyrosine kinase	Homo sapiens
284325	chromosome 19 open reading frame 54	Homo sapiens
5045	furin (paired basic amino acid cleaving enzyme)	Homo sapiens
